# The Regulation Network of Glycerolipid Metabolism as Coregulators of Immunotherapy-Related Myocarditis

**DOI:** 10.1155/2023/8774971

**Published:** 2023-06-21

**Authors:** Xiguang Yang, Xiaopeng Duan, Zhenglin Xia, Rui Huang, Ke He, Guoan Xiang

**Affiliations:** ^1^Department of Gastrointestinal Surgery, Guigang City People's Hospital, Guigang, Guangxi 537100, China; ^2^Department of General Surgery, Guangdong Provincial Second People's Hospital, Guangzhou, Guangdong 510317, China

## Abstract

**Background:**

To date, immunotherapy for patients with malignant tumors has shown a significant association with myocarditis. However, the mechanism of metabolic reprogramming changes for immunotherapy-related cardiotoxicity is still not well understood.

**Methods:**

The CD45^+^ single-cell RNA sequencing (scRNA-seq) of the Pdcd1^−/−^Ctla4^+/-^ and wild-type mouse heart in GSE213486 was downloaded to demonstrate the heterogeneity of immunocyte atlas in immunotherapy-related myocarditis. The liquid chromatography–tandem mass spectrometry (LC-MS/MS) spectrum metabolomics analysis detects the metabolic network differences. The drug prediction, organelle level interaction, mitochondrial level regulatory network, and phosphorylation site prediction for key regulators have also been screened via multibioinformatics analysis methods.

**Results:**

The scRNA analysis shows that the T cell is the main regulatory cell subpopulation in the pathological progress of immunotherapy-related myocarditis. Mitochondrial regulation pathway significantly participated in pseudotime trajectory- (PTT-) related differential expressed genes (DEGs) in the T cell subpopulation. Additionally, both the gene set enrichment analysis (GSEA) of PTT-related DEGs and LC-MS/MS metabolomics analysis showed that mitochondrial-regulated glycerolipid metabolism plays a central role in metabolic reprogramming changes for immunotherapy-related cardiotoxicity. Finally, the hub-regulated protease of diacylglycerol kinase zeta (Dgkz) was significantly identified and widely played various roles in glycerolipid metabolism, oxidative phosphorylation, and lipid kinase activation.

**Conclusion:**

Mitochondrial-regulated glycerolipid metabolism, especially the DGKZ protein, plays a key role in the metabolic reprogramming of immunotherapy-related myocarditis.

## 1. Introduction

Myocarditis is an inflammatory disease caused by infection, physical, chemical, and other factors. It is characterized by inflammatory cell infiltration in the myocardium and cardiomyocyte degeneration and necrosis [1, 2]. Viruses, bacteria, parasites, chlamydia, radiation damage, exogenous toxins, and autoimmune diseases can all be the pathogenic factors [1]. The Global Burden of Disease Study 2013 indicated that the global incidence of myocarditis was approximately 2.2‰, while there is still a lack of information about other epidemiological data [1, 3]. Myocarditis is difficult to diagnose early. At present, there are no recognized and unified diagnostic criteria in the world. According to the *position statement of the European Society of Cardiology Working Group on Myocardial and Pericardial Diseases* published in 2013, the clinical diagnosis of myocarditis mainly relies on the comprehensive consideration of symptoms, medical history, myocardial markers, electrocardiographic findings, echocardiographic findings, and cardiac magnetic resonance imaging findings [4]. All this suggests that early diagnosis of myocarditis is still difficult [5]. The vast majority of patients were not diagnosed in time, thus missing the timing of treatment. In recent years, the application of comprehensive chemotherapy and targeted drugs has gradually become prevalent with the increasing number of cancer cases [5]. A type of novel immunotherapy drug, immune checkpoint inhibitors (ICIs), has been widely used in clinical practice [3]. However, more and more attention is being paid to the immune-related cardiotoxicity caused by ICIs. According to statistics, 1.14% of patients receiving ICI treatment developed myocarditis. Moreover, patients have an 8.2 times higher risk of cardiac death, 15 times higher risk of congestive heart failure, 10 times higher risk of cardiovascular diseases, and 9 times higher risk of stroke at 15-25 years after tumor diagnosis [1, 3]. Arguably, myocarditis is a growing threat to human health. There is a need to understand its pathophysiological mechanism.

In terms of pathological typing, myocarditis can be classified as lymphocytic type, lymphohistiocytic type, eosinophilic type, neutrophilic type, giant cell type, granulomatous type, etc., according to the type of inflammatory cells infiltrating in myocardial tissue [1, 3]. However, the specific clinical manifestations of its pathological subtypes are heterogeneous. At present, the mainstream view is that the development of myocarditis mainly results from myocardial damage caused by various factors, leading to myocardial degeneration and necrosis and the release of autoantigens (e.g., myosin), which come in contact with glial cells in myocardial tissue and activate T cell immunity to cause autoimmune inflammation [6, 7]. Autoimmune inflammation can aggravate myocarditis, prolong the recovery time, and damage the normal physiological function of the heart [7]. It can also cause myocardial fibrosis, chronic heart failure, and sudden cardiac death. The course of myocarditis involves three stages. The first one is the acute stage. In this stage, infection, physical damage, autoimmunity caused by other diseases, and other pathogenic factors directly damage the myocardium [8]. The second stage is immune activation. In this stage, as cardiomyocytes are fragmented, antigens in them are released, and various types of immune cells accumulate, causing subacute inflammation. Natural killer cells and macrophages accumulate first, followed by T cells. Myocardial damage gradually reaches its peak with the increasing T cell density. The third stage is regression. In this stage, the myocardial damage can be completely repaired in some patients. However, in a small proportion of patients, the inflammation can persist [8, 9].

Furthermore, the above pathological changes can damage the capillary intima of cardiac tissue, causing endothelial dysfunction and leading to myocardial tissue hypoperfusion and hypoxic injury or activating the upregulation of endothelial cell adhesion factors and leading to T cell accumulation and entrance to myocardial tissue [10]. Viruses often manifest as high aggregation of endothelial cells in myocardial biopsies [11]. Additionally, toll-like receptors on the surface of dendritic cells in cardiac tissue can activate the immune system, interact with various cells of the immune system, especially CD40^+^ T cells, mediate tissue damage repair, promote ventricular remodeling, promote tissue fibrosis, and cause myocardial fibrosis [6]. However, the precise mechanism of fibrosis remains unclear. With the continuous development of mass spectrometry in metabolomics, we can understand deeper mechanisms and gradually realize that metabolic disorders may play an important role in immunotherapy-related myocarditis. This project is aimed at systematically analyzing the metabolic disorder mechanism in myocarditis related to immunotherapy through comprehensive analysis using both single-cell transcriptomics and cardiac metabolomics and at investigating its pathophysiological mechanism, in the hope of providing a reference for the diagnosis of myocarditis and the treatment of autoimmune myocardial damage.

## 2. Methods

### 2.1. Single-Cell Data Download and Preprocessing

We downloaded the “barcodes,” “gene feature,” and “matrix” data of mice with immunotherapy-related myocarditis under the accession number GSE213486 [12] from the Gene Expression Omnibus database (GEO; https://www.ncbi.nlm.nih.gov/geo) [13]. The scRNA-seq of GSM6588008, GSM6588010, GSM6588012, and GSM6588014 transcriptomic single cells with library-type cDNA of the instrument model with Illumina NextSeq 500 was included. And the sample of GSM6588009, GSM6588011, and GSM6588013 of scTCR-seq was excluded. We read the raw expression profile data using the SingleCellExperiment (version 1.16.0), Seurat (version 4.1.0), and scatter (version 3.8) packages [14, 15]. Cell cycle phases were scored for each cell using the “scatter” cell cycle function. Cell cycle phases were defined as follows: G1 score > 0.5 and G2/M score < 0.5 were G1 phase; G2/M score > 0.5 and G1 score < 0.5 were G2/M phase; G1 score < 0.5 and G2/M score < 0.5 were S phase; G1 score > 0.5 and G2/M score > 0.5 was unknown. We selected cell barcodes with a minimum number of cells greater than 3 and the number of detected genes greater than 200 for single-cell analysis. We read the genes from mitochondria (containing “^MT-”) and erythrocytes (e.g., “HBA1,” “HBA2,” “HBB,” “HBD,” “HBE1,” “HBG1,” “HBG2,” “HBM,” “HBQ1,” and “HBZ”) to calculate the fractions of mitochondrial counts and erythrocytic counts, respectively, both of which should be less than 0.05.

**Figure 1:**
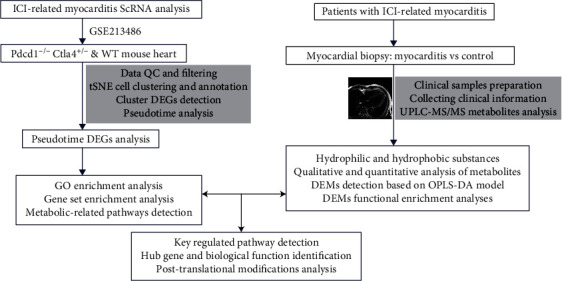
Flow chart depicting the study analysis.

At the same time, we filtered out cells with a number of detected genes less than 200, low-quality/dying cells, and empty droplets. The expression profile data were log-transformed using the LogNormalize function for background correction. We selected highly variable features in single-cell lineages as candidate regulatory genes using the FindVariableFeatures function [14, 15]. Principal component analysis was conducted for linear dimensionality reduction to determine the appropriate dimensions of datasets, followed by nonlinear dimensionality reduction and clustering using Uniform Manifold Approximation and Projection (UMAP) and *t*-distributed stochastic neighbor embedding (tSNE) algorithms. Then, we selected differentially expressed genes (DEGs) between cell clusters using the FindAllMarkers function [14, 15]. The parameter settings for DEGs were as follows: (1) the cutoff for the adjusted *P* value according to Benjamini-Hochberg's (B-H) method was 0.05, and (2) the minimum threshold of log |fold change (FC)| was 0.25. The analysis flow chart is shown in Figure 1. All the analyses are based on R software (version 4.1.3).

### 2.2. Cell Cluster Definition and Pseudotime Analysis

Cell clusters were annotated using the SingleR (version 1.8.1) and Celldex (version 1.1.1) algorithms. For clusters with inconsistent annotations between the two methods, cell markers were visualized to determine cell identities [16]. Here, we identified the type of cells within each cluster according to the following annotation references: “MouseRNAseqData,” “ImmGenData,” “DatabaseImmuneCellExpressionData,” and “MonacoImmuneData” cell type annotation [17]. We compared the percentage and number of cell clusters between groups to further extract core cell clusters for reclustering, followed by reannotation with the above algorithms. Subsequently, we used the monocle algorithm to perform a pseudotime analysis on the core cell clusters to map the trajectory of cells based on gene expression dynamics and define its regulatory nodes and relations [15, 18]. Then, based on pseudotime values, we conducted a differential analysis to determine dynamics-related DEGs and identify core regulators.

### 2.3. Differential Gene Identification for Core Cell Clusters and Functional Enrichment Analysis

We obtained pseudotime trajectory (PTT) DEGs between the core cell clusters using the FindAllMarkers function [15]. For this set of DEGs and the dynamics-related DEGs, we used the clusterProfiler (version 4.2.2) package to perform Gene Ontology (GO) enrichment analysis for information on involved biological processes, molecular functions, and cellular components, as well as gene set enrichment analysis (GSEA) for information on involved pathways [19].

### 2.4. Sample Preparation for Chromatography–Mass Spectrometry

To understand the changes in metabolites and metabolic networks in myocarditis after antitumor immunotherapy, we investigated metabolomic differences between myocarditis samples by ultraperformance liquid chromatography–tandem mass spectrometry (UPLC-MS/MS) in combination with database alignment and multivariate bioinformatics analysis [20, 21]. Samples were collected through myocardial biopsies from three patients who were diagnosed by experienced cardiovascular specialists with myocarditis after targeted immunotherapy for gastrointestinal tumors from December 1, 2020 to December 31, 2021. Normal samples were from heart donors who did not match transplant recipients. The research protocols were approved by the Ethics Committee of Guangdong Provincial Second People's Hospital (approval no. from the ethics committee: 20201113-3DDY-01-01). The age of patients was ranged from 55 to 59 years old, 2 patients are males, and another is male. The patients with advanced or metastatic gastric adenocarcinoma were diagnosed histologically or cytologically. And the 2 patients have received 4 prior systemic chemotherapy and 5 prior systemic chemotherapy for another patient. There was no history of hypertension, diabetes, and coronary heart disease before antitumor treatment. And all the patients are without lung disease, human immunodeficiency virus (HIV) infection, liver failure, or renal insufficiency. All the patient's medications were 3 weeks of antitumor drug treatment with ipilimumab 3 mg/kg, followed by 3 weeks of treatment with nivolumab 1 mg/kg.

All obtained tissues were stored in liquid nitrogen. Hydrophilic and hydrophobic substances were extracted separately from the tissues before UPLC-MS/MS. When extracting hydrophilic substances, we cut a piece of tissue (50 ± 2 mg) for homogenization at 30 Hz for 30 s for four times. After repeated checks of the quality of the homogenate, 1 mL 70% methanol was added into the homogenate-containing centrifuge tube, followed by oscillation for 5 min. Subsequently, the tube was centrifuged at 12000 r/min at 4 centigrade for 10 min for three times and then stood on the ice for 1 h. Finally, 200 *μ*L supernatant was taken into a sample vial insert for UPLC-MS/MS analysis. When extracting hydrophobic substances, we took 20 mg tissue in a 2 mL centrifuge tube and added 1 mL lipid extraction solution (methyl tert-butyl ether: methanol = 3 : 1, V/V, internal standard-containing mixed solution), followed by homogenization, repeated quality checks, and then centrifugation at 4 centigrade at 12000 r/min for 10 min for two times. Subsequently, 300 *μ*L supernatant was taken into a numbered 1.5 mL centrifuge tube for concentration, which was redissolved with 200 *μ*L acetonitrile (containing 0.1% formic acid) for UPLC-MS/MS analysis [22, 23].

### 2.5. UPLC-MS/MS Analysis

The prepared samples were analyzed by UPLC (ExionLC AD, https://sciex.com.cn/) and MS/MS (QTRAP®, https://sciex.com.cn/). MS conditions for hydrophilic substances are as follows: electrospray ionization temperature, 500-celsius degree; voltage, 5500 V (positive) and−4500 V (negative); ion source gas 1, 55 psi; ion source gas 2, 60 psi; curtain gas, 25 psi; collision-activated dissociation, high. MS conditions for hydrophobic substances are as follows: electrospray ionization temperature, 500-celsius degree; voltage, 5500 V (positive) and −4500 V (negative); ion source gas 1, 45 psi; ion source gas 2, 55 psi; curtain gas, 35 psi; collision-activated dissociation, medium. In the triple quadrupole mass spectrometer, each ion pair was detected based on optimized declustering potential and collision energy [24, 25].

### 2.6. Qualitative and Quantitative Analysis of Metabolites

Metabolites were quantified using the multiple reaction monitoring (MRM) modes in triple quadrupole mass spectrometry. We used Analyst 1.6.3 for mass spectra data quality control and metabolite peak detection. Chromatographic peaks were integrated and corrected using MultiQuant software [26]. Each chromatographic peak's integral area represented the corresponding substance's relative content. The integral areas of all chromatographic peaks were exported for subsequent differential and functional enrichment analyses of metabolites.

### 2.7. Differential and Functional Enrichment Analyses of Metabolites

Through principal component analysis and partial least squares-discriminant analysis (PLS-DA), based on MetaboAnalystR (version 1.0.1), we obtained variable importance in projection (VIP) in the orthogonal PLS-DA (OPLS-DA) model. Differentially expressed metabolites (DEMs) were selected using the VIP score (VIP score should be ≥1) in combination with univariable analysis-derived *P* values and fold changes [27, 28]. VIP score represents how much the intergroup difference of the metabolite would affect the model in classifying samples, and metabolites with VIP score ≥ 1 are generally considered significantly different. Then, we performed Kyoto Encyclopedia of Genes and Genomes (KEGG) enrichment analysis and metabolite set enrichment analysis (MSEA) on DEMs using MetaboAnalyst (https://www.metaboanalyst.ca/). Enriched pathways with *P* < 0.05 were considered to be core pathways [29, 30].

### 2.8. Identification of Core Pathways and Genes

Based on the core pathways derived from metabolomics, we further performed Gene Set Enrichment Analysis (GSEA) analysis on the pseudotime-related DEGs, to conversely confirm whether the core metabolomic pathways have regulatory significance. Here, the probability of occurrence of observed enrichment score (ES) is calculated by permutation test based on phenotype without changing the relationship between genes. And normalized enrichment score (NES) is obtained from the ES calculated for each gene subset according to the size of the gene set through multiple hypothesis tests. Then, calculate the false positive rate for NES.

In this GSEA analysis, the gene with the highest enrichment score (i.e., with the most significant regulatory function) would be the target molecule of immunotherapy-related myocarditis in this study. We used the ComPPI database (http://comppi.linkgroup.hu) to predict the interacting regulatory networks of the target molecule at cytoplasm, nucleus, cytosol, and mitochondrion levels, to clarify its potential regulatory mechanisms [31]. For interacting molecules at the mitochondrial level, we used ToppGene Suite (https://toppgene.cchmc.org) to predict the functions, pathways, and interactions of the target molecule [32]. Finally, we used PhosphoSitePlus (v6.6.0.4; https://www.phosphosite.org/homeAction) to analyze the modifications of the target protein, with high throughput papers as the main references [33, 34].

## 3. Results

### 3.1. Single-Cell RNA-seq Analysis

**Figure 2:**
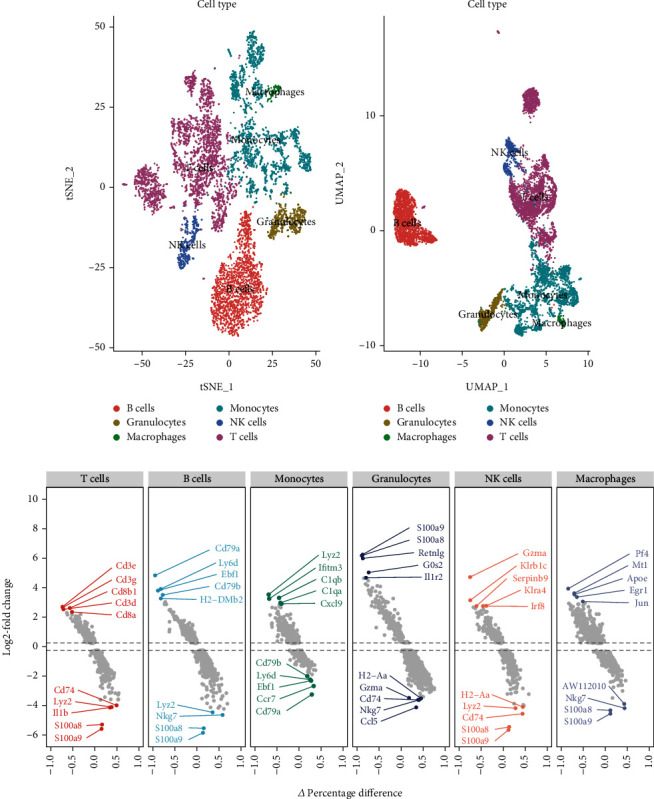
The CD45^+^ single-cell atlas of immunotherapy-related myocarditis among the Pdcd1^−/−^Ctla4^+/-^ and wild-type mouse heart. (a, b) The annotation-cell cluster was presented on tSNE and UMAP maps in immunotherapy-related myocarditis. (c) The scatter diagram shows the top higher/lower expressed differentially expressed genes (DEGs) in each annotation-cell cluster.

**Figure 3:**
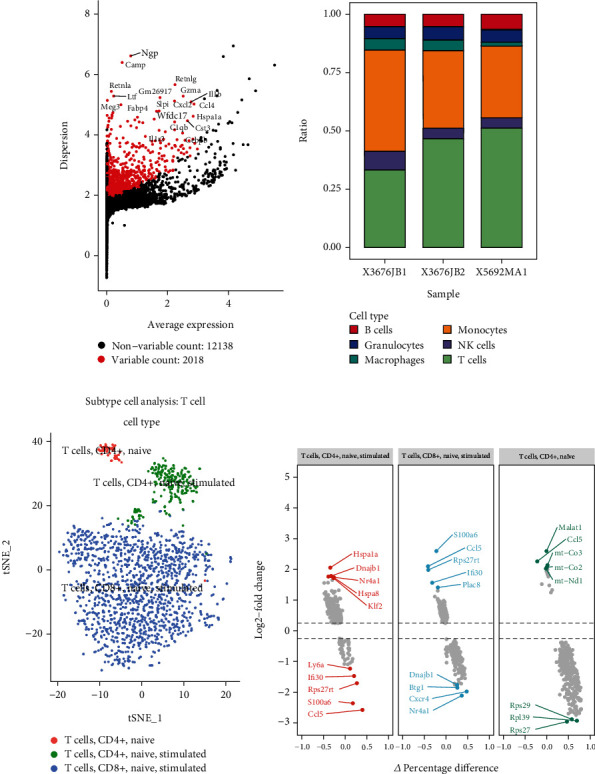
The key cell-cluster detection and reannotation. (a) The top variable marker expression level among the cell atlas of immunotherapy-related myocarditis among the Pdcd1^−/−^Ctla4^+/-^ and wild-type mouse heart. (b) The percentage of the annotated cell population. (c) The reannotation of T cell subtype. (d) The top higher/lower expressed DEGs in each subtype T cell.

Using the GSE213486 data series, we studied the heart tissues of four-week-old healthy C57BL6 mice and Pdcd1^–/–^Ctla4^+/–^ mice with myocarditis (simulating dual PD1/CTLA-targeted anticancer therapy in clinical practice). A total of 23566 CD45^+^ cells were obtained, which were annotated by tSNE/UMAP into six types of immune cell populations: B cells, granulocytes, macrophages, monocytes, NK cells, and T cells. T cells accounted for the largest proportion and revealed different spatial distributions between groups in two-dimensional tSNE and UMAP plots, suggesting a certain heterogeneity (Figures 2(a) and 2(b)). In T cells, Cd3e, Cd3g, Cd8b1, Cd3d, and Cd8a were among the most significantly upregulated DEGs, while Cd74, Lyz2, Il1b, S100a8, and S100a9 were among the most significantly downregulated DEGs. In B cells, the most significantly upregulated genes were Cd79a, Ly6d, Ebf1, Cd79b, and H2-DMb2, while the most significantly downregulated genes were Nkg7, Lyz2, S100a8, and S100a9. In monocytes, the most significantly upregulated genes were Lyz2, C1qb, Ifitm3, C1qa, and Cxcl9, while the most significantly downregulated genes were Cd79b, Ebf1, Ly6d, Ccr7, and Cd79a. In granulocytes, the most significantly upregulated genes were S100a9, S100a8, Retnlg, G0s2, and Il1r2, while the most significantly downregulated genes were H2-Aa, Cd74, Nkg7, Gzma, and Ccl5. In NK cells, the most significantly upregulated genes were Gzma, Klrb1c, Klra4, Serpinb9, and Irf8, while the most significantly downregulated genes were Cd74, H2-Aa, Lyz2, S100a8, and S100a9. In macrophages, the most significantly upregulated genes were Pf4, Mt1, Apoe, Egr1, and Jun, while the most significantly downregulated genes were AW112010, Nkg7, S100a8, and S100a9 (Figure 2(c) and Table S1). In addition, we identified Ngp, Camp, Retnla, Retnlg, and Ltf as highly variable genes in single-cell populations, which were considered to be closely related to cell heterogeneity regulation (Figure 3(a)). Moreover, the percentage of T cells was significantly different between myocarditis and healthy heart tissues (Figure 3(b)), indicating that T cells may play an important role in mediating immunotherapy-associated myocarditis.

### 3.2. Reclustering Analysis of Core Cell Clusters

**Figure 4:**
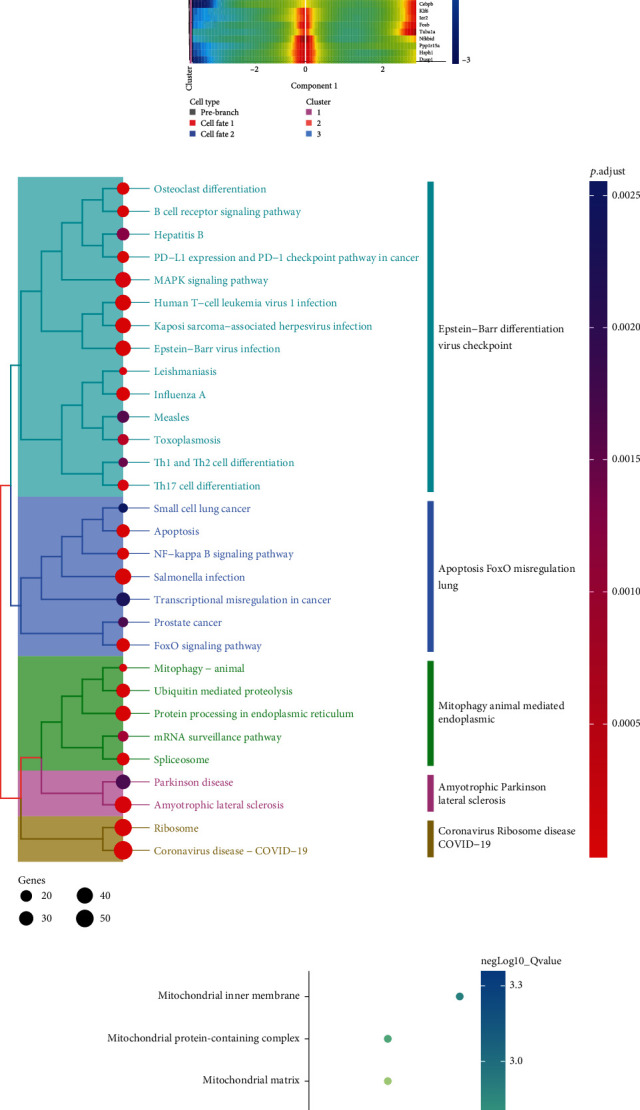
The pseudotime trajectory (PTT) and PTT-related DEG functional enrichment analysis among the T cells. (a) The pseudotime trajectory analysis of T cell subset development in immunotherapy-related myocarditis. (b) The heat map shows the expression level of top PPT-related DEGs. (c) The KEGG terms enrichment results for PTT-related DEGs. (d) The GSEA showed the map of mitochondrion significantly enriched in PTT-related DEGs. (e) GSEA analysis has shown that the mitochondrion-related pathway was significantly enriched, and genes include Hspd1, Mrps12, Map2k1, Uba52, and Fkbp4, which are also detected.

We performed reclustering analysis on T cells, which were divided into three groups in the UMAP plot (Figure 3(c)): CD4^+^ naive T cells, CD4^+^ naive T cells (stimulated), and CD8^+^ naive T cells (stimulated). In CD4^+^ naive T cells (stimulated), Hspa1a, Dnajb1, Nr4a1, Hspa8, and Klf2 were upregulated, while Ly6a, Ifi30, Rps27rt, S100a6, and Ccl5 were downregulated. In CD4^+^ naive T cells, Malat1, Ccl5, mt-Co3, mt-Co2, and mt-Nd1 were upregulated, while Rps29, Rpl39, and Rps27 were downregulated. In CD8+ naive T cells (stimulated), S100a6, Ccl5, Rps27rt, Ifi30, and Plac8 were upregulated, while Dnajb1, Btg1, Cxcr4, and Nr4a1 were downregulated (Figure 3(d)). The pseudotime analysis suggested that T cells were in three developmental stages (Figure 4(a)). The expression of main PPT-associated DEGs is shown in Figure 4(b) and Table S2. Through GO analysis of the development-related genes, we found that their main regulatory pathways involved the MAPK signaling pathway, human T cell leukemia virus 1 infection, Kaposi sarcoma−associated herpesvirus infection, Epstein−Barr virus infection, and PD-L1 expression and PD-1 checkpoint pathway in cancer (Figure 4(c), Table S3, and Table S4). The GSEA analysis suggested that these pseudotime-associated DEGs were mainly enriched in the mitochondrion pathway, where the rps12, Hspd1, Aldh2, P4ha1, and Ndufs6 genes were enriched (enrichment score = 0.541, normalized enrichment score = 2.784, *P* value = 3.13E-06, and adjusted *P* value = 2.406E-04; Figure 4(d) and Table S5).

### 3.3. Identification and Functional Enrichment Analysis of DEMs

**Figure 5:**
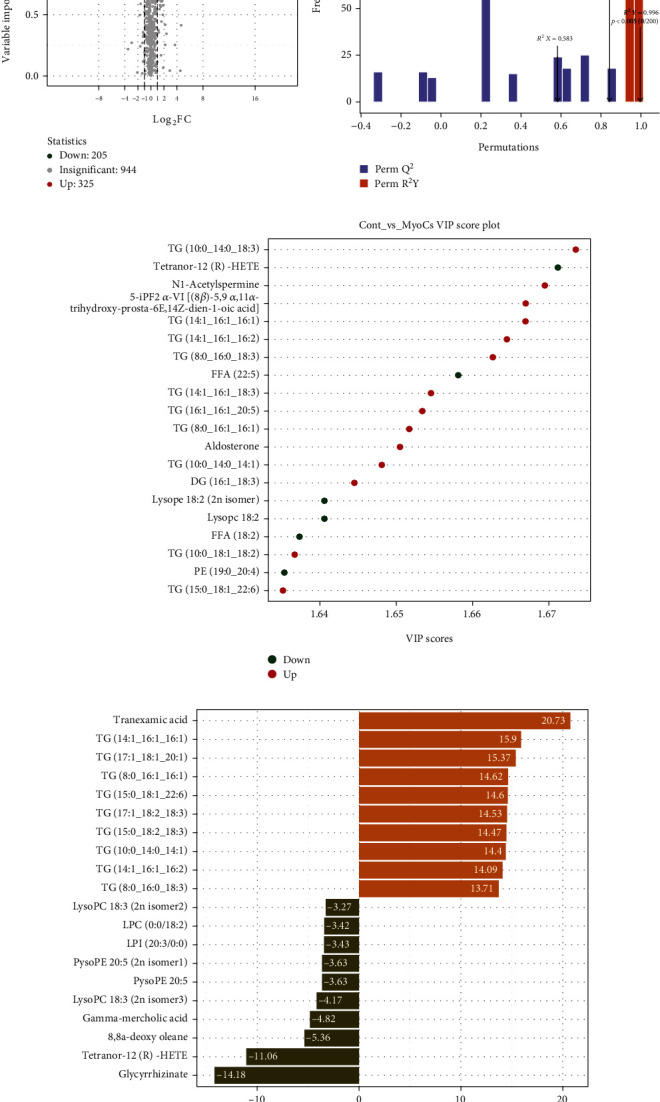
The differential metabolite analysis in immunotherapy-related myocarditis. (a) The differential metabolites (DMs) among the heart tissues of immunotherapy-related myocarditis and control group. (b) The OPLS-DA model for immunotherapy-related myocarditis (Q2 = 0.843, *P* < 0.005; *R*^2^ *Y* = 0.996, *P* < 0.005). (c) The VIP scores of the up and down DMs among the heart tissues of immunotherapy-related myocarditis and control group. (d) Top 20 DMs by Log2FC value with the heart tissues of immunotherapy-related myocarditis-compared control group.

**Figure 6:**
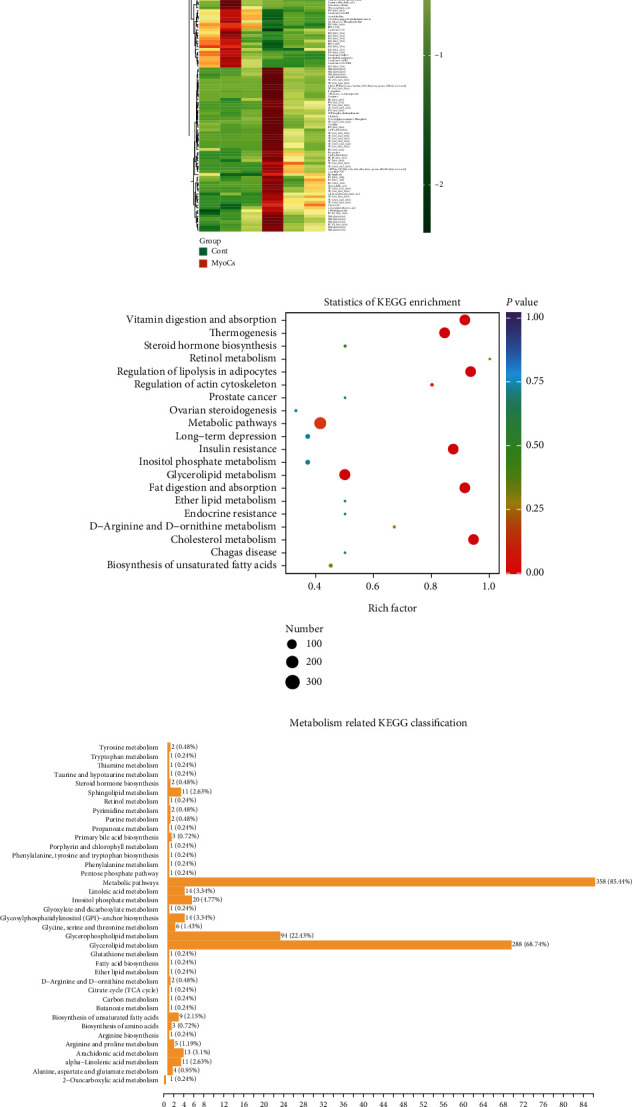
The functional enrichment analysis among the DMs in immunotherapy-related myocarditis. (a) The heat map of DM expression levels in response to immunotherapy-related myocarditis. (b) The KEGG enrichment analysis for the DMs of immunotherapy-related myocarditis. (c) The metabolism-related KEGG classification among the DMs of immunotherapy-related myocarditis. (d) The key metabolism-related pathways were detected via fold enrichment values for DMs.

By widely targeted metabolomic profiling, a total of 1474 metabolites were detected from myocarditis samples. We identified 205 downregulated and 325 upregulated DEMs in immunotherapy-related myocarditis tissues relative to normal heart tissues (Figure 5(a)). The OPLS-DA model had high accuracy, with Q2 being 0.843 (*P* < 0.005) and R2Y being 0.996 (*P* < 0.005) (Figure 5(b)). Based on the VIP score in the OPLS-DA model, we revealed that TG(10:0_14:0_18 : 3), N1-acetylspermine, 5-iPF2*α*-VI ((8*β*)-5,9*α*,11*α*-trihydroxy-prosta-6E,14Z-dien-1-oic acid), TG(14:1_16:1_16:1), and TG(14:1_16:1_16:2) were significantly upregulated metabolites, while FFA(22 : 5), Lysope 18 : 2 (2N isomer), Lysopc 18 : 2, FFA(18 : 2), PE(19:0_20:4), and tetranor-12(R)-HETE were significantly downregulated metabolites (Figure 5(c)). Based on fold changes, tranexamic acid, TG(14:1_16:1_16:1), TG(17:1_18:1_20:1), TG(8:0_16:1_16:1), and TG(15:0_18:1_22:6) were significantly upregulated, while LysoPC 18 : 3(2n isomer3), gamma-mercholic acid, 8,8a-deoxy oleane, tetranor-12(R)-HETE, and glycyrrhizinate were significantly downregulated (Figure 4(d)). The quantified expression of main DEMs is shown in Figure 6(a), Table 1, and Table S6.

We performed a functional enrichment analysis of DEMs based on the above results. From the perspective of rich factors (i.e., the proportion of DEMs enriched in a pathway in all DEMs), we found that retinol metabolism, vitamin digestion and absorption, and cholesterol metabolism were the main enriched pathways (Figure 6(b)). Based on KEGG classification, metabolic pathways (DEMs = 358; 85.44%), glycerophospholipid metabolism (DEMs = 94; 22.43%), and glycerolipid metabolism (DEMs = 288; 68.74%) were the main enriched metabolic pathways (Figure 6(c)). According to the level of significance of enrichment, the main enriched pathways were glycerophospholipid metabolism (*P* = 2.83E − 02), steroid hormone biosynthesis (*P* = 3.61E − 02), steroid biosynthesis (*P* = 3.61E − 02), retinol metabolism (*P* = 4.05E − 02), and drug metabolism—other enzymes (*P* = 4.25E − 02) (Figure 6(d) and Table S7).

### 3.4. GSEA Analysis of Core Pathways

**Figure 7:**
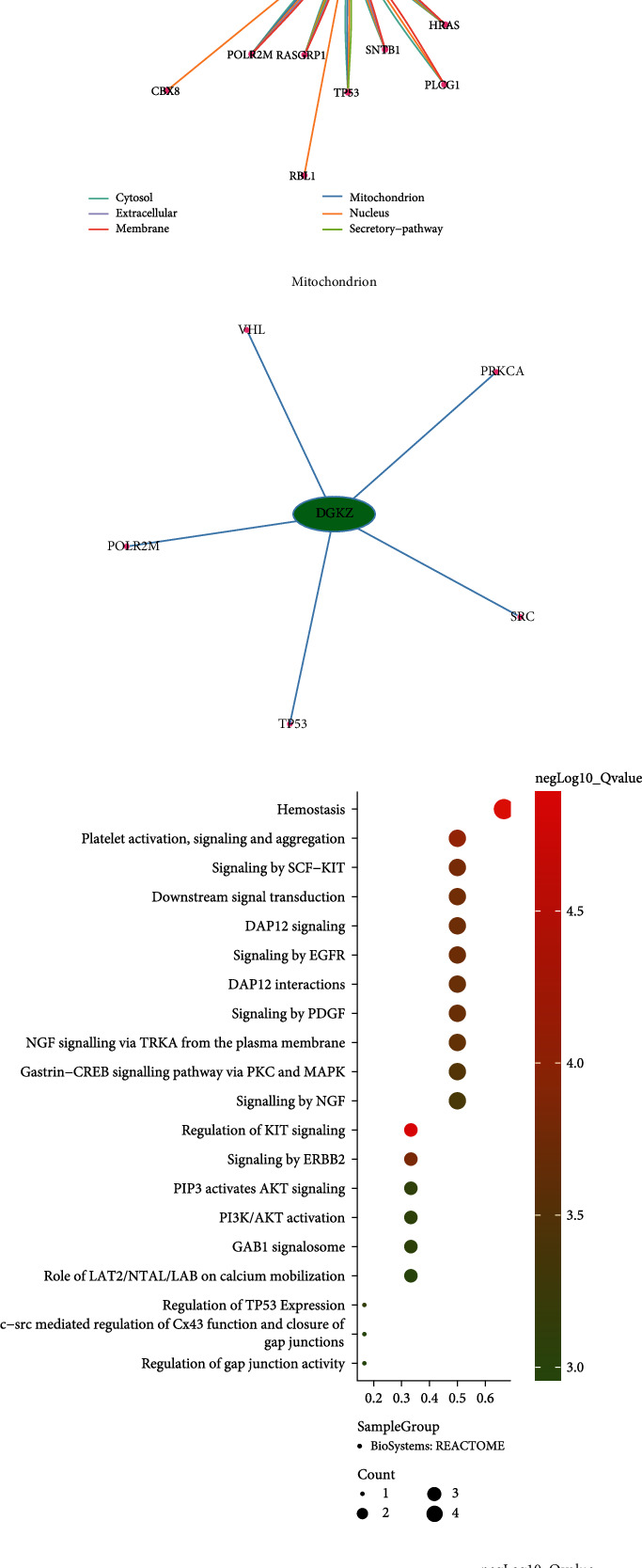
The key regulated pathway detection in immunotherapy-related myocarditis. (a, b) The map of glycerolipids and glycerophospholipids and glycerolipid metabolism was significantly detected. (c) The DGKZ-related cellular compartment-specific protein-protein interaction (ComPPI) was identified via the ComPPI database (URL: http://ComPPI.LinkGroup.hu). (d) The mitochondrion-related subnetwork. (e) DGKZ-related molecular function enrichment results involved in Reactome database (https://reactome.org). (f) The targeted drug molecule prediction in response to Comparative Toxicogenomics Database (CTD; https://ctdbase.org).

The above results suggested that glycerophospholipid metabolism may be an important regulatory pathway in immunotherapy-associated myocarditis. Therefore, we performed GSEA analysis on pseudotime-related DEGs in T cells, showing that glycerolipid metabolism (enrichment score = 0.676, normalized enrichment score = 1.48, and B-H-adjusted *P* value = 0.043) and glycerolipids and glycerophospholipids (enrichment score = 0.772, normalized enrichment score = 2.11, and B-H-adjusted *P* value = 0.002) were significantly enriched. Nfkbia, Dgkz, Pik3r1, and Dgka were among the main enriched genes (Figure 7(a) and 7(b) and Table S5). DGKZ was coenriched in the pathways, which may be an important regulatory target, and was therefore included in subsequent analysis.

### 3.5. DGKZ-Related Regulatory Mechanisms at the Mitochondrial Level

**Figure 8:**
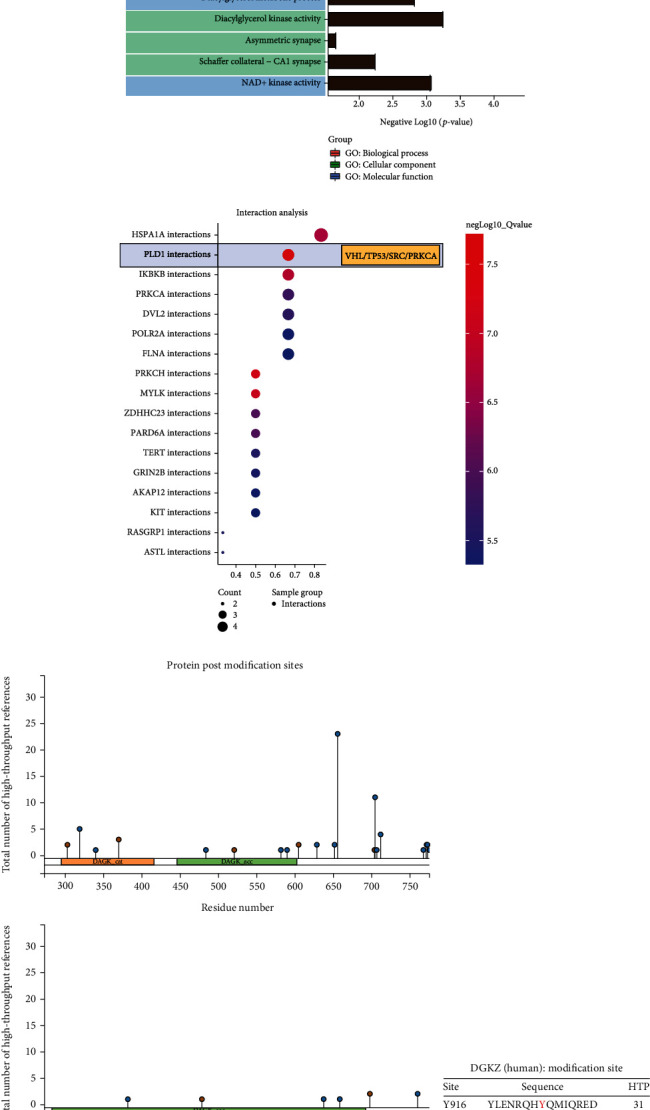
Functional enrichment analysis and protein post modification site prediction results for DGKZ-targeted biomarkers in mitochondrion level. (a) The GO: biological process, GO: cellular component, and GO: molecular function enrichment results for DGKZ-targeted biomarkers in the mitochondrion level. (b) The interaction analysis results. (c, d) The protein post modification site prediction of DGKZ protein.

Through the ComPPI analysis, DGKZ revealed interactions with upstream and downstream regulatory molecules at cytosol, extracellular, membrane, mitochondrion, nucleus, and secretory−pathway levels (Figure 7(c)). At the mitochondrial level, DGKZ had significant regulatory interactions with VHL, PRKCA, POLR2M, TP53, and SRC (Figure 7(d)). In the functional enrichment analysis of DGKZ and its mitochondrion level interacting molecules, we found that they were closely related to hemostasis activity (B-H-adjusted *P* value = 2.32E-05), platelet activation, signaling and aggregation (B-H-adjusted *P* value = 8.54E-05), signaling by SCF-KIT (B-H-adjusted *P* value = 1.45E-04), downstream signal transduction (B-H-adjusted *P* value = 1.65E-04), and regulation of KIT signaling (B-H-adjusted *P* value = 1.29E-05) (Figure 7(e)). According to drug–target interaction prediction, potential drugs targeting DGKZ-related mitochondrial regulation included riddelliine (B-H-adjusted *P* value = 2.80E-07), 1,3-dimethylthiourea (B-H-adjusted *P* value = 3.29E-07), monomethylarsonic acid (B-H-adjusted *P* value = 5.77E-07), azoxymethane (B-H-adjusted *P* value = 8.94E-07), and gallic acid (B-H-adjusted *P* value = 1.49E-06) (Figure 7(f)). In the GO analysis, significantly enriched molecular function terms included protein C-terminus binding (B-H-adjusted *P* value = 3.64E-05, gene count = 3), transferase activity, transferring phosphorus-containing groups (B-H-adjusted *P* value = 2.02E-04, gene count = 4), and DNA-binding transcription factor binding (B-H-adjusted *P* value = 4.41E-04, gene count = 3); significantly enriched cellular component terms included glutamatergic synapse (B-H-adjusted *P* value = 2.97E-04, gene count = 3), extrinsic component of cytoplasmic side of plasma membrane (B-H-adjusted *P* value = 3.90E-04, gene count = 2), and synapse (B-H-adjusted *P* value = 5.47E-04, gene count = 4); significantly enriched biological process terms included positive regulation of outer hair cell apoptotic process (B-H-adjusted *P* value = 6.99E-08, gene count = 2), regulation of outer hair cell apoptotic process (B-H-adjusted *P* value = 2.10E-07, gene count = 2), and outer hair cell apoptotic process (B-H-adjusted *P* value = 6.99E-07, gene count = 2) (Figure 8(a)). The interaction analysis revealed close associations of the genes with PLD1 interactions (B-H-adjusted *P* value = 1.93E-08; genes: VHL, TP53, SRC, and PRKCA), PRKCH interactions (B-H-adjusted *P* value =6.17E-08; genes: VHL, SRC, and PRKCA), and MYLK interactions (B-H-adjusted *P* value = 9.09E-08; genes: TP53, SRC, and PRKCA) (Figure 8(b)).

### 3.6. Identification of DGKZ Posttranslational Modification Sites

DGKZ demonstrated significant regulatory effects at the mitochondrial level, mainly regulating diacylglycerol kinase activity. Therefore, its posttranslational modification may affect metabolic regulation mediated by molecules. Based on the PhosphoSitePlus analysis, we revealed that DGKZ protein had two major domains of DAGK cat and DAGK acc, both of which contained phosphorylation and ubiquitination sites (Figure 8(c)). We found that the amino acids Y916, Y656, and S705 could be the main sites of modification, which were also the most reported in the literature (Figure 8(d)). Therefore, DGKZ may be the main regulatory protein of diacylglycerol kinase, and its activation or inhibition may be related to phosphorylation and ubiquitination, where the confirmed modification sites are at Y916, Y656, and S705.

## 4. Discussion

With the decline of cancer-specific mortality and the aging of the surviving population, more and more patients have both cancer and heart disease. It is reported that an incidence of up to 19% in all patients receiving immunotherapy, who received anticancer therapy, included an anthracycline, cyclophosphamide, trastuzumab, and anti-CTLA4 or anti-PD-1/PD-L1, with an incidence of cardiac insufficiency (with the New York Heart Association (NYHA)III or IV). And this number is expected to increase as more and more patients receive chemotherapy [5]. Myocarditis with cardiogenic shock early after chemotherapy is a rare and life-threatening condition. By contrast, in patients who have no cancer or do not receive chemotherapy, viruses are the most common cause of myocarditis. Chemotherapy-induced immunosuppression and toxicity to leukocytes increase the risk of opportunistic infection [6, 12]. Ammirati et al. proposed that immunosuppression and opportunistic viral infection postchemotherapy may be associated with the risk of cardiac injury and heart failure [35].

Evidence to support the potential for immunotherapy in cardiac remodeling has emerged. It has demonstrated cytotoxicity, with various nontherapeutic and potentially adverse effects. The cardiotoxicity varies according to the chemotherapeutic drug used. CAR T cell therapy includes FDA-approved tisagenlecleucel and axicabtagene ciloleucel, which may significantly correlate with acute toxicities [36, 37]. Their toxicities to the heart include arrhythmia, heart failure, and myocardial injury and are dose-dependent, with the risk rising exponentially as the cumulative dose increases, leading to irreversible structural changes and cell damage of cardiomyocytes in the late stage until the patients die. Cancer immunotherapy-induced lethal cytokine release syndrome can induce changes in the electrocardiogram, showing low QRS voltage and nonspecific T-wave or ST segment abnormalities [2, 3]. Immunotherapy-induced cardiotoxicity showed hemorrhagic myocardial necrosis with interstitial edema and fibrin deposition. The pathologic changes of cardiac fibroblasts may be significantly correlated with T cell activation. In the setting of cardiac remodeling, the immunotherapy-related cardiotoxicity potentially increased heart weight, pericardial effusion, subendocardial hemorrhage, and punctate epicardial lesions [3, 12].

This study found that T cell subsets are the most critical cell population for myocarditis development. Some scholars also suggested that CD4^+^ T cells are the main driver of heart-specific autoimmunity in myocarditis. Moreover, biopsies showed an accumulation of T cells, macrophages, and other inflammatory cells in close contact with injured cardiomyocytes in patients with acute myocarditis. Meanwhile, cellular metabolism and death pathways are important factors for cell fate determination. Once cellular homeostasis is disrupted, metabolites change as a result of cell functions. If their metabolism fails to maintain biosynthesis and homeostasis, the cells cannot keep normal functions and are prone to apoptosis. Based on this, via metabolome sequencing and core pathway screening analyses of clinical samples, we found that the glycerolipid metabolism pathway significantly changed during the progression of myocarditis postchemotherapy. Studies have shown that phospholipase A2 (PLA2) hydrolyzes membrane phosphatidylcholine (PC) to produce lysophosphatidylcholine (LysoPC) during glyceride metabolism, and PLA2 regulates various cellular events, including oxidative stress, differentiation, and inflammation [38]. Fang et al. pointed out that stimulatory factors induce inflammatory cardiac injury by triggering oxidative stress and the nuclear factor erythroid 2-related factor 2 (Nrf2) signaling pathway to interfere with a series of metabolic pathways including glycerolipid metabolism [39]. This metabolic pathway involves a variety of enzymatic lipid metabolites. For example, PC can be hydrolyzed by PLA2 to form LysoPC and polyunsaturated fatty acids (PUFAs). PUFAs are further oxidized by cyclooxygenase, lipoxygenase, and cytochrome P450 enzymes to form prostaglandins, thromboxane, prostacyclin, and hydroxylated fatty acids, which are involved in the pathological process of myocardial injury [40, 41]. Furthermore, studies have found that metabolic syndrome including glyceride metabolism, mitochondrial dysfunction, and oxidative stress plays an important role in the progression of heart failure. The study by Kienesberger et al. showed that the expression of some genes in lipid metabolism pathways changed significantly in a myocardial infarction model [42].

Via a gene set enrichment analysis (GSEA) and the ComPPI database, we found that diacylglycerol kinase zeta (Dgkz) was significantly enriched and widely acted on various organelles. Moreover, its level in mitochondria might be closely related to VHL, PRKCA, POLR2M, SRC, and TP53 [43, 44]. The protein encoded by the DGKZ gene belongs to the eukaryotic diacylglycerol kinase family. It catalyzes the conversion of diacylglycerol (DAG) to phosphatidic acid (PA) by participating in intracellular signal cascades and signal transduction [43]. DAG and PA are lipid molecules with unique biological functions. They usually act as metabolic intermediates, fundamental components of biological membranes, or second messengers [43, 44]. It is reported that DGKZ involved in the phosphatidylinositol signal pathway is closely related to pathway mechanisms in coronary artery diseases [45].

## 5. Conclusion

Immunotherapy-related myocarditis is among the most concerning diseases at present. We integrated single-cell and metabolomics data to systematically expound the immune and metabolic regulation networks in myocarditis and demonstrate the important role of glycerolipid metabolism. Based on protein structure, function, and subcellular organelle regulation, we also found that DGKZ protein may be an important regulatory gene of tumor-related autoimmune myocarditis and can be a potential therapeutic target.

## Figures and Tables

**Table 1:** The quantified expression of the top 50 expressed differential metabolite.

Formula	Compounds	VIP	Log2FC	Type
C8H15NO2	Tranexamic acid	1.23	20.73	Upregulated
C49H88O6	TG(14:1_16:1_16:1)	1.67	15.90	Upregulated
C58H106O6	TG(17:1_18:1_20:1)	1.60	15.37	Upregulated
C43H78O6	TG(8:0_16:1_16:1)	1.65	14.62	Upregulated
C58H98O6	TG(15:0_18:1_22:6)	1.64	14.60	Upregulated
C56H96O6	TG(17:1_18:2_18:3)	1.13	14.53	Upregulated
C54H94O6	TG(15:0_18:2_18:3)	1.60	14.47	Upregulated
C41H76O6	TG(10:0_14:0_14:1)	1.65	14.40	Upregulated
C49H86O6	TG(14:1_16:1_16:2)	1.66	14.09	Upregulated
C45H80O6	TG(8:0_16:0_18:3)	1.66	13.71	Upregulated
C53H90O6	TG(16:0_18:2_16:4)	1.63	13.64	Upregulated
C51H88O6	TG(14:1_16:1_18:3)	1.65	13.30	Upregulated
C55H92O6	TG(16:1_16:1_20:5)	1.65	12.93	Upregulated
C54H94O6	TG(15:0_16:1_20:4)	1.13	12.65	Upregulated
C45H80O6	TG(10:0_14:0_18:3)	1.67	12.54	Upregulated
C20H34O5	5-iPF2_-VI [(8_)-5,9,11-trihydroxy-prosta-6E,14Z-dien-1-oic acid]	1.67	12.14	Upregulated
C61H112O6	TG(16:1_18:1_24:1)	1.13	11.44	Upregulated
C37H64O5	DG(16:1_18:3)	1.64	11.38	Upregulated
C26H45NO7S	Taurocholic acid	1.23	11.17	Upregulated
C20H34O6	6 keto-PGF1_ [6-oxo-9_,11_,15S-trihydroxy-prost-13E-en-1-oic acid]	1.63	11.01	Upregulated
C21H28O5	Aldosterone	1.65	10.86	Upregulated
C12H28N4O	N1-Acetylspermine	1.67	10.49	Upregulated
C54H94O6	TG(16:1_17:1_18:3)	1.43	7.43	Upregulated
C55H94O6	TG(16:0_16:1_20:5)	1.36	7.43	Upregulated
C53H92O6	TG(12:0_16:0_22:5)	1.42	7.28	Upregulated
C55H106O6	TG(14:0_18:0_20:0)	1.16	7.18	Upregulated
C51H90O6	TG(14:0_16:1_18:3)	1.42	6.79	Upregulated
C52H100O6	TG(16:0_16:0_17:0)	1.23	6.75	Upregulated
C56H104O6	TG(16:0_18:1_19:1)	1.36	6.68	Upregulated
C53H92O6	TG(16:1_16:1_18:3)	1.31	6.64	Upregulated
C56H106O6	TG(17:0_17:0_19:1)	1.20	6.63	Upregulated
C58H102O6	TG(19:1_18:2_18:2)	1.24	6.60	Upregulated
C56H102O6	TG(17:1_18:1_18:1)	1.40	6.57	Upregulated
C54H98O6	TG(16:1_17:1_18:1)	1.22	6.47	Upregulated
C56H100O6	TG(15:0_19:2_19:2)	1.32	6.41	Upregulated
C59H100O6	TG(16:1_16:1_24:5)	1.34	6.39	Upregulated
C57H98O6	TG(16:0_16:1_22:5)	1.34	6.37	Upregulated
C54H98O6	TG(16:0_17:1_18:2)	1.27	6.37	Upregulated
C56H106O6	TG(17:0_18:0_18:1)	1.18	6.36	Upregulated
C59H110O6	TG(16:0_18:1_22:1)	1.36	6.32	Upregulated
C57H108O6	TG(16:0_18:0_20:1)	1.27	6.31	Upregulated
C55H106O6	TG(16:0_18:0_18:0)	1.16	6.26	Upregulated
C56H98O6	TG(17:1_18:2_18:2)	1.27	6.26	Upregulated
C43H82O6	TG(8:0_14:0_18:0)	1.27	6.23	Upregulated
C58H104O6	TG(17:0_19:2_19:2)	1.23	6.21	Upregulated
C56H100O6	TG(17:1_18:1_18:2)	1.32	6.20	Upregulated
C61H112O6	TG(18:1_20:1_20:1)	1.20	6.20	Upregulated
C20H36O6	8,8a-deoxy oleane	1.25	-5.36	Downregulated
C16H26O3	Tetranor-12(R)-HETE	1.67	-11.06	Downregulated
C42H62O16	Glycyrrhizinate	1.21	-14.18	Downregulated

## Data Availability

The data that support the findings of this study are available from the corresponding authors upon reasonable request.

## Supplementary Materials

Supplementary 1 Table S1: the differentially expressed genes of annotation-cell clusters in CD45^+^ single-cell atlas of immunotherapy-related myocarditis.

Supplementary 2 Table S2: the pseudotime trajectory- (PTT-) related differentially expressed genes in T cell subpopulation.

Supplementary 3 Table S3: the Gene Ontology (GO) terms enrichment analysis for PTT-related DEGs of T cell subpopulation.

Supplementary 4 Table S4: the Kyoto Encyclopedia of Genes and Genomes (KEGG) pathway enrichment analysis for PTT-related DEGs of T cell subpopulation.

Supplementary 5 Table S5: the Gene Set Enrichment Analysis (GSEA) map enrichment results for PTT-related DEGs of the T cell subpopulation.

Supplementary 6 Table S6: the differentially expressed metabolite results of immunotherapy-related myocarditis via UPLC-MS/MS detection platform.

Supplementary 7 Table S7: The KEGG functional enrichment results for differentially expressed metabolites.
