# Supplementary material for: The Regulation Network of Glycerolipid Metabolism as Coregulators of Immunotherapy-Related Myocarditis
Source: Cardiovasc Ther. 2023 Jun 21;2023:8774971. doi: 10.1155/2023/8774971 (PMC10307211; doi:10.1155/2023/8774971)
Supplement: Supplementary 1 — Table S1: the differentially expressed genes of annotation-cell clusters in CD45+ single-cell atlas of immunotherapy-related myocarditis. [file 8774971.f1.pdf]

p_val	avg_log2FC	pct.1	pct.2	p_val_adj	cluster	gene
0	2.70127338	0.874	0.137	0	T cells	Cd3e
0	2.65888103	0.899	0.171	0	T cells	Cd3g
0	2.61877584	0.704	0.136	0	T cells	Cd8b1
0	2.53100975	0.858	0.145	0	T cells	Cd3d
0	2.35433966	0.64	0.12	0	T cells	Cd8a
0	2.29923125	0.854	0.218	0	T cells	Ms4a4b
0	2.16582599	0.792	0.273	0	T cells	Nkg7
0	2.16476056	0.752	0.13	0	T cells	Lat
0	2.06853317	0.549	0.114	0	T cells	Pdcd1
0	2.05081972	0.476	0.097	0	T cells	Ctla4
0	1.93059767	0.551	0.103	0	T cells	Icos
0	1.9282886	0.668	0.123	0	T cells	Thy1
0	1.80536229	0.527	0.127	0	T cells	Tigit
0	1.79439942	0.548	0.093	0	T cells	Itk
0	1.72900147	0.642	0.137	0	T cells	Lck
0	1.63843067	0.619	0.181	0	T cells	Sh2d2a
0	1.63720695	0.506	0.089	0	T cells	Cd28
0	1.49111725	0.473	0.092	0	T cells	Cd27
0	1.43613713	0.53	0.102	0	T cells	Skap1
0	1.39169424	0.455	0.086	0	T cells	Cd247
0	1.35115002	0.597	0.161	0	T cells	Ctsw
0	-1.6093344	0.879	0.966	0	T cells	Ftl1
0	-1.7129101	0.918	0.98	0	T cells	Fth1
0	-1.7850353	0.062	0.502	0	T cells	Lyn
0	-1.9068328	0.074	0.508	0	T cells	Unc93b1
0	-2.0911416	0.058	0.484	0	T cells	Spi1
0	-2.1156899	0.522	0.779	0	T cells	Gpx1
0	-2.1656671	0.389	0.749	0	T cells	Zfp36
0	-3.1595077	0.152	0.694	0	T cells	H2-Ab1
0	-3.1650783	0.13	0.596	0	T cells	Fcer1g
0	-3.2739869	0.171	0.699	0	T cells	H2-Eb1
0	-3.5753473	0.174	0.705	0	T cells	H2-Aa
0	-3.6035632	0.106	0.634	0	T cells	Tyrobp
0	-3.9889944	0.333	0.826	0	T cells	Cd74
1.05E-304	0.79318901	0.981	0.897	1.49E-300	T cells	Rpsa
1.14E-302	-1.8116726	0.055	0.464	1.61E-298	T cells	Ctsh
8.32E-301	-2.1425784	0.048	0.455	1.18E-296	T cells	Cd83
1.30E-290	1.92077245	0.442	0.093	1.84E-286	T cells	Lag3
4.96E-290	-1.191875	0.803	0.896	7.02E-286	T cells	H3f3a
1.64E-286	-1.7902591	0.056	0.453	2.32E-282	T cells	Ly86
4.91E-286	2.19569897	0.76	0.437	6.95E-282	T cells	Ccl5
2.99E-285	-1.8054352	0.073	0.474	4.24E-281	T cells	Napsa
5.48E-285	-2.8333906	0.06	0.452	7.76E-281	T cells	Alox5ap
6.60E-282	1.57655764	0.517	0.143	9.34E-278	T cells	Cst7
1.12E-281	1.33520347	0.791	0.439	1.59E-277	T cells	H2-Q7
3.04E-277	-3.1033558	0.069	0.453	4.30E-273	T cells	Ifitm2
2.09E-274	-4.1057774	0.11	0.491	2.96E-270	T cells	Lyz2

2.21E-274	-1.619268	0.025	0.399	3.13E-270	T cells	Syk
5.54E-271	1.44803838	0.481	0.125	7.85E-267	T cells	Tnfrsf18
2.16E-266	-3.5810168	0.018	0.379	3.05E-262	T cells	Cd79a
1.30E-263	-1.8525543	0.03	0.394	1.83E-259	T cells	Ncf2
7.03E-263	0.81853933	0.962	0.911	9.96E-259	T cells	Rplp0
2.44E-257	1.68203769	0.42	0.092	3.46E-253	T cells	Cxcr6
2.60E-254	1.52973864	0.732	0.406	3.68E-250	T cells	Lgals1
2.04E-253	1.28305649	0.813	0.424	2.89E-249	T cells	AW112010
2.12E-249	-3.1267975	0.089	0.451	3.00E-245	T cells	Ifitm3
2.00E-246	1.32881052	0.375	0.07	2.83E-242	T cells	Tox
2.09E-246	-2.2187717	0.019	0.361	2.95E-242	T cells	H2-DMb2
9.16E-246	1.33648356	0.325	0.045	1.30E-241	T cells	Cd5
1.09E-243	-1.8604778	0.019	0.358	1.54E-239	T cells	Mef2c
2.12E-243	-2.2654484	0.379	0.658	3.00E-239	T cells	Ctss
6.21E-241	-2.5631798	0.011	0.342	8.79E-237	T cells	Ebf1
1.30E-236	0.78272219	0.956	0.888	1.84E-232	T cells	Rps15a
1.85E-235	-2.2720331	0.054	0.402	2.62E-231	T cells	Plaur
2.21E-235	-4.1356873	0.055	0.397	3.12E-231	T cells	Il1b
1.43E-228	-3.106064	0.05	0.383	2.02E-224	T cells	Ccl6
2.05E-227	1.56721408	0.524	0.179	2.91E-223	T cells	Klrd1
4.44E-226	-1.0710541	0.773	0.856	6.28E-222	T cells	Ly6e
3.55E-221	-3.1511698	0.086	0.421	5.03E-217	T cells	Apoe
2.99E-219	1.13123418	0.678	0.331	4.23E-215	T cells	Hcst
1.28E-218	1.127166	0.706	0.344	1.81E-214	T cells	Cd2
6.26E-218	-1.5888369	0.157	0.493	8.87E-214	T cells	H2-DMa
1.24E-215	1.16831698	0.544	0.199	1.75E-211	T cells	Gimap3
5.08E-214	1.07574607	0.558	0.208	7.19E-210	T cells	Gimap4
1.66E-212	-1.5241131	0.055	0.378	2.35E-208	T cells	Tpd52
4.34E-212	1.00257227	0.649	0.293	6.14E-208	T cells	1-Sep
5.10E-212	-2.2510858	0.363	0.63	7.22E-208	T cells	Psap
7.70E-211	1.12852651	0.45	0.138	1.09E-206	T cells	Zgpat
1.88E-210	-2.7346666	0.019	0.325	2.66E-206	T cells	Ly6d
2.04E-210	-1.5360709	0.024	0.33	2.88E-206	T cells	Cyp4f18
1.74E-209	-1.6030412	0.152	0.482	2.46E-205	T cells	Ctsz
1.10E-208	-2.037182	0.174	0.498	1.56E-204	T cells	Ifi30
3.16E-208	1.09211197	0.736	0.407	4.47E-204	T cells	Ptprcap
1.02E-207	1.04585272	0.468	0.14	1.45E-203	T cells	Il2rb
6.37E-205	-2.2946277	0.051	0.361	9.01E-201	T cells	Cd79b
2.68E-204	-1.6915819	0.077	0.396	3.79E-200	T cells	Cybb
1.32E-202	-2.0697847	0.421	0.658	1.87E-198	T cells	Cst3
1.62E-201	-2.7134583	0.048	0.357	2.30E-197	T cells	Wfdc17
2.01E-201	1.08467025	0.337	0.071	2.84E-197	T cells	Zap70
7.77E-201	1.13130212	0.403	0.109	1.10E-196	T cells	Rnf125
2.99E-200	1.19761842	0.457	0.153	4.23E-196	T cells	Ptpn22
1.17E-199	-1.4702946	0.044	0.347	1.65E-195	T cells	Pld4
3.53E-197	-1.2456562	0.021	0.31	5.00E-193	T cells	Dok3
1.40E-195	-1.6644561	0.051	0.357	1.99E-191	T cells	H2-DMb1
1.09E-194	-1.2042757	0.019	0.306	1.54E-190	T cells	Aldh2

4.04E-193	-2.0878228	0.121	0.441	5.72E-189	T cells	Atf3
9.05E-191	1.08113161	0.273	0.042	1.28E-186	T cells	Cd6
1.31E-190	1.11331862	0.425	0.128	1.85E-186	T cells	Prkca
1.84E-189	1.20046505	0.434	0.142	2.61E-185	T cells	Prkch
4.27E-187	1.11546725	0.418	0.129	6.04E-183	T cells	Gimap7
1.02E-186	0.67091345	0.96	0.878	1.44E-182	T cells	Ppia
2.98E-186	0.82597184	0.889	0.745	4.22E-182	T cells	Rac2
3.98E-185	1.17876208	0.551	0.252	5.64E-181	T cells	Saraf
8.55E-184	-1.8582546	0.008	0.273	1.21E-179	T cells	Fcmr
4.73E-182	-1.7254793	0.049	0.332	6.70E-178	T cells	Fcgr3
1.32E-181	1.20817005	0.399	0.125	1.87E-177	T cells	Dut
7.50E-181	0.88884413	0.267	0.043	1.06E-176	T cells	Klk8
8.63E-180	-1.7379418	0.034	0.309	1.22E-175	T cells	H2-Ob
3.07E-176	-1.4749631	0.293	0.573	4.34E-172	T cells	Serp1
1.81E-175	1.09943189	0.684	0.411	2.57E-171	T cells	Ndfip1
2.20E-175	-1.2681191	0.06	0.346	3.12E-171	T cells	Nrros
8.43E-174	1.28705394	0.576	0.281	1.19E-169	T cells	S100a4
7.23E-173	0.6760617	0.965	0.887	1.02E-168	T cells	H2-K1
3.19E-172	-1.7222099	0.01	0.263	4.52E-168	T cells	Ms4a1
1.03E-171	0.52795668	0.958	0.883	1.46E-167	T cells	Rpl11
1.15E-170	0.90946887	0.232	0.03	1.63E-166	T cells	Bcl11b
9.74E-170	0.55087516	0.961	0.946	1.38E-165	T cells	Tpt1
5.58E-169	-1.5189457	0.512	0.728	7.91E-165	T cells	Fos
2.19E-166	-1.9205357	0.085	0.359	3.10E-162	T cells	Lst1
1.21E-162	-1.4202321	0.029	0.287	1.71E-158	T cells	Cd24a
2.20E-161	0.92990396	0.558	0.273	3.12E-157	T cells	Leprotl1
3.12E-161	-1.0907994	0.044	0.305	4.41E-157	T cells	Pkig
4.06E-161	-2.1318616	0.03	0.281	5.75E-157	T cells	Cd14
6.46E-160	1.10406998	0.201	0.02	9.14E-156	T cells	Tcf7
1.68E-157	-1.2832177	0.286	0.563	2.38E-153	T cells	Ier5
2.71E-157	-1.6177744	0.127	0.407	3.83E-153	T cells	Klf4
1.64E-156	-1.6483278	0.024	0.266	2.32E-152	T cells	Pla2g7
2.77E-156	-1.4822657	0.041	0.293	3.92E-152	T cells	Fcgr2b
6.25E-156	1.14091977	0.263	0.052	8.85E-152	T cells	Izumo1r
4.39E-155	-1.9796373	0.045	0.295	6.21E-151	T cells	Tgfbi
1.36E-154	-1.4184076	0.007	0.237	1.92E-150	T cells	Cd19
3.40E-152	1.13713005	0.213	0.029	4.82E-148	T cells	Lef1
3.95E-152	-1.2430344	0.029	0.268	5.60E-148	T cells	Pirb
9.06E-152	-2.1018524	0.445	0.645	1.28E-147	T cells	Cebpb
3.76E-151	-1.2917828	0.029	0.269	5.32E-147	T cells	Plbd1
7.07E-151	-1.3690782	0.025	0.262	1.00E-146	T cells	Mpeg1
1.28E-150	0.83569743	0.275	0.061	1.81E-146	T cells	Sh2d1a
1.36E-150	-1.6177531	0.05	0.298	1.93E-146	T cells	Lgmn
2.72E-148	1.6973942	0.298	0.075	3.86E-144	T cells	Tnfrsf4
7.57E-148	1.42463964	0.245	0.048	1.07E-143	T cells	Il7r
1.29E-146	-1.419874	0.119	0.39	1.83E-142	T cells	Rhob
4.00E-146	0.95455386	0.291	0.073	5.66E-142	T cells	Inpp4b
3.35E-145	-1.2874565	0.007	0.224	4.75E-141	T cells	Bank1

4.20E-144	1.11055415	0.243	0.048	5.94E-140	T cells	Rgs16
4.93E-144	1.22594076	0.403	0.143	6.98E-140	T cells	Gzmb
1.24E-143	-2.2696416	0.019	0.242	1.75E-139	T cells	Hp
1.40E-142	-0.9474958	0.021	0.246	1.98E-138	T cells	Plcg2
1.22E-141	-1.704963	0.072	0.315	1.72E-137	T cells	Ms4a6c
1.23E-141	0.55363783	0.96	0.898	1.75E-137	T cells	Rps2
2.91E-141	-1.2208817	0.471	0.737	4.12E-137	T cells	Klf2
5.53E-141	-1.1435918	0.041	0.275	7.82E-137	T cells	Csf2ra
5.93E-141	1.26368537	0.247	0.052	8.40E-137	T cells	Gzmk
9.75E-141	0.85154016	0.531	0.254	1.38E-136	T cells	Ms4a6b
5.39E-139	1.21333891	0.595	0.326	7.63E-135	T cells	Nr4a2
5.98E-139	0.96664185	0.775	0.563	8.46E-135	T cells	S100a10
1.27E-138	1.4794575	0.336	0.103	1.80E-134	T cells	Ifng
2.45E-138	-1.4830534	0.036	0.262	3.47E-134	T cells	Csf1r
4.54E-138	0.67009324	0.19	0.024	6.42E-134	T cells	Camk4
1.39E-137	-3.4324692	0.039	0.266	1.96E-133	T cells	Cxcl2
2.61E-137	0.50250119	0.957	0.9	3.69E-133	T cells	Rpl18
2.43E-136	-1.3058171	0.138	0.394	3.44E-132	T cells	Nfe2l2
5.12E-136	-1.5149565	0.07	0.312	7.25E-132	T cells	Marcksl1
1.58E-135	1.211837	0.301	0.087	2.24E-131	T cells	Tnfrsf9
4.39E-135	-1.1866216	0.051	0.285	6.22E-131	T cells	Kctd12
1.84E-134	-0.9433831	0.031	0.252	2.61E-130	T cells	Irf5
4.13E-134	0.79496711	0.271	0.068	5.84E-130	T cells	Mllt3
7.43E-134	-2.3719473	0.18	0.419	1.05E-129	T cells	Msrb1
1.40E-131	1.04484994	0.579	0.32	1.99E-127	T cells	Id2
4.03E-131	-1.0823203	0.037	0.258	5.71E-127	T cells	Themis2
5.17E-131	-1.6431383	0.465	0.658	7.32E-127	T cells	Nr4a1
6.38E-131	-1.3286723	0.005	0.202	9.03E-127	T cells	Fcer2a
3.29E-130	-1.4008866	0.155	0.411	4.66E-126	T cells	Irf8
4.89E-130	0.54235502	0.933	0.855	6.92E-126	T cells	Rpl13a
8.75E-130	-0.9773106	0.032	0.248	1.24E-125	T cells	Rassf4
9.60E-130	0.82906634	0.23	0.047	1.36E-125	T cells	Cd226
2.68E-129	-0.9736212	0.025	0.236	3.80E-125	T cells	Hck
3.35E-129	0.91541677	0.331	0.111	4.74E-125	T cells	Rinl
3.78E-129	0.63796074	0.94	0.886	5.35E-125	T cells	Rplp1
8.92E-129	0.68889676	0.173	0.02	1.26E-124	T cells	Themis
3.38E-128	0.82809971	0.41	0.162	4.79E-124	T cells	Gimap5
8.60E-127	-1.0187103	0.036	0.251	1.22E-122	T cells	Lrrc25
1.55E-126	-1.3894998	0.03	0.242	2.20E-122	T cells	Anxa1
2.19E-125	-1.1050883	0.256	0.497	3.10E-121	T cells	Bri3
3.25E-125	1.08442211	0.723	0.534	4.61E-121	T cells	Arl6ip1
1.70E-124	-0.998679	0.009	0.2	2.40E-120	T cells	Blnk
5.14E-124	-1.9702305	0.015	0.211	7.27E-120	T cells	Tnfaip2
7.32E-124	-1.3691072	0.087	0.312	1.04E-119	T cells	Cd68
9.26E-124	-1.1966304	0.051	0.268	1.31E-119	T cells	Cd72
1.33E-123	0.47726654	0.937	0.888	1.89E-119	T cells	Rps3
7.77E-123	-0.9699353	0.015	0.21	1.10E-118	T cells	Cd300a
3.72E-122	0.54935986	0.905	0.81	5.26E-118	T cells	Rpl3

9.20E-122	0.93366064	0.297	0.094	1.30E-117	T cells	Gtf2i
9.31E-122	-1.2275118	0.325	0.552	1.32E-117	T cells	Mcl1
3.04E-121	-1.7500667	0.195	0.434	4.30E-117	T cells	Plac8
3.28E-121	0.70092784	0.752	0.562	4.64E-117	T cells	Eif3h
4.43E-121	-1.0755792	0.069	0.293	6.27E-117	T cells	Ms4a4c
6.19E-121	0.79170908	0.477	0.217	8.76E-117	T cells	Gimap1
2.21E-119	0.64976575	0.226	0.051	3.13E-115	T cells	Lime1
3.31E-119	-1.1369539	0.067	0.288	4.69E-115	T cells	Cd81
3.57E-119	-1.0080637	0.022	0.217	5.05E-115	T cells	Clec4a3
1.16E-118	0.7075816	0.235	0.055	1.64E-114	T cells	Sla2
1.61E-118	-0.993826	0.093	0.322	2.28E-114	T cells	Prkcd
1.66E-118	-1.6492501	0.011	0.197	2.36E-114	T cells	Clec4e
1.27E-117	-0.8205663	0.037	0.242	1.79E-113	T cells	Rnf130
2.26E-117	-1.9843978	0.009	0.191	3.20E-113	T cells	Trem1
3.26E-117	-1.2816537	0.243	0.49	4.62E-113	T cells	Neat1
9.52E-117	-1.3086825	0.012	0.195	1.35E-112	T cells	C5ar1
1.38E-116	0.9051439	0.342	0.12	1.96E-112	T cells	Ctla2a
2.94E-116	0.65208001	0.951	0.905	4.16E-112	T cells	Gapdh
4.59E-116	-1.0926159	0.329	0.563	6.50E-112	T cells	Pde4b
9.81E-116	-1.1285068	0.006	0.183	1.39E-111	T cells	Fcrla
7.15E-115	-0.9432329	0.352	0.582	1.01E-110	T cells	Serinc3
1.42E-114	-1.2357713	0.014	0.198	2.02E-110	T cells	Mgst1
2.53E-114	0.67908729	0.208	0.043	3.58E-110	T cells	Prkcq
4.08E-114	0.45660801	0.961	0.911	5.78E-110	T cells	Rps3a1
3.76E-113	0.94846032	0.212	0.047	5.32E-109	T cells	Ccr8
3.79E-113	0.90485548	0.38	0.161	5.36E-109	T cells	Smc4
5.99E-113	-1.092275	0.008	0.184	8.47E-109	T cells	Blk
2.97E-112	-0.9618657	0.105	0.333	4.20E-108	T cells	AB124611
1.25E-111	1.09932359	0.218	0.052	1.77E-107	T cells	Ikzf2
1.42E-111	-1.0218574	0.038	0.234	2.01E-107	T cells	Marcks
8.26E-111	-1.0898307	0.012	0.188	1.17E-106	T cells	Gda
8.31E-111	-0.941117	0.023	0.207	1.18E-106	T cells	Sirpa
1.96E-110	0.70833878	0.236	0.062	2.78E-106	T cells	Mgst2
2.58E-109	-0.7809826	0.023	0.206	3.65E-105	T cells	BC028528
4.48E-109	-1.0391666	0.004	0.17	6.34E-105	T cells	Scd1
1.51E-108	-0.9644704	0.011	0.184	2.14E-104	T cells	Mzb1
4.49E-108	-1.4075782	0.422	0.591	6.36E-104	T cells	Sat1
5.09E-108	-0.8188575	0.023	0.204	7.21E-104	T cells	Rnase6
8.80E-107	-1.0546701	0.223	0.461	1.25E-102	T cells	Rel
1.13E-106	-1.2429614	0.018	0.193	1.61E-102	T cells	Cd300ld
1.30E-106	-1.2148193	0.175	0.404	1.85E-102	T cells	Cxcr4
1.57E-106	-1.0205968	0.142	0.374	2.22E-102	T cells	Kdm6b
1.95E-105	-2.8175174	0.017	0.19	2.76E-101	T cells	Slpi
2.18E-105	0.68992017	0.18	0.034	3.09E-101	T cells	Pde4d
3.01E-105	-0.8209432	0.043	0.234	4.26E-101	T cells	Blvrb
3.45E-105	-1.2370778	0.041	0.229	4.89E-101	T cells	Fcgr4
3.86E-105	-0.9358522	0.017	0.19	5.46E-101	T cells	Sirpb1c
6.99E-105	0.83459349	0.651	0.426	9.90E-101	T cells	Mbnl1

1.11E-104	0.43900866	0.944	0.902	1.57E-100	T cells	Rpl9-ps6
1.35E-104	0.83994498	0.301	0.108	1.91E-100	T cells	Pkp3
1.95E-104	0.44982419	0.981	0.97	2.76E-100	T cells	Ubb
2.44E-104	0.82042575	0.323	0.126	3.45E-100	T cells	Acot7
2.28E-103	-1.5021543	0.087	0.289	3.23E-99	T cells	Grn
4.46E-103	-0.409325	0.012	0.177	6.31E-99	T cells	1-Mar
7.65E-103	-0.9672511	0.076	0.278	1.08E-98	T cells	Gns
9.62E-103	-0.954097	0.007	0.169	1.36E-98	T cells	Fam43a
1.42E-102	0.98027035	0.323	0.129	2.01E-98	T cells	Rbpj
2.28E-102	0.78306232	0.541	0.321	3.23E-98	T cells	S100a13
4.30E-102	-1.3979239	0.109	0.316	6.09E-98	T cells	Gngt2
9.56E-102	-1.1921777	0.194	0.404	1.35E-97	T cells	Lamp1
1.69E-101	-0.9251745	0.031	0.21	2.39E-97	T cells	Rasgef1b
1.93E-101	0.9074606	0.425	0.203	2.73E-97	T cells	Il21r
2.36E-101	-0.8993682	0.019	0.188	3.34E-97	T cells	Fgr
3.16E-101	-0.8939491	0.019	0.188	4.47E-97	T cells	Nfam1
6.53E-101	-1.2405169	0.055	0.247	9.24E-97	T cells	Id3
2.29E-100	0.50202531	0.923	0.851	3.24E-96	T cells	Oaz1
5.63E-100	-1.246479	0.01	0.169	7.97E-96	T cells	Nlrp3
1.02E-99	-1.046975	0.369	0.566	1.44E-95	T cells	Samhd1
2.01E-99	0.57864832	0.827	0.723	2.84E-95	T cells	Eef1b2
2.07E-99	-0.9791103	0.029	0.203	2.93E-95	T cells	Sdc4
3.82E-99	-1.4444058	0.531	0.678	5.40E-95	T cells	Dusp1
4.85E-99	0.76630293	0.643	0.381	6.87E-95	T cells	Ltb
8.07E-99	-1.2227355	0.026	0.196	1.14E-94	T cells	Tgm2
1.22E-98	-0.8454023	0.034	0.21	1.73E-94	T cells	Clec12a
3.76E-98	-1.0256646	0.39	0.604	5.32E-94	T cells	Litaf
3.87E-98	-0.93999	0.014	0.174	5.48E-94	T cells	Pilra
4.78E-98	0.44302946	0.913	0.848	6.76E-94	T cells	Rpl24
7.20E-98	-0.8492827	0.015	0.177	1.02E-93	T cells	Csf2rb
2.17E-97	0.61341487	0.171	0.034	3.07E-93	T cells	Sdcbp2
5.46E-97	0.77667024	0.444	0.226	7.72E-93	T cells	Ifi27
8.74E-97	-0.7915117	0.018	0.18	1.24E-92	T cells	Clec4a1
1.26E-96	-1.2372065	0.063	0.252	1.78E-92	T cells	Trib1
4.98E-96	0.43731233	0.894	0.841	7.05E-92	T cells	Rpl27
1.84E-95	-0.7125476	0.038	0.213	2.60E-91	T cells	Scpep1
4.00E-95	-1.4688313	0.008	0.159	5.66E-91	T cells	Clec4d
6.25E-95	-1.0676871	0.142	0.356	8.85E-91	T cells	Sell
6.56E-95	-1.2379746	0.014	0.17	9.28E-91	T cells	Ifitm6
6.96E-95	0.70029129	0.18	0.039	9.86E-91	T cells	Atxn1
2.48E-94	-0.9239543	0.12	0.323	3.51E-90	T cells	Sp140
3.50E-94	-0.8968872	0.103	0.306	4.95E-90	T cells	Zeb2
5.53E-94	-0.9099968	0.04	0.214	7.82E-90	T cells	Myadm
6.67E-94	-0.8487374	0.022	0.183	9.44E-90	T cells	Slc11a1
7.75E-94	-1.5276506	0.011	0.164	1.10E-89	T cells	Csf3r
1.13E-93	0.77924374	0.199	0.051	1.59E-89	T cells	Cxcr3
1.18E-93	-0.8391705	0.05	0.228	1.67E-89	T cells	Skap2
1.50E-93	0.59898752	0.206	0.055	2.12E-89	T cells	Smco4

1.62E-93	-0.8335144	0.056	0.24	2.30E-89	T cells	Ncf1
2.16E-93	0.70150807	0.628	0.42	3.05E-89	T cells	Gstp1
6.19E-93	1.00782944	0.551	0.354	8.76E-89	T cells	Sdf4
6.44E-93	-1.1645453	0.043	0.218	9.11E-89	T cells	Plk2
1.72E-92	-0.8829503	0.188	0.402	2.43E-88	T cells	Ptpn6
3.02E-92	0.57820978	0.162	0.031	4.28E-88	T cells	Cd96
4.26E-92	-0.995506	0.046	0.22	6.02E-88	T cells	App
9.61E-92	0.7949307	0.459	0.241	1.36E-87	T cells	Mrps6
1.00E-91	-0.8464787	0.013	0.165	1.42E-87	T cells	Emilin2
1.15E-91	0.71288209	0.352	0.153	1.63E-87	T cells	D16Ertd472e
1.49E-91	0.65197456	0.422	0.207	2.10E-87	T cells	Tes
5.28E-91	0.52293492	0.16	0.031	7.47E-87	T cells	Ubash3a
5.54E-91	-0.8033592	0.055	0.237	7.85E-87	T cells	Cerk
5.10E-90	-1.4768451	0.049	0.221	7.21E-86	T cells	Mafb
9.40E-90	0.68575711	0.432	0.226	1.33E-85	T cells	Slc25a4
1.22E-89	0.80840108	0.587	0.363	1.72E-85	T cells	Tnfaip3
1.74E-89	0.52618161	0.888	0.797	2.46E-85	T cells	Rps18
4.10E-89	-0.7840825	0.018	0.17	5.81E-85	T cells	Itgam
4.13E-89	0.68152089	0.629	0.428	5.85E-85	T cells	Abracl
5.03E-89	-0.8217161	0.007	0.148	7.12E-85	T cells	Siglecg
6.87E-89	0.4170406	0.946	0.907	9.72E-85	T cells	Rpl30
9.42E-89	0.67409055	0.333	0.145	1.33E-84	T cells	Srpk1
1.10E-88	0.86425562	0.175	0.04	1.56E-84	T cells	Nrn1
1.63E-88	-0.6974996	0.02	0.172	2.30E-84	T cells	Cd40
4.88E-88	0.49038987	0.858	0.762	6.91E-84	T cells	Naca
2.35E-87	-0.8516621	0.095	0.286	3.33E-83	T cells	Trim25
2.57E-87	-0.9372182	0.016	0.163	3.64E-83	T cells	Serpinb1a
2.71E-87	-1.2229443	0.076	0.251	3.84E-83	T cells	Cd55
8.67E-87	0.50624003	0.849	0.739	1.23E-82	T cells	Rps6
1.84E-86	-0.9962113	0.338	0.535	2.60E-82	T cells	Pim1
2.25E-86	0.67027185	0.183	0.045	3.19E-82	T cells	Itm2a
2.87E-86	-0.6049016	0.008	0.148	4.07E-82	T cells	Btk
3.81E-86	-0.9790006	0.015	0.161	5.40E-82	T cells	Cd300lf
4.20E-86	0.57167755	0.206	0.059	5.94E-82	T cells	Fam189b
6.18E-86	-0.6495532	0.019	0.169	8.75E-82	T cells	Fes
1.06E-85	-0.7990722	0.014	0.158	1.51E-81	T cells	Lrp1
3.59E-85	-1.0217755	0.264	0.451	5.08E-81	T cells	Snx5
3.76E-85	-5.2931539	0.051	0.214	5.32E-81	T cells	S100a8
4.42E-85	-0.6702693	0.011	0.151	6.26E-81	T cells	Gm15987
9.21E-85	0.67422525	0.558	0.346	1.30E-80	T cells	Arl6ip5
2.73E-84	-1.108294	0.059	0.229	3.87E-80	T cells	Aif1
3.83E-84	-0.8926826	0.1	0.287	5.42E-80	T cells	Birc3
4.85E-84	-0.6911995	0.035	0.193	6.87E-80	T cells	Apobec1
6.11E-84	0.61205535	0.54	0.328	8.65E-80	T cells	Rnaset2b
1.01E-83	-0.6206687	0.009	0.146	1.43E-79	T cells	Gm6377
1.09E-83	-0.7467094	0.029	0.183	1.54E-79	T cells	Tcf4
1.13E-83	0.42665502	0.91	0.86	1.60E-79	T cells	Rplp2
1.27E-83	-0.9352711	0.044	0.204	1.80E-79	T cells	H2-Oa

2.41E-83	-0.8005233	0.01	0.147	3.42E-79	T cells	Snn
2.66E-83	0.76328395	0.138	0.024	3.76E-79	T cells	Cd4
2.69E-83	0.70520073	0.762	0.625	3.80E-79	T cells	Hmgb1
4.80E-83	0.44698776	0.884	0.793	6.79E-79	T cells	Rpl14
5.12E-83	-0.6605609	0.01	0.147	7.25E-79	T cells	Igsf6
6.19E-83	0.61639355	0.247	0.086	8.77E-79	T cells	Spint2
7.41E-83	0.65126064	0.296	0.118	1.05E-78	T cells	Gimap9
7.96E-83	-1.0061323	0.233	0.422	1.13E-78	T cells	Sdcbp
9.25E-83	0.67050785	0.51	0.312	1.31E-78	T cells	Fkbp1a
1.11E-82	0.999835	0.364	0.178	1.58E-78	T cells	Hilpda
1.50E-82	-0.898989	0.056	0.223	2.12E-78	T cells	Ralgps2
2.34E-82	0.71798913	0.239	0.084	3.31E-78	T cells	Galnt6
2.56E-82	-1.3509121	0.08	0.253	3.62E-78	T cells	Cd9
2.59E-82	0.72930988	0.286	0.109	3.67E-78	T cells	Prf1
5.19E-82	-2.3215467	0.07	0.235	7.35E-78	T cells	C1qc
5.21E-82	-0.6105114	0.018	0.16	7.38E-78	T cells	Fgd2
8.01E-82	-0.7806859	0.008	0.141	1.13E-77	T cells	Sirpb1b
1.02E-81	0.38567845	0.949	0.905	1.44E-77	T cells	Rpl19
1.24E-81	0.40185695	0.949	0.889	1.76E-77	T cells	Rps4x
1.30E-81	0.53129319	0.823	0.714	1.84E-77	T cells	Rpl5
1.49E-81	0.59162636	0.683	0.492	2.11E-77	T cells	Eef1d
2.16E-81	0.65040549	0.159	0.035	3.06E-77	T cells	Slamf1
3.25E-81	0.53387391	0.829	0.671	4.60E-77	T cells	Shisa5
4.39E-81	0.61541755	0.748	0.585	6.21E-77	T cells	Myl12a
6.57E-81	0.45444176	0.891	0.83	9.30E-77	T cells	Rps23
8.11E-81	0.42335797	0.932	0.869	1.15E-76	T cells	Rpl32
1.11E-80	0.45464987	0.906	0.816	1.58E-76	T cells	Rpl10a
1.94E-80	-0.6548305	0.048	0.21	2.75E-76	T cells	Sypl
2.97E-80	0.61000899	0.287	0.112	4.21E-76	T cells	Cd7
3.21E-80	-1.6887719	0.01	0.143	4.55E-76	T cells	Thbs1
4.15E-80	-0.8731399	0.092	0.269	5.88E-76	T cells	Pou2f2
6.74E-80	-0.7718106	0.1	0.28	9.54E-76	T cells	Plekho2
9.08E-80	0.5882686	0.517	0.285	1.29E-75	T cells	Rgs1
1.31E-79	0.36077722	0.972	0.918	1.86E-75	T cells	Rpl13
1.40E-79	-0.7762144	0.006	0.133	1.98E-75	T cells	Cxcr5
2.30E-79	-2.5268703	0.096	0.263	3.25E-75	T cells	C1qb
3.08E-79	0.62238971	0.719	0.552	4.36E-75	T cells	S100a6
3.33E-79	0.60073083	0.765	0.625	4.72E-75	T cells	Npm1
4.40E-79	-0.8236733	0.024	0.167	6.23E-75	T cells	Slc15a3
4.48E-79	-0.8324179	0.021	0.161	6.35E-75	T cells	Nupr1
1.00E-78	0.51144015	0.786	0.651	1.42E-74	T cells	Uqcrh
1.14E-78	0.46064372	0.139	0.027	1.61E-74	T cells	Prkcz
1.40E-78	-0.5454034	0.014	0.148	1.97E-74	T cells	Cd180
1.87E-78	-0.7468433	0.018	0.154	2.64E-74	T cells	Tlr2
4.15E-78	-0.7982893	0.045	0.199	5.88E-74	T cells	Lair1
5.18E-78	-0.6379966	0.013	0.145	7.34E-74	T cells	Klra2
5.35E-78	-0.6308758	0.009	0.138	7.58E-74	T cells	Snx8
7.68E-78	0.7083015	0.358	0.175	1.09E-73	T cells	Batf

1.98E-77	-0.8513929	0.143	0.326	2.81E-73	T cells	Snx2
2.43E-77	0.43776149	0.934	0.886	3.45E-73	T cells	Rps7
2.45E-77	-0.7117519	0.023	0.162	3.47E-73	T cells	Ifi204
3.80E-77	-0.7885297	0.006	0.129	5.38E-73	T cells	Pou2af1
4.27E-77	-0.9483783	0.308	0.504	6.04E-73	T cells	Plek
5.78E-77	-1.0519201	0.098	0.27	8.18E-73	T cells	Plin2
1.92E-76	-0.536221	0.014	0.144	2.72E-72	T cells	Nuak2
5.01E-76	-0.5432145	0.011	0.138	7.09E-72	T cells	Lmo2
5.35E-76	0.58125003	0.228	0.078	7.58E-72	T cells	Fam102a
9.95E-76	-0.7033795	0.003	0.122	1.41E-71	T cells	Pax5
1.29E-75	0.55333646	0.832	0.713	1.82E-71	T cells	Rpl12
1.31E-75	-0.9297799	0.012	0.139	1.85E-71	T cells	Mcemp1
2.07E-75	0.35523709	0.95	0.89	2.92E-71	T cells	Rpl6
2.27E-75	0.58451428	0.735	0.59	3.21E-71	T cells	Sumo2
2.80E-75	-0.6163412	0.023	0.159	3.97E-71	T cells	Pid1
3.06E-75	-0.8234272	0.003	0.121	4.33E-71	T cells	Vpreb3
4.98E-75	-3.6095049	0.015	0.144	7.06E-71	T cells	Retnlg
6.26E-75	0.405269	0.909	0.859	8.86E-71	T cells	Rps14
7.00E-75	0.62255406	0.728	0.55	9.91E-71	T cells	Ldha
9.78E-75	-2.1185258	0.03	0.171	1.38E-70	T cells	Il1r2
1.29E-74	0.66887714	0.393	0.212	1.83E-70	T cells	Dnajc15
2.28E-74	-1.0139518	0.015	0.143	3.23E-70	T cells	Il1rn
2.62E-74	-0.6085684	0.017	0.147	3.71E-70	T cells	Lpcat2
3.30E-74	0.41491473	0.891	0.828	4.67E-70	T cells	Rpl15
7.41E-74	0.39601998	0.951	0.873	1.05E-69	T cells	Rpl8
1.20E-73	-2.3043151	0.081	0.237	1.70E-69	T cells	C1qa
1.93E-73	0.37411912	0.909	0.836	2.74E-69	T cells	Rpl29
2.10E-73	-0.9938604	0.232	0.422	2.97E-69	T cells	Fosb
4.35E-73	-0.5918873	0.048	0.199	6.16E-69	T cells	Plekho1
6.41E-73	0.60292572	0.683	0.503	9.08E-69	T cells	Tmem50a
9.28E-73	-0.8749918	0.067	0.226	1.31E-68	T cells	Adrb2
1.18E-72	0.62194946	0.413	0.218	1.67E-68	T cells	Ccnd2
1.40E-72	-1.0027385	0.011	0.133	1.98E-68	T cells	Ccr1
1.42E-72	-5.5847477	0.07	0.222	2.01E-68	T cells	S100a9
2.21E-72	-0.5854661	0.014	0.139	3.12E-68	T cells	Wdfy4
2.86E-72	-0.6175954	0.003	0.118	4.05E-68	T cells	Cd22
2.89E-72	-1.3088035	0.01	0.13	4.09E-68	T cells	Hcar2
4.71E-72	0.61275779	0.157	0.04	6.66E-68	T cells	Sult2b1
5.79E-72	0.53687478	0.748	0.588	8.20E-68	T cells	Ptpn18
7.12E-72	1.44261289	0.516	0.326	1.01E-67	T cells	Ccl4
1.17E-71	-0.8245948	0.033	0.171	1.66E-67	T cells	Ms4a4a
1.44E-71	-2.7014326	0.018	0.144	2.04E-67	T cells	G0s2
1.49E-71	-0.7134031	0.048	0.195	2.11E-67	T cells	Asah1
1.57E-71	1.13070719	0.238	0.094	2.23E-67	T cells	Dusp10
3.26E-71	-0.7763358	0.092	0.257	4.61E-67	T cells	Lamp2
5.91E-71	0.59072952	0.363	0.188	8.37E-67	T cells	Fundc2
6.87E-71	0.86608379	0.155	0.039	9.73E-67	T cells	Top2a
1.17E-70	0.63470966	0.29	0.13	1.65E-66	T cells	Gnptg

1.20E-70	1.59589524	0.377	0.204	1.70E-66	T cells	Ccl3
1.76E-70	0.66105994	0.41	0.224	2.49E-66	T cells	Bcl2a1d
1.90E-70	0.56237033	0.149	0.036	2.69E-66	T cells	Tnfsf10
1.98E-70	-0.9939302	0.137	0.313	2.80E-66	T cells	Ets2
2.65E-70	-0.7179685	0.024	0.155	3.76E-66	T cells	Cfp
3.08E-70	-0.827685	0.171	0.345	4.37E-66	T cells	Gm2a
3.87E-70	0.46915135	0.135	0.029	5.48E-66	T cells	Plcg1
4.62E-70	0.60315367	0.581	0.389	6.54E-66	T cells	Elf1
7.16E-70	0.65776386	0.618	0.427	1.01E-65	T cells	Ifngr1
2.48E-69	0.57445016	0.685	0.521	3.51E-65	T cells	Ndufa13
3.35E-69	0.52314075	0.728	0.565	4.74E-65	T cells	Ppib
3.66E-69	0.36503631	0.972	0.923	5.18E-65	T cells	Rps8
4.08E-69	0.3767282	0.941	0.886	5.77E-65	T cells	Rps5
4.60E-69	0.51264233	0.149	0.037	6.52E-65	T cells	Stk39
4.82E-69	-0.67379	0.006	0.118	6.82E-65	T cells	Ceacam1
5.00E-69	-1.2691822	0.409	0.526	7.07E-65	T cells	Prdx5
6.07E-69	-1.0262471	0.013	0.132	8.59E-65	T cells	Osm
8.81E-69	0.60415933	0.225	0.082	1.25E-64	T cells	Fasl
1.26E-68	-0.7074175	0.033	0.168	1.79E-64	T cells	Fcgr1
1.29E-68	0.51271523	0.576	0.379	1.82E-64	T cells	Prdx6
1.44E-68	-1.6038164	0.151	0.317	2.04E-64	T cells	Ier3
1.69E-68	-0.6081666	0.005	0.116	2.39E-64	T cells	Bcl11a
2.23E-68	-0.7247248	0.048	0.191	3.16E-64	T cells	Swap70
4.31E-68	-1.0494978	0.024	0.15	6.10E-64	T cells	Ly6i
4.43E-68	0.62068495	0.247	0.099	6.27E-64	T cells	Pitpnc1
4.95E-68	-0.6365418	0.043	0.184	7.00E-64	T cells	Map3k8
4.95E-68	-0.5342805	0.013	0.131	7.00E-64	T cells	Pkib
5.16E-68	0.33782632	0.887	0.649	7.31E-64	T cells	Fxyd5
5.87E-68	0.51944647	0.615	0.413	8.31E-64	T cells	Mif
7.21E-68	0.42195449	0.119	0.022	1.02E-63	T cells	Gpr68
8.46E-68	-0.491641	0.01	0.125	1.20E-63	T cells	Lyl1
9.32E-68	0.53055736	0.23	0.088	1.32E-63	T cells	Rpa2
1.03E-67	-0.6084952	0.058	0.207	1.46E-63	T cells	Pik3ap1
1.26E-67	0.47106319	0.86	0.767	1.79E-63	T cells	Myl6
1.92E-67	-0.890607	0.011	0.125	2.72E-63	T cells	Gcnt2
2.25E-67	0.56558591	0.608	0.433	3.18E-63	T cells	Ppp1r18
3.79E-67	0.52874902	0.195	0.064	5.37E-63	T cells	Actn1
6.29E-67	0.39018011	0.118	0.022	8.91E-63	T cells	1700097N02I
7.43E-67	-0.6527773	0.016	0.135	1.05E-62	T cells	Trf
9.12E-67	0.32854503	0.963	0.933	1.29E-62	T cells	Uba52
1.16E-66	0.56295072	0.181	0.056	1.65E-62	T cells	Sytl3
1.98E-66	0.69186803	0.421	0.253	2.80E-62	T cells	Ech1
4.82E-66	-0.6363256	0.022	0.145	6.83E-62	T cells	Xylt1
5.04E-66	0.62279623	0.248	0.104	7.13E-62	T cells	Serpinb6a
5.87E-66	0.5238149	0.159	0.044	8.31E-62	T cells	Cd160
6.96E-66	-1.0409358	0.083	0.235	9.86E-62	T cells	Rnf149
1.14E-65	-1.3998467	0.01	0.121	1.61E-61	T cells	Slc7a11
1.56E-65	0.56707987	0.418	0.239	2.21E-61	T cells	Fkbp3

2.27E-65	-0.5415454	0.017	0.134	3.21E-61	T cells	Ogfrl1
2.39E-65	-0.4992709	0.014	0.13	3.38E-61	T cells	Rbpms
2.90E-65	0.50302754	0.754	0.623	4.11E-61	T cells	Rpl22l1
3.39E-65	-1.1533631	0.156	0.32	4.80E-61	T cells	Cdkn1a
4.30E-65	-0.7078854	0.009	0.119	6.09E-61	T cells	Pygl
5.83E-65	-0.7494527	0.064	0.211	8.25E-61	T cells	Snap23
9.67E-65	-0.9288356	0.115	0.273	1.37E-60	T cells	Ninj1
1.23E-64	-0.4962737	0.017	0.133	1.74E-60	T cells	Cd302
1.25E-64	-0.8165917	0.027	0.151	1.76E-60	T cells	Pltp
2.51E-64	-0.6196052	0.036	0.165	3.56E-60	T cells	Naaa
4.40E-64	0.50250195	0.708	0.547	6.22E-60	T cells	Arf5
5.89E-64	-1.6960172	0.023	0.143	8.34E-60	T cells	Hdc
7.04E-64	-0.5869604	0.758	0.83	9.97E-60	T cells	Cyba
7.27E-64	0.42940431	0.954	0.95	1.03E-59	T cells	Pfn1
9.14E-64	0.40882759	0.103	0.017	1.29E-59	T cells	Apol7e
1.09E-63	0.6349889	0.633	0.477	1.54E-59	T cells	Ran
1.09E-63	-0.6363165	0.028	0.152	1.54E-59	T cells	Sdc3
1.43E-63	0.3551925	0.933	0.905	2.03E-59	T cells	Rps10
1.70E-63	-0.719976	0.026	0.148	2.40E-59	T cells	C3
2.31E-63	-0.5245041	0.042	0.176	3.27E-59	T cells	Prcp
3.81E-63	-0.5715517	0.019	0.135	5.40E-59	T cells	Clec4a2
4.14E-63	-0.9054734	0.066	0.209	5.87E-59	T cells	Ctsl
4.40E-63	-0.6088643	0.596	0.696	6.22E-59	T cells	Rhoa
7.89E-63	-0.8959865	0.045	0.177	1.12E-58	T cells	Cxcl16
8.56E-63	-0.5572699	0.01	0.117	1.21E-58	T cells	Gga2
1.02E-62	-0.6561043	0.079	0.227	1.44E-58	T cells	Hexa
1.11E-62	0.62741752	0.282	0.132	1.57E-58	T cells	Tiprl
2.73E-62	-0.4023283	0.006	0.109	3.86E-58	T cells	Tifa
3.28E-62	-0.4664543	0.017	0.13	4.65E-58	T cells	P2ry6
3.49E-62	-0.4635231	0.021	0.137	4.94E-58	T cells	Dusp3
3.70E-62	0.31579704	0.958	0.924	5.24E-58	T cells	Rpl18a
3.77E-62	-0.9691531	0.126	0.283	5.34E-58	T cells	Tnfrsf1a
3.80E-62	-0.5223504	0.014	0.123	5.38E-58	T cells	Adgre1
3.90E-62	0.48655624	0.75	0.603	5.52E-58	T cells	Atp5g3
4.36E-62	-0.7489551	0.118	0.272	6.17E-58	T cells	Hexb
5.35E-62	-0.5876212	0.011	0.119	7.57E-58	T cells	Trem2
6.73E-62	-0.7553811	0.01	0.116	9.53E-58	T cells	Gm9733
6.98E-62	0.50407276	0.19	0.064	9.88E-58	T cells	Txk
7.28E-62	0.54787004	0.188	0.064	1.03E-57	T cells	Tbx21
7.65E-62	-1.0596948	0.048	0.18	1.08E-57	T cells	Hmox1
8.22E-62	-0.594644	0.014	0.124	1.16E-57	T cells	Hacd4
1.51E-61	-0.7030886	0.369	0.519	2.14E-57	T cells	Atp6v0b
2.55E-61	-1.7419133	0.057	0.19	3.61E-57	T cells	Cxcl9
2.58E-61	0.31976401	0.94	0.901	3.65E-57	T cells	Rps11
3.19E-61	0.49945187	0.137	0.035	4.51E-57	T cells	Gata3
7.79E-61	0.61026544	0.216	0.085	1.10E-56	T cells	Ier5l
7.98E-61	-0.749969	0.162	0.328	1.13E-56	T cells	Clic4
1.00E-60	-0.5052798	0.03	0.151	1.42E-56	T cells	Fcgrt

1.32E-60	0.42063274	0.629	0.432	1.87E-56	T cells	Selplg
1.39E-60	0.37268185	0.934	0.879	1.97E-56	T cells	Rps26
3.23E-60	0.44266881	0.129	0.032	4.57E-56	T cells	Tyms
3.83E-60	-0.7530179	0.111	0.266	5.43E-56	T cells	Creg1
4.22E-60	-0.5464255	0.081	0.227	5.98E-56	T cells	Stx7
4.65E-60	0.51072819	0.717	0.576	6.58E-56	T cells	Atp5d
6.12E-60	0.65751619	0.5	0.332	8.66E-56	T cells	H2afv
1.28E-59	-0.8046099	0.131	0.284	1.81E-55	T cells	Stap1
1.32E-59	-0.456952	0.008	0.108	1.87E-55	T cells	Pira2
2.19E-59	0.5665161	0.633	0.474	3.11E-55	T cells	Prelid1
2.45E-59	0.55959265	0.452	0.282	3.46E-55	T cells	Sec11a
2.73E-59	0.61866194	0.514	0.344	3.86E-55	T cells	Gm8797
3.39E-59	0.78871241	0.368	0.203	4.80E-55	T cells	Rgcc
3.78E-59	0.56244609	0.191	0.069	5.36E-55	T cells	Grap2
8.07E-59	-0.7166755	0.09	0.235	1.14E-54	T cells	Eif4ebp1
9.56E-59	-1.2644145	0.083	0.22	1.35E-54	T cells	Gsr
1.31E-58	0.50583111	0.649	0.487	1.85E-54	T cells	Dad1
1.69E-58	0.62845509	0.494	0.331	2.40E-54	T cells	Cd48
3.30E-58	-0.7145387	0.03	0.147	4.67E-54	T cells	B3gnt5
3.83E-58	-0.6581804	0.038	0.16	5.42E-54	T cells	Irs2
4.26E-58	0.43885534	0.694	0.504	6.03E-54	T cells	Psme2
4.41E-58	-0.5062423	0.043	0.168	6.24E-54	T cells	Ptprj
6.36E-58	0.48670221	0.635	0.475	9.00E-54	T cells	Cnn2
7.92E-58	0.37957335	0.12	0.028	1.12E-53	T cells	Lockd
9.52E-58	0.42191847	0.963	0.855	1.35E-53	T cells	Hsp90ab1
9.73E-58	-0.4969236	0.01	0.11	1.38E-53	T cells	Dpep2
2.07E-57	-0.4076787	0.01	0.11	2.93E-53	T cells	Ltbr
2.46E-57	0.38408993	0.119	0.028	3.48E-53	T cells	Wdr95
4.66E-57	-0.6923313	0.199	0.366	6.59E-53	T cells	Il10ra
5.85E-57	0.52780178	0.613	0.46	8.28E-53	T cells	Psmb3
7.93E-57	0.73425895	0.118	0.027	1.12E-52	T cells	Hist1h1b
1.02E-56	-0.4496016	0.02	0.127	1.45E-52	T cells	Adssl1
1.28E-56	0.41218339	0.167	0.056	1.81E-52	T cells	Nt5c3b
1.32E-56	-0.6268635	0.038	0.157	1.87E-52	T cells	Cfap43
1.84E-56	0.46713903	0.726	0.593	2.60E-52	T cells	Cox6c
1.87E-56	0.58051325	0.147	0.045	2.64E-52	T cells	Cd200r1
2.02E-56	0.60874706	0.286	0.139	2.86E-52	T cells	Dgka
2.06E-56	-0.6032582	0.023	0.133	2.92E-52	T cells	Oasl2
2.27E-56	-0.4172883	0.009	0.106	3.21E-52	T cells	Hhex
2.49E-56	-1.0996221	0.182	0.331	3.53E-52	T cells	Lilrb4a
3.21E-56	0.5536383	0.367	0.205	4.54E-52	T cells	Vgll4
4.76E-56	-1.1489912	0.01	0.106	6.74E-52	T cells	Ptgs2
6.29E-56	-1.7549499	0.036	0.154	8.90E-52	T cells	Ifitm1
6.54E-56	-1.1520796	0.034	0.149	9.26E-52	T cells	Ccl2
9.39E-56	0.53796949	0.534	0.375	1.33E-51	T cells	Csnk2b
1.32E-55	0.30473871	0.961	0.941	1.86E-51	T cells	Gm10076
1.44E-55	-1.584456	0.008	0.103	2.04E-51	T cells	Wfdc21
1.73E-55	0.49541507	0.169	0.059	2.45E-51	T cells	Rab19

3.65E-55	0.49241526	0.175	0.064	5.17E-51	T cells	Eva1b
4.70E-55	-0.6820872	0.35	0.489	6.66E-51	T cells	Erp29
4.92E-55	0.35566745	0.596	0.397	6.97E-51	T cells	Ctsd
5.38E-55	-0.5436919	0.053	0.178	7.61E-51	T cells	Myo1c
5.85E-55	-0.7093093	0.078	0.213	8.28E-51	T cells	Gsn
1.03E-54	0.64603434	0.295	0.15	1.46E-50	T cells	Chd3
1.10E-54	0.37781859	0.865	0.787	1.56E-50	T cells	Rpl41
1.11E-54	0.61516265	0.246	0.115	1.56E-50	T cells	Ubash3b
1.32E-54	-0.4408383	0.008	0.102	1.87E-50	T cells	Slc2a6
2.01E-54	-0.5024748	0.033	0.147	2.85E-50	T cells	Scimp
2.07E-54	-0.536677	0.033	0.147	2.93E-50	T cells	Gng12
2.48E-54	-0.4641756	0.01	0.106	3.52E-50	T cells	Atp6v0a1
2.77E-54	0.52969953	0.547	0.387	3.92E-50	T cells	Mdh2
3.61E-54	0.52685427	0.55	0.394	5.11E-50	T cells	Erh
4.19E-54	-0.5022598	0.04	0.159	5.93E-50	T cells	D1Ertd622e
5.31E-54	0.33857963	0.916	0.87	7.52E-50	T cells	Rps13
5.46E-54	-0.8736987	0.127	0.273	7.73E-50	T cells	Ndel1
5.68E-54	-0.37216	0.01	0.105	8.04E-50	T cells	Plxnb2
8.50E-54	0.38484226	0.822	0.738	1.20E-49	T cells	Btf3
9.24E-54	-0.7264348	0.13	0.275	1.31E-49	T cells	Prkcb
1.12E-53	0.44038089	0.114	0.027	1.59E-49	T cells	Sit1
1.13E-53	0.58198381	0.168	0.058	1.60E-49	T cells	Klrc1
1.70E-53	0.47711933	0.221	0.095	2.40E-49	T cells	Flt3l
1.93E-53	-0.4467132	0.019	0.121	2.73E-49	T cells	Hfe
2.32E-53	-0.7720987	0.632	0.68	3.29E-49	T cells	Atp6v0c
2.37E-53	-0.4007831	0.009	0.101	3.36E-49	T cells	Sirpb1a
2.43E-53	-0.6119663	0.104	0.244	3.43E-49	T cells	Arhgap17
4.08E-53	-0.628277	0.618	0.703	5.77E-49	T cells	Gabarap
4.91E-53	-0.8105622	0.209	0.361	6.95E-49	T cells	Gpcpd1
5.11E-53	-0.6610391	0.031	0.14	7.24E-49	T cells	Slamf9
5.38E-53	-0.4357766	0.022	0.125	7.61E-49	T cells	Ebi3
7.22E-53	0.35910349	0.955	0.934	1.02E-48	T cells	Rps16
7.98E-53	0.58605821	0.675	0.503	1.13E-48	T cells	Vps37b
8.21E-53	-0.6261053	0.332	0.489	1.16E-48	T cells	Tgfb1
8.27E-53	0.31074052	0.956	0.927	1.17E-48	T cells	Rpl23
1.13E-52	-0.5202193	0.11	0.254	1.60E-48	T cells	Lat2
1.14E-52	-0.6594212	0.085	0.218	1.61E-48	T cells	Ero1lb
1.90E-52	-0.6753942	0.018	0.116	2.69E-48	T cells	Gatm
2.07E-52	-0.682909	0.457	0.598	2.93E-48	T cells	Tsc22d3
5.07E-52	0.86071265	0.278	0.145	7.17E-48	T cells	Eea1
6.58E-52	-0.4328946	0.018	0.118	9.32E-48	T cells	Tcn2
9.40E-52	-0.5064704	0.081	0.216	1.33E-47	T cells	Lmo4
9.85E-52	-0.6328982	0.861	0.886	1.39E-47	T cells	Junb
1.07E-51	-0.633465	0.441	0.575	1.52E-47	T cells	Dazap2
1.15E-51	-0.7420005	0.11	0.25	1.62E-47	T cells	Picalm
1.25E-51	-0.8232745	0.055	0.174	1.77E-47	T cells	Tmem176b
1.42E-51	-0.4314736	0.016	0.113	2.01E-47	T cells	Plekhm3
1.92E-51	-0.4906962	0.027	0.132	2.72E-47	T cells	Atp13a2

1.92E-51	0.56155805	0.118	0.031	2.72E-47	T cells	Rora
2.03E-51	0.53975211	0.322	0.18	2.88E-47	T cells	Naa10
2.48E-51	-0.8736728	0.026	0.129	3.51E-47	T cells	Ms4a7
4.80E-51	0.52822924	0.424	0.266	6.79E-47	T cells	Mettl23
5.64E-51	-0.354451	0.023	0.125	7.99E-47	T cells	5031439G07
6.12E-51	0.54199274	0.312	0.17	8.67E-47	T cells	Rnf138
6.90E-51	0.32860819	0.111	0.027	9.77E-47	T cells	Fam92a
1.06E-50	-0.6016829	0.011	0.102	1.50E-46	T cells	Tnfrsf13c
1.33E-50	0.40498873	0.107	0.026	1.88E-46	T cells	Tmem191c
1.43E-50	-0.4735054	0.048	0.165	2.03E-46	T cells	P2rx4
1.61E-50	0.45753529	0.525	0.352	2.29E-46	T cells	Rps27rt
1.67E-50	0.51186858	0.418	0.267	2.37E-46	T cells	Rwdd1
3.22E-50	0.4847501	0.462	0.302	4.56E-46	T cells	Emg1
3.32E-50	0.77383612	0.409	0.261	4.69E-46	T cells	Hif1a
3.84E-50	-0.6111982	0.171	0.32	5.44E-46	T cells	Ppp3ca
4.09E-50	0.63787119	0.439	0.272	5.78E-46	T cells	Ikzf3
4.09E-50	-0.4450607	0.029	0.134	5.79E-46	T cells	Sh2b3
4.14E-50	-0.413083	0.023	0.122	5.86E-46	T cells	Slamf8
4.60E-50	0.59959388	0.524	0.346	6.51E-46	T cells	Gimap6
5.37E-50	-0.826772	0.285	0.433	7.60E-46	T cells	Foxp1
5.81E-50	0.40691587	0.172	0.065	8.23E-46	T cells	Psph
6.24E-50	-0.6164831	0.139	0.284	8.83E-46	T cells	Gm26532
6.61E-50	0.62573052	0.113	0.029	9.35E-46	T cells	Birc5
7.15E-50	-0.5060608	0.041	0.153	1.01E-45	T cells	Cnn3
7.96E-50	-0.4241342	0.038	0.148	1.13E-45	T cells	Acer3
8.46E-50	0.54735622	0.2	0.086	1.20E-45	T cells	Gna15
9.91E-50	0.41256997	0.129	0.038	1.40E-45	T cells	Pglyrp2
1.31E-49	-0.4803007	0.023	0.121	1.86E-45	T cells	Ciita
1.51E-49	0.39938216	0.78	0.646	2.13E-45	T cells	Psmb8
1.55E-49	0.42597998	0.102	0.023	2.20E-45	T cells	Cdca8
1.72E-49	-0.6754187	0.044	0.155	2.44E-45	T cells	Fchsd2
2.58E-49	-0.3663383	0.012	0.102	3.65E-45	T cells	Aldh3b1
2.60E-49	0.52828611	0.193	0.08	3.68E-45	T cells	Cdk6
2.72E-49	0.40498775	0.296	0.151	3.86E-45	T cells	Dnajc9
2.76E-49	0.51966434	0.265	0.131	3.91E-45	T cells	Btg3
3.82E-49	0.52508071	0.273	0.141	5.40E-45	T cells	Pld3
3.87E-49	0.35207882	0.413	0.236	5.47E-45	T cells	Gm8369
3.93E-49	-0.7010471	0.056	0.171	5.57E-45	T cells	Hvcn1
4.74E-49	-0.3832456	0.021	0.117	6.71E-45	T cells	Rasa4
5.57E-49	0.40770076	0.733	0.616	7.89E-45	T cells	Atp5g2
9.51E-49	0.43665986	0.593	0.393	1.35E-44	T cells	Ets1
9.72E-49	0.3415097	0.873	0.798	1.38E-44	T cells	Rpl7a
1.00E-48	-0.5095917	0.021	0.118	1.42E-44	T cells	Bcl2a1a
1.51E-48	0.46726204	0.211	0.093	2.14E-44	T cells	Mcm6
1.87E-48	0.51895683	0.331	0.192	2.65E-44	T cells	Tmem9b
2.14E-48	0.42935616	0.593	0.436	3.03E-44	T cells	Sri
2.97E-48	0.4181619	0.138	0.045	4.20E-44	T cells	Nmb
3.18E-48	0.3737828	0.113	0.03	4.49E-44	T cells	Tk1

3.18E-48	0.7634424	0.137	0.044	4.51E-44	T cells	Hist1h2ae
4.72E-48	-0.3917449	0.034	0.138	6.68E-44	T cells	Tmem106a
5.20E-48	-0.433515	0.029	0.129	7.36E-44	T cells	Rogdi
1.78E-47	0.5066136	0.347	0.202	2.52E-43	T cells	Npm3
2.23E-47	0.47354594	0.201	0.086	3.15E-43	T cells	Cers4
2.40E-47	0.34304829	0.217	0.096	3.39E-43	T cells	Hsd11b1
2.82E-47	-0.4729804	0.024	0.121	3.99E-43	T cells	Snx30
2.90E-47	0.51931188	0.184	0.076	4.11E-43	T cells	Utrn
3.74E-47	-0.6131272	0.07	0.189	5.30E-43	T cells	Sh3bp5
4.39E-47	-0.9298311	0.359	0.514	6.22E-43	T cells	Jun
4.96E-47	0.45241239	0.546	0.392	7.02E-43	T cells	Nol7
6.54E-47	0.56875537	0.433	0.278	9.25E-43	T cells	Cited2
6.56E-47	0.61387287	0.115	0.032	9.28E-43	T cells	Mki67
8.63E-47	0.47744979	0.207	0.091	1.22E-42	T cells	Adam19
8.67E-47	0.51955609	0.235	0.113	1.23E-42	T cells	Dnajc1
8.68E-47	0.46746187	0.255	0.125	1.23E-42	T cells	Rasal3
9.23E-47	0.48745536	0.396	0.248	1.31E-42	T cells	Anxa6
9.27E-47	-0.9494693	0.297	0.425	1.31E-42	T cells	Ctsc
1.03E-46	0.365911	0.124	0.037	1.46E-42	T cells	Abhd8
1.21E-46	0.41681427	0.204	0.089	1.71E-42	T cells	Agpat3
1.70E-46	-1.0634954	0.191	0.333	2.40E-42	T cells	Ccr7
1.88E-46	0.49913205	0.326	0.182	2.67E-42	T cells	Rhoh
1.93E-46	0.47919662	0.127	0.04	2.73E-42	T cells	Nrgn
2.62E-46	0.46211487	0.129	0.04	3.70E-42	T cells	Ly75
2.66E-46	0.50445139	0.532	0.385	3.77E-42	T cells	Krtcap2
2.82E-46	0.5450803	0.372	0.225	3.99E-42	T cells	Cd82
3.39E-46	0.52402509	0.468	0.311	4.79E-42	T cells	Ptms
3.51E-46	-0.6933406	0.045	0.152	4.97E-42	T cells	Basp1
3.95E-46	-0.4252334	0.024	0.118	5.60E-42	T cells	Tppp3
4.48E-46	-0.5091757	0.015	0.102	6.35E-42	T cells	Rasgrp4
5.85E-46	0.46850997	0.555	0.406	8.28E-42	T cells	Srp14
6.08E-46	0.50697058	0.157	0.059	8.60E-42	T cells	Syt11
6.83E-46	0.58616082	0.292	0.157	9.67E-42	T cells	Bcl2
7.28E-46	0.3673122	0.357	0.207	1.03E-41	T cells	Socs1
7.37E-46	-0.5746125	0.218	0.368	1.04E-41	T cells	Inpp5d
8.04E-46	-0.5045312	0.076	0.196	1.14E-41	T cells	Tmem243
8.63E-46	-0.4790407	0.048	0.158	1.22E-41	T cells	Filip1l
1.03E-45	0.37491397	0.132	0.042	1.46E-41	T cells	Nmrk1
1.34E-45	0.34656042	0.83	0.724	1.90E-41	T cells	Eif3f
2.77E-45	-0.7250247	0.257	0.396	3.92E-41	T cells	Zfand5
3.40E-45	-0.4119235	0.016	0.103	4.81E-41	T cells	Cbfa2t3
3.72E-45	0.54714626	0.425	0.263	5.26E-41	T cells	Ablim1
3.87E-45	-1.1001579	0.092	0.214	5.47E-41	T cells	Slc16a3
3.99E-45	0.60482949	0.45	0.305	5.65E-41	T cells	Peli1
4.00E-45	0.42012368	0.65	0.509	5.66E-41	T cells	Atp5c1
4.39E-45	0.41705401	0.157	0.058	6.22E-41	T cells	Ifitm10
4.46E-45	-0.411492	0.039	0.142	6.32E-41	T cells	Mgrn1
4.60E-45	0.49200114	0.55	0.408	6.51E-41	T cells	1810037I17R

6.10E-45	0.50369161	0.216	0.103	8.63E-41	T cells	Ciapin1
6.33E-45	-0.5544606	0.15	0.285	8.95E-41	T cells	Atp6ap1
6.90E-45	-0.4735816	0.018	0.105	9.77E-41	T cells	C3ar1
8.71E-45	-0.9009169	0.207	0.345	1.23E-40	T cells	Cd44
9.14E-45	0.48928727	0.453	0.303	1.29E-40	T cells	Pebp1
9.24E-45	-0.4762503	0.038	0.139	1.31E-40	T cells	Rassf3
9.48E-45	0.45948087	0.403	0.259	1.34E-40	T cells	Nudt21
1.93E-44	-0.7276892	0.374	0.503	2.73E-40	T cells	Adgre5
2.27E-44	-0.7093347	0.224	0.365	3.21E-40	T cells	Snx20
3.15E-44	0.48054275	0.478	0.332	4.46E-40	T cells	Tecr
3.95E-44	-0.6679782	0.216	0.355	5.59E-40	T cells	Tspan13
4.38E-44	0.40469561	0.711	0.579	6.20E-40	T cells	Atp5h
4.49E-44	0.4937457	0.486	0.335	6.35E-40	T cells	Tomm6
6.22E-44	-0.5771679	0.318	0.452	8.80E-40	T cells	Fam49b
8.50E-44	-0.6593151	0.527	0.641	1.20E-39	T cells	Nfkbia
1.04E-43	0.51415042	0.454	0.307	1.47E-39	T cells	7-Sep
1.24E-43	0.56503161	0.408	0.273	1.75E-39	T cells	Gng2
1.27E-43	0.40000736	0.689	0.553	1.80E-39	T cells	Atp5j2
1.30E-43	-0.2971502	0.017	0.102	1.84E-39	T cells	Adam15
1.30E-43	0.45620364	0.283	0.154	1.85E-39	T cells	Tbrg1
1.50E-43	0.52407724	0.215	0.102	2.12E-39	T cells	5430416N02
1.51E-43	-0.5780243	0.079	0.196	2.14E-39	T cells	Nfkbie
2.74E-43	-0.6088265	0.213	0.351	3.88E-39	T cells	Man2b1
3.73E-43	0.43530425	0.655	0.536	5.27E-39	T cells	Snrpg
4.12E-43	0.58077026	0.352	0.21	5.83E-39	T cells	Smad7
4.29E-43	-0.6341552	0.078	0.194	6.08E-39	T cells	Dmxl1
4.75E-43	0.43069318	0.652	0.528	6.73E-39	T cells	Atp5j
5.93E-43	-0.4664983	0.047	0.149	8.39E-39	T cells	Tubb6
8.19E-43	-0.5025605	0.061	0.171	1.16E-38	T cells	Rnf19b
9.53E-43	0.45359551	0.539	0.392	1.35E-38	T cells	Eif3i
1.75E-42	0.53652765	0.274	0.147	2.48E-38	T cells	Cenpa
1.79E-42	-0.4629918	0.03	0.123	2.53E-38	T cells	Ltb4r1
2.25E-42	0.45588734	0.408	0.267	3.19E-38	T cells	Akr1b3
2.27E-42	0.68858643	0.105	0.029	3.21E-38	T cells	Ube2c
2.63E-42	-0.5603908	0.74	0.815	3.72E-38	T cells	Btg1
3.88E-42	0.28664157	0.556	0.366	5.49E-38	T cells	Fyb
6.01E-42	0.45492948	0.459	0.312	8.51E-38	T cells	H2-T22
7.04E-42	-0.6942368	0.081	0.196	9.96E-38	T cells	Ckb
8.70E-42	-0.2936504	0.018	0.101	1.23E-37	T cells	Milr1
9.37E-42	-0.4710165	0.058	0.164	1.33E-37	T cells	Arap1
9.37E-42	0.31154253	0.909	0.848	1.33E-37	T cells	Rpl36
1.11E-41	-0.4028041	0.069	0.181	1.58E-37	T cells	Cnpy3
1.16E-41	-0.5079148	0.025	0.113	1.64E-37	T cells	Treml2
1.19E-41	-0.4780609	0.051	0.154	1.68E-37	T cells	Notch2
1.67E-41	0.30144267	0.879	0.811	2.37E-37	T cells	Rpl7
1.72E-41	-0.3895567	0.019	0.102	2.43E-37	T cells	Rab31
1.80E-41	-0.6088883	0.09	0.207	2.54E-37	T cells	Baz2b
1.98E-41	0.37326908	0.116	0.036	2.80E-37	T cells	Abcb9

2.04E-41	-0.5708677	0.102	0.221	2.89E-37	T cells	Nadk
2.33E-41	0.47272931	0.106	0.031	3.29E-37	T cells	Tbc1d4
3.28E-41	-0.3126951	0.021	0.105	4.64E-37	T cells	Acvrl1
3.65E-41	0.40291684	0.576	0.424	5.16E-37	T cells	Rnaset2a
4.53E-41	0.45031498	0.622	0.497	6.41E-37	T cells	Edf1
5.20E-41	-0.4725483	0.091	0.209	7.37E-37	T cells	Bcl3
5.54E-41	-0.7172006	0.122	0.243	7.85E-37	T cells	Pmaip1
6.27E-41	0.419444	0.629	0.497	8.87E-37	T cells	Serbp1
6.85E-41	-0.3790499	0.038	0.134	9.69E-37	T cells	Sgk3
7.10E-41	0.5378055	0.154	0.062	1.00E-36	T cells	Smc2
9.42E-41	0.4386821	0.318	0.184	1.33E-36	T cells	Psip1
1.16E-40	0.93434159	0.429	0.304	1.65E-36	T cells	Itgb1
1.21E-40	0.35238213	0.105	0.031	1.71E-36	T cells	Fam78a
1.67E-40	0.29918995	0.1	0.028	2.37E-36	T cells	Rcn1
2.18E-40	-0.4924776	0.039	0.133	3.09E-36	T cells	Rab20
2.54E-40	0.41161024	0.381	0.237	3.60E-36	T cells	Acp5
3.80E-40	0.66722388	0.261	0.145	5.38E-36	T cells	Gramd3
4.09E-40	-0.4748902	0.07	0.179	5.78E-36	T cells	Mpp1
4.58E-40	-0.4841949	0.099	0.217	6.48E-36	T cells	Tm6sf1
6.25E-40	-0.3128563	0.032	0.122	8.85E-36	T cells	Tep1
8.98E-40	-0.5084282	0.138	0.266	1.27E-35	T cells	Runx1
1.26E-39	-0.5134986	0.046	0.144	1.79E-35	T cells	Gla
1.27E-39	-0.6353658	0.222	0.35	1.80E-35	T cells	Ncf4
1.46E-39	0.42546036	0.211	0.101	2.07E-35	T cells	Cdk17
1.48E-39	0.64295279	0.243	0.131	2.09E-35	T cells	AU020206
1.64E-39	0.53480698	0.321	0.191	2.33E-35	T cells	Ankrd12
2.31E-39	-0.3677466	0.039	0.132	3.26E-35	T cells	Qk
2.38E-39	-0.5562716	0.221	0.356	3.37E-35	T cells	Smim14
2.70E-39	0.35679035	0.127	0.045	3.82E-35	T cells	Osbpl3
3.19E-39	0.29204225	0.105	0.031	4.51E-35	T cells	Xlr4b
3.49E-39	-0.3917835	0.03	0.117	4.95E-35	T cells	Bmp2k
4.32E-39	0.42067476	0.549	0.408	6.12E-35	T cells	Gabarapl2
7.15E-39	0.68797219	0.238	0.13	1.01E-34	T cells	Nrp1
7.44E-39	0.52671323	0.284	0.162	1.05E-34	T cells	Itpkb
8.57E-39	-0.9218336	0.489	0.566	1.21E-34	T cells	Taldo1
1.20E-38	0.44976605	0.44	0.303	1.70E-34	T cells	Ostc
1.23E-38	0.39756171	0.483	0.345	1.74E-34	T cells	Ndufv3
1.33E-38	0.3846504	0.6	0.467	1.88E-34	T cells	Psmb1
1.77E-38	0.53789646	0.245	0.131	2.51E-34	T cells	Uhrf2
1.90E-38	-0.2938463	0.025	0.109	2.69E-34	T cells	Plod3
2.84E-38	0.40663592	0.465	0.325	4.01E-34	T cells	Eif3m
3.22E-38	0.43765868	0.292	0.166	4.56E-34	T cells	6-Sep
3.60E-38	-0.3303831	0.022	0.103	5.10E-34	T cells	Rab7b
3.65E-38	0.66005138	0.346	0.217	5.17E-34	T cells	Bhlhe40
4.30E-38	0.39151505	0.631	0.503	6.09E-34	T cells	Tma7
4.61E-38	0.42502586	0.131	0.048	6.53E-34	T cells	Atp2b4
4.89E-38	-0.5044577	0.067	0.171	6.93E-34	T cells	Nampt
5.39E-38	0.36989819	0.696	0.572	7.63E-34	T cells	Nsa2

6.88E-38	-1.0135577	0.155	0.277	9.74E-34	T cells	Nfkbiz
8.96E-38	-0.7011761	0.157	0.272	1.27E-33	T cells	Ptp4a3
9.59E-38	-0.3076783	0.025	0.106	1.36E-33	T cells	Ralb
9.91E-38	0.4357144	0.433	0.297	1.40E-33	T cells	Higd1a
1.09E-37	0.49174496	0.289	0.173	1.55E-33	T cells	Rnf166
1.16E-37	-0.5173552	0.092	0.204	1.65E-33	T cells	Sbno2
1.62E-37	-0.3469804	0.031	0.117	2.29E-33	T cells	Tnfrsf13b
1.66E-37	0.46076381	0.201	0.098	2.35E-33	T cells	Tecpr1
1.76E-37	-0.3272189	0.023	0.103	2.49E-33	T cells	Slc31a2
1.77E-37	0.49023089	0.188	0.091	2.50E-33	T cells	Ptpn7
2.76E-37	0.43043279	0.487	0.352	3.91E-33	T cells	Park7
2.77E-37	-0.5252255	0.168	0.291	3.93E-33	T cells	Epn1
3.38E-37	-0.7143879	0.672	0.734	4.78E-33	T cells	Btg2
3.42E-37	-0.5577076	0.086	0.194	4.84E-33	T cells	Tcirg1
4.31E-37	-0.6336135	0.293	0.408	6.10E-33	T cells	Capza2
4.31E-37	-0.4387672	0.034	0.121	6.10E-33	T cells	Mtss1
4.50E-37	0.42269273	0.163	0.071	6.38E-33	T cells	Frat2
5.45E-37	0.42594557	0.358	0.233	7.72E-33	T cells	Cox20
5.47E-37	0.44058345	0.208	0.103	7.74E-33	T cells	Phf6
7.12E-37	0.32162396	0.913	0.877	1.01E-32	T cells	Cfl1
7.45E-37	-0.7569193	0.273	0.393	1.05E-32	T cells	Adipor1
8.76E-37	-0.2865153	0.028	0.111	1.24E-32	T cells	Slc15a4
1.03E-36	0.68317563	0.361	0.24	1.46E-32	T cells	Emb
1.10E-36	0.27258746	0.193	0.089	1.56E-32	T cells	Gm19585
1.12E-36	0.41368832	0.171	0.078	1.59E-32	T cells	Padi2
1.23E-36	0.36545718	0.457	0.309	1.75E-32	T cells	Bcl2a1b
1.25E-36	-0.5967066	0.219	0.348	1.76E-32	T cells	Tmod3
1.35E-36	-0.4919377	0.105	0.217	1.91E-32	T cells	Snx9
1.51E-36	0.37945314	0.297	0.176	2.14E-32	T cells	Bola3
2.19E-36	0.40154898	0.131	0.05	3.11E-32	T cells	Lig1
2.36E-36	-0.466807	0.133	0.253	3.35E-32	T cells	Uvrag
2.78E-36	0.59168404	0.256	0.145	3.94E-32	T cells	Ptpn11
3.35E-36	0.64712256	0.125	0.047	4.74E-32	T cells	Hist1h2ao
4.32E-36	-0.5036635	0.083	0.187	6.11E-32	T cells	Pxk
4.51E-36	0.42286065	0.149	0.062	6.38E-32	T cells	Rnf19a
5.32E-36	0.80479159	0.602	0.497	7.54E-32	T cells	Hmgb2
6.53E-36	0.43742162	0.384	0.253	9.24E-32	T cells	Glrx
6.54E-36	-0.5314989	0.043	0.131	9.26E-32	T cells	Dusp16
7.56E-36	0.46898817	0.48	0.352	1.07E-31	T cells	Spcs2
9.20E-36	0.32528189	0.128	0.048	1.30E-31	T cells	Il27ra
9.29E-36	0.29054026	0.105	0.034	1.32E-31	T cells	Racgap1
1.30E-35	-0.4717455	0.044	0.133	1.84E-31	T cells	Tmcc1
1.41E-35	-0.4631551	0.085	0.191	1.99E-31	T cells	Rara
1.54E-35	0.3415889	0.14	0.056	2.18E-31	T cells	Rab3ip
1.77E-35	0.41638641	0.313	0.193	2.51E-31	T cells	Fam173a
1.78E-35	-0.5252745	0.494	0.602	2.52E-31	T cells	Rap1b
2.38E-35	0.37637516	0.194	0.096	3.37E-31	T cells	Ebpl
3.27E-35	-0.6349999	0.169	0.288	4.63E-31	T cells	Tiparp

4.71E-35	-0.4831685	0.119	0.233	6.66E-31	T cells	Cd86
5.25E-35	0.39323137	0.145	0.061	7.43E-31	T cells	Ipcef1
5.76E-35	0.42323336	0.623	0.504	8.15E-31	T cells	Ndufa4
5.81E-35	0.51925585	0.404	0.273	8.22E-31	T cells	Rapgef6
5.90E-35	-0.6092036	0.106	0.216	8.36E-31	T cells	Gadd45g
6.10E-35	-0.4741974	0.04	0.126	8.63E-31	T cells	Tmem176a
6.29E-35	0.54181238	0.319	0.201	8.91E-31	T cells	Runx3
6.63E-35	0.34013177	0.184	0.085	9.38E-31	T cells	Gimap8
7.42E-35	-0.5944468	0.163	0.28	1.05E-30	T cells	Per1
9.12E-35	-0.5652868	0.219	0.345	1.29E-30	T cells	Tgif1
1.05E-34	0.34304588	0.172	0.077	1.49E-30	T cells	Jakmip1
1.11E-34	0.4718072	0.45	0.325	1.57E-30	T cells	Nme1
1.13E-34	0.25497984	0.114	0.041	1.60E-30	T cells	Pacsin1
1.19E-34	-0.6207919	0.768	0.787	1.68E-30	T cells	Jund
1.28E-34	0.38398711	0.38	0.248	1.81E-30	T cells	Pttg1
1.68E-34	0.34980159	0.202	0.1	2.38E-30	T cells	Rhof
2.22E-34	-0.3896352	0.059	0.155	3.14E-30	T cells	Bach1
3.52E-34	0.39158577	0.275	0.157	4.98E-30	T cells	Spn
4.38E-34	0.43677487	0.396	0.276	6.20E-30	T cells	Tmem160
4.49E-34	-0.6024737	0.081	0.178	6.35E-30	T cells	Bach2
4.57E-34	0.30583413	0.917	0.878	6.47E-30	T cells	Rpl39
5.12E-34	-0.3772867	0.04	0.124	7.25E-30	T cells	Tfeb
5.88E-34	0.50461391	0.286	0.172	8.32E-30	T cells	Cast
7.17E-34	0.40646724	0.46	0.329	1.01E-29	T cells	BC031181
8.69E-34	0.35956905	0.323	0.202	1.23E-29	T cells	Lsm8
1.10E-33	-0.3508072	0.098	0.211	1.56E-29	T cells	Ifngr2
1.11E-33	-0.5087619	0.179	0.297	1.57E-29	T cells	Mtpn
1.11E-33	0.33594177	0.32	0.191	1.57E-29	T cells	Tbc1d10c
1.18E-33	0.39831967	0.181	0.089	1.67E-29	T cells	Jak3
1.18E-33	0.53939567	0.28	0.169	1.67E-29	T cells	Hopx
1.95E-33	0.37865854	0.502	0.372	2.75E-29	T cells	Spcs1
2.07E-33	0.40124423	0.342	0.222	2.92E-29	T cells	Sugt1
2.07E-33	-0.3774827	0.051	0.14	2.93E-29	T cells	Irf2bp2
2.27E-33	0.35260535	0.256	0.145	3.21E-29	T cells	Pop5
2.45E-33	0.30285622	0.131	0.053	3.47E-29	T cells	Stk26
3.33E-33	0.4108792	0.48	0.352	4.71E-29	T cells	Med28
3.55E-33	0.28607906	0.138	0.057	5.02E-29	T cells	Cmc2
3.64E-33	0.44078942	0.215	0.116	5.15E-29	T cells	Casp3
4.23E-33	0.38223709	0.513	0.392	5.99E-29	T cells	Snrpf
4.73E-33	0.39886807	0.167	0.079	6.70E-29	T cells	Ppm1h
4.99E-33	-0.4172065	0.069	0.164	7.06E-29	T cells	Neu1
5.17E-33	0.42447869	0.327	0.211	7.32E-29	T cells	Lcp2
5.31E-33	0.42660632	0.376	0.257	7.52E-29	T cells	Mrpl4
5.76E-33	-0.5144633	0.106	0.211	8.15E-29	T cells	Trafd1
7.44E-33	0.47933932	0.252	0.144	1.05E-28	T cells	Nr4a3
9.06E-33	0.36093248	0.347	0.227	1.28E-28	T cells	Cyb5a
9.30E-33	0.35434493	0.212	0.112	1.32E-28	T cells	Mri1
9.99E-33	-0.6886582	0.16	0.274	1.41E-28	T cells	Nfkbid

1.24E-32	0.51012733	0.322	0.209	1.76E-28	T cells	Prex1
1.61E-32	0.3581859	0.165	0.078	2.28E-28	T cells	Gmnn
1.81E-32	0.38333636	0.59	0.474	2.57E-28	T cells	Polr1d
1.93E-32	0.38542667	0.592	0.485	2.73E-28	T cells	Snrpe
2.15E-32	-0.5618039	0.262	0.385	3.05E-28	T cells	mt-Nd3
2.21E-32	0.36141302	0.105	0.037	3.13E-28	T cells	Tnik
2.36E-32	0.41737197	0.175	0.087	3.34E-28	T cells	Impa2
2.51E-32	0.39039343	0.274	0.167	3.55E-28	T cells	Exosc8
2.60E-32	0.29405616	0.106	0.038	3.68E-28	T cells	Cdkn2c
2.75E-32	0.40462079	0.143	0.062	3.89E-28	T cells	Atad2
2.89E-32	0.40613416	0.485	0.357	4.09E-28	T cells	Tmed9
2.94E-32	0.26768974	0.857	0.801	4.17E-28	T cells	Rpl22
3.03E-32	-0.5006714	0.119	0.227	4.28E-28	T cells	Ptpre
3.15E-32	-0.3351972	0.029	0.104	4.46E-28	T cells	Myo5a
3.35E-32	-0.4432257	0.064	0.155	4.75E-28	T cells	Casp4
3.68E-32	-0.4861082	0.229	0.349	5.21E-28	T cells	Atp6v0d1
4.43E-32	-0.4822545	0.033	0.109	6.27E-28	T cells	Btla
4.81E-32	0.25492338	0.887	0.842	6.81E-28	T cells	Rps12
5.01E-32	0.28317323	0.136	0.057	7.09E-28	T cells	Slc22a15
5.04E-32	0.48783871	0.298	0.185	7.13E-28	T cells	Cblb
5.35E-32	0.50272288	0.363	0.245	7.57E-28	T cells	Gch1
5.37E-32	0.39267146	0.261	0.148	7.60E-28	T cells	Stat4
7.61E-32	0.30385849	0.83	0.757	1.08E-27	T cells	Cox8a
7.65E-32	0.43229155	0.16	0.073	1.08E-27	T cells	Plcxd2
8.31E-32	-0.3481675	0.079	0.177	1.18E-27	T cells	Etv6
1.19E-31	-0.3325222	0.061	0.152	1.68E-27	T cells	Cd2ap
1.20E-31	-0.5318943	0.727	0.755	1.69E-27	T cells	Ubc
1.40E-31	-1.091294	0.237	0.346	1.98E-27	T cells	Grina
2.02E-31	-0.4622051	0.119	0.225	2.86E-27	T cells	Pten
2.14E-31	0.32917794	0.835	0.762	3.03E-27	T cells	Rpl36a
2.26E-31	-0.3970158	0.091	0.191	3.20E-27	T cells	Irak2
2.54E-31	-0.7099164	0.053	0.137	3.59E-27	T cells	Tsc22d1
3.03E-31	0.32096802	0.713	0.625	4.30E-27	T cells	Rpl23a
3.51E-31	0.42302782	0.117	0.046	4.97E-27	T cells	Il18r1
3.81E-31	-0.44018	0.087	0.183	5.39E-27	T cells	Map2k1
4.32E-31	0.30697894	0.713	0.598	6.11E-27	T cells	Limd2
4.45E-31	0.58906323	0.238	0.138	6.30E-27	T cells	Itgav
4.61E-31	0.33407834	0.33	0.214	6.53E-27	T cells	Churc1
4.74E-31	0.28360568	0.821	0.716	6.71E-27	T cells	Rpl36al
6.66E-31	-0.6418999	0.511	0.589	9.42E-27	T cells	Zfp36l1
6.76E-31	-0.4596985	0.163	0.275	9.57E-27	T cells	Camk2d
7.37E-31	0.34871894	0.127	0.053	1.04E-26	T cells	Hacd3
1.03E-30	0.39158632	0.144	0.065	1.46E-26	T cells	Runx2
1.11E-30	0.51615163	0.243	0.141	1.57E-26	T cells	Nrip1
1.41E-30	0.45692867	0.246	0.142	2.00E-26	T cells	4932438A13I
1.49E-30	0.38768262	0.421	0.301	2.11E-26	T cells	Ssb
1.82E-30	0.35950329	0.144	0.065	2.57E-26	T cells	Aebp2
2.51E-30	0.39470285	0.768	0.694	3.56E-26	T cells	Clic1

2.64E-30	-0.3170396	0.036	0.112	3.74E-26	T cells	Dennd5a
2.75E-30	-0.6413518	0.078	0.167	3.90E-26	T cells	Ms4a6d
3.08E-30	0.41116155	0.379	0.264	4.36E-26	T cells	Nt5c
3.12E-30	-0.4817255	0.078	0.172	4.41E-26	T cells	Lmna
3.94E-30	-0.4966919	0.113	0.214	5.58E-26	T cells	Zc3h12a
4.28E-30	-0.5734394	0.146	0.25	6.07E-26	T cells	Herpud1
5.20E-30	-0.4293052	0.078	0.171	7.36E-26	T cells	Dstn
5.48E-30	0.39419585	0.2	0.106	7.76E-26	T cells	Rasgrp1
7.03E-30	0.41447361	0.111	0.044	9.96E-26	T cells	Cks1b
8.35E-30	-0.3230696	0.047	0.128	1.18E-25	T cells	Tec
9.23E-30	-0.4034617	0.061	0.147	1.31E-25	T cells	Snx10
1.20E-29	0.39672366	0.354	0.243	1.69E-25	T cells	Mrps16
1.36E-29	0.51103559	0.273	0.17	1.92E-25	T cells	Mxd4
1.49E-29	0.36948104	0.16	0.078	2.12E-25	T cells	Prr7
1.53E-29	0.33308299	0.596	0.473	2.16E-25	T cells	Ube2i
1.57E-29	0.42286532	0.302	0.196	2.22E-25	T cells	9-Sep
2.23E-29	0.33387659	0.175	0.088	3.16E-25	T cells	Hdac7
3.24E-29	0.32954873	0.105	0.04	4.59E-25	T cells	Hells
3.98E-29	0.27415533	0.11	0.042	5.63E-25	T cells	Pced1b
4.29E-29	1.35955958	0.107	0.042	6.07E-25	T cells	Areg
4.61E-29	0.61028997	0.208	0.119	6.52E-25	T cells	Havcr2
5.43E-29	0.36172907	0.498	0.383	7.69E-25	T cells	Ndufb7
5.73E-29	0.4030928	0.234	0.134	8.11E-25	T cells	Srpk2
5.98E-29	-0.370946	0.061	0.146	8.47E-25	T cells	Dram2
6.04E-29	-0.427922	0.093	0.188	8.55E-25	T cells	Myd88
6.11E-29	0.39140501	0.209	0.116	8.65E-25	T cells	Prkacb
6.42E-29	0.41082757	0.15	0.072	9.09E-25	T cells	Cish
8.06E-29	0.36004208	0.327	0.217	1.14E-24	T cells	Mrpl18
1.08E-28	0.37941645	0.542	0.43	1.53E-24	T cells	Prkar1a
1.18E-28	0.37347951	0.141	0.065	1.67E-24	T cells	Ttc3
1.35E-28	0.3901911	0.144	0.068	1.92E-24	T cells	Zbtb38
1.53E-28	0.36333413	0.473	0.359	2.17E-24	T cells	Mdh1
1.63E-28	0.31917635	0.677	0.558	2.31E-24	T cells	Sec61b
1.66E-28	-0.7949915	0.223	0.325	2.36E-24	T cells	Socs3
2.02E-28	-0.4186025	0.091	0.183	2.86E-24	T cells	Mapkapk2
2.15E-28	-1.2267921	0.136	0.23	3.04E-24	T cells	Mt1
2.38E-28	0.42435333	0.401	0.284	3.37E-24	T cells	Stk4
2.42E-28	-0.4855479	0.1	0.192	3.43E-24	T cells	Man1a
2.59E-28	-0.4063184	0.039	0.111	3.67E-24	T cells	Cyb561a3
2.65E-28	-0.4821095	0.1	0.195	3.75E-24	T cells	Pgd
2.77E-28	-0.598592	0.337	0.433	3.92E-24	T cells	Atp6v1g1
2.83E-28	0.29550052	0.857	0.82	4.01E-24	T cells	Arhgdib
3.78E-28	-0.4017692	0.171	0.281	5.35E-24	T cells	Ikbkb
3.97E-28	0.35651625	0.27	0.168	5.62E-24	T cells	Acadl
4.34E-28	0.30624058	0.988	0.992	6.15E-24	T cells	Actb
5.02E-28	0.33635157	0.3	0.195	7.10E-24	T cells	Ntan1
6.42E-28	0.36181172	0.145	0.069	9.09E-24	T cells	Ezh2
6.99E-28	-0.3093062	0.053	0.133	9.89E-24	T cells	Dnase2a

7.44E-28	0.49419575	0.437	0.325	1.05E-23	T cells	Atp1b3
9.71E-28	0.279071	0.739	0.615	1.37E-23	T cells	Psme1
1.19E-27	0.7911586	0.249	0.157	1.68E-23	T cells	Stmn1
1.39E-27	-0.5467993	0.097	0.189	1.96E-23	T cells	Fbxl5
1.42E-27	0.43836412	0.514	0.391	2.00E-23	T cells	Tpr
1.46E-27	-0.4064176	0.142	0.244	2.06E-23	T cells	Ehd4
1.69E-27	0.32375796	0.239	0.143	2.39E-23	T cells	Dpy30
1.76E-27	-0.342956	0.082	0.172	2.50E-23	T cells	Gyg
1.95E-27	0.37179478	0.25	0.155	2.76E-23	T cells	Nans
2.00E-27	-0.3101817	0.967	0.957	2.83E-23	T cells	H3f3b
2.36E-27	0.41697922	0.28	0.18	3.34E-23	T cells	Mrps36
2.86E-27	-0.5357486	0.141	0.239	4.05E-23	T cells	Ptp4a1
3.10E-27	0.40051861	0.304	0.203	4.40E-23	T cells	Ebp
4.26E-27	0.3297997	0.132	0.06	6.04E-23	T cells	Mkrn2
4.55E-27	-0.4243441	0.166	0.271	6.44E-23	T cells	Fuca1
4.74E-27	0.36150743	0.421	0.307	6.71E-23	T cells	Txn2
6.05E-27	0.33081235	0.253	0.155	8.57E-23	T cells	Fyn
6.20E-27	0.41287711	0.231	0.139	8.78E-23	T cells	Tmem126a
7.24E-27	0.29321433	0.102	0.04	1.03E-22	T cells	Ncapd3
8.13E-27	0.35364587	0.641	0.54	1.15E-22	T cells	Cox5a
1.12E-26	0.31719743	0.15	0.074	1.59E-22	T cells	Agfg1
1.16E-26	0.35757083	0.234	0.141	1.64E-22	T cells	Pam16
1.17E-26	0.35650798	0.194	0.108	1.66E-22	T cells	Pigp
1.35E-26	0.26269036	0.961	0.927	1.91E-22	T cells	Rps24
1.56E-26	0.34841176	0.149	0.073	2.20E-22	T cells	Aak1
1.58E-26	-0.3013634	0.05	0.126	2.24E-22	T cells	Naga
2.06E-26	-0.6410605	0.215	0.316	2.92E-22	T cells	Csrnp1
2.20E-26	0.34680533	0.688	0.602	3.12E-22	T cells	Ppp1ca
2.35E-26	0.28963785	0.126	0.057	3.33E-22	T cells	Wdyhv1
2.83E-26	0.37982258	0.287	0.184	4.01E-22	T cells	Arhgap31
2.84E-26	0.5558042	0.475	0.374	4.02E-22	T cells	Tubb5
2.85E-26	-0.3609141	0.053	0.129	4.03E-22	T cells	Fbxo11
2.88E-26	-1.561526	0.416	0.491	4.07E-22	T cells	Hspa1a
3.41E-26	-0.4232275	0.077	0.162	4.83E-22	T cells	Xdh
3.50E-26	-0.5381165	0.054	0.129	4.95E-22	T cells	Gadd45a
3.67E-26	-0.3202036	0.043	0.114	5.20E-22	T cells	Lpcat1
4.49E-26	0.25695189	0.131	0.061	6.35E-22	T cells	Gnpda2
4.94E-26	-0.5156772	0.199	0.302	6.99E-22	T cells	Ppp1r2
5.66E-26	-0.3628259	0.087	0.178	8.02E-22	T cells	Il6ra
5.68E-26	-0.3677955	0.066	0.146	8.03E-22	T cells	Synj1
5.72E-26	-0.3422428	0.086	0.174	8.09E-22	T cells	Rnf13
5.98E-26	0.31253067	0.929	0.909	8.46E-22	T cells	Cd52
6.25E-26	0.31396197	0.511	0.387	8.85E-22	T cells	Psmb9
6.37E-26	-0.465083	0.251	0.354	9.02E-22	T cells	Csk
6.84E-26	-0.3379416	0.095	0.184	9.69E-22	T cells	Nfkb2
7.49E-26	-0.4415736	0.172	0.273	1.06E-21	T cells	Fam174a
8.20E-26	0.254637	0.145	0.07	1.16E-21	T cells	Dtd1
9.45E-26	-0.3140146	0.055	0.131	1.34E-21	T cells	Ccdc86

1.01E-25	0.39164017	0.363	0.257	1.43E-21	T cells	Tsn
1.15E-25	-0.458133	0.107	0.197	1.63E-21	T cells	Slc6a6
1.18E-25	0.32010015	0.549	0.439	1.67E-21	T cells	Psma2
1.26E-25	-0.5124971	0.263	0.362	1.79E-21	T cells	Rrbp1
1.51E-25	-0.4272491	0.499	0.569	2.14E-21	T cells	Gnb2
1.57E-25	0.34409962	0.158	0.081	2.23E-21	T cells	Crbn
1.67E-25	0.43585332	0.248	0.153	2.36E-21	T cells	Cntrl
2.18E-25	-0.315639	0.066	0.146	3.08E-21	T cells	Oas1a
2.21E-25	0.36137499	0.328	0.226	3.13E-21	T cells	Aimp1
2.33E-25	0.25695289	0.115	0.051	3.30E-21	T cells	Ssbp3
2.75E-25	0.31872663	0.146	0.071	3.90E-21	T cells	Trim59
2.82E-25	0.31090091	0.142	0.069	3.99E-21	T cells	Dbf4
3.49E-25	0.40174709	0.355	0.251	4.94E-21	T cells	Ralbp1
3.55E-25	0.31317831	0.225	0.133	5.02E-21	T cells	Fam3c
4.03E-25	-0.4896792	0.143	0.238	5.70E-21	T cells	Ppt1
4.15E-25	0.31735545	0.151	0.078	5.87E-21	T cells	Mphosph6
4.20E-25	-0.4542488	0.037	0.103	5.94E-21	T cells	Smox
4.42E-25	0.45449198	0.365	0.262	6.26E-21	T cells	Zc3hav1
4.57E-25	-0.2613533	0.048	0.12	6.47E-21	T cells	Pacsin2
4.85E-25	0.29169674	0.124	0.056	6.86E-21	T cells	Scml4
5.23E-25	0.28785201	0.272	0.176	7.40E-21	T cells	Dctn6
5.48E-25	0.39963425	0.168	0.09	7.76E-21	T cells	Rbl2
6.97E-25	0.33023946	0.117	0.052	9.87E-21	T cells	Igf2r
7.36E-25	-1.0593558	0.105	0.19	1.04E-20	T cells	Ccrl2
7.73E-25	-0.4149005	0.199	0.296	1.09E-20	T cells	Rtn4
8.88E-25	-0.6135995	0.273	0.361	1.26E-20	T cells	Pnp
1.20E-24	-0.4578676	0.182	0.283	1.70E-20	T cells	Cyth1
1.23E-24	-0.5353465	0.106	0.194	1.74E-20	T cells	Sgk1
1.26E-24	0.3587603	0.32	0.221	1.79E-20	T cells	Lypla2
1.41E-24	0.32061572	0.201	0.115	1.99E-20	T cells	Ss18
1.49E-24	0.35318923	0.311	0.214	2.12E-20	T cells	Pstpip1
1.60E-24	0.30354877	0.552	0.437	2.27E-20	T cells	Cox7b
1.85E-24	0.31700652	0.275	0.181	2.62E-20	T cells	Pfdn1
1.93E-24	-0.312537	0.067	0.145	2.73E-20	T cells	Mvp
1.98E-24	0.36205973	0.268	0.175	2.80E-20	T cells	Polr2c
2.22E-24	0.26689607	0.551	0.431	3.14E-20	T cells	Srp9
2.62E-24	-0.6621769	0.295	0.381	3.71E-20	T cells	Efhd2
2.71E-24	-0.3200457	0.039	0.104	3.84E-20	T cells	Plekhm1
2.77E-24	0.3626277	0.32	0.22	3.92E-20	T cells	Chic2
3.08E-24	0.27731059	0.101	0.042	4.37E-20	T cells	Cbx5
3.28E-24	-0.4939067	0.182	0.274	4.64E-20	T cells	Add3
3.38E-24	-0.3433234	0.228	0.346	4.78E-20	T cells	Klf3
3.95E-24	-0.4344274	0.413	0.504	5.60E-20	T cells	Gdi2
4.22E-24	-0.3960789	0.139	0.233	5.97E-20	T cells	Atp6v1b2
4.90E-24	-0.5658703	0.575	0.644	6.93E-20	T cells	Stk17b
4.94E-24	-0.3936396	0.056	0.127	6.99E-20	T cells	Trp53inp2
6.36E-24	0.37820008	0.191	0.11	9.00E-20	T cells	Ttc39b
6.97E-24	0.29766096	0.604	0.495	9.86E-20	T cells	Atp5f1

7.10E-24	-0.4578615	0.206	0.305	1.00E-19	T cells	Rasgrp2
7.94E-24	0.35682568	0.175	0.097	1.12E-19	T cells	Dnmt1
8.85E-24	-0.3375176	0.1	0.187	1.25E-19	T cells	Map7d1
9.34E-24	-0.9697321	0.182	0.273	1.32E-19	T cells	Lilr4b
1.38E-23	0.33846336	0.506	0.406	1.95E-19	T cells	Atp5o
1.62E-23	-0.4113127	0.239	0.341	2.30E-19	T cells	Ubl3
1.83E-23	0.32448362	0.441	0.335	2.59E-19	T cells	Snrpd3
1.86E-23	0.33263153	0.353	0.254	2.63E-19	T cells	Mien1
1.86E-23	-0.4472612	0.198	0.294	2.63E-19	T cells	Wsb1
2.04E-23	0.35927097	0.389	0.29	2.89E-19	T cells	Anapc11
2.05E-23	0.41613351	0.198	0.116	2.91E-19	T cells	Rsbn1
2.13E-23	-0.5240912	0.243	0.339	3.01E-19	T cells	Rab7
2.26E-23	0.29099531	0.668	0.565	3.20E-19	T cells	Eif3k
2.47E-23	0.28548786	0.168	0.093	3.49E-19	T cells	4921524J17R
2.77E-23	-0.4623981	0.048	0.115	3.92E-19	T cells	Rabgef1
3.51E-23	0.34349013	0.437	0.331	4.97E-19	T cells	Prdx2
4.22E-23	0.41892206	0.264	0.174	5.97E-19	T cells	Csnk2a1
4.55E-23	0.29184121	0.128	0.063	6.44E-19	T cells	Rdm1
6.47E-23	-0.4256078	0.329	0.415	9.16E-19	T cells	Laptm4a
9.16E-23	-0.3566637	0.043	0.107	1.30E-18	T cells	Zfp318
9.66E-23	0.34240287	0.413	0.312	1.37E-18	T cells	Tmem167
1.03E-22	0.4482428	0.419	0.33	1.46E-18	T cells	Dek
1.15E-22	-1.0229351	0.06	0.13	1.63E-18	T cells	Cxcl10
1.16E-22	-0.3211839	0.084	0.165	1.64E-18	T cells	Cux1
1.18E-22	-0.329217	0.091	0.172	1.68E-18	T cells	Egln2
1.35E-22	0.35185235	0.261	0.173	1.92E-18	T cells	Sar1b
1.39E-22	-0.3890364	0.092	0.172	1.97E-18	T cells	Parp1
1.51E-22	-0.3991987	0.138	0.228	2.14E-18	T cells	Mef2a
1.57E-22	-0.3830324	0.047	0.112	2.22E-18	T cells	Ttpal
1.63E-22	-0.5026618	0.298	0.383	2.30E-18	T cells	Ctsa
1.63E-22	0.28529335	0.5	0.39	2.31E-18	T cells	Tomm20
1.89E-22	-0.3656827	0.629	0.693	2.67E-18	T cells	Ywhaz
2.05E-22	0.25582702	0.123	0.059	2.91E-18	T cells	Ormdl3
2.40E-22	0.37085222	0.199	0.118	3.40E-18	T cells	Isg20
2.51E-22	0.31748674	0.135	0.069	3.56E-18	T cells	Bzw2
2.91E-22	0.38621728	0.239	0.155	4.11E-18	T cells	Def6
2.94E-22	0.31596326	0.171	0.097	4.17E-18	T cells	Vcpkmt
3.15E-22	-0.3535509	0.139	0.23	4.46E-18	T cells	Kdm7a
3.57E-22	-0.3531329	0.065	0.136	5.06E-18	T cells	Tnrc18
4.53E-22	0.41070867	0.166	0.095	6.41E-18	T cells	Nlrc5
4.62E-22	0.32937122	0.203	0.121	6.53E-18	T cells	Prrc2b
4.89E-22	-0.4234635	0.175	0.264	6.93E-18	T cells	Spop
4.91E-22	0.28338761	0.675	0.6	6.96E-18	T cells	Ubl5
4.96E-22	-0.3844692	0.168	0.262	7.02E-18	T cells	Rasa3
5.02E-22	-0.4201845	0.074	0.147	7.10E-18	T cells	Eif2ak3
5.05E-22	0.25548846	0.106	0.047	7.14E-18	T cells	Gm20186
5.58E-22	0.36823285	0.389	0.293	7.90E-18	T cells	Ranbp1
6.32E-22	-0.3181797	0.061	0.132	8.95E-18	T cells	Tgfbr1

7.28E-22	0.3054371	0.477	0.37	1.03E-17	T cells	Lsm4
8.40E-22	0.45306189	0.338	0.246	1.19E-17	T cells	Dock10
8.41E-22	0.25191239	0.143	0.074	1.19E-17	T cells	Ppil1
9.30E-22	0.35851404	0.176	0.101	1.32E-17	T cells	Nsmaf
9.60E-22	0.30900361	0.268	0.178	1.36E-17	T cells	Ccng1
1.12E-21	-0.2961213	0.041	0.103	1.59E-17	T cells	Hk2
1.31E-21	-0.2559048	0.047	0.112	1.85E-17	T cells	Arf2
1.36E-21	0.31802956	0.227	0.146	1.93E-17	T cells	Ssbp4
1.67E-21	0.31241567	0.138	0.072	2.36E-17	T cells	Galnt2
1.68E-21	0.30519879	0.552	0.45	2.38E-17	T cells	Ndufa2
1.84E-21	0.29409344	0.198	0.12	2.60E-17	T cells	Itgb1bp1
2.04E-21	0.39679354	0.335	0.238	2.88E-17	T cells	Cdkn1b
2.14E-21	0.27946798	0.567	0.468	3.02E-17	T cells	Psma3
2.74E-21	0.27531086	0.204	0.125	3.88E-17	T cells	Pdrg1
2.78E-21	0.29848303	0.366	0.268	3.94E-17	T cells	Ndufv2
3.04E-21	-0.3666186	0.13	0.215	4.31E-17	T cells	Ist1
3.34E-21	-0.2966952	0.069	0.14	4.73E-17	T cells	Colgalt1
3.41E-21	-0.6142579	0.195	0.282	4.83E-17	T cells	Irf7
3.57E-21	-0.3141574	0.116	0.199	5.05E-17	T cells	Hmga1
3.93E-21	0.27712412	0.154	0.084	5.57E-17	T cells	Esyt2
4.35E-21	-0.2923161	0.088	0.165	6.16E-17	T cells	Abhd12
4.48E-21	-0.3245019	0.098	0.176	6.35E-17	T cells	Mfsd1
4.97E-21	0.2541913	0.152	0.082	7.03E-17	T cells	Tmem71
5.16E-21	-0.4947306	0.479	0.539	7.30E-17	T cells	Rhog
5.23E-21	0.29825449	0.238	0.147	7.40E-17	T cells	Acap1
5.24E-21	-0.2975314	0.074	0.146	7.41E-17	T cells	Vps26a
5.67E-21	-0.3986111	0.298	0.394	8.02E-17	T cells	Hcls1
6.44E-21	-0.3749902	0.047	0.109	9.12E-17	T cells	Arrdc3
6.74E-21	0.2894537	0.421	0.321	9.54E-17	T cells	Snrpd2
7.65E-21	-0.4633256	0.067	0.134	1.08E-16	T cells	Irf4
7.69E-21	0.26488915	0.147	0.079	1.09E-16	T cells	Sfxn1
7.77E-21	-0.3189828	0.055	0.121	1.10E-16	T cells	Flcn
9.06E-21	0.28452892	0.417	0.318	1.28E-16	T cells	Dctn3
9.28E-21	0.36184351	0.122	0.062	1.31E-16	T cells	Oxsr1
9.48E-21	-0.2513625	0.054	0.12	1.34E-16	T cells	Mgat1
9.91E-21	-0.4507689	0.272	0.36	1.40E-16	T cells	Syngr2
1.03E-20	-0.4905478	0.202	0.293	1.46E-16	T cells	Mbnl2
1.06E-20	-0.3251075	0.065	0.134	1.50E-16	T cells	Med7
1.27E-20	0.35017506	0.113	0.054	1.80E-16	T cells	Dusp4
1.28E-20	-0.4099019	0.146	0.229	1.82E-16	T cells	Abhd17b
1.29E-20	0.32036474	0.339	0.239	1.83E-16	T cells	Isy1
1.39E-20	0.29984465	0.573	0.466	1.97E-16	T cells	Jak1
1.58E-20	0.29400028	0.139	0.073	2.24E-16	T cells	Zbtb1
1.65E-20	0.33072672	0.397	0.3	2.33E-16	T cells	Cuta
1.71E-20	0.32443529	0.361	0.268	2.42E-16	T cells	Smap1
1.73E-20	-0.426327	0.365	0.454	2.45E-16	T cells	Ccnl1
1.75E-20	0.37649424	0.309	0.224	2.48E-16	T cells	Etfb
1.78E-20	-0.4212074	0.232	0.32	2.52E-16	T cells	Tmbim4

1.99E-20	0.30924768	0.363	0.267	2.81E-16	T cells	Ssr2
2.05E-20	-0.307089	0.632	0.683	2.90E-16	T cells	Rpl10
2.05E-20	-0.5256463	0.047	0.108	2.90E-16	T cells	Iigp1
2.08E-20	0.28313215	0.219	0.139	2.94E-16	T cells	Mdp1
2.12E-20	0.27948573	0.623	0.528	3.01E-16	T cells	Hint1
2.33E-20	-0.2577097	0.045	0.107	3.30E-16	T cells	Cln3
2.39E-20	-0.5427702	0.418	0.494	3.38E-16	T cells	Dusp2
2.47E-20	-0.2545224	0.05	0.113	3.50E-16	T cells	Epb41l2
2.67E-20	0.36950968	0.337	0.246	3.79E-16	T cells	Sh3kbp1
3.13E-20	0.32290298	0.261	0.178	4.44E-16	T cells	Nutf2
3.33E-20	-0.3468225	0.103	0.181	4.71E-16	T cells	Abi3
3.37E-20	0.3745611	0.528	0.427	4.77E-16	T cells	Sec62
3.42E-20	0.28772914	0.233	0.151	4.85E-16	T cells	Mrps26
3.51E-20	-0.4640694	0.111	0.186	4.96E-16	T cells	Cndp2
3.53E-20	0.30613743	0.15	0.084	5.00E-16	T cells	Kcnn4
3.60E-20	-0.3463819	0.13	0.213	5.10E-16	T cells	Nckap1l
3.67E-20	0.30408339	0.169	0.098	5.19E-16	T cells	Mmd
3.70E-20	0.30300585	0.334	0.242	5.24E-16	T cells	Vps29
3.95E-20	-0.4163542	0.223	0.309	5.59E-16	T cells	Pitpna
3.97E-20	-0.4988973	0.307	0.391	5.62E-16	T cells	Mpc1
4.80E-20	0.33214668	0.29	0.204	6.79E-16	T cells	Ssna1
4.85E-20	-0.2794881	0.481	0.571	6.86E-16	T cells	Iqgap1
5.04E-20	0.25950932	0.222	0.143	7.13E-16	T cells	Zbtb8os
5.52E-20	0.35166475	0.358	0.261	7.82E-16	T cells	Pycard
6.66E-20	0.38261652	0.202	0.126	9.43E-16	T cells	Mlec
6.73E-20	0.27344761	0.496	0.392	9.52E-16	T cells	Bsg
7.49E-20	-0.2650325	0.073	0.143	1.06E-15	T cells	Plekhf2
7.71E-20	-0.3305332	0.118	0.196	1.09E-15	T cells	Lmbrd1
7.98E-20	0.42482077	0.3	0.211	1.13E-15	T cells	Nfatc1
8.42E-20	0.30643323	0.269	0.185	1.19E-15	T cells	Denr
8.68E-20	0.28089036	0.377	0.282	1.23E-15	T cells	Bola2
1.01E-19	-0.3568302	0.079	0.149	1.43E-15	T cells	Mylip
1.03E-19	-0.4692464	0.868	0.835	1.46E-15	T cells	mt-Nd4l
1.06E-19	0.31293953	0.237	0.156	1.50E-15	T cells	Fam162a
1.10E-19	-0.5431461	0.167	0.251	1.55E-15	T cells	Sorl1
1.10E-19	0.28982076	0.252	0.166	1.56E-15	T cells	Cbx3
1.22E-19	-0.8197997	0.113	0.185	1.73E-15	T cells	Gbp2
1.33E-19	-0.6874596	0.629	0.644	1.88E-15	T cells	Vim
1.56E-19	-0.3907479	0.053	0.114	2.21E-15	T cells	St6gal1
1.88E-19	-0.376588	0.101	0.175	2.67E-15	T cells	Sgms1
1.95E-19	0.29585676	0.24	0.16	2.76E-15	T cells	Ddt
2.23E-19	0.2790784	0.264	0.181	3.15E-15	T cells	Pigx
2.24E-19	0.30794113	0.424	0.328	3.16E-15	T cells	U2af1
2.52E-19	-0.3096911	0.051	0.113	3.57E-15	T cells	Tbc1d14
3.01E-19	0.28314342	0.423	0.331	4.26E-15	T cells	Calm3
3.03E-19	0.28839638	0.196	0.121	4.29E-15	T cells	Usp1
3.04E-19	0.39668056	0.233	0.157	4.30E-15	T cells	Bscl2
3.04E-19	0.27723659	0.394	0.298	4.30E-15	T cells	Dynlrb1

3.05E-19	0.25295357	0.136	0.073	4.31E-15	T cells	Mcm4
3.20E-19	0.2860649	0.15	0.085	4.53E-15	T cells	Psmg2
3.24E-19	0.31032662	0.324	0.235	4.59E-15	T cells	Emc10
3.26E-19	0.28160081	0.416	0.314	4.62E-15	T cells	Crem
3.30E-19	0.32648046	0.312	0.227	4.67E-15	T cells	Rexo2
3.30E-19	-0.3052623	0.198	0.293	4.68E-15	T cells	Neurl3
3.85E-19	0.27841058	0.204	0.13	5.45E-15	T cells	Surf1
3.91E-19	0.25937352	0.522	0.42	5.54E-15	T cells	Tomm22
3.93E-19	0.25070202	0.216	0.139	5.57E-15	T cells	Dcps
4.00E-19	-0.4083714	0.128	0.207	5.66E-15	T cells	Elmsan1
4.12E-19	-0.4134215	0.733	0.744	5.84E-15	T cells	Ier2
4.36E-19	0.3406618	0.272	0.19	6.18E-15	T cells	Ift20
4.72E-19	0.26260262	0.838	0.773	6.68E-15	T cells	Sub1
4.74E-19	0.30809212	0.306	0.214	6.70E-15	T cells	Cd164
5.44E-19	0.37797102	0.424	0.333	7.70E-15	T cells	Eif3c
6.02E-19	0.32265441	0.227	0.151	8.52E-15	T cells	Borcs8
6.12E-19	-0.3533816	0.224	0.311	8.66E-15	T cells	Grb2
6.17E-19	0.32957171	0.282	0.194	8.73E-15	T cells	Slc1a5
6.43E-19	0.32189716	0.16	0.092	9.11E-15	T cells	Vps13a
6.51E-19	-0.3635137	0.223	0.313	9.21E-15	T cells	Bcl10
6.99E-19	0.33525987	0.261	0.181	9.90E-15	T cells	Dap
7.03E-19	0.25861909	0.224	0.147	9.95E-15	T cells	Cmc1
7.05E-19	0.31513684	0.476	0.38	9.98E-15	T cells	Pak2
7.17E-19	-0.2744572	0.095	0.17	1.02E-14	T cells	Dapp1
7.41E-19	0.26694081	0.305	0.219	1.05E-14	T cells	Ndufs6
9.50E-19	0.30800477	0.34	0.253	1.34E-14	T cells	Cops6
1.02E-18	0.33131048	0.622	0.522	1.45E-14	T cells	Srrm2
1.08E-18	0.27530237	0.381	0.288	1.53E-14	T cells	Ndufb6
1.11E-18	-0.3952476	0.38	0.464	1.57E-14	T cells	Arf6
1.14E-18	0.2892696	0.269	0.186	1.62E-14	T cells	Thoc7
1.24E-18	-0.3048626	0.144	0.228	1.76E-14	T cells	Pkn1
1.25E-18	-0.3940549	0.119	0.194	1.77E-14	T cells	Pnpla2
1.56E-18	0.38786531	0.335	0.247	2.20E-14	T cells	Ppp1r12a
1.62E-18	0.30248759	0.217	0.139	2.29E-14	T cells	Pnn
1.98E-18	-0.2594429	0.074	0.141	2.81E-14	T cells	Pdlim5
2.00E-18	-0.2659689	0.073	0.14	2.83E-14	T cells	Camk1d
2.02E-18	-0.3206629	0.208	0.3	2.86E-14	T cells	Ssh2
2.02E-18	-0.3065208	0.693	0.728	2.86E-14	T cells	Cdc42
2.09E-18	0.29581913	0.286	0.202	2.96E-14	T cells	Imp3
2.21E-18	-0.2977407	0.07	0.134	3.13E-14	T cells	Rere
2.53E-18	-0.324169	0.138	0.217	3.58E-14	T cells	Vav1
2.64E-18	0.29318482	0.765	0.673	3.74E-14	T cells	H2afz
2.74E-18	-0.286156	0.056	0.118	3.88E-14	T cells	Slc12a6
2.80E-18	0.320166	0.259	0.175	3.96E-14	T cells	Rad21
2.84E-18	0.25135328	0.355	0.261	4.02E-14	T cells	Bcas2
2.85E-18	0.28257365	0.108	0.055	4.04E-14	T cells	Cib2
3.12E-18	0.29087817	0.436	0.341	4.42E-14	T cells	Cwc15
3.51E-18	0.36567523	0.211	0.136	4.96E-14	T cells	Fryl

3.57E-18	-0.2878098	0.116	0.191	5.06E-14	T cells	Gusb
3.90E-18	-0.444171	0.269	0.348	5.52E-14	T cells	Tkt
4.15E-18	-0.2972774	0.057	0.116	5.87E-14	T cells	A630001G21
4.19E-18	0.28291283	0.403	0.31	5.93E-14	T cells	Ociad1
4.47E-18	0.40814844	0.435	0.343	6.33E-14	T cells	Prrc2c
5.18E-18	0.25332876	0.248	0.167	7.34E-14	T cells	Rdx
5.42E-18	-0.4612236	0.195	0.277	7.67E-14	T cells	Tcp11l2
5.46E-18	-0.3435615	0.179	0.261	7.73E-14	T cells	Nrbp1
5.50E-18	0.25523715	0.14	0.078	7.78E-14	T cells	Rangap1
5.69E-18	0.2524312	0.58	0.486	8.05E-14	T cells	Snrpb
5.91E-18	-0.3666406	0.049	0.106	8.37E-14	T cells	Grasp
6.13E-18	0.28241996	0.164	0.096	8.68E-14	T cells	Nup210
6.48E-18	0.30117713	0.308	0.225	9.17E-14	T cells	Pa2g4
6.56E-18	0.26101299	0.281	0.197	9.29E-14	T cells	Med10
6.78E-18	0.30091878	0.379	0.29	9.60E-14	T cells	Psmd4
6.82E-18	-0.3261221	0.14	0.219	9.66E-14	T cells	Sh3bgrl
7.12E-18	0.28854982	0.411	0.321	1.01E-13	T cells	Psmb4
7.22E-18	0.27865363	0.125	0.068	1.02E-13	T cells	Mitd1
7.79E-18	-0.2580682	0.058	0.119	1.10E-13	T cells	Cers6
7.96E-18	0.28410078	0.281	0.199	1.13E-13	T cells	Scamp3
8.67E-18	0.27577701	0.447	0.352	1.23E-13	T cells	Ndufa11
8.94E-18	0.25706006	0.468	0.372	1.27E-13	T cells	Raly
9.85E-18	-0.4056045	0.149	0.225	1.39E-13	T cells	Dynlt1b
1.19E-17	0.29854783	0.325	0.24	1.68E-13	T cells	Mea1
1.19E-17	-0.274778	0.087	0.155	1.68E-13	T cells	Hmgcl
1.27E-17	-0.2818406	0.092	0.161	1.80E-13	T cells	Relb
1.40E-17	0.26187191	0.218	0.143	1.98E-13	T cells	Nucks1
1.42E-17	0.33198442	0.153	0.091	2.01E-13	T cells	Hk1
1.79E-17	0.29163754	0.281	0.2	2.53E-13	T cells	Abcf1
1.80E-17	-0.3208962	0.079	0.143	2.55E-13	T cells	Txndc5
1.86E-17	-0.3002166	0.052	0.111	2.63E-13	T cells	Furin
2.00E-17	0.29681524	0.145	0.083	2.83E-13	T cells	Msi2
2.10E-17	-0.2742233	0.066	0.128	2.97E-13	T cells	Mapk3
2.39E-17	0.3024542	0.33	0.245	3.39E-13	T cells	Srp19
2.57E-17	-0.3123426	0.123	0.2	3.65E-13	T cells	Spag9
3.82E-17	0.25577825	0.237	0.161	5.40E-13	T cells	Pcnp
3.83E-17	0.27658032	0.324	0.241	5.43E-13	T cells	Eif4e2
3.90E-17	0.33752723	0.12	0.065	5.52E-13	T cells	Abcb1b
4.08E-17	0.25741506	0.458	0.362	5.78E-13	T cells	Cope
4.44E-17	-0.3692793	0.137	0.211	6.28E-13	T cells	Taf6l
4.47E-17	-0.2874443	0.143	0.222	6.33E-13	T cells	Jmjd1c
4.57E-17	0.53307893	0.289	0.213	6.46E-13	T cells	H2afx
4.83E-17	-0.38585	0.079	0.143	6.84E-13	T cells	Irgm1
5.28E-17	-0.3207245	0.139	0.216	7.48E-13	T cells	Zfp106
6.06E-17	0.32103785	0.266	0.186	8.58E-13	T cells	Hp1bp3
6.17E-17	0.29879141	0.131	0.074	8.73E-13	T cells	Znrf1
6.85E-17	0.29188359	0.295	0.214	9.70E-13	T cells	Prmt1
7.99E-17	0.31336818	0.259	0.181	1.13E-12	T cells	Smc1a

8.47E-17	-0.2672189	0.058	0.115	1.20E-12	T cells	Anxa4
8.68E-17	0.32532695	0.116	0.062	1.23E-12	T cells	Kif13b
9.16E-17	0.26386873	0.39	0.304	1.30E-12	T cells	Snrpd1
1.03E-16	0.3854239	0.187	0.121	1.46E-12	T cells	Ramp1
1.05E-16	0.25341392	0.128	0.072	1.49E-12	T cells	Desi1
1.13E-16	-0.2784302	0.068	0.129	1.59E-12	T cells	Gbp3
1.19E-16	0.26657469	0.246	0.17	1.69E-12	T cells	Mrpl15
1.20E-16	0.28543788	0.37	0.282	1.70E-12	T cells	Plekhj1
1.39E-16	-0.3861379	0.498	0.56	1.96E-12	T cells	Sqstm1
1.51E-16	0.27238099	0.183	0.117	2.13E-12	T cells	Glo1
1.60E-16	0.38720847	0.682	0.582	2.26E-12	T cells	Ifi27l2a
1.60E-16	-0.4294576	0.248	0.325	2.27E-12	T cells	Pold4
1.84E-16	0.28661212	0.166	0.103	2.60E-12	T cells	Dynll2
1.88E-16	-0.3060953	0.123	0.197	2.66E-12	T cells	Ypel5
1.92E-16	-0.5341094	0.244	0.321	2.72E-12	T cells	Card19
2.65E-16	-0.2852378	0.06	0.116	3.75E-12	T cells	Lpgat1
2.68E-16	-0.3337417	0.1	0.167	3.79E-12	T cells	Klhl24
2.71E-16	-0.3213297	0.098	0.165	3.84E-12	T cells	Casp1
2.82E-16	0.30981811	0.475	0.394	3.99E-12	T cells	Ube2s
3.29E-16	0.30637578	0.302	0.223	4.66E-12	T cells	Tpst2
3.36E-16	-0.5834389	0.486	0.498	4.75E-12	T cells	Atox1
3.58E-16	-0.2560681	0.05	0.104	5.07E-12	T cells	Gsap
3.82E-16	0.28176373	0.287	0.211	5.40E-12	T cells	Eif1ax
3.84E-16	0.25277932	0.173	0.108	5.44E-12	T cells	2310033P09I
3.84E-16	-0.3484851	0.245	0.161	5.44E-12	T cells	Gzma
3.86E-16	0.31500192	0.295	0.219	5.47E-12	T cells	Rnaseh2c
4.67E-16	-0.3890024	0.136	0.205	6.61E-12	T cells	Stt3b
4.71E-16	0.33668926	0.165	0.102	6.66E-12	T cells	Lonp2
6.17E-16	0.34046941	0.352	0.269	8.73E-12	T cells	Birc6
7.22E-16	0.29784046	0.223	0.153	1.02E-11	T cells	Dctn4
7.23E-16	0.3520181	0.365	0.28	1.02E-11	T cells	Smarca5
7.63E-16	-0.3935959	0.051	0.102	1.08E-11	T cells	Cpm
7.97E-16	0.26624237	0.142	0.085	1.13E-11	T cells	Thap3
8.20E-16	0.26102593	0.965	0.904	1.16E-11	T cells	Tmsb10
8.39E-16	0.33012373	0.414	0.335	1.19E-11	T cells	Ppp1cc
8.85E-16	0.25253907	0.369	0.284	1.25E-11	T cells	Psma4
9.68E-16	-0.2781581	0.1	0.165	1.37E-11	T cells	Atp6v1a
1.06E-15	0.32977214	0.374	0.29	1.51E-11	T cells	Pole4
1.07E-15	-0.2804932	0.058	0.114	1.51E-11	T cells	Dclre1c
1.07E-15	0.3088787	0.175	0.109	1.52E-11	T cells	Arap2
1.27E-15	-0.3089218	0.164	0.238	1.80E-11	T cells	Hnrnph2
1.36E-15	-0.2520257	0.061	0.117	1.93E-11	T cells	Tmem50b
1.39E-15	0.25421292	0.123	0.069	1.97E-11	T cells	Med14
1.46E-15	0.26931455	0.285	0.21	2.06E-11	T cells	Pdap1
1.51E-15	0.31906277	0.39	0.304	2.14E-11	T cells	Ncor1
1.53E-15	-0.6058246	0.338	0.399	2.16E-11	T cells	Zyx
1.53E-15	0.27370027	0.384	0.301	2.16E-11	T cells	Wdr83os
1.58E-15	0.36434324	0.497	0.412	2.24E-11	T cells	H2-Q4

1.70E-15	-0.3237892	0.109	0.175	2.40E-11	T cells	Oat
1.96E-15	-0.345775	0.142	0.211	2.78E-11	T cells	Dgkd
2.10E-15	0.298788	0.162	0.1	2.97E-11	T cells	11-Sep
2.15E-15	0.33192225	0.563	0.477	3.05E-11	T cells	Akap13
3.62E-15	0.27365461	0.304	0.228	5.13E-11	T cells	Psmc5
3.63E-15	0.26322219	0.365	0.279	5.13E-11	T cells	Dnajc3
3.68E-15	-0.3284725	0.116	0.184	5.21E-11	T cells	Metrnl
4.79E-15	0.26526651	0.267	0.191	6.78E-11	T cells	Fubp1
5.20E-15	-0.2626715	0.06	0.114	7.36E-11	T cells	Rnf216
5.32E-15	-0.3039632	0.113	0.178	7.53E-11	T cells	Stat6
5.47E-15	0.27650086	0.213	0.145	7.74E-11	T cells	Rsl1d1
6.58E-15	0.36485158	0.278	0.204	9.32E-11	T cells	Atp11b
7.57E-15	0.27654815	0.114	0.064	1.07E-10	T cells	Pja1
8.35E-15	0.27269981	0.139	0.084	1.18E-10	T cells	Elk3
9.64E-15	0.31550283	0.223	0.155	1.37E-10	T cells	Chfr
9.80E-15	0.25764727	0.292	0.217	1.39E-10	T cells	Ccdc59
1.00E-14	-0.2505969	0.092	0.155	1.42E-10	T cells	Cyb5r3
1.03E-14	0.42551565	0.152	0.094	1.45E-10	T cells	Hist1h1e
1.03E-14	0.32807006	0.188	0.127	1.46E-10	T cells	Arl4c
1.10E-14	-0.3479206	0.42	0.48	1.56E-10	T cells	Sh3glb1
1.25E-14	-0.2670729	0.073	0.13	1.77E-10	T cells	Fam49a
1.49E-14	-0.3340609	0.175	0.248	2.11E-10	T cells	Tonsl
1.74E-14	0.33799053	0.327	0.249	2.46E-10	T cells	Srpr
1.76E-14	0.27603752	0.25	0.181	2.50E-10	T cells	Actr10
1.99E-14	0.2764017	0.232	0.165	2.82E-10	T cells	Emc4
2.29E-14	-0.2655595	0.088	0.148	3.24E-10	T cells	Fam111a
2.33E-14	-0.3037411	0.133	0.197	3.30E-10	T cells	Vars
2.54E-14	0.25528873	0.301	0.227	3.59E-10	T cells	Ccdc124
2.57E-14	-0.4600204	0.085	0.138	3.64E-10	T cells	Trp53i11
2.72E-14	0.32064559	0.333	0.258	3.86E-10	T cells	Metap2
3.84E-14	-0.3368237	0.237	0.311	5.44E-10	T cells	Ddx3x
4.02E-14	0.25841025	0.366	0.289	5.69E-10	T cells	Bag1
4.05E-14	-0.3393338	0.135	0.201	5.74E-10	T cells	Il17ra
4.12E-14	-0.2705373	0.072	0.126	5.83E-10	T cells	Brwd1
4.49E-14	0.27988285	0.326	0.251	6.35E-10	T cells	Ufc1
4.51E-14	-0.5468596	0.214	0.279	6.38E-10	T cells	Glul
5.51E-14	-0.2961219	0.118	0.181	7.80E-10	T cells	Ddit3
5.66E-14	0.25050414	0.448	0.365	8.01E-10	T cells	Bzw1
6.03E-14	0.25954875	0.128	0.078	8.54E-10	T cells	Lxn
6.10E-14	-0.3100863	0.692	0.626	8.64E-10	T cells	S100a11
8.16E-14	0.32769007	0.304	0.232	1.15E-09	T cells	Anp32e
8.57E-14	0.26223736	0.142	0.089	1.21E-09	T cells	Frmd8
8.66E-14	0.27977215	0.146	0.091	1.23E-09	T cells	Cdkn2aip
8.71E-14	0.29867185	0.42	0.348	1.23E-09	T cells	Banf1
9.73E-14	0.42418861	0.462	0.379	1.38E-09	T cells	Ahnak
1.04E-13	-0.3330948	0.095	0.154	1.47E-09	T cells	Cd274
1.10E-13	0.25963791	0.116	0.067	1.55E-09	T cells	Rif1
1.11E-13	-0.2950769	0.12	0.182	1.57E-09	T cells	Dynlt1c

1.18E-13	0.35452832	0.178	0.119	1.68E-09	T cells	Kdm2b
1.30E-13	0.27260523	0.16	0.102	1.84E-09	T cells	Madd
1.35E-13	0.26281724	0.355	0.278	1.91E-09	T cells	Timm23
1.67E-13	-0.3013566	0.108	0.17	2.36E-09	T cells	Pxn
1.74E-13	-0.2859685	0.124	0.187	2.47E-09	T cells	Gsdmd
1.78E-13	0.2591089	0.457	0.378	2.53E-09	T cells	Skp1a
1.85E-13	0.27348923	0.16	0.104	2.62E-09	T cells	Man1a2
1.97E-13	0.26711673	0.504	0.411	2.78E-09	T cells	Hspd1
2.21E-13	0.26341869	0.252	0.181	3.13E-09	T cells	Sms
2.27E-13	0.39007291	0.459	0.377	3.21E-09	T cells	H2-Q6
2.27E-13	0.2792745	0.275	0.208	3.21E-09	T cells	Nhp2
2.37E-13	-0.28172	0.145	0.211	3.35E-09	T cells	Necap2
2.71E-13	0.27480701	0.25	0.184	3.84E-09	T cells	Pcmt1
2.80E-13	0.27338774	0.48	0.407	3.97E-09	T cells	Sec11c
3.04E-13	-0.3189701	0.101	0.159	4.30E-09	T cells	Chchd10
3.20E-13	0.25624614	0.224	0.162	4.52E-09	T cells	Mpdu1
3.83E-13	-0.5690157	0.261	0.313	5.43E-09	T cells	Anxa5
3.89E-13	0.26379478	0.183	0.125	5.50E-09	T cells	Arl4a
3.90E-13	0.27330716	0.2	0.138	5.52E-09	T cells	Smc3
4.33E-13	0.26271475	0.487	0.407	6.14E-09	T cells	Rgs2
4.49E-13	-0.3114909	0.529	0.592	6.36E-09	T cells	Cytip
4.75E-13	0.26284528	0.209	0.144	6.72E-09	T cells	Suco
4.75E-13	-0.3151354	0.446	0.504	6.73E-09	T cells	Arf1
5.63E-13	0.27678175	0.371	0.299	7.96E-09	T cells	Lman2
5.63E-13	-0.2738913	0.329	0.404	7.97E-09	T cells	Flna
5.94E-13	-0.3637447	0.189	0.256	8.41E-09	T cells	Sde2
6.47E-13	0.2684696	0.175	0.118	9.16E-09	T cells	Supt16
6.51E-13	-0.345372	0.062	0.109	9.22E-09	T cells	Mirt1
6.62E-13	-0.3172159	0.16	0.225	9.37E-09	T cells	Atp6ap2
8.35E-13	0.26098788	0.334	0.257	1.18E-08	T cells	Phf20l1
8.51E-13	-0.2605249	0.364	0.437	1.21E-08	T cells	Ptbp3
8.93E-13	0.26658246	0.309	0.237	1.26E-08	T cells	Esyt1
9.12E-13	0.35876506	0.181	0.122	1.29E-08	T cells	Lyst
9.61E-13	0.2589181	0.505	0.43	1.36E-08	T cells	Tmem258
9.85E-13	0.3652704	0.352	0.28	1.39E-08	T cells	Lrrfip1
1.06E-12	-0.3106874	0.543	0.579	1.51E-08	T cells	Gnai2
1.08E-12	-0.3176836	0.361	0.424	1.54E-08	T cells	Ptpn1
1.22E-12	0.31497073	0.25	0.186	1.72E-08	T cells	Pdcd4
1.34E-12	0.34812269	0.297	0.23	1.89E-08	T cells	Gpr65
1.47E-12	-0.2600443	0.107	0.165	2.09E-08	T cells	Ncoa3
1.89E-12	-0.2541229	0.163	0.228	2.67E-08	T cells	Rcsd1
1.93E-12	-0.399149	0.08	0.131	2.73E-08	T cells	Hist1h2bc
2.07E-12	0.25432245	0.229	0.166	2.93E-08	T cells	Nap1l4
2.07E-12	0.34080122	0.14	0.089	2.94E-08	T cells	Smg6
2.10E-12	-0.2673305	0.134	0.197	2.97E-08	T cells	Il10rb
2.60E-12	0.25498491	0.165	0.112	3.68E-08	T cells	Zdhhc3
2.77E-12	0.25915801	0.349	0.276	3.92E-08	T cells	Dusp11
2.81E-12	-0.6600958	0.331	0.378	3.98E-08	T cells	Cstb

3.16E-12	0.26129358	0.163	0.11	4.48E-08	T cells	Tm2d3
3.58E-12	0.30717909	0.352	0.278	5.07E-08	T cells	Mier1
3.65E-12	0.27528918	0.184	0.125	5.17E-08	T cells	Ccdc88c
3.84E-12	-0.2688902	0.25	0.318	5.44E-08	T cells	Vapa
4.14E-12	0.31000995	0.62	0.556	5.86E-08	T cells	Myh9
4.25E-12	-0.3520017	0.257	0.319	6.01E-08	T cells	M6pr
4.51E-12	-0.2689512	0.693	0.704	6.38E-08	T cells	Gng5
4.70E-12	-0.2529723	0.137	0.199	6.65E-08	T cells	Limd1
5.01E-12	-0.3328966	0.239	0.305	7.10E-08	T cells	Kras
6.39E-12	-0.2519048	0.207	0.276	9.04E-08	T cells	Cltc
6.54E-12	0.26781635	0.222	0.162	9.26E-08	T cells	Gspt1
6.87E-12	0.2548627	0.411	0.338	9.72E-08	T cells	Ddx24
8.67E-12	0.37074645	0.622	0.539	1.23E-07	T cells	Hsp90b1
8.68E-12	0.25631625	0.261	0.199	1.23E-07	T cells	Tmem208
9.60E-12	0.25417912	0.22	0.16	1.36E-07	T cells	Yipf3
9.68E-12	0.30849342	0.261	0.198	1.37E-07	T cells	Fnbp1
9.88E-12	-0.2550304	0.112	0.169	1.40E-07	T cells	Mapk14
1.11E-11	-0.3514861	0.739	0.749	1.57E-07	T cells	Ucp2
1.29E-11	-1.0620302	0.444	0.437	1.83E-07	T cells	Ctsb
1.32E-11	0.25222626	0.249	0.189	1.87E-07	T cells	Dnajc2
1.39E-11	0.26521987	0.169	0.115	1.97E-07	T cells	Cyld
1.48E-11	-0.5104636	0.061	0.105	2.10E-07	T cells	Myc
1.61E-11	-0.2547239	0.132	0.19	2.28E-07	T cells	Cmtm6
1.69E-11	0.26746645	0.26	0.196	2.40E-07	T cells	Rsbn1l
1.76E-11	0.26005804	0.373	0.306	2.49E-07	T cells	Epsti1
1.89E-11	0.28156676	0.267	0.203	2.68E-07	T cells	Actn4
1.98E-11	-0.2807558	0.133	0.192	2.80E-07	T cells	Ttc7
2.54E-11	-0.2511746	0.101	0.156	3.60E-07	T cells	Fem1c
3.28E-11	-0.2731866	0.148	0.206	4.64E-07	T cells	Spg21
3.51E-11	-0.2623252	0.087	0.138	4.98E-07	T cells	Slc43a2
4.02E-11	-0.2768074	0.107	0.161	5.69E-07	T cells	Trim12a
4.10E-11	0.25162928	0.406	0.34	5.80E-07	T cells	Rnasek
4.58E-11	-0.2516846	0.129	0.187	6.49E-07	T cells	Unc119
5.39E-11	0.2501033	0.559	0.491	7.63E-07	T cells	Ncl
6.00E-11	-0.2818178	0.468	0.52	8.49E-07	T cells	Pcbp1
7.47E-11	-0.2864609	0.113	0.168	1.06E-06	T cells	Mdm2
8.20E-11	-0.2584467	0.107	0.161	1.16E-06	T cells	Rin3
8.58E-11	0.34572638	0.301	0.238	1.21E-06	T cells	Rock1
8.86E-11	0.34573629	0.111	0.069	1.25E-06	T cells	Tmem64
8.92E-11	0.2883824	0.138	0.091	1.26E-06	T cells	Casp8ap2
1.26E-10	0.3218152	0.498	0.419	1.78E-06	T cells	Pdia3
1.50E-10	-0.3421953	0.198	0.258	2.13E-06	T cells	Etf1
1.54E-10	0.31533682	0.293	0.231	2.18E-06	T cells	Dock8
1.56E-10	0.38580664	0.28	0.219	2.21E-06	T cells	Atrx
1.61E-10	0.29098407	0.15	0.102	2.27E-06	T cells	Rasa1
1.64E-10	-0.3144649	0.227	0.285	2.32E-06	T cells	Rnh1
1.65E-10	0.26097921	0.249	0.193	2.34E-06	T cells	Mrpl12
2.06E-10	0.28616839	0.25	0.193	2.91E-06	T cells	Rgs10

2.73E-10	0.28353349	0.378	0.31	3.86E-06	T cells	Ythdc1
3.35E-10	-0.2723886	0.157	0.212	4.75E-06	T cells	Cers2
3.43E-10	0.2955341	0.446	0.383	4.85E-06	T cells	Sf3b2
3.66E-10	-0.2964404	0.059	0.101	5.18E-06	T cells	Bambi
3.89E-10	-0.3217455	0.369	0.429	5.50E-06	T cells	Txnip
4.03E-10	0.26075005	0.446	0.388	5.70E-06	T cells	Anp32b
4.46E-10	0.26314644	0.154	0.107	6.31E-06	T cells	Notch1
4.88E-10	-0.3035917	0.08	0.124	6.91E-06	T cells	Hist2h2aa1
5.24E-10	0.28538931	0.295	0.236	7.42E-06	T cells	Itgb7
5.93E-10	0.36357543	0.248	0.193	8.39E-06	T cells	Lnpep
6.15E-10	-0.3428017	0.457	0.507	8.71E-06	T cells	Ezr
6.41E-10	-0.2585954	0.087	0.134	9.07E-06	T cells	Pnkp
7.62E-10	-0.2513192	0.098	0.147	1.08E-05	T cells	Arhgef2
9.49E-10	-0.3224467	0.298	0.352	1.34E-05	T cells	Iscu
1.02E-09	0.25421916	0.124	0.082	1.44E-05	T cells	Xrn1
1.03E-09	-0.4083681	0.271	0.32	1.46E-05	T cells	Pgam1
1.19E-09	-0.2546963	0.106	0.156	1.68E-05	T cells	Phyh
1.42E-09	0.26750069	0.421	0.365	2.02E-05	T cells	Nap1l1
1.50E-09	-0.3984724	0.393	0.437	2.12E-05	T cells	Irf1
1.67E-09	-0.2820998	0.239	0.297	2.36E-05	T cells	Ifnar2
1.74E-09	0.2732129	0.395	0.329	2.46E-05	T cells	Rbm25
2.50E-09	-0.4466749	0.084	0.128	3.54E-05	T cells	Tnf
2.55E-09	-0.252765	0.067	0.108	3.61E-05	T cells	Sephs2
2.93E-09	0.28489703	0.2	0.149	4.14E-05	T cells	Arid5b
4.02E-09	0.35341438	0.258	0.206	5.70E-05	T cells	Baz1a
4.18E-09	-0.3404117	0.241	0.292	5.92E-05	T cells	Hmgn1
4.27E-09	0.25947905	0.269	0.215	6.05E-05	T cells	Ubn1
5.20E-09	-0.2966702	0.21	0.265	7.36E-05	T cells	Dnajc7
6.24E-09	-0.4031688	0.102	0.147	8.83E-05	T cells	Bag3
6.68E-09	-0.2749129	0.241	0.295	9.46E-05	T cells	Cmpk1
7.03E-09	-0.2999431	0.102	0.145	9.95E-05	T cells	Nrm
7.82E-09	0.28005249	0.225	0.172	0.00011074	T cells	Bod1l
1.53E-08	0.27821694	0.132	0.093	0.00021701	T cells	Mdfic
1.73E-08	-0.7131343	0.109	0.151	0.00024503	T cells	Adam8
2.81E-08	0.26249177	0.235	0.188	0.00039788	T cells	Sdf2
3.81E-08	0.39086212	0.42	0.367	0.00053909	T cells	Itga4
4.30E-08	-0.5286531	0.069	0.105	0.00060934	T cells	Sh2d3c
4.46E-08	-0.2754744	0.098	0.139	0.0006311	T cells	Carhsp1
4.74E-08	-0.2859753	0.273	0.32	0.00067049	T cells	Coro1b
5.24E-08	0.25217285	0.142	0.102	0.00074191	T cells	Trp53inp1
1.05E-07	-0.314765	0.15	0.194	0.00148988	T cells	Cdkn2d
1.10E-07	0.25511664	0.298	0.248	0.00155248	T cells	Orai1
1.11E-07	-0.3020256	0.071	0.107	0.00157133	T cells	Abtb1
1.34E-07	-0.397677	0.509	0.538	0.00189138	T cells	Ppp1r15a
2.03E-07	-0.3219279	0.274	0.316	0.00287969	T cells	Rbm38
2.36E-07	-0.3283474	0.376	0.419	0.0033404	T cells	Ifrd1
2.41E-07	-0.2555544	0.206	0.255	0.00340453	T cells	Ehd1
2.68E-07	-0.2730351	0.328	0.374	0.0037982	T cells	Rbms1

2.88E-07	-0.2536514	0.085	0.12	0.00407084	T cells	Cdt1
3.28E-07	-0.3182783	0.377	0.415	0.00464427	T cells	Slfn2
3.73E-07	-0.4238491	0.593	0.582	0.00528005	T cells	Cd37
4.61E-07	-0.3192339	0.246	0.289	0.00652713	T cells	Tmem123
4.64E-07	0.25461243	0.401	0.352	0.00657117	T cells	Hnrnpu
8.58E-07	0.27268509	0.188	0.147	0.01214009	T cells	Kmt2a
1.03E-06	-0.2595098	0.354	0.4	0.01459316	T cells	Cdc42se1
1.23E-06	-0.2693163	0.088	0.124	0.01744928	T cells	Dck
1.63E-06	-0.5976314	0.452	0.347	0.02305068	T cells	Pglyrp1
1.80E-06	0.50603194	0.149	0.116	0.02554431	T cells	Maf
2.51E-06	-0.398082	0.528	0.537	0.0355866	T cells	Pkm
3.81E-06	-0.2619329	0.13	0.165	0.05399435	T cells	Tcf3
5.80E-06	-0.7798911	0.278	0.309	0.08216516	T cells	Mxd1
7.14E-06	0.93863682	0.234	0.199	0.10109164	T cells	Hist1h2ap
1.06E-05	0.2914319	0.432	0.391	0.15015153	T cells	Stat3
1.75E-05	-0.2907709	0.691	0.694	0.24761652	T cells	mt-Nd5
2.42E-05	-0.3204312	0.275	0.311	0.34186824	T cells	Fam32a
4.43E-05	-0.2500524	0.187	0.224	0.62733401	T cells	7-Mar
5.57E-05	-0.3049829	0.168	0.203	0.78811992	T cells	Icam1
0.0001031	-0.4013536	0.927	0.926	1	T cells	Srgn
0.0001035	0.27314386	0.316	0.281	1	T cells	Mycbp2
0.00016881	-0.2684029	0.14	0.165	1	T cells	Atp2a3
0.0002832	-0.279716	0.263	0.292	1	T cells	Sod2
0.0004341	-0.2901735	0.56	0.548	1	T cells	Tagln2
0.00064818	-0.2682768	0.144	0.173	1	T cells	Entpd1
0.00253019	-0.4191775	0.912	0.851	1	T cells	Hspa8
0	4.83551895	0.977	0.026	0	B cells	Cd79a
0	3.90911137	0.845	0.023	0	B cells	Ly6d
0	3.80151333	0.902	0.016	0	B cells	Ebf1
0	3.50972356	0.844	0.068	0	B cells	Cd79b
0	3.27191535	0.866	0.046	0	B cells	H2-DMb2
0	3.034436	0.729	0.009	0	B cells	Fcmr
0	2.95801405	0.843	0.112	0	B cells	Ccr7
0	2.87270068	0.704	0.01	0	B cells	Ms4a1
0	2.8686785	0.773	0.039	0	B cells	H2-Ob
0	2.51804436	0.699	0.094	0	B cells	Mef2c
0	2.48392078	0.635	0.008	0	B cells	Cd19
0	2.38477644	0.64	0.05	0	B cells	Cd55
0	2.33682018	0.537	0.007	0	B cells	Fcer2a
0	2.21018829	0.568	0.017	0	B cells	Bank1
0	2.05972437	0.491	0.007	0	B cells	Blk
0	2.02502398	0.708	0.185	0	B cells	Cd83
0	2.02356828	0.477	0.01	0	B cells	Fcrla
0	2.01023513	0.489	0.01	0	B cells	Mzb1
0	1.93950125	0.748	0.267	0	B cells	Foxp1
0	1.92322377	0.45	0.006	0	B cells	Scd1
0	1.91948767	0.814	0.367	0	B cells	Serp1
0	1.88684101	0.527	0.052	0	B cells	Ralgps2

0	1.85852945	0.999	0.539	0	B cells	Cd74
0	1.84479608	0.58	0.076	0	B cells	Cd24a
0	1.74969057	0.918	0.555	0	B cells	Klf2
0	1.74353533	0.466	0.049	0	B cells	H2-Oa
0	1.73352953	0.457	0.032	0	B cells	Blnk
0	1.68660368	0.989	0.366	0	B cells	H2-Aa
0	1.68300755	0.385	0.026	0	B cells	Serpinb1a
0	1.62191853	0.324	0.004	0	B cells	Vpreb3
0	1.60123349	0.377	0.012	0	B cells	Siglecg
0	1.59141744	0.383	0.011	0	B cells	Snn
0	1.58617913	0.347	0.005	0	B cells	Pou2af1
0	1.58464119	0.361	0.034	0	B cells	Fam43a
0	1.56000185	0.356	0.005	0	B cells	Cxcr5
0	1.55583255	0.868	0.501	0	B cells	Cd37
0	1.5398093	0.42	0.059	0	B cells	Bach2
0	1.49112791	0.981	0.882	0	B cells	Rps27
0	1.41008248	0.322	0.005	0	B cells	Pax5
0	1.26605728	0.97	0.829	0	B cells	H3f3a
0	1.26436957	0.298	0.009	0	B cells	Cd22
0	1.16182464	0.983	0.361	0	B cells	H2-Eb1
0	1.15183999	0.974	0.864	0	B cells	Rps29
0	1.06761174	0.968	0.799	0	B cells	Rps28
0	1.01164923	0.978	0.35	0	B cells	H2-Ab1
0	0.96605027	0.968	0.837	0	B cells	Rpl35a
0	0.93603265	0.98	0.878	0	B cells	Rps20
0	0.91251389	0.972	0.864	0	B cells	Rpl21
0	-1.7897998	0.87	0.938	0	B cells	Gapdh
0	-2.1741996	0.009	0.564	0	B cells	Fyb
0	-2.5869766	0.028	0.586	0	B cells	Anxa2
0	-3.1575437	0.222	0.891	0	B cells	Fxyd5
0	-3.3490641	0.076	0.661	0	B cells	Lgals1
0	-3.3973365	0.009	0.585	0	B cells	Ms4a4b
0	-3.5596169	0.015	0.566	0	B cells	Cd3g
0	-3.8780157	0.082	0.773	0	B cells	S100a6
0	-4.2270345	0.097	0.708	0	B cells	AW112010
0	-4.6399093	0.016	0.598	0	B cells	Nkg7
0	-6.0812901	0.088	0.696	0	B cells	Ccl5
3.59E-304	1.16459022	0.253	0.005	5.08E-300	B cells	Spib
3.70E-304	0.78365741	0.982	0.927	5.24E-300	B cells	Rps24
4.68E-302	0.85310656	0.977	0.869	6.63E-298	B cells	Rps21
2.01E-299	-4.0514674	0.027	0.544	2.84E-295	B cells	Fcer1g
2.47E-299	1.37973951	0.932	0.734	3.50E-295	B cells	Jund
4.04E-296	-2.9913551	0.009	0.527	5.72E-292	B cells	Cd3d
1.38E-295	-3.0234766	0.009	0.527	1.96E-291	B cells	Cd3e
8.11E-290	-2.8048641	0.049	0.556	1.15E-285	B cells	Lgals3
8.68E-290	1.2727232	0.262	0.01	1.23E-285	B cells	Tnfrsf13c
2.48E-282	1.26771836	0.237	0.005	3.51E-278	B cells	Hs3st1
2.31E-281	1.57689039	0.513	0.144	3.27E-277	B cells	Ptp4a3

7.79E-281	0.89292822	0.971	0.833	1.10E-276	B cells	Rps19
2.67E-278	1.16070193	0.277	0.018	3.77E-274	B cells	Gga2
4.53E-278	1.62813004	0.676	0.283	6.41E-274	B cells	Rel
7.76E-273	-0.9931617	0.991	0.991	1.10E-268	B cells	Actb
5.68E-271	-2.6259527	0.013	0.502	8.04E-267	B cells	S100a4
8.31E-270	0.97311808	0.981	0.779	1.18E-265	B cells	Ly6e
9.23E-268	-4.1630133	0.023	0.508	1.31E-263	B cells	Ccl4
2.21E-263	1.37851324	0.341	0.046	3.13E-259	B cells	Fchsd2
1.74E-259	0.81913068	0.97	0.852	2.46E-255	B cells	Rpl38
3.54E-259	1.42513289	0.489	0.117	5.01E-255	B cells	S1pr1
1.06E-256	1.36074453	0.812	0.466	1.50E-252	B cells	Tsc22d3
2.21E-256	1.77084093	0.81	0.52	3.12E-252	B cells	Nr4a1
3.47E-254	0.85216782	0.96	0.828	4.91E-250	B cells	Rpl17
1.51E-251	-1.801209	0.283	0.697	2.13E-247	B cells	Npc2
3.32E-251	1.3596329	0.319	0.04	4.70E-247	B cells	B3gnt5
5.37E-251	-1.8299862	0.135	0.616	7.60E-247	B cells	Selplg
2.13E-250	1.39397601	0.807	0.486	3.02E-246	B cells	Zfp36l1
3.62E-249	1.39151326	0.422	0.094	5.12E-245	B cells	Cerk
3.81E-248	-2.2638663	0.007	0.464	5.39E-244	B cells	Lat
5.94E-246	-1.0296993	0.94	0.954	8.42E-242	B cells	Pfn1
1.36E-244	-1.3271033	0.65	0.846	1.93E-240	B cells	Myl6
8.65E-243	-1.935571	0.109	0.578	1.22E-238	B cells	Ctsd
6.29E-242	-2.1780428	0.06	0.522	8.91E-238	B cells	Id2
7.30E-241	-1.3081215	0.694	0.862	1.03E-236	B cells	Itm2b
2.57E-237	1.41298642	0.546	0.192	3.64E-233	B cells	Hmgn1
1.02E-236	-3.1347992	0.005	0.447	1.44E-232	B cells	Cd8b1
3.55E-236	-2.2390137	0.381	0.731	5.02E-232	B cells	S100a11
2.86E-228	1.42846905	0.347	0.064	4.05E-224	B cells	Hvcn1
1.61E-225	1.26758752	0.535	0.18	2.27E-221	B cells	Syk
3.10E-223	-0.8615018	0.983	0.983	4.38E-219	B cells	Tmsb4x
4.80E-222	0.98960552	0.223	0.013	6.80E-218	B cells	Fcrl1
1.68E-220	1.17133824	0.923	0.747	2.38E-216	B cells	Btg1
2.50E-218	0.69993276	0.97	0.869	3.53E-214	B cells	Rpl39
4.12E-217	-3.6775857	0.144	0.529	5.83E-213	B cells	Tyrobp
6.15E-216	1.1295882	0.279	0.035	8.71E-212	B cells	St6gal1
5.30E-214	1.12817735	0.258	0.028	7.50E-210	B cells	Btla
1.20E-213	1.34924761	0.506	0.185	1.70E-209	B cells	Snx2
1.43E-213	0.88337641	0.183	0.004	2.02E-209	B cells	H2-Eb2
1.75E-212	-2.2681132	0.006	0.416	2.48E-208	B cells	Ctsw
1.02E-211	0.69945609	0.968	0.835	1.44E-207	B cells	Rpl35
1.42E-211	-2.0315243	0.009	0.418	2.01E-207	B cells	Thy1
3.93E-210	-0.8946082	0.911	0.928	5.56E-206	B cells	B2m
3.09E-209	1.14812399	0.272	0.036	4.37E-205	B cells	Snx8
8.10E-209	1.19663316	0.261	0.03	1.15E-204	B cells	Cpm
8.12E-208	1.08324613	0.244	0.024	1.15E-203	B cells	Bcl11a
4.34E-206	1.35893512	0.394	0.102	6.14E-202	B cells	Ero1lb
1.62E-204	1.26721654	0.319	0.058	2.29E-200	B cells	Trp53i11
6.21E-204	1.23761568	0.304	0.051	8.79E-200	B cells	Irf4

1.79E-203	-2.3094791	0.054	0.465	2.53E-199	B cells	Rgs1
5.09E-203	-2.5906199	0.006	0.403	7.21E-199	B cells	Cd8a
7.32E-202	1.30563392	0.441	0.139	1.04E-197	B cells	Pkig
1.45E-200	0.67725798	0.978	0.889	2.06E-196	B cells	Rpl37a
1.07E-198	-1.5446999	0.041	0.45	1.51E-194	B cells	Ms4a6b
3.48E-198	1.11976768	0.466	0.131	4.92E-194	B cells	Satb1
3.10E-197	0.81427679	0.155	0.001	4.39E-193	B cells	Cr2
3.36E-197	-2.3578822	0.008	0.396	4.75E-193	B cells	Klrd1
1.99E-195	-2.2429196	0.133	0.532	2.81E-191	B cells	Ctsb
4.75E-195	1.27589083	0.459	0.15	6.73E-191	B cells	Prkcb
6.74E-195	1.35425531	0.365	0.088	9.54E-191	B cells	Dmxl1
5.84E-194	1.26616295	0.345	0.076	8.27E-190	B cells	Swap70
1.40E-193	-1.6671515	0.146	0.552	1.99E-189	B cells	Hcst
1.49E-193	0.63691293	0.974	0.861	2.11E-189	B cells	Rps13
1.33E-190	-1.5703579	0.305	0.663	1.88E-186	B cells	Tspo
1.53E-190	-1.7395241	0.065	0.459	2.17E-186	B cells	Ptms
3.27E-190	-1.1147273	0.645	0.823	4.63E-186	B cells	Arpc1b
4.30E-189	-3.1388418	0.022	0.399	6.08E-185	B cells	Ifitm2
2.03E-188	-1.7668435	0.029	0.411	2.87E-184	B cells	Lck
8.27E-186	-2.8810434	0.022	0.394	1.17E-181	B cells	Alox5ap
1.92E-184	0.62772363	0.972	0.871	2.72E-180	B cells	Rpl27a
3.18E-183	1.31220903	0.613	0.314	4.50E-179	B cells	Snx5
4.06E-183	1.13453035	0.349	0.084	5.75E-179	B cells	Sh3bp5
3.99E-182	-3.387887	0.034	0.403	5.64E-178	B cells	Ifitm3
1.03E-179	1.19508412	0.701	0.396	1.46E-175	B cells	Dusp2
1.59E-177	0.79145469	0.148	0.002	2.24E-173	B cells	Chst3
3.67E-177	1.13660338	0.798	0.565	5.20E-173	B cells	Stk17b
4.99E-177	0.58212977	0.977	0.914	7.07E-173	B cells	Eef1a1
1.11E-176	-1.8782717	0.006	0.363	1.58E-172	B cells	Cst7
4.90E-176	-1.5980514	0.168	0.551	6.94E-172	B cells	Txn1
6.41E-174	-2.2674872	0.004	0.355	9.07E-170	B cells	Pdcd1
7.96E-174	1.07561568	0.533	0.201	1.13E-169	B cells	Sell
8.08E-174	-4.4618532	0.078	0.433	1.14E-169	B cells	Lyz2
1.70E-173	1.0117933	0.732	0.427	2.41E-169	B cells	Serinc3
2.77E-173	-2.0208649	0.005	0.355	3.93E-169	B cells	Tigit
4.93E-173	1.03997681	0.715	0.391	6.97E-169	B cells	Ets1
7.51E-172	0.36738384	0.993	0.883	1.06E-167	B cells	mt-Atp8
6.13E-170	1.2923029	0.464	0.169	8.68E-166	B cells	P2ry10
7.77E-170	-1.9354583	0.003	0.347	1.10E-165	B cells	Icos
1.07E-169	-1.7489902	0.009	0.357	1.51E-165	B cells	Lilrb4a
3.70E-169	-1.7172533	0.013	0.362	5.24E-165	B cells	Fgl2
3.25E-168	1.19725988	0.528	0.24	4.60E-164	B cells	Smim14
7.89E-168	-1.8985554	0.065	0.425	1.12E-163	B cells	Sh2d2a
2.48E-166	0.4695886	0.968	0.811	3.52E-162	B cells	mt-Nd4l
5.90E-166	0.47438507	0.989	0.977	8.35E-162	B cells	Fau
3.89E-164	-1.5077151	0.113	0.486	5.51E-160	B cells	Itgb2
4.16E-163	1.28614407	0.467	0.181	5.89E-159	B cells	Tcp11l2
1.41E-162	1.08966921	0.569	0.271	1.99E-158	B cells	mt-Nd3

3.77E-161	-1.7834833	0.005	0.337	5.34E-157	B cells	Il2rb
1.04E-160	-2.6869356	0.011	0.345	1.47E-156	B cells	Ccl3
1.85E-160	0.59223089	0.974	0.839	2.61E-156	B cells	Rpl36
1.18E-159	-1.3759673	0.006	0.335	1.68E-155	B cells	Skap1
9.85E-159	-1.5914895	0.007	0.336	1.39E-154	B cells	Itk
2.11E-157	0.55669282	0.975	0.886	2.98E-153	B cells	Rpl34
8.45E-157	-1.2246969	0.004	0.328	1.20E-152	B cells	Lcp2
1.83E-156	0.75843504	0.919	0.786	2.59E-152	B cells	Ddx5
2.24E-155	-2.1441738	0.039	0.379	3.18E-151	B cells	Isg15
9.90E-155	0.87425408	0.43	0.14	1.40E-150	B cells	Cd81
1.01E-153	1.11399752	0.335	0.093	1.42E-149	B cells	Pxk
2.84E-153	-3.0102453	0.013	0.335	4.02E-149	B cells	Ccl6
5.21E-153	1.21103003	0.636	0.347	7.38E-149	B cells	Cd69
1.07E-152	-1.400829	0.04	0.379	1.51E-148	B cells	Glrx
4.91E-152	-2.4003117	0.011	0.329	6.95E-148	B cells	Ier3
1.19E-151	0.92991214	0.598	0.274	1.68E-147	B cells	Unc93b1
4.70E-151	-1.4633361	0.006	0.322	6.65E-147	B cells	Prkch
4.91E-151	-1.602093	0.079	0.431	6.95E-147	B cells	Itgb1
8.64E-151	1.49155045	0.27	0.057	1.22E-146	B cells	Tsc22d1
2.32E-150	-1.5233983	0.002	0.315	3.28E-146	B cells	Cd28
4.45E-150	0.68192032	0.931	0.747	6.29E-146	B cells	Rpl36a
5.91E-150	-1.3112186	0.331	0.647	8.37E-146	B cells	Psme2
8.58E-150	-1.4326541	0.083	0.429	1.21E-145	B cells	Efhd2
8.86E-150	0.55167723	0.977	0.925	1.25E-145	B cells	Rpl18a
7.26E-149	-1.4410211	0.103	0.459	1.03E-144	B cells	Zyx
7.35E-149	0.91688532	0.575	0.253	1.04E-144	B cells	Napsa
9.23E-149	0.9299618	0.211	0.03	1.31E-144	B cells	Txndc16
3.05E-148	0.54887151	0.981	0.881	4.32E-144	B cells	Rps7
3.34E-148	0.71074051	0.914	0.709	4.73E-144	B cells	Rpl12
8.41E-148	-1.5418371	0.034	0.36	1.19E-143	B cells	Emb
8.96E-148	0.6395502	0.132	0.004	1.27E-143	B cells	Bcar3
4.77E-147	-1.4077404	0.239	0.57	6.75E-143	B cells	Atox1
9.58E-147	0.81457864	0.141	0.006	1.36E-142	B cells	B3gnt7
1.00E-145	-1.3385844	0.55	0.754	1.42E-141	B cells	H2afz
1.84E-144	1.01233762	0.545	0.252	2.61E-140	B cells	Cxcr4
2.25E-144	1.18823335	0.323	0.091	3.18E-140	B cells	Rasgef1b
1.66E-143	-2.5117145	0.005	0.308	2.35E-139	B cells	Gzmb
3.54E-143	-4.1246306	0.034	0.343	5.01E-139	B cells	Il1b
1.98E-141	-1.4307884	0.003	0.301	2.81E-137	B cells	Cd27
3.64E-141	1.08404088	0.286	0.07	5.15E-137	B cells	Eif2ak3
4.07E-141	0.51412729	0.981	0.933	5.76E-137	B cells	Uba52
5.33E-141	0.68070307	0.909	0.73	7.54E-137	B cells	Eef2
6.26E-141	1.10580057	0.442	0.179	8.86E-137	B cells	Add3
1.13E-140	-1.7091484	0.009	0.308	1.60E-136	B cells	Lilr4b
2.03E-140	1.05945329	0.676	0.433	2.87E-136	B cells	Ezr
3.36E-140	0.59705923	0.984	0.954	4.75E-136	B cells	H3f3b
4.39E-140	-1.2009855	0.397	0.681	6.22E-136	B cells	Ldha
9.10E-140	1.12465517	0.358	0.121	1.29E-135	B cells	Snx9

1.03E-139	1.08950459	0.337	0.104	1.46E-135	B cells	Man1a
1.29E-139	-1.3290296	0.064	0.398	1.82E-135	B cells	Fosl2
3.71E-138	0.46008159	0.981	0.93	5.26E-134	B cells	Rps16
5.47E-137	-1.9187937	0.027	0.328	7.74E-133	B cells	Lst1
1.91E-136	-0.8501977	0.898	0.953	2.71E-132	B cells	Actg1
5.34E-136	0.8249218	0.617	0.295	7.56E-132	B cells	H2-DMa
1.13E-135	-1.3844621	0.004	0.292	1.60E-131	B cells	Tnfrsf1a
9.38E-135	-1.9771657	0.012	0.304	1.33E-130	B cells	Ctla4
3.67E-134	-1.6910922	0.007	0.294	5.19E-130	B cells	Fcgr3
7.45E-134	-1.7876825	0.003	0.287	1.06E-129	B cells	Lag3
1.63E-133	-1.4734202	0.027	0.324	2.30E-129	B cells	Tnfrsf18
6.98E-133	-1.8694633	0.137	0.46	9.89E-129	B cells	Pglyrp1
8.11E-133	-1.1512972	0.116	0.456	1.15E-128	B cells	Leprotl1
9.81E-133	0.9378029	0.223	0.042	1.39E-128	B cells	Zfp318
2.86E-132	0.70413435	0.177	0.022	4.05E-128	B cells	Aff3
9.46E-132	0.48792223	0.98	0.904	1.34E-127	B cells	Rpl30
2.49E-131	-1.1681608	0.144	0.48	3.53E-127	B cells	S100a13
2.62E-131	-1.0256917	0.581	0.764	3.70E-127	B cells	Clic1
3.65E-131	0.94541951	0.209	0.036	5.17E-127	B cells	Pgap1
8.93E-131	-1.2216639	0.005	0.287	1.26E-126	B cells	Cd247
1.01E-130	-1.6025051	0.239	0.556	1.43E-126	B cells	Prdx5
2.01E-130	-1.4513633	0.056	0.363	2.84E-126	B cells	Bcl2a1d
1.38E-128	0.93099896	0.265	0.066	1.96E-124	B cells	Cnn3
2.00E-128	0.90501607	0.23	0.046	2.83E-124	B cells	Mtss1
4.62E-128	0.83559048	0.138	0.01	6.54E-124	B cells	Dennd5b
2.14E-127	-1.4532751	0.013	0.294	3.03E-123	B cells	Cd68
4.30E-127	-1.7104738	0.003	0.275	6.08E-123	B cells	Cxcr6
6.68E-127	-2.3543707	0.096	0.402	9.46E-123	B cells	Msrb1
6.98E-127	0.89360603	0.268	0.067	9.88E-123	B cells	Cfap43
7.53E-127	0.87751862	0.245	0.054	1.07E-122	B cells	Cmah
6.93E-126	0.47097984	0.985	0.928	9.81E-122	B cells	Rps8
1.04E-124	-1.4521452	0.173	0.5	1.47E-120	B cells	Nr4a2
1.61E-124	0.78804337	0.792	0.587	2.28E-120	B cells	Cnbp
2.26E-124	0.53940358	0.971	0.857	3.20E-120	B cells	Rpl13a
2.30E-124	-1.0768849	0.456	0.701	3.25E-120	B cells	Myl12a
9.17E-124	0.82985975	0.22	0.044	1.30E-119	B cells	Cyb561a3
2.34E-123	0.98889282	0.467	0.209	3.31E-119	B cells	Rasgrp2
9.10E-123	0.48172359	0.973	0.898	1.29E-118	B cells	Rps10
1.08E-122	-0.706038	0.863	0.898	1.52E-118	B cells	Cfl1
3.10E-122	-1.400024	0.123	0.436	4.39E-118	B cells	Bcl2a1b
1.00E-121	0.48467	0.983	0.925	1.42E-117	B cells	Rpl13
1.16E-121	-1.3600591	0.25	0.572	1.64E-117	B cells	Ifngr1
4.12E-121	-1.0708796	0.009	0.276	5.84E-117	B cells	Rgs10
5.01E-121	1.10811373	0.483	0.246	7.09E-117	B cells	Tmod3
5.04E-121	0.44405073	0.977	0.926	7.14E-117	B cells	Rpl23
5.06E-121	-1.3701648	0.159	0.485	7.16E-117	B cells	Ahnak
1.87E-120	0.91679207	0.211	0.041	2.65E-116	B cells	Treml2
2.53E-120	0.49323829	0.971	0.878	3.59E-116	B cells	Rps26

4.29E-120	0.84693382	0.554	0.276	6.08E-116	B cells	Lyn
2.10E-119	0.53548527	0.957	0.821	2.97E-115	B cells	Rpl26
2.22E-119	-1.7446942	0.002	0.262	3.14E-115	B cells	Ctla2a
3.26E-119	0.44309909	0.936	0.789	4.61E-115	B cells	mt-Nd2
1.12E-118	0.45287657	0.977	0.915	1.59E-114	B cells	Rpl37
2.52E-118	1.04276893	0.294	0.091	3.57E-114	B cells	Chchd10
3.40E-118	0.74147746	0.159	0.02	4.81E-114	B cells	Tmem108
7.20E-117	0.48115808	0.978	0.879	1.02E-112	B cells	Rpl8
1.36E-116	-1.8870822	0.006	0.263	1.93E-112	B cells	Tgfbi
1.39E-116	-1.7174134	0.022	0.286	1.96E-112	B cells	Ms4a6c
1.93E-116	0.77968471	0.183	0.03	2.73E-112	B cells	Lrrk2
2.56E-116	0.8955159	0.233	0.056	3.62E-112	B cells	Btk
2.16E-115	0.76582461	0.176	0.028	3.06E-111	B cells	A530040E14I
8.17E-115	0.91292919	0.379	0.147	1.16E-110	B cells	Aldh2
3.86E-114	1.02270464	0.368	0.148	5.47E-110	B cells	Abhd17b
4.46E-114	-1.2658789	0.448	0.696	6.31E-110	B cells	Vim
2.18E-113	1.06571323	0.406	0.175	3.09E-109	B cells	Stap1
3.48E-113	0.50426552	0.977	0.861	4.93E-109	B cells	Ptma
4.50E-113	0.4249367	0.98	0.939	6.37E-109	B cells	Gm10076
6.82E-113	0.60278245	0.113	0.006	9.65E-109	B cells	Rasgrp3
1.50E-112	0.99355267	0.26	0.071	2.12E-108	B cells	Irs2
2.37E-112	0.57963344	0.111	0.006	3.35E-108	B cells	Trim7
5.25E-112	0.57571381	0.112	0.006	7.44E-108	B cells	Pxdc1
1.56E-111	-0.9354908	0.412	0.664	2.20E-107	B cells	Arf5
4.10E-111	0.98722287	0.354	0.136	5.80E-107	B cells	Dgkd
9.37E-111	-0.8326043	0.651	0.801	1.33E-106	B cells	Myl12b
4.74E-110	1.06592806	0.342	0.131	6.71E-106	B cells	Stt3b
3.19E-108	-1.0018677	0.011	0.255	4.51E-104	B cells	Acot7
6.97E-108	-1.8275431	0.005	0.244	9.87E-104	B cells	Ifng
7.31E-108	0.7885094	0.224	0.054	1.04E-103	B cells	Wdfy4
1.16E-107	0.93807038	0.478	0.235	1.65E-103	B cells	Rhob
1.79E-107	-0.7480515	0.904	0.92	2.53E-103	B cells	Cd52
3.31E-107	-0.9952472	0.035	0.297	4.69E-103	B cells	Vsir
3.83E-107	-1.216059	0.001	0.236	5.42E-103	B cells	Tox
7.51E-107	0.74787137	0.652	0.404	1.06E-102	B cells	Ier5
1.90E-106	-3.3251916	0.016	0.257	2.69E-102	B cells	C1qb
9.52E-106	1.34801615	0.308	0.107	1.35E-101	B cells	Plk2
9.76E-106	0.98984805	0.659	0.46	1.38E-101	B cells	Fam107b
2.18E-105	-1.1899501	0.367	0.628	3.09E-101	B cells	H2-Q7
2.47E-105	0.823983	0.214	0.05	3.50E-101	B cells	Ttpal
2.89E-105	1.0243354	0.471	0.248	4.09E-101	B cells	4930523C07F
5.47E-105	0.64483797	0.134	0.015	7.74E-101	B cells	Sbk1
2.47E-104	-0.9967439	0.582	0.729	3.50E-100	B cells	Psmb8
8.07E-104	1.00211271	0.358	0.151	1.14E-99	B cells	Cnp
1.03E-103	-2.0700332	0.023	0.266	1.45E-99	B cells	Ly6c2
1.06E-103	1.06169603	0.784	0.605	1.50E-99	B cells	Hspe1
3.43E-103	-1.4509931	0.059	0.321	4.85E-99	B cells	Cdkn1a
5.37E-103	-1.9572212	0.009	0.243	7.60E-99	B cells	Cd14

8.95E-103	1.00314119	0.48	0.247	1.27E-98	B cells	Gm8369
1.58E-102	0.46559787	0.978	0.885	2.23E-98	B cells	Rps5
1.84E-102	-1.2511735	0.127	0.408	2.60E-98	B cells	Tpi1
6.62E-102	0.99606926	0.429	0.216	9.38E-98	B cells	Ppp3ca
4.77E-101	-0.9459519	0.185	0.495	6.76E-97	B cells	Arl6ip5
5.48E-101	-3.8543106	0.014	0.245	7.76E-97	B cells	Gzma
1.14E-100	0.7322388	0.612	0.351	1.61E-96	B cells	Gimap6
1.28E-100	1.08573269	0.558	0.364	1.81E-96	B cells	Bri3
1.75E-100	-2.9223947	0.006	0.232	2.48E-96	B cells	C1qa
3.65E-100	-1.3915429	0.005	0.231	5.17E-96	B cells	Csf1r
4.01E-100	-1.5055855	0.005	0.229	5.68E-96	B cells	Pla2g7
4.82E-100	-1.5942348	0.004	0.228	6.82E-96	B cells	Cd7
5.00E-100	-0.9406749	0.083	0.361	7.08E-96	B cells	Pycard
7.28E-100	0.803505	0.651	0.425	1.03E-95	B cells	Pde4b
1.53E-99	-0.9954105	0.019	0.255	2.16E-95	B cells	Spn
2.03E-99	0.99321653	0.434	0.225	2.88E-95	B cells	Mif4gd
3.13E-99	-0.9517885	0.014	0.245	4.43E-95	B cells	Rinl
3.42E-99	-1.2722106	0.024	0.262	4.84E-95	B cells	Plin2
5.01E-99	-1.8584708	0.018	0.249	7.09E-95	B cells	Mt1
5.82E-99	-0.8915106	0.041	0.293	8.23E-95	B cells	9-Sep
9.74E-99	0.84071454	0.229	0.061	1.38E-94	B cells	Xylt1
1.32E-98	-1.478277	0.096	0.369	1.87E-94	B cells	Grina
1.80E-98	-1.0079732	0.237	0.524	2.55E-94	B cells	Vamp8
5.02E-98	0.9172397	0.296	0.109	7.11E-94	B cells	Tmem243
5.61E-98	-1.1009504	0.193	0.49	7.94E-94	B cells	Mrpl33
8.78E-98	-1.5003678	0.005	0.225	1.24E-93	B cells	Prf1
1.12E-97	-1.0484653	0.065	0.325	1.59E-93	B cells	Ptpn22
1.67E-97	-0.9635313	0.037	0.282	2.37E-93	B cells	Glipr1
2.77E-97	-0.9798525	0.002	0.22	3.92E-93	B cells	Nrp1
3.02E-97	-2.7558859	0.005	0.225	4.28E-93	B cells	C1qc
4.79E-97	-0.9540023	0.002	0.219	6.79E-93	B cells	Zap70
9.81E-97	0.96729519	0.283	0.1	1.39E-92	B cells	Parp1
1.02E-96	-0.8644279	0.726	0.819	1.45E-92	B cells	Rac2
1.27E-96	-1.0004492	0.046	0.294	1.80E-92	B cells	Nfil3
1.63E-96	-0.8453779	0.021	0.253	2.31E-92	B cells	Itm2c
5.70E-96	0.50952193	0.953	0.782	8.07E-92	B cells	Rpl22
7.89E-96	-1.0752021	0.09	0.355	1.12E-91	B cells	Anxa5
1.19E-95	-0.8521096	0.756	0.818	1.69E-91	B cells	Cyba
1.24E-95	0.90553685	0.322	0.129	1.76E-91	B cells	Vars
2.68E-95	1.0054382	0.362	0.158	3.80E-91	B cells	Foxo1
4.51E-95	-0.876916	0.016	0.243	6.38E-91	B cells	Myo1f
1.05E-94	-1.2754965	0.002	0.215	1.49E-90	B cells	Tnfrsf9
1.19E-94	0.56591759	0.124	0.014	1.69E-90	B cells	Kmo
1.42E-94	0.85910466	0.583	0.388	2.01E-90	B cells	Arf6
1.50E-94	0.61453508	0.827	0.656	2.12E-90	B cells	Rpl4
1.76E-94	-0.9804491	0.006	0.222	2.50E-90	B cells	Lrrc25
2.13E-94	1.61024098	0.66	0.491	3.01E-90	B cells	Dnajb1
2.44E-94	-1.1067232	0.344	0.6	3.45E-90	B cells	Prr13

2.98E-94	0.87816688	0.263	0.086	4.22E-90	B cells	Tcf4
6.90E-94	-0.9490043	0.099	0.369	9.77E-90	B cells	Cyth4
1.13E-93	1.2605653	0.519	0.313	1.61E-89	B cells	Hsph1
2.12E-93	0.65145009	0.141	0.021	2.99E-89	B cells	Slc9a7
2.24E-93	0.88392702	0.467	0.251	3.16E-89	B cells	Rbm38
3.88E-93	-1.4101582	0.006	0.218	5.49E-89	B cells	Slc16a3
4.98E-93	0.41818382	0.984	0.913	7.05E-89	B cells	Rps3a1
6.89E-93	0.80327166	0.625	0.424	9.75E-89	B cells	Gdi2
2.28E-92	0.45306441	0.97	0.856	3.23E-88	B cells	Rpl28
3.26E-92	-0.7458429	0.025	0.253	4.61E-88	B cells	Hpcal1
7.24E-92	-0.9292659	0.054	0.3	1.02E-87	B cells	Zbp1
2.78E-91	0.47644358	0.831	0.651	3.93E-87	B cells	mt-Nd5
4.67E-91	0.95899483	0.916	0.86	6.62E-87	B cells	Hspa8
9.38E-91	-0.8871887	0.184	0.472	1.33E-86	B cells	Rnaset2b
1.30E-90	-0.8530838	0.058	0.305	1.85E-86	B cells	Arrb2
5.79E-90	-1.2324608	0.007	0.214	8.20E-86	B cells	Aif1
1.43E-89	-0.8387772	0.08	0.338	2.03E-85	B cells	Dnajc15
3.68E-89	-1.3698242	0.01	0.218	5.21E-85	B cells	Gsr
5.45E-89	0.77165559	0.718	0.524	7.72E-85	B cells	Cytip
6.43E-89	-0.9088805	0.291	0.565	9.10E-85	B cells	Abracl
6.96E-89	-0.8128836	0.459	0.677	9.85E-85	B cells	Capzb
2.59E-88	0.93233955	0.369	0.169	3.67E-84	B cells	Trim25
2.97E-88	-1.0805877	0.005	0.206	4.21E-84	B cells	Klrk1
6.46E-88	-1.2794258	0.005	0.206	9.14E-84	B cells	Fcgr4
1.48E-87	0.82174371	0.197	0.051	2.09E-83	B cells	Snx30
2.43E-87	-0.9491847	0.373	0.619	3.44E-83	B cells	Emp3
6.39E-87	-3.4139559	0.022	0.231	9.04E-83	B cells	Cxcl2
7.44E-87	0.48066289	0.936	0.806	1.05E-82	B cells	Rpl7
2.50E-86	-0.8496376	0.014	0.22	3.54E-82	B cells	Lgals3bp
2.68E-86	-1.0881858	0.341	0.592	3.79E-82	B cells	Pkm
3.73E-86	-2.0971941	0.007	0.206	5.28E-82	B cells	Hp
5.32E-86	-0.9220915	0.068	0.308	7.53E-82	B cells	Zgpat
8.13E-86	-1.5257741	0.006	0.204	1.15E-81	B cells	Mafb
2.36E-85	1.20872688	0.201	0.055	3.34E-81	B cells	Myc
5.57E-85	-1.1710232	0.102	0.352	7.89E-81	B cells	Cd44
6.94E-85	0.572253	0.115	0.014	9.82E-81	B cells	Zbtb10
7.33E-85	-0.815434	0.007	0.205	1.04E-80	B cells	Metrnl
1.30E-84	-0.8882866	0.003	0.196	1.84E-80	B cells	Havcr2
2.13E-84	0.9992314	0.394	0.205	3.01E-80	B cells	Sp140
2.87E-84	-1.0089214	0.006	0.201	4.07E-80	B cells	App
4.05E-84	0.60573148	0.792	0.633	5.73E-80	B cells	Ywhaz
4.31E-84	-0.6623933	0.698	0.795	6.10E-80	B cells	Arpc2
1.12E-83	0.83415185	0.294	0.115	1.59E-79	B cells	Atp2a3
1.39E-83	0.40835449	0.972	0.868	1.97E-79	B cells	Rpl32
1.72E-83	0.94787611	0.421	0.229	2.43E-79	B cells	Tmem123
8.33E-83	-0.8621844	0.377	0.627	1.18E-78	B cells	Tmem50a
1.57E-82	-1.0080821	0.006	0.198	2.23E-78	B cells	Ckb
1.58E-82	-0.9375629	0.554	0.703	2.24E-78	B cells	Aldoa

1.58E-82	-1.5905003	0.008	0.202	2.24E-78	B cells	Tnfrsf4
2.42E-82	0.5675332	0.128	0.02	3.42E-78	B cells	Cnr2
2.66E-82	-1.1531858	0.02	0.222	3.76E-78	B cells	Mpeg1
2.02E-81	-0.9055107	0.348	0.588	2.85E-77	B cells	Prelid1
2.91E-81	-0.8675892	0.065	0.296	4.12E-77	B cells	Batf
3.59E-81	-0.9606648	0.03	0.237	5.09E-77	B cells	Csf2ra
5.79E-81	0.92312283	0.379	0.19	8.20E-77	B cells	Camk2d
5.99E-81	-0.8246263	0.181	0.449	8.48E-77	B cells	Tmem14c
1.34E-80	-0.9265051	0.003	0.188	1.90E-76	B cells	Clec4a3
1.44E-80	0.40375256	0.981	0.891	2.04E-76	B cells	Rpl6
1.46E-80	-0.8116353	0.172	0.444	2.07E-76	B cells	Chmp4b
1.68E-80	-0.8508492	0.054	0.278	2.38E-76	B cells	D16Ertd472e
3.75E-80	-0.8261813	0.53	0.699	5.31E-76	B cells	Psme1
5.86E-80	-2.0420793	0.451	0.606	8.30E-76	B cells	Cst3
1.06E-79	-0.7597741	0.09	0.332	1.50E-75	B cells	Tmem256
1.12E-79	-0.9540462	0.005	0.191	1.58E-75	B cells	Cd5
1.44E-79	-0.8411587	0.072	0.304	2.04E-75	B cells	Prex1
3.46E-79	-0.7954893	0.004	0.188	4.90E-75	B cells	Ramp1
3.90E-79	0.71644042	0.192	0.054	5.52E-75	B cells	Tnfrsf13b
4.16E-79	0.60644323	0.728	0.541	5.89E-75	B cells	Pcbp2
5.41E-79	-0.9248403	0.039	0.249	7.65E-75	B cells	Rbpj
6.93E-79	0.87833403	0.391	0.202	9.81E-75	B cells	Cyth1
7.40E-79	-1.3471126	0.012	0.202	1.05E-74	B cells	Ccrl2
8.05E-79	0.68941995	0.663	0.485	1.14E-74	B cells	Hnrnpf
1.45E-78	0.60020641	0.125	0.02	2.06E-74	B cells	Cxxc5
1.54E-78	-0.7855587	0.001	0.181	2.19E-74	B cells	Sh2d1a
1.70E-78	-1.0163173	0.09	0.324	2.41E-74	B cells	Itgal
7.92E-78	0.86165461	0.447	0.254	1.12E-73	B cells	Tgif1
2.38E-77	-1.965612	0.004	0.182	3.37E-73	B cells	Cxcl9
8.64E-77	-0.448119	0.98	0.972	1.22E-72	B cells	Ubb
9.89E-77	0.36518696	0.981	0.903	1.40E-72	B cells	Rpl19
1.10E-76	0.89408844	0.279	0.115	1.56E-72	B cells	Tcf3
1.19E-76	0.54730597	0.847	0.704	1.69E-72	B cells	Rps15
1.97E-76	0.48369906	0.943	0.833	2.78E-72	B cells	Rps12
2.08E-76	-0.7723529	0.018	0.207	2.95E-72	B cells	Ubash3b
3.21E-76	0.78732796	0.221	0.073	4.54E-72	B cells	Cdt1
5.08E-76	0.7923079	0.464	0.273	7.19E-72	B cells	Eif4a2
5.24E-76	-0.9553777	0.261	0.528	7.42E-72	B cells	Tln1
5.29E-76	0.8650706	0.39	0.206	7.49E-72	B cells	Rabgap1l
5.51E-76	-0.8609154	0.358	0.595	7.80E-72	B cells	Ostf1
7.40E-76	-0.8337388	0.052	0.265	1.05E-71	B cells	Ifi47
9.48E-76	0.38033978	0.966	0.927	1.34E-71	B cells	Rps9
1.22E-75	-0.8813839	0.109	0.348	1.73E-71	B cells	Card19
2.10E-75	-0.837465	0.006	0.183	2.98E-71	B cells	Lair1
2.16E-75	-0.9053462	0.005	0.181	3.06E-71	B cells	Ccr2
3.93E-75	-0.8563749	0.018	0.205	5.57E-71	B cells	Entpd1
4.47E-75	-1.7921267	0.005	0.18	6.33E-71	B cells	Tnfaip2
1.52E-74	-0.7006779	0.011	0.191	2.16E-70	B cells	Arl4c

2.16E-74	0.5129694	0.485	0.253	3.06E-70	B cells	Ly86
2.45E-74	0.38731296	0.977	0.891	3.47E-70	B cells	Rps4x
2.67E-74	0.50300806	0.867	0.721	3.78E-70	B cells	Rpl9
3.03E-74	-1.1526832	0.003	0.175	4.29E-70	B cells	Adam8
5.11E-74	-0.9146472	0.002	0.174	7.23E-70	B cells	Fasl
6.44E-74	0.84666292	0.461	0.287	9.11E-70	B cells	Ppdpf
1.32E-73	-0.8458262	0.006	0.179	1.87E-69	B cells	Sirpa
2.26E-73	-0.8571526	0.003	0.174	3.20E-69	B cells	Ms4a6d
4.83E-73	0.75241506	0.316	0.134	6.84E-69	B cells	Id3
7.90E-73	0.77855926	0.292	0.125	1.12E-68	B cells	Plcg2
9.21E-73	-0.8374885	0.005	0.177	1.30E-68	B cells	Slfn1
1.43E-72	-1.285031	0.04	0.234	2.03E-68	B cells	Cd9
1.65E-72	-0.8021103	0.251	0.512	2.34E-68	B cells	Prdx6
1.70E-72	-1.123436	0.004	0.173	2.41E-68	B cells	Tgm2
1.86E-72	0.73442841	0.831	0.694	2.63E-68	B cells	Hsp90aa1
2.82E-72	-0.9671265	0.148	0.388	4.00E-68	B cells	Bst2
3.57E-72	-0.8133819	0.207	0.46	5.06E-68	B cells	Akr1a1
3.65E-72	1.06321719	0.347	0.172	5.17E-68	B cells	Gpr171
7.73E-72	-0.7530092	0.075	0.295	1.09E-67	B cells	Runx3
2.10E-71	-0.8588912	0.007	0.177	2.97E-67	B cells	Cd300a
4.83E-71	-1.3396999	0.061	0.261	6.84E-67	B cells	Grn
6.96E-71	-0.7885222	0.133	0.37	9.85E-67	B cells	Ech1
1.11E-70	-0.7914691	0.415	0.631	1.56E-66	B cells	Ndufa13
2.02E-70	-0.9099335	0.003	0.167	2.86E-66	B cells	Izumo1r
2.17E-70	-0.7904048	0.002	0.165	3.07E-66	B cells	Cd6
2.18E-70	0.84148423	0.354	0.178	3.09E-66	B cells	Birc3
4.45E-70	-0.6583537	0.004	0.168	6.30E-66	B cells	Sema4a
5.24E-70	-0.7368879	0.004	0.169	7.41E-66	B cells	Xdh
8.53E-70	-1.0619961	0	0.16	1.21E-65	B cells	Gzmk
2.97E-69	-0.7822792	0.671	0.767	4.20E-65	B cells	Ucp2
3.93E-69	0.70594238	0.215	0.074	5.56E-65	B cells	Brwd1
4.90E-69	0.83493283	0.237	0.089	6.94E-65	B cells	Traf4
7.67E-69	0.68513749	0.187	0.058	1.09E-64	B cells	Lpcat1
9.23E-69	-0.6487998	0.002	0.163	1.31E-64	B cells	Klk8
1.28E-68	-0.6521695	0.017	0.191	1.82E-64	B cells	Fam89b
1.48E-68	-1.4643505	0.004	0.166	2.09E-64	B cells	Clec4e
1.88E-68	-0.7735067	0.054	0.255	2.66E-64	B cells	Creg1
2.31E-68	-0.8479651	0.003	0.163	3.27E-64	B cells	Sirpb1c
3.21E-68	0.53377656	0.103	0.015	4.55E-64	B cells	Prr5
4.79E-68	-1.0166823	0.04	0.224	6.78E-64	B cells	Plbd1
5.69E-68	-0.7435326	0.128	0.361	8.06E-64	B cells	Lamtor4
6.60E-68	-0.837056	0.933	0.924	9.35E-64	B cells	Srgn
1.15E-67	0.62709793	0.16	0.042	1.62E-63	B cells	Pecam1
1.39E-67	-0.8262889	0.205	0.445	1.96E-63	B cells	Psmb10
1.87E-67	-0.9359374	0.005	0.166	2.64E-63	B cells	Cxcl16
1.90E-67	-0.8730117	0.095	0.312	2.69E-63	B cells	Socs1
2.28E-67	-0.5835784	0.836	0.848	3.23E-63	B cells	Sh3bgrl3
3.27E-67	0.55639835	0.125	0.025	4.63E-63	B cells	Rubcn

3.96E-67	-1.0836681	0.024	0.198	5.61E-63	B cells	Gbp2
4.99E-67	-1.1401947	0.005	0.165	7.07E-63	B cells	C5ar1
5.93E-67	-0.8027575	0.003	0.16	8.40E-63	B cells	Slc11a1
1.02E-66	-0.7637947	0.246	0.488	1.45E-62	B cells	Psmb9
1.31E-66	-1.0800118	0.006	0.165	1.85E-62	B cells	Cd300ld
1.57E-66	-0.7463253	0.043	0.231	2.23E-62	B cells	Ptpre
2.38E-66	-0.7471652	0.344	0.568	3.37E-62	B cells	Psmb3
2.42E-66	-0.6371579	0.061	0.262	3.43E-62	B cells	Rnf166
4.44E-66	-0.831747	0.178	0.417	6.29E-62	B cells	Saraf
5.12E-66	-0.6440669	0.002	0.157	7.24E-62	B cells	Sla2
7.31E-66	-0.9573182	0.004	0.159	1.03E-61	B cells	Gda
8.50E-66	0.83730729	0.369	0.191	1.20E-61	B cells	Gem
8.69E-66	-0.768309	0.624	0.75	1.23E-61	B cells	Crip1
9.11E-66	-1.0226586	0.182	0.414	1.29E-61	B cells	Cstb
1.10E-65	0.52449236	0.48	0.264	1.55E-61	B cells	Ctsh
1.39E-65	-0.7231881	0.215	0.463	1.96E-61	B cells	Capns1
1.83E-65	-0.6777418	0.003	0.157	2.59E-61	B cells	Cx3cr1
1.92E-65	-0.728868	0.001	0.153	2.72E-61	B cells	Fcgr1
2.64E-65	0.82690635	0.438	0.28	3.73E-61	B cells	Csk
3.22E-65	0.7131822	0.204	0.07	4.56E-61	B cells	Trp53inp2
3.48E-65	0.74251339	0.268	0.116	4.93E-61	B cells	Sypl
4.71E-65	0.66196156	0.564	0.392	6.67E-61	B cells	Tgfb1
7.15E-65	-0.9012209	0.002	0.155	1.01E-60	B cells	Rgs16
1.01E-64	-0.8468054	0.039	0.222	1.42E-60	B cells	Rtp4
1.18E-64	-0.6425587	0.08	0.288	1.67E-60	B cells	Ebp
1.86E-64	0.6208219	0.471	0.278	2.63E-60	B cells	Ablim1
2.58E-64	-1.2405353	0.13	0.347	3.65E-60	B cells	Mxd1
2.77E-64	-0.7650344	0.312	0.553	3.92E-60	B cells	Ppp1r18
2.90E-64	-0.7297678	0.2	0.44	4.11E-60	B cells	Fkbp1a
3.14E-64	-0.7033892	0.004	0.156	4.44E-60	B cells	Clec4a1
3.64E-64	-0.7672839	0.179	0.41	5.15E-60	B cells	Aprt
5.44E-64	-0.73642	0	0.148	7.70E-60	B cells	Cd226
5.79E-64	-1.7702472	0.006	0.16	8.19E-60	B cells	Trem1
1.01E-63	0.3398672	0.975	0.9	1.43E-59	B cells	Rpl9-ps6
1.32E-63	-0.6786101	0.05	0.238	1.87E-59	B cells	Sdf2l1
1.54E-63	0.51394261	0.802	0.656	2.18E-59	B cells	Rps25
1.77E-63	-0.7206255	0.089	0.298	2.51E-59	B cells	Pstpip1
1.91E-63	-0.7946787	0.215	0.443	2.70E-59	B cells	Tomm6
1.92E-63	-0.6547697	0.016	0.178	2.72E-59	B cells	Hsd11b1
2.07E-63	-0.7000941	0.141	0.368	2.93E-59	B cells	Psma4
2.84E-63	0.72741488	0.187	0.062	4.02E-59	B cells	Lmo2
3.27E-63	-0.5327542	0.037	0.217	4.63E-59	B cells	Ssbp4
4.40E-63	-0.9961033	0.041	0.22	6.23E-59	B cells	Rnf149
5.03E-63	-0.6291083	0.744	0.824	7.11E-59	B cells	Laptm5
5.95E-63	0.5499333	0.736	0.58	8.42E-59	B cells	Il2rg
1.11E-62	-0.806053	0.159	0.387	1.57E-58	B cells	Cited2
1.12E-62	-1.0343255	0.011	0.167	1.59E-58	B cells	Hmox1
1.61E-62	-0.7701093	0.05	0.233	2.28E-58	B cells	Dhrs7

2.21E-62	-0.5944018	0.009	0.164	3.13E-58	B cells	Gna15
2.31E-62	-1.0848505	0.382	0.584	3.27E-58	B cells	Taldo1
2.50E-62	-0.8671369	0.022	0.187	3.53E-58	B cells	Fabp5
3.39E-62	0.31033238	0.978	0.894	4.79E-58	B cells	Rps15a
4.48E-62	-1.2052083	0.082	0.277	6.34E-58	B cells	Nfkbiz
4.77E-62	-0.5415979	0.039	0.217	6.75E-58	B cells	Cmc1
7.30E-62	-0.9232638	0.245	0.481	1.03E-57	B cells	Sdf4
7.70E-62	-2.5954808	0.01	0.161	1.09E-57	B cells	Slpi
8.55E-62	-0.7513307	0.294	0.519	1.21E-57	B cells	Pomp
9.16E-62	-0.671618	0.11	0.326	1.30E-57	B cells	Ifnar2
9.26E-62	0.68042984	0.606	0.469	1.31E-57	B cells	Pcbp1
1.15E-61	-0.7303857	0.053	0.239	1.63E-57	B cells	Rab8b
2.06E-61	-0.6977141	0.293	0.536	2.91E-57	B cells	Rnaset2a
2.16E-61	0.57098395	0.134	0.032	3.06E-57	B cells	Strbp
2.29E-61	-0.5551617	0.002	0.146	3.24E-57	B cells	Dok2
2.86E-61	-0.9083126	0.002	0.146	4.04E-57	B cells	Ikzf2
2.98E-61	0.74092876	0.252	0.108	4.22E-57	B cells	Csnk1g3
3.64E-61	-0.8380914	0.004	0.149	5.15E-57	B cells	Pilra
3.78E-61	-0.6347055	0.021	0.182	5.35E-57	B cells	Gbp7
7.29E-61	-0.6417199	0.095	0.303	1.03E-56	B cells	Etfb
1.03E-60	-0.5498338	0.002	0.145	1.46E-56	B cells	Ppm1h
2.14E-60	-0.5172389	0.841	0.844	3.03E-56	B cells	Serf2
2.16E-60	0.53526764	0.138	0.035	3.05E-56	B cells	Pold1
2.85E-60	0.73161918	0.444	0.262	4.03E-56	B cells	Klf4
3.21E-60	-0.9720705	0.561	0.665	4.54E-56	B cells	S100a10
3.74E-60	-1.1227548	0.036	0.203	5.29E-56	B cells	Anxa1
4.03E-60	0.62093373	0.317	0.15	5.70E-56	B cells	Cd72
5.15E-60	-0.5501398	0.936	0.91	7.29E-56	B cells	H2-K1
1.09E-59	-0.8222617	0.001	0.14	1.55E-55	B cells	Ccr8
1.10E-59	-0.6803365	0.535	0.68	1.55E-55	B cells	Ptpn18
1.25E-59	-0.6810702	0.002	0.143	1.77E-55	B cells	Ifi204
1.27E-59	-0.5954103	0.004	0.146	1.79E-55	B cells	Fam129a
1.36E-59	-0.9361997	0.014	0.165	1.92E-55	B cells	Tmem176b
1.51E-59	0.59670548	0.701	0.564	2.14E-55	B cells	Gnas
1.56E-59	-1.9800785	0.002	0.143	2.21E-55	B cells	Ifitm1
1.90E-59	0.90209404	0.289	0.137	2.70E-55	B cells	Gpr183
2.23E-59	-0.6775073	0.03	0.195	3.16E-55	B cells	Serpinb6a
2.47E-59	-0.7331425	0.153	0.374	3.50E-55	B cells	Gng2
4.25E-59	0.46055873	0.831	0.699	6.01E-55	B cells	Shisa5
4.49E-59	0.7284987	0.253	0.112	6.36E-55	B cells	Map4k1
4.71E-59	0.81529217	0.534	0.391	6.66E-55	B cells	Tuba1a
7.37E-59	-0.7073521	0.005	0.147	1.04E-54	B cells	Itgam
8.11E-59	-1.3782952	0.001	0.139	1.15E-54	B cells	Csf3r
9.40E-59	0.75969988	0.279	0.133	1.33E-54	B cells	Lmbrd1
9.98E-59	0.59370448	0.135	0.034	1.41E-54	B cells	Neil1
1.37E-58	0.41568569	0.859	0.742	1.95E-54	B cells	Rbm39
1.62E-58	-1.1114071	0.005	0.145	2.29E-54	B cells	Ifitm6
2.30E-58	0.6609927	0.435	0.264	3.26E-54	B cells	Tspan13

2.31E-58	-0.9584437	0.122	0.332	3.27E-54	B cells	Egr1
2.34E-58	0.37891862	0.963	0.814	3.31E-54	B cells	Rpl10a
2.35E-58	-1.1918714	0.379	0.574	3.33E-54	B cells	Sat1
2.72E-58	0.64888214	0.464	0.297	3.86E-54	B cells	Macf1
2.74E-58	-0.8559987	0.005	0.145	3.88E-54	B cells	Serpinb6b
3.13E-58	-0.6818835	0.225	0.461	4.43E-54	B cells	Ccdc12
3.30E-58	-0.5301556	0.019	0.173	4.67E-54	B cells	Gsto1
3.65E-58	-0.9491998	0.002	0.139	5.17E-54	B cells	Serpinb9
4.93E-58	-0.5859777	0.003	0.141	6.98E-54	B cells	Pid1
7.70E-58	-0.4914347	0.031	0.194	1.09E-53	B cells	Ap1s1
8.09E-58	-0.6352514	0.015	0.165	1.15E-53	B cells	Hip1
9.28E-58	-0.5371695	0.006	0.148	1.31E-53	B cells	Lime1
9.52E-58	-0.6603812	0.004	0.142	1.35E-53	B cells	Tbx21
1.18E-57	-0.7838789	0.004	0.141	1.67E-53	B cells	Nupr1
1.30E-57	-0.7304977	0.506	0.657	1.84E-53	B cells	Eno1
1.46E-57	-1.0961076	0.004	0.142	2.07E-53	B cells	Nlrp3
1.69E-57	-0.6155079	0.001	0.135	2.39E-53	B cells	Bcl11b
1.80E-57	-0.8377577	0.217	0.439	2.55E-53	B cells	Samsn1
1.81E-57	0.53576128	0.127	0.031	2.57E-53	B cells	Gpd1l
3.83E-57	-0.7146047	0.006	0.145	5.42E-53	B cells	Basp1
4.29E-57	-0.661817	0.495	0.667	6.08E-53	B cells	Atp5h
6.73E-57	-0.6736817	0.537	0.673	9.53E-53	B cells	Cox6c
7.46E-57	-0.7406415	0.338	0.562	1.06E-52	B cells	Cotl1
7.90E-57	-0.9162502	0.341	0.531	1.12E-52	B cells	Mif
7.97E-57	-0.6077774	0.711	0.781	1.13E-52	B cells	Arpc3
1.20E-56	0.74016321	0.323	0.168	1.69E-52	B cells	Pou2f2
1.39E-56	-5.5412941	0.036	0.19	1.97E-52	B cells	S100a8
1.41E-56	-0.6650242	0.242	0.473	2.00E-52	B cells	Spcs1
1.45E-56	-0.6681482	0.002	0.137	2.06E-52	B cells	Cxcr3
1.95E-56	0.77521021	0.293	0.143	2.76E-52	B cells	Elmsan1
1.95E-56	0.64024961	0.197	0.072	2.76E-52	B cells	Fbxo11
2.15E-56	-0.7125523	0.002	0.136	3.04E-52	B cells	Lrp1
2.24E-56	-0.5893372	0.092	0.288	3.18E-52	B cells	Tmem9b
2.92E-56	-0.582575	0.002	0.135	4.14E-52	B cells	Timp2
3.22E-56	-0.7697892	0.338	0.544	4.56E-52	B cells	Gstp1
3.34E-56	-0.7970507	0.007	0.145	4.73E-52	B cells	Ccl9
3.46E-56	-0.5950889	0.112	0.319	4.90E-52	B cells	Cyb5a
3.81E-56	-0.7523765	0.195	0.417	5.39E-52	B cells	Atp1b3
3.89E-56	-0.6725063	0.04	0.205	5.51E-52	B cells	Ccr5
4.05E-56	-0.9186176	0.114	0.309	5.74E-52	B cells	Bhlhe40
4.37E-56	-0.7334626	0.005	0.14	6.19E-52	B cells	Emilin2
4.38E-56	-0.6767062	0.001	0.132	6.20E-52	B cells	Sytl3
6.49E-56	-0.7302591	0.015	0.159	9.19E-52	B cells	Fgr
7.80E-56	-0.6561021	0.502	0.661	1.10E-51	B cells	Ppib
8.99E-56	-0.6647839	0.199	0.422	1.27E-51	B cells	Prdx2
9.05E-56	-0.5762003	0.071	0.255	1.28E-51	B cells	Znhit1
1.00E-55	-0.6436543	0.212	0.439	1.42E-51	B cells	Esd
1.39E-55	-0.6692894	0.07	0.252	1.97E-51	B cells	Hopx

1.40E-55	0.77642642	0.375	0.218	1.98E-51	B cells	Aff4
1.46E-55	-0.8772344	0	0.13	2.06E-51	B cells	Klre1
1.64E-55	-0.7314804	0.077	0.259	2.32E-51	B cells	Rnf125
1.98E-55	-1.200398	0.004	0.138	2.80E-51	B cells	Ccl2
2.34E-55	0.73645023	0.432	0.277	3.32E-51	B cells	Inpp5d
3.80E-55	-0.7254134	0.002	0.133	5.38E-51	B cells	C3
4.52E-55	-0.5761156	0.002	0.134	6.39E-51	B cells	Prkcq
5.06E-55	-1.6356252	0.003	0.135	7.16E-51	B cells	Cxcl10
7.46E-55	-0.6820159	0.028	0.181	1.06E-50	B cells	Clec12a
8.22E-55	-1.1758478	0.102	0.29	1.16E-50	B cells	Lmnb1
1.04E-54	-0.5691162	0.008	0.144	1.47E-50	B cells	Padi2
1.15E-54	0.67599311	0.471	0.322	1.63E-50	B cells	Cirbp
1.26E-54	0.69794838	0.267	0.125	1.79E-50	B cells	Etnk1
1.31E-54	0.72587736	0.32	0.17	1.86E-50	B cells	Nfatc3
2.17E-54	-0.7022349	0.147	0.354	3.07E-50	B cells	Ifi27
2.17E-54	-0.5897541	0.025	0.176	3.07E-50	B cells	Galnt6
2.18E-54	-0.6309698	0.043	0.206	3.08E-50	B cells	Glipr2
2.33E-54	-1.0186737	0.376	0.584	3.30E-50	B cells	Hmgb2
3.04E-54	-0.9311957	0.337	0.554	4.30E-50	B cells	Cdk2ap2
5.03E-54	-0.5194573	0.043	0.207	7.12E-50	B cells	Rps6ka1
5.85E-54	0.75813751	0.239	0.104	8.27E-50	B cells	Sesn1
6.50E-54	-0.6528361	0.262	0.485	9.19E-50	B cells	Csnk2b
6.77E-54	-0.8065855	0.001	0.128	9.58E-50	B cells	Klrc1
7.37E-54	-0.6751536	0.03	0.183	1.04E-49	B cells	Isg20
7.87E-54	-0.6559733	0.053	0.22	1.11E-49	B cells	Eif4ebp1
8.45E-54	-0.6692359	0.226	0.446	1.20E-49	B cells	Ndufv3
9.55E-54	0.645246	0.488	0.341	1.35E-49	B cells	Sf1
1.12E-53	-0.5542708	0.036	0.193	1.58E-49	B cells	Igtp
1.15E-53	-0.9835781	0.004	0.134	1.63E-49	B cells	Ly6i
2.23E-53	-0.9624932	0.062	0.231	3.16E-49	B cells	Plk3
2.40E-53	-1.2897829	0.004	0.133	3.39E-49	B cells	Clec4d
2.98E-53	0.82807731	0.551	0.414	4.21E-49	B cells	Hspd1
3.27E-53	-0.4934663	0.069	0.249	4.63E-49	B cells	Stard3nl
4.00E-53	-0.5448882	0.059	0.231	5.66E-49	B cells	Fyn
4.48E-53	-0.6255411	0.028	0.177	6.35E-49	B cells	Mllt3
4.55E-53	-0.5922575	0.001	0.126	6.44E-49	B cells	Igsf6
4.76E-53	-0.8118001	0.56	0.693	6.73E-49	B cells	Atp6v0c
5.29E-53	-0.663706	0.2	0.418	7.49E-49	B cells	Crlf2
6.59E-53	-0.7662176	0.127	0.323	9.34E-49	B cells	Tnfrsf1b
9.04E-53	-1.0025282	0.022	0.163	1.28E-48	B cells	Mgst1
2.20E-52	-0.7058854	0.488	0.637	3.11E-48	B cells	Prdx1
2.21E-52	-0.7789438	0.272	0.485	3.13E-48	B cells	Rgs2
2.58E-52	0.68055111	0.194	0.075	3.66E-48	B cells	Tfam
2.80E-52	-0.452405	0.006	0.136	3.96E-48	B cells	Ppt2
3.87E-52	0.65573741	0.432	0.279	5.48E-48	B cells	Hnrnph1
4.40E-52	-0.6548191	0.132	0.332	6.23E-48	B cells	Tes
4.57E-52	-0.6855568	0.302	0.53	6.47E-48	B cells	Gpsm3
4.58E-52	-0.5265994	0.727	0.771	6.49E-48	B cells	Cox4i1

4.81E-52	-0.7861492	0.251	0.472	6.81E-48	B cells	Irf1
6.92E-52	-0.5312911	0.751	0.793	9.79E-48	B cells	Cox8a
8.64E-52	0.67170871	0.343	0.193	1.22E-47	B cells	Rasa3
8.93E-52	-0.9881959	0.058	0.22	1.26E-47	B cells	Trib1
9.59E-52	-0.7147698	0.002	0.126	1.36E-47	B cells	AA467197
1.04E-51	-0.606011	0.008	0.136	1.47E-47	B cells	Sdc3
1.06E-51	-0.5464805	0.001	0.123	1.50E-47	B cells	Ifitm10
1.41E-51	-0.6744812	0.149	0.354	2.00E-47	B cells	Lrrfip1
1.51E-51	-0.5317559	0.121	0.323	2.14E-47	B cells	Vps29
1.84E-51	-0.6284548	0.37	0.579	2.61E-47	B cells	Rac1
1.92E-51	-0.5594503	0.059	0.228	2.71E-47	B cells	Gnptg
2.16E-51	-0.502871	0.015	0.151	3.05E-47	B cells	Impa2
2.58E-51	-0.6833811	0.128	0.324	3.65E-47	B cells	Cd82
2.80E-51	-0.8240812	0.091	0.266	3.96E-47	B cells	Dut
3.06E-51	-0.526892	0.102	0.295	4.34E-47	B cells	Cnih4
4.20E-51	-0.7774698	0.098	0.276	5.95E-47	B cells	Gimap7
5.08E-51	-0.5257935	0.158	0.369	7.19E-47	B cells	Tmem160
5.32E-51	-0.4872247	0.004	0.127	7.53E-47	B cells	Iqgap2
7.96E-51	0.56358223	0.162	0.055	1.13E-46	B cells	Plekhm1
1.30E-50	-0.6905966	0.093	0.272	1.85E-46	B cells	Smpdl3a
1.43E-50	0.75956178	0.212	0.091	2.02E-46	B cells	Txndc5
1.51E-50	-0.5741494	0.003	0.125	2.14E-46	B cells	Klra2
2.55E-50	-0.6855097	0.453	0.614	3.61E-46	B cells	Cox5a
2.76E-50	-0.9224806	0.513	0.65	3.91E-46	B cells	Ifi27l2a
2.81E-50	-0.4939423	0.058	0.223	3.98E-46	B cells	Bscl2
3.09E-50	-0.4755645	0.012	0.141	4.37E-46	B cells	Tubb6
3.24E-50	-0.5435196	0.163	0.376	4.59E-46	B cells	Ap2s1
3.50E-50	-0.4769898	0.044	0.199	4.95E-46	B cells	Ap3s1
3.58E-50	-0.6971959	0.24	0.456	5.06E-46	B cells	Gm8797
4.53E-50	0.65919056	0.205	0.085	6.41E-46	B cells	Pml
6.33E-50	-0.4944428	0.076	0.252	8.97E-46	B cells	Pigx
6.44E-50	-0.4569988	0.005	0.128	9.12E-46	B cells	Tmem106a
7.03E-50	-0.6670455	0.225	0.441	9.96E-46	B cells	Cd48
8.20E-50	-0.8246439	0.001	0.12	1.16E-45	B cells	Mcemp1
9.43E-50	-1.9970794	0.018	0.15	1.34E-45	B cells	Il1r2
1.02E-49	0.74505471	0.224	0.101	1.45E-45	B cells	Nrm
2.25E-49	-2.53133	0.005	0.125	3.18E-45	B cells	G0s2
2.91E-49	-0.52026	0.016	0.147	4.12E-45	B cells	Abr
3.14E-49	-0.4377299	0.01	0.136	4.44E-45	B cells	Spr
3.39E-49	0.50575643	0.627	0.496	4.80E-45	B cells	Dazap2
3.61E-49	-1.1966626	0.18	0.366	5.10E-45	B cells	Atf3
5.02E-49	-0.6302439	0.013	0.142	7.11E-45	B cells	Actn1
7.17E-49	0.44018302	0.754	0.631	1.01E-44	B cells	Rhoa
1.47E-48	-0.4285293	0.884	0.876	2.08E-44	B cells	Oaz1
1.60E-48	-0.6747981	0.002	0.119	2.27E-44	B cells	Sirpb1b
1.64E-48	0.74182905	0.175	0.065	2.33E-44	B cells	Sfn
1.74E-48	-0.699275	0.018	0.147	2.46E-44	B cells	Csf2rb
2.08E-48	0.25031502	0.978	0.935	2.94E-44	B cells	Eif1

2.16E-48	-0.9040754	0.005	0.123	3.05E-44	B cells	Il1rn
2.74E-48	0.47031733	0.104	0.024	3.88E-44	B cells	Snx29
3.01E-48	-0.6309243	0.496	0.634	4.26E-44	B cells	Cox6b1
3.11E-48	0.33909084	0.93	0.8	4.40E-44	B cells	Rps18
3.27E-48	0.65382626	0.226	0.102	4.63E-44	B cells	Helz2
4.05E-48	-0.5380495	0.003	0.119	5.73E-44	B cells	Clec4a2
7.80E-48	-0.5989613	0.112	0.297	1.10E-43	B cells	Gpr65
9.59E-48	0.4607634	0.11	0.028	1.36E-43	B cells	Acsf2
1.17E-47	-0.5779562	0.001	0.115	1.66E-43	B cells	Ltb4r1
1.48E-47	-0.8335191	0.003	0.118	2.10E-43	B cells	Ms4a7
1.58E-47	-0.5208731	0.004	0.121	2.23E-43	B cells	Runx2
1.75E-47	-0.68453	0.002	0.116	2.48E-43	B cells	Nrn1
1.79E-47	-0.7564656	0.07	0.231	2.53E-43	B cells	Eea1
1.96E-47	-1.3310076	0.353	0.492	2.77E-43	B cells	Ly6a
2.00E-47	0.50266236	0.127	0.037	2.84E-43	B cells	Dus2
2.44E-47	0.49115149	0.118	0.032	3.46E-43	B cells	Gucd1
2.72E-47	-0.5964508	0.174	0.378	3.85E-43	B cells	Epsti1
2.76E-47	-0.6054511	0.051	0.204	3.90E-43	B cells	Gtf2i
3.12E-47	-0.6272847	0.011	0.132	4.42E-43	B cells	Tlr2
3.16E-47	-0.5153757	0.079	0.25	4.48E-43	B cells	Dap
3.78E-47	0.61360826	0.373	0.228	5.35E-43	B cells	Tgfbr2
5.13E-47	0.60919925	0.315	0.171	7.26E-43	B cells	Dok3
5.62E-47	-0.5915106	0.298	0.507	7.96E-43	B cells	Ssr4
6.04E-47	0.2591405	0.972	0.922	8.55E-43	B cells	Rps27a
6.28E-47	-0.3761362	0.006	0.123	8.89E-43	B cells	Lxn
7.07E-47	-0.4958523	0.001	0.112	1.00E-42	B cells	Slc22a15
7.50E-47	-0.5013874	0.005	0.12	1.06E-42	B cells	Gbp8
7.62E-47	-0.8320831	0.013	0.136	1.08E-42	B cells	Cd300lf
7.84E-47	0.56465498	0.174	0.066	1.11E-42	B cells	Heg1
7.98E-47	-0.7820659	0.361	0.556	1.13E-42	B cells	Ndfip1
8.99E-47	-0.6123562	0.144	0.334	1.27E-42	B cells	Ccnd2
1.01E-46	-1.590842	0.008	0.126	1.43E-42	B cells	Hdc
1.13E-46	0.59678403	0.17	0.064	1.60E-42	B cells	Rabep2
1.14E-46	-0.4341946	0.019	0.147	1.61E-42	B cells	Capn2
1.24E-46	-0.4469766	0.003	0.116	1.75E-42	B cells	Cd302
1.36E-46	-0.4157152	0.058	0.216	1.92E-42	B cells	Tmed3
1.36E-46	-0.9050864	0.125	0.305	1.93E-42	B cells	Rgcc
1.65E-46	0.59443403	0.261	0.129	2.34E-42	B cells	Map4k2
1.65E-46	-0.4217938	0.002	0.115	2.34E-42	B cells	P2ry6
1.72E-46	0.39143488	0.206	0.09	2.44E-42	B cells	1-Mar
1.74E-46	-0.4864193	0.006	0.123	2.46E-42	B cells	Cers6
1.89E-46	-0.596866	0.06	0.215	2.67E-42	B cells	Pkp3
1.97E-46	-0.4566818	0	0.11	2.80E-42	B cells	Sdcbp2
2.00E-46	-0.6324197	0.35	0.542	2.83E-42	B cells	Psma7
2.08E-46	-0.4562658	0.011	0.132	2.95E-42	B cells	Ccdc88b
2.56E-46	-0.5895026	0.004	0.118	3.62E-42	B cells	Trf
2.73E-46	0.45301749	0.616	0.436	3.87E-42	B cells	Ltb
2.82E-46	-0.541003	0.001	0.112	3.99E-42	B cells	Cd160

3.07E-46	-0.518502	0.84	0.847	4.34E-42	B cells	Calm1
3.53E-46	-0.686697	0.015	0.139	5.00E-42	B cells	Txk
3.62E-46	1.42108304	0.539	0.441	5.13E-42	B cells	Hspa1a
4.07E-46	-0.4303568	0.015	0.139	5.76E-42	B cells	Smco4
4.52E-46	-0.5289121	0.048	0.197	6.40E-42	B cells	Ap1s2
4.68E-46	-0.6780123	0.197	0.397	6.62E-42	B cells	Stat1
4.92E-46	-0.5115744	0.202	0.408	6.97E-42	B cells	Dbi
4.96E-46	0.66894806	0.357	0.218	7.02E-42	B cells	Plekha2
5.76E-46	-0.4845855	0.055	0.209	8.16E-42	B cells	Pfkp
6.54E-46	-0.7487508	0.017	0.141	9.26E-42	B cells	Tnf
8.32E-46	-0.5892517	0.575	0.682	1.18E-41	B cells	Atp5g3
9.18E-46	-0.7295144	0.002	0.112	1.30E-41	B cells	Tcf7
1.10E-45	0.58503577	0.178	0.07	1.56E-41	B cells	Lpgat1
1.13E-45	0.61959328	0.168	0.064	1.60E-41	B cells	Pkib
1.14E-45	-0.5497571	0.159	0.358	1.61E-41	B cells	H13
1.15E-45	-1.4866793	0.006	0.12	1.62E-41	B cells	Thbs1
1.55E-45	0.70058873	0.347	0.215	2.19E-41	B cells	Smap2
1.73E-45	-0.3799103	0.037	0.178	2.44E-41	B cells	Blvra
2.20E-45	-1.5473361	0.459	0.605	3.11E-41	B cells	Cebpb
2.26E-45	0.51581112	0.615	0.491	3.20E-41	B cells	Hnrnpk
3.10E-45	-0.3748874	0.02	0.147	4.39E-41	B cells	Il3ra
3.21E-45	-0.5622492	0.177	0.38	4.54E-41	B cells	Dynlrb1
4.21E-45	-3.5276205	0.009	0.123	5.96E-41	B cells	Retnlg
4.40E-45	-0.4998	0.07	0.231	6.23E-41	B cells	Lfng
4.73E-45	-0.5704059	0.245	0.453	6.70E-41	B cells	Sdhb
4.79E-45	-0.5813387	0.156	0.346	6.78E-41	B cells	Slc25a4
5.57E-45	0.6788594	0.356	0.221	7.88E-41	B cells	Matr3
5.96E-45	0.48063029	0.176	0.066	8.44E-41	B cells	Mirt1
6.26E-45	-0.4244439	0.001	0.109	8.86E-41	B cells	Hfe
6.72E-45	-0.7430136	0.091	0.252	9.51E-41	B cells	Ninj1
1.05E-44	-0.4726361	0.001	0.107	1.49E-40	B cells	Cd200r1
1.20E-44	-0.8009906	0.001	0.108	1.70E-40	B cells	Gcnt2
1.21E-44	-0.4615446	0.002	0.109	1.72E-40	B cells	Rhoc
1.30E-44	-5.8513866	0.061	0.197	1.84E-40	B cells	S100a9
1.39E-44	0.30090586	0.975	0.885	1.96E-40	B cells	Rps3
1.60E-44	-0.9159152	0.004	0.114	2.26E-40	B cells	Osm
1.67E-44	-0.458296	0.055	0.205	2.36E-40	B cells	Evi2a
1.85E-44	0.70514433	0.348	0.209	2.63E-40	B cells	Nrros
1.93E-44	-0.8450345	0.082	0.24	2.73E-40	B cells	Dgat1
2.19E-44	-0.9607153	0.12	0.284	3.10E-40	B cells	Hilpda
2.27E-44	0.5824248	0.181	0.074	3.21E-40	B cells	Tec
2.34E-44	-0.4761478	0.016	0.136	3.32E-40	B cells	Kcnn4
2.89E-44	-1.1412957	0.003	0.111	4.09E-40	B cells	Hcar2
3.33E-44	-0.646174	0.027	0.156	4.71E-40	B cells	Nfam1
3.38E-44	0.5723155	0.32	0.178	4.78E-40	B cells	Ms4a4c
4.07E-44	-0.4037632	0.003	0.11	5.76E-40	B cells	Slamf8
4.50E-44	-0.5196941	0.02	0.143	6.37E-40	B cells	Fes
4.53E-44	-0.3977862	0.021	0.146	6.41E-40	B cells	Oas1a

6.96E-44	-0.4080888	0.027	0.157	9.85E-40	B cells	Ggh
7.86E-44	0.52390641	0.144	0.049	1.11E-39	B cells	Cbx7
8.89E-44	0.6359296	0.182	0.075	1.26E-39	B cells	Rpia
9.78E-44	-0.4848955	0.15	0.344	1.38E-39	B cells	Txndc17
1.09E-43	-1.2630419	0.513	0.657	1.54E-39	B cells	Dusp1
1.14E-43	-0.4674723	0.001	0.105	1.61E-39	B cells	Ctla2b
1.25E-43	-0.4855202	0.004	0.111	1.77E-39	B cells	Atp8b4
1.44E-43	-0.3834377	0.014	0.132	2.04E-39	B cells	Eva1b
1.52E-43	-0.5852812	0.05	0.193	2.15E-39	B cells	Ass1
1.52E-43	-0.5271118	0.002	0.107	2.15E-39	B cells	Bcl2a1a
1.67E-43	0.58206927	0.199	0.087	2.36E-39	B cells	Med13
1.88E-43	0.52633485	0.575	0.462	2.66E-39	B cells	Eef1g
1.90E-43	-0.7518586	0.172	0.358	2.68E-39	B cells	Hif1a
2.17E-43	0.49210065	0.143	0.049	3.08E-39	B cells	Atp6v0a1
2.22E-43	0.64038231	0.305	0.174	3.14E-39	B cells	Rcsd1
2.38E-43	-0.5835225	0.009	0.12	3.38E-39	B cells	Tmem176a
2.73E-43	-0.5865342	0.305	0.507	3.87E-39	B cells	1810037I17R
2.84E-43	-0.5507527	0.575	0.687	4.02E-39	B cells	Atp5l
2.99E-43	-0.5293695	0.003	0.108	4.23E-39	B cells	Hacd4
3.56E-43	0.50193708	0.142	0.049	5.03E-39	B cells	Hhex
3.67E-43	-0.5428083	0.05	0.191	5.20E-39	B cells	Mbp
4.18E-43	-0.7829284	0.068	0.214	5.92E-39	B cells	Pirb
4.19E-43	-0.6317104	0.004	0.111	5.93E-39	B cells	Iigp1
5.75E-43	-0.436144	0.004	0.109	8.13E-39	B cells	Camk4
7.22E-43	-0.4701963	0.001	0.103	1.02E-38	B cells	Cd96
8.41E-43	-0.9555978	0.026	0.149	1.19E-38	B cells	Il7r
8.65E-43	-0.5085096	0.017	0.135	1.22E-38	B cells	Tgtp2
8.70E-43	0.77308285	0.389	0.269	1.23E-38	B cells	Pold4
8.82E-43	-0.6758704	0.079	0.234	1.25E-38	B cells	Picalm
9.36E-43	-0.5196105	0.173	0.367	1.33E-38	B cells	Rwdd1
9.46E-43	-0.4702086	0.022	0.144	1.34E-38	B cells	Pik3r5
1.03E-42	-1.2815661	0.001	0.104	1.45E-38	B cells	Slc7a11
1.07E-42	0.562497	0.162	0.062	1.52E-38	B cells	Bmp2k
1.29E-42	0.5530262	0.146	0.052	1.82E-38	B cells	Clec2i
1.32E-42	-0.5105373	0.003	0.107	1.87E-38	B cells	Sult2b1
1.43E-42	0.55436746	0.553	0.436	2.02E-38	B cells	Kmt2e
1.71E-42	-0.4540516	0.022	0.144	2.42E-38	B cells	Snx10
1.75E-42	-0.5758283	0.489	0.637	2.48E-38	B cells	Atp5j2
2.09E-42	-0.5096502	0.001	0.102	2.96E-38	B cells	Atp2b4
2.36E-42	-0.4122086	0.043	0.18	3.34E-38	B cells	Xaf1
2.56E-42	-0.8163571	0.051	0.188	3.62E-38	B cells	Ctsl
3.33E-42	-0.3712377	0.022	0.144	4.71E-38	B cells	Eif4e3
3.59E-42	-0.407559	0.001	0.101	5.08E-38	B cells	Ubash3a
4.43E-42	0.61318225	0.389	0.251	6.27E-38	B cells	Crlf3
5.49E-42	-0.6416455	0.022	0.142	7.77E-38	B cells	Slc15a3
5.64E-42	0.38966582	0.906	0.868	7.99E-38	B cells	Junb
5.90E-42	-0.5927621	0.042	0.175	8.35E-38	B cells	Svil
8.25E-42	-0.5312429	0.102	0.267	1.17E-37	B cells	Phf11b

9.72E-42	-0.3556284	0.041	0.178	1.38E-37	B cells	Comt
1.22E-41	-0.5428591	0.239	0.445	1.72E-37	B cells	Cope
1.30E-41	-0.5154833	0.011	0.121	1.84E-37	B cells	Itm2a
1.31E-41	-0.6237019	0.002	0.103	1.85E-37	B cells	Gatm
1.31E-41	0.5293824	0.55	0.428	1.85E-37	B cells	Arhgef1
1.31E-41	-0.6728216	0.032	0.157	1.86E-37	B cells	Maf
1.59E-41	-1.6225527	0.448	0.558	2.24E-37	B cells	Psap
1.63E-41	-0.4491986	0.028	0.153	2.31E-37	B cells	Flot1
1.77E-41	0.62916583	0.219	0.106	2.51E-37	B cells	Myo1c
2.44E-41	-0.5931163	0.456	0.609	3.45E-37	B cells	Atp5j
2.52E-41	-0.3865987	0.002	0.102	3.56E-37	B cells	Hnrnpll
2.91E-41	-0.5168213	0.052	0.191	4.12E-37	B cells	Cndp2
3.13E-41	-0.4688795	0.015	0.127	4.43E-37	B cells	Cish
3.65E-41	0.53897916	0.148	0.054	5.17E-37	B cells	Fgfr1op
3.77E-41	-0.4870059	0.05	0.187	5.33E-37	B cells	Map7d1
4.38E-41	-0.4669017	0.051	0.19	6.20E-37	B cells	Dnajc1
4.83E-41	-0.5267316	0.404	0.596	6.83E-37	B cells	Tagln2
5.90E-41	0.61998145	0.286	0.165	8.36E-37	B cells	Arhgap17
6.12E-41	-0.8526522	0.008	0.112	8.66E-37	B cells	Ccr1
6.60E-41	-0.7418547	0.187	0.367	9.34E-37	B cells	Rrbp1
9.73E-41	-0.6714216	0.002	0.1	1.38E-36	B cells	Gm9733
1.04E-40	-0.5195725	0.216	0.415	1.48E-36	B cells	Chmp2a
1.08E-40	-0.5016054	0.019	0.133	1.53E-36	B cells	Arsb
1.39E-40	-0.4321129	0.012	0.12	1.97E-36	B cells	St3gal4
1.40E-40	-0.4616155	0.027	0.149	1.99E-36	B cells	Nlrc5
1.41E-40	0.49795903	0.11	0.032	1.99E-36	B cells	Icosl
1.62E-40	0.57865347	0.172	0.071	2.30E-36	B cells	A630001G21
1.80E-40	-0.5474083	0.054	0.192	2.55E-36	B cells	Snx18
2.06E-40	-0.3834009	0.002	0.101	2.91E-36	B cells	Stk39
2.25E-40	-0.5441004	0.075	0.225	3.18E-36	B cells	Icam1
2.27E-40	-0.5399512	0.212	0.406	3.21E-36	B cells	Slc3a2
3.27E-40	0.62355365	0.244	0.124	4.63E-36	B cells	Lamb3
3.57E-40	0.70593632	0.248	0.129	5.05E-36	B cells	Gpr18
3.90E-40	-0.5625197	0.03	0.151	5.52E-36	B cells	Nabp1
4.43E-40	0.5694443	0.179	0.075	6.28E-36	B cells	Gm15987
4.94E-40	-0.6349348	0.072	0.217	6.99E-36	B cells	Nr4a3
5.24E-40	-0.5122655	0.004	0.102	7.42E-36	B cells	Trem2
1.11E-39	-0.5755319	0.02	0.133	1.57E-35	B cells	Cfp
1.27E-39	0.57755718	0.224	0.109	1.80E-35	B cells	Avl9
1.72E-39	-0.6333006	0.035	0.159	2.44E-35	B cells	Bag3
1.73E-39	0.54045568	0.548	0.449	2.45E-35	B cells	Zfp706
1.80E-39	-0.4428817	0.02	0.134	2.54E-35	B cells	Mdfic
2.07E-39	0.58755137	0.231	0.116	2.94E-35	B cells	Klhl24
2.64E-39	-0.4646154	0.202	0.401	3.74E-35	B cells	Dctn3
3.51E-39	-0.6659079	0.058	0.193	4.97E-35	B cells	Marcks
4.14E-39	-0.5531132	0.087	0.238	5.86E-35	B cells	Ppt1
4.21E-39	-0.53604	0.004	0.101	5.96E-35	B cells	Smox
4.71E-39	-0.5036975	0.19	0.38	6.66E-35	B cells	Cuta

4.86E-39	0.60355	0.53	0.419	6.88E-35	B cells	Tubb4b
5.70E-39	-0.4918625	0.011	0.115	8.06E-35	B cells	Atxn1
7.63E-39	-0.4126857	0.007	0.107	1.08E-34	B cells	Tppp3
8.64E-39	-1.5942965	0.127	0.278	1.22E-34	B cells	Wfdc17
8.75E-39	-0.510242	0.051	0.184	1.24E-34	B cells	Pitpnc1
9.44E-39	-0.5183697	0.2	0.391	1.34E-34	B cells	Higd1a
1.28E-38	-0.3903912	0.033	0.154	1.81E-34	B cells	Ptpn7
2.71E-38	0.31240574	0.813	0.67	3.84E-34	B cells	Pabpc1
3.15E-38	-0.5706701	0.394	0.562	4.45E-34	B cells	Reep5
3.24E-38	-0.4224328	0.101	0.26	4.59E-34	B cells	Nmi
3.28E-38	0.5090371	0.146	0.055	4.64E-34	B cells	Card6
3.37E-38	-0.439572	0.131	0.3	4.77E-34	B cells	Tbcb
3.37E-38	-0.5168264	0.279	0.469	4.78E-34	B cells	Ndufb7
3.58E-38	0.76424181	0.304	0.184	5.07E-34	B cells	Herpud1
4.29E-38	-0.666497	0.113	0.266	6.07E-34	B cells	Zeb2
4.81E-38	0.61008715	0.289	0.17	6.81E-34	B cells	Grk6
6.35E-38	-0.5385971	0.136	0.302	8.99E-34	B cells	Rnh1
6.82E-38	-0.3129253	0.047	0.177	9.65E-34	B cells	Mpv17l2
6.86E-38	-0.5164536	0.998	0.998	9.71E-34	B cells	Gm42418
7.14E-38	-0.4921472	0.017	0.123	1.01E-33	B cells	Rab20
7.65E-38	-0.3522283	0.036	0.157	1.08E-33	B cells	Mettl9
7.67E-38	-0.4340841	0.169	0.355	1.09E-33	B cells	Ier3ip1
1.09E-37	-0.4752063	0.078	0.223	1.54E-33	B cells	Pld3
1.09E-37	-0.6139461	0.005	0.1	1.55E-33	B cells	Pygl
1.55E-37	-0.32732	0.009	0.108	2.19E-33	B cells	Ctnnbip1
1.56E-37	-0.2908891	0.04	0.165	2.20E-33	B cells	Fbxo6
1.64E-37	-0.365277	0.022	0.133	2.33E-33	B cells	Acer3
2.11E-37	-0.3288145	0.006	0.101	2.99E-33	B cells	Vps26b
2.24E-37	-0.3952917	0.009	0.107	3.17E-33	B cells	H1f0
3.04E-37	-0.4656505	0.012	0.112	4.30E-33	B cells	Rab27a
3.42E-37	-0.4110607	0.009	0.108	4.84E-33	B cells	Rgs3
3.75E-37	-0.5204	0.495	0.634	5.32E-33	B cells	Sec61b
3.91E-37	-0.5141349	0.295	0.485	5.53E-33	B cells	Mrpl52
4.17E-37	0.51521199	0.13	0.046	5.91E-33	B cells	Haao
4.95E-37	0.68976399	0.331	0.219	7.01E-33	B cells	Dnajc7
5.99E-37	0.56595201	0.453	0.337	8.47E-33	B cells	Stk24
6.89E-37	-0.5270252	0.611	0.709	9.75E-33	B cells	Eif5a
7.53E-37	-0.5578099	0.424	0.585	1.07E-32	B cells	Arpc4
1.16E-36	0.6146179	0.224	0.117	1.65E-32	B cells	Egln2
1.23E-36	-0.4766627	0.183	0.364	1.74E-32	B cells	Ndufb6
1.30E-36	0.64316445	0.397	0.283	1.83E-32	B cells	Mat2a
1.48E-36	-0.4646091	0.1	0.253	2.09E-32	B cells	Os9
1.53E-36	-0.3528283	0.048	0.175	2.17E-32	B cells	Rsu1
1.66E-36	-0.3844046	0.083	0.229	2.35E-32	B cells	Elovl1
1.84E-36	-0.3767482	0.016	0.118	2.61E-32	B cells	Anxa4
2.00E-36	-0.3833896	0.083	0.231	2.83E-32	B cells	Fkbp2
2.27E-36	-0.4311523	0.009	0.105	3.21E-32	B cells	Adgre1
2.39E-36	0.41132504	0.721	0.605	3.38E-32	B cells	Rpl31

3.09E-36	-0.4085412	0.027	0.138	4.38E-32	B cells	Fam189b
3.19E-36	-0.3597825	0.095	0.246	4.51E-32	B cells	Dctn6
3.38E-36	0.46820672	0.151	0.06	4.78E-32	B cells	Rras2
3.40E-36	-0.3050119	0.022	0.13	4.81E-32	B cells	Htatip2
3.68E-36	-0.4629277	0.184	0.369	5.20E-32	B cells	Anapc11
4.22E-36	-0.6209243	0.298	0.49	5.98E-32	B cells	Tnfaip3
4.40E-36	-0.4452568	0.033	0.148	6.23E-32	B cells	Cdk6
4.66E-36	-0.4472724	0.2	0.388	6.60E-32	B cells	Sec11a
5.17E-36	-0.5505758	0.443	0.596	7.31E-32	B cells	Atp5c1
5.51E-36	0.64345795	0.321	0.206	7.80E-32	B cells	Fli1
5.63E-36	0.27458611	0.947	0.814	7.96E-32	B cells	Rpl3
6.15E-36	-0.3476752	0.028	0.139	8.70E-32	B cells	Pea15a
6.39E-36	-0.4182955	0.159	0.334	9.04E-32	B cells	Pdcd6
7.85E-36	0.51413279	0.433	0.317	1.11E-31	B cells	Polr2a
8.15E-36	-0.4663861	0.188	0.372	1.15E-31	B cells	Timm10b
8.28E-36	0.54886359	0.177	0.08	1.17E-31	B cells	Syvn1
9.08E-36	-0.4441878	0.011	0.107	1.28E-31	B cells	Abcb1b
9.75E-36	-0.535918	0.075	0.212	1.38E-31	B cells	Serpina3g
1.01E-35	-0.4921728	0.016	0.116	1.44E-31	B cells	Oasl2
1.11E-35	-0.3568712	0.13	0.296	1.57E-31	B cells	Mrpl18
1.38E-35	-0.4255526	0.166	0.342	1.95E-31	B cells	Mrpl4
1.78E-35	-0.4346122	0.036	0.15	2.51E-31	B cells	Notch1
1.99E-35	0.669893	0.353	0.238	2.81E-31	B cells	Tob2
2.01E-35	-0.5662376	0.021	0.124	2.85E-31	B cells	Slamf9
2.22E-35	-0.4922971	0.179	0.356	3.14E-31	B cells	Ccnd3
2.39E-35	0.32952646	0.867	0.776	3.38E-31	B cells	Sub1
2.52E-35	-0.3624107	0.112	0.27	3.56E-31	B cells	Mrpl42
2.86E-35	0.4314872	0.797	0.723	4.06E-31	B cells	Ier2
3.28E-35	-0.4634937	0.016	0.115	4.64E-31	B cells	Ipcef1
3.52E-35	-0.5186622	0.379	0.561	4.98E-31	B cells	H2afj
3.74E-35	-0.5329359	0.036	0.15	5.30E-31	B cells	Cdc42ep3
3.95E-35	-0.3713102	0.081	0.222	5.59E-31	B cells	Nans
5.41E-35	-0.5129611	0.342	0.52	7.65E-31	B cells	Cox7b
5.75E-35	-0.3605434	0.013	0.11	8.13E-31	B cells	Adssl1
7.41E-35	0.27890119	0.956	0.819	1.05E-30	B cells	Rpl15
1.20E-34	0.56286526	0.228	0.122	1.70E-30	B cells	Rnf145
1.45E-34	0.49376784	0.136	0.052	2.05E-30	B cells	Pik3ip1
1.54E-34	-0.4020466	0.158	0.332	2.18E-30	B cells	Ndufa8
1.57E-34	-0.3587004	0.078	0.217	2.22E-30	B cells	Yipf1
1.61E-34	-0.4101184	0.021	0.123	2.28E-30	B cells	Rab19
1.93E-34	-0.4277016	0.014	0.11	2.74E-30	B cells	Pde4d
2.23E-34	-0.4033487	0.171	0.346	3.16E-30	B cells	Nt5c
2.69E-34	-0.4945453	0.176	0.348	3.81E-30	B cells	Pgk1
2.80E-34	-0.4842648	0.264	0.458	3.97E-30	B cells	Apbb1ip
3.22E-34	-0.4853232	0.225	0.406	4.55E-30	B cells	Rnasek
4.14E-34	-0.3585409	0.129	0.292	5.86E-30	B cells	Ccdc124
4.20E-34	-0.3132615	0.043	0.16	5.95E-30	B cells	Bak1
4.23E-34	-0.6730922	0.216	0.373	5.99E-30	B cells	Gimap4

4.28E-34	-0.4146588	0.039	0.152	6.06E-30	B cells	Mgst2
4.29E-34	0.55087945	0.233	0.126	6.07E-30	B cells	Nfkb2
4.43E-34	-0.5183201	0.26	0.443	6.27E-30	B cells	Slfn2
4.60E-34	-0.507342	0.262	0.44	6.51E-30	B cells	Ndufb8
4.68E-34	-0.4004031	0.134	0.296	6.62E-30	B cells	Rexo2
4.87E-34	-0.4139217	0.133	0.295	6.89E-30	B cells	Gltp
5.69E-34	-0.42517	0.019	0.118	8.06E-30	B cells	Syt11
5.71E-34	-0.3996088	0.106	0.255	8.09E-30	B cells	Ift20
5.81E-34	-0.3087626	0.089	0.233	8.23E-30	B cells	Commd7
6.55E-34	-0.3307236	0.064	0.193	9.27E-30	B cells	Gusb
1.29E-33	0.45584299	0.126	0.047	1.82E-29	B cells	Abca1
1.33E-33	0.51092611	0.572	0.485	1.89E-29	B cells	Srsf3
2.03E-33	0.52157169	0.158	0.068	2.87E-29	B cells	Ephx1
2.27E-33	-0.5964172	0.133	0.285	3.21E-29	B cells	Irf7
2.52E-33	-0.4281531	0.17	0.344	3.57E-29	B cells	Ndufv2
3.53E-33	-0.6225596	0.027	0.129	5.00E-29	B cells	Pltp
3.83E-33	-0.4455506	0.224	0.407	5.41E-29	B cells	Uqcr11
3.99E-33	0.49420247	0.167	0.076	5.65E-29	B cells	Ctso
4.58E-33	-0.3675538	0.119	0.273	6.48E-29	B cells	Fam173a
4.97E-33	-0.5010625	0.56	0.661	7.04E-29	B cells	Cox5b
5.03E-33	-0.3383174	0.023	0.123	7.11E-29	B cells	Rap2b
5.80E-33	-0.4066352	0.053	0.172	8.21E-29	B cells	Il6ra
8.21E-33	-0.4017215	0.078	0.211	1.16E-28	B cells	Arhgap9
9.61E-33	-0.4058832	0.034	0.14	1.36E-28	B cells	Plscr1
9.83E-33	-0.2621169	0.041	0.154	1.39E-28	B cells	Ccs
1.32E-32	0.44223911	0.104	0.035	1.87E-28	B cells	N4bp2
2.17E-32	-0.6803801	0.023	0.119	3.07E-28	B cells	Lef1
2.27E-32	-0.6282775	0.167	0.324	3.22E-28	B cells	Socs3
3.16E-32	0.59373045	0.225	0.125	4.47E-28	B cells	Map2k1
3.17E-32	0.28435646	0.928	0.795	4.49E-28	B cells	Rpl14
3.20E-32	-0.4172634	0.071	0.199	4.54E-28	B cells	Nab1
3.38E-32	-0.4959019	0.061	0.182	4.79E-28	B cells	Tcirg1
3.53E-32	-0.2888719	0.105	0.252	5.00E-28	B cells	Tomm5
4.04E-32	-0.2934845	0.035	0.142	5.72E-28	B cells	Rnpep
4.36E-32	-0.548971	0.061	0.181	6.17E-28	B cells	Inpp4b
4.53E-32	-0.481588	0.632	0.72	6.41E-28	B cells	Gng5
5.34E-32	-0.4713668	0.28	0.457	7.56E-28	B cells	Smdt1
6.47E-32	-0.4313599	0.036	0.142	9.15E-28	B cells	Naaa
6.71E-32	-0.3858637	0.046	0.159	9.50E-28	B cells	Errfi1
6.88E-32	0.27318616	0.933	0.81	9.75E-28	B cells	Rpl10-ps3
7.28E-32	-0.2887305	0.038	0.147	1.03E-27	B cells	Cnpy2
7.36E-32	-0.3151682	0.03	0.134	1.04E-27	B cells	Gmnn
7.74E-32	0.60328383	0.223	0.125	1.10E-27	B cells	Ubqln1
7.97E-32	0.62064352	0.284	0.177	1.13E-27	B cells	Phip
8.22E-32	-0.3248853	0.067	0.193	1.16E-27	B cells	Gsdmd
8.76E-32	-0.407229	0.039	0.146	1.24E-27	B cells	Cyfip1
9.31E-32	-0.445165	0.18	0.347	1.32E-27	B cells	Atp1a1
1.06E-31	-0.4854467	0.222	0.402	1.49E-27	B cells	7-Sep

1.12E-31	-0.2861115	0.045	0.158	1.59E-27	B cells	Sap30
1.48E-31	-0.3940054	0.104	0.246	2.10E-27	B cells	Srpk1
1.55E-31	-0.2983288	0.055	0.173	2.19E-27	B cells	2210016F16F
1.65E-31	-0.4160276	0.052	0.167	2.33E-27	B cells	Ttc39b
1.99E-31	-0.4038072	0.029	0.128	2.81E-27	B cells	Gng12
1.99E-31	-0.3256704	0.065	0.189	2.82E-27	B cells	Sh3bp1
2.15E-31	-0.5088928	0.354	0.517	3.04E-27	B cells	Psma2
2.17E-31	0.53532993	0.173	0.083	3.07E-27	B cells	Mgrn1
2.63E-31	-0.4698345	0.067	0.188	3.73E-27	B cells	Pgd
2.78E-31	-0.4030361	0.174	0.341	3.93E-27	B cells	Ssr2
2.92E-31	-0.3358503	0.04	0.149	4.14E-27	B cells	Ube2l6
3.16E-31	-0.2648842	0.047	0.161	4.47E-27	B cells	Cltb
4.35E-31	-0.3272195	0.101	0.242	6.16E-27	B cells	Polr2c
4.52E-31	0.46683579	0.146	0.063	6.40E-27	B cells	Cdc25b
5.18E-31	-0.3275587	0.037	0.141	7.34E-27	B cells	Ehbp1l1
5.58E-31	-0.3290101	0.079	0.209	7.90E-27	B cells	Borcs8
5.95E-31	0.4143183	0.124	0.047	8.42E-27	B cells	Cd200
6.02E-31	0.40864295	0.434	0.304	8.52E-27	B cells	Ikzf3
7.38E-31	0.61884815	0.226	0.128	1.05E-26	B cells	Oat
8.03E-31	-0.2515948	0.044	0.154	1.14E-26	B cells	Tpgs1
9.55E-31	-0.4928475	0.357	0.523	1.35E-26	B cells	Uqcrq
1.00E-30	-0.4293395	0.209	0.378	1.42E-26	B cells	Ndufc2
1.06E-30	-0.3519505	0.971	0.889	1.50E-26	B cells	Ppia
1.44E-30	-0.3306775	0.076	0.202	2.04E-26	B cells	Tmem126a
1.46E-30	-0.5080689	0.585	0.691	2.07E-26	B cells	Tmbim6
1.47E-30	-0.3234151	0.042	0.149	2.07E-26	B cells	Vcpkmt
1.63E-30	-0.379315	0.058	0.174	2.30E-26	B cells	Pxn
1.96E-30	0.56737841	0.336	0.23	2.78E-26	B cells	Apobec3
1.99E-30	-0.4478608	0.23	0.405	2.82E-26	B cells	Calm3
2.06E-30	-0.4070522	0.026	0.121	2.92E-26	B cells	Lpcat2
2.14E-30	-0.349332	0.014	0.1	3.03E-26	B cells	Hk2
2.34E-30	-0.3238419	0.088	0.221	3.31E-26	B cells	Emc4
2.43E-30	-0.312788	0.037	0.142	3.45E-26	B cells	Dtx3l
2.46E-30	0.50312056	0.294	0.184	3.49E-26	B cells	Malt1
2.70E-30	-0.3011662	0.075	0.201	3.83E-26	B cells	Zbtb8os
3.86E-30	-0.3122639	0.162	0.327	5.47E-26	B cells	Eif6
4.23E-30	-0.3829369	0.093	0.226	5.99E-26	B cells	Lasp1
4.48E-30	-0.3547293	0.095	0.228	6.35E-26	B cells	Dynlt1b
5.34E-30	-0.3172506	0.113	0.257	7.56E-26	B cells	Tex261
5.39E-30	0.49360714	0.167	0.081	7.63E-26	B cells	Farsb
5.69E-30	-0.3375078	0.065	0.184	8.06E-26	B cells	Surf1
5.70E-30	-0.4766814	0.233	0.4	8.06E-26	B cells	Atpif1
9.20E-30	0.46759894	0.148	0.067	1.30E-25	B cells	Traf5
9.22E-30	-0.4580719	0.071	0.19	1.30E-25	B cells	Skap2
1.00E-29	0.50981674	0.173	0.084	1.42E-25	B cells	Myo1e
1.02E-29	0.31728349	0.719	0.629	1.44E-25	B cells	Hspa5
1.03E-29	-0.7740271	0.258	0.413	1.45E-25	B cells	Gadd45b
1.08E-29	0.40300118	0.569	0.477	1.52E-25	B cells	Eif4g2

1.15E-29	0.66435363	0.257	0.161	1.62E-25	B cells	Gorasp2
1.37E-29	-0.3616349	0.024	0.116	1.94E-25	B cells	Znrf1
1.39E-29	-0.4794176	0.295	0.471	1.97E-25	B cells	Bsg
1.83E-29	-0.3654556	0.048	0.156	2.59E-25	B cells	Agpat3
1.84E-29	0.48323435	0.148	0.066	2.61E-25	B cells	Ciita
2.07E-29	-0.4166341	0.044	0.15	2.93E-25	B cells	Gbp4
2.27E-29	-0.281441	0.043	0.149	3.21E-25	B cells	Tapbpl
2.51E-29	-0.4228602	0.088	0.215	3.55E-25	B cells	Vmp1
2.74E-29	0.61982778	0.292	0.193	3.88E-25	B cells	Stip1
2.75E-29	-0.3449966	0.084	0.211	3.89E-25	B cells	Siva1
2.83E-29	-0.4397318	0.278	0.454	4.01E-25	B cells	Ndufb9
2.91E-29	-0.2942583	0.13	0.281	4.12E-25	B cells	AI413582
2.92E-29	-0.4324831	0.089	0.215	4.13E-25	B cells	Ptpn11
3.34E-29	-0.3185213	0.057	0.171	4.72E-25	B cells	Ciapin1
3.55E-29	0.57098287	0.211	0.118	5.02E-25	B cells	Rfk
3.79E-29	-0.3192513	0.13	0.279	5.37E-25	B cells	Hmox2
4.28E-29	-0.303442	0.124	0.272	6.06E-25	B cells	Pdap1
5.43E-29	0.51401544	0.18	0.092	7.69E-25	B cells	Rcc2
6.52E-29	0.55794345	0.192	0.103	9.23E-25	B cells	Pmf1
7.77E-29	-0.3212478	0.047	0.154	1.10E-24	B cells	Mapkapk3
7.90E-29	-0.2968945	0.018	0.104	1.12E-24	B cells	Gsap
7.93E-29	-0.3114959	0.109	0.248	1.12E-24	B cells	Snapc5
8.01E-29	0.47979353	0.184	0.095	1.13E-24	B cells	Card11
8.86E-29	0.47607274	0.277	0.172	1.25E-24	B cells	Sptbn1
9.04E-29	-0.3601163	0.09	0.218	1.28E-24	B cells	Krcc1
9.89E-29	-0.3773248	0.198	0.366	1.40E-24	B cells	Ube2l3
1.00E-28	-0.2962901	0.017	0.102	1.42E-24	B cells	Inpp1
1.02E-28	-0.2571632	0.029	0.124	1.44E-24	B cells	3830406C13F
1.15E-28	-0.4671925	0.228	0.392	1.63E-24	B cells	Rps27l
1.16E-28	-0.3597297	0.183	0.349	1.64E-24	B cells	Dnajc3
1.18E-28	-0.3979078	0.035	0.132	1.66E-24	B cells	Fkbp5
1.19E-28	-0.4322245	0.176	0.332	1.69E-24	B cells	Pttg1
1.32E-28	-0.3439821	0.105	0.24	1.87E-24	B cells	Nutf2
1.41E-28	-0.5343598	0.132	0.269	1.99E-24	B cells	Prkca
1.43E-28	-0.4192185	0.083	0.207	2.02E-24	B cells	Nrip1
1.46E-28	0.55148127	0.263	0.163	2.07E-24	B cells	Pten
1.66E-28	-0.3849519	0.069	0.185	2.35E-24	B cells	Chd9
1.95E-28	-0.4399665	0.078	0.196	2.76E-24	B cells	Rassf4
2.14E-28	-0.3734761	0.169	0.326	3.03E-24	B cells	Ralbp1
2.15E-28	-0.2747615	0.11	0.249	3.05E-24	B cells	Mcts1
2.20E-28	-0.5746183	0.018	0.101	3.11E-24	B cells	Top2a
2.87E-28	-0.3118584	0.026	0.117	4.06E-24	B cells	Galnt2
2.97E-28	-0.3330731	0.041	0.141	4.20E-24	B cells	Slk
3.10E-28	-0.2796931	0.134	0.281	4.39E-24	B cells	Mrpl57
3.11E-28	0.3530582	0.756	0.637	4.40E-24	B cells	Rpl10
3.40E-28	-0.2714937	0.057	0.169	4.81E-24	B cells	Med8
3.53E-28	-0.404098	0.182	0.339	4.99E-24	B cells	Anxa6
4.00E-28	-0.4250821	0.313	0.486	5.66E-24	B cells	Mdh2

4.30E-28	-0.4759702	0.458	0.598	6.09E-24	B cells	Gnai2
4.59E-28	-0.4034539	0.04	0.138	6.49E-24	B cells	Utrn
4.72E-28	-0.3083354	0.018	0.101	6.68E-24	B cells	Rasa4
5.12E-28	-0.280197	0.027	0.119	7.25E-24	B cells	Naga
5.66E-28	-0.3424831	0.165	0.321	8.01E-24	B cells	Cops6
5.80E-28	-0.2713225	0.062	0.175	8.21E-24	B cells	Itgb1bp1
5.97E-28	0.46442153	0.488	0.405	8.45E-24	B cells	Ube2d2a
6.33E-28	-0.3921507	0.048	0.151	8.96E-24	B cells	Gm19585
6.35E-28	0.55910604	0.208	0.117	8.99E-24	B cells	Siah2
8.78E-28	-0.353531	0.065	0.178	1.24E-23	B cells	Casp3
9.46E-28	-0.399301	0.255	0.425	1.34E-23	B cells	Tecr
1.02E-27	-0.3385488	0.183	0.346	1.44E-23	B cells	Mlf2
1.35E-27	0.56141661	0.237	0.142	1.91E-23	B cells	Baz2b
1.41E-27	-0.5054014	0.395	0.553	2.00E-23	B cells	Rhog
1.46E-27	-0.4935167	0.235	0.387	2.07E-23	B cells	Ctsa
1.62E-27	-0.3901923	0.046	0.147	2.29E-23	B cells	Mmd
1.63E-27	-0.5565597	0.277	0.445	2.31E-23	B cells	Stat3
1.67E-27	-0.4616278	0.239	0.404	2.37E-23	B cells	H2-T22
1.93E-27	-0.4602737	0.113	0.243	2.74E-23	B cells	Lnpep
2.52E-27	-0.3299225	0.113	0.248	3.56E-23	B cells	Mrps36
2.72E-27	-0.2534107	0.04	0.138	3.85E-23	B cells	Echs1
2.74E-27	-0.3259022	0.039	0.136	3.88E-23	B cells	Hk1
3.23E-27	0.57218843	0.261	0.165	4.57E-23	B cells	Grap
3.31E-27	-0.2871333	0.089	0.213	4.69E-23	B cells	Zmat5
3.71E-27	-0.5670167	0.321	0.486	5.25E-23	B cells	Pdia3
3.86E-27	-0.2871227	0.117	0.254	5.47E-23	B cells	Acaa1a
4.06E-27	-0.4881485	0.442	0.597	5.75E-23	B cells	Gpx4
4.15E-27	-0.3048102	0.055	0.161	5.87E-23	B cells	Dstn
4.57E-27	-0.3176705	0.135	0.279	6.47E-23	B cells	Lamtor5
4.71E-27	-0.4317891	0.083	0.2	6.66E-23	B cells	Trafd1
4.88E-27	0.40597289	0.108	0.041	6.90E-23	B cells	Fam214a
4.93E-27	-0.4434593	0.166	0.314	6.97E-23	B cells	Ppp1r12a
5.40E-27	-0.3741058	0.178	0.335	7.64E-23	B cells	Psma5
5.70E-27	-0.3123763	0.068	0.181	8.06E-23	B cells	Cflar
5.83E-27	-0.3107844	0.034	0.127	8.25E-23	B cells	Ppp1r9b
5.91E-27	-0.2948446	0.128	0.268	8.37E-23	B cells	Ssna1
7.38E-27	0.51425073	0.161	0.08	1.04E-22	B cells	Trim11
8.23E-27	-0.4252881	0.044	0.142	1.17E-22	B cells	Irgm1
8.47E-27	-0.3541985	0.068	0.179	1.20E-22	B cells	Myd88
1.02E-26	-0.4787278	0.562	0.647	1.45E-22	B cells	Atp5d
1.17E-26	-0.2746982	0.043	0.142	1.66E-22	B cells	Lman1
1.26E-26	-0.430014	0.231	0.395	1.78E-22	B cells	Rbms1
1.27E-26	-0.4410713	0.115	0.244	1.80E-22	B cells	Cast
1.38E-26	0.4978499	0.168	0.085	1.96E-22	B cells	Prkd2
1.43E-26	-0.4887469	0.103	0.225	2.02E-22	B cells	Lamp2
1.43E-26	-0.3633906	0.098	0.221	2.02E-22	B cells	Pip4k2a
1.47E-26	-0.3594719	0.153	0.3	2.08E-22	B cells	Sugt1
1.48E-26	-0.4557411	0.293	0.454	2.09E-22	B cells	Psmb5

2.03E-26	-0.2543642	0.084	0.204	2.87E-22	B cells	Mydgf
2.21E-26	-0.2975174	0.176	0.333	3.13E-22	B cells	Hsbp1
2.31E-26	-0.3408879	0.036	0.129	3.27E-22	B cells	Txnrd1
2.31E-26	0.64024505	0.312	0.208	3.28E-22	B cells	Nfkbid
2.36E-26	-0.2705608	0.114	0.249	3.33E-22	B cells	Twf2
2.41E-26	0.5158176	0.201	0.113	3.41E-22	B cells	Map3k1
3.12E-26	0.32144297	0.113	0.044	4.42E-22	B cells	Rnf144a
3.13E-26	-0.2845721	0.06	0.166	4.43E-22	B cells	Flt3l
3.50E-26	-0.3414438	0.047	0.146	4.95E-22	B cells	N4bp1
3.53E-26	-0.3386893	0.134	0.274	5.00E-22	B cells	Mrps15
3.73E-26	0.44905556	0.151	0.072	5.28E-22	B cells	Dnajb4
3.85E-26	-0.2891183	0.072	0.185	5.45E-22	B cells	Mthfsl
3.97E-26	0.50909096	0.156	0.077	5.62E-22	B cells	Sh2b3
4.39E-26	-0.3569072	0.198	0.36	6.21E-22	B cells	Psmd4
4.78E-26	-0.2830195	0.157	0.306	6.77E-22	B cells	Eif4e2
5.06E-26	-0.3374739	0.11	0.24	7.16E-22	B cells	Bnip2
5.69E-26	0.4653059	0.351	0.25	8.06E-22	B cells	Sik1
6.17E-26	0.48808967	0.475	0.391	8.74E-22	B cells	Brd2
7.22E-26	0.77978906	0.567	0.473	1.02E-21	B cells	Hspa1b
7.82E-26	-0.5109588	0.17	0.309	1.11E-21	B cells	Tap1
8.16E-26	-0.4467737	0.138	0.272	1.15E-21	B cells	Smc4
8.17E-26	-0.2647806	0.13	0.269	1.16E-21	B cells	Hcfc1r1
1.03E-25	-0.2508041	0.087	0.206	1.46E-21	B cells	Dctn2
1.14E-25	0.47143502	0.144	0.069	1.62E-21	B cells	Cse1l
1.18E-25	-0.2783535	0.155	0.305	1.67E-21	B cells	Mrpl20
1.19E-25	-0.2576547	0.053	0.154	1.68E-21	B cells	Dhrs1
1.20E-25	0.33721723	0.755	0.646	1.70E-21	B cells	Rpl22l1
1.46E-25	-0.4803232	0.133	0.262	2.07E-21	B cells	Slamf7
1.47E-25	-0.273543	0.151	0.297	2.08E-21	B cells	Aimp1
1.57E-25	-0.2686594	0.103	0.229	2.22E-21	B cells	Mrpl14
1.85E-25	0.44869996	0.191	0.105	2.62E-21	B cells	Sesn3
1.87E-25	-0.2777367	0.023	0.105	2.64E-21	B cells	Vamp5
1.90E-25	0.36050229	0.105	0.041	2.69E-21	B cells	Tfrc
2.07E-25	0.27645846	0.963	0.874	2.94E-21	B cells	Hsp90ab1
2.21E-25	-0.6304952	0.608	0.714	3.13E-21	B cells	Lcp1
2.38E-25	-0.3511287	0.212	0.374	3.37E-21	B cells	1810058I24R
2.56E-25	0.500613	0.199	0.114	3.62E-21	B cells	Rftn1
2.58E-25	-0.3307769	0.186	0.344	3.65E-21	B cells	Psma6
2.64E-25	0.41139585	0.351	0.252	3.74E-21	B cells	Ddx6
3.28E-25	-0.3514406	0.027	0.11	4.64E-21	B cells	Ogfrl1
3.66E-25	0.46010002	0.173	0.092	5.19E-21	B cells	Rere
3.83E-25	0.43821956	0.385	0.281	5.42E-21	B cells	Gpcpd1
4.27E-25	-0.4334149	0.31	0.467	6.04E-21	B cells	Psmb2
4.72E-25	-0.283078	0.061	0.164	6.68E-21	B cells	Casp1
5.34E-25	-0.324478	0.056	0.156	7.56E-21	B cells	Ier5l
5.63E-25	-0.2595636	0.029	0.113	7.98E-21	B cells	Dpysl2
6.00E-25	-0.3182219	0.145	0.284	8.49E-21	B cells	Tpst2
6.12E-25	0.48947292	0.281	0.185	8.66E-21	B cells	Mef2d

6.33E-25	0.49029089	0.202	0.118	8.96E-21	B cells	Cat
6.50E-25	-0.4162335	0.221	0.375	9.19E-21	B cells	Glud1
6.73E-25	0.37200396	0.3	0.198	9.53E-21	B cells	Tonsl
6.89E-25	-0.2832797	0.037	0.126	9.75E-21	B cells	Mapk3
6.97E-25	0.52056616	0.211	0.124	9.86E-21	B cells	Ivns1abp
7.09E-25	-0.2945093	0.051	0.149	1.00E-20	B cells	Dynll2
7.37E-25	-0.2605131	0.099	0.221	1.04E-20	B cells	Psmb7
8.02E-25	0.58520104	0.271	0.181	1.14E-20	B cells	Pcmtd1
8.13E-25	-0.2756072	0.13	0.263	1.15E-20	B cells	Naa10
8.55E-25	-0.3446391	0.209	0.368	1.21E-20	B cells	Wdr83os
1.01E-24	0.35825181	0.107	0.043	1.44E-20	B cells	Slc25a37
1.08E-24	-0.3093235	0.025	0.105	1.53E-20	B cells	Ebi3
1.25E-24	0.49488252	0.738	0.68	1.77E-20	B cells	Dnaja1
1.38E-24	-0.3448467	0.96	0.941	1.95E-20	B cells	H2-D1
1.40E-24	-0.4841202	0.926	0.929	1.97E-20	B cells	Lars2
1.46E-24	-0.2653904	0.022	0.1	2.07E-20	B cells	Camk2g
1.64E-24	-0.578371	0.102	0.217	2.32E-20	B cells	Cks2
1.74E-24	0.44325829	0.12	0.053	2.46E-20	B cells	1110059E24F
1.76E-24	0.42551836	0.472	0.392	2.50E-20	B cells	Ptbp3
2.09E-24	-0.4049795	0.172	0.314	2.96E-20	B cells	Sod2
2.20E-24	-0.2940583	0.095	0.213	3.12E-20	B cells	Fam162a
2.22E-24	0.49877829	0.327	0.239	3.15E-20	B cells	Wbp2
2.23E-24	-0.3450576	0.26	0.425	3.15E-20	B cells	Ndufa11
2.44E-24	-0.2538415	0.11	0.235	3.46E-20	B cells	Exosc8
2.73E-24	-0.2811556	0.037	0.124	3.87E-20	B cells	Nfic
3.73E-24	-0.250145	0.138	0.275	5.28E-20	B cells	Mvb12a
3.85E-24	-0.4258725	0.089	0.2	5.45E-20	B cells	Itgav
4.54E-24	-0.3425105	0.027	0.108	6.42E-20	B cells	Furin
4.70E-24	-0.5197012	0.068	0.168	6.66E-20	B cells	Nudt4
4.77E-24	0.45697229	0.155	0.078	6.75E-20	B cells	Tmem163
4.97E-24	-0.3750379	0.262	0.421	7.03E-20	B cells	Bax
5.94E-24	0.56119573	0.32	0.233	8.41E-20	B cells	Scaf11
6.05E-24	0.4800698	0.205	0.121	8.56E-20	B cells	Srebf2
6.82E-24	-0.2698398	0.123	0.252	9.66E-20	B cells	Tmem208
7.80E-24	-0.3887566	0.081	0.19	1.10E-19	B cells	Aplp2
8.63E-24	-0.3493438	0.23	0.392	1.22E-19	B cells	Scp2
8.66E-24	-0.3986182	0.065	0.164	1.23E-19	B cells	Asah1
9.20E-24	-0.3647861	0.118	0.241	1.30E-19	B cells	Traf1
1.24E-23	0.5053143	0.191	0.11	1.75E-19	B cells	Neu1
1.45E-23	0.53033317	0.262	0.174	2.05E-19	B cells	Ccm2
1.45E-23	-0.3721204	0.228	0.385	2.05E-19	B cells	Tmem167
1.46E-23	0.47979616	0.415	0.337	2.06E-19	B cells	Sfpq
1.49E-23	-0.4982003	0.051	0.141	2.11E-19	B cells	Ms4a4a
1.53E-23	-0.4974369	0.097	0.209	2.16E-19	B cells	Phlda1
1.53E-23	-0.3061447	0.146	0.282	2.17E-19	B cells	Ndufs6
1.87E-23	-0.4377272	0.071	0.172	2.64E-19	B cells	2310001H17I
1.87E-23	-0.3885107	0.203	0.348	2.65E-19	B cells	Bin2
1.88E-23	-0.2797183	0.122	0.248	2.66E-19	B cells	Lims1

1.89E-23	-0.4825164	0.068	0.167	2.68E-19	B cells	Lyst
1.96E-23	-0.3935822	0.236	0.389	2.77E-19	B cells	Psmb4
2.02E-23	-0.3864135	0.234	0.389	2.86E-19	B cells	Ostc
2.05E-23	-0.4061937	0.256	0.405	2.90E-19	B cells	Nme1
2.56E-23	-0.3578272	0.089	0.199	3.62E-19	B cells	Tm6sf1
2.61E-23	-0.3170977	0.075	0.18	3.70E-19	B cells	Trps1
2.63E-23	-0.2697283	0.047	0.138	3.72E-19	B cells	Mvp
2.72E-23	-0.436552	0.284	0.436	3.84E-19	B cells	Ptpn1
2.90E-23	0.27535479	0.302	0.199	4.11E-19	B cells	Marcksl1
2.94E-23	-0.2908582	0.064	0.163	4.16E-19	B cells	Rpa2
2.98E-23	-0.3258544	0.065	0.164	4.22E-19	B cells	Etv6
3.14E-23	-0.4660128	0.602	0.646	4.44E-19	B cells	Nme2
3.33E-23	0.36482025	0.463	0.37	4.71E-19	B cells	Hmgn2
3.70E-23	-0.2768843	0.152	0.291	5.24E-19	B cells	Aip
3.86E-23	-0.3687384	0.267	0.427	5.47E-19	B cells	Mrps21
4.14E-23	0.49584988	0.347	0.26	5.86E-19	B cells	Bcl10
4.50E-23	-0.4235735	0.061	0.156	6.37E-19	B cells	Adam19
4.65E-23	-0.4480815	0.158	0.282	6.58E-19	B cells	Gimap5
6.65E-23	-0.3146481	0.215	0.367	9.41E-19	B cells	Mkrn1
6.74E-23	-0.2737042	0.026	0.103	9.54E-19	B cells	Mxi1
7.26E-23	-0.3814185	0.117	0.236	1.03E-18	B cells	Mxd4
8.27E-23	-0.2715528	0.199	0.353	1.17E-18	B cells	Bag1
8.55E-23	0.38103281	0.481	0.394	1.21E-18	B cells	Clk1
8.90E-23	-0.3014376	0.15	0.283	1.26E-18	B cells	Fundc2
1.09E-22	-0.3036293	0.026	0.103	1.54E-18	B cells	Herc6
1.17E-22	-0.3024633	0.064	0.161	1.66E-18	B cells	Gyg
1.24E-22	-0.5301452	0.267	0.409	1.75E-18	B cells	Flna
1.36E-22	-0.2559832	0.092	0.204	1.92E-18	B cells	Atxn10
1.46E-22	-0.3959292	0.275	0.438	2.07E-18	B cells	Ywhab
1.49E-22	-0.3249899	0.153	0.286	2.11E-18	B cells	Npm3
1.50E-22	-0.4516096	0.228	0.374	2.12E-18	B cells	Atp2b1
1.61E-22	-0.2533346	0.129	0.256	2.28E-18	B cells	Tnfaip8l2
1.69E-22	0.53569491	0.169	0.093	2.39E-18	B cells	Tbc1d1
1.71E-22	-0.3979121	0.375	0.529	2.42E-18	B cells	Sri
2.05E-22	0.60401756	0.298	0.211	2.90E-18	B cells	Gm26532
2.28E-22	-0.281996	0.027	0.104	3.22E-18	B cells	Clip1
2.33E-22	-0.3364891	0.1	0.213	3.30E-18	B cells	Tiprl
2.56E-22	0.46561896	0.154	0.082	3.62E-18	B cells	Cd180
2.77E-22	0.37481531	0.111	0.049	3.92E-18	B cells	Rrs1
3.09E-22	-0.4099915	0.091	0.196	4.37E-18	B cells	AU020206
3.87E-22	-0.2788816	0.172	0.315	5.48E-18	B cells	Pet100
4.05E-22	-0.4108182	0.28	0.435	5.73E-18	B cells	Spcs2
4.21E-22	0.51513939	0.286	0.2	5.96E-18	B cells	Bptf
5.03E-22	-0.3217897	0.1	0.211	7.13E-18	B cells	Def6
5.85E-22	0.32382629	0.274	0.177	8.28E-18	B cells	Kctd12
6.47E-22	-0.2741323	0.044	0.128	9.16E-18	B cells	Fam49a
7.35E-22	0.4890162	0.238	0.153	1.04E-17	B cells	Pde7a
8.74E-22	-0.3851831	0.429	0.567	1.24E-17	B cells	Atp5f1

9.21E-22	0.38654107	0.502	0.433	1.30E-17	B cells	Hnrnpab
9.79E-22	0.48188391	0.222	0.14	1.39E-17	B cells	Rnf44
1.46E-21	-0.3896134	0.348	0.5	2.06E-17	B cells	Nedd8
1.73E-21	-0.2632336	0.173	0.311	2.45E-17	B cells	Cox20
1.78E-21	0.35218516	0.122	0.057	2.52E-17	B cells	Itpr3
2.06E-21	0.46839803	0.174	0.099	2.92E-17	B cells	Cd40
2.09E-21	0.41721839	0.498	0.434	2.96E-17	B cells	Srsf2
2.31E-21	0.40384039	0.306	0.217	3.28E-17	B cells	Per1
2.89E-21	-0.2674893	0.173	0.311	4.10E-17	B cells	Ufc1
2.90E-21	-0.4417737	0.081	0.179	4.11E-17	B cells	Cwc25
2.96E-21	-0.40114	0.46	0.597	4.19E-17	B cells	Atp6v0e
3.09E-21	-0.3014216	0.209	0.357	4.37E-17	B cells	Ssu72
3.46E-21	-0.3115594	0.09	0.195	4.90E-17	B cells	Map2k3
3.77E-21	-0.2922873	0.165	0.297	5.34E-17	B cells	Orai1
4.15E-21	-0.3673528	0.208	0.349	5.87E-17	B cells	Klf13
4.54E-21	-0.2918273	0.232	0.384	6.43E-17	B cells	Txn2
5.01E-21	-0.2797351	0.144	0.27	7.09E-17	B cells	Scamp2
5.39E-21	0.50454599	0.273	0.193	7.63E-17	B cells	Sipa1
5.86E-21	-0.2596936	0.096	0.204	8.29E-17	B cells	Dctn4
7.00E-21	0.52572635	0.342	0.262	9.91E-17	B cells	Kras
7.08E-21	0.4487807	0.42	0.355	1.00E-16	B cells	Paip2
8.36E-21	-0.4091938	0.447	0.571	1.18E-16	B cells	Edf1
8.38E-21	0.39713262	0.274	0.183	1.19E-16	B cells	Gns
9.38E-21	0.48601026	0.261	0.179	1.33E-16	B cells	Itsn2
1.02E-20	-0.4169972	0.044	0.125	1.44E-16	B cells	Ccpg1
1.12E-20	-0.3534207	0.284	0.437	1.58E-16	B cells	Park7
1.20E-20	-0.3555346	0.12	0.233	1.69E-16	B cells	Itpkb
1.40E-20	-0.4924013	0.312	0.449	1.98E-16	B cells	Tuba1c
1.59E-20	0.44050453	0.447	0.381	2.24E-16	B cells	Atf4
1.68E-20	-0.3310724	0.087	0.188	2.38E-16	B cells	Slc6a6
1.92E-20	0.3870308	0.149	0.079	2.72E-16	B cells	Slc12a6
2.30E-20	0.47477329	0.164	0.093	3.26E-16	B cells	Pafah1b3
2.55E-20	-0.3173131	0.199	0.334	3.61E-16	B cells	Coro1b
2.86E-20	0.41207012	0.233	0.148	4.06E-16	B cells	Adrb2
2.87E-20	-0.3061013	0.092	0.195	4.06E-16	B cells	Parp14
2.88E-20	-0.2723956	0.186	0.322	4.08E-16	B cells	Mien1
2.99E-20	0.34080625	0.565	0.501	4.23E-16	B cells	Ncl
3.21E-20	-0.2766964	0.192	0.332	4.54E-16	B cells	Psmc3
3.28E-20	-0.3926395	0.18	0.309	4.64E-16	B cells	Diaph1
3.64E-20	0.37629147	0.474	0.386	5.16E-16	B cells	Txnip
3.81E-20	0.41670116	0.138	0.072	5.39E-16	B cells	Slc30a5
3.85E-20	-0.2724991	0.178	0.314	5.45E-16	B cells	Ddost
3.94E-20	0.54439214	0.275	0.198	5.58E-16	B cells	Fkbp4
4.19E-20	-0.2656638	0.194	0.334	5.93E-16	B cells	Smap1
4.66E-20	0.49985913	0.196	0.121	6.59E-16	B cells	AI467606
4.76E-20	-0.2694297	0.174	0.307	6.74E-16	B cells	Srp19
4.90E-20	-0.2818049	0.202	0.344	6.93E-16	B cells	Mpc2
6.92E-20	-0.3942879	0.078	0.171	9.79E-16	B cells	Ddit4

7.89E-20	0.34431287	0.13	0.065	1.12E-15	B cells	Arhgef18
8.08E-20	0.52372293	0.177	0.105	1.14E-15	B cells	Nsf
8.38E-20	0.30830123	0.599	0.548	1.19E-15	B cells	Xist
9.58E-20	-0.3215955	0.102	0.204	1.36E-15	B cells	Plec
1.07E-19	0.3630413	0.104	0.047	1.52E-15	B cells	Trim34a
1.17E-19	0.39041814	0.474	0.412	1.66E-15	B cells	Hnrnpa1
1.21E-19	0.53305475	0.244	0.167	1.72E-15	B cells	Gpbp1
1.24E-19	0.55843647	0.135	0.07	1.76E-15	B cells	Grasp
1.32E-19	-0.4095691	0.573	0.65	1.87E-15	B cells	Eif3h
1.50E-19	-0.3805823	0.316	0.46	2.13E-15	B cells	Uqcr10
1.84E-19	0.34702324	0.103	0.047	2.61E-15	B cells	Tpst1
2.44E-19	0.40342005	0.162	0.092	3.45E-15	B cells	Ppp3cc
3.39E-19	-0.3412469	0.261	0.407	4.80E-15	B cells	Swi5
3.44E-19	-0.257521	0.163	0.291	4.87E-15	B cells	Ifi35
3.49E-19	0.36866391	0.125	0.063	4.93E-15	B cells	Plekhm3
3.74E-19	-0.3066468	0.246	0.391	5.29E-15	B cells	Bloc1s1
4.58E-19	0.47242117	0.193	0.119	6.48E-15	B cells	Il16
4.72E-19	-0.312168	0.29	0.44	6.68E-15	B cells	Ndufa1
5.55E-19	0.25713515	0.625	0.544	7.86E-15	B cells	Rap1b
6.34E-19	0.53265402	0.252	0.179	8.97E-15	B cells	Bin1
6.56E-19	-0.2643658	0.07	0.158	9.29E-15	B cells	Lta4h
6.68E-19	-0.320181	0.039	0.112	9.46E-15	B cells	Smc2
6.83E-19	-0.5106414	0.677	0.718	9.67E-15	B cells	Lsp1
8.11E-19	0.45256869	0.288	0.214	1.15E-14	B cells	Nrbp1
8.65E-19	0.42658017	0.179	0.106	1.23E-14	B cells	Hist1h1c
8.66E-19	-0.3069526	0.108	0.21	1.23E-14	B cells	Trim30a
9.04E-19	-0.312003	0.26	0.41	1.28E-14	B cells	Tmem234
1.09E-18	0.44563184	0.173	0.102	1.55E-14	B cells	Filip1l
1.29E-18	0.40177236	0.495	0.439	1.83E-14	B cells	Nop10
1.61E-18	-0.314856	0.236	0.377	2.28E-14	B cells	Tmem59
1.66E-18	0.41921213	0.191	0.119	2.35E-14	B cells	Far1
1.77E-18	0.42435885	0.244	0.167	2.50E-14	B cells	Add1
1.78E-18	0.45329222	0.448	0.388	2.53E-14	B cells	Sp100
1.90E-18	-0.2600388	0.058	0.141	2.69E-14	B cells	Casp4
2.35E-18	-0.2503562	0.163	0.286	3.32E-14	B cells	Lypla2
2.79E-18	-0.3291462	0.331	0.476	3.95E-14	B cells	Atp5o
3.58E-18	0.40903802	0.151	0.085	5.06E-14	B cells	Ilf3
3.60E-18	-0.5037864	0.404	0.54	5.10E-14	B cells	Mbnl1
3.68E-18	0.43169197	0.177	0.107	5.21E-14	B cells	Mylip
4.19E-18	0.46790005	0.292	0.219	5.93E-14	B cells	Oser1
4.53E-18	-0.3921565	0.399	0.519	6.42E-14	B cells	Uqcrb
4.66E-18	-0.5730863	0.213	0.326	6.60E-14	B cells	Nfe2l2
4.78E-18	0.45844275	0.191	0.121	6.76E-14	B cells	Clp1
4.94E-18	0.35343203	0.55	0.495	6.99E-14	B cells	Srsf5
5.07E-18	0.43389654	0.205	0.132	7.18E-14	B cells	Scpep1
5.60E-18	0.40130361	0.228	0.154	7.93E-14	B cells	Mfap1b
5.68E-18	-0.2747407	0.071	0.156	8.05E-14	B cells	Mcm6
5.74E-18	-0.3482019	0.406	0.549	8.13E-14	B cells	Anp32a

6.12E-18	-0.7713554	0.161	0.264	8.67E-14	B cells	Gngt2
6.34E-18	0.39907633	0.206	0.133	8.97E-14	B cells	5031425E22I
7.99E-18	0.43454335	0.135	0.073	1.13E-13	B cells	Icam2
9.99E-18	-0.2710963	0.215	0.35	1.41E-13	B cells	Akr1b3
1.33E-17	-0.2511254	0.1	0.196	1.88E-13	B cells	Zmiz1
1.37E-17	-0.2517176	0.226	0.368	1.94E-13	B cells	Rer1
1.45E-17	-0.2520386	0.157	0.274	2.05E-13	B cells	Rnaseh2c
1.48E-17	-0.3660943	0.302	0.449	2.09E-13	B cells	Pak2
1.70E-17	0.37879322	0.142	0.079	2.41E-13	B cells	Tcf12
1.73E-17	-0.3763372	0.45	0.563	2.45E-13	B cells	Cox7a2
2.01E-17	-0.3330949	0.337	0.488	2.85E-13	B cells	Psenen
2.11E-17	-0.2817621	0.079	0.166	2.99E-13	B cells	Spint2
2.55E-17	0.43066417	0.188	0.119	3.61E-13	B cells	Relb
2.93E-17	-0.6252363	0.542	0.622	4.15E-13	B cells	Arl6ip1
3.04E-17	-0.2558269	0.162	0.28	4.31E-13	B cells	Rab5c
3.16E-17	0.52567326	0.375	0.316	4.47E-13	B cells	Cacybp
3.26E-17	-0.8537194	0.264	0.347	4.62E-13	B cells	Spi1
3.54E-17	-0.556989	0.335	0.461	5.02E-13	B cells	Plek
4.24E-17	-0.3038161	0.104	0.199	6.00E-13	B cells	Nadk
4.29E-17	-0.2670651	0.18	0.304	6.07E-13	B cells	Ak2
5.28E-17	-0.4179814	0.348	0.483	7.47E-13	B cells	Cox17
5.41E-17	-0.358271	0.566	0.646	7.66E-13	B cells	Ubl5
5.58E-17	-0.2743463	0.154	0.267	7.90E-13	B cells	Nptn
6.49E-17	0.39775304	0.207	0.133	9.19E-13	B cells	S1pr4
7.05E-17	0.39254673	0.344	0.274	9.98E-13	B cells	Slbp
7.21E-17	-0.3068114	0.365	0.509	1.02E-12	B cells	Srp9
7.74E-17	0.38525964	0.24	0.167	1.10E-12	B cells	Taf6l
8.73E-17	-1.0337588	0.691	0.683	1.24E-12	B cells	Gpx1
8.82E-17	0.3667622	0.152	0.087	1.25E-12	B cells	Gadd45a
1.41E-16	0.45853905	0.163	0.1	2.00E-12	B cells	Ube2d1
1.45E-16	0.26964483	0.701	0.622	2.05E-12	B cells	Limd2
1.50E-16	-0.3181669	0.119	0.218	2.12E-12	B cells	Mef2a
1.54E-16	-0.3515278	0.203	0.322	2.17E-12	B cells	Plp2
1.63E-16	-0.3025361	0.349	0.494	2.30E-12	B cells	Srp14
1.68E-16	-0.4700879	0.242	0.351	2.37E-12	B cells	Gimap3
1.70E-16	-0.3717068	0.095	0.183	2.41E-12	B cells	Gsn
1.76E-16	-0.3331192	0.379	0.52	2.49E-12	B cells	Ndufa2
1.81E-16	-0.4781816	0.642	0.681	2.56E-12	B cells	Gabarap
1.90E-16	0.46360173	0.204	0.136	2.69E-12	B cells	Alkbh1
1.96E-16	0.25567502	0.132	0.07	2.77E-12	B cells	Tmem64
2.02E-16	0.40253593	0.157	0.094	2.87E-12	B cells	Chtf8
2.06E-16	-0.3532943	0.457	0.573	2.92E-12	B cells	Fis1
2.31E-16	-0.29812	0.102	0.194	3.27E-12	B cells	Hexa
2.40E-16	-0.3246823	0.348	0.494	3.40E-12	B cells	Gabarapl2
2.89E-16	-0.287923	0.074	0.154	4.10E-12	B cells	Fem1c
3.25E-16	0.38401496	0.252	0.182	4.60E-12	B cells	Slc38a1
3.25E-16	0.41491331	0.209	0.141	4.60E-12	B cells	Eml4
3.44E-16	-0.3119952	0.258	0.394	4.87E-12	B cells	Brk1

3.88E-16	0.40195746	0.154	0.091	5.50E-12	B cells	Pnpla8
3.91E-16	0.49516246	0.264	0.193	5.53E-12	B cells	Smg1
4.34E-16	0.40888613	0.172	0.108	6.14E-12	B cells	Snap29
4.48E-16	-0.3827383	0.443	0.561	6.35E-12	B cells	Cnn2
4.51E-16	-0.2716693	0.211	0.338	6.38E-12	B cells	Snrpc
4.59E-16	-0.2862723	0.296	0.436	6.49E-12	B cells	Tmed9
4.60E-16	-0.3050283	0.285	0.427	6.51E-12	B cells	Psmb6
4.92E-16	0.50320947	0.282	0.212	6.97E-12	B cells	Odc1
5.46E-16	-0.2741346	0.038	0.102	7.72E-12	B cells	Peak1
5.51E-16	-0.4001338	0.095	0.181	7.80E-12	B cells	Sgk1
7.69E-16	-0.3307211	0.093	0.178	1.09E-11	B cells	Hck
1.02E-15	-0.3558529	0.25	0.377	1.44E-11	B cells	Cap1
1.07E-15	0.44119558	0.18	0.117	1.52E-11	B cells	Ddx41
1.09E-15	-0.3364052	0.511	0.603	1.54E-11	B cells	Slc25a5
1.14E-15	0.40244781	0.264	0.195	1.61E-11	B cells	Hnrnph2
1.23E-15	0.46163619	0.402	0.336	1.73E-11	B cells	Crem
2.11E-15	-0.3096448	0.17	0.283	2.99E-11	B cells	Atg3
2.70E-15	0.39337717	0.199	0.133	3.82E-11	B cells	Sgms1
3.01E-15	0.38552633	0.427	0.377	4.26E-11	B cells	Luc7l2
3.34E-15	0.3774997	0.269	0.199	4.73E-11	B cells	Hexb
3.42E-15	0.39684905	0.204	0.138	4.84E-11	B cells	Nfkbie
4.31E-15	-1.7413017	0.246	0.314	6.10E-11	B cells	Apoe
4.97E-15	-0.5102775	0.601	0.7	7.03E-11	B cells	Ptprc
6.02E-15	0.51381691	0.315	0.259	8.52E-11	B cells	Arpc5l
7.79E-15	0.37069423	0.117	0.065	1.10E-10	B cells	Phc2
8.06E-15	0.32665161	0.156	0.094	1.14E-10	B cells	Hip1r
9.81E-15	-0.2892834	0.411	0.532	1.39E-10	B cells	Psma3
1.20E-14	-0.2687707	0.235	0.357	1.70E-10	B cells	Ranbp1
1.36E-14	0.44084716	0.351	0.299	1.92E-10	B cells	Rnf187
1.60E-14	0.44196848	0.288	0.226	2.27E-10	B cells	Ikbkb
1.68E-14	-0.2994383	0.322	0.449	2.37E-10	B cells	Atp5g1
1.87E-14	-0.2676738	0.2	0.315	2.65E-10	B cells	Slc9a3r1
1.88E-14	0.43819468	0.235	0.173	2.66E-10	B cells	Uchl3
2.02E-14	0.40491215	0.346	0.29	2.85E-10	B cells	Ubl3
2.08E-14	-0.2882998	0.276	0.408	2.95E-10	B cells	BC031181
2.59E-14	0.33696453	0.104	0.055	3.66E-10	B cells	Fam220a
2.80E-14	0.39139884	0.179	0.118	3.96E-10	B cells	Zmynd11
3.51E-14	0.43925993	0.24	0.176	4.96E-10	B cells	Cd86
3.82E-14	-0.49175	0.148	0.241	5.40E-10	B cells	Ndel1
4.33E-14	0.44046519	0.299	0.24	6.14E-10	B cells	Mtpn
4.34E-14	0.37294085	0.152	0.094	6.15E-10	B cells	Slamf6
4.80E-14	0.46883823	0.281	0.223	6.79E-10	B cells	Ctbp1
6.00E-14	-0.3077867	0.352	0.481	8.49E-10	B cells	Erh
6.62E-14	-0.3460538	0.242	0.355	9.37E-10	B cells	Pnp
7.67E-14	0.49205397	0.222	0.163	1.09E-09	B cells	Limd1
7.71E-14	0.3911547	0.163	0.104	1.09E-09	B cells	Ppp1r16b
8.88E-14	-0.2807957	0.236	0.347	1.26E-09	B cells	Mrps6
1.05E-13	-0.3602075	0.134	0.223	1.49E-09	B cells	Pik3r1

1.18E-13	-0.3185121	0.487	0.586	1.67E-09	B cells	Hint1
1.20E-13	0.4135324	0.183	0.123	1.70E-09	B cells	Rnase6
1.21E-13	0.36670332	0.131	0.077	1.71E-09	B cells	Donson
1.36E-13	0.42619154	0.199	0.14	1.92E-09	B cells	Brix1
1.41E-13	-0.2663101	0.162	0.262	2.00E-09	B cells	Ankrd12
1.56E-13	-0.3865666	0.139	0.226	2.21E-09	B cells	Bcl2
1.70E-13	0.36932812	0.258	0.193	2.40E-09	B cells	Atf7ip
1.83E-13	0.3727244	0.486	0.452	2.59E-09	B cells	Eif5
1.92E-13	-0.4137206	0.307	0.425	2.71E-09	B cells	Atp6v1g1
2.05E-13	0.29438754	0.102	0.055	2.90E-09	B cells	Dennd6a
2.11E-13	0.3876049	0.211	0.15	2.98E-09	B cells	Blvrb
2.44E-13	0.34976517	0.119	0.069	3.45E-09	B cells	Slc15a4
2.47E-13	0.40516358	0.33	0.269	3.49E-09	B cells	Arid5a
2.49E-13	0.35949136	0.12	0.069	3.53E-09	B cells	Cdk19
2.53E-13	0.44108926	0.31	0.254	3.59E-09	B cells	Cdc42se2
3.09E-13	0.33493126	0.272	0.202	4.38E-09	B cells	Tob1
3.36E-13	-0.2870331	0.427	0.543	4.75E-09	B cells	Psmb1
5.23E-13	0.35747244	0.128	0.076	7.41E-09	B cells	Snhg20
7.52E-13	0.3747499	0.121	0.072	1.07E-08	B cells	Lyl1
7.68E-13	0.37894756	0.267	0.205	1.09E-08	B cells	Runx1
7.72E-13	0.34632834	0.126	0.074	1.09E-08	B cells	Lncpint
8.19E-13	0.32533255	0.128	0.077	1.16E-08	B cells	Fam120b
8.73E-13	-0.4729158	0.365	0.442	1.24E-08	B cells	1-Sep
9.81E-13	0.37873472	0.309	0.252	1.39E-08	B cells	Lbh
1.07E-12	-0.2883741	0.571	0.635	1.51E-08	B cells	Clta
1.10E-12	0.25964314	0.575	0.542	1.56E-08	B cells	Ube2b
1.15E-12	0.269707	0.564	0.516	1.63E-08	B cells	Ppp1r15a
1.22E-12	0.29937027	0.187	0.125	1.72E-08	B cells	Sdc4
1.34E-12	0.4138589	0.247	0.184	1.89E-08	B cells	Pmaip1
1.60E-12	0.40375357	0.137	0.085	2.26E-08	B cells	Aff1
1.63E-12	0.38935944	0.148	0.096	2.31E-08	B cells	Tex30
1.65E-12	0.29085098	0.114	0.065	2.34E-08	B cells	Mir142hg
1.88E-12	0.4051184	0.188	0.131	2.67E-08	B cells	Cd38
1.95E-12	-0.2802199	0.235	0.344	2.76E-08	B cells	Tkt
2.87E-12	-0.3368262	0.478	0.568	4.06E-08	B cells	Ndufa4
2.98E-12	0.42486186	0.26	0.207	4.21E-08	B cells	Srsf6
3.38E-12	0.36075448	0.232	0.173	4.79E-08	B cells	Dnajb9
3.45E-12	0.3947907	0.195	0.139	4.88E-08	B cells	Pik3ap1
3.84E-12	0.36563194	0.326	0.279	5.43E-08	B cells	Tcp1
4.16E-12	0.29540499	0.454	0.408	5.89E-08	B cells	Slc38a2
4.29E-12	-0.2611931	0.121	0.202	6.08E-08	B cells	Gimap9
4.76E-12	0.38574919	0.186	0.13	6.74E-08	B cells	Setd2
4.76E-12	0.38198709	0.258	0.201	6.74E-08	B cells	Plekho2
5.03E-12	0.35962703	0.193	0.135	7.12E-08	B cells	Crebrf
5.32E-12	0.71155783	0.183	0.128	7.52E-08	B cells	Rrad
5.83E-12	0.27508398	0.233	0.171	8.26E-08	B cells	mt-Nd6
6.17E-12	-0.6151829	0.309	0.399	8.73E-08	B cells	Ctsc
6.33E-12	0.34254168	0.147	0.096	8.96E-08	B cells	Fgd2

7.74E-12	0.33833077	0.112	0.066	1.10E-07	B cells	Myo5a
9.13E-12	0.39146129	0.16	0.109	1.29E-07	B cells	Pnrc2
9.93E-12	0.43909766	0.305	0.258	1.41E-07	B cells	Ppp1r11
1.01E-11	-0.2814928	0.079	0.146	1.42E-07	B cells	Atg2a
1.14E-11	0.35567674	0.132	0.082	1.61E-07	B cells	Rnf216
1.17E-11	-0.7044821	0.963	0.925	1.66E-07	B cells	Ftl1
1.31E-11	-0.2884454	0.081	0.148	1.86E-07	B cells	Cd274
1.34E-11	0.4390532	0.269	0.216	1.89E-07	B cells	Rbm5
1.37E-11	0.33769916	0.137	0.088	1.93E-07	B cells	Rnf2
1.50E-11	0.41345577	0.211	0.158	2.13E-07	B cells	Polr2m
1.71E-11	0.28005579	0.106	0.061	2.42E-07	B cells	Clcf1
2.06E-11	0.416524	0.207	0.155	2.92E-07	B cells	Rraga
2.11E-11	0.36495358	0.184	0.131	2.99E-07	B cells	Ncoa3
2.39E-11	0.35629418	0.191	0.138	3.39E-07	B cells	Cpsf6
2.46E-11	0.31304892	0.231	0.173	3.48E-07	B cells	Chd2
2.50E-11	0.41288793	0.257	0.204	3.54E-07	B cells	Ctnnb1
2.59E-11	0.40544267	0.2	0.146	3.67E-07	B cells	Dedd2
2.78E-11	0.31661369	0.138	0.088	3.94E-07	B cells	Elk4
2.86E-11	0.40394414	0.146	0.097	4.05E-07	B cells	Med7
2.87E-11	0.26892848	0.498	0.465	4.06E-07	B cells	Atp5a1
3.58E-11	-0.2625487	0.152	0.238	5.07E-07	B cells	Med21
4.03E-11	-0.2767215	0.187	0.282	5.70E-07	B cells	Mbnl2
4.28E-11	0.42516551	0.169	0.12	6.06E-07	B cells	Rbmxl1
4.32E-11	-0.2789885	0.433	0.545	6.12E-07	B cells	Atp6v1f
4.34E-11	-0.3656419	0.467	0.555	6.14E-07	B cells	Ran
4.87E-11	-0.2751989	0.204	0.303	6.89E-07	B cells	Dock10
5.43E-11	0.33098659	0.182	0.128	7.69E-07	B cells	Trim12a
5.51E-11	0.35715048	0.112	0.068	7.80E-07	B cells	Rnf41
6.32E-11	-0.2893409	0.589	0.647	8.95E-07	B cells	Ppp1ca
6.45E-11	0.50917725	0.171	0.121	9.13E-07	B cells	Map3k8
6.65E-11	0.32901808	0.122	0.076	9.42E-07	B cells	Trmt10c
6.82E-11	0.31668717	0.154	0.104	9.65E-07	B cells	Kpna1
7.11E-11	-0.3492082	0.522	0.612	1.01E-06	B cells	Gmfg
7.44E-11	0.38828283	0.187	0.137	1.05E-06	B cells	Ap1m1
7.61E-11	0.38796345	0.163	0.114	1.08E-06	B cells	Gt(ROSA)26S
8.08E-11	0.27015403	0.116	0.07	1.14E-06	B cells	Fbxl12
8.45E-11	0.41742135	0.221	0.169	1.20E-06	B cells	Clk4
8.50E-11	0.40708288	0.116	0.072	1.20E-06	B cells	Csrp2
9.26E-11	0.37594285	0.188	0.137	1.31E-06	B cells	Pim3
9.87E-11	0.28718142	0.435	0.402	1.40E-06	B cells	Eif3e
1.04E-10	0.3841522	0.236	0.185	1.47E-06	B cells	Ifnar1
1.13E-10	0.25854188	0.194	0.137	1.59E-06	B cells	Myadm
1.16E-10	0.31269276	0.138	0.09	1.64E-06	B cells	Tsc22d2
1.18E-10	-0.3271919	0.229	0.322	1.67E-06	B cells	Man2b1
1.41E-10	0.41947506	0.266	0.222	2.00E-06	B cells	U2af2
1.58E-10	0.3220476	0.1	0.059	2.24E-06	B cells	Foxp4
1.64E-10	0.32765604	0.11	0.067	2.33E-06	B cells	Tusc2
1.72E-10	0.27125574	0.139	0.091	2.43E-06	B cells	Rassf3

1.78E-10	0.40220095	0.243	0.196	2.52E-06	B cells	Cct3
1.89E-10	0.2705488	0.114	0.07	2.68E-06	B cells	Prr14l
2.15E-10	0.40481464	0.219	0.169	3.05E-06	B cells	Isca1
2.43E-10	0.32334053	0.378	0.333	3.44E-06	B cells	B4galnt1
2.60E-10	0.30598878	0.178	0.128	3.68E-06	B cells	Vamp2
2.63E-10	-0.2709456	0.28	0.388	3.72E-06	B cells	Dek
2.89E-10	0.31257997	0.113	0.071	4.09E-06	B cells	Kansl2
3.12E-10	0.3481	0.173	0.124	4.41E-06	B cells	Vezf1
3.14E-10	0.38076579	0.153	0.107	4.44E-06	B cells	4933434E20I
3.17E-10	0.30431591	0.107	0.064	4.49E-06	B cells	Fam169b
3.56E-10	0.26877018	0.431	0.396	5.04E-06	B cells	Tmed2
3.79E-10	-0.2504221	0.218	0.316	5.37E-06	B cells	Tor1aip1
4.05E-10	0.30764548	0.137	0.09	5.73E-06	B cells	Senp7
4.23E-10	-0.2575507	0.114	0.184	5.99E-06	B cells	Suco
4.30E-10	0.30199331	0.128	0.083	6.08E-06	B cells	Tfeb
4.73E-10	0.38065388	0.237	0.19	6.70E-06	B cells	Lat2
5.09E-10	0.38936988	0.279	0.238	7.21E-06	B cells	Emc6
5.41E-10	0.28815293	0.357	0.311	7.66E-06	B cells	Tra2a
6.03E-10	0.3846342	0.152	0.107	8.53E-06	B cells	Fh1
7.53E-10	-0.2585224	0.304	0.408	1.07E-05	B cells	Laptm4a
8.30E-10	0.35283731	0.333	0.296	1.18E-05	B cells	Atp6v0d1
1.04E-09	0.35581925	0.263	0.215	1.47E-05	B cells	Tuba4a
1.06E-09	0.37602256	0.353	0.323	1.50E-05	B cells	Cct7
1.28E-09	0.38597029	0.137	0.094	1.82E-05	B cells	Dnase2a
1.29E-09	0.30019061	0.108	0.067	1.82E-05	B cells	Zfp868
1.33E-09	0.27081971	0.206	0.152	1.88E-05	B cells	Gm26740
1.71E-09	0.35611805	0.202	0.152	2.42E-05	B cells	Kif21b
1.71E-09	0.30174576	0.277	0.229	2.42E-05	B cells	Prrc2a
1.80E-09	0.40515109	0.183	0.138	2.55E-05	B cells	Rfc1
1.87E-09	0.38830159	0.248	0.203	2.65E-05	B cells	Nop56
1.96E-09	0.25920132	0.134	0.089	2.78E-05	B cells	Mga
2.10E-09	0.35732658	0.195	0.149	2.97E-05	B cells	Fam168b
2.12E-09	-0.4006748	0.506	0.588	3.00E-05	B cells	Hsp90b1
2.34E-09	0.29496275	0.205	0.158	3.31E-05	B cells	Cmtm6
2.95E-09	0.39780933	0.237	0.197	4.17E-05	B cells	Sf3b4
4.08E-09	-0.2934141	0.106	0.17	5.78E-05	B cells	Fbxl5
4.51E-09	-0.5961316	0.993	0.981	6.39E-05	B cells	Malat1
4.65E-09	0.26827431	0.307	0.254	6.58E-05	B cells	Ssh2
6.05E-09	-0.3139447	0.128	0.196	8.57E-05	B cells	4932438A13I
6.29E-09	0.37657993	0.214	0.17	8.90E-05	B cells	Krit1
6.78E-09	0.34112074	0.21	0.168	9.60E-05	B cells	Rtcb
7.51E-09	0.27191837	0.109	0.07	0.00010626	B cells	Slc25a25
9.30E-09	0.27585687	0.18	0.133	0.0001317	B cells	Samd9l
9.35E-09	0.30257761	0.113	0.074	0.00013233	B cells	Atg16l2
9.39E-09	0.28344329	0.211	0.165	0.00013296	B cells	Morf4l2
1.15E-08	0.32195081	0.337	0.305	0.00016222	B cells	Ptges3
1.16E-08	0.33984299	0.195	0.152	0.0001644	B cells	Trim28
1.22E-08	-0.2762205	0.489	0.565	0.0001724	B cells	Cox6a1

1.35E-08	0.34692508	0.132	0.092	0.00019054	B cells	Srp54b
1.46E-08	-0.3970931	0.136	0.205	0.00020635	B cells	Tubb2a
1.47E-08	0.32661806	0.32	0.286	0.00020819	B cells	Vapa
1.54E-08	0.3625367	0.211	0.167	0.00021803	B cells	Zc3h12a
1.56E-08	0.33169059	0.286	0.242	0.00022045	B cells	Nedd9
1.62E-08	-0.2510524	0.262	0.358	0.00022895	B cells	Ncor1
1.69E-08	0.33055824	0.133	0.093	0.00023976	B cells	Rnf146
1.82E-08	0.34355063	0.225	0.182	0.0002579	B cells	Hnrnph3
2.09E-08	0.32602174	0.233	0.189	0.0002956	B cells	Arid4b
2.14E-08	0.33515181	0.195	0.15	0.00030298	B cells	St8sia4
2.23E-08	-0.2748947	0.398	0.493	0.00031564	B cells	Gpi1
2.30E-08	-0.5566903	0.67	0.723	0.00032619	B cells	Btg2
2.33E-08	0.28558031	0.126	0.086	0.0003292	B cells	Zmym5
2.47E-08	0.28071022	0.352	0.315	0.00034987	B cells	Ptpn6
2.49E-08	0.36329468	0.366	0.348	0.00035284	B cells	Mrfap1
2.52E-08	0.27463183	0.149	0.106	0.00035719	B cells	Notch2
2.83E-08	0.3506522	0.275	0.237	0.00040046	B cells	Epn1
3.28E-08	0.3416523	0.4	0.381	0.00046454	B cells	Hspa4
3.28E-08	-0.4564414	0.058	0.102	0.00046487	B cells	Sh2d3c
3.71E-08	0.37046225	0.268	0.231	0.00052575	B cells	Srsf10
4.28E-08	0.36346163	0.172	0.132	0.00060636	B cells	Yrdc
4.60E-08	0.32932715	0.232	0.193	0.00065158	B cells	Sdha
5.21E-08	0.36918304	0.151	0.111	0.00073782	B cells	Epb41l4aos
5.41E-08	0.40304914	0.284	0.255	0.00076612	B cells	Cct4
6.58E-08	0.3374972	0.166	0.126	0.00093102	B cells	Wtap
6.59E-08	0.29012073	0.327	0.297	0.00093222	B cells	Rsrc2
6.65E-08	0.31785527	0.278	0.24	0.00094156	B cells	Mknk2
6.69E-08	0.32295556	0.127	0.088	0.00094658	B cells	Flcn
6.96E-08	0.34252572	0.188	0.147	0.00098489	B cells	Pdia4
7.05E-08	0.30697825	0.104	0.068	0.00099753	B cells	Lpp
7.64E-08	0.27151335	0.306	0.27	0.00108088	B cells	Khdrbs1
8.22E-08	0.47420899	0.172	0.135	0.00116406	B cells	Plekho1
8.55E-08	0.29693489	0.107	0.071	0.00121021	B cells	Rbm48
9.25E-08	0.31656416	0.178	0.136	0.00130972	B cells	Arhgap4
9.85E-08	0.35214739	0.255	0.22	0.00139481	B cells	G3bp1
1.04E-07	0.33538798	0.194	0.154	0.00147508	B cells	Fam204a
1.05E-07	0.31854114	0.18	0.138	0.00149233	B cells	Gdi1
1.09E-07	0.30546586	0.351	0.327	0.00154726	B cells	Rab11b
1.10E-07	0.3610881	0.263	0.229	0.00156361	B cells	Yeats4
1.14E-07	0.2538096	0.134	0.095	0.00160783	B cells	Zbtb20
1.16E-07	-0.2536521	0.222	0.299	0.00164838	B cells	Gm2a
1.18E-07	0.36661459	0.223	0.188	0.00166347	B cells	Bccip
1.18E-07	-0.3057036	0.616	0.651	0.00167611	B cells	Sumo2
1.24E-07	0.28761276	0.291	0.249	0.00175815	B cells	Wsb1
1.34E-07	0.28879795	0.365	0.341	0.00190272	B cells	Cct2
1.51E-07	0.37189027	0.206	0.17	0.00214189	B cells	Ddx54
1.69E-07	0.33766104	0.27	0.237	0.00239776	B cells	Ppm1g
1.76E-07	0.3344537	0.165	0.127	0.00249727	B cells	Aars

1.85E-07	0.3252868	0.215	0.175	0.00261544	B cells	Ep400
1.88E-07	0.29619201	0.214	0.171	0.00266036	B cells	Acap1
1.93E-07	0.32859748	0.231	0.193	0.00273654	B cells	Nxf1
1.96E-07	0.34763231	0.148	0.111	0.00276957	B cells	Mrpl38
2.19E-07	0.29734122	0.128	0.091	0.00309763	B cells	Rest
2.43E-07	0.30684663	0.148	0.11	0.00344478	B cells	Gpkow
2.47E-07	-0.5568917	0.529	0.581	0.0034962	B cells	Zfp36l2
2.48E-07	0.3490256	0.15	0.113	0.00351227	B cells	P2rx4
2.72E-07	0.32013204	0.132	0.096	0.00384626	B cells	Pex13
2.72E-07	0.40259607	0.263	0.231	0.00385492	B cells	Atg101
2.98E-07	0.31161062	0.195	0.159	0.00422024	B cells	Ppp6c
2.98E-07	0.27758457	0.365	0.339	0.00422089	B cells	Ywhaq
3.03E-07	0.27037171	0.134	0.095	0.00428982	B cells	Pim2
3.16E-07	0.2583228	0.119	0.084	0.00447536	B cells	Ctps2
3.52E-07	0.25982895	0.218	0.175	0.00497587	B cells	Amd1
3.72E-07	0.3867284	0.113	0.078	0.00527027	B cells	Bambi
3.83E-07	0.25755848	0.118	0.083	0.00542582	B cells	Aida
4.02E-07	-0.3085185	0.335	0.423	0.00568607	B cells	Ifrd1
4.55E-07	0.30141027	0.286	0.253	0.00644163	B cells	Ptk2b
4.65E-07	0.30479345	0.244	0.208	0.00657575	B cells	Rlim
4.84E-07	0.31743109	0.204	0.169	0.00685377	B cells	Rbm7
4.87E-07	0.32495164	0.169	0.133	0.00688912	B cells	Galnt1
5.31E-07	0.30512752	0.11	0.077	0.00751712	B cells	Trmt1
6.31E-07	0.30754889	0.224	0.187	0.00893318	B cells	Ccnt1
6.75E-07	0.39108731	0.225	0.193	0.00954971	B cells	Nsmce4a
7.01E-07	0.3811354	0.182	0.148	0.00992349	B cells	Ythdf2
7.26E-07	0.28965668	0.125	0.091	0.01027073	B cells	Fbxo38
7.56E-07	0.29150873	0.214	0.177	0.01070048	B cells	Hbp1
7.96E-07	0.32479403	0.277	0.248	0.01127514	B cells	Maf1
9.30E-07	0.31626511	0.294	0.264	0.01317099	B cells	Cebpz
9.34E-07	0.33518849	0.184	0.148	0.01321522	B cells	Chkb
1.00E-06	-0.5856125	0.613	0.66	0.01422673	B cells	Fos
1.02E-06	0.32472167	0.254	0.223	0.01448068	B cells	Wbp11
1.03E-06	0.25902917	0.111	0.078	0.01458998	B cells	Fbxl3
1.07E-06	0.31642263	0.143	0.109	0.01519866	B cells	Rnpepl1
1.11E-06	0.37101997	0.22	0.185	0.01565593	B cells	N4bp2l2
1.14E-06	0.3290242	0.313	0.283	0.0161149	B cells	Acp5
1.57E-06	0.30747268	0.154	0.119	0.02216859	B cells	Setd5
1.57E-06	0.3348945	0.204	0.169	0.02217598	B cells	Mfap1a
1.81E-06	0.25700441	0.225	0.188	0.02559405	B cells	Kdm7a
1.82E-06	0.31640233	0.145	0.111	0.02570267	B cells	Cd2ap
1.98E-06	0.26668038	0.273	0.24	0.02801176	B cells	Cd164
2.02E-06	0.41087114	0.229	0.201	0.02852804	B cells	Impdh2
2.09E-06	0.28979609	0.141	0.108	0.02954863	B cells	Atl3
2.24E-06	0.26899133	0.393	0.382	0.0317172	B cells	Pgls
2.81E-06	0.3631678	0.218	0.188	0.03971196	B cells	Strap
3.63E-06	0.3029353	0.131	0.099	0.05139048	B cells	Zmiz2
3.77E-06	0.2859047	0.236	0.204	0.05331168	B cells	Top2b

3.88E-06	0.30496676	0.197	0.164	0.05491915	B cells	Tm9sf2
4.37E-06	0.2790104	0.144	0.111	0.06182643	B cells	Qrich1
5.19E-06	0.34189822	0.174	0.143	0.07352657	B cells	Lats2
5.93E-06	0.26084955	0.272	0.245	0.08398498	B cells	Thrap3
5.94E-06	0.26250935	0.118	0.087	0.08413164	B cells	Dynlt3
6.24E-06	0.26653529	0.244	0.213	0.08836992	B cells	Zdhhc20
6.44E-06	0.28271849	0.127	0.096	0.09111976	B cells	Mtf2
6.56E-06	0.27942252	0.31	0.286	0.09288627	B cells	Lrp10
6.88E-06	0.25976315	0.391	0.381	0.0973756	B cells	Cdc42se1
7.18E-06	0.26229843	0.291	0.267	0.10162581	B cells	Eif4b
7.30E-06	0.33755397	0.162	0.131	0.10338246	B cells	Ccz1
7.70E-06	0.34583248	0.347	0.336	0.10904784	B cells	Ahsa1
8.03E-06	0.32485295	0.116	0.086	0.11361234	B cells	Coq2
8.86E-06	0.26591114	0.101	0.072	0.12548151	B cells	Prps1
9.90E-06	0.30870951	0.24	0.212	0.14018722	B cells	Elavl1
9.98E-06	0.29749799	0.128	0.096	0.14127134	B cells	Ahctf1
1.03E-05	0.31285115	0.13	0.099	0.14639342	B cells	Trim27
1.06E-05	0.27494326	0.346	0.329	0.14986343	B cells	Mndal
1.19E-05	0.30287969	0.114	0.084	0.16815835	B cells	Usp36
1.23E-05	0.33616129	0.256	0.227	0.173557	B cells	Il4ra
1.24E-05	0.26227878	0.104	0.075	0.17595573	B cells	Ttc5
1.38E-05	0.26304427	0.109	0.08	0.19516473	B cells	Isg20l2
1.48E-05	0.3107267	0.134	0.104	0.20949863	B cells	Gle1
1.62E-05	0.25219019	0.109	0.08	0.22891209	B cells	Hsf1
1.64E-05	0.26346746	0.128	0.098	0.23159838	B cells	Tia1
1.71E-05	0.32288399	0.218	0.189	0.24223865	B cells	Prpf8
1.74E-05	0.28999019	0.159	0.13	0.24581583	B cells	Smarcd2
1.76E-05	0.28128421	0.118	0.089	0.24882097	B cells	Pgam5
1.84E-05	0.36897597	0.174	0.145	0.26076324	B cells	Gatad2b
1.85E-05	0.346502	0.221	0.194	0.26259065	B cells	Atxn2l
2.06E-05	0.26940566	0.118	0.089	0.2912493	B cells	Gm6377
2.24E-05	0.32245766	0.155	0.125	0.31742513	B cells	Trim35
2.33E-05	0.30558731	0.127	0.098	0.32997358	B cells	Prorsd1
2.41E-05	0.26421942	0.18	0.15	0.34109652	B cells	Pias1
2.51E-05	0.26352859	0.186	0.155	0.35592898	B cells	Herc4
2.63E-05	0.32714301	0.279	0.264	0.37186013	B cells	Cct8
2.73E-05	0.26588168	0.134	0.105	0.38688784	B cells	Cnot2
2.88E-05	0.27106054	0.101	0.074	0.40722548	B cells	Kti12
2.92E-05	0.30740838	0.144	0.115	0.41266798	B cells	Cep57
3.04E-05	0.25876415	0.146	0.117	0.42988132	B cells	Prr14
3.18E-05	0.26105793	0.348	0.336	0.4507674	B cells	Mtdh
3.78E-05	0.3148005	0.213	0.186	0.5349496	B cells	Sf3b3
4.09E-05	0.28207012	0.204	0.176	0.57863694	B cells	Ttc14
4.67E-05	0.27491109	0.166	0.138	0.66127478	B cells	Nek7
4.87E-05	-0.3083912	0.201	0.246	0.68958619	B cells	Pld4
4.99E-05	-0.2895915	0.327	0.405	0.70636068	B cells	Itga4
5.19E-05	0.29922133	0.163	0.135	0.73409472	B cells	Swt1
5.37E-05	0.25568572	0.202	0.174	0.76084499	B cells	Ppp1cb

5.82E-05	0.27294	0.166	0.139	0.8242727	B cells	Trim26
5.90E-05	0.27726577	0.251	0.225	0.83545812	B cells	Epc1
6.41E-05	0.28304295	0.155	0.128	0.90776888	B cells	Prpf38a
6.88E-05	-0.3284616	0.4	0.466	0.97402503	B cells	Dusp5
7.63E-05	0.2750332	0.223	0.193	1	B cells	Tagap
7.76E-05	0.27512236	0.361	0.361	1	B cells	Llph
8.15E-05	0.25073448	0.266	0.241	1	B cells	Arhgap15
8.98E-05	0.27946784	0.118	0.092	1	B cells	Sfswap
9.52E-05	0.31515508	0.195	0.171	1	B cells	Hspa9
9.60E-05	0.30773195	0.164	0.139	1	B cells	Dcaf12
9.82E-05	0.2727395	0.145	0.116	1	B cells	Arhgap25
0.00011178	0.28840371	0.258	0.24	1	B cells	Rbbp4
0.00015574	-0.3652242	0.534	0.593	1	B cells	Myh9
0.00015888	0.25450663	0.271	0.252	1	B cells	Bclaf1
0.00017905	0.29780357	0.217	0.195	1	B cells	Azin1
0.00018055	0.27991276	0.196	0.171	1	B cells	Kdm5a
0.00018452	0.3321284	0.168	0.144	1	B cells	Trappc5
0.00019155	0.25742436	0.141	0.115	1	B cells	Lin7c
0.00019389	0.32457438	0.197	0.175	1	B cells	Zfp622
0.00019828	0.43435794	0.325	0.321	1	B cells	Rilpl2
0.00023235	0.32428276	0.142	0.117	1	B cells	Arl5c
0.00024146	0.25090314	0.134	0.108	1	B cells	Abt1
0.00025436	0.26398259	0.103	0.079	1	B cells	Tex10
0.0002619	0.26671742	0.111	0.087	1	B cells	Cops3
0.00030478	0.28531515	0.288	0.277	1	B cells	St13
0.00030831	0.28058831	0.219	0.199	1	B cells	Serinc1
0.00031957	0.34333602	0.208	0.188	1	B cells	Gm11808
0.00034029	0.27896304	0.104	0.081	1	B cells	Heatr6
0.00034413	-0.4499028	0.161	0.199	1	B cells	Stmn1
0.00035795	0.30011806	0.255	0.242	1	B cells	Ubap2l
0.00039313	0.3147233	0.25	0.23	1	B cells	Rhoh
0.00050546	0.26514048	0.241	0.221	1	B cells	Slc1a5
0.00054983	0.273377	0.289	0.286	1	B cells	Hypk
0.00058001	0.25531419	0.15	0.127	1	B cells	Akap8l
0.00064045	0.28713643	0.114	0.093	1	B cells	Ppm1m
0.00065181	0.25917552	0.145	0.122	1	B cells	Mybbp1a
0.00071954	0.30180257	0.144	0.122	1	B cells	Mphosph10
0.00082834	0.26938076	0.18	0.161	1	B cells	Naa50
0.00089704	0.28812418	0.212	0.195	1	B cells	Anapc5
0.00089888	0.25360363	0.113	0.091	1	B cells	Nuak2
0.00099167	0.26873975	0.222	0.205	1	B cells	Uvrag
0.0010172	0.31776576	0.234	0.221	1	B cells	Ogdh
0.0012161	0.3077192	0.177	0.159	1	B cells	Bcl7b
0.00130176	0.32678822	0.189	0.173	1	B cells	Nsmce1
0.00139281	0.26377156	0.128	0.107	1	B cells	Herpud2
0.00164182	0.30314967	0.19	0.175	1	B cells	Rbm17
0.00167589	0.25300384	0.149	0.129	1	B cells	Rbm33
0.00182869	0.30560737	0.132	0.113	1	B cells	Txndc11

0.00185332	0.29629593	0.177	0.16	1	B cells	Rad23b
0.00216147	-0.5674599	0.597	0.599	1	B cells	Nfkbia
0.00246881	-0.3920305	0.216	0.258	1	B cells	Ets2
0.00266115	0.29871799	0.183	0.166	1	B cells	B3gnt2
0.00272592	0.26920514	0.151	0.133	1	B cells	Nelfb
0.00338762	0.29392918	0.299	0.298	1	B cells	Slc50a1
0.0037735	0.266403	0.153	0.136	1	B cells	Cnpy3
0.00600556	0.27447921	0.242	0.234	1	B cells	Chd1
0.00663496	0.25024168	0.143	0.126	1	B cells	Tspan32
0.00706809	0.27624211	0.278	0.279	1	B cells	Puf60
0	3.53439969	0.853	0.163	0	Monocytes	Lyz2
0	3.30979075	0.531	0.078	0	Monocytes	C1qb
0	3.25137669	0.811	0.134	0	Monocytes	Ifitm3
0	2.97263353	0.487	0.064	0	Monocytes	C1qa
0	2.90883555	0.431	0.032	0	Monocytes	Cxcl9
0	2.8916013	0.484	0.058	0	Monocytes	C1qc
0	2.78674112	0.881	0.433	0	Monocytes	Ctss
0	2.69547599	0.851	0.413	0	Monocytes	Psap
0	2.65990393	0.67	0.059	0	Monocytes	Ms4a6c
0	2.65130112	0.871	0.458	0	Monocytes	Cst3
0	2.60993793	0.685	0.153	0	Monocytes	Apoe
0	2.39033058	0.552	0.073	0	Monocytes	Tgfbi
0	2.26607717	0.743	0.242	0	Monocytes	Ifi30
0	2.25581164	0.785	0.31	0	Monocytes	Ctsb
0	2.19104523	0.553	0.088	0	Monocytes	Grn
0	2.15444416	0.887	0.609	0	Monocytes	Gpx1
0	2.15105111	0.902	0.246	0	Monocytes	Fcer1g
0	1.99310088	0.458	0.045	0	Monocytes	Mafb
0	1.95837514	0.487	0.046	0	Monocytes	Aif1
0	1.94680567	0.62	0.083	0	Monocytes	Cd68
0	1.93454331	0.473	0.042	0	Monocytes	Fcgr4
0	1.87546778	0.538	0.129	0	Monocytes	Gngt2
0	1.86343278	0.778	0.312	0	Monocytes	Lgals3
0	1.86299275	0.627	0.148	0	Monocytes	Cybb
0	1.81003453	0.529	0.047	0	Monocytes	Csf1r
0	1.78473127	0.336	0.017	0	Monocytes	Ly6i
0	1.75762094	0.698	0.234	0	Monocytes	Ctsz
0	1.75020199	0.504	0.052	0	Monocytes	Mpeg1
0	1.73357819	0.593	0.102	0	Monocytes	Pld4
0	1.68832329	0.573	0.099	0	Monocytes	Fcgr3
0	1.6398724	0.46	0.027	0	Monocytes	Clec4a3
0	1.59423285	0.755	0.14	0	Monocytes	Alox5ap
0	1.570592	0.373	0.037	0	Monocytes	Cxcl16
0	1.54680638	0.499	0.062	0	Monocytes	Plbd1
0	1.5306307	0.566	0.145	0	Monocytes	Cdkn1a
0	1.51505433	0.501	0.097	0	Monocytes	Plin2
0	1.50058005	0.762	0.143	0	Monocytes	Ifitm2
0	1.48638528	0.353	0.018	0	Monocytes	Nupr1

0	1.43927654	0.607	0.128	0	Monocytes	Lst1
0	1.3798274	0.411	0.041	0	Monocytes	Lair1
0	1.37473457	0.472	0.055	0	Monocytes	Rassf4
0	1.36653952	0.337	0.019	0	Monocytes	Lrp1
0	1.33445627	0.385	0.021	0	Monocytes	Clec4a1
0	1.32824547	0.886	0.273	0	Monocytes	Tyrobp
0	1.32354375	0.365	0.026	0	Monocytes	Fcgr1
0	1.26183349	0.477	0.081	0	Monocytes	Cd14
0	1.26011953	0.417	0.044	0	Monocytes	Clec12a
0	1.20514998	0.375	0.033	0	Monocytes	Sirpb1c
0	1.19818785	0.486	0.078	0	Monocytes	Csf2ra
0	1.19264783	0.343	0.024	0	Monocytes	Ifi204
0	1.11536903	0.966	0.922	0	Monocytes	Ftl1
0	1.08811893	0.469	0.061	0	Monocytes	Lrrc25
0	0.78775447	0.587	0.115	0	Monocytes	Wfdc17
0	0.68386046	0.616	0.127	0	Monocytes	Ccl6
0	0.36915327	0.628	0.137	0	Monocytes	Il1b
2.45E-302	1.25492875	0.317	0.023	3.47E-298	Monocytes	C3
3.51E-302	1.39020716	0.452	0.074	4.97E-298	Monocytes	Pla2g7
4.38E-301	1.11039937	0.667	0.201	6.20E-297	Monocytes	Spi1
5.32E-300	1.40832861	0.406	0.059	7.52E-296	Monocytes	Ckb
6.02E-294	1.28354037	0.656	0.199	8.52E-290	Monocytes	Atf3
1.52E-293	1.15444085	0.32	0.027	2.15E-289	Monocytes	Sdc3
2.59E-292	1.09808667	0.379	0.049	3.67E-288	Monocytes	BC028528
1.21E-290	1.20871725	0.409	0.061	1.71E-286	Monocytes	App
4.24E-288	0.98656732	0.324	0.029	6.00E-284	Monocytes	Pid1
1.24E-282	0.97069837	0.377	0.048	1.75E-278	Monocytes	Cd300a
2.33E-281	1.4193871	0.329	0.032	3.30E-277	Monocytes	Ifitm6
8.95E-281	1.39302458	0.362	0.049	1.27E-276	Monocytes	Ms4a6d
1.27E-277	1.08294598	0.335	0.036	1.80E-273	Monocytes	Naaa
1.64E-277	1.61287892	0.315	0.029	2.32E-273	Monocytes	Ccl2
7.46E-276	0.84868804	0.273	0.015	1.06E-271	Monocytes	Slamf8
2.40E-274	1.59428576	0.457	0.098	3.39E-270	Monocytes	Mt1
2.77E-273	0.96960811	0.375	0.051	3.92E-269	Monocytes	Sirpa
6.23E-273	0.91135649	0.281	0.018	8.82E-269	Monocytes	Cd302
1.75E-264	1.06114286	0.402	0.067	2.47E-260	Monocytes	Hck
4.94E-262	1.31428415	0.545	0.147	6.99E-258	Monocytes	Ier3
6.84E-261	1.53169332	0.74	0.386	9.68E-257	Monocytes	Prdx5
5.64E-259	1.6780147	0.456	0.105	7.98E-255	Monocytes	Fcgr2b
9.64E-259	1.01142225	0.34	0.043	1.37E-254	Monocytes	Slc11a1
2.47E-258	1.00321422	0.436	0.084	3.50E-254	Monocytes	Pirb
4.82E-257	0.86837882	0.29	0.024	6.82E-253	Monocytes	Klra2
1.44E-254	1.38356975	0.571	0.19	2.04E-250	Monocytes	Anxa5
2.30E-254	1.86151827	0.461	0.111	3.25E-250	Monocytes	Lgmn
7.44E-253	1.31643014	0.328	0.042	1.05E-248	Monocytes	Ms4a4a
1.42E-251	0.8385394	0.292	0.028	2.02E-247	Monocytes	Tmem106a
2.02E-251	1.47294985	0.84	0.563	2.86E-247	Monocytes	Vim
5.78E-250	0.87528195	0.261	0.017	8.19E-246	Monocytes	Tppp3

3.09E-248	0.9287458	0.355	0.051	4.37E-244	Monocytes	Tgm2
4.93E-246	1.664261	0.502	0.148	6.98E-242	Monocytes	H2-DMb1
2.27E-245	1.35714509	0.724	0.407	3.22E-241	Monocytes	Atox1
2.24E-244	1.02576174	0.25	0.015	3.17E-240	Monocytes	Trem2
8.04E-243	1.28775301	0.603	0.205	1.14E-238	Monocytes	Ctsh
1.18E-242	0.85846931	0.258	0.019	1.66E-238	Monocytes	Hfe
2.50E-241	1.64530841	0.382	0.074	3.53E-237	Monocytes	Gbp2
1.95E-237	1.03316647	0.344	0.05	2.76E-233	Monocytes	Mgst1
1.95E-235	0.93909886	0.234	0.012	2.76E-231	Monocytes	Dpep2
2.09E-234	0.94261058	0.253	0.019	2.96E-230	Monocytes	Bcl2a1a
2.93E-233	1.03467138	0.223	0.01	4.15E-229	Monocytes	Cfb
7.27E-233	0.74638945	0.222	0.009	1.03E-228	Monocytes	Sirpb1a
8.61E-233	1.52145295	0.767	0.44	1.22E-228	Monocytes	Sat1
4.95E-232	1.33861051	0.589	0.201	7.00E-228	Monocytes	Ly86
1.95E-229	1.15529907	0.493	0.133	2.77E-225	Monocytes	Zeb2
4.00E-229	0.99834657	0.875	0.776	5.66E-225	Monocytes	Cyba
2.50E-228	0.74669528	0.263	0.023	3.54E-224	Monocytes	P2ry6
3.78E-228	1.20004096	0.339	0.053	5.34E-224	Monocytes	Hmox1
1.17E-227	1.16865512	0.245	0.018	1.65E-223	Monocytes	Gatm
1.78E-227	1.42820512	0.266	0.026	2.52E-223	Monocytes	Ms4a7
7.38E-227	2.02892303	0.598	0.252	1.04E-222	Monocytes	Plac8
5.47E-225	0.72956079	0.215	0.009	7.75E-221	Monocytes	Gpr141
4.29E-221	0.70263368	0.98	0.949	6.08E-217	Monocytes	Fth1
4.03E-220	1.17243048	0.333	0.054	5.71E-216	Monocytes	Tmem176b
4.93E-220	0.79063648	0.301	0.04	6.98E-216	Monocytes	Tubb6
2.66E-219	1.26275515	0.373	0.075	3.76E-215	Monocytes	Ctsl
1.89E-217	1.89948747	0.206	0.008	2.67E-213	Monocytes	Chil3
8.18E-217	1.1138973	0.868	0.699	1.16E-212	Monocytes	Ucp2
1.23E-216	0.76455274	0.292	0.038	1.74E-212	Monocytes	Acer3
3.00E-214	2.11895955	0.284	0.037	4.25E-210	Monocytes	Cxcl10
3.86E-214	1.31964103	0.19	0.005	5.46E-210	Monocytes	Ace
2.26E-213	0.77795424	0.326	0.051	3.21E-209	Monocytes	Fgr
1.07E-212	1.07820439	0.287	0.037	1.51E-208	Monocytes	Pltp
3.24E-212	-1.6046319	0.194	0.568	4.59E-208	Monocytes	Ets1
2.19E-210	1.40966596	0.195	0.007	3.10E-206	Monocytes	Ear2
6.36E-209	0.4573056	0.618	0.224	9.01E-205	Monocytes	Msrb1
1.54E-208	1.21201673	0.783	0.534	2.18E-204	Monocytes	Npc2
2.90E-206	0.84527411	0.382	0.079	4.10E-202	Monocytes	Marcks
3.47E-206	0.61633785	0.226	0.016	4.91E-202	Monocytes	Ltbr
4.95E-205	-0.8771193	0.863	0.891	7.01E-201	Monocytes	Rpl13a
2.86E-204	0.96027835	0.391	0.088	4.05E-200	Monocytes	Irf5
1.11E-203	-1.8888171	0.35	0.647	1.57E-199	Monocytes	Vps37b
6.77E-202	0.67763551	0.217	0.015	9.58E-198	Monocytes	Plxnb2
2.38E-201	1.05024998	0.272	0.036	3.37E-197	Monocytes	Slamf9
6.24E-201	0.73893036	0.206	0.012	8.84E-197	Monocytes	Il18bp
6.59E-201	0.85957848	0.449	0.119	9.33E-197	Monocytes	Creg1
7.02E-199	0.68355066	0.299	0.045	9.94E-195	Monocytes	Fes
2.20E-198	0.64209681	0.268	0.033	3.12E-194	Monocytes	Igsf6

2.77E-198	0.85823694	0.333	0.061	3.92E-194	Monocytes	Apobec1
6.97E-196	0.60385062	0.201	0.012	9.87E-192	Monocytes	Tmem51
2.09E-195	0.79241692	0.541	0.177	2.96E-191	Monocytes	Lilrb4a
3.21E-193	1.17301862	0.796	0.612	4.54E-189	Monocytes	Atp6v0c
8.10E-193	0.97422033	0.336	0.068	1.15E-188	Monocytes	Asah1
3.69E-192	1.13583663	0.242	0.027	5.23E-188	Monocytes	Iigp1
4.80E-191	2.2002702	0.21	0.016	6.79E-187	Monocytes	Ccl8
6.14E-190	1.56624719	0.711	0.414	8.70E-186	Monocytes	H2-Ab1
1.15E-189	0.71619939	0.235	0.024	1.63E-185	Monocytes	Rasa4
1.70E-189	0.85595457	0.192	0.01	2.40E-185	Monocytes	Ifi205
4.93E-188	1.1565497	0.576	0.232	6.99E-184	Monocytes	Rrbp1
7.20E-188	-1.2917632	0.868	0.919	1.02E-183	Monocytes	Rps27
1.04E-187	0.6004767	0.218	0.019	1.48E-183	Monocytes	Acvrl1
8.52E-187	-0.8431905	0.916	0.912	1.21E-182	Monocytes	Rps15a
9.19E-187	0.82623642	0.291	0.047	1.30E-182	Monocytes	Ccl9
1.01E-186	0.5872317	0.3	0.049	1.43E-182	Monocytes	Csf2rb
3.32E-186	0.7281213	0.254	0.032	4.69E-182	Monocytes	Clec4a2
8.99E-186	0.75030355	0.4	0.1	1.27E-181	Monocytes	Eif4ebp1
3.90E-184	1.4398342	0.615	0.289	5.53E-180	Monocytes	Ctsc
1.94E-183	0.65713915	0.199	0.014	2.74E-179	Monocytes	Cebpa
3.18E-182	0.77543745	0.461	0.137	4.50E-178	Monocytes	Tnfrsf1a
1.77E-181	0.76679508	0.272	0.041	2.50E-177	Monocytes	Tlr2
5.56E-181	0.69329407	0.276	0.043	7.87E-177	Monocytes	Fcgrt
1.54E-180	-2.6768138	0.039	0.371	2.18E-176	Monocytes	Ccr7
3.31E-180	0.92533503	0.176	0.008	4.69E-176	Monocytes	Treml4
1.00E-179	0.48791015	0.186	0.011	1.42E-175	Monocytes	Tifab
2.28E-179	0.71124558	0.244	0.03	3.22E-175	Monocytes	Ltb4r1
1.86E-177	0.55990958	0.191	0.013	2.63E-173	Monocytes	Ptpro
2.15E-177	0.64218198	0.214	0.021	3.04E-173	Monocytes	Rab7b
2.79E-177	0.59127311	0.205	0.018	3.95E-173	Monocytes	Batf3
1.19E-175	0.70522536	0.28	0.045	1.68E-171	Monocytes	Emilin2
9.38E-174	0.74118726	0.365	0.086	1.33E-169	Monocytes	Skap2
1.30E-173	-1.5776135	0.17	0.501	1.84E-169	Monocytes	Gimap6
9.57E-173	0.92139785	0.22	0.024	1.35E-168	Monocytes	Gm9733
1.45E-170	-1.2547958	0.269	0.625	2.05E-166	Monocytes	Ptprcap
1.20E-169	-0.8418423	0.869	0.897	1.70E-165	Monocytes	Rpl21
1.52E-169	1.00019235	0.345	0.083	2.15E-165	Monocytes	Tcirg1
8.82E-169	0.9663366	0.516	0.193	1.25E-164	Monocytes	Gm2a
8.93E-169	0.6809453	0.229	0.028	1.26E-164	Monocytes	Adgre1
1.12E-167	0.66452367	0.287	0.053	1.59E-163	Monocytes	Oas1a
2.72E-167	0.68826596	0.271	0.045	3.85E-163	Monocytes	Cfp
8.17E-167	-0.825548	0.864	0.906	1.16E-162	Monocytes	Rps21
2.44E-165	0.42391158	0.301	0.056	3.46E-161	Monocytes	Gda
3.50E-165	0.8721045	0.916	0.543	4.96E-161	Monocytes	Cd74
4.05E-162	1.42535827	0.715	0.426	5.73E-158	Monocytes	H2-Eb1
1.43E-161	-0.7471071	0.951	0.935	2.03E-157	Monocytes	Rps24
2.65E-161	0.54821495	0.234	0.033	3.75E-157	Monocytes	Adssl1
1.70E-160	-1.0750461	0.672	0.788	2.41E-156	Monocytes	Rpl12

1.92E-160	0.51476347	0.203	0.021	2.71E-156	Monocytes	Adam15
3.56E-160	-1.3258085	0.393	0.658	5.04E-156	Monocytes	Cd37
3.62E-160	0.54929218	0.217	0.026	5.13E-156	Monocytes	Plod3
1.82E-159	0.68533678	0.171	0.012	2.58E-155	Monocytes	Gm21188
1.88E-159	0.88545164	0.464	0.16	2.67E-155	Monocytes	Zbp1
4.05E-158	-0.6998346	0.86	0.897	5.73E-154	Monocytes	Rps13
4.09E-158	0.96570892	0.342	0.09	5.79E-154	Monocytes	Cndp2
6.62E-157	0.92829628	0.537	0.211	9.37E-153	Monocytes	Nfe2l2
1.47E-156	0.6726194	0.276	0.052	2.08E-152	Monocytes	Basp1
3.17E-156	0.67845567	0.143	0.004	4.49E-152	Monocytes	F10
1.05E-155	0.58396494	0.167	0.011	1.48E-151	Monocytes	Mmp14
5.65E-155	0.50359769	0.174	0.014	8.00E-151	Monocytes	Ppfia4
8.31E-155	0.49784796	0.184	0.017	1.18E-150	Monocytes	Tbxas1
3.30E-154	-3.2515049	0.034	0.325	4.67E-150	Monocytes	Cd79a
7.20E-154	0.65200554	0.203	0.024	1.02E-149	Monocytes	C3ar1
1.24E-153	0.69899883	0.277	0.055	1.75E-149	Monocytes	Snx10
1.17E-152	0.53631046	0.201	0.023	1.65E-148	Monocytes	Tifa
3.40E-152	0.49958265	0.297	0.062	4.82E-148	Monocytes	Nfam1
3.71E-152	0.61934185	0.248	0.042	5.25E-148	Monocytes	Naga
3.75E-152	0.55549601	0.188	0.018	5.31E-148	Monocytes	Tlr13
4.58E-152	0.87126172	0.362	0.102	6.48E-148	Monocytes	Trafd1
5.19E-152	0.86042065	0.858	0.811	7.35E-148	Monocytes	Itm2b
4.09E-151	0.50769472	0.148	0.006	5.80E-147	Monocytes	Tnfsf13
8.03E-151	0.58260214	0.186	0.019	1.14E-146	Monocytes	Ccdc88a
1.42E-150	0.6377685	0.301	0.067	2.01E-146	Monocytes	Xdh
1.75E-150	0.75832878	0.365	0.1	2.47E-146	Monocytes	Hexa
1.91E-150	0.55271425	0.231	0.035	2.70E-146	Monocytes	Tep1
1.22E-149	0.43307068	0.301	0.064	1.73E-145	Monocytes	Clec4e
1.97E-149	0.486783	0.176	0.015	2.79E-145	Monocytes	Itgb5
1.99E-148	0.67857246	0.157	0.01	2.82E-144	Monocytes	Gbp2b
2.83E-148	0.45681388	0.181	0.018	4.01E-144	Monocytes	Scarb1
3.05E-148	0.59815371	0.202	0.025	4.32E-144	Monocytes	Slc31a2
1.17E-147	0.50800447	0.152	0.008	1.65E-143	Monocytes	Tcf7l2
1.12E-146	-1.0164718	0.616	0.772	1.59E-142	Monocytes	Shisa5
2.09E-146	0.58311585	0.175	0.016	2.95E-142	Monocytes	Ldlr
4.21E-146	0.47113201	0.154	0.009	5.96E-142	Monocytes	Cttnbp2nl
6.18E-146	0.44451335	0.181	0.018	8.75E-142	Monocytes	Zeb2os
1.51E-145	0.49476937	0.35	0.089	2.14E-141	Monocytes	Hp
3.16E-145	-0.6266072	0.903	0.929	4.47E-141	Monocytes	Rpl30
3.55E-145	0.45741312	0.168	0.014	5.03E-141	Monocytes	Itga5
7.73E-144	1.39031218	0.143	0.007	1.09E-139	Monocytes	Arg1
1.04E-143	-1.21382	0.245	0.564	1.48E-139	Monocytes	Cd2
1.67E-143	0.8048365	0.237	0.041	2.36E-139	Monocytes	Tmem176a
3.37E-143	-2.3146255	0.021	0.295	4.77E-139	Monocytes	Ebf1
5.30E-143	0.50738065	0.136	0.005	7.50E-139	Monocytes	Anpep
7.81E-142	1.22511328	0.534	0.249	1.11E-137	Monocytes	Lamp1
1.69E-141	0.87262125	0.576	0.264	2.39E-137	Monocytes	Efhd2
2.69E-141	0.68840247	0.127	0.003	3.81E-137	Monocytes	Adgre4

2.09E-140	0.63796163	0.312	0.077	2.96E-136	Monocytes	Etv6
2.11E-140	-0.6997274	0.9	0.905	2.99E-136	Monocytes	Rps7
3.64E-140	0.77545217	0.392	0.123	5.15E-136	Monocytes	Lamp2
4.01E-140	0.93650597	0.139	0.007	5.68E-136	Monocytes	Apoc2
3.99E-139	0.53280231	0.167	0.015	5.64E-135	Monocytes	Myof
4.27E-139	0.50535293	0.335	0.092	6.04E-135	Monocytes	Ccrl2
4.30E-138	-0.6026253	0.923	0.932	6.09E-134	Monocytes	Rps3a1
1.84E-137	0.52298422	0.161	0.014	2.60E-133	Monocytes	Creb5
7.91E-137	0.77232645	0.495	0.194	1.12E-132	Monocytes	Ifnar2
9.50E-137	0.67554679	0.232	0.043	1.34E-132	Monocytes	Anxa4
2.42E-136	0.53306763	0.268	0.056	3.42E-132	Monocytes	Itgam
3.04E-136	0.67927763	0.304	0.074	4.30E-132	Monocytes	Rnase6
5.77E-136	-1.2712899	0.261	0.558	8.17E-132	Monocytes	Ltb
5.79E-136	0.4553998	0.271	0.057	8.19E-132	Monocytes	Pilra
6.35E-135	0.98359754	0.137	0.007	8.99E-131	Monocytes	Fn1
8.51E-135	0.91621466	0.567	0.271	1.20E-130	Monocytes	Ctsa
1.75E-134	-0.7063434	0.889	0.894	2.48E-130	Monocytes	Rpl39
3.95E-134	0.8612684	0.468	0.187	5.60E-130	Monocytes	Rnh1
1.07E-132	0.83326517	0.826	0.583	1.52E-128	Monocytes	Fos
2.97E-132	0.46010825	0.372	0.112	4.21E-128	Monocytes	Trib1
3.47E-132	0.51403986	0.167	0.017	4.92E-128	Monocytes	Atp1a3
1.26E-131	-0.7985731	0.815	0.875	1.78E-127	Monocytes	Rpl17
2.65E-131	0.41733471	0.189	0.025	3.75E-127	Monocytes	Milr1
3.03E-130	-0.6509689	0.844	0.88	4.29E-126	Monocytes	Rpl36
3.31E-130	0.49340122	0.178	0.022	4.68E-126	Monocytes	Hk3
4.81E-130	0.42743866	0.13	0.006	6.81E-126	Monocytes	Sh3pxd2b
6.44E-130	0.78748787	0.268	0.063	9.12E-126	Monocytes	Irgm1
1.03E-129	0.74750149	0.41	0.142	1.46E-125	Monocytes	Ninj1
5.03E-129	0.4514054	0.203	0.032	7.12E-125	Monocytes	Cmtm3
2.05E-128	0.40796278	0.18	0.023	2.91E-124	Monocytes	Rab32
3.34E-128	0.4397189	0.217	0.038	4.73E-124	Monocytes	Ebi3
4.54E-128	0.45957999	0.167	0.018	6.42E-124	Monocytes	Gpr35
4.93E-127	-1.0438748	0.216	0.502	6.98E-123	Monocytes	1-Sep
9.25E-127	0.70364176	0.227	0.044	1.31E-122	Monocytes	Atp13a2
1.11E-126	0.60674418	0.335	0.099	1.57E-122	Monocytes	Gusb
1.18E-126	0.58764272	0.343	0.1	1.68E-122	Monocytes	Lmo4
2.68E-126	0.44439779	0.137	0.009	3.79E-122	Monocytes	Msr1
9.17E-126	0.60870525	0.309	0.083	1.30E-121	Monocytes	Il6ra
4.12E-125	1.57611054	0.133	0.009	5.83E-121	Monocytes	Fabp4
6.45E-125	0.55860074	0.246	0.052	9.13E-121	Monocytes	Scimp
9.01E-125	1.00026557	0.687	0.421	1.27E-120	Monocytes	Samhd1
1.22E-124	-2.2409531	0.029	0.282	1.73E-120	Monocytes	Ly6d
1.32E-124	-1.4428795	0.028	0.276	1.87E-120	Monocytes	Satb1
2.55E-124	0.39812867	0.14	0.011	3.62E-120	Monocytes	Shtn1
3.52E-124	0.48402545	0.149	0.013	4.98E-120	Monocytes	Tns3
3.87E-123	-1.2655966	0.129	0.395	5.48E-119	Monocytes	Ablim1
4.68E-123	-0.5778412	0.902	0.92	6.62E-119	Monocytes	Rps10
8.68E-123	0.41556887	0.358	0.112	1.23E-118	Monocytes	Rnf149

1.06E-122	0.85405777	0.47	0.191	1.50E-118	Monocytes	Clic4
1.27E-122	-1.0532838	0.674	0.82	1.79E-118	Monocytes	Jund
2.05E-122	0.55055158	0.942	0.918	2.91E-118	Monocytes	B2m
2.94E-122	0.31980848	0.145	0.012	4.17E-118	Monocytes	Ugt1a7c
8.72E-122	0.60020163	0.202	0.035	1.23E-117	Monocytes	Hk2
9.85E-122	0.49206302	0.131	0.009	1.40E-117	Monocytes	Tbc1d9
2.74E-121	0.54925907	0.23	0.046	3.88E-117	Monocytes	Il1rn
2.75E-121	-0.5372785	0.887	0.967	3.89E-117	Monocytes	Eif1
4.70E-121	-0.6041924	0.874	0.912	6.65E-117	Monocytes	Rpl8
5.76E-121	0.45567552	0.235	0.048	8.16E-117	Monocytes	Lpcat2
6.94E-121	-0.5368318	0.941	0.945	9.83E-117	Monocytes	Uba52
1.62E-120	0.94463291	0.726	0.526	2.29E-116	Monocytes	Tspo
1.81E-120	0.94137338	0.538	0.248	2.56E-116	Monocytes	Napsa
8.24E-120	-0.5581139	0.928	0.94	1.17E-115	Monocytes	Rpl18a
9.06E-120	0.97447727	0.528	0.254	1.28E-115	Monocytes	Pnp
1.32E-119	0.47034171	0.123	0.007	1.87E-115	Monocytes	Arhgef10l
1.72E-119	-1.7485096	0.008	0.239	2.44E-115	Monocytes	Fcmr
2.28E-119	1.00297561	0.562	0.297	3.22E-115	Monocytes	H2-DMa
2.47E-119	0.82637382	0.158	0.019	3.50E-115	Monocytes	Eno3
2.55E-119	0.38269317	0.182	0.027	3.61E-115	Monocytes	Aldh3b1
2.94E-119	0.46206277	0.107	0.002	4.16E-115	Monocytes	Zfyve9
3.49E-119	0.78078542	0.782	0.555	4.93E-115	Monocytes	Zfp36
5.09E-119	-0.6553235	0.903	0.908	7.21E-115	Monocytes	Rplp1
1.35E-118	0.71572002	0.295	0.082	1.91E-114	Monocytes	Ccr2
2.15E-118	0.42485399	0.12	0.006	3.04E-114	Monocytes	Adap2
2.31E-118	0.50851213	0.248	0.055	3.27E-114	Monocytes	Cd300lf
3.86E-118	0.46580479	0.192	0.032	5.47E-114	Monocytes	S100a1
3.97E-118	0.35844444	0.131	0.009	5.61E-114	Monocytes	Csf2rb2
5.07E-118	0.93989999	0.55	0.266	7.18E-114	Monocytes	Tpi1
6.23E-118	1.30035129	0.62	0.4	8.81E-114	Monocytes	Ly6a
1.18E-117	-1.4182913	0.017	0.25	1.67E-113	Monocytes	Cd55
1.45E-117	0.45709486	0.336	0.1	2.05E-113	Monocytes	Anxa1
3.57E-117	0.30389678	0.223	0.043	5.05E-113	Monocytes	Sirpb1b
3.71E-117	0.63082046	0.295	0.083	5.25E-113	Monocytes	Casp1
1.13E-116	0.36898464	0.281	0.071	1.60E-112	Monocytes	Cd300ld
1.60E-116	0.3410765	0.219	0.043	2.26E-112	Monocytes	Ogfrl1
2.09E-116	0.75708836	0.517	0.225	2.95E-112	Monocytes	Il10ra
2.94E-116	0.85128273	0.176	0.027	4.17E-112	Monocytes	Ecm1
3.32E-116	0.60474163	0.183	0.029	4.69E-112	Monocytes	Cbfa2t3
3.92E-116	0.44535789	0.12	0.006	5.55E-112	Monocytes	Gm4951
4.97E-116	0.42965703	0.177	0.026	7.03E-112	Monocytes	Camk1
9.53E-116	0.48285364	0.15	0.016	1.35E-111	Monocytes	Ccnd1
2.45E-115	0.98959845	0.722	0.431	3.47E-111	Monocytes	H2-Aa
2.52E-115	-0.7357667	0.919	0.896	3.56E-111	Monocytes	Rps20
3.86E-115	0.32776937	0.122	0.007	5.47E-111	Monocytes	F11r
7.32E-115	0.64732774	0.312	0.093	1.04E-110	Monocytes	Abi3
1.61E-114	-1.6056527	0.008	0.231	2.28E-110	Monocytes	Ms4a1
1.91E-114	0.39558785	0.221	0.045	2.70E-110	Monocytes	Oasl2

2.98E-114	0.58695933	0.264	0.067	4.22E-110	Monocytes	Cyfip1
5.45E-114	1.04689841	0.481	0.215	7.72E-110	Monocytes	Socs3
7.91E-114	0.32972098	0.257	0.061	1.12E-109	Monocytes	Slc15a3
1.04E-113	-1.4578369	0.034	0.267	1.48E-109	Monocytes	S1pr1
2.01E-113	0.63110023	0.397	0.144	2.85E-109	Monocytes	Znhit1
2.08E-113	0.48078217	0.163	0.022	2.95E-109	Monocytes	Aoah
5.96E-113	0.5129605	0.138	0.013	8.44E-109	Monocytes	Cdc42ep2
7.26E-113	-1.1664011	0.713	0.815	1.03E-108	Monocytes	Btg1
9.23E-113	0.37240581	0.129	0.01	1.31E-108	Monocytes	Phf11d
8.21E-112	-0.5428709	0.893	0.927	1.16E-107	Monocytes	Rpl9-ps6
8.90E-112	0.86289758	0.631	0.342	1.26E-107	Monocytes	Irf1
8.92E-112	0.85921396	0.389	0.144	1.26E-107	Monocytes	Ms4a4c
2.10E-111	0.51507931	0.731	0.511	2.97E-107	Monocytes	Cebpb
2.11E-111	0.35745584	0.124	0.009	2.99E-107	Monocytes	Tnfrsf21
2.79E-111	0.56497485	0.361	0.123	3.95E-107	Monocytes	Ptpre
3.44E-111	0.89585638	0.409	0.163	4.86E-107	Monocytes	Smpdl3a
3.76E-111	0.90105041	0.591	0.33	5.32E-107	Monocytes	Akr1a1
7.84E-111	0.56064121	0.29	0.082	1.11E-106	Monocytes	Gyg
3.30E-110	0.28466079	0.118	0.007	4.67E-106	Monocytes	Guca1a
4.35E-110	0.67986038	0.402	0.148	6.16E-106	Monocytes	Cyp4f18
4.50E-110	0.39247079	0.466	0.182	6.37E-106	Monocytes	Tpd52
5.56E-110	-0.5415249	0.932	0.945	7.87E-106	Monocytes	Rps16
1.08E-109	0.36594749	0.19	0.034	1.53E-105	Monocytes	Ralb
1.24E-109	0.40812818	0.172	0.027	1.76E-105	Monocytes	Rbm47
1.25E-109	0.55444666	0.161	0.022	1.77E-105	Monocytes	Id1
1.39E-109	0.42094828	0.239	0.055	1.96E-105	Monocytes	Gng12
2.37E-109	0.57284258	0.262	0.069	3.35E-105	Monocytes	Casp4
3.71E-109	0.39179265	0.181	0.03	5.26E-105	Monocytes	Pira2
4.40E-109	0.53976524	0.195	0.036	6.24E-105	Monocytes	Slfn5
8.73E-109	0.44451763	0.22	0.047	1.24E-104	Monocytes	Dusp3
1.11E-108	0.4294183	0.144	0.016	1.58E-104	Monocytes	Axl
1.13E-108	0.30861919	0.123	0.009	1.60E-104	Monocytes	Hsd3b7
1.49E-108	0.77113705	0.656	0.384	2.11E-104	Monocytes	Vamp8
1.89E-108	0.68178939	0.378	0.137	2.67E-104	Monocytes	Ppt1
2.41E-108	0.43530215	0.15	0.019	3.41E-104	Monocytes	Acp2
2.97E-108	-0.4838994	0.936	0.953	4.20E-104	Monocytes	Gm10076
2.15E-107	-0.2593942	0.295	0.081	3.04E-103	Monocytes	Tnfaip2
2.25E-107	0.4302162	0.103	0.004	3.19E-103	Monocytes	Ms4a8a
2.66E-107	0.82970263	0.551	0.289	3.77E-103	Monocytes	Cstb
4.51E-107	-0.5473319	0.882	0.898	6.38E-103	Monocytes	Rpl27a
5.75E-107	0.73432052	0.423	0.168	8.14E-103	Monocytes	Prkcd
6.98E-107	0.54809279	0.259	0.068	9.89E-103	Monocytes	Bach1
7.16E-107	0.39156239	0.124	0.01	1.01E-102	Monocytes	Stom
1.07E-106	-0.5827248	0.901	0.914	1.51E-102	Monocytes	Rpl37a
3.03E-106	0.36772521	0.164	0.024	4.29E-102	Monocytes	Pdlim4
3.63E-106	0.31473042	0.143	0.016	5.14E-102	Monocytes	Pak1
3.72E-106	0.37199384	0.215	0.045	5.27E-102	Monocytes	Trf
1.07E-105	0.61609018	0.325	0.104	1.51E-101	Monocytes	Blvrb

1.54E-105	-1.1642449	0.127	0.366	2.18E-101	Monocytes	Gm8369
1.77E-105	0.87771212	0.528	0.26	2.50E-101	Monocytes	Bst2
2.39E-105	0.60897459	0.372	0.133	3.38E-101	Monocytes	Picalm
3.77E-105	0.56333522	0.955	0.91	5.34E-101	Monocytes	Gapdh
5.75E-105	0.64316354	0.277	0.079	8.13E-101	Monocytes	Nampt
2.42E-104	-0.6657284	0.855	0.888	3.42E-100	Monocytes	Rpl38
3.13E-104	0.40357187	0.148	0.019	4.43E-100	Monocytes	Eps8
4.51E-104	0.70457257	0.382	0.145	6.38E-100	Monocytes	Ly6c2
1.29E-103	0.50186102	0.251	0.066	1.82E-99	Monocytes	Mvp
2.04E-103	0.67982328	0.336	0.116	2.88E-99	Monocytes	Gadd45g
2.29E-103	-0.4965876	0.951	0.933	3.24E-99	Monocytes	Rpl13
3.80E-103	-0.5552558	0.949	0.92	5.38E-99	Monocytes	Rpsa
4.54E-103	0.50429156	0.332	0.11	6.43E-99	Monocytes	Evi2a
4.76E-103	0.33083819	0.134	0.014	6.74E-99	Monocytes	Zfp385a
6.27E-103	-0.5281517	0.894	0.911	8.87E-99	Monocytes	Rps3
3.25E-102	0.39507515	0.12	0.01	4.60E-98	Monocytes	Sash1
7.37E-102	-0.8937846	0.616	0.754	1.04E-97	Monocytes	Pnrc1
9.98E-102	0.33903674	0.112	0.008	1.41E-97	Monocytes	Krt80
1.23E-101	0.4769011	0.218	0.051	1.74E-97	Monocytes	Cers6
1.23E-101	-1.3061664	0.006	0.207	1.74E-97	Monocytes	Cd19
1.31E-101	0.27412967	0.111	0.007	1.86E-97	Monocytes	Cpq
1.76E-101	0.37838011	0.136	0.016	2.49E-97	Monocytes	Dram1
2.36E-101	0.77440364	0.581	0.317	3.34E-97	Monocytes	Psmb10
3.63E-101	0.74393718	0.639	0.36	5.14E-97	Monocytes	Fyb
9.64E-101	0.33632045	0.174	0.031	1.36E-96	Monocytes	Dnase1l1
2.67E-100	0.38944877	0.221	0.052	3.79E-96	Monocytes	Rab20
3.09E-100	0.53896063	0.317	0.106	4.37E-96	Monocytes	Gsdmd
5.00E-100	0.33240103	0.125	0.012	7.08E-96	Monocytes	I830077J02Ri
5.07E-100	0.53936942	0.316	0.105	7.18E-96	Monocytes	Ap1s2
1.02E-99	0.86963264	0.279	0.087	1.45E-95	Monocytes	Sdc4
1.03E-99	0.36405458	0.146	0.02	1.46E-95	Monocytes	Sh2d1b1
1.14E-99	0.39790493	0.105	0.006	1.61E-95	Monocytes	Mertk
1.47E-99	0.76329186	0.551	0.272	2.08E-95	Monocytes	Unc93b1
2.33E-99	0.95772386	0.697	0.473	3.29E-95	Monocytes	Pkm
2.35E-99	0.58333088	0.235	0.061	3.33E-95	Monocytes	Fam49a
4.85E-99	0.36800846	0.156	0.024	6.87E-95	Monocytes	Cpne2
9.23E-99	-1.5686063	0.052	0.267	1.31E-94	Monocytes	H2-Ob
1.09E-98	0.75417207	0.573	0.313	1.54E-94	Monocytes	Laptm4a
1.46E-98	0.33261356	0.122	0.012	2.07E-94	Monocytes	Nfix
2.18E-98	0.46425747	0.249	0.068	3.09E-94	Monocytes	Ehbp1l1
2.26E-98	0.3185919	0.134	0.016	3.20E-94	Monocytes	Cln8
2.62E-98	0.67449594	0.999	0.998	3.71E-94	Monocytes	Gm42418
2.67E-98	0.49202472	0.216	0.051	3.78E-94	Monocytes	Gm6377
4.83E-98	0.30684804	0.107	0.007	6.84E-94	Monocytes	Batf2
5.43E-98	0.87406285	0.495	0.233	7.69E-94	Monocytes	Klf4
1.02E-97	0.41414754	0.12	0.011	1.45E-93	Monocytes	4930430E12F
1.41E-97	0.48163242	0.111	0.009	2.00E-93	Monocytes	Zmynd15
1.47E-97	0.53298344	0.387	0.149	2.07E-93	Monocytes	Rgs10

1.64E-97	0.39605763	0.217	0.051	2.33E-93	Monocytes	Nuak2
4.03E-97	0.59603479	0.315	0.106	5.71E-93	Monocytes	Gsn
4.50E-97	0.36144498	0.291	0.088	6.37E-93	Monocytes	Plekho1
4.60E-97	0.39285837	0.148	0.022	6.51E-93	Monocytes	Tmem86a
4.87E-97	0.51420548	0.269	0.079	6.89E-93	Monocytes	Rnf19b
5.31E-97	0.7489989	0.644	0.4	7.51E-93	Monocytes	Pomp
9.16E-97	-0.4650489	0.929	0.941	1.30E-92	Monocytes	Rpl23
1.25E-96	0.68585987	0.266	0.08	1.77E-92	Monocytes	Bag3
1.39E-96	1.0915346	0.289	0.096	1.97E-92	Monocytes	Fabp5
1.56E-96	0.25720417	0.131	0.015	2.21E-92	Monocytes	Ubtd1
8.59E-96	0.41423987	0.248	0.069	1.22E-91	Monocytes	Rnpep
2.46E-95	0.32333365	0.145	0.021	3.48E-91	Monocytes	Bst1
2.55E-95	0.39946826	0.119	0.012	3.61E-91	Monocytes	Sowahc
2.69E-95	0.76505334	0.181	0.037	3.80E-91	Monocytes	Ceacam1
2.77E-95	-0.4775482	0.905	0.908	3.92E-91	Monocytes	Rpl34
4.47E-95	-0.4767696	0.89	0.944	6.33E-91	Monocytes	Rpl37
1.11E-94	0.79330364	0.13	0.016	1.57E-90	Monocytes	Hpgd
1.72E-94	0.41951664	0.158	0.027	2.44E-90	Monocytes	Slc8b1
2.32E-94	0.7815334	0.515	0.257	3.28E-90	Monocytes	Syngr2
1.19E-93	0.412977	0.268	0.081	1.68E-89	Monocytes	Sap30
3.80E-93	0.27887933	0.115	0.011	5.38E-89	Monocytes	Aph1c
5.48E-93	0.67299615	0.424	0.185	7.76E-89	Monocytes	Irf7
6.25E-93	-0.6982658	0.75	0.804	8.85E-89	Monocytes	Rpl36a
1.31E-92	0.55269827	0.372	0.144	1.86E-88	Monocytes	Ehd4
2.02E-92	0.55502	0.24	0.068	2.86E-88	Monocytes	Dram2
5.82E-92	-1.0525593	0.161	0.388	8.24E-88	Monocytes	Gimap3
5.98E-92	0.53788643	0.412	0.17	8.46E-88	Monocytes	Fam174a
1.11E-91	0.60339809	0.124	0.015	1.58E-87	Monocytes	F13a1
1.23E-91	0.35063053	0.149	0.024	1.73E-87	Monocytes	Tlr7
3.19E-91	0.26806896	0.126	0.015	4.52E-87	Monocytes	Gab2
3.79E-91	0.70901647	0.32	0.117	5.36E-87	Monocytes	Lgals3bp
3.95E-91	0.47655831	0.484	0.223	5.59E-87	Monocytes	Cd44
6.88E-91	0.45156224	0.274	0.085	9.74E-87	Monocytes	Dstn
7.36E-91	0.34027015	0.105	0.008	1.04E-86	Monocytes	Tbc1d8
7.45E-91	-0.4763091	0.946	0.939	1.05E-86	Monocytes	Rps8
2.86E-90	0.30352647	0.122	0.014	4.04E-86	Monocytes	Cyp4f16
4.04E-90	0.52089926	0.304	0.105	5.72E-86	Monocytes	Metrnl
4.28E-90	-1.0749334	0.515	0.658	6.06E-86	Monocytes	Stk17b
7.16E-90	0.27534862	0.193	0.043	1.01E-85	Monocytes	Hacd4
7.23E-90	0.44787294	0.297	0.099	1.02E-85	Monocytes	Myd88
9.69E-90	0.29336001	0.131	0.018	1.37E-85	Monocytes	Tarm1
2.16E-89	0.67372168	0.481	0.232	3.06E-85	Monocytes	Man2b1
2.45E-89	0.69304894	0.402	0.175	3.46E-85	Monocytes	Nfil3
2.52E-89	0.80801293	0.303	0.108	3.57E-85	Monocytes	Sgk1
3.11E-89	0.72984339	0.543	0.292	4.40E-85	Monocytes	Dbi
5.06E-89	0.28655756	0.135	0.019	7.17E-85	Monocytes	Renbp
5.43E-89	0.31276962	0.113	0.011	7.69E-85	Monocytes	Fgd4
8.70E-89	0.47360604	0.356	0.138	1.23E-84	Monocytes	Dynlt1b

1.42E-88	0.35002884	0.171	0.035	2.00E-84	Monocytes	Dnajb14
1.44E-88	0.50845611	0.356	0.137	2.03E-84	Monocytes	Fkbp2
1.83E-88	0.39615993	0.125	0.016	2.60E-84	Monocytes	Lacc1
3.25E-88	0.28376749	0.165	0.031	4.60E-84	Monocytes	Slc2a6
3.52E-88	0.49898153	0.29	0.096	4.98E-84	Monocytes	Scpep1
3.65E-88	0.68714361	0.133	0.02	5.16E-84	Monocytes	Ccl12
5.07E-88	0.44175173	0.197	0.047	7.17E-84	Monocytes	Rbpms
7.98E-88	-0.6843421	0.749	0.842	1.13E-83	Monocytes	Ddx5
1.68E-87	0.52310727	0.828	0.764	2.38E-83	Monocytes	Arpc1b
1.88E-87	0.60903466	0.537	0.267	2.66E-83	Monocytes	Lyn
2.09E-87	0.66760517	0.762	0.572	2.96E-83	Monocytes	Dusp1
2.21E-87	0.51022018	0.241	0.07	3.13E-83	Monocytes	Cd40
3.55E-87	0.28535519	0.138	0.021	5.03E-83	Monocytes	St3gal5
4.31E-87	0.70056414	0.591	0.329	6.10E-83	Monocytes	Slfn2
6.26E-87	-0.5006498	0.914	0.904	8.86E-83	Monocytes	Rps5
6.78E-87	0.69004059	0.611	0.373	9.59E-83	Monocytes	Erp29
8.14E-87	0.480339	0.27	0.087	1.15E-82	Monocytes	Gsto1
1.06E-86	0.53876725	0.224	0.063	1.50E-82	Monocytes	Gbp3
1.39E-86	0.7485356	0.53	0.275	1.96E-82	Monocytes	Zfand5
1.70E-86	-1.1117787	0.087	0.293	2.41E-82	Monocytes	P2ry10
2.02E-86	0.31302666	0.13	0.019	2.86E-82	Monocytes	Parp12
2.26E-86	0.53089333	0.424	0.185	3.19E-82	Monocytes	Cnih4
4.90E-86	0.31824487	0.102	0.009	6.93E-82	Monocytes	Fam129b
5.61E-86	0.33062279	0.126	0.017	7.94E-82	Monocytes	Rnase4
5.79E-86	0.31049135	0.105	0.01	8.19E-82	Monocytes	Eng
6.12E-86	-1.7088472	0.089	0.29	8.67E-82	Monocytes	H2-DMb2
6.78E-86	-0.9637576	0.157	0.371	9.60E-82	Monocytes	Gimap1
1.63E-85	0.97486693	0.116	0.014	2.31E-81	Monocytes	Ccl7
1.75E-85	0.4191132	0.282	0.094	2.48E-81	Monocytes	Rsu1
1.83E-85	0.3545618	0.155	0.029	2.59E-81	Monocytes	Pilrb2
1.88E-85	0.6648375	0.643	0.396	2.67E-81	Monocytes	Atp6v0b
2.71E-85	0.46802527	0.426	0.182	3.84E-81	Monocytes	AB124611
3.38E-85	0.67435831	0.26	0.084	4.78E-81	Monocytes	Cd274
4.56E-85	0.45707531	0.329	0.124	6.46E-81	Monocytes	Tmed3
5.62E-85	0.30099215	0.104	0.01	7.95E-81	Monocytes	Dock4
1.17E-84	-0.6994176	0.71	0.831	1.66E-80	Monocytes	Rac2
1.68E-84	0.49959122	0.267	0.088	2.37E-80	Monocytes	Abhd12
2.11E-84	0.56953413	0.352	0.142	2.99E-80	Monocytes	Itm2c
3.81E-84	-1.1981386	0.006	0.176	5.39E-80	Monocytes	Fcer2a
4.41E-84	0.47693607	0.171	0.037	6.24E-80	Monocytes	Frmd4b
6.02E-84	0.42236008	0.249	0.077	8.52E-80	Monocytes	Ube2l6
6.14E-84	0.47801007	0.143	0.025	8.70E-80	Monocytes	Gbp5
1.68E-83	-1.1323499	0.016	0.192	2.38E-79	Monocytes	Bank1
1.74E-83	0.36060099	0.159	0.032	2.47E-79	Monocytes	Tmbim1
2.23E-83	0.71817479	0.53	0.292	3.15E-79	Monocytes	Aprt
9.67E-83	0.49974146	0.245	0.076	1.37E-78	Monocytes	Ptprj
9.72E-83	0.50782937	0.889	0.801	1.38E-78	Monocytes	Ly6e
1.82E-82	-0.4843463	0.913	0.885	2.57E-78	Monocytes	Rpl32

2.52E-82	0.37460146	0.159	0.032	3.56E-78	Monocytes	Adam9
6.72E-82	0.40030039	0.22	0.063	9.51E-78	Monocytes	Ppt2
1.15E-81	0.32927053	0.131	0.02	1.62E-77	Monocytes	Il13ra1
1.25E-81	0.28708597	0.108	0.012	1.77E-77	Monocytes	Dna2
1.48E-81	0.71853594	0.144	0.027	2.09E-77	Monocytes	Cd36
1.84E-81	0.42453124	0.101	0.01	2.60E-77	Monocytes	Upp1
2.11E-81	0.60358821	0.446	0.211	2.99E-77	Monocytes	Ak2
2.17E-81	-0.5255466	0.834	0.849	3.07E-77	Monocytes	Rpl3
2.95E-81	0.315992	0.122	0.017	4.17E-77	Monocytes	Cmklr1
6.64E-81	0.38734159	0.126	0.019	9.40E-77	Monocytes	Rap1gap2
7.98E-81	0.71599269	0.724	0.558	1.13E-76	Monocytes	Prdx1
2.34E-80	0.46088525	0.333	0.131	3.32E-76	Monocytes	Tmem219
2.58E-80	0.46598209	0.223	0.064	3.65E-76	Monocytes	Fgd2
1.59E-79	0.31882298	0.108	0.013	2.26E-75	Monocytes	Mitf
2.15E-79	0.30178816	0.103	0.011	3.05E-75	Monocytes	Amz1
2.27E-79	-2.0043148	0.11	0.298	3.21E-75	Monocytes	Cd79b
2.75E-79	0.28874045	0.106	0.012	3.89E-75	Monocytes	Rab3il1
2.75E-79	0.44222339	0.359	0.146	3.89E-75	Monocytes	Ube2f
3.25E-79	0.31319148	0.194	0.05	4.60E-75	Monocytes	Elmo2
3.34E-79	0.61657793	0.141	0.026	4.73E-75	Monocytes	Tnip3
3.47E-79	-0.42286	0.942	0.955	4.92E-75	Monocytes	Tpt1
3.61E-79	0.44526895	0.25	0.081	5.11E-75	Monocytes	Ggh
9.03E-79	0.32761445	0.132	0.022	1.28E-74	Monocytes	Clec4n
1.53E-78	0.28889498	0.311	0.117	2.16E-74	Monocytes	Gsr
2.57E-78	-0.4798608	0.908	0.896	3.63E-74	Monocytes	Rps26
3.69E-78	0.3277073	0.195	0.052	5.22E-74	Monocytes	Ntpcr
1.26E-77	0.50721608	0.307	0.117	1.78E-73	Monocytes	Rnf213
1.90E-77	0.35365347	0.253	0.083	2.69E-73	Monocytes	Hmgcl
3.60E-77	-0.536747	0.929	0.973	5.10E-73	Monocytes	H3f3b
4.69E-77	0.39739853	0.255	0.085	6.63E-73	Monocytes	Soat1
4.79E-77	-0.893871	0.184	0.395	6.79E-73	Monocytes	Gimap4
7.89E-77	-0.4096248	0.931	0.918	1.12E-72	Monocytes	Rpl19
1.30E-76	0.29007043	0.137	0.025	1.85E-72	Monocytes	Slc12a9
1.40E-76	0.44436833	0.409	0.178	1.98E-72	Monocytes	Nrros
1.45E-76	0.30852427	0.153	0.032	2.05E-72	Monocytes	Gpr137b
3.78E-76	-0.7113383	0.733	0.786	5.36E-72	Monocytes	Eef2
4.23E-76	-1.0217194	0.006	0.162	5.98E-72	Monocytes	Blk
4.97E-76	-0.7522656	0.557	0.664	7.04E-72	Monocytes	Cnbp
7.63E-76	-0.7961553	0.81	0.849	1.08E-71	Monocytes	Rps28
1.52E-75	0.4258319	0.318	0.125	2.16E-71	Monocytes	Nadk
3.18E-75	0.25479232	0.115	0.017	4.50E-71	Monocytes	Pla2g15
4.23E-75	0.4644659	0.312	0.121	5.99E-71	Monocytes	Tm6sf1
5.71E-75	0.80038081	0.525	0.306	8.08E-71	Monocytes	Capza2
1.13E-74	0.30566532	0.162	0.037	1.60E-70	Monocytes	Rab31
1.20E-74	0.89042774	0.199	0.059	1.70E-70	Monocytes	AA467197
1.61E-74	0.38124663	0.287	0.106	2.28E-70	Monocytes	Map7d1
3.17E-74	0.62065853	0.468	0.241	4.48E-70	Monocytes	Coro1b
3.48E-74	0.40386161	0.299	0.114	4.92E-70	Monocytes	Slc6a6

6.71E-74	0.31042865	0.186	0.049	9.49E-70	Monocytes	G6pdx
7.04E-74	0.44623572	0.19	0.052	9.97E-70	Monocytes	Furin
7.92E-74	0.38924334	0.335	0.136	1.12E-69	Monocytes	Myo1f
1.14E-73	0.59672299	0.291	0.111	1.62E-69	Monocytes	Ass1
1.86E-73	0.26061136	0.104	0.013	2.63E-69	Monocytes	Anxa3
1.94E-73	0.61823647	0.741	0.545	2.75E-69	Monocytes	Nfkbia
1.98E-73	0.68541917	0.713	0.522	2.80E-69	Monocytes	Psme2
2.34E-73	0.63291604	0.524	0.286	3.31E-69	Monocytes	Stat1
7.01E-73	0.39220282	0.275	0.101	9.93E-69	Monocytes	Xaf1
8.43E-73	0.64278877	0.571	0.335	1.19E-68	Monocytes	Itgb2
8.52E-73	0.54090882	0.441	0.214	1.21E-68	Monocytes	Tmem256
1.27E-72	0.3174605	0.122	0.021	1.80E-68	Monocytes	Il15ra
1.49E-72	0.61315801	0.557	0.324	2.10E-68	Monocytes	Tmem14c
1.55E-72	0.52157807	0.558	0.309	2.20E-68	Monocytes	Zyx
1.72E-72	0.35315071	0.358	0.147	2.44E-68	Monocytes	Dok3
1.78E-72	0.26996571	0.122	0.021	2.52E-68	Monocytes	Tmem104
2.08E-72	0.33432525	0.157	0.036	2.94E-68	Monocytes	Daglb
3.23E-72	0.62925981	0.484	0.257	4.58E-68	Monocytes	Tkt
9.75E-72	-0.9756652	0.191	0.387	1.38E-67	Monocytes	Ikzf3
1.90E-71	0.70324961	0.343	0.152	2.68E-67	Monocytes	Gns
4.64E-71	0.33135537	0.117	0.019	6.56E-67	Monocytes	Dab2
6.82E-71	0.78251299	0.355	0.164	9.66E-67	Monocytes	Ifi47
7.55E-71	0.37850883	0.166	0.042	1.07E-66	Monocytes	Lap3
9.72E-71	0.53618568	0.471	0.24	1.38E-66	Monocytes	Ncf4
9.82E-71	-0.9603557	0.4	0.545	1.39E-66	Monocytes	Fam107b
1.03E-70	0.38529424	0.31	0.122	1.45E-66	Monocytes	Stx7
1.34E-70	0.65449192	0.44	0.222	1.89E-66	Monocytes	Fgl2
1.59E-70	0.60505088	0.784	0.662	2.25E-66	Monocytes	Psmb8
1.81E-70	0.3816639	0.261	0.093	2.57E-66	Monocytes	Mpp1
1.86E-70	-0.5225398	0.83	0.856	2.64E-66	Monocytes	Rpl10a
2.25E-70	0.28665004	0.227	0.074	3.18E-66	Monocytes	Eif4e3
2.28E-70	-1.2732138	0.065	0.24	3.23E-66	Monocytes	Cd24a
2.29E-70	0.38364663	0.15	0.034	3.23E-66	Monocytes	Pfkfb3
2.57E-70	0.31108591	0.137	0.028	3.63E-66	Monocytes	Cyp4v3
2.70E-70	-1.0427255	0.011	0.162	3.82E-66	Monocytes	Mzb1
3.68E-70	0.39936316	0.244	0.083	5.21E-66	Monocytes	Prcp
4.41E-70	0.25375439	0.116	0.019	6.24E-66	Monocytes	Vwa5a
1.02E-69	0.26995969	0.18	0.049	1.45E-65	Monocytes	Ctnnbip1
1.29E-69	0.50394256	0.115	0.019	1.82E-65	Monocytes	Stab1
1.38E-69	0.33858491	0.275	0.102	1.95E-65	Monocytes	Mapkapk2
1.52E-69	0.28935151	0.121	0.021	2.15E-65	Monocytes	Ly96
5.19E-69	0.31068204	0.133	0.027	7.34E-65	Monocytes	Slc29a3
7.78E-69	0.51764885	0.335	0.143	1.10E-64	Monocytes	Mef2a
1.11E-68	0.43945741	0.104	0.015	1.56E-64	Monocytes	Dst
4.29E-68	0.33248448	0.316	0.129	6.07E-64	Monocytes	Rtp4
5.81E-68	0.4281892	0.377	0.17	8.23E-64	Monocytes	Tnfaip8l2
8.86E-68	-0.6130668	0.845	0.875	1.25E-63	Monocytes	Rpl35a
9.15E-68	0.2974973	0.109	0.017	1.29E-63	Monocytes	Tnfsf13b

1.16E-67	0.48212026	0.761	0.565	1.64E-63	Monocytes	Ifi27l2a
1.17E-67	0.46119062	0.651	0.385	1.66E-63	Monocytes	Anxa2
1.97E-67	0.46408183	0.23	0.08	2.78E-63	Monocytes	Cx3cr1
3.70E-67	0.28077483	0.154	0.037	5.23E-63	Monocytes	Vav3
4.09E-67	-0.5505368	0.816	0.836	5.80E-63	Monocytes	Rps18
4.31E-67	0.42617745	0.226	0.076	6.10E-63	Monocytes	Synj1
4.45E-67	0.31020362	0.133	0.028	6.30E-63	Monocytes	Plod1
4.93E-67	-0.4201767	0.936	0.901	6.98E-63	Monocytes	Rps4x
1.47E-66	0.25028516	0.102	0.015	2.08E-62	Monocytes	Rras
1.69E-66	-0.416756	0.903	0.928	2.40E-62	Monocytes	Rpl18
1.94E-66	0.54866447	0.21	0.069	2.74E-62	Monocytes	Tgtp2
2.01E-66	-0.4957966	0.943	0.924	2.84E-62	Monocytes	Eef1a1
2.49E-66	0.3352846	0.298	0.117	3.52E-62	Monocytes	Rnf130
2.96E-66	-0.9045154	0.007	0.147	4.19E-62	Monocytes	Scd1
5.74E-66	0.43213138	0.304	0.126	8.12E-62	Monocytes	Bri3bp
5.94E-66	-1.0244601	0.19	0.373	8.41E-62	Monocytes	Lck
1.04E-65	0.39269682	0.187	0.056	1.48E-61	Monocytes	Stat2
1.06E-65	0.40171892	0.34	0.147	1.51E-61	Monocytes	Mrpl14
1.72E-65	0.29838068	0.118	0.022	2.44E-61	Monocytes	Idh1
1.82E-65	-0.8931699	0.032	0.184	2.57E-61	Monocytes	Bach2
3.39E-65	0.54400641	0.323	0.14	4.80E-61	Monocytes	Sh3bgrl
5.05E-65	0.5370761	0.569	0.338	7.15E-61	Monocytes	Ptpn1
7.74E-65	0.27368259	0.117	0.021	1.10E-60	Monocytes	Zfp710
8.33E-65	0.35315271	0.457	0.242	1.18E-60	Monocytes	Isg15
9.61E-65	-0.6847786	0.609	0.695	1.36E-60	Monocytes	Rpl22l1
1.66E-64	-0.9320404	0.014	0.156	2.34E-60	Monocytes	Fcrla
2.14E-64	0.46201246	0.396	0.188	3.04E-60	Monocytes	Hmox2
2.80E-64	0.34617168	0.233	0.08	3.96E-60	Monocytes	P2rx4
3.54E-64	-0.381786	0.917	0.91	5.01E-60	Monocytes	Rpl6
3.83E-64	0.59057802	0.215	0.074	5.42E-60	Monocytes	Tnf
4.30E-64	-0.4807172	0.836	0.867	6.09E-60	Monocytes	Rps12
5.75E-64	0.37449893	0.117	0.022	8.14E-60	Monocytes	Gk
6.11E-64	0.25793548	0.189	0.056	8.64E-60	Monocytes	Rogdi
6.90E-64	0.30278774	0.206	0.066	9.76E-60	Monocytes	Nfic
2.90E-63	0.35080498	0.207	0.068	4.11E-59	Monocytes	Ddi2
3.34E-63	0.38609279	0.244	0.091	4.72E-59	Monocytes	Bak1
4.49E-63	0.44486477	0.107	0.019	6.35E-59	Monocytes	Mrc1
1.03E-62	0.34577455	0.22	0.076	1.45E-58	Monocytes	Echs1
1.95E-62	0.60824626	0.527	0.309	2.76E-58	Monocytes	Ptms
2.29E-62	-0.9904946	0.291	0.46	3.24E-58	Monocytes	Cd69
2.35E-62	0.55264474	0.855	0.842	3.33E-58	Monocytes	Calm1
2.54E-62	0.44387933	0.264	0.103	3.59E-58	Monocytes	Piezo1
3.60E-62	0.52866082	0.208	0.072	5.10E-58	Monocytes	Fkbp5
4.32E-62	0.33907378	0.378	0.177	6.12E-58	Monocytes	Nfkbiz
7.24E-62	0.29262179	0.11	0.02	1.03E-57	Monocytes	Asph
8.15E-62	0.30484854	0.426	0.217	1.15E-57	Monocytes	Plaur
8.25E-62	-0.4530696	0.818	0.843	1.17E-57	Monocytes	Rpl7
9.04E-62	0.34301029	0.23	0.083	1.28E-57	Monocytes	Pmvk

1.25E-61	-0.4010819	0.843	0.867	1.77E-57	Monocytes	Rpl27
1.31E-61	0.32563308	0.216	0.074	1.86E-57	Monocytes	Ap2a2
1.99E-61	0.25146716	0.301	0.121	2.81E-57	Monocytes	Ifngr2
2.85E-61	0.29838853	0.103	0.017	4.03E-57	Monocytes	Naip5
4.34E-61	0.25577287	0.144	0.036	6.15E-57	Monocytes	Sh3bp2
4.81E-61	0.32653511	0.301	0.127	6.81E-57	Monocytes	Lrpap1
5.49E-61	0.28446939	0.309	0.128	7.77E-57	Monocytes	Themis2
7.45E-61	0.70135425	0.518	0.305	1.06E-56	Monocytes	Bcl2a1b
1.32E-60	0.67601265	0.45	0.246	1.87E-56	Monocytes	Pgam1
1.46E-60	0.4038681	0.275	0.112	2.07E-56	Monocytes	Igtp
1.92E-60	0.48503361	0.319	0.142	2.72E-56	Monocytes	Icam1
2.37E-60	0.27889174	0.287	0.116	3.35E-56	Monocytes	Sbno2
2.54E-60	0.30993516	0.128	0.029	3.60E-56	Monocytes	Luzp1
3.48E-60	0.66388566	0.425	0.227	4.92E-56	Monocytes	Sod2
1.07E-59	0.29684981	0.195	0.062	1.52E-55	Monocytes	Cd180
1.14E-59	0.5535603	0.757	0.624	1.61E-55	Monocytes	Psme1
1.48E-59	0.29098793	0.122	0.026	2.10E-55	Monocytes	Clcn7
1.79E-59	0.27770869	0.194	0.062	2.54E-55	Monocytes	Pgap2
1.86E-59	0.25504904	0.281	0.115	2.64E-55	Monocytes	Pgd
3.50E-59	0.51158595	0.425	0.222	4.96E-55	Monocytes	Pitpna
3.55E-59	0.38910714	0.388	0.186	5.02E-55	Monocytes	Scamp2
3.63E-59	0.3836276	0.155	0.044	5.14E-55	Monocytes	Smpdl3b
4.03E-59	0.40237262	0.232	0.086	5.70E-55	Monocytes	Adam17
4.81E-59	-1.1409178	0.332	0.497	6.81E-55	Monocytes	Ms4a4b
1.09E-58	0.31441206	0.877	0.684	1.54E-54	Monocytes	Fxyd5
1.15E-58	0.31495103	0.253	0.099	1.63E-54	Monocytes	Atp6v1a
1.25E-58	0.27897338	0.2	0.067	1.77E-54	Monocytes	Ccdc86
1.36E-58	0.31559396	0.171	0.051	1.93E-54	Monocytes	H1f0
1.62E-58	0.29204569	0.259	0.102	2.30E-54	Monocytes	Blvra
1.75E-58	0.42676379	0.324	0.146	2.48E-54	Monocytes	Sdf2l1
4.77E-58	-0.5040425	0.705	0.793	6.75E-54	Monocytes	Rbm39
1.38E-57	0.3946387	0.662	0.491	1.96E-53	Monocytes	Taldo1
2.03E-57	0.25950681	0.138	0.035	2.87E-53	Monocytes	Extl3
2.11E-57	0.26219128	0.268	0.108	2.98E-53	Monocytes	Havcr2
5.42E-57	-0.8512696	0.367	0.495	7.67E-53	Monocytes	Elf1
5.46E-57	0.40525987	0.487	0.262	7.73E-53	Monocytes	Ptpn6
6.54E-57	0.31540103	0.201	0.069	9.25E-53	Monocytes	Mapk3
7.28E-57	0.49181145	0.459	0.249	1.03E-52	Monocytes	Cyth4
8.67E-57	0.36067227	0.135	0.034	1.23E-52	Monocytes	Gstm1
8.80E-57	-0.9432384	0.231	0.407	1.25E-52	Monocytes	Lat
1.12E-56	-1.1024107	0.29	0.452	1.59E-52	Monocytes	Cd3e
1.88E-56	0.2852792	0.184	0.06	2.66E-52	Monocytes	Ufsp2
2.12E-56	0.3254413	0.169	0.052	3.01E-52	Monocytes	Gsap
2.92E-56	0.35938166	0.389	0.193	4.13E-52	Monocytes	Tm2d2
3.65E-56	0.41552767	0.291	0.126	5.16E-52	Monocytes	Parp14
3.97E-56	0.29478096	0.112	0.023	5.62E-52	Monocytes	P2ry14
4.81E-56	-0.5224941	0.723	0.766	6.81E-52	Monocytes	Rpl5
1.41E-55	0.2947652	0.125	0.03	1.99E-51	Monocytes	Lpar6

2.54E-55	-0.4814321	0.233	0.084	3.59E-51	Monocytes	Trem1
4.83E-55	0.6104185	0.628	0.449	6.84E-51	Monocytes	Psma7
4.87E-55	-0.6985959	0.003	0.117	6.90E-51	Monocytes	Cxcr5
5.07E-55	0.53912642	0.535	0.317	7.18E-51	Monocytes	Flna
6.24E-55	0.45919134	0.362	0.179	8.84E-51	Monocytes	Phf11b
8.17E-55	0.29364787	0.446	0.235	1.16E-50	Monocytes	Card19
9.33E-55	0.36224082	0.312	0.14	1.32E-50	Monocytes	Necap2
1.25E-54	0.26100258	0.253	0.102	1.77E-50	Monocytes	Tor1a
1.27E-54	-0.808493	0.144	0.303	1.80E-50	Monocytes	Skap1
2.13E-54	0.27191805	0.125	0.031	3.02E-50	Monocytes	Dse
2.17E-54	0.37434161	0.199	0.071	3.07E-50	Monocytes	Tgfbr1
2.38E-54	0.2761147	0.574	0.32	3.36E-50	Monocytes	S100a4
2.89E-54	-0.4403972	0.797	0.837	4.09E-50	Monocytes	Rpl14
3.48E-54	0.33847301	0.338	0.159	4.92E-50	Monocytes	Stard3nl
5.36E-54	0.32381537	0.167	0.053	7.59E-50	Monocytes	Htra2
6.83E-54	0.32336496	0.145	0.041	9.67E-50	Monocytes	Itgax
7.41E-54	0.44465631	0.459	0.25	1.05E-49	Monocytes	Lamtor4
1.11E-53	0.27617899	0.243	0.097	1.57E-49	Monocytes	Fbxo6
1.25E-53	-0.4282601	0.796	0.831	1.77E-49	Monocytes	Rpl22
1.91E-53	0.45811132	0.39	0.202	2.71E-49	Monocytes	Rab5c
2.48E-53	0.3795113	0.19	0.066	3.50E-49	Monocytes	Gm15987
2.53E-53	0.31273085	0.255	0.105	3.59E-49	Monocytes	Comt
3.07E-53	-0.7083177	0.011	0.129	4.34E-49	Monocytes	Snn
3.50E-53	0.26787631	0.189	0.065	4.95E-49	Monocytes	Sgk3
3.68E-53	0.29950288	0.209	0.078	5.21E-49	Monocytes	Akt1
8.29E-53	0.30497176	0.223	0.085	1.17E-48	Monocytes	Nenf
1.11E-52	-0.554722	0.529	0.649	1.56E-48	Monocytes	Il2rg
2.02E-52	0.5447787	0.503	0.299	2.87E-48	Monocytes	Rps27l
3.18E-52	0.25287864	0.137	0.037	4.50E-48	Monocytes	Acsl1
3.81E-52	0.51486704	0.416	0.225	5.39E-48	Monocytes	Tap1
6.62E-52	0.40130115	0.434	0.233	9.38E-48	Monocytes	Tmbim4
8.36E-52	0.33718721	0.374	0.186	1.18E-47	Monocytes	Hcfc1r1
9.95E-52	-0.706807	0.004	0.114	1.41E-47	Monocytes	Pou2af1
1.09E-51	-0.827061	0.079	0.219	1.55E-47	Monocytes	Acap1
1.24E-51	0.41382388	0.441	0.243	1.76E-47	Monocytes	Pycard
1.70E-51	0.38555395	0.6	0.369	2.41E-47	Monocytes	Plek
2.35E-51	-0.5723947	0.013	0.13	3.33E-47	Monocytes	Cmah
2.48E-51	-0.3302267	0.901	0.914	3.50E-47	Monocytes	Rpl11
2.70E-51	-0.7826904	0.429	0.533	3.83E-47	Monocytes	Jak1
2.82E-51	0.29674696	0.355	0.173	4.00E-47	Monocytes	Acaa1a
3.18E-51	-0.3605654	0.858	0.886	4.50E-47	Monocytes	Rplp2
3.23E-51	0.33001485	0.211	0.08	4.57E-47	Monocytes	Camk1d
3.61E-51	0.45165661	0.463	0.26	5.12E-47	Monocytes	Psma4
3.69E-51	-0.9752114	0.149	0.302	5.22E-47	Monocytes	Itk
3.96E-51	-1.0927253	0.203	0.365	5.60E-47	Monocytes	Ctsw
5.30E-51	0.64894589	0.94	0.924	7.51E-47	Monocytes	Lars2
5.63E-51	-0.948769	0.123	0.273	7.98E-47	Monocytes	Gem
5.71E-51	0.28698534	0.127	0.034	8.08E-47	Monocytes	Ifih1

6.73E-51	0.3348696	0.265	0.114	9.53E-47	Monocytes	Trps1
8.61E-51	-0.9128408	0.063	0.199	1.22E-46	Monocytes	Ralgps2
1.56E-50	0.27725363	0.213	0.082	2.21E-46	Monocytes	Dtx3l
1.86E-50	0.25530018	0.1	0.021	2.63E-46	Monocytes	Dgat2
2.47E-50	-0.8691594	0.412	0.518	3.49E-46	Monocytes	Ezr
2.53E-50	0.26673198	0.988	0.981	3.58E-46	Monocytes	Tmsb4x
3.17E-50	0.54997634	0.719	0.586	4.49E-46	Monocytes	Eno1
5.96E-50	0.52331006	0.76	0.635	8.44E-46	Monocytes	Aldoa
6.32E-50	0.30281337	0.195	0.071	8.95E-46	Monocytes	Ppp1r9b
1.16E-49	0.34373067	0.147	0.045	1.64E-45	Monocytes	Camkk2
1.22E-49	0.26376907	0.214	0.083	1.73E-45	Monocytes	Cd2ap
1.23E-49	0.47009119	0.338	0.169	1.74E-45	Monocytes	Hexb
1.28E-49	-0.8423355	0.142	0.288	1.81E-45	Monocytes	Rabgap1l
1.28E-49	0.52918719	0.559	0.362	1.81E-45	Monocytes	Smdt1
2.18E-49	-0.6896881	0.03	0.152	3.09E-45	Monocytes	Trp53i11
2.34E-49	-0.558779	0.565	0.668	3.31E-45	Monocytes	Limd2
2.68E-49	-0.3278169	0.914	0.944	3.79E-45	Monocytes	Rps9
3.53E-49	-0.4658087	0.711	0.771	5.00E-45	Monocytes	Rpl9
3.63E-49	0.52861051	0.713	0.607	5.14E-45	Monocytes	Nme2
3.72E-49	0.38953789	0.113	0.028	5.26E-45	Monocytes	Tgtp1
3.76E-49	0.27436674	0.222	0.089	5.33E-45	Monocytes	Lipa
5.32E-49	0.3837359	0.251	0.108	7.53E-45	Monocytes	Mfsd1
5.85E-49	0.31273392	0.326	0.156	8.28E-45	Monocytes	Slc25a39
6.02E-49	-0.9506532	0.293	0.45	8.52E-45	Monocytes	Cd3d
8.69E-49	0.25324824	0.176	0.061	1.23E-44	Monocytes	Pepd
1.07E-48	0.38303474	0.272	0.123	1.52E-44	Monocytes	Agpat4
1.10E-48	0.45111638	0.608	0.408	1.56E-44	Monocytes	Txn1
1.24E-48	-0.7408119	0.003	0.106	1.76E-44	Monocytes	Vpreb3
1.49E-48	0.51037946	0.458	0.265	2.11E-44	Monocytes	Gng10
1.55E-48	0.44884212	0.45	0.255	2.19E-44	Monocytes	Atp1a1
2.08E-48	0.32617307	0.298	0.138	2.94E-44	Monocytes	Siva1
2.16E-48	-1.0615596	0.289	0.412	3.06E-44	Monocytes	Foxp1
2.33E-48	0.30892596	0.573	0.348	3.29E-44	Monocytes	Ahnak
2.88E-48	-0.2801704	0.969	0.984	4.08E-44	Monocytes	Fau
2.99E-48	-0.8096895	0.131	0.277	4.24E-44	Monocytes	Prkca
4.47E-48	-0.7422728	0.876	0.894	6.32E-44	Monocytes	Rps29
6.16E-48	0.30482388	0.106	0.025	8.72E-44	Monocytes	Spp1
6.37E-48	-0.9084258	0.107	0.242	9.02E-44	Monocytes	Foxo1
6.41E-48	0.6035341	0.795	0.693	9.08E-44	Monocytes	Crip1
6.80E-48	0.33835303	0.341	0.169	9.63E-44	Monocytes	Ssr3
8.62E-48	-0.8972324	0.172	0.311	1.22E-43	Monocytes	Hmgn1
1.36E-47	0.36185126	0.44	0.239	1.92E-43	Monocytes	Tor1aip1
1.73E-47	0.32032838	0.342	0.169	2.44E-43	Monocytes	Xbp1
1.81E-47	0.25712817	0.302	0.141	2.56E-43	Monocytes	Trappc1
1.86E-47	0.28002194	0.365	0.188	2.64E-43	Monocytes	Vsir
1.87E-47	0.48935943	0.323	0.159	2.64E-43	Monocytes	Pou2f2
2.20E-47	0.43763574	0.52	0.314	3.11E-43	Monocytes	Prdx2
2.61E-47	-0.6906495	0.134	0.278	3.70E-43	Monocytes	Tbc1d10c

5.17E-47	-0.3899212	0.929	0.918	7.31E-43	Monocytes	Rps2
6.77E-47	-0.6553569	0.008	0.112	9.59E-43	Monocytes	Cpm
6.87E-47	0.33192404	0.332	0.162	9.73E-43	Monocytes	Uvrag
7.39E-47	-0.5960604	0.005	0.106	1.05E-42	Monocytes	Pax5
1.30E-46	0.76896945	0.282	0.137	1.84E-42	Monocytes	Id3
1.75E-46	0.50161141	0.131	0.039	2.48E-42	Monocytes	Cd63
1.84E-46	0.31384755	0.182	0.066	2.61E-42	Monocytes	Qk
2.15E-46	0.45267784	0.518	0.313	3.05E-42	Monocytes	Chmp2a
2.49E-46	0.5067791	0.529	0.333	3.52E-42	Monocytes	Esd
3.35E-46	-1.3268091	0.458	0.557	4.75E-42	Monocytes	Dnajb1
5.89E-46	0.38055946	0.355	0.186	8.34E-42	Monocytes	Slamf7
9.70E-46	0.33343753	0.21	0.085	1.37E-41	Monocytes	Slc43a2
1.05E-45	0.37449101	0.399	0.22	1.48E-41	Monocytes	Fermt3
2.13E-45	-0.5067109	0.872	0.862	3.02E-41	Monocytes	Rps19
2.31E-45	-0.9674998	0.175	0.316	3.27E-41	Monocytes	Sell
3.33E-45	0.32599737	0.273	0.126	4.72E-41	Monocytes	Nmt1
4.53E-45	0.26262814	0.105	0.026	6.41E-41	Monocytes	Atp7a
4.73E-45	-0.5329651	0.663	0.708	6.69E-41	Monocytes	Rpl4
5.38E-45	0.31766199	0.188	0.072	7.62E-41	Monocytes	Cept1
5.49E-45	-0.8693831	0.133	0.265	7.78E-41	Monocytes	Ptp4a3
5.70E-45	0.48668154	0.179	0.068	8.07E-41	Monocytes	Dusp16
9.25E-45	0.32932478	0.315	0.155	1.31E-40	Monocytes	Atp6v1b2
1.10E-44	0.39564121	0.326	0.164	1.56E-40	Monocytes	Degs1
1.15E-44	0.27637977	0.236	0.101	1.63E-40	Monocytes	Phyh
1.19E-44	0.33575636	0.182	0.069	1.68E-40	Monocytes	Parp9
1.27E-44	0.54597754	0.287	0.141	1.80E-40	Monocytes	Serpina3g
1.28E-44	0.26127767	0.243	0.106	1.81E-40	Monocytes	Med8
1.87E-44	0.52317044	0.67	0.508	2.65E-40	Monocytes	Tagln2
2.06E-44	0.71198433	0.292	0.149	2.92E-40	Monocytes	Cd72
2.85E-44	0.26431152	0.251	0.113	4.03E-40	Monocytes	Fam89b
2.95E-44	0.39429571	0.62	0.409	4.18E-40	Monocytes	Tln1
3.23E-44	0.25772035	0.257	0.117	4.57E-40	Monocytes	Fbxl5
3.46E-44	0.54763984	0.12	0.035	4.90E-40	Monocytes	Hes1
4.08E-44	0.25258961	0.163	0.058	5.78E-40	Monocytes	Abcd1
5.74E-44	0.25661102	0.166	0.059	8.13E-40	Monocytes	Arf2
5.78E-44	-0.699718	0.02	0.125	8.18E-40	Monocytes	Siglecg
6.40E-44	-0.8376176	0.12	0.253	9.06E-40	Monocytes	Rnf125
7.30E-44	-0.9787658	0.36	0.477	1.03E-39	Monocytes	Hspd1
8.62E-44	0.63266528	0.372	0.212	1.22E-39	Monocytes	Glul
9.77E-44	-0.579702	0.016	0.119	1.38E-39	Monocytes	St6gal1
1.28E-43	0.42162004	0.522	0.328	1.82E-39	Monocytes	Chmp4b
1.50E-43	0.37941091	0.607	0.417	2.13E-39	Monocytes	Mcl1
1.63E-43	0.41898566	0.364	0.195	2.31E-39	Monocytes	Myo1g
1.76E-43	0.52214328	0.422	0.244	2.49E-39	Monocytes	Bcl2a1d
2.95E-43	-0.6954713	0.153	0.291	4.17E-39	Monocytes	Gimap5
3.36E-43	-0.4527566	0.71	0.747	4.76E-39	Monocytes	Rps15
4.14E-43	0.3853385	0.39	0.211	5.85E-39	Monocytes	Mbnl2
4.55E-43	0.34816867	0.328	0.166	6.44E-39	Monocytes	Bnip2

6.11E-43	-0.7697207	0.021	0.125	8.64E-39	Monocytes	Lef1
8.21E-43	0.36699329	0.269	0.128	1.16E-38	Monocytes	Ccr5
1.47E-42	-0.6826414	0.095	0.221	2.08E-38	Monocytes	Grap
1.97E-42	0.58910203	0.479	0.305	2.78E-38	Monocytes	Sdcbp
2.09E-42	-0.8501641	0.234	0.382	2.96E-38	Monocytes	Sh2d2a
2.73E-42	-0.4118555	0.75	0.791	3.86E-38	Monocytes	Rps6
2.93E-42	0.49314007	0.676	0.52	4.15E-38	Monocytes	Emp3
4.01E-42	0.30082227	0.102	0.026	5.68E-38	Monocytes	Tnfsf9
9.40E-42	-0.6939239	0.068	0.189	1.33E-37	Monocytes	Atp2a3
1.17E-41	0.26103298	0.211	0.09	1.65E-37	Monocytes	N4bp1
1.19E-41	0.27237403	0.168	0.063	1.69E-37	Monocytes	Lrrk1
1.34E-41	0.2875042	0.147	0.051	1.90E-37	Monocytes	Pot1b
1.72E-41	-0.8292044	0.221	0.361	2.43E-37	Monocytes	Thy1
1.81E-41	0.26343125	0.135	0.044	2.56E-37	Monocytes	Nod1
1.83E-41	0.26708611	0.183	0.072	2.59E-37	Monocytes	Usp8
1.93E-41	0.32202985	0.437	0.25	2.73E-37	Monocytes	Glrx
2.62E-41	0.27946322	0.273	0.13	3.70E-37	Monocytes	Sppl2a
3.62E-41	0.27700551	0.333	0.169	5.12E-37	Monocytes	Plekho2
4.72E-41	0.36819147	0.387	0.214	6.68E-37	Monocytes	Tbcb
1.05E-40	0.25806573	0.292	0.143	1.49E-36	Monocytes	Ist1
1.45E-40	0.45108238	0.538	0.347	2.05E-36	Monocytes	Ndufb8
1.61E-40	0.2574758	0.323	0.165	2.29E-36	Monocytes	Sdhd
2.03E-40	-0.6892196	0.373	0.472	2.87E-36	Monocytes	Tubb4b
2.60E-40	-0.26178	0.548	0.339	3.67E-36	Monocytes	Ccl4
2.97E-40	-0.6143238	0.065	0.182	4.20E-36	Monocytes	S1pr4
2.98E-40	0.3219589	0.3	0.15	4.21E-36	Monocytes	Cd86
4.24E-40	0.46105815	0.21	0.093	6.00E-36	Monocytes	Gbp4
5.76E-40	0.28557542	0.353	0.187	8.16E-36	Monocytes	Fuca1
9.69E-40	0.25724714	0.256	0.122	1.37E-35	Monocytes	Chd9
9.87E-40	0.36731569	0.311	0.16	1.40E-35	Monocytes	Atp6ap2
1.16E-39	-0.3777529	0.881	0.86	1.65E-35	Monocytes	Rpl35
1.47E-39	-0.3559775	0.844	0.853	2.08E-35	Monocytes	Rpl15
2.18E-39	0.36179733	0.478	0.289	3.09E-35	Monocytes	Atp2b1
2.58E-39	0.28460987	0.187	0.077	3.65E-35	Monocytes	Fcho2
2.73E-39	-0.7960665	0.383	0.487	3.86E-35	Monocytes	Hcst
3.69E-39	-0.7207396	0.343	0.455	5.22E-35	Monocytes	Tuba1a
4.25E-39	0.29389853	0.138	0.048	6.02E-35	Monocytes	Noct
4.85E-39	0.34244563	0.466	0.275	6.86E-35	Monocytes	Ap2s1
5.44E-39	-0.5561912	0.043	0.151	7.70E-35	Monocytes	Gimap8
5.45E-39	0.48889354	0.418	0.25	7.71E-35	Monocytes	M6pr
6.05E-39	-0.328218	0.872	0.879	8.57E-35	Monocytes	Rps14
6.10E-39	0.90845922	0.826	0.681	8.63E-35	Monocytes	AY036118
6.19E-39	0.28817201	0.343	0.181	8.77E-35	Monocytes	Psmd7
7.64E-39	-0.7913919	0.388	0.495	1.08E-34	Monocytes	Dusp2
1.42E-38	-0.7267864	0.106	0.225	2.01E-34	Monocytes	Dgka
2.29E-38	0.36274261	0.378	0.216	3.23E-34	Monocytes	Rtn4
3.23E-38	0.39036602	0.782	0.679	4.57E-34	Monocytes	H2afz
3.36E-38	-0.7726298	0.597	0.665	4.75E-34	Monocytes	Hspe1

3.92E-38	-0.7426064	0.131	0.256	5.55E-34	Monocytes	Cd247
5.53E-38	0.37047309	0.529	0.348	7.83E-34	Monocytes	Atp6v1g1
7.14E-38	0.27952206	0.187	0.078	1.01E-33	Monocytes	Irf2bp2
8.03E-38	0.30420918	0.201	0.087	1.14E-33	Monocytes	Filip1l
9.79E-38	0.34253197	0.475	0.289	1.39E-33	Monocytes	Glud1
1.14E-37	0.3803472	0.503	0.313	1.62E-33	Monocytes	Uqcr11
1.31E-37	0.37265108	0.471	0.289	1.85E-33	Monocytes	Ndufc2
1.33E-37	-0.7594064	0.213	0.333	1.88E-33	Monocytes	Rbm38
1.40E-37	0.30838122	0.468	0.282	1.98E-33	Monocytes	Iscu
1.43E-37	-0.9521699	0.339	0.476	2.03E-33	Monocytes	Cd3g
1.71E-37	-0.3492581	0.854	0.852	2.42E-33	Monocytes	Rps23
1.86E-37	0.51278163	0.508	0.347	2.63E-33	Monocytes	Capg
2.51E-37	-0.6505033	0.07	0.182	3.55E-33	Monocytes	Lamb3
2.93E-37	-0.4799313	0.016	0.105	4.15E-33	Monocytes	Rras2
3.49E-37	0.31884677	0.633	0.473	4.95E-33	Monocytes	Rhog
3.86E-37	0.28723631	0.229	0.106	5.46E-33	Monocytes	Klf10
3.92E-37	-0.5801928	0.499	0.569	5.54E-33	Monocytes	Ube2b
4.79E-37	0.43559937	0.559	0.385	6.78E-33	Monocytes	Psmb9
4.89E-37	0.26976236	0.162	0.063	6.92E-33	Monocytes	Epb41l2
7.47E-37	0.45068205	0.644	0.488	1.06E-32	Monocytes	Rac1
7.76E-37	0.31531682	0.351	0.192	1.10E-32	Monocytes	Taok3
1.58E-36	-0.5124065	0.039	0.14	2.24E-32	Monocytes	Jakmip1
1.65E-36	-0.7005511	0.475	0.573	2.34E-32	Monocytes	Tsc22d3
1.99E-36	0.31665499	0.79	0.696	2.81E-32	Monocytes	Clic1
2.83E-36	0.26223193	0.46	0.28	4.00E-32	Monocytes	Timm10b
3.53E-36	0.34727828	0.722	0.653	5.00E-32	Monocytes	Gabarap
4.25E-36	0.28883545	0.338	0.182	6.01E-32	Monocytes	Trappc2l
4.94E-36	-0.9764868	0.171	0.294	6.99E-32	Monocytes	Il2rb
7.97E-36	-0.7848739	0.154	0.276	1.13E-31	Monocytes	Cd28
8.48E-36	0.29283811	0.214	0.099	1.20E-31	Monocytes	Hip1
9.46E-36	0.25865836	0.142	0.053	1.34E-31	Monocytes	Stx12
1.69E-35	-0.5892919	0.048	0.151	2.40E-31	Monocytes	Traf4
2.05E-35	-0.318341	0.876	0.885	2.90E-31	Monocytes	Rpl28
2.39E-35	-0.9347172	0.278	0.392	3.38E-31	Monocytes	Hsph1
2.60E-35	-0.656732	0.101	0.214	3.68E-31	Monocytes	Gimap9
3.59E-35	0.41340662	0.591	0.407	5.09E-31	Monocytes	Jun
4.62E-35	-0.583969	0.513	0.597	6.53E-31	Monocytes	Ypel3
4.97E-35	0.29952963	0.173	0.073	7.04E-31	Monocytes	Man2a1
5.68E-35	-0.8044858	0.164	0.278	8.04E-31	Monocytes	Tcp11l2
5.86E-35	0.62728288	0.481	0.329	8.29E-31	Monocytes	Rgs1
6.75E-35	-0.6565182	0.039	0.137	9.56E-31	Monocytes	Txk
7.60E-35	0.27254555	0.164	0.067	1.08E-30	Monocytes	Gbp8
9.45E-35	0.35636163	0.367	0.21	1.34E-30	Monocytes	Pdia6
1.03E-34	-0.7133629	0.056	0.158	1.46E-30	Monocytes	Blnk
2.14E-34	0.30570017	0.188	0.084	3.02E-30	Monocytes	Fdps
2.38E-34	0.40608921	0.694	0.593	3.37E-30	Monocytes	Clta
3.64E-34	0.43873254	0.467	0.301	5.16E-30	Monocytes	Tapbp
3.76E-34	0.35542197	0.485	0.307	5.32E-30	Monocytes	Ms4a6b

6.02E-34	-0.5256392	0.482	0.553	8.52E-30	Monocytes	Cnn2
6.04E-34	0.31731268	0.489	0.308	8.56E-30	Monocytes	Rbms1
6.23E-34	0.31660359	0.182	0.079	8.81E-30	Monocytes	Ripk2
7.53E-34	0.35842152	0.433	0.26	1.07E-29	Monocytes	Psma6
7.75E-34	0.25360808	0.262	0.133	1.10E-29	Monocytes	Psen2
7.78E-34	0.25934286	0.385	0.219	1.10E-29	Monocytes	Ywhag
1.61E-33	-0.4492183	0.673	0.765	2.28E-29	Monocytes	Ier2
1.73E-33	-0.9997759	0.621	0.646	2.44E-29	Monocytes	Klf2
1.81E-33	0.34041645	0.54	0.355	2.57E-29	Monocytes	Capns1
1.87E-33	-0.7241122	0.04	0.135	2.64E-29	Monocytes	Serpinb1a
3.27E-33	0.2517967	0.129	0.047	4.63E-29	Monocytes	Bnip3
3.47E-33	0.32998434	0.358	0.207	4.92E-29	Monocytes	Arrb2
4.56E-33	0.35394731	0.661	0.53	6.46E-29	Monocytes	Gnai2
1.54E-32	-0.717129	0.129	0.243	2.18E-28	Monocytes	Gpr171
3.02E-32	-0.3983251	0.766	0.809	4.28E-28	Monocytes	Sub1
4.30E-32	0.32676285	0.796	0.764	6.09E-28	Monocytes	Arpc2
4.35E-32	0.25366758	0.181	0.081	6.15E-28	Monocytes	Ppp1r12c
4.77E-32	0.37418313	0.426	0.264	6.75E-28	Monocytes	Pgk1
8.29E-32	0.33489625	0.416	0.251	1.17E-27	Monocytes	Ndufc1
1.20E-31	0.41852428	0.563	0.396	1.70E-27	Monocytes	Mrpl52
1.74E-31	0.44576832	0.515	0.355	2.47E-27	Monocytes	Vdac2
1.75E-31	0.25507424	0.857	0.841	2.47E-27	Monocytes	Sh3bgrl3
1.77E-31	0.37799373	0.712	0.595	2.51E-27	Monocytes	Capzb
2.51E-31	-0.5661996	0.425	0.489	3.56E-27	Monocytes	Prkar1a
2.87E-31	-0.675302	0.409	0.483	4.06E-27	Monocytes	Kmt2e
3.48E-31	-0.6642413	0.405	0.475	4.92E-27	Monocytes	Arhgef1
3.98E-31	0.2622441	0.184	0.084	5.64E-27	Monocytes	Dr1
4.40E-31	0.25598351	0.203	0.096	6.23E-27	Monocytes	Arap1
5.24E-31	0.25855638	0.227	0.114	7.42E-27	Monocytes	Gbp7
5.72E-31	-0.6668371	0.072	0.173	8.10E-27	Monocytes	H2-Oa
7.62E-31	-0.669418	0.026	0.109	1.08E-26	Monocytes	Tcf7
7.98E-31	0.40882058	0.521	0.346	1.13E-26	Monocytes	Psmb6
8.96E-31	-0.6145117	0.111	0.219	1.27E-26	Monocytes	Stat4
1.54E-30	-0.6827421	0.384	0.475	2.19E-26	Monocytes	Dusp5
1.84E-30	-0.7665921	0.208	0.096	2.61E-26	Monocytes	Slpi
5.52E-30	-0.2885747	0.846	0.87	7.81E-26	Monocytes	Rpl29
6.38E-30	-0.7168911	0.281	0.378	9.03E-26	Monocytes	Crem
2.98E-29	-0.8105587	0.305	0.383	4.22E-25	Monocytes	Saraf
3.44E-29	0.2593117	0.376	0.224	4.88E-25	Monocytes	Uqcc2
3.56E-29	0.31784634	0.417	0.255	5.04E-25	Monocytes	Txndc17
6.23E-29	0.25383227	0.354	0.205	8.81E-25	Monocytes	AI413582
6.38E-29	-0.4501262	0.648	0.685	9.03E-25	Monocytes	Hmgb1
7.07E-29	0.52829664	0.522	0.354	1.00E-24	Monocytes	Neat1
7.17E-29	-0.3636123	0.736	0.771	1.01E-24	Monocytes	Eef1b2
8.09E-29	0.37867824	0.526	0.36	1.14E-24	Monocytes	Sdhb
9.43E-29	0.31639461	0.49	0.315	1.34E-24	Monocytes	Brk1
1.14E-28	0.34097105	0.54	0.372	1.62E-24	Monocytes	Tuba1c
1.26E-28	0.26448531	0.205	0.101	1.78E-24	Monocytes	Arhgef2

1.55E-28	-0.5643769	0.046	0.133	2.19E-24	Monocytes	Irf4
1.88E-28	-0.6969329	0.253	0.347	2.66E-24	Monocytes	Rapgef6
1.91E-28	0.25257085	0.386	0.227	2.70E-24	Monocytes	Ndufb3
2.28E-28	0.32052844	0.414	0.255	3.23E-24	Monocytes	Psma5
3.64E-28	0.40329637	0.479	0.317	5.15E-24	Monocytes	Atpif1
3.74E-28	0.29065606	0.237	0.124	5.30E-24	Monocytes	Cflar
3.81E-28	-0.5964728	0.177	0.275	5.40E-24	Monocytes	Cd164
4.32E-28	0.27454025	0.385	0.234	6.12E-24	Monocytes	Cmpk1
4.36E-28	0.31815268	0.478	0.313	6.17E-24	Monocytes	H2afy
4.65E-28	0.3555311	0.575	0.415	6.58E-24	Monocytes	Ssr4
5.76E-28	-0.6340271	0.176	0.276	8.15E-24	Monocytes	Lbr
6.84E-28	-0.4810499	0.037	0.118	9.68E-24	Monocytes	Tmem163
6.84E-28	-0.4296024	0.149	0.062	9.69E-24	Monocytes	Hcar2
1.34E-27	0.31813063	0.476	0.313	1.90E-23	Monocytes	Bloc1s1
1.41E-27	-0.4193481	0.047	0.131	1.99E-23	Monocytes	Ppp3cc
1.79E-27	0.31708007	0.289	0.165	2.53E-23	Monocytes	Pmaip1
2.01E-27	-0.9263326	0.081	0.172	2.85E-23	Monocytes	Dusp10
2.88E-27	-0.5927242	0.104	0.199	4.07E-23	Monocytes	Pde7a
2.90E-27	-0.5422282	0.044	0.128	4.11E-23	Monocytes	Plcxd2
3.11E-27	0.35058781	0.678	0.539	4.40E-23	Monocytes	Cox5a
3.44E-27	-0.487765	0.173	0.077	4.87E-23	Monocytes	Clec4d
4.07E-27	0.33932315	0.599	0.443	5.76E-23	Monocytes	Scand1
6.86E-27	-0.7337453	0.153	0.247	9.71E-23	Monocytes	Prkcb
6.88E-27	0.43349506	0.276	0.158	9.74E-23	Monocytes	Cks2
7.24E-27	-0.6076942	0.179	0.081	1.02E-22	Monocytes	Csf3r
7.35E-27	0.38356854	0.516	0.361	1.04E-22	Monocytes	Fam49b
7.41E-27	-1.0345358	0.417	0.481	1.05E-22	Monocytes	Nkg7
8.74E-27	-0.6609371	0.195	0.286	1.24E-22	Monocytes	Tgfbr2
9.73E-27	0.2893691	0.527	0.358	1.38E-22	Monocytes	Cmtm7
1.08E-26	0.27533592	0.43	0.272	1.53E-22	Monocytes	Atp6v1e1
1.33E-26	-0.4931647	0.062	0.148	1.89E-22	Monocytes	Sesn3
2.26E-26	-0.4263882	0.623	0.671	3.21E-22	Monocytes	Calm2
2.78E-26	0.30059115	0.421	0.273	3.94E-22	Monocytes	P4hb
3.17E-26	0.36383112	0.677	0.574	4.49E-22	Monocytes	Cox6b1
4.50E-26	-0.5461558	0.143	0.238	6.37E-22	Monocytes	6-Sep
6.19E-26	-0.5669057	0.845	0.888	8.76E-22	Monocytes	Junb
8.29E-26	0.25381734	0.556	0.393	1.17E-21	Monocytes	Tuba1b
9.56E-26	0.33192891	0.355	0.221	1.35E-21	Monocytes	Gltp
9.94E-26	-0.6743851	0.167	0.26	1.41E-21	Monocytes	Rhoh
1.73E-25	0.25662085	0.424	0.27	2.45E-21	Monocytes	H13
2.01E-25	0.25613051	0.502	0.34	2.85E-21	Monocytes	Bax
2.77E-25	-0.3342865	0.946	0.923	3.92E-21	Monocytes	Rplp0
3.34E-25	0.29640436	0.312	0.183	4.73E-21	Monocytes	Med21
4.37E-25	-0.4468666	0.036	0.112	6.18E-21	Monocytes	Mirt1
4.90E-25	0.37263976	0.592	0.454	6.94E-21	Monocytes	Uqcrb
5.93E-25	-0.475963	0.645	0.712	8.40E-21	Monocytes	Dnaja1
9.32E-25	-0.552545	0.055	0.136	1.32E-20	Monocytes	Fchsd2
1.46E-24	-0.3812835	0.884	0.899	2.07E-20	Monocytes	Hsp90ab1

1.68E-24	-0.540345	0.094	0.184	2.38E-20	Monocytes	Map4k2
1.87E-24	0.32267762	0.596	0.443	2.64E-20	Monocytes	Uqcrq
2.54E-24	0.31927116	0.515	0.353	3.60E-20	Monocytes	Cope
2.63E-24	0.30785436	0.732	0.688	3.73E-20	Monocytes	Gng5
3.25E-24	0.27725288	0.543	0.382	4.61E-20	Monocytes	Tmed10
4.74E-24	-0.4320591	0.048	0.125	6.71E-20	Monocytes	Prkd2
4.82E-24	-0.469171	0.548	0.597	6.82E-20	Monocytes	Map1lc3b
8.13E-24	0.25467037	0.528	0.361	1.15E-19	Monocytes	Ccdc12
9.69E-24	-0.6933489	0.199	0.286	1.37E-19	Monocytes	Smad7
9.95E-24	-0.5411739	0.476	0.524	1.41E-19	Monocytes	Ndfip1
1.21E-23	-0.5003984	0.526	0.585	1.71E-19	Monocytes	Cytip
1.25E-23	0.25289664	0.385	0.239	1.77E-19	Monocytes	Ndufs3
1.30E-23	-0.7390068	0.221	0.308	1.84E-19	Monocytes	Il21r
1.45E-23	-0.5913219	0.139	0.229	2.05E-19	Monocytes	Nfatc3
1.76E-23	-0.5464594	0.516	0.577	2.50E-19	Monocytes	Zfp36l1
1.82E-23	0.32266858	0.53	0.375	2.58E-19	Monocytes	Psmb5
2.43E-23	-0.3912711	0.676	0.713	3.44E-19	Monocytes	Pabpc1
3.51E-23	-0.4522725	0.031	0.1	4.98E-19	Monocytes	Btla
4.42E-23	0.3481799	0.606	0.478	6.25E-19	Monocytes	Cycs
4.62E-23	-0.5085889	0.469	0.509	6.55E-19	Monocytes	Eif4g2
6.17E-23	0.26205758	0.322	0.198	8.74E-19	Monocytes	Spop
8.09E-23	-0.5644064	0.307	0.375	1.15E-18	Monocytes	Cirbp
3.43E-22	-0.4009246	0.546	0.598	4.86E-18	Monocytes	Pcbp2
3.63E-22	0.38043795	0.361	0.236	5.14E-18	Monocytes	Itgal
4.19E-22	0.36110203	0.238	0.135	5.92E-18	Monocytes	Chka
4.23E-22	0.31514297	0.664	0.551	5.99E-18	Monocytes	Slc25a5
4.34E-22	0.2570622	0.371	0.234	6.14E-18	Monocytes	Cyb5a
4.44E-22	0.33560166	0.714	0.641	6.29E-18	Monocytes	Atp5l
4.63E-22	-0.4550958	0.076	0.155	6.55E-18	Monocytes	Fam102a
4.79E-22	-0.3510737	0.034	0.101	6.78E-18	Monocytes	Cdc25b
5.42E-22	0.31724158	0.63	0.51	7.67E-18	Monocytes	Gnb2
6.76E-22	-0.3862147	0.043	0.115	9.57E-18	Monocytes	Lax1
7.36E-22	-0.4425881	0.051	0.123	1.04E-17	Monocytes	Prkcq
7.64E-22	-0.4482703	0.142	0.228	1.08E-17	Monocytes	Dnajc9
9.96E-22	-0.6918197	0.229	0.303	1.41E-17	Monocytes	Crlf3
1.00E-21	-0.4891822	0.167	0.26	1.42E-17	Monocytes	Gimap7
1.68E-21	0.32111481	0.51	0.363	2.38E-17	Monocytes	Ifrd1
1.78E-21	-0.5126039	0.346	0.412	2.51E-17	Monocytes	H2afv
3.39E-21	0.31497678	0.636	0.515	4.79E-17	Monocytes	Arpc4
4.62E-21	-0.4815575	0.037	0.102	6.54E-17	Monocytes	Tmem64
1.28E-20	-0.5972304	0.177	0.254	1.82E-16	Monocytes	Rnf167
1.51E-20	-0.4512052	0.063	0.135	2.14E-16	Monocytes	Card11
1.54E-20	-0.6867528	0.193	0.279	2.18E-16	Monocytes	Tnfrsf18
1.56E-20	-0.7743489	0.132	0.208	2.21E-16	Monocytes	Gramd3
2.11E-20	0.28123578	0.778	0.754	2.98E-16	Monocytes	Cox4i1
3.45E-20	-0.6540548	0.247	0.32	4.89E-16	Monocytes	4930523C07F
3.68E-20	0.28086662	0.905	0.909	5.21E-16	Monocytes	mt-Atp8
3.79E-20	0.31561135	0.561	0.419	5.36E-16	Monocytes	Sh3glb1

4.65E-20	0.28219995	0.533	0.392	6.58E-16	Monocytes	Psmb2
5.06E-20	-0.3077199	0.635	0.665	7.17E-16	Monocytes	Rpl23a
5.59E-20	-0.5692075	0.168	0.256	7.91E-16	Monocytes	Cd27
6.60E-20	-0.5923324	0.534	0.581	9.34E-16	Monocytes	H2-Q7
7.01E-20	-0.4960069	0.117	0.194	9.92E-16	Monocytes	Rasal3
7.58E-20	-0.5554364	0.054	0.123	1.07E-15	Monocytes	B3gnt5
8.25E-20	-0.953268	0.292	0.364	1.17E-15	Monocytes	Cd8b1
9.75E-20	-0.6944109	0.211	0.285	1.38E-15	Monocytes	Ptpn22
1.01E-19	-0.3936377	0.458	0.499	1.43E-15	Monocytes	Eef1g
1.15E-19	-0.5734013	0.326	0.378	1.63E-15	Monocytes	Ppp1cc
1.20E-19	0.25991057	0.492	0.349	1.69E-15	Monocytes	Calr
1.66E-19	-0.3740112	0.057	0.128	2.34E-15	Monocytes	Hip1r
2.09E-19	-0.5348682	0.193	0.275	2.95E-15	Monocytes	Zgpat
3.00E-19	-0.53156	0.352	0.406	4.25E-15	Monocytes	Hmgn2
6.03E-19	-0.5976727	0.405	0.46	8.53E-15	Monocytes	Tnfaip3
7.32E-19	-0.5803918	0.221	0.292	1.04E-14	Monocytes	Tmem123
7.69E-19	-0.3523115	0.663	0.681	1.09E-14	Monocytes	Npm1
8.02E-19	0.27031051	0.457	0.314	1.14E-14	Monocytes	Ywhae
1.11E-18	-0.6205404	0.553	0.599	1.57E-14	Monocytes	Nr4a1
1.16E-18	0.48136651	0.559	0.461	1.65E-14	Monocytes	Mif
1.20E-18	-0.5506431	0.07	0.139	1.71E-14	Monocytes	Eif2ak3
1.22E-18	-0.5539293	0.146	0.218	1.73E-14	Monocytes	Cnp
1.31E-18	-0.587855	0.132	0.206	1.86E-14	Monocytes	Dgkd
1.33E-18	-0.4386414	0.085	0.157	1.88E-14	Monocytes	Rhof
2.41E-18	-0.5232019	0.115	0.189	3.41E-14	Monocytes	Zap70
2.56E-18	-0.4628583	0.081	0.153	3.63E-14	Monocytes	Arap2
3.47E-18	-0.3808587	0.575	0.603	4.91E-14	Monocytes	Gnas
3.50E-18	1.16045564	0.484	0.346	4.95E-14	Monocytes	Gm26917
5.12E-18	-0.7051845	0.207	0.29	7.25E-14	Monocytes	Icos
6.53E-18	-0.6960419	0.058	0.124	9.24E-14	Monocytes	Tsc22d1
6.96E-18	0.27884924	0.702	0.613	9.85E-14	Monocytes	Cox5b
7.09E-18	-0.5818491	0.2	0.269	1.00E-13	Monocytes	Plekha2
7.60E-18	-0.5265752	0.132	0.204	1.08E-13	Monocytes	Add1
7.75E-18	-0.4165481	0.057	0.122	1.10E-13	Monocytes	Pim2
8.02E-18	0.31134193	0.515	0.384	1.14E-13	Monocytes	Atp5g1
1.03E-17	-0.6401408	0.153	0.227	1.45E-13	Monocytes	Bcl2
1.11E-17	-1.1956093	0.222	0.129	1.57E-13	Monocytes	S100a8
1.17E-17	-0.4419007	0.041	0.1	1.66E-13	Monocytes	Zfp318
1.26E-17	-0.5873199	0.044	0.105	1.79E-13	Monocytes	Myc
2.03E-17	-0.5616214	0.046	0.107	2.87E-13	Monocytes	Sfn
2.35E-17	-0.4488157	0.078	0.146	3.32E-13	Monocytes	Gm19585
2.97E-17	-0.4783855	0.336	0.383	4.21E-13	Monocytes	Paip2
3.28E-17	-0.389944	0.293	0.357	4.64E-13	Monocytes	mt-Nd3
3.75E-17	-0.258559	0.061	0.126	5.31E-13	Monocytes	Phgdh
4.50E-17	-0.4455368	0.662	0.682	6.37E-13	Monocytes	Ptprc
5.83E-17	-0.4315932	0.483	0.518	8.25E-13	Monocytes	Mbnl1
6.11E-17	-0.8584262	0.121	0.194	8.65E-13	Monocytes	Prf1
6.47E-17	-0.2800619	0.648	0.678	9.16E-13	Monocytes	Ywhaz

6.85E-17	-0.5176429	0.186	0.26	9.70E-13	Monocytes	Add3
7.22E-17	-0.6187589	0.098	0.167	1.02E-12	Monocytes	Cd5
1.34E-16	-0.6787511	0.123	0.19	1.89E-12	Monocytes	Gpr183
1.36E-16	-0.3102355	0.534	0.573	1.93E-12	Monocytes	Eef1d
1.94E-16	-0.4119144	0.071	0.136	2.75E-12	Monocytes	Ppp1r16b
2.72E-16	-0.4318055	0.076	0.139	3.84E-12	Monocytes	Zdhhc18
2.88E-16	-0.2778267	0.614	0.639	4.08E-12	Monocytes	Rpl31
2.90E-16	-0.9169483	0.159	0.231	4.10E-12	Monocytes	Hist1h2ap
5.43E-16	-0.3723691	0.534	0.55	7.69E-12	Monocytes	Serbp1
5.64E-16	-0.4675397	0.443	0.472	7.98E-12	Monocytes	Sec62
7.63E-16	0.26828704	0.653	0.53	1.08E-11	Monocytes	Hint1
9.52E-16	-0.8130169	0.261	0.33	1.35E-11	Monocytes	Cd8a
9.71E-16	-0.4181489	0.047	0.103	1.37E-11	Monocytes	Pde4d
1.04E-15	-0.5010814	0.219	0.281	1.48E-11	Monocytes	Vgll4
1.29E-15	-0.4018347	0.35	0.237	1.83E-11	Monocytes	Ccl3
1.74E-15	-0.6326651	0.226	0.301	2.46E-11	Monocytes	Cst7
1.83E-15	-0.4453847	0.115	0.182	2.59E-11	Monocytes	Kif21b
1.92E-15	-0.460938	0.105	0.171	2.72E-11	Monocytes	Inpp4b
1.99E-15	-0.8456612	0.225	0.292	2.82E-11	Monocytes	Pdcd1
2.01E-15	-0.7526678	0.075	0.137	2.84E-11	Monocytes	Il7r
2.07E-15	-0.5907452	0.084	0.146	2.92E-11	Monocytes	Hvcn1
2.35E-15	-0.4108184	0.094	0.158	3.33E-11	Monocytes	Rasgrp1
2.96E-15	-0.4197308	0.705	0.734	4.19E-11	Monocytes	Hsp90aa1
3.04E-15	-0.4786582	0.207	0.269	4.31E-11	Monocytes	Matr3
3.15E-15	0.27142266	0.206	0.128	4.46E-11	Monocytes	Ddit4
3.25E-15	-0.3289281	0.047	0.102	4.60E-11	Monocytes	Gpr174
3.28E-15	-0.7117689	0.224	0.293	4.65E-11	Monocytes	Tigit
3.71E-15	-0.9342886	0.193	0.249	5.25E-11	Monocytes	Mef2c
4.97E-15	0.32611713	0.536	0.404	7.03E-11	Monocytes	Rap1a
6.46E-15	-0.3910539	0.504	0.535	9.15E-11	Monocytes	Hnrnpf
6.94E-15	0.28456887	0.49	0.36	9.82E-11	Monocytes	Ndufv3
9.91E-15	-0.5513674	0.167	0.226	1.40E-10	Monocytes	Pdcd4
1.13E-14	-0.4067282	0.486	0.517	1.60E-10	Monocytes	Akap13
1.53E-14	-0.3824994	0.06	0.117	2.17E-10	Monocytes	Sytl3
1.56E-14	-0.4898518	0.279	0.332	2.21E-10	Monocytes	Eif4a2
2.93E-14	-0.375187	0.094	0.156	4.14E-10	Monocytes	Sh2d1a
3.16E-14	-0.2655876	0.559	0.588	4.48E-10	Monocytes	Snrpg
4.16E-14	-0.4129175	0.064	0.12	5.89E-10	Monocytes	Frat2
7.15E-14	-0.4615889	0.154	0.214	1.01E-09	Monocytes	Grk6
8.00E-14	0.30626986	0.653	0.593	1.13E-09	Monocytes	Sec61g
8.56E-14	-0.4581517	0.093	0.152	1.21E-09	Monocytes	Tecpr1
1.10E-13	-0.5442723	0.091	0.151	1.56E-09	Monocytes	Fasl
1.15E-13	-0.4514376	0.248	0.305	1.62E-09	Monocytes	Acp5
1.22E-13	-0.4819016	0.588	0.609	1.72E-09	Monocytes	Arl6ip1
1.68E-13	-0.3911944	0.08	0.137	2.38E-09	Monocytes	Sla2
1.90E-13	-0.476936	0.149	0.211	2.69E-09	Monocytes	Cenpa
1.93E-13	-0.4730804	0.142	0.203	2.73E-09	Monocytes	Chd2
1.95E-13	-0.5186216	0.377	0.418	2.76E-09	Monocytes	H2-Q6

2.74E-13	-0.4766249	0.111	0.168	3.88E-09	Monocytes	Tcf3
3.06E-13	-0.3613132	0.052	0.103	4.33E-09	Monocytes	Ephx1
3.55E-13	-0.5740802	0.061	0.114	5.03E-09	Monocytes	Klre1
3.91E-13	-0.4362642	0.115	0.174	5.53E-09	Monocytes	Man1a
4.41E-13	0.25990469	0.705	0.64	6.24E-09	Monocytes	Ybx1
4.50E-13	-0.3694021	0.487	0.503	6.37E-09	Monocytes	Tra2b
4.77E-13	0.25510074	0.564	0.447	6.75E-09	Monocytes	Cox7b
4.89E-13	-0.3643541	0.504	0.52	6.92E-09	Monocytes	Ncl
5.49E-13	-1.2443972	0.194	0.255	7.77E-09	Monocytes	Gzmb
5.76E-13	-0.3684364	0.06	0.113	8.15E-09	Monocytes	Trim59
1.14E-12	-0.4838925	0.227	0.285	1.61E-08	Monocytes	Rasgrp2
1.20E-12	-0.5249549	0.071	0.124	1.70E-08	Monocytes	Fam43a
1.32E-12	-0.4537314	0.185	0.241	1.86E-08	Monocytes	Slc1a5
1.44E-12	-0.5273796	0.333	0.368	2.04E-08	Monocytes	Peli1
1.53E-12	-0.5208533	0.203	0.256	2.16E-08	Monocytes	Smc4
1.64E-12	0.25995486	0.568	0.449	2.32E-08	Monocytes	Rbx1
1.80E-12	-0.4199918	0.875	0.873	2.55E-08	Monocytes	Hspa8
2.24E-12	-0.4603279	0.105	0.16	3.17E-08	Monocytes	Sh3bp5
2.43E-12	-0.3804192	0.086	0.14	3.44E-08	Monocytes	Klk8
3.90E-12	-0.3955817	0.067	0.118	5.52E-08	Monocytes	Bcl11b
4.91E-12	-0.3612832	0.07	0.121	6.96E-08	Monocytes	Cdt1
5.26E-12	-0.3783555	0.105	0.158	7.45E-08	Monocytes	Arl2bp
6.02E-12	-0.3644699	0.27	0.317	8.52E-08	Monocytes	Fkbp3
6.73E-12	-0.4783667	0.108	0.16	9.53E-08	Monocytes	Rnf145
7.72E-12	-0.4080093	0.393	0.422	1.09E-07	Monocytes	Clk1
7.76E-12	-0.4265437	0.383	0.409	1.10E-07	Monocytes	Sp100
1.03E-11	-0.5518443	0.206	0.254	1.46E-07	Monocytes	H2afx
1.04E-11	-0.3726215	0.493	0.499	1.48E-07	Monocytes	Ppp1r18
1.22E-11	-0.418469	0.195	0.248	1.72E-07	Monocytes	Psip1
1.28E-11	-1.0828359	0.169	0.101	1.81E-07	Monocytes	Il1r2
1.44E-11	-0.2945468	0.744	0.615	2.04E-07	Monocytes	S100a11
1.59E-11	-0.5075084	0.132	0.185	2.26E-07	Monocytes	Srpk2
1.77E-11	-0.3267379	0.187	0.116	2.51E-07	Monocytes	Adam8
1.90E-11	-0.6613391	0.471	0.47	2.70E-07	Monocytes	Serp1
2.06E-11	-0.4215697	0.25	0.3	2.91E-07	Monocytes	Tes
2.44E-11	-0.4082538	0.091	0.142	3.45E-07	Monocytes	Cers4
3.38E-11	-0.4070201	0.416	0.427	4.78E-07	Monocytes	Ube2s
4.63E-11	-0.4920286	0.091	0.141	6.56E-07	Monocytes	Cd6
6.49E-11	-0.7539132	0.268	0.32	9.19E-07	Monocytes	Klrd1
7.14E-11	-0.495539	0.187	0.232	1.01E-06	Monocytes	Zdhhc20
1.14E-10	-0.5380041	0.352	0.379	1.61E-06	Monocytes	Dennd4a
1.29E-10	-0.4768334	0.188	0.239	1.83E-06	Monocytes	Dut
1.32E-10	-0.4060698	0.251	0.305	1.87E-06	Monocytes	Ccnd2
1.41E-10	-0.4845483	0.201	0.244	1.99E-06	Monocytes	Fli1
1.65E-10	-0.5218219	0.114	0.162	2.33E-06	Monocytes	Pxk
2.77E-10	-0.4104796	0.342	0.372	3.92E-06	Monocytes	Stk24
2.78E-10	-1.0211232	0.138	0.082	3.94E-06	Monocytes	G0s2
2.95E-10	-0.3517173	0.085	0.134	4.18E-06	Monocytes	Nup210

3.05E-10	-0.4085446	0.261	0.302	4.31E-06	Monocytes	Slbp
3.13E-10	-0.3992169	0.075	0.12	4.43E-06	Monocytes	Slamf6
3.31E-10	-0.401442	0.118	0.167	4.68E-06	Monocytes	Pitpnc1
4.10E-10	-0.3965768	0.303	0.336	5.81E-06	Monocytes	Ppdpf
4.29E-10	-0.4663837	0.147	0.193	6.07E-06	Monocytes	Isca1
4.37E-10	-0.4284588	0.425	0.436	6.19E-06	Monocytes	Arf6
4.73E-10	-0.5239602	0.118	0.165	6.69E-06	Monocytes	Dmxl1
5.89E-10	-0.4170587	0.143	0.197	8.33E-06	Monocytes	Tox
5.97E-10	-0.4139256	0.101	0.148	8.45E-06	Monocytes	Sesn1
6.51E-10	-0.4985183	0.079	0.125	9.21E-06	Monocytes	Serpinb6b
7.93E-10	-0.4370817	0.219	0.265	1.12E-05	Monocytes	Nedd9
8.12E-10	-0.4409855	0.146	0.193	1.15E-05	Monocytes	Btg3
1.05E-09	-0.4479525	0.082	0.125	1.49E-05	Monocytes	Cfap43
1.21E-09	-0.6280419	0.163	0.216	1.72E-05	Monocytes	Ctla2a
1.43E-09	-0.3992805	0.303	0.332	2.03E-05	Monocytes	Mettl23
1.44E-09	-0.3260956	0.456	0.462	2.04E-05	Monocytes	Gabarapl2
1.57E-09	-0.4441489	0.185	0.228	2.22E-05	Monocytes	Fkbp4
1.65E-09	-0.4732708	0.146	0.192	2.33E-05	Monocytes	Stt3b
1.67E-09	-0.4144342	0.08	0.124	2.36E-05	Monocytes	Actn1
1.71E-09	-0.4949113	0.226	0.265	2.42E-05	Monocytes	Aff4
1.75E-09	-0.3864552	0.358	0.381	2.47E-05	Monocytes	Sf1
2.20E-09	-0.4263852	0.357	0.374	3.12E-05	Monocytes	Dnajb6
2.74E-09	-0.3059786	0.669	0.472	3.88E-05	Monocytes	Lgals1
2.86E-09	-0.4342206	0.405	0.424	4.05E-05	Monocytes	Slc38a2
3.22E-09	-0.3305761	0.112	0.158	4.56E-05	Monocytes	Spint2
3.23E-09	-0.4342894	0.249	0.282	4.58E-05	Monocytes	Mif4gd
3.56E-09	-0.4315357	0.145	0.189	5.04E-05	Monocytes	Sun2
3.91E-09	-0.3053292	0.109	0.155	5.53E-05	Monocytes	Mllt3
4.43E-09	-0.5300223	0.14	0.182	6.27E-05	Monocytes	Cerk
4.44E-09	-1.2181595	0.213	0.148	6.29E-05	Monocytes	S100a9
4.66E-09	-0.3978457	0.079	0.122	6.60E-05	Monocytes	Tbx21
4.73E-09	-0.4021215	0.518	0.53	6.70E-05	Monocytes	Ppp1r15a
5.62E-09	-0.3783709	0.073	0.114	7.95E-05	Monocytes	Tfam
6.91E-09	-0.3570797	0.135	0.179	9.78E-05	Monocytes	Fam3c
8.25E-09	-0.4587182	0.169	0.209	0.00011675	Monocytes	Abhd17b
1.01E-08	-0.4286902	0.165	0.208	0.00014272	Monocytes	Sptbn1
1.02E-08	-0.320001	0.109	0.154	0.00014455	Monocytes	Cdk17
1.10E-08	-0.536565	0.219	0.26	0.00015585	Monocytes	Prkch
1.23E-08	-0.7733972	0.202	0.249	0.00017352	Monocytes	Ctla4
1.41E-08	-0.3941665	0.084	0.127	0.00019949	Monocytes	Hist1h1e
1.72E-08	-0.3188363	0.078	0.119	0.00024292	Monocytes	Tmem71
1.72E-08	-0.3640446	0.389	0.398	0.00024299	Monocytes	Bzw1
1.87E-08	-0.4729776	0.252	0.285	0.00026417	Monocytes	Isy1
2.11E-08	-0.4323137	0.305	0.327	0.0002981	Monocytes	Pole4
2.33E-08	-0.4230021	0.106	0.147	0.00033032	Monocytes	Avl9
2.91E-08	-0.3206669	0.419	0.426	0.00041261	Monocytes	Ube2d2a
4.25E-08	-0.3387606	0.42	0.422	0.00060142	Monocytes	Hnrnpl
4.28E-08	-0.3298614	0.47	0.471	0.00060537	Monocytes	Srsf7

4.74E-08	-0.3397172	0.33	0.349	0.00067151	Monocytes	Polr2a
5.35E-08	-0.3897327	0.109	0.147	0.00075676	Monocytes	Clp1
5.58E-08	-0.4143837	0.384	0.39	0.00078956	Monocytes	Luc7l2
5.83E-08	0.33786295	0.317	0.242	0.0008247	Monocytes	Socs1
6.21E-08	-0.3207159	0.097	0.137	0.00087954	Monocytes	Mgst2
7.09E-08	-0.4178337	0.094	0.134	0.00100391	Monocytes	Hist1h1c
7.15E-08	-0.3143311	0.45	0.449	0.00101255	Monocytes	Hnrnpab
7.27E-08	-0.3200558	0.067	0.105	0.00102872	Monocytes	Ifitm10
7.77E-08	-0.3950841	0.292	0.332	0.00110021	Monocytes	Mrps6
9.49E-08	-0.4982074	0.294	0.311	0.00134401	Monocytes	Smim14
1.12E-07	-0.4716329	0.111	0.148	0.00158742	Monocytes	Chchd10
1.18E-07	-0.2965624	0.085	0.123	0.00167318	Monocytes	Fam189b
1.26E-07	-0.3710024	0.315	0.343	0.00178762	Monocytes	Macf1
1.87E-07	-0.2571676	0.423	0.428	0.00265262	Monocytes	Hnrnpa1
1.94E-07	-0.2578362	0.564	0.557	0.00274824	Monocytes	Srrm2
2.16E-07	-0.4398806	0.22	0.252	0.00305255	Monocytes	Nfatc1
2.78E-07	-0.5353447	0.144	0.178	0.00394198	Monocytes	Ero1lb
2.88E-07	-0.3766333	0.344	0.36	0.00407839	Monocytes	Sfpq
2.92E-07	-0.4076866	0.248	0.27	0.00412701	Monocytes	Tprgl
2.98E-07	-0.4705561	0.227	0.252	0.00421914	Monocytes	Smap2
3.02E-07	-0.4668076	0.178	0.213	0.00427359	Monocytes	Chd3
3.21E-07	-0.4221128	0.131	0.168	0.00454309	Monocytes	Etnk1
3.49E-07	-0.3093946	0.07	0.105	0.00493572	Monocytes	Itm2a
3.62E-07	-0.3834098	0.186	0.221	0.00512082	Monocytes	Cbx4
3.84E-07	-0.4082856	0.319	0.334	0.00543067	Monocytes	Cacybp
3.95E-07	-0.4289506	0.395	0.411	0.00558742	Monocytes	Txnip
4.61E-07	-0.2935178	0.076	0.111	0.0065194	Monocytes	Senp7
4.94E-07	-0.4781979	0.152	0.187	0.0069973	Monocytes	Elmsan1
6.55E-07	-0.3682208	0.132	0.168	0.00926865	Monocytes	Dedd2
6.81E-07	-0.4768945	0.301	0.327	0.00963738	Monocytes	Cxcr4
8.47E-07	-0.3470609	0.088	0.123	0.01199382	Monocytes	Grap2
9.98E-07	-0.3038988	0.081	0.115	0.01412391	Monocytes	Unc45a
1.02E-06	-0.3650066	0.227	0.251	0.0144897	Monocytes	Rbbp4
1.06E-06	-0.4329115	0.182	0.213	0.01494414	Monocytes	Rcsd1
1.14E-06	-0.371245	0.16	0.194	0.0161167	Monocytes	Amd1
1.15E-06	-0.377809	0.149	0.183	0.01631643	Monocytes	Vars
1.45E-06	-0.3269119	0.126	0.161	0.02050785	Monocytes	Prrc2b
1.48E-06	-0.4124	0.193	0.227	0.02091678	Monocytes	Tob1
1.79E-06	-0.286349	0.069	0.102	0.02528747	Monocytes	Ttc3
1.92E-06	-0.3949972	0.203	0.235	0.02715217	Monocytes	D16Ertd472e
1.97E-06	-0.2635369	0.451	0.448	0.02795164	Monocytes	Nol7
2.00E-06	-0.36828	0.301	0.315	0.02830407	Monocytes	Rnf187
2.02E-06	-0.4432059	0.203	0.229	0.02862166	Monocytes	Rnf138
2.19E-06	-0.5290175	0.557	0.528	0.03095942	Monocytes	Hmgb2
2.89E-06	-0.2953104	0.092	0.124	0.04089879	Monocytes	Ccng2
3.54E-06	-0.3449685	0.093	0.125	0.05004242	Monocytes	Hmces
4.12E-06	-0.3519617	0.118	0.15	0.05828946	Monocytes	Swt1
4.38E-06	-0.4155512	0.146	0.177	0.06204168	Monocytes	Gtf2i

4.51E-06	-0.4119132	0.208	0.236	0.06379866	Monocytes	Odc1
4.68E-06	-0.518791	0.141	0.175	0.06622699	Monocytes	Tnfrsf9
4.90E-06	-0.3367052	0.112	0.145	0.06943463	Monocytes	Il16
5.28E-06	-0.2870465	0.522	0.503	0.07469891	Monocytes	Srsf5
5.35E-06	-0.3212373	0.101	0.133	0.07573803	Monocytes	Ccdc117
5.76E-06	-0.4077814	0.343	0.345	0.08157375	Monocytes	Ywhaq
6.39E-06	-0.4487707	0.096	0.128	0.09049473	Monocytes	Rgs16
6.43E-06	-0.3679978	0.19	0.219	0.09108903	Monocytes	Top2b
7.53E-06	-0.4147742	0.149	0.178	0.10653989	Monocytes	B3gnt2
7.74E-06	-0.6246523	0.107	0.07	0.10951281	Monocytes	Slc7a11
8.15E-06	-0.3687568	0.207	0.233	0.11533244	Monocytes	Tuba4a
8.78E-06	-0.293035	0.383	0.38	0.12426724	Monocytes	Syf2
9.52E-06	-0.374708	0.322	0.329	0.13477277	Monocytes	Stk4
9.92E-06	-0.3587806	0.379	0.376	0.14043276	Monocytes	BC031181
1.06E-05	-0.3421505	0.167	0.195	0.15017185	Monocytes	Uchl3
1.12E-05	-0.2705606	0.084	0.114	0.15872206	Monocytes	Msi2
1.14E-05	-0.3190878	0.417	0.403	0.1609432	Monocytes	Skp1a
1.15E-05	-0.2960986	0.091	0.123	0.16294319	Monocytes	Cd226
1.16E-05	-0.4232804	0.262	0.279	0.16370291	Monocytes	Ddx6
1.18E-05	-0.3323847	0.084	0.115	0.16769045	Monocytes	Arsb
1.31E-05	-0.4962297	0.289	0.3	0.1850045	Monocytes	Cdk11b
1.44E-05	-0.3334652	0.122	0.153	0.20406876	Monocytes	Ogt
1.60E-05	-0.4067811	0.344	0.343	0.22616512	Monocytes	B4galnt1
1.67E-05	-0.3754847	0.127	0.156	0.23694893	Monocytes	Crebrf
1.79E-05	-0.45302	0.118	0.146	0.25359289	Monocytes	Swap70
1.80E-05	-0.4065596	0.29	0.303	0.2546111	Monocytes	Zc3hav1
2.58E-05	-0.3697076	0.122	0.149	0.36458938	Monocytes	Csnk1g3
2.72E-05	-0.31066	0.303	0.318	0.38439382	Monocytes	Hnrnph1
2.72E-05	-0.4512424	0.137	0.164	0.38503725	Monocytes	Gpr18
2.92E-05	-0.3820442	0.168	0.194	0.41367938	Monocytes	Tiprl
2.99E-05	-0.3370629	0.125	0.153	0.42260021	Monocytes	Arhgap4
3.25E-05	-0.3788772	0.2	0.222	0.46040806	Monocytes	Stip1
3.49E-05	-0.4304457	0.173	0.199	0.49357558	Monocytes	Rinl
3.70E-05	-0.7395311	0.204	0.228	0.52377734	Monocytes	Lag3
4.17E-05	-0.4080147	0.194	0.218	0.58990631	Monocytes	Tnrc6b
4.47E-05	-0.371831	0.143	0.171	0.63296837	Monocytes	Fryl
4.78E-05	-0.3851197	0.226	0.244	0.67633045	Monocytes	Akna
4.80E-05	-0.3873735	0.333	0.337	0.67994553	Monocytes	Ncor1
4.94E-05	-0.2897215	0.16	0.188	0.69950204	Monocytes	4932438A13I
5.11E-05	-0.4319754	0.297	0.302	0.72313868	Monocytes	Tmod3
5.52E-05	-0.3003515	0.115	0.143	0.78148	Monocytes	Traf3ip3
5.70E-05	-0.2707505	0.471	0.457	0.80732116	Monocytes	Srp14
5.76E-05	-0.3509743	0.148	0.176	0.81575106	Monocytes	Suco
6.19E-05	-0.3261504	0.418	0.402	0.87577421	Monocytes	Sf3b2
6.57E-05	-0.45824	0.146	0.171	0.9301272	Monocytes	Gm26740
6.66E-05	-0.3221622	0.27	0.281	0.94272696	Monocytes	Khdrbs1
6.72E-05	-0.2975855	0.153	0.178	0.95143569	Monocytes	Mfap1b
7.31E-05	-0.904565	0.7	0.501	1	Monocytes	Ccl5

7.42E-05	-0.3378224	0.123	0.148	1	Monocytes	Flt3l
7.64E-05	-0.2554945	0.117	0.145	1	Monocytes	Usp38
8.63E-05	-0.4087343	0.394	0.382	1	Monocytes	Hspa4
8.69E-05	-0.3698348	0.272	0.287	1	Monocytes	Arid5a
8.89E-05	-0.3986405	0.16	0.183	1	Monocytes	Mfap1a
9.27E-05	-0.314588	0.191	0.213	1	Monocytes	Sms
0.00010274	-0.2570781	0.085	0.112	1	Monocytes	Myo1e
0.00011164	-0.3523768	0.301	0.303	1	Monocytes	Anxa6
0.0001164	-0.2799932	0.363	0.36	1	Monocytes	Emg1
0.00012624	-0.405584	0.207	0.224	1	Monocytes	Bptf
0.00013627	-0.3151534	0.118	0.144	1	Monocytes	Clcn3
0.00014062	-0.2826987	0.517	0.495	1	Monocytes	Pcbp1
0.00014311	-0.3140848	0.475	0.454	1	Monocytes	Eif5
0.00014728	-0.2960673	0.154	0.177	1	Monocytes	Polr2m
0.00015665	-0.2853657	0.162	0.187	1	Monocytes	Krit1
0.00015688	-0.4076712	0.188	0.207	1	Monocytes	Pcmtd1
0.00016879	0.2956509	0.862	0.866	1	Monocytes	mt-Nd1
0.00017807	-0.2541606	0.465	0.447	1	Monocytes	Cox17
0.00018327	-0.3570298	0.235	0.252	1	Monocytes	Arhgap15
0.00018543	-0.416181	0.236	0.249	1	Monocytes	Cyth1
0.00019335	-0.3221631	0.113	0.137	1	Monocytes	Helz2
0.00019946	-0.3100146	0.084	0.107	1	Monocytes	Trp53inp2
0.00020439	-0.3044217	0.083	0.106	1	Monocytes	Rpia
0.00021514	-0.3566272	0.296	0.299	1	Monocytes	Slc50a1
0.00021807	-0.3517559	0.184	0.205	1	Monocytes	Tagap
0.00021891	-0.2855775	0.265	0.275	1	Monocytes	Eif4b
0.00022035	-0.5623484	0.189	0.21	1	Monocytes	Dgat1
0.00025918	-0.2958483	0.441	0.435	1	Monocytes	Tpr
0.00026489	-0.3523296	0.144	0.165	1	Monocytes	Rnf44
0.00029963	-0.4377391	0.209	0.227	1	Monocytes	Arhgap31
0.00030223	-0.3712184	0.28	0.288	1	Monocytes	Phf20l1
0.0003102	-0.2708165	0.089	0.113	1	Monocytes	Brwd1
0.00032777	-0.2938191	0.123	0.145	1	Monocytes	Rfk
0.00044039	-0.2560427	0.134	0.156	1	Monocytes	5031425E22F
0.00044065	-0.3235725	0.363	0.352	1	Monocytes	Rp9
0.00046494	-0.4701051	0.09	0.114	1	Monocytes	Serpinb9
0.0004776	-0.6298237	0.157	0.184	1	Monocytes	Cd7
0.00048786	-0.4703915	0.379	0.363	1	Monocytes	Ubald2
0.00050328	-0.2850164	0.083	0.106	1	Monocytes	Elk4
0.00051454	-0.329171	0.139	0.161	1	Monocytes	Sidt2
0.00057585	-0.284983	0.379	0.376	1	Monocytes	Prrc2c
0.00060051	-0.3631977	0.174	0.197	1	Monocytes	Stmn1
0.00063213	-0.3379047	0.133	0.153	1	Monocytes	Gdi1
0.00066394	-0.5123668	0.197	0.218	1	Monocytes	Cxcr6
0.00067524	-0.3781202	0.229	0.242	1	Monocytes	Ankrd12
0.00071697	-0.352986	0.312	0.311	1	Monocytes	Smarca5
0.00074754	-0.3435906	0.165	0.184	1	Monocytes	Pkp3
0.0007628	-0.2933867	0.095	0.117	1	Monocytes	Cdkn2aip

0.0007729	-0.3109494	0.123	0.143	1	Monocytes	Nelfb
0.00078493	-0.3822765	0.191	0.206	1	Monocytes	Phip
0.00078736	-0.2901372	0.298	0.309	1	Monocytes	Ifi27
0.00080958	-0.3179584	0.31	0.306	1	Monocytes	Mob4
0.00085869	-0.2937852	0.136	0.155	1	Monocytes	Prkacb
0.00086034	-0.4658827	0.271	0.274	1	Monocytes	Sik1
0.00096452	-0.2606268	0.172	0.192	1	Monocytes	Dnajb9
0.00097825	-0.2875405	0.094	0.115	1	Monocytes	Esyt2
0.00098659	-0.4614203	0.13	0.15	1	Monocytes	Nudt4
0.00100429	-0.3218692	0.298	0.306	1	Monocytes	Tspan13
0.00102834	-0.2707729	0.194	0.211	1	Monocytes	Rad21
0.00118513	-0.3014832	0.13	0.149	1	Monocytes	Map4k1
0.00120389	-0.3157902	0.181	0.196	1	Monocytes	Hnrnph3
0.00123204	-0.3570722	0.184	0.2	1	Monocytes	Ccnt1
0.00123208	-0.3558813	0.256	0.26	1	Monocytes	Snx2
0.00123895	-0.3683327	0.572	0.568	1	Monocytes	Zfp36l2
0.00137701	-0.3283078	0.129	0.149	1	Monocytes	Samd9l
0.00138463	-0.2749179	0.083	0.104	1	Monocytes	Klrc1
0.00145872	-0.3099889	0.125	0.143	1	Monocytes	Siah2
0.00146677	-0.2793063	0.255	0.261	1	Monocytes	Wbp2
0.00146938	-0.2698251	0.107	0.127	1	Monocytes	Cep57
0.00147678	-0.2788442	0.135	0.154	1	Monocytes	Numa1
0.00149714	-0.3291937	0.256	0.259	1	Monocytes	Anp32e
0.00152721	-0.310515	0.393	0.372	1	Monocytes	Leprotl1
0.00166612	-0.3206023	0.159	0.174	1	Monocytes	Pink1
0.00177538	-0.2687089	0.184	0.142	1	Monocytes	Plk2
0.00181831	-0.3109578	0.306	0.302	1	Monocytes	Dusp11
0.00184984	-0.3655535	0.308	0.303	1	Monocytes	Rsrc2
0.00194804	-0.3162285	0.131	0.149	1	Monocytes	5430416N02I
0.00200014	-0.2644565	0.16	0.177	1	Monocytes	Cnot6l
0.0020097	-0.3465602	0.149	0.167	1	Monocytes	Kmt2a
0.00209316	-0.3799702	0.171	0.184	1	Monocytes	Senp6
0.00211644	-0.259984	0.297	0.294	1	Monocytes	Bcas2
0.00223351	-0.2778602	0.34	0.326	1	Monocytes	Cct7
0.00225402	-0.4363848	0.233	0.238	1	Monocytes	Tgoln1
0.00252914	-0.3279477	0.186	0.198	1	Monocytes	Chordc1
0.00261072	-0.2892771	0.183	0.197	1	Monocytes	N4bp2l2
0.00267003	-0.3849179	0.225	0.233	1	Monocytes	Epc1
0.00286952	-0.3183	0.233	0.238	1	Monocytes	Ctbp1
0.0029155	0.42133397	0.852	0.845	1	Monocytes	mt-Nd4l
0.00295742	-0.4238986	0.148	0.162	1	Monocytes	Ugcg
0.00322386	-0.4319051	0.169	0.178	1	Monocytes	Snx9
0.0034723	-0.2590898	0.48	0.446	1	Monocytes	Supt4a
0.00354708	-0.3174937	0.237	0.248	1	Monocytes	Cyfip2
0.0036266	-0.2606738	0.144	0.161	1	Monocytes	Gramd1a
0.003679	-0.2802661	0.188	0.201	1	Monocytes	Cbx3
0.00385873	-0.474245	0.11	0.128	1	Monocytes	Gzmk
0.00393003	-0.3338669	0.2	0.211	1	Monocytes	Atf7ip

0.00403613	-0.2753566	0.129	0.146	1	Monocytes	Phf6
0.00427375	-0.2674051	0.14	0.156	1	Monocytes	Alkbh1
0.00448526	-0.3218203	0.709	0.712	1	Monocytes	Btg2
0.00468329	-0.2812789	0.207	0.216	1	Monocytes	Srpk1
0.00514564	-0.307676	0.093	0.11	1	Monocytes	Cxcr3
0.00538216	-0.2775995	0.098	0.115	1	Monocytes	Gle1
0.00577924	0.38718899	0.755	0.714	1	Monocytes	mt-Nd4
0.00606323	-0.3337428	0.264	0.263	1	Monocytes	Esyt1
0.00610203	-0.2803845	0.276	0.273	1	Monocytes	Ptbp1
0.00646314	-0.3478047	0.189	0.198	1	Monocytes	Prpf8
0.00653631	-0.2592515	0.633	0.644	1	Monocytes	S100a10
0.00781749	-0.2500972	0.19	0.201	1	Monocytes	Slc38a1
0.00787567	-0.39702	0.117	0.134	1	Monocytes	Izumo1r
0.00828701	-0.2573164	0.204	0.211	1	Monocytes	Wdr33
0.00871261	-0.3399812	0.224	0.228	1	Monocytes	Ddx50
0.0088309	-0.3156291	0.168	0.18	1	Monocytes	Kdm5a
0.00936469	-0.2587752	0.118	0.132	1	Monocytes	Nsmaf
0	6.24491028	0.982	0.108	0	Granulocytes	S100a9
0	6.18062808	0.988	0.096	0	Granulocytes	S100a8
0	5.99303311	0.908	0.039	0	Granulocytes	Retnlg
0	5.03653457	0.785	0.049	0	Granulocytes	G0s2
0	4.67665455	0.863	0.067	0	Granulocytes	Il1r2
0	4.63867923	0.916	0.071	0	Granulocytes	Slpi
0	4.48089125	0.971	0.222	0	Granulocytes	Il1b
0	4.25224217	0.853	0.046	0	Granulocytes	Hdc
0	4.15322207	0.836	0.074	0	Granulocytes	Trem1
0	4.08316362	0.501	0.029	0	Granulocytes	Lcn2
0	3.98413623	0.748	0.096	0	Granulocytes	Tnfaip2
0	3.83417563	0.8	0.059	0	Granulocytes	Csf3r
0	3.82225873	0.92	0.214	0	Granulocytes	Ccl6
0	3.75654436	0.609	0.03	0	Granulocytes	Wfdc21
0	3.74568098	0.978	0.286	0	Granulocytes	Msrb1
0	3.73088987	0.708	0.036	0	Granulocytes	Slc7a11
0	3.67938741	0.769	0.056	0	Granulocytes	Clec4d
0	3.61031022	0.832	0.112	0	Granulocytes	Hp
0	3.49598431	0.708	0.018	0	Granulocytes	Mmp9
0	3.42938589	0.591	0.05	0	Granulocytes	Hcar2
0	3.33764032	0.476	0.027	0	Granulocytes	Lrg1
0	3.12287306	0.593	0.015	0	Granulocytes	Cxcr2
0	3.06742997	0.352	0.012	0	Granulocytes	Asprv1
0	3.00975849	0.307	0.01	0	Granulocytes	Stfa2l1
0	2.70899441	0.47	0.018	0	Granulocytes	H2-Q10
0	2.21329154	0.335	0.008	0	Granulocytes	Il1f9
0	2.10652041	0.344	0.01	0	Granulocytes	Dhrs9
0	1.85627231	0.299	0.005	0	Granulocytes	Slc40a1
2.43E-304	2.77234848	0.935	0.264	3.44E-300	Granulocytes	Alox5ap
1.46E-298	2.68407273	0.998	0.401	2.07E-294	Granulocytes	Tyrobp
7.39E-295	2.95108753	0.708	0.132	1.05E-290	Granulocytes	Gsr

6.21E-290	2.33716195	0.378	0.024	8.80E-286	Granulocytes	Arg2
6.96E-290	2.95461283	0.632	0.1	9.86E-286	Granulocytes	Adam8
1.52E-287	3.03085459	0.92	0.269	2.16E-283	Granulocytes	Ifitm2
5.50E-286	1.8204744	0.26	0.006	7.78E-282	Granulocytes	Chil1
2.48E-282	2.74171349	0.613	0.094	3.52E-278	Granulocytes	C5ar1
1.84E-277	3.22551999	0.849	0.268	2.61E-273	Granulocytes	Grina
1.38E-273	2.68511497	0.505	0.059	1.96E-269	Granulocytes	Ccr1
2.88E-271	3.34551653	0.524	0.064	4.07E-267	Granulocytes	Thbs1
6.44E-268	2.97228236	0.982	0.627	9.11E-264	Granulocytes	S100a11
1.25E-267	3.11045498	0.675	0.134	1.77E-263	Granulocytes	Slc16a3
1.92E-265	3.1758683	0.781	0.209	2.72E-261	Granulocytes	Lmnb1
2.96E-262	3.65984419	0.562	0.079	4.19E-258	Granulocytes	Ifitm1
1.29E-261	3.11446142	0.599	0.095	1.83E-257	Granulocytes	Clec4e
9.64E-258	2.48208501	0.479	0.055	1.36E-253	Granulocytes	Gcnt2
3.53E-253	3.03461356	0.826	0.26	5.00E-249	Granulocytes	Mxd1
3.79E-248	2.66274816	0.583	0.096	5.36E-244	Granulocytes	Cd300ld
1.26E-246	4.12402421	0.689	0.147	1.79E-242	Granulocytes	Cxcl2
2.16E-244	2.25038712	0.996	0.921	3.06E-240	Granulocytes	Srgn
4.90E-243	-3.1010987	0.323	0.971	6.94E-239	Granulocytes	Rpsa
1.80E-242	3.06060213	0.429	0.045	2.55E-238	Granulocytes	Ptgs2
6.95E-238	2.96945342	0.53	0.081	9.84E-234	Granulocytes	Nlrp3
6.45E-234	2.09945353	0.307	0.019	9.13E-230	Granulocytes	Mmp8
1.78E-232	-2.650704	0.495	0.969	2.52E-228	Granulocytes	Rpl13
7.99E-232	3.18453165	0.953	0.544	1.13E-227	Granulocytes	Cebpb
8.29E-231	2.42171379	0.481	0.065	1.17E-226	Granulocytes	Mcemp1
2.52E-228	-2.8361919	0.464	0.961	3.57E-224	Granulocytes	Eef1a1
1.77E-226	-2.868355	0.321	0.952	2.50E-222	Granulocytes	Rps4x
6.64E-226	-3.0286969	0.256	0.937	9.40E-222	Granulocytes	Rpl32
7.14E-226	-2.8023346	0.297	0.951	1.01E-221	Granulocytes	Ppia
2.15E-224	2.6436954	0.673	0.155	3.04E-220	Granulocytes	Cd9
1.53E-223	-2.4215748	0.564	0.968	2.17E-219	Granulocytes	Rps8
1.50E-220	2.4498961	0.92	0.51	2.12E-216	Granulocytes	Taldo1
2.18E-219	-2.9083741	0.382	0.951	3.09E-215	Granulocytes	Rps15a
3.90E-218	-2.5853965	0.403	0.958	5.52E-214	Granulocytes	Rps2
1.21E-217	-2.4725351	0.464	0.954	1.71E-213	Granulocytes	Rpl19
1.55E-217	-2.9216297	0.282	0.946	2.20E-213	Granulocytes	Rps20
1.66E-217	2.283595	0.297	0.02	2.35E-213	Granulocytes	Slfn4
3.64E-217	-2.8788221	0.368	0.966	5.15E-213	Granulocytes	Tmsb10
2.63E-216	-2.5164035	0.387	0.949	3.73E-212	Granulocytes	Rpl6
2.46E-215	-2.5759369	0.595	0.964	3.48E-211	Granulocytes	Rps24
1.89E-214	2.89936005	0.72	0.205	2.68E-210	Granulocytes	Lilr4b
2.50E-212	2.71847248	0.738	0.226	3.54E-208	Granulocytes	Ncf2
1.03E-211	-2.622412	0.38	0.944	1.46E-207	Granulocytes	Rps5
1.82E-211	-2.4246895	0.423	0.95	2.58E-207	Granulocytes	Rps11
1.05E-210	-2.3319597	0.413	0.945	1.49E-206	Granulocytes	Rpl11
2.95E-210	-2.4725567	0.493	0.96	4.17E-206	Granulocytes	Rplp0
2.41E-209	2.78520998	0.847	0.353	3.42E-205	Granulocytes	Pglyrp1
6.81E-209	1.3089993	0.168	0.002	9.64E-205	Granulocytes	Gm20406

5.53E-206	1.7820152	0.319	0.026	7.82E-202	Granulocytes	Trim30b
8.43E-206	-2.3148066	0.546	0.956	1.19E-201	Granulocytes	Rps3a1
1.07E-205	2.71230403	0.448	0.063	1.51E-201	Granulocytes	Osm
3.33E-205	-2.6584893	0.258	0.933	4.72E-201	Granulocytes	Ptma
6.65E-205	-2.9096727	0.143	0.894	9.41E-201	Granulocytes	Rpl3
2.58E-204	-2.5239376	0.37	0.937	3.65E-200	Granulocytes	Rps26
6.56E-203	-2.8071625	0.274	0.939	9.28E-199	Granulocytes	Hsp90ab1
1.19E-200	-2.0885448	0.683	0.962	1.69E-196	Granulocytes	Uba52
3.92E-200	1.78578169	0.327	0.029	5.55E-196	Granulocytes	Cd33
1.33E-199	-2.8056075	0.145	0.898	1.89E-195	Granulocytes	Rpl10a
2.29E-199	-2.0523787	0.501	0.98	3.25E-195	Granulocytes	mt-Co2
5.19E-198	-2.3708784	0.462	0.938	7.35E-194	Granulocytes	Rplp1
5.28E-198	2.43679074	0.519	0.095	7.47E-194	Granulocytes	Gda
1.62E-197	-2.4603746	0.425	0.937	2.29E-193	Granulocytes	Rps7
2.64E-196	-2.5169337	0.337	0.922	3.73E-192	Granulocytes	Rpl13a
2.30E-195	-2.7449689	0.231	0.91	3.26E-191	Granulocytes	Rps19
2.36E-194	-2.2627879	0.411	0.936	3.34E-190	Granulocytes	Rpl8
1.36E-193	-2.6063944	0.284	0.907	1.92E-189	Granulocytes	Rpl35
8.75E-193	-2.0171006	0.499	0.98	1.24E-188	Granulocytes	mt-Co3
1.22E-192	-2.4995558	0.331	0.908	1.73E-188	Granulocytes	Rpl36
1.64E-192	-2.5817464	0.319	0.95	2.32E-188	Granulocytes	mt-Atp8
1.56E-191	-2.8096676	0.164	0.877	2.22E-187	Granulocytes	Rps18
4.47E-191	-2.1591851	0.489	0.935	6.33E-187	Granulocytes	Rps3
1.63E-190	1.61228963	0.198	0.007	2.31E-186	Granulocytes	Mrgpra2b
1.39E-188	-2.3798859	0.46	0.923	1.97E-184	Granulocytes	Rpl39
2.52E-188	-1.9035332	0.677	0.982	3.57E-184	Granulocytes	mt-Co1
1.96E-187	2.15310711	0.405	0.055	2.78E-183	Granulocytes	Pygl
9.80E-187	-2.3300632	0.454	0.92	1.39E-182	Granulocytes	Rpl21
7.14E-185	-2.1548256	0.456	0.925	1.01E-180	Granulocytes	Rpl27a
7.66E-185	2.29196916	0.789	0.295	1.08E-180	Granulocytes	Spi1
9.47E-185	-2.4641564	0.239	0.894	1.34E-180	Granulocytes	Rpl15
6.85E-184	1.25428104	0.184	0.006	9.70E-180	Granulocytes	Phospho1
5.70E-183	-2.1038236	0.519	0.938	8.07E-179	Granulocytes	Rpl37a
5.95E-182	1.58530922	0.996	0.929	8.43E-178	Granulocytes	Ftl1
1.72E-181	-2.0024146	0.556	0.932	2.44E-177	Granulocytes	Rpl34
3.19E-180	-1.8729119	0.681	0.955	4.51E-176	Granulocytes	Rpl18a
6.81E-178	2.53609736	0.685	0.217	9.64E-174	Granulocytes	Ets2
1.11E-176	2.25067905	0.939	0.695	1.57E-172	Granulocytes	Btg2
4.21E-175	-1.7345522	0.697	0.955	5.97E-171	Granulocytes	Rpl23
4.87E-175	-2.1167359	0.476	0.916	6.89E-171	Granulocytes	Rps13
8.44E-174	-1.8622811	0.566	0.946	1.19E-169	Granulocytes	Rpl18
2.18E-171	-2.2212031	0.313	0.89	3.08E-167	Granulocytes	Rps23
4.04E-171	-1.8374735	0.624	0.943	5.72E-167	Granulocytes	Rpl30
4.76E-171	-2.0629758	0.417	0.915	6.74E-167	Granulocytes	Rpl28
5.03E-170	-2.3501878	0.231	0.863	7.12E-166	Granulocytes	Rpl22
7.71E-170	-2.3188604	0.217	0.869	1.09E-165	Granulocytes	Rpl14
1.08E-169	-2.0940089	0.372	0.96	1.53E-165	Granulocytes	mt-Cytb
5.63E-169	1.40399234	0.194	0.009	7.97E-165	Granulocytes	Fpr1

2.41E-168	-2.1050698	0.339	0.9	3.41E-164	Granulocytes	Rpl29
4.19E-168	-2.2570861	0.286	0.878	5.94E-164	Granulocytes	Rpl10-ps3
5.85E-168	1.61149587	0.284	0.026	8.28E-164	Granulocytes	Pi16
1.98E-167	2.32009622	0.706	0.227	2.80E-163	Granulocytes	Lst1
2.71E-166	2.13686148	0.47	0.09	3.84E-162	Granulocytes	Pilra
3.47E-166	-1.6829169	0.751	0.955	4.91E-162	Granulocytes	Rps16
5.27E-166	-1.9805304	0.397	0.911	7.46E-162	Granulocytes	Rps14
6.15E-166	2.32373113	0.71	0.246	8.70E-162	Granulocytes	Lilrb4a
8.56E-166	1.49759922	0.996	0.936	1.21E-161	Granulocytes	Actg1
2.81E-165	2.16347682	0.456	0.083	3.97E-161	Granulocytes	Cd300lf
4.26E-165	-2.0902626	0.339	0.888	6.03E-161	Granulocytes	Rpl26
1.50E-164	-1.7760282	0.634	0.935	2.12E-160	Granulocytes	Rps10
6.41E-164	-2.1837925	0.292	0.875	9.07E-160	Granulocytes	Rpl7
1.96E-162	-2.465964	0.225	0.891	2.78E-158	Granulocytes	mt-Nd4l
3.50E-162	1.40806768	0.998	0.983	4.95E-158	Granulocytes	Malat1
4.18E-162	-2.663185	0.112	0.802	5.91E-158	Granulocytes	Rpl12
8.67E-161	-1.7049246	0.644	0.937	1.23E-156	Granulocytes	Rpl9-ps6
1.03E-160	2.54120171	0.579	0.155	1.45E-156	Granulocytes	Trib1
2.07E-160	2.07016395	0.413	0.07	2.93E-156	Granulocytes	Sirpb1b
2.77E-160	2.15879079	0.855	0.502	3.92E-156	Granulocytes	Litaf
1.52E-159	-1.9598314	0.519	0.921	2.16E-155	Granulocytes	Rps21
1.52E-158	1.72264572	0.235	0.018	2.16E-154	Granulocytes	Ankrd33b
1.04E-157	-2.0193194	0.47	0.908	1.47E-153	Granulocytes	Rpl38
2.39E-156	1.89447014	0.965	0.589	3.38E-152	Granulocytes	S100a6
2.54E-156	-1.8783749	0.384	0.906	3.60E-152	Granulocytes	Rpl24
4.88E-156	-2.1211586	0.262	0.908	6.91E-152	Granulocytes	mt-Nd1
8.03E-156	1.89508859	0.99	0.955	1.14E-151	Granulocytes	Fth1
5.45E-155	2.35001745	0.665	0.231	7.72E-151	Granulocytes	Tpd52
1.54E-153	-2.3968578	0.35	0.873	2.18E-149	Granulocytes	Rps28
1.95E-153	-1.8828163	0.376	0.894	2.76E-149	Granulocytes	Rpl27
3.45E-152	-1.4587406	0.808	0.958	4.89E-148	Granulocytes	Gm10076
3.55E-151	-2.2783453	0.172	0.833	5.02E-147	Granulocytes	Rpl36a
1.39E-150	2.40382806	0.401	0.07	1.97E-146	Granulocytes	Sh2d3c
2.36E-150	-1.8527961	0.325	0.922	3.35E-146	Granulocytes	mt-Atp6
6.61E-150	-1.7445503	0.452	0.909	9.36E-146	Granulocytes	Rplp2
6.34E-149	-1.9997825	0.272	0.864	8.98E-145	Granulocytes	Rpl7a
1.38E-148	-2.2123682	0.123	0.799	1.96E-144	Granulocytes	Rpl36al
8.92E-147	1.68842365	0.303	0.037	1.26E-142	Granulocytes	Trem3
1.52E-146	1.51397225	0.965	0.72	2.15E-142	Granulocytes	Fxyd5
6.56E-146	1.24702146	0.176	0.009	9.29E-142	Granulocytes	Sgms2
5.85E-143	2.32320136	0.599	0.193	8.28E-139	Granulocytes	Ndel1
5.86E-143	-2.1814498	0.151	0.797	8.29E-139	Granulocytes	Rpl5
3.86E-141	-1.7028024	0.517	0.944	5.46E-137	Granulocytes	H2-K1
1.13E-140	-2.7455433	0.102	0.764	1.59E-136	Granulocytes	Crip1
5.28E-137	-1.9393982	0.231	0.838	7.48E-133	Granulocytes	Naca
1.10E-136	-1.8767323	0.476	0.895	1.56E-132	Granulocytes	Rpl35a
2.72E-136	2.26378567	0.675	0.267	3.85E-132	Granulocytes	Cd44
7.14E-136	-2.0265089	0.227	0.818	1.01E-131	Granulocytes	Rps6

8.82E-136	1.00430292	0.129	0.004	1.25E-131	Granulocytes	Nlrp12
2.82E-135	0.95040387	0.121	0.003	3.99E-131	Granulocytes	9830107B12I
4.71E-134	1.52862383	0.147	0.006	6.67E-130	Granulocytes	Ly6g
1.85E-132	-1.8869921	0.233	0.865	2.63E-128	Granulocytes	mt-Nd2
2.49E-132	-2.176306	0.223	0.81	3.53E-128	Granulocytes	Eef2
2.26E-130	1.91242667	0.344	0.056	3.20E-126	Granulocytes	Slc2a3
1.55E-129	1.09710288	0.117	0.003	2.19E-125	Granulocytes	Chac1
2.14E-129	1.91886323	0.546	0.151	3.03E-125	Granulocytes	Pla2g7
1.47E-128	1.42569026	0.213	0.019	2.08E-124	Granulocytes	Igf1r
1.73E-128	-2.1574367	0.088	0.718	2.45E-124	Granulocytes	Npm1
1.75E-128	1.76044892	0.268	0.033	2.48E-124	Granulocytes	Cass4
1.01E-127	-2.0595627	0.571	0.912	1.43E-123	Granulocytes	Rps29
2.43E-127	1.29272077	0.953	0.388	3.44E-123	Granulocytes	Fcer1g
2.82E-126	-2.1024114	0.123	0.736	3.99E-122	Granulocytes	Rpl4
2.64E-125	-1.730936	0.499	0.884	3.73E-121	Granulocytes	Rpl17
7.61E-124	-1.921555	0.442	0.904	1.08E-119	Granulocytes	Hspa8
1.90E-123	-1.6130005	0.474	0.886	2.69E-119	Granulocytes	Rps12
1.32E-122	-1.8351124	0.227	0.801	1.87E-118	Granulocytes	Eif3f
5.67E-121	-2.4719725	0.078	0.687	8.02E-117	Granulocytes	Hspe1
5.00E-119	-2.4716345	0.078	0.681	7.08E-115	Granulocytes	S100a10
1.75E-118	2.33735958	0.497	0.141	2.47E-114	Granulocytes	Anxa1
3.17E-118	1.21747573	0.184	0.015	4.48E-114	Granulocytes	Padi4
8.67E-117	2.26043819	0.863	0.607	1.23E-112	Granulocytes	Dusp1
1.96E-116	1.54906123	0.239	0.028	2.77E-112	Granulocytes	Cd300lb
4.43E-116	-2.0071199	0.108	0.698	6.26E-112	Granulocytes	Atp5g2
4.49E-116	2.42204502	0.507	0.156	6.35E-112	Granulocytes	Rnf149
2.67E-115	-2.1819779	0.08	0.675	3.78E-111	Granulocytes	Nme2
5.21E-113	2.02694281	0.454	0.123	7.37E-109	Granulocytes	Nudt4
1.54E-112	2.04614381	0.401	0.094	2.19E-108	Granulocytes	Slc15a3
3.94E-112	-1.7305882	0.198	0.775	5.58E-108	Granulocytes	Rps15
5.28E-112	-1.1293896	0.843	0.959	7.48E-108	Granulocytes	Tpt1
3.29E-110	2.41998599	0.515	0.169	4.65E-106	Granulocytes	Plk3
3.46E-110	2.01532937	0.716	0.4	4.89E-106	Granulocytes	Mrpl33
2.43E-109	-1.989918	0.151	0.731	3.44E-105	Granulocytes	mt-Nd5
3.06E-109	-2.0070656	0.078	0.654	4.34E-105	Granulocytes	Il2rg
9.93E-108	1.58829323	0.274	0.042	1.41E-103	Granulocytes	Gm5150
1.10E-107	2.72229708	0.618	0.25	1.55E-103	Granulocytes	Plaur
2.46E-107	1.80938705	0.245	0.033	3.49E-103	Granulocytes	Rdh12
6.17E-107	2.14209219	0.656	0.327	8.73E-103	Granulocytes	Adipor1
1.34E-106	1.98004138	0.751	0.449	1.89E-102	Granulocytes	Mcl1
1.49E-105	-1.8720458	0.137	0.694	2.11E-101	Granulocytes	Atp5g3
2.85E-105	1.88817807	0.413	0.106	4.03E-101	Granulocytes	Nfam1
4.50E-105	0.81657896	0.104	0.003	6.37E-101	Granulocytes	Ceacam10
2.39E-104	2.012456	0.552	0.202	3.38E-100	Granulocytes	Tnfrsf1a
2.50E-104	1.95121767	0.446	0.128	3.54E-100	Granulocytes	2310001H17I
3.25E-104	1.32025875	0.172	0.015	4.60E-100	Granulocytes	A530064D06I
2.71E-103	-1.9868741	0.082	0.64	3.84E-99	Granulocytes	Prdx1
5.98E-103	2.11677291	0.358	0.077	8.47E-99	Granulocytes	Il1rn

1.44E-102	2.02075373	0.542	0.197	2.03E-98	Granulocytes	Sorl1
1.49E-102	-1.7882555	0.143	0.709	2.11E-98	Granulocytes	Rpl22l1
1.47E-101	-1.8066019	0.119	0.668	2.09E-97	Granulocytes	Eif3h
1.60E-101	-1.5745277	0.26	0.797	2.26E-97	Granulocytes	Eef1b2
4.45E-101	-1.7421879	0.233	0.764	6.31E-97	Granulocytes	Shisa5
3.99E-100	1.40208546	0.239	0.034	5.65E-96	Granulocytes	Siglece
5.15E-100	1.66257379	0.767	0.436	7.29E-96	Granulocytes	Anxa2
1.37E-98	0.85304157	0.117	0.006	1.94E-94	Granulocytes	Rab44
5.84E-98	-2.1862444	0.065	0.604	8.26E-94	Granulocytes	H2-Q7
1.13E-97	1.56819003	0.252	0.039	1.60E-93	Granulocytes	1600010M07
1.73E-97	1.51128895	0.834	0.661	2.44E-93	Granulocytes	Gabarap
5.55E-97	-1.6471943	0.123	0.673	7.85E-93	Granulocytes	Rps17
3.51E-96	1.57666634	0.89	0.632	4.97E-92	Granulocytes	Fos
2.38E-94	-3.4041605	0.098	0.6	3.36E-90	Granulocytes	AW112010
7.14E-94	-1.6581607	0.131	0.667	1.01E-89	Granulocytes	Rpl31
9.56E-94	-1.8062221	0.061	0.581	1.35E-89	Granulocytes	Dad1
9.70E-94	2.26301991	0.501	0.167	1.37E-89	Granulocytes	Cd14
1.30E-93	-1.6180087	0.115	0.653	1.84E-89	Granulocytes	Nsa2
3.08E-93	0.91256868	0.108	0.005	4.36E-89	Granulocytes	Ptgs2os2
1.22E-92	1.69832521	0.734	0.443	1.73E-88	Granulocytes	Txn1
2.41E-92	-1.9029743	0.245	0.76	3.41E-88	Granulocytes	Hsp90aa1
7.82E-92	1.29280106	0.192	0.023	1.11E-87	Granulocytes	Scnn1a
4.40E-90	-2.2168657	0.055	0.561	6.23E-86	Granulocytes	Ptprcap
6.69E-90	-1.5096768	0.434	0.853	9.47E-86	Granulocytes	Ly6e
2.37E-89	-1.5657771	0.168	0.692	3.36E-85	Granulocytes	Rpl23a
4.07E-89	-1.3785777	0.307	0.801	5.76E-85	Granulocytes	Btf3
3.93E-88	-2.944528	0.067	0.558	5.57E-84	Granulocytes	Lgals1
7.02E-88	1.0813677	0.151	0.014	9.94E-84	Granulocytes	Pbx1
8.24E-88	0.91884427	0.123	0.008	1.17E-83	Granulocytes	Acta2
2.27E-87	0.82886903	0.982	0.942	3.22E-83	Granulocytes	Eif1
1.13E-86	-1.8351939	0.09	0.603	1.60E-82	Granulocytes	Hsp90b1
2.34E-86	-1.6829622	0.09	0.597	3.32E-82	Granulocytes	Hint1
1.11E-85	-1.5559536	0.1	0.613	1.57E-81	Granulocytes	Snrpg
1.51E-85	-1.4974211	0.164	0.677	2.14E-81	Granulocytes	Sumo2
3.97E-85	-1.9387214	0.094	0.592	5.62E-81	Granulocytes	Xist
1.04E-84	-1.6015608	0.094	0.595	1.48E-80	Granulocytes	Eef1d
2.41E-84	1.71397664	0.294	0.064	3.41E-80	Granulocytes	Smox
4.05E-84	1.72660613	0.656	0.357	5.74E-80	Granulocytes	Zyx
7.85E-84	-1.4673492	0.184	0.691	1.11E-79	Granulocytes	Ybx1
9.14E-83	-1.5090085	0.082	0.578	1.29E-78	Granulocytes	Serbp1
1.85E-82	-1.5080964	0.73	0.917	2.62E-78	Granulocytes	Rps27
5.69E-82	-2.0459317	0.033	0.508	8.06E-78	Granulocytes	Cd2
9.88E-82	1.71207849	0.509	0.197	1.40E-77	Granulocytes	Cyp4f18
1.22E-80	-1.5109104	0.065	0.557	1.73E-76	Granulocytes	Snrpe
1.80E-80	-0.9222009	0.814	0.937	2.54E-76	Granulocytes	Rpl37
1.93E-80	-1.1808573	0.419	0.844	2.73E-76	Granulocytes	Rpl41
1.95E-80	0.90940686	0.115	0.008	2.76E-76	Granulocytes	Tlr6
2.91E-80	-1.6655614	0.082	0.566	4.12E-76	Granulocytes	Ran

1.29E-79	0.94415991	0.129	0.011	1.82E-75	Granulocytes	Gm43661
2.44E-79	2.07633123	0.387	0.116	3.46E-75	Granulocytes	Tgm2
2.61E-79	-3.5195669	0.088	0.54	3.70E-75	Granulocytes	H2-Aa
5.17E-79	-2.4518094	0.11	0.587	7.32E-75	Granulocytes	Ctss
1.29E-78	1.55663745	0.346	0.092	1.82E-74	Granulocytes	Emilin2
3.39E-78	1.71120035	0.444	0.158	4.80E-74	Granulocytes	Themis2
3.64E-78	-3.6124861	0.282	0.67	5.16E-74	Granulocytes	Cd74
7.07E-78	1.4441394	0.941	0.872	1.00E-73	Granulocytes	Junb
9.03E-78	1.12811476	0.157	0.018	1.28E-73	Granulocytes	Lilra6
1.09E-77	1.7866034	0.356	0.101	1.55E-73	Granulocytes	Csf2rb
3.81E-77	1.6090896	0.294	0.069	5.40E-73	Granulocytes	Hacd4
6.78E-77	1.85849952	0.38	0.113	9.59E-73	Granulocytes	Mgst1
9.36E-77	-3.3218676	0.08	0.524	1.32E-72	Granulocytes	H2-Ab1
1.57E-76	1.2845056	0.834	0.699	2.22E-72	Granulocytes	Lsp1
4.11E-76	-1.4134193	0.168	0.657	5.82E-72	Granulocytes	Ppib
4.25E-76	1.44559014	0.734	0.465	6.02E-72	Granulocytes	Prdx5
6.44E-76	1.99208184	0.556	0.235	9.11E-72	Granulocytes	Ier3
6.88E-76	-1.2723929	0.331	0.785	9.74E-72	Granulocytes	Rpl9
8.51E-76	1.18556363	0.83	0.679	1.20E-71	Granulocytes	Lcp1
1.49E-75	1.55362768	0.245	0.048	2.11E-71	Granulocytes	Ptafr
6.47E-75	-2.8465074	0.037	0.481	9.15E-71	Granulocytes	Ms4a4b
9.89E-75	0.96286774	0.141	0.015	1.40E-70	Granulocytes	Alox5
1.38E-74	-1.4849499	0.059	0.518	1.96E-70	Granulocytes	Eef1g
2.83E-74	1.65942882	0.264	0.057	4.01E-70	Granulocytes	Slc25a33
3.38E-74	-1.5001706	0.137	0.609	4.78E-70	Granulocytes	Hnrnpa3
5.53E-74	-3.1785362	0.102	0.533	7.82E-70	Granulocytes	H2-Eb1
1.89E-73	-1.3517352	0.211	0.692	2.68E-69	Granulocytes	Psme1
1.12E-72	-1.5439409	0.031	0.465	1.59E-68	Granulocytes	Snrpf
1.37E-72	-1.9171244	0.051	0.496	1.93E-68	Granulocytes	Ets1
2.23E-72	-2.0006708	0.035	0.474	3.16E-68	Granulocytes	Hspd1
2.29E-72	1.02698779	0.149	0.017	3.24E-68	Granulocytes	Fpr2
3.36E-72	1.64699116	0.329	0.09	4.76E-68	Granulocytes	Ccpg1
1.19E-71	2.09402485	0.501	0.213	1.68E-67	Granulocytes	Nfkbiz
2.73E-71	-1.6514787	0.182	0.631	3.86E-67	Granulocytes	Npc2
2.85E-71	-2.691319	0.053	0.488	4.03E-67	Granulocytes	Ly6a
2.86E-71	-1.3097892	0.264	0.726	4.05E-67	Granulocytes	Psmb8
3.84E-71	1.016514	0.135	0.014	5.43E-67	Granulocytes	F5
4.29E-71	0.77203703	0.102	0.007	6.08E-67	Granulocytes	Bmx
4.65E-71	1.52956326	0.734	0.573	6.58E-67	Granulocytes	Map1lc3b
3.61E-70	1.67147639	0.436	0.162	5.11E-66	Granulocytes	Pirb
4.13E-70	1.63565248	0.297	0.076	5.84E-66	Granulocytes	Rabgef1
9.57E-70	-1.2395499	0.186	0.666	1.36E-65	Granulocytes	Cnbp
1.23E-69	1.57763097	0.358	0.109	1.74E-65	Granulocytes	Fgr
2.61E-69	1.15100667	0.17	0.024	3.69E-65	Granulocytes	Trpm2
6.42E-69	-3.6251759	0.086	0.49	9.09E-65	Granulocytes	Nkg7
1.15E-68	-1.515106	0.041	0.467	1.63E-64	Granulocytes	Krtcap2
1.87E-68	-1.1735009	0.254	0.72	2.64E-64	Granulocytes	Rps25
5.28E-68	1.49259065	0.254	0.057	7.47E-64	Granulocytes	Rasgrp4

1.22E-67	-1.1961181	0.174	0.64	1.73E-63	Granulocytes	Sec61g
1.23E-67	-1.4370057	0.086	0.52	1.74E-63	Granulocytes	Uqcrb
2.66E-67	-1.4638615	0.053	0.479	3.76E-63	Granulocytes	Erh
8.42E-67	-2.6394285	0.025	0.435	1.19E-62	Granulocytes	Cd3e
1.13E-66	1.76889582	0.796	0.604	1.60E-62	Granulocytes	Zfp36
1.56E-66	-1.4309982	0.049	0.474	2.21E-62	Granulocytes	Eif3i
1.99E-66	-1.2601548	0.196	0.658	2.82E-62	Granulocytes	Atp5d
6.89E-66	-1.1450736	0.297	0.756	9.76E-62	Granulocytes	mt-Nd4
9.24E-66	-1.3756603	0.088	0.526	1.31E-61	Granulocytes	Gstp1
9.61E-66	0.81709189	0.117	0.011	1.36E-61	Granulocytes	Pram1
1.65E-65	-1.3683836	0.051	0.477	2.33E-61	Granulocytes	Nol7
2.74E-65	0.6215876	0.998	0.982	3.87E-61	Granulocytes	Tmsb4x
6.18E-65	0.86005404	0.108	0.009	8.75E-61	Granulocytes	Tacstd2
7.23E-65	1.55469463	0.268	0.066	1.02E-60	Granulocytes	Rab11fip1
8.10E-65	-2.7971375	0.063	0.465	1.15E-60	Granulocytes	Cd3g
8.25E-65	-2.405999	0.104	0.522	1.17E-60	Granulocytes	Hspa1b
9.19E-65	-1.5530984	0.016	0.417	1.30E-60	Granulocytes	Cd48
1.74E-64	-1.6575904	0.047	0.465	2.46E-60	Granulocytes	Tuba1b
2.29E-64	-1.4042563	0.1	0.537	3.25E-60	Granulocytes	Mbnl1
4.77E-64	1.75335719	0.658	0.449	6.75E-60	Granulocytes	Pim1
1.08E-63	1.53707804	0.292	0.08	1.53E-59	Granulocytes	Abtb1
1.42E-63	-1.8898264	0.035	0.438	2.01E-59	Granulocytes	Gimap6
1.74E-63	1.63901823	0.266	0.066	2.47E-59	Granulocytes	Stx11
2.16E-63	-1.0857568	0.219	0.688	3.06E-59	Granulocytes	Dynll1
2.72E-63	-4.1241462	0.235	0.578	3.86E-59	Granulocytes	Ccl5
3.55E-63	-1.060583	0.249	0.715	5.03E-59	Granulocytes	Cox7c
4.98E-63	-1.2870446	0.061	0.479	7.06E-59	Granulocytes	Nop10
5.54E-63	-1.4276533	0.17	0.602	7.84E-59	Granulocytes	Psme2
6.65E-63	-1.3437681	0.033	0.436	9.41E-59	Granulocytes	Eif3e
9.44E-63	-1.4932291	0.086	0.516	1.34E-58	Granulocytes	Mif
1.37E-62	1.32184876	0.73	0.582	1.94E-58	Granulocytes	Gmfg
1.58E-62	-1.3620095	0.049	0.453	2.24E-58	Granulocytes	Hnrnpa1
1.75E-62	-1.5758557	0.157	0.589	2.48E-58	Granulocytes	Zfp36l1
1.79E-62	1.62942837	0.632	0.383	2.53E-58	Granulocytes	Neat1
4.51E-62	-1.2532812	0.084	0.5	6.38E-58	Granulocytes	Atp5a1
7.10E-62	-1.2673092	0.119	0.549	1.01E-57	Granulocytes	Snrpb
2.09E-61	1.6603954	0.358	0.122	2.96E-57	Granulocytes	Slfn1
2.39E-61	-1.4267649	0.02	0.41	3.38E-57	Granulocytes	Fkbp1a
2.49E-61	-1.5518061	0.039	0.438	3.53E-57	Granulocytes	Tubb5
3.89E-61	0.78137931	0.669	0.293	5.51E-57	Granulocytes	Ifitm3
5.87E-61	1.30902497	0.231	0.051	8.31E-57	Granulocytes	Pilrb2
7.95E-61	-1.1780688	0.256	0.704	1.13E-56	Granulocytes	Hmgb1
1.36E-60	-1.1289473	0.339	0.749	1.92E-56	Granulocytes	Rbm3
2.84E-60	1.03291282	0.141	0.019	4.02E-56	Granulocytes	Il1rap
3.50E-60	1.78655195	0.419	0.175	4.95E-56	Granulocytes	Dhrs7
4.52E-60	2.20409936	0.524	0.267	6.40E-56	Granulocytes	Egr1
4.61E-60	-1.537986	0.037	0.433	6.53E-56	Granulocytes	H2-Q6
6.40E-60	1.72246568	0.54	0.298	9.07E-56	Granulocytes	Snx20

1.06E-59	-1.5376865	0.057	0.45	1.50E-55	Granulocytes	1-Sep
1.64E-59	-1.2991945	0.063	0.468	2.32E-55	Granulocytes	Mrpl52
3.97E-59	-1.2042188	0.149	0.576	5.62E-55	Granulocytes	Ndufa4
5.21E-59	0.98911769	0.131	0.016	7.38E-55	Granulocytes	F630028O10I
5.27E-59	-1.7321036	0.043	0.435	7.46E-55	Granulocytes	Ahnak
5.50E-59	1.47972451	0.675	0.505	7.79E-55	Granulocytes	Rhog
6.38E-59	1.27290193	0.495	0.209	9.03E-55	Granulocytes	Fcgr3
9.20E-59	-2.7565272	0.094	0.49	1.30E-54	Granulocytes	Hspa1a
1.14E-58	-1.3213047	0.041	0.426	1.61E-54	Granulocytes	Mdh1
1.50E-58	-1.0761241	0.307	0.728	2.12E-54	Granulocytes	Uqcrh
1.55E-58	-0.6982716	0.861	0.939	2.19E-54	Granulocytes	Rps27a
2.84E-58	-1.1572757	0.188	0.624	4.02E-54	Granulocytes	Gnas
2.90E-58	0.9850172	0.139	0.019	4.10E-54	Granulocytes	Dmxl2
4.64E-58	-1.1047332	0.194	0.631	6.57E-54	Granulocytes	Eif3k
6.04E-58	-2.2838728	0.055	0.432	8.55E-54	Granulocytes	Cd3d
8.73E-58	-1.1892785	0.18	0.605	1.24E-53	Granulocytes	Cox5a
1.05E-57	1.98121606	0.663	0.492	1.48E-53	Granulocytes	Cdk2ap2
1.58E-57	-1.8760201	0.061	0.45	2.24E-53	Granulocytes	Nr4a2
1.72E-57	-1.5857239	0.033	0.407	2.43E-53	Granulocytes	Snx5
2.16E-57	-1.198765	0.125	0.543	3.06E-53	Granulocytes	Ncl
4.28E-57	1.6108477	0.446	0.199	6.06E-53	Granulocytes	Ninj1
6.28E-57	-1.0406892	0.258	0.693	8.89E-53	Granulocytes	Rpl10
7.45E-57	1.7490939	0.577	0.305	1.05E-52	Granulocytes	Atf3
1.04E-56	-1.1964883	0.125	0.54	1.47E-52	Granulocytes	Cycs
1.37E-56	0.96160664	0.139	0.02	1.94E-52	Granulocytes	Ppp1r3b
1.76E-56	1.41113001	0.524	0.26	2.49E-52	Granulocytes	Sell
2.06E-56	-1.7950776	0.061	0.439	2.92E-52	Granulocytes	Cd69
2.32E-56	0.9837606	0.153	0.024	3.28E-52	Granulocytes	Nfe2
2.67E-56	-1.1898861	0.045	0.429	3.77E-52	Granulocytes	Tmed9
7.84E-56	1.92994535	0.429	0.188	1.11E-51	Granulocytes	Dgat1
8.66E-56	-1.7815473	0.035	0.402	1.23E-51	Granulocytes	Ctsc
2.45E-55	-1.0044591	0.452	0.821	3.47E-51	Granulocytes	Sub1
3.12E-55	-1.7611289	0.155	0.557	4.41E-51	Granulocytes	Dnajb1
4.53E-55	1.47271528	0.237	0.06	6.41E-51	Granulocytes	4833407H14I
5.00E-55	-1.1484791	0.067	0.447	7.08E-51	Granulocytes	Tbca
1.04E-54	-2.0001284	0.045	0.413	1.47E-50	Granulocytes	S100a4
1.91E-54	-1.7113039	0.035	0.393	2.70E-50	Granulocytes	H2-DMa
3.06E-54	-1.3497176	0.088	0.473	4.33E-50	Granulocytes	Pdia3
1.26E-53	-1.2604487	0.072	0.449	1.79E-49	Granulocytes	Arl6ip5
2.64E-53	-1.1846046	0.067	0.444	3.74E-49	Granulocytes	Atp5g1
4.60E-53	-1.1607391	0.112	0.505	6.51E-49	Granulocytes	Cox7b
7.48E-53	-1.3054877	0.035	0.394	1.06E-48	Granulocytes	Nme1
9.26E-53	1.25370431	0.708	0.517	1.31E-48	Granulocytes	Sat1
1.10E-52	1.5609345	0.174	0.033	1.55E-48	Granulocytes	Csf1
1.31E-52	-1.2618466	0.041	0.398	1.85E-48	Granulocytes	Banf1
1.67E-52	-1.3383866	0.039	0.394	2.36E-48	Granulocytes	Prdx2
1.75E-52	-2.1652774	0.047	0.407	2.48E-48	Granulocytes	Gm26917
2.32E-52	0.87950257	0.129	0.018	3.29E-48	Granulocytes	Arap3

3.63E-52	-1.1212423	0.09	0.475	5.14E-48	Granulocytes	Hnrnpab
4.05E-52	1.19143901	0.221	0.053	5.74E-48	Granulocytes	Cd63
4.77E-52	1.21840467	0.434	0.175	6.75E-48	Granulocytes	Cd24a
5.28E-52	1.71942407	0.503	0.278	7.48E-48	Granulocytes	Card19
5.70E-52	-1.2755628	0.031	0.381	8.07E-48	Granulocytes	Pebp1
8.82E-52	-1.2618962	0.09	0.468	1.25E-47	Granulocytes	H2-Q4
8.83E-52	-1.1814477	0.063	0.434	1.25E-47	Granulocytes	Anp32b
9.74E-52	1.63008733	0.37	0.148	1.38E-47	Granulocytes	Entpd1
1.62E-51	1.66741271	0.417	0.184	2.29E-47	Granulocytes	Kctd12
1.92E-51	-1.542474	0.014	0.356	2.71E-47	Granulocytes	Ikzf3
2.06E-51	-1.1654771	0.053	0.412	2.92E-47	Granulocytes	Ndufb5
2.20E-51	1.67703984	0.62	0.419	3.12E-47	Granulocytes	Plek
2.34E-51	-1.0382782	0.145	0.542	3.32E-47	Granulocytes	Psmb1
4.95E-51	-0.9367711	0.127	0.525	7.00E-47	Granulocytes	Tra2b
5.57E-51	-1.0872768	0.119	0.507	7.88E-47	Granulocytes	Atp5b
7.02E-51	-1.7545369	0.035	0.381	9.93E-47	Granulocytes	Lat
8.14E-51	-1.0076044	0.264	0.669	1.15E-46	Granulocytes	Cox6c
8.78E-51	-1.1855382	0.19	0.589	1.24E-46	Granulocytes	Emp3
1.04E-50	-1.872461	0.025	0.364	1.47E-46	Granulocytes	Sh2d2a
1.29E-50	-1.406032	0.033	0.378	1.83E-46	Granulocytes	Ms4a6b
1.51E-50	-1.2942292	0.035	0.383	2.14E-46	Granulocytes	Dbi
1.66E-50	-0.9837401	0.221	0.63	2.35E-46	Granulocytes	Atp5j2
2.11E-50	-1.2060219	0.07	0.429	2.99E-46	Granulocytes	Rnaset2b
2.23E-50	-1.2353781	0.233	0.629	3.16E-46	Granulocytes	Arl6ip1
2.37E-50	-1.1579536	0.037	0.383	3.36E-46	Granulocytes	Emg1
2.92E-50	-1.7496331	0.039	0.383	4.13E-46	Granulocytes	Hsph1
9.19E-50	-0.9979361	0.331	0.729	1.30E-45	Granulocytes	Pabpc1
1.09E-49	-1.0597465	0.096	0.467	1.54E-45	Granulocytes	Atp5o
1.81E-49	-1.4107867	0.022	0.359	2.56E-45	Granulocytes	Gimap4
1.82E-49	-1.2609953	0.055	0.413	2.58E-45	Granulocytes	Psmb10
2.24E-49	-1.2945448	0.02	0.35	3.17E-45	Granulocytes	Ranbp1
4.37E-49	-1.156313	0.061	0.41	6.18E-45	Granulocytes	Ndufa11
5.20E-49	-1.6252018	0.016	0.344	7.36E-45	Granulocytes	Lck
9.33E-49	-1.1686859	0.045	0.389	1.32E-44	Granulocytes	Eif3c
1.02E-48	-1.1338411	0.039	0.381	1.45E-44	Granulocytes	Manf
1.20E-48	1.48522169	0.673	0.531	1.69E-44	Granulocytes	Prr13
1.76E-48	-1.2936203	0.027	0.355	2.49E-44	Granulocytes	Mndal
2.24E-48	-1.4801769	0.02	0.348	3.17E-44	Granulocytes	Gimap3
4.70E-48	-1.9153466	0.059	0.401	6.65E-44	Granulocytes	Ifi30
6.25E-48	-1.0636788	0.027	0.357	8.85E-44	Granulocytes	Snrpd1
6.36E-48	-1.467562	0.059	0.401	9.00E-44	Granulocytes	Foxp1
7.76E-48	-1.169991	0.016	0.34	1.10E-43	Granulocytes	Akr1b3
7.90E-48	-0.9653949	0.09	0.455	1.12E-43	Granulocytes	Tomm20
8.43E-48	-1.3192036	0.012	0.334	1.19E-43	Granulocytes	Gimap1
1.04E-47	-0.7315701	0.763	0.936	1.48E-43	Granulocytes	B2m
1.07E-47	1.52224252	0.405	0.184	1.51E-43	Granulocytes	Picalm
1.50E-47	-1.0486762	0.053	0.399	2.12E-43	Granulocytes	Eif3m
1.85E-47	-1.8619253	0.055	0.393	2.62E-43	Granulocytes	Rgs1

2.48E-47	-1.9403653	0.02	0.342	3.51E-43	Granulocytes	Ctsw
2.92E-47	-0.976483	0.194	0.586	4.13E-43	Granulocytes	Atp5c1
3.00E-47	-2.4773872	0.043	0.366	4.25E-43	Granulocytes	Cd8b1
3.76E-47	-1.1283436	0.076	0.422	5.32E-43	Granulocytes	Spcs2
6.58E-47	1.64991099	0.35	0.143	9.31E-43	Granulocytes	Snap23
7.25E-47	-1.7122145	0.025	0.344	1.03E-42	Granulocytes	Thy1
7.81E-47	-1.4045237	0.022	0.344	1.11E-42	Granulocytes	Ablim1
1.05E-46	-0.9803555	0.145	0.529	1.49E-42	Granulocytes	Psma3
1.07E-46	-0.8314977	0.509	0.829	1.52E-42	Granulocytes	Chchd2
1.52E-46	-1.1061114	0.1	0.46	2.15E-42	Granulocytes	Tpr
1.67E-46	-1.0966323	0.096	0.444	2.37E-42	Granulocytes	Uqcrfs1
1.84E-46	-0.9102161	0.16	0.543	2.61E-42	Granulocytes	Ube2i
1.92E-46	-1.2689005	0.025	0.347	2.72E-42	Granulocytes	Ifi203
2.03E-46	1.38714167	0.294	0.102	2.88E-42	Granulocytes	Itgam
3.71E-46	-1.1383401	0.055	0.385	5.26E-42	Granulocytes	Dek
3.87E-46	-1.0166708	0.112	0.472	5.48E-42	Granulocytes	Srsf2
4.27E-46	-1.03829	0.092	0.445	6.05E-42	Granulocytes	Hnrnpl
5.04E-46	1.40007351	0.329	0.124	7.13E-42	Granulocytes	Cd300a
5.38E-46	-1.5674037	0.045	0.371	7.62E-42	Granulocytes	Itgb1
7.97E-46	1.76805923	0.348	0.143	1.13E-41	Granulocytes	Cwc25
8.83E-46	1.51500013	0.507	0.285	1.25E-41	Granulocytes	Nfe2l2
1.62E-45	-1.1080913	0.08	0.418	2.29E-41	Granulocytes	Ndufv3
1.77E-45	-1.1636619	0.027	0.342	2.50E-41	Granulocytes	Pole4
3.57E-45	-1.1060025	0.041	0.36	5.06E-41	Granulocytes	Ndufc2
5.80E-45	-1.6416904	0.025	0.337	8.20E-41	Granulocytes	Irf8
6.40E-45	-0.9650779	0.033	0.358	9.06E-41	Granulocytes	Set
7.93E-45	-0.9757546	0.076	0.416	1.12E-40	Granulocytes	Psmb6
8.09E-45	-0.8693457	0.16	0.542	1.14E-40	Granulocytes	Polr1d
1.16E-44	-2.0495981	0.018	0.326	1.64E-40	Granulocytes	Klrd1
1.20E-44	-1.1135392	0.168	0.535	1.69E-40	Granulocytes	Ndfip1
1.24E-44	-1.0814738	0.07	0.41	1.75E-40	Granulocytes	Calr
1.90E-44	-1.0044629	0.121	0.482	2.69E-40	Granulocytes	Ssr4
2.08E-44	-1.0296074	0.286	0.676	2.95E-40	Granulocytes	Hspa5
2.86E-44	-0.9276453	0.123	0.484	4.04E-40	Granulocytes	Elf1
3.89E-44	-2.2202834	0.027	0.331	5.51E-40	Granulocytes	Cd8a
4.25E-44	-1.0111274	0.108	0.453	6.02E-40	Granulocytes	Psmb2
9.89E-44	-1.0842365	0.02	0.326	1.40E-39	Granulocytes	Nt5c
1.10E-43	-0.9095603	0.135	0.495	1.56E-39	Granulocytes	Zfp706
1.52E-43	-0.9026275	0.139	0.503	2.15E-39	Granulocytes	Psma2
2.52E-43	1.4767524	0.254	0.082	3.57E-39	Granulocytes	Oasl2
2.53E-43	-1.0342939	0.057	0.379	3.59E-39	Granulocytes	Snrpd2
2.77E-43	-0.906284	0.235	0.606	3.91E-39	Granulocytes	Slc25a5
6.43E-43	-0.8050765	0.141	0.514	9.10E-39	Granulocytes	Trmt112
7.11E-43	-1.3171485	0.041	0.353	1.01E-38	Granulocytes	Bst2
7.90E-43	-1.2534227	0.168	0.521	1.12E-38	Granulocytes	Ifngr1
8.33E-43	-0.8833078	0.088	0.432	1.18E-38	Granulocytes	Lsm4
1.79E-42	-1.7521585	0.027	0.325	2.54E-38	Granulocytes	Cd83
1.81E-42	-1.1673436	0.043	0.356	2.56E-38	Granulocytes	Macf1

2.68E-42	-1.0111882	0.049	0.365	3.79E-38	Granulocytes	Sec11a
2.83E-42	1.11086577	0.166	0.037	4.01E-38	Granulocytes	Pilrb1
3.80E-42	0.85507436	0.115	0.018	5.38E-38	Granulocytes	Mapk13
7.21E-42	-1.0706751	0.037	0.34	1.02E-37	Granulocytes	Tmem160
7.80E-42	-1.1641313	0.037	0.341	1.10E-37	Granulocytes	Mrps6
8.83E-42	-0.980735	0.082	0.407	1.25E-37	Granulocytes	Nap1l1
1.03E-41	-1.0243963	0.029	0.324	1.46E-37	Granulocytes	Nudc
1.09E-41	-0.9750541	0.09	0.424	1.54E-37	Granulocytes	Park7
1.39E-41	-1.0875023	0.025	0.322	1.97E-37	Granulocytes	Slc25a4
1.77E-41	-1.6537224	0.012	0.299	2.51E-37	Granulocytes	Cst7
1.78E-41	-1.1083773	0.029	0.324	2.52E-37	Granulocytes	Fkbp3
3.94E-41	-1.0548096	0.045	0.347	5.57E-37	Granulocytes	Ppdpf
4.61E-41	-0.9791679	0.043	0.349	6.52E-37	Granulocytes	Ddx39b
5.12E-41	0.5908868	0.955	0.914	7.24E-37	Granulocytes	Cd52
1.11E-40	-0.9829349	0.115	0.453	1.57E-36	Granulocytes	Bsg
1.24E-40	-0.8102404	0.061	0.374	1.75E-36	Granulocytes	Tcea1
1.40E-40	-0.8774758	0.082	0.405	1.99E-36	Granulocytes	Bax
1.58E-40	-0.8589736	0.225	0.598	2.23E-36	Granulocytes	Cd47
1.66E-40	1.46575237	0.548	0.387	2.36E-36	Granulocytes	Atp6v1g1
1.79E-40	-0.6958235	0.141	0.489	2.54E-36	Granulocytes	Dusp2
2.20E-40	-0.7794878	0.325	0.715	3.11E-36	Granulocytes	Hnrnpa2b1
3.62E-40	-1.2699918	0.11	0.437	5.12E-36	Granulocytes	Id2
4.45E-40	-1.4142597	0.037	0.326	6.30E-36	Granulocytes	Ly86
4.48E-40	-1.0185071	0.031	0.322	6.35E-36	Granulocytes	Fbl
4.54E-40	-0.8879917	0.047	0.354	6.43E-36	Granulocytes	Timm13
5.49E-40	-0.9430417	0.104	0.432	7.77E-36	Granulocytes	Brd2
5.65E-40	-1.1043569	0.022	0.308	8.00E-36	Granulocytes	Acp5
6.44E-40	-0.909774	0.055	0.361	9.11E-36	Granulocytes	Sarnp
6.45E-40	1.53171957	0.505	0.308	9.13E-36	Granulocytes	Fosl2
7.70E-40	1.94599432	0.413	0.209	1.09E-35	Granulocytes	Marcksl1
1.08E-39	-0.8147553	0.211	0.574	1.52E-35	Granulocytes	Tma7
1.35E-39	-1.1348364	0.031	0.316	1.91E-35	Granulocytes	Pycard
1.36E-39	-1.2306888	0.033	0.32	1.92E-35	Granulocytes	Gm8369
1.60E-39	-0.8219923	0.297	0.671	2.27E-35	Granulocytes	Ptpn18
1.92E-39	1.35154229	0.56	0.375	2.72E-35	Granulocytes	Samsn1
1.94E-39	-1.2337194	0.014	0.292	2.74E-35	Granulocytes	Hmgn1
2.54E-39	-0.7495086	0.155	0.512	3.59E-35	Granulocytes	Cox7a2l
3.22E-39	-0.8624995	0.086	0.406	4.56E-35	Granulocytes	Pgls
4.21E-39	-1.113795	0.018	0.298	5.96E-35	Granulocytes	Dock10
4.77E-39	-0.9065293	0.049	0.345	6.75E-35	Granulocytes	Phb2
5.09E-39	-0.9865802	0.027	0.308	7.20E-35	Granulocytes	Tcp1
5.59E-39	-0.9852835	0.027	0.309	7.91E-35	Granulocytes	Ndufa5
5.76E-39	1.07640873	0.732	0.611	8.16E-35	Granulocytes	Stk17b
5.77E-39	-0.7550812	0.09	0.414	8.17E-35	Granulocytes	Eif2s2
5.77E-39	-0.8857967	0.043	0.337	8.17E-35	Granulocytes	Bola2
6.45E-39	1.07857899	0.19	0.051	9.13E-35	Granulocytes	Rnf144a
7.89E-39	-1.0057281	0.102	0.423	1.12E-34	Granulocytes	Akr1a1
8.35E-39	-0.8589299	0.204	0.566	1.18E-34	Granulocytes	Edf1

1.03E-38	-1.0150079	0.063	0.363	1.46E-34	Granulocytes	B4galnt1
1.15E-38	-0.9969634	0.104	0.423	1.63E-34	Granulocytes	S100a13
1.17E-38	-0.9330151	0.065	0.366	1.66E-34	Granulocytes	Cct2
1.25E-38	-0.9493588	0.039	0.325	1.77E-34	Granulocytes	Mrpl54
1.44E-38	-1.5615165	0.039	0.323	2.03E-34	Granulocytes	Klf4
2.00E-38	-0.8616226	0.07	0.381	2.84E-34	Granulocytes	Llph
2.10E-38	-1.666016	0.014	0.285	2.97E-34	Granulocytes	Icos
2.13E-38	-0.8921437	0.031	0.314	3.02E-34	Granulocytes	Bcas2
2.17E-38	-0.8711936	0.063	0.369	3.07E-34	Granulocytes	Txn2
3.40E-38	-0.706854	0.143	0.494	4.81E-34	Granulocytes	Sf3b6
3.48E-38	-1.0160506	0.025	0.302	4.92E-34	Granulocytes	Ddx21
4.22E-38	-0.8461437	0.084	0.395	5.97E-34	Granulocytes	Snrpd3
5.39E-38	-0.7178732	0.585	0.864	7.63E-34	Granulocytes	Calm1
5.89E-38	1.26674137	0.249	0.086	8.34E-34	Granulocytes	Igsf6
6.40E-38	-0.7656498	0.168	0.529	9.06E-34	Granulocytes	Srsf3
6.68E-38	-0.8909585	0.063	0.364	9.46E-34	Granulocytes	Ociad1
8.93E-38	-0.7943178	0.239	0.597	1.26E-33	Granulocytes	Atp5j
9.77E-38	-1.6089337	0.02	0.292	1.38E-33	Granulocytes	Tigit
9.99E-38	1.51588056	0.466	0.276	1.41E-33	Granulocytes	Kdm6b
1.00E-37	-0.8886906	0.182	0.525	1.42E-33	Granulocytes	Abracl
1.19E-37	-0.8567044	0.082	0.392	1.69E-33	Granulocytes	Sfr1
1.21E-37	-0.857127	0.174	0.52	1.72E-33	Granulocytes	Psma7
1.40E-37	-1.0223971	0.088	0.397	1.98E-33	Granulocytes	Prrc2c
1.43E-37	-0.9126395	0.045	0.331	2.03E-33	Granulocytes	Nudt21
1.62E-37	-0.7908696	0.147	0.494	2.30E-33	Granulocytes	Srsf7
2.04E-37	-0.9807389	0.022	0.294	2.88E-33	Granulocytes	Tmem256
2.07E-37	-0.9437021	0.047	0.334	2.93E-33	Granulocytes	Psma4
2.74E-37	0.8107602	0.1	0.016	3.88E-33	Granulocytes	B430306N03
2.96E-37	-0.8871047	0.051	0.34	4.19E-33	Granulocytes	Cct5
3.31E-37	-1.1824061	0.029	0.302	4.69E-33	Granulocytes	Il21r
3.34E-37	-0.929282	0.035	0.317	4.73E-33	Granulocytes	Slc50a1
3.35E-37	-0.9378046	0.055	0.343	4.74E-33	Granulocytes	Mettl23
4.21E-37	-0.9751882	0.031	0.304	5.95E-33	Granulocytes	Metap2
4.28E-37	-0.8301264	0.123	0.446	6.06E-33	Granulocytes	Tuba1a
5.26E-37	-1.8359346	0.022	0.291	7.45E-33	Granulocytes	Pdcd1
5.43E-37	-1.5532626	0.014	0.278	7.68E-33	Granulocytes	Il2rb
6.20E-37	-0.8856102	0.01	0.274	8.78E-33	Granulocytes	Churc1
6.91E-37	-1.044579	0.025	0.292	9.78E-33	Granulocytes	Smc6
9.23E-37	-1.022031	0.047	0.328	1.31E-32	Granulocytes	Mat2a
9.87E-37	-0.808527	0.09	0.396	1.40E-32	Granulocytes	Cwc15
9.96E-37	-0.9628877	0.043	0.327	1.41E-32	Granulocytes	Atp1a1
1.06E-36	-0.8187476	0.11	0.421	1.51E-32	Granulocytes	Hnrnpdl
1.63E-36	-0.9160804	0.141	0.466	2.31E-32	Granulocytes	Tubb4b
1.64E-36	-0.8794616	0.041	0.321	2.33E-32	Granulocytes	Ssr2
1.76E-36	1.44902946	0.329	0.148	2.50E-32	Granulocytes	Pgd
2.25E-36	1.17430193	0.209	0.064	3.18E-32	Granulocytes	Tmem154
2.58E-36	-0.7557488	0.106	0.428	3.66E-32	Granulocytes	Skp1a
2.74E-36	-0.8273094	0.078	0.378	3.88E-32	Granulocytes	H2afy

3.54E-36	0.76776718	0.834	0.778	5.01E-32	Granulocytes	Arpc1b
4.07E-36	-0.6903989	0.168	0.514	5.76E-32	Granulocytes	Ndufb11
4.23E-36	-0.8200863	0.041	0.318	5.99E-32	Granulocytes	Psmc3
4.38E-36	1.36489931	0.27	0.101	6.20E-32	Granulocytes	Hist1h2bc
5.51E-36	-0.8904527	0.033	0.3	7.80E-32	Granulocytes	Ddost
5.63E-36	-0.7883959	0.102	0.416	7.97E-32	Granulocytes	Bzw1
5.84E-36	-0.791234	0.303	0.65	8.27E-32	Granulocytes	Atp5h
6.57E-36	-0.8695094	0.078	0.373	9.31E-32	Granulocytes	Ostc
7.19E-36	-2.3194299	0.035	0.298	1.02E-31	Granulocytes	Ccr7
8.82E-36	-0.9690797	0.012	0.269	1.25E-31	Granulocytes	Fundc2
1.25E-35	-0.7380742	0.067	0.362	1.77E-31	Granulocytes	Pdcd5
1.33E-35	1.03317362	0.64	0.495	1.89E-31	Granulocytes	Selplg
1.40E-35	1.19128148	0.247	0.088	1.98E-31	Granulocytes	Lpcat2
1.45E-35	-0.8549116	0.037	0.307	2.06E-31	Granulocytes	Cox16
1.51E-35	-0.8512505	0.074	0.364	2.13E-31	Granulocytes	Ssb
1.52E-35	-0.7716612	0.108	0.42	2.16E-31	Granulocytes	Med28
1.68E-35	-0.6635469	0.1	0.411	2.38E-31	Granulocytes	Ndufb10
1.71E-35	-0.8021056	0.059	0.344	2.42E-31	Granulocytes	Uqcrc1
1.99E-35	-0.724509	0.178	0.524	2.82E-31	Granulocytes	Pcbp1
2.87E-35	-0.8010036	0.049	0.327	4.06E-31	Granulocytes	Ube2v1
3.41E-35	-0.8066846	0.063	0.351	4.83E-31	Granulocytes	Wdr83os
3.58E-35	-0.8318164	0.164	0.493	5.07E-31	Granulocytes	Gdi2
4.23E-35	-0.7440448	0.084	0.381	5.98E-31	Granulocytes	Ptp4a2
4.94E-35	-0.8050499	0.074	0.364	6.99E-31	Granulocytes	Ywhaq
5.45E-35	-0.7640591	0.076	0.374	7.72E-31	Granulocytes	Psmb4
5.71E-35	1.2143127	0.364	0.177	8.08E-31	Granulocytes	Csf2ra
5.83E-35	-0.7938796	0.047	0.325	8.26E-31	Granulocytes	Timm23
6.81E-35	1.45060376	0.342	0.161	9.64E-31	Granulocytes	Ncf1
7.73E-35	-1.3124873	0.02	0.277	1.09E-30	Granulocytes	Itk
8.18E-35	0.92715329	0.147	0.035	1.16E-30	Granulocytes	Dgat2
9.23E-35	1.27961049	0.209	0.067	1.31E-30	Granulocytes	Tctex1d2
9.74E-35	0.52228161	0.922	0.858	1.38E-30	Granulocytes	H3f3a
1.21E-34	0.80878213	0.671	0.526	1.72E-30	Granulocytes	Hmgb2
1.39E-34	-0.9458643	0.088	0.381	1.96E-30	Granulocytes	Saraf
1.41E-34	-2.3351661	0.008	0.254	2.00E-30	Granulocytes	Gzmb
1.44E-34	-0.9155414	0.029	0.291	2.04E-30	Granulocytes	Ptbp1
1.66E-34	-0.7560538	0.317	0.66	2.35E-30	Granulocytes	Cox5b
1.68E-34	-1.1008705	0.02	0.276	2.38E-30	Granulocytes	Skap1
1.69E-34	1.17997392	0.673	0.621	2.39E-30	Granulocytes	D8Ertd738e
2.11E-34	-1.0616454	0.016	0.269	2.98E-30	Granulocytes	Zgpat
2.15E-34	-0.8324774	0.043	0.312	3.04E-30	Granulocytes	Mrps14
2.23E-34	0.93877786	0.121	0.024	3.15E-30	Granulocytes	Il15
2.61E-34	-0.6823163	0.108	0.418	3.69E-30	Granulocytes	Psmd8
2.76E-34	-0.8317278	0.07	0.348	3.90E-30	Granulocytes	Romo1
3.23E-34	-0.7499192	0.172	0.502	4.57E-30	Granulocytes	Rnaset2a
3.68E-34	-0.7966534	0.088	0.384	5.21E-30	Granulocytes	Ddx24
4.46E-34	-0.8914953	0.02	0.272	6.31E-30	Granulocytes	Pa2g4
5.33E-34	-0.8197151	0.027	0.284	7.54E-30	Granulocytes	Idh3b

6.31E-34	-0.7903751	0.065	0.345	8.94E-30	Granulocytes	Vcp
7.30E-34	-0.9322987	0.084	0.37	1.03E-29	Granulocytes	Crem
7.45E-34	-0.702232	0.213	0.548	1.05E-29	Granulocytes	Hnrnpf
8.63E-34	0.96163034	0.127	0.027	1.22E-29	Granulocytes	Gm14548
9.98E-34	-0.8444514	0.016	0.265	1.41E-29	Granulocytes	Lsm5
1.24E-33	-0.9760889	0.078	0.358	1.76E-29	Granulocytes	mt-Nd3
1.33E-33	-0.8069109	0.072	0.348	1.88E-29	Granulocytes	Cct7
1.41E-33	-0.8109134	0.043	0.304	2.00E-29	Granulocytes	Mrpl23
1.49E-33	-1.7701658	0.01	0.252	2.11E-29	Granulocytes	Ctla4
1.56E-33	-0.827235	0.119	0.417	2.21E-29	Granulocytes	Cope
1.58E-33	-0.7667938	0.047	0.314	2.24E-29	Granulocytes	Tsn
1.70E-33	-0.8675099	0.043	0.305	2.41E-29	Granulocytes	Cyc1
1.75E-33	-1.2463523	0.022	0.272	2.48E-29	Granulocytes	Tnfrsf18
1.87E-33	-1.0996448	0.102	0.393	2.64E-29	Granulocytes	Rel
1.94E-33	-0.6979302	0.051	0.322	2.75E-29	Granulocytes	Ndufv2
1.96E-33	-0.9188506	0.027	0.28	2.78E-29	Granulocytes	Esyt1
1.98E-33	-0.7906419	0.07	0.348	2.80E-29	Granulocytes	Cacybp
2.09E-33	-1.1934349	0.01	0.251	2.96E-29	Granulocytes	Gimap7
2.12E-33	-0.8146845	0.072	0.348	3.00E-29	Granulocytes	Cdc37
2.64E-33	-0.8745457	0.057	0.329	3.73E-29	Granulocytes	Dnajc3
2.73E-33	-0.7351391	0.168	0.488	3.86E-29	Granulocytes	Pomp
2.97E-33	1.32403542	0.247	0.091	4.21E-29	Granulocytes	Gadd45a
3.26E-33	-0.8270901	0.049	0.313	4.61E-29	Granulocytes	Ndufc1
3.43E-33	-0.8819655	0.025	0.275	4.86E-29	Granulocytes	Anp32e
4.23E-33	-0.8105304	0.086	0.373	5.98E-29	Granulocytes	Rps27l
4.44E-33	-0.7410943	0.188	0.52	6.29E-29	Granulocytes	Eif4g2
4.58E-33	0.74147203	0.873	0.782	6.48E-29	Granulocytes	Btg1
5.58E-33	-1.0585207	0.022	0.269	7.90E-29	Granulocytes	Gimap5
5.85E-33	0.84558957	0.783	0.763	8.28E-29	Granulocytes	Arpc3
6.46E-33	-0.6424329	0.157	0.479	9.14E-29	Granulocytes	Tmem258
6.92E-33	-1.9631148	0.02	0.263	9.80E-29	Granulocytes	Cd79b
7.04E-33	-1.0645738	0.051	0.311	9.96E-29	Granulocytes	Anxa5
7.83E-33	-0.8779184	0.014	0.256	1.11E-28	Granulocytes	C1qbp
7.84E-33	-0.8189029	0.033	0.284	1.11E-28	Granulocytes	Emc10
7.91E-33	-0.8349256	0.09	0.376	1.12E-28	Granulocytes	Aprt
9.70E-33	-0.8889284	0.039	0.295	1.37E-28	Granulocytes	Khdrbs1
1.04E-32	-0.7860258	0.049	0.313	1.47E-28	Granulocytes	Ndufb2
1.18E-32	-0.8805625	0.074	0.346	1.67E-28	Granulocytes	Syngr2
1.29E-32	-0.7667122	0.215	0.537	1.83E-28	Granulocytes	Psmb3
1.30E-32	-0.8273477	0.027	0.275	1.84E-28	Granulocytes	Rexo2
1.36E-32	1.42690722	0.323	0.151	1.93E-28	Granulocytes	Marcks
1.41E-32	0.83898132	0.748	0.697	2.00E-28	Granulocytes	Gng5
1.46E-32	-1.1538234	0.006	0.241	2.06E-28	Granulocytes	Dut
1.57E-32	-0.6807915	0.082	0.361	2.23E-28	Granulocytes	Mrpl30
1.73E-32	-1.1183026	0.014	0.253	2.45E-28	Granulocytes	Prkca
1.87E-32	-0.8250656	0.029	0.275	2.65E-28	Granulocytes	Glrx3
2.53E-32	-1.4602148	0.02	0.26	3.59E-28	Granulocytes	H2-DMb1
2.55E-32	1.11607574	0.648	0.569	3.61E-28	Granulocytes	Ypel3

2.87E-32	-0.773869	0.033	0.284	4.07E-28	Granulocytes	Cct8
3.07E-32	-1.2118597	0.018	0.258	4.35E-28	Granulocytes	Cd28
3.18E-32	-0.9034329	0.039	0.292	4.51E-28	Granulocytes	Ak2
3.54E-32	-0.7998098	0.043	0.301	5.01E-28	Granulocytes	Mrps16
3.80E-32	-1.0145836	0.149	0.446	5.38E-28	Granulocytes	Sdf4
3.83E-32	-0.8507757	0.098	0.384	5.42E-28	Granulocytes	H2-T22
4.62E-32	-0.7386538	0.094	0.383	6.54E-28	Granulocytes	U2af1
5.02E-32	3.26680568	0.411	0.232	7.10E-28	Granulocytes	Wfdc17
5.98E-32	-0.6253628	0.209	0.544	8.46E-28	Granulocytes	Arhgdia
6.21E-32	-0.7888337	0.047	0.302	8.79E-28	Granulocytes	Cops6
6.39E-32	-0.7126673	0.119	0.419	9.05E-28	Granulocytes	Ndufb8
8.20E-32	-1.459666	0.17	0.459	1.16E-27	Granulocytes	Ctsb
8.35E-32	-0.8150079	0.039	0.289	1.18E-27	Granulocytes	Eif4b
8.85E-32	-0.8739886	0.033	0.278	1.25E-27	Granulocytes	Xrn2
9.07E-32	-0.7040628	0.078	0.354	1.28E-27	Granulocytes	Cuta
9.45E-32	-1.2112838	0.016	0.251	1.34E-27	Granulocytes	Pld4
9.96E-32	0.32021888	1	0.99	1.41E-27	Granulocytes	Actb
1.00E-31	1.20334041	0.211	0.072	1.42E-27	Granulocytes	Tcn2
1.11E-31	-1.2099157	0.014	0.247	1.57E-27	Granulocytes	Cd27
1.21E-31	-0.7934464	0.037	0.29	1.72E-27	Granulocytes	Mif4gd
1.24E-31	-1.05036	0.035	0.281	1.76E-27	Granulocytes	Ptpn22
1.38E-31	-0.4719082	0.106	0.394	1.96E-27	Granulocytes	2-Mar
1.40E-31	-0.7260952	0.041	0.296	1.98E-27	Granulocytes	Eif5b
1.41E-31	-0.5666302	0.137	0.443	2.00E-27	Granulocytes	Ywhah
1.42E-31	-0.7805237	0.139	0.439	2.02E-27	Granulocytes	Spcs1
1.83E-31	-0.8132684	0.035	0.279	2.59E-27	Granulocytes	Aimp1
1.96E-31	-0.890643	0.031	0.271	2.77E-27	Granulocytes	Npm3
2.13E-31	-0.684846	0.133	0.435	3.02E-27	Granulocytes	Atp5k
2.51E-31	-1.1382213	0.018	0.253	3.55E-27	Granulocytes	P2ry10
2.55E-31	-0.7108348	0.053	0.316	3.61E-27	Granulocytes	Psma5
2.63E-31	-0.7224226	0.149	0.453	3.73E-27	Granulocytes	Arf6
2.66E-31	-0.8173473	0.033	0.274	3.77E-27	Granulocytes	Lsm7
2.98E-31	-0.9874398	0.012	0.242	4.22E-27	Granulocytes	Cblb
2.99E-31	1.1888174	0.245	0.095	4.23E-27	Granulocytes	Gng12
3.15E-31	-0.7418852	0.082	0.356	4.46E-27	Granulocytes	Ahsa1
3.63E-31	-1.0885922	0.016	0.247	5.14E-27	Granulocytes	Slamf7
4.02E-31	-0.5785936	0.08	0.355	5.68E-27	Granulocytes	Ghitm
4.10E-31	-0.7557475	0.043	0.296	5.81E-27	Granulocytes	Ndufs3
4.20E-31	1.04606215	0.153	0.041	5.95E-27	Granulocytes	Tarm1
4.42E-31	-0.9032333	0.018	0.251	6.25E-27	Granulocytes	Dhx9
4.92E-31	-0.6002292	0.211	0.541	6.97E-27	Granulocytes	Hnrnpk
5.07E-31	-0.7890324	0.027	0.265	7.18E-27	Granulocytes	Dnajc19
5.08E-31	1.67774284	0.427	0.268	7.20E-27	Granulocytes	Csrnp1
6.55E-31	-0.7007053	0.072	0.34	9.27E-27	Granulocytes	Eif3a
8.93E-31	-1.4780653	0.01	0.236	1.26E-26	Granulocytes	Lag3
1.16E-30	0.86745931	0.123	0.028	1.65E-26	Granulocytes	6430548M08
1.30E-30	-0.7561099	0.045	0.296	1.84E-26	Granulocytes	St13
1.32E-30	-0.656881	0.057	0.316	1.87E-26	Granulocytes	Emc7

1.33E-30	0.72322999	0.804	0.764	1.88E-26	Granulocytes	Myl12b
1.36E-30	-0.7877862	0.037	0.277	1.93E-26	Granulocytes	Sec13
1.57E-30	-0.6527104	0.458	0.791	2.22E-26	Granulocytes	Rbm39
1.62E-30	-0.62283	0.17	0.485	2.29E-26	Granulocytes	Sec62
1.71E-30	-0.8083942	0.041	0.281	2.43E-26	Granulocytes	Uqcc2
1.75E-30	-0.6444858	0.108	0.392	2.48E-26	Granulocytes	Swi5
1.96E-30	-0.8602367	0.031	0.269	2.77E-26	Granulocytes	Pdia6
2.03E-30	-1.4077064	0.02	0.248	2.87E-26	Granulocytes	Mef2c
2.22E-30	1.4353042	0.303	0.139	3.15E-26	Granulocytes	Myadm
2.26E-30	-0.7856197	0.02	0.252	3.19E-26	Granulocytes	Eny2
2.43E-30	-0.8883185	0.027	0.26	3.44E-26	Granulocytes	Runx3
2.97E-30	-0.9168198	0.029	0.264	4.20E-26	Granulocytes	Rabgap1l
3.09E-30	2.08990587	0.458	0.289	4.37E-26	Granulocytes	Isg15
3.14E-30	-1.1228901	0.031	0.264	4.45E-26	Granulocytes	Prkch
3.34E-30	-0.609901	0.092	0.371	4.73E-26	Granulocytes	Mrps24
3.35E-30	-2.6615891	0.033	0.261	4.74E-26	Granulocytes	Cd79a
3.70E-30	-1.1147051	0.006	0.227	5.23E-26	Granulocytes	Gpr171
4.94E-30	-0.729132	0.115	0.393	6.99E-26	Granulocytes	Srsf11
5.03E-30	-0.8907584	0.018	0.244	7.12E-26	Granulocytes	Phf11b
5.15E-30	-0.771566	0.047	0.292	7.30E-26	Granulocytes	Ndufab1
5.48E-30	-0.692114	0.137	0.424	7.76E-26	Granulocytes	Sdhb
5.79E-30	-1.6863899	0.025	0.251	8.19E-26	Granulocytes	H2-DMb2
5.91E-30	-0.7331839	0.012	0.235	8.37E-26	Granulocytes	Bola3
7.09E-30	-1.4852358	0.008	0.227	1.00E-25	Granulocytes	Cxcr6
9.40E-30	-0.7696525	0.047	0.295	1.33E-25	Granulocytes	Dnajc15
9.95E-30	1.09316447	0.245	0.098	1.41E-25	Granulocytes	Hist2h2aa1
1.13E-29	-0.6532497	0.129	0.418	1.61E-25	Granulocytes	Vdac2
1.14E-29	-0.8080296	0.031	0.259	1.61E-25	Granulocytes	Prmt1
1.27E-29	-0.5995873	0.121	0.406	1.80E-25	Granulocytes	Esd
1.44E-29	-1.1993876	0.02	0.247	2.04E-25	Granulocytes	Gem
1.49E-29	-0.7266621	0.045	0.287	2.11E-25	Granulocytes	Commd4
1.66E-29	-0.731876	0.155	0.451	2.35E-25	Granulocytes	Tgfb1
1.80E-29	-0.777592	0.022	0.248	2.54E-25	Granulocytes	Imp3
1.82E-29	-0.6802726	0.055	0.303	2.57E-25	Granulocytes	Hypk
1.89E-29	-0.899541	0.434	0.726	2.67E-25	Granulocytes	H2afz
2.07E-29	-0.7343955	0.072	0.328	2.92E-25	Granulocytes	Smarca5
2.17E-29	-1.5093153	0.018	0.24	3.07E-25	Granulocytes	Ms4a6c
2.55E-29	-2.0124279	0.016	0.235	3.62E-25	Granulocytes	Ebf1
2.57E-29	-1.0317799	0.016	0.236	3.64E-25	Granulocytes	Cd247
2.70E-29	-0.5590869	0.487	0.78	3.83E-25	Granulocytes	Cox4i1
2.80E-29	-0.6148499	0.053	0.301	3.97E-25	Granulocytes	Mrps33
2.82E-29	-0.7291271	0.051	0.295	3.99E-25	Granulocytes	Cox20
3.44E-29	-0.5826168	0.182	0.496	4.87E-25	Granulocytes	Srp9
3.66E-29	-0.3431754	0.223	0.558	5.18E-25	Granulocytes	Sap18
3.90E-29	-0.6597584	0.147	0.443	5.51E-25	Granulocytes	Ube2s
4.35E-29	-1.003499	0.137	0.409	6.16E-25	Granulocytes	Capg
5.01E-29	1.1895729	0.569	0.47	7.09E-25	Granulocytes	Gpsm3
5.19E-29	-0.5931266	0.194	0.517	7.35E-25	Granulocytes	Ndufa6

5.36E-29	1.23762933	0.288	0.129	7.58E-25	Granulocytes	Sirpa
5.45E-29	-0.7226667	0.1	0.372	7.72E-25	Granulocytes	Rbm25
6.18E-29	0.4676026	0.808	0.676	8.74E-25	Granulocytes	Gpx1
6.72E-29	1.20235237	0.233	0.091	9.52E-25	Granulocytes	Tmcc1
6.96E-29	-0.7330462	0.039	0.271	9.85E-25	Granulocytes	Psmc5
7.32E-29	-0.8535539	0.057	0.306	1.04E-24	Granulocytes	Ccnd2
7.75E-29	-0.6245643	0.07	0.323	1.10E-24	Granulocytes	Ndufs4
9.00E-29	-0.6510264	0.249	0.569	1.27E-24	Granulocytes	Cox6a1
1.05E-28	-0.6694063	0.127	0.413	1.48E-24	Granulocytes	H2afv
1.11E-28	-0.7390128	0.051	0.292	1.57E-24	Granulocytes	Srp19
1.12E-28	-0.5792682	0.172	0.481	1.59E-24	Granulocytes	1810037I17R
1.26E-28	-0.6929638	0.037	0.27	1.78E-24	Granulocytes	Ergic3
1.33E-28	-0.8470282	0.035	0.265	1.88E-24	Granulocytes	Plekha2
1.46E-28	-0.7367063	0.043	0.277	2.07E-24	Granulocytes	Cct4
1.47E-28	-0.7719676	0.006	0.216	2.07E-24	Granulocytes	Phb
1.64E-28	-0.647835	0.123	0.404	2.32E-24	Granulocytes	Hspa4
2.00E-28	-0.6904446	0.104	0.37	2.83E-24	Granulocytes	Ywhae
2.22E-28	-0.6270939	0.237	0.556	3.15E-24	Granulocytes	Atp5f1
2.30E-28	-0.7684266	0.016	0.231	3.25E-24	Granulocytes	Mrps36
2.31E-28	1.33277276	0.397	0.238	3.28E-24	Granulocytes	AB124611
2.94E-28	-0.610802	0.139	0.426	4.16E-24	Granulocytes	Raly
2.96E-28	-0.7059479	0.053	0.293	4.19E-24	Granulocytes	Dcun1d5
2.99E-28	-0.9274969	0.012	0.223	4.23E-24	Granulocytes	Pkig
3.10E-28	-0.6768584	0.01	0.221	4.39E-24	Granulocytes	Stard3nl
3.67E-28	-0.9652183	0.025	0.244	5.20E-24	Granulocytes	Ptp4a3
3.74E-28	-0.7857241	0.004	0.21	5.29E-24	Granulocytes	Bin1
4.03E-28	1.27834813	0.217	0.082	5.70E-24	Granulocytes	Ogfrl1
4.18E-28	1.05160615	0.188	0.064	5.92E-24	Granulocytes	Msra
4.19E-28	-0.6986888	0.043	0.274	5.93E-24	Granulocytes	Lsm6
4.52E-28	-0.7301557	0.055	0.293	6.39E-24	Granulocytes	Canx
4.69E-28	-0.6665415	0.035	0.26	6.63E-24	Granulocytes	Ndufa12
5.29E-28	-1.050064	0.047	0.279	7.49E-24	Granulocytes	Bhlhe40
5.47E-28	1.16334134	0.593	0.461	7.74E-24	Granulocytes	Ctsd
6.28E-28	-0.8668057	0.029	0.249	8.88E-24	Granulocytes	Rhoh
6.41E-28	-0.7564101	0.016	0.227	9.07E-24	Granulocytes	Mrpl12
6.47E-28	-0.6029712	0.067	0.317	9.15E-24	Granulocytes	Mrpl4
6.59E-28	-0.5486908	0.096	0.365	9.33E-24	Granulocytes	Higd1a
7.01E-28	-0.7058317	0.033	0.258	9.93E-24	Granulocytes	1110004F10R
7.65E-28	-0.5726169	0.092	0.354	1.08E-23	Granulocytes	Dpm3
8.81E-28	-0.6847229	0.098	0.361	1.25E-23	Granulocytes	Acin1
8.86E-28	-0.8851324	0.025	0.24	1.25E-23	Granulocytes	Slc1a5
9.96E-28	-0.6891061	0.037	0.266	1.41E-23	Granulocytes	Thrap3
1.05E-27	-0.739601	0.029	0.248	1.48E-23	Granulocytes	Mrpl42
1.05E-27	-0.6941736	0.022	0.239	1.48E-23	Granulocytes	Naa38
1.06E-27	-0.9510313	0.031	0.248	1.50E-23	Granulocytes	Camk2d
1.07E-27	0.89576157	0.732	0.715	1.51E-23	Granulocytes	Pnrc1
1.27E-27	-0.6171696	0.084	0.339	1.79E-23	Granulocytes	Rwdd1
1.43E-27	-0.7783903	0.012	0.221	2.03E-23	Granulocytes	Sms

1.83E-27	-0.6668847	0.051	0.286	2.60E-23	Granulocytes	Ddrgk1
1.89E-27	-0.7789986	0.01	0.215	2.67E-23	Granulocytes	H2-M3
2.53E-27	-0.7363513	0.02	0.231	3.59E-23	Granulocytes	Ak6
2.65E-27	-0.760821	0.072	0.316	3.75E-23	Granulocytes	4930523C07R
2.77E-27	-0.6700668	0.053	0.288	3.93E-23	Granulocytes	Ndufs8
3.51E-27	-0.6020654	0.088	0.342	4.97E-23	Granulocytes	Lman2
3.54E-27	-0.6579573	0.065	0.305	5.01E-23	Granulocytes	Eif6
3.61E-27	-0.7336723	0.084	0.33	5.11E-23	Granulocytes	Hnrnph1
4.06E-27	-0.5517211	0.106	0.375	5.75E-23	Granulocytes	Csnk1a1
4.63E-27	-0.7279233	0.022	0.233	6.55E-23	Granulocytes	Rpn2
4.89E-27	-0.7131945	0.047	0.275	6.92E-23	Granulocytes	Tram1
4.97E-27	-0.8018012	0.02	0.23	7.03E-23	Granulocytes	Stip1
4.99E-27	-0.6803291	0.119	0.384	7.06E-23	Granulocytes	Atp1b3
5.28E-27	-0.8657115	0.049	0.277	7.48E-23	Granulocytes	Smad7
5.32E-27	1.11951989	0.198	0.072	7.53E-23	Granulocytes	Rnf11
5.61E-27	-0.8245806	0.014	0.22	7.95E-23	Granulocytes	Ehd4
5.87E-27	1.23418378	0.587	0.47	8.31E-23	Granulocytes	Pde4b
6.48E-27	-0.7354101	0.031	0.247	9.17E-23	Granulocytes	Nhp2
6.85E-27	-0.7151013	0.049	0.28	9.70E-23	Granulocytes	Lbh
7.59E-27	-1.3514015	0.016	0.222	1.07E-22	Granulocytes	H2-Ob
8.36E-27	-0.7792711	0.006	0.204	1.18E-22	Granulocytes	Fyn
8.78E-27	-0.6759792	0.02	0.229	1.24E-22	Granulocytes	Pfdn1
9.25E-27	-0.6810967	0.035	0.254	1.31E-22	Granulocytes	Mtch2
9.31E-27	-0.7122658	0.014	0.217	1.32E-22	Granulocytes	Nubp1
1.10E-26	-0.617553	0.151	0.436	1.55E-22	Granulocytes	Psmb5
1.16E-26	-0.7146636	0.016	0.22	1.64E-22	Granulocytes	Exosc8
1.17E-26	-0.7566452	0.02	0.225	1.66E-22	Granulocytes	Dnajc2
1.29E-26	-0.7941476	0.049	0.274	1.82E-22	Granulocytes	Snx2
1.29E-26	-0.9456697	0.016	0.219	1.83E-22	Granulocytes	Foxo1
1.46E-26	-0.9110902	0.016	0.22	2.06E-22	Granulocytes	Bcl2
1.58E-26	-0.6533118	0.025	0.234	2.24E-22	Granulocytes	Ndufa10
1.70E-26	-0.7468602	0.051	0.279	2.40E-22	Granulocytes	Vgll4
1.82E-26	-0.7011299	0.029	0.242	2.58E-22	Granulocytes	Med10
1.89E-26	-0.6203418	0.098	0.356	2.67E-22	Granulocytes	Mtdh
1.90E-26	-0.6770377	0.016	0.221	2.69E-22	Granulocytes	Impdh2
1.95E-26	-0.5847177	0.082	0.329	2.76E-22	Granulocytes	Ptges3
2.03E-26	1.15694592	0.438	0.27	2.88E-22	Granulocytes	Fgl2
2.07E-26	-2.2713229	0.022	0.226	2.92E-22	Granulocytes	Ly6d
2.17E-26	-0.7476691	0.027	0.237	3.08E-22	Granulocytes	U2surp
2.23E-26	-0.8210187	0.037	0.255	3.15E-22	Granulocytes	H2afx
2.30E-26	-0.7085157	0.061	0.292	3.25E-22	Granulocytes	Ppig
2.37E-26	-0.6860675	0.057	0.286	3.36E-22	Granulocytes	Cyb5a
2.39E-26	-0.7446637	0.018	0.224	3.39E-22	Granulocytes	Eif4g1
2.46E-26	-0.7101333	0.051	0.279	3.48E-22	Granulocytes	Tob2
2.90E-26	-0.6030514	0.053	0.284	4.10E-22	Granulocytes	Phf5a
2.95E-26	-0.7623007	0.02	0.227	4.18E-22	Granulocytes	Nop56
3.22E-26	-0.6748034	0.018	0.223	4.56E-22	Granulocytes	Adh5
3.26E-26	-0.6415628	0.047	0.268	4.61E-22	Granulocytes	Adrm1

3.33E-26	-0.5206533	0.188	0.489	4.72E-22	Granulocytes	Snx3
3.85E-26	-0.7974014	0.055	0.282	5.45E-22	Granulocytes	Clic4
3.91E-26	-0.6375471	0.057	0.287	5.54E-22	Granulocytes	Arpc5l
4.07E-26	-0.8375523	0.027	0.233	5.76E-22	Granulocytes	Fubp1
4.19E-26	-0.7872853	0.016	0.218	5.93E-22	Granulocytes	Got1
4.47E-26	-0.6848243	0.018	0.221	6.32E-22	Granulocytes	Cct3
4.48E-26	-0.6287653	0.043	0.262	6.34E-22	Granulocytes	Emc6
4.56E-26	-0.7541668	0.012	0.212	6.45E-22	Granulocytes	Cnp
4.68E-26	-0.4716202	0.157	0.449	6.63E-22	Granulocytes	Aup1
4.80E-26	-1.2257024	0.119	0.362	6.79E-22	Granulocytes	Plac8
4.94E-26	-0.7335261	0.047	0.269	7.00E-22	Granulocytes	Apobec3
5.48E-26	-0.580692	0.057	0.287	7.76E-22	Granulocytes	Eif4e2
6.08E-26	-0.7246858	0.045	0.265	8.61E-22	Granulocytes	Taf1d
7.62E-26	-0.5745753	0.09	0.339	1.08E-21	Granulocytes	Ndufb6
8.13E-26	-0.7165908	0.051	0.272	1.15E-21	Granulocytes	Mrpl18
8.16E-26	1.03215765	0.194	0.069	1.15E-21	Granulocytes	Gm9733
8.41E-26	-0.7453712	0.022	0.227	1.19E-21	Granulocytes	Srpk1
8.87E-26	-0.7186834	0.027	0.233	1.26E-21	Granulocytes	Gm9844
1.06E-25	-0.7727776	0.008	0.202	1.50E-21	Granulocytes	Pld3
1.15E-25	-0.6392817	0.237	0.543	1.63E-21	Granulocytes	Reep5
1.23E-25	-0.6402944	0.022	0.227	1.75E-21	Granulocytes	Timm17a
1.29E-25	-0.7109071	0.053	0.276	1.82E-21	Granulocytes	Tbcb
1.37E-25	-1.3432916	0.016	0.215	1.95E-21	Granulocytes	Ctla2a
1.48E-25	-0.8797633	0.018	0.216	2.09E-21	Granulocytes	Chd3
1.49E-25	-0.6648339	0.006	0.197	2.11E-21	Granulocytes	Eif2s1
1.51E-25	-0.546683	0.115	0.38	2.14E-21	Granulocytes	Polr2l
1.61E-25	-0.5965204	0.059	0.289	2.28E-21	Granulocytes	Eif4a3
1.67E-25	-0.5359036	0.108	0.369	2.36E-21	Granulocytes	Bcap31
1.75E-25	-0.6216215	0.02	0.22	2.48E-21	Granulocytes	Snrpb2
1.75E-25	0.92123367	0.1	0.022	2.48E-21	Granulocytes	Tnfrsf23
1.78E-25	-0.8734411	0.112	0.359	2.53E-21	Granulocytes	Tpi1
1.81E-25	-0.5756685	0.08	0.323	2.57E-21	Granulocytes	Psma6
1.87E-25	-0.5343918	0.701	0.89	2.65E-21	Granulocytes	Oaz1
1.91E-25	-0.5782573	0.076	0.31	2.71E-21	Granulocytes	Zc3h15
1.93E-25	-0.6724474	0.016	0.212	2.73E-21	Granulocytes	Mrpl28
1.96E-25	0.87572999	0.153	0.048	2.77E-21	Granulocytes	Bst1
2.04E-25	-1.6226338	0.01	0.201	2.89E-21	Granulocytes	Ifng
2.30E-25	-0.6028344	0.153	0.434	3.26E-21	Granulocytes	Rps27rt
2.58E-25	-0.8292352	0.014	0.207	3.66E-21	Granulocytes	Sdf2l1
2.68E-25	-0.5502375	0.057	0.285	3.79E-21	Granulocytes	Ndufb3
2.69E-25	-0.7569191	0.02	0.218	3.81E-21	Granulocytes	Dnajc9
2.75E-25	-1.2196784	0.02	0.216	3.90E-21	Granulocytes	S1pr1
2.79E-25	-0.7140507	0.016	0.214	3.95E-21	Granulocytes	Lat2
3.08E-25	-0.6482151	0.014	0.209	4.36E-21	Granulocytes	Bccip
3.56E-25	-0.6353315	0.137	0.401	5.04E-21	Granulocytes	Laptm4a
3.94E-25	-0.6300077	0.01	0.202	5.57E-21	Granulocytes	Ddt
4.28E-25	-0.5374265	0.084	0.327	6.06E-21	Granulocytes	Rnf187
4.52E-25	1.33818037	0.423	0.289	6.40E-21	Granulocytes	Fam32a

4.91E-25	1.24300511	0.268	0.126	6.95E-21	Granulocytes	Fem1c
4.97E-25	-0.5058957	0.076	0.313	7.03E-21	Granulocytes	Zcrb1
5.18E-25	-0.8266513	0.018	0.213	7.34E-21	Granulocytes	Spn
5.59E-25	-0.6832882	0.053	0.272	7.92E-21	Granulocytes	Itgb7
5.62E-25	1.26745066	0.442	0.296	7.95E-21	Granulocytes	Gpcpd1
5.66E-25	-0.7584289	0.055	0.277	8.02E-21	Granulocytes	Socs1
5.72E-25	2.50583352	0.292	0.149	8.10E-21	Granulocytes	Ccrl2
6.35E-25	-0.5198823	0.198	0.481	8.99E-21	Granulocytes	Kmt2e
6.37E-25	-1.3326042	0.002	0.186	9.02E-21	Granulocytes	Prf1
8.35E-25	-0.9643449	0.1	0.329	1.18E-20	Granulocytes	Ctsh
8.41E-25	-0.6534911	0.039	0.245	1.19E-20	Granulocytes	Chrac1
8.69E-25	-0.9599979	0.027	0.224	1.23E-20	Granulocytes	Ms4a4c
8.76E-25	-0.7429741	0.055	0.27	1.24E-20	Granulocytes	Bclaf1
9.99E-25	-0.5344297	0.16	0.434	1.41E-20	Granulocytes	Smdt1
1.01E-24	-1.0289048	0.012	0.201	1.42E-20	Granulocytes	Tubb2a
1.02E-24	-0.64241	0.018	0.212	1.45E-20	Granulocytes	Cfdp1
1.09E-24	-0.6692317	0.01	0.198	1.55E-20	Granulocytes	Polr2d
1.17E-24	-0.5220557	0.211	0.507	1.65E-20	Granulocytes	Ndufa2
1.31E-24	-0.5034498	0.258	0.58	1.85E-20	Granulocytes	Srrm2
1.34E-24	-0.5978097	0.035	0.244	1.89E-20	Granulocytes	Abcf1
1.44E-24	-0.794971	0.012	0.199	2.03E-20	Granulocytes	Grap
1.44E-24	-0.8661819	0.08	0.307	2.04E-20	Granulocytes	Bcl2a1d
1.51E-24	-0.521975	0.123	0.378	2.14E-20	Granulocytes	Atpif1
1.54E-24	-0.6290906	0.039	0.248	2.18E-20	Granulocytes	Rassf1
1.55E-24	-0.5065588	0.067	0.294	2.20E-20	Granulocytes	Rnf7
1.56E-24	-0.6451869	0.037	0.241	2.20E-20	Granulocytes	Eif3g
1.57E-24	-0.5854915	0.106	0.356	2.23E-20	Granulocytes	Glud1
1.63E-24	1.31759246	0.389	0.247	2.31E-20	Granulocytes	Atg3
1.66E-24	1.28687138	0.309	0.162	2.35E-20	Granulocytes	Lrrc25
1.71E-24	-0.7133789	0.02	0.214	2.42E-20	Granulocytes	Evl
1.83E-24	-0.6441128	0.041	0.251	2.59E-20	Granulocytes	Fam173a
1.87E-24	-0.625455	0.342	0.635	2.65E-20	Granulocytes	Ldha
1.98E-24	1.2766559	0.245	0.103	2.80E-20	Granulocytes	Ifitm6
1.98E-24	-0.592727	0.025	0.222	2.81E-20	Granulocytes	Nutf2
2.01E-24	-0.550187	0.102	0.347	2.85E-20	Granulocytes	Epsti1
2.07E-24	-0.5444056	0.102	0.342	2.92E-20	Granulocytes	Anapc11
2.17E-24	-0.6597733	0.037	0.239	3.07E-20	Granulocytes	Psmd7
2.24E-24	0.97753601	0.188	0.069	3.17E-20	Granulocytes	St3gal6
2.24E-24	-0.537854	0.055	0.272	3.17E-20	Granulocytes	Polr2e
2.36E-24	-0.6142177	0.055	0.273	3.34E-20	Granulocytes	Glmp
2.44E-24	1.12724932	0.133	0.038	3.46E-20	Granulocytes	Rsad2
2.61E-24	-0.5684038	0.045	0.254	3.69E-20	Granulocytes	Psmc4
2.82E-24	-0.489448	0.082	0.318	3.99E-20	Granulocytes	Anxa6
2.94E-24	1.28466225	0.276	0.135	4.17E-20	Granulocytes	Svil
3.42E-24	-0.5241294	0.1	0.343	4.85E-20	Granulocytes	Gtf2b
3.50E-24	-0.6551223	0.061	0.281	4.95E-20	Granulocytes	Cdc42se2
3.51E-24	-0.6215147	0.092	0.327	4.98E-20	Granulocytes	Ewsr1
3.76E-24	-0.6154919	0.043	0.251	5.32E-20	Granulocytes	Pdap1

3.85E-24	-0.6229275	0.082	0.312	5.45E-20	Granulocytes	G3bp2
3.88E-24	-0.6359429	0.027	0.224	5.49E-20	Granulocytes	Cdk4
3.88E-24	-0.8211628	0.382	0.656	5.50E-20	Granulocytes	Vim
3.90E-24	-0.5451984	0.078	0.308	5.52E-20	Granulocytes	Eif3j1
4.38E-24	-0.6455947	0.02	0.211	6.21E-20	Granulocytes	Mrpl15
4.48E-24	-0.6697838	0.018	0.208	6.35E-20	Granulocytes	Tcof1
4.52E-24	-0.5263034	0.323	0.641	6.40E-20	Granulocytes	Clta
4.63E-24	-0.497384	0.254	0.558	6.56E-20	Granulocytes	Sqstm1
4.68E-24	-0.7714278	0.027	0.221	6.62E-20	Granulocytes	Mxd4
5.16E-24	-0.5281735	0.053	0.27	7.30E-20	Granulocytes	Grpel1
5.30E-24	-0.5781603	0.059	0.278	7.50E-20	Granulocytes	Hprt
5.36E-24	-0.9367453	0.01	0.193	7.58E-20	Granulocytes	Serpina3g
5.49E-24	-0.5554865	0.043	0.249	7.77E-20	Granulocytes	Ube2k
5.50E-24	-0.5946674	0.025	0.218	7.78E-20	Granulocytes	Eif3d
5.55E-24	-0.5292312	0.057	0.274	7.86E-20	Granulocytes	Kxd1
5.56E-24	-0.5007278	0.202	0.501	7.87E-20	Granulocytes	Rbx1
5.65E-24	1.3448189	0.346	0.204	8.00E-20	Granulocytes	R3hdm4
6.33E-24	-0.5757837	0.047	0.259	8.96E-20	Granulocytes	Ccdc59
6.34E-24	-0.3909519	0.08	0.317	8.97E-20	Granulocytes	Ube2n
6.36E-24	-0.5818125	0.033	0.231	9.00E-20	Granulocytes	Tomm5
6.67E-24	-0.6534221	0.014	0.199	9.45E-20	Granulocytes	AI662270
7.63E-24	-0.6163354	0.01	0.193	1.08E-19	Granulocytes	Mrps26
7.67E-24	1.392576	0.423	0.295	1.09E-19	Granulocytes	Rab7
7.91E-24	-0.5740646	0.096	0.333	1.12E-19	Granulocytes	Gng10
7.98E-24	-0.6730411	0.022	0.211	1.13E-19	Granulocytes	Mrps7
8.02E-24	-0.5370893	0.063	0.283	1.14E-19	Granulocytes	Lsm12
8.02E-24	0.93274178	0.174	0.061	1.14E-19	Granulocytes	Iqsec1
8.09E-24	-0.6592552	0.004	0.182	1.15E-19	Granulocytes	Nucks1
8.54E-24	-0.5453921	0.157	0.423	1.21E-19	Granulocytes	Capns1
8.69E-24	-0.6381103	0.055	0.268	1.23E-19	Granulocytes	Tacc1
9.21E-24	-0.5825165	0.037	0.238	1.30E-19	Granulocytes	Psmd11
9.39E-24	-0.665868	0.051	0.26	1.33E-19	Granulocytes	Lsm8
9.97E-24	-0.7517649	0.022	0.211	1.41E-19	Granulocytes	Slc38a1
1.03E-23	-0.6174185	0.016	0.204	1.46E-19	Granulocytes	Nsfl1c
1.08E-23	-0.6635	0.033	0.229	1.53E-19	Granulocytes	Fkbp4
1.09E-23	-0.5340163	0.072	0.293	1.54E-19	Granulocytes	Ufc1
1.09E-23	-0.6111661	0.016	0.202	1.55E-19	Granulocytes	Emc4
1.18E-23	-0.5507678	0.141	0.397	1.67E-19	Granulocytes	Srrm1
1.32E-23	-0.5757701	0.084	0.309	1.87E-19	Granulocytes	Rab10
1.40E-23	-0.6601591	0.055	0.265	1.98E-19	Granulocytes	Nfkbib
1.41E-23	0.89658127	0.141	0.043	1.99E-19	Granulocytes	Cpd
1.41E-23	-0.4501909	0.133	0.392	2.00E-19	Granulocytes	Tmem234
1.43E-23	-0.5510398	0.065	0.282	2.02E-19	Granulocytes	Polr2k
1.43E-23	-0.590983	0.049	0.256	2.03E-19	Granulocytes	Vdac3
1.46E-23	-0.6560468	0.016	0.202	2.07E-19	Granulocytes	Trp53
1.46E-23	-0.4694214	0.098	0.338	2.07E-19	Granulocytes	Psmd4
1.61E-23	1.21862761	0.423	0.286	2.28E-19	Granulocytes	Plp2
1.63E-23	-0.5410923	0.051	0.258	2.31E-19	Granulocytes	Rpp21

1.73E-23	-0.5778468	0.084	0.314	2.44E-19	Granulocytes	Birc6
1.75E-23	-0.7318485	0.006	0.184	2.47E-19	Granulocytes	Lgals3bp
2.07E-23	-0.5387874	0.037	0.236	2.93E-19	Granulocytes	Polr2f
2.16E-23	-0.6961305	0.02	0.208	3.06E-19	Granulocytes	Snrnp48
2.25E-23	-0.5869616	0.057	0.269	3.18E-19	Granulocytes	Etfb
2.26E-23	-0.5026637	0.051	0.259	3.19E-19	Granulocytes	Jtb
2.33E-23	0.91957534	0.634	0.561	3.29E-19	Granulocytes	Gnai2
2.57E-23	-0.456504	0.123	0.368	3.64E-19	Granulocytes	Ndufb4
2.65E-23	-0.5207865	0.045	0.251	3.75E-19	Granulocytes	Rnps1
2.88E-23	-0.6358312	0.1	0.33	4.08E-19	Granulocytes	Ech1
2.93E-23	-0.649376	0.031	0.222	4.14E-19	Granulocytes	Mrpl21
3.01E-23	-0.6918077	0.149	0.404	4.26E-19	Granulocytes	Itga4
3.25E-23	-0.5565344	0.115	0.354	4.60E-19	Granulocytes	Gna13
3.36E-23	-0.6025152	0.031	0.222	4.75E-19	Granulocytes	Chchd1
3.51E-23	0.8426804	0.141	0.044	4.97E-19	Granulocytes	Il13ra1
3.71E-23	-0.9862172	0.014	0.194	5.25E-19	Granulocytes	Tox
3.75E-23	-0.5791038	0.055	0.263	5.31E-19	Granulocytes	Snw1
3.84E-23	-0.5245089	0.18	0.451	5.44E-19	Granulocytes	Csnk2b
3.90E-23	-0.5295678	0.272	0.572	5.52E-19	Granulocytes	Tagln2
4.01E-23	-1.0507976	0.018	0.202	5.68E-19	Granulocytes	Stmn1
4.17E-23	-1.3834608	0.01	0.188	5.90E-19	Granulocytes	Cd7
4.35E-23	-0.6503188	0.016	0.197	6.15E-19	Granulocytes	Bscl2
4.35E-23	-0.5901594	0.051	0.256	6.16E-19	Granulocytes	Commd3
4.65E-23	1.17705332	0.227	0.1	6.59E-19	Granulocytes	Gla
4.99E-23	-0.9822979	0.219	0.474	7.06E-19	Granulocytes	Jun
5.01E-23	-0.7688333	0.012	0.19	7.10E-19	Granulocytes	Nrip1
5.25E-23	-3.4835711	0.022	0.204	7.43E-19	Granulocytes	Gzma
5.27E-23	-0.6133002	0.033	0.225	7.46E-19	Granulocytes	Hdgf
5.31E-23	-0.5814471	0.041	0.241	7.52E-19	Granulocytes	G3bp1
5.34E-23	0.68968949	0.11	0.029	7.56E-19	Granulocytes	Mtus1
5.44E-23	-0.752714	0.012	0.191	7.69E-19	Granulocytes	Pkp3
5.55E-23	-0.657431	0.012	0.192	7.85E-19	Granulocytes	Btg3
5.70E-23	-0.5506939	0.045	0.247	8.06E-19	Granulocytes	Ntan1
5.85E-23	-0.8272441	0.145	0.384	8.28E-19	Granulocytes	Ptms
5.87E-23	-0.7926249	0.112	0.342	8.31E-19	Granulocytes	Lamp1
6.21E-23	-0.5769576	0.084	0.312	8.79E-19	Granulocytes	Pold4
6.26E-23	-0.5737664	0.016	0.198	8.86E-19	Granulocytes	Psmc1
6.27E-23	-0.5661899	0.049	0.253	8.88E-19	Granulocytes	Srsf10
6.39E-23	-0.7847094	0.01	0.184	9.05E-19	Granulocytes	Pde7a
6.63E-23	-0.5168109	0.157	0.418	9.39E-19	Granulocytes	Ywhab
6.66E-23	-0.7699631	0.037	0.228	9.43E-19	Granulocytes	Hexb
7.16E-23	-0.5394505	0.037	0.233	1.01E-18	Granulocytes	Cdc5l
8.13E-23	-0.6277261	0.022	0.205	1.15E-18	Granulocytes	Ssrp1
8.21E-23	-0.8921798	0.018	0.199	1.16E-18	Granulocytes	Gramd3
8.26E-23	-0.718625	0.035	0.227	1.17E-18	Granulocytes	Rgs10
8.30E-23	-0.5753205	0.029	0.217	1.17E-18	Granulocytes	Hnrnpa0
8.44E-23	-0.5861749	0.086	0.309	1.20E-18	Granulocytes	Snrnp70
8.67E-23	-0.5489925	0.057	0.267	1.23E-18	Granulocytes	Rtf1

8.80E-23	-1.2607921	0.033	0.219	1.25E-18	Granulocytes	Lgmn
8.91E-23	-0.6111587	0.029	0.217	1.26E-18	Granulocytes	Sptssa
1.00E-22	-0.5757309	0.043	0.242	1.42E-18	Granulocytes	Scamp3
1.03E-22	-0.4327417	0.268	0.564	1.46E-18	Granulocytes	Eif4a1
1.05E-22	-0.6590038	0.115	0.35	1.49E-18	Granulocytes	Cited2
1.20E-22	-0.7872452	0.008	0.18	1.70E-18	Granulocytes	Gtf2i
1.29E-22	-0.6851794	0.02	0.201	1.83E-18	Granulocytes	Gnptg
1.34E-22	-0.6299547	0.008	0.181	1.90E-18	Granulocytes	Rsl1d1
1.38E-22	-0.6003636	0.108	0.344	1.95E-18	Granulocytes	Pnp
1.38E-22	-0.8578046	0.031	0.216	1.96E-18	Granulocytes	Pou2f2
1.40E-22	1.45921354	0.282	0.146	1.99E-18	Granulocytes	Fbxl5
1.45E-22	-0.493352	0.022	0.208	2.05E-18	Granulocytes	Etfa
1.47E-22	1.01691857	0.168	0.061	2.08E-18	Granulocytes	Slc2a6
1.47E-22	-0.5750428	0.055	0.26	2.08E-18	Granulocytes	Tm2d2
1.49E-22	-0.5798723	0.025	0.209	2.10E-18	Granulocytes	Fkbp2
1.50E-22	-0.3810187	0.151	0.414	2.13E-18	Granulocytes	Sf3b5
1.66E-22	-0.7048365	0.035	0.225	2.34E-18	Granulocytes	6-Sep
1.86E-22	-0.6809659	0.025	0.206	2.63E-18	Granulocytes	Chordc1
1.90E-22	-0.6305731	0.018	0.197	2.68E-18	Granulocytes	Rbbp7
1.96E-22	-0.5329918	0.02	0.201	2.78E-18	Granulocytes	Psmc2
1.98E-22	0.93468557	0.597	0.534	2.80E-18	Granulocytes	Iqgap1
2.01E-22	-0.7849903	0.008	0.18	2.84E-18	Granulocytes	Zap70
2.02E-22	-0.5113186	0.033	0.224	2.86E-18	Granulocytes	Hsd17b10
2.03E-22	-0.5505742	0.051	0.254	2.88E-18	Granulocytes	Ebp
2.08E-22	-0.5367121	0.012	0.187	2.94E-18	Granulocytes	Cmc1
2.09E-22	-0.7378295	0.037	0.229	2.96E-18	Granulocytes	Ifi47
2.09E-22	-0.5410226	0.076	0.293	2.96E-18	Granulocytes	Puf60
2.29E-22	-0.6269996	0.025	0.211	3.24E-18	Granulocytes	Abhd17b
2.30E-22	-0.5083361	0.125	0.365	3.26E-18	Granulocytes	Tmem167
2.32E-22	-0.4573836	0.035	0.229	3.28E-18	Granulocytes	Thoc7
2.56E-22	-0.549966	0.037	0.229	3.62E-18	Granulocytes	Mcts1
2.58E-22	-0.494821	0.063	0.276	3.65E-18	Granulocytes	Ifi35
2.59E-22	-0.6329411	0.01	0.184	3.67E-18	Granulocytes	Nop58
2.63E-22	-0.6948083	0.055	0.254	3.73E-18	Granulocytes	Smc4
2.65E-22	-0.4468858	0.174	0.445	3.75E-18	Granulocytes	Uqcr10
2.97E-22	-0.5021647	0.067	0.278	4.20E-18	Granulocytes	Ywhag
3.01E-22	-0.5152848	0.084	0.303	4.26E-18	Granulocytes	Gch1
3.08E-22	-0.7121049	0.035	0.224	4.36E-18	Granulocytes	Top2b
3.29E-22	-0.4770863	0.123	0.365	4.66E-18	Granulocytes	Psma1
3.50E-22	-0.5051308	0.067	0.279	4.95E-18	Granulocytes	Tmem128
3.64E-22	-0.5529725	0.057	0.26	5.16E-18	Granulocytes	Stub1
3.74E-22	-0.4643214	0.057	0.263	5.30E-18	Granulocytes	Nono
3.94E-22	-0.5592227	0.004	0.172	5.57E-18	Granulocytes	Alg5
4.35E-22	-0.6223079	0.029	0.21	6.16E-18	Granulocytes	Cetn2
4.97E-22	-0.7996313	0.027	0.208	7.03E-18	Granulocytes	Sptbn1
5.08E-22	-1.0730269	0.008	0.177	7.19E-18	Granulocytes	Tnfrsf9
5.15E-22	-0.514968	0.08	0.296	7.29E-18	Granulocytes	Rrp1
5.18E-22	-0.4868255	0.342	0.654	7.34E-18	Granulocytes	Ppp1ca

5.73E-22	-0.5331513	0.086	0.305	8.12E-18	Granulocytes	Slbp
5.75E-22	1.21793672	0.211	0.09	8.15E-18	Granulocytes	Rab20
6.26E-22	-0.5623847	0.029	0.212	8.87E-18	Granulocytes	Exosc3
6.27E-22	-0.7841342	0.012	0.183	8.88E-18	Granulocytes	AU020206
6.63E-22	-0.5880252	0.033	0.218	9.39E-18	Granulocytes	Babam1
6.63E-22	-0.8668877	0.481	0.738	9.39E-18	Granulocytes	AY036118
6.76E-22	0.93621175	0.129	0.039	9.57E-18	Granulocytes	Oas3
6.86E-22	-0.4980183	0.112	0.342	9.71E-18	Granulocytes	Ap2s1
7.21E-22	-0.7910983	0.065	0.269	1.02E-17	Granulocytes	Glul
7.34E-22	-0.5365115	0.02	0.196	1.04E-17	Granulocytes	Psmd6
7.39E-22	-0.3776495	0.209	0.495	1.05E-17	Granulocytes	Arpp19
7.42E-22	-1.4270288	0.016	0.187	1.05E-17	Granulocytes	Fcmr
7.52E-22	-0.5778024	0.043	0.235	1.06E-17	Granulocytes	Caprin1
7.73E-22	-0.7988182	0.01	0.178	1.09E-17	Granulocytes	Ccr5
7.80E-22	-0.613025	0.041	0.232	1.10E-17	Granulocytes	Dhx15
7.87E-22	-0.4347788	0.088	0.314	1.11E-17	Granulocytes	Txndc17
8.48E-22	-0.488299	0.135	0.377	1.20E-17	Granulocytes	7-Sep
8.90E-22	-0.5674557	0.057	0.258	1.26E-17	Granulocytes	Dnajc7
9.30E-22	-0.5067816	0.02	0.198	1.32E-17	Granulocytes	Kdelr2
9.49E-22	-0.5041978	0.041	0.231	1.34E-17	Granulocytes	Mrpl36
9.50E-22	-0.479976	0.112	0.349	1.34E-17	Granulocytes	Dynlrb1
1.03E-21	-0.6564966	0.01	0.178	1.46E-17	Granulocytes	Fam3c
1.06E-21	-0.5423879	0.045	0.24	1.50E-17	Granulocytes	Taf15
1.09E-21	-1.0743263	0.022	0.198	1.55E-17	Granulocytes	Cd55
1.12E-21	-0.466823	0.047	0.243	1.58E-17	Granulocytes	Txndc9
1.20E-21	-0.6518658	0.025	0.202	1.70E-17	Granulocytes	Sh3bgrl
1.25E-21	-0.9625596	0.008	0.173	1.77E-17	Granulocytes	Ralgps2
1.26E-21	-0.5830339	0.012	0.182	1.79E-17	Granulocytes	Chchd7
1.31E-21	-0.902857	0.025	0.2	1.86E-17	Granulocytes	Cd72
1.35E-21	-0.532351	0.02	0.196	1.91E-17	Granulocytes	Mrps18a
1.35E-21	-0.5406226	0.027	0.208	1.91E-17	Granulocytes	Hnrnpd
1.38E-21	0.92120294	0.153	0.053	1.96E-17	Granulocytes	Cpne2
1.74E-21	-0.5024405	0.033	0.217	2.46E-17	Granulocytes	Sar1b
1.75E-21	-0.4119886	0.127	0.373	2.48E-17	Granulocytes	Cirbp
1.77E-21	-0.5213589	0.182	0.442	2.51E-17	Granulocytes	Ndufb7
1.78E-21	-0.6439755	0.01	0.176	2.53E-17	Granulocytes	Snrpa1
1.83E-21	-0.4732186	0.09	0.309	2.58E-17	Granulocytes	Pfdn2
1.97E-21	-0.5663143	0.027	0.204	2.79E-17	Granulocytes	Arl6ip4
2.05E-21	-0.6018341	0.025	0.201	2.90E-17	Granulocytes	Nans
2.19E-21	1.0507467	0.538	0.45	3.10E-17	Granulocytes	Adgre5
2.37E-21	-0.474842	0.19	0.457	3.35E-17	Granulocytes	Rap1a
2.38E-21	-0.4563985	0.153	0.405	3.37E-17	Granulocytes	Luc7l2
2.49E-21	-0.4789958	0.057	0.254	3.52E-17	Granulocytes	Taf10
2.49E-21	-0.5343828	0.012	0.18	3.52E-17	Granulocytes	Mdp1
2.54E-21	-0.5236278	0.051	0.245	3.60E-17	Granulocytes	U2af2
2.70E-21	-1.307338	0.014	0.181	3.82E-17	Granulocytes	Ms4a1
2.76E-21	-0.5400607	0.055	0.249	3.90E-17	Granulocytes	Ctbp1
2.79E-21	-0.6058604	0.059	0.254	3.95E-17	Granulocytes	Myo1g

2.80E-21	-0.5214629	0.016	0.186	3.96E-17	Granulocytes	Pam16
2.85E-21	-0.6499132	0.049	0.241	4.04E-17	Granulocytes	Odc1
2.98E-21	-0.5103714	0.057	0.258	4.21E-17	Granulocytes	Ppm1g
2.99E-21	-0.5983393	0.037	0.221	4.23E-17	Granulocytes	Prpf38b
3.10E-21	-0.6045747	0.012	0.18	4.39E-17	Granulocytes	Ccdc50
3.41E-21	-0.5541222	0.035	0.217	4.83E-17	Granulocytes	Acadl
3.55E-21	-0.5161271	0.037	0.221	5.02E-17	Granulocytes	Smarce1
3.70E-21	-0.6995137	0.016	0.184	5.23E-17	Granulocytes	Vars
3.81E-21	-0.5791116	0.016	0.186	5.39E-17	Granulocytes	Gadd45gip1
3.82E-21	-0.3825773	0.059	0.259	5.41E-17	Granulocytes	Gm16286
3.88E-21	-0.5109482	0.031	0.211	5.49E-17	Granulocytes	Mrpl32
3.88E-21	-0.5620547	0.027	0.202	5.49E-17	Granulocytes	Nfu1
4.05E-21	-0.4476185	0.08	0.293	5.74E-17	Granulocytes	Gtf2h5
4.26E-21	-0.5284592	0.094	0.31	6.03E-17	Granulocytes	M6pr
4.41E-21	-0.5004662	0.063	0.264	6.24E-17	Granulocytes	Ndufs6
4.42E-21	-0.5587717	0.018	0.189	6.26E-17	Granulocytes	Rtcb
4.46E-21	0.51842018	0.836	0.795	6.31E-17	Granulocytes	Rac2
4.52E-21	-0.5274662	0.016	0.186	6.41E-17	Granulocytes	Mrps34
4.56E-21	-0.5822344	0.008	0.169	6.46E-17	Granulocytes	Paics
4.91E-21	-0.5921332	0.027	0.204	6.95E-17	Granulocytes	Hnrnph3
5.19E-21	1.02985966	0.18	0.071	7.34E-17	Granulocytes	Smim3
5.19E-21	-0.4329429	0.168	0.42	7.35E-17	Granulocytes	Tmed2
5.29E-21	-0.6797492	0.016	0.184	7.49E-17	Granulocytes	Rasal3
5.61E-21	-0.5397952	0.057	0.252	7.94E-17	Granulocytes	Eif1ax
5.76E-21	-0.7557922	0.008	0.168	8.15E-17	Granulocytes	Man1a
5.94E-21	-2.5642591	0.039	0.213	8.41E-17	Granulocytes	C1qb
6.42E-21	-0.4606852	0.333	0.621	9.09E-17	Granulocytes	Cox6b1
6.77E-21	-0.6011381	0.016	0.184	9.58E-17	Granulocytes	Tmem126a
6.99E-21	-0.6014166	0.025	0.197	9.90E-17	Granulocytes	Snrpa
7.00E-21	-0.5853971	0.02	0.189	9.91E-17	Granulocytes	Dpy30
7.18E-21	-0.5968874	0.082	0.291	1.02E-16	Granulocytes	Tap1
7.68E-21	-0.6033058	0.022	0.192	1.09E-16	Granulocytes	Snhg12
8.02E-21	-0.3803918	0.223	0.503	1.13E-16	Granulocytes	Uqcrq
8.02E-21	-0.7280365	0.025	0.195	1.13E-16	Granulocytes	Gimap9
8.36E-21	-0.5076352	0.151	0.394	1.18E-16	Granulocytes	Leprotl1
8.54E-21	-0.485664	0.065	0.264	1.21E-16	Granulocytes	Zbtb7a
8.58E-21	-0.5377252	0.072	0.275	1.21E-16	Granulocytes	Tor1aip2
8.60E-21	0.53804586	0.36	0.192	1.22E-16	Granulocytes	Tgfbi
8.69E-21	-0.515256	0.018	0.187	1.23E-16	Granulocytes	Mydgf
8.71E-21	-0.5746476	0.045	0.231	1.23E-16	Granulocytes	Foxn3
8.77E-21	-0.4380605	0.084	0.293	1.24E-16	Granulocytes	Prpf40a
8.99E-21	-0.4133467	0.133	0.37	1.27E-16	Granulocytes	Dctn3
9.09E-21	-0.590643	0.025	0.197	1.29E-16	Granulocytes	Pop5
9.16E-21	-0.9359934	0.01	0.17	1.30E-16	Granulocytes	Klrk1
9.69E-21	-0.7764449	0.016	0.18	1.37E-16	Granulocytes	Nrp1
1.02E-20	1.01911023	0.172	0.066	1.44E-16	Granulocytes	Pdlim7
1.04E-20	-0.4390153	0.082	0.291	1.48E-16	Granulocytes	Rbm8a
1.05E-20	-0.5064364	0.215	0.481	1.49E-16	Granulocytes	H2-T23

1.08E-20	-0.495831	0.053	0.242	1.53E-16	Granulocytes	Uqcrc2
1.10E-20	-0.7804023	0.039	0.216	1.56E-16	Granulocytes	Nfatc3
1.16E-20	-0.5372855	0.018	0.185	1.64E-16	Granulocytes	Acbd6
1.20E-20	-0.5854294	0.051	0.239	1.70E-16	Granulocytes	Ddx50
1.24E-20	0.8566671	0.587	0.537	1.75E-16	Granulocytes	Ostf1
1.28E-20	-0.5051933	0.049	0.239	1.81E-16	Granulocytes	Actn4
1.29E-20	-0.5051635	0.033	0.21	1.83E-16	Granulocytes	Glrx5
1.35E-20	-0.4266825	0.074	0.28	1.91E-16	Granulocytes	Sugt1
1.37E-20	-0.5483508	0.415	0.706	1.94E-16	Granulocytes	Eif5a
1.39E-20	-0.5903986	0.027	0.198	1.96E-16	Granulocytes	Uchl3
1.47E-20	-0.5665389	0.049	0.234	2.08E-16	Granulocytes	Tmem208
1.51E-20	0.99005336	0.176	0.069	2.14E-16	Granulocytes	Mir142hg
1.57E-20	-0.510166	0.022	0.192	2.23E-16	Granulocytes	Osgep
1.58E-20	-0.7442981	0.041	0.221	2.24E-16	Granulocytes	Malt1
1.65E-20	-0.4942955	0.029	0.204	2.33E-16	Granulocytes	Psmb7
1.82E-20	-0.7633342	0.027	0.197	2.57E-16	Granulocytes	mt-Nd6
1.87E-20	-0.6134557	0.012	0.173	2.65E-16	Granulocytes	Ddx18
1.91E-20	-0.6430349	0.783	0.938	2.71E-16	Granulocytes	Lars2
1.91E-20	-0.5035768	0.049	0.236	2.71E-16	Granulocytes	Tex261
1.92E-20	-0.6050331	0.031	0.204	2.72E-16	Granulocytes	Gm11808
1.97E-20	-0.5755165	0.004	0.159	2.78E-16	Granulocytes	Lpxn
2.13E-20	0.97110578	0.151	0.054	3.01E-16	Granulocytes	Pfkfb4
2.22E-20	1.17613822	0.284	0.154	3.14E-16	Granulocytes	Gsn
2.39E-20	-1.1129154	0.022	0.187	3.39E-16	Granulocytes	Id3
2.46E-20	-0.4878559	0.035	0.21	3.48E-16	Granulocytes	Eif3l
2.47E-20	-1.0387455	0.039	0.212	3.50E-16	Granulocytes	Fcgr2b
2.54E-20	-0.5397674	0.031	0.204	3.59E-16	Granulocytes	Cbx1
2.71E-20	1.2232518	0.36	0.229	3.84E-16	Granulocytes	Per1
2.74E-20	-0.3832556	0.123	0.352	3.88E-16	Granulocytes	Ndufs7
3.16E-20	-0.5236052	0.319	0.6	4.47E-16	Granulocytes	Ndufa13
3.27E-20	-0.5764818	0.117	0.34	4.63E-16	Granulocytes	Rrbp1
3.40E-20	-0.5589264	0.037	0.213	4.81E-16	Granulocytes	Tbrg1
3.49E-20	-0.6462893	0.002	0.154	4.94E-16	Granulocytes	Cd38
3.57E-20	-0.5416295	0.051	0.236	5.05E-16	Granulocytes	Bub3
3.59E-20	-0.8342308	0.008	0.164	5.09E-16	Granulocytes	Ckb
3.66E-20	-0.4806481	0.035	0.21	5.18E-16	Granulocytes	Psmd14
3.71E-20	-0.3916091	0.059	0.25	5.25E-16	Granulocytes	Ndufs2
3.72E-20	-0.4333481	0.194	0.455	5.27E-16	Granulocytes	Erp29
4.16E-20	-0.5927503	0.008	0.162	5.89E-16	Granulocytes	Csrp1
4.25E-20	-0.5824295	0.008	0.164	6.02E-16	Granulocytes	Brix1
4.33E-20	1.03239189	0.562	0.489	6.13E-16	Granulocytes	Samhd1
4.67E-20	-0.5291246	0.065	0.261	6.62E-16	Granulocytes	Cd164
4.77E-20	-0.4756409	0.055	0.245	6.75E-16	Granulocytes	Naa10
5.07E-20	-0.6184404	0.043	0.222	7.17E-16	Granulocytes	Pdcd4
5.09E-20	-0.5606452	0.022	0.186	7.21E-16	Granulocytes	Ssbp4
5.14E-20	1.08765242	0.215	0.096	7.27E-16	Granulocytes	Tlr2
5.16E-20	0.7090378	0.687	0.676	7.30E-16	Granulocytes	Ptprc
5.26E-20	-0.478576	0.147	0.379	7.44E-16	Granulocytes	Stk24

5.34E-20	-0.9079838	0.02	0.182	7.56E-16	Granulocytes	Gpr183
5.38E-20	-0.5644151	0.027	0.195	7.61E-16	Granulocytes	Gspt1
5.52E-20	-0.5428858	0.041	0.218	7.81E-16	Granulocytes	Ube3a
5.83E-20	-0.5043951	0.014	0.174	8.25E-16	Granulocytes	Slirp
6.54E-20	-0.4602248	0.106	0.322	9.26E-16	Granulocytes	Mlf2
6.64E-20	-0.5133991	0.014	0.173	9.40E-16	Granulocytes	Samm50
6.76E-20	-0.4002044	0.065	0.262	9.57E-16	Granulocytes	Gps2
6.77E-20	-0.5872868	0.027	0.194	9.59E-16	Granulocytes	Gorasp2
6.82E-20	-0.4801745	0.215	0.473	9.66E-16	Granulocytes	Arhgef1
7.15E-20	-0.5990803	0.072	0.266	1.01E-15	Granulocytes	Scaf11
7.44E-20	-0.8433882	0.16	0.375	1.05E-15	Granulocytes	Ctsz
7.50E-20	-0.4980469	0.008	0.163	1.06E-15	Granulocytes	Clns1a
7.71E-20	-0.4906685	0.049	0.232	1.09E-15	Granulocytes	Cetn3
7.95E-20	-0.4721186	0.025	0.192	1.13E-15	Granulocytes	Suclg1
9.21E-20	-0.3230444	0.098	0.309	1.30E-15	Granulocytes	Dnaja2
9.26E-20	-0.645251	0.012	0.168	1.31E-15	Granulocytes	Dnajc1
9.35E-20	-0.4248816	0.049	0.234	1.32E-15	Granulocytes	Rnf138
1.02E-19	-0.5008034	0.018	0.179	1.44E-15	Granulocytes	Psmd2
1.03E-19	-0.5803282	0.072	0.265	1.46E-15	Granulocytes	Matr3
1.05E-19	-0.3594388	0.157	0.401	1.48E-15	Granulocytes	Lamtor2
1.14E-19	-0.5171153	0.037	0.21	1.61E-15	Granulocytes	Anapc5
1.15E-19	-1.3698086	0.012	0.167	1.62E-15	Granulocytes	Tnfrsf4
1.23E-19	-0.628286	0.035	0.204	1.74E-15	Granulocytes	Sf3b3
1.25E-19	-0.4571597	0.074	0.271	1.77E-15	Granulocytes	Gltp
1.26E-19	-0.7276236	0.014	0.17	1.79E-15	Granulocytes	Ass1
1.35E-19	-0.4873772	0.178	0.42	1.91E-15	Granulocytes	Cmtm7
1.35E-19	-2.1885807	0.025	0.185	1.91E-15	Granulocytes	C1qc
1.38E-19	-0.4684082	0.049	0.229	1.96E-15	Granulocytes	Polr2j
1.39E-19	-2.3010279	0.029	0.19	1.96E-15	Granulocytes	C1qa
1.45E-19	-0.6218376	0.45	0.711	2.05E-15	Granulocytes	Dnaja1
1.47E-19	-0.5391998	0.047	0.226	2.08E-15	Granulocytes	Ddx39
1.50E-19	-0.5498366	0.014	0.171	2.13E-15	Granulocytes	Gtpbp4
1.51E-19	-0.3708217	0.112	0.331	2.13E-15	Granulocytes	Ap2m1
1.54E-19	-1.9128654	0.121	0.311	2.17E-15	Granulocytes	Apoe
1.54E-19	-0.5029059	0.02	0.182	2.18E-15	Granulocytes	Ddx47
1.62E-19	-0.6993708	0.01	0.163	2.30E-15	Granulocytes	Inpp4b
1.65E-19	-0.4576165	0.104	0.317	2.34E-15	Granulocytes	Dusp11
1.65E-19	-0.5841241	0.041	0.216	2.34E-15	Granulocytes	Tpp2
1.71E-19	1.40450941	0.354	0.224	2.42E-15	Granulocytes	Nfkbid
1.72E-19	-0.6023391	0.012	0.167	2.44E-15	Granulocytes	Pdia4
1.74E-19	-0.62786	0.008	0.159	2.47E-15	Granulocytes	Scpep1
1.76E-19	-0.4397791	0.037	0.212	2.49E-15	Granulocytes	Nsmce4a
1.89E-19	-0.5496195	0.012	0.165	2.68E-15	Granulocytes	Mrpl13
2.03E-19	-0.6711632	0.039	0.211	2.87E-15	Granulocytes	Tagap
2.15E-19	-0.5832881	0.016	0.173	3.05E-15	Granulocytes	Fam204a
2.16E-19	-0.5402991	0.043	0.217	3.06E-15	Granulocytes	Sf3b4
2.25E-19	-0.7384741	0.01	0.162	3.19E-15	Granulocytes	Havcr2
2.60E-19	-0.4598228	0.047	0.225	3.68E-15	Granulocytes	Idnk

2.65E-19	-0.4521558	0.008	0.159	3.75E-15	Granulocytes	Prdx3
2.73E-19	-0.4133561	0.057	0.241	3.87E-15	Granulocytes	Coa3
2.89E-19	-0.5803445	0.008	0.158	4.09E-15	Granulocytes	Mri1
2.94E-19	-0.4752256	0.047	0.223	4.16E-15	Granulocytes	Dctn6
3.09E-19	-0.4861229	0.09	0.292	4.37E-15	Granulocytes	Sh3kbp1
3.12E-19	-0.5339259	0.014	0.167	4.42E-15	Granulocytes	Ndufa9
3.32E-19	-0.9017603	0.047	0.218	4.70E-15	Granulocytes	Cd81
3.40E-19	-0.5883562	0.016	0.174	4.81E-15	Granulocytes	Ubash3b
3.48E-19	-0.7256462	0.029	0.19	4.92E-15	Granulocytes	Stt3b
3.52E-19	-0.5621458	0.025	0.184	4.99E-15	Granulocytes	Chchd3
3.66E-19	-0.6136077	0.022	0.181	5.18E-15	Granulocytes	Polr2m
3.92E-19	-0.5827908	0.035	0.203	5.55E-15	Granulocytes	Bod1l
4.07E-19	-0.4018868	0.039	0.21	5.76E-15	Granulocytes	Sltm
4.07E-19	-0.5156764	0.029	0.193	5.77E-15	Granulocytes	Ttc14
4.08E-19	-0.313071	0.125	0.35	5.77E-15	Granulocytes	Rer1
4.15E-19	-0.5227595	0.025	0.185	5.87E-15	Granulocytes	Cops8
4.19E-19	-0.3234244	0.174	0.418	5.93E-15	Granulocytes	Sp100
4.50E-19	-1.0603461	0.012	0.162	6.37E-15	Granulocytes	Cd19
4.53E-19	-0.3642251	0.112	0.329	6.42E-15	Granulocytes	Plekhj1
4.63E-19	-0.476243	0.022	0.181	6.55E-15	Granulocytes	Ola1
4.75E-19	-0.5845658	0.051	0.23	6.72E-15	Granulocytes	Med21
4.90E-19	-0.9320453	0.008	0.157	6.94E-15	Granulocytes	Dusp10
4.95E-19	-0.5003578	0.153	0.385	7.00E-15	Granulocytes	Hnrnpu
4.98E-19	-0.4500669	0.061	0.244	7.05E-15	Granulocytes	Eif4h
5.42E-19	-0.4798181	0.055	0.236	7.67E-15	Granulocytes	Ogdh
5.45E-19	-0.6573156	0.004	0.149	7.71E-15	Granulocytes	Sh2d1a
5.50E-19	-0.5222675	0.01	0.161	7.79E-15	Granulocytes	Prrc2b
5.55E-19	-0.406295	0.063	0.248	7.85E-15	Granulocytes	Ssna1
5.73E-19	-0.4837426	0.039	0.21	8.12E-15	Granulocytes	Tm2d1
5.84E-19	-0.3951925	0.051	0.233	8.27E-15	Granulocytes	Lsm3
5.89E-19	-0.6174216	0.006	0.152	8.33E-15	Granulocytes	Parp1
5.91E-19	1.24705587	0.284	0.16	8.37E-15	Granulocytes	Glipr2
6.07E-19	-0.6015501	0.025	0.184	8.59E-15	Granulocytes	Syncrip
6.12E-19	-0.5594296	0.02	0.178	8.66E-15	Granulocytes	Pnn
6.17E-19	-0.4701166	0.029	0.19	8.73E-15	Granulocytes	Mrpl40
6.45E-19	-0.5093484	0.025	0.185	9.13E-15	Granulocytes	Lrpap1
6.45E-19	-0.6312763	0.025	0.184	9.13E-15	Granulocytes	Uhrf2
6.59E-19	-0.6234233	0.006	0.152	9.33E-15	Granulocytes	Mllt3
6.78E-19	-0.7991267	0.008	0.154	9.59E-15	Granulocytes	Rasgef1b
6.81E-19	-0.6319162	0.041	0.212	9.63E-15	Granulocytes	Rbpj
7.03E-19	-0.6048464	0.012	0.162	9.95E-15	Granulocytes	Alkbh1
7.21E-19	-0.4923343	0.037	0.205	1.02E-14	Granulocytes	Dnajb11
7.46E-19	-0.5012937	0.067	0.257	1.06E-14	Granulocytes	Rbbp4
7.50E-19	-0.3888516	0.143	0.374	1.06E-14	Granulocytes	Peli1
7.51E-19	-0.5608997	0.049	0.225	1.06E-14	Granulocytes	Traf1
7.58E-19	-0.7872437	0.01	0.158	1.07E-14	Granulocytes	Cd5
7.62E-19	-0.8109317	0.002	0.144	1.08E-14	Granulocytes	Fasl
8.01E-19	-0.5773544	0.029	0.189	1.13E-14	Granulocytes	Ddx54

8.03E-19	-0.5520254	0.041	0.208	1.14E-14	Granulocytes	Ubac2
8.06E-19	-0.3951706	0.065	0.25	1.14E-14	Granulocytes	Trappc6b
8.26E-19	0.98796889	0.581	0.548	1.17E-14	Granulocytes	Ube2b
8.40E-19	-0.449976	0.037	0.205	1.19E-14	Granulocytes	Sf3a2
8.58E-19	-0.4496283	0.2	0.449	1.21E-14	Granulocytes	Psmb9
8.88E-19	-0.5982014	0.022	0.179	1.26E-14	Granulocytes	B4galt1
8.96E-19	-0.5030531	0.022	0.179	1.27E-14	Granulocytes	Tmem192
9.70E-19	-0.5880818	0.02	0.176	1.37E-14	Granulocytes	Nolc1
1.02E-18	-0.5262122	0.088	0.281	1.44E-14	Granulocytes	Fermt3
1.08E-18	-0.4191603	0.057	0.237	1.53E-14	Granulocytes	Ngdn
1.09E-18	-0.4732592	0.037	0.204	1.54E-14	Granulocytes	Guk1
1.10E-18	-0.4901376	0.012	0.162	1.55E-14	Granulocytes	Nip7
1.11E-18	-0.6283516	0.004	0.147	1.58E-14	Granulocytes	Itpr1
1.14E-18	-0.4826705	0.053	0.231	1.61E-14	Granulocytes	Srsf6
1.18E-18	-0.5294774	0.02	0.174	1.67E-14	Granulocytes	Rad23b
1.23E-18	-0.4383323	0.053	0.23	1.74E-14	Granulocytes	Lims1
1.24E-18	-0.6317856	0.006	0.15	1.75E-14	Granulocytes	Kdm2b
1.30E-18	-0.7678137	0.135	0.34	1.84E-14	Granulocytes	Napsa
1.36E-18	-0.5231129	0.008	0.154	1.93E-14	Granulocytes	Oxa1l
1.37E-18	-0.3907012	0.041	0.211	1.94E-14	Granulocytes	Commd7
1.44E-18	-0.8005192	0	0.139	2.03E-14	Granulocytes	Izumo1r
1.51E-18	-0.5271258	0.022	0.179	2.14E-14	Granulocytes	Pon2
1.59E-18	-0.5287121	0.016	0.166	2.25E-14	Granulocytes	1110038F14R
1.63E-18	-0.5042526	0.018	0.17	2.31E-14	Granulocytes	Snrnp40
1.65E-18	-0.6562502	0.027	0.185	2.33E-14	Granulocytes	Itgav
1.66E-18	-0.4172851	0.045	0.217	2.35E-14	Granulocytes	Fcf1
1.66E-18	-0.5144541	0.035	0.199	2.35E-14	Granulocytes	Yipf5
1.67E-18	-0.5525092	0.008	0.152	2.36E-14	Granulocytes	Actl6a
1.75E-18	-0.5549928	0.018	0.17	2.48E-14	Granulocytes	Pop7
1.77E-18	-0.4581713	0.035	0.198	2.51E-14	Granulocytes	Ndufv1
1.90E-18	-0.7152268	0.025	0.18	2.69E-14	Granulocytes	Cerk
1.92E-18	1.09453747	0.497	0.416	2.72E-14	Granulocytes	Ccnl1
1.96E-18	1.02978181	0.135	0.047	2.77E-14	Granulocytes	Osgin1
1.97E-18	-0.4400552	0.055	0.232	2.79E-14	Granulocytes	Ift20
1.98E-18	-0.9015174	0.022	0.177	2.80E-14	Granulocytes	Aif1
2.05E-18	-0.5120942	0.006	0.149	2.90E-14	Granulocytes	Immt
2.10E-18	-0.5133803	0.025	0.181	2.97E-14	Granulocytes	Phax
2.10E-18	-0.3288827	0.29	0.58	2.97E-14	Granulocytes	Morf4l1
2.14E-18	-0.6770755	0.016	0.167	3.03E-14	Granulocytes	Gpr18
2.18E-18	-0.539423	0.047	0.218	3.08E-14	Granulocytes	Csnk2a1
2.20E-18	-0.8388715	0.1	0.291	3.12E-14	Granulocytes	Cybb
2.35E-18	-0.5024597	0.025	0.179	3.33E-14	Granulocytes	Psmg4
2.36E-18	-0.5639861	0.016	0.166	3.34E-14	Granulocytes	Luc7l3
2.45E-18	-0.4285399	0.037	0.2	3.47E-14	Granulocytes	Mrps12
2.55E-18	-0.5043649	0.084	0.274	3.61E-14	Granulocytes	Tgfbr2
2.61E-18	-0.5485705	0.02	0.172	3.69E-14	Granulocytes	Trim28
2.68E-18	-0.5359552	0.27	0.514	3.79E-14	Granulocytes	Serinc3
2.95E-18	-0.4478509	0.072	0.257	4.17E-14	Granulocytes	Ube2j1

2.98E-18	-0.4808082	0.076	0.258	4.21E-14	Granulocytes	AI413582
3.11E-18	-0.5142948	0.039	0.204	4.41E-14	Granulocytes	N4bp2l2
3.12E-18	-0.4106513	0.033	0.196	4.42E-14	Granulocytes	Fip1l1
3.22E-18	-0.443778	0.072	0.255	4.56E-14	Granulocytes	Mvb12a
3.26E-18	-0.5275555	0.067	0.248	4.61E-14	Granulocytes	Chd1
3.48E-18	-0.616824	0.025	0.179	4.93E-14	Granulocytes	Rassf4
3.65E-18	-0.3954374	0.274	0.555	5.17E-14	Granulocytes	Cox7a2
3.75E-18	-0.5583091	0.01	0.153	5.31E-14	Granulocytes	Plekho1
3.75E-18	-0.4547724	0.031	0.189	5.31E-14	Granulocytes	Srp72
3.81E-18	-0.5089068	0.037	0.198	5.39E-14	Granulocytes	Rpn1
3.82E-18	-0.3995085	0.029	0.188	5.41E-14	Granulocytes	Eprs
4.03E-18	-0.5784768	0.02	0.17	5.70E-14	Granulocytes	Adcy7
4.05E-18	-0.5041899	0.027	0.181	5.74E-14	Granulocytes	Slc35b1
4.12E-18	-0.4659424	0.035	0.196	5.84E-14	Granulocytes	0610012G03I
4.13E-18	-0.586536	0.006	0.146	5.84E-14	Granulocytes	Mcm6
4.19E-18	-0.5362018	0.039	0.202	5.93E-14	Granulocytes	Cd86
4.29E-18	-0.4289092	0.086	0.276	6.08E-14	Granulocytes	Rnh1
4.50E-18	-0.4688309	0.002	0.139	6.37E-14	Granulocytes	Idh3a
4.63E-18	-0.3662519	0.045	0.214	6.55E-14	Granulocytes	Slc25a39
4.78E-18	-0.5373501	0.008	0.149	6.76E-14	Granulocytes	Pigp
5.35E-18	-0.4724157	0.051	0.223	7.58E-14	Granulocytes	Cbx4
5.37E-18	-0.5269235	0.004	0.141	7.61E-14	Granulocytes	Ebpl
5.40E-18	-0.4941866	0.033	0.19	7.64E-14	Granulocytes	Eif3b
5.55E-18	-0.7826793	0.01	0.152	7.86E-14	Granulocytes	Bach2
5.63E-18	-0.4428011	0.029	0.186	7.97E-14	Granulocytes	Lsm2
5.81E-18	-1.1688812	0.055	0.223	8.22E-14	Granulocytes	Hist1h2ap
6.08E-18	-0.6034481	0.016	0.162	8.61E-14	Granulocytes	Hivep2
6.19E-18	1.15623564	0.411	0.295	8.77E-14	Granulocytes	Klf3
6.39E-18	-0.3524688	0.18	0.421	9.04E-14	Granulocytes	Ndufa1
6.49E-18	-0.4168136	0.047	0.217	9.19E-14	Granulocytes	Sec61a1
6.68E-18	-0.4006061	0.072	0.255	9.46E-14	Granulocytes	Zbp1
6.77E-18	-0.3201451	0.121	0.331	9.59E-14	Granulocytes	Bag1
7.11E-18	-0.4613904	0.012	0.156	1.01E-13	Granulocytes	Immp1l
7.17E-18	0.50875733	0.838	0.844	1.02E-13	Granulocytes	Serf2
7.18E-18	-0.4464712	0.014	0.16	1.02E-13	Granulocytes	Dohh
7.21E-18	-0.4683235	0.117	0.32	1.02E-13	Granulocytes	Smim14
7.28E-18	-0.5712216	0.016	0.162	1.03E-13	Granulocytes	Tcf3
7.80E-18	-0.500869	0.018	0.165	1.10E-13	Granulocytes	Ythdf2
7.86E-18	-0.4926257	0.002	0.137	1.11E-13	Granulocytes	Wdr18
8.26E-18	-0.4751501	0.045	0.21	1.17E-13	Granulocytes	Itm2c
8.32E-18	-0.5223576	0.02	0.168	1.18E-13	Granulocytes	Chd9
8.52E-18	-0.5493736	0.008	0.146	1.21E-13	Granulocytes	Cat
8.64E-18	-0.5011212	0.041	0.203	1.22E-13	Granulocytes	Hdac1
8.70E-18	-0.5785869	0.004	0.14	1.23E-13	Granulocytes	Agpat3
9.03E-18	-0.529445	0.016	0.161	1.28E-13	Granulocytes	Smarca4
9.30E-18	-0.524364	0.033	0.189	1.32E-13	Granulocytes	Sars
9.43E-18	-0.4185617	0.076	0.26	1.34E-13	Granulocytes	Pdcd6ip
9.52E-18	-0.4623916	0.039	0.202	1.35E-13	Granulocytes	Psmc6

1.01E-17	-0.4043061	0.072	0.253	1.43E-13	Granulocytes	Pcna
1.03E-17	-0.4517534	0.012	0.155	1.46E-13	Granulocytes	Blvra
1.05E-17	-0.4912655	0.014	0.158	1.49E-13	Granulocytes	Tmem147
1.05E-17	-0.5813252	0.02	0.168	1.49E-13	Granulocytes	Cndp2
1.06E-17	-0.4698275	0.09	0.278	1.50E-13	Granulocytes	Kif5b
1.07E-17	-0.3797039	0.135	0.353	1.51E-13	Granulocytes	Map2k2
1.08E-17	-0.503404	0.016	0.161	1.52E-13	Granulocytes	Casp8
1.09E-17	-0.5114101	0.018	0.163	1.54E-13	Granulocytes	Pdrg1
1.10E-17	-0.3377473	0.041	0.206	1.55E-13	Granulocytes	Strap
1.18E-17	-0.3654281	0.16	0.385	1.67E-13	Granulocytes	Paip2
1.20E-17	-0.398269	0.07	0.25	1.70E-13	Granulocytes	Znrd1
1.21E-17	-0.4073414	0.078	0.262	1.71E-13	Granulocytes	Trappc4
1.21E-17	-0.5196022	0.053	0.219	1.71E-13	Granulocytes	Pcmt1
1.23E-17	-0.5752182	0.012	0.153	1.74E-13	Granulocytes	5430416N02I
1.31E-17	-0.5016396	0.088	0.276	1.86E-13	Granulocytes	Sla
1.34E-17	-0.4011202	0.354	0.619	1.90E-13	Granulocytes	Sec61b
1.41E-17	-0.467014	0.01	0.15	2.00E-13	Granulocytes	Cisd1
1.46E-17	-0.5592896	0.033	0.186	2.06E-13	Granulocytes	Limd1
1.46E-17	-0.5070372	0.031	0.183	2.06E-13	Granulocytes	Trappc6a
1.46E-17	-0.4729691	0.004	0.138	2.06E-13	Granulocytes	Timm50
1.47E-17	-0.8334171	0.014	0.154	2.08E-13	Granulocytes	H2-Oa
1.49E-17	-0.3505398	0.104	0.305	2.10E-13	Granulocytes	Ndufa8
1.51E-17	-0.5376077	0.01	0.149	2.14E-13	Granulocytes	Rpa2
1.52E-17	-0.4347443	0.033	0.188	2.15E-13	Granulocytes	Ssbp1
1.52E-17	-0.526149	0.065	0.24	2.15E-13	Granulocytes	Rbm5
1.54E-17	0.97638586	0.19	0.086	2.17E-13	Granulocytes	Sephs2
1.58E-17	-0.5368746	0.018	0.163	2.24E-13	Granulocytes	Tmem173
1.58E-17	-0.529823	0.014	0.156	2.24E-13	Granulocytes	Tcerg1
1.59E-17	-0.6936049	0.008	0.144	2.25E-13	Granulocytes	Sesn1
1.59E-17	-0.4171875	0.025	0.175	2.25E-13	Granulocytes	Cdc123
1.64E-17	-0.4716606	0.01	0.15	2.33E-13	Granulocytes	Plaa
1.69E-17	-0.4125325	0.051	0.22	2.40E-13	Granulocytes	Dda1
1.77E-17	-0.448815	0.041	0.202	2.51E-13	Granulocytes	Wbp4
1.79E-17	-0.5590931	0.006	0.142	2.53E-13	Granulocytes	Ier5l
1.89E-17	0.88321688	0.303	0.173	2.67E-13	Granulocytes	Plbd1
1.89E-17	-0.2810545	0.162	0.39	2.68E-13	Granulocytes	Sf1
1.90E-17	-0.6224724	0.016	0.16	2.68E-13	Granulocytes	S1pr4
1.94E-17	-0.5480479	0.043	0.204	2.75E-13	Granulocytes	Cenpa
1.98E-17	0.93885171	0.16	0.065	2.80E-13	Granulocytes	Antxr2
2.07E-17	-0.6144401	0.012	0.15	2.93E-13	Granulocytes	Rasgrp1
2.11E-17	-0.3263795	0.235	0.5	2.98E-13	Granulocytes	Arf1
2.15E-17	-0.3329534	0.104	0.305	3.04E-13	Granulocytes	Lrp10
2.25E-17	0.94625273	0.235	0.118	3.19E-13	Granulocytes	Sirpb1c
2.26E-17	-0.4387683	0.059	0.23	3.20E-13	Granulocytes	Elavl1
2.29E-17	-0.424083	0.022	0.169	3.24E-13	Granulocytes	Get4
2.31E-17	-0.5779723	0.014	0.153	3.26E-13	Granulocytes	Map4k1
2.31E-17	-0.5476898	0.029	0.179	3.27E-13	Granulocytes	Lrmp
2.33E-17	0.86885043	0.141	0.053	3.29E-13	Granulocytes	Gpr35

2.34E-17	-0.4221201	0.008	0.146	3.31E-13	Granulocytes	Lyar
2.37E-17	-0.386319	0.035	0.192	3.36E-13	Granulocytes	Utp3
2.39E-17	-0.3375478	0.096	0.289	3.38E-13	Granulocytes	Vps29
2.44E-17	-0.2827656	0.092	0.284	3.45E-13	Granulocytes	Mea1
2.60E-17	-0.4412292	0.02	0.165	3.68E-13	Granulocytes	Magohb
2.64E-17	-0.3710868	0.07	0.245	3.74E-13	Granulocytes	Mrpl24
2.67E-17	-0.4173346	0.092	0.281	3.78E-13	Granulocytes	Tap2
2.67E-17	-0.5651127	0.014	0.154	3.78E-13	Granulocytes	Lrrc8c
2.68E-17	-0.3450223	0.125	0.331	3.80E-13	Granulocytes	Pdcd10
2.80E-17	-0.539736	0.004	0.136	3.97E-13	Granulocytes	Mybbp1a
2.90E-17	-0.4061493	0.084	0.269	4.11E-13	Granulocytes	Plgrkt
2.94E-17	-0.4254155	0.033	0.187	4.16E-13	Granulocytes	Nsmce1
2.94E-17	0.76466372	0.139	0.052	4.17E-13	Granulocytes	Atp1a3
2.96E-17	-0.2787041	0.145	0.369	4.19E-13	Granulocytes	Rbm42
2.98E-17	-0.527183	0.004	0.137	4.21E-13	Granulocytes	Nsmaf
3.12E-17	-0.5311286	0.01	0.147	4.42E-13	Granulocytes	Kcnab2
3.15E-17	-0.4204871	0.035	0.191	4.46E-13	Granulocytes	Sbds
3.27E-17	-1.0312779	0.047	0.206	4.62E-13	Granulocytes	Mt1
3.45E-17	-0.4135134	0.149	0.366	4.88E-13	Granulocytes	Ctsa
3.52E-17	-0.4368953	0.059	0.227	4.99E-13	Granulocytes	Ssr3
3.59E-17	-0.808806	0.012	0.148	5.08E-13	Granulocytes	Sdc4
3.60E-17	0.93384288	0.209	0.1	5.10E-13	Granulocytes	Cfp
3.65E-17	-0.4585413	0.014	0.155	5.16E-13	Granulocytes	Acd
3.74E-17	-0.5073777	0.033	0.184	5.30E-13	Granulocytes	B230219D22
3.85E-17	-0.4513379	0.016	0.157	5.46E-13	Granulocytes	Gtf3a
3.98E-17	0.7464572	0.112	0.037	5.64E-13	Granulocytes	Cd101
4.00E-17	-0.5963786	0.01	0.147	5.67E-13	Granulocytes	Chchd10
4.08E-17	-0.3921985	0.02	0.166	5.77E-13	Granulocytes	Ap1s1
4.09E-17	-0.465257	0.076	0.252	5.79E-13	Granulocytes	Ikbkb
4.19E-17	-0.4802338	0.025	0.169	5.94E-13	Granulocytes	Mrpl51
4.21E-17	-0.5264123	0.012	0.149	5.95E-13	Granulocytes	Efr3a
4.25E-17	-0.3798972	0.02	0.165	6.01E-13	Granulocytes	Acp1
4.27E-17	1.23496733	0.29	0.174	6.05E-13	Granulocytes	Eif4ebp1
4.28E-17	-0.421173	0.031	0.182	6.07E-13	Granulocytes	Psmd12
4.32E-17	-0.6512337	0.008	0.142	6.11E-13	Granulocytes	Arap2
4.40E-17	-0.4388427	0.051	0.216	6.22E-13	Granulocytes	Sdf2
4.40E-17	-0.4515838	0.039	0.196	6.24E-13	Granulocytes	Fam162a
4.41E-17	-0.3631039	0.041	0.2	6.24E-13	Granulocytes	Asna1
4.47E-17	-0.4901105	0.027	0.175	6.32E-13	Granulocytes	Naa50
4.48E-17	-0.5634673	0.029	0.178	6.34E-13	Granulocytes	Arid5b
4.51E-17	-0.3925042	0.006	0.14	6.38E-13	Granulocytes	Qdpr
4.58E-17	-0.276957	0.168	0.401	6.49E-13	Granulocytes	Tecr
4.65E-17	-0.4594875	0.018	0.162	6.58E-13	Granulocytes	Kpnb1
5.25E-17	-0.4859436	0.012	0.149	7.43E-13	Granulocytes	Ccdc85b
5.32E-17	-0.3695317	0.072	0.248	7.53E-13	Granulocytes	Mrps18c
5.33E-17	-0.4002988	0.074	0.253	7.55E-13	Granulocytes	Mrps15
5.46E-17	-0.5028977	0.012	0.15	7.73E-13	Granulocytes	Irf2bpl
5.57E-17	-0.5057514	0.018	0.159	7.88E-13	Granulocytes	Prkacb

5.61E-17	-0.4576811	0.045	0.204	7.94E-13	Granulocytes	Rbck1
6.31E-17	-0.4696846	0.006	0.138	8.93E-13	Granulocytes	Tm2d3
6.35E-17	-0.4203382	0.027	0.175	8.99E-13	Granulocytes	Ube2e1
6.51E-17	-0.4947087	0.016	0.155	9.22E-13	Granulocytes	Luc7l
6.75E-17	1.12529124	0.254	0.14	9.56E-13	Granulocytes	Pxn
6.92E-17	-0.5117492	0.016	0.155	9.79E-13	Granulocytes	Srrt
6.98E-17	-0.425537	0.065	0.237	9.88E-13	Granulocytes	Baz1a
7.08E-17	-0.4001231	0.043	0.203	1.00E-12	Granulocytes	Supt5
7.16E-17	-0.3872695	0.055	0.221	1.01E-12	Granulocytes	Polr2c
7.17E-17	-0.3781504	0.057	0.227	1.01E-12	Granulocytes	Rnf166
7.67E-17	-0.4675725	0.008	0.14	1.09E-12	Granulocytes	Mrpl11
7.67E-17	-0.4473235	0.006	0.137	1.09E-12	Granulocytes	Tex264
8.07E-17	-0.6357455	0.045	0.205	1.14E-12	Granulocytes	Eea1
8.07E-17	-0.4157835	0.008	0.141	1.14E-12	Granulocytes	Faim
8.12E-17	-0.3216035	0.045	0.204	1.15E-12	Granulocytes	Commd1
8.25E-17	-0.4807921	0.022	0.164	1.17E-12	Granulocytes	Pqbp1
8.26E-17	-0.552436	0.008	0.141	1.17E-12	Granulocytes	Trim35
8.62E-17	-0.4541433	0.022	0.165	1.22E-12	Granulocytes	Yif1b
8.69E-17	1.36582122	0.417	0.313	1.23E-12	Granulocytes	Cxcr4
8.79E-17	-0.4164436	0.016	0.155	1.24E-12	Granulocytes	Comt
8.80E-17	1.06901136	0.372	0.254	1.25E-12	Granulocytes	Syk
9.05E-17	-0.4041656	0.047	0.207	1.28E-12	Granulocytes	Pin4
9.07E-17	-0.4512931	0.321	0.587	1.28E-12	Granulocytes	Tmem50a
9.08E-17	-0.5061757	0.022	0.166	1.29E-12	Granulocytes	Eml4
9.12E-17	1.08707815	0.286	0.17	1.29E-12	Granulocytes	Nadk
9.18E-17	1.01851457	0.211	0.104	1.30E-12	Granulocytes	Dck
9.39E-17	-0.3618809	0.198	0.43	1.33E-12	Granulocytes	Ubxn1
9.57E-17	0.68597873	0.742	0.74	1.35E-12	Granulocytes	Ier2
9.62E-17	-0.4071901	0.016	0.155	1.36E-12	Granulocytes	2210016F16F
9.98E-17	-0.4954009	0.022	0.163	1.41E-12	Granulocytes	Psmd1
9.99E-17	-0.3631651	0.016	0.155	1.41E-12	Granulocytes	Abhd17a
1.06E-16	-0.5244818	0.029	0.173	1.51E-12	Granulocytes	Bcl7b
1.08E-16	-0.5440498	0.014	0.15	1.52E-12	Granulocytes	Phf6
1.08E-16	-0.4353173	0.006	0.136	1.53E-12	Granulocytes	Ccs
1.13E-16	-0.3819702	0.031	0.181	1.60E-12	Granulocytes	Idh3g
1.16E-16	-0.3434812	0.123	0.325	1.64E-12	Granulocytes	H13
1.19E-16	1.0178348	0.198	0.095	1.69E-12	Granulocytes	Rassf3
1.20E-16	-0.3425546	0.057	0.222	1.69E-12	Granulocytes	Svbp
1.21E-16	-0.5728869	0.008	0.138	1.72E-12	Granulocytes	Klhl6
1.22E-16	-0.4808503	0.018	0.158	1.73E-12	Granulocytes	Akap8
1.23E-16	-0.5089136	0.012	0.147	1.74E-12	Granulocytes	Siah2
1.27E-16	-0.493137	0.025	0.168	1.80E-12	Granulocytes	Hnrnpr
1.29E-16	-0.4343261	0.053	0.218	1.83E-12	Granulocytes	Itpkb
1.39E-16	-0.5414091	0.041	0.196	1.97E-12	Granulocytes	Ptpn11
1.42E-16	-0.3787304	0.033	0.181	2.02E-12	Granulocytes	Polr2i
1.43E-16	-0.3333571	0.07	0.244	2.03E-12	Granulocytes	BC004004
1.54E-16	-0.5776765	0.006	0.136	2.18E-12	Granulocytes	Gm19585
1.54E-16	-0.428479	0.027	0.17	2.18E-12	Granulocytes	Pbdc1

1.59E-16	-0.535869	0.358	0.602	2.25E-12	Granulocytes	Cd37
1.59E-16	-0.520931	0.004	0.131	2.26E-12	Granulocytes	11-Sep
1.60E-16	-0.4395119	0.018	0.157	2.26E-12	Granulocytes	Mad2l1bp
1.62E-16	-0.4055099	0.029	0.174	2.29E-12	Granulocytes	Zcchc17
1.66E-16	-0.4932795	0.01	0.142	2.35E-12	Granulocytes	Rftn1
1.77E-16	-1.0632487	0.027	0.167	2.51E-12	Granulocytes	Mafb
1.78E-16	-0.3898989	0.031	0.179	2.52E-12	Granulocytes	Stx4a
1.80E-16	-0.4538314	0.008	0.139	2.55E-12	Granulocytes	Prkcsh
1.81E-16	-0.3940614	0.065	0.234	2.57E-12	Granulocytes	Sp110
1.82E-16	-0.3160285	0.041	0.194	2.58E-12	Granulocytes	C1d
1.86E-16	-0.5490781	0.016	0.151	2.63E-12	Granulocytes	Ilf2
1.87E-16	-0.4068008	0.031	0.178	2.65E-12	Granulocytes	Mrps17
1.90E-16	-0.4777573	0.025	0.166	2.70E-12	Granulocytes	Serpinb6a
1.94E-16	-0.3388157	0.045	0.202	2.74E-12	Granulocytes	Vdac1
1.97E-16	-0.4892194	0.01	0.141	2.79E-12	Granulocytes	Me2
1.99E-16	-0.30263	0.104	0.294	2.81E-12	Granulocytes	Sod2
1.99E-16	-0.4554738	0.049	0.208	2.82E-12	Granulocytes	Cbx3
2.00E-16	-0.460522	0.115	0.306	2.83E-12	Granulocytes	Mycbp2
2.06E-16	-0.4225947	0.057	0.221	2.91E-12	Granulocytes	Smc1a
2.14E-16	-0.4902219	0.012	0.145	3.03E-12	Granulocytes	Aars
2.17E-16	-0.430724	0.016	0.152	3.08E-12	Granulocytes	Gars
2.23E-16	-0.4168636	0.045	0.2	3.16E-12	Granulocytes	Ebna1bp2
2.28E-16	-0.5340358	0.008	0.136	3.23E-12	Granulocytes	Gna15
2.29E-16	-0.3479492	0.033	0.181	3.24E-12	Granulocytes	Arl1
2.29E-16	-0.4709541	0.051	0.207	3.25E-12	Granulocytes	Rdx
2.30E-16	-0.348821	0.082	0.259	3.25E-12	Granulocytes	Mrpl57
2.31E-16	-0.4733704	0.008	0.137	3.27E-12	Granulocytes	Prpf19
2.61E-16	-1.5901957	0.016	0.15	3.70E-12	Granulocytes	Cxcl9
2.64E-16	-0.5508087	0.008	0.137	3.73E-12	Granulocytes	Cers4
2.64E-16	-0.4891855	0.012	0.144	3.74E-12	Granulocytes	Mak16
2.65E-16	-0.4169953	0.049	0.205	3.75E-12	Granulocytes	Dazap1
2.66E-16	-0.5461947	0.006	0.133	3.76E-12	Granulocytes	Sesn3
2.66E-16	-0.3226211	0.063	0.228	3.76E-12	Granulocytes	Polr2g
2.68E-16	-0.4356225	0.01	0.14	3.79E-12	Granulocytes	Mrpl35
2.73E-16	-0.7115225	0.008	0.137	3.87E-12	Granulocytes	Maf
2.84E-16	-0.5744697	0.033	0.179	4.02E-12	Granulocytes	Ero1lb
2.85E-16	-0.7589724	0.008	0.137	4.04E-12	Granulocytes	Cxcl16
2.89E-16	-0.4425454	0.012	0.143	4.09E-12	Granulocytes	Cltb
2.92E-16	-0.3781578	0.027	0.171	4.13E-12	Granulocytes	Eif2s3x
2.99E-16	-0.3018392	0.274	0.54	4.24E-12	Granulocytes	Tomm7
3.02E-16	-0.4161078	0.029	0.172	4.28E-12	Granulocytes	Ogfr
3.12E-16	-0.5482292	0.027	0.165	4.42E-12	Granulocytes	Elovl5
3.17E-16	-0.69523	0.012	0.143	4.49E-12	Granulocytes	Ms4a6d
3.18E-16	-0.9900364	0.01	0.138	4.50E-12	Granulocytes	Fcer2a
3.25E-16	-0.4345515	0.031	0.176	4.60E-12	Granulocytes	Nmt1
3.50E-16	0.7369804	0.129	0.048	4.95E-12	Granulocytes	St3gal5
3.61E-16	-0.4219656	0.016	0.152	5.11E-12	Granulocytes	E2f4
3.68E-16	1.142341	0.556	0.525	5.21E-12	Granulocytes	Ppp1r15a

3.74E-16	-0.9403096	0.004	0.129	5.30E-12	Granulocytes	Mzb1
3.78E-16	-0.3221362	0.041	0.194	5.36E-12	Granulocytes	Smim11
3.91E-16	-0.2890557	0.221	0.461	5.54E-12	Granulocytes	Tnfaip3
3.98E-16	-0.6221361	0.008	0.135	5.63E-12	Granulocytes	Tcf4
4.12E-16	-0.4681632	0.014	0.146	5.83E-12	Granulocytes	Gsto1
4.16E-16	-0.4515588	0.02	0.157	5.89E-12	Granulocytes	Mtx1
4.17E-16	-0.553333	0.006	0.131	5.91E-12	Granulocytes	Arl5c
4.18E-16	1.1624312	0.317	0.207	5.92E-12	Granulocytes	Anxa11
4.32E-16	-0.3451594	0.063	0.226	6.11E-12	Granulocytes	Denr
4.34E-16	-0.4053565	0.008	0.136	6.14E-12	Granulocytes	Clpp
4.40E-16	1.29527846	0.313	0.203	6.23E-12	Granulocytes	7-Mar
4.44E-16	-0.7731741	0.004	0.128	6.28E-12	Granulocytes	Rgs16
4.45E-16	-0.5610492	0.02	0.156	6.29E-12	Granulocytes	Piezo1
4.63E-16	-0.475103	0.051	0.206	6.55E-12	Granulocytes	Prpf8
4.64E-16	-0.713607	0.01	0.139	6.56E-12	Granulocytes	Bag3
4.76E-16	-0.4787249	0.02	0.156	6.74E-12	Granulocytes	Otulin
4.93E-16	-0.5011126	0.01	0.138	6.98E-12	Granulocytes	Rabggtb
4.93E-16	-0.5280754	0.016	0.15	6.98E-12	Granulocytes	Galnt6
5.06E-16	-0.3338842	0.151	0.36	7.16E-12	Granulocytes	Tapbp
5.15E-16	-0.5215458	0.004	0.127	7.30E-12	Granulocytes	Txndc5
5.21E-16	-0.3035625	0.086	0.266	7.37E-12	Granulocytes	Maf1
5.21E-16	-0.3585701	0.041	0.192	7.37E-12	Granulocytes	Siva1
5.27E-16	1.33547449	0.286	0.172	7.47E-12	Granulocytes	Rtp4
5.41E-16	-0.4754583	0.008	0.135	7.66E-12	Granulocytes	Dynll2
5.54E-16	-0.4752541	0.037	0.183	7.84E-12	Granulocytes	0610010K14I
5.61E-16	-0.3944539	0.027	0.168	7.94E-12	Granulocytes	Copb2
5.62E-16	-0.3667803	0.047	0.201	7.96E-12	Granulocytes	Emc2
5.82E-16	-0.4445393	0.016	0.149	8.24E-12	Granulocytes	Psmd9
5.95E-16	-0.4530267	0.004	0.126	8.43E-12	Granulocytes	Eef1e1
6.08E-16	-0.3504416	0.055	0.214	8.61E-12	Granulocytes	Ufm1
6.17E-16	-0.4537538	0.014	0.144	8.73E-12	Granulocytes	Cnih1
6.17E-16	-0.334043	0.194	0.421	8.73E-12	Granulocytes	Sf3b2
6.27E-16	-0.3964581	0.025	0.162	8.87E-12	Granulocytes	Ormdl2
6.27E-16	-0.3552272	0.121	0.313	8.88E-12	Granulocytes	Tmod3
6.53E-16	-0.5149803	0.209	0.409	9.25E-12	Granulocytes	Ccl4
6.57E-16	-0.4293787	0.012	0.141	9.31E-12	Granulocytes	Vma21
6.69E-16	-0.4351243	0.112	0.295	9.47E-12	Granulocytes	Crlf3
6.70E-16	-0.2509688	0.082	0.257	9.48E-12	Granulocytes	Lamtor5
6.70E-16	-0.4712148	0.008	0.134	9.49E-12	Granulocytes	Dhps
6.89E-16	-0.4069449	0.301	0.549	9.75E-12	Granulocytes	Prelid1
6.91E-16	0.73047187	0.579	0.531	9.79E-12	Granulocytes	Pkm
6.98E-16	-0.3676582	0.016	0.15	9.88E-12	Granulocytes	Mrto4
7.02E-16	-0.4372104	0.004	0.127	9.94E-12	Granulocytes	Pop4
7.21E-16	-0.6100228	0.014	0.146	1.02E-11	Granulocytes	Lmna
7.39E-16	-0.3471683	0.067	0.235	1.05E-11	Granulocytes	Nmi
7.52E-16	-0.3953196	0.031	0.172	1.07E-11	Granulocytes	Lsm1
7.57E-16	-0.346414	0.01	0.138	1.07E-11	Granulocytes	Hscb
7.81E-16	0.91287552	0.145	0.059	1.11E-11	Granulocytes	Tlr13

8.06E-16	-0.312819	0.098	0.282	1.14E-11	Granulocytes	Mrpl20
8.07E-16	-0.348548	0.051	0.206	1.14E-11	Granulocytes	Szrd1
8.10E-16	-0.4800181	0.039	0.185	1.15E-11	Granulocytes	Prpf4b
8.12E-16	-0.299625	0.28	0.55	1.15E-11	Granulocytes	Grcc10
8.24E-16	-0.3979808	0.037	0.184	1.17E-11	Granulocytes	Fxr1
8.33E-16	-0.4969877	0.022	0.157	1.18E-11	Granulocytes	Oxct1
8.37E-16	-0.3785495	0.037	0.183	1.18E-11	Granulocytes	Dpm2
8.55E-16	-0.4090065	0.014	0.143	1.21E-11	Granulocytes	Mrpl2
8.66E-16	-0.5074514	0.006	0.13	1.23E-11	Granulocytes	Sla2
8.68E-16	-0.3962064	0.037	0.184	1.23E-11	Granulocytes	Gtf2f1
9.13E-16	-0.3747452	0.022	0.157	1.29E-11	Granulocytes	Wdr61
9.75E-16	-0.4163647	0.004	0.125	1.38E-11	Granulocytes	Il3ra
1.01E-15	-1.0045277	0.006	0.128	1.44E-11	Granulocytes	Il7r
1.02E-15	-0.5084646	0.018	0.149	1.45E-11	Granulocytes	Srebf2
1.04E-15	-0.4144654	0.029	0.167	1.48E-11	Granulocytes	Elof1
1.06E-15	-0.354975	0.033	0.178	1.50E-11	Granulocytes	Psen2
1.09E-15	-0.5797223	0	0.118	1.54E-11	Granulocytes	Tbx21
1.09E-15	-0.4288438	0.016	0.147	1.55E-11	Granulocytes	Mrpl9
1.13E-15	-0.4961544	0.055	0.213	1.59E-11	Granulocytes	Pik3r1
1.13E-15	-0.3063507	0.166	0.379	1.61E-11	Granulocytes	Uqcr11
1.18E-15	-0.4530498	0.018	0.15	1.67E-11	Granulocytes	Flt3l
1.22E-15	-0.4542289	0.022	0.156	1.73E-11	Granulocytes	Gtf3c6
1.24E-15	-0.7013605	0.012	0.138	1.76E-11	Granulocytes	Blnk
1.25E-15	-0.4403653	0.01	0.136	1.77E-11	Granulocytes	Rce1
1.25E-15	-0.8246583	0.012	0.139	1.77E-11	Granulocytes	Tmem176b
1.28E-15	-0.4196861	0.006	0.128	1.81E-11	Granulocytes	Rpp25l
1.28E-15	-0.8645283	0.008	0.132	1.82E-11	Granulocytes	Gzmk
1.29E-15	-0.8472212	0.006	0.127	1.82E-11	Granulocytes	Blk
1.32E-15	-0.7561093	0.002	0.12	1.87E-11	Granulocytes	Serpinb6b
1.35E-15	-0.3600989	0.018	0.152	1.91E-11	Granulocytes	Nsmce2
1.40E-15	-0.3430261	0.059	0.22	1.99E-11	Granulocytes	Wdr33
1.42E-15	-0.6946571	0.012	0.137	2.01E-11	Granulocytes	Hvcn1
1.45E-15	-0.4021308	0.076	0.245	2.06E-11	Granulocytes	Psip1
1.46E-15	-0.3014482	0.07	0.234	2.06E-11	Granulocytes	Eif1b
1.47E-15	-0.509644	0.022	0.155	2.09E-11	Granulocytes	Rsbn1
1.50E-15	1.29162547	0.209	0.106	2.12E-11	Granulocytes	Basp1
1.51E-15	1.00094122	0.213	0.11	2.14E-11	Granulocytes	Pnkp
1.52E-15	-0.4202891	0.043	0.19	2.15E-11	Granulocytes	Erp44
1.54E-15	-0.5333878	0.01	0.134	2.18E-11	Granulocytes	Klk8
1.59E-15	-0.343667	0.057	0.216	2.25E-11	Granulocytes	Rad21
1.60E-15	-0.4359497	0.006	0.127	2.26E-11	Granulocytes	Gar1
1.60E-15	-0.4432407	0.006	0.128	2.27E-11	Granulocytes	Atic
1.61E-15	-0.4750552	0.031	0.17	2.28E-11	Granulocytes	Smc3
1.62E-15	-0.3017088	0.055	0.21	2.29E-11	Granulocytes	Tmco1
1.72E-15	-0.4644877	0.018	0.149	2.44E-11	Granulocytes	Yrdc
1.74E-15	-0.4908581	0.006	0.127	2.46E-11	Granulocytes	Lman1
1.80E-15	-0.4085983	0.035	0.178	2.55E-11	Granulocytes	Hmga1
1.81E-15	-0.4610749	0.008	0.131	2.56E-11	Granulocytes	Ncor2

1.88E-15	-0.394659	0.01	0.135	2.66E-11	Granulocytes	Lage3
1.93E-15	-0.398016	0.022	0.157	2.73E-11	Granulocytes	Aggf1
1.94E-15	-0.505121	0.041	0.185	2.75E-11	Granulocytes	Snx9
1.94E-15	0.42309331	0.838	0.801	2.75E-11	Granulocytes	Cyba
1.95E-15	-0.4976621	0.039	0.181	2.76E-11	Granulocytes	Nasp
1.96E-15	-0.468544	0.016	0.143	2.78E-11	Granulocytes	Smarcb1
1.97E-15	-0.3285849	0.006	0.127	2.79E-11	Granulocytes	Polr2h
2.00E-15	-0.5172475	0.018	0.147	2.83E-11	Granulocytes	Supt16
2.21E-15	-0.4591344	0.006	0.127	3.13E-11	Granulocytes	Cd2ap
2.21E-15	-0.2829688	0.092	0.268	3.14E-11	Granulocytes	Chic2
2.24E-15	-0.5596312	0.01	0.133	3.17E-11	Granulocytes	Gbp4
2.25E-15	1.0142618	0.309	0.197	3.19E-11	Granulocytes	Dok3
2.31E-15	-0.3463731	0.025	0.159	3.27E-11	Granulocytes	Alyref
2.32E-15	-0.292555	0.057	0.215	3.28E-11	Granulocytes	Pin1
2.33E-15	-0.3951061	0.02	0.151	3.30E-11	Granulocytes	Mrpl41
2.39E-15	-0.3860027	0.045	0.195	3.38E-11	Granulocytes	Gpbp1
2.62E-15	-0.4790941	0.008	0.129	3.71E-11	Granulocytes	Eftud2
2.65E-15	-0.427858	0.047	0.195	3.75E-11	Granulocytes	1700123O20I
2.65E-15	-0.3663555	0.018	0.149	3.75E-11	Granulocytes	Josd2
2.73E-15	-0.4275801	0.004	0.122	3.86E-11	Granulocytes	Rpa1
2.77E-15	-0.5830662	0.566	0.758	3.92E-11	Granulocytes	Ucp2
2.81E-15	-0.4150922	0.039	0.182	3.98E-11	Granulocytes	Susd3
2.81E-15	-0.5060406	0.01	0.134	3.98E-11	Granulocytes	Dnmt1
2.88E-15	-0.7138943	0.02	0.149	4.07E-11	Granulocytes	Rrad
2.89E-15	-0.4089091	0	0.114	4.09E-11	Granulocytes	Bcap29
2.99E-15	-0.4315178	0.025	0.157	4.23E-11	Granulocytes	Parl
3.08E-15	-0.4440009	0.037	0.177	4.36E-11	Granulocytes	5-Mar
3.15E-15	-0.6988011	0.02	0.149	4.47E-11	Granulocytes	Ccr2
3.27E-15	-0.3971175	0.012	0.137	4.63E-11	Granulocytes	Trnau1ap
3.28E-15	-0.3527426	0.074	0.236	4.65E-11	Granulocytes	Mapk1
3.29E-15	0.40353311	0.857	0.863	4.65E-11	Granulocytes	Coro1a
3.31E-15	-0.3676042	0.02	0.15	4.69E-11	Granulocytes	Sdhc
3.37E-15	-0.4954822	0.012	0.135	4.78E-11	Granulocytes	Ptpn7
3.54E-15	-0.4369035	0.012	0.135	5.01E-11	Granulocytes	Ppie
3.64E-15	-0.46831	0.025	0.155	5.15E-11	Granulocytes	Mgat2
3.69E-15	-0.4302024	0.025	0.157	5.23E-11	Granulocytes	Usp1
3.80E-15	-0.3961598	0.043	0.188	5.38E-11	Granulocytes	Rbm17
3.83E-15	-0.4758337	0.035	0.173	5.43E-11	Granulocytes	Blvrb
3.84E-15	-0.3349051	0.029	0.166	5.43E-11	Granulocytes	Anapc16
3.84E-15	-0.5253232	0.004	0.122	5.44E-11	Granulocytes	Cd226
3.99E-15	-0.3201976	0.047	0.195	5.65E-11	Granulocytes	Trappc1
4.15E-15	-0.3931534	0.047	0.193	5.87E-11	Granulocytes	Mrps23
4.17E-15	-0.4896107	0.004	0.12	5.91E-11	Granulocytes	Fam189b
4.28E-15	-0.3815022	0.035	0.175	6.06E-11	Granulocytes	Tpd52l2
4.34E-15	-0.4073579	0.047	0.194	6.14E-11	Granulocytes	Ep400
4.37E-15	-0.4690796	0.008	0.128	6.18E-11	Granulocytes	Mrpl38
4.38E-15	-0.3679182	0.098	0.274	6.21E-11	Granulocytes	Tnfaip8
4.57E-15	-0.4207598	0.012	0.134	6.47E-11	Granulocytes	Tmem242

4.58E-15	-0.4183432	0.031	0.168	6.48E-11	Granulocytes	Ppil2
4.63E-15	-0.4428217	0.601	0.82	6.56E-11	Granulocytes	Laptm5
4.68E-15	-0.4539734	0.072	0.23	6.63E-11	Granulocytes	Bptf
4.74E-15	1.04261343	0.397	0.297	6.71E-11	Granulocytes	Tspan13
4.76E-15	-0.4265837	0.025	0.157	6.74E-11	Granulocytes	Xaf1
4.85E-15	-0.3643152	0.025	0.156	6.87E-11	Granulocytes	Pabpn1
4.89E-15	-0.4292242	0.049	0.195	6.92E-11	Granulocytes	Hdlbp
4.95E-15	-0.4656396	0.037	0.179	7.01E-11	Granulocytes	Huwe1
4.96E-15	-0.3688858	0.037	0.177	7.02E-11	Granulocytes	Dcps
4.97E-15	-0.3252627	0.078	0.243	7.04E-11	Granulocytes	Fli1
4.98E-15	-0.4701906	0.045	0.19	7.05E-11	Granulocytes	Plec
5.02E-15	-0.2809942	0.045	0.192	7.11E-11	Granulocytes	Eif4e
5.18E-15	-0.3934915	0.027	0.161	7.34E-11	Granulocytes	Cdv3
5.19E-15	-0.4204651	0.012	0.134	7.35E-11	Granulocytes	Tomm40
5.28E-15	-0.4610447	0.033	0.17	7.47E-11	Granulocytes	Tnks2
5.44E-15	-0.641089	0.008	0.126	7.71E-11	Granulocytes	Trp53i11
5.50E-15	-0.4944496	0.053	0.202	7.78E-11	Granulocytes	Rinl
5.52E-15	-0.3105871	0.094	0.267	7.81E-11	Granulocytes	Psmd13
5.60E-15	-0.3845584	0.072	0.23	7.93E-11	Granulocytes	Zdhhc20
5.65E-15	-0.285958	0.063	0.221	8.01E-11	Granulocytes	Dap
5.66E-15	-0.3833584	0.035	0.172	8.01E-11	Granulocytes	Nucb1
5.67E-15	1.00209135	0.221	0.117	8.02E-11	Granulocytes	Slc11a1
5.68E-15	-0.4003648	0.041	0.183	8.04E-11	Granulocytes	Sirt2
5.78E-15	-0.4260231	0.027	0.16	8.18E-11	Granulocytes	Hadhb
5.85E-15	-0.38267	0.002	0.116	8.28E-11	Granulocytes	Ybx3
5.91E-15	-0.3833245	0.002	0.116	8.36E-11	Granulocytes	Hint2
5.92E-15	-0.4405529	0.065	0.22	8.39E-11	Granulocytes	Hopx
6.02E-15	-0.3874615	0.01	0.131	8.52E-11	Granulocytes	Nenf
6.14E-15	-0.4783149	0.014	0.138	8.69E-11	Granulocytes	Tspan32
6.30E-15	0.90980316	0.209	0.105	8.91E-11	Granulocytes	Actn1
6.36E-15	-0.3657131	0.039	0.179	9.01E-11	Granulocytes	B3gnt2
6.48E-15	-0.434305	0.014	0.136	9.17E-11	Granulocytes	Tufm
6.63E-15	-0.4464241	0.016	0.139	9.39E-11	Granulocytes	Ubl4a
6.64E-15	-0.462014	0.002	0.115	9.39E-11	Granulocytes	Tex30
6.78E-15	-0.4278233	0.051	0.199	9.60E-11	Granulocytes	Nap1l4
6.90E-15	-0.4238311	0.012	0.134	9.77E-11	Granulocytes	D5Ertd579e
7.17E-15	-0.5757207	0.027	0.158	1.01E-10	Granulocytes	Ddit4
7.19E-15	-0.420383	0.016	0.14	1.02E-10	Granulocytes	Pdcd2
7.26E-15	-0.4072494	0.008	0.126	1.03E-10	Granulocytes	Dcp2
7.31E-15	-0.4399912	0.016	0.14	1.04E-10	Granulocytes	Sf3a3
7.40E-15	-0.4172825	0.018	0.145	1.05E-10	Granulocytes	Cops2
7.74E-15	-0.3558788	0.031	0.166	1.10E-10	Granulocytes	Nosip
7.80E-15	-0.4540722	0.059	0.212	1.10E-10	Granulocytes	Pcmtd1
8.17E-15	-0.6465001	0.029	0.161	1.16E-10	Granulocytes	Dmxl1
8.29E-15	0.91063984	0.151	0.065	1.17E-10	Granulocytes	Rab31
8.31E-15	-0.5117152	0.07	0.227	1.18E-10	Granulocytes	Rnf125
8.43E-15	-0.4949726	0.055	0.202	1.19E-10	Granulocytes	Dgka
8.56E-15	-0.4414794	0.02	0.147	1.21E-10	Granulocytes	Rfk

8.61E-15	-0.3660773	0.035	0.172	1.22E-10	Granulocytes	Gusb
8.62E-15	-0.5363667	0.01	0.129	1.22E-10	Granulocytes	Gimap8
8.64E-15	-0.4452371	0.012	0.132	1.22E-10	Granulocytes	Vcpkmt
8.77E-15	-0.3300747	0.061	0.214	1.24E-10	Granulocytes	Ube2f
8.93E-15	-0.4949713	0.022	0.149	1.26E-10	Granulocytes	Wasl
8.98E-15	-0.4476202	0.02	0.146	1.27E-10	Granulocytes	Nudcd3
9.05E-15	-0.4128762	0.022	0.151	1.28E-10	Granulocytes	Stt3a
9.33E-15	1.0723055	0.262	0.155	1.32E-10	Granulocytes	Skap2
9.52E-15	-0.3821402	0.074	0.231	1.35E-10	Granulocytes	Sharpin
9.53E-15	-0.288043	0.043	0.187	1.35E-10	Granulocytes	Glrx2
9.71E-15	-0.4275661	0.006	0.121	1.37E-10	Granulocytes	Pea15a
9.90E-15	-0.4692466	0.016	0.14	1.40E-10	Granulocytes	2310033P09R
1.00E-14	-0.3352096	0.031	0.165	1.42E-10	Granulocytes	Cs
1.01E-14	-0.42367	0.049	0.194	1.43E-10	Granulocytes	Dync1h1
1.06E-14	-0.5196325	0.008	0.126	1.50E-10	Granulocytes	Ppp1r16b
1.07E-14	-0.3471724	0.012	0.134	1.51E-10	Granulocytes	1500011B03R
1.09E-14	-0.3812651	0.012	0.133	1.54E-10	Granulocytes	Nubp2
1.09E-14	-0.4035364	0.008	0.125	1.55E-10	Granulocytes	Capn2
1.09E-14	-0.4158657	0.016	0.141	1.55E-10	Granulocytes	Errfi1
1.09E-14	-0.4412548	0.02	0.146	1.55E-10	Granulocytes	Ddb1
1.11E-14	-0.4333096	0.016	0.139	1.58E-10	Granulocytes	Tigd2
1.13E-14	-0.4604356	0.025	0.154	1.60E-10	Granulocytes	Spint2
1.17E-14	-0.4841965	0.022	0.15	1.66E-10	Granulocytes	Csnk1g3
1.18E-14	-0.3857485	0.027	0.157	1.66E-10	Granulocytes	Atraid
1.18E-14	-0.4241492	0.02	0.145	1.68E-10	Granulocytes	Pigt
1.23E-14	-0.5254079	0.041	0.18	1.74E-10	Granulocytes	Irf5
1.23E-14	-0.322253	0.029	0.161	1.74E-10	Granulocytes	Rab18
1.25E-14	-0.4632843	0.006	0.12	1.77E-10	Granulocytes	Dok2
1.37E-14	-0.3280896	0.029	0.16	1.94E-10	Granulocytes	Imp4
1.38E-14	-0.4262014	0.059	0.21	1.95E-10	Granulocytes	Nfkb1
1.38E-14	-0.3117861	0.129	0.315	1.95E-10	Granulocytes	Coro1b
1.39E-14	-0.42639	0.004	0.117	1.97E-10	Granulocytes	Psmg2
1.39E-14	-0.3500137	0.031	0.164	1.97E-10	Granulocytes	Cops5
1.41E-14	-0.3896253	0.016	0.139	2.00E-10	Granulocytes	Bcl7c
1.43E-14	-0.459161	0.012	0.131	2.02E-10	Granulocytes	Naa15
1.44E-14	-0.4174831	0.025	0.153	2.04E-10	Granulocytes	Ciapin1
1.49E-14	-0.3755035	0.016	0.139	2.11E-10	Granulocytes	Gmps
1.49E-14	-0.4015529	0.002	0.113	2.11E-10	Granulocytes	Afg3l1
1.52E-14	-0.3941973	0.039	0.176	2.15E-10	Granulocytes	Rraga
1.52E-14	-0.2876532	0.047	0.191	2.16E-10	Granulocytes	Mrpl17
1.53E-14	-0.3167461	0.039	0.176	2.16E-10	Granulocytes	Stx8
1.55E-14	-0.3173659	0.086	0.252	2.19E-10	Granulocytes	Scamp2
1.59E-14	-0.3810206	0.029	0.16	2.25E-10	Granulocytes	Dguok
1.61E-14	-0.512316	0.006	0.12	2.28E-10	Granulocytes	Jakmip1
1.62E-14	-0.5074142	0.025	0.149	2.30E-10	Granulocytes	Galnt1
1.63E-14	-0.331095	0.02	0.146	2.31E-10	Granulocytes	Emc8
1.64E-14	-0.4442173	0.029	0.159	2.32E-10	Granulocytes	Oat
1.66E-14	1.10810053	0.354	0.258	2.36E-10	Granulocytes	Ppp1r2

1.67E-14	-0.2514475	0.141	0.336	2.37E-10	Granulocytes	Sys1
1.69E-14	-0.3938455	0.02	0.145	2.39E-10	Granulocytes	Abhd12
1.78E-14	-0.2952028	0.057	0.208	2.53E-10	Granulocytes	Anapc13
1.91E-14	-0.5131954	0.022	0.147	2.70E-10	Granulocytes	BC028528
1.93E-14	-0.357717	0.096	0.266	2.73E-10	Granulocytes	Gpr65
1.95E-14	-0.3981168	0.002	0.112	2.75E-10	Granulocytes	Psph
1.97E-14	0.77415309	0.395	0.277	2.79E-10	Granulocytes	Emb
2.00E-14	-0.380828	0.008	0.124	2.83E-10	Granulocytes	Rbm28
2.02E-14	-0.4532236	0.008	0.123	2.86E-10	Granulocytes	Lime1
2.06E-14	-0.435281	0.037	0.171	2.91E-10	Granulocytes	Dnttip2
2.10E-14	-0.4033798	0.014	0.134	2.97E-10	Granulocytes	Hint3
2.15E-14	-0.6869728	0.004	0.115	3.05E-10	Granulocytes	Ccr8
2.16E-14	-0.4705055	0.004	0.115	3.06E-10	Granulocytes	Mdfic
2.20E-14	-0.4221058	0.037	0.171	3.11E-10	Granulocytes	Kmt2a
2.21E-14	-0.810489	0.01	0.125	3.13E-10	Granulocytes	Fcrla
2.27E-14	-0.3851426	0.029	0.157	3.22E-10	Granulocytes	Tmem11
2.28E-14	-0.5405519	0.012	0.129	3.23E-10	Granulocytes	Nup210
2.40E-14	-0.3214354	0.029	0.159	3.40E-10	Granulocytes	Commd6
2.44E-14	-0.4970794	0.012	0.129	3.45E-10	Granulocytes	Cdk6
2.46E-14	-0.4071232	0.025	0.149	3.49E-10	Granulocytes	Glo1
2.47E-14	-0.8041172	0.004	0.115	3.50E-10	Granulocytes	Serpinb9
2.48E-14	-0.4285065	0.006	0.118	3.52E-10	Granulocytes	Smco4
2.48E-14	-0.3835141	0.022	0.147	3.52E-10	Granulocytes	Rsl24d1
2.49E-14	-0.3778597	0.025	0.151	3.52E-10	Granulocytes	Commd2
2.52E-14	-0.36742	0.02	0.144	3.56E-10	Granulocytes	Wdr43
2.53E-14	-0.4796986	0.016	0.135	3.58E-10	Granulocytes	Mphosph10
2.54E-14	-0.3800211	0.004	0.115	3.60E-10	Granulocytes	Elp5
2.55E-14	-0.5625173	0.01	0.126	3.60E-10	Granulocytes	Fcgr1
2.55E-14	-0.3873974	0.022	0.147	3.61E-10	Granulocytes	Eif1ad
2.56E-14	-0.487828	0.053	0.194	3.63E-10	Granulocytes	Amd1
2.59E-14	-0.4280832	0.043	0.181	3.67E-10	Granulocytes	Dcaf7
2.60E-14	-0.3135309	0.031	0.164	3.68E-10	Granulocytes	Chtop
2.68E-14	-0.4348525	0.059	0.207	3.80E-10	Granulocytes	Ifnar1
2.69E-14	0.49338446	0.957	0.961	3.81E-10	Granulocytes	H3f3b
2.77E-14	-0.3599445	0.004	0.114	3.92E-10	Granulocytes	Mrps30
2.79E-14	-0.4833359	0.033	0.164	3.95E-10	Granulocytes	P4ha1
2.86E-14	-0.4523979	0.012	0.13	4.05E-10	Granulocytes	Cep57
3.17E-14	1.00063054	0.264	0.157	4.48E-10	Granulocytes	Gm26740
3.21E-14	-0.4056138	0.012	0.129	4.55E-10	Granulocytes	Acat1
3.25E-14	-0.2963849	0.045	0.184	4.60E-10	Granulocytes	Ppp2r2a
3.31E-14	-0.4072056	0.027	0.153	4.68E-10	Granulocytes	Uba2
3.49E-14	-0.354883	0.053	0.196	4.95E-10	Granulocytes	Tiprl
3.57E-14	-0.4904645	0.012	0.129	5.05E-10	Granulocytes	Cx3cr1
3.58E-14	-0.4228959	0.047	0.186	5.07E-10	Granulocytes	Pbrm1
3.59E-14	-0.3442133	0.047	0.186	5.09E-10	Granulocytes	Emc3
3.61E-14	-0.26788	0.057	0.204	5.10E-10	Granulocytes	Gtf2a2
3.62E-14	-0.4379648	0.049	0.189	5.12E-10	Granulocytes	Krit1
3.72E-14	-0.2642454	0.049	0.193	5.26E-10	Granulocytes	Cdc26

3.74E-14	-0.2857721	0.043	0.183	5.30E-10	Granulocytes	Pfkp
3.86E-14	-0.5793218	0.014	0.131	5.47E-10	Granulocytes	Traf4
3.89E-14	-0.3624406	0.006	0.118	5.50E-10	Granulocytes	Mrps9
4.03E-14	-0.3798887	0.092	0.254	5.71E-10	Granulocytes	Nfatc1
4.06E-14	-0.3287581	0.061	0.211	5.75E-10	Granulocytes	Nxf1
4.12E-14	-0.2960366	0.049	0.19	5.83E-10	Granulocytes	Otub1
4.16E-14	-0.445485	0.014	0.132	5.89E-10	Granulocytes	Ccdc117
4.19E-14	-0.3883455	0.029	0.157	5.94E-10	Granulocytes	Aco2
4.23E-14	-0.4235263	0.027	0.152	5.99E-10	Granulocytes	Ivns1abp
4.24E-14	-0.4104353	0.008	0.119	6.00E-10	Granulocytes	Zfp593
4.26E-14	-0.3862727	0.022	0.146	6.03E-10	Granulocytes	Zfp91
4.33E-14	0.70685171	0.72	0.747	6.12E-10	Granulocytes	Ubc
4.37E-14	-0.3620607	0.112	0.284	6.19E-10	Granulocytes	Tmem123
4.39E-14	-0.7525788	0.008	0.12	6.21E-10	Granulocytes	Ikzf2
4.39E-14	-0.4332863	0.029	0.156	6.21E-10	Granulocytes	Ubqln1
4.41E-14	-0.5774724	0.002	0.109	6.24E-10	Granulocytes	Sytl3
4.50E-14	-0.3904317	0.02	0.142	6.38E-10	Granulocytes	Pura
4.67E-14	-0.4545315	0.014	0.131	6.62E-10	Granulocytes	Zfp644
4.70E-14	-0.6586406	0.006	0.117	6.65E-10	Granulocytes	Fam43a
4.80E-14	-0.2610436	0.078	0.235	6.79E-10	Granulocytes	Trappc2l
4.89E-14	-0.3600246	0.01	0.124	6.93E-10	Granulocytes	Mrps28
4.91E-14	-0.550569	0.008	0.119	6.95E-10	Granulocytes	Cnn3
4.95E-14	-0.3485686	0.016	0.135	7.00E-10	Granulocytes	Gps1
4.95E-14	-0.416343	0.029	0.154	7.01E-10	Granulocytes	Arl4a
5.30E-14	-0.3595284	0.018	0.137	7.50E-10	Granulocytes	Dtymk
5.37E-14	-0.4631196	0.006	0.116	7.60E-10	Granulocytes	Ppp3cc
5.57E-14	-0.3734868	0.006	0.116	7.89E-10	Granulocytes	Aarsd1
5.67E-14	-0.4928645	0.01	0.123	8.03E-10	Granulocytes	Card11
5.72E-14	-0.4144216	0.002	0.108	8.10E-10	Granulocytes	Rangap1
5.78E-14	-0.2658016	0.078	0.237	8.18E-10	Granulocytes	Mapre2
5.78E-14	-0.3503279	0.027	0.152	8.19E-10	Granulocytes	Sike1
5.80E-14	-0.4202772	0.012	0.127	8.20E-10	Granulocytes	Slc38a10
5.81E-14	-0.2552531	0.1	0.269	8.22E-10	Granulocytes	Lypla2
6.01E-14	-0.4183168	0.006	0.116	8.51E-10	Granulocytes	Phgdh
6.07E-14	-0.3358144	0.027	0.151	8.59E-10	Granulocytes	Rpl7l1
6.20E-14	-0.3187713	0.076	0.23	8.78E-10	Granulocytes	Rsbn1l
6.23E-14	-0.3309894	0.035	0.166	8.82E-10	Granulocytes	Rnf4
6.29E-14	-0.3535207	0.037	0.167	8.90E-10	Granulocytes	Eif2b2
6.56E-14	-0.4828025	0.006	0.115	9.29E-10	Granulocytes	Kcnn4
6.60E-14	-0.4067609	0.043	0.179	9.34E-10	Granulocytes	Eif1a
6.74E-14	-0.4106265	0.002	0.108	9.54E-10	Granulocytes	Farsb
6.74E-14	-0.3971273	0.288	0.521	9.54E-10	Granulocytes	Fam107b
6.82E-14	-0.4215337	0.008	0.119	9.66E-10	Granulocytes	Dr1
6.99E-14	-0.3899143	0.012	0.125	9.89E-10	Granulocytes	Wdr74
7.09E-14	-0.508718	0.004	0.111	1.00E-09	Granulocytes	Bcl11b
7.25E-14	-0.3674416	0.027	0.153	1.03E-09	Granulocytes	Rnf6
7.37E-14	0.89412723	0.139	0.059	1.04E-09	Granulocytes	Apobr
7.99E-14	-0.4004238	0.008	0.118	1.13E-09	Granulocytes	Snrnp25

8.13E-14	-0.3783644	0.031	0.157	1.15E-09	Granulocytes	Adss
8.15E-14	-0.4076502	0.01	0.123	1.15E-09	Granulocytes	Atl3
8.16E-14	-0.429511	0.039	0.169	1.16E-09	Granulocytes	Clint1
8.23E-14	-0.363937	0.012	0.125	1.17E-09	Granulocytes	Mrpl27
8.34E-14	-0.4473198	0.01	0.122	1.18E-09	Granulocytes	Pdlim1
8.37E-14	-0.3586237	0.047	0.186	1.18E-09	Granulocytes	Rbm7
8.48E-14	-0.3978625	0.004	0.111	1.20E-09	Granulocytes	Elk3
8.54E-14	-0.2608249	0.053	0.196	1.21E-09	Granulocytes	Nabp2
9.12E-14	-0.3433338	0.018	0.136	1.29E-09	Granulocytes	Psmd3
9.22E-14	-0.402383	0.012	0.125	1.31E-09	Granulocytes	Fh1
9.24E-14	-0.4798152	0.006	0.115	1.31E-09	Granulocytes	Tgtp2
9.29E-14	-0.4889824	0.004	0.11	1.32E-09	Granulocytes	Prkcq
9.37E-14	-0.3875427	0.016	0.132	1.33E-09	Granulocytes	Tapbpl
9.40E-14	-0.4147743	0.004	0.111	1.33E-09	Granulocytes	Ankrd10
9.43E-14	-0.3653841	0.012	0.125	1.33E-09	Granulocytes	Mrpl16
9.61E-14	-0.4180221	0.002	0.106	1.36E-09	Granulocytes	Ppil1
9.66E-14	-0.3973556	0.006	0.114	1.37E-09	Granulocytes	Spr
9.71E-14	-0.3411543	0.039	0.172	1.37E-09	Granulocytes	Glg1
9.95E-14	-0.4358838	0.008	0.117	1.41E-09	Granulocytes	Ccdc107
1.01E-13	-0.3683905	0.014	0.129	1.43E-09	Granulocytes	Ddx27
1.06E-13	-0.4416407	0	0.103	1.50E-09	Granulocytes	Ahr
1.07E-13	-0.3256133	0.178	0.377	1.51E-09	Granulocytes	Ppp1cc
1.07E-13	-0.6291177	0.008	0.117	1.52E-09	Granulocytes	Txk
1.07E-13	-0.3136265	0.17	0.363	1.52E-09	Granulocytes	Stat1
1.08E-13	-0.5127861	0.012	0.124	1.53E-09	Granulocytes	Cd40
1.08E-13	-0.3645139	0.004	0.11	1.53E-09	Granulocytes	Apex1
1.09E-13	-0.3673137	0.063	0.208	1.54E-09	Granulocytes	Acot7
1.09E-13	-0.3182492	0.033	0.16	1.55E-09	Granulocytes	Smim20
1.10E-13	-0.4203324	0.043	0.174	1.55E-09	Granulocytes	Hadha
1.10E-13	-0.4686678	0.037	0.165	1.55E-09	Granulocytes	Igtp
1.13E-13	-0.3848598	0.012	0.126	1.60E-09	Granulocytes	Qrich1
1.14E-13	-0.3826143	0.008	0.117	1.61E-09	Granulocytes	Mrps25
1.17E-13	-0.4357284	0.006	0.113	1.65E-09	Granulocytes	Fcgrt
1.18E-13	1.10596423	0.329	0.23	1.67E-09	Granulocytes	Tgoln1
1.21E-13	-0.3933799	0.035	0.163	1.71E-09	Granulocytes	Baz1b
1.21E-13	-0.366772	0.006	0.113	1.71E-09	Granulocytes	Ppih
1.22E-13	-0.3632787	0.025	0.146	1.73E-09	Granulocytes	Cops4
1.25E-13	-0.3040509	0.014	0.13	1.77E-09	Granulocytes	Lrrc59
1.26E-13	-0.3799065	0.006	0.113	1.78E-09	Granulocytes	Timm22
1.28E-13	-0.4082115	0.006	0.114	1.81E-09	Granulocytes	Sdc3
1.34E-13	-0.3905426	0.012	0.124	1.90E-09	Granulocytes	Pgrmc1
1.39E-13	-0.4074774	0.008	0.117	1.96E-09	Granulocytes	Rabep1
1.43E-13	-0.4567627	0.006	0.113	2.03E-09	Granulocytes	Msi2
1.44E-13	1.04087524	0.282	0.181	2.04E-09	Granulocytes	Ptpre
1.45E-13	-0.5319626	0.025	0.143	2.05E-09	Granulocytes	Tecpr1
1.49E-13	-0.4514516	0.01	0.119	2.11E-09	Granulocytes	Padi2
1.52E-13	-0.4590025	0.01	0.12	2.15E-09	Granulocytes	Pml
1.53E-13	-0.3322849	0.098	0.262	2.17E-09	Granulocytes	Ccnl2

1.54E-13	-0.3799879	0.029	0.152	2.17E-09	Granulocytes	Nek7
1.56E-13	-0.5366942	0.014	0.125	2.21E-09	Granulocytes	Filip1l
1.56E-13	-0.354636	0.012	0.124	2.21E-09	Granulocytes	Fnta
1.62E-13	-0.4134654	0.008	0.116	2.29E-09	Granulocytes	Esf1
1.63E-13	-0.4978575	0.041	0.17	2.31E-09	Granulocytes	Sgk1
1.65E-13	-0.2902108	0.051	0.189	2.33E-09	Granulocytes	Tmed3
1.65E-13	-0.3444313	0.006	0.113	2.34E-09	Granulocytes	Ube2v2
1.68E-13	-0.3442743	0.016	0.131	2.38E-09	Granulocytes	Tgs1
1.71E-13	-0.3539512	0.025	0.145	2.42E-09	Granulocytes	Taf11
1.77E-13	-0.2623009	0.174	0.368	2.51E-09	Granulocytes	Sfpq
1.78E-13	-0.3821137	0	0.101	2.52E-09	Granulocytes	Hars
1.79E-13	-0.4359245	0.016	0.131	2.53E-09	Granulocytes	Mmd
1.84E-13	-0.2948178	0.084	0.241	2.61E-09	Granulocytes	Epc1
1.86E-13	-0.4605906	0.012	0.122	2.63E-09	Granulocytes	Kpna1
1.87E-13	-0.3442991	0.053	0.191	2.65E-09	Granulocytes	Yipf3
1.91E-13	-0.2998694	0.031	0.156	2.70E-09	Granulocytes	Btf3l4
1.91E-13	-0.3571275	0.027	0.148	2.70E-09	Granulocytes	Brms1
1.93E-13	-0.4379533	0.018	0.135	2.73E-09	Granulocytes	Cd6
1.96E-13	-0.2746634	0.231	0.461	2.77E-09	Granulocytes	Mdh2
2.06E-13	-0.3393098	0.035	0.162	2.91E-09	Granulocytes	Larp7
2.10E-13	-0.3384985	0.051	0.187	2.98E-09	Granulocytes	Smim7
2.11E-13	-0.3595108	0.025	0.145	2.98E-09	Granulocytes	Rhof
2.17E-13	-0.2980489	0.051	0.189	3.08E-09	Granulocytes	Chfr
2.19E-13	-0.4217978	0.002	0.104	3.10E-09	Granulocytes	Ezh2
2.24E-13	-0.3915937	0.004	0.107	3.18E-09	Granulocytes	Tusc3
2.26E-13	-0.3033135	0.031	0.155	3.20E-09	Granulocytes	Rbm22
2.26E-13	-0.3425277	0.022	0.141	3.20E-09	Granulocytes	Zcchc9
2.30E-13	-0.6615252	0.016	0.127	3.25E-09	Granulocytes	Ms4a4a
2.31E-13	-0.4397664	0	0.1	3.27E-09	Granulocytes	Gbp8
2.32E-13	-0.4413611	0.016	0.129	3.28E-09	Granulocytes	P2rx4
2.34E-13	-0.3009146	0.051	0.188	3.32E-09	Granulocytes	Ctcf
2.38E-13	-0.2755837	0.061	0.203	3.37E-09	Granulocytes	Nars
2.40E-13	0.86116986	0.176	0.086	3.40E-09	Granulocytes	Mirt1
2.46E-13	0.90067414	0.431	0.347	3.48E-09	Granulocytes	Sdcbp
2.47E-13	-0.5076308	0.012	0.121	3.50E-09	Granulocytes	Grap2
2.48E-13	-0.362198	0.006	0.112	3.51E-09	Granulocytes	Nsun2
2.48E-13	-0.4395072	0.041	0.17	3.52E-09	Granulocytes	Akap9
2.51E-13	-0.387379	0.035	0.161	3.56E-09	Granulocytes	Fam133b
2.52E-13	-0.3744962	0.016	0.129	3.57E-09	Granulocytes	Mphosph8
2.56E-13	-0.4664562	0.008	0.115	3.62E-09	Granulocytes	Slamf6
2.59E-13	-0.3515386	0.049	0.181	3.66E-09	Granulocytes	Kansl1
2.70E-13	-0.413705	0.016	0.127	3.82E-09	Granulocytes	Epb41l4aos
2.71E-13	-0.4561327	0.059	0.197	3.83E-09	Granulocytes	Cntrl
2.73E-13	-0.3273648	0.049	0.184	3.87E-09	Granulocytes	Copa
2.74E-13	-0.3898718	0.043	0.172	3.88E-09	Granulocytes	Chka
2.75E-13	-0.2991056	0.376	0.622	3.90E-09	Granulocytes	Arf5
2.77E-13	-0.3832441	0.006	0.111	3.91E-09	Granulocytes	Sfxn1
2.82E-13	-0.3074861	0.029	0.15	3.99E-09	Granulocytes	Isca2

2.84E-13	-0.3691355	0.022	0.139	4.02E-09	Granulocytes	Cyb5b
2.91E-13	-0.3338393	0.016	0.13	4.12E-09	Granulocytes	Eci1
2.92E-13	-0.4876381	0.006	0.111	4.14E-09	Granulocytes	Pim2
2.94E-13	-0.4470524	0.006	0.11	4.16E-09	Granulocytes	Dnm1l
3.00E-13	-0.3718296	0.035	0.161	4.24E-09	Granulocytes	Tomm34
3.07E-13	-0.3672326	0.035	0.16	4.35E-09	Granulocytes	Tlk1
3.09E-13	-0.4114894	0.002	0.103	4.37E-09	Granulocytes	Arl5a
3.10E-13	-0.4403066	0.014	0.126	4.39E-09	Granulocytes	Rbl2
3.14E-13	-0.500598	0.002	0.102	4.44E-09	Granulocytes	Tmem163
3.16E-13	-0.4851806	0.027	0.145	4.47E-09	Granulocytes	Clcn3
3.16E-13	-0.386756	0.018	0.131	4.48E-09	Granulocytes	Apopt1
3.20E-13	-0.622342	0.01	0.116	4.53E-09	Granulocytes	Irf4
3.22E-13	-0.3111129	0.08	0.231	4.56E-09	Granulocytes	Fnbp1
3.24E-13	-0.4488624	0.008	0.114	4.58E-09	Granulocytes	Ly9
3.30E-13	-0.6914191	0.031	0.152	4.67E-09	Granulocytes	Bank1
3.34E-13	-0.2788354	0.022	0.14	4.73E-09	Granulocytes	Bak1
3.34E-13	-0.3985132	0.035	0.158	4.73E-09	Granulocytes	Acly
3.37E-13	-0.2882203	0.047	0.179	4.77E-09	Granulocytes	Ten1
3.38E-13	-0.414514	0.014	0.124	4.79E-09	Granulocytes	Zfp800
3.42E-13	-0.3955612	0.018	0.132	4.83E-09	Granulocytes	Gpatch8
3.43E-13	-0.4311058	0.01	0.117	4.86E-09	Granulocytes	Gmnn
3.45E-13	-0.3344086	0.012	0.121	4.88E-09	Granulocytes	Psmf1
3.47E-13	-0.3947881	0.045	0.176	4.92E-09	Granulocytes	Lmbrd1
3.58E-13	-0.4721379	0.002	0.102	5.06E-09	Granulocytes	Lax1
3.67E-13	-0.3230214	0.025	0.142	5.20E-09	Granulocytes	Pnkd
3.68E-13	-0.8355939	0.082	0.224	5.21E-09	Granulocytes	Grn
3.70E-13	-0.5953269	0.076	0.218	5.24E-09	Granulocytes	Satb1
3.75E-13	-0.4537239	0.016	0.127	5.31E-09	Granulocytes	Hdac7
3.90E-13	-0.3128616	0.014	0.125	5.52E-09	Granulocytes	Ube2m
3.90E-13	-0.366137	0.014	0.125	5.52E-09	Granulocytes	Zfp326
3.92E-13	-0.3761357	0.01	0.116	5.55E-09	Granulocytes	Mrps35
3.93E-13	-0.4043883	0.008	0.113	5.57E-09	Granulocytes	Trim27
4.00E-13	-0.723259	0.01	0.116	5.67E-09	Granulocytes	Scd1
4.16E-13	-0.3643779	0.047	0.179	5.90E-09	Granulocytes	Map4
4.24E-13	-0.394349	0.031	0.152	6.00E-09	Granulocytes	Pum1
4.29E-13	-0.3734058	0.01	0.117	6.07E-09	Granulocytes	Pafah1b3
4.36E-13	-0.3926788	0.094	0.252	6.17E-09	Granulocytes	Atrx
4.39E-13	-0.6895471	0.004	0.105	6.21E-09	Granulocytes	Klrc1
4.47E-13	-0.347058	0.018	0.13	6.32E-09	Granulocytes	Ppa1
4.48E-13	-0.3587815	0.022	0.136	6.35E-09	Granulocytes	Sumo3
4.64E-13	-0.3221399	0.072	0.219	6.57E-09	Granulocytes	Smg1
4.72E-13	-0.4002038	0.027	0.143	6.67E-09	Granulocytes	Vezf1
4.84E-13	-0.3784677	0.016	0.126	6.85E-09	Granulocytes	Nr2c2ap
4.88E-13	-0.4031085	0.002	0.101	6.91E-09	Granulocytes	Uri1
5.01E-13	-0.3883898	0.016	0.127	7.10E-09	Granulocytes	Med15
5.06E-13	-0.2877262	0.027	0.146	7.16E-09	Granulocytes	Faf2
5.06E-13	-0.4376222	0.02	0.133	7.17E-09	Granulocytes	Mgst2
5.10E-13	-0.3602916	0.029	0.147	7.22E-09	Granulocytes	Nxt1

5.14E-13	-0.3422214	0.006	0.109	7.28E-09	Granulocytes	Srm
5.21E-13	-0.4044681	0.016	0.127	7.37E-09	Granulocytes	Magt1
5.22E-13	-0.2606261	0.067	0.212	7.38E-09	Granulocytes	Sdha
5.30E-13	-0.3175539	0.014	0.123	7.50E-09	Granulocytes	A430005L14R
5.43E-13	-0.3682332	0.008	0.112	7.69E-09	Granulocytes	Mphosph6
5.50E-13	-0.2599946	0.067	0.211	7.79E-09	Granulocytes	Eif4g3
5.86E-13	-0.3690195	0.008	0.112	8.29E-09	Granulocytes	Ddi2
5.86E-13	-0.3379653	0.012	0.119	8.30E-09	Granulocytes	Mbd3
5.87E-13	-0.3372006	0.031	0.15	8.31E-09	Granulocytes	Psme3
5.90E-13	-0.4476486	0.041	0.166	8.36E-09	Granulocytes	Etnk1
5.91E-13	-0.3105139	0.033	0.155	8.36E-09	Granulocytes	Ubxn6
5.99E-13	-0.3676768	0.043	0.172	8.48E-09	Granulocytes	Fryl
5.99E-13	-0.3256119	0.018	0.129	8.49E-09	Granulocytes	Mrpl55
6.04E-13	-0.486755	0.008	0.112	8.55E-09	Granulocytes	Cxcr3
6.14E-13	-0.4947354	0.008	0.112	8.68E-09	Granulocytes	Myo1e
6.21E-13	-0.4512846	0.002	0.1	8.79E-09	Granulocytes	Tcf12
6.31E-13	1.05536312	0.384	0.305	8.93E-09	Granulocytes	Coq10b
6.38E-13	-0.3991994	0.012	0.118	9.03E-09	Granulocytes	Gnl3
6.50E-13	-0.3670136	0.004	0.104	9.21E-09	Granulocytes	Hspbp1
6.54E-13	-0.3962847	0.029	0.147	9.26E-09	Granulocytes	Cldnd1
6.58E-13	-0.3329062	0.012	0.119	9.32E-09	Granulocytes	Blmh
6.61E-13	-0.4298714	0.072	0.213	9.36E-09	Granulocytes	Gns
6.68E-13	-0.2806039	0.059	0.197	9.46E-09	Granulocytes	Snrnp27
6.69E-13	-0.3821723	0.01	0.115	9.47E-09	Granulocytes	Brcc3
6.77E-13	-0.3491674	0.02	0.134	9.58E-09	Granulocytes	Sec31a
6.92E-13	-0.3644414	0.016	0.126	9.80E-09	Granulocytes	Sae1
7.04E-13	-0.3321737	0.041	0.168	9.96E-09	Granulocytes	Golgb1
7.29E-13	-0.2614718	0.033	0.155	1.03E-08	Granulocytes	Mpv17l2
7.31E-13	-0.3387942	0.018	0.13	1.03E-08	Granulocytes	Jak3
7.50E-13	-0.2714541	0.08	0.228	1.06E-08	Granulocytes	Os9
7.51E-13	-0.3955382	0.002	0.1	1.06E-08	Granulocytes	Bzw2
7.83E-13	1.03030867	0.256	0.159	1.11E-08	Granulocytes	Cmip
7.84E-13	-0.7264584	0.006	0.107	1.11E-08	Granulocytes	Klre1
7.85E-13	-0.3939964	0.008	0.111	1.11E-08	Granulocytes	R3hdm1
7.87E-13	-0.3448744	0.045	0.174	1.11E-08	Granulocytes	Ccar1
8.16E-13	-0.3664134	0.012	0.118	1.15E-08	Granulocytes	Cfap20
8.29E-13	1.03986167	0.466	0.403	1.17E-08	Granulocytes	Txnip
8.37E-13	-0.3061126	0.004	0.104	1.18E-08	Granulocytes	Ift27
8.39E-13	-0.3925566	0.047	0.174	1.19E-08	Granulocytes	Mta2
8.65E-13	-0.4127735	0.02	0.133	1.22E-08	Granulocytes	Gt(ROSA)26S
8.71E-13	-0.4077345	0.041	0.165	1.23E-08	Granulocytes	Cnot4
8.80E-13	-0.4724624	0.01	0.114	1.25E-08	Granulocytes	Cdt1
8.84E-13	-0.3937276	0.008	0.11	1.25E-08	Granulocytes	Htatsf1
9.07E-13	-0.2633427	0.018	0.131	1.28E-08	Granulocytes	Manbal
9.10E-13	-0.4909712	0.018	0.127	1.29E-08	Granulocytes	Gm4070
9.11E-13	-0.4512589	0.008	0.111	1.29E-08	Granulocytes	Asxl2
9.26E-13	-0.2720986	0.045	0.175	1.31E-08	Granulocytes	Rab10os
9.44E-13	-0.3946595	0.016	0.124	1.34E-08	Granulocytes	Hsd17b12

9.61E-13	-0.274041	0.08	0.225	1.36E-08	Granulocytes	Ppp2r1a
9.63E-13	-0.3651004	0.029	0.146	1.36E-08	Granulocytes	Rad23a
9.86E-13	-0.3728191	0.014	0.121	1.40E-08	Granulocytes	Ube2d1
9.90E-13	-0.3084216	0.014	0.121	1.40E-08	Granulocytes	Ccnh
9.90E-13	-0.3179042	0.037	0.161	1.40E-08	Granulocytes	Zfr
1.01E-12	-0.430314	0.014	0.121	1.43E-08	Granulocytes	Riok1
1.04E-12	1.11830226	0.299	0.203	1.47E-08	Granulocytes	Creg1
1.09E-12	-0.3250067	0.006	0.106	1.54E-08	Granulocytes	Mrps18b
1.10E-12	-0.3938295	0.01	0.113	1.56E-08	Granulocytes	Gbp3
1.12E-12	-0.3964533	0.027	0.142	1.59E-08	Granulocytes	2410004B18I
1.20E-12	-0.4074347	0.006	0.106	1.70E-08	Granulocytes	Rab19
1.20E-12	-0.3584781	0.008	0.11	1.70E-08	Granulocytes	Mad2l2
1.21E-12	-0.3958804	0.051	0.181	1.71E-08	Granulocytes	Cnot6l
1.21E-12	-0.323272	0.049	0.18	1.71E-08	Granulocytes	Parp14
1.21E-12	-0.3467295	0.008	0.109	1.71E-08	Granulocytes	3830406C13F
1.22E-12	-0.325162	0.018	0.128	1.73E-08	Granulocytes	Cebpzos
1.24E-12	-0.3914632	0.006	0.105	1.76E-08	Granulocytes	Bmyc
1.25E-12	-0.3785196	0.006	0.105	1.77E-08	Granulocytes	Mrps5
1.26E-12	-0.2966624	0.039	0.161	1.78E-08	Granulocytes	Smu1
1.27E-12	-0.2932761	0.031	0.147	1.80E-08	Granulocytes	Igbp1
1.29E-12	-0.3836743	0.065	0.202	1.82E-08	Granulocytes	Arhgap17
1.31E-12	-0.2819212	0.153	0.333	1.86E-08	Granulocytes	Rapgef6
1.33E-12	-0.3063604	0.047	0.175	1.88E-08	Granulocytes	Cbfb
1.33E-12	-0.3415079	0.018	0.128	1.89E-08	Granulocytes	BC003965
1.34E-12	-0.3620585	0.008	0.109	1.90E-08	Granulocytes	Polb
1.34E-12	-0.3017307	0.027	0.142	1.90E-08	Granulocytes	Ptpra
1.36E-12	-0.4091319	0.014	0.119	1.93E-08	Granulocytes	Rcc2
1.40E-12	-0.3968159	0.008	0.109	1.98E-08	Granulocytes	Mink1
1.43E-12	-0.3441135	0.006	0.105	2.02E-08	Granulocytes	Dera
1.43E-12	-0.3935417	0.014	0.119	2.03E-08	Granulocytes	Fdps
1.46E-12	0.63093966	0.112	0.044	2.06E-08	Granulocytes	Cd80
1.55E-12	1.02691174	0.247	0.152	2.19E-08	Granulocytes	Ddit3
1.56E-12	-0.3729319	0.029	0.144	2.21E-08	Granulocytes	Creld2
1.58E-12	-0.2771445	0.029	0.144	2.23E-08	Granulocytes	Fbxo6
1.67E-12	-0.3699743	0.029	0.143	2.36E-08	Granulocytes	Cd2bp2
1.70E-12	-0.3213719	0.057	0.191	2.41E-08	Granulocytes	Zfp292
1.72E-12	-0.4191677	0.027	0.14	2.43E-08	Granulocytes	Traf6
1.73E-12	-0.4091199	0.025	0.137	2.45E-08	Granulocytes	Cabin1
1.73E-12	-0.480626	0.039	0.16	2.45E-08	Granulocytes	Lamb3
1.74E-12	-0.3540134	0.004	0.101	2.46E-08	Granulocytes	Noc2l
1.79E-12	-0.2729138	0.057	0.191	2.54E-08	Granulocytes	2310011J03R
1.85E-12	-0.3008112	0.102	0.257	2.62E-08	Granulocytes	Ikzf1
1.86E-12	-0.2749573	0.041	0.165	2.63E-08	Granulocytes	Surf1
1.86E-12	-0.3065042	0.037	0.158	2.64E-08	Granulocytes	Cpsf6
1.86E-12	-0.3228293	0.004	0.101	2.64E-08	Granulocytes	Rnaseh2a
1.92E-12	-0.4428364	0.01	0.112	2.72E-08	Granulocytes	Plcxd2
1.93E-12	1.45681141	0.442	0.373	2.73E-08	Granulocytes	Gadd45b
2.03E-12	1.137896	0.266	0.171	2.87E-08	Granulocytes	Cdkn2d

2.04E-12	-0.3222312	0.012	0.116	2.89E-08	Granulocytes	Rrp7a
2.05E-12	-0.299265	0.029	0.143	2.90E-08	Granulocytes	Pdha1
2.13E-12	-0.3366715	0.012	0.115	3.01E-08	Granulocytes	Vkorc1
2.17E-12	-0.342196	0.004	0.1	3.08E-08	Granulocytes	Alad
2.18E-12	1.22823156	0.425	0.347	3.09E-08	Granulocytes	Fosb
2.22E-12	-0.2896643	0.006	0.104	3.15E-08	Granulocytes	Hat1
2.26E-12	-0.2859073	0.051	0.18	3.20E-08	Granulocytes	Golga7
2.27E-12	-0.2795115	0.027	0.14	3.21E-08	Granulocytes	Nrbf2
2.32E-12	-0.4163444	0.02	0.129	3.29E-08	Granulocytes	Nsf
2.34E-12	0.88772382	0.247	0.152	3.31E-08	Granulocytes	Mbp
2.34E-12	-0.3650825	0.004	0.1	3.32E-08	Granulocytes	Rsrc1
2.37E-12	-0.2595502	0.074	0.213	3.36E-08	Granulocytes	Vps36
2.48E-12	-0.3204162	0.029	0.143	3.51E-08	Granulocytes	Paf1
2.48E-12	0.90561592	0.174	0.089	3.52E-08	Granulocytes	Por
2.51E-12	-0.2584991	0.137	0.311	3.55E-08	Granulocytes	Ubxn4
2.54E-12	-0.364746	0.01	0.111	3.60E-08	Granulocytes	Nop14
2.56E-12	1.18884082	0.317	0.23	3.63E-08	Granulocytes	Etf1
2.58E-12	-0.3239802	0.016	0.122	3.65E-08	Granulocytes	Pes1
2.59E-12	-0.3225644	0.031	0.146	3.66E-08	Granulocytes	Ccz1
2.59E-12	-0.3702838	0.016	0.122	3.66E-08	Granulocytes	Dram2
2.64E-12	-0.3167433	0.027	0.139	3.73E-08	Granulocytes	Cyb5r3
2.68E-12	-0.3230594	0.02	0.129	3.80E-08	Granulocytes	Lactb
2.69E-12	-0.3382019	0.012	0.114	3.81E-08	Granulocytes	Snx12
2.69E-12	-0.3151815	0.01	0.11	3.81E-08	Granulocytes	Naa20
2.71E-12	1.01871071	0.117	0.048	3.84E-08	Granulocytes	Clec4n
2.74E-12	-0.2907139	0.047	0.173	3.89E-08	Granulocytes	Cdc40
2.76E-12	-0.293668	0.033	0.148	3.90E-08	Granulocytes	Zfp207
2.78E-12	-0.387266	0.012	0.114	3.94E-08	Granulocytes	Fgd2
2.80E-12	0.81289894	0.137	0.061	3.96E-08	Granulocytes	Hdac4
2.82E-12	-0.5409566	0.008	0.106	3.99E-08	Granulocytes	Slamf9
2.83E-12	-0.3929268	0.006	0.102	4.00E-08	Granulocytes	Nob1
2.90E-12	-0.3414349	0.029	0.143	4.10E-08	Granulocytes	Arhgef6
2.91E-12	-0.3761058	0.018	0.125	4.13E-08	Granulocytes	Naaa
2.97E-12	-0.3698976	0.008	0.107	4.20E-08	Granulocytes	Aldh9a1
2.98E-12	-0.2993701	0.018	0.125	4.21E-08	Granulocytes	Larp1
3.01E-12	-0.2598714	0.041	0.164	4.26E-08	Granulocytes	Ubtf
3.08E-12	-0.2714264	0.143	0.317	4.36E-08	Granulocytes	Ifi27
3.26E-12	-0.4656187	0.012	0.113	4.61E-08	Granulocytes	Arsb
3.29E-12	0.99835523	0.217	0.125	4.66E-08	Granulocytes	Atg2a
3.32E-12	-0.3696056	0.006	0.102	4.69E-08	Granulocytes	Galnt2
3.34E-12	0.85632159	0.143	0.066	4.74E-08	Granulocytes	Hgsnat
3.45E-12	-0.3553942	0.014	0.117	4.88E-08	Granulocytes	Ehmt2
3.45E-12	-0.7779446	0.012	0.113	4.88E-08	Granulocytes	Tsc22d1
3.45E-12	-0.2720239	0.018	0.125	4.88E-08	Granulocytes	Cmas
3.61E-12	-0.286601	0.055	0.184	5.10E-08	Granulocytes	Dgkz
3.65E-12	-0.2699867	0.053	0.182	5.17E-08	Granulocytes	Elf2
3.69E-12	-0.2880499	0.057	0.187	5.22E-08	Granulocytes	Ddx46
3.76E-12	-0.3176027	0.008	0.106	5.32E-08	Granulocytes	Tfg

3.76E-12	-0.3413024	0.039	0.156	5.33E-08	Granulocytes	Rfc1
3.77E-12	-0.3238457	0.016	0.121	5.34E-08	Granulocytes	Dars
3.81E-12	-0.3023181	0.006	0.102	5.39E-08	Granulocytes	Prdx4
3.83E-12	-0.3692856	0.008	0.105	5.41E-08	Granulocytes	Panx1
3.83E-12	-0.26635	0.008	0.107	5.43E-08	Granulocytes	Hes6
3.86E-12	-0.2852534	0.106	0.262	5.46E-08	Granulocytes	Tpst2
3.90E-12	-0.41112	0.01	0.109	5.53E-08	Granulocytes	Tfam
3.91E-12	-0.2750614	0.063	0.198	5.53E-08	Granulocytes	Pcnp
3.96E-12	-0.2968078	0.016	0.121	5.61E-08	Granulocytes	Ppil3
4.00E-12	-0.3332835	0.008	0.106	5.66E-08	Granulocytes	Phpt1
4.00E-12	-0.29244	0.006	0.102	5.66E-08	Granulocytes	Galk1
4.08E-12	-0.2734031	0.043	0.163	5.77E-08	Granulocytes	Derl2
4.08E-12	-0.3627428	0.018	0.123	5.78E-08	Granulocytes	Eif4e3
4.14E-12	-0.3161278	0.006	0.102	5.86E-08	Granulocytes	Tsr3
4.36E-12	-0.3816691	0.02	0.127	6.17E-08	Granulocytes	Ccnt2
4.45E-12	-0.6229194	0.012	0.112	6.30E-08	Granulocytes	Pltp
4.47E-12	-0.3338181	0.012	0.113	6.33E-08	Granulocytes	Ppp5c
4.48E-12	-0.2899222	0.047	0.17	6.35E-08	Granulocytes	Nras
4.49E-12	-0.428293	0.008	0.105	6.36E-08	Granulocytes	Srek1
4.52E-12	-0.2785932	0.022	0.131	6.40E-08	Granulocytes	4930453N24I
4.61E-12	-0.3304524	0.008	0.105	6.53E-08	Granulocytes	Hmgn5
4.62E-12	0.82083542	0.16	0.078	6.54E-08	Granulocytes	Dennd5a
4.69E-12	-0.3097329	0.008	0.106	6.64E-08	Granulocytes	Tmem60
4.70E-12	-0.358406	0.02	0.126	6.66E-08	Granulocytes	U2af1l4
4.72E-12	-0.2990479	0.039	0.157	6.68E-08	Granulocytes	Tmem222
4.77E-12	-0.2522217	0.018	0.125	6.75E-08	Granulocytes	Ccny
4.87E-12	-0.3086277	0.037	0.154	6.89E-08	Granulocytes	Kin
4.88E-12	-0.3469626	0.018	0.122	6.90E-08	Granulocytes	Echs1
4.98E-12	-0.3537391	0.025	0.132	7.05E-08	Granulocytes	Prpsap1
5.08E-12	-0.308138	0.02	0.127	7.20E-08	Granulocytes	4921524J17R
5.11E-12	-0.5979746	0.008	0.103	7.24E-08	Granulocytes	Snn
5.12E-12	-0.2841178	0.037	0.154	7.25E-08	Granulocytes	Iah1
5.16E-12	-0.2549795	0.045	0.167	7.31E-08	Granulocytes	Tmem248
5.38E-12	-0.2907874	0.027	0.137	7.61E-08	Granulocytes	Med11
5.41E-12	-0.3488032	0.01	0.108	7.65E-08	Granulocytes	Rpa3
5.41E-12	-0.3561351	0.02	0.127	7.66E-08	Granulocytes	Rasa1
5.42E-12	-0.3798079	0.008	0.104	7.67E-08	Granulocytes	Zpr1
5.45E-12	-0.3195119	0.01	0.109	7.71E-08	Granulocytes	Slc25a17
5.66E-12	-0.2996603	0.049	0.175	8.01E-08	Granulocytes	Mia3
5.67E-12	-0.3978758	0.006	0.101	8.02E-08	Granulocytes	A630001G21
5.67E-12	-0.2834936	0.029	0.141	8.03E-08	Granulocytes	Ap2b1
5.75E-12	-0.4061366	0.006	0.101	8.14E-08	Granulocytes	Syt11
5.75E-12	-0.3680384	0.008	0.105	8.15E-08	Granulocytes	Atp2a2
5.88E-12	-0.4258646	0.01	0.107	8.32E-08	Granulocytes	Cish
5.98E-12	-0.2887176	0.016	0.119	8.46E-08	Granulocytes	Pdzd11
6.03E-12	-0.3905277	0.01	0.107	8.54E-08	Granulocytes	Cul5
6.04E-12	-0.2843987	0.029	0.141	8.55E-08	Granulocytes	Bloc1s4
6.08E-12	-0.3389469	0.025	0.133	8.61E-08	Granulocytes	Atp6v0a2

6.11E-12	-0.3028356	0.018	0.123	8.66E-08	Granulocytes	Nup50
6.12E-12	-0.2868264	0.027	0.136	8.66E-08	Granulocytes	Mapkapk3
6.16E-12	-0.3152233	0.01	0.107	8.71E-08	Granulocytes	Mrps11
6.18E-12	-0.2581059	0.037	0.153	8.74E-08	Granulocytes	Txnl4a
6.18E-12	-0.2636838	0.041	0.161	8.75E-08	Granulocytes	Ssr1
6.24E-12	-0.3968617	0.022	0.128	8.83E-08	Granulocytes	Ahsa2
6.38E-12	-0.2700752	0.364	0.6	9.03E-08	Granulocytes	Pcbp2
7.02E-12	-0.367579	0.012	0.111	9.94E-08	Granulocytes	Nol12
7.05E-12	-0.3451444	0.012	0.111	9.98E-08	Granulocytes	Eif2a
7.07E-12	-0.3545555	0.033	0.143	1.00E-07	Granulocytes	Tomm70a
7.11E-12	-0.2989266	0.043	0.163	1.01E-07	Granulocytes	Ddx42
7.22E-12	-0.3413882	0.01	0.107	1.02E-07	Granulocytes	Mthfd2
7.32E-12	-0.3438207	0.008	0.103	1.04E-07	Granulocytes	Nt5c3b
7.43E-12	1.03305454	0.207	0.118	1.05E-07	Granulocytes	Carhsp1
7.50E-12	-0.3666638	0.043	0.161	1.06E-07	Granulocytes	Ubr2
7.57E-12	-0.2963234	0.022	0.13	1.07E-07	Granulocytes	Cdkn2aipnl
7.58E-12	-0.4109906	0.045	0.164	1.07E-07	Granulocytes	Atp2a3
7.63E-12	-0.3425405	0.01	0.106	1.08E-07	Granulocytes	Sf3a1
7.77E-12	-0.281951	0.035	0.15	1.10E-07	Granulocytes	Egln2
7.88E-12	-0.3242364	0.029	0.138	1.12E-07	Granulocytes	Rbmxl1
7.94E-12	-0.305827	0.012	0.111	1.12E-07	Granulocytes	Eva1b
8.02E-12	-0.285843	0.006	0.1	1.14E-07	Granulocytes	Bcat2
8.05E-12	-0.3309632	0.027	0.135	1.14E-07	Granulocytes	Hmg20b
8.22E-12	-0.3239919	0.025	0.131	1.16E-07	Granulocytes	Anxa7
8.28E-12	-0.5591881	0.018	0.121	1.17E-07	Granulocytes	Fchsd2
8.84E-12	-0.3202763	0.041	0.156	1.25E-07	Granulocytes	Azi2
8.84E-12	-0.3689862	0.008	0.103	1.25E-07	Granulocytes	Mcm4
8.85E-12	-0.3071872	0.016	0.118	1.25E-07	Granulocytes	Tubb6
8.91E-12	-0.2552894	0.02	0.125	1.26E-07	Granulocytes	Mtfr1l
8.93E-12	1.22657486	0.327	0.238	1.26E-07	Granulocytes	Tiparp
8.97E-12	-0.2783292	0.053	0.177	1.27E-07	Granulocytes	Rps6ka1
9.10E-12	-0.3324228	0.008	0.102	1.29E-07	Granulocytes	Lxn
9.30E-12	-0.2987395	0.055	0.18	1.32E-07	Granulocytes	Pdhb
9.52E-12	-0.3218514	0.031	0.142	1.35E-07	Granulocytes	Traf3ip3
9.59E-12	0.59069003	0.681	0.717	1.36E-07	Granulocytes	Cdc42
9.66E-12	-0.2813566	0.055	0.181	1.37E-07	Granulocytes	Pkn2
9.67E-12	-0.2822012	0.039	0.156	1.37E-07	Granulocytes	Hspa14
9.83E-12	-0.3113733	0.008	0.103	1.39E-07	Granulocytes	Erap1
1.03E-11	-0.3582088	0.016	0.117	1.45E-07	Granulocytes	Snd1
1.04E-11	-0.2772814	0.02	0.124	1.47E-07	Granulocytes	Zfp330
1.04E-11	-0.3370245	0.01	0.106	1.47E-07	Granulocytes	Exosc7
1.08E-11	-0.3260351	0.01	0.106	1.53E-07	Granulocytes	Pycr2
1.10E-11	-0.2901826	0.049	0.173	1.56E-07	Granulocytes	Aplp2
1.11E-11	-0.4235536	0.012	0.109	1.57E-07	Granulocytes	Slc4a7
1.12E-11	-0.305198	0.031	0.141	1.59E-07	Granulocytes	Mrpl48
1.13E-11	-0.2963226	0.022	0.126	1.60E-07	Granulocytes	Copg1
1.13E-11	-0.3564995	0.008	0.102	1.61E-07	Granulocytes	Ncoa7
1.14E-11	-0.4503809	0.125	0.273	1.61E-07	Granulocytes	Rgcc

1.14E-11	-0.3351109	0.356	0.584	1.62E-07	Granulocytes	Zfp36l2
1.15E-11	-0.2863069	0.037	0.151	1.63E-07	Granulocytes	Vps72
1.18E-11	-0.2965553	0.031	0.14	1.67E-07	Granulocytes	Mapk1ip1l
1.23E-11	-0.3537261	0.029	0.136	1.74E-07	Granulocytes	Zranb2
1.24E-11	-0.4306563	0.008	0.102	1.76E-07	Granulocytes	Itm2a
1.28E-11	-0.3188446	0.098	0.244	1.82E-07	Granulocytes	9-Sep
1.31E-11	-0.2789557	0.027	0.135	1.85E-07	Granulocytes	Stk19
1.31E-11	1.04100676	0.417	0.344	1.86E-07	Granulocytes	Efhd2
1.34E-11	-0.377976	0.031	0.14	1.90E-07	Granulocytes	Map3k1
1.36E-11	-0.3771698	0.008	0.102	1.92E-07	Granulocytes	Gm6377
1.39E-11	-0.344371	0.018	0.119	1.96E-07	Granulocytes	Zfp511
1.43E-11	-0.3167371	0.012	0.109	2.02E-07	Granulocytes	Ap1b1
1.43E-11	-0.2725639	0.037	0.151	2.03E-07	Granulocytes	Ubn2
1.48E-11	-0.3893915	0.014	0.112	2.10E-07	Granulocytes	Tia1
1.50E-11	-0.2689167	0.02	0.123	2.13E-07	Granulocytes	9130401M01
1.51E-11	-0.251593	0.053	0.176	2.14E-07	Granulocytes	Ppp6c
1.51E-11	-0.34826	0.025	0.129	2.14E-07	Granulocytes	1700017B05I
1.57E-11	-0.3176351	0.012	0.108	2.22E-07	Granulocytes	Eci2
1.57E-11	0.87699174	0.149	0.073	2.22E-07	Granulocytes	Fas
1.57E-11	-0.2774196	0.025	0.131	2.23E-07	Granulocytes	Man1a2
1.62E-11	-0.3431295	0.012	0.108	2.29E-07	Granulocytes	Exosc10
1.68E-11	-0.3272471	0.01	0.104	2.38E-07	Granulocytes	Tpm1
1.75E-11	-0.3121061	0.014	0.111	2.48E-07	Granulocytes	Polr3k
1.76E-11	0.80482577	0.104	0.042	2.49E-07	Granulocytes	Sesn2
1.78E-11	-0.3328137	0.025	0.129	2.52E-07	Granulocytes	Jmjd6
1.80E-11	-0.3446367	0.027	0.132	2.55E-07	Granulocytes	Lrrc58
1.81E-11	-0.3263696	0.033	0.143	2.56E-07	Granulocytes	Avl9
1.81E-11	-0.2793212	0.569	0.772	2.56E-07	Granulocytes	Atp5e
1.82E-11	-0.2835945	0.051	0.173	2.57E-07	Granulocytes	Ndfip2
1.85E-11	-0.3768171	0.55	0.733	2.61E-07	Granulocytes	Clic1
1.90E-11	-0.3336062	0.022	0.125	2.70E-07	Granulocytes	Tmub1
1.94E-11	-0.3580128	0.014	0.111	2.75E-07	Granulocytes	Topbp1
1.95E-11	-0.3560963	0.031	0.14	2.77E-07	Granulocytes	Zcchc7
1.96E-11	-0.4059514	0.01	0.103	2.77E-07	Granulocytes	Ctso
1.96E-11	-0.300296	0.074	0.206	2.77E-07	Granulocytes	Grk6
1.97E-11	-0.3490369	0.008	0.1	2.78E-07	Granulocytes	Mfng
2.01E-11	-0.4281059	0.112	0.257	2.84E-07	Granulocytes	Tcp11l2
2.10E-11	-0.3496179	0.012	0.107	2.97E-07	Granulocytes	Ncbp1
2.13E-11	-0.3224524	0.012	0.107	3.02E-07	Granulocytes	Pfdn4
2.15E-11	-0.34141	0.008	0.1	3.04E-07	Granulocytes	Dpysl2
2.16E-11	-0.3230053	0.049	0.167	3.06E-07	Granulocytes	Snx1
2.16E-11	-0.3198163	0.029	0.134	3.06E-07	Granulocytes	Ggh
2.19E-11	-0.4477414	0.098	0.237	3.09E-07	Granulocytes	Stap1
2.21E-11	-0.3375201	0.027	0.131	3.13E-07	Granulocytes	Ube2l6
2.23E-11	-0.2800717	0.039	0.151	3.16E-07	Granulocytes	Gtpbp2
2.26E-11	-0.390633	0.016	0.115	3.20E-07	Granulocytes	Casp8ap2
2.27E-11	-0.3404317	0.018	0.118	3.21E-07	Granulocytes	Ube2g2
2.35E-11	-0.2946064	0.008	0.1	3.32E-07	Granulocytes	Mocs2

2.38E-11	-0.6242297	0.01	0.103	3.37E-07	Granulocytes	Lef1
2.38E-11	0.80213679	0.135	0.063	3.37E-07	Granulocytes	Usp32
2.42E-11	-0.3343389	0.01	0.103	3.43E-07	Granulocytes	Parp2
2.44E-11	-0.3255547	0.037	0.149	3.45E-07	Granulocytes	Zc3h13
2.45E-11	-0.2816054	0.029	0.136	3.47E-07	Granulocytes	Rexo1
2.45E-11	-0.3606131	0.014	0.11	3.47E-07	Granulocytes	Hdac2
2.46E-11	-0.4338806	0.016	0.113	3.48E-07	Granulocytes	Gfpt1
2.46E-11	-0.3317313	0.022	0.125	3.49E-07	Granulocytes	Cpsf2
2.51E-11	-0.3238643	0.012	0.106	3.55E-07	Granulocytes	Urm1
2.52E-11	-0.3801383	0.043	0.156	3.57E-07	Granulocytes	Arcn1
2.57E-11	-0.3260157	0.022	0.124	3.64E-07	Granulocytes	Trmt6
2.65E-11	-0.8595826	0.016	0.113	3.74E-07	Granulocytes	Ccl2
2.65E-11	-0.341962	0.014	0.11	3.75E-07	Granulocytes	Dnase2a
2.77E-11	-0.4038972	0.027	0.13	3.92E-07	Granulocytes	Pcm1
2.78E-11	-0.3468972	0.014	0.108	3.94E-07	Granulocytes	Polr1c
2.80E-11	-0.2998896	0.016	0.113	3.97E-07	Granulocytes	Gfer
2.89E-11	-0.3338532	0.022	0.124	4.09E-07	Granulocytes	Vps13a
2.93E-11	-0.2581032	0.014	0.111	4.15E-07	Granulocytes	Dnpep
3.00E-11	-0.3203628	0.02	0.121	4.24E-07	Granulocytes	2610507B11I
3.01E-11	-0.3272927	0.025	0.127	4.26E-07	Granulocytes	Wdr82
3.05E-11	-0.350539	0.059	0.182	4.32E-07	Granulocytes	Ranbp2
3.13E-11	-0.3239412	0.01	0.102	4.44E-07	Granulocytes	Sgf29
3.15E-11	-0.3663188	0.047	0.162	4.45E-07	Granulocytes	Tcirg1
3.17E-11	-0.2602394	0.012	0.106	4.48E-07	Granulocytes	Tmem109
3.33E-11	-0.2581407	0.106	0.252	4.72E-07	Granulocytes	Batf
3.41E-11	-0.347944	0.045	0.16	4.83E-07	Granulocytes	Casp3
3.47E-11	-0.251957	0.055	0.176	4.91E-07	Granulocytes	Aph1a
3.56E-11	0.93069146	0.436	0.379	5.03E-07	Granulocytes	Cdc42se1
3.67E-11	-0.3564935	0.01	0.102	5.19E-07	Granulocytes	Dbf4
3.70E-11	-0.3458752	0.045	0.16	5.23E-07	Granulocytes	Tmem243
3.79E-11	-0.2895102	0.029	0.134	5.36E-07	Granulocytes	Dap3
3.79E-11	-0.3142316	0.012	0.105	5.36E-07	Granulocytes	Exosc1
3.81E-11	-0.3283928	0.014	0.109	5.40E-07	Granulocytes	Smarcc1
3.83E-11	-0.2902399	0.014	0.109	5.43E-07	Granulocytes	2610001J05R
3.87E-11	-0.2624283	0.022	0.123	5.49E-07	Granulocytes	Tpp1
3.92E-11	-0.4533328	0.037	0.144	5.56E-07	Granulocytes	Rnase6
4.01E-11	-0.2982081	0.016	0.112	5.68E-07	Granulocytes	Tbl1x
4.08E-11	1.04160997	0.233	0.148	5.77E-07	Granulocytes	Preb
4.11E-11	0.8752185	0.112	0.048	5.81E-07	Granulocytes	Jdp2
4.12E-11	-0.2926645	0.018	0.116	5.83E-07	Granulocytes	Crbn
4.14E-11	-0.3671131	0.016	0.111	5.86E-07	Granulocytes	Scimp
4.24E-11	-0.2804501	0.016	0.113	6.00E-07	Granulocytes	2300009A05I
4.25E-11	-0.4850542	0.027	0.127	6.02E-07	Granulocytes	Clec4a1
4.32E-11	-0.2952513	0.035	0.143	6.11E-07	Granulocytes	Lypla1
4.32E-11	-0.3560659	0.02	0.119	6.11E-07	Granulocytes	Rab3gap1
4.35E-11	-0.255005	0.014	0.109	6.16E-07	Granulocytes	Rps19bp1
4.38E-11	-0.3540613	0.016	0.112	6.20E-07	Granulocytes	Impa1
4.40E-11	-0.4792172	0.022	0.122	6.22E-07	Granulocytes	Hist1h1e

4.73E-11	-0.3016093	0.01	0.101	6.69E-07	Granulocytes	Cyb561d2
4.75E-11	-0.3467459	0.016	0.111	6.72E-07	Granulocytes	Pex16
4.89E-11	-0.3326688	0.014	0.108	6.92E-07	Granulocytes	Znrf2
5.01E-11	-0.3001867	0.029	0.133	7.09E-07	Granulocytes	Cul1
5.10E-11	-0.3641994	0.012	0.104	7.22E-07	Granulocytes	Cd180
5.15E-11	0.94849248	0.192	0.109	7.28E-07	Granulocytes	Fes
5.53E-11	-0.3355931	0.014	0.107	7.83E-07	Granulocytes	Srp54b
5.54E-11	-0.3123323	0.01	0.1	7.84E-07	Granulocytes	Mrpl37
5.61E-11	-0.3921928	0.035	0.14	7.95E-07	Granulocytes	Jarid2
5.66E-11	-0.3285583	0.025	0.126	8.02E-07	Granulocytes	Fam114a2
5.85E-11	-0.3192872	0.01	0.1	8.29E-07	Granulocytes	Anxa4
5.86E-11	-0.3472397	0.018	0.114	8.30E-07	Granulocytes	Smg6
6.02E-11	-0.3287481	0.022	0.121	8.52E-07	Granulocytes	Secisbp2
6.03E-11	-0.2570057	0.057	0.177	8.53E-07	Granulocytes	Nab1
6.31E-11	-0.3386625	0.018	0.113	8.93E-07	Granulocytes	Nelfe
6.31E-11	-0.281287	0.104	0.249	8.93E-07	Granulocytes	Prrc2a
6.41E-11	-0.3085042	0.106	0.249	9.08E-07	Granulocytes	Add3
6.49E-11	-0.3249937	0.018	0.115	9.19E-07	Granulocytes	Pds5a
6.98E-11	-0.308807	0.029	0.13	9.89E-07	Granulocytes	Rpf1
7.18E-11	-0.320948	0.202	0.375	1.02E-06	Granulocytes	Bcl2a1b
7.26E-11	-0.3253267	0.016	0.109	1.03E-06	Granulocytes	Ccdc86
7.42E-11	-0.2564707	0.041	0.151	1.05E-06	Granulocytes	Arl2bp
7.52E-11	-0.2652801	0.067	0.193	1.06E-06	Granulocytes	Add1
7.58E-11	-0.3353011	0.037	0.146	1.07E-06	Granulocytes	Swap70
8.06E-11	-0.2813545	0.039	0.148	1.14E-06	Granulocytes	Casp1
8.29E-11	-0.3516729	0.016	0.109	1.17E-06	Granulocytes	Mtf2
8.42E-11	-0.459487	0.012	0.101	1.19E-06	Granulocytes	Smc2
8.47E-11	-0.2931248	0.02	0.117	1.20E-06	Granulocytes	Cers5
8.70E-11	-0.26769	0.037	0.144	1.23E-06	Granulocytes	Clp1
9.09E-11	-0.3220559	0.02	0.117	1.29E-06	Granulocytes	Gle1
9.18E-11	-0.3232674	0.018	0.112	1.30E-06	Granulocytes	Fbxl6
9.24E-11	-0.3538148	0.012	0.101	1.31E-06	Granulocytes	Exoc4
9.49E-11	-0.3231002	0.016	0.11	1.34E-06	Granulocytes	Ahctf1
9.72E-11	-0.2778266	0.045	0.157	1.38E-06	Granulocytes	Sash3
1.00E-10	-0.2854594	0.07	0.195	1.42E-06	Granulocytes	Csnk1d
1.04E-10	-0.2626499	0.025	0.124	1.48E-06	Granulocytes	Txndc11
1.04E-10	-0.2896374	0.031	0.133	1.48E-06	Granulocytes	Prcp
1.06E-10	-0.2796095	0.043	0.153	1.49E-06	Granulocytes	Tial1
1.12E-10	0.65673993	0.104	0.043	1.58E-06	Granulocytes	Slc35a5
1.12E-10	-0.3129359	0.027	0.126	1.59E-06	Granulocytes	Topors
1.14E-10	-0.3199349	0.02	0.115	1.61E-06	Granulocytes	Rae1
1.15E-10	-0.2722615	0.045	0.154	1.63E-06	Granulocytes	Rbbp6
1.16E-10	-0.4943929	0.012	0.1	1.64E-06	Granulocytes	Tmem176a
1.16E-10	-0.289543	0.012	0.102	1.65E-06	Granulocytes	Fam207a
1.20E-10	-0.266829	0.035	0.14	1.70E-06	Granulocytes	Fam102a
1.20E-10	-0.2756838	0.031	0.132	1.70E-06	Granulocytes	Tank
1.25E-10	-0.3260298	0.012	0.101	1.77E-06	Granulocytes	Vps41
1.26E-10	-0.2844938	0.016	0.108	1.79E-06	Granulocytes	Ppm1b

1.27E-10	-0.3581058	0.033	0.136	1.80E-06	Granulocytes	Ipo7
1.27E-10	-0.3212362	0.027	0.126	1.80E-06	Granulocytes	Dcaf8
1.29E-10	-0.3164038	0.029	0.128	1.82E-06	Granulocytes	Ptprj
1.29E-10	-0.3113829	0.029	0.129	1.83E-06	Granulocytes	Rbm6
1.30E-10	-0.2900488	0.016	0.108	1.84E-06	Granulocytes	Spcs3
1.31E-10	-0.3244155	0.02	0.114	1.86E-06	Granulocytes	Smim15
1.31E-10	1.03334974	0.301	0.22	1.86E-06	Granulocytes	Sbno1
1.33E-10	0.81473015	0.137	0.067	1.89E-06	Granulocytes	Pira2
1.34E-10	-0.2963171	0.102	0.239	1.89E-06	Granulocytes	Smpdl3a
1.35E-10	-0.2973514	0.016	0.108	1.91E-06	Granulocytes	BC029722
1.38E-10	-0.4094033	0.02	0.113	1.96E-06	Granulocytes	Ripk2
1.39E-10	-0.3511621	0.012	0.1	1.97E-06	Granulocytes	Dusp3
1.40E-10	-0.2952102	0.016	0.107	1.98E-06	Granulocytes	Lin37
1.48E-10	-0.3852169	0.018	0.11	2.10E-06	Granulocytes	Prkd2
1.50E-10	-0.3060704	0.012	0.1	2.13E-06	Granulocytes	Ercc1
1.55E-10	-0.2848945	0.031	0.132	2.20E-06	Granulocytes	Rab22a
1.59E-10	-0.255018	0.014	0.105	2.25E-06	Granulocytes	Zfx
1.61E-10	-0.324667	0.022	0.118	2.27E-06	Granulocytes	Srsf4
1.65E-10	-0.3186265	0.049	0.161	2.34E-06	Granulocytes	Mlec
1.68E-10	-0.3070797	0.033	0.135	2.37E-06	Granulocytes	Poldip3
1.78E-10	1.01091426	0.313	0.231	2.52E-06	Granulocytes	Vsir
1.85E-10	-0.349297	0.016	0.107	2.62E-06	Granulocytes	Nfx1
1.86E-10	-0.3539208	0.018	0.11	2.63E-06	Granulocytes	Ktn1
1.87E-10	-0.2737716	0.014	0.103	2.64E-06	Granulocytes	Nop16
1.88E-10	-0.3069621	0.012	0.1	2.66E-06	Granulocytes	Sec63
1.92E-10	-0.5493923	0.014	0.102	2.72E-06	Granulocytes	Siglecg
1.95E-10	-0.3486353	0.016	0.107	2.76E-06	Granulocytes	Emsy
1.98E-10	0.74079223	0.131	0.062	2.80E-06	Granulocytes	Il18rap
1.99E-10	-0.3118166	0.012	0.1	2.82E-06	Granulocytes	Aebp2
2.00E-10	-0.3503772	0.016	0.106	2.83E-06	Granulocytes	Aak1
2.01E-10	1.00587336	0.272	0.186	2.85E-06	Granulocytes	Pag1
2.02E-10	-0.3330433	0.031	0.131	2.86E-06	Granulocytes	Bdp1
2.11E-10	-0.315063	0.014	0.102	2.99E-06	Granulocytes	Utp23
2.15E-10	-0.3277345	0.02	0.113	3.05E-06	Granulocytes	Zmiz2
2.20E-10	-0.2973855	0.012	0.1	3.11E-06	Granulocytes	Dynlt3
2.25E-10	-0.445865	0.053	0.166	3.19E-06	Granulocytes	Gbp2
2.28E-10	-0.3101324	0.014	0.103	3.23E-06	Granulocytes	Pigk
2.31E-10	-0.2998828	0.055	0.168	3.27E-06	Granulocytes	Sh3gl1
2.31E-10	-0.2502043	0.022	0.117	3.27E-06	Granulocytes	Slc48a1
2.35E-10	-0.3764517	0.018	0.11	3.33E-06	Granulocytes	Zbtb20
2.38E-10	-0.3070684	0.018	0.109	3.38E-06	Granulocytes	Ncbp2
2.39E-10	-0.2984268	0.072	0.194	3.38E-06	Granulocytes	Dgkd
2.43E-10	-0.3058952	0.016	0.107	3.43E-06	Granulocytes	Lsg1
2.52E-10	-0.2752869	0.035	0.136	3.57E-06	Granulocytes	Vps51
2.64E-10	-0.2792972	0.018	0.109	3.74E-06	Granulocytes	Gtf2f2
2.66E-10	-0.3117448	0.047	0.156	3.77E-06	Granulocytes	Numa1
2.78E-10	-0.2972941	0.037	0.139	3.93E-06	Granulocytes	Akap8l
2.82E-10	-0.3033032	0.031	0.129	4.00E-06	Granulocytes	Brd9

2.85E-10	-0.2758589	0.037	0.139	4.04E-06	Granulocytes	Clptm1
2.86E-10	-0.3120098	0.016	0.106	4.05E-06	Granulocytes	Tmem106a
2.92E-10	1.02131925	0.162	0.087	4.14E-06	Granulocytes	Trf
2.94E-10	-0.2882476	0.02	0.112	4.17E-06	Granulocytes	Mplkip
2.98E-10	-0.2614442	0.047	0.156	4.21E-06	Granulocytes	Map3k2
3.03E-10	0.71203603	0.125	0.058	4.30E-06	Granulocytes	Map3k5
3.09E-10	-0.3311848	0.031	0.129	4.37E-06	Granulocytes	Sertad2
3.09E-10	-0.308787	0.027	0.122	4.38E-06	Granulocytes	Rcn2
3.10E-10	-0.3071365	0.014	0.102	4.38E-06	Granulocytes	Pgam5
3.14E-10	-0.263672	0.057	0.172	4.44E-06	Granulocytes	Dync1i2
3.20E-10	-0.2910915	0.037	0.138	4.53E-06	Granulocytes	Desi2
3.21E-10	-0.2707654	0.047	0.154	4.55E-06	Granulocytes	Mfsd1
3.26E-10	-0.2679277	0.033	0.131	4.61E-06	Granulocytes	Qars
3.36E-10	-0.3029413	0.043	0.149	4.75E-06	Granulocytes	Lair1
3.39E-10	-0.2601408	0.031	0.129	4.80E-06	Granulocytes	Zrsr2
3.50E-10	0.74659455	0.493	0.462	4.96E-06	Granulocytes	Atp6v0b
3.53E-10	-0.3196695	0.033	0.133	5.00E-06	Granulocytes	Tle4
3.55E-10	-0.3443092	0.027	0.121	5.02E-06	Granulocytes	Herc2
3.73E-10	-0.2797377	0.499	0.731	5.27E-06	Granulocytes	Pfdn5
3.74E-10	-0.33698	0.027	0.122	5.30E-06	Granulocytes	Ubxn2a
3.78E-10	-0.3778678	0.076	0.194	5.36E-06	Granulocytes	Chd2
3.88E-10	-0.4565168	0.828	0.929	5.50E-06	Granulocytes	Gapdh
3.91E-10	-0.3065674	0.027	0.121	5.53E-06	Granulocytes	Usp48
3.96E-10	-0.3626414	0.022	0.115	5.60E-06	Granulocytes	Hip1r
3.99E-10	-0.2812914	0.02	0.112	5.65E-06	Granulocytes	Ldb1
4.03E-10	-0.2943825	0.037	0.138	5.71E-06	Granulocytes	Ddx41
4.14E-10	-0.3451985	0.018	0.107	5.86E-06	Granulocytes	Secisbp2l
4.31E-10	-0.2687767	0.016	0.104	6.10E-06	Granulocytes	Synj2bp
4.36E-10	-0.3017075	0.022	0.115	6.17E-06	Granulocytes	Pid1
4.42E-10	0.86536918	0.276	0.191	6.26E-06	Granulocytes	Lamp2
4.80E-10	-0.2952192	0.031	0.127	6.79E-06	Granulocytes	Cyfip1
4.84E-10	0.84767695	0.135	0.067	6.85E-06	Granulocytes	Man1b1
5.13E-10	-0.2710835	0.067	0.184	7.26E-06	Granulocytes	Kdm5a
5.15E-10	-0.354527	0.016	0.103	7.29E-06	Granulocytes	Uhmk1
5.27E-10	-0.3244367	0.018	0.106	7.45E-06	Granulocytes	Trnt1
5.47E-10	-0.4441426	0.018	0.105	7.74E-06	Granulocytes	Gm15987
5.63E-10	-0.2573861	0.016	0.102	7.97E-06	Granulocytes	Rab9
5.68E-10	-0.2520507	0.016	0.104	8.03E-06	Granulocytes	Sfswap
5.85E-10	-0.3690939	0.02	0.109	8.28E-06	Granulocytes	Golga4
5.87E-10	1.00267432	0.256	0.176	8.31E-06	Granulocytes	Chp1
5.90E-10	-0.3238178	0.031	0.127	8.35E-06	Granulocytes	Nlrc5
6.20E-10	-0.5084077	0.022	0.111	8.77E-06	Granulocytes	Lrp1
6.40E-10	-0.2597583	0.027	0.119	9.06E-06	Granulocytes	Zdhhc4
6.43E-10	-0.2621577	0.078	0.197	9.10E-06	Granulocytes	Rnf10
6.61E-10	0.87127507	0.344	0.265	9.35E-06	Granulocytes	Itgal
6.62E-10	-0.2703116	0.047	0.15	9.37E-06	Granulocytes	Tor1a
6.95E-10	-0.3386939	0.018	0.106	9.83E-06	Granulocytes	Rpia
7.17E-10	-0.2739522	0.033	0.13	1.01E-05	Granulocytes	Ncaph2

7.24E-10	-0.2770262	0.018	0.106	1.03E-05	Granulocytes	Ilf3
7.90E-10	-0.3194685	0.016	0.101	1.12E-05	Granulocytes	Asf1a
8.70E-10	-0.3478033	0.016	0.102	1.23E-05	Granulocytes	Zbtb38
8.76E-10	-0.3109977	0.027	0.118	1.24E-05	Granulocytes	Atp9b
8.98E-10	-0.2635978	0.027	0.119	1.27E-05	Granulocytes	Mysm1
9.12E-10	-0.2845567	0.022	0.112	1.29E-05	Granulocytes	Uso1
9.53E-10	-0.2953151	0.033	0.128	1.35E-05	Granulocytes	4833420G17
9.68E-10	1.08124769	0.393	0.342	1.37E-05	Granulocytes	Emd
9.84E-10	-0.308627	0.049	0.153	1.39E-05	Granulocytes	Rnf145
9.97E-10	-0.2673302	0.027	0.118	1.41E-05	Granulocytes	Chd8
1.01E-09	0.79537064	0.147	0.077	1.43E-05	Granulocytes	Tmem43
1.05E-09	-0.2552043	0.045	0.148	1.49E-05	Granulocytes	Cdk17
1.08E-09	0.79783097	0.153	0.082	1.53E-05	Granulocytes	Arrdc3
1.11E-09	-0.2923415	0.039	0.137	1.58E-05	Granulocytes	Celf1
1.15E-09	-0.3508703	0.027	0.117	1.63E-05	Granulocytes	Cdkn2aip
1.19E-09	0.95200838	0.137	0.071	1.68E-05	Granulocytes	Arrdc4
1.25E-09	-0.2864334	0.016	0.1	1.78E-05	Granulocytes	Lars
1.26E-09	-0.2518614	0.02	0.107	1.78E-05	Granulocytes	Ganab
1.27E-09	-0.4973105	0.029	0.119	1.79E-05	Granulocytes	Ccl9
1.34E-09	-0.2821176	0.824	0.895	1.90E-05	Granulocytes	Cfl1
1.41E-09	-0.301543	0.039	0.135	2.00E-05	Granulocytes	Coro7
1.43E-09	-0.2643311	0.027	0.118	2.02E-05	Granulocytes	Ppm1h
1.44E-09	0.6596583	0.125	0.06	2.03E-05	Granulocytes	Hk3
1.51E-09	-0.2852598	0.025	0.112	2.14E-05	Granulocytes	Abhd16a
1.53E-09	0.72821455	0.108	0.049	2.16E-05	Granulocytes	Fbxo31
1.64E-09	-0.3039158	0.053	0.159	2.32E-05	Granulocytes	Gatad2b
1.68E-09	0.98251252	0.374	0.319	2.38E-05	Granulocytes	Vasp
1.74E-09	-0.5323146	0.027	0.115	2.46E-05	Granulocytes	Serpinb1a
1.80E-09	-0.291559	0.02	0.106	2.55E-05	Granulocytes	Coro2a
1.82E-09	-0.297724	0.031	0.122	2.58E-05	Granulocytes	Epb41
1.83E-09	-0.2815087	0.045	0.145	2.59E-05	Granulocytes	Arfgap2
1.85E-09	-0.3192877	0.022	0.109	2.62E-05	Granulocytes	Araf
1.87E-09	-0.3593154	0.02	0.105	2.64E-05	Granulocytes	Hbs1l
1.94E-09	-0.2690053	0.131	0.268	2.75E-05	Granulocytes	Wsb1
2.15E-09	-0.4549968	0.051	0.152	3.05E-05	Granulocytes	Clec4a3
2.25E-09	-0.2971168	0.025	0.112	3.18E-05	Granulocytes	Brwd1
2.51E-09	-0.2506041	0.037	0.131	3.56E-05	Granulocytes	Alkbh5
2.57E-09	-0.3190936	0.992	0.999	3.64E-05	Granulocytes	Gm42418
2.83E-09	0.82385246	0.219	0.139	4.00E-05	Granulocytes	Lyst
2.89E-09	-0.292413	0.051	0.153	4.10E-05	Granulocytes	Ccdc88c
2.96E-09	-0.3010047	0.055	0.16	4.19E-05	Granulocytes	Pitpnc1
3.01E-09	-0.3125599	0.018	0.1	4.26E-05	Granulocytes	Gtf3c2
3.10E-09	-0.2539159	0.025	0.111	4.38E-05	Granulocytes	Rbbp8
3.19E-09	-0.2600526	0.027	0.115	4.52E-05	Granulocytes	Fkbp5
3.20E-09	-0.3035371	0.02	0.104	4.53E-05	Granulocytes	Stag1
3.23E-09	-0.3072744	0.022	0.107	4.57E-05	Granulocytes	Erlec1
3.49E-09	-0.3228346	0.022	0.106	4.93E-05	Granulocytes	Nup153
3.75E-09	0.83077533	0.147	0.079	5.31E-05	Granulocytes	Cln3

4.05E-09	-0.2734808	0.049	0.148	5.73E-05	Granulocytes	Swt1
4.05E-09	0.73443247	0.235	0.15	5.73E-05	Granulocytes	App
4.07E-09	0.91241031	0.172	0.1	5.76E-05	Granulocytes	Timp2
4.17E-09	-0.2717684	0.027	0.114	5.90E-05	Granulocytes	Prr7
4.25E-09	-0.3040307	0.454	0.658	6.01E-05	Granulocytes	Myl12a
4.57E-09	-0.2708091	0.045	0.143	6.47E-05	Granulocytes	Osbpl8
4.62E-09	-0.2774554	0.035	0.126	6.54E-05	Granulocytes	Usp9x
4.67E-09	-0.3326367	0.02	0.102	6.61E-05	Granulocytes	Mepce
4.99E-09	-0.2748364	0.022	0.105	7.06E-05	Granulocytes	Ddx23
5.28E-09	-0.2781957	0.029	0.115	7.47E-05	Granulocytes	Diablo
5.53E-09	1.17586461	0.192	0.119	7.82E-05	Granulocytes	Cdc42ep3
6.22E-09	-0.2589043	0.953	0.976	8.80E-05	Granulocytes	Ubb
6.23E-09	1.04615869	0.239	0.162	8.82E-05	Granulocytes	Adrb2
6.78E-09	-0.3175661	0.025	0.107	9.59E-05	Granulocytes	Xpo1
7.07E-09	-1.0902561	0.027	0.11	0.00010005	Granulocytes	Cxcl10
7.33E-09	-0.4784112	0.143	0.276	0.00010375	Granulocytes	Ccl3
7.40E-09	-0.2501519	0.029	0.115	0.00010477	Granulocytes	Chtf8
7.47E-09	-0.2625339	0.027	0.111	0.00010571	Granulocytes	Thap3
7.56E-09	-0.2881478	0.025	0.107	0.00010705	Granulocytes	Sec24c
8.51E-09	-0.4991607	0.031	0.115	0.00012046	Granulocytes	Nupr1
9.55E-09	0.82934756	0.151	0.084	0.00013515	Granulocytes	Impact
9.74E-09	1.02466774	0.319	0.256	0.00013787	Granulocytes	Mbnl2
9.94E-09	-0.2650197	0.027	0.11	0.00014073	Granulocytes	Pex13
1.13E-08	0.84347259	0.176	0.105	0.00015986	Granulocytes	Tnrc18
1.61E-08	-0.2519029	0.027	0.107	0.00022825	Granulocytes	Rnf146
1.61E-08	-0.2730164	0.033	0.117	0.00022847	Granulocytes	Atf6
1.77E-08	-0.6892635	0.534	0.646	0.00025078	Granulocytes	Klf2
1.77E-08	-0.2778678	0.035	0.121	0.0002509	Granulocytes	Utrn
1.88E-08	-0.2729354	0.031	0.113	0.00026616	Granulocytes	Gpbp1l1
2.25E-08	0.84486915	0.303	0.232	0.00031907	Granulocytes	Ehd1
2.33E-08	0.48421589	0.658	0.663	0.00032974	Granulocytes	Atp6v0c
2.42E-08	-0.2825691	0.035	0.12	0.00034188	Granulocytes	Hmgcr
2.45E-08	-0.3128536	0.022	0.1	0.00034648	Granulocytes	Ifitm10
2.58E-08	-0.2642568	0.031	0.113	0.00036583	Granulocytes	Irf2bp2
3.18E-08	-0.2564897	0.067	0.168	0.00045056	Granulocytes	Thoc2
5.20E-08	-0.2673985	0.027	0.103	0.0007355	Granulocytes	Trim59
5.93E-08	-0.2971929	0.041	0.125	0.00083965	Granulocytes	Eif2ak3
8.41E-08	1.07428265	0.266	0.199	0.00119001	Granulocytes	Ptp4a1
8.95E-08	0.87982345	0.153	0.091	0.00126689	Granulocytes	St3gal4
9.28E-08	0.95000364	0.454	0.452	0.00131348	Granulocytes	Cox17
1.10E-07	-0.2699106	0.029	0.104	0.00155584	Granulocytes	Gtf3c1
1.43E-07	-0.312896	0.029	0.103	0.002026	Granulocytes	Cmah
1.61E-07	0.73306962	0.155	0.092	0.00227973	Granulocytes	Klra2
1.79E-07	1.03187865	0.11	0.057	0.0025296	Granulocytes	Id1
1.83E-07	-0.2639033	0.029	0.102	0.00259464	Granulocytes	Irf3
2.10E-07	0.79074529	0.143	0.083	0.00296616	Granulocytes	Ebi3
2.28E-07	0.78531183	0.254	0.186	0.00322251	Granulocytes	Myo1f
2.81E-07	0.79157616	0.176	0.112	0.00398225	Granulocytes	Abr

3.30E-07	0.67075111	0.519	0.549	0.00466664	Granulocytes	Arpc5
3.56E-07	0.65461043	0.489	0.46	0.00504468	Granulocytes	Ier5
3.61E-07	0.98747692	0.28	0.221	0.00511125	Granulocytes	Glipr1
3.79E-07	-0.259921	0.031	0.102	0.00536643	Granulocytes	Xrn1
3.99E-07	0.85503537	0.38	0.345	0.0056436	Granulocytes	Cap1
4.48E-07	0.84110604	0.315	0.254	0.00633833	Granulocytes	Neurl3
5.01E-07	0.8207964	0.364	0.321	0.00708844	Granulocytes	Ptpn6
5.90E-07	0.74039469	0.17	0.109	0.00835775	Granulocytes	Fam129a
6.51E-07	0.94945528	0.223	0.16	0.00921355	Granulocytes	Slc6a6
7.90E-07	0.63363463	0.517	0.545	0.0111792	Granulocytes	Gnb2
8.06E-07	-0.4297811	0.037	0.109	0.01140492	Granulocytes	Ly6i
8.22E-07	0.69466288	0.139	0.082	0.01163908	Granulocytes	Vamp5
9.60E-07	0.76083871	0.119	0.066	0.01359313	Granulocytes	Rnasel
1.16E-06	0.90153427	0.389	0.366	0.01642473	Granulocytes	Fmnl1
1.18E-06	0.87530801	0.188	0.127	0.01663478	Granulocytes	Reep3
1.31E-06	0.94072378	0.358	0.327	0.01859708	Granulocytes	Timm10b
1.34E-06	0.72610353	0.17	0.109	0.01902848	Granulocytes	Cfap43
1.70E-06	0.75349014	0.141	0.085	0.02405751	Granulocytes	Ltb4r1
1.76E-06	0.91545938	0.239	0.183	0.02486827	Granulocytes	Trim30a
1.83E-06	1.32383379	0.384	0.359	0.02587698	Granulocytes	Cstb
2.08E-06	0.92927322	0.178	0.12	0.02940033	Granulocytes	Flot1
3.10E-06	0.85622581	0.153	0.098	0.04386985	Granulocytes	Med31
3.14E-06	0.77363777	0.184	0.126	0.0444405	Granulocytes	Sema4a
3.48E-06	0.76899666	0.135	0.082	0.04923999	Granulocytes	Wbp1l
3.75E-06	0.90450049	0.382	0.359	0.05303995	Granulocytes	Mpc1
3.92E-06	0.71638445	0.139	0.086	0.05549555	Granulocytes	Stk40
5.81E-06	0.60250078	0.151	0.096	0.08225648	Granulocytes	Xylt1
6.82E-06	0.66242463	0.141	0.088	0.09650634	Granulocytes	Snrk
9.10E-06	0.67656347	0.141	0.089	0.12879764	Granulocytes	Clec4a2
9.14E-06	0.77554172	0.227	0.173	0.12938805	Granulocytes	Il17ra
1.06E-05	0.6248126	0.115	0.067	0.14983937	Granulocytes	Trim30d
1.09E-05	0.74763849	0.127	0.078	0.1545025	Granulocytes	Pnpla7
1.30E-05	0.77684337	0.178	0.125	0.1836208	Granulocytes	Mettl9
1.51E-05	-0.2656362	0.045	0.108	0.21418765	Granulocytes	B3gnt5
1.76E-05	0.80480895	0.151	0.1	0.24985193	Granulocytes	Ell2
1.81E-05	0.80480016	0.139	0.09	0.25644547	Granulocytes	Dclre1c
2.39E-05	0.62955605	0.133	0.085	0.33775617	Granulocytes	Adssl1
2.44E-05	0.79272217	0.288	0.246	0.34555704	Granulocytes	Lbr
2.69E-05	-0.5236833	0.133	0.215	0.38113055	Granulocytes	Ly6c2
2.84E-05	0.58403582	0.106	0.063	0.40253529	Granulocytes	Rab32
3.37E-05	0.79235826	0.301	0.264	0.47700354	Granulocytes	Ssh2
3.42E-05	0.94133706	0.305	0.272	0.48374667	Granulocytes	Ddx6
4.33E-05	0.91246812	0.178	0.127	0.61316584	Granulocytes	Xdh
5.04E-05	0.58613923	0.417	0.398	0.71317184	Granulocytes	Itgb2
5.09E-05	0.6752179	0.145	0.098	0.72060272	Granulocytes	Mboat7
6.87E-05	0.63055914	0.489	0.516	0.97184407	Granulocytes	Cd53
7.56E-05	0.85434656	0.35	0.336	1	Granulocytes	1810058I24R
7.94E-05	0.62994673	0.519	0.564	1	Granulocytes	Gpx4

8.81E-05	0.62452324	0.102	0.062	1	Granulocytes	Braf
9.06E-05	0.65653539	0.485	0.529	1	Granulocytes	Dazap2
9.08E-05	0.80186171	0.213	0.168	1	Granulocytes	Map2k3
0.00010364	0.61467827	0.44	0.459	1	Granulocytes	Sh3glb1
0.00011065	0.70439303	0.147	0.103	1	Granulocytes	Ttyh3
0.00011657	0.76602015	0.192	0.146	1	Granulocytes	Mapkapk2
0.00011823	0.76785276	0.117	0.075	1	Granulocytes	Klf7
0.00013643	0.80556827	0.194	0.15	1	Granulocytes	Myd88
0.00014887	-0.3419709	0.112	0.183	1	Granulocytes	Csf1r
0.00017388	0.30040737	0.237	0.379	1	Granulocytes	Dnajb6
0.00017903	0.90185493	0.374	0.372	1	Granulocytes	Dennd4a
0.00019256	0.57075822	0.374	0.338	1	Granulocytes	Lyn
0.00025061	0.48933994	0.1	0.062	1	Granulocytes	Tmem38b
0.00025422	0.74373234	0.18	0.136	1	Granulocytes	Mpp1
0.00027801	0.81068339	0.202	0.16	1	Granulocytes	Sbno2
0.00027938	0.76796545	0.391	0.392	1	Granulocytes	Hmgn2
0.00029051	0.30049491	0.231	0.363	1	Granulocytes	Rp9
0.00029069	0.75169326	0.229	0.189	1	Granulocytes	Cd84
0.00029083	0.72131706	0.233	0.194	1	Granulocytes	Kdm7a
0.00032617	0.80066672	0.192	0.153	1	Granulocytes	Sidt2
0.00038099	0.60642307	0.104	0.067	1	Granulocytes	Bnip3
0.00047666	0.7971859	0.229	0.195	1	Granulocytes	Dynlt1b
0.00048898	0.50768944	0.11	0.073	1	Granulocytes	Rnf40
0.00058832	0.74887307	0.235	0.201	1	Granulocytes	Ppt1
0.00060432	0.26182225	0.417	0.592	1	Granulocytes	Tspo
0.00073021	0.67183387	0.401	0.414	1	Granulocytes	Apbb1ip
0.00081508	0.28255183	0.072	0.13	1	Granulocytes	Mbd2
0.00091236	0.26167553	0.217	0.329	1	Granulocytes	Tra2a
0.00094681	0.50489593	0.217	0.172	1	Granulocytes	Mpeg1
0.00098771	0.66815392	0.182	0.146	1	Granulocytes	Mapk14
0.00110121	0.76053815	0.356	0.357	1	Granulocytes	Rbms1
0.00113206	0.63483588	0.272	0.246	1	Granulocytes	Arrb2
0.00117928	0.48651394	0.121	0.084	1	Granulocytes	G6pdx
0.0012304	0.80920788	0.227	0.198	1	Granulocytes	Taf7
0.00123983	0.59950882	0.123	0.086	1	Granulocytes	Rab27a
0.00134275	0.61499297	0.133	0.096	1	Granulocytes	Dusp16
0.00146383	0.56936013	0.1	0.067	1	Granulocytes	Aldh3b1
0.00148814	0.68604002	0.119	0.084	1	Granulocytes	Slc22a15
0.00151459	0.2755688	0.137	0.214	1	Granulocytes	Mat2b
0.00151675	0.74407965	0.182	0.146	1	Granulocytes	Isg20
0.00158529	0.71323814	0.217	0.184	1	Granulocytes	Pten
0.00161165	0.25810215	0.217	0.33	1	Granulocytes	Lgals9
0.00165857	0.62178474	0.151	0.115	1	Granulocytes	Msl1
0.00176088	0.34996184	0.571	0.588	1	Granulocytes	Nr4a1
0.00191327	0.98342389	0.219	0.188	1	Granulocytes	Cks2
0.00209981	0.70584014	0.311	0.302	1	Granulocytes	Ncf4
0.0022761	0.50353768	0.106	0.074	1	Granulocytes	Ralb
0.00233383	0.78610589	0.427	0.452	1	Granulocytes	Dusp5

0.00235385	0.72061488	0.327	0.333	1	Granulocytes	Iscu
0.00248694	0.33157615	0.29	0.43	1	Granulocytes	Irf1
0.00285863	0.2811869	0.188	0.282	1	Granulocytes	Isy1
0.00307387	0.48958073	0.108	0.075	1	Granulocytes	Nbeal2
0.0031554	0.66588915	0.227	0.199	1	Granulocytes	Pbxip1
0.00318191	0.57715149	0.19	0.156	1	Granulocytes	Hck
0.00324661	0.66342054	0.19	0.158	1	Granulocytes	Snx18
0.00329218	0.62735873	0.117	0.085	1	Granulocytes	Sec14l1
0.00336854	0.71010004	0.35	0.36	1	Granulocytes	Hcls1
0.00361318	0.2710302	0.157	0.239	1	Granulocytes	Pik3cd
0.003895	0.68342679	0.198	0.168	1	Granulocytes	Ttc7
0.00454935	0.28476361	0.09	0.145	1	Granulocytes	Atp6v1a
0.00461161	0.69687902	0.266	0.248	1	Granulocytes	Pstpip1
0.00500101	0.60005404	0.119	0.088	1	Granulocytes	Tbc1d14
0.00545307	0.32308244	0.155	0.233	1	Granulocytes	Cib1
0.0054552	0.66604461	0.164	0.135	1	Granulocytes	Dstn
0.00563794	0.54307006	0.1	0.072	1	Granulocytes	Samd4b
0.00612153	0.80169491	0.301	0.297	1	Granulocytes	Cdk11b
0.00657637	0.66021954	0.143	0.114	1	Granulocytes	Notch2
0.0068841	0.64792471	0.192	0.165	1	Granulocytes	Rnf130
0.00692014	0.27012017	0.164	0.243	1	Granulocytes	Frg1
0.0069416	0.32497362	0.129	0.197	1	Granulocytes	Rassf5
0.00747342	0.57149401	0.188	0.157	1	Granulocytes	Map4k2
0.0083269	0.63907445	0.149	0.121	1	Granulocytes	N4bp1
0	4.72174577	0.905	0.154	0	NK cells	Gzma
0	3.14717649	0.757	0.01	0	NK cells	Klrb1c
0	2.77118821	0.386	0.004	0	NK cells	Klra4
0	2.75221279	0.684	0.077	0	NK cells	Serpinb9
0	2.66891678	0.741	0.066	0	NK cells	Klre1
0	2.59444878	0.635	0.003	0	NK cells	Ncr1
0	2.5273885	0.451	0.002	0	NK cells	Klra8
0	2.37047601	0.392	0.005	0	NK cells	Klra7
0	2.34912767	0.414	0.006	0	NK cells	Klra9
0	1.99003945	0.346	0.001	0	NK cells	Cma1
0	1.83213998	0.405	0.02	0	NK cells	Eomes
0	1.81123116	0.389	0.008	0	NK cells	Klrc2
0	1.77187611	0.397	0.014	0	NK cells	Il12rb2
0	1.51002141	0.324	0.003	0	NK cells	Klrb1a
2.31E-283	1.15811037	0.262	0.006	3.27E-279	NK cells	Klrb1f
9.13E-258	1.94008329	0.284	0.01	1.29E-253	NK cells	Klra3
6.51E-249	1.47756591	0.176	0.001	9.22E-245	NK cells	Serpinb9b
7.02E-244	0.77435093	0.165	0	9.94E-240	NK cells	Khdc1a
2.94E-243	2.2958452	0.73	0.13	4.16E-239	NK cells	Klrk1
1.02E-234	1.62458458	0.351	0.022	1.45E-230	NK cells	Klri2
1.64E-228	1.95060352	0.373	0.027	2.31E-224	NK cells	Klrb1b
2.99E-223	1.60062712	0.316	0.017	4.23E-219	NK cells	Klrg1
2.41E-199	2.21268074	0.568	0.089	3.41E-195	NK cells	Serpinb6b
1.99E-198	1.02269035	0.265	0.013	2.81E-194	NK cells	Adamts14

3.45E-189	2.33705911	0.695	0.147	4.88E-185	NK cells	Prf1
8.43E-186	1.56165102	0.446	0.052	1.19E-181	NK cells	Atp1b1
1.01E-169	0.53262856	0.149	0.003	1.43E-165	NK cells	Clnk
1.10E-163	2.12627543	0.786	0.233	1.56E-159	NK cells	Il2rb
1.14E-151	0.92420813	0.208	0.011	1.61E-147	NK cells	Samd3
1.25E-151	1.9989168	0.865	0.292	1.76E-147	NK cells	Ctsw
2.09E-144	1.70300633	0.503	0.089	2.96E-140	NK cells	Txk
4.72E-136	2.75034551	0.751	0.294	6.68E-132	NK cells	Irf8
9.36E-131	2.4334337	0.714	0.213	1.32E-126	NK cells	Gzmb
1.98E-125	1.51015519	0.981	0.396	2.80E-121	NK cells	Fcer1g
2.84E-124	1.41609187	0.395	0.064	4.02E-120	NK cells	Ctla2b
1.18E-123	1.66540242	0.522	0.114	1.67E-119	NK cells	Fasl
6.10E-114	1.33696587	0.957	0.413	8.64E-110	NK cells	Tyrobp
2.24E-113	0.7312714	0.141	0.006	3.17E-109	NK cells	Gpc1
3.30E-109	0.57491596	0.135	0.006	4.67E-105	NK cells	Styk1
3.58E-108	1.50604559	0.432	0.089	5.07E-104	NK cells	Arsb
2.37E-105	1.53711915	0.473	0.109	3.35E-101	NK cells	Gm19585
4.26E-104	1.50815738	0.524	0.139	6.03E-100	NK cells	Ugcg
1.91E-100	1.79478712	0.359	0.061	2.70E-96	NK cells	Xcl1
7.09E-99	1.775831	0.581	0.183	1.00E-94	NK cells	Pik3r1
1.72E-97	1.39264858	0.981	0.545	2.44E-93	NK cells	AW112010
2.85E-95	0.75766889	0.162	0.012	4.04E-91	NK cells	Spry2
1.55E-93	1.15051168	0.986	0.436	2.20E-89	NK cells	Nkg7
6.54E-92	1.95783099	0.581	0.182	9.26E-88	NK cells	Ctla2a
3.44E-91	-3.2277481	0.065	0.647	4.87E-87	NK cells	Ifi27l2a
1.88E-88	1.05710691	0.303	0.049	2.66E-84	NK cells	Pde2a
1.55E-87	2.3695965	0.53	0.158	2.19E-83	NK cells	Cd7
6.58E-86	0.99269628	0.105	0.004	9.31E-82	NK cells	Klra1
6.83E-85	1.0456128	0.986	0.533	9.67E-81	NK cells	Ccl5
1.52E-83	1.24218237	0.749	0.283	2.15E-79	NK cells	Klrd1
9.18E-83	1.16777122	0.284	0.047	1.30E-78	NK cells	Car2
1.38E-82	1.67877601	0.819	0.481	1.95E-78	NK cells	Ifngr1
1.01E-74	1.12189002	0.373	0.088	1.43E-70	NK cells	Sytl3
1.16E-74	-4.5595144	0.227	0.667	1.65E-70	NK cells	Cd74
4.28E-74	1.67701691	0.846	0.554	6.05E-70	NK cells	Zfp36l2
6.01E-74	0.63610219	0.143	0.012	8.51E-70	NK cells	Qrfp
1.54E-72	1.20807839	0.827	0.438	2.18E-68	NK cells	Anxa2
5.91E-70	0.56088007	0.135	0.011	8.37E-66	NK cells	Emid1
8.06E-69	0.87088891	0.989	0.923	1.14E-64	NK cells	Tmsb10
7.89E-68	1.00713543	0.222	0.034	1.12E-63	NK cells	Nrarp
1.76E-67	1.27158955	0.811	0.488	2.49E-63	NK cells	Selplg
2.30E-65	1.16825402	0.368	0.096	3.26E-61	NK cells	Tbx21
2.74E-65	0.9845878	0.278	0.055	3.87E-61	NK cells	Il18rap
6.22E-65	0.63090212	0.157	0.017	8.80E-61	NK cells	S1pr5
7.03E-65	-4.0173076	0.059	0.528	9.96E-61	NK cells	H2-Eb1
2.41E-63	1.04562376	0.335	0.082	3.41E-59	NK cells	Ifitm10
7.70E-63	-2.8384805	0.132	0.578	1.09E-58	NK cells	Ctss
9.59E-63	-3.9574267	0.062	0.517	1.36E-58	NK cells	H2-Ab1

6.37E-62	-4.0954925	0.086	0.532	9.02E-58	NK cells	H2-Aa
1.29E-61	-1.4768602	0.951	0.958	1.82E-57	NK cells	Fth1
7.62E-61	0.56307607	0.111	0.009	1.08E-56	NK cells	Rac3
9.91E-61	1.12265347	0.757	0.407	1.40E-56	NK cells	1-Sep
2.25E-60	1.0850782	0.903	0.697	3.18E-56	NK cells	H2afz
1.00E-58	0.7719388	0.168	0.023	1.42E-54	NK cells	Sulf2
5.04E-58	1.17364949	0.351	0.1	7.13E-54	NK cells	Dok2
3.90E-57	1.02391276	0.27	0.059	5.52E-53	NK cells	Itgax
2.89E-56	1.00726527	0.281	0.065	4.09E-52	NK cells	Adora2a
3.08E-55	0.84836358	0.224	0.044	4.35E-51	NK cells	1700025G04
3.21E-55	0.92814963	0.195	0.033	4.54E-51	NK cells	Lpin1
6.34E-55	1.02324563	0.884	0.507	8.97E-51	NK cells	Lgals1
2.48E-53	0.84554624	0.205	0.038	3.50E-49	NK cells	Olfm1
2.37E-52	1.19216662	0.386	0.127	3.36E-48	NK cells	Hsd11b1
3.08E-51	1.07624714	0.87	0.667	4.36E-47	NK cells	Ptprc
2.30E-50	0.97382268	0.765	0.442	3.26E-46	NK cells	Hcst
2.46E-49	1.17523891	0.805	0.554	3.49E-45	NK cells	Vps37b
7.93E-49	0.88991223	0.476	0.174	1.12E-44	NK cells	Cd9
1.70E-47	1.09980814	0.703	0.394	2.41E-43	NK cells	Ahnak
1.86E-47	0.81328093	0.23	0.051	2.63E-43	NK cells	Camk2n1
2.45E-47	1.08254669	0.651	0.334	3.47E-43	NK cells	Itgb1
1.11E-45	0.86231094	0.678	0.324	1.57E-41	NK cells	Sh2d2a
2.55E-45	0.49574652	0.119	0.015	3.61E-41	NK cells	Pogk
1.05E-44	1.45013963	0.497	0.218	1.49E-40	NK cells	Gem
1.19E-44	0.76928493	0.932	0.794	1.68E-40	NK cells	Myl6
4.67E-44	-1.5852457	0.105	0.497	6.61E-40	NK cells	Ltb
5.07E-44	0.92104681	0.822	0.538	7.17E-40	NK cells	Tagln2
9.30E-44	-2.1264029	0.097	0.479	1.32E-39	NK cells	Ly6a
2.58E-43	0.4211677	0.116	0.015	3.65E-39	NK cells	Cuedc1
1.18E-42	1.19580849	0.651	0.386	1.68E-38	NK cells	Itgb2
1.48E-42	1.12479695	0.538	0.25	2.09E-38	NK cells	Bhlhe40
1.54E-42	-2.193068	0.059	0.426	2.17E-38	NK cells	Cd3e
3.33E-42	0.9502987	0.224	0.054	4.72E-38	NK cells	Tmem37
1.79E-41	1.24241535	0.616	0.355	2.53E-37	NK cells	Ubald2
5.42E-41	0.76998028	0.151	0.026	7.67E-37	NK cells	Vegfa
7.29E-41	0.49516661	0.154	0.027	1.03E-36	NK cells	Rnf157
1.62E-40	0.89099739	0.192	0.042	2.30E-36	NK cells	Ern1
2.98E-40	0.99140887	0.749	0.492	4.22E-36	NK cells	Jak1
4.97E-40	0.97655469	0.562	0.276	7.04E-36	NK cells	Ccnd2
2.25E-39	0.62596603	0.97	0.95	3.19E-35	NK cells	Pfn1
4.10E-39	-1.8944916	0.011	0.344	5.80E-35	NK cells	Spi1
9.20E-39	1.21471296	0.573	0.333	1.30E-34	NK cells	Ywhaq
8.84E-38	0.52892421	0.151	0.028	1.25E-33	NK cells	Smad3
1.43E-37	0.74669895	0.235	0.063	2.02E-33	NK cells	Il18r1
3.72E-37	1.19947076	0.432	0.195	5.27E-33	NK cells	Bcl2
3.78E-37	0.69668982	0.143	0.026	5.35E-33	NK cells	Arl4d
5.67E-37	-2.522386	0.035	0.361	8.02E-33	NK cells	Cd8b1
7.96E-37	0.58407964	0.151	0.029	1.13E-32	NK cells	Ppm1j

4.19E-36	0.61273667	0.838	0.432	5.93E-32	NK cells	Ms4a4b
5.08E-36	0.89931759	0.522	0.257	7.19E-32	NK cells	Itgal
1.30E-35	-1.5474968	0.016	0.329	1.85E-31	NK cells	Ctsh
1.63E-35	-1.85367	0.5	0.694	2.31E-31	NK cells	Gpx1
1.71E-35	-2.7056853	0.014	0.323	2.41E-31	NK cells	Alox5ap
4.56E-35	-1.5863087	0.014	0.322	6.46E-31	NK cells	Ly86
8.72E-35	1.10794865	0.276	0.089	1.23E-30	NK cells	Klrc1
1.33E-34	0.68560045	0.741	0.456	1.88E-30	NK cells	Ctsd
2.45E-34	0.84617992	0.646	0.393	3.47E-30	NK cells	Rnaset2b
2.80E-34	1.02128663	0.673	0.439	3.96E-30	NK cells	Dusp5
4.83E-34	-3.0917545	0.03	0.333	6.84E-30	NK cells	Ifitm3
5.72E-34	-2.2672717	0.024	0.326	8.10E-30	NK cells	Cd8a
5.76E-34	-1.9465365	0.154	0.454	8.15E-30	NK cells	Lgals3
5.95E-34	-1.8637838	0.108	0.423	8.42E-30	NK cells	Cd3d
9.49E-34	0.94787139	0.584	0.324	1.34E-29	NK cells	Gimap4
9.69E-34	0.96145163	0.335	0.127	1.37E-29	NK cells	Lmna
1.02E-33	-2.4066237	0.324	0.583	1.44E-29	NK cells	Cst3
1.23E-33	-2.8288533	0.027	0.327	1.74E-29	NK cells	Ifitm2
1.24E-33	0.67904696	0.141	0.028	1.75E-29	NK cells	As3mt
1.41E-33	1.1061446	0.538	0.304	1.99E-29	NK cells	Ccnd3
2.09E-32	-1.9825936	0.162	0.454	2.96E-28	NK cells	Ctsb
3.57E-32	0.81502339	0.292	0.103	5.05E-28	NK cells	Padi2
5.68E-32	-1.648589	0.024	0.32	8.05E-28	NK cells	Cd83
7.08E-32	-1.3248159	0.024	0.316	1.00E-27	NK cells	Gm8369
1.99E-31	0.90540274	0.335	0.13	2.81E-27	NK cells	Ccr2
1.99E-31	0.86863782	0.554	0.319	2.82E-27	NK cells	Epsti1
2.17E-31	0.79944542	0.165	0.039	3.07E-27	NK cells	Dhrs3
2.30E-31	-1.917499	0.097	0.393	3.25E-27	NK cells	Ifi30
4.40E-31	-4.1814265	0.084	0.365	6.23E-27	NK cells	Lyz2
5.28E-31	0.8954949	0.305	0.114	7.47E-27	NK cells	Nabp1
5.96E-31	-1.6101347	0.014	0.293	8.44E-27	NK cells	Cybb
6.39E-31	-1.7466365	0.149	0.454	9.04E-27	NK cells	Cd3g
1.47E-30	0.85973959	0.408	0.19	2.09E-26	NK cells	Spn
2.01E-30	-1.4872382	0.086	0.375	2.84E-26	NK cells	Ctsz
2.11E-30	0.8365198	0.478	0.237	2.98E-26	NK cells	Prkch
3.13E-30	-3.1041617	0.035	0.312	4.43E-26	NK cells	Apoe
5.24E-30	0.6882085	0.246	0.079	7.42E-26	NK cells	Rbpms
9.97E-30	0.82091438	0.662	0.402	1.41E-25	NK cells	Id2
1.26E-29	0.92884673	0.3	0.115	1.79E-25	NK cells	Mmd
2.63E-29	-2.7994487	0.008	0.274	3.72E-25	NK cells	Ccl6
2.63E-29	0.7249009	0.684	0.411	3.72E-25	NK cells	Nr4a2
6.39E-29	-1.3347331	0.097	0.384	9.05E-25	NK cells	H2-DMa
6.68E-29	-1.9139425	0.076	0.36	9.45E-25	NK cells	Plac8
1.70E-28	-1.8036728	0.011	0.272	2.40E-24	NK cells	Lst1
1.79E-28	-1.3395193	0.23	0.507	2.54E-24	NK cells	Samhd1
1.88E-28	-1.3960778	0.092	0.378	2.67E-24	NK cells	Bcl2a1b
2.66E-28	-1.0996006	0.924	0.934	3.76E-24	NK cells	Ftl1
3.14E-28	-2.4072302	0.03	0.294	4.44E-24	NK cells	Ccr7

3.16E-28	0.52296063	0.978	0.913	4.47E-24	NK cells	H2-K1
4.17E-28	0.43403274	0.138	0.03	5.91E-24	NK cells	Dapk2
4.38E-28	0.86630215	0.249	0.087	6.20E-24	NK cells	Klhdc2
7.55E-27	0.45293838	0.135	0.03	1.07E-22	NK cells	Rab37
1.07E-26	-1.463483	0.022	0.273	1.51E-22	NK cells	Ncf2
1.31E-26	0.77963918	0.341	0.15	1.85E-22	NK cells	Metrnl
2.27E-26	-3.8732864	0.035	0.284	3.22E-22	NK cells	Il1b
2.39E-26	0.75930138	0.211	0.068	3.38E-22	NK cells	Osbpl3
3.54E-26	-2.0095662	0.014	0.259	5.02E-22	NK cells	Cd79b
4.19E-26	0.68678087	0.232	0.08	5.94E-22	NK cells	Peak1
7.46E-26	0.79586413	0.614	0.396	1.06E-21	NK cells	H2-Q6
2.21E-25	-1.2695978	0.011	0.248	3.13E-21	NK cells	Pld4
2.59E-25	-1.0995121	0.043	0.294	3.67E-21	NK cells	Gm2a
3.08E-25	-2.4408379	0.016	0.255	4.36E-21	NK cells	Wfdc17
3.33E-25	0.82481166	0.454	0.242	4.71E-21	NK cells	Gimap5
4.17E-25	-1.329324	0.105	0.361	5.90E-21	NK cells	Unc93b1
7.21E-25	-1.2595809	0	0.229	1.02E-20	NK cells	Cyp4f18
1.06E-24	-2.0877695	0.03	0.268	1.50E-20	NK cells	Ier3
1.12E-24	0.78631375	0.495	0.292	1.58E-20	NK cells	Anxa6
1.14E-24	0.45518709	0.13	0.03	1.62E-20	NK cells	Pear1
1.38E-24	0.85045741	0.616	0.414	1.95E-20	NK cells	Arl6ip5
4.17E-24	0.51085908	0.524	0.268	5.90E-20	NK cells	Cst7
4.97E-24	-1.2374566	0.446	0.66	7.04E-20	NK cells	Fos
6.23E-24	-1.3066227	0.014	0.241	8.82E-20	NK cells	Cd68
1.05E-23	0.47339598	0.941	0.79	1.49E-19	NK cells	Rac2
1.07E-23	-1.3321087	0.049	0.288	1.51E-19	NK cells	Lilrb4a
1.46E-23	-1.6804888	0.019	0.247	2.07E-19	NK cells	H2-DMb2
1.92E-23	0.77999328	0.297	0.13	2.72E-19	NK cells	Kcnab2
1.97E-23	-0.9688825	0.143	0.404	2.79E-19	NK cells	Capg
2.55E-23	-1.407099	0.027	0.256	3.61E-19	NK cells	H2-DMb1
2.76E-23	0.6450976	0.83	0.631	3.91E-19	NK cells	S100a10
3.24E-23	-2.8346266	0.032	0.257	4.58E-19	NK cells	Cd79a
4.20E-23	0.44692527	0.135	0.034	5.95E-19	NK cells	Baiap3
6.43E-23	0.59009836	0.803	0.65	9.10E-19	NK cells	Calm2
6.98E-23	-2.0731342	0.014	0.231	9.88E-19	NK cells	Ebf1
1.81E-22	0.75799099	0.73	0.588	2.56E-18	NK cells	Gnas
2.44E-22	0.54865189	0.157	0.045	3.45E-18	NK cells	Nedd4
3.10E-22	0.73060737	0.411	0.217	4.39E-18	NK cells	D16Ertd472e
3.21E-22	0.99689548	0.419	0.23	4.55E-18	NK cells	Bcl2l11
3.55E-22	0.652241	0.554	0.338	5.03E-18	NK cells	Efhd2
4.14E-22	-1.7200006	0.005	0.214	5.86E-18	NK cells	Tgfbi
4.26E-22	-0.9699555	0.005	0.215	6.03E-18	NK cells	Dok3
4.89E-22	0.69061576	0.286	0.123	6.92E-18	NK cells	Agpat3
5.03E-22	0.58482773	0.643	0.383	7.12E-18	NK cells	Ccl4
7.14E-22	0.78410336	0.443	0.254	1.01E-17	NK cells	Esyt1
8.29E-22	0.51269342	0.965	0.939	1.17E-17	NK cells	Actg1
1.01E-21	0.46680343	0.978	0.944	1.43E-17	NK cells	H2-D1
1.14E-21	0.67296162	0.841	0.678	1.61E-17	NK cells	Eif5a

1.20E-21	-1.0859873	0.008	0.217	1.70E-17	NK cells	Cd81
1.23E-21	-0.8113696	0.832	0.863	1.75E-17	NK cells	H3f3a
1.25E-21	-0.5289609	0.684	0.833	1.77E-17	NK cells	Ly6e
1.43E-21	-2.2721255	0.014	0.223	2.02E-17	NK cells	Ly6d
1.62E-21	-1.3714801	0.022	0.233	2.29E-17	NK cells	Marcksl1
1.64E-21	-0.6680159	0.876	0.864	2.33E-17	NK cells	Rps19
7.19E-21	0.54494935	0.189	0.064	1.02E-16	NK cells	Hhex
7.20E-21	0.69301091	0.53	0.312	1.02E-16	NK cells	Lck
7.28E-21	-0.8438883	0.032	0.248	1.03E-16	NK cells	Ehd1
8.52E-21	0.39522678	0.124	0.032	1.21E-16	NK cells	Syne3
1.07E-20	-1.4067229	0.016	0.218	1.52E-16	NK cells	H2-Ob
1.22E-20	-1.3994138	0.005	0.202	1.73E-16	NK cells	Cd24a
1.57E-20	-1.307191	0.041	0.251	2.22E-16	NK cells	Gngt2
4.00E-20	-1.0153027	0	0.19	5.66E-16	NK cells	Pirb
4.20E-20	0.64874907	0.238	0.096	5.94E-16	NK cells	Prkcq
6.56E-20	-1.3463045	0.019	0.217	9.28E-16	NK cells	Lgmn
7.28E-20	-1.0967206	0.081	0.304	1.03E-15	NK cells	Bcl2a1d
7.75E-20	-1.4163671	0.032	0.235	1.10E-15	NK cells	Ms4a6c
8.02E-20	-1.7313031	0.073	0.285	1.14E-15	NK cells	Plaur
9.04E-20	0.5416621	0.451	0.249	1.28E-15	NK cells	Skap1
9.94E-20	0.56207662	0.786	0.607	1.41E-15	NK cells	Il2rg
1.04E-19	-1.0631685	0.003	0.191	1.48E-15	NK cells	Plbd1
1.22E-19	-0.7652124	0.008	0.201	1.72E-15	NK cells	Cd86
2.05E-19	-1.2311976	0.003	0.188	2.90E-15	NK cells	Csf1r
2.83E-19	-0.847164	0.568	0.716	4.01E-15	NK cells	Lsp1
3.37E-19	-1.3186222	0.027	0.225	4.76E-15	NK cells	Grn
4.78E-19	0.51641429	0.865	0.733	6.77E-15	NK cells	Ier2
5.75E-19	0.69946833	0.678	0.526	8.14E-15	NK cells	Cnn2
7.59E-19	0.62165641	0.708	0.538	1.07E-14	NK cells	Dad1
9.14E-19	-0.8444102	0.03	0.224	1.29E-14	NK cells	Rgs10
1.02E-18	-0.9010172	0.005	0.186	1.44E-14	NK cells	Themis2
1.04E-18	-0.8682102	0.022	0.214	1.48E-14	NK cells	Pou2f2
1.05E-18	0.64337634	0.719	0.566	1.48E-14	NK cells	Cd47
1.27E-18	-0.8566039	0.003	0.181	1.80E-14	NK cells	Lrrc25
1.42E-18	0.50983862	0.178	0.063	2.02E-14	NK cells	Lpcat4
1.62E-18	0.75531926	0.324	0.166	2.29E-14	NK cells	Zmiz1
1.66E-18	-0.9081091	0.014	0.198	2.35E-14	NK cells	Csf2ra
1.95E-18	0.55531746	0.203	0.078	2.76E-14	NK cells	Rhoc
2.02E-18	-1.3663641	0.038	0.231	2.87E-14	NK cells	Lag3
2.64E-18	0.72655136	0.276	0.131	3.74E-14	NK cells	Lta4h
2.71E-18	-1.9711105	0.135	0.342	3.84E-14	NK cells	Msrb1
2.71E-18	-0.740922	0.003	0.179	3.84E-14	NK cells	Ifngr2
3.03E-18	0.72869177	0.627	0.458	4.29E-14	NK cells	Dusp2
3.11E-18	0.5350911	0.759	0.558	4.41E-14	NK cells	H2-Q7
3.23E-18	-1.0983599	0.011	0.192	4.57E-14	NK cells	Trib1
3.67E-18	0.53379858	0.735	0.517	5.20E-14	NK cells	Ptprcap
3.89E-18	-0.6802274	0.062	0.273	5.50E-14	NK cells	Tnfaip8
4.13E-18	-1.246488	0.008	0.186	5.84E-14	NK cells	Pla2g7

4.94E-18	-3.1907992	0.014	0.192	7.00E-14	NK cells	Cxcl2
5.06E-18	-1.0853792	0.008	0.184	7.17E-14	NK cells	Mpeg1
7.31E-18	0.76108957	0.338	0.175	1.03E-13	NK cells	Gimap9
8.98E-18	0.6411374	0.676	0.53	1.27E-13	NK cells	Sqstm1
9.03E-18	-0.824479	0.005	0.178	1.28E-13	NK cells	Nrp1
9.26E-18	0.55235044	0.808	0.697	1.31E-13	NK cells	Pabpc1
9.52E-18	-1.6985881	0.019	0.198	1.35E-13	NK cells	Cd14
1.12E-17	-1.1242868	0	0.168	1.58E-13	NK cells	Fcgr4
1.67E-17	-2.5250965	0.011	0.183	2.37E-13	NK cells	C1qc
1.72E-17	-1.1086555	0.062	0.258	2.44E-13	NK cells	Ets2
1.96E-17	0.70409081	0.551	0.382	2.78E-13	NK cells	Tpm4
1.99E-17	-1.0635562	0.073	0.269	2.82E-13	NK cells	Tpd52
2.37E-17	0.62376453	0.541	0.368	3.35E-13	NK cells	Zyx
2.39E-17	0.38631329	0.119	0.033	3.38E-13	NK cells	Kcnq5
2.51E-17	0.40564162	0.143	0.046	3.56E-13	NK cells	Fhl2
2.59E-17	-1.1131721	0.054	0.249	3.67E-13	NK cells	Lilr4b
3.17E-17	-1.0186635	0.265	0.479	4.49E-13	NK cells	Mcl1
3.20E-17	-1.4563026	0.014	0.184	4.53E-13	NK cells	Fcmr
3.27E-17	-1.4658213	0.057	0.246	4.63E-13	NK cells	Ctla4
3.35E-17	-1.1768954	0.008	0.178	4.75E-13	NK cells	Slc16a3
4.15E-17	-0.7746817	0.008	0.177	5.87E-13	NK cells	Rassf4
4.40E-17	0.55225908	0.316	0.156	6.22E-13	NK cells	Anxa1
5.44E-17	-1.3510227	0.011	0.178	7.69E-13	NK cells	Ms4a1
5.46E-17	-0.7715029	0.003	0.166	7.73E-13	NK cells	Hck
5.72E-17	0.44616738	0.157	0.054	8.09E-13	NK cells	2700038G22I
5.85E-17	-1.3531065	0.003	0.166	8.27E-13	NK cells	Mafb
6.50E-17	-1.0544646	0.008	0.175	9.20E-13	NK cells	Aif1
8.14E-17	0.7435524	0.397	0.236	1.15E-12	NK cells	Runx3
9.95E-17	0.54700564	0.697	0.54	1.41E-12	NK cells	Ndufa4
1.12E-16	-1.8629953	0.005	0.168	1.59E-12	NK cells	Hp
1.15E-16	0.72625305	0.292	0.148	1.63E-12	NK cells	Map7d1
1.24E-16	-0.7779604	0.008	0.171	1.76E-12	NK cells	Gsn
2.05E-16	0.55779359	0.376	0.204	2.90E-12	NK cells	Gpr171
2.05E-16	-1.0018519	0.17	0.384	2.91E-12	NK cells	Rel
2.21E-16	0.31636081	0.843	0.704	3.12E-12	NK cells	Btg2
2.31E-16	-2.5780792	0.022	0.188	3.27E-12	NK cells	C1qa
2.51E-16	-0.853353	0	0.156	3.55E-12	NK cells	Cd5
2.63E-16	-0.8257691	0.008	0.17	3.72E-12	NK cells	Marcks
2.94E-16	-0.8816646	0.005	0.164	4.17E-12	NK cells	App
3.03E-16	0.42781341	0.47	0.288	4.29E-12	NK cells	Mxd1
3.15E-16	-0.9191784	0.043	0.219	4.45E-12	NK cells	Ms4a4c
3.18E-16	0.61302233	0.468	0.302	4.50E-12	NK cells	Dnajc3
3.40E-16	0.80274073	0.27	0.134	4.81E-12	NK cells	Irf2bpl
3.61E-16	-0.7563598	0.003	0.16	5.10E-12	NK cells	Havcr2
4.78E-16	0.82019218	0.332	0.182	6.76E-12	NK cells	Stat4
5.82E-16	-0.928715	0.011	0.17	8.23E-12	NK cells	Ralgps2
5.99E-16	-0.7654175	0.016	0.181	8.48E-12	NK cells	Ncf1
6.61E-16	-1.1116229	0.005	0.16	9.36E-12	NK cells	Cd19

7.53E-16	0.34116596	1	0.99	1.07E-11	NK cells	Actb
9.39E-16	0.6512322	0.443	0.278	1.33E-11	NK cells	Tes
1.02E-15	0.54029486	0.211	0.089	1.45E-11	NK cells	Lax1
1.32E-15	-0.7372831	0.884	0.924	1.86E-11	NK cells	Gapdh
1.52E-15	-0.8018154	0.151	0.361	2.15E-11	NK cells	Stat1
1.74E-15	0.68991259	0.354	0.202	2.47E-11	NK cells	Pdcd4
1.83E-15	-0.7119836	0.003	0.153	2.60E-11	NK cells	Clec12a
1.87E-15	-0.7868642	0.003	0.153	2.64E-11	NK cells	Clec4a3
1.95E-15	0.71736761	0.286	0.147	2.76E-11	NK cells	Irak2
2.08E-15	-0.9340062	0.278	0.481	2.95E-11	NK cells	Serp1
2.09E-15	-2.9078568	0.046	0.21	2.96E-11	NK cells	C1qb
2.41E-15	0.61663435	0.162	0.06	3.41E-11	NK cells	Abcb9
2.46E-15	-1.3900593	0.011	0.165	3.48E-11	NK cells	Tnfrsf4
2.61E-15	-0.7413271	0	0.147	3.69E-11	NK cells	Sirpa
2.67E-15	-0.8395318	0.059	0.237	3.78E-11	NK cells	Stap1
3.51E-15	-0.6537505	0.092	0.283	4.97E-11	NK cells	Mif4gd
3.55E-15	-0.6270825	0.005	0.156	5.03E-11	NK cells	Sgms1
4.07E-15	-0.6391716	0.914	0.927	5.77E-11	NK cells	Srgn
4.22E-15	-0.7200661	0.014	0.168	5.97E-11	NK cells	Ass1
4.36E-15	0.67708644	0.195	0.084	6.18E-11	NK cells	Elmo2
4.75E-15	0.5864252	0.243	0.115	6.72E-11	NK cells	Sla2
4.78E-15	0.51889133	0.776	0.638	6.76E-11	NK cells	Myl12a
5.01E-15	-0.7182451	0.643	0.75	7.09E-11	NK cells	Ucp2
6.16E-15	0.52016594	0.314	0.158	8.72E-11	NK cells	Tnfrsf9
6.41E-15	0.70090249	0.359	0.213	9.07E-11	NK cells	Ift20
6.46E-15	-0.543339	0.205	0.414	9.14E-11	NK cells	Cmtm7
9.04E-15	-0.3454537	0.919	0.936	1.28E-10	NK cells	Rps9
9.69E-15	-0.6089475	0.003	0.146	1.37E-10	NK cells	BC028528
1.08E-14	0.59151898	0.251	0.124	1.54E-10	NK cells	Sema4a
1.13E-14	-0.9849562	0	0.141	1.60E-10	NK cells	Tgm2
1.18E-14	-0.7553522	0.003	0.145	1.67E-10	NK cells	Cd300a
1.23E-14	0.41528536	0.135	0.046	1.74E-10	NK cells	Sidt1
1.28E-14	0.57313456	0.276	0.142	1.81E-10	NK cells	Azi2
1.31E-14	0.61490131	0.411	0.257	1.86E-10	NK cells	Ptpn22
1.39E-14	-0.7397562	0.003	0.144	1.96E-10	NK cells	Slfn1
1.59E-14	-1.547352	0.043	0.204	2.24E-10	NK cells	Mt1
1.66E-14	-0.7852184	0.184	0.388	2.36E-10	NK cells	Gadd45b
1.84E-14	0.51820847	0.186	0.079	2.60E-10	NK cells	Gsap
1.92E-14	0.70327077	0.286	0.149	2.72E-10	NK cells	Atp2a3
2.11E-14	0.57143085	0.484	0.318	2.98E-10	NK cells	Gimap3
2.21E-14	0.38935266	0.154	0.058	3.13E-10	NK cells	Chn2
2.21E-14	-0.6323211	0.008	0.152	3.14E-10	NK cells	Il6ra
2.27E-14	-0.6422687	0.414	0.601	3.21E-10	NK cells	Gmfg
2.51E-14	0.36140312	0.138	0.048	3.55E-10	NK cells	Sytl2
2.76E-14	-0.8154335	0.132	0.325	3.90E-10	NK cells	Hif1a
2.79E-14	-0.7285043	0.046	0.209	3.95E-10	NK cells	Rbpj
2.81E-14	-1.5631531	0.005	0.146	3.98E-10	NK cells	Tnfaip2
3.06E-14	-1.5781827	0.405	0.539	4.33E-10	NK cells	Psap

3.22E-14	-0.9953533	0.046	0.208	4.56E-10	NK cells	Fcgr2b
3.23E-14	-0.5387587	0.986	0.961	4.58E-10	NK cells	mt-Co1
3.61E-14	0.63740591	0.238	0.115	5.10E-10	NK cells	Nup210
4.53E-14	-0.9698471	0	0.135	6.42E-10	NK cells	Cd300ld
4.74E-14	0.49661067	0.168	0.066	6.71E-10	NK cells	Clcf1
5.00E-14	0.52834119	0.678	0.558	7.08E-10	NK cells	Tpm3
5.37E-14	0.62332275	0.235	0.114	7.61E-10	NK cells	Gm4070
5.58E-14	0.40399236	0.178	0.072	7.90E-10	NK cells	St3gal6
5.97E-14	-0.6726606	0	0.134	8.45E-10	NK cells	Cd6
6.65E-14	-0.6283026	0.03	0.183	9.41E-10	NK cells	Snx9
7.09E-14	-2.4261917	0	0.133	1.00E-09	NK cells	Slpi
7.17E-14	-0.9525283	0.003	0.138	1.02E-09	NK cells	Hmox1
7.86E-14	-0.7342841	0	0.133	1.11E-09	NK cells	Sirpb1c
8.25E-14	-0.9627019	0.003	0.137	1.17E-09	NK cells	Mgst1
9.32E-14	0.55817223	0.424	0.272	1.32E-09	NK cells	Ppp1r12a
9.70E-14	-0.9696958	0.132	0.313	1.37E-09	NK cells	Klf4
1.03E-13	0.56327323	0.219	0.103	1.46E-09	NK cells	Ppp3cc
1.07E-13	0.50914728	0.316	0.173	1.51E-09	NK cells	Acap1
1.47E-13	-0.825609	0.014	0.152	2.08E-09	NK cells	H2-Oa
1.54E-13	-1.004815	0.003	0.135	2.18E-09	NK cells	C5ar1
1.54E-13	0.75726372	0.405	0.27	2.19E-09	NK cells	Ppig
1.59E-13	-0.610678	0.008	0.144	2.25E-09	NK cells	Rnase6
1.67E-13	0.50759468	0.654	0.505	2.37E-09	NK cells	Cycs
1.71E-13	0.50875564	0.186	0.081	2.42E-09	NK cells	Atp8b4
1.72E-13	0.35417608	0.897	0.84	2.44E-09	NK cells	Serf2
1.73E-13	-0.8312461	0	0.129	2.44E-09	NK cells	Gda
1.73E-13	0.34518443	0.943	0.887	2.46E-09	NK cells	Cfl1
2.34E-13	0.5384259	0.308	0.174	3.31E-09	NK cells	Btg3
2.37E-13	-0.7083946	0.219	0.41	3.36E-09	NK cells	Slfn2
2.51E-13	-0.6891208	0.003	0.133	3.55E-09	NK cells	Nfam1
2.58E-13	0.44054505	0.178	0.076	3.65E-09	NK cells	Chsy1
2.61E-13	-1.1342825	0.481	0.624	3.70E-09	NK cells	Zfp36
2.84E-13	-0.7241876	0.008	0.141	4.02E-09	NK cells	Ms4a6d
2.86E-13	-0.7561387	0.146	0.331	4.05E-09	NK cells	Rilpl2
3.14E-13	0.43658284	0.138	0.051	4.44E-09	NK cells	Gramd4
3.19E-13	-0.6142939	0	0.127	4.51E-09	NK cells	Clec4a1
3.33E-13	0.62473825	0.538	0.362	4.71E-09	NK cells	Rgs1
3.59E-13	-1.7038542	0.014	0.148	5.08E-09	NK cells	Cxcl9
3.93E-13	0.48101519	0.614	0.464	5.57E-09	NK cells	Prkar1a
4.07E-13	-1.5456303	0.003	0.131	5.76E-09	NK cells	Trem1
4.55E-13	-1.2702856	0.005	0.135	6.44E-09	NK cells	Clec4e
5.01E-13	-0.6305124	0.119	0.297	7.09E-09	NK cells	Gch1
5.43E-13	-5.4538716	0.024	0.161	7.68E-09	NK cells	S100a8
5.75E-13	-0.7605819	0.559	0.668	8.13E-09	NK cells	Atp6v0c
5.93E-13	-0.9017167	0.016	0.151	8.40E-09	NK cells	Bank1
6.07E-13	-1.367907	0.162	0.332	8.59E-09	NK cells	Atf3
6.37E-13	-0.6420152	0.016	0.152	9.02E-09	NK cells	Sh3bp5
7.19E-13	-0.6591189	0	0.123	1.02E-08	NK cells	Csf2rb

7.21E-13	0.51295059	0.349	0.205	1.02E-08	NK cells	6-Sep
7.46E-13	0.50215925	0.224	0.11	1.06E-08	NK cells	Plscr1
7.56E-13	-0.735818	0.008	0.136	1.07E-08	NK cells	Blnk
7.94E-13	0.45129791	0.157	0.064	1.12E-08	NK cells	Mmgt2
8.34E-13	0.63449159	0.278	0.154	1.18E-08	NK cells	Snx18
8.41E-13	0.41633919	0.927	0.841	1.19E-08	NK cells	Sh3bgrl3
8.41E-13	0.63838893	0.184	0.081	1.19E-08	NK cells	Bambi
8.80E-13	-0.2972754	0	0.123	1.25E-08	NK cells	1-Mar
9.18E-13	-0.6088578	0.03	0.171	1.30E-08	NK cells	Blvrb
1.05E-12	0.46830833	0.276	0.144	1.49E-08	NK cells	S1pr4
1.21E-12	-0.6982308	0.186	0.365	1.71E-08	NK cells	Ms4a6b
1.23E-12	-0.7216984	0	0.121	1.75E-08	NK cells	Pilra
1.31E-12	-1.881737	0.003	0.125	1.86E-08	NK cells	Il1r2
1.37E-12	-0.6242296	0.035	0.178	1.95E-08	NK cells	Irf5
1.38E-12	-0.6516167	0.008	0.136	1.96E-08	NK cells	Izumo1r
1.40E-12	0.52966201	0.168	0.072	1.99E-08	NK cells	Tmcc3
1.76E-12	0.4206456	0.108	0.036	2.49E-08	NK cells	F2r
1.77E-12	0.34364232	0.1	0.032	2.51E-08	NK cells	Acss2
1.80E-12	-0.6094764	0.003	0.124	2.54E-08	NK cells	Fcgr1
1.84E-12	0.48810603	0.808	0.725	2.60E-08	NK cells	Shisa5
1.93E-12	0.44653563	0.132	0.05	2.73E-08	NK cells	Sh2d1b1
1.95E-12	-0.7345811	0.086	0.249	2.75E-08	NK cells	Nrros
2.01E-12	0.26964277	0.959	0.905	2.85E-08	NK cells	Ppia
2.18E-12	-0.9237592	0.014	0.142	3.08E-08	NK cells	Adam8
2.21E-12	-0.8863892	0.041	0.183	3.13E-08	NK cells	Id3
2.26E-12	0.5025645	0.711	0.555	3.19E-08	NK cells	Emp3
2.26E-12	-0.9790752	0	0.119	3.19E-08	NK cells	Ifitm6
2.45E-12	-0.5672468	0.014	0.142	3.46E-08	NK cells	Apobec1
2.51E-12	0.54752403	0.195	0.092	3.55E-08	NK cells	Cdk2ap1
2.62E-12	-1.0953586	0.089	0.241	3.71E-08	NK cells	Mef2c
2.62E-12	1.01524286	0.338	0.203	3.71E-08	NK cells	Ly6c2
2.77E-12	-0.5381477	0.124	0.298	3.92E-08	NK cells	Acp5
2.93E-12	0.73593704	0.373	0.246	4.14E-08	NK cells	Tpst2
3.00E-12	-0.5426512	0.003	0.122	4.25E-08	NK cells	Cd40
3.03E-12	-0.8960311	0.065	0.22	4.28E-08	NK cells	Cxcr6
3.06E-12	-0.4934495	0.019	0.15	4.34E-08	NK cells	Plekho1
3.13E-12	0.59199567	0.597	0.448	4.43E-08	NK cells	Adgre5
3.13E-12	0.48394968	0.657	0.516	4.43E-08	NK cells	Reep5
3.24E-12	-0.9331196	0.005	0.126	4.59E-08	NK cells	Mzb1
3.69E-12	0.53615562	0.227	0.114	5.23E-08	NK cells	Spata13
3.98E-12	-1.7661801	0	0.116	5.64E-08	NK cells	Ifitm1
4.08E-12	-0.7734529	0.011	0.135	5.77E-08	NK cells	Cxcl16
4.21E-12	0.6687925	0.384	0.25	5.95E-08	NK cells	Wipf1
4.42E-12	0.55889437	0.143	0.058	6.26E-08	NK cells	Tnik
4.57E-12	0.75384029	0.386	0.251	6.48E-08	NK cells	Neurl3
4.70E-12	0.52248575	0.543	0.402	6.65E-08	NK cells	Anp32b
4.99E-12	-0.6419397	0.008	0.13	7.06E-08	NK cells	Slc11a1
5.02E-12	0.32726267	0.886	0.776	7.11E-08	NK cells	Arpc1b

5.14E-12	-0.6544355	0.003	0.12	7.27E-08	NK cells	Slc15a3
5.20E-12	-0.6739046	0	0.115	7.36E-08	NK cells	Nupr1
5.56E-12	-0.4983528	0	0.115	7.87E-08	NK cells	Pid1
6.01E-12	0.47850552	0.743	0.642	8.51E-08	NK cells	Ptpn18
6.77E-12	-0.5029248	0.032	0.169	9.58E-08	NK cells	Ap1s2
6.84E-12	0.52861719	0.511	0.365	9.68E-08	NK cells	Dennd4a
7.35E-12	-0.719882	0.073	0.223	1.04E-07	NK cells	Trim25
7.78E-12	0.58402923	0.176	0.08	1.10E-07	NK cells	Abcb1b
8.12E-12	0.66329792	0.305	0.185	1.15E-07	NK cells	Fyn
8.28E-12	-1.2096392	0	0.113	1.17E-07	NK cells	Csf3r
9.19E-12	-0.4569424	0.008	0.128	1.30E-07	NK cells	P2rx4
9.29E-12	-0.9058299	0.014	0.136	1.31E-07	NK cells	Fcer2a
9.42E-12	-0.7379338	0.222	0.398	1.33E-07	NK cells	S100a4
1.01E-11	-0.6708582	0.151	0.322	1.43E-07	NK cells	Snx20
1.08E-11	-0.8332353	0.008	0.125	1.53E-07	NK cells	Blk
1.19E-11	-0.5865412	0.019	0.144	1.68E-07	NK cells	Chchd10
1.31E-11	-0.9508339	0.003	0.116	1.85E-07	NK cells	Nlrp3
1.53E-11	-0.7554283	0.189	0.348	2.17E-07	NK cells	Lyn
1.57E-11	0.48500109	0.608	0.485	2.23E-07	NK cells	Ndufb11
1.61E-11	0.56336636	0.435	0.308	2.28E-07	NK cells	Bin2
1.62E-11	-0.6389909	0.284	0.446	2.30E-07	NK cells	Erp29
1.63E-11	0.51240745	0.568	0.432	2.31E-07	NK cells	Krtcap2
1.70E-11	-0.4588603	0.011	0.13	2.40E-07	NK cells	Ube2l6
1.83E-11	-0.6700164	0.024	0.151	2.59E-07	NK cells	Rasgef1b
1.84E-11	0.59787651	0.457	0.331	2.61E-07	NK cells	Dpm3
1.88E-11	0.53546367	0.165	0.074	2.65E-07	NK cells	Nt5c3
1.89E-11	0.40505113	0.784	0.661	2.68E-07	NK cells	Tmbim6
1.90E-11	0.55774403	0.289	0.167	2.69E-07	NK cells	Tm6sf1
2.15E-11	0.42276579	0.535	0.395	3.04E-07	NK cells	S100a13
2.26E-11	0.61521309	0.403	0.272	3.19E-07	NK cells	Dnajc15
2.39E-11	-1.1365533	0	0.109	3.38E-07	NK cells	Clec4d
2.53E-11	0.44166653	0.141	0.058	3.58E-07	NK cells	Gstm1
2.79E-11	-0.4388028	0.986	0.947	3.95E-07	NK cells	mt-Co2
2.81E-11	-0.6813018	0.005	0.118	3.98E-07	NK cells	Tnf
2.89E-11	0.55500946	0.805	0.688	4.10E-07	NK cells	Dnaja1
2.92E-11	0.49688773	0.465	0.33	4.13E-07	NK cells	1810058I24R
2.98E-11	-1.0528786	0.003	0.112	4.22E-07	NK cells	Ccl2
2.99E-11	-0.6882424	0.049	0.186	4.23E-07	NK cells	Rtp4
3.06E-11	0.54064597	0.227	0.116	4.33E-07	NK cells	Gimap8
3.33E-11	0.31966709	0.105	0.037	4.72E-07	NK cells	Gm14029
3.65E-11	-0.8633698	0.07	0.21	5.17E-07	NK cells	S1pr1
3.74E-11	-0.5491427	0.073	0.22	5.29E-07	NK cells	Znhit1
3.91E-11	-0.4365481	0.003	0.112	5.54E-07	NK cells	Fcgrt
3.93E-11	0.47753528	0.265	0.147	5.56E-07	NK cells	Pitpnc1
4.10E-11	-0.9912613	0.035	0.165	5.81E-07	NK cells	Ccrl2
4.10E-11	0.52086394	0.305	0.179	5.81E-07	NK cells	Amd1
4.26E-11	-0.6025685	0.003	0.111	6.03E-07	NK cells	Lrp1
4.41E-11	-0.5173798	0.138	0.305	6.25E-07	NK cells	Pold4

4.62E-11	-1.0002144	0.4	0.536	6.54E-07	NK cells	Sat1
5.10E-11	-0.6244658	0.005	0.115	7.22E-07	NK cells	Irf4
5.12E-11	-0.6454818	0.095	0.245	7.25E-07	NK cells	Prkcd
5.32E-11	0.52594881	0.214	0.111	7.53E-07	NK cells	Pik3r5
6.08E-11	-0.5061183	0.003	0.11	8.61E-07	NK cells	Bcl11b
6.24E-11	-0.8543392	0.003	0.109	8.83E-07	NK cells	Ly6i
6.56E-11	-0.5607374	0.003	0.109	9.29E-07	NK cells	Tlr2
6.87E-11	0.45337995	0.143	0.061	9.72E-07	NK cells	Golm1
7.48E-11	-0.6254546	0.068	0.208	1.06E-06	NK cells	Aldh2
7.76E-11	0.47963035	0.362	0.23	1.10E-06	NK cells	Nfil3
7.77E-11	-0.432597	0	0.104	1.10E-06	NK cells	Cd180
8.49E-11	-0.5515594	0.008	0.119	1.20E-06	NK cells	Basp1
9.30E-11	-0.6560199	0.014	0.126	1.32E-06	NK cells	Ms4a4a
9.57E-11	-0.5749682	0.005	0.112	1.36E-06	NK cells	Cfp
1.06E-10	0.44531209	0.284	0.168	1.51E-06	NK cells	Pfkp
1.12E-10	0.43814785	0.714	0.63	1.58E-06	NK cells	Cnbp
1.12E-10	-0.6357048	0.068	0.205	1.59E-06	NK cells	Pmaip1
1.15E-10	-0.4974022	0	0.102	1.63E-06	NK cells	Igsf6
1.16E-10	-0.621466	0.589	0.673	1.64E-06	NK cells	Aldoa
1.19E-10	-0.4907321	0	0.102	1.68E-06	NK cells	Klra2
1.31E-10	0.35025822	0.146	0.063	1.85E-06	NK cells	Ak3
1.32E-10	-0.7151047	0.532	0.605	1.87E-06	NK cells	Npc2
1.35E-10	-0.5299908	0.008	0.116	1.91E-06	NK cells	Ifi204
1.35E-10	0.4380532	0.724	0.622	1.92E-06	NK cells	Atp5h
1.36E-10	0.47151751	0.132	0.055	1.93E-06	NK cells	Vopp1
1.40E-10	-0.7960075	0	0.101	1.98E-06	NK cells	Il1rn
1.48E-10	-0.696968	0.027	0.145	2.10E-06	NK cells	Sdc4
1.51E-10	-0.7300888	0.032	0.155	2.14E-06	NK cells	Fabp5
1.57E-10	0.28547351	0.749	0.572	2.22E-06	NK cells	Tspo
1.63E-10	0.41980325	0.962	0.914	2.31E-06	NK cells	Cd52
1.64E-10	-0.5748364	0.003	0.105	2.33E-06	NK cells	Slamf9
1.65E-10	-0.4017315	0.003	0.105	2.34E-06	NK cells	Tmem106a
1.70E-10	-0.7713729	0.014	0.123	2.40E-06	NK cells	Fcrla
1.78E-10	0.60151203	0.311	0.195	2.53E-06	NK cells	Lat2
1.82E-10	-0.5187575	0.035	0.159	2.58E-06	NK cells	Inpp4b
1.83E-10	-0.5771672	0.178	0.341	2.59E-06	NK cells	Bst2
2.11E-10	-0.3452561	1	0.998	2.98E-06	NK cells	Gm42418
2.15E-10	-0.5961635	0.011	0.118	3.04E-06	NK cells	Actn1
2.18E-10	-1.4340344	0.003	0.104	3.08E-06	NK cells	Hdc
2.36E-10	-1.0225124	0.132	0.275	3.34E-06	NK cells	Icos
2.40E-10	-0.5276957	0.011	0.117	3.39E-06	NK cells	Cnn3
2.45E-10	-0.4560674	0.008	0.113	3.47E-06	NK cells	Fgd2
2.50E-10	-0.3853778	0.003	0.103	3.54E-06	NK cells	Bmyc
2.67E-10	-0.9472977	0.424	0.543	3.78E-06	NK cells	Taldo1
2.68E-10	0.54431652	0.516	0.395	3.79E-06	NK cells	Mdh1
2.70E-10	0.55219641	0.365	0.244	3.82E-06	NK cells	Prex1
2.73E-10	0.40594214	0.138	0.059	3.86E-06	NK cells	Gramd1b
2.78E-10	-5.6564131	0.051	0.172	3.94E-06	NK cells	S100a9

2.84E-10	1.23540573	0.351	0.245	4.03E-06	NK cells	Irf7
2.98E-10	0.46901887	0.576	0.454	4.22E-06	NK cells	Gabarapl2
3.01E-10	-2.3003172	0.003	0.102	4.25E-06	NK cells	G0s2
3.47E-10	0.42453098	0.732	0.622	4.91E-06	NK cells	Atp5d
3.79E-10	0.43989331	0.641	0.541	5.36E-06	NK cells	Serbp1
4.00E-10	-0.4238524	0.003	0.102	5.66E-06	NK cells	Btk
4.08E-10	0.45058763	0.6	0.474	5.77E-06	NK cells	Rnaset2a
4.08E-10	-0.6784575	0.011	0.115	5.78E-06	NK cells	Fam43a
4.67E-10	-0.4504193	0.003	0.101	6.61E-06	NK cells	Gm6377
5.06E-10	-0.4830999	0.016	0.123	7.17E-06	NK cells	Naaa
5.24E-10	-0.4860306	0.046	0.17	7.41E-06	NK cells	Agpat4
5.64E-10	-0.3849638	0.016	0.124	7.98E-06	NK cells	Cd2ap
5.80E-10	0.50811716	0.305	0.191	8.21E-06	NK cells	Ubac2
6.49E-10	0.47402588	0.184	0.091	9.19E-06	NK cells	Mical1
6.97E-10	-0.4058726	0.989	0.946	9.87E-06	NK cells	mt-Co3
7.12E-10	-0.4650508	0.005	0.104	1.01E-05	NK cells	Lpcat2
7.52E-10	-0.5619917	0.008	0.108	1.07E-05	NK cells	C3
7.63E-10	0.55012983	0.47	0.355	1.08E-05	NK cells	Slc3a2
7.77E-10	-0.487567	0.222	0.394	1.10E-05	NK cells	Nap1l1
8.05E-10	0.48507447	0.5	0.371	1.14E-05	NK cells	Leprotl1
8.78E-10	-0.295325	0.005	0.104	1.24E-05	NK cells	Tspan31
8.85E-10	0.46667142	0.443	0.314	1.25E-05	NK cells	Fosl2
8.93E-10	-0.4508879	0.492	0.648	1.26E-05	NK cells	Limd2
9.66E-10	-0.3341854	0.051	0.179	1.37E-05	NK cells	Susd3
9.81E-10	-0.53234	0.957	0.919	1.39E-05	NK cells	mt-Cytb
1.03E-09	0.57977332	0.295	0.184	1.46E-05	NK cells	Pld3
1.08E-09	0.45307877	0.241	0.134	1.53E-05	NK cells	Sh2d1a
1.13E-09	0.35207851	0.622	0.491	1.59E-05	NK cells	Ppp1r18
1.14E-09	0.44795845	0.57	0.438	1.62E-05	NK cells	Tubb4b
1.14E-09	-3.2165476	0.005	0.101	1.62E-05	NK cells	Retnlg
1.18E-09	0.46035784	0.459	0.338	1.67E-05	NK cells	B4galnt1
1.27E-09	-0.8269345	0.062	0.185	1.80E-05	NK cells	Rnf149
1.31E-09	0.45946813	0.122	0.05	1.85E-05	NK cells	Mgat5
1.33E-09	-0.5921048	0.005	0.101	1.89E-05	NK cells	Siglecg
1.35E-09	0.43038473	0.216	0.118	1.91E-05	NK cells	11-Sep
1.45E-09	-0.5897601	0.011	0.11	2.06E-05	NK cells	Pltp
1.46E-09	-0.6968822	0.014	0.114	2.07E-05	NK cells	Scd1
1.47E-09	0.40958842	0.565	0.439	2.08E-05	NK cells	Tnfaip3
1.48E-09	0.46447153	0.208	0.111	2.09E-05	NK cells	D1Ertd622e
1.49E-09	0.31152813	0.416	0.274	2.11E-05	NK cells	Fgl2
1.52E-09	-0.534917	0.073	0.203	2.14E-05	NK cells	Lamp2
1.54E-09	-0.8206929	0.07	0.198	2.18E-05	NK cells	Plk3
1.54E-09	0.40968459	0.181	0.093	2.18E-05	NK cells	Ube2e3
1.56E-09	0.6875075	0.17	0.086	2.20E-05	NK cells	Borcs7
1.79E-09	0.39038088	0.17	0.083	2.54E-05	NK cells	Phf1
1.84E-09	0.3929753	0.638	0.512	2.61E-05	NK cells	H2afj
1.87E-09	0.49369712	0.232	0.131	2.65E-05	NK cells	Tecpr1
2.09E-09	-0.3784954	0.135	0.282	2.96E-05	NK cells	Ak2

2.11E-09	0.4703699	0.205	0.11	2.99E-05	NK cells	Usp48
2.14E-09	-0.5210039	0.062	0.188	3.03E-05	NK cells	Eif4ebp1
2.16E-09	-0.4439487	0.014	0.112	3.06E-05	NK cells	Slamf6
2.25E-09	0.52930991	0.292	0.183	3.19E-05	NK cells	Gnptg
2.41E-09	-0.4980698	0.014	0.111	3.42E-05	NK cells	Sdc3
2.66E-09	-0.4095427	0.03	0.139	3.77E-05	NK cells	Adam19
2.71E-09	-0.6111566	0.159	0.301	3.83E-05	NK cells	Anxa5
2.91E-09	0.52150893	0.305	0.193	4.11E-05	NK cells	Acot7
3.16E-09	-0.3677335	0.03	0.139	4.47E-05	NK cells	Rftn1
3.65E-09	0.5046154	0.238	0.136	5.16E-05	NK cells	Rin3
3.80E-09	-0.7784457	0.049	0.164	5.38E-05	NK cells	Gbp2
3.85E-09	0.51101205	0.643	0.558	5.45E-05	NK cells	Rap1b
4.01E-09	-0.4701274	0.057	0.178	5.68E-05	NK cells	Gpr183
4.50E-09	-0.4738335	0.178	0.331	6.37E-05	NK cells	Ptpn6
4.69E-09	-0.5856245	0.016	0.114	6.64E-05	NK cells	Serpinb1a
4.88E-09	0.5879705	0.362	0.255	6.90E-05	NK cells	Tram1
4.89E-09	0.45394536	0.459	0.347	6.92E-05	NK cells	Dock2
5.05E-09	-0.5405045	0.214	0.364	7.14E-05	NK cells	Aprt
5.19E-09	0.5238727	0.189	0.099	7.35E-05	NK cells	Pim2
5.39E-09	0.3272541	0.141	0.065	7.63E-05	NK cells	Scyl1
5.42E-09	0.51739993	0.357	0.243	7.67E-05	NK cells	Mknk2
5.80E-09	-0.5542421	0.141	0.28	8.22E-05	NK cells	Hmgn1
5.81E-09	0.31703725	0.884	0.836	8.22E-05	NK cells	Rpl10-ps3
5.89E-09	-0.5519147	0.089	0.215	8.34E-05	NK cells	Pkig
6.07E-09	-0.3778604	0.97	0.898	8.60E-05	NK cells	Rps20
6.34E-09	-0.5289396	0.051	0.165	8.98E-05	NK cells	Map4k2
6.68E-09	-0.6678544	0.17	0.308	9.46E-05	NK cells	Glrx
6.72E-09	0.54185808	0.268	0.161	9.52E-05	NK cells	Rnf130
6.87E-09	0.55664716	0.165	0.082	9.72E-05	NK cells	Cd160
6.95E-09	-0.4181951	0.03	0.136	9.83E-05	NK cells	Rnf19b
7.06E-09	-0.3441906	0.011	0.103	9.99E-05	NK cells	Sgk3
7.77E-09	0.44020176	0.476	0.356	0.00011004	NK cells	Saraf
8.25E-09	0.52952196	0.254	0.154	0.00011673	NK cells	Gatad2a
8.34E-09	-0.6757396	0.105	0.232	0.000118	NK cells	Dut
8.47E-09	0.51560358	0.235	0.139	0.00011992	NK cells	Sike1
8.56E-09	-0.4610023	0.427	0.582	0.00012116	NK cells	Ypel3
8.87E-09	-0.5575208	0.011	0.102	0.00012563	NK cells	Snn
9.24E-09	0.48613147	0.289	0.187	0.0001308	NK cells	Cers2
9.45E-09	-0.4277392	0.019	0.115	0.00013384	NK cells	Dck
9.57E-09	-0.4275975	0.027	0.128	0.00013551	NK cells	Arl5c
9.69E-09	-0.3695709	0.019	0.116	0.00013712	NK cells	Smco4
9.74E-09	-0.3931375	0.141	0.286	0.00013786	NK cells	Grb2
9.95E-09	-0.8502193	0.078	0.192	0.00014086	NK cells	Cd55
1.01E-08	0.42960125	0.17	0.088	0.00014364	NK cells	Lrrk1
1.02E-08	0.55144202	0.319	0.213	0.00014387	NK cells	Os9
1.15E-08	0.54897944	0.349	0.242	0.00016217	NK cells	Ikzf1
1.15E-08	0.42689878	0.211	0.12	0.00016283	NK cells	Vcpkmt
1.16E-08	-0.5646064	0.019	0.114	0.00016355	NK cells	Emilin2

1.20E-08	0.32419866	0.841	0.781	0.00016938	NK cells	Cox8a
1.26E-08	-0.3877019	0.027	0.127	0.00017839	NK cells	Ptprj
1.29E-08	0.54480344	0.211	0.121	0.000182	NK cells	Atp6v0a2
1.32E-08	-0.529729	0.046	0.156	0.00018725	NK cells	Myadm
1.41E-08	-0.4422093	0.065	0.18	0.00020022	NK cells	Bri3bp
1.44E-08	-0.5071448	0.146	0.284	0.0002038	NK cells	Tap1
1.47E-08	-0.4401694	0.014	0.104	0.00020763	NK cells	Gm15987
1.56E-08	-0.535481	0.149	0.289	0.00022018	NK cells	Smchd1
1.57E-08	-0.2545778	0.011	0.1	0.00022248	NK cells	Prdx4
1.58E-08	-0.5430521	0.124	0.255	0.00022343	NK cells	Sp140
1.62E-08	-0.5488655	0.046	0.153	0.00022868	NK cells	Isg20
1.62E-08	-0.6679646	0.019	0.112	0.00022941	NK cells	Cd300lf
1.68E-08	-0.566859	0.343	0.478	0.00023812	NK cells	Gdi2
1.72E-08	-0.3885611	0.022	0.118	0.00024325	NK cells	Ccl9
1.90E-08	-0.5701019	0.051	0.16	0.00026835	NK cells	Fbxl5
2.00E-08	0.44598932	0.495	0.385	0.0002826	NK cells	Cd48
2.03E-08	0.30404584	0.865	0.802	0.00028695	NK cells	Laptm5
2.09E-08	-0.4664859	0.051	0.161	0.00029553	NK cells	Trps1
2.15E-08	0.41387398	0.535	0.426	0.00030463	NK cells	Plek
2.26E-08	-0.9677622	0.127	0.253	0.00032058	NK cells	Lmnb1
2.27E-08	-0.3600694	0.165	0.314	0.00032167	NK cells	Mrpl54
2.28E-08	0.28314574	0.735	0.628	0.00032246	NK cells	Ppp1ca
2.47E-08	0.53636782	0.184	0.101	0.00034913	NK cells	Scimp
2.52E-08	-0.4952492	0.108	0.237	0.00035628	NK cells	Smpdl3a
2.59E-08	-0.7025892	0.054	0.161	0.00036601	NK cells	Ctsl
2.88E-08	-0.4748345	0.197	0.342	0.0004071	NK cells	Irf2
2.88E-08	0.48040971	0.286	0.187	0.00040773	NK cells	Rinl
3.12E-08	0.40442214	0.586	0.477	0.00044159	NK cells	Arf1
3.19E-08	-0.5670166	0.049	0.154	0.00045207	NK cells	2310001H17I
3.20E-08	-0.3085738	0.022	0.114	0.00045293	NK cells	Pafah1b3
3.21E-08	-0.551541	0.051	0.159	0.00045455	NK cells	Ckb
3.33E-08	0.56579335	0.297	0.191	0.00047132	NK cells	Sptbn1
3.46E-08	-0.4612893	0.065	0.179	0.00049001	NK cells	Uhrf2
3.60E-08	0.4406132	0.376	0.249	0.00050911	NK cells	Tnfrsf18
3.72E-08	-0.3993351	0.119	0.251	0.00052648	NK cells	Hmox2
3.76E-08	-0.3144077	0.062	0.176	0.00053261	NK cells	Evi2a
3.82E-08	0.41188722	0.681	0.563	0.000541	NK cells	Hsp90b1
3.97E-08	0.29963769	0.743	0.638	0.00056178	NK cells	Sumo2
4.00E-08	0.42606699	0.186	0.102	0.00056685	NK cells	Tinf2
4.06E-08	0.6479714	0.381	0.284	0.00057408	NK cells	Slc9a3r1
4.08E-08	-0.4259543	0.046	0.149	0.00057822	NK cells	Map4k1
4.09E-08	-0.3330884	0.062	0.174	0.00057927	NK cells	Rnf213
4.10E-08	0.31664964	0.581	0.46	0.00058092	NK cells	Tln1
4.17E-08	0.33182597	0.154	0.078	0.00058978	NK cells	Camk2g
4.35E-08	-0.3784694	0.049	0.154	0.000616	NK cells	Lpxn
4.56E-08	-0.9811646	0.397	0.487	0.00064617	NK cells	Prdx5
4.68E-08	-0.527949	0.154	0.286	0.00066193	NK cells	Sh3kbp1
4.89E-08	-0.464089	0.059	0.168	0.00069205	NK cells	Sbno2

5.07E-08	-0.3800817	0.038	0.137	0.00071794	NK cells	Myo1c
5.11E-08	-0.693793	0.354	0.484	0.00072286	NK cells	Pde4b
5.17E-08	-0.4623035	0.03	0.124	0.00073159	NK cells	Irgm1
5.19E-08	0.25394371	0.865	0.755	0.00073505	NK cells	Cox4i1
5.34E-08	-0.53703	0.022	0.112	0.00075637	NK cells	Ccr8
5.70E-08	-0.3482597	0.032	0.13	0.00080746	NK cells	Tank
5.75E-08	-0.6007334	0.054	0.157	0.00081463	NK cells	Dmxl1
5.93E-08	0.6329728	0.311	0.214	0.00083887	NK cells	Tmem30a
6.50E-08	0.43719539	0.473	0.37	0.00092049	NK cells	Tmem234
6.58E-08	0.43959636	0.284	0.185	0.00093167	NK cells	Myo1f
6.85E-08	-0.4766021	0.046	0.146	0.0009694	NK cells	Asah1
7.63E-08	0.46717884	0.13	0.062	0.00108052	NK cells	Adamts10
7.82E-08	-0.3472058	0.019	0.106	0.00110666	NK cells	Rassf3
7.84E-08	-0.6681404	0.078	0.188	0.00110999	NK cells	Tox
7.99E-08	0.42583418	0.343	0.233	0.0011314	NK cells	Tbc1d10c
8.09E-08	0.47264182	0.238	0.145	0.00114584	NK cells	Prkacb
8.28E-08	0.48276656	0.165	0.087	0.00117171	NK cells	Dnajb4
8.78E-08	-0.5112036	0.173	0.312	0.00124282	NK cells	Gpcpd1
8.87E-08	-0.6417321	0.211	0.333	0.00125591	NK cells	Napsa
9.16E-08	0.45314589	0.522	0.414	0.00129676	NK cells	Slc38a2
9.89E-08	0.34660261	0.616	0.469	0.00139986	NK cells	Cd2
1.04E-07	0.2871789	0.111	0.049	0.00146905	NK cells	Ptpn4
1.05E-07	0.48201121	0.224	0.136	0.00148663	NK cells	Ttc39b
1.14E-07	0.45319098	0.127	0.061	0.00161046	NK cells	Apobr
1.17E-07	-0.5052745	0.368	0.492	0.00165894	NK cells	Scand1
1.19E-07	0.37788016	0.53	0.405	0.00169018	NK cells	Gimap6
1.24E-07	0.57458052	0.257	0.163	0.00176157	NK cells	Suco
1.35E-07	-0.3149415	0.022	0.108	0.00191741	NK cells	Dnase2a
1.43E-07	-0.3363106	0.092	0.211	0.00202142	NK cells	Got1
1.48E-07	-0.541902	0.038	0.13	0.00209077	NK cells	Fgr
1.50E-07	0.46601339	0.254	0.159	0.00212919	NK cells	Fryl
1.55E-07	-0.4557177	0.019	0.103	0.00219157	NK cells	Dusp16
1.55E-07	0.34747414	0.17	0.093	0.00219423	NK cells	Lrrfip2
1.58E-07	-0.4321499	0.103	0.221	0.00223692	NK cells	Hexb
1.60E-07	-0.4335693	0.054	0.154	0.00227083	NK cells	Pxk
1.65E-07	-0.6489536	0.397	0.534	0.00233428	NK cells	Ppp1r15a
1.65E-07	0.52962347	0.292	0.197	0.00233867	NK cells	Evl
1.79E-07	0.4555089	0.273	0.176	0.00253815	NK cells	Isca1
1.92E-07	0.37178089	0.192	0.108	0.00272496	NK cells	Cpne3
1.95E-07	-0.3953666	0.176	0.309	0.00276061	NK cells	Slc25a4
1.99E-07	0.27188823	0.727	0.621	0.0028207	NK cells	Capzb
2.09E-07	-0.3344457	0.041	0.136	0.0029586	NK cells	Soat1
2.14E-07	-0.310708	0.059	0.162	0.00302259	NK cells	Igtp
2.26E-07	0.44031366	0.173	0.097	0.00320627	NK cells	Coro2a
2.27E-07	-0.3618223	0.054	0.154	0.00320713	NK cells	Scpep1
2.35E-07	-0.3591412	0.019	0.102	0.00332933	NK cells	Alcam
2.36E-07	0.46235283	0.192	0.113	0.00334003	NK cells	Capn2
2.39E-07	0.38622572	0.138	0.07	0.00338364	NK cells	Crot

2.53E-07	-0.5930579	0.481	0.605	0.00357711	NK cells	Nfkbia
2.57E-07	-0.4929221	0.041	0.133	0.00363366	NK cells	Maf
2.65E-07	-0.2657603	0.046	0.142	0.00374466	NK cells	Phyh
2.70E-07	0.3820517	0.416	0.307	0.00381639	NK cells	Gimap1
2.74E-07	-0.3643611	0.035	0.126	0.00388578	NK cells	Cdk6
2.76E-07	0.57978648	0.327	0.227	0.00390714	NK cells	Slamf7
2.94E-07	0.41138821	0.392	0.292	0.00415676	NK cells	Pycard
3.16E-07	-0.3384451	0.065	0.17	0.00447166	NK cells	Slc6a6
3.39E-07	0.46797777	0.314	0.217	0.0047957	NK cells	Tonsl
3.40E-07	0.47259915	0.546	0.443	0.00480601	NK cells	Pdia3
3.59E-07	-0.5998419	0.381	0.493	0.00508083	NK cells	Mif
3.61E-07	-0.3350627	0.114	0.231	0.00510383	NK cells	Baz1a
3.67E-07	-0.5093771	0.032	0.118	0.0051895	NK cells	Fchsd2
3.96E-07	-0.731869	0.105	0.212	0.00560822	NK cells	Plin2
4.02E-07	0.26043245	0.786	0.711	0.00569312	NK cells	Cdc42
4.07E-07	0.50158179	0.208	0.128	0.00576212	NK cells	Me2
4.19E-07	0.38709365	0.376	0.274	0.00593566	NK cells	Cd82
4.31E-07	-0.3158221	0.03	0.116	0.00609583	NK cells	Tubb6
4.40E-07	-0.3238078	0.843	0.863	0.00622957	NK cells	Coro1a
4.43E-07	0.54509122	0.278	0.189	0.0062715	NK cells	Lfng
4.69E-07	-0.4537449	0.346	0.47	0.00664174	NK cells	Atp6v0b
4.99E-07	-0.6750422	0.246	0.367	0.0070706	NK cells	Cstb
5.01E-07	-0.4532273	0.181	0.311	0.00708618	NK cells	Il10ra
5.03E-07	0.32433097	0.132	0.067	0.00711528	NK cells	Arhgap18
5.27E-07	0.44475464	0.165	0.092	0.00745474	NK cells	Galnt2
5.30E-07	0.48024912	0.335	0.243	0.00750428	NK cells	Pdcd6ip
5.30E-07	-0.4374864	0.065	0.164	0.00750535	NK cells	Cndp2
5.42E-07	-0.3929678	0.37	0.516	0.00767516	NK cells	Akap13
5.56E-07	0.35600205	0.592	0.493	0.00787303	NK cells	Tra2b
5.57E-07	-0.5886133	0.097	0.203	0.00787947	NK cells	Picalm
5.93E-07	-0.308318	0.043	0.134	0.00839545	NK cells	Hmgcl
5.94E-07	0.44735432	0.232	0.148	0.00841282	NK cells	Ssr1
6.19E-07	-1.0568727	0.027	0.108	0.00875901	NK cells	Cxcl10
6.20E-07	0.35153897	0.595	0.504	0.00877764	NK cells	Mbnl1
6.33E-07	0.45191168	0.165	0.092	0.00895993	NK cells	Dbf4
6.54E-07	0.3733667	0.511	0.418	0.00926452	NK cells	Ywhah
6.77E-07	0.3882968	0.162	0.089	0.00958803	NK cells	Sephs2
6.81E-07	-0.5918436	0.214	0.333	0.00963681	NK cells	Lamp1
6.92E-07	0.42245226	0.308	0.214	0.00979417	NK cells	Runx1
7.00E-07	-0.3133152	0.022	0.1	0.00991107	NK cells	Nuak2
7.54E-07	-0.3081593	0.038	0.125	0.01067995	NK cells	Cyfip1
8.04E-07	-0.4591057	0.035	0.117	0.0113859	NK cells	Cfap43
8.11E-07	-0.3047195	0.049	0.142	0.01148152	NK cells	Itpr1
8.56E-07	-0.4696631	0.324	0.438	0.01212405	NK cells	Psmb9
8.72E-07	0.38719021	0.178	0.103	0.01233991	NK cells	Ly9
8.93E-07	0.30578642	0.141	0.074	0.01264574	NK cells	1700047I17R
9.12E-07	-0.6295461	0.27	0.384	0.01290801	NK cells	Foxp1
9.46E-07	0.34417563	0.651	0.559	0.01339546	NK cells	Ndufa3

9.68E-07	-0.5523744	0.119	0.227	0.0136986	NK cells	Arhgap31
1.10E-06	-0.5099343	0.03	0.108	0.01560059	NK cells	B3gnt5
1.12E-06	-0.3384201	0.062	0.157	0.01587221	NK cells	Abi3
1.13E-06	0.26345288	0.732	0.632	0.01594347	NK cells	Cox5b
1.15E-06	0.38172659	0.203	0.124	0.01632104	NK cells	Prpf19
1.16E-06	-0.935192	0.081	0.174	0.01637712	NK cells	Gsr
1.18E-06	-0.3508494	0.051	0.142	0.01674896	NK cells	Gsto1
1.19E-06	-0.4773618	0.057	0.148	0.01689349	NK cells	Lyst
1.21E-06	-0.2733238	0.03	0.111	0.01714901	NK cells	Acer3
1.22E-06	-0.4352617	0.054	0.143	0.01731664	NK cells	Swap70
1.37E-06	-0.2639377	0.908	0.864	0.01941935	NK cells	Rpl35
1.40E-06	0.37460405	0.305	0.215	0.01975827	NK cells	Gm9844
1.40E-06	-0.4660592	0.186	0.309	0.01978027	NK cells	Ncf4
1.41E-06	-0.4390659	0.111	0.219	0.01990982	NK cells	Plekho2
1.43E-06	0.32497812	0.146	0.078	0.0202402	NK cells	Tatdn2
1.47E-06	0.4872829	0.414	0.332	0.02076999	NK cells	Cuta
1.53E-06	-0.3040878	0.054	0.146	0.02171605	NK cells	Etv6
1.56E-06	-0.296732	0.097	0.205	0.02212734	NK cells	Mrpl14
1.57E-06	-0.4055843	0.097	0.199	0.0222628	NK cells	Cenpa
1.58E-06	0.30908871	0.146	0.079	0.02239509	NK cells	Inpp1
1.59E-06	-0.3938862	0.105	0.214	0.02244365	NK cells	Uvrag
1.63E-06	-0.4523212	0.159	0.28	0.02311018	NK cells	Pdcd1
1.66E-06	-0.537905	0.043	0.128	0.02343829	NK cells	Gzmk
1.70E-06	0.46930972	0.251	0.171	0.02412319	NK cells	Cmc1
1.75E-06	-0.5108388	0.159	0.267	0.02471086	NK cells	Syk
1.76E-06	0.45946849	0.289	0.204	0.02484663	NK cells	Mxd4
1.80E-06	-0.3570413	0.1	0.204	0.02542234	NK cells	Cnp
1.83E-06	-0.4760159	0.238	0.358	0.02587512	NK cells	Ctsa
1.84E-06	-0.4391882	0.116	0.224	0.02605056	NK cells	Birc3
1.84E-06	-0.3209138	0.054	0.143	0.02609809	NK cells	Mpp1
1.86E-06	-0.3656113	0.078	0.178	0.02639596	NK cells	Hexa
1.88E-06	0.35721061	0.222	0.139	0.0266166	NK cells	Arl2bp
1.99E-06	0.45886373	0.216	0.137	0.02810464	NK cells	Flt3l
2.02E-06	-0.3869684	0.068	0.162	0.02853878	NK cells	Serpinb6a
2.02E-06	0.50435383	0.222	0.143	0.0285534	NK cells	Otulin
2.02E-06	0.38660528	0.181	0.11	0.02861655	NK cells	Pea15a
2.08E-06	0.39553124	0.341	0.244	0.02939767	NK cells	Lbr
2.09E-06	0.41665295	0.246	0.162	0.02964454	NK cells	Ccr5
2.10E-06	-0.3862364	0.189	0.313	0.0296672	NK cells	Smim14
2.10E-06	0.44565136	0.522	0.439	0.0297601	NK cells	H2-Q4
2.13E-06	0.51424812	0.503	0.429	0.03008767	NK cells	Arf6
2.19E-06	-0.3047425	0.041	0.123	0.03096285	NK cells	Slc43a2
2.26E-06	-0.3481198	0.046	0.13	0.03196505	NK cells	Mgst2
2.28E-06	-0.6787571	0.13	0.238	0.03233822	NK cells	Nfkbid
2.39E-06	-0.5149713	0.027	0.101	0.03387114	NK cells	AA467197
2.40E-06	0.34398654	0.678	0.576	0.03399962	NK cells	Ndufa13
2.46E-06	-0.6702535	0.043	0.124	0.03477001	NK cells	Il7r
2.47E-06	0.45396911	0.154	0.088	0.03492328	NK cells	Gnptab

2.49E-06	-0.4527789	0.173	0.29	0.03518718	NK cells	Il21r
2.51E-06	-0.4435179	0.132	0.242	0.03547187	NK cells	Vsir
2.55E-06	-0.4773871	0.081	0.174	0.03609695	NK cells	Ero1lb
2.56E-06	-0.3591548	0.027	0.102	0.03627893	NK cells	Rab20
2.57E-06	-0.3070108	0.046	0.13	0.036369	NK cells	Fam111a
2.58E-06	0.50421736	0.303	0.219	0.03646986	NK cells	Tex261
2.66E-06	-0.3385108	0.062	0.152	0.03765981	NK cells	Map2k1
2.69E-06	-0.4675349	0.368	0.463	0.03807939	NK cells	Vamp8
2.77E-06	0.41276129	0.37	0.282	0.03922169	NK cells	Hypk
2.80E-06	-0.256883	0.943	0.897	0.0396911	NK cells	Rps26
2.89E-06	-0.4318883	0.138	0.25	0.04089362	NK cells	Tiparp
3.05E-06	0.54416498	0.381	0.272	0.04318628	NK cells	Sell
3.12E-06	0.25360711	0.176	0.101	0.04411737	NK cells	Plcxd2
3.14E-06	0.44501354	0.192	0.119	0.0444718	NK cells	Sertad2
3.25E-06	0.319089	0.105	0.051	0.04594632	NK cells	Prr5l
3.25E-06	-0.462516	0.035	0.112	0.04599896	NK cells	Gla
3.35E-06	-0.6165681	0.535	0.623	0.04743568	NK cells	Stk17b
3.36E-06	0.44672471	0.146	0.082	0.04753289	NK cells	Rgs3
3.44E-06	-0.2924995	0.046	0.13	0.04866367	NK cells	Klk8
3.62E-06	0.48385439	0.181	0.11	0.05129585	NK cells	Riok1
3.63E-06	0.45233195	0.184	0.111	0.05136475	NK cells	Itgam
3.65E-06	-0.3249737	0.097	0.198	0.05165057	NK cells	Pip4k2a
3.65E-06	-0.3552731	0.332	0.471	0.0516909	NK cells	Hspa1a
3.69E-06	0.25945573	0.751	0.686	0.05226975	NK cells	Hnrnpa2b1
3.71E-06	-0.3218238	0.051	0.137	0.05252796	NK cells	Nampt
3.82E-06	0.28104281	0.251	0.164	0.05402767	NK cells	Zap70
3.84E-06	0.36747237	0.114	0.058	0.05436477	NK cells	Aoah
3.94E-06	0.39501363	0.211	0.133	0.05575338	NK cells	Rhof
4.13E-06	-0.3990713	0.189	0.307	0.05852172	NK cells	Rbm38
4.34E-06	0.41882143	0.922	0.871	0.06141379	NK cells	Hspa8
4.39E-06	0.34843224	0.286	0.2	0.06216372	NK cells	Dgat1
4.47E-06	0.36016724	0.178	0.108	0.06321316	NK cells	Ube2g2
4.53E-06	0.4033117	0.422	0.34	0.06410292	NK cells	Ociad1
4.77E-06	-0.2824663	0.043	0.123	0.06757586	NK cells	Vrk1
4.79E-06	-0.3509906	0.081	0.177	0.06787044	NK cells	AU020206
4.82E-06	0.31346309	0.138	0.076	0.06818438	NK cells	Agtrap
4.87E-06	0.53260804	0.319	0.243	0.06900196	NK cells	Lsm8
4.95E-06	-0.3002367	0.046	0.127	0.07005189	NK cells	Pmvk
5.11E-06	0.36636162	0.449	0.362	0.07227582	NK cells	Atp1b3
5.26E-06	0.38157481	0.208	0.132	0.07447819	NK cells	Vezf1
5.41E-06	-0.5747933	0.378	0.467	0.07665257	NK cells	Txn1
5.52E-06	0.27784396	0.646	0.535	0.07821169	NK cells	Ostf1
5.66E-06	-0.4242083	0.095	0.192	0.0801162	NK cells	Trim30a
6.06E-06	0.39054881	0.378	0.29	0.08575812	NK cells	Ankrd44
6.11E-06	-0.3326057	0.046	0.126	0.08648287	NK cells	Nsf
6.13E-06	0.37498284	0.743	0.633	0.0867387	NK cells	Klf2
6.16E-06	0.33262335	0.157	0.091	0.08718875	NK cells	St3gal4
6.23E-06	-0.421151	0.032	0.105	0.08819542	NK cells	Gadd45a

6.28E-06	-0.2677068	0.035	0.111	0.08896692	NK cells	Mdfic
6.58E-06	-0.6814228	0.919	0.929	0.09308569	NK cells	Lars2
6.65E-06	0.34504198	0.33	0.239	0.09414879	NK cells	Nfatc1
6.68E-06	-0.2706466	0.035	0.111	0.09456176	NK cells	Irf2bp2
6.74E-06	0.43241291	0.419	0.344	0.09546542	NK cells	Cap1
6.88E-06	-0.2847018	0.068	0.158	0.09737099	NK cells	Larp4b
6.93E-06	-0.2951439	0.062	0.148	0.09807094	NK cells	Cd38
7.34E-06	0.33991434	0.47	0.382	0.10394125	NK cells	Arf4
7.40E-06	-0.375774	0.184	0.299	0.10470876	NK cells	Tor1aip1
7.52E-06	0.33433816	0.349	0.258	0.10651311	NK cells	Socs1
7.92E-06	-0.4864513	0.181	0.294	0.11209948	NK cells	Kdm6b
8.18E-06	0.33637126	0.116	0.061	0.11576398	NK cells	Slc9a9
8.19E-06	0.32866632	0.143	0.08	0.1158693	NK cells	Sult2b1
8.39E-06	0.27043786	0.114	0.059	0.11870286	NK cells	Prkd3
8.66E-06	-0.2874638	0.959	0.911	0.12258942	NK cells	Rps15a
8.80E-06	-0.3152148	0.043	0.12	0.12451671	NK cells	Notch2
8.95E-06	-0.3159507	0.081	0.171	0.12666748	NK cells	Fam3c
9.22E-06	0.39145413	0.446	0.36	0.13056908	NK cells	Stk24
1.04E-05	-0.62185	0.073	0.158	0.14692326	NK cells	Plk2
1.04E-05	0.37841009	0.314	0.227	0.14788572	NK cells	Gm26532
1.08E-05	-0.3062796	0.043	0.12	0.1534106	NK cells	Camk1d
1.10E-05	-0.3616269	0.065	0.147	0.15541405	NK cells	Parp1
1.11E-05	0.27379473	0.138	0.078	0.15747247	NK cells	Cdk5rap3
1.16E-05	-0.3867726	0.032	0.101	0.16391062	NK cells	Cmah
1.16E-05	-0.3010644	0.111	0.209	0.16440029	NK cells	Ube2f
1.16E-05	0.42913821	0.295	0.216	0.1648413	NK cells	Rsbn1l
1.20E-05	0.36506719	0.405	0.326	0.16975599	NK cells	Cdc37
1.20E-05	-0.3062362	0.159	0.27	0.17019956	NK cells	Ywhag
1.29E-05	0.3982088	0.468	0.391	0.18316865	NK cells	Bzw1
1.34E-05	0.33751694	0.389	0.307	0.19026278	NK cells	Rnf187
1.38E-05	-0.3090374	0.038	0.111	0.1958156	NK cells	Tgtp2
1.39E-05	0.32748772	0.476	0.403	0.19613119	NK cells	Skp1a
1.53E-05	0.46448097	0.338	0.26	0.21721033	NK cells	Vgll4
1.64E-05	-0.423854	0.035	0.103	0.23146342	NK cells	Xylt1
1.64E-05	0.26807079	0.586	0.498	0.2326149	NK cells	Abracl
1.68E-05	-0.3273937	0.143	0.245	0.2375764	NK cells	Ikbkb
1.74E-05	0.34908024	0.181	0.112	0.24563376	NK cells	Stim1
1.74E-05	0.43412118	0.392	0.319	0.24650882	NK cells	Vasp
1.79E-05	-0.4632191	0.311	0.427	0.25280513	NK cells	Ccnl1
1.80E-05	-0.456155	0.227	0.334	0.25419859	NK cells	Pnp
1.88E-05	0.28320138	0.492	0.401	0.26616545	NK cells	Capns1
1.88E-05	0.3893383	0.224	0.152	0.26634341	NK cells	Gramd1a
1.94E-05	0.28482206	0.124	0.068	0.27492514	NK cells	Sppl3
2.01E-05	-0.3177772	0.035	0.104	0.28475998	NK cells	Tmcc1
2.10E-05	-0.4495224	0.068	0.146	0.29739542	NK cells	Lair1
2.19E-05	0.30755078	0.384	0.303	0.31006673	NK cells	Lrrfip1
2.28E-05	0.4016955	0.192	0.125	0.32230545	NK cells	Gna15
2.29E-05	0.39531693	0.238	0.165	0.32366412	NK cells	B4galt1

2.41E-05	0.2540032	0.116	0.062	0.34176866	NK cells	Eml3
2.50E-05	-0.3579328	0.049	0.122	0.35433806	NK cells	Filip1l
2.55E-05	0.26748651	0.646	0.575	0.36098465	NK cells	Hnrnpa3
2.57E-05	-0.3262708	0.068	0.147	0.36385228	NK cells	Ncoa3
2.60E-05	0.43724186	0.314	0.237	0.36808122	NK cells	Nptn
2.64E-05	-0.3180327	0.57	0.639	0.37421075	NK cells	Nme2
2.64E-05	0.32988811	0.33	0.251	0.37437778	NK cells	Rab1b
2.67E-05	0.27742358	0.627	0.529	0.37736473	NK cells	Ran
2.93E-05	-0.2968503	0.257	0.376	0.41473928	NK cells	Prdx2
2.96E-05	-0.5495042	0.476	0.565	0.41853701	NK cells	Zfp36l1
2.96E-05	-0.3845294	0.173	0.274	0.41885926	NK cells	Rasgrp2
3.03E-05	0.46065528	0.23	0.158	0.42909276	NK cells	Sgk1
3.23E-05	0.35543297	0.5	0.415	0.45734134	NK cells	Spcs1
3.23E-05	-0.2924209	0.116	0.209	0.45737946	NK cells	Dnajc9
3.41E-05	0.43571087	0.462	0.398	0.48231329	NK cells	Ywhab
3.63E-05	-0.7055853	0.292	0.362	0.5132155	NK cells	Lat
3.63E-05	0.25424389	0.335	0.248	0.51455253	NK cells	Zgpat
3.65E-05	0.41397426	0.376	0.309	0.51717996	NK cells	Cript
3.78E-05	-0.4728267	0.3	0.403	0.53520929	NK cells	Atp6v1g1
4.27E-05	-0.4256014	0.095	0.179	0.60516594	NK cells	Itgav
4.38E-05	0.38647976	0.154	0.096	0.62062039	NK cells	Dtnbp1
4.59E-05	-0.4582218	0.184	0.279	0.64943474	NK cells	Ddx6
4.70E-05	-0.382064	0.143	0.239	0.66543715	NK cells	Camk2d
4.73E-05	-0.7344267	0.297	0.382	0.66927805	NK cells	Ctsc
4.76E-05	0.34028935	0.468	0.402	0.67391919	NK cells	Ndufa1
4.76E-05	-0.4785926	0.13	0.219	0.67401126	NK cells	Ninj1
4.82E-05	-0.2776326	0.149	0.248	0.68161966	NK cells	Batf
4.83E-05	0.38240358	0.149	0.091	0.68360114	NK cells	Pacsin2
4.85E-05	-0.3456515	0.089	0.172	0.68593363	NK cells	Irf9
4.98E-05	-0.3629163	0.362	0.477	0.70502596	NK cells	Gpi1
5.16E-05	0.34558843	0.168	0.106	0.73074144	NK cells	Chtf8
5.17E-05	0.30571422	0.597	0.534	0.73140191	NK cells	Cox7a2
5.18E-05	-0.3361322	0.297	0.412	0.73383161	NK cells	Txnip
5.18E-05	-0.325203	0.205	0.311	0.73392673	NK cells	Mier1
5.32E-05	-0.3916189	0.262	0.366	0.75252944	NK cells	Atpif1
5.36E-05	-0.257919	0.065	0.141	0.75928875	NK cells	Abhd12
5.73E-05	0.31473513	0.17	0.109	0.81169854	NK cells	Nck1
5.83E-05	-0.2968424	0.07	0.148	0.82512551	NK cells	Svil
5.88E-05	0.33754338	0.241	0.171	0.83224874	NK cells	Il10rb
5.90E-05	0.40635953	0.224	0.153	0.83458029	NK cells	Gpr18
6.09E-05	0.40154012	0.192	0.129	0.86181238	NK cells	Cyb5r3
6.10E-05	-0.3120138	0.154	0.25	0.86335648	NK cells	Smap2
6.36E-05	-0.305222	0.049	0.118	0.90012398	NK cells	Fes
6.54E-05	0.29079287	0.222	0.148	0.92550397	NK cells	Lamb3
6.61E-05	0.35356308	0.186	0.121	0.93608964	NK cells	Ccdc117
6.98E-05	-0.3879481	0.124	0.213	0.98759447	NK cells	Creg1
7.08E-05	-0.2530609	0.059	0.134	1	NK cells	Hip1
7.09E-05	-0.3365738	0.062	0.133	1	NK cells	Nrm

7.11E-05	-0.3224914	0.905	0.881	1	NK cells	mt-Atp6
7.19E-05	0.32016885	0.459	0.396	1	NK cells	Capza1
7.54E-05	-0.3995659	0.211	0.305	1	NK cells	Man2b1
7.64E-05	-0.3044542	0.054	0.123	1	NK cells	Pcf11
7.90E-05	0.47016242	0.189	0.126	1	NK cells	Klhl6
8.08E-05	0.38257402	0.259	0.192	1	NK cells	Lasp1
8.09E-05	0.32424814	0.468	0.404	1	NK cells	Raly
8.10E-05	-0.294439	0.17	0.27	1	NK cells	Lbh
8.11E-05	-0.6790404	0.157	0.232	1	NK cells	Fcgr3
8.20E-05	-0.2941147	0.086	0.166	1	NK cells	Skap2
8.24E-05	-0.4495234	0.238	0.34	1	NK cells	Cited2
8.24E-05	-0.4011906	0.173	0.264	1	NK cells	Snx2
8.38E-05	0.31573139	0.481	0.412	1	NK cells	Atp5k
8.60E-05	-0.3274374	0.146	0.239	1	NK cells	Rhoh
8.74E-05	0.33706402	0.492	0.419	1	NK cells	Tbca
9.07E-05	0.29375584	0.197	0.13	1	NK cells	Arap2
9.09E-05	0.39793806	0.222	0.155	1	NK cells	Dnajc1
9.57E-05	-0.2939653	0.168	0.266	1	NK cells	Tgfbr2
0.0001005	-0.3012188	0.086	0.166	1	NK cells	Entpd1
0.00010732	-0.4650177	0.322	0.416	1	NK cells	Tubb5
0.00010768	-0.5705319	0.078	0.15	1	NK cells	Dusp10
0.00011156	-0.2851256	0.073	0.146	1	NK cells	Egln2
0.00011318	0.30933285	0.227	0.162	1	NK cells	Unc119
0.00011326	0.37412865	0.343	0.279	1	NK cells	Ddost
0.00011407	-0.3724601	0.116	0.197	1	NK cells	Dgka
0.00011702	-0.3884706	0.227	0.328	1	NK cells	Gng2
0.00012274	0.39986219	0.481	0.429	1	NK cells	Tgfb1
0.00012372	-0.2930747	0.108	0.19	1	NK cells	Pten
0.00012564	0.37101193	0.368	0.298	1	NK cells	Rab8a
0.00013105	0.51478363	0.295	0.228	1	NK cells	Atp11b
0.00013502	-0.29902	0.051	0.116	1	NK cells	Pml
0.00014064	0.31532714	0.286	0.218	1	NK cells	Rnf138
0.00014301	0.42186983	0.322	0.254	1	NK cells	Itgb7
0.00014317	0.28337207	0.149	0.094	1	NK cells	Itpk1
0.00014389	-0.4427726	0.238	0.326	1	NK cells	Mrps6
0.00014502	0.42390123	0.408	0.349	1	NK cells	Bcap31
0.00015191	0.4138597	0.259	0.195	1	NK cells	Cetn2
0.00016309	0.38021184	0.273	0.206	1	NK cells	Hopx
0.00016551	0.4393699	0.362	0.299	1	NK cells	Ssr2
0.0001663	-0.9383418	0.665	0.649	1	NK cells	S100a11
0.0001705	0.35567555	0.151	0.097	1	NK cells	Rap2b
0.00017459	0.38565191	0.335	0.272	1	NK cells	Cmpk1
0.00018484	0.38334345	0.319	0.255	1	NK cells	Glrx3
0.0001921	-0.255318	0.119	0.201	1	NK cells	Dynlt1b
0.00019449	0.30195496	0.197	0.134	1	NK cells	Cab39
0.00019537	0.27986102	0.132	0.082	1	NK cells	Acadvl
0.00021144	-0.9982206	0.286	0.389	1	NK cells	Gm26917
0.00021351	-0.2501925	0.119	0.2	1	NK cells	Kdm7a

0.00021446	-0.3201876	0.07	0.139	1	NK cells	Fem1c
0.00022142	-0.2716467	0.068	0.136	1	NK cells	Map3k8
0.00022389	0.2667826	0.462	0.401	1	NK cells	Tmed9
0.00022576	0.25409968	0.357	0.289	1	NK cells	Card19
0.00022605	0.35353639	0.189	0.129	1	NK cells	Tle3
0.00022866	0.29966284	0.343	0.274	1	NK cells	Tnfrsf1b
0.00023377	0.31130466	0.381	0.319	1	NK cells	Lgals9
0.00023509	0.425389	0.349	0.289	1	NK cells	Lrp10
0.00023741	-0.3346601	0.595	0.667	1	NK cells	Klf6
0.00024104	0.32769939	0.195	0.136	1	NK cells	Mrpl9
0.00025212	-0.3127302	0.162	0.251	1	NK cells	BC005537
0.00026376	-0.257384	0.111	0.19	1	NK cells	Tmem219
0.0002727	-0.2683873	0.046	0.105	1	NK cells	Cmtr1
0.00031244	-0.2784984	0.059	0.123	1	NK cells	Bach1
0.00033357	-0.2959929	0.127	0.207	1	NK cells	Ppt1
0.00034096	0.32753106	0.459	0.405	1	NK cells	Sumo1
0.00034116	0.41784893	0.219	0.16	1	NK cells	Lsm1
0.00034746	0.41502822	0.411	0.348	1	NK cells	Crem
0.0003488	-0.2570106	0.068	0.132	1	NK cells	Neu1
0.00035672	0.35293873	0.227	0.169	1	NK cells	Polr2i
0.0003587	-0.4642569	0.122	0.198	1	NK cells	Eea1
0.00035931	0.32538751	0.154	0.101	1	NK cells	Golga4
0.00037136	-0.4360392	0.146	0.224	1	NK cells	Sorl1
0.00037231	0.35278808	0.2	0.142	1	NK cells	Chst12
0.00037387	0.35944466	0.295	0.23	1	NK cells	Psip1
0.000376	0.36100086	0.132	0.084	1	NK cells	Sfxn3
0.00037844	-0.2787676	0.219	0.313	1	NK cells	Pgk1
0.00038316	0.39619458	0.395	0.332	1	NK cells	Ncor1
0.00039758	-0.3000652	0.151	0.233	1	NK cells	Phf11b
0.00041544	-0.3134608	0.176	0.263	1	NK cells	Wsb1
0.00043323	-0.2776304	0.168	0.254	1	NK cells	Pstpip1
0.00044737	0.36151019	0.286	0.227	1	NK cells	Ube2j2
0.00044837	0.28797954	0.341	0.273	1	NK cells	Isy1
0.0004546	0.31414425	0.127	0.078	1	NK cells	Arhgef18
0.00047055	0.47871967	0.297	0.242	1	NK cells	Ppm1g
0.00048412	0.48749249	0.184	0.13	1	NK cells	Errfi1
0.00048831	0.29567301	0.116	0.07	1	NK cells	Gata3
0.00050113	-0.3170957	0.165	0.245	1	NK cells	Smc4
0.00051087	0.25360635	0.116	0.071	1	NK cells	Sec23b
0.00051095	0.29232092	0.308	0.245	1	NK cells	Lsm5
0.00051287	-0.5282889	0.486	0.536	1	NK cells	Pkm
0.00052642	-0.9801468	0.232	0.304	1	NK cells	Isg15
0.0005452	-0.2510065	0.057	0.115	1	NK cells	Rcc2
0.00055712	-0.2738583	0.089	0.159	1	NK cells	P4ha1
0.00056545	0.27886361	0.141	0.091	1	NK cells	Alad
0.00056827	0.27060388	0.708	0.62	1	NK cells	Ppib
0.0005765	-0.3355995	0.216	0.303	1	NK cells	Tgif1
0.00057728	0.28655222	0.203	0.144	1	NK cells	Ss18

0.00057903	-0.349492	0.568	0.605	1	NK cells	Prdx1
0.00058166	0.27812674	0.168	0.113	1	NK cells	Ccng2
0.00058673	-0.2654225	0.07	0.134	1	NK cells	Tmem176b
0.0006141	-0.2781144	0.051	0.107	1	NK cells	Prkd2
0.00062486	0.31112567	0.281	0.215	1	NK cells	Tob1
0.00062867	0.29005184	0.505	0.455	1	NK cells	Tmem258
0.00064775	0.30891486	0.1	0.059	1	NK cells	Wdr95
0.00065358	0.30233446	0.168	0.115	1	NK cells	Txndc11
0.00066069	0.3224928	0.127	0.081	1	NK cells	Nop9
0.00066278	0.38700799	0.446	0.402	1	NK cells	Tmed2
0.00067747	0.34777415	0.195	0.14	1	NK cells	Stt3a
0.00068098	-0.4499122	0.154	0.229	1	NK cells	Tnfrsf1a
0.00068386	0.4217529	0.27	0.211	1	NK cells	Tm9sf3
0.00069247	0.28833148	0.176	0.124	1	NK cells	Ptpn7
0.00069702	-0.2709109	0.149	0.23	1	NK cells	Stk38
0.00070728	0.31409995	0.322	0.264	1	NK cells	Idh3b
0.00073494	0.34008418	0.286	0.225	1	NK cells	Odc1
0.00073669	0.33360655	0.103	0.062	1	NK cells	Ssh1
0.00076395	0.29130765	0.297	0.234	1	NK cells	Tgoln1
0.00078299	0.31929396	0.351	0.293	1	NK cells	M6pr
0.00080299	-0.3512469	0.189	0.267	1	NK cells	Rnh1
0.00082244	0.2692477	0.473	0.419	1	NK cells	Hnrnpl
0.00082478	0.27667257	0.362	0.295	1	NK cells	Slc50a1
0.00083018	-0.3465469	0.073	0.132	1	NK cells	Hvcn1
0.00083303	-0.2911077	0.87	0.858	1	NK cells	Rpl17
0.00085401	0.31303898	0.416	0.359	1	NK cells	Polr2l
0.0008601	0.28706207	0.224	0.168	1	NK cells	Map4
0.00087279	0.26206931	0.551	0.496	1	NK cells	Eif4g2
0.00087948	-0.3486438	0.684	0.696	1	NK cells	Psmb8
0.00090461	0.41254073	0.2	0.147	1	NK cells	Acly
0.00093152	0.29315847	0.178	0.127	1	NK cells	Arhgef2
0.00096536	0.38263442	0.197	0.144	1	NK cells	Ccdc88c
0.00097024	0.3210137	0.497	0.447	1	NK cells	Hnrnpab
0.00100153	-0.38787	0.173	0.25	1	NK cells	Hilpda
0.00105127	0.41155141	0.214	0.162	1	NK cells	Ndfip2
0.00105687	0.39364183	0.708	0.647	1	NK cells	Hspa5
0.00115354	-0.2539507	0.065	0.122	1	NK cells	Rgs16
0.00115699	-0.2633294	0.162	0.241	1	NK cells	Per1
0.00118936	0.40792642	0.216	0.162	1	NK cells	Aplp2
0.00120905	0.27835303	0.276	0.214	1	NK cells	Rnf125
0.0012137	-0.3473738	0.17	0.247	1	NK cells	Zbp1
0.00122227	0.36601082	0.378	0.325	1	NK cells	Sft2d1
0.00123087	0.32021129	0.154	0.106	1	NK cells	Slc35b2
0.00126538	0.26745243	0.211	0.156	1	NK cells	Ppil2
0.00127134	0.29329886	0.368	0.312	1	NK cells	Plekhj1
0.00127802	0.34872913	0.189	0.137	1	NK cells	Efr3a
0.00128447	0.28359967	0.219	0.161	1	NK cells	Kif21b
0.00129182	-0.2878524	0.073	0.133	1	NK cells	Bag3

0.00132509	0.28885036	0.565	0.524	1	NK cells	Hnrnpf
0.00134043	0.43468194	0.408	0.369	1	NK cells	Nme1
0.00134573	0.33111688	0.284	0.228	1	NK cells	Zeb2
0.00136959	-0.3016615	0.265	0.349	1	NK cells	Zfand5
0.00137987	-0.2677998	0.189	0.271	1	NK cells	Clic4
0.00138167	-0.5159045	0.351	0.424	1	NK cells	Irf1
0.00140913	-0.4885945	0.23	0.304	1	NK cells	Nfe2l2
0.00145927	-0.25031	0.097	0.16	1	NK cells	Pias1
0.00149555	-0.2704572	0.219	0.306	1	NK cells	Lamtor1
0.00156537	0.27813501	0.162	0.112	1	NK cells	Pdlim1
0.00167485	0.26181478	0.408	0.358	1	NK cells	7-Sep
0.00170413	0.27382368	0.146	0.1	1	NK cells	Ppm1b
0.00175373	0.33699494	0.151	0.105	1	NK cells	Txnrd1
0.00176729	0.27815852	0.159	0.11	1	NK cells	Jakmip1
0.00178015	-0.2850609	0.327	0.42	1	NK cells	Rps27rt
0.00178533	0.28180005	0.403	0.351	1	NK cells	Rbm42
0.00180708	-0.3230234	0.07	0.122	1	NK cells	Eif2ak3
0.001864	0.2647946	0.362	0.304	1	NK cells	Mob4
0.00189311	-0.3064395	0.062	0.115	1	NK cells	Hist1h2bc
0.00190153	0.31725896	0.214	0.16	1	NK cells	Fam204a
0.00193523	0.30491099	0.154	0.107	1	NK cells	Esyt2
0.00195708	0.32450526	0.251	0.198	1	NK cells	Exosc3
0.00200662	0.27199098	0.3	0.244	1	NK cells	Rnaseh2c
0.00208006	0.30226595	0.203	0.154	1	NK cells	Pdcl3
0.00209893	0.36545226	0.111	0.071	1	NK cells	Snip1
0.00211436	0.25186595	0.511	0.462	1	NK cells	Sec62
0.00218092	0.29328214	0.132	0.089	1	NK cells	Fam53b
0.00218378	0.35907023	0.3	0.248	1	NK cells	Trappc4
0.00225665	0.29132828	0.381	0.333	1	NK cells	Rer1
0.00227684	-0.3466754	0.457	0.52	1	NK cells	Rhog
0.00227906	0.33846952	0.289	0.242	1	NK cells	Prmt1
0.00228195	0.31996081	0.354	0.303	1	NK cells	Nt5c
0.00248574	0.3950969	0.178	0.134	1	NK cells	Prkag1
0.00257431	0.30364147	0.146	0.102	1	NK cells	Sfxn1
0.00265023	0.26922195	0.151	0.105	1	NK cells	Trabd
0.0028234	-0.5231264	0.281	0.356	1	NK cells	Fosb
0.00303898	0.29236355	0.273	0.217	1	NK cells	Zdhhc20
0.00304076	0.28597272	0.132	0.09	1	NK cells	Fcho1
0.00310626	0.25899744	0.362	0.315	1	NK cells	Bag1
0.00328299	0.31570231	0.322	0.274	1	NK cells	Vps29
0.00334763	0.34037716	0.243	0.198	1	NK cells	Hpcal1
0.0033832	0.28128375	0.549	0.503	1	NK cells	Fam107b
0.00356612	0.27914677	0.224	0.175	1	NK cells	Midn
0.00366286	0.30881268	0.262	0.217	1	NK cells	Rpn2
0.00373175	0.32612292	0.219	0.171	1	NK cells	Syncrip
0.00388559	0.3901513	0.259	0.214	1	NK cells	Stip1
0.00393923	0.32881646	0.327	0.282	1	NK cells	Ddx21
0.00401685	-0.2668021	0.795	0.789	1	NK cells	Rpl36a

0.00406761	0.38522802	0.338	0.298	1	NK cells	Ubxn4
0.00422023	0.32222121	0.181	0.135	1	NK cells	Clcn3
0.0042282	0.26434481	0.114	0.076	1	NK cells	Tbc1d9b
0.00428518	-0.2547672	0.227	0.303	1	NK cells	Kpna4
0.00500936	0.32677175	0.216	0.168	1	NK cells	Polr2m
0.00504647	-0.2855778	0.192	0.263	1	NK cells	Mbnl2
0.00513645	-0.2505509	0.232	0.311	1	NK cells	Wnk1
0.00524228	0.25009072	0.303	0.255	1	NK cells	Polr2e
0.00541187	-0.9796817	0.23	0.286	1	NK cells	Egr1
0.00548465	0.28891383	0.108	0.072	1	NK cells	Fbxo34
0.00581987	0.30562682	0.224	0.174	1	NK cells	Sun2
0.0058528	0.25548354	0.122	0.084	1	NK cells	Qtrt1
0.00595561	0.28174402	0.278	0.234	1	NK cells	Eny2
0.00599099	0.25445968	0.108	0.072	1	NK cells	D10Wsu102e
0.00604814	0.29519364	0.273	0.227	1	NK cells	Scamp3
0.00622009	-0.5199401	0.173	0.235	1	NK cells	Nfkbiz
0.00626931	0.34669821	0.27	0.225	1	NK cells	Taf15
0.00630261	-0.3271818	0.116	0.172	1	NK cells	Gtf2i
0.00636228	0.25255899	0.17	0.128	1	NK cells	Stk16
0.00644562	0.25321617	0.178	0.135	1	NK cells	Nelfb
0.00644565	0.27628492	0.227	0.184	1	NK cells	Kdelr2
0.00646536	0.35075085	0.208	0.168	1	NK cells	Rsl1d1
0.00654657	0.2946622	0.178	0.138	1	NK cells	Josd2
0.00683954	-0.3916109	0.508	0.548	1	NK cells	Tsc22d3
0.00693732	0.33378428	0.249	0.209	1	NK cells	Cdk4
0.00741817	0.26067757	0.327	0.283	1	NK cells	Mrps33
0.00747759	-0.2746146	0.186	0.254	1	NK cells	Cltc
0.00751613	0.30430795	0.3	0.263	1	NK cells	Tmem128
0.00757758	0.25707508	0.111	0.076	1	NK cells	Nbeal2
0.00763786	0.25881349	0.465	0.423	1	NK cells	Ndufb7
0.00772404	0.26236311	0.246	0.2	1	NK cells	Sptan1
0.00777226	0.33912452	0.284	0.243	1	NK cells	Rbbp4
0.00800329	0.29109743	0.23	0.187	1	NK cells	Nans
0.00802433	-0.2649511	0.349	0.404	1	NK cells	Akr1a1
0.00823623	0.27681129	0.143	0.104	1	NK cells	Ldb1
0.00889989	0.28272408	0.338	0.288	1	NK cells	Slbp
0.00903256	-0.3804763	0.989	0.983	1	NK cells	Malat1
0.009324	-0.3738994	0.4	0.466	1	NK cells	Pim1
0.00961952	0.29978751	0.295	0.252	1	NK cells	Gpr65
0.00975669	-0.3108129	0.151	0.206	1	NK cells	Gns
0	3.93447674	0.859	0.018	0	Macrophage:	Pf4
0	2.51062241	0.515	0.009	0	Macrophage:	Cbr2
0	2.45851822	0.657	0.012	0	Macrophage:	Mgl2
0	2.0787684	0.556	0.002	0	Macrophage:	Fcrls
0	1.74158422	0.475	0.004	0	Macrophage:	Gas6
0	1.51532318	0.424	0.003	0	Macrophage:	Pmp22
0	1.15257837	0.465	0.002	0	Macrophage:	Slco2b1
2.73E-280	2.15598179	0.778	0.036	3.87E-276	Macrophage:	Dab2

3.58E-237	0.99428236	0.242	0.002	5.07E-233	Macrophage:	Cd163
2.90E-223	1.28249203	0.414	0.011	4.10E-219	Macrophage:	Igf1
5.23E-222	2.05821845	0.697	0.036	7.41E-218	Macrophage:	Stab1
2.87E-217	2.07658661	0.606	0.028	4.06E-213	Macrophage:	Cfh
1.64E-215	2.1955284	0.667	0.034	2.33E-211	Macrophage:	Mrc1
4.58E-215	2.21565142	0.707	0.038	6.49E-211	Macrophage:	Hpgd
4.09E-208	1.29725277	0.212	0.002	5.78E-204	Macrophage:	Mmp13
2.82E-203	1.05235888	0.273	0.004	3.99E-199	Macrophage:	Ccl24
4.05E-192	0.97431174	0.283	0.005	5.74E-188	Macrophage:	Fxyd2
1.51E-179	0.99311514	0.293	0.006	2.14E-175	Macrophage:	Gpr34
5.56E-179	2.10862153	0.525	0.025	7.86E-175	Macrophage:	Lpl
9.07E-161	1.63039081	0.515	0.027	1.28E-156	Macrophage:	Lilra5
1.24E-158	1.10173507	0.162	0.001	1.76E-154	Macrophage:	Lyve1
1.26E-158	1.47433025	0.444	0.02	1.78E-154	Macrophage:	Trim47
1.90E-158	1.5798309	0.273	0.006	2.69E-154	Macrophage:	Folr2
1.63E-152	2.32587001	0.818	0.082	2.31E-148	Macrophage:	Trf
1.89E-149	0.66011989	0.172	0.002	2.67E-145	Macrophage:	Col14a1
3.20E-149	0.49082023	0.172	0.002	4.53E-145	Macrophage:	Kitl
2.83E-142	2.24413439	0.707	0.064	4.01E-138	Macrophage:	C3ar1
9.84E-142	0.41352626	0.162	0.002	1.39E-137	Macrophage:	Etv1
2.01E-141	0.97612594	0.343	0.013	2.85E-137	Macrophage:	Abca9
5.16E-131	0.63861536	0.242	0.006	7.30E-127	Macrophage:	Xlr
7.41E-131	0.38672927	0.111	0.001	1.05E-126	Macrophage:	P3h2
1.46E-129	2.20574907	0.202	0.004	2.06E-125	Macrophage:	Cxcl1
1.64E-129	2.66950381	0.889	0.12	2.32E-125	Macrophage:	Tmem176b
3.06E-129	1.29557659	0.263	0.008	4.33E-125	Macrophage:	Ltc4s
6.82E-129	1.91612236	0.727	0.074	9.65E-125	Macrophage:	Adgre1
2.92E-124	2.2483448	0.525	0.038	4.13E-120	Macrophage:	F13a1
7.71E-124	0.49210682	0.131	0.001	1.09E-119	Macrophage:	Fcna
2.82E-122	1.45452396	0.556	0.041	3.99E-118	Macrophage:	Igfbp4
7.70E-120	1.21568031	0.525	0.039	1.09E-115	Macrophage:	Dhrs3
5.77E-118	1.91380668	0.747	0.086	8.16E-114	Macrophage:	Tmem176a
3.54E-117	2.93192997	0.99	0.163	5.01E-113	Macrophage:	C1qc
6.66E-115	0.88695029	0.303	0.012	9.43E-111	Macrophage:	Ptgs1
1.59E-110	2.73951825	0.949	0.168	2.26E-106	Macrophage:	Csf1r
3.00E-108	1.3923519	0.505	0.041	4.24E-104	Macrophage:	Rnase4
1.37E-106	0.57786189	0.172	0.004	1.94E-102	Macrophage:	Nes
2.70E-106	1.71344876	0.768	0.1	3.82E-102	Macrophage:	Pid1
4.83E-106	1.61690612	0.596	0.057	6.84E-102	Macrophage:	Cd63
1.07E-105	1.61793687	0.687	0.081	1.51E-101	Macrophage:	P2ry6
2.28E-105	2.82697431	0.96	0.169	3.23E-101	Macrophage:	C1qa
1.89E-103	1.21309113	0.596	0.06	2.68E-99	Macrophage:	Camk1
3.77E-101	0.70687561	0.101	0.001	5.34E-97	Macrophage:	Bmp2
4.20E-101	0.44240029	0.141	0.002	5.95E-97	Macrophage:	Ctsf
3.10E-98	2.46766036	0.717	0.097	4.39E-94	Macrophage:	Pltp
4.68E-98	2.71297015	0.879	0.148	6.62E-94	Macrophage:	Mafb
5.68E-98	2.56257198	0.96	0.196	8.04E-94	Macrophage:	Cd81
1.36E-94	2.17665789	0.798	0.119	1.93E-90	Macrophage:	C5ar1

5.00E-94	2.53462834	0.98	0.191	7.08E-90	Macrophage	C1qb
6.47E-93	0.35745514	0.131	0.002	9.16E-89	Macrophage	Reps2
2.21E-92	0.4249987	0.121	0.002	3.13E-88	Macrophage	Gm15726
1.03E-91	0.34976394	0.141	0.003	1.45E-87	Macrophage	Mxra7
3.24E-91	1.28714071	0.535	0.053	4.59E-87	Macrophage	Cd36
1.17E-90	0.8232086	0.263	0.012	1.65E-86	Macrophage	Tanc2
6.02E-90	0.70277028	0.131	0.002	8.52E-86	Macrophage	Adam33
1.23E-89	1.35098058	0.485	0.045	1.74E-85	Macrophage	Axl
2.11E-89	1.06294717	0.414	0.033	2.98E-85	Macrophage	Rab3il1
8.43E-89	1.04989002	0.414	0.033	1.19E-84	Macrophage	Anxa3
3.54E-87	1.06211212	0.414	0.033	5.02E-83	Macrophage	Zfhx3
1.03E-86	1.81869071	0.677	0.092	1.46E-82	Macrophage	Slamf9
4.34E-86	0.99738718	0.333	0.021	6.14E-82	Macrophage	Plxdc2
1.59E-85	0.84331374	0.162	0.004	2.25E-81	Macrophage	Il1a
2.23E-84	3.62358778	0.889	0.186	3.15E-80	Macrophage	Mt1
1.77E-83	2.21388238	0.697	0.104	2.51E-79	Macrophage	Tnf
7.81E-83	0.65711234	0.313	0.019	1.11E-78	Macrophage	Alox5
2.04E-82	3.54015274	0.99	0.289	2.89E-78	Macrophage	Apoe
4.19E-81	0.88174383	0.354	0.026	5.94E-77	Macrophage	Hpgds
9.31E-81	0.30983753	0.111	0.002	1.32E-76	Macrophage	Rhobtb1
1.69E-80	0.57715096	0.162	0.005	2.39E-76	Macrophage	Timd4
8.72E-80	2.87824262	0.727	0.123	1.23E-75	Macrophage	Hmox1
2.97E-75	1.58300389	0.646	0.097	4.20E-71	Macrophage	Timp2
3.90E-75	1.88944915	0.788	0.147	5.52E-71	Macrophage	App
1.14E-73	2.45577457	0.859	0.18	1.62E-69	Macrophage	Cd14
5.15E-73	0.32366534	0.101	0.002	7.28E-69	Macrophage	Colec12
8.05E-71	0.92305806	0.414	0.041	1.14E-66	Macrophage	Lacc1
1.57E-70	1.46980689	0.131	0.003	2.22E-66	Macrophage	Mmp12
1.98E-70	1.07593685	0.394	0.037	2.80E-66	Macrophage	Rgl1
4.14E-70	3.36453943	0.919	0.275	5.86E-66	Macrophage	Egr1
4.98E-70	0.68627496	0.253	0.015	7.05E-66	Macrophage	Rab11fip5
1.60E-67	1.63169411	0.879	0.198	2.26E-63	Macrophage	Lgmn
3.42E-67	1.58668165	0.667	0.114	4.84E-63	Macrophage	Cx3cr1
1.98E-65	2.54314179	0.859	0.224	2.81E-61	Macrophage	Nfkbiz
3.40E-65	0.76883315	0.333	0.029	4.81E-61	Macrophage	Gas7
9.56E-65	1.57559389	0.576	0.085	1.35E-60	Macrophage	Ms4a7
1.16E-64	0.57055075	0.263	0.018	1.65E-60	Macrophage	Arhgap22
2.49E-64	1.23437917	0.424	0.048	3.52E-60	Macrophage	Ccnd1
5.74E-64	0.77517739	0.263	0.018	8.13E-60	Macrophage	Stard8
2.50E-63	1.72066513	0.758	0.165	3.55E-59	Macrophage	Hexa
7.81E-63	0.53981485	0.222	0.013	1.11E-58	Macrophage	Serpinb8
2.67E-62	2.88304376	0.899	0.282	3.78E-58	Macrophage	Rhob
1.73E-60	1.21602472	0.434	0.054	2.44E-56	Macrophage	Itgb5
5.65E-60	1.66453632	0.899	0.219	8.00E-56	Macrophage	Fcgr3
1.50E-59	2.55246114	0.98	0.343	2.12E-55	Macrophage	Lyz2
5.92E-59	1.24602982	0.455	0.059	8.38E-55	Macrophage	Cebpa
7.12E-59	1.26699103	0.606	0.107	1.01E-54	Macrophage	Pea15a
1.18E-58	0.8785109	0.424	0.051	1.67E-54	Macrophage	Dse

6.76E-58	2.48998196	0.808	0.215	9.57E-54	Macrophage:	Marcksl1
4.10E-57	1.32480772	0.404	0.049	5.80E-53	Macrophage:	Creb5
4.89E-57	1.5954153	0.808	0.206	6.93E-53	Macrophage:	Rgs10
5.87E-57	1.47555364	0.646	0.121	8.31E-53	Macrophage:	Maf
1.36E-56	0.94052925	0.303	0.027	1.93E-52	Macrophage:	Lifr
4.81E-56	2.59073223	0.929	0.315	6.80E-52	Macrophage:	Atf3
5.60E-56	0.61244417	0.242	0.017	7.93E-52	Macrophage:	Serpinf1
6.38E-56	1.59401666	0.869	0.221	9.02E-52	Macrophage:	Cd68
8.97E-56	0.92076384	0.343	0.035	1.27E-51	Macrophage:	Mid1
1.42E-54	1.37921425	0.848	0.206	2.01E-50	Macrophage:	Grn
2.17E-54	1.52140547	0.566	0.1	3.07E-50	Macrophage:	Fcgrt
8.01E-54	0.88492526	0.384	0.045	1.13E-49	Macrophage:	Cd33
9.29E-54	0.65847677	0.263	0.022	1.31E-49	Macrophage:	Cysltr1
1.06E-53	2.03961465	0.636	0.131	1.50E-49	Macrophage:	Lmna
1.43E-53	2.56516358	0.616	0.124	2.03E-49	Macrophage:	Bag3
2.43E-53	2.52182182	0.838	0.248	3.44E-49	Macrophage:	Ier3
4.89E-53	0.70852444	0.273	0.023	6.93E-49	Macrophage:	Itsn1
8.36E-53	3.0585542	0.96	0.45	1.18E-48	Macrophage:	Jun
7.73E-52	1.11434656	0.646	0.13	1.09E-47	Macrophage:	Abhd12
8.91E-52	0.36876718	0.131	0.005	1.26E-47	Macrophage:	Rnf150
3.10E-51	0.27791262	0.101	0.003	4.39E-47	Macrophage:	Nuak1
3.92E-51	0.49665961	0.212	0.015	5.55E-47	Macrophage:	Sult1a1
6.39E-51	1.96251314	0.97	0.37	9.05E-47	Macrophage:	Ctsc
4.18E-50	0.52658108	0.182	0.011	5.92E-46	Macrophage:	Ang
5.82E-50	0.28599249	0.121	0.005	8.24E-46	Macrophage:	Zfp658
1.31E-49	0.95978266	0.444	0.066	1.85E-45	Macrophage:	Adam15
1.43E-49	1.13122631	0.657	0.134	2.02E-45	Macrophage:	Asah1
5.90E-49	0.94825611	0.596	0.115	8.36E-45	Macrophage:	Cyfip1
7.04E-49	1.75631843	0.758	0.208	9.97E-45	Macrophage:	Hexb
2.92E-48	1.10701413	0.374	0.048	4.13E-44	Macrophage:	Clec4n
9.11E-48	1.00940883	0.444	0.068	1.29E-43	Macrophage:	Rab7b
2.11E-47	1.1842477	0.667	0.143	2.99E-43	Macrophage:	Myadm
4.15E-47	1.45926154	0.838	0.252	5.87E-43	Macrophage:	Rtn4
5.29E-47	1.19251974	0.273	0.027	7.49E-43	Macrophage:	Rnd3
6.71E-47	1.10699946	0.636	0.133	9.51E-43	Macrophage:	Sirpa
1.19E-46	1.40842376	0.657	0.147	1.68E-42	Macrophage:	Ckb
1.90E-46	1.23745362	0.747	0.189	2.69E-42	Macrophage:	Lamp2
7.96E-46	0.93781582	0.808	0.195	1.13E-41	Macrophage:	Tgfbi
8.77E-46	0.45967873	0.182	0.012	1.24E-41	Macrophage:	Soga1
1.04E-45	0.78744145	0.273	0.027	1.48E-41	Macrophage:	Rin2
1.32E-45	1.60075018	0.899	0.319	1.87E-41	Macrophage:	Lamp1
6.17E-45	0.8934681	0.394	0.057	8.74E-41	Macrophage:	Ptpro
9.94E-45	2.78290561	0.677	0.176	1.41E-40	Macrophage:	Cxcl2
2.18E-44	0.45831129	0.232	0.02	3.09E-40	Macrophage:	Sbf2
5.01E-44	1.57704229	0.525	0.106	7.09E-40	Macrophage:	Tubb6
7.40E-44	1.09065669	0.737	0.181	1.05E-39	Macrophage:	Csf2ra
7.79E-44	0.65367613	0.333	0.042	1.10E-39	Macrophage:	Cmklr1
9.20E-44	1.1882848	0.596	0.122	1.30E-39	Macrophage:	Cxcl16

9.95E-44	0.54584958	0.202	0.016	1.41E-39	Macrophage:	Lima1
1.04E-43	2.37032295	0.798	0.253	1.47E-39	Macrophage:	Cdkn1a
1.50E-43	0.57179017	0.212	0.017	2.13E-39	Macrophage:	B4galt6
2.00E-43	0.78926328	0.364	0.05	2.84E-39	Macrophage:	Eps8
6.56E-43	1.59729946	0.99	0.565	9.29E-39	Macrophage:	Cst3
1.46E-42	1.04395418	0.444	0.072	2.07E-38	Macrophage:	St3gal6
2.47E-42	2.31236268	0.97	0.612	3.49E-38	Macrophage:	Zfp36
3.62E-42	2.9547436	0.919	0.458	5.12E-38	Macrophage:	Hspa1a
6.17E-42	1.15918092	0.333	0.044	8.73E-38	Macrophage:	Egr2
6.94E-42	0.84220067	0.414	0.066	9.82E-38	Macrophage:	Idh2
7.53E-42	1.53907063	1	0.933	1.07E-37	Macrophage:	Ftl1
8.32E-42	1.14326206	0.505	0.099	1.18E-37	Macrophage:	Nfic
4.19E-41	2.45007227	0.99	0.644	5.94E-37	Macrophage:	Fos
4.39E-41	2.21253766	0.768	0.248	6.22E-37	Macrophage:	Glul
5.48E-41	1.41811401	0.636	0.15	7.76E-37	Macrophage:	Ctsl
6.92E-41	0.68846471	0.313	0.04	9.79E-37	Macrophage:	Pla2g15
7.09E-41	0.43180914	0.182	0.014	1.00E-36	Macrophage:	Kcnk13
8.53E-41	0.40925041	0.141	0.008	1.21E-36	Macrophage:	Psd3
2.74E-40	1.73338424	0.939	0.433	3.88E-36	Macrophage:	Ctsb
2.76E-40	1.08622759	0.646	0.155	3.91E-36	Macrophage:	Marcks
5.34E-40	1.95460319	0.667	0.184	7.55E-36	Macrophage:	Icam1
6.95E-40	0.31452177	0.111	0.005	9.84E-36	Macrophage:	Spaca6
1.53E-39	0.64057296	0.192	0.016	2.16E-35	Macrophage:	Olfml3
2.30E-39	1.08231282	0.737	0.193	3.26E-35	Macrophage:	Fcgr2b
2.45E-39	0.31723735	0.111	0.005	3.46E-35	Macrophage:	B3galnt1
3.30E-39	0.29159148	0.131	0.007	4.67E-35	Macrophage:	Cracr2b
3.33E-39	1.17317975	0.434	0.075	4.71E-35	Macrophage:	Gatm
3.75E-39	0.84905726	0.444	0.079	5.30E-35	Macrophage:	Hacd4
7.95E-39	0.99533473	0.465	0.088	1.13E-34	Macrophage:	Clec4a2
8.31E-39	1.44699534	0.909	0.341	1.18E-34	Macrophage:	Unc93b1
8.41E-39	0.59150849	0.293	0.037	1.19E-34	Macrophage:	Sash1
1.09E-38	1.02043259	0.667	0.165	1.55E-34	Macrophage:	Lrrc25
1.54E-38	2.0952715	0.828	0.346	2.17E-34	Macrophage:	Fosb
1.74E-38	0.34942394	0.111	0.005	2.46E-34	Macrophage:	Trpv4
2.06E-38	0.25713862	0.111	0.005	2.91E-34	Macrophage:	Scamp5
3.92E-38	1.07526287	0.424	0.075	5.54E-34	Macrophage:	Trem2
4.23E-38	0.88294514	0.414	0.074	5.99E-34	Macrophage:	Cmtm3
5.03E-38	0.98434923	0.495	0.099	7.11E-34	Macrophage:	Tlr2
7.76E-38	0.69271745	0.232	0.024	1.10E-33	Macrophage:	Pros1
1.91E-37	2.00674689	0.818	0.297	2.71E-33	Macrophage:	Klf4
3.47E-37	1.26204008	0.687	0.2	4.92E-33	Macrophage:	Ehd4
4.33E-37	0.90581287	0.475	0.094	6.13E-33	Macrophage:	Lpcat2
5.04E-37	1.8144448	1	0.741	7.13E-33	Macrophage:	Ubc
5.06E-37	0.77512112	0.556	0.118	7.16E-33	Macrophage:	Slc11a1
8.97E-37	1.40377433	0.505	0.108	1.27E-32	Macrophage:	Ccl9
9.31E-37	0.53296146	0.162	0.012	1.32E-32	Macrophage:	Rac3
1.03E-36	0.70464094	0.364	0.058	1.46E-32	Macrophage:	Tbxas1
1.06E-36	2.70602962	0.485	0.102	1.50E-32	Macrophage:	Ccl2

1.15E-36	0.29832528	0.172	0.014	1.63E-32	Macrophage:	Scamp1
1.20E-36	0.78474782	0.303	0.041	1.71E-32	Macrophage:	Mob3c
1.99E-36	0.9583572	0.515	0.111	2.82E-32	Macrophage:	Tpp1
2.55E-36	1.40358592	0.636	0.161	3.61E-32	Macrophage:	Adrb2
2.56E-36	1.28690702	1	0.526	3.62E-32	Macrophage:	Psap
2.71E-36	1.96484028	0.939	0.62	3.83E-32	Macrophage:	Dusp1
2.74E-36	0.9043255	0.667	0.168	3.88E-32	Macrophage:	Mpeg1
5.63E-36	-2.4005123	0.808	0.928	7.97E-32	Macrophage:	Tmsb10
1.17E-35	0.5091014	0.232	0.025	1.66E-31	Macrophage:	Ctbp2
1.87E-35	0.75769386	0.364	0.059	2.65E-31	Macrophage:	Tmem37
3.55E-35	0.61928183	0.293	0.04	5.03E-31	Macrophage:	Cryl1
4.01E-35	1.18307871	0.808	0.269	5.68E-31	Macrophage:	Lilrb4a
4.84E-35	1.9013151	0.879	0.456	6.85E-31	Macrophage:	Ier5
4.86E-35	1.31575602	0.869	0.345	6.89E-31	Macrophage:	Ctsa
5.01E-35	0.9715612	0.606	0.157	7.09E-31	Macrophage:	Gusb
5.36E-35	0.28118558	0.141	0.01	7.58E-31	Macrophage:	Cav2
7.35E-35	0.35090491	0.162	0.013	1.04E-30	Macrophage:	Fblim1
7.40E-35	0.94601897	0.253	0.03	1.05E-30	Macrophage:	Cyp27a1
1.08E-34	0.43547062	0.182	0.016	1.53E-30	Macrophage:	Gas2l1
1.10E-34	0.36933837	0.141	0.01	1.56E-30	Macrophage:	Gpr84
1.86E-34	1.00930328	0.747	0.224	2.64E-30	Macrophage:	Zeb2
5.66E-34	0.80176	0.434	0.085	8.01E-30	Macrophage:	Cd302
6.97E-34	0.94202188	0.596	0.153	9.87E-30	Macrophage:	Cndp2
1.16E-33	0.98850907	0.657	0.184	1.64E-29	Macrophage:	Sh3bgrl
2.15E-33	0.88255649	0.525	0.121	3.04E-29	Macrophage:	Prcp
3.11E-33	1.87773049	0.737	0.281	4.40E-29	Macrophage:	Socs3
3.51E-33	1.32902498	0.111	0.006	4.97E-29	Macrophage:	Lyz1
4.57E-33	0.6646694	0.414	0.079	6.46E-29	Macrophage:	Hfe
6.16E-33	0.68860405	0.303	0.045	8.72E-29	Macrophage:	Cttnbp2nl
8.69E-33	0.50359584	0.222	0.025	1.23E-28	Macrophage:	Dapk1
8.86E-33	0.84126224	0.424	0.086	1.25E-28	Macrophage:	Ntpcr
1.82E-32	1.47222151	0.293	0.044	2.57E-28	Macrophage:	Tnfsf9
1.83E-32	0.85482565	0.364	0.065	2.59E-28	Macrophage:	Daglb
2.28E-32	1.0905147	0.384	0.071	3.23E-28	Macrophage:	Maff
2.84E-32	0.61034037	0.333	0.054	4.01E-28	Macrophage:	Gnaq
3.99E-32	0.6493835	0.263	0.036	5.65E-28	Macrophage:	Clec5a
4.29E-32	1.86983658	0.303	0.047	6.08E-28	Macrophage:	Ccl12
4.71E-32	0.5431065	0.232	0.028	6.67E-28	Macrophage:	Spred1
7.31E-32	0.41023591	0.202	0.021	1.04E-27	Macrophage:	Mfhas1
2.90E-31	0.8853571	0.606	0.162	4.10E-27	Macrophage:	Rassf4
2.91E-31	1.36605609	0.778	0.288	4.12E-27	Macrophage:	Anxa5
3.64E-31	0.76881298	0.404	0.08	5.15E-27	Macrophage:	Rhoc
4.00E-31	0.78127609	0.424	0.088	5.67E-27	Macrophage:	Pepd
5.36E-31	0.97611164	0.818	0.272	7.58E-27	Macrophage:	Cybb
5.76E-31	0.74127138	0.465	0.102	8.15E-27	Macrophage:	Sdc3
6.48E-31	0.757627	0.374	0.069	9.18E-27	Macrophage:	Dpep2
7.78E-31	0.53513606	0.303	0.047	1.10E-26	Macrophage:	Il13ra1
7.85E-31	0.69665921	0.939	0.299	1.11E-26	Macrophage:	Alox5ap

8.57E-31	0.79033154	0.556	0.142	1.21E-26	Macrophage:	Mfsd1
9.94E-31	0.7547353	0.566	0.14	1.41E-26	Macrophage:	Clec4a3
1.32E-30	0.63121608	0.636	0.16	1.86E-26	Macrophage:	Aif1
1.69E-30	0.46318636	0.192	0.02	2.39E-26	Macrophage:	Fads1
1.97E-30	0.82776405	0.293	0.046	2.79E-26	Macrophage:	Wwp1
4.27E-30	0.65630822	0.424	0.087	6.04E-26	Macrophage:	Lmo2
5.25E-30	0.60802685	0.364	0.068	7.43E-26	Macrophage:	Tfe3
5.90E-30	0.97275864	0.737	0.229	8.35E-26	Macrophage:	Pld4
7.86E-30	0.70882324	0.374	0.072	1.11E-25	Macrophage:	Ralb
9.62E-30	0.78605264	0.455	0.101	1.36E-25	Macrophage:	Lrp1
9.65E-30	0.49176452	0.242	0.032	1.37E-25	Macrophage:	Dpp7
1.24E-29	0.65519502	0.192	0.021	1.76E-25	Macrophage:	8-Mar
1.31E-29	0.54532184	0.364	0.067	1.85E-25	Macrophage:	Rab31
1.33E-29	0.40735775	0.202	0.023	1.89E-25	Macrophage:	Jup
1.59E-29	0.98423849	0.485	0.116	2.25E-25	Macrophage:	Clec4a1
1.64E-29	1.01997077	0.717	0.242	2.32E-25	Macrophage:	Arrb2
2.03E-29	0.49996185	0.222	0.028	2.87E-25	Macrophage:	Tnfrsf11a
2.27E-29	0.73485716	0.354	0.066	3.21E-25	Macrophage:	Plxnb2
3.55E-29	0.63591706	0.131	0.01	5.02E-25	Macrophage:	Arc
3.67E-29	0.57946664	0.172	0.017	5.19E-25	Macrophage:	Cdr2
4.21E-29	0.46162687	0.333	0.058	5.96E-25	Macrophage:	Ptafr
5.34E-29	0.94005884	0.677	0.213	7.55E-25	Macrophage:	Runx1
1.46E-28	1.63278837	0.929	0.522	2.07E-24	Macrophage:	Ppp1r15a
1.72E-28	1.32953116	0.97	0.875	2.43E-24	Macrophage:	Junb
2.73E-28	0.83515797	0.444	0.104	3.86E-24	Macrophage:	Vkorc1
3.85E-28	1.38762978	0.929	0.492	5.44E-24	Macrophage:	Serinc3
8.00E-28	0.98683058	0.525	0.134	1.13E-23	Macrophage:	Sdc4
1.27E-27	0.53881305	0.152	0.014	1.79E-23	Macrophage:	Etv5
1.43E-27	0.66507909	0.323	0.058	2.03E-23	Macrophage:	Dusp6
1.57E-27	0.90471894	0.576	0.168	2.23E-23	Macrophage:	Tm6sf1
1.69E-27	0.75099908	0.364	0.073	2.39E-23	Macrophage:	Myo5a
2.18E-27	0.47127397	0.192	0.023	3.09E-23	Macrophage:	Selenbp1
2.87E-27	1.02028986	0.808	0.3	4.07E-23	Macrophage:	Ly86
3.25E-27	0.60729258	0.162	0.016	4.60E-23	Macrophage:	Plau
4.38E-27	1.00076294	0.687	0.233	6.20E-23	Macrophage:	Lilr4b
4.77E-27	1.19889849	0.98	0.821	6.75E-23	Macrophage:	Itm2b
4.78E-27	0.64130971	0.242	0.035	6.77E-23	Macrophage:	Cd93
4.91E-27	1.11645248	0.111	0.008	6.95E-23	Macrophage:	Gdf15
8.54E-27	1.69306742	0.929	0.659	1.21E-22	Macrophage:	Klf6
1.00E-26	1.56573418	0.333	0.064	1.42E-22	Macrophage:	Ecm1
1.87E-26	1.23775729	0.687	0.253	2.65E-22	Macrophage:	Wsb1
2.10E-26	0.69749331	0.313	0.059	2.97E-22	Macrophage:	Pdlim4
2.35E-26	1.86272663	0.859	0.49	3.32E-22	Macrophage:	Hspa1b
3.07E-26	0.32312847	0.141	0.013	4.34E-22	Macrophage:	Pald1
3.30E-26	0.62958072	0.364	0.076	4.68E-22	Macrophage:	Mknk1
3.69E-26	1.00697598	0.404	0.091	5.23E-22	Macrophage:	Gm6377
3.92E-26	0.7178123	0.374	0.079	5.55E-22	Macrophage:	Lyl1
4.59E-26	0.50249937	0.222	0.031	6.49E-22	Macrophage:	Clec4b1

5.22E-26	0.35992526	0.192	0.023	7.39E-22	Macrophage:	Ext1
6.22E-26	0.55531194	0.242	0.037	8.81E-22	Macrophage:	Gna12
6.94E-26	0.51505187	0.263	0.043	9.83E-22	Macrophage:	Ptov1
8.69E-26	1.27415279	0.939	0.594	1.23E-21	Macrophage:	Nfkbia
9.01E-26	0.5835559	0.313	0.059	1.28E-21	Macrophage:	Zeb2os
9.83E-26	0.65578058	0.444	0.106	1.39E-21	Macrophage:	Nlrp3
1.04E-25	0.95310544	0.596	0.19	1.47E-21	Macrophage:	Mef2a
1.05E-25	1.24581992	0.798	0.347	1.48E-21	Macrophage:	Sdcbp
1.60E-25	0.3605282	0.222	0.031	2.26E-21	Macrophage:	Mfge8
2.24E-25	0.79676424	0.303	0.057	3.17E-21	Macrophage:	Smagp
2.36E-25	0.64791286	0.556	0.158	3.34E-21	Macrophage:	Gsn
2.43E-25	1.31782365	0.596	0.194	3.43E-21	Macrophage:	Kctd12
2.67E-25	-1.296166	0.97	0.927	3.78E-21	Macrophage:	Rpsa
2.84E-25	0.40284445	0.222	0.032	4.02E-21	Macrophage:	Mtus1
3.23E-25	0.87364691	0.545	0.159	4.57E-21	Macrophage:	Blvrb
4.57E-25	1.10310819	0.626	0.201	6.47E-21	Macrophage:	Plin2
5.17E-25	0.91363972	0.586	0.184	7.32E-21	Macrophage:	Tubb2a
5.54E-25	0.5433623	0.182	0.022	7.84E-21	Macrophage:	Man1c1
6.95E-25	0.61872098	0.444	0.11	9.84E-21	Macrophage:	Fes
7.02E-25	0.7833265	0.616	0.193	9.93E-21	Macrophage:	Itm2c
9.09E-25	0.54245414	0.303	0.057	1.29E-20	Macrophage:	Aoah
9.53E-25	0.6712474	0.263	0.044	1.35E-20	Macrophage:	Vat1
1.07E-24	0.45213167	0.768	0.254	1.52E-20	Macrophage:	Ccl6
1.26E-24	0.53858006	0.263	0.045	1.79E-20	Macrophage:	Itpripl2
1.38E-24	1.22895937	0.707	0.261	1.96E-20	Macrophage:	Clic4
1.41E-24	0.74328626	0.333	0.067	1.99E-20	Macrophage:	Atp6v0a1
1.72E-24	0.68679261	0.323	0.066	2.43E-20	Macrophage:	Dnase1l1
1.86E-24	0.2597809	0.131	0.012	2.63E-20	Macrophage:	Snta1
2.13E-24	1.06122543	0.848	0.394	3.01E-20	Macrophage:	Neat1
2.74E-24	0.42450861	0.212	0.03	3.89E-20	Macrophage:	Wdfy3
3.09E-24	0.90320901	0.687	0.245	4.38E-20	Macrophage:	Cltc
4.00E-24	0.58424332	0.444	0.113	5.66E-20	Macrophage:	Rnpep
6.08E-24	0.40247571	0.212	0.03	8.61E-20	Macrophage:	Arsa
6.93E-24	1.40647657	0.758	0.299	9.81E-20	Macrophage:	Cd83
7.12E-24	0.2897316	0.111	0.009	1.01E-19	Macrophage:	2510009E07F
7.30E-24	0.98515643	0.798	0.307	1.03E-19	Macrophage:	Ctsh
8.18E-24	0.94083471	0.424	0.109	1.16E-19	Macrophage:	Basp1
1.19E-23	0.626287	0.394	0.096	1.68E-19	Macrophage:	Tmem106a
1.75E-23	0.59834356	0.354	0.078	2.48E-19	Macrophage:	Rasa4
1.87E-23	0.91152888	0.515	0.154	2.65E-19	Macrophage:	Metrnl
2.05E-23	0.60021245	0.323	0.066	2.90E-19	Macrophage:	Sgpp1
2.26E-23	1.69971813	0.404	0.103	3.21E-19	Macrophage:	Cfp
2.40E-23	0.28754421	0.101	0.008	3.39E-19	Macrophage:	Clmp
3.38E-23	0.61643827	0.586	0.172	4.79E-19	Macrophage:	Pla2g7
3.40E-23	0.87515302	0.444	0.121	4.81E-19	Macrophage:	Lipa
3.72E-23	0.85643123	0.535	0.165	5.27E-19	Macrophage:	Nrp1
3.99E-23	0.61062537	0.414	0.103	5.65E-19	Macrophage:	Acer3
4.09E-23	0.26537831	0.131	0.013	5.78E-19	Macrophage:	Pld1

8.41E-23	0.71047106	0.556	0.165	1.19E-18	Macrophage:	Irf5
8.83E-23	0.58534889	0.465	0.122	1.25E-18	Macrophage:	Tcf4
1.57E-22	0.63119569	0.475	0.131	2.22E-18	Macrophage:	Apobec1
1.60E-22	0.98032137	0.364	0.085	2.27E-18	Macrophage:	Ephx1
1.85E-22	0.50879448	0.192	0.027	2.62E-18	Macrophage:	Slc7a8
2.51E-22	0.2655402	0.141	0.015	3.55E-18	Macrophage:	Pld2
3.75E-22	0.99170298	0.798	0.372	5.31E-18	Macrophage:	Gadd45b
3.75E-22	1.11098953	0.768	0.323	5.31E-18	Macrophage:	Pnp
3.81E-22	0.63533424	0.545	0.169	5.40E-18	Macrophage:	Il10rb
3.99E-22	0.67556172	0.535	0.166	5.65E-18	Macrophage:	Evi2a
4.43E-22	-6.053879	0.04	0.562	6.27E-18	Macrophage:	Ccl5
4.77E-22	0.77925792	0.475	0.137	6.76E-18	Macrophage:	Rnf13
5.53E-22	0.27036222	0.859	0.304	7.82E-18	Macrophage:	Ifitm2
6.77E-22	0.50307831	0.242	0.043	9.58E-18	Macrophage:	Arl11
7.13E-22	0.36901663	0.192	0.028	1.01E-17	Macrophage:	P2rx7
8.04E-22	0.49074634	0.293	0.059	1.14E-17	Macrophage:	Igsf8
8.41E-22	0.83553882	0.444	0.123	1.19E-17	Macrophage:	Ppp1r10
8.57E-22	-1.0112387	0.99	0.92	1.21E-17	Macrophage:	Rpl19
9.71E-22	0.38209898	0.152	0.018	1.38E-17	Macrophage:	Sord
1.00E-21	0.43061921	0.242	0.043	1.42E-17	Macrophage:	Vwa5a
1.06E-21	0.33307614	0.172	0.022	1.50E-17	Macrophage:	Tle1
1.20E-21	0.62401987	0.465	0.132	1.70E-17	Macrophage:	Dstn
1.22E-21	2.11526806	0.525	0.179	1.73E-17	Macrophage:	Phlda1
1.27E-21	0.28293397	0.121	0.012	1.80E-17	Macrophage:	Agap1
1.33E-21	0.61358334	0.333	0.074	1.89E-17	Macrophage:	Klf7
1.41E-21	0.89663442	0.485	0.143	2.00E-17	Macrophage:	Mdm2
1.66E-21	0.60800062	0.515	0.158	2.35E-17	Macrophage:	Skap2
1.97E-21	1.06314044	0.232	0.039	2.79E-17	Macrophage:	Ccl7
2.24E-21	0.88221546	0.475	0.141	3.17E-17	Macrophage:	Clec12a
2.27E-21	0.58042592	0.394	0.097	3.21E-17	Macrophage:	Rap1gds1
2.70E-21	0.4495287	0.313	0.067	3.83E-17	Macrophage:	Arhgap18
2.71E-21	0.52936083	0.434	0.115	3.83E-17	Macrophage:	Vps26a
2.85E-21	1.41039547	0.939	0.635	4.04E-17	Macrophage:	Klf2
3.36E-21	0.45276803	0.273	0.054	4.75E-17	Macrophage:	Myof
3.58E-21	0.89107839	0.798	0.334	5.06E-17	Macrophage:	mt-Nd3
3.81E-21	0.6664335	0.192	0.029	5.40E-17	Macrophage:	Mt2
4.50E-21	0.75356797	0.404	0.109	6.38E-17	Macrophage:	Fam129a
4.83E-21	0.27098783	0.111	0.01	6.83E-17	Macrophage:	Itga9
6.40E-21	-1.754539	0.606	0.801	9.06E-17	Macrophage:	Rac2
6.75E-21	0.30674163	0.121	0.012	9.55E-17	Macrophage:	P2ry12
7.60E-21	0.7212066	0.364	0.092	1.08E-16	Macrophage:	Prdx4
8.91E-21	0.52325019	0.232	0.041	1.26E-16	Macrophage:	Glb1
1.03E-20	0.64848016	0.313	0.07	1.45E-16	Macrophage:	Acvrl1
1.09E-20	0.8576258	0.566	0.196	1.55E-16	Macrophage:	Atp6ap2
1.24E-20	0.40241658	0.212	0.035	1.76E-16	Macrophage:	Plscr3
1.37E-20	0.99166218	0.667	0.239	1.94E-16	Macrophage:	H2-DMb1
1.61E-20	0.86057782	0.828	0.354	2.28E-16	Macrophage:	Ctsz
2.11E-20	0.53788124	0.222	0.038	2.99E-16	Macrophage:	Sowahc

2.22E-20	-1.0051096	0.97	0.92	3.15E-16	Macrophage:	Rpl18
2.47E-20	0.59876938	0.293	0.063	3.49E-16	Macrophage:	Fam234a
2.58E-20	0.5678861	0.828	0.321	3.65E-16	Macrophage:	Spi1
3.35E-20	-1.2649429	0.99	0.912	4.74E-16	Macrophage:	Rps15a
3.74E-20	-1.6261688	0.636	0.799	5.30E-16	Macrophage:	Sub1
4.37E-20	0.95116147	0.99	0.68	6.18E-16	Macrophage:	Gpx1
5.13E-20	0.40884917	0.222	0.038	7.26E-16	Macrophage:	Plekhg3
7.33E-20	-0.9556176	1	0.943	1.04E-15	Macrophage:	Uba52
8.93E-20	0.5025726	0.323	0.075	1.26E-15	Macrophage:	Ppp1r21
9.23E-20	0.50745525	0.242	0.046	1.31E-15	Macrophage:	Idh1
9.88E-20	-1.2848585	0.889	0.917	1.40E-15	Macrophage:	Cd52
1.15E-19	0.49667185	0.465	0.134	1.63E-15	Macrophage:	Cd300a
1.17E-19	0.69431235	0.414	0.114	1.66E-15	Macrophage:	Fcgr1
1.37E-19	0.87432558	0.677	0.279	1.93E-15	Macrophage:	Ddx3x
1.50E-19	0.28273053	0.131	0.015	2.12E-15	Macrophage:	Ctnnd1
2.56E-19	0.67320349	0.394	0.109	3.63E-15	Macrophage:	Dok2
2.76E-19	0.7205388	0.636	0.235	3.91E-15	Macrophage:	Nrros
2.95E-19	-1.0134874	0.939	0.901	4.18E-15	Macrophage:	Rpl8
3.04E-19	0.71091642	0.434	0.13	4.31E-15	Macrophage:	Ptpra
3.35E-19	0.37962219	0.232	0.044	4.74E-15	Macrophage:	Shtn1
3.92E-19	0.42865396	0.162	0.023	5.54E-15	Macrophage:	Parvb
3.93E-19	0.54538681	0.515	0.162	5.56E-15	Macrophage:	Rnf130
4.02E-19	-3.8917426	0.141	0.573	5.68E-15	Macrophage:	AW112010
4.36E-19	0.48691582	0.242	0.048	6.17E-15	Macrophage:	Pak1
4.56E-19	0.28844852	0.111	0.011	6.46E-15	Macrophage:	Zdhhc14
4.83E-19	0.77746576	0.909	0.567	6.83E-15	Macrophage:	Cebpb
5.58E-19	0.29932364	0.192	0.031	7.90E-15	Macrophage:	Mertk
5.58E-19	-0.9441723	1	0.936	7.91E-15	Macrophage:	Rpl18a
5.98E-19	-2.3096493	0.071	0.534	8.46E-15	Macrophage:	Ptprcap
6.42E-19	0.88136007	0.404	0.118	9.09E-15	Macrophage:	Casp4
7.31E-19	0.6631987	0.525	0.178	1.03E-14	Macrophage:	Nckap1l
8.78E-19	0.51382125	0.354	0.091	1.24E-14	Macrophage:	Anxa4
9.74E-19	0.66135221	0.455	0.138	1.38E-14	Macrophage:	Lair1
1.17E-18	0.26424288	0.121	0.013	1.65E-14	Macrophage:	Ston2
1.19E-18	0.93541562	0.667	0.295	1.68E-14	Macrophage:	Man2b1
1.20E-18	0.56349064	0.384	0.104	1.70E-14	Macrophage:	Fgd2
1.26E-18	0.48663583	0.677	0.254	1.78E-14	Macrophage:	Tpd52
1.55E-18	0.50780276	0.222	0.042	2.19E-14	Macrophage:	Msr1
1.78E-18	0.49776721	0.616	0.22	2.52E-14	Macrophage:	Tnfrsf1a
1.90E-18	0.5174557	0.404	0.112	2.69E-14	Macrophage:	D1Ertd622e
2.30E-18	0.92816259	0.838	0.463	3.25E-14	Macrophage:	Mcl1
2.61E-18	0.75955541	0.354	0.095	3.69E-14	Macrophage:	Rab20
2.63E-18	0.28713058	0.101	0.01	3.73E-14	Macrophage:	Slc13a3
2.75E-18	1.11249938	0.475	0.159	3.90E-14	Macrophage:	Chka
3.39E-18	-0.9148487	0.949	0.917	4.80E-14	Macrophage:	Rpl9-ps6
3.97E-18	0.47568422	0.232	0.045	5.62E-14	Macrophage:	Siglece
4.30E-18	0.36581481	0.152	0.021	6.09E-14	Macrophage:	Slc35f5
4.41E-18	-1.2378862	0.828	0.834	6.25E-14	Macrophage:	Arhgdib

5.59E-18	0.41491323	0.99	0.433	7.91E-14	Macrophage:	Tyrobp
5.67E-18	-1.0724007	0.96	0.903	8.03E-14	Macrophage:	Rps7
5.88E-18	0.67257741	0.606	0.231	8.33E-14	Macrophage:	Vsir
6.04E-18	0.3586512	0.162	0.024	8.56E-14	Macrophage:	Inppl1
7.11E-18	0.47039817	0.273	0.06	1.01E-13	Macrophage:	Slc9a9
1.06E-17	0.91519398	1	0.845	1.50E-13	Macrophage:	mt-Nd4l
1.14E-17	0.65682853	0.929	0.55	1.61E-13	Macrophage:	Ctss
1.29E-17	0.45285768	0.192	0.034	1.83E-13	Macrophage:	Eng
1.69E-17	0.55063422	0.394	0.114	2.40E-13	Macrophage:	Eps15
1.70E-17	-1.0511494	0.98	0.929	2.40E-13	Macrophage:	Rplp0
1.86E-17	0.55034695	0.323	0.084	2.63E-13	Macrophage:	Ebi3
1.95E-17	-0.9533754	0.98	0.92	2.75E-13	Macrophage:	Rps2
1.99E-17	0.33312583	0.111	0.012	2.82E-13	Macrophage:	Fnbp1l
2.37E-17	0.72005769	0.525	0.187	3.36E-13	Macrophage:	Cd86
2.45E-17	0.73785496	0.788	0.355	3.47E-13	Macrophage:	Atpif1
2.50E-17	0.49193244	0.313	0.078	3.54E-13	Macrophage:	Tcn2
2.68E-17	0.48944752	0.202	0.037	3.79E-13	Macrophage:	Dst
2.85E-17	0.48003268	0.626	0.22	4.03E-13	Macrophage:	Ms4a6c
2.90E-17	0.46304838	0.222	0.045	4.10E-13	Macrophage:	Ubtd1
3.22E-17	-0.9137253	1	0.928	4.56E-13	Macrophage:	Rps3a1
3.29E-17	-0.8490234	1	0.94	4.65E-13	Macrophage:	Rps8
3.45E-17	0.51806188	0.343	0.092	4.89E-13	Macrophage:	Ski
3.58E-17	0.37774129	0.172	0.028	5.07E-13	Macrophage:	Tdrd7
4.12E-17	-1.1054697	0.949	0.883	5.83E-13	Macrophage:	Rpl13a
4.17E-17	0.47669719	0.232	0.047	5.90E-13	Macrophage:	Acox1
4.91E-17	0.73908567	1	0.957	6.95E-13	Macrophage:	Fth1
4.92E-17	-4.1804895	0.03	0.469	6.97E-13	Macrophage:	Nkg7
5.38E-17	0.63457951	0.556	0.196	7.61E-13	Macrophage:	Aldh2
5.51E-17	0.45871677	0.404	0.118	7.79E-13	Macrophage:	Ptprj
5.51E-17	0.58951414	0.283	0.067	7.80E-13	Macrophage:	Zfp703
6.13E-17	0.40235415	0.273	0.064	8.68E-13	Macrophage:	Rbm47
6.33E-17	0.44959922	0.222	0.045	8.96E-13	Macrophage:	Zfp385a
6.60E-17	0.70738812	0.323	0.085	9.35E-13	Macrophage:	Arf2
6.99E-17	0.69530983	0.626	0.238	9.90E-13	Macrophage:	Wfdc17
7.48E-17	0.52703638	0.384	0.114	1.06E-12	Macrophage:	Ehbp1l1
7.72E-17	0.45958383	0.172	0.028	1.09E-12	Macrophage:	Sulf2
9.09E-17	-0.804873	1	0.937	1.29E-12	Macrophage:	Rpl13
1.00E-16	0.45179686	0.495	0.164	1.42E-12	Macrophage:	Cmtm6
1.14E-16	-0.8804355	0.98	0.905	1.61E-12	Macrophage:	Rps3
1.16E-16	-0.837228	1	0.941	1.64E-12	Macrophage:	Rps16
1.30E-16	0.54329821	0.99	0.417	1.85E-12	Macrophage:	Fcer1g
1.34E-16	0.99444565	0.697	0.34	1.89E-12	Macrophage:	Zfand5
1.46E-16	0.45651536	0.343	0.095	2.06E-12	Macrophage:	Tspan31
1.48E-16	0.5967739	0.394	0.116	2.09E-12	Macrophage:	Ms4a4a
1.48E-16	0.54654963	0.434	0.141	2.10E-12	Macrophage:	Rsu1
1.53E-16	0.96768788	0.909	0.708	2.17E-12	Macrophage:	Btg2
1.59E-16	0.49518612	0.354	0.097	2.26E-12	Macrophage:	Tmcc1
1.91E-16	1.00102763	0.404	0.129	2.71E-12	Macrophage:	Errfi1

1.96E-16	0.5012588	0.333	0.09	2.78E-12	Macrophage:	Etv3
2.06E-16	0.48543662	0.444	0.14	2.92E-12	Macrophage:	Il6ra
2.35E-16	0.55603513	0.202	0.039	3.33E-12	Macrophage:	4930430E12F
2.55E-16	0.39101159	0.323	0.084	3.60E-12	Macrophage:	5031439G07I
2.56E-16	0.40116804	0.313	0.081	3.63E-12	Macrophage:	Dennd5a
2.78E-16	0.98128578	0.99	0.872	3.93E-12	Macrophage:	Hspa8
2.83E-16	0.42699197	0.434	0.135	4.00E-12	Macrophage:	BC028528
2.99E-16	0.47272448	0.172	0.029	4.23E-12	Macrophage:	Pdgfb
3.24E-16	0.69503034	0.556	0.207	4.58E-12	Macrophage:	Herpud1
3.44E-16	0.8577228	0.687	0.304	4.87E-12	Macrophage:	Mat2a
3.58E-16	0.79002662	0.687	0.301	5.06E-12	Macrophage:	Cyth4
3.59E-16	0.52480863	0.374	0.112	5.09E-12	Macrophage:	Camk1d
3.77E-16	0.46035459	0.455	0.15	5.33E-12	Macrophage:	Tcirg1
3.89E-16	0.31880494	0.263	0.059	5.51E-12	Macrophage:	Gstm1
4.17E-16	0.38372191	0.242	0.054	5.90E-12	Macrophage:	Slc29a3
4.39E-16	0.42455504	0.343	0.094	6.21E-12	Macrophage:	Qk
4.39E-16	0.66232451	0.596	0.24	6.21E-12	Macrophage:	Hmox2
4.48E-16	0.41285882	0.222	0.046	6.34E-12	Macrophage:	Ly96
4.60E-16	0.52974028	0.313	0.084	6.51E-12	Macrophage:	Sec14l1
4.81E-16	0.36976591	0.162	0.026	6.81E-12	Macrophage:	Tlr4
5.26E-16	-1.6164344	0.313	0.644	7.45E-12	Macrophage:	Limd2
5.29E-16	0.31149584	0.141	0.021	7.49E-12	Macrophage:	Il11ra1
6.62E-16	-0.6145087	1	0.98	9.38E-12	Macrophage:	Fau
6.63E-16	-0.9010865	0.949	0.859	9.38E-12	Macrophage:	Rpl27
7.47E-16	0.86244407	0.929	0.646	1.06E-11	Macrophage:	Hspa5
7.91E-16	0.54270339	0.606	0.233	1.12E-11	Macrophage:	Prkcd
8.32E-16	0.40659416	0.192	0.037	1.18E-11	Macrophage:	Tspan4
8.68E-16	0.4479185	0.323	0.087	1.23E-11	Macrophage:	Dnajc13
9.59E-16	0.52650537	0.657	0.272	1.36E-11	Macrophage:	Canx
9.93E-16	-0.7709088	0.96	0.948	1.41E-11	Macrophage:	Gm10076
1.03E-15	0.70873214	0.455	0.149	1.46E-11	Macrophage:	Plk2
1.21E-15	0.71552522	0.667	0.295	1.71E-11	Macrophage:	Nfe2l2
1.33E-15	0.54909732	0.354	0.102	1.88E-11	Macrophage:	Tgfbr1
1.40E-15	0.51436273	0.394	0.123	1.98E-11	Macrophage:	Ncstn
1.64E-15	-0.8293252	0.97	0.921	2.32E-11	Macrophage:	Rpl30
1.67E-15	0.52426662	0.222	0.049	2.36E-11	Macrophage:	Renbp
1.86E-15	0.4085052	0.404	0.125	2.64E-11	Macrophage:	Cd300ld
1.98E-15	0.39140936	0.182	0.034	2.81E-11	Macrophage:	Manba
2.01E-15	0.57946528	0.273	0.069	2.84E-11	Macrophage:	Pira2
2.09E-15	0.46414418	0.222	0.048	2.96E-11	Macrophage:	Dbndd2
2.54E-15	0.67197284	0.758	0.36	3.59E-11	Macrophage:	Capza2
3.23E-15	0.446273	0.263	0.062	4.57E-11	Macrophage:	Abca1
3.24E-15	0.53086905	0.354	0.104	4.59E-11	Macrophage:	Irf2bp2
3.81E-15	0.33364639	0.152	0.025	5.40E-11	Macrophage:	Tmem141
3.87E-15	0.43200197	0.141	0.022	5.47E-11	Macrophage:	Tceal8
4.16E-15	0.27491667	0.152	0.025	5.88E-11	Macrophage:	Pparg
4.58E-15	0.34037023	0.101	0.012	6.48E-11	Macrophage:	Disc1
4.78E-15	0.26408114	0.131	0.019	6.76E-11	Macrophage:	Gm20481

4.81E-15	0.44036997	0.333	0.097	6.81E-11	Macrophage:	Tmem109
5.04E-15	0.40261681	0.293	0.077	7.13E-11	Macrophage:	Cep170
5.87E-15	0.31463916	0.182	0.034	8.31E-11	Macrophage:	Zmynd15
6.91E-15	0.48737435	0.414	0.138	9.79E-11	Macrophage:	Atp6v1a
7.21E-15	0.56707519	0.404	0.135	1.02E-10	Macrophage:	Mpp1
7.30E-15	0.44240296	0.273	0.069	1.03E-10	Macrophage:	Hgsnat
7.63E-15	0.53730317	0.242	0.056	1.08E-10	Macrophage:	Itga6
7.97E-15	0.28029376	0.646	0.255	1.13E-10	Macrophage:	Ncf2
8.59E-15	0.41199355	0.343	0.102	1.22E-10	Macrophage:	Mapk3
8.60E-15	0.59486909	0.354	0.11	1.22E-10	Macrophage:	Ap2a2
9.59E-15	0.37994122	0.303	0.082	1.36E-10	Macrophage:	Abcb1b
1.07E-14	0.78272751	0.455	0.169	1.51E-10	Macrophage:	Nop58
1.08E-14	0.30996981	0.152	0.025	1.53E-10	Macrophage:	Evi5
1.08E-14	-3.0113317	0.051	0.444	1.53E-10	Macrophage:	Cd3g
1.16E-14	0.40546779	0.263	0.066	1.64E-10	Macrophage:	Pisd
1.18E-14	0.46654045	0.424	0.14	1.67E-10	Macrophage:	Ncoa3
1.20E-14	-1.9861213	0.101	0.482	1.70E-10	Macrophage:	Cd2
1.28E-14	0.45403547	0.535	0.205	1.81E-10	Macrophage:	Creg1
1.35E-14	0.2614847	0.455	0.153	1.92E-10	Macrophage:	Snap23
1.38E-14	0.43714885	0.222	0.05	1.95E-10	Macrophage:	Rreb1
1.42E-14	0.51996315	0.303	0.085	2.00E-10	Macrophage:	Slc29a1
1.44E-14	0.52941664	0.303	0.086	2.04E-10	Macrophage:	Tep1
1.72E-14	0.52948235	0.475	0.169	2.43E-10	Macrophage:	Stx7
1.76E-14	0.55661904	0.394	0.129	2.49E-10	Macrophage:	Map3k8
1.78E-14	-0.7965542	0.96	0.915	2.52E-10	Macrophage:	Rps10
1.81E-14	0.53778415	0.566	0.231	2.56E-10	Macrophage:	Taok3
1.98E-14	0.52036295	0.606	0.253	2.80E-10	Macrophage:	Gltp
2.01E-14	0.59207342	0.646	0.284	2.84E-10	Macrophage:	Kdm6b
2.05E-14	0.42404971	0.283	0.075	2.90E-10	Macrophage:	Tbc1d9b
2.25E-14	0.31754022	0.263	0.065	3.19E-10	Macrophage:	Ncln
2.29E-14	-0.7320882	0.99	0.911	3.24E-10	Macrophage:	Rpl6
3.07E-14	0.3786781	0.232	0.054	4.34E-10	Macrophage:	Plod1
3.08E-14	0.36309401	0.293	0.08	4.37E-10	Macrophage:	Rcbtb2
3.10E-14	0.64877615	0.758	0.378	4.39E-10	Macrophage:	Snx5
3.42E-14	0.39839982	0.202	0.043	4.84E-10	Macrophage:	Gab2
3.49E-14	0.85346887	0.535	0.227	4.94E-10	Macrophage:	Gm26532
3.49E-14	0.30423592	0.162	0.029	4.94E-10	Macrophage:	Il15
3.58E-14	-1.904464	0.101	0.482	5.07E-10	Macrophage:	Ltb
3.97E-14	0.40244772	0.111	0.015	5.62E-10	Macrophage:	Tmem119
4.08E-14	-2.6184593	0.081	0.465	5.78E-10	Macrophage:	Ly6a
4.08E-14	0.33189036	0.141	0.023	5.78E-10	Macrophage:	Hlx
5.48E-14	0.50945831	0.535	0.21	7.76E-10	Macrophage:	Plekho2
5.60E-14	0.34199167	0.131	0.02	7.93E-10	Macrophage:	Nfxl1
5.87E-14	0.45633894	0.182	0.037	8.30E-10	Macrophage:	Mitf
6.14E-14	-0.6850706	0.98	0.909	8.69E-10	Macrophage:	Rpl11
6.22E-14	0.25432633	0.141	0.023	8.80E-10	Macrophage:	Slc35e4
7.06E-14	0.40771308	0.253	0.063	9.99E-10	Macrophage:	Nab2
7.49E-14	0.3512728	0.121	0.018	1.06E-09	Macrophage:	Itpripl1

8.34E-14	1.11996866	0.828	0.526	1.18E-09	Macrophage:	Dnajb1
8.47E-14	-1.494485	0.313	0.62	1.20E-09	Macrophage:	Il2rg
1.11E-13	0.37160238	0.273	0.073	1.57E-09	Macrophage:	Map3k11
1.14E-13	0.75384443	0.929	0.659	1.61E-09	Macrophage:	Atp6v0c
1.20E-13	0.45161158	0.505	0.194	1.70E-09	Macrophage:	Picalm
1.21E-13	0.49279152	0.293	0.084	1.72E-09	Macrophage:	Phf23
1.22E-13	0.51589609	0.424	0.151	1.72E-09	Macrophage:	Map7d1
1.29E-13	0.43110604	0.222	0.052	1.83E-09	Macrophage:	Diaph2
1.32E-13	0.42465304	0.111	0.016	1.86E-09	Macrophage:	Ch25h
1.38E-13	0.63934174	0.414	0.145	1.95E-09	Macrophage:	Scpep1
1.48E-13	-0.968218	0.99	0.901	2.10E-09	Macrophage:	Rps20
1.52E-13	0.59684124	0.556	0.237	2.16E-09	Macrophage:	Nptn
1.70E-13	0.45351837	0.485	0.182	2.40E-09	Macrophage:	Vmp1
1.79E-13	0.38455487	0.313	0.091	2.54E-09	Macrophage:	Atp13a2
2.06E-13	0.55205978	0.232	0.058	2.91E-09	Macrophage:	Tom1
2.06E-13	-1.6347475	0.192	0.571	2.92E-09	Macrophage:	Vps37b
2.09E-13	0.35351165	0.212	0.049	2.96E-09	Macrophage:	Vps37c
2.18E-13	0.44864624	0.354	0.111	3.09E-09	Macrophage:	Itgam
2.24E-13	-0.6563599	1	0.933	3.18E-09	Macrophage:	Rps27a
2.33E-13	0.32904113	0.152	0.027	3.30E-09	Macrophage:	Nckipsd
2.47E-13	-2.595166	0.051	0.412	3.49E-09	Macrophage:	Cd3d
2.50E-13	0.30530039	0.172	0.034	3.54E-09	Macrophage:	Klf11
2.56E-13	0.32657014	0.283	0.078	3.63E-09	Macrophage:	Ctla2b
2.66E-13	0.61761549	0.788	0.416	3.76E-09	Macrophage:	Ccnl1
2.79E-13	-2.6078219	0.101	0.457	3.94E-09	Macrophage:	Ms4a4b
2.92E-13	0.31713301	0.141	0.024	4.13E-09	Macrophage:	Mmp19
3.37E-13	0.28673332	0.273	0.074	4.78E-09	Macrophage:	Stx12
3.44E-13	0.48905345	0.283	0.081	4.87E-09	Macrophage:	Htra2
3.48E-13	0.46400254	0.202	0.046	4.92E-09	Macrophage:	Pmepa1
3.69E-13	0.54510255	0.434	0.159	5.23E-09	Macrophage:	Sbno2
3.89E-13	0.47789819	0.263	0.073	5.51E-09	Macrophage:	S100a1
4.32E-13	0.41053494	0.222	0.054	6.12E-09	Macrophage:	Itga5
4.44E-13	-2.2980314	0.03	0.39	6.28E-09	Macrophage:	Pglyrp1
4.44E-13	0.41363382	0.232	0.058	6.29E-09	Macrophage:	Pfkfb4
4.56E-13	1.12236121	0.929	0.641	6.46E-09	Macrophage:	Cd74
4.59E-13	0.71981867	0.475	0.184	6.50E-09	Macrophage:	Cd72
4.87E-13	0.29902869	0.232	0.058	6.90E-09	Macrophage:	Bcl2l1
4.91E-13	-1.6512045	0.071	0.429	6.96E-09	Macrophage:	1-Sep
5.01E-13	0.27572049	0.172	0.035	7.09E-09	Macrophage:	Tnfaip1
5.02E-13	-1.2433294	0.737	0.827	7.11E-09	Macrophage:	Ly6e
5.12E-13	0.6745948	0.778	0.427	7.25E-09	Macrophage:	Plek
5.40E-13	-0.6969675	0.99	0.914	7.65E-09	Macrophage:	Rps11
5.48E-13	0.29746971	0.152	0.028	7.76E-09	Macrophage:	Clec10a
5.52E-13	1.28343694	0.818	0.534	7.82E-09	Macrophage:	Sqstm1
5.85E-13	0.31647698	0.222	0.055	8.28E-09	Macrophage:	Ppfia4
6.30E-13	0.70425775	0.697	0.344	8.91E-09	Macrophage:	Efhd2
6.32E-13	0.54365957	0.162	0.032	8.95E-09	Macrophage:	Emp1
6.41E-13	0.3368251	0.303	0.089	9.07E-09	Macrophage:	Npepl1

6.67E-13	0.46423739	0.515	0.199	9.44E-09	Macrophage:	Gns
6.68E-13	0.47571512	0.343	0.111	9.46E-09	Macrophage:	Gapvd1
6.85E-13	0.96930633	0.848	0.556	9.70E-09	Macrophage:	Zfp36l1
7.24E-13	0.52172332	0.323	0.102	1.02E-08	Macrophage:	Mafg
8.50E-13	0.37742421	0.202	0.047	1.20E-08	Macrophage:	Parp12
8.70E-13	0.42940772	0.242	0.064	1.23E-08	Macrophage:	Tmbim1
8.70E-13	0.47788215	0.202	0.047	1.23E-08	Macrophage:	Lipe
8.83E-13	-0.8081724	1	0.939	1.25E-08	Macrophage:	Rps24
9.13E-13	0.65695563	0.384	0.14	1.29E-08	Macrophage:	Plekho1
9.28E-13	0.35562523	0.394	0.132	1.31E-08	Macrophage:	Relb
9.37E-13	0.33123687	0.182	0.039	1.33E-08	Macrophage:	Stom
9.79E-13	0.43171487	0.384	0.131	1.39E-08	Macrophage:	Ms4a6d
1.01E-12	-0.8826858	0.919	0.916	1.42E-08	Macrophage:	H2-K1
1.02E-12	0.2539888	0.192	0.042	1.44E-08	Macrophage:	Abcc5
1.16E-12	0.57330366	0.848	0.466	1.64E-08	Macrophage:	Gdi2
1.16E-12	-0.7959335	0.97	0.899	1.64E-08	Macrophage:	Rps26
1.23E-12	0.25522615	0.111	0.017	1.75E-08	Macrophage:	Abcd2
1.30E-12	0.33540823	0.242	0.063	1.84E-08	Macrophage:	Cyb5r1
1.31E-12	0.50156104	0.343	0.114	1.85E-08	Macrophage:	Oas1a
1.34E-12	0.3110961	0.434	0.159	1.90E-08	Macrophage:	Entpd1
1.38E-12	0.48428036	0.394	0.143	1.95E-08	Macrophage:	Comt
1.40E-12	0.29357399	0.202	0.046	1.99E-08	Macrophage:	Cass4
1.43E-12	0.4154685	0.525	0.211	2.02E-08	Macrophage:	Usf2
1.43E-12	-2.3969704	0.061	0.412	2.02E-08	Macrophage:	Cd3e
1.66E-12	0.27730232	0.101	0.014	2.35E-08	Macrophage:	Ophn1
1.77E-12	0.43693066	0.253	0.069	2.51E-08	Macrophage:	Tifa
1.82E-12	-0.6860331	1	0.906	2.58E-08	Macrophage:	Rpl34
1.86E-12	0.55504673	0.263	0.074	2.64E-08	Macrophage:	Tmcc3
1.92E-12	0.37186279	0.172	0.037	2.71E-08	Macrophage:	F11r
1.98E-12	0.30451178	0.283	0.081	2.80E-08	Macrophage:	Rb1
2.03E-12	0.39152697	0.192	0.045	2.87E-08	Macrophage:	Zfp467
2.08E-12	0.92823455	0.646	0.33	2.94E-08	Macrophage:	Cited2
2.16E-12	0.40703595	0.152	0.029	3.06E-08	Macrophage:	Gm10138
2.17E-12	0.40325777	0.444	0.169	3.07E-08	Macrophage:	Lgals3bp
2.30E-12	0.3754907	0.182	0.04	3.25E-08	Macrophage:	Pvr
2.31E-12	0.40559431	0.222	0.056	3.27E-08	Macrophage:	Tlr7
2.49E-12	0.32306208	0.232	0.06	3.53E-08	Macrophage:	Slc8b1
2.57E-12	0.47364044	0.384	0.134	3.63E-08	Macrophage:	Cat
2.90E-12	0.35521695	0.222	0.056	4.11E-08	Macrophage:	Zswim6
2.97E-12	0.46000193	0.646	0.304	4.20E-08	Macrophage:	Atp1a1
2.98E-12	0.4278696	0.364	0.122	4.22E-08	Macrophage:	Arap1
3.18E-12	0.56001014	0.545	0.231	4.51E-08	Macrophage:	Atp6ap1
3.22E-12	0.56129634	0.354	0.119	4.56E-08	Macrophage:	P2rx4
3.24E-12	0.35110572	0.172	0.037	4.58E-08	Macrophage:	Stard9
4.02E-12	0.50192745	0.242	0.068	5.69E-08	Macrophage:	Milr1
4.02E-12	0.60326062	0.162	0.034	5.70E-08	Macrophage:	Rcan1
4.55E-12	0.4770972	0.707	0.328	6.44E-08	Macrophage:	Bst2
4.58E-12	0.36386762	0.222	0.057	6.49E-08	Macrophage:	Nkiras2

5.17E-12	0.55818837	0.667	0.336	7.32E-08	Macrophage:	Atp2b1
5.18E-12	0.31275777	0.172	0.037	7.33E-08	Macrophage:	Aph1c
5.22E-12	0.43985022	0.293	0.091	7.39E-08	Macrophage:	Pacsin2
5.23E-12	0.30799315	0.131	0.023	7.40E-08	Macrophage:	Pon3
6.54E-12	0.33110905	0.253	0.071	9.26E-08	Macrophage:	Frmd4b
6.59E-12	0.28449151	0.111	0.017	9.32E-08	Macrophage:	Nlrc4
7.02E-12	0.37890918	0.131	0.024	9.94E-08	Macrophage:	Nav1
7.23E-12	-1.3220321	0.677	0.73	1.02E-07	Macrophage:	Shisa5
8.22E-12	0.40746422	0.444	0.171	1.16E-07	Macrophage:	Zmiz1
8.60E-12	0.28325056	0.232	0.062	1.22E-07	Macrophage:	Hk3
8.73E-12	0.32552099	0.465	0.176	1.24E-07	Macrophage:	Pirb
8.87E-12	0.98997827	0.939	0.723	1.26E-07	Macrophage:	Hsp90aa1
1.00E-11	2.20518543	0.192	0.048	1.42E-07	Macrophage:	Hspb1
1.18E-11	0.47362766	0.687	0.34	1.66E-07	Macrophage:	Tmem59
1.21E-11	0.53888637	0.576	0.255	1.71E-07	Macrophage:	Snx2
1.29E-11	0.46316059	0.333	0.114	1.83E-07	Macrophage:	Mvp
1.30E-11	0.27231141	0.152	0.03	1.84E-07	Macrophage:	Myo9a
1.40E-11	0.41637968	0.253	0.073	1.99E-07	Macrophage:	Baiap2
1.41E-11	0.53720426	0.525	0.228	1.99E-07	Macrophage:	Fuca1
1.41E-11	0.27132213	0.152	0.031	2.00E-07	Macrophage:	Tspan17
1.54E-11	0.55825329	0.424	0.166	2.18E-07	Macrophage:	Eif1a
1.77E-11	0.34050361	0.141	0.028	2.50E-07	Macrophage:	Fkbp1b
1.97E-11	-1.9455471	0.02	0.346	2.79E-07	Macrophage:	Sh2d2a
2.01E-11	0.37920502	0.283	0.088	2.85E-07	Macrophage:	Ogfrl1
2.15E-11	0.5071165	0.293	0.094	3.05E-07	Macrophage:	Ube2e3
2.26E-11	0.42506165	0.475	0.189	3.20E-07	Macrophage:	Jmjd1c
2.35E-11	0.25937809	0.131	0.024	3.32E-07	Macrophage:	Dusp23
2.42E-11	0.50517542	0.354	0.129	3.43E-07	Macrophage:	Sap30
2.49E-11	-0.5972339	0.99	0.937	3.52E-07	Macrophage:	Rpl23
2.57E-11	0.3406104	0.242	0.069	3.63E-07	Macrophage:	Dnajb14
2.58E-11	0.33615555	0.333	0.112	3.65E-07	Macrophage:	Pilra
2.64E-11	0.281792	0.101	0.015	3.74E-07	Macrophage:	Plxna4os1
2.70E-11	0.33217532	0.293	0.092	3.82E-07	Macrophage:	2-Sep
2.83E-11	0.29120303	0.111	0.018	4.00E-07	Macrophage:	Gamt
3.35E-11	0.6116066	0.626	0.309	4.75E-07	Macrophage:	P4hb
3.45E-11	-1.6554439	0.152	0.471	4.89E-07	Macrophage:	Ets1
3.58E-11	0.47124917	0.323	0.111	5.06E-07	Macrophage:	Colgalt1
3.68E-11	0.66233553	0.283	0.09	5.20E-07	Macrophage:	Zfand2a
3.85E-11	0.42620673	0.222	0.061	5.45E-07	Macrophage:	Mfsd11
3.95E-11	0.99202028	0.818	0.506	5.59E-07	Macrophage:	H2-Aa
4.22E-11	0.44055526	0.414	0.154	5.98E-07	Macrophage:	Man1a
4.50E-11	0.46300913	0.242	0.069	6.37E-07	Macrophage:	Vps18
4.56E-11	0.26524311	0.152	0.032	6.45E-07	Macrophage:	As3mt
4.97E-11	0.56145571	0.939	0.661	7.03E-07	Macrophage:	Rpl10
4.97E-11	0.94903917	0.818	0.49	7.04E-07	Macrophage:	H2-Ab1
5.02E-11	-0.748997	0.919	0.85	7.11E-07	Macrophage:	Rpl15
5.18E-11	0.39263488	0.242	0.071	7.34E-07	Macrophage:	Uap1l1
5.21E-11	0.42318905	0.576	0.257	7.37E-07	Macrophage:	Tgfbr2

5.27E-11	0.38007338	0.232	0.066	7.47E-07	Macrophage:	Arl8a
5.29E-11	0.59457011	0.525	0.243	7.49E-07	Macrophage:	BC005537
5.34E-11	0.83802502	0.717	0.417	7.56E-07	Macrophage:	Irf1
5.86E-11	-0.6896526	0.98	0.886	8.29E-07	Macrophage:	Rps13
6.06E-11	0.48291104	0.293	0.096	8.57E-07	Macrophage:	Bmyc
6.31E-11	0.25875667	0.141	0.029	8.93E-07	Macrophage:	Oas2
6.36E-11	0.67238199	0.485	0.213	9.00E-07	Macrophage:	Skil
7.02E-11	0.80248486	0.374	0.141	9.93E-07	Macrophage:	Rasgef1b
7.05E-11	0.463756	0.333	0.12	9.97E-07	Macrophage:	Nenf
7.19E-11	-1.3906403	0.677	0.696	1.02E-06	Macrophage:	Psmb8
7.26E-11	0.33778383	0.121	0.022	1.03E-06	Macrophage:	Spg20
8.04E-11	0.56659421	0.485	0.215	1.14E-06	Macrophage:	Rab1a
8.14E-11	0.3959185	0.263	0.082	1.15E-06	Macrophage:	H1f0
8.20E-11	0.30616096	0.192	0.048	1.16E-06	Macrophage:	Lrp6
8.43E-11	0.31089999	0.283	0.086	1.19E-06	Macrophage:	Mtss1
9.71E-11	0.48070237	0.899	0.538	1.37E-06	Macrophage:	Gnb2
1.04E-10	0.40673738	0.293	0.096	1.48E-06	Macrophage:	Cd180
1.08E-10	0.34919695	0.202	0.053	1.53E-06	Macrophage:	Rp2
1.21E-10	0.32943534	0.192	0.048	1.72E-06	Macrophage:	Icosl
1.22E-10	0.57258817	0.455	0.201	1.72E-06	Macrophage:	Ilk
1.32E-10	0.43611887	0.364	0.137	1.87E-06	Macrophage:	Cnpy3
1.33E-10	0.33181001	0.323	0.112	1.88E-06	Macrophage:	Sgpl1
1.33E-10	0.40753673	0.333	0.117	1.88E-06	Macrophage:	Bach1
1.33E-10	0.31459764	0.384	0.144	1.89E-06	Macrophage:	Pxn
1.39E-10	0.43222129	0.384	0.15	1.96E-06	Macrophage:	Myd88
1.45E-10	0.53018819	0.394	0.154	2.05E-06	Macrophage:	Serpinb6a
1.47E-10	0.27738153	0.192	0.049	2.08E-06	Macrophage:	Tbc1d5
1.51E-10	0.5530975	0.566	0.275	2.14E-06	Macrophage:	Csrnp1
1.59E-10	0.48373115	0.414	0.167	2.24E-06	Macrophage:	Tmed7
1.64E-10	0.42376853	0.444	0.184	2.32E-06	Macrophage:	Ptpre
1.72E-10	0.53522351	0.313	0.108	2.44E-06	Macrophage:	Ifi204
1.83E-10	0.34250283	0.323	0.112	2.59E-06	Macrophage:	Dram2
2.05E-10	0.26598953	0.202	0.054	2.91E-06	Macrophage:	Mpv17
2.12E-10	0.45092855	0.818	0.443	3.00E-06	Macrophage:	Pdia3
2.14E-10	0.32777512	0.263	0.082	3.03E-06	Macrophage:	Edem2
2.21E-10	-1.5087283	0.253	0.5	3.13E-06	Macrophage:	Gstp1
2.31E-10	0.51532279	0.828	0.459	3.28E-06	Macrophage:	Atp6v0b
2.32E-10	0.2768863	0.152	0.034	3.28E-06	Macrophage:	Cpq
2.32E-10	0.35792098	0.364	0.127	3.29E-06	Macrophage:	Blnk
2.32E-10	0.2970842	0.192	0.05	3.29E-06	Macrophage:	Slc25a45
2.37E-10	-2.6451475	0.051	0.349	3.35E-06	Macrophage:	Cd8b1
2.43E-10	-1.6967748	0.364	0.617	3.44E-06	Macrophage:	S100a6
2.44E-10	-0.7649888	0.879	0.862	3.45E-06	Macrophage:	Coro1a
2.75E-10	0.49553655	0.707	0.379	3.89E-06	Macrophage:	Laptm4a
2.81E-10	0.51550402	0.394	0.161	3.97E-06	Macrophage:	Slc6a6
2.89E-10	-2.0787008	0.01	0.31	4.09E-06	Macrophage:	Klrd1
3.15E-10	-0.6593028	1	0.905	4.46E-06	Macrophage:	Rps5
3.39E-10	1.13756702	0.778	0.501	4.81E-06	Macrophage:	H2-Eb1

3.41E-10	0.36750852	0.444	0.184	4.82E-06	Macrophage:	Vav1
3.45E-10	0.27978533	0.242	0.073	4.89E-06	Macrophage:	Polr3gl
3.61E-10	0.55391487	0.758	0.397	5.11E-06	Macrophage:	Akr1a1
3.85E-10	-1.6403541	0.384	0.571	5.44E-06	Macrophage:	H2-Q7
3.85E-10	-0.6248826	0.99	0.877	5.45E-06	Macrophage:	Rplp2
3.89E-10	-1.9694748	0.03	0.325	5.50E-06	Macrophage:	Ctsw
4.11E-10	-1.8043867	0.071	0.363	5.81E-06	Macrophage:	Lat
4.14E-10	0.40762782	0.333	0.125	5.87E-06	Macrophage:	Ggh
4.17E-10	0.30805754	0.283	0.094	5.91E-06	Macrophage:	Rab9
4.58E-10	0.49941706	0.293	0.102	6.48E-06	Macrophage:	Scimp
4.66E-10	0.44730905	0.535	0.242	6.60E-06	Macrophage:	Epn1
4.97E-10	0.49061207	0.394	0.162	7.04E-06	Macrophage:	Naa50
4.99E-10	0.43173085	0.253	0.081	7.06E-06	Macrophage:	Nagk
5.05E-10	-2.2161676	0.02	0.315	7.15E-06	Macrophage:	Cd8a
5.34E-10	-0.6637343	0.98	0.891	7.56E-06	Macrophage:	Rpl32
5.48E-10	-1.4732529	0.04	0.338	7.76E-06	Macrophage:	Ikzf3
5.79E-10	0.42425253	0.303	0.106	8.20E-06	Macrophage:	Ppp1r12c
5.82E-10	0.29738761	0.263	0.083	8.23E-06	Macrophage:	Mapk6
5.82E-10	0.41413228	0.212	0.062	8.23E-06	Macrophage:	Dusp22
6.47E-10	0.26424299	0.343	0.128	9.16E-06	Macrophage:	Leprot
6.73E-10	0.36251934	0.212	0.061	9.53E-06	Macrophage:	Mcfd2
7.29E-10	0.37615212	0.293	0.101	1.03E-05	Macrophage:	Dnase2a
7.57E-10	0.38121381	0.152	0.035	1.07E-05	Macrophage:	Tmem9
8.28E-10	0.54144421	0.192	0.053	1.17E-05	Macrophage:	Mid1ip1
8.35E-10	0.3802332	0.141	0.031	1.18E-05	Macrophage:	Tcta
8.68E-10	-1.3139562	0.03	0.316	1.23E-05	Macrophage:	Gimap1
9.74E-10	0.36167968	0.222	0.067	1.38E-05	Macrophage:	Aldh3b1
9.88E-10	0.47715575	0.374	0.152	1.40E-05	Macrophage:	Cflar
1.02E-09	0.39405179	0.273	0.094	1.44E-05	Macrophage:	Pdcd2l
1.02E-09	0.39680688	0.394	0.158	1.45E-05	Macrophage:	Nisch
1.03E-09	-0.7287396	0.98	0.951	1.46E-05	Macrophage:	Pfn1
1.03E-09	-0.8233699	0.919	0.829	1.46E-05	Macrophage:	Rps18
1.07E-09	0.39299698	0.364	0.142	1.51E-05	Macrophage:	Scarb2
1.16E-09	0.39266921	0.212	0.063	1.64E-05	Macrophage:	Smim10l1
1.17E-09	0.33423327	0.121	0.025	1.66E-05	Macrophage:	Snx24
1.19E-09	0.65159089	0.687	0.356	1.68E-05	Macrophage:	Cstb
1.24E-09	0.30993636	0.263	0.087	1.76E-05	Macrophage:	Stard3
1.26E-09	0.31459051	0.121	0.025	1.78E-05	Macrophage:	Cadm1
1.44E-09	-0.873614	0.707	0.764	2.04E-05	Macrophage:	Eif3f
1.66E-09	0.66479579	0.707	0.445	2.34E-05	Macrophage:	Srsf2
1.66E-09	-0.6583252	0.919	0.863	2.35E-05	Macrophage:	Rpl29
1.72E-09	0.3214085	0.242	0.078	2.44E-05	Macrophage:	Hk2
1.86E-09	0.31564667	0.121	0.025	2.63E-05	Macrophage:	Rab40c
1.88E-09	0.40074048	0.374	0.148	2.67E-05	Macrophage:	Oat
1.90E-09	-0.6068481	0.97	0.871	2.69E-05	Macrophage:	Rpl24
1.90E-09	0.37660279	0.576	0.274	2.70E-05	Macrophage:	Pitpna
2.05E-09	0.2879373	0.172	0.045	2.91E-05	Macrophage:	Rnf141
2.10E-09	0.28714543	0.162	0.041	2.97E-05	Macrophage:	Prkra

2.15E-09	0.30065418	0.354	0.135	3.05E-05	Macrophage:	Commd8
2.41E-09	0.29026513	0.162	0.041	3.41E-05	Macrophage:	Ctbs
2.60E-09	0.37557064	0.192	0.054	3.68E-05	Macrophage:	Tmem86a
2.86E-09	0.4641655	0.556	0.272	4.05E-05	Macrophage:	Ifnar2
2.95E-09	0.40833016	0.172	0.045	4.18E-05	Macrophage:	Slc5a3
3.05E-09	0.51621936	0.384	0.162	4.32E-05	Macrophage:	Aplp2
3.06E-09	0.78654423	0.414	0.183	4.33E-05	Macrophage:	Dnajb9
3.09E-09	0.27219704	0.475	0.2	4.37E-05	Macrophage:	Ptp4a1
3.17E-09	0.52025529	0.576	0.299	4.49E-05	Macrophage:	Coro1b
3.42E-09	0.33885581	0.131	0.029	4.84E-05	Macrophage:	Ssh3
3.50E-09	-0.5871225	0.98	0.91	4.95E-05	Macrophage:	Rps4x
3.52E-09	0.2751875	0.374	0.149	4.98E-05	Macrophage:	Pik3ap1
3.68E-09	0.38890791	0.545	0.26	5.21E-05	Macrophage:	Hprt
3.80E-09	-0.5847277	0.99	0.893	5.38E-05	Macrophage:	Rpl27a
4.02E-09	0.38494066	0.242	0.078	5.69E-05	Macrophage:	Slc15a4
4.13E-09	-1.7737911	0.01	0.278	5.85E-05	Macrophage:	Tigit
4.66E-09	-0.6766801	0.939	0.877	6.59E-05	Macrophage:	Oaz1
4.66E-09	0.26836569	0.172	0.046	6.60E-05	Macrophage:	Gm20707
4.71E-09	-1.9618417	0.01	0.277	6.67E-05	Macrophage:	Pdcd1
5.01E-09	0.37423052	0.283	0.101	7.09E-05	Macrophage:	Naa20
5.08E-09	0.30712628	0.192	0.055	7.19E-05	Macrophage:	Tnip2
5.09E-09	0.36503794	0.232	0.075	7.21E-05	Macrophage:	Gabarapl1
5.12E-09	0.34922359	0.313	0.117	7.24E-05	Macrophage:	Rassf2
5.24E-09	0.3892505	0.152	0.038	7.42E-05	Macrophage:	Gcc1
5.33E-09	0.38662183	0.273	0.097	7.55E-05	Macrophage:	Ripk1
5.38E-09	0.6337018	0.677	0.366	7.62E-05	Macrophage:	H2-DMa
5.49E-09	0.30657595	0.303	0.112	7.77E-05	Macrophage:	2610507B11I
5.49E-09	-1.5282784	0.061	0.326	7.77E-05	Macrophage:	Lck
5.68E-09	0.30323553	0.687	0.336	8.05E-05	Macrophage:	Lyn
5.87E-09	0.33735698	0.242	0.079	8.31E-05	Macrophage:	Ptger4
6.01E-09	0.51368331	0.747	0.434	8.51E-05	Macrophage:	Tuba1b
6.08E-09	-1.6306771	0.02	0.284	8.60E-05	Macrophage:	Cst7
6.13E-09	-0.6386185	0.96	0.869	8.68E-05	Macrophage:	Rpl36
6.14E-09	0.29977008	0.434	0.18	8.69E-05	Macrophage:	Taf6l
6.14E-09	0.3497174	0.182	0.051	8.70E-05	Macrophage:	1700025G04I
6.17E-09	0.31463018	0.253	0.085	8.73E-05	Macrophage:	Man2b2
6.19E-09	0.26166069	0.111	0.022	8.77E-05	Macrophage:	Xylt2
6.22E-09	0.29367239	0.263	0.089	8.81E-05	Macrophage:	Fnip1
6.54E-09	0.37888069	0.303	0.112	9.26E-05	Macrophage:	Pdlim1
7.19E-09	-1.3894409	0	0.259	0.00010173	Macrophage:	Tnfrsf18
7.32E-09	0.3191936	0.121	0.026	0.00010367	Macrophage:	Mib2
7.46E-09	0.37688435	0.404	0.175	0.00010563	Macrophage:	Dctn2
7.54E-09	0.40833554	0.434	0.193	0.00010668	Macrophage:	Bin1
7.72E-09	0.35542057	0.394	0.17	0.00010922	Macrophage:	Sirt2
8.02E-09	-1.6153946	0.061	0.327	0.00011358	Macrophage:	Thy1
8.68E-09	-0.806507	0.879	0.797	0.00012285	Macrophage:	Naca
8.87E-09	0.56002413	0.96	0.778	0.0001255	Macrophage:	Jund
9.02E-09	0.26244002	0.515	0.23	0.00012765	Macrophage:	Mef2c

9.11E-09	0.43411547	0.354	0.145	0.00012901	Macrophage:	Mapk14
9.21E-09	0.26765987	0.323	0.123	0.00013038	Macrophage:	Nfam1
9.38E-09	0.33846952	0.394	0.165	0.00013272	Macrophage:	Pon2
9.46E-09	-1.3795672	0.374	0.572	0.00013391	Macrophage:	Cytip
9.51E-09	0.28375827	0.273	0.094	0.00013465	Macrophage:	Btk
9.90E-09	-0.5103792	1	0.928	0.00014015	Macrophage:	Rpl37
1.02E-08	0.42876973	0.657	0.348	0.00014486	Macrophage:	Mrfap1
1.12E-08	-0.5251119	0.99	0.935	0.000159	Macrophage:	Rps9
1.14E-08	0.3437982	0.303	0.114	0.00016128	Macrophage:	Synj1
1.18E-08	0.34428773	0.475	0.215	0.00016672	Macrophage:	Birc3
1.21E-08	0.3690405	0.202	0.061	0.00017157	Macrophage:	Bbc3
1.22E-08	0.42426484	0.556	0.278	0.0001721	Macrophage:	Sod2
1.23E-08	0.48250462	0.273	0.101	0.00017452	Macrophage:	Ap1b1
1.24E-08	0.30788313	0.495	0.233	0.00017544	Macrophage:	Etf1
1.26E-08	-1.6777128	0.545	0.651	0.00017875	Macrophage:	S100a11
1.32E-08	-0.5772849	0.96	0.876	0.00018635	Macrophage:	Rps14
1.45E-08	0.6428235	0.687	0.414	0.00020563	Macrophage:	Tuba1c
1.70E-08	0.38581283	0.556	0.279	0.00024032	Macrophage:	Ddost
1.74E-08	0.32667681	0.343	0.135	0.00024566	Macrophage:	Siah2
1.82E-08	0.324255	0.152	0.039	0.00025745	Macrophage:	Naip5
1.86E-08	0.46942569	0.636	0.349	0.00026304	Macrophage:	Ywhae
1.87E-08	-1.4180387	0.283	0.509	0.00026402	Macrophage:	Fam107b
1.88E-08	0.35135427	0.768	0.427	0.00026573	Macrophage:	Tgfb1
1.90E-08	-2.0948002	0.091	0.35	0.00026866	Macrophage:	Plac8
1.90E-08	0.34373953	0.283	0.106	0.00026956	Macrophage:	Slc35b2
2.06E-08	0.35088071	0.354	0.147	0.00029118	Macrophage:	Mapkapk2
2.19E-08	0.25167797	0.212	0.067	0.00031022	Macrophage:	Lonp1
2.22E-08	0.2569791	0.212	0.069	0.00031475	Macrophage:	Dcxr
2.38E-08	0.3070154	0.202	0.063	0.00033634	Macrophage:	Sh3bp2
2.40E-08	0.49048262	0.848	0.54	0.00034006	Macrophage:	Eif4a1
2.67E-08	0.32598736	0.424	0.19	0.00037777	Macrophage:	Pip4k2a
2.82E-08	0.31613463	0.323	0.129	0.00039919	Macrophage:	Reep3
2.99E-08	-1.5190345	0.242	0.461	0.00042256	Macrophage:	Hcst
2.99E-08	0.59531476	0.414	0.186	0.00042366	Macrophage:	Cd9
3.05E-08	0.31716923	0.242	0.083	0.00043246	Macrophage:	Bmp2k
3.06E-08	0.28295126	0.253	0.086	0.00043254	Macrophage:	Myc
3.06E-08	-0.876329	0.768	0.766	0.00043327	Macrophage:	Myl12b
3.16E-08	-1.2540271	0.051	0.304	0.00044705	Macrophage:	Gm8369
3.36E-08	0.25177802	0.172	0.049	0.00047596	Macrophage:	Man2c1
3.39E-08	-0.4839555	0.99	0.951	0.00047923	Macrophage:	Tpt1
3.70E-08	0.4715529	0.475	0.232	0.00052413	Macrophage:	Oser1
3.82E-08	0.28788668	0.202	0.063	0.00054071	Macrophage:	Myo18a
3.90E-08	-0.7710513	0.838	0.768	0.00055173	Macrophage:	Btf3
4.17E-08	0.51945959	0.677	0.406	0.0005901	Macrophage:	Ahnak
4.30E-08	-0.6481191	0.929	0.825	0.00060843	Macrophage:	Rpl14
4.39E-08	0.66529994	0.697	0.432	0.00062164	Macrophage:	Rsrp1
4.41E-08	-1.5164411	0.02	0.264	0.00062412	Macrophage:	Il2rb
4.58E-08	0.35868543	0.343	0.141	0.00064892	Macrophage:	Ivns1abp

4.97E-08	0.34368714	0.222	0.075	0.00070387	Macrophage:	Mrpl3
5.04E-08	0.27990613	0.182	0.054	0.00071351	Macrophage:	Kat5
5.33E-08	-1.1805315	0.212	0.455	0.00075478	Macrophage:	Prdx6
5.74E-08	0.34057979	0.192	0.06	0.00081209	Macrophage:	Scarb1
6.28E-08	0.4192284	0.394	0.174	0.00088951	Macrophage:	Zc3h12a
6.49E-08	0.32977492	0.202	0.065	0.00091853	Macrophage:	Adam9
6.58E-08	0.32883447	0.172	0.051	0.00093095	Macrophage:	Dync1li2
6.63E-08	0.26588219	0.273	0.103	0.00093895	Macrophage:	Ppp1r9b
6.95E-08	0.35724885	0.859	0.565	0.00098419	Macrophage:	Zfp36l2
7.22E-08	0.28419885	0.152	0.042	0.00102244	Macrophage:	Frmd4a
7.45E-08	0.49093933	0.465	0.238	0.00105465	Macrophage:	Scamp2
7.53E-08	0.51869788	0.909	0.723	0.0010659	Macrophage:	mt-Nd4
7.74E-08	-1.2533009	0.081	0.329	0.00109592	Macrophage:	Gimap3
7.95E-08	-1.4041785	0.172	0.414	0.00112494	Macrophage:	Gimap6
7.99E-08	-1.272397	0.091	0.34	0.00113098	Macrophage:	Gimap4
8.59E-08	0.29468346	0.384	0.171	0.0012163	Macrophage:	Rela
8.69E-08	0.29221092	0.192	0.061	0.00122949	Macrophage:	Mcoln1
8.92E-08	0.26315746	0.121	0.029	0.00126214	Macrophage:	Maml2
9.37E-08	-0.7679633	0.869	0.782	0.00132648	Macrophage:	Cox8a
9.48E-08	0.32452863	0.242	0.087	0.00134144	Macrophage:	Furin
9.51E-08	0.2905863	0.131	0.034	0.00134684	Macrophage:	Tmem159
9.95E-08	-2.2594191	0.01	0.241	0.00140906	Macrophage:	Gzmb
1.04E-07	0.61843959	0.838	0.496	0.00146804	Macrophage:	Pcbp1
1.11E-07	-1.7610689	0.01	0.239	0.0015741	Macrophage:	Ctla4
1.13E-07	0.47174863	0.444	0.216	0.0015966	Macrophage:	Rpn2
1.13E-07	0.25259137	0.212	0.072	0.00159876	Macrophage:	Ltbr
1.15E-07	0.2634051	0.253	0.091	0.00162135	Macrophage:	Tfeb
1.24E-07	-1.132927	0.03	0.262	0.00175666	Macrophage:	Skap1
1.26E-07	0.2720427	0.364	0.156	0.00178005	Macrophage:	Hexim1
1.28E-07	-0.7642878	0.848	0.753	0.00180653	Macrophage:	Rpl36al
1.32E-07	0.27842294	0.111	0.026	0.00187026	Macrophage:	Akr1e1
1.36E-07	0.26320347	0.384	0.169	0.00192932	Macrophage:	Tm9sf2
1.37E-07	0.29596169	0.172	0.053	0.00194287	Macrophage:	Prkar2a
1.43E-07	0.40861695	0.182	0.056	0.00202748	Macrophage:	Hes1
1.47E-07	0.27829081	0.242	0.088	0.00207785	Macrophage:	Epb41l2
1.55E-07	0.31905978	0.677	0.364	0.00218792	Macrophage:	Ptms
1.55E-07	0.33208614	0.232	0.083	0.00220056	Macrophage:	Rock2
1.56E-07	0.33241194	0.121	0.029	0.00220144	Macrophage:	Thbd
1.58E-07	0.4995545	0.657	0.384	0.0022376	Macrophage:	Calr
1.64E-07	-1.168063	0.081	0.326	0.00232544	Macrophage:	Ablim1
1.66E-07	0.32720684	0.172	0.053	0.00234443	Macrophage:	Acp2
1.71E-07	0.29734813	0.212	0.072	0.0024251	Macrophage:	Pik3c2a
1.71E-07	0.32203624	0.212	0.074	0.00242591	Macrophage:	Plbd2
1.82E-07	-1.5738607	0.04	0.271	0.00257162	Macrophage:	Icos
1.82E-07	0.41300023	0.303	0.128	0.00257287	Macrophage:	Sema4a
1.86E-07	0.40672178	0.253	0.098	0.00263244	Macrophage:	Rap2b
1.89E-07	0.30149351	0.182	0.058	0.00268254	Macrophage:	Nagpa
1.94E-07	-0.5693975	0.949	0.851	0.00273948	Macrophage:	Rps23

1.94E-07	0.29885227	0.162	0.049	0.00274334	Macrophage:	Tns3
2.05E-07	-1.4479848	0.192	0.428	0.00290257	Macrophage:	Nr4a2
2.06E-07	0.30836972	0.232	0.085	0.00291476	Macrophage:	Abcd1
2.08E-07	0.68642895	0.636	0.4	0.0029476	Macrophage:	Ifrd1
2.09E-07	-1.4616956	0.101	0.33	0.00295241	Macrophage:	Napsa
2.12E-07	0.82823513	0.354	0.162	0.00299998	Macrophage:	Anxa1
2.16E-07	0.29547368	0.242	0.091	0.00305379	Macrophage:	Dpysl2
2.18E-07	-0.678259	0.859	0.815	0.00308237	Macrophage:	Rpl41
2.27E-07	-1.1032934	0.03	0.256	0.00321511	Macrophage:	Gimap5
2.28E-07	0.39313773	0.545	0.301	0.00323019	Macrophage:	Rab7
2.32E-07	0.31162224	0.343	0.151	0.00328556	Macrophage:	Tmem173
2.41E-07	0.41798271	0.283	0.115	0.00340706	Macrophage:	Srsf9
2.42E-07	0.37900879	0.202	0.07	0.00342678	Macrophage:	Dennd1a
2.48E-07	0.49239465	0.99	0.863	0.00351108	Macrophage:	mt-Nd1
2.55E-07	-0.9941902	0.657	0.671	0.00361492	Macrophage:	Rpl22l1
2.64E-07	0.32620665	0.222	0.08	0.0037329	Macrophage:	Ctnna1
2.64E-07	-1.111261	0.04	0.273	0.00373543	Macrophage:	Itgal
2.67E-07	0.31273081	0.596	0.315	0.00378043	Macrophage:	Tkt
2.72E-07	0.44655052	0.374	0.169	0.00385456	Macrophage:	Gpr183
2.73E-07	0.38295416	0.182	0.058	0.00386076	Macrophage:	Ralgds
2.83E-07	-1.0377961	0.404	0.548	0.00400166	Macrophage:	Fis1
2.83E-07	0.70021301	0.859	0.691	0.00400674	Macrophage:	Dnaja1
3.00E-07	0.34220825	0.566	0.301	0.00424465	Macrophage:	Il10ra
3.03E-07	0.33233825	0.394	0.178	0.00428434	Macrophage:	Plbd1
3.04E-07	-0.9628481	0.626	0.658	0.00430696	Macrophage:	Atp5g3
3.08E-07	0.59217528	0.96	0.822	0.0043568	Macrophage:	mt-Nd2
3.25E-07	0.32328162	0.232	0.085	0.00459628	Macrophage:	Nbr1
3.37E-07	-1.147439	0.02	0.238	0.00477112	Macrophage:	Gimap7
3.53E-07	-1.5380073	0.01	0.224	0.00499662	Macrophage:	Lag3
3.56E-07	0.31822141	0.162	0.049	0.00503724	Macrophage:	Slc16a10
3.66E-07	0.3480329	0.919	0.669	0.00517762	Macrophage:	Gabarap
3.84E-07	0.39774539	0.343	0.156	0.00543605	Macrophage:	Ccrl2
4.40E-07	-0.9786434	0.04	0.26	0.00622558	Macrophage:	Itgb7
4.41E-07	0.41065973	0.465	0.241	0.006239	Macrophage:	Tiparp
4.46E-07	0.29528025	0.263	0.103	0.0063131	Macrophage:	Ddi2
4.50E-07	0.31577422	0.222	0.082	0.00636648	Macrophage:	Rabl6
4.59E-07	0.63841213	0.242	0.094	0.00649119	Macrophage:	Il1rn
4.70E-07	0.26449763	0.283	0.114	0.00664902	Macrophage:	Pdlim5
4.73E-07	0.37923413	0.303	0.13	0.00669166	Macrophage:	Clptm1
4.74E-07	0.33508306	0.263	0.106	0.00670982	Macrophage:	Usf1
4.80E-07	0.33146834	0.283	0.117	0.00680026	Macrophage:	Rnf5
4.82E-07	0.285676	0.242	0.092	0.00682357	Macrophage:	Dusp3
4.96E-07	-0.7903905	0.798	0.761	0.00702611	Macrophage:	Eef1b2
5.20E-07	0.40426904	0.838	0.56	0.00736426	Macrophage:	Tpm3
5.62E-07	0.2649417	0.172	0.055	0.00796118	Macrophage:	Mpp6
6.02E-07	0.2595896	0.222	0.081	0.00851981	Macrophage:	Strn4
6.17E-07	0.2738569	0.131	0.036	0.00874079	Macrophage:	Dag1
6.44E-07	0.25372612	0.212	0.076	0.00911657	Macrophage:	Pcyt1a

6.45E-07	0.41838118	1	0.92	0.00912963	Macrophage:	mt-Cytb
6.66E-07	0.32000131	0.485	0.25	0.00943352	Macrophage:	Rab5c
6.92E-07	-1.2954162	0.475	0.588	0.00980297	Macrophage:	Cd37
7.04E-07	0.26020699	0.232	0.088	0.00996596	Macrophage:	Fbxw11
7.35E-07	-0.6002538	0.96	0.847	0.01040636	Macrophage:	Rpl10a
7.76E-07	0.43916045	0.697	0.407	0.01098793	Macrophage:	Brd2
7.95E-07	0.34056552	0.758	0.465	0.01125303	Macrophage:	Snx3
8.03E-07	0.32341935	0.202	0.072	0.01136615	Macrophage:	Smpdl3b
8.07E-07	0.30238334	0.808	0.525	0.01142263	Macrophage:	Sat1
8.13E-07	-0.7654277	0.828	0.764	0.01151048	Macrophage:	Arpc3
8.50E-07	0.29974268	0.657	0.356	0.012032	Macrophage:	Dbi
8.56E-07	0.28826259	0.152	0.047	0.01211158	Macrophage:	Tmem106c
8.88E-07	-1.0676691	0.081	0.303	0.01257277	Macrophage:	4930523C07I
8.89E-07	0.46910906	0.172	0.057	0.01259016	Macrophage:	Tifab
9.31E-07	-1.3990922	0.01	0.215	0.01318335	Macrophage:	Cxcr6
9.47E-07	0.25810112	0.121	0.032	0.01340597	Macrophage:	Wdfy2
9.94E-07	0.28202685	0.202	0.072	0.01406842	Macrophage:	Orai3
1.01E-06	0.32148223	0.323	0.146	0.0143263	Macrophage:	Ube2r2
1.01E-06	-1.2208287	0.182	0.417	0.01435288	Macrophage:	Cd69
1.15E-06	0.25926834	0.172	0.056	0.01625935	Macrophage:	Abl2
1.21E-06	-0.9897736	0.04	0.248	0.0171437	Macrophage:	Cyfip2
1.28E-06	-1.1725075	0.212	0.418	0.01806959	Macrophage:	Rps27rt
1.36E-06	0.3954223	0.242	0.098	0.01929216	Macrophage:	Aldh9a1
1.40E-06	0.30584416	0.172	0.057	0.01988159	Macrophage:	Lman2l
1.41E-06	-0.5593996	0.97	0.891	0.01999762	Macrophage:	Rpl39
1.45E-06	0.37404119	0.424	0.213	0.02051089	Macrophage:	Xbp1
1.50E-06	-0.5443528	1	0.923	0.02128921	Macrophage:	B2m
1.51E-06	-1.0149012	0.02	0.22	0.02140364	Macrophage:	Rnf125
1.61E-06	0.47930622	0.869	0.548	0.02276607	Macrophage:	Tagln2
1.61E-06	-0.9186236	0.96	0.922	0.02283783	Macrophage:	Gapdh
1.62E-06	0.25044483	0.172	0.058	0.02287151	Macrophage:	Apaf1
1.62E-06	-1.6189561	0	0.191	0.02289463	Macrophage:	Ifng
2.00E-06	0.34617392	0.182	0.063	0.02825946	Macrophage:	Gpr137b
2.01E-06	0.26095781	0.677	0.405	0.02841007	Macrophage:	Bri3
2.01E-06	0.26250036	0.273	0.116	0.02850356	Macrophage:	Dctn5
2.12E-06	0.27473883	0.313	0.14	0.02999579	Macrophage:	Stt3a
2.12E-06	0.36035472	0.909	0.686	0.03007906	Macrophage:	Lcp1
2.16E-06	0.3375875	0.283	0.121	0.03057058	Macrophage:	Nabp1
2.26E-06	0.25695528	0.121	0.034	0.03192467	Macrophage:	Pvt1
2.30E-06	0.272674	0.232	0.089	0.03261698	Macrophage:	Dnajb4
2.39E-06	0.27651786	0.131	0.039	0.03380885	Macrophage:	Sh3pxd2b
2.43E-06	0.28954421	0.111	0.03	0.03438375	Macrophage:	Smad1
2.53E-06	0.35082185	0.253	0.105	0.03575631	Macrophage:	Trim8
2.60E-06	0.27627335	0.253	0.106	0.03686911	Macrophage:	Mdfic
2.62E-06	0.3069692	0.323	0.149	0.03712197	Macrophage:	Casp8
2.62E-06	0.25059171	0.394	0.198	0.03712328	Macrophage:	Hpcal1
2.67E-06	0.36447674	0.485	0.255	0.0377823	Macrophage:	Neurl3
2.77E-06	-0.6762063	0.848	0.779	0.03920999	Macrophage:	Rps6

2.98E-06	-0.9353016	0	0.183	0.04216978	Macrophage:	Serpina3g
3.01E-06	-1.162086	0.04	0.235	0.04266565	Macrophage:	Cd27
3.05E-06	0.25952279	0.273	0.117	0.04311609	Macrophage:	Slc43a2
3.09E-06	-0.5693368	0.96	0.888	0.04379356	Macrophage:	Rpl21
3.26E-06	-0.7960694	0.778	0.721	0.04610376	Macrophage:	Rbm3
3.26E-06	0.31254531	0.333	0.158	0.04620842	Macrophage:	Sh3bp1
3.33E-06	0.51671564	0.646	0.383	0.04713944	Macrophage:	Arf4
3.36E-06	0.26512868	0.172	0.059	0.04751003	Macrophage:	Dennd4b
3.76E-06	-1.7339593	0.02	0.212	0.0532399	Macrophage:	Ly6c2
3.77E-06	-1.1518352	0.061	0.263	0.05342549	Macrophage:	Itk
3.92E-06	1.04116531	0.343	0.174	0.05542959	Macrophage:	Gadd45g
4.06E-06	-1.2495181	0.081	0.28	0.05750852	Macrophage:	Sell
4.12E-06	0.29894674	0.283	0.124	0.05835786	Macrophage:	Fam111a
4.12E-06	0.25795304	0.404	0.202	0.05836933	Macrophage:	Sptssa
4.26E-06	0.27357019	0.909	0.58	0.06026179	Macrophage:	Pcbp2
4.27E-06	0.29594966	0.616	0.355	0.06041427	Macrophage:	Hcls1
4.35E-06	0.29031677	0.273	0.119	0.06153894	Macrophage:	Dlgap4
4.39E-06	0.31796377	0.182	0.067	0.06214143	Macrophage:	Batf3
4.42E-06	0.41709342	0.889	0.617	0.06255133	Macrophage:	Clta
4.43E-06	0.35400206	0.182	0.066	0.06268467	Macrophage:	B4galt3
4.53E-06	0.35364084	0.273	0.122	0.06412677	Macrophage:	Fibp
4.58E-06	0.34536198	0.283	0.128	0.06488273	Macrophage:	Rnf19b
4.63E-06	0.25755873	0.111	0.03	0.06555312	Macrophage:	Ank
4.92E-06	-1.302095	0	0.176	0.06969663	Macrophage:	Prf1
4.95E-06	0.33301806	0.444	0.234	0.07009522	Macrophage:	Ehd1
4.95E-06	0.34104327	0.798	0.527	0.07011677	Macrophage:	Rac1
5.00E-06	-2.7573125	0.061	0.248	0.07075495	Macrophage:	Cd79a
5.10E-06	0.28850709	0.263	0.115	0.07213488	Macrophage:	Pgrmc1
5.14E-06	0.32407047	0.293	0.134	0.07281571	Macrophage:	Aars
5.42E-06	-1.1142969	0.212	0.41	0.07675979	Macrophage:	H2-Q6
5.69E-06	0.30671749	0.424	0.217	0.08052872	Macrophage:	Srsf6
5.70E-06	0.38307442	0.889	0.656	0.08063502	Macrophage:	Rhoa
5.93E-06	0.29332946	0.202	0.078	0.08397602	Macrophage:	Stx6
5.98E-06	0.25097996	0.141	0.045	0.08467107	Macrophage:	Trim41
5.98E-06	-0.9151467	0.04	0.24	0.08471727	Macrophage:	P2ry10
6.18E-06	0.31910394	0.303	0.143	0.08749872	Macrophage:	Blvra
6.42E-06	-0.9793678	0.081	0.279	0.09086496	Macrophage:	Isy1
6.46E-06	0.3261342	0.828	0.559	0.09138838	Macrophage:	Rap1b
6.92E-06	-1.1293955	0.525	0.575	0.09800397	Macrophage:	Psme2
7.40E-06	-0.4846725	0.99	0.909	0.10481554	Macrophage:	Rpl37a
7.44E-06	0.42745871	0.131	0.042	0.10529813	Macrophage:	Phf11d
7.75E-06	-1.0307629	0.081	0.272	0.10974753	Macrophage:	Rasgrp2
7.80E-06	-0.485013	1	0.907	0.11038847	Macrophage:	Ppia
7.85E-06	-0.8837406	0.071	0.259	0.1111803	Macrophage:	1110008P14F
7.87E-06	0.25200036	0.263	0.115	0.11135692	Macrophage:	Hsd17b12
8.28E-06	-0.998854	0.01	0.184	0.11725243	Macrophage:	Tox
8.32E-06	0.29147338	0.364	0.185	0.11774764	Macrophage:	Necap2
8.36E-06	0.38617361	0.212	0.086	0.11832756	Macrophage:	Insig1

8.84E-06	-1.0939571	0	0.168	0.12515646	Macrophage:	Tnfrsf9
9.00E-06	-1.0033673	0.071	0.255	0.12736665	Macrophage:	Zgpat
9.14E-06	-0.7979304	0.01	0.183	0.12934604	Macrophage:	Acap1
9.19E-06	0.45571825	0.596	0.36	0.13007114	Macrophage:	Bcl2a1b
9.44E-06	-1.010192	0.545	0.578	0.13361993	Macrophage:	Cox5a
9.52E-06	0.32996038	0.162	0.057	0.13469812	Macrophage:	Nbeal1
9.56E-06	-1.1697803	0.061	0.245	0.13537237	Macrophage:	Cd28
1.02E-05	0.35468416	0.172	0.063	0.14484161	Macrophage:	Plekha1
1.07E-05	0.43990716	0.646	0.403	0.15144308	Macrophage:	Txnip
1.08E-05	-0.8149466	0.828	0.755	0.15303896	Macrophage:	Rpl12
1.13E-05	0.42240823	0.333	0.165	0.15954958	Macrophage:	Rraga
1.16E-05	0.25138464	0.99	0.907	0.16485659	Macrophage:	mt-Atp8
1.16E-05	-1.4092635	0.01	0.178	0.1648855	Macrophage:	Cd7
1.17E-05	0.33463757	0.838	0.562	0.16558418	Macrophage:	Gnai2
1.23E-05	-2.3673346	0.04	0.215	0.17392385	Macrophage:	Ly6d
1.23E-05	-0.5138005	0.909	0.82	0.17397446	Macrophage:	Rpl22
1.30E-05	-0.8841746	0.081	0.276	0.18467224	Macrophage:	Sik1
1.33E-05	0.30534791	0.374	0.198	0.18795097	Macrophage:	Tmco1
1.39E-05	-0.9289009	0	0.162	0.19646917	Macrophage:	Klrk1
1.40E-05	-0.8321361	0.04	0.224	0.19863432	Macrophage:	Cd247
1.46E-05	0.25254999	0.303	0.147	0.20609856	Macrophage:	Lamtor3
1.50E-05	0.27885421	0.202	0.081	0.21272026	Macrophage:	Bcl2a1a
1.62E-05	-0.5009678	0.919	0.844	0.22983382	Macrophage:	Rpl3
1.63E-05	0.37551068	0.869	0.637	0.23028776	Macrophage:	Actr3
1.69E-05	-1.4353248	0	0.159	0.23867399	Macrophage:	Tnfrsf4
1.84E-05	0.2919217	0.323	0.162	0.26009129	Macrophage:	Dync1i2
1.84E-05	0.31600253	0.333	0.166	0.26083998	Macrophage:	Aph1a
1.86E-05	0.27612171	0.96	0.803	0.2637515	Macrophage:	Laptm5
2.00E-05	-1.0256458	0.263	0.453	0.28284854	Macrophage:	Dusp5
2.02E-05	-1.8312344	0.051	0.223	0.28649217	Macrophage:	Ebf1
2.04E-05	0.29540074	0.758	0.507	0.28885452	Macrophage:	Cotl1
2.05E-05	-0.883191	0.727	0.659	0.2904245	Macrophage:	Psme1
2.13E-05	0.29682395	0.394	0.216	0.30169524	Macrophage:	Twf2
2.15E-05	-0.7828302	0.01	0.171	0.30497529	Macrophage:	Zap70
2.15E-05	0.2669931	0.333	0.167	0.30498868	Macrophage:	Stx4a
2.24E-05	0.28690993	0.172	0.066	0.31750195	Macrophage:	Tmem115
2.41E-05	-0.718488	0.97	0.86	0.34130532	Macrophage:	H3f3a
2.50E-05	0.2609906	0.212	0.089	0.35341637	Macrophage:	Anapc2
2.54E-05	0.86532527	0.828	0.636	0.35965134	Macrophage:	Vim
2.57E-05	0.38190178	0.96	0.815	0.36418534	Macrophage:	Ddx5
2.72E-05	0.27547307	0.152	0.055	0.38457901	Macrophage:	Vps9d1
2.73E-05	0.32964496	1	0.974	0.38628807	Macrophage:	Ubb
2.85E-05	-0.835465	0.141	0.326	0.40333071	Macrophage:	Sys1
2.87E-05	-3.4846863	0.03	0.194	0.40659884	Macrophage:	Gzma
2.94E-05	0.27803338	0.141	0.049	0.41618445	Macrophage:	Pde12
3.14E-05	0.25049258	0.263	0.123	0.44481753	Macrophage:	Slc39a7
3.18E-05	-0.8293187	0	0.15	0.45082705	Macrophage:	Cd5
3.27E-05	-0.8870369	0.03	0.192	0.46330812	Macrophage:	Stat4

3.30E-05	0.28837971	0.313	0.156	0.46742675	Macrophage:	Ddit3
3.30E-05	-0.9832894	0.333	0.453	0.46751207	Macrophage:	Erh
3.38E-05	-0.3808981	0.97	0.945	0.47872912	Macrophage:	Eif1
3.38E-05	-1.9464103	0.111	0.283	0.47917315	Macrophage:	Ccr7
3.44E-05	-0.7427765	0.02	0.181	0.48719382	Macrophage:	Pkp3
3.45E-05	0.54222738	0.909	0.718	0.48867565	Macrophage:	Crip1
3.78E-05	-0.8399278	0.04	0.201	0.53565787	Macrophage:	Acot7
3.93E-05	-1.0750931	0.061	0.234	0.55633408	Macrophage:	Gem
3.95E-05	0.45381702	0.394	0.223	0.55925242	Macrophage:	Glipr1
4.16E-05	-0.84364	0.071	0.241	0.58827279	Macrophage:	Tbc1d10c
4.20E-05	0.25328969	0.172	0.067	0.59409648	Macrophage:	Gmppa
4.33E-05	0.34244395	0.253	0.116	0.61269916	Macrophage:	4933434E20I
4.60E-05	0.27605469	0.313	0.161	0.65143069	Macrophage:	Nucb1
4.67E-05	0.35363134	0.172	0.068	0.6611463	Macrophage:	Vav3
4.67E-05	0.31919089	0.162	0.064	0.66148019	Macrophage:	Gipc1
4.78E-05	0.25132807	0.758	0.479	0.67657402	Macrophage:	Arf1
4.93E-05	-1.3134923	0.02	0.172	0.69747755	Macrophage:	Ms4a1
5.27E-05	-1.7341142	0	0.143	0.74629006	Macrophage:	Cxcl9
5.86E-05	-0.8336923	0.03	0.189	0.82960664	Macrophage:	Gramd3
6.04E-05	0.26273466	0.172	0.069	0.85533326	Macrophage:	Rasgrp4
6.30E-05	-0.7160256	0.051	0.214	0.89164283	Macrophage:	6-Sep
7.03E-05	-0.8571035	0.404	0.514	0.99585901	Macrophage:	Cycs
7.18E-05	0.66928912	0.828	0.739	1	Macrophage:	Ier2
7.36E-05	0.4373996	0.99	0.96	1	Macrophage:	H3f3b
7.52E-05	-0.506177	0.98	0.893	1	Macrophage:	Rps21
7.80E-05	0.30579859	0.394	0.215	1	Macrophage:	Tob1
8.01E-05	-0.9228725	0.091	0.25	1	Macrophage:	Rabgap1l
8.27E-05	-0.7372861	0.051	0.212	1	Macrophage:	Hopx
9.28E-05	0.25037195	0.374	0.204	1	Macrophage:	Uba1
0.00010163	-0.805865	0.97	0.904	1	Macrophage:	Rps27
0.00010243	-0.4446852	0.929	0.835	1	Macrophage:	Rpl7
0.00010514	-1.0881945	0.606	0.603	1	Macrophage:	Arl6ip1
0.00010587	-0.8284891	0.505	0.551	1	Macrophage:	Tma7
0.00010693	-0.4298548	0.99	0.864	1	Macrophage:	Rpl35
0.0001113	0.39925108	0.232	0.107	1	Macrophage:	Emilin2
0.000118	-0.7674269	0	0.131	1	Macrophage:	Izumo1r
0.00011885	-1.0048378	0.111	0.267	1	Macrophage:	Ptpn22
0.00011901	-0.8740808	0.071	0.227	1	Macrophage:	Dut
0.00012188	0.29179586	0.152	0.06	1	Macrophage:	Rnft1
0.00012962	-0.9038013	0.475	0.517	1	Macrophage:	Psmb3
0.00015224	-0.8307335	0.081	0.239	1	Macrophage:	Prkca
0.00015721	-0.5816696	0	0.127	1	Macrophage:	Gbp4
0.00017885	-0.8859223	0	0.125	1	Macrophage:	Gzmk
0.00018017	-0.3890627	0.97	0.851	1	Macrophage:	Rpl26
0.00018556	-0.8176246	0.707	0.627	1	Macrophage:	Atp5d
0.00018725	-0.6238067	0.01	0.141	1	Macrophage:	Sh2d1a
0.00019181	-1.0105718	0.02	0.154	1	Macrophage:	Cd19
0.00019614	0.25464292	0.162	0.066	1	Macrophage:	Sirt1

0.00020755	-1.0764661	0.111	0.266	1	Macrophage:	Bhlhe40
0.00022183	0.2525168	0.212	0.1	1	Macrophage:	Arhgap1
0.0002276	-0.9116097	0.374	0.461	1	Macrophage:	Gabarapl2
0.00023241	-0.6752778	0.02	0.153	1	Macrophage:	Lamb3
0.00023334	-0.7569945	0	0.121	1	Macrophage:	Rgs16
0.00023735	-1.1730347	0.091	0.249	1	Macrophage:	Lmnb1
0.00025439	-0.7540216	0.01	0.136	1	Macrophage:	Fasl
0.00025801	-0.5630148	0	0.12	1	Macrophage:	Ppp1r16b
0.00029158	-1.0092435	0.404	0.505	1	Macrophage:	Cdk2ap2
0.0002943	-0.9178102	0.071	0.214	1	Macrophage:	Gpr171
0.00029865	-0.6873989	0.01	0.134	1	Macrophage:	Cd274
0.00030356	-0.6503406	0.939	0.926	1	Macrophage:	Srgn
0.00033922	-0.7652048	0.131	0.288	1	Macrophage:	Tes
0.00034357	0.30898364	0.101	0.035	1	Macrophage:	Arl10
0.00034378	0.26526148	0.444	0.261	1	Macrophage:	Rnh1
0.00034518	-0.7170331	0.697	0.676	1	Macrophage:	Npm1
0.00034901	-0.41327	0.96	0.889	1	Macrophage:	Cfl1
0.00036793	-0.3746889	0.96	0.881	1	Macrophage:	Rpl28
0.00036947	-0.5539507	0.02	0.147	1	Macrophage:	Spint2
0.00037243	-1.0943745	0.04	0.177	1	Macrophage:	Fcmr
0.00039055	-0.7300425	0.051	0.185	1	Macrophage:	Gimap9
0.00039198	0.32016976	0.212	0.102	1	Macrophage:	Tet3
0.00041507	-0.603223	0.01	0.129	1	Macrophage:	Gm19585
0.00043402	-1.0653665	0.071	0.21	1	Macrophage:	H2-Ob
0.00043499	-1.6308884	0.111	0.249	1	Macrophage:	Cd79b
0.00043818	-0.6445629	0.848	0.699	1	Macrophage:	Uqcrh
0.00044297	-0.8191463	0.091	0.228	1	Macrophage:	D16Ertd472e
0.00044912	-0.5160756	0.01	0.128	1	Macrophage:	Mgst2
0.00047228	-0.5804125	0.02	0.142	1	Macrophage:	Hsd11b1
0.00048146	-0.9340385	0.384	0.49	1	Macrophage:	Ezr
0.00048358	-0.9460384	0.222	0.353	1	Macrophage:	Crem
0.00049099	-0.521079	0	0.11	1	Macrophage:	Hip1r
0.0004916	-1.3524228	0.071	0.203	1	Macrophage:	Ctla2a
0.00050637	-0.6956897	0.717	0.641	1	Macrophage:	Cox6c
0.00050899	-0.6796202	0	0.109	1	Macrophage:	Ccr8
0.00053598	-0.608013	0.586	0.642	1	Macrophage:	S100a10
0.00053678	-0.5703148	0.02	0.139	1	Macrophage:	Rhof
0.00054385	-0.8612695	0.343	0.435	1	Macrophage:	Csnk2b
0.000561	-0.4666483	0.455	0.269	1	Macrophage:	Il1b
0.00056337	-0.4763251	0.98	0.863	1	Macrophage:	Rps19
0.00056743	-0.5837854	0.01	0.125	1	Macrophage:	Traf4
0.00062244	-0.9160584	0.01	0.122	1	Macrophage:	Mzb1
0.00062564	0.2694226	0.394	0.246	1	Macrophage:	Csde1
0.00064046	0.33507227	0.232	0.119	1	Macrophage:	Atp6v1h
0.00066138	-0.6202899	0.576	0.602	1	Macrophage:	Sec61b
0.00066617	-0.8383022	0	0.105	1	Macrophage:	Ly6i
0.00067097	-0.4445168	0.98	0.887	1	Macrophage:	Ptma
0.00068953	-0.8552614	0.111	0.265	1	Macrophage:	Rgcc

0.00069165	-0.4755001	0.01	0.121	1	Macrophage:	Impa2
0.00070462	0.46072111	0.192	0.093	1	Macrophage:	Slc20a1
0.00072899	-0.7694418	0.505	0.543	1	Macrophage:	Edf1
0.00073024	0.27971866	0.111	0.042	1	Macrophage:	Mroh1
0.00076393	-0.5228123	0.889	0.788	1	Macrophage:	Rpl36a
0.00080774	-0.7685135	0.354	0.457	1	Macrophage:	Tax1bp1
0.00088201	-0.7658296	0.03	0.147	1	Macrophage:	H2-Oa
0.00089381	-0.6680701	0.687	0.633	1	Macrophage:	Ppp1ca
0.00090263	-0.573061	0.909	0.799	1	Macrophage:	Myl6
0.0009482	-0.4437093	0.01	0.116	1	Macrophage:	Lime1
0.00095827	-0.7954424	0.313	0.418	1	Macrophage:	Psmb5
0.00095925	-0.6123215	0.03	0.144	1	Macrophage:	Mllt3
0.00096863	-0.6675682	0	0.1	1	Macrophage:	Klrc1
0.00101156	-0.5274559	0.808	0.715	1	Macrophage:	Pfdn5
0.00102801	-0.980241	0.152	0.299	1	Macrophage:	Mxd1
0.00103377	-0.8528841	0.192	0.323	1	Macrophage:	Rapgef6
0.0010421	-0.4787386	0.99	0.857	1	Macrophage:	Rpl17
0.00104393	-0.7679291	0.323	0.468	1	Macrophage:	Dusp2
0.00105401	-0.784589	0.465	0.519	1	Macrophage:	H2afj
0.00107313	0.6140758	0.131	0.057	1	Macrophage:	Tnip3
0.00107713	-0.7167606	0.01	0.114	1	Macrophage:	Serpinb6b
0.00108127	-0.9325708	0.414	0.489	1	Macrophage:	Mif
0.00110551	-0.7529908	0.01	0.114	1	Macrophage:	Ikzf2
0.00113386	-0.5446842	0.01	0.115	1	Macrophage:	Cd226
0.00114654	-0.6581271	0.081	0.206	1	Macrophage:	Dnajc9
0.00116759	-0.4365148	0.899	0.757	1	Macrophage:	Atp5e
0.00119561	-4.3273954	0.04	0.156	1	Macrophage:	S100a8
0.00119986	-0.7445336	0.182	0.31	1	Macrophage:	Pgk1
0.00121172	-1.046698	0.071	0.188	1	Macrophage:	Cd55
0.00122688	-0.8692931	0.131	0.264	1	Macrophage:	Socs1
0.00123804	-0.7420227	0.242	0.367	1	Macrophage:	H2-T22
0.00129129	-0.544547	0.01	0.111	1	Macrophage:	Tbx21
0.00129651	-1.6774216	0.01	0.112	1	Macrophage:	Ifitm1
0.00131226	-4.5526494	0.051	0.168	1	Macrophage:	S100a9
0.00132447	-0.7170325	0.111	0.236	1	Macrophage:	9-Sep
0.00134762	-0.5070397	0.808	0.754	1	Macrophage:	Rpl5
0.00136752	-0.7284754	0.01	0.11	1	Macrophage:	Scd1
0.00142776	-0.9314891	0.081	0.206	1	Macrophage:	Dgat1
0.00145943	-0.7913734	0.646	0.615	1	Macrophage:	Ldha
0.00147303	-0.6894075	0.475	0.517	1	Macrophage:	Psmb1
0.00147627	-0.6580403	0.111	0.24	1	Macrophage:	Atg101
0.00150249	-0.2608302	0.515	0.314	1	Macrophage:	Irf8
0.00153803	-1.4065465	0.121	0.237	1	Macrophage:	H2-DMb2
0.00160871	0.29247241	0.162	0.078	1	Macrophage:	Tor3a
0.00168288	-0.6070826	0.04	0.152	1	Macrophage:	S1pr4
0.00169489	0.27882181	0.566	0.399	1	Macrophage:	Slfn2
0.00171528	-0.3586534	0.636	0.375	1	Macrophage:	Ifi30
0.00175392	-0.4925019	0.02	0.123	1	Macrophage:	Sla2

0.00179749	-0.7570573	0.424	0.482	1	Macrophage:	Rbx1
0.00179823	-0.7597514	0.636	0.581	1	Macrophage:	Ndufa13
0.00180348	-0.9259377	0.515	0.541	1	Macrophage:	Prr13
0.00182419	-0.6709386	0.242	0.364	1	Macrophage:	Polr2l
0.00186923	-0.3979136	0.02	0.122	1	Macrophage:	Rpp25l
0.00187524	-0.8661053	0.101	0.221	1	Macrophage:	Sorl1
0.00189169	-0.7164327	0.374	0.453	1	Macrophage:	Nop10
0.00191536	-0.6025592	0.03	0.135	1	Macrophage:	Arap2
0.00196023	-0.5332982	0.03	0.135	1	Macrophage:	Fam102a
0.00199738	-0.7908257	0.02	0.119	1	Macrophage:	Fcrla
0.00207045	0.69249234	0.222	0.118	1	Macrophage:	Irgm1
0.0020914	-0.6025346	0.04	0.148	1	Macrophage:	Ccdc88c
0.00211981	-0.6144339	0.636	0.628	1	Macrophage:	Ubl5
0.00217743	0.32652808	0.394	0.252	1	Macrophage:	Gpr65
0.00220257	-0.6836861	0.091	0.215	1	Macrophage:	Traf1
0.00220308	-0.5442317	0.01	0.103	1	Macrophage:	Sytl3
0.00222458	-0.6476265	0.667	0.602	1	Macrophage:	Eif3k
0.00222755	0.3926026	0.283	0.16	1	Macrophage:	Sgk1
0.00224114	-1.1304461	0.172	0.302	1	Macrophage:	Isg15
0.0023023	-0.649154	0.162	0.291	1	Macrophage:	Acp5
0.00231475	-1.0001927	0.293	0.388	1	Macrophage:	Itga4
0.00235156	-0.6774722	0.737	0.642	1	Macrophage:	Sumo2
0.0023585	-0.9394939	0.616	0.619	1	Macrophage:	Stk17b
0.00237265	-0.6841346	0.717	0.668	1	Macrophage:	Aldoa
0.00240158	-0.4753501	0.919	0.753	1	Macrophage:	Rpl9
0.00240894	0.30236418	0.121	0.053	1	Macrophage:	Gart
0.00247358	-0.7948089	0.374	0.44	1	Macrophage:	Krtcap2
0.00248589	-0.6596507	0.051	0.158	1	Macrophage:	Gpr18
0.00249363	-0.7075746	0.444	0.494	1	Macrophage:	Sri
0.00256565	-0.7807851	0.253	0.348	1	Macrophage:	Higd1a
0.00258102	-0.7680354	0.455	0.48	1	Macrophage:	Psma2
0.00258432	-0.6949212	0.01	0.101	1	Macrophage:	Klre1
0.00263203	-0.6612075	0.071	0.181	1	Macrophage:	Chfr
0.00264463	-0.7500731	0.384	0.456	1	Macrophage:	Supt4a
0.00270345	-0.8275986	0.434	0.477	1	Macrophage:	Gpsm3
0.00272967	-0.5019577	0.02	0.115	1	Macrophage:	Grap2
0.00273592	-0.7689245	0.394	0.446	1	Macrophage:	Mdh2
0.00276255	-0.6545489	0.242	0.36	1	Macrophage:	Pebp1
0.00277381	-0.3933885	0.04	0.151	1	Macrophage:	Ddit4
0.00281728	-0.7089038	0.374	0.447	1	Macrophage:	Eif3i
0.00285063	-0.9486836	0.101	0.21	1	Macrophage:	Satb1
0.00286588	-0.3537737	0.01	0.1	1	Macrophage:	Panx1
0.00288107	-0.4985597	0.03	0.13	1	Macrophage:	Cers4
0.00297428	-0.9941976	0.455	0.494	1	Macrophage:	Samhd1
0.00299476	-0.5956534	0.172	0.295	1	Macrophage:	Pdcd6
0.00304467	-0.7001279	0.111	0.228	1	Macrophage:	Cblb
0.00307073	-0.4738794	0.02	0.113	1	Macrophage:	Jakmip1
0.00314808	-0.5733461	0.03	0.128	1	Macrophage:	Cd6

0.00331919	-0.6993681	0.121	0.235	1	Macrophage:	Psip1
0.00352297	-0.6279182	0.141	0.252	1	Macrophage:	Ndufs6
0.00353536	-0.8487951	0.313	0.391	1	Macrophage:	Tomm6
0.00357098	-0.6375865	0.717	0.631	1	Macrophage:	Eif3h
0.00358919	-0.9522869	0.253	0.352	1	Macrophage:	Stat1
0.00366589	-0.7097062	0.889	0.734	1	Macrophage:	Fxyd5
0.00367344	-0.6577724	0.404	0.476	1	Macrophage:	Srp9
0.00367974	-0.4584821	0.949	0.844	1	Macrophage:	Calm1
0.00373436	-0.9003961	0.283	0.389	1	Macrophage:	Samsn1
0.00383017	-0.3796925	0.98	0.878	1	Macrophage:	Rpl38
0.00387904	-0.5872046	0.768	0.685	1	Macrophage:	Eif5a
0.00397893	-0.4182847	0.03	0.127	1	Macrophage:	Klk8
0.00399142	-0.5260566	0.091	0.2	1	Macrophage:	Cetn2
0.00408161	-0.542879	0.101	0.215	1	Macrophage:	Srpk1
0.00421363	-0.373863	0.02	0.11	1	Macrophage:	Phgdh
0.00427049	-0.697463	0.02	0.109	1	Macrophage:	Cd300lf
0.00443514	-0.7693934	0.03	0.12	1	Macrophage:	Blk
0.00444369	-0.4481265	0.03	0.123	1	Macrophage:	Zdhhc18
0.00474987	-0.4961493	0.071	0.175	1	Macrophage:	Chchd3
0.00476122	-0.7223874	0.263	0.356	1	Macrophage:	Rp9
0.00483672	0.37743134	0.162	0.087	1	Macrophage:	Eid1
0.00486729	-0.646442	0.222	0.325	1	Macrophage:	Mettl23
0.00488123	-0.7261978	0.545	0.547	1	Macrophage:	Dad1
0.00495198	-0.6675672	0.071	0.173	1	Macrophage:	AU020206
0.00496237	-0.6739635	0.667	0.634	1	Macrophage:	Cnbp
0.00498799	-0.7182834	0.081	0.185	1	Macrophage:	Nr4a3
0.00504643	-0.7231051	0.283	0.372	1	Macrophage:	Nme1
0.00512096	-0.4946167	0.02	0.107	1	Macrophage:	Cxcr3
0.00522386	-0.7632525	0.293	0.379	1	Macrophage:	Leprotl1
0.00527436	-0.6953381	0.444	0.477	1	Macrophage:	Arpp19
0.00530941	-0.7625612	0.556	0.532	1	Macrophage:	Prelid1
0.00530991	-0.512947	0.051	0.146	1	Macrophage:	Map4k1
0.00557221	-0.4473962	0.02	0.105	1	Macrophage:	Bcl11b
0.00558859	-0.6406675	0.586	0.581	1	Macrophage:	Slc25a5
0.00560318	-0.6179339	0.727	0.626	1	Macrophage:	Atp5h
0.00568601	-0.5325067	0.02	0.105	1	Macrophage:	B3gnt5
0.00571121	-0.6923574	0.172	0.273	1	Macrophage:	Arpc5l
0.00576667	-0.6502096	0.545	0.537	1	Macrophage:	Cox7a2
0.00584325	-0.5112704	0.101	0.206	1	Macrophage:	Pin1
0.00585858	-0.883241	0.283	0.373	1	Macrophage:	Dennd4a
0.00587951	-0.5841535	0.071	0.172	1	Macrophage:	Srpk2
0.00590201	-0.6760665	0.051	0.145	1	Macrophage:	Lyst
0.00605657	-0.3148409	0.99	0.991	1	Macrophage:	Actb
0.00614104	-0.500683	0.111	0.221	1	Macrophage:	Gm9844
0.00653154	-0.6597675	0.596	0.563	1	Macrophage:	Hint1
0.00664459	-0.7501733	0.111	0.211	1	Macrophage:	Malt1
0.00664883	-0.6159817	0.182	0.285	1	Macrophage:	Mrps16
0.00670203	-0.3329984	0.929	0.842	1	Macrophage:	Serf2

0.00673077	-0.5905682	0.505	0.525	1	Macrophage:	Snrpe
0.00679171	-0.7826455	0.263	0.345	1	Macrophage:	B4galnt1
0.00693054	-0.8758746	0.424	0.463	1	Macrophage:	Txn1
0.00700295	-0.4579595	0.051	0.145	1	Macrophage:	5430416N02I
0.00704818	-0.3817114	0.04	0.131	1	Macrophage:	Xpa
0.00709251	-0.8276164	0.141	0.248	1	Macrophage:	Hilpda
0.00710121	-1.0151502	0.556	0.537	1	Macrophage:	Taldo1
0.00712605	-0.5654476	0.152	0.259	1	Macrophage:	Mrpl18
0.00715745	-0.5577654	0.03	0.117	1	Macrophage:	Hist1h1e
0.00733081	0.34300369	0.242	0.148	1	Macrophage:	Arl4c
0.00740022	-0.4369423	0.03	0.114	1	Macrophage:	Fam189b
0.0075026	0.3546504	0.495	0.359	1	Macrophage:	Hsph1
0.00774985	-0.3833417	0.02	0.1	1	Macrophage:	Rab19
0.0078062	-1.043552	0.111	0.213	1	Macrophage:	Hist1h2ap
0.00784716	-0.4679594	0.101	0.205	1	Macrophage:	Nubp1
0.00787589	-0.6337998	0.212	0.308	1	Macrophage:	Timm23
0.00793663	-0.6027591	0.667	0.605	1	Macrophage:	Arf5
0.00799708	-0.5633096	0.051	0.139	1	Macrophage:	Chchd10
0.00819732	-0.6510424	0.232	0.329	1	Macrophage:	Ppdpf
0.00821029	-0.4725387	0.03	0.113	1	Macrophage:	Cnn3
0.00824325	-0.6253266	0.212	0.308	1	Macrophage:	Mrpl54
0.00829506	-1.6305319	0.071	0.161	1	Macrophage:	Hp
0.00843598	-0.7275493	0.091	0.194	1	Macrophage:	Cd24a
0.00850119	-0.4749686	0.737	0.658	1	Macrophage:	Atp5g2
0.00855619	-0.6454151	0.212	0.306	1	Macrophage:	Fkbp3
0.00858317	-0.6346297	0.616	0.579	1	Macrophage:	Snrpg
0.00860657	-0.3098375	0.99	0.905	1	Macrophage:	Rplp1
0.00884384	-0.5171951	0.051	0.138	1	Macrophage:	Rnase6
0.0089254	-0.7289001	0.182	0.28	1	Macrophage:	Cd82
0.00895325	-0.5949304	0.414	0.471	1	Macrophage:	Sf3b6
0.00905709	-0.4520611	0.04	0.126	1	Macrophage:	Sesn3
0.00931516	-0.4682392	0.081	0.174	1	Macrophage:	Ubl7
0.0097604	-0.7705566	0.768	0.676	1	Macrophage:	Ptprc
0.00996834	-0.5903621	0.071	0.16	1	Macrophage:	Map4k2
0.00997927	-0.6749929	0.111	0.206	1	Macrophage:	Nfatc3

Rik

Rik

ik

Rik

Rik

:ik

Rik

Rik

Rik

Rik

ik

Rik

Rik

Rik

ik

Rik

Rik

ꞩik

Sor

[illegible]ik

[illegible]ik

ik

Rik

Rik

Rik

Rik

Rik

Rik

Rik

Rik

'Rik

Rik

Rik

Rik

Rik

ik

Rik

Rik

Rik

Rik

Rik

Rik

Rik

Rik

Rik

Rik

Rik

Rik

Sor

Rik

Rik

Rik

Rik

:ik

Rik

.Rik

Rik

Rik

ik

Rik

Rik

ik

Rik

Rik

ik

Rik

ik2

Rik

Rik
Rik

Rik

Rik

Rik

Rik

Rik

Rik
