# Supplementary material for: The Regulation Network of Glycerolipid Metabolism as Coregulators of Immunotherapy-Related Myocarditis
Source: Cardiovasc Ther. 2023 Jun 21;2023:8774971. doi: 10.1155/2023/8774971 (PMC10307211; doi:10.1155/2023/8774971)
Supplement: Supplementary 2 — Table S2: the pseudotime trajectory- (PTT-) related differentially expressed genes in T cell subpopulation. [file 8774971.f2.pdf]

Table S2

gene	p_val	avg_log2FC	pct.1	pct.2	p_val_adj	cluster
**Ccl5**	2.67E-110	-2.57694129	0.018	0.416	3.78E-106	T cells, CD8+, naive, stimulated
**Rps29**	9.28E-105	1.320332606	0.999	0.852	1.31E-100	T cells, CD8+, naive, stimulated
**Eef1a1**	8.78E-89	1.057047483	0.999	0.872	1.24E-84	T cells, CD8+, naive, stimulated
**Rps27**	6.69E-85	1.048338516	1	0.889	9.47E-81	T cells, CD8+, naive, stimulated
**Btg1**	6.82E-78	1.651852945	0.975	0.678	9.65E-74	T cells, CD8+, naive, stimulated
**Rpl35a**	2.71E-75	0.943064313	1	0.815	3.84E-71	T cells, CD8+, naive, stimulated
**Rps28**	1.21E-73	0.990717655	0.999	0.819	1.72E-69	T cells, CD8+, naive, stimulated
**Klf2**	1.85E-70	1.717577584	0.965	0.695	2.62E-66	T cells, CD8+, naive, stimulated
**Hspa8**	1.66E-60	1.736227339	0.961	0.701	2.36E-56	T cells, CD8+, naive, stimulated
**Jund**	2.24E-58	1.227875053	0.979	0.711	3.17E-54	T cells, CD8+, naive, stimulated
**Nr4a1**	3.08E-58	1.766411709	0.877	0.49	4.35E-54	T cells, CD8+, naive, stimulated
**Rpl38**	4.44E-56	0.823274285	0.999	0.832	6.28E-52	T cells, CD8+, naive, stimulated
**Eef2**	1.53E-52	1.004865677	0.974	0.601	2.17E-48	T cells, CD8+, naive, stimulated
**Tsc22d3**	2.05E-50	1.26214362	0.891	0.44	2.91E-46	T cells, CD8+, naive, stimulated
**Dusp2**	3.08E-48	1.215770532	0.784	0.305	4.36E-44	T cells, CD8+, naive, stimulated
**Cxcr4**	2.17E-47	1.55858789	0.629	0.144	3.07E-43	T cells, CD8+, naive, stimulated
**Serp1**	1.16E-46	1.098078432	0.889	0.456	1.65E-42	T cells, CD8+, naive, stimulated
**Junb**	9.55E-46	1.279984488	0.947	0.711	1.35E-41	T cells, CD8+, naive, stimulated
**Ier5**	3.37E-39	1.192274244	0.731	0.275	4.77E-35	T cells, CD8+, naive, stimulated
**Stk17b**	3.61E-39	0.983127329	0.868	0.463	5.1E-35	T cells, CD8+, naive, stimulated
**Pcbp2**	8.34E-38	1.023829488	0.809	0.346	1.18E-33	T cells, CD8+, naive, stimulated
**Cytip**	1.07E-37	1.035886887	0.795	0.349	1.51E-33	T cells, CD8+, naive, stimulated
**Hnrnpf**	4.54E-37	1.136607794	0.737	0.309	6.43E-33	T cells, CD8+, naive, stimulated
**Dazap2**	4.89E-36	1.135560836	0.698	0.285	6.93E-32	T cells, CD8+, naive, stimulated
**Ccr7**	1.59E-35	0.986347051	0.9	0.574	2.26E-31	T cells, CD8+, naive, stimulated
**Rap1b**	6.45E-35	1.044720354	0.7	0.268	9.13E-31	T cells, CD8+, naive, stimulated
**Fam107b**	8.83E-35	1.151250057	0.729	0.326	1.25E-30	T cells, CD8+, naive, stimulated
**Pabpc1**	9.48E-35	0.885346916	0.885	0.47	1.34E-30	T cells, CD8+, naive, stimulated
**Ywhaz**	1.21E-34	0.925518621	0.866	0.443	1.72E-30	T cells, CD8+, naive, stimulated
**Rhob**	2.19E-34	1.56832249	0.547	0.151	3.1E-30	T cells, CD8+, naive, stimulated
**Rps27rt**	7.8E-34	-1.706681342	0.29	0.56	1.1E-29	T cells, CD8+, naive, stimulated
**Hspa1a**	3.85E-33	2.05564667	0.599	0.255	5.46E-29	T cells, CD8+, naive, stimulated

**Actg1**	7.58E-33	0.816803539	0.956	0.628	1.07E-28	T cells, CD8+, naive, stimulated
**H3f3b**	1.02E-31	0.658988562	0.996	0.93	1.44E-27	T cells, CD8+, naive, stimulated
**Zfp36**	1.12E-30	0.970859392	0.81	0.413	1.58E-26	T cells, CD8+, naive, stimulated
**Dnajb1**	4.34E-30	1.782699779	0.717	0.393	6.14E-26	T cells, CD8+, naive, stimulated
**Gnas**	8.22E-30	0.93124452	0.768	0.383	1.16E-25	T cells, CD8+, naive, stimulated
**Dusp1**	2.42E-29	1.167959951	0.581	0.191	3.43E-25	T cells, CD8+, naive, stimulated
**Fcer2a**	2.9E-29	1.00936175	0.609	0.198	4.11E-25	T cells, CD8+, naive, stimulated
**Cnbp**	3.07E-29	0.781639534	0.861	0.466	4.35E-25	T cells, CD8+, naive, stimulated
**Rpl17**	2.27E-28	0.5711111	0.989	0.822	3.22E-24	T cells, CD8+, naive, stimulated
**Zfp36l2**	3.95E-28	1.051677598	0.595	0.218	5.59E-24	T cells, CD8+, naive, stimulated
**Serinc3**	4.55E-28	0.832250841	0.804	0.393	6.45E-24	T cells, CD8+, naive, stimulated
**mt-Co2**	7.65E-28	-0.939430346	0.999	0.997	1.08E-23	T cells, CD8+, naive, stimulated
**Ets1**	2.06E-27	0.616093613	0.787	0.376	2.92E-23	T cells, CD8+, naive, stimulated
**mt-Nd3**	2.44E-27	0.547278595	0.638	0.242	3.46E-23	T cells, CD8+, naive, stimulated
**S100a6**	2.76E-26	-2.3609815	0.051	0.228	3.91E-22	T cells, CD8+, naive, stimulated
**Snx5**	8.25E-26	0.846302781	0.68	0.292	1.17E-21	T cells, CD8+, naive, stimulated
**Pnrc1**	3.17E-25	0.758882531	0.832	0.507	4.49E-21	T cells, CD8+, naive, stimulated
**Rps21**	3.35E-25	0.492726622	0.999	0.872	4.74E-21	T cells, CD8+, naive, stimulated
**Hnrnpk**	4.86E-25	0.765541753	0.68	0.305	6.88E-21	T cells, CD8+, naive, stimulated
**Ptp4a3**	5.33E-25	0.895973202	0.577	0.211	7.54E-21	T cells, CD8+, naive, stimulated
**Tcp11l2**	2.54E-24	1.054606047	0.527	0.185	3.6E-20	T cells, CD8+, naive, stimulated
**mt-Co3**	7.82E-24	-0.956574842	0.999	0.993	1.11E-19	T cells, CD8+, naive, stimulated
**Ubc**	8.87E-24	0.770216163	0.805	0.473	1.26E-19	T cells, CD8+, naive, stimulated
**Arf6**	2.08E-23	0.784339002	0.648	0.279	2.95E-19	T cells, CD8+, naive, stimulated
**Tgfb1**	2.22E-23	0.795825552	0.625	0.272	3.14E-19	T cells, CD8+, naive, stimulated
**Hsph1**	2.37E-23	0.908964662	0.577	0.245	3.36E-19	T cells, CD8+, naive, stimulated
**Ddx5**	3.05E-23	0.452755171	0.965	0.698	4.32E-19	T cells, CD8+, naive, stimulated
**Ezr**	3.64E-23	0.678463216	0.741	0.366	5.15E-19	T cells, CD8+, naive, stimulated
**Vps37b**	6.56E-23	0.851358334	0.692	0.346	9.28E-19	T cells, CD8+, naive, stimulated
**Rhoa**	1.09E-22	0.669972584	0.82	0.443	1.54E-18	T cells, CD8+, naive, stimulated
**P2ry10**	1.61E-22	0.878483585	0.521	0.191	2.28E-18	T cells, CD8+, naive, stimulated
**Klf4**	1.76E-22	1.299928769	0.498	0.188	2.48E-18	T cells, CD8+, naive, stimulated
**Ifi30**	2.02E-22	-1.473568177	0.376	0.581	2.86E-18	T cells, CD8+, naive, stimulated
**Klf6**	6.1E-22	0.799691059	0.712	0.366	8.64E-18	T cells, CD8+, naive, stimulated
**Rplp0**	1.68E-21	-0.604433982	0.994	0.876	2.38E-17	T cells, CD8+, naive, stimulated

**Lgals1**	2.65E-21	-0.999000253	0.049	0.205	3.76E-17	T cells, CD8+, naive, stimulated
**Plk2**	2.81E-21	1.279021761	0.358	0.07	3.98E-17	T cells, CD8+, naive, stimulated
**Cfap43**	4.31E-21	0.855647886	0.316	0.044	6.1E-17	T cells, CD8+, naive, stimulated
**Hspa1b**	7.9E-21	1.036109668	0.617	0.326	1.12E-16	T cells, CD8+, naive, stimulated
**Rpsa**	1.03E-20	-0.452577192	0.997	0.906	1.46E-16	T cells, CD8+, naive, stimulated
**Add3**	1.26E-20	0.716527062	0.498	0.178	1.78E-16	T cells, CD8+, naive, stimulated
**Tonsl**	1.81E-20	1.040761314	0.345	0.087	2.56E-16	T cells, CD8+, naive, stimulated
**Pcbp1**	3.37E-20	0.751012974	0.662	0.336	4.77E-16	T cells, CD8+, naive, stimulated
**Bach2**	1.46E-19	0.541265186	0.476	0.154	2.06E-15	T cells, CD8+, naive, stimulated
**Cnn3**	1.47E-19	1.031767429	0.309	0.057	2.08E-15	T cells, CD8+, naive, stimulated
**Csk**	2.24E-19	0.742361219	0.49	0.191	3.17E-15	T cells, CD8+, naive, stimulated
**Snn**	2.33E-19	0.915428302	0.434	0.144	3.3E-15	T cells, CD8+, naive, stimulated
**Tgif1**	2.4E-19	0.867635599	0.498	0.205	3.39E-15	T cells, CD8+, naive, stimulated
**Hmgn2**	9.55E-19	0.866827449	0.514	0.221	1.35E-14	T cells, CD8+, naive, stimulated
**Ube2d3**	9.64E-19	0.640537009	0.7	0.366	1.37E-14	T cells, CD8+, naive, stimulated
**Sqstm1**	1.58E-18	0.652887275	0.638	0.312	2.24E-14	T cells, CD8+, naive, stimulated
**Anxa2**	1.87E-18	-0.636852009	0.012	0.104	2.65E-14	T cells, CD8+, naive, stimulated
**Rbm38**	2.49E-18	0.632048661	0.52	0.218	3.53E-14	T cells, CD8+, naive, stimulated
**Syk**	2.58E-18	0.42112303	0.595	0.248	3.65E-14	T cells, CD8+, naive, stimulated
**Arf1**	2.68E-18	0.547722248	0.569	0.252	3.79E-14	T cells, CD8+, naive, stimulated
**Ptp4a1**	3.82E-18	1.108329408	0.271	0.047	5.4E-14	T cells, CD8+, naive, stimulated
**Actr3**	5.27E-18	0.572023538	0.698	0.356	7.46E-14	T cells, CD8+, naive, stimulated
**Tgfbr2**	6.28E-18	0.662496872	0.42	0.148	8.88E-14	T cells, CD8+, naive, stimulated
**Gdi2**	8.84E-18	0.563579174	0.682	0.356	1.25E-13	T cells, CD8+, naive, stimulated
**Rpl4**	1.05E-17	0.574174563	0.88	0.577	1.48E-13	T cells, CD8+, naive, stimulated
**Sdcbp**	1.35E-17	0.863898313	0.379	0.121	1.91E-13	T cells, CD8+, naive, stimulated
**Hnrnpab**	1.44E-17	0.723995626	0.554	0.255	2.03E-13	T cells, CD8+, naive, stimulated
**Rpl13**	1.61E-17	-0.350806064	0.999	0.906	2.28E-13	T cells, CD8+, naive, stimulated
**Cd37**	1.83E-17	-0.787769815	0.875	0.836	2.59E-13	T cells, CD8+, naive, stimulated
**Rasgef1b**	2.1E-17	0.981727915	0.368	0.111	2.97E-13	T cells, CD8+, naive, stimulated
**Ppp1r15a**	2.67E-17	0.644593644	0.616	0.315	3.78E-13	T cells, CD8+, naive, stimulated
**Cd19**	2.98E-17	0.438266951	0.696	0.346	4.21E-13	T cells, CD8+, naive, stimulated
**Hsp90aa1**	4.08E-17	0.662960806	0.869	0.651	5.77E-13	T cells, CD8+, naive, stimulated
**Rps2**	4.48E-17	-0.461756413	0.999	0.919	6.34E-13	T cells, CD8+, naive, stimulated
**Eif4a1**	8.72E-17	0.599441915	0.589	0.268	1.23E-12	T cells, CD8+, naive, stimulated

**Pim1**	1.05E-16	0.695307439	0.532	0.248	1.49E-12	T cells, CD8+, naive, stimulated
**Gga2**	1.86E-16	0.827158479	0.318	0.081	2.64E-12	T cells, CD8+, naive, stimulated
**Cd83**	1.88E-16	0.558958625	0.761	0.456	2.66E-12	T cells, CD8+, naive, stimulated
**Dusp5**	2.38E-16	0.934984644	0.446	0.181	3.37E-12	T cells, CD8+, naive, stimulated
**Scd1**	2.59E-16	0.608785091	0.499	0.221	3.66E-12	T cells, CD8+, naive, stimulated
**Brd2**	3.84E-16	0.65983135	0.524	0.245	5.44E-12	T cells, CD8+, naive, stimulated
**Rnf187**	5.65E-16	0.827979351	0.393	0.151	7.99E-12	T cells, CD8+, naive, stimulated
**Cd79a**	1.06E-15	-0.569102668	0.994	0.899	1.5E-11	T cells, CD8+, naive, stimulated
**Sell**	1.36E-15	0.319709663	0.585	0.285	1.92E-11	T cells, CD8+, naive, stimulated
**Lamp1**	1.36E-15	0.654108639	0.389	0.141	1.92E-11	T cells, CD8+, naive, stimulated
**Cd24a**	2.31E-15	0.631493938	0.631	0.336	3.27E-11	T cells, CD8+, naive, stimulated
**Lyn**	3.81E-15	0.517402452	0.606	0.309	5.39E-11	T cells, CD8+, naive, stimulated
**Sf1**	4.96E-15	0.527520391	0.537	0.255	7.02E-11	T cells, CD8+, naive, stimulated
**Cd53**	7.29E-15	0.448159566	0.607	0.309	1.03E-10	T cells, CD8+, naive, stimulated
**Fam43a**	8.39E-15	0.773793659	0.404	0.158	1.19E-10	T cells, CD8+, naive, stimulated
**Capza1**	9.91E-15	0.719126823	0.372	0.141	1.4E-10	T cells, CD8+, naive, stimulated
**Cebpb**	1.03E-14	0.841453298	0.504	0.245	1.45E-10	T cells, CD8+, naive, stimulated
**Fam49b**	1.17E-14	0.644335443	0.456	0.208	1.66E-10	T cells, CD8+, naive, stimulated
**Slbp**	1.39E-14	0.76068523	0.384	0.151	1.97E-10	T cells, CD8+, naive, stimulated
**Rpl10**	1.75E-14	0.513557977	0.81	0.497	2.48E-10	T cells, CD8+, naive, stimulated
**Polr2a**	2.06E-14	0.501763611	0.478	0.218	2.92E-10	T cells, CD8+, naive, stimulated
**Tmed2**	3.53E-14	0.574339455	0.476	0.218	5E-10	T cells, CD8+, naive, stimulated
**Cd9**	3.58E-14	-0.574629202	0.023	0.117	5.07E-10	T cells, CD8+, naive, stimulated
**Tob1**	5.17E-14	0.661951743	0.308	0.101	7.32E-10	T cells, CD8+, naive, stimulated
**Rps4x**	5.56E-14	-0.330113173	0.999	0.872	7.86E-10	T cells, CD8+, naive, stimulated
**Srsf3**	6.23E-14	0.521757367	0.62	0.342	8.82E-10	T cells, CD8+, naive, stimulated
**Sh3bp5**	7.01E-14	0.679634436	0.391	0.154	9.92E-10	T cells, CD8+, naive, stimulated
**Foxo1**	7.82E-14	0.62973309	0.404	0.164	1.11E-09	T cells, CD8+, naive, stimulated
**Cd79b**	8.09E-14	-0.863418989	0.852	0.802	1.14E-09	T cells, CD8+, naive, stimulated
**Mcl1**	8.23E-14	0.651743366	0.52	0.262	1.17E-09	T cells, CD8+, naive, stimulated
**Oser1**	9.88E-14	0.774644583	0.328	0.121	1.4E-09	T cells, CD8+, naive, stimulated
**Pmaip1**	1.01E-13	0.959174691	0.282	0.081	1.42E-09	T cells, CD8+, naive, stimulated
**Tsc22d1**	1.37E-13	1.061566049	0.308	0.091	1.93E-09	T cells, CD8+, naive, stimulated
**Arhgef1**	1.63E-13	0.417463512	0.599	0.319	2.31E-09	T cells, CD8+, naive, stimulated
**Khdrbs1**	1.69E-13	0.5730999	0.344	0.124	2.4E-09	T cells, CD8+, naive, stimulated

**H2-D1**	2.2E-13	0.290553598	0.98	0.862	3.12E-09	T cells, CD8+, naive, stimulated
**Zfp36l1**	2.37E-13	0.40070132	0.852	0.597	3.35E-09	T cells, CD8+, naive, stimulated
**Gapdh**	2.89E-13	-0.728901109	0.886	0.792	4.09E-09	T cells, CD8+, naive, stimulated
**Crlf3**	4.68E-13	0.413262067	0.43	0.191	6.62E-09	T cells, CD8+, naive, stimulated
**Tomm6**	4.95E-13	-0.914454502	0.185	0.356	7E-09	T cells, CD8+, naive, stimulated
**Cd55**	5.56E-13	0.292379587	0.696	0.376	7.87E-09	T cells, CD8+, naive, stimulated
**Rpl39**	6.47E-13	0.347117654	0.997	0.839	9.16E-09	T cells, CD8+, naive, stimulated
**Herpud1**	6.89E-13	0.815063651	0.34	0.134	9.75E-09	T cells, CD8+, naive, stimulated
**Rpl10a**	7.47E-13	-0.437081211	0.985	0.859	1.06E-08	T cells, CD8+, naive, stimulated
**Rbm3**	7.9E-13	0.482831677	0.845	0.55	1.12E-08	T cells, CD8+, naive, stimulated
**Clic1**	8.2E-13	-0.902840778	0.57	0.631	1.16E-08	T cells, CD8+, naive, stimulated
**Sfpq**	8.63E-13	0.467768748	0.457	0.218	1.22E-08	T cells, CD8+, naive, stimulated
**Rpl11**	9.08E-13	-0.337585552	0.993	0.886	1.29E-08	T cells, CD8+, naive, stimulated
**Eif4a2**	1.16E-12	0.506604168	0.508	0.255	1.65E-08	T cells, CD8+, naive, stimulated
**Myh9**	1.22E-12	0.473541475	0.58	0.315	1.73E-08	T cells, CD8+, naive, stimulated
**Jun**	1.29E-12	0.470405018	0.545	0.295	1.83E-08	T cells, CD8+, naive, stimulated
**Cyth1**	2.25E-12	0.402089897	0.432	0.198	3.18E-08	T cells, CD8+, naive, stimulated
**Wipf1**	2.3E-12	0.635855539	0.241	0.057	3.25E-08	T cells, CD8+, naive, stimulated
**Cbx4**	2.38E-12	0.695097896	0.244	0.06	3.38E-08	T cells, CD8+, naive, stimulated
**Srsf2**	2.49E-12	0.498626387	0.541	0.292	3.53E-08	T cells, CD8+, naive, stimulated
**Cxcr5**	2.53E-12	0.554062895	0.393	0.178	3.59E-08	T cells, CD8+, naive, stimulated
**Rnf145**	2.71E-12	0.648261416	0.26	0.074	3.83E-08	T cells, CD8+, naive, stimulated
**Taf6l**	2.8E-12	0.693197407	0.272	0.087	3.96E-08	T cells, CD8+, naive, stimulated
**Mrfap1**	2.99E-12	0.610792524	0.403	0.195	4.23E-08	T cells, CD8+, naive, stimulated
**Pou2af1**	3.18E-12	0.511661372	0.386	0.161	4.5E-08	T cells, CD8+, naive, stimulated
**Rps13**	3.2E-12	0.332038789	0.999	0.856	4.52E-08	T cells, CD8+, naive, stimulated
**Snx9**	3.39E-12	0.613454328	0.395	0.178	4.8E-08	T cells, CD8+, naive, stimulated
**Prkcb**	3.53E-12	0.421078092	0.502	0.252	5E-08	T cells, CD8+, naive, stimulated
**Amd1**	3.99E-12	0.744311122	0.249	0.07	5.64E-08	T cells, CD8+, naive, stimulated
**Rpl18**	4.12E-12	-0.389882335	0.996	0.876	5.83E-08	T cells, CD8+, naive, stimulated
**Cpm**	4.13E-12	0.546737819	0.297	0.094	5.85E-08	T cells, CD8+, naive, stimulated
**Tpm4**	4.14E-12	0.501201033	0.408	0.195	5.86E-08	T cells, CD8+, naive, stimulated
**Rps8**	4.94E-12	-0.273002104	0.999	0.916	7E-08	T cells, CD8+, naive, stimulated
**Ppp1cc**	5.9E-12	0.424004863	0.412	0.191	8.35E-08	T cells, CD8+, naive, stimulated
**Cacybp**	5.91E-12	0.595492868	0.414	0.191	8.36E-08	T cells, CD8+, naive, stimulated

**Pxk**	6.12E-12	0.343461564	0.373	0.154	8.66E-08	T cells, CD8+, naive, stimulated
**Eif2ak3**	7.26E-12	0.459552484	0.322	0.114	1.03E-07	T cells, CD8+, naive, stimulated
**Eif4g2**	8.06E-12	0.278302243	0.617	0.342	1.14E-07	T cells, CD8+, naive, stimulated
**Rps3a1**	8.09E-12	-0.285306357	0.999	0.909	1.15E-07	T cells, CD8+, naive, stimulated
**Dnajb6**	9.06E-12	0.658363939	0.367	0.158	1.28E-07	T cells, CD8+, naive, stimulated
**Ncf2**	9.59E-12	0.578538823	0.334	0.134	1.36E-07	T cells, CD8+, naive, stimulated
**Tmem123**	9.81E-12	0.518664754	0.463	0.221	1.39E-07	T cells, CD8+, naive, stimulated
**Cnn2**	1.22E-11	0.572410398	0.483	0.252	1.73E-07	T cells, CD8+, naive, stimulated
**Wsb1**	1.25E-11	0.504289736	0.325	0.128	1.77E-07	T cells, CD8+, naive, stimulated
**Gpr171**	1.32E-11	0.533621903	0.385	0.168	1.86E-07	T cells, CD8+, naive, stimulated
**Hist1h2ap**	1.32E-11	0.762240619	0.327	0.131	1.86E-07	T cells, CD8+, naive, stimulated
**Fmnl1**	1.57E-11	0.494838721	0.381	0.171	2.22E-07	T cells, CD8+, naive, stimulated
**Eif3e**	1.67E-11	0.433644692	0.476	0.238	2.36E-07	T cells, CD8+, naive, stimulated
**Stk24**	1.86E-11	0.361673609	0.495	0.252	2.64E-07	T cells, CD8+, naive, stimulated
**Tubb4b**	1.92E-11	0.376459737	0.576	0.312	2.71E-07	T cells, CD8+, naive, stimulated
**Cd22**	1.93E-11	0.552839337	0.333	0.131	2.73E-07	T cells, CD8+, naive, stimulated
**Rps11**	2.34E-11	-0.371005409	0.996	0.872	3.31E-07	T cells, CD8+, naive, stimulated
**Adipor1**	2.45E-11	0.472819138	0.34	0.141	3.47E-07	T cells, CD8+, naive, stimulated
**Tuba1a**	2.56E-11	0.598578552	0.576	0.339	3.63E-07	T cells, CD8+, naive, stimulated
**Atf4**	2.8E-11	0.387070447	0.488	0.255	3.97E-07	T cells, CD8+, naive, stimulated
**Trp53i11**	2.85E-11	0.570432588	0.355	0.148	4.04E-07	T cells, CD8+, naive, stimulated
**Mknk2**	2.96E-11	0.59884253	0.31	0.124	4.19E-07	T cells, CD8+, naive, stimulated
**Rpl21**	3.01E-11	0.332809153	1	0.839	4.26E-07	T cells, CD8+, naive, stimulated
**Nrros**	3.17E-11	0.606089352	0.383	0.181	4.48E-07	T cells, CD8+, naive, stimulated
**Nrbp1**	3.23E-11	0.571171599	0.32	0.134	4.58E-07	T cells, CD8+, naive, stimulated
**Lbr**	3.9E-11	0.399256048	0.307	0.114	5.52E-07	T cells, CD8+, naive, stimulated
**Ywhaq**	4.38E-11	0.442870791	0.402	0.188	6.2E-07	T cells, CD8+, naive, stimulated
**Peli1**	4.64E-11	0.306792723	0.346	0.144	6.57E-07	T cells, CD8+, naive, stimulated
**Man1a**	4.64E-11	0.419782679	0.371	0.178	6.57E-07	T cells, CD8+, naive, stimulated
**Hnrnpl**	4.77E-11	0.460521565	0.475	0.248	6.75E-07	T cells, CD8+, naive, stimulated
**Ubald2**	5.19E-11	0.792723017	0.343	0.158	7.35E-07	T cells, CD8+, naive, stimulated
**Akap13**	5.61E-11	0.288020073	0.564	0.315	7.94E-07	T cells, CD8+, naive, stimulated
**Tagap**	5.61E-11	0.738672901	0.252	0.084	7.94E-07	T cells, CD8+, naive, stimulated
**Rps5**	5.64E-11	-0.325868938	0.998	0.883	7.98E-07	T cells, CD8+, naive, stimulated
**Rnf167**	5.76E-11	0.629784381	0.284	0.104	8.16E-07	T cells, CD8+, naive, stimulated

**S1pr1**	5.82E-11	0.415028546	0.532	0.289	8.24E-07	T cells, CD8+, naive, stimulated
**B4galnt1**	6.66E-11	0.468353851	0.415	0.201	9.43E-07	T cells, CD8+, naive, stimulated
**Unc93b1**	6.86E-11	0.391729737	0.643	0.383	9.71E-07	T cells, CD8+, naive, stimulated
**Ap2m1**	7.18E-11	0.596832914	0.33	0.144	1.02E-06	T cells, CD8+, naive, stimulated
**Calr**	7.57E-11	0.430096539	0.417	0.198	1.07E-06	T cells, CD8+, naive, stimulated
**Prrc2a**	8.01E-11	0.469299474	0.309	0.121	1.13E-06	T cells, CD8+, naive, stimulated
**Lmo2**	8.15E-11	0.557986527	0.215	0.054	1.15E-06	T cells, CD8+, naive, stimulated
**AW11201C**	8.64E-11	-1.046311547	0.077	0.191	1.22E-06	T cells, CD8+, naive, stimulated
**Dnaja1**	8.74E-11	0.445364017	0.778	0.55	1.24E-06	T cells, CD8+, naive, stimulated
**Ctbp1**	9.06E-11	0.614500145	0.312	0.134	1.28E-06	T cells, CD8+, naive, stimulated
**Mef2d**	1.02E-10	0.462688781	0.312	0.134	1.44E-06	T cells, CD8+, naive, stimulated
**Tnfaip3**	1.03E-10	0.711926072	0.33	0.144	1.45E-06	T cells, CD8+, naive, stimulated
**Hnrnpa2b**	1.14E-10	0.259820301	0.77	0.477	1.62E-06	T cells, CD8+, naive, stimulated
**Fosb**	1.22E-10	0.47611797	0.415	0.208	1.73E-06	T cells, CD8+, naive, stimulated
**Cd164**	1.24E-10	0.5661303	0.304	0.124	1.76E-06	T cells, CD8+, naive, stimulated
**Mfap1b**	1.75E-10	0.575278889	0.258	0.084	2.48E-06	T cells, CD8+, naive, stimulated
**Tprgl**	1.78E-10	0.6490128	0.287	0.114	2.52E-06	T cells, CD8+, naive, stimulated
**Ly6a**	1.86E-10	-1.23200544	0.333	0.446	2.63E-06	T cells, CD8+, naive, stimulated
**Cd74**	1.91E-10	-0.343834305	1	0.993	2.71E-06	T cells, CD8+, naive, stimulated
**Bcl10**	1.91E-10	0.498436756	0.381	0.185	2.71E-06	T cells, CD8+, naive, stimulated
**Kctd12**	2.32E-10	0.389011889	0.306	0.121	3.28E-06	T cells, CD8+, naive, stimulated
**Hnrnph2**	2.43E-10	0.466440803	0.295	0.114	3.44E-06	T cells, CD8+, naive, stimulated
**Morf4l2**	2.5E-10	0.58534065	0.239	0.077	3.54E-06	T cells, CD8+, naive, stimulated
**Rps9**	2.77E-10	-0.317939116	0.987	0.866	3.92E-06	T cells, CD8+, naive, stimulated
**Traf4**	2.95E-10	0.630808292	0.267	0.097	4.18E-06	T cells, CD8+, naive, stimulated
**Irf4**	3.21E-10	0.410249212	0.338	0.141	4.54E-06	T cells, CD8+, naive, stimulated
**Azin1**	3.44E-10	0.555452896	0.246	0.081	4.86E-06	T cells, CD8+, naive, stimulated
**Cat**	3.49E-10	0.582207688	0.23	0.07	4.94E-06	T cells, CD8+, naive, stimulated
**Ier2**	3.83E-10	0.391614031	0.837	0.607	5.42E-06	T cells, CD8+, naive, stimulated
**Hvcn1**	4.21E-10	0.43137983	0.382	0.178	5.95E-06	T cells, CD8+, naive, stimulated
**Rpl37a**	4.38E-10	0.297694941	0.995	0.899	6.2E-06	T cells, CD8+, naive, stimulated
**Eif4b**	4.94E-10	0.493289439	0.323	0.138	6.99E-06	T cells, CD8+, naive, stimulated
**Polr2m**	5.12E-10	0.436018838	0.24	0.074	7.25E-06	T cells, CD8+, naive, stimulated
**Stip1**	5.15E-10	0.375949326	0.325	0.138	7.29E-06	T cells, CD8+, naive, stimulated
**Map2k1**	6.33E-10	0.54971503	0.253	0.091	8.97E-06	T cells, CD8+, naive, stimulated

**Ptbp3**	6.62E-10	0.381121184	0.51	0.292	9.37E-06	T cells, CD8+, naive, stimulated
**Per1**	6.91E-10	0.530117601	0.338	0.154	9.78E-06	T cells, CD8+, naive, stimulated
**Ubl3**	7.08E-10	0.468335566	0.379	0.188	1E-05	T cells, CD8+, naive, stimulated
**Fkbp4**	7.43E-10	0.511724096	0.306	0.128	1.05E-05	T cells, CD8+, naive, stimulated
**Rbm7**	7.56E-10	0.598173275	0.231	0.077	1.07E-05	T cells, CD8+, naive, stimulated
**Siah2**	7.87E-10	0.605085981	0.234	0.081	1.11E-05	T cells, CD8+, naive, stimulated
**Tob2**	7.89E-10	0.48897395	0.387	0.191	1.12E-05	T cells, CD8+, naive, stimulated
**Abhd17b**	7.95E-10	0.380477556	0.403	0.205	1.13E-05	T cells, CD8+, naive, stimulated
**Ptk2b**	9.46E-10	0.366628538	0.317	0.138	1.34E-05	T cells, CD8+, naive, stimulated
**Cyba**	1.01E-09	-0.77945067	0.765	0.715	1.43E-05	T cells, CD8+, naive, stimulated
**Ubqln1**	1.02E-09	0.476335097	0.251	0.087	1.44E-05	T cells, CD8+, naive, stimulated
**Epn1**	1.2E-09	0.626768031	0.303	0.141	1.7E-05	T cells, CD8+, naive, stimulated
**Ass1**	1.24E-09	-0.376159339	0.035	0.121	1.76E-05	T cells, CD8+, naive, stimulated
**Srgn**	1.34E-09	-0.601046478	0.946	0.869	1.89E-05	T cells, CD8+, naive, stimulated
**Gem**	1.43E-09	0.31985275	0.404	0.205	2.03E-05	T cells, CD8+, naive, stimulated
**H2-T23**	1.46E-09	0.250445395	0.524	0.285	2.07E-05	T cells, CD8+, naive, stimulated
**Atp5b**	1.59E-09	0.292639782	0.529	0.302	2.25E-05	T cells, CD8+, naive, stimulated
**Elmsan1**	1.62E-09	0.456353099	0.323	0.148	2.29E-05	T cells, CD8+, naive, stimulated
**Usp38**	1.62E-09	0.55071527	0.183	0.044	2.3E-05	T cells, CD8+, naive, stimulated
**Ctnnb1**	1.64E-09	0.297221985	0.287	0.114	2.33E-05	T cells, CD8+, naive, stimulated
**Gm26532**	1.73E-09	0.337725329	0.33	0.144	2.45E-05	T cells, CD8+, naive, stimulated
**Ppp4c**	2.01E-09	0.437714771	0.41	0.218	2.84E-05	T cells, CD8+, naive, stimulated
**Gm42418**	2.04E-09	-0.325287963	0.998	1	2.89E-05	T cells, CD8+, naive, stimulated
**Gpi1**	2.12E-09	0.375175857	0.434	0.228	3E-05	T cells, CD8+, naive, stimulated
**AI467606**	2.17E-09	0.616237907	0.222	0.07	3.08E-05	T cells, CD8+, naive, stimulated
**Prkcd**	2.33E-09	0.520117961	0.287	0.121	3.29E-05	T cells, CD8+, naive, stimulated
**Wbp2**	2.6E-09	0.528468412	0.358	0.181	3.68E-05	T cells, CD8+, naive, stimulated
**Neurl3**	2.69E-09	0.293039529	0.292	0.111	3.81E-05	T cells, CD8+, naive, stimulated
**Oat**	2.74E-09	0.361734875	0.254	0.094	3.87E-05	T cells, CD8+, naive, stimulated
**Ly6e**	2.79E-09	-0.480392534	0.99	0.936	3.95E-05	T cells, CD8+, naive, stimulated
**Arhgdia**	2.84E-09	0.349593319	0.53	0.322	4.03E-05	T cells, CD8+, naive, stimulated
**Adrb2**	2.87E-09	0.488200094	0.261	0.101	4.07E-05	T cells, CD8+, naive, stimulated
**Ago2**	2.9E-09	0.427959906	0.242	0.084	4.1E-05	T cells, CD8+, naive, stimulated
**Hmgb1**	3.15E-09	0.338328787	0.717	0.453	4.46E-05	T cells, CD8+, naive, stimulated
**Itsn2**	3.18E-09	0.485092886	0.29	0.124	4.5E-05	T cells, CD8+, naive, stimulated

**Blk**	3.25E-09	0.335153484	0.532	0.299	4.6E-05	T cells, CD8+, naive, stimulated
**Ost4**	4.11E-09	0.384863118	0.552	0.336	5.81E-05	T cells, CD8+, naive, stimulated
**Tspan13**	4.14E-09	0.379135953	0.469	0.272	5.86E-05	T cells, CD8+, naive, stimulated
**Rpl8**	4.36E-09	-0.271374779	0.996	0.889	6.17E-05	T cells, CD8+, naive, stimulated
**Tram1**	4.37E-09	0.382767603	0.264	0.104	6.18E-05	T cells, CD8+, naive, stimulated
**Ccm2**	4.46E-09	0.478704804	0.291	0.124	6.31E-05	T cells, CD8+, naive, stimulated
**Blnk**	4.49E-09	0.3338876	0.493	0.285	6.35E-05	T cells, CD8+, naive, stimulated
**Ppp3ca**	4.52E-09	0.358402131	0.466	0.252	6.4E-05	T cells, CD8+, naive, stimulated
**Csrnp1**	4.55E-09	0.57709226	0.274	0.114	6.44E-05	T cells, CD8+, naive, stimulated
**Arid5a**	4.57E-09	0.4155453	0.361	0.181	6.47E-05	T cells, CD8+, naive, stimulated
**Il16**	4.79E-09	0.455104465	0.218	0.07	6.78E-05	T cells, CD8+, naive, stimulated
**Sesn3**	5.55E-09	0.383772185	0.218	0.067	7.86E-05	T cells, CD8+, naive, stimulated
**Ube2d2a**	5.85E-09	0.306036732	0.524	0.319	8.28E-05	T cells, CD8+, naive, stimulated
**Tpm3**	6.18E-09	0.266947032	0.547	0.322	8.75E-05	T cells, CD8+, naive, stimulated
**Cap1**	6.32E-09	0.41131443	0.279	0.114	8.95E-05	T cells, CD8+, naive, stimulated
**Marcksl1**	6.64E-09	0.690389346	0.332	0.158	9.4E-05	T cells, CD8+, naive, stimulated
**Vapa**	6.69E-09	0.461983871	0.35	0.178	9.47E-05	T cells, CD8+, naive, stimulated
**Spop**	7.46E-09	0.622843425	0.26	0.111	0.00010567	T cells, CD8+, naive, stimulated
**Ehd1**	8.88E-09	0.533302835	0.268	0.111	0.00012574	T cells, CD8+, naive, stimulated
**Cdc42**	8.99E-09	0.320487999	0.733	0.47	0.00012729	T cells, CD8+, naive, stimulated
**Hspe1**	9.13E-09	0.521579428	0.818	0.624	0.00012928	T cells, CD8+, naive, stimulated
**Fli1**	9.57E-09	0.3470382	0.353	0.168	0.00013549	T cells, CD8+, naive, stimulated
**Rsrp1**	1.02E-08	0.291177958	0.403	0.208	0.00014407	T cells, CD8+, naive, stimulated
**Capza2**	1.04E-08	0.456408275	0.344	0.178	0.00014666	T cells, CD8+, naive, stimulated
**Ewsr1**	1.07E-08	0.452040499	0.333	0.164	0.00015105	T cells, CD8+, naive, stimulated
**Ywhah**	1.09E-08	0.490430187	0.454	0.265	0.00015418	T cells, CD8+, naive, stimulated
**Actb**	1.21E-08	-0.383627748	0.999	0.956	0.00017063	T cells, CD8+, naive, stimulated
**Zfp318**	1.24E-08	0.250627586	0.252	0.087	0.00017530	T cells, CD8+, naive, stimulated
**Chtf8**	1.26E-08	0.586573124	0.179	0.05	0.00017828	T cells, CD8+, naive, stimulated
**Rpl29**	1.44E-08	-0.385194346	0.979	0.842	0.00020360	T cells, CD8+, naive, stimulated
**Set**	1.5E-08	0.378716475	0.372	0.205	0.00021284	T cells, CD8+, naive, stimulated
**Ptges3**	1.53E-08	0.526726475	0.367	0.195	0.00021725	T cells, CD8+, naive, stimulated
**Cnp**	1.56E-08	0.45752595	0.39	0.208	0.00022071	T cells, CD8+, naive, stimulated
**Vars**	1.56E-08	0.296848068	0.352	0.181	0.00022131	T cells, CD8+, naive, stimulated
**Sipa1**	1.71E-08	0.324743392	0.302	0.134	0.00024139	T cells, CD8+, naive, stimulated

**Snx2**	1.72E-08	0.304281455	0.545	0.322	0.00024302	T cells, CD8+, naive, stimulated
**Bri3**	1.74E-08	0.437926613	0.593	0.389	0.00024627	T cells, CD8+, naive, stimulated
**Tiparp**	1.81E-08	0.512873265	0.263	0.111	0.00025586	T cells, CD8+, naive, stimulated
**Atp6v0d1**	1.89E-08	0.503897459	0.363	0.191	0.00026717	T cells, CD8+, naive, stimulated
**Irs2**	2.29E-08	0.307688979	0.288	0.128	0.00032392	T cells, CD8+, naive, stimulated
**Lpgat1**	2.3E-08	0.504936292	0.201	0.067	0.00032606	T cells, CD8+, naive, stimulated
**Plekhm1**	2.35E-08	0.446013788	0.185	0.054	0.00033197	T cells, CD8+, naive, stimulated
**Hnrnpdl**	2.45E-08	0.325536046	0.451	0.258	0.00034678	T cells, CD8+, naive, stimulated
**Mta2**	2.46E-08	0.588650257	0.202	0.07	0.00034799	T cells, CD8+, naive, stimulated
**Wdr1**	2.46E-08	0.505119497	0.275	0.124	0.00034886	T cells, CD8+, naive, stimulated
**Arpc5**	2.65E-08	0.332296981	0.536	0.315	0.00037564	T cells, CD8+, naive, stimulated
**Tnfrsf13c**	2.75E-08	0.519277302	0.29	0.128	0.00038921	T cells, CD8+, naive, stimulated
**Cirbp**	2.78E-08	0.361481153	0.507	0.299	0.00039339	T cells, CD8+, naive, stimulated
**Ptp4a2**	2.83E-08	0.329213964	0.391	0.205	0.00040064	T cells, CD8+, naive, stimulated
**Rab11b**	2.87E-08	0.396032209	0.381	0.208	0.00040688	T cells, CD8+, naive, stimulated
**St6gal1**	3.04E-08	0.307541478	0.308	0.141	0.00043061	T cells, CD8+, naive, stimulated
**Trp53inp2**	3.13E-08	0.495477494	0.228	0.087	0.00044368	T cells, CD8+, naive, stimulated
**Atp6ap1**	3.85E-08	0.493721021	0.274	0.124	0.00054478	T cells, CD8+, naive, stimulated
**Parp1**	4.3E-08	0.442159471	0.311	0.151	0.00060864	T cells, CD8+, naive, stimulated
**Csde1**	4.51E-08	0.410139159	0.283	0.138	0.00063894	T cells, CD8+, naive, stimulated
**Hnrnpa3**	4.76E-08	0.263786765	0.581	0.366	0.00067377	T cells, CD8+, naive, stimulated
**Ssrp1**	4.97E-08	0.470164269	0.219	0.081	0.00070345	T cells, CD8+, naive, stimulated
**Esyt1**	5.14E-08	0.308033102	0.246	0.104	0.00072813	T cells, CD8+, naive, stimulated
**Rnf44**	5.29E-08	0.448367571	0.247	0.104	0.00074853	T cells, CD8+, naive, stimulated
**Cmtm6**	5.33E-08	0.462176054	0.229	0.094	0.00075411	T cells, CD8+, naive, stimulated
**Srp54b**	5.58E-08	0.554430608	0.152	0.037	0.00079007	T cells, CD8+, naive, stimulated
**Birc3**	5.71E-08	0.386018003	0.383	0.215	0.00080776	T cells, CD8+, naive, stimulated
**Smap2**	5.81E-08	0.370051419	0.376	0.205	0.00082261	T cells, CD8+, naive, stimulated
**M6pr**	5.84E-08	0.284394506	0.275	0.124	0.00082672	T cells, CD8+, naive, stimulated
**Rras2**	6.24E-08	0.456717144	0.172	0.047	0.00088356	T cells, CD8+, naive, stimulated
**Nfkb2**	6.68E-08	0.315805851	0.258	0.111	0.00094493	T cells, CD8+, naive, stimulated
**Pgam1**	6.69E-08	0.400941152	0.254	0.104	0.00094729	T cells, CD8+, naive, stimulated
**Vamp2**	7.38E-08	0.580480147	0.2	0.074	0.00104495	T cells, CD8+, naive, stimulated
**U2af2**	7.74E-08	0.360447048	0.293	0.138	0.00109569	T cells, CD8+, naive, stimulated
**Ccni**	7.74E-08	0.471648303	0.251	0.111	0.00109599	T cells, CD8+, naive, stimulated

**Tcf3**	8.35E-08	0.457108124	0.305	0.154	0.00118236	T cells, CD8+, naive, stimulated
**Nsmce4a**	8.4E-08	0.463884374	0.249	0.107	0.00118932	T cells, CD8+, naive, stimulated
**Helz2**	8.45E-08	0.430978953	0.251	0.104	0.00119603	T cells, CD8+, naive, stimulated
**Myc**	8.82E-08	0.694228974	0.225	0.087	0.00124856	T cells, CD8+, naive, stimulated
**Pim3**	8.85E-08	0.506780954	0.212	0.077	0.00125309	T cells, CD8+, naive, stimulated
**Traf5**	8.98E-08	0.472797363	0.169	0.047	0.00127191	T cells, CD8+, naive, stimulated
**Plac8**	9.65E-08	-1.094165313	0.417	0.477	0.00136658	T cells, CD8+, naive, stimulated
**Rps3**	1.02E-07	-0.268530371	0.996	0.876	0.00144139	T cells, CD8+, naive, stimulated
**Myo1c**	1.02E-07	0.334074158	0.243	0.107	0.00144752	T cells, CD8+, naive, stimulated
**Tubb5**	1.07E-07	0.308494849	0.428	0.245	0.00151473	T cells, CD8+, naive, stimulated
**Dedd2**	1.07E-07	0.431783705	0.224	0.084	0.00151490	T cells, CD8+, naive, stimulated
**Prkar1a**	1.07E-07	0.350058806	0.469	0.279	0.00151817	T cells, CD8+, naive, stimulated
**Lsm14a**	1.11E-07	0.501762635	0.194	0.07	0.00157507	T cells, CD8+, naive, stimulated
**Map4k1**	1.18E-07	0.472477367	0.278	0.134	0.00166719	T cells, CD8+, naive, stimulated
**Ppp1cb**	1.2E-07	0.302761798	0.227	0.087	0.00170268	T cells, CD8+, naive, stimulated
**Ets2**	1.23E-07	0.524563345	0.24	0.104	0.00173754	T cells, CD8+, naive, stimulated
**Cmpk1**	1.23E-07	0.586366677	0.253	0.117	0.00174711	T cells, CD8+, naive, stimulated
**Snx8**	1.37E-07	0.448480302	0.299	0.144	0.00194239	T cells, CD8+, naive, stimulated
**Rnf144a**	1.38E-07	0.322474975	0.132	0.023	0.00195514	T cells, CD8+, naive, stimulated
**Isca1**	1.39E-07	0.571210097	0.242	0.107	0.00196932	T cells, CD8+, naive, stimulated
**Fbxo11**	1.41E-07	0.394029767	0.219	0.091	0.00200259	T cells, CD8+, naive, stimulated
**Rasa3**	1.44E-07	0.357403507	0.372	0.205	0.00203429	T cells, CD8+, naive, stimulated
**Sf3b4**	1.48E-07	0.392838763	0.262	0.117	0.00209512	T cells, CD8+, naive, stimulated
**Atf7ip**	1.49E-07	0.405780946	0.283	0.138	0.00210547	T cells, CD8+, naive, stimulated
**Pxdc1**	1.71E-07	0.400233938	0.131	0.023	0.00241424	T cells, CD8+, naive, stimulated
**Ptpn6**	1.75E-07	0.386535808	0.383	0.208	0.00247924	T cells, CD8+, naive, stimulated
**Ccng2**	1.78E-07	0.510666424	0.148	0.037	0.00251693	T cells, CD8+, naive, stimulated
**Pax5**	1.78E-07	0.283638622	0.352	0.181	0.00252268	T cells, CD8+, naive, stimulated
**Vasp**	1.85E-07	0.462639094	0.271	0.131	0.00261393	T cells, CD8+, naive, stimulated
**H2-Eb2**	1.86E-07	0.405504931	0.206	0.074	0.00263073	T cells, CD8+, naive, stimulated
**Aldh2**	1.89E-07	0.374498913	0.409	0.238	0.00266856	T cells, CD8+, naive, stimulated
**Hspd1**	1.9E-07	0.273281242	0.583	0.396	0.00268816	T cells, CD8+, naive, stimulated
**Erp29**	2.04E-07	-0.733526819	0.452	0.517	0.00288725	T cells, CD8+, naive, stimulated
**Sgms1**	2.06E-07	0.35108484	0.222	0.087	0.00291810	T cells, CD8+, naive, stimulated
**G3bp1**	2.11E-07	0.379143241	0.28	0.138	0.00298779	T cells, CD8+, naive, stimulated

**Plcg2**	2.13E-07	0.295601515	0.319	0.168	0.00301129	T cells, CD8+, naive, stimulated
**Trim28**	2.16E-07	0.479949791	0.217	0.091	0.00306303	T cells, CD8+, naive, stimulated
**Coq10b**	2.26E-07	0.373600439	0.328	0.174	0.00319379	T cells, CD8+, naive, stimulated
**Csnk1g3**	2.28E-07	0.345676967	0.278	0.131	0.00323098	T cells, CD8+, naive, stimulated
**Tpd52**	2.31E-07	0.493086812	0.266	0.128	0.00326645	T cells, CD8+, naive, stimulated
**Neu1**	2.31E-07	0.425866352	0.213	0.084	0.00327048	T cells, CD8+, naive, stimulated
**Wtap**	2.43E-07	0.439632801	0.186	0.067	0.00344012	T cells, CD8+, naive, stimulated
**Kpna4**	2.51E-07	0.304998294	0.319	0.161	0.00354639	T cells, CD8+, naive, stimulated
**Mirt1**	2.54E-07	0.464958463	0.197	0.074	0.00359362	T cells, CD8+, naive, stimulated
**Add1**	2.67E-07	0.465759051	0.268	0.131	0.00378426	T cells, CD8+, naive, stimulated
**Cdt1**	2.68E-07	0.353428787	0.245	0.104	0.00379478	T cells, CD8+, naive, stimulated
**Malat1**	2.73E-07	-1.080874102	0.996	0.977	0.00386857	T cells, CD8+, naive, stimulated
**Rftn1**	2.87E-07	0.446212056	0.221	0.094	0.00405981	T cells, CD8+, naive, stimulated
**Hnrnpm**	2.9E-07	0.332783618	0.279	0.128	0.00410214	T cells, CD8+, naive, stimulated
**Zc3hav1**	3.09E-07	0.32446636	0.277	0.131	0.00438104	T cells, CD8+, naive, stimulated
**Litaf**	3.22E-07	0.412772555	0.536	0.352	0.00456290	T cells, CD8+, naive, stimulated
**Nxf1**	3.26E-07	0.324611531	0.255	0.117	0.00460845	T cells, CD8+, naive, stimulated
**Rfk**	3.3E-07	0.51962175	0.233	0.104	0.00466781	T cells, CD8+, naive, stimulated
**Plaur**	3.5E-07	0.44690034	0.414	0.242	0.00495449	T cells, CD8+, naive, stimulated
**Ctps2**	3.51E-07	0.507389759	0.137	0.034	0.00497264	T cells, CD8+, naive, stimulated
**Rasgrp2**	3.68E-07	0.297429715	0.502	0.302	0.00520910	T cells, CD8+, naive, stimulated
**Ctdsp1**	3.89E-07	0.510974935	0.152	0.044	0.00551283	T cells, CD8+, naive, stimulated
**Bin1**	3.97E-07	0.33061807	0.278	0.131	0.00561788	T cells, CD8+, naive, stimulated
**Zdhhc18**	4E-07	0.430525831	0.155	0.044	0.00566254	T cells, CD8+, naive, stimulated
**Dgka**	4.05E-07	0.35817382	0.231	0.097	0.00572818	T cells, CD8+, naive, stimulated
**Galnt1**	4.11E-07	0.428649648	0.19	0.07	0.00581631	T cells, CD8+, naive, stimulated
**Sypl**	4.19E-07	0.401935957	0.292	0.151	0.00592566	T cells, CD8+, naive, stimulated
**Fos**	4.21E-07	0.456466366	0.643	0.473	0.00596156	T cells, CD8+, naive, stimulated
**Cct3**	4.23E-07	0.400846923	0.267	0.131	0.00598431	T cells, CD8+, naive, stimulated
**Vezf1**	4.27E-07	0.428507317	0.194	0.074	0.00603851	T cells, CD8+, naive, stimulated
**Lrrk2**	4.31E-07	0.280310162	0.206	0.074	0.00609573	T cells, CD8+, naive, stimulated
**Cbx7**	4.31E-07	0.399998706	0.164	0.05	0.00609832	T cells, CD8+, naive, stimulated
**Plekho2**	4.32E-07	0.507613234	0.282	0.144	0.00611119	T cells, CD8+, naive, stimulated
**Tfeb**	4.36E-07	0.47377236	0.146	0.04	0.00617520	T cells, CD8+, naive, stimulated
**Elavl1**	4.68E-07	0.45129762	0.263	0.131	0.00661818	T cells, CD8+, naive, stimulated

**Ndufa3**	4.69E-07	0.262901627	0.57	0.352	0.00663335	T cells, CD8+, naive, stimulated
**Dcaf12**	4.7E-07	0.445137104	0.184	0.067	0.00665602	T cells, CD8+, naive, stimulated
**Rab7**	4.71E-07	0.516066514	0.274	0.144	0.00667176	T cells, CD8+, naive, stimulated
**Lpcat1**	4.88E-07	0.332650776	0.209	0.081	0.00690415	T cells, CD8+, naive, stimulated
**Pink1**	4.91E-07	0.570583237	0.203	0.084	0.00695165	T cells, CD8+, naive, stimulated
**Tfrc**	5.16E-07	0.358452498	0.122	0.023	0.00730639	T cells, CD8+, naive, stimulated
**Thrap3**	5.21E-07	0.357198726	0.297	0.151	0.00737120	T cells, CD8+, naive, stimulated
**Gorasp2**	5.37E-07	0.368156674	0.282	0.138	0.00760404	T cells, CD8+, naive, stimulated
**Cdc25b**	5.43E-07	0.360603553	0.166	0.054	0.00768647	T cells, CD8+, naive, stimulated
**Hexb**	5.47E-07	0.397528767	0.294	0.151	0.00774345	T cells, CD8+, naive, stimulated
**Gpr132**	5.53E-07	0.282105489	0.321	0.164	0.00782184	T cells, CD8+, naive, stimulated
**S1pr4**	5.62E-07	0.421812163	0.23	0.097	0.00794969	T cells, CD8+, naive, stimulated
**Grk6**	5.69E-07	0.371910209	0.314	0.171	0.00805370	T cells, CD8+, naive, stimulated
**Pnrc2**	5.69E-07	0.45997391	0.18	0.067	0.00805488	T cells, CD8+, naive, stimulated
**Tm9sf2**	5.78E-07	0.326479045	0.219	0.094	0.00817511	T cells, CD8+, naive, stimulated
**Smarcd2**	6.16E-07	0.467767881	0.179	0.067	0.00872480	T cells, CD8+, naive, stimulated
**Prpf38a**	6.21E-07	0.416366092	0.174	0.06	0.00879574	T cells, CD8+, naive, stimulated
**Ddx3x**	6.24E-07	0.294238328	0.314	0.168	0.00883834	T cells, CD8+, naive, stimulated
**Mat2b**	6.61E-07	0.418259832	0.191	0.074	0.00935527	T cells, CD8+, naive, stimulated
**Mob1a**	6.72E-07	0.353449628	0.218	0.091	0.00950852	T cells, CD8+, naive, stimulated
**Tcp1**	7.35E-07	0.408882637	0.351	0.205	0.01041070	T cells, CD8+, naive, stimulated
**Srek1**	7.43E-07	-0.570354868	0.044	0.114	0.01051405	T cells, CD8+, naive, stimulated
**Brd4**	7.44E-07	0.474865187	0.321	0.185	0.01053647	T cells, CD8+, naive, stimulated
**Ppp1r2**	7.57E-07	0.389440166	0.292	0.151	0.01072099	T cells, CD8+, naive, stimulated
**Sik1**	7.59E-07	0.267241084	0.379	0.218	0.01074579	T cells, CD8+, naive, stimulated
**Anp32e**	7.85E-07	0.402666878	0.286	0.144	0.01110647	T cells, CD8+, naive, stimulated
**Gt(ROSA)2**	8.14E-07	0.393254212	0.184	0.067	0.01152729	T cells, CD8+, naive, stimulated
**Golph3**	8.27E-07	0.554838191	0.155	0.05	0.01170920	T cells, CD8+, naive, stimulated
**Btk**	8.62E-07	0.487764148	0.256	0.128	0.01220057	T cells, CD8+, naive, stimulated
**Skil**	8.63E-07	0.406860031	0.204	0.084	0.01221333	T cells, CD8+, naive, stimulated
**Kras**	8.7E-07	0.34132678	0.369	0.218	0.01231393	T cells, CD8+, naive, stimulated
**mt-Atp6**	9.02E-07	-0.694953839	0.975	0.923	0.01277358	T cells, CD8+, naive, stimulated
**Dnaja2**	9.11E-07	0.35759366	0.307	0.161	0.01289177	T cells, CD8+, naive, stimulated
**Skp1a**	9.14E-07	0.274211647	0.41	0.248	0.01294178	T cells, CD8+, naive, stimulated
**Ifnar1**	9.19E-07	0.410990373	0.259	0.128	0.01300411	T cells, CD8+, naive, stimulated

**Ptbp1**	9.26E-07	0.375975452	0.299	0.154	0.01311184	T cells, CD8+, naive, stimulated
**Syf2**	9.3E-07	0.283354537	0.407	0.242	0.01316255	T cells, CD8+, naive, stimulated
**Canx**	9.51E-07	0.26476015	0.29	0.148	0.01346470	T cells, CD8+, naive, stimulated
**Ginm1**	9.56E-07	0.497973289	0.145	0.044	0.01353846	T cells, CD8+, naive, stimulated
**Ywhae**	9.71E-07	0.351140182	0.371	0.221	0.01374003	T cells, CD8+, naive, stimulated
**Supt5**	9.84E-07	0.280317208	0.199	0.081	0.01392656	T cells, CD8+, naive, stimulated
**Pcna**	1.09E-06	0.461770313	0.237	0.111	0.01545655	T cells, CD8+, naive, stimulated
**Srebf2**	1.09E-06	0.386798202	0.226	0.107	0.01546415	T cells, CD8+, naive, stimulated
**Kif5b**	1.13E-06	0.386096812	0.287	0.151	0.01604557	T cells, CD8+, naive, stimulated
**Bmp2k**	1.13E-06	0.292049287	0.181	0.067	0.01606656	T cells, CD8+, naive, stimulated
**Tmem108**	1.17E-06	0.320196104	0.18	0.06	0.01650844	T cells, CD8+, naive, stimulated
**BC005537**	1.22E-06	0.410497863	0.249	0.124	0.01726168	T cells, CD8+, naive, stimulated
**Rpia**	1.29E-06	0.637974322	0.201	0.091	0.01826530	T cells, CD8+, naive, stimulated
**Irak2**	1.42E-06	0.467512643	0.172	0.064	0.02006940	T cells, CD8+, naive, stimulated
**Evi2a**	1.43E-06	-0.296435666	0.043	0.114	0.02027950	T cells, CD8+, naive, stimulated
**Ptms**	1.51E-06	-0.404894608	0.052	0.128	0.02144580	T cells, CD8+, naive, stimulated
**Cmah**	1.54E-06	0.27856382	0.269	0.131	0.02181795	T cells, CD8+, naive, stimulated
**Cbfb**	1.71E-06	0.406552352	0.193	0.081	0.02421547	T cells, CD8+, naive, stimulated
**Gucd1**	1.75E-06	0.378633564	0.136	0.037	0.02482706	T cells, CD8+, naive, stimulated
**Atp5k**	1.8E-06	0.392485173	0.377	0.232	0.02554366	T cells, CD8+, naive, stimulated
**Zc3h12a**	1.81E-06	0.286551551	0.232	0.107	0.02559781	T cells, CD8+, naive, stimulated
**Zfp622**	1.87E-06	0.437701645	0.218	0.097	0.02650686	T cells, CD8+, naive, stimulated
**Paip2**	1.87E-06	0.401837089	0.448	0.285	0.02651522	T cells, CD8+, naive, stimulated
**Msi2**	1.87E-06	0.356917331	0.124	0.03	0.02652552	T cells, CD8+, naive, stimulated
**Heg1**	1.94E-06	0.370189373	0.194	0.077	0.02740194	T cells, CD8+, naive, stimulated
**Ifrd1**	1.97E-06	0.321972953	0.364	0.201	0.02786098	T cells, CD8+, naive, stimulated
**Abi1**	2.03E-06	0.281205341	0.207	0.087	0.02875051	T cells, CD8+, naive, stimulated
**Tm9sf3**	2.31E-06	0.446125446	0.198	0.087	0.03263529	T cells, CD8+, naive, stimulated
**Npc2**	2.37E-06	-0.790748583	0.266	0.366	0.03360802	T cells, CD8+, naive, stimulated
**Mcrs1**	2.4E-06	0.355686528	0.144	0.044	0.03394738	T cells, CD8+, naive, stimulated
**Eif3l**	2.49E-06	0.396951122	0.206	0.094	0.03517950	T cells, CD8+, naive, stimulated
**Rac2**	2.49E-06	-0.641662471	0.74	0.661	0.03528863	T cells, CD8+, naive, stimulated
**Mapk1**	2.5E-06	0.301578875	0.241	0.121	0.03543073	T cells, CD8+, naive, stimulated
**Myadm**	2.52E-06	0.545351075	0.213	0.101	0.03566596	T cells, CD8+, naive, stimulated
**Rpl28**	2.57E-06	-0.252177323	0.993	0.862	0.03632912	T cells, CD8+, naive, stimulated

**Rassf5**	2.64E-06	0.352713077	0.143	0.044	0.0374327	T cells, CD8+, naive, stimulated
**Ppp2r1a**	2.66E-06	0.332691165	0.234	0.117	0.0376466	T cells, CD8+, naive, stimulated
**Wac**	2.85E-06	0.382395052	0.227	0.111	0.0403382	T cells, CD8+, naive, stimulated
**Tmem243**	2.94E-06	0.399251238	0.32	0.185	0.0416290	T cells, CD8+, naive, stimulated
**Rassf4**	2.94E-06	-0.449031841	0.064	0.144	0.0416588	T cells, CD8+, naive, stimulated
**Fam220a**	3.02E-06	0.420256146	0.119	0.03	0.0427368	T cells, CD8+, naive, stimulated
**Map3k1**	3.02E-06	0.257929043	0.222	0.101	0.0427750	T cells, CD8+, naive, stimulated
**Mtpn**	3.17E-06	0.427735361	0.32	0.198	0.0448430	T cells, CD8+, naive, stimulated
**Rnf130**	3.28E-06	0.457982537	0.187	0.081	0.0464107	T cells, CD8+, naive, stimulated
**Txndc5**	3.3E-06	0.284684959	0.234	0.111	0.0466628	T cells, CD8+, naive, stimulated
**Cd200**	3.33E-06	0.457767456	0.141	0.044	0.0471570	T cells, CD8+, naive, stimulated
**Spib**	3.36E-06	0.425002295	0.275	0.144	0.0475349	T cells, CD8+, naive, stimulated
**Ccl5**	3.01E-98	2.09945865	0.445	0.03	4.26E-94	T cells, CD4+, naive, stimulated
**Btg1**	3.61E-71	-1.853404152	0.697	0.959	5.1E-67	T cells, CD4+, naive, stimulated
**Rps29**	4.62E-67	-1.037596704	0.962	0.976	6.53E-63	T cells, CD4+, naive, stimulated
**Rplp0**	5.69E-62	0.928887449	1	0.97	8.06E-58	T cells, CD4+, naive, stimulated
**Rpsa**	6.9E-57	0.745193981	1	0.978	9.77E-53	T cells, CD4+, naive, stimulated
**Klf2**	1.01E-55	-1.718987534	0.748	0.946	1.43E-51	T cells, CD4+, naive, stimulated
**Eef1a1**	2.23E-55	-0.828783844	0.958	0.98	3.16E-51	T cells, CD4+, naive, stimulated
**Rps27rt**	2.94E-55	1.983520136	0.689	0.28	4.17E-51	T cells, CD4+, naive, stimulated
**Nr4a1**	4.41E-54	-2.112406968	0.496	0.86	6.24E-50	T cells, CD4+, naive, stimulated
**Rpl13**	5.59E-54	0.65173067	1	0.98	7.91E-50	T cells, CD4+, naive, stimulated
**Rps4x**	2.25E-50	0.664447509	1	0.974	3.19E-46	T cells, CD4+, naive, stimulated
**Jund**	9.84E-49	-1.286762161	0.765	0.959	1.39E-44	T cells, CD4+, naive, stimulated
**Rps27**	3.13E-48	-0.745864878	0.979	0.981	4.43E-44	T cells, CD4+, naive, stimulated
**Hspa8**	6.19E-48	-1.75687086	0.739	0.944	8.77E-44	T cells, CD4+, naive, stimulated
**Rps2**	3.04E-46	0.724415041	0.996	0.983	4.3E-42	T cells, CD4+, naive, stimulated
**Rps8**	4.84E-45	0.584213578	1	0.982	6.85E-41	T cells, CD4+, naive, stimulated
**Junb**	2.98E-43	-1.427326545	0.723	0.936	4.22E-39	T cells, CD4+, naive, stimulated
**Rps3a1**	7.56E-43	0.583305736	1	0.981	1.07E-38	T cells, CD4+, naive, stimulated
**Cxcr4**	1.84E-42	-1.979066279	0.134	0.611	2.61E-38	T cells, CD4+, naive, stimulated
**Stk17b**	5.35E-42	-1.418845189	0.458	0.852	7.58E-38	T cells, CD4+, naive, stimulated
**Rps28**	5.63E-41	-0.698493057	0.945	0.972	7.97E-37	T cells, CD4+, naive, stimulated
**Rps5**	1.04E-40	0.636181141	0.996	0.975	1.47E-36	T cells, CD4+, naive, stimulated
**Rpl18**	1.05E-40	0.689922458	0.996	0.972	1.48E-36	T cells, CD4+, naive, stimulated

**Rpl35a**	2.32E-40	-0.608680726	0.975	0.967	3.29E-36	T cells, CD4+, naive, stimulated
**Dusp2**	5.22E-40	-1.546835099	0.324	0.762	7.39E-36	T cells, CD4+, naive, stimulated
**Eef2**	1.52E-39	-0.931654004	0.668	0.948	2.16E-35	T cells, CD4+, naive, stimulated
**Rps7**	2.74E-38	0.556242198	1	0.978	3.88E-34	T cells, CD4+, naive, stimulated
**Tsc22d3**	2.78E-38	-1.228670149	0.487	0.864	3.93E-34	T cells, CD4+, naive, stimulated
**Rps9**	1.56E-37	0.625384476	0.987	0.962	2.21E-33	T cells, CD4+, naive, stimulated
**Cytip**	2.05E-37	-1.422467842	0.345	0.778	2.91E-33	T cells, CD4+, naive, stimulated
**Rpl11**	2.42E-37	0.597172884	0.987	0.972	3.43E-33	T cells, CD4+, naive, stimulated
**Rps11**	4.99E-37	0.661709106	0.987	0.972	7.07E-33	T cells, CD4+, naive, stimulated
**Ccr7**	1.37E-35	-1.306705258	0.584	0.885	1.94E-31	T cells, CD4+, naive, stimulated
**Rpl9-ps6**	3.68E-35	0.545957583	0.996	0.972	5.2E-31	T cells, CD4+, naive, stimulated
**Rpl8**	4.09E-35	0.56434231	0.996	0.975	5.79E-31	T cells, CD4+, naive, stimulated
**Ets1**	1.76E-34	-1.222092378	0.345	0.775	2.48E-30	T cells, CD4+, naive, stimulated
**Rpl10a**	1.81E-34	0.700237593	0.975	0.961	2.56E-30	T cells, CD4+, naive, stimulated
**Ifi30**	3.15E-34	1.563469354	0.685	0.367	4.45E-30	T cells, CD4+, naive, stimulated
**S100a6**	1.24E-33	2.597059909	0.273	0.051	1.75E-29	T cells, CD4+, naive, stimulated
**Rps3**	4.23E-33	0.575768998	0.992	0.973	5.99E-29	T cells, CD4+, naive, stimulated
**Rpl19**	8.5E-33	0.483678977	0.996	0.979	1.2E-28	T cells, CD4+, naive, stimulated
**mt-Nd4l**	1.48E-32	-0.800514287	0.849	0.987	2.1E-28	T cells, CD4+, naive, stimulated
**Rpl6**	3.06E-32	0.540057797	1	0.978	4.33E-28	T cells, CD4+, naive, stimulated
**Ier5**	1.79E-31	-1.270117107	0.294	0.709	2.53E-27	T cells, CD4+, naive, stimulated
**Rpl18a**	2.65E-31	0.536807385	0.996	0.974	3.75E-27	T cells, CD4+, naive, stimulated
**Rps10**	4.16E-31	0.497691755	0.992	0.97	5.89E-27	T cells, CD4+, naive, stimulated
**mt-Nd3**	1.06E-30	-1.323693629	0.214	0.627	1.5E-26	T cells, CD4+, naive, stimulated
**Hspa1a**	1.56E-30	-3.320935889	0.244	0.587	2.21E-26	T cells, CD4+, naive, stimulated
**Gapdh**	3.57E-30	0.982196844	0.916	0.862	5.06E-26	T cells, CD4+, naive, stimulated
**Rap1b**	1.4E-29	-1.255985542	0.282	0.68	1.99E-25	T cells, CD4+, naive, stimulated
**Ddx5**	1.79E-29	-0.803986884	0.706	0.953	2.53E-25	T cells, CD4+, naive, stimulated
**Rpl28**	2.04E-29	0.559176035	0.983	0.968	2.88E-25	T cells, CD4+, naive, stimulated
**Rpl29**	2.45E-29	0.674151005	0.971	0.953	3.47E-25	T cells, CD4+, naive, stimulated
**Ywhaz**	1.38E-28	-0.99265293	0.475	0.843	1.96E-24	T cells, CD4+, naive, stimulated
**Hnrnpf**	4.8E-28	-1.153789793	0.34	0.715	6.8E-24	T cells, CD4+, naive, stimulated
**Serp1**	1.14E-27	-0.91677607	0.542	0.858	1.61E-23	T cells, CD4+, naive, stimulated
**Fam107b**	3.97E-27	-1.2580875	0.353	0.708	5.63E-23	T cells, CD4+, naive, stimulated
**Pabpc1**	8.32E-27	-0.922026087	0.508	0.862	1.18E-22	T cells, CD4+, naive, stimulated

**Rpl38**	1.36E-26	-0.531250906	0.941	0.974	1.92E-22	T cells, CD4+, naive, stimulated
**Vps37b**	1.77E-26	-1.379247382	0.324	0.682	2.5E-22	T cells, CD4+, naive, stimulated
**Fcer2a**	3.07E-26	-1.263585337	0.193	0.593	4.35E-22	T cells, CD4+, naive, stimulated
**Snx5**	3.68E-26	-1.181845189	0.282	0.666	5.21E-22	T cells, CD4+, naive, stimulated
**mt-Atp8**	3.75E-26	-0.60267521	0.958	0.999	5.31E-22	T cells, CD4+, naive, stimulated
**Hsph1**	9.99E-26	-1.722162695	0.218	0.568	1.41E-21	T cells, CD4+, naive, stimulated
**Dusp1**	1.06E-25	-1.463860792	0.189	0.566	1.5E-21	T cells, CD4+, naive, stimulated
**Lgals1**	1.39E-25	1.00396364	0.239	0.049	1.96E-21	T cells, CD4+, naive, stimulated
**Serinc3**	1.49E-25	-0.936695055	0.403	0.785	2.12E-21	T cells, CD4+, naive, stimulated
**Cd79a**	2.43E-25	0.629505419	0.979	0.977	3.44E-21	T cells, CD4+, naive, stimulated
**Rps20**	2.85E-25	0.412009166	0.996	0.978	4.03E-21	T cells, CD4+, naive, stimulated
**Rhob**	3.52E-25	-1.546283437	0.172	0.527	4.99E-21	T cells, CD4+, naive, stimulated
**Pcbp2**	2.42E-24	-0.923822023	0.399	0.781	3.42E-20	T cells, CD4+, naive, stimulated
**Clic1**	2.61E-24	1.041166447	0.744	0.554	3.7E-20	T cells, CD4+, naive, stimulated
**Cnbp**	3.99E-24	-0.863947033	0.504	0.839	5.65E-20	T cells, CD4+, naive, stimulated
**Zfp36l2**	1.35E-23	-1.287792774	0.227	0.578	1.92E-19	T cells, CD4+, naive, stimulated
**Rpl15**	6.41E-23	0.583177285	0.962	0.955	9.08E-19	T cells, CD4+, naive, stimulated
**Rpl24**	6.85E-23	0.581735877	0.958	0.936	9.7E-19	T cells, CD4+, naive, stimulated
**Satb1**	8.3E-23	-1.160660144	0.164	0.515	1.18E-18	T cells, CD4+, naive, stimulated
**Ezr**	9.91E-23	-0.996368592	0.37	0.725	1.4E-18	T cells, CD4+, naive, stimulated
**Tomm6**	3.52E-22	1.115025549	0.437	0.179	4.99E-18	T cells, CD4+, naive, stimulated
**Dazap2**	5.23E-22	-0.973275393	0.345	0.672	7.4E-18	T cells, CD4+, naive, stimulated
**Actg1**	5.84E-22	-0.726398913	0.702	0.93	8.27E-18	T cells, CD4+, naive, stimulated
**Zfp36**	6.81E-22	-0.973385347	0.458	0.787	9.64E-18	T cells, CD4+, naive, stimulated
**Rpl32**	1.08E-21	0.436226356	0.983	0.97	1.53E-17	T cells, CD4+, naive, stimulated
**Bach2**	2.34E-21	-1.193382106	0.126	0.467	3.32E-17	T cells, CD4+, naive, stimulated
**Anxa2**	3.39E-21	0.665589667	0.122	0.013	4.79E-17	T cells, CD4+, naive, stimulated
**Hspa1b**	3.72E-21	-1.684650661	0.307	0.608	5.27E-17	T cells, CD4+, naive, stimulated
**P2ry10**	5.03E-21	-1.170707287	0.185	0.509	7.12E-17	T cells, CD4+, naive, stimulated
**Tcp11l2**	7.48E-21	-1.208376141	0.185	0.513	1.06E-16	T cells, CD4+, naive, stimulated
**Actb**	9.89E-21	0.563294452	0.996	0.991	1.4E-16	T cells, CD4+, naive, stimulated
**Rpl23**	9.9E-21	0.384900468	0.996	0.974	1.4E-16	T cells, CD4+, naive, stimulated
**Syk**	1.85E-20	-1.0099696	0.227	0.585	2.62E-16	T cells, CD4+, naive, stimulated
**Dnajb1**	2.09E-20	-1.761793248	0.441	0.696	2.96E-16	T cells, CD4+, naive, stimulated
**Naca**	2.5E-20	0.690879993	0.895	0.866	3.54E-16	T cells, CD4+, naive, stimulated

**Cfap43**	4.11E-20	-1.261768112	0.025	0.308	5.81E-16	T cells, CD4+, naive, stimulated
**Rps12**	5.15E-20	0.608661805	0.954	0.942	7.29E-16	T cells, CD4+, naive, stimulated
**Ubc**	6.43E-20	-0.957165385	0.504	0.787	9.1E-16	T cells, CD4+, naive, stimulated
**H2-D1**	1.06E-19	-0.530218081	0.878	0.973	1.5E-15	T cells, CD4+, naive, stimulated
**Klf6**	1.23E-19	-1.08614151	0.378	0.696	1.74E-15	T cells, CD4+, naive, stimulated
**Cd19**	1.74E-19	-0.877144334	0.332	0.684	2.46E-15	T cells, CD4+, naive, stimulated
**Rps26**	2.13E-19	0.415350847	0.992	0.968	3.01E-15	T cells, CD4+, naive, stimulated
**Plac8**	3.12E-19	1.410423491	0.588	0.401	4.41E-15	T cells, CD4+, naive, stimulated
**Pnrc1**	4.46E-19	-0.818333984	0.559	0.81	6.32E-15	T cells, CD4+, naive, stimulated
**Rpl14**	4.48E-19	0.554892659	0.929	0.928	6.34E-15	T cells, CD4+, naive, stimulated
**Plk2**	5.67E-19	-1.593864553	0.059	0.348	8.03E-15	T cells, CD4+, naive, stimulated
**Arf6**	5.74E-19	-0.881641796	0.294	0.63	8.12E-15	T cells, CD4+, naive, stimulated
**Cd79b**	6.02E-19	0.803197422	0.882	0.837	8.52E-15	T cells, CD4+, naive, stimulated
**Rpl10-ps3**	6.63E-19	0.512971304	0.937	0.933	9.39E-15	T cells, CD4+, naive, stimulated
**H3f3b**	1.88E-18	-0.536221486	0.971	0.986	2.66E-14	T cells, CD4+, naive, stimulated
**Klf4**	6.2E-18	-1.373435805	0.197	0.484	8.78E-14	T cells, CD4+, naive, stimulated
**Rps16**	1.02E-17	0.340320804	1	0.978	1.44E-13	T cells, CD4+, naive, stimulated
**Gnas**	1.32E-17	-0.806029739	0.45	0.742	1.86E-13	T cells, CD4+, naive, stimulated
**Hspa5**	1.56E-17	-0.838972073	0.466	0.759	2.21E-13	T cells, CD4+, naive, stimulated
**Cd74**	1.83E-17	0.317768541	1	0.999	2.59E-13	T cells, CD4+, naive, stimulated
**Rps27a**	1.87E-17	0.352702791	0.983	0.97	2.65E-13	T cells, CD4+, naive, stimulated
**Scd1**	2.04E-17	-1.098873367	0.206	0.49	2.89E-13	T cells, CD4+, naive, stimulated
**Rac2**	2.29E-17	0.878245903	0.782	0.717	3.24E-13	T cells, CD4+, naive, stimulated
**Hnrnpk**	3.52E-17	-0.779322286	0.345	0.659	4.98E-13	T cells, CD4+, naive, stimulated
**Cd37**	5.5E-17	0.674275892	0.887	0.865	7.79E-13	T cells, CD4+, naive, stimulated
**Oaz1**	6.4E-17	0.673382289	0.899	0.881	9.06E-13	T cells, CD4+, naive, stimulated
**Cd55**	6.96E-17	-0.691041427	0.353	0.686	9.86E-13	T cells, CD4+, naive, stimulated
**Cd83**	7.4E-17	-0.877665133	0.466	0.747	1.05E-12	T cells, CD4+, naive, stimulated
**Tgfb1**	1.16E-16	-0.791344649	0.303	0.606	1.64E-12	T cells, CD4+, naive, stimulated
**Ptprc**	1.21E-16	-0.876223476	0.34	0.643	1.71E-12	T cells, CD4+, naive, stimulated
**Ly6a**	1.96E-16	1.3032395	0.517	0.327	2.78E-12	T cells, CD4+, naive, stimulated
**Hsp90aa1**	2.2E-16	-0.948206682	0.693	0.854	3.11E-12	T cells, CD4+, naive, stimulated
**Tgfbr2**	2.65E-16	-0.952389263	0.143	0.41	3.75E-12	T cells, CD4+, naive, stimulated
**Rps18**	2.83E-16	0.522552808	0.937	0.929	4.01E-12	T cells, CD4+, naive, stimulated
**Fau**	3.12E-16	0.376016732	1	0.988	4.42E-12	T cells, CD4+, naive, stimulated

**Ptp4a3**	5.85E-16	-0.816722893	0.248	0.556	8.29E-12	T cells, CD4+, naive, stimulated
**Gdi2**	7.01E-16	-0.760184798	0.37	0.667	9.92E-12	T cells, CD4+, naive, stimulated
**Cd9**	8.9E-16	0.477109071	0.134	0.024	1.26E-11	T cells, CD4+, naive, stimulated
**Polr2a**	1.12E-15	-0.968588893	0.202	0.47	1.58E-11	T cells, CD4+, naive, stimulated
**Ppp1r15a**	1.28E-15	-0.896314805	0.324	0.602	1.81E-11	T cells, CD4+, naive, stimulated
**Add3**	1.75E-15	-0.750997521	0.193	0.482	2.48E-11	T cells, CD4+, naive, stimulated
**Cox4i1**	2.9E-15	0.676605998	0.794	0.716	4.11E-11	T cells, CD4+, naive, stimulated
**Csk**	3.55E-15	-0.896201634	0.206	0.476	5.03E-11	T cells, CD4+, naive, stimulated
**Srgn**	3.87E-15	0.544206938	0.95	0.93	5.48E-11	T cells, CD4+, naive, stimulated
**Arhgef1**	5.11E-15	-0.807092423	0.311	0.589	7.23E-11	T cells, CD4+, naive, stimulated
**Eef1b2**	1.11E-14	0.653450327	0.828	0.826	1.57E-10	T cells, CD4+, naive, stimulated
**Tonsl**	1.75E-14	-0.997756728	0.105	0.332	2.47E-10	T cells, CD4+, naive, stimulated
**Neat1**	1.95E-14	-1.048089323	0.185	0.439	2.76E-10	T cells, CD4+, naive, stimulated
**Pim1**	2.13E-14	-0.969042484	0.256	0.52	3.01E-10	T cells, CD4+, naive, stimulated
**Cnn3**	2.31E-14	-1.005970535	0.067	0.297	3.27E-10	T cells, CD4+, naive, stimulated
**Ass1**	2.53E-14	0.502680741	0.151	0.034	3.58E-10	T cells, CD4+, naive, stimulated
**Hnrnph1**	2.82E-14	-0.748706974	0.202	0.469	4E-10	T cells, CD4+, naive, stimulated
**Actr3**	2.93E-14	-0.708034192	0.382	0.68	4.15E-10	T cells, CD4+, naive, stimulated
**Uba52**	3.4E-14	0.335229791	1	0.978	4.82E-10	T cells, CD4+, naive, stimulated
**Sell**	3.72E-14	-0.711667396	0.29	0.572	5.26E-10	T cells, CD4+, naive, stimulated
**Rbm38**	4E-14	-0.798970269	0.235	0.505	5.67E-10	T cells, CD4+, naive, stimulated
**Clk1**	4.1E-14	-0.84721449	0.248	0.519	5.81E-10	T cells, CD4+, naive, stimulated
**Sqstm1**	6.5E-14	-0.706582737	0.34	0.62	9.2E-10	T cells, CD4+, naive, stimulated
**Lamp1**	7.43E-14	-0.906287541	0.139	0.379	1.05E-09	T cells, CD4+, naive, stimulated
**Rplp1**	8.47E-14	0.382646446	0.979	0.97	1.2E-09	T cells, CD4+, naive, stimulated
**AW11201(**	9.24E-14	0.965686755	0.223	0.077	1.31E-09	T cells, CD4+, naive, stimulated
**Brd2**	1.07E-13	-0.885505361	0.256	0.511	1.52E-09	T cells, CD4+, naive, stimulated
**Eif4a1**	1.13E-13	-0.722020419	0.282	0.574	1.59E-09	T cells, CD4+, naive, stimulated
**Snn**	1.14E-13	-0.90324816	0.164	0.419	1.61E-09	T cells, CD4+, naive, stimulated
**Sdcbp**	1.36E-13	-0.936625657	0.13	0.367	1.93E-09	T cells, CD4+, naive, stimulated
**Sf1**	1.79E-13	-0.813799777	0.261	0.524	2.54E-09	T cells, CD4+, naive, stimulated
**Rhoa**	2.29E-13	-0.572785681	0.504	0.795	3.24E-09	T cells, CD4+, naive, stimulated
**Ube2d3**	2.65E-13	-0.680861393	0.403	0.68	3.75E-09	T cells, CD4+, naive, stimulated
**Arf1**	2.8E-13	-0.587828676	0.277	0.551	3.96E-09	T cells, CD4+, naive, stimulated
**Hnrnpa2b**	3.06E-13	-0.595829812	0.475	0.759	4.34E-09	T cells, CD4+, naive, stimulated

**Ccdc28b**	3.6E-13	0.481273393	0.109	0.02	5.09E-09	T cells, CD4+, naive, stimulated
**Sfpq**	3.76E-13	-0.850243884	0.21	0.449	5.32E-09	T cells, CD4+, naive, stimulated
**Cyba**	3.88E-13	0.717520711	0.79	0.751	5.49E-09	T cells, CD4+, naive, stimulated
**Ptp4a1**	4.48E-13	-1.049445321	0.059	0.26	6.35E-09	T cells, CD4+, naive, stimulated
**Cfl1**	4.8E-13	0.510636466	0.891	0.858	6.8E-09	T cells, CD4+, naive, stimulated
**Psme1**	5.02E-13	0.844887047	0.647	0.511	7.11E-09	T cells, CD4+, naive, stimulated
**Lbr**	5.06E-13	-0.969321158	0.088	0.304	7.16E-09	T cells, CD4+, naive, stimulated
**Ubb**	5.08E-13	0.397380918	0.992	0.978	7.18E-09	T cells, CD4+, naive, stimulated
**Tgif1**	5.1E-13	-0.846974252	0.235	0.481	7.22E-09	T cells, CD4+, naive, stimulated
**Btf3**	5.89E-13	0.582602474	0.815	0.809	8.34E-09	T cells, CD4+, naive, stimulated
**Tmsb4x**	6.3E-13	0.397851248	0.992	0.982	8.92E-09	T cells, CD4+, naive, stimulated
**Rpl35**	7.15E-13	0.327866318	0.992	0.965	1.01E-08	T cells, CD4+, naive, stimulated
**Ppia**	7.19E-13	0.436607384	0.971	0.971	1.02E-08	T cells, CD4+, naive, stimulated
**Cd52**	7.93E-13	0.608682467	0.941	0.898	1.12E-08	T cells, CD4+, naive, stimulated
**Rps15a**	7.96E-13	0.326138696	0.992	0.976	1.13E-08	T cells, CD4+, naive, stimulated
**Foxo1**	9.7E-13	-0.898051233	0.16	0.395	1.37E-08	T cells, CD4+, naive, stimulated
**S1pr1**	1.28E-12	-0.876540916	0.273	0.524	1.81E-08	T cells, CD4+, naive, stimulated
**Fam43a**	1.42E-12	-0.942169215	0.16	0.394	2.01E-08	T cells, CD4+, naive, stimulated
**Rps24**	1.73E-12	0.293267234	1	0.98	2.45E-08	T cells, CD4+, naive, stimulated
**Laptm5**	1.95E-12	-0.551523287	0.504	0.783	2.76E-08	T cells, CD4+, naive, stimulated
**Ly6d**	1.97E-12	0.836486203	0.87	0.841	2.79E-08	T cells, CD4+, naive, stimulated
**Cd53**	2.43E-12	-0.659375382	0.328	0.592	3.43E-08	T cells, CD4+, naive, stimulated
**Rasgef1b**	2.57E-12	-0.90704627	0.126	0.355	3.64E-08	T cells, CD4+, naive, stimulated
**Lyn**	2.81E-12	-0.626868233	0.328	0.591	3.97E-08	T cells, CD4+, naive, stimulated
**Srsf3**	3.05E-12	-0.680460462	0.357	0.606	4.32E-08	T cells, CD4+, naive, stimulated
**Hmgn2**	3.29E-12	-0.847557315	0.256	0.497	4.66E-08	T cells, CD4+, naive, stimulated
**Arhgdib**	3.4E-12	0.489003571	0.908	0.873	4.82E-08	T cells, CD4+, naive, stimulated
**Ly6e**	3.85E-12	0.481300722	0.983	0.98	5.45E-08	T cells, CD4+, naive, stimulated
**Fosb**	3.88E-12	-1.186131791	0.189	0.41	5.5E-08	T cells, CD4+, naive, stimulated
**Pfn1**	4.19E-12	0.538865675	0.95	0.938	5.94E-08	T cells, CD4+, naive, stimulated
**Pxk**	4.87E-12	-0.834098622	0.143	0.366	6.89E-08	T cells, CD4+, naive, stimulated
**Eif4a2**	5.18E-12	-0.784160774	0.256	0.497	7.34E-08	T cells, CD4+, naive, stimulated
**Tob1**	5.35E-12	-0.946749197	0.101	0.299	7.57E-08	T cells, CD4+, naive, stimulated
**Arpc3**	5.73E-12	0.607465369	0.777	0.701	8.11E-08	T cells, CD4+, naive, stimulated
**Akap13**	5.92E-12	-0.692276837	0.311	0.554	8.38E-08	T cells, CD4+, naive, stimulated

**Cpm**	6.04E-12	-0.952884328	0.08	0.291	8.54E-08	T cells, CD4+, naive, stimulated
**Gga2**	7.38E-12	-0.780785736	0.092	0.306	1.04E-07	T cells, CD4+, naive, stimulated
**Psmb8**	7.4E-12	0.864291608	0.66	0.57	1.05E-07	T cells, CD4+, naive, stimulated
**Rpl41**	8.82E-12	0.516783615	0.849	0.875	1.25E-07	T cells, CD4+, naive, stimulated
**Crlf3**	1.17E-11	-0.759413236	0.193	0.42	1.65E-07	T cells, CD4+, naive, stimulated
**Dmxl1**	1.28E-11	-0.927563505	0.168	0.397	1.81E-07	T cells, CD4+, naive, stimulated
**Dusp5**	1.31E-11	-0.936966683	0.202	0.432	1.86E-07	T cells, CD4+, naive, stimulated
**Btg2**	1.37E-11	-0.730707997	0.487	0.7	1.93E-07	T cells, CD4+, naive, stimulated
**Fus**	1.69E-11	-0.678967869	0.277	0.516	2.39E-07	T cells, CD4+, naive, stimulated
**Cyth1**	2.13E-11	-0.798717265	0.197	0.423	3.02E-07	T cells, CD4+, naive, stimulated
**Zfp36l1**	2.28E-11	-0.538947657	0.639	0.835	3.23E-07	T cells, CD4+, naive, stimulated
**Pcbp1**	2.74E-11	-0.611272433	0.395	0.64	3.88E-07	T cells, CD4+, naive, stimulated
**Eif2ak3**	3.36E-11	-0.850052076	0.105	0.315	4.76E-07	T cells, CD4+, naive, stimulated
**Rpl22**	3.76E-11	0.357191723	0.962	0.952	5.32E-07	T cells, CD4+, naive, stimulated
**Capg**	3.77E-11	0.82869496	0.534	0.382	5.34E-07	T cells, CD4+, naive, stimulated
**Nme2**	3.9E-11	0.804208982	0.664	0.592	5.53E-07	T cells, CD4+, naive, stimulated
**Prkcb**	4.12E-11	-0.673535719	0.256	0.491	5.83E-07	T cells, CD4+, naive, stimulated
**Gpr171**	4.23E-11	-0.973594531	0.16	0.377	5.99E-07	T cells, CD4+, naive, stimulated
**Eif3f**	4.24E-11	0.596888883	0.845	0.854	6.01E-07	T cells, CD4+, naive, stimulated
**Erp29**	4.99E-11	0.690227768	0.584	0.444	7.06E-07	T cells, CD4+, naive, stimulated
**Foxp1**	5.23E-11	-0.545735368	0.521	0.785	7.41E-07	T cells, CD4+, naive, stimulated
**Sh3bp5**	6.27E-11	-0.779315786	0.164	0.379	8.88E-07	T cells, CD4+, naive, stimulated
**Cebpb**	6.39E-11	-0.877687428	0.265	0.491	9.04E-07	T cells, CD4+, naive, stimulated
**Kctd12**	6.62E-11	-0.889591242	0.105	0.301	9.37E-07	T cells, CD4+, naive, stimulated
**Tsc22d1**	7.44E-11	-1.147605875	0.092	0.299	1.05E-06	T cells, CD4+, naive, stimulated
**Khdrbs1**	7.55E-11	-0.776247568	0.13	0.334	1.07E-06	T cells, CD4+, naive, stimulated
**Gm8797**	7.62E-11	0.929835116	0.387	0.217	1.08E-06	T cells, CD4+, naive, stimulated
**Ppp1cc**	1.02E-10	-0.653481009	0.193	0.402	1.44E-06	T cells, CD4+, naive, stimulated
**Timm8a1**	1.11E-10	0.710677943	0.122	0.031	1.58E-06	T cells, CD4+, naive, stimulated
**Rnf187**	1.13E-10	-0.785377895	0.176	0.379	1.6E-06	T cells, CD4+, naive, stimulated
**Cd24a**	1.18E-10	-0.720342259	0.37	0.614	1.67E-06	T cells, CD4+, naive, stimulated
**Elmsan1**	1.18E-10	-0.990125611	0.13	0.319	1.67E-06	T cells, CD4+, naive, stimulated
**Mzb1**	1.36E-10	0.894223929	0.571	0.476	1.92E-06	T cells, CD4+, naive, stimulated
**Fam49b**	1.39E-10	-0.735813989	0.231	0.442	1.96E-06	T cells, CD4+, naive, stimulated
**Hnrnpab**	1.43E-10	-0.661668349	0.298	0.535	2.03E-06	T cells, CD4+, naive, stimulated

**H2-T23**	1.57E-10	-0.692962282	0.277	0.516	2.22E-06	T cells, CD4+, naive, stimulated
**Jun**	1.81E-10	-0.962637266	0.307	0.533	2.56E-06	T cells, CD4+, naive, stimulated
**Man1a**	1.85E-10	-0.885227804	0.176	0.363	2.61E-06	T cells, CD4+, naive, stimulated
**Cxcr5**	2.16E-10	-0.850591872	0.185	0.383	3.06E-06	T cells, CD4+, naive, stimulated
**B2m**	2.34E-10	0.393322084	0.899	0.913	3.31E-06	T cells, CD4+, naive, stimulated
**Wsb1**	2.52E-10	-0.811799002	0.126	0.317	3.57E-06	T cells, CD4+, naive, stimulated
**Lcp1**	3.53E-10	-0.594360515	0.399	0.642	5E-06	T cells, CD4+, naive, stimulated
**Rps14**	3.89E-10	0.358192211	0.945	0.961	5.5E-06	T cells, CD4+, naive, stimulated
**Slbp**	4.56E-10	-0.745852892	0.172	0.371	6.45E-06	T cells, CD4+, naive, stimulated
**Bank1**	4.9E-10	-0.540008663	0.353	0.603	6.94E-06	T cells, CD4+, naive, stimulated
**Rnf145**	5.1E-10	-0.803596504	0.076	0.252	7.22E-06	T cells, CD4+, naive, stimulated
**Stk24**	5.13E-10	-0.633393943	0.261	0.484	7.27E-06	T cells, CD4+, naive, stimulated
**Serf2**	5.25E-10	0.504259949	0.84	0.841	7.43E-06	T cells, CD4+, naive, stimulated
**Gm26532**	5.68E-10	-0.902892762	0.13	0.325	8.04E-06	T cells, CD4+, naive, stimulated
**Capza1**	6.53E-10	-0.679387213	0.164	0.359	9.25E-06	T cells, CD4+, naive, stimulated
**Rpl7**	7.35E-10	0.355772156	0.937	0.936	1.04E-05	T cells, CD4+, naive, stimulated
**Nrros**	7.62E-10	-0.889928206	0.185	0.374	1.08E-05	T cells, CD4+, naive, stimulated
**Fmnl1**	7.96E-10	-0.732278599	0.176	0.371	1.13E-05	T cells, CD4+, naive, stimulated
**Tmed2**	8.57E-10	-0.63115109	0.244	0.461	1.21E-05	T cells, CD4+, naive, stimulated
**Taf6l**	8.82E-10	-0.851304339	0.092	0.264	1.25E-05	T cells, CD4+, naive, stimulated
**Emb**	9.01E-10	0.464380989	0.101	0.023	1.28E-05	T cells, CD4+, naive, stimulated
**Rpl30**	9.89E-10	0.282487123	0.996	0.978	1.4E-05	T cells, CD4+, naive, stimulated
**Tnfaip3**	1.12E-09	-1.046040132	0.143	0.322	1.58E-05	T cells, CD4+, naive, stimulated
**Calm1**	1.13E-09	0.545598802	0.845	0.839	1.61E-05	T cells, CD4+, naive, stimulated
**Adgre5**	1.15E-09	-0.631970805	0.303	0.533	1.62E-05	T cells, CD4+, naive, stimulated
**Ptms**	1.22E-09	0.491253999	0.155	0.051	1.73E-05	T cells, CD4+, naive, stimulated
**Myh9**	1.4E-09	-0.55075197	0.34	0.565	1.99E-05	T cells, CD4+, naive, stimulated
**Kdm6b**	1.52E-09	-0.873046176	0.16	0.341	2.15E-05	T cells, CD4+, naive, stimulated
**Npc2**	1.71E-09	0.780712004	0.42	0.261	2.42E-05	T cells, CD4+, naive, stimulated
**Matr3**	1.77E-09	-0.653658386	0.181	0.384	2.5E-05	T cells, CD4+, naive, stimulated
**Rpl7a**	1.8E-09	0.431920131	0.866	0.899	2.54E-05	T cells, CD4+, naive, stimulated
**Gm10076**	1.8E-09	0.254080506	0.987	0.978	2.55E-05	T cells, CD4+, naive, stimulated
**Pou2af1**	2.04E-09	-0.669673827	0.172	0.375	2.88E-05	T cells, CD4+, naive, stimulated
**Peli1**	2.12E-09	-0.707719795	0.147	0.337	2.99E-05	T cells, CD4+, naive, stimulated
**Rsrp1**	2.13E-09	-0.716766408	0.197	0.397	3.01E-05	T cells, CD4+, naive, stimulated

**Pmaip1**	2.16E-09	-0.915406465	0.097	0.271	3.06E-05	T cells, CD4+, naive, stimulated
**Rps19**	2.53E-09	0.306372103	0.987	0.968	3.59E-05	T cells, CD4+, naive, stimulated
**Il2rg**	2.66E-09	-0.439558186	0.546	0.767	3.76E-05	T cells, CD4+, naive, stimulated
**Ctnnb1**	2.73E-09	-0.83078489	0.105	0.282	3.86E-05	T cells, CD4+, naive, stimulated
**Brwd1**	2.76E-09	-0.763533831	0.067	0.239	3.9E-05	T cells, CD4+, naive, stimulated
**Cbx4**	2.84E-09	-0.734429704	0.067	0.235	4.01E-05	T cells, CD4+, naive, stimulated
**Mcl1**	3.35E-09	-0.650420338	0.294	0.504	4.75E-05	T cells, CD4+, naive, stimulated
**Herpud1**	3.38E-09	-0.863374118	0.151	0.329	4.78E-05	T cells, CD4+, naive, stimulated
**Oser1**	3.6E-09	-0.740662257	0.143	0.316	5.1E-05	T cells, CD4+, naive, stimulated
**Snx9**	3.7E-09	-0.684716165	0.193	0.384	5.24E-05	T cells, CD4+, naive, stimulated
**Sh3bgrl3**	3.78E-09	0.444220562	0.836	0.836	5.35E-05	T cells, CD4+, naive, stimulated
**Polr2m**	3.86E-09	-0.7125748	0.067	0.234	5.47E-05	T cells, CD4+, naive, stimulated
**Fchsd2**	4.42E-09	-0.641405379	0.164	0.37	6.25E-05	T cells, CD4+, naive, stimulated
**Sat1**	4.43E-09	0.725280179	0.508	0.358	6.27E-05	T cells, CD4+, naive, stimulated
**Calr**	4.97E-09	-0.672364422	0.206	0.407	7.04E-05	T cells, CD4+, naive, stimulated
**Hnrnph2**	5.26E-09	-0.732756628	0.113	0.288	7.45E-05	T cells, CD4+, naive, stimulated
**Ptk2b**	5.35E-09	-0.703493467	0.134	0.31	7.58E-05	T cells, CD4+, naive, stimulated
**Srek1**	6.44E-09	0.570867148	0.134	0.043	9.11E-05	T cells, CD4+, naive, stimulated
**Tpm4**	6.56E-09	-0.697041198	0.214	0.396	9.28E-05	T cells, CD4+, naive, stimulated
**C130026I2**	6.6E-09	0.515959934	0.122	0.036	9.35E-05	T cells, CD4+, naive, stimulated
**Zfp318**	6.74E-09	-0.569825642	0.071	0.248	9.54E-05	T cells, CD4+, naive, stimulated
**Trim25**	6.91E-09	-0.546404956	0.189	0.398	9.79E-05	T cells, CD4+, naive, stimulated
**Sec61b**	7.26E-09	0.667413161	0.592	0.479	0.0001027	T cells, CD4+, naive, stimulated
**Rpl17**	7.44E-09	-0.275821737	0.95	0.962	0.0001052	T cells, CD4+, naive, stimulated
**Trp53i11**	7.47E-09	-0.650806321	0.155	0.346	0.0001057	T cells, CD4+, naive, stimulated
**Rassf4**	7.62E-09	0.487873472	0.172	0.062	0.0001078	T cells, CD4+, naive, stimulated
**Lmo2**	8.61E-09	-0.740375277	0.055	0.209	0.0001218	T cells, CD4+, naive, stimulated
**Prrc2a**	9.12E-09	-0.694597397	0.126	0.301	0.0001290	T cells, CD4+, naive, stimulated
**Ncf2**	1.02E-08	-0.633257296	0.147	0.324	0.0001439	T cells, CD4+, naive, stimulated
**Tmem123**	1.04E-08	-0.585561791	0.239	0.45	0.0001478	T cells, CD4+, naive, stimulated
**Ptbp3**	1.17E-08	-0.586757664	0.307	0.499	0.0001650	T cells, CD4+, naive, stimulated
**Prdx1**	1.17E-08	0.768234147	0.576	0.474	0.0001653	T cells, CD4+, naive, stimulated
**Prr13**	1.17E-08	0.811611529	0.471	0.323	0.0001662	T cells, CD4+, naive, stimulated
**B4galnt1**	1.19E-08	-0.634515512	0.214	0.404	0.0001689	T cells, CD4+, naive, stimulated
**Mknk2**	1.21E-08	-0.712836973	0.134	0.301	0.0001715	T cells, CD4+, naive, stimulated

**Irf4**	1.22E-08	-0.575063486	0.143	0.33	0.0001720[illegible]	T cells, CD4+, naive, stimulated
**Hist1h2ap**	1.23E-08	-0.788328074	0.143	0.317	0.0001746[illegible]	T cells, CD4+, naive, stimulated
**Rpl34**	1.32E-08	0.250633863	0.992	0.972	0.0001871[illegible]	T cells, CD4+, naive, stimulated
**Nfatc3**	1.37E-08	-0.754826864	0.168	0.345	0.0001933[illegible]	T cells, CD4+, naive, stimulated
**Mef2d**	1.43E-08	-0.767840229	0.143	0.304	0.0002030[illegible]	T cells, CD4+, naive, stimulated
**Ywhaq**	1.54E-08	-0.581056901	0.202	0.391	0.0002176[illegible]	T cells, CD4+, naive, stimulated
**Evi2a**	1.55E-08	0.317947436	0.134	0.043	0.0002195[illegible]	T cells, CD4+, naive, stimulated
**Msn**	1.56E-08	-0.391167332	0.462	0.696	0.0002209[illegible]	T cells, CD4+, naive, stimulated
**Srsf2**	1.62E-08	-0.568593419	0.324	0.526	0.0002288[illegible]	T cells, CD4+, naive, stimulated
**Psme2**	1.65E-08	0.78904159	0.454	0.311	0.0002337[illegible]	T cells, CD4+, naive, stimulated
**Eif4g2**	1.67E-08	-0.43784648	0.374	0.601	0.0002357[illegible]	T cells, CD4+, naive, stimulated
**Ndufb11**	1.76E-08	0.640192311	0.563	0.427	0.0002491[illegible]	T cells, CD4+, naive, stimulated
**Csnk2b**	1.89E-08	0.732358635	0.387	0.242	0.0002679[illegible]	T cells, CD4+, naive, stimulated
**Irs2**	1.9E-08	-0.803160441	0.118	0.283	0.0002694[illegible]	T cells, CD4+, naive, stimulated
**Amd1**	1.95E-08	-0.713241801	0.084	0.24	0.0002765[illegible]	T cells, CD4+, naive, stimulated
**Dnaja1**	2E-08	-0.540930346	0.592	0.762	0.0002837[illegible]	T cells, CD4+, naive, stimulated
**Tpt1**	2.04E-08	0.267039194	1	0.97	0.0002882[illegible]	T cells, CD4+, naive, stimulated
**Atf4**	2.1E-08	-0.578199792	0.277	0.475	0.0002968[illegible]	T cells, CD4+, naive, stimulated
**Tob2**	2.1E-08	-0.728259832	0.197	0.378	0.0002975[illegible]	T cells, CD4+, naive, stimulated
**Lgals3**	2.12E-08	0.470272729	0.122	0.037	0.0003002[illegible]	T cells, CD4+, naive, stimulated
**Ivns1abp**	2.15E-08	-0.747495569	0.08	0.232	0.0003050[illegible]	T cells, CD4+, naive, stimulated
**Hnrnpl**	2.26E-08	-0.60249463	0.269	0.462	0.0003196[illegible]	T cells, CD4+, naive, stimulated
**Tagap**	2.58E-08	-0.830677414	0.092	0.244	0.0003657[illegible]	T cells, CD4+, naive, stimulated
**Wipf1**	2.77E-08	-0.521738923	0.071	0.231	0.0003921[illegible]	T cells, CD4+, naive, stimulated
**Blk**	3E-08	-0.521397489	0.307	0.521	0.0004244[illegible]	T cells, CD4+, naive, stimulated
**Mrfap1**	3.4E-08	-0.66053052	0.223	0.39	0.0004807[illegible]	T cells, CD4+, naive, stimulated
**Abhd17b**	3.54E-08	-0.570813545	0.214	0.393	0.0005012[illegible]	T cells, CD4+, naive, stimulated
**Mat2a**	3.67E-08	-0.640751792	0.244	0.422	0.0005195[illegible]	T cells, CD4+, naive, stimulated
**Cd164**	3.76E-08	-0.689958106	0.134	0.295	0.0005322[illegible]	T cells, CD4+, naive, stimulated
**Unc93b1**	3.93E-08	-0.502268249	0.416	0.627	0.0005559[illegible]	T cells, CD4+, naive, stimulated
**Gpr132**	4.37E-08	-0.756691474	0.147	0.317	0.0006188[illegible]	T cells, CD4+, naive, stimulated
**Gpr137b**	4.68E-08	0.406358401	0.134	0.044	0.0006624[illegible]	T cells, CD4+, naive, stimulated
**Psmb9**	4.87E-08	0.922631179	0.366	0.227	0.0006897[illegible]	T cells, CD4+, naive, stimulated
**Tiparp**	5.21E-08	-0.811792906	0.105	0.258	0.0007369[illegible]	T cells, CD4+, naive, stimulated
**Nrbp1**	5.4E-08	-0.663589739	0.151	0.31	0.0007640[illegible]	T cells, CD4+, naive, stimulated

**Npm1**	5.4E-08	0.540716274	0.718	0.717	0.00076470	T cells, CD4+, naive, stimulated
**Fos**	5.73E-08	-0.770555192	0.479	0.635	0.00081085	T cells, CD4+, naive, stimulated
**Il16**	5.88E-08	-0.659463046	0.067	0.213	0.00083197	T cells, CD4+, naive, stimulated
**Mef2c**	6.27E-08	-0.436894126	0.517	0.728	0.00088806	T cells, CD4+, naive, stimulated
**Morf4l2**	6.61E-08	-0.677839368	0.084	0.232	0.00093586	T cells, CD4+, naive, stimulated
**Ptprcap**	6.85E-08	0.614354323	0.668	0.61	0.00096927	T cells, CD4+, naive, stimulated
**Napsa**	6.99E-08	0.732851589	0.63	0.566	0.00098965	T cells, CD4+, naive, stimulated
**Gem**	7.01E-08	-0.678313996	0.214	0.394	0.00099270	T cells, CD4+, naive, stimulated
**Dnajb6**	7.84E-08	-0.671091757	0.181	0.354	0.00110920	T cells, CD4+, naive, stimulated
**Ppp3ca**	7.9E-08	-0.564253539	0.261	0.456	0.00111761	T cells, CD4+, naive, stimulated
**Iqgap1**	8.42E-08	-0.494951251	0.345	0.551	0.00119183	T cells, CD4+, naive, stimulated
**Ehd1**	8.53E-08	-0.748102624	0.109	0.262	0.00120776	T cells, CD4+, naive, stimulated
**Mfap1b**	9.49E-08	-0.581286641	0.092	0.25	0.00134359	T cells, CD4+, naive, stimulated
**Rnasek**	1.11E-07	0.721798071	0.345	0.205	0.00157621	T cells, CD4+, naive, stimulated
**Azin1**	1.13E-07	-0.626635057	0.088	0.238	0.00160037	T cells, CD4+, naive, stimulated
**Phip**	1.2E-07	-0.588849876	0.139	0.308	0.00169883	T cells, CD4+, naive, stimulated
**Atp5g2**	1.2E-07	0.498692301	0.697	0.68	0.00170401	T cells, CD4+, naive, stimulated
**Rpl4**	1.26E-07	-0.39763893	0.676	0.852	0.00178020	T cells, CD4+, naive, stimulated
**Ubqln1**	1.3E-07	-0.601812022	0.092	0.244	0.00184087	T cells, CD4+, naive, stimulated
**Oat**	1.31E-07	-0.506229494	0.097	0.247	0.00185709	T cells, CD4+, naive, stimulated
**Hint1**	1.35E-07	0.633901657	0.563	0.474	0.00190693	T cells, CD4+, naive, stimulated
**Ier2**	1.36E-07	-0.45874334	0.651	0.82	0.00192038	T cells, CD4+, naive, stimulated
**Tm9sf2**	1.38E-07	-0.768490456	0.08	0.216	0.00194743	T cells, CD4+, naive, stimulated
**Inpp5d**	1.4E-07	-0.507782147	0.261	0.46	0.00198767	T cells, CD4+, naive, stimulated
**Cerk**	1.42E-07	-0.451145456	0.256	0.449	0.00200827	T cells, CD4+, naive, stimulated
**Atp5b**	1.43E-07	-0.446108641	0.324	0.516	0.00202877	T cells, CD4+, naive, stimulated
**Cd22**	1.51E-07	-0.494456182	0.151	0.321	0.00214165	T cells, CD4+, naive, stimulated
**Eif3i**	1.57E-07	0.668943103	0.496	0.369	0.00221662	T cells, CD4+, naive, stimulated
**Rpl5**	1.6E-07	0.460211519	0.849	0.85	0.00227069	T cells, CD4+, naive, stimulated
**Pafah1b3**	1.61E-07	0.581115354	0.282	0.145	0.00227350	T cells, CD4+, naive, stimulated
**Arid5a**	1.61E-07	-0.61093519	0.189	0.352	0.00227720	T cells, CD4+, naive, stimulated
**Ssh2**	1.62E-07	-0.633941928	0.155	0.331	0.00229695	T cells, CD4+, naive, stimulated
**Neurl3**	1.64E-07	-0.487856742	0.113	0.285	0.00232035	T cells, CD4+, naive, stimulated
**Nfkb2**	1.69E-07	-0.632139183	0.105	0.253	0.00238658	T cells, CD4+, naive, stimulated
**Sik1**	1.88E-07	-0.611916437	0.21	0.374	0.00265485	T cells, CD4+, naive, stimulated

**Rpl3**	1.93E-07	0.3067768	0.945	0.948	0.00272765	T cells, CD4+, naive, stimulated
**Sdhc**	1.99E-07	0.481382042	0.134	0.049	0.00282071	T cells, CD4+, naive, stimulated
**Cat**	2E-07	-0.581011141	0.08	0.222	0.00283284	T cells, CD4+, naive, stimulated
**Sesn3**	2E-07	-0.538974388	0.067	0.211	0.00283326	T cells, CD4+, naive, stimulated
**Smdt1**	2.01E-07	0.688880954	0.399	0.261	0.00284797	T cells, CD4+, naive, stimulated
**Trp53inp2**	2.16E-07	-0.71117798	0.084	0.223	0.00305331	T cells, CD4+, naive, stimulated
**Traf4**	2.17E-07	-0.623475173	0.109	0.258	0.00307831	T cells, CD4+, naive, stimulated
**Adipor1**	2.22E-07	-0.471153096	0.164	0.328	0.00314845	T cells, CD4+, naive, stimulated
**Pax5**	2.24E-07	-0.603292407	0.176	0.346	0.00317038	T cells, CD4+, naive, stimulated
**Rnf167**	2.3E-07	-0.625408198	0.122	0.274	0.00325794	T cells, CD4+, naive, stimulated
**Bcl2a1b**	2.46E-07	0.76818499	0.218	0.107	0.00348837	T cells, CD4+, naive, stimulated
**Hvcn1**	2.48E-07	-0.493591801	0.193	0.371	0.00351347	T cells, CD4+, naive, stimulated
**Txndc16**	2.58E-07	-0.620674009	0.084	0.231	0.00365772	T cells, CD4+, naive, stimulated
**Map2k1**	2.62E-07	-0.573279973	0.101	0.245	0.00371367	T cells, CD4+, naive, stimulated
**Ago2**	2.71E-07	-0.519817893	0.088	0.235	0.00384121	T cells, CD4+, naive, stimulated
**Stip1**	2.83E-07	-0.568082257	0.151	0.315	0.00400875	T cells, CD4+, naive, stimulated
**Cacybp**	2.86E-07	-0.574963246	0.223	0.4	0.00404472	T cells, CD4+, naive, stimulated
**Tram1**	2.87E-07	-0.624273449	0.109	0.257	0.00405683	T cells, CD4+, naive, stimulated
**Nxf1**	2.98E-07	-0.671890176	0.109	0.251	0.00421500	T cells, CD4+, naive, stimulated
**AI467606**	3.11E-07	-0.66292959	0.076	0.215	0.00439885	T cells, CD4+, naive, stimulated
**Ralgps2**	3.27E-07	-0.460127329	0.349	0.556	0.00463009	T cells, CD4+, naive, stimulated
**Plaur**	3.4E-07	-0.787392558	0.239	0.407	0.00480744	T cells, CD4+, naive, stimulated
**Tra2b**	3.46E-07	-0.487113787	0.378	0.547	0.00490165	T cells, CD4+, naive, stimulated
**Rps6**	3.57E-07	0.382796276	0.857	0.858	0.00505120	T cells, CD4+, naive, stimulated
**Prkcd**	3.65E-07	-0.654060656	0.13	0.278	0.00516448	T cells, CD4+, naive, stimulated
**Atp5c1**	4.06E-07	0.59439396	0.542	0.428	0.00574787	T cells, CD4+, naive, stimulated
**St6gal1**	4.15E-07	-0.528113998	0.143	0.301	0.00587047	T cells, CD4+, naive, stimulated
**B3gnt5**	4.17E-07	-0.646583836	0.181	0.341	0.00590705	T cells, CD4+, naive, stimulated
**Rbm7**	4.44E-07	-0.585340148	0.088	0.223	0.00628626	T cells, CD4+, naive, stimulated
**Stt3b**	4.47E-07	-0.660305622	0.206	0.364	0.00633246	T cells, CD4+, naive, stimulated
**Lrrk2**	4.48E-07	-0.547662574	0.063	0.202	0.00633885	T cells, CD4+, naive, stimulated
**Rassf3**	4.6E-07	-0.648917825	0.038	0.156	0.00650503	T cells, CD4+, naive, stimulated
**Hspd1**	4.86E-07	-0.706157243	0.412	0.573	0.00687514	T cells, CD4+, naive, stimulated
**Sipa1**	5.1E-07	-0.575815236	0.139	0.295	0.00721991	T cells, CD4+, naive, stimulated
**G3bp1**	5.14E-07	-0.667327244	0.134	0.274	0.00728173	T cells, CD4+, naive, stimulated

**Adrb2**	5.15E-07	-0.647442758	0.109	0.253	0.0072931	T cells, CD4+, naive, stimulated
**Usp38**	5.16E-07	-0.535277119	0.05	0.176	0.0073017	T cells, CD4+, naive, stimulated
**Siah2**	5.17E-07	-0.61458342	0.092	0.226	0.0073245	T cells, CD4+, naive, stimulated
**Tspan13**	5.4E-07	-0.519614076	0.294	0.457	0.0076476	T cells, CD4+, naive, stimulated
**Gtf3c6**	5.55E-07	0.519206535	0.176	0.077	0.0078584	T cells, CD4+, naive, stimulated
**Fli1**	5.79E-07	-0.502379748	0.176	0.344	0.0082027	T cells, CD4+, naive, stimulated
**Macf1**	6.22E-07	-0.465308319	0.311	0.489	0.0088062	T cells, CD4+, naive, stimulated
**Zc3hav1**	6.31E-07	-0.623550627	0.126	0.272	0.0089322	T cells, CD4+, naive, stimulated
**Per1**	6.45E-07	-0.57506831	0.172	0.328	0.0091295	T cells, CD4+, naive, stimulated
**Rel**	6.5E-07	-0.440891457	0.538	0.698	0.0092072	T cells, CD4+, naive, stimulated
**Esyt1**	6.96E-07	-0.585887013	0.105	0.241	0.0098592	T cells, CD4+, naive, stimulated
**Mdh1**	7.12E-07	0.620982613	0.424	0.295	0.0100837	T cells, CD4+, naive, stimulated
**Atp5f1**	7.65E-07	0.608511571	0.521	0.415	0.0108341	T cells, CD4+, naive, stimulated
**Plekhm1**	7.81E-07	-0.577856201	0.055	0.18	0.0110491	T cells, CD4+, naive, stimulated
**Ptp4a2**	7.97E-07	-0.512338756	0.214	0.381	0.0112770	T cells, CD4+, naive, stimulated
**Zc3h12a**	8.65E-07	-0.71609983	0.097	0.229	0.0122408	T cells, CD4+, naive, stimulated
**Nme3**	8.67E-07	0.403020266	0.109	0.037	0.0122781	T cells, CD4+, naive, stimulated
**Pgap1**	8.7E-07	-0.581113564	0.088	0.229	0.0123095	T cells, CD4+, naive, stimulated
**Ctbp1**	8.72E-07	-0.560879888	0.16	0.301	0.0123461	T cells, CD4+, naive, stimulated
**Samhd1**	8.77E-07	-0.459483461	0.353	0.527	0.0124160	T cells, CD4+, naive, stimulated
**Vars**	9E-07	-0.542707129	0.193	0.343	0.0127415	T cells, CD4+, naive, stimulated
**Ifrd1**	9.62E-07	-0.621450369	0.193	0.358	0.0136149	T cells, CD4+, naive, stimulated
**Pycard**	1.02E-06	0.449142552	0.164	0.07	0.0144871	T cells, CD4+, naive, stimulated
**Ewsr1**	1.04E-06	-0.552366209	0.176	0.325	0.0146890	T cells, CD4+, naive, stimulated
**Gimap6**	1.1E-06	-0.509902557	0.462	0.636	0.0155187	T cells, CD4+, naive, stimulated
**Srsf5**	1.13E-06	-0.416694771	0.391	0.576	0.0159316	T cells, CD4+, naive, stimulated
**Tcf3**	1.19E-06	-0.700619622	0.16	0.298	0.0168889	T cells, CD4+, naive, stimulated
**Ap2m1**	1.21E-06	-0.503147755	0.172	0.318	0.0171556	T cells, CD4+, naive, stimulated
**Sgms1**	1.24E-06	-0.56399821	0.084	0.217	0.0175856	T cells, CD4+, naive, stimulated
**Myo1c**	1.25E-06	-0.70105206	0.109	0.237	0.0177636	T cells, CD4+, naive, stimulated
**Tmem14c**	1.27E-06	0.690215876	0.286	0.164	0.0179120	T cells, CD4+, naive, stimulated
**Crip1**	1.27E-06	0.961224445	0.66	0.619	0.0179242	T cells, CD4+, naive, stimulated
**Csnk1g3**	1.28E-06	-0.625634951	0.13	0.272	0.0180859	T cells, CD4+, naive, stimulated
**Eif5a**	1.28E-06	0.699907202	0.63	0.608	0.0181376	T cells, CD4+, naive, stimulated
**Cox5a**	1.31E-06	0.622859914	0.538	0.439	0.0184797	T cells, CD4+, naive, stimulated

**Canx**	1.31E-06	-0.611418429	0.143	0.285	0.0186089	T cells, CD4+, naive, stimulated
**Bcl2a1d**	1.34E-06	0.524702012	0.122	0.045	0.0190381	T cells, CD4+, naive, stimulated
**Atp5g3**	1.39E-06	0.552337335	0.626	0.566	0.0197419	T cells, CD4+, naive, stimulated
**Ubald2**	1.4E-06	-0.729262259	0.189	0.331	0.0197982	T cells, CD4+, naive, stimulated
**Lpgat1**	1.4E-06	-0.586752925	0.071	0.195	0.0198866	T cells, CD4+, naive, stimulated
**Tfrc**	1.42E-06	-0.531122441	0.017	0.119	0.0201542	T cells, CD4+, naive, stimulated
**Birc3**	1.47E-06	-0.655283974	0.227	0.374	0.0208326	T cells, CD4+, naive, stimulated
**Ets2**	1.49E-06	-0.742971205	0.105	0.234	0.0210233	T cells, CD4+, naive, stimulated
**Lrp10**	1.58E-06	-0.577771342	0.176	0.332	0.0223306	T cells, CD4+, naive, stimulated
**Mrpl57**	1.58E-06	0.53349823	0.231	0.118	0.0223510	T cells, CD4+, naive, stimulated
**Mirt1**	1.69E-06	-0.634733111	0.071	0.192	0.0238811	T cells, CD4+, naive, stimulated
**Cuta**	1.69E-06	0.536459687	0.298	0.172	0.0238883	T cells, CD4+, naive, stimulated
**Srp54b**	1.69E-06	-0.618991335	0.038	0.147	0.0239030	T cells, CD4+, naive, stimulated
**Aldoa**	1.69E-06	0.551321154	0.613	0.545	0.0239603	T cells, CD4+, naive, stimulated
**Sptbn1**	1.74E-06	-0.624530616	0.155	0.297	0.0246205	T cells, CD4+, naive, stimulated
**Atp5o**	1.75E-06	0.656796462	0.433	0.314	0.0247882	T cells, CD4+, naive, stimulated
**Setd2**	1.76E-06	-0.659008145	0.08	0.203	0.0249258	T cells, CD4+, naive, stimulated
**Bcl10**	1.8E-06	-0.552277971	0.214	0.368	0.0254363	T cells, CD4+, naive, stimulated
**Rnf144a**	1.85E-06	-0.465763369	0.021	0.128	0.0262184	T cells, CD4+, naive, stimulated
**Eif4b**	1.91E-06	-0.432613696	0.16	0.312	0.0270766	T cells, CD4+, naive, stimulated
**Tprgl**	1.94E-06	-0.583702081	0.139	0.276	0.0274974	T cells, CD4+, naive, stimulated
**H2-DMb1**	2.01E-06	-0.399504729	0.185	0.358	0.0283976	T cells, CD4+, naive, stimulated
**Nfkb1**	2.02E-06	-0.615245173	0.105	0.233	0.0285849	T cells, CD4+, naive, stimulated
**Ccm2**	2.03E-06	-0.528069619	0.139	0.282	0.0287652	T cells, CD4+, naive, stimulated
**Tubb4b**	2.04E-06	-0.309148325	0.357	0.558	0.0289401	T cells, CD4+, naive, stimulated
**Ddx3x**	2.1E-06	-0.624713905	0.168	0.308	0.0297059	T cells, CD4+, naive, stimulated
**Ppp1cb**	2.15E-06	-0.460256355	0.088	0.221	0.0303828	T cells, CD4+, naive, stimulated
**M6pr**	2.17E-06	-0.555657208	0.13	0.268	0.0307400	T cells, CD4+, naive, stimulated
**Plekha2**	2.19E-06	-0.519475644	0.227	0.378	0.0310604	T cells, CD4+, naive, stimulated
**Epn1**	2.43E-06	-0.656084448	0.164	0.293	0.0343685	T cells, CD4+, naive, stimulated
**Rps23**	2.56E-06	0.267425912	0.958	0.932	0.0362385	T cells, CD4+, naive, stimulated
**Atxn2l**	2.58E-06	-0.634249014	0.109	0.238	0.0365627	T cells, CD4+, naive, stimulated
**Rpl10**	2.63E-06	-0.335043503	0.58	0.784	0.0372254	T cells, CD4+, naive, stimulated
**Supt5**	2.65E-06	-0.638286553	0.076	0.195	0.0375492	T cells, CD4+, naive, stimulated
**Med13**	2.81E-06	-0.61905881	0.092	0.216	0.0397899	T cells, CD4+, naive, stimulated

**Tpm3**	2.87E-06	-0.360923637	0.353	0.533	0.0406932[illegible]	T cells, CD4+, naive, stimulated
**Scand1**	2.89E-06	0.620812787	0.508	0.402	0.0408464[illegible]	T cells, CD4+, naive, stimulated
**Coq10b**	2.89E-06	-0.604325474	0.181	0.321	0.0408778[illegible]	T cells, CD4+, naive, stimulated
**Cmah**	2.9E-06	-0.529159189	0.126	0.264	0.0410358[illegible]	T cells, CD4+, naive, stimulated
**Neu1**	2.91E-06	-0.585586477	0.084	0.208	0.0412431[illegible]	T cells, CD4+, naive, stimulated
**Snx2**	2.99E-06	-0.393481784	0.349	0.532	0.0422897[illegible]	T cells, CD4+, naive, stimulated
**Il21r**	3.05E-06	-0.592230062	0.118	0.257	0.0431373[illegible]	T cells, CD4+, naive, stimulated
**Cox8a**	3.11E-06	0.414109729	0.752	0.751	0.0440527[illegible]	T cells, CD4+, naive, stimulated
**Pgam1**	3.12E-06	-0.478434286	0.109	0.247	0.0441116[illegible]	T cells, CD4+, naive, stimulated
**Srebf2**	3.15E-06	-0.698759243	0.105	0.222	0.0445886[illegible]	T cells, CD4+, naive, stimulated
**Jak1**	3.19E-06	-0.465535969	0.345	0.511	0.0451589[illegible]	T cells, CD4+, naive, stimulated
**Blnk**	3.24E-06	-0.417249407	0.315	0.48	0.0458792[illegible]	T cells, CD4+, naive, stimulated
**Gm15987**	3.27E-06	0.623617184	0.282	0.163	0.0463355[illegible]	T cells, CD4+, naive, stimulated
**Csrnp1**	3.34E-06	-0.553526632	0.13	0.265	0.0472847[illegible]	T cells, CD4+, naive, stimulated
**Cnn2**	3.35E-06	-0.496402129	0.298	0.466	0.0473808[illegible]	T cells, CD4+, naive, stimulated
**Serinc1**	3.48E-06	-0.555559898	0.109	0.237	0.0492109[illegible]	T cells, CD4+, naive, stimulated
**Anp32a**	3.48E-06	0.684212189	0.508	0.39	0.0492886[illegible]	T cells, CD4+, naive, stimulated
**Rps24**	3.81E-38	-2.696910849	0.5	1	5.4E-34	T cells, CD4+, naive
**Rpl35a**	1.01E-37	-3.577355138	0.183	0.996	1.42E-33	T cells, CD4+, naive
**Rps20**	1.13E-37	-2.646777825	0.45	0.999	1.59E-33	T cells, CD4+, naive
**Rps27**	1.5E-37	-2.959578322	0.533	0.997	2.13E-33	T cells, CD4+, naive
**Rpl39**	2.01E-36	-2.929883993	0.3	0.994	2.85E-32	T cells, CD4+, naive
**Rps4x**	2.78E-36	-2.671061898	0.367	0.999	3.93E-32	T cells, CD4+, naive
**mt-Co1**	3.05E-36	1.997916372	0.983	0.999	4.31E-32	T cells, CD4+, naive
**Rpl35**	8.52E-36	-3.016926422	0.217	0.996	1.21E-31	T cells, CD4+, naive
**Uba52**	1.06E-35	-2.551752822	0.467	1	1.5E-31	T cells, CD4+, naive
**Rpl38**	1.35E-35	-2.606756715	0.4	0.99	1.91E-31	T cells, CD4+, naive
**Rps16**	1.37E-35	-2.387327132	0.483	0.999	1.94E-31	T cells, CD4+, naive
**Rpl37**	2.76E-35	-2.429441147	0.417	0.998	3.9E-31	T cells, CD4+, naive
**Rpl21**	3.83E-35	-2.634162269	0.283	0.997	5.43E-31	T cells, CD4+, naive
**Tpt1**	6.24E-35	-2.677099973	0.333	0.997	8.84E-31	T cells, CD4+, naive
**mt-Co3**	7.33E-35	2.13779805	0.983	0.999	1.04E-30	T cells, CD4+, naive
**Fau**	7.5E-35	-2.080418056	0.7	1	1.06E-30	T cells, CD4+, naive
**Rps29**	1.31E-34	-2.882513815	0.417	0.994	1.85E-30	T cells, CD4+, naive
**Rpl9-ps6**	1.41E-34	-2.549682112	0.333	0.999	1.99E-30	T cells, CD4+, naive

**Rps8**	1.62E-34	-2.15901935	0.583	0.999	2.3E-30	T cells, CD4+, naive
**Rps13**	1.81E-34	-2.549772324	0.317	0.998	2.57E-30	T cells, CD4+, naive
**mt-Co2**	1.9E-34	2.066322527	1	0.998	2.69E-30	T cells, CD4+, naive
**Rpl32**	2.57E-34	-2.633161574	0.3	0.996	3.64E-30	T cells, CD4+, naive
**Rps28**	2.71E-34	-2.745192816	0.317	0.992	3.83E-30	T cells, CD4+, naive
**Rps10**	5.64E-34	-2.529220269	0.333	0.996	7.99E-30	T cells, CD4+, naive
**Gm10076**	7.65E-34	-2.264151278	0.5	0.997	1.08E-29	T cells, CD4+, naive
**Rpl23**	9.78E-34	-2.364612867	0.4	0.998	1.38E-29	T cells, CD4+, naive
**Rpl6**	1.37E-33	-2.064090526	0.5	0.998	1.94E-29	T cells, CD4+, naive
**Rpl18a**	2.3E-33	-2.502407948	0.35	0.999	3.26E-29	T cells, CD4+, naive
**Rps15a**	2.9E-33	-2.210398065	0.483	0.996	4.1E-29	T cells, CD4+, naive
**Rps27a**	4.03E-33	-2.465098476	0.317	0.996	5.71E-29	T cells, CD4+, naive
**Rps19**	4.04E-33	-2.390446995	0.383	0.992	5.72E-29	T cells, CD4+, naive
**Rps7**	4.24E-33	-2.205006417	0.467	0.999	6E-29	T cells, CD4+, naive
**Rps26**	7.62E-33	-2.685770773	0.25	0.998	1.08E-28	T cells, CD4+, naive
**Rpl17**	1.15E-32	-2.460876433	0.317	0.984	1.63E-28	T cells, CD4+, naive
**Malat1**	1.43E-32	2.595699148	1	0.993	2.02E-28	T cells, CD4+, naive
**Rps3a1**	1.03E-31	-1.913470326	0.55	0.999	1.46E-27	T cells, CD4+, naive
**Eef1a1**	1.26E-31	-2.059307181	0.533	0.993	1.79E-27	T cells, CD4+, naive
**Rpl34**	1.41E-31	-2.316257838	0.35	0.998	1.99E-27	T cells, CD4+, naive
**Rpl37a**	2.48E-31	-2.030722418	0.533	0.995	3.51E-27	T cells, CD4+, naive
**Rps5**	3.15E-31	-2.109673612	0.433	0.998	4.46E-27	T cells, CD4+, naive
**Rplp0**	3.36E-31	-2.258004848	0.383	0.995	4.75E-27	T cells, CD4+, naive
**Rpl19**	4.42E-31	-1.960211454	0.5	0.999	6.26E-27	T cells, CD4+, naive
**Rps3**	5.67E-31	-2.085868052	0.417	0.996	8.02E-27	T cells, CD4+, naive
**Rpl27a**	8.04E-31	-2.173299037	0.367	0.994	1.14E-26	T cells, CD4+, naive
**Rpl13**	8.5E-31	-1.923598113	0.533	0.999	1.2E-26	T cells, CD4+, naive
**Rpl30**	9.54E-31	-2.034430263	0.467	0.999	1.35E-26	T cells, CD4+, naive
**Rpl28**	1.48E-30	-2.091405196	0.383	0.992	2.1E-26	T cells, CD4+, naive
**Rps21**	1.74E-30	-2.160158774	0.433	0.997	2.46E-26	T cells, CD4+, naive
**Rpl27**	1.87E-30	-2.550813954	0.183	0.975	2.65E-26	T cells, CD4+, naive
**Tmsb4x**	7.33E-30	-1.91973581	0.633	0.996	1.04E-25	T cells, CD4+, naive
**Fth1**	1.32E-29	-2.208877319	0.517	1	1.87E-25	T cells, CD4+, naive
**Rplp1**	1.47E-29	-2.138530947	0.383	0.992	2.08E-25	T cells, CD4+, naive
**Rpl36**	1.95E-29	-1.995731477	0.367	0.996	2.76E-25	T cells, CD4+, naive

**Rps12**	5.48E-29	-2.547294454	0.183	0.971	7.76E-25	T cells, CD4+, naive
**Rpl8**	6.55E-29	-1.840849972	0.467	0.996	9.27E-25	T cells, CD4+, naive
**H3f3a**	9.96E-29	-2.073494215	0.383	0.992	1.41E-24	T cells, CD4+, naive
**Rps9**	1.65E-28	-2.059891965	0.383	0.987	2.34E-24	T cells, CD4+, naive
**Rpl26**	2.89E-28	-2.268122615	0.233	0.984	4.09E-24	T cells, CD4+, naive
**Tmsb10**	1.16E-27	-1.813006234	0.6	0.998	1.64E-23	T cells, CD4+, naive
**Rpl22**	1.16E-27	-2.277139321	0.2	0.981	1.65E-23	T cells, CD4+, naive
**Rpl18**	2.27E-27	-1.885514554	0.4	0.996	3.21E-23	T cells, CD4+, naive
**mt-Nd1**	2.5E-27	2.025489167	0.983	0.963	3.55E-23	T cells, CD4+, naive
**mt-Atp8**	3.39E-27	1.932638597	1	0.993	4.81E-23	T cells, CD4+, naive
**mt-Cytb**	1.25E-26	1.876603827	0.967	0.991	1.77E-22	T cells, CD4+, naive
**Rpsa**	1.57E-26	-1.728328668	0.533	0.998	2.22E-22	T cells, CD4+, naive
**Rpl24**	7.56E-26	-2.237099604	0.183	0.966	1.07E-21	T cells, CD4+, naive
**Rps23**	8.55E-26	-2.185840558	0.233	0.961	1.21E-21	T cells, CD4+, naive
**Rpl10-ps3**	1.19E-25	-2.182419338	0.233	0.959	1.69E-21	T cells, CD4+, naive
**Rpl7**	9.12E-25	-2.040232761	0.267	0.961	1.29E-20	T cells, CD4+, naive
**Rps25**	1.86E-24	-2.577952822	0.05	0.829	2.63E-20	T cells, CD4+, naive
**Rplp2**	2.36E-24	-1.767311386	0.3	0.985	3.34E-20	T cells, CD4+, naive
**Rps11**	3.74E-24	-1.746011405	0.417	0.995	5.29E-20	T cells, CD4+, naive
**Rpl36a**	1.09E-23	-1.9466586	0.2	0.958	1.55E-19	T cells, CD4+, naive
**Rpl13a**	4.37E-22	-1.580884018	0.467	0.989	6.19E-18	T cells, CD4+, naive
**Atp5e**	1.04E-21	-2.203171315	0.067	0.811	1.47E-17	T cells, CD4+, naive
**Naca**	1.33E-21	-1.850428928	0.117	0.897	1.89E-17	T cells, CD4+, naive
**Rps2**	1.41E-21	-1.318272307	0.617	0.998	2E-17	T cells, CD4+, naive
**mt-Atp6**	1.55E-21	1.807606465	0.933	0.967	2.2E-17	T cells, CD4+, naive
**Rpl29**	2.87E-21	-1.711191574	0.333	0.978	4.07E-17	T cells, CD4+, naive
**Rpl11**	1.48E-20	-1.355651566	0.483	0.992	2.09E-16	T cells, CD4+, naive
**mt-Nd2**	1.72E-20	1.832922375	0.917	0.937	2.44E-16	T cells, CD4+, naive
**Rpl23a**	2.38E-20	-2.210230337	0.033	0.748	3.37E-16	T cells, CD4+, naive
**Rpl3**	3.57E-20	-1.595605437	0.35	0.969	5.06E-16	T cells, CD4+, naive
**Rpl14**	3.69E-20	-1.691126759	0.217	0.954	5.23E-16	T cells, CD4+, naive
**Ftl1**	4.4E-20	-1.614213678	0.4	0.984	6.22E-16	T cells, CD4+, naive
**Eif1**	9.71E-20	-1.272654508	0.567	0.993	1.38E-15	T cells, CD4+, naive
**Serp1**	1.21E-19	-1.729549136	0.117	0.839	1.71E-15	T cells, CD4+, naive
**Rpl15**	1.93E-19	-1.562506932	0.333	0.978	2.73E-15	T cells, CD4+, naive

**Btf3**	3.18E-19	-1.81803085	0.1	0.836	4.5E-15	T cells, CD4+, naive
**Eef1b2**	4.37E-19	-1.853240014	0.133	0.851	6.18E-15	T cells, CD4+, naive
**Chchd2**	1.15E-18	-1.868455126	0.167	0.871	1.63E-14	T cells, CD4+, naive
**Rpl31**	1.17E-18	-1.919488873	0.05	0.745	1.66E-14	T cells, CD4+, naive
**Ubb**	1.67E-18	-1.241484547	0.567	0.995	2.36E-14	T cells, CD4+, naive
**Rpl9**	2.07E-18	-1.645814677	0.167	0.892	2.94E-14	T cells, CD4+, naive
**Pfdn5**	2.08E-18	-1.760120358	0.067	0.77	2.95E-14	T cells, CD4+, naive
**Rps14**	2.64E-18	-1.49407673	0.333	0.981	3.74E-14	T cells, CD4+, naive
**Rps18**	2.74E-18	-1.572025112	0.35	0.951	3.89E-14	T cells, CD4+, naive
**Gabarap**	7.53E-18	-2.118497762	0.017	0.665	1.07E-13	T cells, CD4+, naive
**Sub1**	1.03E-17	-1.727291823	0.2	0.891	1.46E-13	T cells, CD4+, naive
**Arhgdib**	1.12E-17	-1.513021463	0.183	0.903	1.58E-13	T cells, CD4+, naive
**Gpx1**	1.19E-17	-2.142151558	0.05	0.714	1.68E-13	T cells, CD4+, naive
**Sh3bgrl3**	1.34E-17	-1.687968253	0.15	0.861	1.9E-13	T cells, CD4+, naive
**Cox8a**	1.44E-17	-1.624318545	0.083	0.775	2.04E-13	T cells, CD4+, naive
**Limd2**	4.06E-17	-1.964497519	0.067	0.724	5.75E-13	T cells, CD4+, naive
**Eif3f**	4.87E-17	-1.574525195	0.167	0.877	6.89E-13	T cells, CD4+, naive
**Rpl36al**	7.77E-17	-1.533912469	0.15	0.846	1.1E-12	T cells, CD4+, naive
**Rpl7a**	8.9E-17	-1.403657088	0.217	0.919	1.26E-12	T cells, CD4+, naive
**Rps17**	1.24E-16	-1.822072279	0.067	0.72	1.75E-12	T cells, CD4+, naive
**mt-Nd4l**	1.45E-16	1.765220475	0.933	0.969	2.06E-12	T cells, CD4+, naive
**Rpl10a**	1.59E-16	-1.338346621	0.4	0.984	2.24E-12	T cells, CD4+, naive
**Gng5**	1.73E-16	-2.10419182	0.033	0.654	2.45E-12	T cells, CD4+, naive
**Cfl1**	2.66E-16	-1.588968764	0.233	0.886	3.76E-12	T cells, CD4+, naive
**Ppia**	3.61E-16	-1.237137482	0.433	0.99	5.1E-12	T cells, CD4+, naive
**mt-Nd5**	5.57E-15	1.858224446	0.817	0.832	7.88E-11	T cells, CD4+, naive
**Rpl4**	1.12E-14	-1.272476619	0.183	0.851	1.58E-10	T cells, CD4+, naive
**Rpl12**	1.34E-14	-1.328887737	0.317	0.935	1.9E-10	T cells, CD4+, naive
**Dazap2**	1.35E-14	-1.638367665	0.05	0.648	1.92E-10	T cells, CD4+, naive
**Serf2**	1.36E-14	-1.357076485	0.183	0.865	1.93E-10	T cells, CD4+, naive
**H3f3b**	1.66E-14	-1.022539994	0.767	0.992	2.34E-10	T cells, CD4+, naive
**Rpl22l1**	2.2E-14	-1.349715032	0.133	0.778	3.11E-10	T cells, CD4+, naive
**Rps15**	2.41E-14	-1.307883801	0.183	0.871	3.41E-10	T cells, CD4+, naive
**Oaz1**	2.46E-14	-1.32406022	0.233	0.907	3.48E-10	T cells, CD4+, naive
**Cox4i1**	7.64E-14	-1.236822438	0.117	0.749	1.08E-09	T cells, CD4+, naive

**Lsp1**	1.33E-13	-1.376993585	0.1	0.698	1.89E-09	T cells, CD4+, naive
**Nsa2**	1.9E-13	-1.418384899	0.05	0.62	2.69E-09	T cells, CD4+, naive
**Ubl5**	1.91E-13	-1.63054205	0.033	0.585	2.7E-09	T cells, CD4+, naive
**Pcbp2**	2.02E-13	-1.208204608	0.133	0.75	2.86E-09	T cells, CD4+, naive
**Gnas**	2.6E-13	-1.256659502	0.117	0.722	3.68E-09	T cells, CD4+, naive
**Rps6**	3.95E-13	-1.221509496	0.25	0.88	5.59E-09	T cells, CD4+, naive
**Sec61g**	5.48E-13	-1.349605945	0.067	0.632	7.76E-09	T cells, CD4+, naive
**Zfp706**	5.84E-13	-1.752964427	0.033	0.567	8.27E-09	T cells, CD4+, naive
**Hmgb1**	6.84E-13	-1.232513719	0.1	0.692	9.69E-09	T cells, CD4+, naive
**Uqcrh**	8.61E-13	-1.153776019	0.117	0.716	1.22E-08	T cells, CD4+, naive
**Pfn1**	1.09E-12	-1.188642182	0.35	0.961	1.55E-08	T cells, CD4+, naive
**Npm1**	1.37E-12	-1.238404236	0.133	0.738	1.94E-08	T cells, CD4+, naive
**Myl12b**	2.1E-12	-1.186258472	0.1	0.671	2.98E-08	T cells, CD4+, naive
**Klf2**	2.45E-12	-1.412093888	0.483	0.934	3.47E-08	T cells, CD4+, naive
**Rpl10**	3.13E-12	-1.238808898	0.167	0.777	4.43E-08	T cells, CD4+, naive
**Atp5h**	5.51E-12	-1.732751316	0.017	0.513	7.8E-08	T cells, CD4+, naive
**Rpl5**	1.32E-11	-1.057759285	0.233	0.872	1.87E-07	T cells, CD4+, naive
**Hspe1**	1.62E-11	-1.490107464	0.25	0.804	2.29E-07	T cells, CD4+, naive
**Eif3h**	1.71E-11	-1.217814057	0.067	0.591	2.42E-07	T cells, CD4+, naive
**Ucp2**	1.79E-11	-1.2970289	0.133	0.69	2.54E-07	T cells, CD4+, naive
**Ybx1**	2.07E-11	-0.922530081	0.117	0.678	2.93E-07	T cells, CD4+, naive
**Eef2**	2.1E-11	-1.071152982	0.333	0.93	2.97E-07	T cells, CD4+, naive
**Cox6c**	2.74E-11	-1.311244038	0.05	0.554	3.88E-07	T cells, CD4+, naive
**Grcc10**	3.92E-11	-1.454934575	0.05	0.544	5.55E-07	T cells, CD4+, naive
**Bri3**	5.63E-11	-1.271256935	0.067	0.576	7.97E-07	T cells, CD4+, naive
**Actg1**	8.47E-11	-0.991479931	0.333	0.919	1.2E-06	T cells, CD4+, naive
**Hspa8**	9.97E-11	-1.366215183	0.55	0.929	1.41E-06	T cells, CD4+, naive
**Calm2**	1.02E-10	-1.021131412	0.15	0.71	1.44E-06	T cells, CD4+, naive
**Pcbp1**	1.09E-10	-1.188048689	0.1	0.624	1.55E-06	T cells, CD4+, naive
**Tsc22d3**	1.12E-10	-1.131450944	0.25	0.833	1.59E-06	T cells, CD4+, naive
**Ccl5**	1.25E-10	2.260052187	0.3	0.08	1.77E-06	T cells, CD4+, naive
**Rhoa**	1.34E-10	-0.904177053	0.2	0.774	1.89E-06	T cells, CD4+, naive
**Gapdh**	1.34E-10	-1.067443796	0.3	0.891	1.9E-06	T cells, CD4+, naive
**Sap18**	1.37E-10	-1.552643625	0.033	0.497	1.94E-06	T cells, CD4+, naive
**Paip2**	1.63E-10	-1.742263942	0	0.435	2.31E-06	T cells, CD4+, naive

**Ost4**	1.66E-10	-1.200101722	0.05	0.531	2.35E-06	T cells, CD4+, naive
**Cox7a2**	1.85E-10	-1.357188957	0.017	0.466	2.62E-06	T cells, CD4+, naive
**Atp5g3**	2.19E-10	-0.82811982	0.083	0.593	3.1E-06	T cells, CD4+, naive
**Slc25a5**	2.26E-10	-1.161831692	0.05	0.527	3.2E-06	T cells, CD4+, naive
**Tomm7**	2.39E-10	-1.524106109	0.017	0.462	3.38E-06	T cells, CD4+, naive
**Crip1**	2.76E-10	-1.504239701	0.133	0.642	3.91E-06	T cells, CD4+, naive
**Atp5d**	4.16E-10	-1.085426227	0.083	0.579	5.89E-06	T cells, CD4+, naive
**Rbm3**	4.17E-10	-1.055759852	0.25	0.813	5.9E-06	T cells, CD4+, naive
**Rac2**	4.34E-10	-0.940845296	0.183	0.746	6.14E-06	T cells, CD4+, naive
**Atp5l**	7.5E-10	-1.084366104	0.1	0.593	1.06E-05	T cells, CD4+, naive
**Atp5g2**	8.56E-10	-0.874475842	0.15	0.701	1.21E-05	T cells, CD4+, naive
**Tuba1a**	1.08E-09	-1.371647224	0.083	0.551	1.52E-05	T cells, CD4+, naive
**Coro1a**	1.12E-09	-0.776129614	0.333	0.919	1.59E-05	T cells, CD4+, naive
**Dnajb1**	1.18E-09	-1.548487711	0.2	0.677	1.67E-05	T cells, CD4+, naive
**Ptp4a3**	1.29E-09	-1.00650493	0.067	0.53	1.82E-05	T cells, CD4+, naive
**Cox7c**	1.76E-09	-0.683688183	0.167	0.71	2.5E-05	T cells, CD4+, naive
**Edf1**	1.96E-09	-1.357058557	0.033	0.463	2.78E-05	T cells, CD4+, naive
**Ptpn18**	2.05E-09	-1.09971834	0.083	0.551	2.9E-05	T cells, CD4+, naive
**Ndufa7**	2.25E-09	-1.378237746	0.033	0.458	3.19E-05	T cells, CD4+, naive
**Ptma**	2.43E-09	-0.817038294	0.6	0.991	3.44E-05	T cells, CD4+, naive
**mt-Nd4**	2.91E-09	1.477751512	0.75	0.807	4.12E-05	T cells, CD4+, naive
**Ndufa2**	2.92E-09	-1.592345868	0	0.393	4.13E-05	T cells, CD4+, naive
**Calm1**	3.29E-09	-0.623104747	0.267	0.861	4.66E-05	T cells, CD4+, naive
**Ebf1**	3.3E-09	1.327722614	0.783	0.906	4.67E-05	T cells, CD4+, naive
**Pkig**	3.41E-09	-1.149826185	0.033	0.456	4.82E-05	T cells, CD4+, naive
**Snrpf**	3.43E-09	-1.262666447	0.017	0.423	4.86E-05	T cells, CD4+, naive
**Rasgrp2**	3.61E-09	-1.285783061	0.05	0.482	5.12E-05	T cells, CD4+, naive
**Eif3k**	3.76E-09	-1.002109846	0.1	0.567	5.33E-05	T cells, CD4+, naive
**Ndufa3**	3.78E-09	-0.921785974	0.083	0.548	5.35E-05	T cells, CD4+, naive
**S100a10**	4E-09	-0.976184697	0.1	0.578	5.67E-05	T cells, CD4+, naive
**Ifi27l2a**	4.05E-09	-1.595222573	0.083	0.528	5.74E-05	T cells, CD4+, naive
**Cox5b**	4.3E-09	-0.693331686	0.1	0.577	6.09E-05	T cells, CD4+, naive
**Plac8**	4.39E-09	-1.846609321	0.033	0.441	6.22E-05	T cells, CD4+, naive
**Tuba1b**	4.4E-09	-1.002233607	0.033	0.451	6.22E-05	T cells, CD4+, naive
**Ppp1ca**	4.48E-09	-0.881166303	0.117	0.606	6.35E-05	T cells, CD4+, naive

**Cox6b1**	5.15E-09	-0.977990204	0.067	0.511	7.29E-05	T cells, CD4+, naive
**Actb**	6.04E-09	-0.623980639	0.8	0.998	8.55E-05	T cells, CD4+, naive
**Sec61b**	6.43E-09	-0.925102461	0.067	0.51	9.11E-05	T cells, CD4+, naive
**Eif5a**	6.55E-09	-0.799401007	0.133	0.629	9.28E-05	T cells, CD4+, naive
**Sumo2**	7.49E-09	-0.757748425	0.133	0.634	0.00010608	T cells, CD4+, naive
**Atp5j2**	7.68E-09	-1.053140073	0.067	0.505	0.00010874	T cells, CD4+, naive
**D8Ertd738**	8.93E-09	-0.881649646	0.133	0.625	0.00012634	T cells, CD4+, naive
**Snrpg**	1.09E-08	-0.649077269	0.133	0.629	0.00015443	T cells, CD4+, naive
**Rhob**	1.12E-08	-1.359371807	0.067	0.493	0.00015858	T cells, CD4+, naive
**Gnb2**	1.14E-08	-0.967063308	0.067	0.5	0.00016087	T cells, CD4+, naive
**Rbx1**	1.26E-08	-1.175344711	0.033	0.434	0.00017837	T cells, CD4+, naive
**Hmgn1**	1.37E-08	-0.8269314	0.1	0.562	0.00019404	T cells, CD4+, naive
**Eif3i**	1.5E-08	-1.212089224	0.017	0.4	0.00021175	T cells, CD4+, naive
**Zfp36**	1.61E-08	-0.753085506	0.233	0.759	0.00022744	T cells, CD4+, naive
**Nme2**	1.97E-08	-0.622454917	0.133	0.619	0.00027909	T cells, CD4+, naive
**Cd52**	1.97E-08	-0.654603539	0.367	0.924	0.00027912	T cells, CD4+, naive
**Jund**	2E-08	-0.797030227	0.5	0.948	0.00028257	T cells, CD4+, naive
**Cirbp**	2.23E-08	-0.954781159	0.067	0.485	0.00031636	T cells, CD4+, naive
**Rbm39**	2.26E-08	1.247277426	0.767	0.862	0.00032046	T cells, CD4+, naive
**Hnrnpf**	2.5E-08	-0.845871703	0.183	0.68	0.00035377	T cells, CD4+, naive
**Arpc1b**	2.66E-08	-0.78248671	0.167	0.663	0.00037630	T cells, CD4+, naive
**Hnrnpab**	3.22E-08	-0.792792197	0.083	0.518	0.00045589	T cells, CD4+, naive
**Tmem258**	3.49E-08	-1.058300304	0.033	0.417	0.00049350	T cells, CD4+, naive
**Cox7a2l**	3.88E-08	-1.009555274	0.067	0.476	0.00054948	T cells, CD4+, naive
**Eif3e**	3.98E-08	-1.041891065	0.05	0.449	0.00056343	T cells, CD4+, naive
**Gmfg**	4.01E-08	-0.83709458	0.1	0.537	0.00056831	T cells, CD4+, naive
**Rpl41**	4.55E-08	-0.810396402	0.333	0.891	0.00064441	T cells, CD4+, naive
**Hnrnpk**	4.87E-08	-0.543938039	0.15	0.632	0.00068969	T cells, CD4+, naive
**Psmb8**	6.53E-08	-0.554114492	0.133	0.599	0.00092448	T cells, CD4+, naive
**Banf1**	6.77E-08	-1.426936135	0	0.343	0.00095887	T cells, CD4+, naive
**Fis1**	7E-08	-0.78597335	0.067	0.472	0.00099045	T cells, CD4+, naive
**Trmt112**	7.27E-08	-1.045683512	0.05	0.434	0.00102952	T cells, CD4+, naive
**Myl12a**	7.39E-08	-0.697037061	0.067	0.47	0.00104595	T cells, CD4+, naive
**Pgls**	7.97E-08	-0.924594419	0.033	0.406	0.00112817	T cells, CD4+, naive
**Atp5j**	8.36E-08	-0.672509555	0.067	0.47	0.00118325	T cells, CD4+, naive

**H2afv**	1.05E-07	-1.136851447	0.017	0.368	0.0014909	T cells, CD4+, naive
**Polr1d**	1.06E-07	-0.941349178	0.083	0.492	0.0015020	T cells, CD4+, naive
**Atp5c1**	1.32E-07	-0.789121515	0.067	0.457	0.0018663	T cells, CD4+, naive
**Smim14**	1.33E-07	-0.858825758	0.117	0.543	0.0018790	T cells, CD4+, naive
**Ndufa4**	1.42E-07	-0.68406039	0.083	0.493	0.0020160	T cells, CD4+, naive
**Nol7**	1.43E-07	-1.036578797	0.05	0.426	0.0020285	T cells, CD4+, naive
**Arpc3**	1.58E-07	-0.589810259	0.217	0.729	0.0022334	T cells, CD4+, naive
**Cox17**	1.68E-07	-1.019887206	0.017	0.36	0.0023827	T cells, CD4+, naive
**Cnn2**	1.69E-07	-0.73001621	0.067	0.456	0.0023904	T cells, CD4+, naive
**Prelid1**	1.72E-07	-1.181066025	0.017	0.36	0.0024392	T cells, CD4+, naive
**Psme1**	1.87E-07	-0.611992249	0.117	0.545	0.0026487	T cells, CD4+, naive
**Tbca**	1.88E-07	-1.253828653	0.017	0.357	0.0026626	T cells, CD4+, naive
**Pabpc1**	2.11E-07	-0.570141809	0.317	0.831	0.0029937	T cells, CD4+, naive
**Prkar1a**	2.51E-07	-0.810260702	0.067	0.449	0.0035463	T cells, CD4+, naive
**Ran**	2.65E-07	-0.649951918	0.083	0.481	0.0037484	T cells, CD4+, naive
**Capg**	2.66E-07	-0.950642084	0.05	0.416	0.0037598	T cells, CD4+, naive
**Nop10**	2.81E-07	-0.762649261	0.1	0.509	0.0039815	T cells, CD4+, naive
**Hmgn2**	2.97E-07	-0.742456611	0.083	0.477	0.0042050	T cells, CD4+, naive
**Myl6**	3.03E-07	-0.557082629	0.183	0.667	0.0042850	T cells, CD4+, naive
**Ndufa6**	3.68E-07	-0.820845684	0.083	0.467	0.0052100	T cells, CD4+, naive
**Uqcrb**	3.7E-07	-0.733852977	0.05	0.412	0.0052328	T cells, CD4+, naive
**Cox6a1**	3.78E-07	-0.505469526	0.1	0.503	0.0053448	T cells, CD4+, naive
**Atp5f1**	3.89E-07	-0.772939438	0.067	0.443	0.0055041	T cells, CD4+, naive
**Fam107b**	3.93E-07	-0.598781519	0.217	0.675	0.0055589	T cells, CD4+, naive
**Ier5**	4.41E-07	-0.712199404	0.2	0.668	0.0062394	T cells, CD4+, naive
**Psmb1**	4.55E-07	-0.654843748	0.067	0.44	0.0064363	T cells, CD4+, naive
**Vim**	4.87E-07	-0.713396793	0.083	0.461	0.0068907	T cells, CD4+, naive
**Hint1**	5.15E-07	-0.729761975	0.1	0.501	0.0072844	T cells, CD4+, naive
**Ostf1**	5.84E-07	-1.020140139	0.033	0.369	0.0082665	T cells, CD4+, naive
**Cnp**	6.08E-07	-0.929725635	0.033	0.37	0.0086035	T cells, CD4+, naive
**Ndufa13**	6.62E-07	-0.880137033	0.067	0.428	0.0093681	T cells, CD4+, naive
**Btg1**	6.89E-07	-0.826062171	0.6	0.935	0.0097534	T cells, CD4+, naive
**Supt4a**	7.17E-07	-0.799168047	0.05	0.398	0.0101565	T cells, CD4+, naive
**Tgif1**	7.21E-07	-0.748194424	0.083	0.46	0.0102000	T cells, CD4+, naive
**Psma2**	7.3E-07	-0.979048519	0.033	0.366	0.0103326	T cells, CD4+, naive

**Tgfb1**	7.39E-07	-0.630980817	0.15	0.579	0.01045925	T cells, CD4+, naive
**Atp5g1**	7.86E-07	-1.082901077	0.017	0.333	0.01113190	T cells, CD4+, naive
**Atp5k**	9.05E-07	-0.782352407	0.033	0.363	0.01281325	T cells, CD4+, naive
**Tubb4b**	9.29E-07	-0.539925707	0.133	0.545	0.01315781	T cells, CD4+, naive
**Nedd8**	1.08E-06	-0.828398848	0.033	0.36	0.01535067	T cells, CD4+, naive
**Eef1g**	1.15E-06	-0.381002151	0.15	0.59	0.01632382	T cells, CD4+, naive
**Ubl3**	1.25E-06	-0.384253978	0.033	0.357	0.01769084	T cells, CD4+, naive
**Hmgb2**	1.27E-06	-0.925194947	0.05	0.388	0.01798122	T cells, CD4+, naive
**Gm8369**	1.41E-06	-0.848040315	0.117	0.493	0.01996353	T cells, CD4+, naive
**Anp32a**	1.54E-06	-0.37532023	0.067	0.418	0.02185347	T cells, CD4+, naive
**Ppdpf**	1.61E-06	-0.731067177	0.1	0.475	0.02282497	T cells, CD4+, naive
**Med28**	1.69E-06	-1.015747202	0.017	0.319	0.02388701	T cells, CD4+, naive
**Ptges3**	1.76E-06	-0.978549349	0.033	0.348	0.02492353	T cells, CD4+, naive
**Cdc42**	1.82E-06	-0.479207948	0.217	0.704	0.02570414	T cells, CD4+, naive
**Atp6v1g1**	1.86E-06	-0.97294693	0.017	0.317	0.02635178	T cells, CD4+, naive
**Snrpd2**	1.91E-06	-1.131887147	0.017	0.316	0.02710563	T cells, CD4+, naive
**Cox5a**	1.95E-06	-0.719441022	0.1	0.466	0.02756689	T cells, CD4+, naive
**Psenen**	2.07E-06	-0.674518072	0.033	0.348	0.02923284	T cells, CD4+, naive
**Tomm22**	2.1E-06	-0.79652909	0.05	0.379	0.02965801	T cells, CD4+, naive
**Eif3m**	2.14E-06	-0.606523693	0.017	0.315	0.03026595	T cells, CD4+, naive
**Marcksl1**	2.4E-06	-1.307061089	0.017	0.312	0.03397829	T cells, CD4+, naive
**Msmo1**	2.45E-06	1.51990511	0.15	0.035	0.03472852	T cells, CD4+, naive
**Atp5o**	2.55E-06	-1.030006544	0.033	0.342	0.03604062	T cells, CD4+, naive
**Tubb5**	2.58E-06	-0.615471196	0.067	0.408	0.03656894	T cells, CD4+, naive
**Tonsl**	2.67E-06	-0.977805194	0.017	0.311	0.03779258	T cells, CD4+, naive
**Scand1**	2.68E-06	-0.866820015	0.083	0.429	0.03794662	T cells, CD4+, naive
**Tma7**	2.8E-06	-0.406575378	0.117	0.503	0.03959379	T cells, CD4+, naive
**Rp9**	2.88E-06	-0.791728247	0.033	0.342	0.04080254	T cells, CD4+, naive
**Dynll1**	3.23E-06	-0.56163598	0.25	0.721	0.04571682	T cells, CD4+, naive
**Ube2s**	3.24E-06	-0.500886748	0.083	0.437	0.04586725	T cells, CD4+, naive
**Nfkbia**	3.25E-06	-0.396224486	0.183	0.612	0.04600556	T cells, CD4+, naive
**Tmem243**	3.35E-06	-1.00552805	0.017	0.307	0.04741444	T cells, CD4+, naive
**Snx3**	3.4E-06	-0.455066984	0.083	0.431	0.04809645	T cells, CD4+, naive
**Wbp2**	3.47E-06	-0.890093826	0.033	0.338	0.04913496	T cells, CD4+, naive
