# Supplementary material for: The Regulation Network of Glycerolipid Metabolism as Coregulators of Immunotherapy-Related Myocarditis
Source: Cardiovasc Ther. 2023 Jun 21;2023:8774971. doi: 10.1155/2023/8774971 (PMC10307211; doi:10.1155/2023/8774971)
Supplement: Supplementary 3 — Table S3: the Gene Ontology (GO) terms enrichment analysis for PTT-related DEGs of T cell subpopulation. [file 8774971.f3.pdf]

Table S3

	ONTOLOGY	ID	Description	GeneRatio	BgRatio	pvalue	p.adjust	qvalue
**GO:0022613**	BP	GO:0022613	ribonucleoprotein complex biogenesis	73/1133	412/29008	1.12E-27	5.96E-24	3.99E-24
**GO:0008380**	BP	GO:0008380	RNA splicing	68/1133	398/29008	6.62E-25	1.76E-21	1.18E-21
**GO:0043484**	BP	GO:0043484	regulation of RNA splicing	41/1133	157/29008	1.2E-22	2.12E-19	1.42E-19
**GO:1903311**	BP	GO:1903311	regulation of mRNA metabolic process	54/1133	293/29008	1.33E-21	1.76E-18	1.18E-18
**GO:0043161**	BP	GO:0043161	proteasome-mediated ubiquitin-dependent protein catabolic process	63/1133	423/29008	5.95E-20	6.33E-17	4.24E-17
**GO:0006397**	BP	GO:0006397	mRNA processing	66/1133	469/29008	1.64E-19	1.46E-16	9.76E-17
**GO:0042254**	BP	GO:0042254	ribosome biogenesis	50/1133	296/29008	2.01E-18	1.53E-15	1.02E-15
**GO:0016570**	BP	GO:0016570	histone modification	63/1133	461/29008	4.94E-18	3.29E-15	2.2E-15
**GO:0006325**	BP	GO:0006325	chromatin organization	64/1133	477/29008	6.83E-18	4.03E-15	2.7E-15
**GO:0042274**	BP	GO:0042274	ribosomal small subunit biogenesis	24/1133	72/29008	1.66E-16	8.82E-14	5.9E-14
**GO:1903320**	BP	GO:1903320	regulation of protein modification by small protein conjugation or removal	43/1133	248/29008	1.85E-16	8.93E-14	5.98E-14
**GO:0034976**	BP	GO:0034976	response to endoplasmic reticulum stress	43/1133	254/29008	4.54E-16	2.01E-13	1.35E-13
**GO:0022618**	BP	GO:0022618	ribonucleoprotein complex assembly	35/1133	171/29008	6.13E-16	2.05E-13	1.37E-13
**GO:0000375**	BP	GO:0000375	RNA splicing, via transesterification reactions	45/1133	279/29008	6.18E-16	2.05E-13	1.37E-13
**GO:0000377**	BP	GO:0000377	RNA splicing, via transesterification reactions with bulged adenosine as nucleophile	45/1133	279/29008	6.18E-16	2.05E-13	1.37E-13
**GO:0000398**	BP	GO:0000398	mRNA splicing, via spliceosome	45/1133	279/29008	6.18E-16	2.05E-13	1.37E-13
**GO:0006986**	BP	GO:0006986	response to unfolded protein	29/1133	116/29008	7.11E-16	2.23E-13	1.49E-13
**GO:0035966**	BP	GO:0035966	response to topologically incorrect protein	31/1133	138/29008	1.83E-15	5.41E-13	3.62E-13
**GO:0071826**	BP	GO:0071826	ribonucleoprotein complex subunit organization	35/1133	178/29008	2.25E-15	6.29E-13	4.21E-13
**GO:0031331**	BP	GO:0031331	positive regulation of cellular catabolic process	55/1133	425/29008	7.09E-15	1.89E-12	1.26E-12
**GO:0009896**	BP	GO:0009896	positive regulation of catabolic process	60/1133	494/29008	7.69E-15	1.95E-12	1.3E-12
**GO:0006913**	BP	GO:0006913	nucleocytoplasmic transport	47/1133	327/29008	1.29E-14	2.98E-12	2E-12
**GO:0051169**	BP	GO:0051169	nuclear transport	47/1133	327/29008	1.29E-14	2.98E-12	2E-12
**GO:0034248**	BP	GO:0034248	regulation of cellular amide metabolic process	56/1133	448/29008	1.8E-14	4E-12	2.68E-12
**GO:0002181**	BP	GO:0002181	cytoplasmic translation	25/1133	96/29008	2.53E-14	5.26E-12	3.52E-12
**GO:0006364**	BP	GO:0006364	rRNA processing	37/1133	214/29008	2.57E-14	5.26E-12	3.52E-12
**GO:0030099**	BP	GO:0030099	myeloid cell differentiation	54/1133	426/29008	2.98E-14	5.87E-12	3.93E-12
**GO:0006457**	BP	GO:0006457	protein folding	32/1133	163/29008	3.71E-14	7.04E-12	4.72E-12
**GO:2001233**	BP	GO:2001233	regulation of apoptotic signaling pathway	52/1133	402/29008	3.96E-14	7.27E-12	4.87E-12
**GO:0050684**	BP	GO:0050684	regulation of mRNA processing	30/1133	148/29008	9.57E-14	1.7E-11	1.14E-11
**GO:0016072**	BP	GO:0016072	rRNA metabolic process	37/1133	224/29008	1.12E-13	1.92E-11	1.29E-11
**GO:0006417**	BP	GO:0006417	regulation of translation	50/1133	390/29008	1.74E-13	2.89E-11	1.94E-11
**GO:0016032**	BP	GO:0016032	viral process	46/1133	346/29008	4.4E-13	7.08E-11	4.75E-11
**GO:0140694**	BP	GO:0140694	non-membrane-bounded organelle assembly	48/1133	373/29008	4.63E-13	7.24E-11	4.85E-11
**GO:0042176**	BP	GO:0042176	regulation of protein catabolic process	49/1133	389/29008	5.93E-13	9.01E-11	6.04E-11
**GO:0030098**	BP	GO:0030098	lymphocyte differentiation	52/1133	441/29008	1.55E-12	2.3E-10	1.54E-10
**GO:0043281**	BP	GO:0043281	regulation of cysteine-type endopeptidase activity involved in apoptotic process	34/1133	210/29008	1.99E-12	2.86E-10	1.91E-10
**GO:0034620**	BP	GO:0034620	cellular response to unfolded protein	22/1133	88/29008	2.21E-12	3.09E-10	2.07E-10
**GO:1903131**	BP	GO:1903131	mononuclear cell differentiation	55/1133	489/29008	2.37E-12	3.23E-10	2.16E-10
**GO:0035967**	BP	GO:0035967	cellular response to topologically incorrect protein	24/1133	108/29008	3.5E-12	4.6E-10	3.08E-10
**GO:0046631**	BP	GO:0046631	alpha-beta T cell activation	31/1133	180/29008	3.55E-12	4.6E-10	3.08E-10
**GO:2000116**	BP	GO:2000116	regulation of cysteine-type endopeptidase activity	36/1133	239/29008	4.01E-12	5.08E-10	3.4E-10
**GO:0007264**	BP	GO:0007264	small GTPase mediated signal transduction	50/1133	425/29008	4.55E-12	5.63E-10	3.77E-10
**GO:0006338**	BP	GO:0006338	chromatin remodeling	33/1133	208/29008	7.42E-12	8.97E-10	6.01E-10
**GO:0018205**	BP	GO:0018205	peptidyl-lysine modification	46/1133	375/29008	7.72E-12	9.03E-10	6.05E-10
**GO:0002683**	BP	GO:0002683	negative regulation of immune system process	52/1133	460/29008	7.81E-12	9.03E-10	6.05E-10
**GO:0031396**	BP	GO:0031396	regulation of protein ubiquitination	33/1133	214/29008	1.64E-11	1.86E-09	1.24E-09
**GO:0030522**	BP	GO:0030522	intracellular receptor signaling pathway	33/1133	217/29008	2.41E-11	2.67E-09	1.79E-09
**GO:0006402**	BP	GO:0006402	mRNA catabolic process	34/1133	231/29008	3.03E-11	3.29E-09	2.2E-09
**GO:0048024**	BP	GO:0048024	regulation of mRNA splicing, via spliceosome	23/1133	110/29008	3.71E-11	3.95E-09	2.65E-09
**GO:0043434**	BP	GO:0043434	response to peptide hormone	43/1133	351/29008	3.88E-11	4.04E-09	2.71E-09
**GO:0006401**	BP	GO:0006401	RNA catabolic process	37/1133	272/29008	4.36E-11	4.46E-09	2.99E-09
**GO:0009615**	BP	GO:0009615	response to virus	40/1133	312/29008	4.6E-11	4.53E-09	3.04E-09
**GO:0010563**	BP	GO:0010563	negative regulation of phosphorus metabolic process	50/1133	453/29008	4.69E-11	4.53E-09	3.04E-09
**GO:0045936**	BP	GO:0045936	negative regulation of phosphate metabolic process	50/1133	453/29008	4.69E-11	4.53E-09	3.04E-09
**GO:1901652**	BP	GO:1901652	response to peptide	47/1133	412/29008	5.82E-11	5.52E-09	3.7E-09
**GO:1903322**	BP	GO:1903322	positive regulation of protein modification by small protein conjugation or removal	26/1133	145/29008	7.65E-11	7.14E-09	4.78E-09
**GO:0007249**	BP	GO:0007249	I-kappaB kinase/NF-kappaB signaling	33/1133	228/29008	9.29E-11	8.45E-09	5.66E-09
**GO:0036503**	BP	GO:0036503	ERAD pathway	22/1133	105/29008	9.45E-11	8.45E-09	5.66E-09
**GO:0072594**	BP	GO:0072594	establishment of protein localization to organelle	47/1133	418/29008	9.54E-11	8.45E-09	5.66E-09
**GO:0071824**	BP	GO:0071824	protein-DNA complex subunit organization	30/1133	192/29008	9.74E-11	8.49E-09	5.69E-09
**GO:0043543**	BP	GO:0043543	protein acylation	34/1133	243/29008	1.23E-10	1.06E-08	7.09E-09
**GO:0006333**	BP	GO:0006333	chromatin assembly or disassembly	24/1133	127/29008	1.31E-10	1.11E-08	7.4E-09
**GO:0006914**	BP	GO:0006914	autophagy	48/1133	437/29008	1.35E-10	1.11E-08	7.4E-09
**GO:0061919**	BP	GO:0061919	process utilizing autophagic mechanism	48/1133	437/29008	1.35E-10	1.11E-08	7.4E-09
**GO:0018209**	BP	GO:0018209	peptidyl-serine modification	43/1133	365/29008	1.37E-10	1.11E-08	7.4E-09
**GO:0061013**	BP	GO:0061013	regulation of mRNA catabolic process	27/1133	161/29008	1.63E-10	1.29E-08	8.64E-09
**GO:0001819**	BP	GO:0001819	positive regulation of cytokine production	52/1133	500/29008	1.7E-10	1.33E-08	8.92E-09
**GO:0043087**	BP	GO:0043087	regulation of GTPase activity	40/1133	326/29008	1.77E-10	1.37E-08	9.14E-09
**GO:1903037**	BP	GO:1903037	regulation of leukocyte cell-cell adhesion	40/1133	333/29008	3.37E-10	2.56E-08	1.71E-08
**GO:0097193**	BP	GO:0097193	intrinsic apoptotic signaling pathway	38/1133	307/29008	3.91E-10	2.93E-08	1.96E-08
**GO:0030433**	BP	GO:0030433	ubiquitin-dependent ERAD pathway	19/1133	84/29008	4.47E-10	3.3E-08	2.21E-08
**GO:0045862**	BP	GO:0045862	positive regulation of proteolysis	41/1133	351/29008	4.76E-10	3.47E-08	2.32E-08
**GO:0051090**	BP	GO:0051090	regulation of DNA-binding transcription factor activity	46/1133	425/29008	5.32E-10	3.82E-08	2.56E-08
**GO:0042326**	BP	GO:0042326	negative regulation of phosphorylation	44/1133	396/29008	5.52E-10	3.92E-08	2.62E-08
**GO:0018105**	BP	GO:0018105	peptidyl-serine phosphorylation	40/1133	340/29008	6.27E-10	4.38E-08	2.94E-08
**GO:0006354**	BP	GO:0006354	DNA-templated transcription, elongation	19/1133	86/29008	6.87E-10	4.74E-08	3.18E-08
**GO:1903362**	BP	GO:1903362	regulation of cellular protein catabolic process	33/1133	246/29008	7E-10	4.77E-08	3.2E-08
**GO:0006413**	BP	GO:0006413	translational initiation	22/1133	117/29008	8.48E-10	5.71E-08	3.82E-08
**GO:0007159**	BP	GO:0007159	leukocyte cell-cell adhesion	42/1133	373/29008	9.07E-10	6.03E-08	4.04E-08
**GO:0031056**	BP	GO:0031056	regulation of histone modification	27/1133	174/29008	9.77E-10	6.38E-08	4.27E-08
**GO:0034504**	BP	GO:0034504	protein localization to nucleus	38/1133	317/29008	9.83E-10	6.38E-08	4.27E-08
**GO:0071375**	BP	GO:0071375	cellular response to peptide hormone stimulus	34/1133	264/29008	1.15E-09	7.37E-08	4.94E-08
**GO:0043488**	BP	GO:0043488	regulation of mRNA stability	25/1133	152/29008	1.17E-09	7.4E-08	4.96E-08
**GO:0048872**	BP	GO:0048872	homeostasis of number of cells	41/1133	363/29008	1.31E-09	8.19E-08	5.49E-08
**GO:0006473**	BP	GO:0006473	protein acetylation	29/1133	201/29008	1.36E-09	8.42E-08	5.64E-08
**GO:0031647**	BP	GO:0031647	regulation of protein stability	37/1133	307/29008	1.4E-09	8.58E-08	5.74E-08
**GO:1901653**	BP	GO:1901653	cellular response to peptide	37/1133	308/29008	1.54E-09	9.28E-08	6.22E-08
**GO:0043487**	BP	GO:0043487	regulation of RNA stability	26/1133	166/29008	1.62E-09	9.68E-08	6.48E-08
**GO:0051168**	BP	GO:0051168	nuclear export	25/1133	155/29008	1.78E-09	1.05E-07	7.04E-08
**GO:0022407**	BP	GO:0022407	regulation of cell-cell adhesion	47/1133	457/29008	1.85E-09	1.08E-07	7.25E-08
**GO:0032868**	BP	GO:0032868	response to insulin	32/1133	244/29008	2.24E-09	1.29E-07	8.66E-08
**GO:0030183**	BP	GO:0030183	B cell differentiation	26/1133	170/29008	2.73E-09	1.56E-07	1.04E-07
**GO:0032869**	BP	GO:0032869	cellular response to insulin stimulus	29/1133	208/29008	3.05E-09	1.73E-07	1.16E-07
**GO:0034728**	BP	GO:0034728	nucleosome organization	22/1133	125/29008	3.12E-09	1.74E-07	1.17E-07
**GO:0030490**	BP	GO:0030490	maturation of SSU-rRNA	14/1133	49/29008	3.34E-09	1.82E-07	1.22E-07
**GO:0006605**	BP	GO:0006605	protein targeting	34/1133	275/29008	3.35E-09	1.82E-07	1.22E-07
**GO:0000209**	BP	GO:0000209	protein polyubiquitination	32/1133	248/29008	3.36E-09	1.82E-07	1.22E-07
**GO:0034470**	BP	GO:0034470	ncRNA processing	40/1133	362/29008	3.92E-09	2.11E-07	1.41E-07
**GO:0008286**	BP	GO:0008286	insulin receptor signaling pathway	22/1133	127/29008	4.24E-09	2.25E-07	1.51E-07
**GO:0043401**	BP	GO:0043401	steroid hormone mediated signaling pathway	21/1133	117/29008	4.91E-09	2.58E-07	1.73E-07
**GO:0033119**	BP	GO:0033119	negative regulation of RNA splicing	11/1133	29/29008	5.57E-09	2.9E-07	1.94E-07
**GO:2001242**	BP	GO:2001242	regulation of intrinsic apoptotic signaling pathway	26/1133	176/29008	5.78E-09	2.98E-07	2E-07
**GO:0050863**	BP	GO:0050863	regulation of T cell activation	38/1133	339/29008	6.45E-09	3.3E-07	2.21E-07
**GO:0043122**	BP	GO:0043122	regulation of I-kappaB kinase/NF-kappaB signaling	27/1133	190/29008	6.98E-09	3.53E-07	2.37E-07
**GO:0042026**	BP	GO:0042026	protein refolding	9/1133	18/29008	7.25E-09	3.64E-07	2.44E-07
**GO:0030217**	BP	GO:0030217	T cell differentiation	35/1133	299/29008	8.33E-09	4.14E-07	2.77E-07
**GO:0052548**	BP	GO:0052548	regulation of endopeptidase activity	39/1133	357/29008	8.42E-09	4.14E-07	2.78E-07
**GO:0070936**	BP	GO:0070936	protein K48-linked ubiquitination	16/1133	70/29008	8.99E-09	4.39E-07	2.94E-07
**GO:0007265**	BP	GO:0007265	Ras protein signal transduction	37/1133	330/29008	1.01E-08	4.89E-07	3.27E-07
**GO:0032479**	BP	GO:0032479	regulation of type I interferon production	19/1133	101/29008	1.18E-08	5.62E-07	3.77E-07
**GO:0034660**	BP	GO:0034660	ncRNA metabolic process	45/1133	453/29008	1.18E-08	5.62E-07	3.77E-07
**GO:0045732**	BP	GO:0045732	positive regulation of protein catabolic process	29/1133	221/29008	1.23E-08	5.78E-07	3.87E-07
**GO:0018394**	BP	GO:0018394	peptidyl-lysine acetylation	25/1133	170/29008	1.24E-08	5.78E-07	3.87E-07
**GO:0002697**	BP	GO:0002697	regulation of immune effector process	43/1133	424/29008	1.36E-08	6.31E-07	4.22E-07
**GO:0032386**	BP	GO:0032386	regulation of intracellular transport	37/1133	335/29008	1.51E-08	6.92E-07	4.64E-07
**GO:0019827**	BP	GO:0019827	stem cell population maintenance	25/1133	172/29008	1.58E-08	7.16E-07	4.8E-07
**GO:0016573**	BP	GO:0016573	histone acetylation	23/1133	148/29008	1.63E-08	7.33E-07	4.91E-07
**GO:0032606**	BP	GO:0032606	type I interferon production	19/1133	103/29008	1.65E-08	7.36E-07	4.93E-07
**GO:1903312**	BP	GO:1903312	negative regulation of mRNA metabolic process	18/1133	93/29008	1.78E-08	7.9E-07	5.29E-07
**GO:0046700**	BP	GO:0046700	heterocycle catabolic process	42/1133	415/29008	2.13E-08	9.36E-07	6.27E-07
**GO:0050854**	BP	GO:0050854	regulation of antigen receptor-mediated signaling pathway	15/1133	65/29008	2.27E-08	9.91E-07	6.64E-07
**GO:0038061**	BP	GO:0038061	NIK/NF-kappaB signaling	20/1133	116/29008	2.31E-08	9.99E-07	6.69E-07
**GO:0000028**	BP	GO:0000028	ribosomal small subunit assembly	9/1133	20/29008	2.33E-08	1E-06	6.7E-07
**GO:0034655**	BP	GO:0034655	nucleobase-containing compound catabolic process	40/1133	386/29008	2.4E-08	1.02E-06	6.81E-07
**GO:0051607**	BP	GO:0051607	defense response to virus	31/1133	255/29008	2.43E-08	1.02E-06	6.81E-07
**GO:0140546**	BP	GO:0140546	defense response to symbiont	31/1133	255/29008	2.43E-08	1.02E-06	6.81E-07
**GO:0098727**	BP	GO:0098727	maintenance of cell number	25/1133	176/29008	2.52E-08	1.05E-06	7.01E-07
**GO:0090150**	BP	GO:0090150	establishment of protein localization to membrane	31/1133	256/29008	2.66E-08	1.1E-06	7.35E-07
**GO:0071383**	BP	GO:0071383	cellular response to steroid hormone stimulus	24/1133	165/29008	3E-08	1.23E-06	8.21E-07
**GO:0030968**	BP	GO:0030968	endoplasmic reticulum unfolded protein response	15/1133	67/29008	3.52E-08	1.43E-06	9.58E-07
**GO:0061077**	BP	GO:0061077	chaperone-mediated protein folding	14/1133	58/29008	3.61E-08	1.45E-06	9.75E-07
**GO:0031058**	BP	GO:0031058	positive regulation of histone modification	19/1133	108/29008	3.68E-08	1.47E-06	9.85E-07
**GO:0048193**	BP	GO:0048193	Golgi vesicle transport	32/1133	274/29008	3.79E-08	1.51E-06	1.01E-06
**GO:0048588**	BP	GO:0048588	developmental cell growth	32/1133	275/29008	4.14E-08	1.63E-06	1.09E-06
**GO:0032943**	BP	GO:0032943	mononuclear cell proliferation	36/1133	334/29008	4.41E-08	1.73E-06	1.16E-06

**GO:0030518**	BP	GO:0030518	intracellular steroid hormone receptor signaling pathway	19/1133	110/29008	5E-08	1.94E-06	1.3E-06
**GO:0001818**	BP	GO:0001818	negative regulation of cytokine production	33/1133	292/29008	5.18E-08	1.99E-06	1.34E-06
**GO:0019080**	BP	GO:0019080	viral gene expression	15/1133	69/29008	5.37E-08	2.05E-06	1.38E-06
**GO:0034249**	BP	GO:0034249	negative regulation of cellular amide metabolic process	27/1133	209/29008	5.42E-08	2.06E-06	1.38E-06
**GO:1903706**	BP	GO:1903706	regulation of hemopoiesis	40/1133	398/29008	5.55E-08	2.09E-06	1.4E-06
**GO:0006475**	BP	GO:0006475	internal protein amino acid acetylation	23/1133	158/29008	5.69E-08	2.11E-06	1.41E-06
**GO:0018393**	BP	GO:0018393	internal peptidyl-lysine acetylation	23/1133	158/29008	5.69E-08	2.11E-06	1.41E-06
**GO:0044270**	BP	GO:0044270	cellular nitrogen compound catabolic process	41/1133	414/29008	5.71E-08	2.11E-06	1.41E-06
**GO:0006446**	BP	GO:0006446	regulation of translational initiation	14/1133	60/29008	5.74E-08	2.11E-06	1.41E-06
**GO:0000956**	BP	GO:0000956	nuclear-transcribed mRNA catabolic process	19/1133	111/29008	5.82E-08	2.12E-06	1.42E-06
**GO:0042594**	BP	GO:0042594	response to starvation	25/1133	184/29008	6.17E-08	2.23E-06	1.49E-06
**GO:0048545**	BP	GO:0048545	response to steroid hormone	28/1133	226/29008	7.62E-08	2.74E-06	1.83E-06
**GO:0070661**	BP	GO:0070661	leukocyte proliferation	37/1133	358/29008	8.54E-08	3.05E-06	2.04E-06
**GO:1903050**	BP	GO:1903050	regulation of proteolysis involved in cellular protein catabolic process	27/1133	214/29008	8.88E-08	3.15E-06	2.11E-06
**GO:0019439**	BP	GO:0019439	aromatic compound catabolic process	42/1133	437/29008	9.13E-08	3.22E-06	2.15E-06
**GO:2001234**	BP	GO:2001234	negative regulation of apoptotic signaling pathway	29/1133	242/29008	9.33E-08	3.26E-06	2.19E-06
**GO:0042273**	BP	GO:0042273	ribosomal large subunit biogenesis	15/1133	72/29008	9.8E-08	3.41E-06	2.28E-06
**GO:0046651**	BP	GO:0046651	lymphocyte proliferation	35/1133	330/29008	1.01E-07	3.48E-06	2.33E-06
**GO:0009267**	BP	GO:0009267	cellular response to starvation	23/1133	164/29008	1.15E-07	3.93E-06	2.63E-06
**GO:2000117**	BP	GO:2000117	negative regulation of cysteine-type endopeptidase activity	17/1133	94/29008	1.25E-07	4.28E-06	2.86E-06
**GO:0051028**	BP	GO:0051028	mRNA transport	19/1133	117/29008	1.38E-07	4.66E-06	3.12E-06
**GO:0043154**	BP	GO:0043154	negative regulation of cysteine-type endopeptidase activity involved in apoptotic pro	16/1133	84/29008	1.39E-07	4.66E-06	3.12E-06
**GO:1903313**	BP	GO:1903313	positive regulation of mRNA metabolic process	20/1133	129/29008	1.44E-07	4.78E-06	3.2E-06
**GO:0043620**	BP	GO:0043620	regulation of DNA-templated transcription in response to stress	13/1133	55/29008	1.45E-07	4.78E-06	3.2E-06
**GO:0051170**	BP	GO:0051170	import into nucleus	24/1133	179/29008	1.45E-07	4.78E-06	3.2E-06
**GO:0051056**	BP	GO:0051056	regulation of small GTPase mediated signal transduction	28/1133	233/29008	1.46E-07	4.78E-06	3.2E-06
**GO:0016571**	BP	GO:0016571	histone methylation	22/1133	154/29008	1.54E-07	5.04E-06	3.37E-06
**GO:0043433**	BP	GO:0043433	negative regulation of DNA-binding transcription factor activity	23/1133	167/29008	1.6E-07	5.2E-06	3.48E-06
**GO:1901361**	BP	GO:1901361	organic cyclic compound catabolic process	43/1133	463/29008	1.68E-07	5.42E-06	3.63E-06
**GO:0034101**	BP	GO:0034101	erythrocyte homeostasis	22/1133	155/29008	1.73E-07	5.55E-06	3.72E-06
**GO:1903364**	BP	GO:1903364	positive regulation of cellular protein catabolic process	21/1133	143/29008	1.83E-07	5.82E-06	3.9E-06
**GO:0001558**	BP	GO:0001558	regulation of cell growth	42/1133	450/29008	2.04E-07	6.46E-06	4.33E-06
**GO:0042113**	BP	GO:0042113	B cell activation	41/1133	434/29008	2.06E-07	6.48E-06	4.34E-06
**GO:0002699**	BP	GO:0002699	positive regulation of immune effector process	32/1133	296/29008	2.28E-07	7.14E-06	4.78E-06
**GO:0065004**	BP	GO:0065004	protein-DNA complex assembly	21/1133	145/29008	2.32E-07	7.19E-06	4.81E-06
**GO:0050686**	BP	GO:0050686	negative regulation of mRNA processing	10/1133	32/29008	2.34E-07	7.19E-06	4.81E-06
**GO:0051085**	BP	GO:0051085	chaperone cofactor-dependent protein refolding	10/1133	32/29008	2.34E-07	7.19E-06	4.81E-06
**GO:0045785**	BP	GO:0045785	positive regulation of cell adhesion	43/1133	471/29008	2.69E-07	8.23E-06	5.52E-06
**GO:0016601**	BP	GO:0016601	Rac protein signal transduction	12/1133	49/29008	2.88E-07	8.75E-06	5.86E-06
**GO:0050657**	BP	GO:0050657	nucleic acid transport	21/1133	147/29008	2.94E-07	8.83E-06	5.91E-06
**GO:0050658**	BP	GO:0050658	RNA transport	21/1133	147/29008	2.94E-07	8.83E-06	5.91E-06
**GO:0017148**	BP	GO:0017148	negative regulation of translation	24/1133	186/29008	2.99E-07	8.92E-06	5.98E-06
**GO:0046634**	BP	GO:0046634	regulation of alpha-beta T cell activation	18/1133	111/29008	3E-07	8.92E-06	5.98E-06
**GO:0031398**	BP	GO:0031398	positive regulation of protein ubiquitination	19/1133	123/29008	3.11E-07	9.18E-06	6.15E-06
**GO:0052547**	BP	GO:0052547	regulation of peptidase activity	41/1133	441/29008	3.15E-07	9.25E-06	6.2E-06
**GO:0001666**	BP	GO:0001666	response to hypoxia	26/1133	214/29008	3.24E-07	9.47E-06	6.34E-06
**GO:0009755**	BP	GO:0009755	hormone-mediated signaling pathway	22/1133	161/29008	3.4E-07	9.89E-06	6.62E-06
**GO:0007519**	BP	GO:0007519	skeletal muscle tissue development	25/1133	201/29008	3.47E-07	1E-05	6.72E-06
**GO:0046626**	BP	GO:0046626	regulation of insulin receptor signaling pathway	15/1133	79/29008	3.54E-07	1.02E-05	6.81E-06
**GO:0017038**	BP	GO:0017038	protein import	27/1133	229/29008	3.56E-07	1.02E-05	6.81E-06
**GO:0019083**	BP	GO:0019083	viral transcription	12/1133	50/29008	3.66E-07	1.03E-05	6.89E-06
**GO:0043618**	BP	GO:0043618	regulation of transcription from RNA polymerase II promoter in response to stress	12/1133	50/29008	3.66E-07	1.03E-05	6.89E-06
**GO:0060964**	BP	GO:0060964	regulation of gene silencing by miRNA	12/1133	50/29008	3.66E-07	1.03E-05	6.89E-06
**GO:0032481**	BP	GO:0032481	positive regulation of type I interferon production	14/1133	69/29008	3.69E-07	1.03E-05	6.91E-06
**GO:2000058**	BP	GO:2000058	regulation of ubiquitin-dependent protein catabolic process	22/1133	162/29008	3.79E-07	1.06E-05	7.08E-06
**GO:0001933**	BP	GO:0001933	negative regulation of protein phosphorylation	35/1133	349/29008	3.9E-07	1.08E-05	7.24E-06
**GO:0051348**	BP	GO:0051348	negative regulation of transferase activity	29/1133	259/29008	3.99E-07	1.1E-05	7.37E-06
**GO:0043409**	BP	GO:0043409	negative regulation of MAPK cascade	24/1133	189/29008	4.04E-07	1.11E-05	7.41E-06
**GO:0051236**	BP	GO:0051236	establishment of RNA localization	21/1133	150/29008	4.15E-07	1.13E-05	7.58E-06
**GO:0031668**	BP	GO:0031668	cellular response to extracellular stimulus	27/1133	231/29008	4.23E-07	1.15E-05	7.7E-06
**GO:0050821**	BP	GO:0050821	protein stabilization	24/1133	190/29008	4.45E-07	1.2E-05	8.05E-06
**GO:0002831**	BP	GO:0002831	regulation of response to biotic stimulus	34/1133	337/29008	4.95E-07	1.33E-05	8.91E-06
**GO:0032922**	BP	GO:0032922	circadian regulation of gene expression	14/1133	71/29008	5.34E-07	1.43E-05	9.56E-06
**GO:0050777**	BP	GO:0050777	negative regulation of immune response	25/1133	206/29008	5.54E-07	1.47E-05	9.87E-06
**GO:0022411**	BP	GO:0022411	cellular component disassembly	38/1133	402/29008	5.6E-07	1.47E-05	9.88E-06
**GO:0006984**	BP	GO:0006984	ER-nucleus signaling pathway	11/1133	43/29008	5.6E-07	1.47E-05	9.88E-06
**GO:0031497**	BP	GO:0031497	chromatin assembly	17/1133	104/29008	5.67E-07	1.48E-05	9.95E-06
**GO:0033674**	BP	GO:0033674	positive regulation of kinase activity	40/1133	435/29008	5.8E-07	1.51E-05	1.01E-05
**GO:0042098**	BP	GO:0042098	T cell proliferation	26/1133	221/29008	6.07E-07	1.58E-05	1.06E-05
**GO:0060537**	BP	GO:0060537	muscle tissue development	43/1133	486/29008	6.29E-07	1.62E-05	1.09E-05
**GO:0002287**	BP	GO:0002287	alpha-beta T cell activation involved in immune response	14/1133	72/29008	6.39E-07	1.63E-05	1.09E-05
**GO:0098586**	BP	GO:0098586	cellular response to virus	14/1133	72/29008	6.39E-07	1.63E-05	1.09E-05
**GO:0071456**	BP	GO:0071456	cellular response to hypoxia	17/1133	105/29008	6.52E-07	1.66E-05	1.11E-05
**GO:0043558**	BP	GO:0043558	regulation of translational initiation in response to stress	7/1133	15/29008	6.65E-07	1.68E-05	1.13E-05
**GO:0009895**	BP	GO:0009895	negative regulation of catabolic process	32/1133	311/29008	6.91E-07	1.74E-05	1.17E-05
**GO:0060147**	BP	GO:0060147	regulation of posttranscriptional gene silencing	12/1133	53/29008	7.21E-07	1.81E-05	1.21E-05
**GO:0050866**	BP	GO:0050866	negative regulation of cell activation	26/1133	223/29008	7.23E-07	1.81E-05	1.21E-05
**GO:0042255**	BP	GO:0042255	ribosome assembly	13/1133	63/29008	7.83E-07	1.94E-05	1.3E-05
**GO:0002262**	BP	GO:0002262	myeloid cell homeostasis	24/1133	196/29008	7.88E-07	1.94E-05	1.3E-05
**GO:0034612**	BP	GO:0034612	response to tumor necrosis factor	24/1133	196/29008	7.88E-07	1.94E-05	1.3E-05
**GO:0030218**	BP	GO:0030218	erythrocyte differentiation	20/1133	143/29008	7.9E-07	1.94E-05	1.3E-05
**GO:0007517**	BP	GO:0007517	muscle organ development	35/1133	360/29008	8.1E-07	1.97E-05	1.32E-05
**GO:0006612**	BP	GO:0006612	protein targeting to membrane	18/1133	119/29008	8.68E-07	2.1E-05	1.41E-05
**GO:0018107**	BP	GO:0018107	peptidyl-threonine phosphorylation	18/1133	119/29008	8.68E-07	2.1E-05	1.41E-05
**GO:2001235**	BP	GO:2001235	positive regulation of apoptotic signaling pathway	21/1133	157/29008	8.94E-07	2.14E-05	1.43E-05
**GO:0060966**	BP	GO:0060966	regulation of gene silencing by RNA	12/1133	54/29008	8.94E-07	2.14E-05	1.43E-05
**GO:0016051**	BP	GO:0016051	carbohydrate biosynthetic process	24/1133	198/29008	9.49E-07	2.25E-05	1.51E-05
**GO:0031060**	BP	GO:0031060	regulation of histone methylation	15/1133	85/29008	9.49E-07	2.25E-05	1.51E-05
**GO:0060538**	BP	GO:0060538	skeletal muscle organ development	25/1133	212/29008	9.51E-07	2.25E-05	1.51E-05
**GO:0014706**	BP	GO:0014706	striated muscle tissue development	41/1133	461/29008	9.99E-07	2.35E-05	1.57E-05
**GO:0072659**	BP	GO:0072659	protein localization to plasma membrane	31/1133	301/29008	1.01E-06	2.36E-05	1.58E-05
**GO:0031669**	BP	GO:0031669	cellular response to nutrient levels	24/1133	199/29008	1.04E-06	2.42E-05	1.62E-05
**GO:0006458**	BP	GO:0006458	'de novo' protein folding	10/1133	37/29008	1.06E-06	2.44E-05	1.64E-05
**GO:0051084**	BP	GO:0051084	'de novo' posttranslational protein folding	10/1133	37/29008	1.06E-06	2.44E-05	1.64E-05
**GO:0062197**	BP	GO:0062197	cellular response to chemical stress	33/1133	333/29008	1.08E-06	2.49E-05	1.67E-05
**GO:0019058**	BP	GO:0019058	viral life cycle	28/1133	257/29008	1.09E-06	2.49E-05	1.67E-05
**GO:0034250**	BP	GO:0034250	positive regulation of cellular amide metabolic process	21/1133	159/29008	1.1E-06	2.49E-05	1.67E-05
**GO:0051098**	BP	GO:0051098	regulation of binding	37/1133	397/29008	1.1E-06	2.49E-05	1.67E-05
**GO:0071900**	BP	GO:0071900	regulation of protein serine/threonine kinase activity	34/1133	349/29008	1.11E-06	2.49E-05	1.67E-05
**GO:0050779**	BP	GO:0050779	RNA destabilization	15/1133	86/29008	1.11E-06	2.49E-05	1.67E-05
**GO:0061014**	BP	GO:0061014	positive regulation of mRNA catabolic process	15/1133	86/29008	1.11E-06	2.49E-05	1.67E-05
**GO:0002695**	BP	GO:0002695	negative regulation of leukocyte activation	24/1133	200/29008	1.14E-06	2.53E-05	1.7E-05
**GO:0046578**	BP	GO:0046578	regulation of Ras protein signal transduction	24/1133	200/29008	1.14E-06	2.53E-05	1.7E-05
**GO:0000245**	BP	GO:0000245	spliceosomal complex assembly	11/1133	46/29008	1.17E-06	2.59E-05	1.73E-05
**GO:0022409**	BP	GO:0022409	positive regulation of cell-cell adhesion	30/1133	288/29008	1.18E-06	2.61E-05	1.75E-05
**GO:0050852**	BP	GO:0050852	T cell receptor signaling pathway	19/1133	134/29008	1.2E-06	2.63E-05	1.76E-05
**GO:0006403**	BP	GO:0006403	RNA localization	22/1133	174/29008	1.31E-06	2.83E-05	1.9E-05
**GO:0050792**	BP	GO:0050792	regulation of viral process	22/1133	174/29008	1.31E-06	2.83E-05	1.9E-05
**GO:0071356**	BP	GO:0071356	cellular response to tumor necrosis factor	22/1133	174/29008	1.31E-06	2.83E-05	1.9E-05
**GO:0031330**	BP	GO:0031330	negative regulation of cellular catabolic process	27/1133	245/29008	1.35E-06	2.92E-05	1.96E-05
**GO:0006368**	BP	GO:0006368	transcription elongation from RNA polymerase II promoter	13/1133	66/29008	1.37E-06	2.95E-05	1.97E-05
**GO:0032535**	BP	GO:0032535	regulation of cellular component size	38/1133	417/29008	1.37E-06	2.95E-05	1.97E-05
**GO:0051604**	BP	GO:0051604	protein maturation	32/1133	321/29008	1.38E-06	2.95E-05	1.98E-05
**GO:1990778**	BP	GO:1990778	protein localization to cell periphery	35/1133	369/29008	1.43E-06	3.05E-05	2.04E-05
**GO:0006606**	BP	GO:0006606	protein import into nucleus	22/1133	175/29008	1.44E-06	3.05E-05	2.04E-05
**GO:0043967**	BP	GO:0043967	histone H4 acetylation	13/1133	67/29008	1.64E-06	3.46E-05	2.32E-05
**GO:0051052**	BP	GO:0051052	regulation of DNA metabolic process	36/1133	388/29008	1.71E-06	3.58E-05	2.4E-05
**GO:0000723**	BP	GO:0000723	telomere maintenance	18/1133	125/29008	1.81E-06	3.79E-05	2.54E-05
**GO:0031062**	BP	GO:0031062	positive regulation of histone methylation	11/1133	48/29008	1.84E-06	3.84E-05	2.57E-05
**GO:0002822**	BP	GO:0002822	regulation of adaptive immune response based on somatic recombination of immune	25/1133	220/29008	1.89E-06	3.92E-05	2.62E-05
**GO:0045860**	BP	GO:0045860	positive regulation of protein kinase activity	34/1133	358/29008	1.96E-06	4.05E-05	2.71E-05
**GO:0031503**	BP	GO:0031503	protein-containing complex localization	28/1133	265/29008	2.01E-06	4.12E-05	2.76E-05
**GO:0044403**	BP	GO:0044403	biological process involved in symbiotic interaction	28/1133	265/29008	2.01E-06	4.12E-05	2.76E-05
**GO:0071426**	BP	GO:0071426	ribonucleoprotein complex export from nucleus	14/1133	79/29008	2.06E-06	4.22E-05	2.83E-05
**GO:0034341**	BP	GO:0034341	response to interferon-gamma	19/1133	139/29008	2.1E-06	4.28E-05	2.87E-05
**GO:0002819**	BP	GO:0002819	regulation of adaptive immune response	26/1133	236/29008	2.12E-06	4.31E-05	2.88E-05
**GO:0048025**	BP	GO:0048025	negative regulation of mRNA splicing, via spliceosome	8/1133	24/29008	2.22E-06	4.48E-05	3E-05
**GO:0051054**	BP	GO:0051054	positive regulation of DNA metabolic process	25/1133	222/29008	2.22E-06	4.48E-05	3E-05
**GO:0018210**	BP	GO:0018210	peptidyl-threonine modification	18/1133	127/29008	2.28E-06	4.57E-05	3.06E-05
**GO:0043280**	BP	GO:0043280	positive regulation of cysteine-type endopeptidase activity involved in apoptotic proc	18/1133	127/29008	2.28E-06	4.57E-05	3.06E-05
**GO:0042093**	BP	GO:0042093	T-helper cell differentiation	13/1133	69/29008	2.33E-06	4.59E-05	3.07E-05
**GO:0070972**	BP	GO:0070972	protein localization to endoplasmic reticulum	13/1133	69/29008	2.33E-06	4.59E-05	3.07E-05
**GO:0006479**	BP	GO:0006479	protein methylation	23/1133	194/29008	2.33E-06	4.59E-05	3.07E-05
**GO:0008213**	BP	GO:0008213	protein alkylation	23/1133	194/29008	2.33E-06	4.59E-05	3.07E-05
**GO:0016311**	BP	GO:0016311	dephosphorylation	33/1133	345/29008	2.36E-06	4.64E-05	3.11E-05
**GO:0035710**	BP	GO:0035710	CD4-positive, alpha-beta T cell activation	17/1133	115/29008	2.4E-06	4.68E-05	3.14E-05
**GO:0032088**	BP	GO:0032088	negative regulation of NF-kappaB transcription factor activity	14/1133	80/29008	2.41E-06	4.68E-05	3.14E-05
**GO:0071166**	BP	GO:0071166	ribonucleoprotein complex localization	14/1133	80/29008	2.41E-06	4.68E-05	3.14E-05
**GO:0002573**	BP	GO:0002573	myeloid leukocyte differentiation	26/1133	238/29008	2.48E-06	4.79E-05	3.21E-05

**GO:0097191**	BP	GO:0097191	extrinsic apoptotic signaling pathway	26/1133	238/29008	2.48E-06	4.79E-05	3.21E-05
**GO:0006260**	BP	GO:0006260	DNA replication	27/1133	253/29008	2.51E-06	4.82E-05	3.23E-05
**GO:0061136**	BP	GO:0061136	regulation of proteasomal protein catabolic process	22/1133	181/29008	2.54E-06	4.83E-05	3.23E-05
**GO:0015931**	BP	GO:0015931	nucleobase-containing compound transport	23/1133	195/29008	2.54E-06	4.83E-05	3.23E-05
**GO:0030511**	BP	GO:0030511	positive regulation of transforming growth factor beta receptor signaling pathway	9/1133	32/29008	2.55E-06	4.83E-05	3.23E-05
**GO:1903846**	BP	GO:1903846	positive regulation of cellular response to transforming growth factor beta stimulus	9/1133	32/29008	2.55E-06	4.83E-05	3.23E-05
**GO:0010998**	BP	GO:0010998	regulation of translational initiation by eIF2 alpha phosphorylation	6/1133	12/29008	2.64E-06	4.99E-05	3.34E-05
**GO:0002294**	BP	GO:0002294	CD4-positive, alpha-beta T cell differentiation involved in immune response	13/1133	70/29008	2.75E-06	5.18E-05	3.47E-05
**GO:0002711**	BP	GO:0002711	positive regulation of T cell mediated immunity	14/1133	81/29008	2.81E-06	5.27E-05	3.53E-05
**GO:0006323**	BP	GO:0006323	DNA packaging	20/1133	155/29008	2.85E-06	5.31E-05	3.56E-05
**GO:0046632**	BP	GO:0046632	alpha-beta T cell differentiation	18/1133	129/29008	2.87E-06	5.34E-05	3.57E-05
**GO:1903039**	BP	GO:1903039	positive regulation of leukocyte cell-cell adhesion	26/1133	240/29008	2.9E-06	5.38E-05	3.6E-05
**GO:0006474**	BP	GO:0006474	N-terminal protein amino acid acetylation	7/1133	18/29008	2.97E-06	5.48E-05	3.67E-05
**GO:0033673**	BP	GO:0033673	negative regulation of kinase activity	25/1133	226/29008	3.07E-06	5.66E-05	3.79E-05
**GO:0034599**	BP	GO:0034599	cellular response to oxidative stress	28/1133	271/29008	3.11E-06	5.7E-05	3.82E-05
**GO:0036293**	BP	GO:0036293	response to decreased oxygen levels	26/1133	241/29008	3.13E-06	5.73E-05	3.84E-05
**GO:0032200**	BP	GO:0032200	telomere organization	18/1133	130/29008	3.21E-06	5.85E-05	3.92E-05
**GO:0002293**	BP	GO:0002293	alpha-beta T cell differentiation involved in immune response	13/1133	71/29008	3.25E-06	5.9E-05	3.95E-05
**GO:0006334**	BP	GO:0006334	nucleosome assembly	14/1133	82/29008	3.27E-06	5.9E-05	3.95E-05
**GO:0061157**	BP	GO:0061157	mRNA destabilization	14/1133	82/29008	3.27E-06	5.9E-05	3.95E-05
**GO:0051235**	BP	GO:0051235	maintenance of location	32/1133	335/29008	3.44E-06	6.18E-05	4.14E-05
**GO:0022604**	BP	GO:0022604	regulation of cell morphogenesis	32/1133	337/29008	3.9E-06	6.98E-05	4.68E-05
**GO:1903038**	BP	GO:1903038	negative regulation of leukocyte cell-cell adhesion	19/1133	145/29008	3.98E-06	7.1E-05	4.75E-05
**GO:0071559**	BP	GO:0071559	response to transforming growth factor beta	25/1133	230/29008	4.21E-06	7.49E-05	5.02E-05
**GO:0032944**	BP	GO:0032944	regulation of mononuclear cell proliferation	26/1133	245/29008	4.24E-06	7.52E-05	5.04E-05
**GO:0043255**	BP	GO:0043255	regulation of carbohydrate biosynthetic process	16/1133	108/29008	4.59E-06	8.11E-05	5.43E-05
**GO:0002253**	BP	GO:0002253	activation of immune response	38/1133	439/29008	4.65E-06	8.19E-05	5.49E-05
**GO:1901990**	BP	GO:1901990	regulation of mitotic cell cycle phase transition	29/1133	293/29008	4.89E-06	8.58E-05	5.75E-05
**GO:0071103**	BP	GO:0071103	DNA conformation change	23/1133	203/29008	5.04E-06	8.82E-05	5.91E-05
**GO:0045861**	BP	GO:0045861	negative regulation of proteolysis	33/1133	358/29008	5.23E-06	9.12E-05	6.11E-05
**GO:0031098**	BP	GO:0031098	stress-activated protein kinase signaling cascade	26/1133	248/29008	5.3E-06	9.21E-05	6.17E-05
**GO:2001056**	BP	GO:2001056	positive regulation of cysteine-type endopeptidase activity	19/1133	148/29008	5.4E-06	9.35E-05	6.26E-05
**GO:0071496**	BP	GO:0071496	cellular response to external stimulus	29/1133	295/29008	5.58E-06	9.64E-05	6.46E-05
**GO:0000462**	BP	GO:0000462	maturation of SSU-rRNA from tricistronic rRNA transcript (SSU-rRNA, 5.8S rRNA, LS	9/1133	35/29008	5.78E-06	9.95E-05	6.66E-05
**GO:0043491**	BP	GO:0043491	protein kinase B signaling	23/1133	205/29008	5.95E-06	0.0001019	6.83E-05
**GO:0034063**	BP	GO:0034063	stress granule assembly	8/1133	27/29008	6.04E-06	0.0001033	6.92E-05
**GO:0042692**	BP	GO:0042692	muscle cell differentiation	37/1133	428/29008	6.37E-06	0.0001084	7.27E-05
**GO:0022408**	BP	GO:0022408	negative regulation of cell-cell adhesion	23/1133	206/29008	6.45E-06	0.0001095	7.34E-05
**GO:0045638**	BP	GO:0045638	negative regulation of myeloid cell differentiation	14/1133	87/29008	6.71E-06	0.0001136	7.61E-05
**GO:0034968**	BP	GO:0034968	histone lysine methylation	17/1133	124/29008	6.85E-06	0.0001155	7.74E-05
**GO:0007623**	BP	GO:0007623	circadian rhythm	24/1133	222/29008	7.14E-06	0.0001198	8.03E-05
**GO:0016236**	BP	GO:0016236	macroautophagy	24/1133	222/29008	7.14E-06	0.0001198	8.03E-05
**GO:0043547**	BP	GO:0043547	positive regulation of GTPase activity	25/1133	237/29008	7.17E-06	0.0001198	8.03E-05
**GO:0071346**	BP	GO:0071346	cellular response to interferon-gamma	16/1133	112/29008	7.42E-06	0.0001236	8.28E-05
**GO:0051403**	BP	GO:0051403	stress-activated MAPK cascade	25/1133	238/29008	7.72E-06	0.0001282	8.59E-05
**GO:0006470**	BP	GO:0006470	protein dephosphorylation	25/1133	239/29008	8.3E-06	0.0001375	9.21E-05
**GO:0043254**	BP	GO:0043254	regulation of protein-containing complex assembly	38/1133	451/29008	8.66E-06	0.0001430	9.58E-05
**GO:2001243**	BP	GO:2001243	negative regulation of intrinsic apoptotic signaling pathway	15/1133	101/29008	8.77E-06	0.0001444	9.67E-05
**GO:0008631**	BP	GO:0008631	intrinsic apoptotic signaling pathway in response to oxidative stress	10/1133	46/29008	8.98E-06	0.0001469	9.84E-05
**GO:0051898**	BP	GO:0051898	negative regulation of protein kinase B signaling	10/1133	46/29008	8.98E-06	0.0001469	9.84E-05
**GO:0002292**	BP	GO:0002292	T cell differentiation involved in immune response	13/1133	78/29008	9.59E-06	0.0001561	0.0001046
**GO:0050670**	BP	GO:0050670	regulation of lymphocyte proliferation	25/1133	241/29008	9.6E-06	0.0001561	0.0001046
**GO:0007163**	BP	GO:0007163	establishment or maintenance of cell polarity	24/1133	226/29008	9.68E-06	0.0001564	0.0001047
**GO:0071560**	BP	GO:0071560	cellular response to transforming growth factor beta stimulus	24/1133	226/29008	9.68E-06	0.0001564	0.0001047
**GO:0039531**	BP	GO:0039531	regulation of viral-induced cytoplasmic pattern recognition receptor signaling pathwa	7/1133	21/29008	9.78E-06	0.0001564	0.0001047
**GO:0043555**	BP	GO:0043555	regulation of translation in response to stress	7/1133	21/29008	9.78E-06	0.0001564	0.0001047
**GO:2001236**	BP	GO:2001236	regulation of extrinsic apoptotic signaling pathway	20/1133	168/29008	9.79E-06	0.0001564	0.0001047
**GO:0051817**	BP	GO:0051817	modulation of process of other organism involved in symbiotic interaction	12/1133	67/29008	9.8E-06	0.0001564	0.0001047
**GO:1902105**	BP	GO:1902105	regulation of leukocyte differentiation	30/1133	320/29008	9.93E-06	0.0001581	0.0001059
**GO:0031365**	BP	GO:0031365	N-terminal protein amino acid modification	8/1133	29/29008	1.09E-05	0.0001725	0.0001155
**GO:0090502**	BP	GO:0090502	RNA phosphodiester bond hydrolysis, endonucleolytic	8/1133	29/29008	1.09E-05	0.0001725	0.0001155
**GO:0006094**	BP	GO:0006094	gluconeogenesis	13/1133	79/29008	1.11E-05	0.0001741	0.0001166
**GO:0033143**	BP	GO:0033143	regulation of intracellular steroid hormone receptor signaling pathway	13/1133	79/29008	1.11E-05	0.0001741	0.0001166
**GO:0008361**	BP	GO:0008361	regulation of cell size	23/1133	213/29008	1.12E-05	0.0001759	0.0001178
**GO:0046034**	BP	GO:0046034	ATP metabolic process	26/1133	259/29008	1.16E-05	0.0001807	0.0001210
**GO:0044770**	BP	GO:0044770	cell cycle phase transition	40/1133	492/29008	1.18E-05	0.0001837	0.0001230
**GO:1905475**	BP	GO:1905475	regulation of protein localization to membrane	22/1133	199/29008	1.19E-05	0.0001846	0.0001236
**GO:0044772**	BP	GO:0044772	mitotic cell cycle phase transition	35/1133	406/29008	1.19E-05	0.0001846	0.0001236
**GO:0048511**	BP	GO:0048511	rhythmic process	29/1133	307/29008	1.2E-05	0.0001858	0.0001244
**GO:0006979**	BP	GO:0006979	response to oxidative stress	35/1133	407/29008	1.26E-05	0.0001934	0.0001295
**GO:0000478**	BP	GO:0000478	endonucleolytic cleavage involved in rRNA processing	6/1133	15/29008	1.29E-05	0.0001971	0.0001320
**GO:0000479**	BP	GO:0000479	endonucleolytic cleavage of tricistronic rRNA transcript (SSU-rRNA, 5.8S rRNA, LSU	6/1133	15/29008	1.29E-05	0.0001971	0.0001320
**GO:0043923**	BP	GO:0043923	positive regulation by host of viral transcription	6/1133	15/29008	1.29E-05	0.0001971	0.0001320
**GO:1900153**	BP	GO:1900153	positive regulation of nuclear-transcribed mRNA catabolic process, deadenylation-de	6/1133	15/29008	1.29E-05	0.0001971	0.0001320
**GO:0036294**	BP	GO:0036294	cellular response to decreased oxygen levels	17/1133	130/29008	1.3E-05	0.0001971	0.0001320
**GO:0043367**	BP	GO:0043367	CD4-positive, alpha-beta T cell differentiation	14/1133	92/29008	1.3E-05	0.0001976	0.0001323
**GO:0070663**	BP	GO:0070663	regulation of leukocyte proliferation	26/1133	261/29008	1.32E-05	0.0002000	0.0001340
**GO:0150116**	BP	GO:0150116	regulation of cell-substrate junction organization	12/1133	69/29008	1.34E-05	0.0002016	0.0001350
**GO:0050856**	BP	GO:0050856	regulation of T cell receptor signaling pathway	10/1133	48/29008	1.34E-05	0.0002016	0.0001350
**GO:0007179**	BP	GO:0007179	transforming growth factor beta receptor signaling pathway	21/1133	186/29008	1.35E-05	0.0002020	0.0001353
**GO:1903829**	BP	GO:1903829	positive regulation of cellular protein localization	27/1133	277/29008	1.35E-05	0.0002020	0.0001353
**GO:0050870**	BP	GO:0050870	positive regulation of T cell activation	23/1133	216/29008	1.41E-05	0.0002099	0.0001406
**GO:0050868**	BP	GO:0050868	negative regulation of T cell activation	17/1133	131/29008	1.44E-05	0.0002135	0.0001430
**GO:0007162**	BP	GO:0007162	negative regulation of cell adhesion	29/1133	310/29008	1.45E-05	0.0002140	0.0001433
**GO:0007346**	BP	GO:0007346	regulation of mitotic cell cycle	38/1133	462/29008	1.49E-05	0.0002202	0.0001475
**GO:0032946**	BP	GO:0032946	positive regulation of mononuclear cell proliferation	18/1133	145/29008	1.51E-05	0.0002219	0.0001486
**GO:0072331**	BP	GO:0072331	signal transduction by p53 class mediator	18/1133	145/29008	1.51E-05	0.0002219	0.0001486
**GO:0051896**	BP	GO:0051896	regulation of protein kinase B signaling	20/1133	173/29008	1.52E-05	0.0002221	0.0001488
**GO:0000381**	BP	GO:0000381	regulation of alternative mRNA splicing, via spliceosome	11/1133	59/29008	1.54E-05	0.0002247	0.0001505
**GO:1901987**	BP	GO:1901987	regulation of cell cycle phase transition	33/1133	378/29008	1.62E-05	0.0002359	0.0001580
**GO:0042149**	BP	GO:0042149	cellular response to glucose starvation	10/1133	49/29008	1.63E-05	0.0002362	0.0001582
**GO:1990138**	BP	GO:1990138	neuron projection extension	22/1133	203/29008	1.63E-05	0.0002362	0.0001582
**GO:0071453**	BP	GO:0071453	cellular response to oxygen levels	18/1133	146/29008	1.66E-05	0.0002400	0.0001608
**GO:0008625**	BP	GO:0008625	extrinsic apoptotic signaling pathway via death domain receptors	13/1133	82/29008	1.68E-05	0.0002413	0.0001616
**GO:0019319**	BP	GO:0019319	hexose biosynthetic process	13/1133	82/29008	1.68E-05	0.0002413	0.0001616
**GO:0009314**	BP	GO:0009314	response to radiation	36/1133	430/29008	1.7E-05	0.0002433	0.0001630
**GO:0010212**	BP	GO:0010212	response to ionizing radiation	17/1133	133/29008	1.76E-05	0.0002506	0.0001679
**GO:0032434**	BP	GO:0032434	regulation of proteasomal ubiquitin-dependent protein catabolic process	17/1133	133/29008	1.76E-05	0.0002506	0.0001679
**GO:0019221**	BP	GO:0019221	cytokine-mediated signaling pathway	34/1133	397/29008	1.81E-05	0.0002571	0.0001722
**GO:0006406**	BP	GO:0006406	mRNA export from nucleus	11/1133	60/29008	1.82E-05	0.0002571	0.0001722
**GO:0071427**	BP	GO:0071427	mRNA-containing ribonucleoprotein complex export from nucleus	11/1133	60/29008	1.82E-05	0.0002571	0.0001722
**GO:2000637**	BP	GO:2000637	positive regulation of gene silencing by miRNA	8/1133	31/29008	1.87E-05	0.0002636	0.0001765
**GO:0051651**	BP	GO:0051651	maintenance of location in cell	23/1133	220/29008	1.9E-05	0.0002668	0.0001787
**GO:0033120**	BP	GO:0033120	positive regulation of RNA splicing	10/1133	50/29008	1.96E-05	0.0002756	0.0001846
**GO:0032970**	BP	GO:0032970	regulation of actin filament-based process	35/1133	416/29008	1.99E-05	0.0002789	0.0001868
**GO:0031348**	BP	GO:0031348	negative regulation of defense response	24/1133	236/29008	2E-05	0.0002789	0.0001868
**GO:0030307**	BP	GO:0030307	positive regulation of cell growth	22/1133	206/29008	2.05E-05	0.0002856	0.0001913
**GO:0051851**	BP	GO:0051851	modulation by host of symbiont process	11/1133	61/29008	2.14E-05	0.0002971	0.0001990
**GO:0010256**	BP	GO:0010256	endomembrane system organization	39/1133	488/29008	2.22E-05	0.0003073	0.0002058
**GO:0007015**	BP	GO:0007015	actin filament organization	37/1133	453/29008	2.24E-05	0.0003089	0.0002069
**GO:0002768**	BP	GO:0002768	immune response-regulating cell surface receptor signaling pathway	31/1133	351/29008	2.31E-05	0.0003184	0.0002133
**GO:0032956**	BP	GO:0032956	regulation of actin cytoskeleton organization	32/1133	368/29008	2.34E-05	0.0003214	0.0002152
**GO:0060148**	BP	GO:0060148	positive regulation of posttranscriptional gene silencing	8/1133	32/29008	2.41E-05	0.0003298	0.0002209
**GO:0040029**	BP	GO:0040029	regulation of gene expression, epigenetic	16/1133	123/29008	2.47E-05	0.0003377	0.0002262
**GO:0016525**	BP	GO:0016525	negative regulation of angiogenesis	15/1133	110/29008	2.5E-05	0.0003404	0.0002280
**GO:0010952**	BP	GO:0010952	positive regulation of peptidase activity	20/1133	179/29008	2.5E-05	0.0003404	0.0002280
**GO:0033157**	BP	GO:0033157	regulation of intracellular protein transport	23/1133	224/29008	2.53E-05	0.0003430	0.0002297
**GO:0045787**	BP	GO:0045787	positive regulation of cell cycle	30/1133	336/29008	2.53E-05	0.0003430	0.0002297
**GO:0034405**	BP	GO:0034405	response to fluid shear stress	7/1133	24/29008	2.63E-05	0.0003545	0.0002374
**GO:0050851**	BP	GO:0050851	antigen receptor-mediated signaling pathway	28/1133	304/29008	2.67E-05	0.0003598	0.0002410
**GO:0002709**	BP	GO:0002709	regulation of T cell mediated immunity	15/1133	111/29008	2.79E-05	0.0003726	0.0002495
**GO:1903707**	BP	GO:1903707	negative regulation of hemopoiesis	15/1133	111/29008	2.79E-05	0.0003726	0.0002495
**GO:0060968**	BP	GO:0060968	regulation of gene silencing	12/1133	74/29008	2.79E-05	0.0003726	0.0002495
**GO:0000288**	BP	GO:0000288	nuclear-transcribed mRNA catabolic process, deadenylation-dependent decay	10/1133	52/29008	2.82E-05	0.0003756	0.0002516
**GO:0032527**	BP	GO:0032527	protein exit from endoplasmic reticulum	9/1133	42/29008	2.85E-05	0.0003775	0.0002528
**GO:0043001**	BP	GO:0043001	Golgi to plasma membrane protein transport	9/1133	42/29008	2.85E-05	0.0003775	0.0002528
**GO:0072538**	BP	GO:0072538	T-helper 17 type immune response	9/1133	42/29008	2.85E-05	0.0003775	0.0002528
**GO:0006893**	BP	GO:0006893	Golgi to plasma membrane transport	11/1133	63/29008	2.94E-05	0.0003873	0.0002594
**GO:0007000**	BP	GO:0007000	nucleolus organization	6/1133	17/29008	2.99E-05	0.0003939	0.0002638
**GO:2000181**	BP	GO:2000181	negative regulation of blood vessel morphogenesis	15/1133	112/29008	3.1E-05	0.0004075	0.0002729
**GO:0002429**	BP	GO:0002429	immune response-activating cell surface receptor signaling pathway	30/1133	340/29008	3.17E-05	0.0004147	0.0002778
**GO:0009991**	BP	GO:0009991	response to extracellular stimulus	33/1133	391/29008	3.2E-05	0.0004164	0.0002789
**GO:0000380**	BP	GO:0000380	alternative mRNA splicing, via spliceosome	12/1133	75/29008	3.2E-05	0.0004164	0.0002789
**GO:1905897**	BP	GO:1905897	regulation of response to endoplasmic reticulum stress	12/1133	75/29008	3.2E-05	0.0004164	0.0002789
**GO:0051250**	BP	GO:0051250	negative regulation of lymphocyte activation	19/1133	168/29008	3.34E-05	0.0004317	0.0002891
**GO:0002757**	BP	GO:0002757	immune response-activating signal transduction	30/1133	341/29008	3.34E-05	0.0004317	0.0002891
**GO:0032784**	BP	GO:0032784	regulation of DNA-templated transcription, elongation	10/1133	53/29008	3.35E-05	0.0004317	0.0002891
**GO:0038066**	BP	GO:0038066	p38MAPK cascade	10/1133	53/29008	3.35E-05	0.0004317	0.0002891
**GO:0001836**	BP	GO:0001836	release of cytochrome c from mitochondria	11/1133	64/29008	3.42E-05	0.0004383	0.0002935

**GO:0070534**	BP	GO:0070534	protein K63-linked ubiquitination	11/1133	64/29008	3.42E-05	0.0004383	0.0002935
**GO:0032680**	BP	GO:0032680	regulation of tumor necrosis factor production	20/1133	183/29008	3.45E-05	0.0004393	0.0002943
**GO:1901343**	BP	GO:1901343	negative regulation of vasculature development	15/1133	113/29008	3.45E-05	0.0004393	0.0002943
**GO:0002460**	BP	GO:0002460	adaptive immune response based on somatic recombination of immune receptors bu	38/1133	480/29008	3.45E-05	0.0004393	0.0002943
**GO:0032872**	BP	GO:0032872	regulation of stress-activated MAPK cascade	21/1133	198/29008	3.48E-05	0.0004411	0.0002954
**GO:0046633**	BP	GO:0046633	alpha-beta T cell proliferation	9/1133	43/29008	3.48E-05	0.0004412	0.0002955
**GO:0010950**	BP	GO:0010950	positive regulation of endopeptidase activity	19/1133	169/29008	3.63E-05	0.0004583	0.0003070
**GO:0051101**	BP	GO:0051101	regulation of DNA binding	16/1133	127/29008	3.69E-05	0.0004643	0.0003110
**GO:0018022**	BP	GO:0018022	peptidyl-lysine methylation	17/1133	141/29008	3.77E-05	0.0004745	0.0003178
**GO:0051702**	BP	GO:0051702	biological process involved in interaction with symbiont	15/1133	114/29008	3.83E-05	0.0004806	0.0003219
**GO:0006111**	BP	GO:0006111	regulation of gluconeogenesis	10/1133	54/29008	3.97E-05	0.0004959	0.0003322
**GO:0043331**	BP	GO:0043331	response to dsRNA	11/1133	65/29008	3.97E-05	0.0004959	0.0003322
**GO:0046364**	BP	GO:0046364	monosaccharide biosynthetic process	13/1133	89/29008	4.11E-05	0.0005122	0.0003431
**GO:0061458**	BP	GO:0061458	reproductive system development	38/1133	484/29008	4.13E-05	0.0005130	0.0003436
**GO:0016579**	BP	GO:0016579	protein deubiquitination	15/1133	115/29008	4.25E-05	0.0005266	0.0003527
**GO:0006352**	BP	GO:0006352	DNA-templated transcription, initiation	14/1133	102/29008	4.29E-05	0.0005266	0.0003527
**GO:0048525**	BP	GO:0048525	negative regulation of viral process	14/1133	102/29008	4.29E-05	0.0005266	0.0003527
**GO:0150115**	BP	GO:0150115	cell-substrate junction organization	14/1133	102/29008	4.29E-05	0.0005266	0.0003527
**GO:2000060**	BP	GO:2000060	positive regulation of ubiquitin-dependent protein catabolic process	14/1133	102/29008	4.29E-05	0.0005266	0.0003527
**GO:0030575**	BP	GO:0030575	nuclear body organization	6/1133	18/29008	4.34E-05	0.0005271	0.0003531
**GO:0039535**	BP	GO:0039535	regulation of RIG-I signaling pathway	6/1133	18/29008	4.34E-05	0.0005271	0.0003531
**GO:1900151**	BP	GO:1900151	regulation of nuclear-transcribed mRNA catabolic process, deadenylation-dependent	6/1133	18/29008	4.34E-05	0.0005271	0.0003531
**GO:0070302**	BP	GO:0070302	regulation of stress-activated protein kinase signaling cascade	21/1133	201/29008	4.34E-05	0.0005271	0.0003531
**GO:0032388**	BP	GO:0032388	positive regulation of intracellular transport	20/1133	186/29008	4.35E-05	0.0005271	0.0003531
**GO:1903555**	BP	GO:1903555	regulation of tumor necrosis factor superfamily cytokine production	20/1133	186/29008	4.35E-05	0.0005271	0.0003531
**GO:0070482**	BP	GO:0070482	response to oxygen levels	27/1133	296/29008	4.37E-05	0.0005282	0.0003538
**GO:0070665**	BP	GO:0070665	positive regulation of leukocyte proliferation	18/1133	157/29008	4.45E-05	0.0005362	0.0003591
**GO:1901216**	BP	GO:1901216	positive regulation of neuron death	16/1133	129/29008	4.47E-05	0.0005378	0.0003602
**GO:0050671**	BP	GO:0050671	positive regulation of lymphocyte proliferation	17/1133	143/29008	4.52E-05	0.0005431	0.0003637
**GO:0071478**	BP	GO:0071478	cellular response to radiation	19/1133	172/29008	4.63E-05	0.0005530	0.0003704
**GO:0033233**	BP	GO:0033233	regulation of protein sumoylation	7/1133	26/29008	4.66E-05	0.0005530	0.0003704
**GO:0035020**	BP	GO:0035020	regulation of Rac protein signal transduction	7/1133	26/29008	4.66E-05	0.0005530	0.0003704
**GO:0046685**	BP	GO:0046685	response to arsenic-containing substance	7/1133	26/29008	4.66E-05	0.0005530	0.0003704
**GO:0050857**	BP	GO:0050857	positive regulation of antigen receptor-mediated signaling pathway	7/1133	26/29008	4.66E-05	0.0005530	0.0003704
**GO:0030308**	BP	GO:0030308	negative regulation of cell growth	21/1133	202/29008	4.67E-05	0.0005530	0.0003704
**GO:0090068**	BP	GO:0090068	positive regulation of cell cycle process	24/1133	249/29008	4.78E-05	0.0005639	0.0003777
**GO:0006919**	BP	GO:0006919	activation of cysteine-type endopeptidase activity involved in apoptotic process	12/1133	78/29008	4.78E-05	0.0005639	0.0003777
**GO:0002698**	BP	GO:0002698	negative regulation of immune effector process	16/1133	130/29008	4.92E-05	0.0005783	0.0003874
**GO:0032640**	BP	GO:0032640	tumor necrosis factor production	20/1133	188/29008	5.07E-05	0.0005948	0.0003984
**GO:0008637**	BP	GO:0008637	apoptotic mitochondrial changes	15/1133	117/29008	5.21E-05	0.0006100	0.0004085
**GO:0045637**	BP	GO:0045637	regulation of myeloid cell differentiation	22/1133	219/29008	5.24E-05	0.0006129	0.0004105
**GO:0002821**	BP	GO:0002821	positive regulation of adaptive immune response	18/1133	159/29008	5.26E-05	0.0006134	0.0004108
**GO:0045727**	BP	GO:0045727	positive regulation of translation	16/1133	131/29008	5.4E-05	0.0006277	0.0004204
**GO:0051091**	BP	GO:0051091	positive regulation of DNA-binding transcription factor activity	25/1133	267/29008	5.41E-05	0.0006277	0.0004204
**GO:0010594**	BP	GO:0010594	regulation of endothelial cell migration	19/1133	174/29008	5.42E-05	0.0006285	0.0004210
**GO:0006405**	BP	GO:0006405	RNA export from nucleus	12/1133	79/29008	5.44E-05	0.0006290	0.0004213
**GO:0018023**	BP	GO:0018023	peptidyl-lysine trimethylation	10/1133	56/29008	5.51E-05	0.0006352	0.0004255
**GO:0036490**	BP	GO:0036490	regulation of translation in response to endoplasmic reticulum stress	5/1133	12/29008	5.67E-05	0.0006514	0.0004363
**GO:0097201**	BP	GO:0097201	negative regulation of transcription from RNA polymerase II promoter in response to	5/1133	12/29008	5.67E-05	0.0006514	0.0004363
**GO:0043542**	BP	GO:0043542	endothelial cell migration	22/1133	221/29008	6.01E-05	0.0006875	0.0004605
**GO:0048639**	BP	GO:0048639	positive regulation of developmental growth	22/1133	221/29008	6.01E-05	0.0006875	0.0004605
**GO:0000289**	BP	GO:0000289	nuclear-transcribed mRNA poly(A) tail shortening	7/1133	27/29008	6.08E-05	0.0006911	0.0004629
**GO:0000469**	BP	GO:0000469	cleavage involved in rRNA processing	7/1133	27/29008	6.08E-05	0.0006911	0.0004629
**GO:0006376**	BP	GO:0006376	mRNA splice site selection	7/1133	27/29008	6.08E-05	0.0006911	0.0004629
**GO:1900076**	BP	GO:1900076	regulation of cellular response to insulin stimulus	12/1133	80/29008	6.18E-05	0.0007003	0.0004690
**GO:0071706**	BP	GO:0071706	tumor necrosis factor superfamily cytokine production	20/1133	191/29008	6.34E-05	0.0007169	0.0004802
**GO:0032496**	BP	GO:0032496	response to lipopolysaccharide	32/1133	388/29008	6.5E-05	0.0007324	0.0004906
**GO:1902903**	BP	GO:1902903	regulation of supramolecular fiber organization	32/1133	388/29008	6.5E-05	0.0007324	0.0004906
**GO:0051146**	BP	GO:0051146	striated muscle cell differentiation	28/1133	320/29008	6.63E-05	0.0007453	0.0004992
**GO:0006281**	BP	GO:0006281	DNA repair	38/1133	495/29008	6.65E-05	0.0007464	0.0004999
**GO:0048638**	BP	GO:0048638	regulation of developmental growth	33/1133	406/29008	6.68E-05	0.0007474	0.0005006
**GO:0007033**	BP	GO:0007033	vacuole organization	19/1133	177/29008	6.85E-05	0.0007649	0.0005124
**GO:0090257**	BP	GO:0090257	regulation of muscle system process	24/1133	255/29008	6.97E-05	0.0007769	0.0005204
**GO:0045927**	BP	GO:0045927	positive regulation of growth	28/1133	321/29008	7E-05	0.0007785	0.0005215
**GO:0051099**	BP	GO:0051099	positive regulation of binding	20/1133	193/29008	7.33E-05	0.0008140	0.0005452
**GO:0007369**	BP	GO:0007369	gastrulation	19/1133	178/29008	7.39E-05	0.0008178	0.0005477
**GO:0019076**	BP	GO:0019076	viral release from host cell	8/1133	37/29008	7.43E-05	0.0008178	0.0005477
**GO:0035890**	BP	GO:0035890	exit from host	8/1133	37/29008	7.43E-05	0.0008178	0.0005477
**GO:0035891**	BP	GO:0035891	exit from host cell	8/1133	37/29008	7.43E-05	0.0008178	0.0005477
**GO:0048608**	BP	GO:0048608	reproductive structure development	37/1133	480/29008	7.58E-05	0.0008333	0.0005581
**GO:0009411**	BP	GO:0009411	response to UV	17/1133	149/29008	7.62E-05	0.0008356	0.0005597
**GO:0002237**	BP	GO:0002237	response to molecule of bacterial origin	33/1133	409/29008	7.69E-05	0.0008401	0.0005627
**GO:0010631**	BP	GO:0010631	epithelial cell migration	27/1133	306/29008	7.71E-05	0.0008401	0.0005627
**GO:1901342**	BP	GO:1901342	regulation of vasculature development	27/1133	306/29008	7.71E-05	0.0008401	0.0005627
**GO:0032469**	BP	GO:0032469	endoplasmic reticulum calcium ion homeostasis	7/1133	28/29008	7.84E-05	0.0008522	0.0005708
**GO:0006006**	BP	GO:0006006	glucose metabolic process	22/1133	225/29008	7.85E-05	0.0008522	0.0005708
**GO:0000082**	BP	GO:0000082	G1/S transition of mitotic cell cycle	20/1133	194/29008	7.88E-05	0.0008535	0.0005717
**GO:0010632**	BP	GO:0010632	regulation of epithelial cell migration	23/1133	241/29008	7.92E-05	0.0008559	0.0005733
**GO:2001244**	BP	GO:2001244	positive regulation of intrinsic apoptotic signaling pathway	11/1133	70/29008	8.04E-05	0.0008672	0.0005809
**GO:0031667**	BP	GO:0031667	response to nutrient levels	30/1133	358/29008	8.16E-05	0.0008784	0.0005883
**GO:0070997**	BP	GO:0070997	neuron death	34/1133	428/29008	8.18E-05	0.0008784	0.0005883
**GO:0090132**	BP	GO:0090132	epithelium migration	27/1133	308/29008	8.6E-05	0.0009224	0.0006178
**GO:0010656**	BP	GO:0010656	negative regulation of muscle cell apoptotic process	10/1133	59/29008	8.74E-05	0.0009352	0.0006264
**GO:0006983**	BP	GO:0006983	ER overload response	5/1133	13/29008	8.92E-05	0.0009506	0.0006367
**GO:0060213**	BP	GO:0060213	positive regulation of nuclear-transcribed mRNA poly(A) tail shortening	5/1133	13/29008	8.92E-05	0.0009506	0.0006367
**GO:1902106**	BP	GO:1902106	negative regulation of leukocyte differentiation	14/1133	109/29008	8.98E-05	0.0009551	0.0006397
**GO:0030010**	BP	GO:0030010	establishment of cell polarity	17/1133	151/29008	9E-05	0.0009558	0.0006402
**GO:0051402**	BP	GO:0051402	neuron apoptotic process	27/1133	309/29008	9.08E-05	0.0009608	0.0006435
**GO:0045622**	BP	GO:0045622	regulation of T-helper cell differentiation	8/1133	38/29008	9.09E-05	0.0009608	0.0006435
**GO:0009408**	BP	GO:0009408	response to heat	13/1133	96/29008	9.18E-05	0.0009650	0.0006463
**GO:1901222**	BP	GO:1901222	regulation of NIK/NF-kappaB signaling	13/1133	96/29008	9.18E-05	0.0009650	0.0006463
**GO:0030888**	BP	GO:0030888	regulation of B cell proliferation	11/1133	71/29008	9.18E-05	0.0009650	0.0006463
**GO:0070646**	BP	GO:0070646	protein modification by small protein removal	16/1133	137/29008	9.28E-05	0.0009726	0.0006514
**GO:1903052**	BP	GO:1903052	positive regulation of proteolysis involved in cellular protein catabolic process	15/1133	123/29008	9.31E-05	0.0009726	0.0006514
**GO:0010720**	BP	GO:0010720	positive regulation of cell development	31/1133	378/29008	9.31E-05	0.0009726	0.0006514
**GO:0090130**	BP	GO:0090130	tissue migration	27/1133	310/29008	9.59E-05	0.0009999	0.0006697
**GO:0006109**	BP	GO:0006109	regulation of carbohydrate metabolic process	20/1133	197/29008	9.75E-05	0.0010134	0.0006788
**GO:0002824**	BP	GO:0002824	positive regulation of adaptive immune response based on somatic recombination of	17/1133	152/29008	9.78E-05	0.0010134	0.0006788
**GO:0051092**	BP	GO:0051092	positive regulation of NF-kappaB transcription factor activity	17/1133	152/29008	9.78E-05	0.0010134	0.0006788
**GO:0006275**	BP	GO:0006275	regulation of DNA replication	14/1133	110/29008	9.93E-05	0.0010269	0.0006878
**GO:0032647**	BP	GO:0032647	regulation of interferon-alpha production	7/1133	29/29008	9.98E-05	0.0010290	0.0006893
**GO:0009266**	BP	GO:0009266	response to temperature stimulus	18/1133	167/29008	9.99E-05	0.0010290	0.0006893
**GO:0002274**	BP	GO:0002274	myeloid leukocyte activation	24/1133	261/29008	0.0001002	0.0010306	0.0006903
**GO:0051656**	BP	GO:0051656	establishment of organelle localization	33/1133	415/29008	0.0001014	0.0010413	0.0006975
**GO:0002286**	BP	GO:0002286	T cell activation involved in immune response	15/1133	124/29008	0.0001020	0.0010459	0.0007005
**GO:0042158**	BP	GO:0042158	lipoprotein biosynthetic process	13/1133	97/29008	0.0001022	0.0010459	0.0007005
**GO:0050767**	BP	GO:0050767	regulation of neurogenesis	35/1133	451/29008	0.0001027	0.0010482	0.0007021
**GO:0043124**	BP	GO:0043124	negative regulation of I-kappaB kinase/NF-kappaB signaling	9/1133	49/29008	0.0001028	0.0010483	0.0007021
**GO:1901989**	BP	GO:1901989	positive regulation of cell cycle phase transition	14/1133	111/29008	0.0001095	0.0011140	0.0007461
**GO:0010639**	BP	GO:0010639	negative regulation of organelle organization	30/1133	364/29008	0.0001098	0.0011144	0.0007464
**GO:0046640**	BP	GO:0046640	regulation of alpha-beta T cell proliferation	8/1133	39/29008	0.0001104	0.0011187	0.0007493
**GO:0045619**	BP	GO:0045619	regulation of lymphocyte differentiation	20/1133	199/29008	0.0001120	0.0011327	0.0007587
**GO:0060560**	BP	GO:0060560	developmental growth involved in morphogenesis	25/1133	280/29008	0.0001160	0.0011705	0.0007840
**GO:0002703**	BP	GO:0002703	regulation of leukocyte mediated immunity	26/1133	297/29008	0.0001179	0.0011878	0.0007956
**GO:0043414**	BP	GO:0043414	macromolecule methylation	27/1133	314/29008	0.0001186	0.0011925	0.0007988
**GO:0046635**	BP	GO:0046635	positive regulation of alpha-beta T cell activation	11/1133	73/29008	0.0001188	0.0011925	0.0007988
**GO:0044843**	BP	GO:0044843	cell cycle G1/S phase transition	21/1133	216/29008	0.0001225	0.0012272	0.0008220
**GO:1901214**	BP	GO:1901214	regulation of neuron death	31/1133	384/29008	0.0001238	0.0012380	0.0008292
**GO:0039528**	BP	GO:0039528	cytoplasmic pattern recognition receptor signaling pathway in response to virus	7/1133	30/29008	0.0001258	0.0012556	0.0008410
**GO:0006997**	BP	GO:0006997	nucleus organization	16/1133	141/29008	0.0001307	0.0012993	0.0008703
**GO:1904375**	BP	GO:1904375	regulation of protein localization to cell periphery	16/1133	141/29008	0.0001307	0.0012993	0.0008703
**GO:0008630**	BP	GO:0008630	intrinsic apoptotic signaling pathway in response to DNA damage	14/1133	113/29008	0.0001329	0.0013171	0.0008822
**GO:0030521**	BP	GO:0030521	androgen receptor signaling pathway	8/1133	40/29008	0.0001333	0.0013171	0.0008822
**GO:0043500**	BP	GO:0043500	muscle adaptation	15/1133	127/29008	0.0001339	0.0013171	0.0008822
**GO:0045926**	BP	GO:0045926	negative regulation of growth	24/1133	266/29008	0.0001341	0.0013171	0.0008822
**GO:0036499**	BP	GO:0036499	PERK-mediated unfolded protein response	5/1133	14/29008	0.0001342	0.0013171	0.0008822
**GO:2000644**	BP	GO:2000644	regulation of receptor catabolic process	5/1133	14/29008	0.0001342	0.0013171	0.0008822
**GO:0043407**	BP	GO:0043407	negative regulation of MAP kinase activity	10/1133	62/29008	0.0001345	0.0013171	0.0008822
**GO:0010611**	BP	GO:0010611	regulation of cardiac muscle hypertrophy	11/1133	74/29008	0.0001347	0.0013171	0.0008822
**GO:0050688**	BP	GO:0050688	regulation of defense response to virus	11/1133	74/29008	0.0001347	0.0013171	0.0008822
**GO:0032984**	BP	GO:0032984	protein-containing complex disassembly	21/1133	218/29008	0.0001395	0.0013612	0.0009117
**GO:0006892**	BP	GO:0006892	post-Golgi vesicle-mediated transport	13/1133	100/29008	0.0001400	0.0013644	0.0009139
**GO:0061515**	BP	GO:0061515	myeloid cell development	12/1133	87/29008	0.0001415	0.0013765	0.0009220
**GO:0010951**	BP	GO:0010951	negative regulation of endopeptidase activity	19/1133	187/29008	0.0001426	0.0013839	0.0009270
**GO:0031346**	BP	GO:0031346	positive regulation of cell projection organization	34/1133	441/29008	0.0001451	0.0014059	0.0009417
**GO:0006469**	BP	GO:0006469	negative regulation of protein kinase activity	20/1133	203/29008	0.0001469	0.0014213	0.0009520
**GO:0016973**	BP	GO:0016973	poly(A)+ mRNA export from nucleus	6/1133	22/29008	0.0001525	0.0014724	0.0009862
**GO:0045765**	BP	GO:0045765	regulation of angiogenesis	26/1133	302/29008	0.0001542	0.0014783	0.0009902
**GO:0032655**	BP	GO:0032655	regulation of interleukin-12 production	10/1133	63/29008	0.0001544	0.0014783	0.0009902

**GO:0036473**	BP	GO:0036473	cell death in response to oxidative stress	13/1133	101/29008	0.0001551	0.0014783	0.0009902
**GO:0042116**	BP	GO:0042116	macrophage activation	13/1133	101/29008	0.0001551	0.0014783	0.0009902
**GO:0043502**	BP	GO:0043502	regulation of muscle adaptation	13/1133	101/29008	0.0001551	0.0014783	0.0009902
**GO:0045185**	BP	GO:0045185	maintenance of protein location	13/1133	101/29008	0.0001551	0.0014783	0.0009902
**GO:0050772**	BP	GO:0050772	positive regulation of axonogenesis	13/1133	101/29008	0.0001551	0.0014783	0.0009902
**GO:0032607**	BP	GO:0032607	interferon-alpha production	7/1133	31/29008	0.0001571	0.0014893	0.0009975
**GO:0032801**	BP	GO:0032801	receptor catabolic process	7/1133	31/29008	0.0001571	0.0014893	0.0009975
**GO:0072539**	BP	GO:0072539	T-helper 17 cell differentiation	7/1133	31/29008	0.0001571	0.0014893	0.0009975
**GO:0032436**	BP	GO:0032436	positive regulation of proteasomal ubiquitin-dependent protein catabolic process	12/1133	88/29008	0.0001581	0.0014969	0.0010026
**GO:0010613**	BP	GO:0010613	positive regulation of cardiac muscle hypertrophy	8/1133	41/29008	0.0001600	0.0015103	0.0010116
**GO:0071216**	BP	GO:0071216	cellular response to biotic stimulus	28/1133	337/29008	0.0001601	0.0015103	0.0010116
**GO:0001667**	BP	GO:0001667	ameboidal-type cell migration	34/1133	444/29008	0.0001649	0.0015524	0.0010398
**GO:0032715**	BP	GO:0032715	negative regulation of interleukin-6 production	9/1133	52/29008	0.0001659	0.0015590	0.0010442
**GO:1904950**	BP	GO:1904950	negative regulation of establishment of protein localization	16/1133	144/29008	0.0001673	0.0015697	0.0010514
**GO:0050900**	BP	GO:0050900	leukocyte migration	30/1133	373/29008	0.0001686	0.0015789	0.0010576
**GO:0032103**	BP	GO:0032103	positive regulation of response to external stimulus	33/1133	427/29008	0.0001726	0.0016135	0.0010807
**GO:0030316**	BP	GO:0030316	osteoclast differentiation	14/1133	116/29008	0.0001761	0.0016409	0.0010991
**GO:0043534**	BP	GO:0043534	blood vessel endothelial cell migration	14/1133	116/29008	0.0001761	0.0016409	0.0010991
**GO:0051346**	BP	GO:0051346	negative regulation of hydrolase activity	30/1133	374/29008	0.0001766	0.0016425	0.0011002
**GO:0002456**	BP	GO:0002456	T cell mediated immunity	16/1133	145/29008	0.0001814	0.0016837	0.0011278
**GO:0010906**	BP	GO:0010906	regulation of glucose metabolic process	15/1133	131/29008	0.0001897	0.0017575	0.0011772
**GO:0014742**	BP	GO:0014742	positive regulation of muscle hypertrophy	8/1133	42/29008	0.0001910	0.0017635	0.0011812
**GO:1900744**	BP	GO:1900744	regulation of p38MAPK cascade	8/1133	42/29008	0.0001910	0.0017635	0.0011812
**GO:0006888**	BP	GO:0006888	endoplasmic reticulum to Golgi vesicle-mediated transport	14/1133	117/29008	0.0001930	0.0017762	0.0011897
**GO:0014812**	BP	GO:0014812	muscle cell migration	14/1133	117/29008	0.0001930	0.0017762	0.0011897
**GO:0014743**	BP	GO:0014743	regulation of muscle hypertrophy	11/1133	77/29008	0.0001936	0.0017764	0.0011898
**GO:1901532**	BP	GO:1901532	regulation of hematopoietic progenitor cell differentiation	7/1133	32/29008	0.0001943	0.0017764	0.0011898
**GO:1905898**	BP	GO:1905898	positive regulation of response to endoplasmic reticulum stress	7/1133	32/29008	0.0001943	0.0017764	0.0011898
**GO:0033235**	BP	GO:0033235	positive regulation of protein sumoylation	5/1133	15/29008	0.0001949	0.0017764	0.0011898
**GO:0060211**	BP	GO:0060211	regulation of nuclear-transcribed mRNA poly(A) tail shortening	5/1133	15/29008	0.0001949	0.0017764	0.0011898
**GO:0019318**	BP	GO:0019318	hexose metabolic process	23/1133	256/29008	0.0001950	0.0017764	0.0011898
**GO:1901992**	BP	GO:1901992	positive regulation of mitotic cell cycle phase transition	12/1133	90/29008	0.0001964	0.0017858	0.0011962
**GO:0002366**	BP	GO:0002366	leukocyte activation involved in immune response	27/1133	324/29008	0.0001982	0.0017988	0.0012049
**GO:0000413**	BP	GO:0000413	protein peptidyl-prolyl isomerization	6/1133	23/29008	0.0001996	0.0018052	0.0012092
**GO:1900101**	BP	GO:1900101	regulation of endoplasmic reticulum unfolded protein response	6/1133	23/29008	0.0001996	0.0018052	0.0012092
**GO:0032615**	BP	GO:0032615	interleukin-12 production	10/1133	65/29008	0.0002015	0.0018198	0.0012189
**GO:0050727**	BP	GO:0050727	regulation of inflammatory response	28/1133	342/29008	0.0002045	0.0018436	0.0012349
**GO:0007030**	BP	GO:0007030	Golgi organization	15/1133	132/29008	0.0002064	0.0018575	0.0012442
**GO:0002700**	BP	GO:0002700	regulation of production of molecular mediator of immune response	18/1133	177/29008	0.0002087	0.0018726	0.0012543
**GO:0003300**	BP	GO:0003300	cardiac muscle hypertrophy	13/1133	104/29008	0.0002088	0.0018726	0.0012543
**GO:0044262**	BP	GO:0044262	cellular carbohydrate metabolic process	25/1133	291/29008	0.0002113	0.0018925	0.0012676
**GO:0035303**	BP	GO:0035303	regulation of dephosphorylation	15/1133	133/29008	0.0002244	0.0020057	0.0013435
**GO:0016925**	BP	GO:0016925	protein sumoylation	8/1133	43/29008	0.0002267	0.0020194	0.0013526
**GO:0046636**	BP	GO:0046636	negative regulation of alpha-beta T cell activation	8/1133	43/29008	0.0002267	0.0020194	0.0013526
**GO:0060761**	BP	GO:0060761	negative regulation of response to cytokine stimulus	10/1133	66/29008	0.0002293	0.0020395	0.0013661
**GO:0006816**	BP	GO:0006816	calcium ion transport	33/1133	434/29008	0.0002324	0.0020632	0.0013820
**GO:0032102**	BP	GO:0032102	negative regulation of response to external stimulus	31/1133	398/29008	0.0002337	0.0020714	0.0013874
**GO:0043523**	BP	GO:0043523	regulation of neuron apoptotic process	24/1133	276/29008	0.0002341	0.0020722	0.0013880
**GO:0048641**	BP	GO:0048641	regulation of skeletal muscle tissue development	7/1133	33/29008	0.0002384	0.0021061	0.0014107
**GO:0016458**	BP	GO:0016458	gene silencing	22/1133	243/29008	0.0002388	0.0021061	0.0014107
**GO:0002263**	BP	GO:0002263	cell activation involved in immune response	27/1133	328/29008	0.0002414	0.0021259	0.0014239
**GO:0006497**	BP	GO:0006497	protein lipidation	12/1133	92/29008	0.0002423	0.0021300	0.0014267
**GO:0000466**	BP	GO:0000466	maturation of 5.8S rRNA from tricistronic rRNA transcript (SSU-rRNA, 5.8S rRNA, LS	6/1133	24/29008	0.0002573	0.0022513	0.0015079
**GO:0016578**	BP	GO:0016578	histone deubiquitination	6/1133	24/29008	0.0002573	0.0022513	0.0015079
**GO:0032727**	BP	GO:0032727	positive regulation of interferon-alpha production	6/1133	24/29008	0.0002573	0.0022513	0.0015079
**GO:0045580**	BP	GO:0045580	regulation of T cell differentiation	17/1133	165/29008	0.0002652	0.0023158	0.0015512
**GO:0032651**	BP	GO:0032651	regulation of interleukin-1 beta production	12/1133	93/29008	0.0002684	0.0023368	0.0015652
**GO:0043535**	BP	GO:0043535	regulation of blood vessel endothelial cell migration	12/1133	93/29008	0.0002684	0.0023368	0.0015652
**GO:0001771**	BP	GO:0001771	immunological synapse formation	5/1133	16/29008	0.0002744	0.0023812	0.0015950
**GO:0042921**	BP	GO:0042921	glucocorticoid receptor signaling pathway	5/1133	16/29008	0.0002744	0.0023812	0.0015950
**GO:0017015**	BP	GO:0017015	regulation of transforming growth factor beta receptor signaling pathway	14/1133	121/29008	0.0002752	0.0023835	0.0015965
**GO:1901800**	BP	GO:1901800	positive regulation of proteasomal protein catabolic process	13/1133	107/29008	0.0002776	0.0024012	0.0016083
**GO:0032675**	BP	GO:0032675	regulation of interleukin-6 production	17/1133	166/29008	0.0002849	0.0024597	0.0016475
**GO:0010922**	BP	GO:0010922	positive regulation of phosphatase activity	7/1133	34/29008	0.0002901	0.0025011	0.0016753
**GO:1901224**	BP	GO:1901224	positive regulation of NIK/NF-kappaB signaling	10/1133	68/29008	0.0002945	0.0025349	0.0016979
**GO:1903321**	BP	GO:1903321	negative regulation of protein modification by small protein conjugation or removal	12/1133	94/29008	0.0002969	0.0025516	0.0017091
**GO:0043405**	BP	GO:0043405	regulation of MAP kinase activity	19/1133	198/29008	0.0002984	0.0025597	0.0017145
**GO:0051271**	BP	GO:0051271	negative regulation of cellular component movement	26/1133	315/29008	0.0002991	0.0025599	0.0017146
**GO:0042177**	BP	GO:0042177	negative regulation of protein catabolic process	14/1133	122/29008	0.0002998	0.0025599	0.0017146
**GO:0042542**	BP	GO:0042542	response to hydrogen peroxide	14/1133	122/29008	0.0002998	0.0025599	0.0017146
**GO:0014897**	BP	GO:0014897	striated muscle hypertrophy	13/1133	108/29008	0.0003045	0.0025946	0.0017379
**GO:0045913**	BP	GO:0045913	positive regulation of carbohydrate metabolic process	11/1133	81/29008	0.0003049	0.0025946	0.0017379
**GO:0042129**	BP	GO:0042129	regulation of T cell proliferation	18/1133	183/29008	0.0003151	0.0026771	0.0017931
**GO:0062012**	BP	GO:0062012	regulation of small molecule metabolic process	29/1133	369/29008	0.0003157	0.0026779	0.0017937
**GO:0050860**	BP	GO:0050860	negative regulation of T cell receptor signaling pathway	6/1133	25/29008	0.0003275	0.0027735	0.0018577
**GO:0010660**	BP	GO:0010660	regulation of muscle cell apoptotic process	12/1133	95/29008	0.0003280	0.0027735	0.0018577
**GO:1903900**	BP	GO:1903900	regulation of viral life cycle	16/1133	153/29008	0.0003358	0.0028351	0.0018990
**GO:0002064**	BP	GO:0002064	epithelial cell development	23/1133	266/29008	0.0003390	0.0028578	0.0019142
**GO:0031648**	BP	GO:0031648	protein destabilization	9/1133	57/29008	0.0003408	0.0028631	0.0019177
**GO:0043666**	BP	GO:0043666	regulation of phosphoprotein phosphatase activity	9/1133	57/29008	0.0003408	0.0028631	0.0019177
**GO:1901991**	BP	GO:1901991	negative regulation of mitotic cell cycle phase transition	17/1133	169/29008	0.0003518	0.0029516	0.0019770
**GO:1903844**	BP	GO:1903844	regulation of cellular response to transforming growth factor beta stimulus	14/1133	124/29008	0.0003550	0.0029731	0.0019914
**GO:0033044**	BP	GO:0033044	regulation of chromosome organization	18/1133	185/29008	0.0003597	0.0030036	0.0020118
**GO:0050770**	BP	GO:0050770	regulation of axonogenesis	18/1133	185/29008	0.0003597	0.0030036	0.0020118
**GO:0034502**	BP	GO:0034502	protein localization to chromosome	12/1133	96/29008	0.0003617	0.0030156	0.0020199
**GO:0031341**	BP	GO:0031341	regulation of cell killing	15/1133	139/29008	0.0003633	0.0030242	0.0020256
**GO:0014896**	BP	GO:0014896	muscle hypertrophy	13/1133	110/29008	0.0003650	0.0030288	0.0020288
**GO:0032760**	BP	GO:0032760	positive regulation of tumor necrosis factor production	13/1133	110/29008	0.0003650	0.0030288	0.0020288
**GO:0010907**	BP	GO:0010907	positive regulation of glucose metabolic process	8/1133	46/29008	0.0003678	0.0030375	0.0020346
**GO:0030890**	BP	GO:0030890	positive regulation of B cell proliferation	8/1133	46/29008	0.0003678	0.0030375	0.0020346
**GO:0045005**	BP	GO:0045005	DNA-dependent DNA replication maintenance of fidelity	8/1133	46/29008	0.0003678	0.0030375	0.0020346
**GO:2000514**	BP	GO:2000514	regulation of CD4-positive, alpha-beta T cell activation	10/1133	70/29008	0.0003746	0.0030706	0.0020567
**GO:0031958**	BP	GO:0031958	corticosteroid receptor signaling pathway	5/1133	17/29008	0.0003763	0.0030706	0.0020567
**GO:0032986**	BP	GO:0032986	protein-DNA complex disassembly	5/1133	17/29008	0.0003763	0.0030706	0.0020567
**GO:0070262**	BP	GO:0070262	peptidyl-serine dephosphorylation	5/1133	17/29008	0.0003763	0.0030706	0.0020567
**GO:0071243**	BP	GO:0071243	cellular response to arsenic-containing substance	5/1133	17/29008	0.0003763	0.0030706	0.0020567
**GO:0071498**	BP	GO:0071498	cellular response to fluid shear stress	5/1133	17/29008	0.0003763	0.0030706	0.0020567
**GO:1902916**	BP	GO:1902916	positive regulation of protein polyubiquitination	5/1133	17/29008	0.0003763	0.0030706	0.0020567
**GO:0002244**	BP	GO:0002244	hematopoietic progenitor cell differentiation	17/1133	170/29008	0.0003770	0.0030706	0.0020567
**GO:0007254**	BP	GO:0007254	JNK cascade	17/1133	170/29008	0.0003770	0.0030706	0.0020567
**GO:0008154**	BP	GO:0008154	actin polymerization or depolymerization	20/1133	218/29008	0.0003783	0.0030765	0.0020607
**GO:0007059**	BP	GO:0007059	chromosome segregation	27/1133	338/29008	0.0003881	0.0031511	0.0021107
**GO:0006754**	BP	GO:0006754	ATP biosynthetic process	9/1133	58/29008	0.0003895	0.0031534	0.0021122
**GO:0045058**	BP	GO:0045058	T cell selection	9/1133	58/29008	0.0003895	0.0031534	0.0021122
**GO:0034614**	BP	GO:0034614	cellular response to reactive oxygen species	15/1133	140/29008	0.0003925	0.0031679	0.0021219
**GO:0071887**	BP	GO:0071887	leukocyte apoptotic process	15/1133	140/29008	0.0003925	0.0031679	0.0021219
**GO:0002718**	BP	GO:0002718	regulation of cytokine production involved in immune response	12/1133	97/29008	0.0003984	0.0032103	0.0021503
**GO:1900246**	BP	GO:1900246	positive regulation of RIG-I signaling pathway	4/1133	10/29008	0.0004024	0.0032328	0.0021654
**GO:2000646**	BP	GO:2000646	positive regulation of receptor catabolic process	4/1133	10/29008	0.0004024	0.0032328	0.0021654
**GO:0002221**	BP	GO:0002221	pattern recognition receptor signaling pathway	17/1133	171/29008	0.0004037	0.0032384	0.0021691
**GO:0030970**	BP	GO:0030970	retrograde protein transport, ER to cytosol	6/1133	26/29008	0.0004118	0.0032784	0.0021959
**GO:0035278**	BP	GO:0035278	miRNA mediated inhibition of translation	6/1133	26/29008	0.0004118	0.0032784	0.0021959
**GO:0039529**	BP	GO:0039529	RIG-I signaling pathway	6/1133	26/29008	0.0004118	0.0032784	0.0021959
**GO:1903513**	BP	GO:1903513	endoplasmic reticulum to cytosol transport	6/1133	26/29008	0.0004118	0.0032784	0.0021959
**GO:2000316**	BP	GO:2000316	regulation of T-helper 17 type immune response	6/1133	26/29008	0.0004118	0.0032784	0.0021959
**GO:0019079**	BP	GO:0019079	viral genome replication	14/1133	126/29008	0.0004185	0.0033153	0.0022206
**GO:0019882**	BP	GO:0019882	antigen processing and presentation	14/1133	126/29008	0.0004185	0.0033153	0.0022206
**GO:0071214**	BP	GO:0071214	cellular response to abiotic stimulus	26/1133	322/29008	0.0004189	0.0033153	0.0022206
**GO:0104004**	BP	GO:0104004	cellular response to environmental stimulus	26/1133	322/29008	0.0004189	0.0033153	0.0022206
**GO:0050766**	BP	GO:0050766	positive regulation of phagocytosis	11/1133	84/29008	0.0004201	0.0033196	0.0022235
**GO:0048536**	BP	GO:0048536	spleen development	8/1133	47/29008	0.0004282	0.0033792	0.0022634
**GO:0009743**	BP	GO:0009743	response to carbohydrate	19/1133	204/29008	0.0004343	0.0034210	0.0022915
**GO:1903557**	BP	GO:1903557	positive regulation of tumor necrosis factor superfamily cytokine production	13/1133	112/29008	0.0004355	0.0034210	0.0022915
**GO:2000106**	BP	GO:2000106	regulation of leukocyte apoptotic process	13/1133	112/29008	0.0004355	0.0034210	0.0022915
**GO:0000819**	BP	GO:0000819	sister chromatid segregation	18/1133	188/29008	0.0004370	0.0034276	0.0022959
**GO:0007409**	BP	GO:0007409	axonogenesis	35/1133	487/29008	0.0004378	0.0034290	0.0022968
**GO:0043388**	BP	GO:0043388	positive regulation of DNA binding	9/1133	59/29008	0.0004439	0.0034721	0.0023257
**GO:0046486**	BP	GO:0046486	glycerolipid metabolic process	29/1133	377/29008	0.0004482	0.0035001	0.0023444
**GO:0002833**	BP	GO:0002833	positive regulation of response to biotic stimulus	17/1133	173/29008	0.0004620	0.0036030	0.0024133
**GO:0000302**	BP	GO:0000302	response to reactive oxygen species	18/1133	189/29008	0.0004657	0.0036161	0.0024221
**GO:0010921**	BP	GO:0010921	regulation of phosphatase activity	11/1133	85/29008	0.0004657	0.0036161	0.0024221
**GO:0051881**	BP	GO:0051881	regulation of mitochondrial membrane potential	11/1133	85/29008	0.0004657	0.0036161	0.0024221
**GO:0090501**	BP	GO:0090501	RNA phosphodiester bond hydrolysis	10/1133	72/29008	0.0004720	0.0036593	0.0024511
**GO:0032611**	BP	GO:0032611	interleukin-1 beta production	12/1133	99/29008	0.0004811	0.0037192	0.0024912
**GO:0062207**	BP	GO:0062207	regulation of pattern recognition receptor signaling pathway	12/1133	99/29008	0.0004811	0.0037192	0.0024912
**GO:0005996**	BP	GO:0005996	monosaccharide metabolic process	23/1133	273/29008	0.0004889	0.0037684	0.0025241
**GO:0006650**	BP	GO:0006650	glycerophospholipid metabolic process	23/1133	273/29008	0.0004889	0.0037684	0.0025241
**GO:0071902**	BP	GO:0071902	positive regulation of protein serine/threonine kinase activity	19/1133	206/29008	0.0004902	0.0037724	0.0025268
**GO:0061387**	BP	GO:0061387	regulation of extent of cell growth	14/1133	128/29008	0.0004915	0.0037724	0.0025268

**GO:2000045**	BP	GO:2000045	regulation of G1/S transition of mitotic cell cycle	14/1133	128/29008	0.000491	0.003772	0.002526
**GO:0001890**	BP	GO:0001890	placenta development	17/1133	174/29008	0.000493	0.003778	0.002531
**GO:0032635**	BP	GO:0032635	interleukin-6 production	17/1133	174/29008	0.000493	0.003778	0.002531
**GO:0050855**	BP	GO:0050855	regulation of B cell receptor signaling pathway	5/1133	18/29008	0.000504	0.003837	0.002570
**GO:1900037**	BP	GO:1900037	regulation of cellular response to hypoxia	5/1133	18/29008	0.000504	0.003837	0.002570
**GO:1902176**	BP	GO:1902176	negative regulation of oxidative stress-induced intrinsic apoptotic signaling pathway	5/1133	18/29008	0.000504	0.003837	0.002570
**GO:2000319**	BP	GO:2000319	regulation of T-helper 17 cell differentiation	5/1133	18/29008	0.000504	0.003837	0.002570
**GO:0002685**	BP	GO:0002685	regulation of leukocyte migration	20/1133	223/29008	0.000506	0.003847	0.002577
**GO:0007064**	BP	GO:0007064	mitotic sister chromatid cohesion	6/1133	27/29008	0.000512	0.003868	0.002591
**GO:0032781**	BP	GO:0032781	positive regulation of ATPase activity	6/1133	27/29008	0.000512	0.003868	0.002591
**GO:0040033**	BP	GO:0040033	negative regulation of translation, ncRNA-mediated	6/1133	27/29008	0.000512	0.003868	0.002591
**GO:0045974**	BP	GO:0045974	regulation of translation, ncRNA-mediated	6/1133	27/29008	0.000512	0.003868	0.002591
**GO:0010657**	BP	GO:0010657	muscle cell apoptotic process	12/1133	100/29008	0.000527	0.003977	0.002664
**GO:0033209**	BP	GO:0033209	tumor necrosis factor-mediated signaling pathway	10/1133	73/29008	0.000528	0.003977	0.002664
**GO:0030336**	BP	GO:0030336	negative regulation of cell migration	24/1133	293/29008	0.000559	0.004199	0.002812
**GO:0051258**	BP	GO:0051258	protein polymerization	24/1133	293/29008	0.000559	0.004199	0.002812
**GO:0002832**	BP	GO:0002832	negative regulation of response to biotic stimulus	13/1133	115/29008	0.000562	0.004213	0.002822
**GO:0050764**	BP	GO:0050764	regulation of phagocytosis	13/1133	115/29008	0.000562	0.004213	0.002822
**GO:0060324**	BP	GO:0060324	face development	9/1133	61/29008	0.000571	0.004270	0.002860
**GO:0010332**	BP	GO:0010332	response to gamma radiation	8/1133	49/29008	0.000573	0.004270	0.002860
**GO:0032873**	BP	GO:0032873	negative regulation of stress-activated MAPK cascade	8/1133	49/29008	0.000573	0.004270	0.002860
**GO:0070303**	BP	GO:0070303	negative regulation of stress-activated protein kinase signaling cascade	8/1133	49/29008	0.000573	0.004270	0.002860
**GO:0010506**	BP	GO:0010506	regulation of autophagy	22/1133	259/29008	0.000574	0.004273	0.002862
**GO:0016441**	BP	GO:0016441	posttranscriptional gene silencing	16/1133	161/29008	0.000592	0.004401	0.002948
**GO:2000144**	BP	GO:2000144	positive regulation of DNA-templated transcription, initiation	7/1133	38/29008	0.000594	0.004407	0.002952
**GO:0045444**	BP	GO:0045444	fat cell differentiation	22/1133	260/29008	0.000605	0.004481	0.003001
**GO:0071888**	BP	GO:0071888	macrophage apoptotic process	4/1133	11/29008	0.000612	0.004532	0.003036
**GO:1903828**	BP	GO:1903828	negative regulation of cellular protein localization	14/1133	131/29008	0.000621	0.004589	0.003073
**GO:0016577**	BP	GO:0016577	histone demethylation	6/1133	28/29008	0.000630	0.004636	0.003105
**GO:0070076**	BP	GO:0070076	histone lysine demethylation	6/1133	28/29008	0.000630	0.004636	0.003105
**GO:1902175**	BP	GO:1902175	regulation of oxidative stress-induced intrinsic apoptotic signaling pathway	6/1133	28/29008	0.000630	0.004636	0.003105
**GO:0001704**	BP	GO:0001704	formation of primary germ layer	12/1133	102/29008	0.000632	0.004636	0.003105
**GO:0006476**	BP	GO:0006476	protein deacetylation	12/1133	102/29008	0.000632	0.004636	0.003105
**GO:0043112**	BP	GO:0043112	receptor metabolic process	18/1133	194/29008	0.000635	0.004653	0.003116
**GO:0001960**	BP	GO:0001960	negative regulation of cytokine-mediated signaling pathway	9/1133	62/29008	0.000645	0.004723	0.003163
**GO:0007492**	BP	GO:0007492	endoderm development	10/1133	75/29008	0.000656	0.004791	0.003209
**GO:1901796**	BP	GO:1901796	regulation of signal transduction by p53 class mediator	10/1133	75/29008	0.000656	0.004791	0.003209
**GO:0002753**	BP	GO:0002753	cytoplasmic pattern recognition receptor signaling pathway	8/1133	50/29008	0.000659	0.004801	0.003216
**GO:0007039**	BP	GO:0007039	protein catabolic process in the vacuole	5/1133	19/29008	0.000662	0.004801	0.003216
**GO:0051571**	BP	GO:0051571	positive regulation of histone H3-K4 methylation	5/1133	19/29008	0.000662	0.004801	0.003216
**GO:0071786**	BP	GO:0071786	endoplasmic reticulum tubular network organization	5/1133	19/29008	0.000662	0.004801	0.003216
**GO:0140467**	BP	GO:0140467	integrated stress response signaling	5/1133	19/29008	0.000662	0.004801	0.003216
**GO:0042100**	BP	GO:0042100	B cell proliferation	13/1133	117/29008	0.000663	0.004801	0.003216
**GO:0007178**	BP	GO:0007178	transmembrane receptor protein serine/threonine kinase signaling pathway	29/1133	387/29008	0.000681	0.004921	0.003296
**GO:0034644**	BP	GO:0034644	cellular response to UV	11/1133	89/29008	0.000692	0.004990	0.003342
**GO:0010959**	BP	GO:0010959	regulation of metal ion transport	32/1133	443/29008	0.000692	0.004990	0.003342
**GO:0001706**	BP	GO:0001706	endoderm formation	7/1133	39/29008	0.000700	0.005017	0.003360
**GO:0001782**	BP	GO:0001782	B cell homeostasis	7/1133	39/29008	0.000700	0.005017	0.003360
**GO:0018208**	BP	GO:0018208	peptidyl-proline modification	7/1133	39/29008	0.000700	0.005017	0.003360
**GO:0031297**	BP	GO:0031297	replication fork processing	7/1133	39/29008	0.000700	0.005017	0.003360
**GO:0003012**	BP	GO:0003012	muscle system process	31/1133	425/29008	0.000710	0.005083	0.003404
**GO:0003158**	BP	GO:0003158	endothelium development	14/1133	133/29008	0.000723	0.005167	0.003461
**GO:0010675**	BP	GO:0010675	regulation of cellular carbohydrate metabolic process	16/1133	164/29008	0.000724	0.005174	0.003466
**GO:0030865**	BP	GO:0030865	cortical cytoskeleton organization	9/1133	63/29008	0.000727	0.005180	0.003469
**GO:0043966**	BP	GO:0043966	histone H3 acetylation	9/1133	63/29008	0.000727	0.005180	0.003469
**GO:0002704**	BP	GO:0002704	negative regulation of leukocyte mediated immunity	10/1133	76/29008	0.000730	0.005185	0.003473
**GO:2000573**	BP	GO:2000573	positive regulation of DNA biosynthetic process	10/1133	76/29008	0.000730	0.005185	0.003473
**GO:0045930**	BP	GO:0045930	negative regulation of mitotic cell cycle	20/1133	231/29008	0.000789	0.005598	0.003750
**GO:0042593**	BP	GO:0042593	glucose homeostasis	23/1133	283/29008	0.000802	0.005679	0.003804
**GO:0051147**	BP	GO:0051147	regulation of muscle cell differentiation	15/1133	150/29008	0.000813	0.005742	0.003846
**GO:1902806**	BP	GO:1902806	regulation of cell cycle G1/S phase transition	15/1133	150/29008	0.000813	0.005742	0.003846
**GO:0051893**	BP	GO:0051893	regulation of focal adhesion assembly	9/1133	64/29008	0.000817	0.005758	0.003857
**GO:0090109**	BP	GO:0090109	regulation of cell-substrate junction assembly	9/1133	64/29008	0.000817	0.005758	0.003857
**GO:0033144**	BP	GO:0033144	negative regulation of intracellular steroid hormone receptor signaling pathway	7/1133	40/29008	0.000820	0.005758	0.003857
**GO:0090224**	BP	GO:0090224	regulation of spindle organization	7/1133	40/29008	0.000820	0.005758	0.003857
**GO:1902749**	BP	GO:1902749	regulation of cell cycle G2/M phase transition	12/1133	105/29008	0.000820	0.005758	0.003857
**GO:0033135**	BP	GO:0033135	regulation of peptidyl-serine phosphorylation	16/1133	166/29008	0.000826	0.005790	0.003878
**GO:1903578**	BP	GO:1903578	regulation of ATP metabolic process	11/1133	91/29008	0.000835	0.005848	0.003917
**GO:0032609**	BP	GO:0032609	interferon-gamma production	14/1133	135/29008	0.000838	0.005859	0.003924
**GO:0033500**	BP	GO:0033500	carbohydrate homeostasis	23/1133	284/29008	0.000841	0.005871	0.003932
**GO:0001776**	BP	GO:0001776	leukocyte homeostasis	13/1133	120/29008	0.000843	0.005880	0.003938
**GO:0002643**	BP	GO:0002643	regulation of tolerance induction	5/1133	20/29008	0.000855	0.005907	0.003956
**GO:0003159**	BP	GO:0003159	morphogenesis of an endothelium	5/1133	20/29008	0.000855	0.005907	0.003956
**GO:0032695**	BP	GO:0032695	negative regulation of interleukin-12 production	5/1133	20/29008	0.000855	0.005907	0.003956
**GO:0033962**	BP	GO:0033962	P-body assembly	5/1133	20/29008	0.000855	0.005907	0.003956
**GO:0045623**	BP	GO:0045623	negative regulation of T-helper cell differentiation	5/1133	20/29008	0.000855	0.005907	0.003956
**GO:0045722**	BP	GO:0045722	positive regulation of gluconeogenesis	5/1133	20/29008	0.000855	0.005907	0.003956
**GO:0061154**	BP	GO:0061154	endothelial tube morphogenesis	5/1133	20/29008	0.000855	0.005907	0.003956
**GO:0006023**	BP	GO:0006023	aminoglycan biosynthetic process	8/1133	52/29008	0.000862	0.005936	0.003976
**GO:0045581**	BP	GO:0045581	negative regulation of T cell differentiation	8/1133	52/29008	0.000862	0.005936	0.003976
**GO:2000059**	BP	GO:2000059	negative regulation of ubiquitin-dependent protein catabolic process	8/1133	52/29008	0.000862	0.005936	0.003976
**GO:0016055**	BP	GO:0016055	Wnt signaling pathway	32/1133	449/29008	0.000866	0.005951	0.003986
**GO:0007051**	BP	GO:0007051	spindle organization	17/1133	183/29008	0.000875	0.006004	0.004022
**GO:0061418**	BP	GO:0061418	regulation of transcription from RNA polymerase II promoter in response to hypoxia	4/1133	12/29008	0.000890	0.006105	0.004089
**GO:0016575**	BP	GO:0016575	histone deacetylation	10/1133	78/29008	0.000897	0.006135	0.004109
**GO:0071277**	BP	GO:0071277	cellular response to calcium ion	10/1133	78/29008	0.000897	0.006135	0.004109
**GO:0010821**	BP	GO:0010821	regulation of mitochondrion organization	14/1133	136/29008	0.000901	0.006156	0.004123
**GO:0006611**	BP	GO:0006611	protein export from nucleus	9/1133	65/29008	0.000916	0.006250	0.004186
**GO:0051924**	BP	GO:0051924	regulation of calcium ion transport	23/1133	286/29008	0.000924	0.006291	0.004214
**GO:0032271**	BP	GO:0032271	regulation of protein polymerization	20/1133	234/29008	0.000926	0.006291	0.004214
**GO:0000470**	BP	GO:0000470	maturation of LSU-rRNA	6/1133	30/29008	0.000929	0.006291	0.004214
**GO:1902751**	BP	GO:1902751	positive regulation of cell cycle G2/M phase transition	6/1133	30/29008	0.000929	0.006291	0.004214
**GO:1902914**	BP	GO:1902914	regulation of protein polyubiquitination	6/1133	30/29008	0.000929	0.006291	0.004214
**GO:0048659**	BP	GO:0048659	smooth muscle cell proliferation	17/1133	184/29008	0.000929	0.006291	0.004214
**GO:0198738**	BP	GO:0198738	cell-cell signaling by wnt	32/1133	451/29008	0.000931	0.006297	0.004218
**GO:0043368**	BP	GO:0043368	positive T cell selection	7/1133	41/29008	0.000956	0.006445	0.004317
**GO:0046627**	BP	GO:0046627	negative regulation of insulin receptor signaling pathway	7/1133	41/29008	0.000956	0.006445	0.004317
**GO:0006874**	BP	GO:0006874	cellular calcium ion homeostasis	34/1133	490/29008	0.000964	0.006494	0.004350
**GO:0033555**	BP	GO:0033555	multicellular organismal response to stress	12/1133	107/29008	0.000971	0.006528	0.004373
**GO:0040013**	BP	GO:0040013	negative regulation of locomotion	26/1133	341/29008	0.000979	0.006577	0.004405
**GO:0043370**	BP	GO:0043370	regulation of CD4-positive, alpha-beta T cell differentiation	8/1133	53/29008	0.000982	0.006585	0.004411
**GO:2000146**	BP	GO:2000146	negative regulation of cell motility	24/1133	306/29008	0.001025	0.006859	0.004594
**GO:1903051**	BP	GO:1903051	negative regulation of proteolysis involved in cellular protein catabolic process	9/1133	66/29008	0.001025	0.006859	0.004594
**GO:0002285**	BP	GO:0002285	lymphocyte activation involved in immune response	19/1133	219/29008	0.001029	0.006876	0.004605
**GO:0009749**	BP	GO:0009749	response to glucose	17/1133	186/29008	0.001048	0.006994	0.004685
**GO:0007006**	BP	GO:0007006	mitochondrial membrane organization	12/1133	108/29008	0.001054	0.007017	0.004700
**GO:0071901**	BP	GO:0071901	negative regulation of protein serine/threonine kinase activity	12/1133	108/29008	0.001054	0.007017	0.004700
**GO:0051251**	BP	GO:0051251	positive regulation of lymphocyte activation	31/1133	436/29008	0.001074	0.007140	0.004782
**GO:0046777**	BP	GO:0046777	protein autophosphorylation	20/1133	237/29008	0.001083	0.007179	0.004809
**GO:0035264**	BP	GO:0035264	multicellular organism growth	19/1133	220/29008	0.001086	0.007179	0.004809
**GO:0032516**	BP	GO:0032516	positive regulation of phosphoprotein phosphatase activity	5/1133	21/29008	0.001086	0.007179	0.004809
**GO:0034724**	BP	GO:0034724	DNA replication-independent nucleosome organization	5/1133	21/29008	0.001086	0.007179	0.004809
**GO:0035994**	BP	GO:0035994	response to muscle stretch	5/1133	21/29008	0.001086	0.007179	0.004809
**GO:0032204**	BP	GO:0032204	regulation of telomere maintenance	10/1133	80/29008	0.001095	0.007225	0.004839
**GO:0007616**	BP	GO:0007616	long-term memory	7/1133	42/29008	0.001109	0.007292	0.004884
**GO:0072595**	BP	GO:0072595	maintenance of protein localization in organelle	7/1133	42/29008	0.001109	0.007292	0.004884
**GO:0002823**	BP	GO:0002823	negative regulation of adaptive immune response based on somatic recombination o	8/1133	54/29008	0.001113	0.007292	0.004884
**GO:0045773**	BP	GO:0045773	positive regulation of axon extension	8/1133	54/29008	0.001113	0.007292	0.004884
**GO:0090311**	BP	GO:0090311	regulation of protein deacetylation	8/1133	54/29008	0.001113	0.007292	0.004884
**GO:0050858**	BP	GO:0050858	negative regulation of antigen receptor-mediated signaling pathway	6/1133	31/29008	0.001114	0.007292	0.004884
**GO:1902186**	BP	GO:1902186	regulation of viral release from host cell	6/1133	31/29008	0.001114	0.007292	0.004884
**GO:0051224**	BP	GO:0051224	negative regulation of protein transport	14/1133	139/29008	0.001116	0.007293	0.004885
**GO:0032259**	BP	GO:0032259	methylation	27/1133	363/29008	0.001142	0.007438	0.004982
**GO:0010595**	BP	GO:0010595	positive regulation of endothelial cell migration	12/1133	109/29008	0.001143	0.007438	0.004982
**GO:0034446**	BP	GO:0034446	substrate adhesion-dependent cell spreading	12/1133	109/29008	0.001143	0.007438	0.004982
**GO:0061951**	BP	GO:0061951	establishment of protein localization to plasma membrane	9/1133	67/29008	0.001144	0.007438	0.004982
**GO:0000045**	BP	GO:0000045	autophagosome assembly	11/1133	95/29008	0.001197	0.007777	0.005209
**GO:2001020**	BP	GO:2001020	regulation of response to DNA damage stimulus	19/1133	222/29008	0.001208	0.007837	0.005249
**GO:0010038**	BP	GO:0010038	response to metal ion	23/1133	292/29008	0.001219	0.007900	0.005291
**GO:0002702**	BP	GO:0002702	positive regulation of production of molecular mediator of immune response	13/1133	125/29008	0.001234	0.007974	0.005341
**GO:0045931**	BP	GO:0045931	positive regulation of mitotic cell cycle	13/1133	125/29008	0.001234	0.007974	0.005341
**GO:0001731**	BP	GO:0001731	formation of translation preinitiation complex	4/1133	13/29008	0.001247	0.008001	0.005359
**GO:0048385**	BP	GO:0048385	regulation of retinoic acid receptor signaling pathway	4/1133	13/29008	0.001247	0.008001	0.005359
**GO:0060368**	BP	GO:0060368	regulation of Fc receptor mediated stimulatory signaling pathway	4/1133	13/29008	0.001247	0.008001	0.005359
**GO:0090085**	BP	GO:0090085	regulation of protein deubiquitination	4/1133	13/29008	0.001247	0.008001	0.005359
**GO:1902931**	BP	GO:1902931	negative regulation of alcohol biosynthetic process	4/1133	13/29008	0.001247	0.008001	0.005359
**GO:1905214**	BP	GO:1905214	regulation of RNA binding	4/1133	13/29008	0.001247	0.008001	0.005359
**GO:0009746**	BP	GO:0009746	response to hexose	17/1133	189/29008	0.001249	0.008007	0.005363
**GO:0007062**	BP	GO:0007062	sister chromatid cohesion	8/1133	55/29008	0.001259	0.008060	0.005399

**GO:0032608**	BP	GO:0032608	interferon-beta production	9/1133	68/29008	0.0012736	0.0081314	0.0054465
**GO:0032648**	BP	GO:0032648	regulation of interferon-beta production	9/1133	68/29008	0.0012736	0.0081314	0.0054465
**GO:0001824**	BP	GO:0001824	blastocyst development	15/1133	157/29008	0.0012956	0.0082417	0.0055204
**GO:0008360**	BP	GO:0008360	regulation of cell shape	15/1133	157/29008	0.0012956	0.0082417	0.0055204
**GO:0071322**	BP	GO:0071322	cellular response to carbohydrate stimulus	15/1133	157/29008	0.0012956	0.0082417	0.0055204
**GO:0007044**	BP	GO:0007044	cell-substrate junction assembly	11/1133	96/29008	0.0013058	0.0082971	0.0055576
**GO:0034284**	BP	GO:0034284	response to monosaccharide	17/1133	190/29008	0.0013238	0.0083667	0.0056042
**GO:0002260**	BP	GO:0002260	lymphocyte homeostasis	10/1133	82/29008	0.0013266	0.0083667	0.0056042
**GO:0072332**	BP	GO:0072332	intrinsic apoptotic signaling pathway by p53 class mediator	10/1133	82/29008	0.0013266	0.0083667	0.0056042
**GO:0006482**	BP	GO:0006482	protein demethylation	6/1133	32/29008	0.0013273	0.0083667	0.0056042
**GO:0008214**	BP	GO:0008214	protein dealkylation	6/1133	32/29008	0.0013273	0.0083667	0.0056042
**GO:0022037**	BP	GO:0022037	metencephalon development	13/1133	126/29008	0.0013278	0.0083667	0.0056042
**GO:0043123**	BP	GO:0043123	positive regulation of I-kappaB kinase/NF-kappaB signaling	13/1133	126/29008	0.0013278	0.0083667	0.0056042
**GO:0060326**	BP	GO:0060326	cell chemotaxis	24/1133	312/29008	0.0013349	0.0084014	0.0056274
**GO:0002367**	BP	GO:0002367	cytokine production involved in immune response	12/1133	111/29008	0.0013407	0.0084177	0.0056383
**GO:0071482**	BP	GO:0071482	cellular response to light stimulus	12/1133	111/29008	0.0013407	0.0084177	0.0056383
**GO:0007035**	BP	GO:0007035	vacuolar acidification	5/1133	22/29008	0.0013615	0.0085387	0.0057193
**GO:0006261**	BP	GO:0006261	DNA-dependent DNA replication	14/1133	142/29008	0.0013722	0.0085852	0.0057505
**GO:0048675**	BP	GO:0048675	axon extension	14/1133	142/29008	0.0013722	0.0085852	0.0057505
**GO:0016482**	BP	GO:0016482	cytosolic transport	15/1133	158/29008	0.0013810	0.0086300	0.0057806
**GO:0030041**	BP	GO:0030041	actin filament polymerization	17/1133	191/29008	0.0014016	0.0087485	0.0058599
**GO:0002706**	BP	GO:0002706	regulation of lymphocyte mediated immunity	19/1133	225/29008	0.0014137	0.0088002	0.0058945
**GO:0009206**	BP	GO:0009206	purine ribonucleoside triphosphate biosynthetic process	9/1133	69/29008	0.0014148	0.0088002	0.0058945
**GO:0032507**	BP	GO:0032507	maintenance of protein location in cell	9/1133	69/29008	0.0014148	0.0088002	0.0058945
**GO:0002209**	BP	GO:0002209	behavioral defense response	8/1133	56/29008	0.0014200	0.0088015	0.0058954
**GO:0034340**	BP	GO:0034340	response to type I interferon	8/1133	56/29008	0.0014200	0.0088015	0.0058954
**GO:1901861**	BP	GO:1901861	regulation of muscle tissue development	8/1133	56/29008	0.0014200	0.0088015	0.0058954
**GO:0010389**	BP	GO:0010389	regulation of G2/M transition of mitotic cell cycle	11/1133	97/29008	0.0014219	0.0088032	0.0058966
**GO:0001906**	BP	GO:0001906	cell killing	21/1133	260/29008	0.0014302	0.0088439	0.0059238
**GO:0140014**	BP	GO:0140014	mitotic nuclear division	22/1133	278/29008	0.0014507	0.0089605	0.0060019
**GO:0031397**	BP	GO:0031397	negative regulation of protein ubiquitination	10/1133	83/29008	0.0014565	0.0089860	0.0060190
**GO:0060348**	BP	GO:0060348	bone development	20/1133	243/29008	0.0014654	0.0090302	0.0060486
**GO:0046328**	BP	GO:0046328	regulation of JNK cascade	14/1133	143/29008	0.0014677	0.0090338	0.0060510
**GO:0030219**	BP	GO:0030219	megakaryocyte differentiation	7/1133	44/29008	0.0014725	0.0090426	0.0060569
**GO:0072599**	BP	GO:0072599	establishment of protein localization to endoplasmic reticulum	7/1133	44/29008	0.0014725	0.0090426	0.0060569
**GO:0010810**	BP	GO:0010810	regulation of cell-substrate adhesion	19/1133	226/29008	0.0014883	0.0091190	0.0061081
**GO:0030595**	BP	GO:0030595	leukocyte chemotaxis	19/1133	226/29008	0.0014883	0.0091190	0.0061081
**GO:0110053**	BP	GO:0110053	regulation of actin filament organization	22/1133	279/29008	0.0015183	0.0092920	0.0062240
**GO:0001952**	BP	GO:0001952	regulation of cell-matrix adhesion	13/1133	128/29008	0.0015333	0.0093726	0.0062779
**GO:0030516**	BP	GO:0030516	regulation of axon extension	12/1133	113/29008	0.0015651	0.0095315	0.0063844
**GO:0009145**	BP	GO:0009145	purine nucleoside triphosphate biosynthetic process	9/1133	70/29008	0.0015683	0.0095315	0.0063844
**GO:0090316**	BP	GO:0090316	positive regulation of intracellular protein transport	14/1133	144/29008	0.0015686	0.0095315	0.0063844
**GO:0032786**	BP	GO:0032786	positive regulation of DNA-templated transcription, elongation	6/1133	33/29008	0.0015693	0.0095315	0.0063844
**GO:0150117**	BP	GO:0150117	positive regulation of cell-substrate junction organization	6/1133	33/29008	0.0015693	0.0095315	0.0063844
**GO:0007568**	BP	GO:0007568	aging	21/1133	262/29008	0.0015718	0.0095315	0.0063844
**GO:0032886**	BP	GO:0032886	regulation of microtubule-based process	21/1133	262/29008	0.0015718	0.0095315	0.0063844
**GO:0050769**	BP	GO:0050769	positive regulation of neurogenesis	23/1133	298/29008	0.0015919	0.0096422	0.0064585
**GO:0031529**	BP	GO:0031529	ruffle organization	8/1133	57/29008	0.0015962	0.0096487	0.0064629
**GO:0006809**	BP	GO:0006809	nitric oxide biosynthetic process	10/1133	84/29008	0.0015966	0.0096487	0.0064629
**GO:0042157**	BP	GO:0042157	lipoprotein metabolic process	13/1133	129/29008	0.0016455	0.0099329	0.0066533
**GO:0030111**	BP	GO:0030111	regulation of Wnt signaling pathway	24/1133	317/29008	0.0016515	0.0099467	0.0066625
**GO:0051222**	BP	GO:0051222	positive regulation of protein transport	24/1133	317/29008	0.0016515	0.0099467	0.0066625
**GO:0022626**	CC	GO:0022626	cytosolic ribosome	46/1136	114/28886	1.34E-34	8.24E-32	5.16E-32
**GO:0005840**	CC	GO:0005840	ribosome	57/1136	239/28886	1.36E-28	3.62E-26	2.27E-26
**GO:0044391**	CC	GO:0044391	ribosomal subunit	52/1136	197/28886	1.77E-28	3.62E-26	2.27E-26
**GO:0022627**	CC	GO:0022627	cytosolic small ribosomal subunit	23/1136	47/28886	2.51E-20	3.86E-18	2.42E-18
**GO:0015935**	CC	GO:0015935	small ribosomal subunit	28/1136	80/28886	1.33E-19	1.63E-17	1.02E-17
**GO:0022625**	CC	GO:0022625	cytosolic large ribosomal subunit	24/1136	63/28886	5.34E-18	5.47E-16	3.43E-16
**GO:0005844**	CC	GO:0005844	polysome	24/1136	77/28886	1.1E-15	9.68E-14	6.06E-14
**GO:0016607**	CC	GO:0016607	nuclear speck	50/1136	343/28886	1.34E-15	1.03E-13	6.47E-14
**GO:0035770**	CC	GO:0035770	ribonucleoprotein granule	40/1136	250/28886	4.2E-14	2.87E-12	1.8E-12
**GO:0042788**	CC	GO:0042788	polysomal ribosome	16/1136	35/28886	5.92E-14	3.64E-12	2.28E-12
**GO:0036464**	CC	GO:0036464	cytoplasmic ribonucleoprotein granule	38/1136	232/28886	8.48E-14	4.74E-12	2.97E-12
**GO:0015934**	CC	GO:0015934	large ribosomal subunit	27/1136	122/28886	2.06E-13	1.06E-11	6.61E-12
**GO:0005635**	CC	GO:0005635	nuclear envelope	49/1136	423/28886	1.66E-11	7.86E-10	4.92E-10
**GO:0031248**	CC	GO:0031248	protein acetyltransferase complex	20/1136	90/28886	2.47E-10	1.01E-08	6.35E-09
**GO:1902493**	CC	GO:1902493	acetyltransferase complex	20/1136	90/28886	2.47E-10	1.01E-08	6.35E-09
**GO:0043209**	CC	GO:0043209	myelin sheath	30/1136	215/28886	1.88E-09	7.23E-08	4.53E-08
**GO:0005667**	CC	GO:0005667	transcription regulator complex	48/1136	475/28886	2.76E-09	9.99E-08	6.26E-08
**GO:0010494**	CC	GO:0010494	cytoplasmic stress granule	17/1136	74/28886	3.16E-09	1.08E-07	6.76E-08
**GO:0005774**	CC	GO:0005774	vacuolar membrane	30/1136	228/28886	7.61E-09	2.46E-07	1.54E-07
**GO:0034399**	CC	GO:0034399	nuclear periphery	20/1136	115/28886	2.22E-08	6.83E-07	4.28E-07
**GO:0031965**	CC	GO:0031965	nuclear membrane	30/1136	241/28886	2.76E-08	8.07E-07	5.06E-07
**GO:0016605**	CC	GO:0016605	PML body	16/1136	76/28886	3.46E-08	9.68E-07	6.06E-07
**GO:0030139**	CC	GO:0030139	endocytic vesicle	27/1136	205/28886	4.14E-08	1.11E-06	6.93E-07
**GO:0031300**	CC	GO:0031300	intrinsic component of organelle membrane	37/1136	366/28886	1.77E-07	4.54E-06	2.84E-06
**GO:0061695**	CC	GO:0061695	transferase complex, transferring phosphorus-containing groups	28/1136	242/28886	3.68E-07	8.81E-06	5.52E-06
**GO:0031301**	CC	GO:0031301	integral component of organelle membrane	35/1136	346/28886	3.72E-07	8.81E-06	5.52E-06
**GO:0005776**	CC	GO:0005776	autophagosome	14/1136	75/28886	1.17E-06	2.66E-05	1.66E-05
**GO:0031414**	CC	GO:0031414	N-terminal protein acetyltransferase complex	6/1136	11/28886	1.42E-06	3.13E-05	1.96E-05
**GO:0016363**	CC	GO:0016363	nuclear matrix	15/1136	89/28886	1.89E-06	4.02E-05	2.52E-05
**GO:0045335**	CC	GO:0045335	phagocytic vesicle	17/1136	113/28886	2.06E-06	4.22E-05	2.64E-05
**GO:0005681**	CC	GO:0005681	spliceosomal complex	24/1136	206/28886	2.18E-06	4.3E-05	2.69E-05
**GO:0000123**	CC	GO:0000123	histone acetyltransferase complex	14/1136	79/28886	2.23E-06	4.3E-05	2.69E-05
**GO:0098562**	CC	GO:0098562	cytoplasmic side of membrane	23/1136	194/28886	2.61E-06	4.86E-05	3.05E-05
**GO:0001650**	CC	GO:0001650	fibrillar center	19/1136	142/28886	3.21E-06	5.8E-05	3.63E-05
**GO:0000932**	CC	GO:0000932	P-body	14/1136	83/28886	4.1E-06	7.21E-05	4.52E-05
**GO:0005684**	CC	GO:0005684	U2-type spliceosomal complex	15/1136	95/28886	4.4E-06	7.51E-05	4.7E-05
**GO:0008023**	CC	GO:0008023	transcription elongation factor complex	11/1136	53/28886	5.54E-06	9.21E-05	5.77E-05
**GO:0070603**	CC	GO:0070603	SWI/SNF superfamily-type complex	13/1136	75/28886	6.6E-06	0.0001068	6.69E-05
**GO:1904949**	CC	GO:1904949	ATPase complex	13/1136	76/28886	7.68E-06	0.0001211	7.59E-05
**GO:0019866**	CC	GO:0019866	organelle inner membrane	41/1136	500/28886	8.81E-06	0.0001354	8.49E-05
**GO:0034708**	CC	GO:0034708	methyltransferase complex	15/1136	101/28886	9.51E-06	0.0001417	8.88E-05
**GO:0001726**	CC	GO:0001726	ruffle	19/1136	153/28886	9.68E-06	0.0001417	8.88E-05
**GO:0032279**	CC	GO:0032279	asymmetric synapse	31/1136	337/28886	1.19E-05	0.0001643	0.0001029
**GO:0016234**	CC	GO:0016234	inclusion body	13/1136	79/28886	1.19E-05	0.0001643	0.0001029
**GO:0005769**	CC	GO:0005769	early endosome	30/1136	321/28886	1.2E-05	0.0001643	0.0001029
**GO:0030684**	CC	GO:0030684	preribosome	13/1136	80/28886	1.37E-05	0.0001832	0.0001147
**GO:0140534**	CC	GO:0140534	endoplasmic reticulum protein-containing complex	17/1136	130/28886	1.42E-05	0.0001853	0.0001161
**GO:0090575**	CC	GO:0090575	RNA polymerase II transcription regulator complex	20/1136	172/28886	1.54E-05	0.0001966	0.0001232
**GO:0014069**	CC	GO:0014069	postsynaptic density	30/1136	328/28886	1.82E-05	0.0002286	0.0001432
**GO:0071013**	CC	GO:0071013	catalytic step 2 spliceosome	14/1136	95/28886	2.05E-05	0.0002518	0.0001577
**GO:0000139**	CC	GO:0000139	Golgi membrane	26/1136	267/28886	2.21E-05	0.0002670	0.0001673
**GO:0009898**	CC	GO:0009898	cytoplasmic side of plasma membrane	20/1136	177/28886	2.34E-05	0.0002731	0.0001710
**GO:1902554**	CC	GO:1902554	serine/threonine protein kinase complex	13/1136	84/28886	2.35E-05	0.0002731	0.0001710
**GO:0099572**	CC	GO:0099572	postsynaptic specialization	31/1136	351/28886	2.63E-05	0.0002996	0.0001877
**GO:0016514**	CC	GO:0016514	SWI/SNF complex	6/1136	17/28886	3.11E-05	0.0003478	0.0002178
**GO:0005770**	CC	GO:0005770	late endosome	24/1136	242/28886	3.36E-05	0.0003691	0.0002312
**GO:0030496**	CC	GO:0030496	midbody	20/1136	183/28886	3.79E-05	0.0004091	0.0002563
**GO:0098984**	CC	GO:0098984	neuron to neuron synapse	31/1136	359/28886	4.05E-05	0.0004289	0.0002686
**GO:1902911**	CC	GO:1902911	protein kinase complex	14/1136	101/28886	4.13E-05	0.0004305	0.0002697
**GO:0071011**	CC	GO:0071011	precatalytic spliceosome	10/1136	54/28886	4.21E-05	0.0004313	0.0002702
**GO:0005765**	CC	GO:0005765	lysosomal membrane	19/1136	171/28886	4.68E-05	0.0004642	0.0002908
**GO:0098852**	CC	GO:0098852	lytic vacuole membrane	19/1136	171/28886	4.68E-05	0.0004642	0.0002908
**GO:0030532**	CC	GO:0030532	small nuclear ribonucleoprotein complex	11/1136	66/28886	4.89E-05	0.0004777	0.0002993
**GO:0005643**	CC	GO:0005643	nuclear pore	11/1136	67/28886	5.65E-05	0.0005429	0.0003400
**GO:0097525**	CC	GO:0097525	spliceosomal snRNP complex	10/1136	58/28886	7.96E-05	0.0007530	0.0004717
**GO:0005874**	CC	GO:0005874	microtubule	34/1136	427/28886	8.91E-05	0.0008297	0.0005198
**GO:0044754**	CC	GO:0044754	autolysosome	5/1136	13/28886	9.21E-05	0.0008458	0.0005298
**GO:0032587**	CC	GO:0032587	ruffle membrane	11/1136	73/28886	0.0001262	0.0011249	0.0007047
**GO:0035097**	CC	GO:0035097	histone methyltransferase complex	11/1136	73/28886	0.0001262	0.0011249	0.0007047
**GO:0005743**	CC	GO:0005743	mitochondrial inner membrane	35/1136	456/28886	0.0001450	0.0012743	0.0007983
**GO:0000792**	CC	GO:0000792	heterochromatin	13/1136	101/28886	0.0001658	0.0014369	0.0009001
**GO:0071005**	CC	GO:0071005	U2-type precatalytic spliceosome	9/1136	52/28886	0.0001746	0.0014918	0.0009345
**GO:0005938**	CC	GO:0005938	cell cortex	26/1136	304/28886	0.0001906	0.0016058	0.0010059
**GO:0031227**	CC	GO:0031227	intrinsic component of endoplasmic reticulum membrane	17/1136	160/28886	0.0001987	0.0016310	0.0010217
**GO:0071565**	CC	GO:0071565	nBAF complex	5/1136	15/28886	0.0002013	0.0016310	0.0010217
**GO:0045121**	CC	GO:0045121	membrane raft	31/1136	392/28886	0.0002015	0.0016310	0.0010217
**GO:0098857**	CC	GO:0098857	membrane microdomain	31/1136	393/28886	0.0002107	0.0016746	0.0010491
**GO:0031252**	CC	GO:0031252	cell leading edge	32/1136	411/28886	0.0002124	0.0016746	0.0010491
**GO:0098791**	CC	GO:0098791	Golgi apparatus subcompartment	26/1136	307/28886	0.0002225	0.0017322	0.0010851
**GO:0120114**	CC	GO:0120114	Sm-like protein family complex	11/1136	78/28886	0.0002307	0.0017741	0.0011113
**GO:0000307**	CC	GO:0000307	cyclin-dependent protein kinase holoenzyme complex	8/1136	43/28886	0.0002374	0.0018031	0.0011295
**GO:1902562**	CC	GO:1902562	H4 histone acetyltransferase complex	7/1136	33/28886	0.0002486	0.0018646	0.0011680
**GO:0098798**	CC	GO:0098798	mitochondrial protein-containing complex	23/1136	263/28886	0.0003174	0.0023521	0.0014734
**GO:0005819**	CC	GO:0005819	spindle	27/1136	332/28886	0.0003257	0.0023847	0.0014939
**GO:0030176**	CC	GO:0030176	integral component of endoplasmic reticulum membrane	16/1136	152/28886	0.0003362	0.0024299	0.0015222
**GO:0030687**	CC	GO:0030687	preribosome, large subunit precursor	6/1136	25/28886	0.0003397	0.0024299	0.0015222
**GO:0030135**	CC	GO:0030135	coated vesicle	19/1136	200/28886	0.0003686	0.0026060	0.0016325

GO:0000791	CC	GO:0000791	euchromatin	8/1136	46/28886	0.0003850	0.0026842	0.0016815
GO:0005767	CC	GO:0005767	secondary lysosome	5/1136	17/28886	0.0003884	0.0026842	0.0016815
GO:0005801	CC	GO:0005801	cis-Golgi network	10/1136	73/28886	0.0005569	0.0038057	0.0023840
GO:0008287	CC	GO:0008287	protein serine/threonine phosphatase complex	8/1136	49/28886	0.0005997	0.0040092	0.0025115
GO:1903293	CC	GO:1903293	phosphatase complex	8/1136	49/28886	0.0005997	0.0040092	0.0025115
GO:0042611	CC	GO:0042611	MHC protein complex	7/1136	38/28886	0.0006189	0.0040928	0.0025639
GO:0072686	CC	GO:0072686	mitotic spindle	16/1136	161/28886	0.0006375	0.0041712	0.0026130
GO:1905368	CC	GO:1905368	peptidase complex	12/1136	102/28886	0.0006711	0.0043344	0.0027153
GO:0032045	CC	GO:0032045	guanyl-nucleotide exchange factor complex	5/1136	19/28886	0.0006836	0.0043344	0.0027153
GO:1902773	CC	GO:1902773	GTPase activator complex	5/1136	19/28886	0.0006836	0.0043344	0.0027153
GO:0032993	CC	GO:0032993	protein-DNA complex	16/1136	163/28886	0.0007293	0.0045772	0.0028673
GO:0005759	CC	GO:0005759	mitochondrial matrix	23/1136	280/28886	0.0007603	0.0047236	0.0029591
GO:0033106	CC	GO:0033106	cis-Golgi network membrane	6/1136	29/28886	0.0007968	0.0049004	0.0030698
GO:0000803	CC	GO:0000803	sex chromosome	7/1136	40/28886	0.0008539	0.0051752	0.0032420
GO:0000502	CC	GO:0000502	proteasome complex	9/1136	64/28886	0.0008583	0.0051752	0.0032420
GO:0071564	CC	GO:0071564	npBAF complex	4/1136	12/28886	0.0009141	0.0054581	0.0034192
GO:0032806	CC	GO:0032806	carboxy-terminal domain protein kinase complex	5/1136	21/28886	0.0011206	0.0066269	0.0041514
GO:0031228	CC	GO:0031228	intrinsic component of Golgi membrane	8/1136	54/28886	0.0011639	0.0068171	0.0042705
GO:0005657	CC	GO:0005657	replication fork	9/1136	67/28886	0.0011999	0.0069621	0.0043614
GO:0019908	CC	GO:0019908	nuclear cyclin-dependent protein kinase holoenzyme complex	4/1136	13/28886	0.0012794	0.0073082	0.0045782
GO:0019898	CC	GO:0019898	extrinsic component of membrane	24/1136	309/28886	0.0012833	0.0073082	0.0045782
GO:0030134	CC	GO:0030134	COPII-coated ER to Golgi transport vesicle	8/1136	55/28886	0.0013157	0.0074239	0.0046506
GO:0001741	CC	GO:0001741	XY body	5/1136	22/28886	0.0014036	0.0077771	0.0048719
GO:0035145	CC	GO:0035145	exon-exon junction complex	5/1136	22/28886	0.0014036	0.0077771	0.0048719
GO:0031256	CC	GO:0031256	leading edge membrane	15/1136	158/28886	0.0014766	0.0081086	0.0050795
GO:1905369	CC	GO:1905369	endopeptidase complex	9/1136	70/28886	0.0016437	0.0089461	0.0056042
GO:0030662	CC	GO:0030662	coated vesicle membrane	10/1136	84/28886	0.0016793	0.0090598	0.0056754
GO:0150034	CC	GO:0150034	distal axon	27/1136	371/28886	0.0017249	0.0092024	0.0057648
GO:0005652	CC	GO:0005652	nuclear lamina	4/1136	14/28886	0.0017357	0.0092024	0.0057648
GO:0003735	MF	GO:0003735	structural constituent of ribosome	52/1102	166/28438	7.76E-33	6.66E-30	4.79E-30
GO:0044389	MF	GO:0044389	ubiquitin-like protein ligase binding	65/1102	330/28438	1.13E-27	4.84E-25	3.48E-25
GO:0031625	MF	GO:0031625	ubiquitin protein ligase binding	62/1102	313/28438	1.37E-26	3.92E-24	2.82E-24
GO:0140297	MF	GO:0140297	DNA-binding transcription factor binding	67/1102	431/28438	2.19E-22	4.7E-20	3.38E-20
GO:0003729	MF	GO:0003729	mRNA binding	59/1102	366/28438	1.2E-20	2.06E-18	1.48E-18
GO:0061629	MF	GO:0061629	RNA polymerase II-specific DNA-binding transcription factor binding	55/1102	329/28438	4.39E-20	6.28E-18	4.52E-18
GO:0003712	MF	GO:0003712	transcription coregulator activity	61/1102	480/28438	3.77E-16	4.62E-14	3.32E-14
GO:0030695	MF	GO:0030695	GTPase regulator activity	52/1102	421/28438	1.84E-13	1.75E-11	1.26E-11
GO:0060589	MF	GO:0060589	nucleoside-triphosphatase regulator activity	52/1102	421/28438	1.84E-13	1.75E-11	1.26E-11
GO:0051082	MF	GO:0051082	unfolded protein binding	23/1102	88/28438	2.13E-13	1.75E-11	1.26E-11
GO:0019787	MF	GO:0019787	ubiquitin-like protein transferase activity	53/1102	437/28438	2.24E-13	1.75E-11	1.26E-11
GO:0004842	MF	GO:0004842	ubiquitin-protein transferase activity	51/1102	415/28438	3.83E-13	2.74E-11	1.97E-11
GO:0005096	MF	GO:0005096	GTPase activator activity	50/1102	405/28438	5.48E-13	3.62E-11	2.6E-11
GO:0042393	MF	GO:0042393	histone binding	40/1102	287/28438	2.55E-12	1.56E-10	1.12E-10
GO:0016922	MF	GO:0016922	nuclear receptor binding	28/1102	155/28438	9.91E-12	5.67E-10	4.07E-10
GO:0005085	MF	GO:0005085	guanyl-nucleotide exchange factor activity	31/1102	197/28438	3.23E-11	1.73E-09	1.25E-09
GO:0031072	MF	GO:0031072	heat shock protein binding	26/1102	147/28438	8.82E-11	4.45E-09	3.2E-09
GO:0051020	MF	GO:0051020	GTPase binding	39/1102	313/28438	1.5E-10	7.13E-09	5.13E-09
GO:0003713	MF	GO:0003713	transcription coactivator activity	34/1102	253/28438	2.99E-10	1.35E-08	9.72E-09
GO:0003730	MF	GO:0003730	mRNA 3'-UTR binding	21/1102	106/28438	6.5E-10	2.79E-08	2E-08
GO:0061659	MF	GO:0061659	ubiquitin-like protein ligase activity	38/1102	321/28438	1.12E-09	4.59E-08	3.3E-08
GO:0061630	MF	GO:0061630	ubiquitin protein ligase activity	37/1102	310/28438	1.48E-09	5.76E-08	4.14E-08
GO:0031267	MF	GO:0031267	small GTPase binding	34/1102	277/28438	3.3E-09	1.23E-07	8.85E-08
GO:0008022	MF	GO:0008022	protein C-terminus binding	30/1102	232/28438	8.18E-09	2.92E-07	2.1E-07
GO:0140658	MF	GO:0140658	ATP-dependent chromatin remodeler activity	11/1102	31/28438	1.17E-08	4.01E-07	2.88E-07
GO:0043021	MF	GO:0043021	ribonucleoprotein complex binding	24/1102	168/28438	3.67E-08	1.21E-06	8.71E-07
GO:0047485	MF	GO:0047485	protein N-terminus binding	21/1102	133/28438	4.46E-08	1.42E-06	1.02E-06
GO:0140097	MF	GO:0140097	catalytic activity, acting on DNA	28/1102	223/28438	4.84E-08	1.48E-06	1.07E-06
GO:0001221	MF	GO:0001221	transcription coregulator binding	17/1102	90/28438	5.75E-08	1.7E-06	1.22E-06
GO:0002039	MF	GO:0002039	p53 binding	15/1102	76/28438	1.88E-07	5.38E-06	3.87E-06
GO:0051787	MF	GO:0051787	misfolded protein binding	9/1102	25/28438	2.22E-07	6.15E-06	4.42E-06
GO:0050681	MF	GO:0050681	androgen receptor binding	10/1102	33/28438	3.01E-07	8.06E-06	5.8E-06
GO:0005543	MF	GO:0005543	phospholipid binding	41/1102	457/28438	6.5E-07	1.69E-05	1.21E-05
GO:0004674	MF	GO:0004674	protein serine/threonine kinase activity	40/1102	441/28438	6.72E-07	1.7E-05	1.22E-05
GO:0046332	MF	GO:0046332	SMAD binding	15/1102	86/28438	1E-06	2.46E-05	1.77E-05
GO:0003714	MF	GO:0003714	transcription corepressor activity	23/1102	187/28438	1.07E-06	2.56E-05	1.84E-05
GO:0008094	MF	GO:0008094	ATP-dependent activity, acting on DNA	17/1102	110/28438	1.15E-06	2.66E-05	1.91E-05
GO:0044183	MF	GO:0044183	protein folding chaperone	10/1102	38/28438	1.29E-06	2.91E-05	2.09E-05
GO:0019207	MF	GO:0019207	kinase regulator activity	25/1102	229/28438	3.39E-06	7.45E-05	5.36E-05
GO:0106310	MF	GO:0106310	protein serine kinase activity	26/1102	246/28438	3.96E-06	8.38E-05	6.03E-05
GO:0003725	MF	GO:0003725	double-stranded RNA binding	14/1102	84/28438	4.01E-06	8.38E-05	6.03E-05
GO:0090079	MF	GO:0090079	translation regulator activity, nucleic acid binding	16/1102	108/28438	4.15E-06	8.47E-05	6.09E-05
GO:0051087	MF	GO:0051087	chaperone binding	16/1102	109/28438	4.69E-06	9.36E-05	6.73E-05
GO:0016410	MF	GO:0016410	N-acyltransferase activity	15/1102	99/28438	6.21E-06	0.0001210	8.7E-05
GO:0004386	MF	GO:0004386	helicase activity	19/1102	151/28438	6.48E-06	0.0001234	8.88E-05
GO:0051721	MF	GO:0051721	protein phosphatase 2A binding	9/1102	37/28438	8.9E-06	0.0001660	0.0001193
GO:0046982	MF	GO:0046982	protein heterodimerization activity	29/1102	307/28438	1.03E-05	0.0001888	0.0001357
GO:0019902	MF	GO:0019902	phosphatase binding	24/1102	230/28438	1.14E-05	0.0002040	0.0001466
GO:0048156	MF	GO:0048156	tau protein binding	7/1102	22/28438	1.32E-05	0.0002288	0.0001645
GO:0140030	MF	GO:0140030	modification-dependent protein binding	22/1102	202/28438	1.33E-05	0.0002288	0.0001645
GO:0032182	MF	GO:0032182	ubiquitin-like protein binding	15/1102	106/28438	1.45E-05	0.0002442	0.0001755
GO:0001046	MF	GO:0001046	core promoter sequence-specific DNA binding	10/1102	49/28438	1.52E-05	0.0002507	0.0001802
GO:0030331	MF	GO:0030331	estrogen receptor binding	10/1102	50/28438	1.83E-05	0.0002914	0.0002095
GO:0034212	MF	GO:0034212	peptide N-acetyltransferase activity	10/1102	50/28438	1.83E-05	0.0002914	0.0002095
GO:0003743	MF	GO:0003743	translation initiation factor activity	11/1102	61/28438	1.99E-05	0.0003046	0.0002190
GO:0070063	MF	GO:0070063	RNA polymerase binding	11/1102	61/28438	1.99E-05	0.0003046	0.0002190
GO:0019903	MF	GO:0019903	protein phosphatase binding	20/1102	182/28438	2.85E-05	0.0004145	0.0002980
GO:0032183	MF	GO:0032183	SUMO binding	6/1102	17/28438	2.86E-05	0.0004145	0.0002980
GO:0043422	MF	GO:0043422	protein kinase B binding	6/1102	17/28438	2.86E-05	0.0004145	0.0002980
GO:0051019	MF	GO:0051019	mitogen-activated protein kinase binding	8/1102	33/28438	2.9E-05	0.0004145	0.0002980
GO:0008080	MF	GO:0008080	N-acetyltransferase activity	12/1102	75/28438	2.96E-05	0.0004168	0.0002996
GO:1990782	MF	GO:1990782	protein tyrosine kinase binding	15/1102	113/28438	3.15E-05	0.0004358	0.0003133
GO:0045182	MF	GO:0045182	translation regulator activity	17/1102	141/28438	3.42E-05	0.0004653	0.0003345
GO:0008135	MF	GO:0008135	translation factor activity, RNA binding	13/1102	90/28438	4.27E-05	0.0005729	0.0004119
GO:0042826	MF	GO:0042826	histone deacetylase binding	16/1102	132/28438	5.39E-05	0.0007119	0.0005118
GO:0016887	MF	GO:0016887	ATP hydrolysis activity	22/1102	223/28438	6.12E-05	0.0007955	0.0005719
GO:0097718	MF	GO:0097718	disordered domain specific binding	8/1102	37/28438	7.03E-05	0.0008997	0.0006468
GO:0046966	MF	GO:0046966	thyroid hormone receptor binding	7/1102	28/28438	7.46E-05	0.0009406	0.0006762
GO:0001227	MF	GO:0001227	DNA-binding transcription repressor activity, RNA polymerase II-specific	28/1102	328/28438	8.86E-05	0.0011018	0.0007921
GO:0031490	MF	GO:0031490	chromatin DNA binding	13/1102	97/28438	9.45E-05	0.0011577	0.0008323
GO:0001217	MF	GO:0001217	DNA-binding transcription repressor activity	28/1102	330/28438	9.83E-05	0.0011882	0.0008542
GO:0035091	MF	GO:0035091	phosphatidylinositol binding	24/1102	268/28438	0.0001334	0.0015901	0.0011431
GO:0019843	MF	GO:0019843	rRNA binding	11/1102	75/28438	0.0001421	0.0016709	0.0012012
GO:0017069	MF	GO:0017069	snRNA binding	8/1102	42/28438	0.0001809	0.0020698	0.0014880
GO:0044390	MF	GO:0044390	ubiquitin-like protein conjugating enzyme binding	8/1102	42/28438	0.0001809	0.0020698	0.0014880
GO:0043425	MF	GO:0043425	bHLH transcription factor binding	7/1102	32/28438	0.0001850	0.0020896	0.0015023
GO:0140035	MF	GO:0140035	ubiquitination-like modification-dependent protein binding	5/1102	15/28438	0.0001878	0.0020934	0.0015050
GO:0061650	MF	GO:0061650	ubiquitin-like protein conjugating enzyme activity	8/1102	43/28438	0.0002147	0.0023627	0.0016986
GO:0042608	MF	GO:0042608	T cell receptor binding	7/1102	33/28438	0.0002271	0.0024667	0.0017734
GO:0071889	MF	GO:0071889	14-3-3 protein binding	9/1102	55/28438	0.0002435	0.0026117	0.0018776
GO:0019887	MF	GO:0019887	protein kinase regulator activity	19/1102	197/28438	0.0002532	0.0026830	0.0019288
GO:0008013	MF	GO:0008013	beta-catenin binding	12/1102	94/28438	0.0002763	0.0028911	0.0020785
GO:0043022	MF	GO:0043022	ribosome binding	10/1102	69/28438	0.0003123	0.0032283	0.0023209
GO:0033218	MF	GO:0033218	amide binding	33/1102	446/28438	0.0003289	0.0033602	0.0024157
GO:0001223	MF	GO:0001223	transcription coactivator binding	7/1102	35/28438	0.0003341	0.0033731	0.0024250
GO:0051219	MF	GO:0051219	phosphoprotein binding	13/1102	110/28438	0.0003384	0.0033764	0.0024274
GO:0035613	MF	GO:0035613	RNA stem-loop binding	5/1102	17/28438	0.0003628	0.0035786	0.0025728
GO:0003755	MF	GO:0003755	peptidyl-prolyl cis-trans isomerase activity	7/1102	36/28438	0.0004010	0.0039104	0.0028113
GO:1990841	MF	GO:1990841	promoter-specific chromatin binding	10/1102	72/28438	0.0004435	0.0042764	0.0030744
GO:0035035	MF	GO:0035035	histone acetyltransferase binding	6/1102	27/28438	0.0004910	0.0046296	0.0033283
GO:0016407	MF	GO:0016407	acetyltransferase activity	12/1102	100/28438	0.0004917	0.0046296	0.0033283
GO:0043621	MF	GO:0043621	protein self-association	10/1102	73/28438	0.0004964	0.0046296	0.0033283
GO:0016746	MF	GO:0016746	acyltransferase activity	22/1102	260/28438	0.0005444	0.0050226	0.0036109
GO:0031369	MF	GO:0031369	translation initiation factor binding	7/1102	38/28438	0.0005668	0.0051739	0.0037197
GO:0016747	MF	GO:0016747	acyltransferase activity, transferring groups other than amino-acyl groups	20/1102	228/28438	0.0006075	0.0054868	0.0039446
GO:0030544	MF	GO:0030544	Hsp70 protein binding	8/1102	50/28438	0.0006260	0.0055375	0.0039810
GO:0031491	MF	GO:0031491	nucleosome binding	8/1102	50/28438	0.0006260	0.0055375	0.0039810
GO:0061631	MF	GO:0061631	ubiquitin conjugating enzyme activity	7/1102	39/28438	0.0006679	0.0058482	0.0042044
GO:0048027	MF	GO:0048027	mRNA 5'-UTR binding	6/1102	29/28438	0.0007375	0.0063919	0.0045953
GO:0016859	MF	GO:0016859	cis-trans isomerase activity	7/1102	40/28438	0.0007828	0.0067171	0.0048291
GO:0005525	MF	GO:0005525	GTP binding	27/1102	358/28438	0.0008276	0.0070309	0.0050547
GO:0004596	MF	GO:0004596	peptide alpha-N-acetyltransferase activity	4/1102	12/28438	0.0008648	0.0072043	0.0051794
GO:0031386	MF	GO:0031386	protein tag	4/1102	12/28438	0.0008648	0.0072043	0.0051794
GO:0061733	MF	GO:0061733	peptide-lysine-N-acetyltransferase activity	7/1102	41/28438	0.0009128	0.0075311	0.0054143
GO:0004721	MF	GO:0004721	phosphoprotein phosphatase activity	16/1102	170/28438	0.0009813	0.0080194	0.0057653
GO:0030971	MF	GO:0030971	receptor tyrosine kinase binding	10/1102	80/28438	0.0010313	0.0082996	0.0059668
GO:0140678	MF	GO:0140678	molecular function inhibitor activity	14/1102	139/28438	0.0010350	0.0082996	0.0059668
GO:0051059	MF	GO:0051059	NF-kappaB binding	6/1102	31/28438	0.0010701	0.0085016	0.0061120
GO:0005078	MF	GO:0005078	MAP-kinase scaffold activity	4/1102	13/28438	0.0012110	0.0092775	0.0066699
GO:0016018	MF	GO:0016018	cyclosporin A binding	4/1102	13/28438	0.0012110	0.0092775	0.0066699

**GO:1990226**	MF	GO:1990226	histone methyltransferase binding	4/1102	13/28438	0.001211[illegible]	0.009277[illegible]	0.006669[illegible]
**GO:1990405**	MF	GO:1990405	protein antigen binding	4/1102	13/28438	0.001211[illegible]	0.009277[illegible]	0.006669[illegible]
**GO:0042288**	MF	GO:0042288	MHC class I protein binding	7/1102	43/28438	0.001223[illegible]	0.009289[illegible]	0.006678[illegible]
**GO:0070182**	MF	GO:0070182	DNA polymerase binding	5/1102	22/28438	0.001314[illegible]	0.009892[illegible]	0.007112[illegible]

geneID	Count
Hsp90ab1/Hsp90aa1/Rps8/Rps5/Rpsa/Rps24/Mbnl1/Rpl38/Rps21/Rps7/Rps27/Rps16/Rps2/Rpl13a/Rps19/Rpl35/Rpl24/Rpl14/Ptges3/Rpl35a/Rps17/Rps28/Fcf1/Rpl7a/Sf3b1/Sart1/Bms1/Eif3a/Snrpd3/Isy1/Lsm2/Sart3/R	73
Hspa8/Rps26/Mbnl1/Rps13/Malat1/Fam172a/Rbm39/Dazap1/Son/Pnn/Sf3b1/Srsf7/Sap18/Clk1/Sart1/Ahnak/Srrm2/Snrpd3/Isy1/Lsm2/Acin1/Rbm17/Hnrnpl/Sart3/Rbm5/Hnrnph3/Scaf11/Celf1/Polr2a/Hnrnph2/Npm1/Snrn	68
Hspa8/Rps26/Mbnl1/Rps13/Malat1/Fam172a/Rbm39/Dazap1/Son/Srsf7/Sap18/Clk1/Ahnak/Acin1/Rbm17/Hnrnpl/Rbm5/Hnrnph3/Celf1/Polr2a/Hnrnph2/Npm1/Snrnp70/Celf2/Akt2/Snw1/Zfp326/Magoh/C1qbp/Ptbp3/Pik3r	41
Hspa8/Mbnl1/Zfp36/Malat1/Fam172a/Rbm39/Rc3h2/Pcid2/Dazap1/Son/Srsf7/Sap18/Dhx36/Acin1/Pabpc4/Hnrnpl/Rbm5/Zfp36l2/Celf1/Hnrnpa0/Npm1/Snrnp70/Celf2/Zc3hav1/Snw1/Cdk9/Taf15/Iws1/Rock1/Magoh/Tnrc6	54
Hspa1a/Hspa1b/Hsp90ab1/Trim25/Smad7/Fbxl20/Stt3b/Arih1/Ccdc47/Psmb3/Rnf4/Aup1/Os9/Erlin1/Ubr1/Sirt1/Csnk1d/Itch/Tbl1x/Arrb2/Fbxl3/Ube2j2/Xpo1/Nfe2l2/Fbxo38/Siah2/Nrros/Birc2/Gabarap/Ddrgk1/Smarcc1/Dn	63
Hspa8/Mbnl1/Malat1/Fam172a/Rbm39/Dazap1/Son/Pnn/Sf3b1/Srsf7/Sap18/Pcbp1/Sart1/Dhx36/Srrm2/Snrpd3/Isy1/Lsm2/Acin1/Ssu72/Rbm17/Hnrnpl/Sart3/Cmtr1/Rbm5/Scaf11/Celf1/Npm1/Snrnp70/Celf2/Fip1l1/Snw1/	66
Rps8/Rps5/Rpsa/Rps24/Rpl38/Rps21/Rps7/Rps27/Rps16/Rps2/Rps19/Rpl35/Rpl24/Rpl14/Rpl35a/Rps17/Rps28/Fcf1/Rpl7a/Sart1/Bms1/Wdr74/Pop7/Rrp1/Usp36/Xpo1/Nifk/Npm1/Abt1/Exosc1/Nup88/Rrs1/Dhx30/Wdr43	50
Phf14/Kdm5a/Kmt2e/Arid4b/Arid5a/Satb1/Atf2/Morf4l2/Kdm6b/Naa50/Pbxip1/Crebbp/Setd2/Irf4/Sart3/Sirt1/Tbl1x/Ldb1/Jmjd1c/Phf20/Daxx/Usp36/Mtf2/Ube2e1/Ep300/Prkcb/Kmt2c/Ogt/Ube2b/Usp7/Snw1/Cdk9/Mecp2/	63
Kdm5a/Kmt2e/Arid4b/Satb1/Fam172a/Hltf/Nap1l1/Morf4l2/Rere/Smchd1/Kdm6b/Arid1b/Chd1/Chd8/Nr3c1/Setd2/Myc/Ubtf/Smarca5/Sart3/Rsf1/Emsy/Sirt1/Jmjd1c/Phf20/Daxx/Mtf2/Smarce1/Brd9/Prkcb/Npm1/Smarcc1/	64
Rps8/Rps5/Rpsa/Rps24/Rpl38/Rps21/Rps7/Rps27/Rps16/Rps2/Rps19/Rps17/Rps28/Fcf1/Bms1/Npm1/Abt1/Rrs1/Mrps7/Riok1/Gtf2h5/Nol11/Riok3/Ddx52	24
Ubb/Hsp90ab1/Hsp90aa1/Dnaja1/Birc3/Rps7/Rps2/Smad7/Mul1/Rps3/Laptm5/Egr1/Nmi/Sart3/Arnt/Itch/Arrb2/Daxx/Ube2d1/Cblb/Siah2/Npm1/Birc2/Gabarap/Ogt/Cdk9/Herpud1/Ivns1abp/Tspo/Uba2/Usp4/Dnajb2/Pttg1i	43
Jun/Ppp1r15a/Trim25/Stt3b/Atf3/H13/Pmaip1/Ccdc47/Ppp1r15b/Tmem259/Aup1/Os9/Bcl2/Erlin1/Sirt1/Tmed2/Ube2j2/Nfe2l2/Ep300/Nrros/Erp44/Ddrgk1/Dnajb9/Ptpn2/Herpud1/Ube4b/Eif2ak3/Dnajb2/Derl2/Pik3r1/Tmub1	43
Hsp90ab1/Hsp90aa1/Rps5/Rpsa/Mbnl1/Rpl38/Rps27/Rps2/Rpl13a/Rps19/Rpl24/Ptges3/Rps28/Sf3b1/Eif3a/Snrpd3/Isy1/Lsm2/Sart3/Rbm5/Scaf11/Celf1/Abt1/Celf2/Rrs1/Dhx30/Mrps7/Usp4/Luc7l2/Srsf10/Setx/Prpf8/Srp	35
Hspa8/Mbnl1/Malat1/Fam172a/Rbm39/Dazap1/Son/Sf3b1/Srsf7/Sap18/Sart1/Srrm2/Snrpd3/Isy1/Lsm2/Acin1/Rbm17/Hnrnpl/Sart3/Rbm5/Scaf11/Celf1/Npm1/Snrnp70/Celf2/Snw1/Casc3/Magoh/Usp4/Luc7l2/C1qbp/Srrm	45
Hspa8/Mbnl1/Malat1/Fam172a/Rbm39/Dazap1/Son/Sf3b1/Srsf7/Sap18/Sart1/Srrm2/Snrpd3/Isy1/Lsm2/Acin1/Rbm17/Hnrnpl/Sart3/Rbm5/Scaf11/Celf1/Npm1/Snrnp70/Celf2/Snw1/Casc3/Magoh/Usp4/Luc7l2/C1qbp/Srrm	45
Hspa8/Mbnl1/Malat1/Fam172a/Rbm39/Dazap1/Son/Sf3b1/Srsf7/Sap18/Sart1/Srrm2/Snrpd3/Isy1/Lsm2/Acin1/Rbm17/Hnrnpl/Sart3/Rbm5/Scaf11/Celf1/Npm1/Snrnp70/Celf2/Snw1/Casc3/Magoh/Usp4/Luc7l2/C1qbp/Srrm	45
Hspa8/Hspa1a/Hspa1b/Hsp90aa1/Ppp1r15a/Hsph1/Hspd1/Stt3b/Atf3/Hspb1/Tmed2/Daxx/Ube2j2/Nfe2l2/Ep300/Erp44/Ddrgk1/Dnajb9/Ptpn2/Herpud1/Eif2ak3/Derl2/Pik3r1/Hspa9/Hspa14/Atf6/Dnajc3/Crebrf/Ptpn1	29
Hspa8/Hspa1a/Hspa1b/Hsp90aa1/Ppp1r15a/Hsph1/Hspd1/Stt3b/Atf3/Hspb1/Aup1/Tmed2/Daxx/Ube2j2/Nfe2l2/Ep300/Erp44/Ddrgk1/Dnajb9/Ptpn2/Herpud1/Eif2ak3/Derl2/Pik3r1/Hspa9/Ngly1/Hspa14/Atf6/Dnajc3/Crebrf/	31
Hsp90ab1/Hsp90aa1/Rps5/Rpsa/Mbnl1/Rpl38/Rps27/Rps2/Rpl13a/Rps19/Rpl24/Ptges3/Rps28/Sf3b1/Eif3a/Snrpd3/Isy1/Lsm2/Sart3/Rbm5/Scaf11/Celf1/Abt1/Celf2/Rrs1/Dhx30/Mrps7/Usp4/Luc7l2/Srsf10/Setx/Prpf8/Srp	35
Hspa1a/Hspa1b/Hsp90aa1/Zfp36/Smad7/Irs2/Rc3h2/Laptm5/Arih1/Dhx36/Tmem259/Map2k1/Myc/Sirt1/Csnk1d/Itch/Zfp36l2/Foxo1/Celf1/Zbtb20/Gabarap/Ddrgk1/Akt2/Zc3hav1/Herpud1/Zfand2a/Rock1/Tnrc6a/Dnajb2/C	55
Hspa1a/Hspa1b/Hsp90aa1/Nsf/Zfp36/Smad7/Irs2/Rc3h2/Laptm5/Arih1/Dhx36/Tmem259/Map2k1/Myc/Sirt1/Csnk1d/Itch/Zfp36l2/Foxo1/Celf1/Cblb/Csnk2a1/Zbtb20/Gabarap/Ddrgk1/Akt2/Zc3hav1/Herpud1/Zfand2a/Mylip	60
Hspa8/Hsp90ab1/Nfkbia/Syk/Akap13/Bach2/Atf2/Pcid2/Malt1/Adam10/Sp100/Setd2/Mbtps1/Nup50/Rae1/Xpo1/Npm1/Ipo7/Nup88/Rrs1/Prkag1/Kpnb1/Anp32b/Iws1/Ppp3ca/Xpo6/Poldip3/Pttg1ip/Ing1/Pik3r1/Sfn/Eny2/H	47
Hspa8/Hsp90ab1/Nfkbia/Syk/Akap13/Bach2/Atf2/Pcid2/Malt1/Adam10/Sp100/Setd2/Mbtps1/Nup50/Rae1/Xpo1/Npm1/Ipo7/Nup88/Rrs1/Prkag1/Kpnb1/Anp32b/Iws1/Ppp3ca/Xpo6/Poldip3/Pttg1ip/Ing1/Pik3r1/Sfn/Eny2/H	47
Ppp1r15a/Gapdh/Rpl38/Eif1/Zfp36/Rpl13a/Rc3h2/Rps3/Ifngr1/Eif3k/Ppp1r15b/Dhx36/Hnrnpl/Ddx6/Tmed2/Zfp36l2/Eif4g2/Celf1/Nfe2l2/Eif3e/Npm1/Bank1/Akt2/Rock1/Casc3/Eif2ak3/Magoh/Tnrc6a/Bin1/Cnot1/C1qbp/Pol	56
Rpl18/Rplp2/Rpsa/Rpl19/Rpl9-ps6/Rps26/Rpl8/Rpl38/Rps21/Rpl30/Rps2/Rpl13a/Rpl18a/Rpl24/Rpl31/Rpl35a/Rps28/Rpl15/Rpl36a/Rpl22l1/Rpl39/Eif3a/Dhx36/Eif2s2/Eif2s3x	25
Rps8/Rps24/Rps21/Rps7/Rps27/Rps16/Rps19/Rpl35/Rpl14/Rpl35a/Rps17/Rps28/Fcf1/Rpl7a/Sart1/Bms1/Wdr74/Pop7/Rrp1/Usp36/Nifk/Npm1/Abt1/Exosc1/Rrs1/Wdr43/Nhp2/Riok1/Pes1/Ddx47/Gtf2h5/Nol11/Mphosph1	37
Klf2/Jun/Fos/Nfkbia/Mef2c/Foxp1/Zfp36/Gpr171/Junb/Rps19/Kmt2e/Clec2d/Inpp5d/Tmod3/Tcea1/Klf10/Tcirg1/Prdx3/Cited2/Sbno2/Irf4/Pabpc4/Myc/Sirt1/Ets1/Ldb1/Nfatc1/Ep300/Sh2b3/Nrros/Ptpn2/Stap1/Cebpg/Hax1/	54
Dnajb1/Hspa8/Hspe1/Hspa1a/Hspa1b/Hsp90ab1/Hsp90aa1/Dnaja1/Hsph1/Hspd1/Ptges3/Ahsa1/Hspb1/Fkbp4/Nudcd3/Nudc/St13/Dnajb11/Erp44/Fkbp1a/Ube4b/Tbca/Nktr/Dnajb2/Tbce/Hspa9/Ppig/Hspa14/Ranbp2/Dna	32
Ubb/Dnaja1/Hsph1/Rps7/Gpx1/Atf3/Rps3/Plaur/Tpt1/Pmaip1/Casp2/Hspb1/Sgk3/Sp100/Map2k1/Myc/Bcl2/Dedd2/Parp1/Sirt1/Arrb2/Daxx/Rb1cc1/Traf1/Nfe2l2/Fis1/Siah2/Eno1/Ptpn2/Ghitm/Herpud1/Ivns1abp/Eif2ak3/P	52
Hspa8/Mbnl1/Malat1/Fam172a/Rbm39/Dazap1/Son/Srsf7/Sap18/Dhx36/Acin1/Hnrnpl/Rbm5/Celf1/Npm1/Snrnp70/Celf2/Snw1/Cdk9/Iws1/Magoh/Ccnt1/C1qbp/Ptbp3/Paf1/Srsf10/Nsrp1/Mbnl2/Srpk2/Safb	30
Rps8/Rps24/Rps21/Rps7/Rps27/Rps16/Rps19/Rpl35/Rpl14/Rpl35a/Rps17/Rps28/Fcf1/Rpl7a/Sart1/Bms1/Wdr74/Pop7/Rrp1/Usp36/Nifk/Npm1/Abt1/Exosc1/Rrs1/Wdr43/Nhp2/Riok1/Pes1/Ddx47/Gtf2h5/Nol11/Mphosph1	37
Ppp1r15a/Gapdh/Rpl38/Eif1/Zfp36/Rpl13a/Rc3h2/Rps3/Eif3k/Ppp1r15b/Dhx36/Hnrnpl/Ddx6/Tmed2/Zfp36l2/Eif4g2/Celf1/Eif3e/Npm1/Bank1/Akt2/Rock1/Casc3/Eif2ak3/Magoh/Tnrc6a/Cnot1/C1qbp/Poldip3/Per1/Pan3/Cn	50
Hspa8/Jun/Hsp90ab1/Vps37b/Trim25/Zfp36/Eif3f/Top2b/Rab43/Pcbp1/Eif3a/Chd1/Crebbp/Sp100/Trim59/Vapa/Bcl2/Rsf1/Ddx6/Arl8b/Dynlt1c/Ist1/Ep300/Ccnt2/Bsg/Zc3hav1/Inpp5k/Cul1/Snw1/Csde1/Atg16l2/Fam111a/St	46
Hspa1a/Hspa1b/Rps5/Rpsa/Mef2c/Cfl2/Actg1/Rpl38/Tubb5/Rps27/Foxp1/Rps2/Rps19/Akap13/Rpl24/Cenpa/Rps3/Rps28/Tmod3/G3bp1/Limd1/Senp6/Rnf4/Aup1/Ddx6/Csrp1/Csnk1d/Npm1/Prrc2c/Abt1/Rrs1/Dhx30/Kpn	48
Hspa1a/Hspa1b/Ubb/Hsp90ab1/Hsp90aa1/Nsf/Rps7/Smad7/Gpx1/Fbxl20/Azin1/Laptm5/Arih1/Tmem259/Snx12/Psmd3/Csnk1d/Itch/Foxo1/Xpo1/Cblb/Csnk2a1/Gabarap/Ddrgk1/Ctsa/Smarcc1/Ogt/Usp7/Herpud1/Psmd2/	49
Hsp90aa1/Ly6d/Foxp1/Smad7/Syk/Ms4a1/Satb1/Rc3h2/H2-Ab1/Atf2/Pcid2/Nfatc3/Laptm5/Inpp5d/Hspb1/Sart1/Tcirg1/Malt1/Bcl11a/Egr1/Irf4/Bcl2/Zfp36l2/Nfkbid/Ptprj/Nfatc1/Ep300/Dclre1c/Sh2b3/Dnajb9/Ptpn2/Gadd4	52
Hspa1b/Nr4a1/Hspd1/Klf4/Syk/Gpx1/Mul1/Rps3/Plaur/Laptm5/Pmaip1/Casp2/Pdcd2/Malt1/Myc/Sirt1/Arl6ip1/Arrb2/Fis1/Siah2/Csnk2a1/Akt2/Mical1/Herpud1/Anp32b/Eif2ak3/Fnip1/Stat1/Rnf34/Sfn/Fcmr/Ppp2ca/Pml/Bcl	34
Hspa8/Hspa1a/Hspa1b/Ppp1r15a/Hspd1/Atf3/Tmed2/Daxx/Nfe2l2/Ep300/Ddrgk1/Ptpn2/Herpud1/Eif2ak3/Derl2/Pik3r1/Hspa9/Hspa14/Atf6/Dnajc3/Crebrf/Ptpn1	22
Jun/Hsp90aa1/Mef2c/Ly6d/Foxp1/Smad7/Syk/Ms4a1/Satb1/Rc3h2/H2-Ab1/Atf2/Pcid2/Nfatc3/Laptm5/Inpp5d/Hspb1/Sart1/Tcirg1/Malt1/Bcl11a/Egr1/Irf4/Myc/Bcl2/Zfp36l2/Nfkbid/Ptprj/Nfatc1/Ep300/Dclre1c/Sh2b3/Dnajb	55
Hspa8/Hspa1a/Hspa1b/Ppp1r15a/Hspd1/Atf3/Aup1/Tmed2/Daxx/Nfe2l2/Ep300/Ddrgk1/Ptpn2/Herpud1/Eif2ak3/Derl2/Pik3r1/Hspa9/Ngly1/Hspa14/Atf6/Dnajc3/Crebrf/Ptpn1	24
Hsph1/Foxp1/Smad7/Syk/Satb1/Rc3h2/H2-Ab1/Atf2/Tcirg1/Malt1/Rasal3/Irf4/Bcl2/Itch/Nfkbid/Wdfy4/Cblb/H2-M3/Btla/Gadd45g/Cd55/Ly9/Il4ra/Psap/Ccr7/Dock2/Cd81/Relb/Zc3h12a/Ptprc/Bcl6	31
Hspa1b/Nr4a1/Hspd1/Klf4/Syk/Gpx1/Mul1/Rps3/Plaur/Pcid2/Laptm5/Pmaip1/Casp2/Pdcd2/Malt1/Myc/Sirt1/Arl6ip1/Arrb2/Fis1/Siah2/Csnk2a1/Akt2/Mical1/Herpud1/Anp32b/Eif2ak3/Fnip1/Stat1/Rnf34/Sfn/Fcmr/Ppp2ca/P	36
Jun/Cyth1/P2ry10/Rhob/Dennd4a/Ralgapa1/Akap13/Cdc42se2/Dgkz/Rasal3/Map2k1/Dynlt1c/Brap/Bcar3/Gdi2/Rala/Kras/Gabarap/Rap1a/Camk2d/Ogt/Elmo1/Kpnb1/Arfgef1/Rasa2/Rock1/Cd2ap/Rhog/Nisch/Dock8/Arhga	50
Kdm5a/Satb1/Nap1l1/Rere/Kdm6b/Arid1b/Chd1/Chd8/Nr3c1/Setd2/Myc/Smarca5/Sart3/Rsf1/Daxx/Smarce1/Npm1/Smarcc1/Cabin1/Anp32b/Cebpg/Bptf/Atrx/Arid1a/Ubn1/Smarca2/Paf1/Nap1l4/Kdm6a/Nasp/Supt16/Chc	33
Phf14/Mul1/Kmt2e/Arid4b/Arid5a/Atf2/Morf4l2/Naa50/Pbxip1/Crebbp/Senp6/Bcl11a/Egr1/Dhps/Setd2/Irf4/Arnt/Sirt1/Ldb1/Phf20/Mtf2/Ep300/Dip2b/Kmt2c/Ogt/Snw1/Mecp2/Ehmt1/Iws1/Uba2/Atrx/Per1/Brd8/Epc1/Pax5/R	46
Smad7/Gpx1/Mul1/Rps19/Tsc22d3/Dusp1/Rc3h2/H2-Ab1/Pag1/Laptm5/Tnfaip8l2/Clec2d/Inpp5d/Hspb1/Rnf125/Dgkz/Nmi/Myc/Elf1/Cr1l/Itch/Cd84/Nfkbid/Arrb2/Siglecg/Ptprj/Cblb/Bank1/Tspan32/Ptpn2/Btla/Cd55/Stap1/	52
Ubb/Hsp90ab1/Hsp90aa1/Dnaja1/Birc3/Rps7/Rps2/Smad7/Rps3/Laptm5/Nmi/Arrb2/Daxx/Ube2d1/Cblb/Siah2/Npm1/Birc2/Gabarap/Ogt/Cdk9/Herpud1/Ivns1abp/Tspo/Usp4/Dnajb2/Pttg1ip/Chfr/Fbxo33/Pten/Mycbp2/Peli	33
Klf2/Dnaja1/Nfkbia/Foxp1/Ptges3/Rnf6/Rnf125/Cited2/Nr3c1/Fkbp4/Parp1/Sirt1/Daxx/Ep300/Ddrgk1/Zc3hav1/Snw1/Lbh/Cnot1/Ankrd17/Arid1a/C1qbp/Per1/Ncoa2/Rnf34/Cnot2/Usp15/Pml/Riok3/Pum2/Prcp/Safb/Calr	33
Zfp36/Rc3h2/Pcid2/Dhx36/Lsm2/Pabpc4/Zfp36l2/Celf1/Hnrnpa0/Eif3e/Smg5/Smg1/Npm1/Zc3hav1/Csde1/Taf15/Rock1/Casc3/Magoh/Tnrc6a/Cnot1/Pan3/Myd88/Cnot2/Ythdf3/Fus/Larp1/Tnrc6c/Secisbp2/Zc3h12a/Pum2	34
Hspa8/Mbnl1/Malat1/Fam172a/Rbm39/Dazap1/Son/Srsf7/Sap18/Acin1/Hnrnpl/Rbm5/Celf1/Npm1/Snrnp70/Celf2/Snw1/Magoh/C1qbp/Ptbp3/Srsf10/Nsrp1/Mbnl2	23
Nr4a1/Cacybp/Atp2b1/Irs2/Myo1c/Egr1/Parp1/Sirt1/Bcar3/Foxo1/Zfp106/Ptprj/Nfe2l2/Pde3b/Prkcb/Ikbkb/Eif2b2/Lmbrd1/Sik2/Stat4/Smarcc1/Akt2/Inpp5k/Ogt/Ptpn2/Nfkb1/Rock1/Vamp2/Stat1/Hadha/Pik3r1/Ccnd3/Ctnn	43
Zfp36/Rc3h2/Pcid2/Dhx36/Lsm2/Pabpc4/Zfp36l2/Celf1/Hnrnpa0/Eif3e/Smg5/Smg1/Npm1/Zc3hav1/Csde1/Taf15/Rock1/Casc3/Magoh/Tnrc6a/Cnot1/Pan3/Myd88/Sidt2/Cnot2/Ythdf3/Fus/Rnaset2a/Larp1/Tnrc6c/Secisbp2	37
Hsp90aa1/Trim25/Mul1/Atf2/Pmaip1/Ifngr1/Rnf125/G3bp1/Crebbp/Dhx36/Nmi/Setd2/Aup1/Bcl2/Itch/Wdfy4/Tspan32/Tagap/Zc3hav1/Nfkb1/Ivns1abp/Ifnar1/Ankrd17/Cd86/C1qbp/Zmynd11/Stat1/Myd88/Usp15/Bst2/Trim1	40
Jun/Dnaja1/Ppp1r15a/Wnk1/Birc3/Smad7/Dynll1/Dusp1/Irs2/Hspb1/Bod1l/Prdx3/Ppp1r15b/Gadd45b/Hipk3/Parp1/Sirt1/Tmed2/Arrb2/Ptprj/Ikbkb/Cblb/Sh2b3/Npm1/Iqgap1/Pik3ip1/Mical1/Inpp5k/Ogt/Ptpn2/Ube2b/Gadd4	50
Jun/Dnaja1/Ppp1r15a/Wnk1/Birc3/Smad7/Dynll1/Dusp1/Irs2/Hspb1/Bod1l/Prdx3/Ppp1r15b/Gadd45b/Hipk3/Parp1/Sirt1/Tmed2/Arrb2/Ptprj/Ikbkb/Cblb/Sh2b3/Npm1/Iqgap1/Pik3ip1/Mical1/Inpp5k/Ogt/Ptpn2/Ube2b/Gadd4	50
Nr4a1/Nfkbia/Cacybp/Atp2b1/Itga4/Irs2/Myo1c/Egr1/Parp1/Sirt1/Bcar3/Foxo1/Zfp106/Ptprj/Nfe2l2/Pde3b/Prkcb/Ikbkb/Fis1/Eif2b2/Lmbrd1/Sik2/Stat4/Smarcc1/Akt2/Inpp5k/Ogt/Ptpn2/Nfkb1/Rock1/Vamp2/Stat1/Hadha/Pi	47
Ubb/Birc3/Rps2/Smad7/Mul1/Laptm5/Nmi/Sart3/Arnt/Ube2d1/Cblb/Npm1/Birc2/Gabarap/Cdk9/Uba2/Dnajb2/Pttg1ip/Chfr/Fbxo33/Pten/Sae1/Mycbp2/Peli1/Zc3h12a/Gnl3	26
Gapdh/Nfkbia/Trim25/Akap13/Traf4/Rel/Hspb1/Malt1/Dhx36/Trim59/Sirt1/Nfkbid/Card6/Traf1/Prkcb/Ikbkb/Ddrgk1/Zc3hav1/Rock1/Ankrd17/Per1/Zmynd11/Stat1/Myd88/Ctnnb1/Trim12a/Zfand6/Rhoh/Peli1/Relb/Riok3/Chuk	33
Trim25/Stt3b/H13/Ccdc47/Tmem259/Aup1/Os9/Erlin1/Ube2j2/Nrros/Dnajb9/Herpud1/Ube4b/Eif2ak3/Dnajb2/Derl2/Tmub1/Sel1l/Ngly1/Hsp90b1/Usp19/Calr	22
Hspa8/Hsp90ab1/Hsp90aa1/Dnaja1/Nfkbia/Hsph1/Hspd1/Macf1/Vps37b/Syk/Il10ra/Atf2/Laptm5/Pmaip1/Pmpcb/Mbtps1/Nup50/Rala/Fis1/Ipo7/Nup88/Pik3r4/Kpnb1/Herpud1/Zfand2a/Ppp3ca/Tspo/Vps13a/Srpr/Pex7/Pttg	47
Cenpa/Nap1l1/Senp6/Rnf4/Setd2/Myc/Smarca5/Sart3/Rsf1/Daxx/Tcf4/Smarce1/Npm1/Gtf2a2/Smarcc1/Cabin1/Anp32b/Cebpg/Bptf/Atrx/Arid1a/Gtf2h5/Bdp1/Ubn1/Smarca2/Paf1/Nap1l4/Nasp/Supt16/Kat6a	30
Phf14/Arid5a/Atf2/Naa35/Morf4l2/Zdhhc3/Gm16286/Naa50/Pbxip1/Crebbp/Naa10/Irf4/Zdhhc18/Sirt1/Ldb1/Phf20/Ep300/Dip2b/Nmt2/Ogt/Mecp2/Iws1/Naa15/Ogdh/Per1/Brd8/Epc1/Ep400/Golga7/Flcn/Kmt2a/Phf20l1/Kat	34
Nap1l1/Chd1/Ubtf/Smarca5/Sart3/Rsf1/Sirt1/Daxx/Smarce1/Npm1/Smarcc1/Cabin1/Anp32b/Cebpg/Atrx/Arid1a/Tlk1/Ubn1/Smarca2/Paf1/Nap1l4/Nasp/Supt16/Kat6a	24
Hspa8/Mul1/Il10ra/Rab43/Atp6v0a1/Tcirg1/Anxa7/Tmem208/Herc1/Atg12/Map2k1/Aup1/Bcl2/Arl8b/Sirt1/Cd84/Eif4g2/Foxo1/Usp36/Rb1cc1/Pik3c3/Wdfy4/Snapin/Fis1/Smg1/Gabarap/Ddrgk1/Ctsa/Nsfl1c/Pik3r4/Rock1/Ts	48
Hspa8/Mul1/Il10ra/Rab43/Atp6v0a1/Tcirg1/Anxa7/Tmem208/Herc1/Atg12/Map2k1/Aup1/Bcl2/Arl8b/Sirt1/Cd84/Eif4g2/Foxo1/Usp36/Rb1cc1/Pik3c3/Wdfy4/Snapin/Fis1/Smg1/Gabarap/Ddrgk1/Ctsa/Nsfl1c/Pik3r4/Rock1/Ts	48
Hsp90ab1/Hsp90aa1/Ppp1r15a/Wnk1/Smad7/Syk/Clk1/Sgk3/Gm16286/Naa10/Cab39/Bcl2/Hipk3/Parp1/Csnk1d/Bcar3/Arrb2/Prkcb/Ikbkb/Csnk1g3/Smg1/Csnk2a1/Camk2d/Akt2/Inpp5k/Ogt/Stk38/Rock1/Eif2ak3/Hax1/Tl	43
Zfp36/Rc3h2/Pcid2/Dhx36/Pabpc4/Zfp36l2/Celf1/Hnrnpa0/Npm1/Zc3hav1/Taf15/Rock1/Tnrc6a/Cnot1/Pan3/Myd88/Cnot2/Ythdf3/Fus/Larp1/Tnrc6c/Secisbp2/Zc3h12a/Pum2/Larp4b/Vim/Tnrc6b	27
Hsp90aa1/Pde4b/Gapdh/Hspd1/Foxp1/Syk/Arid5a/Atf2/Rps3/Laptm5/Sptbn1/Gpsm3/Rel/Ifngr1/Hspb1/G3bp1/Malt1/Dhx36/Egr1/Setd2/Irf4/Cd2/Arnt/Sirt1/Cd84/1810058I24Rik/Ptprj/Bsg/Zbtb20/Tnfrsf13c/Dennd1b/H2-M	52
Wnk1/Ralgapa1/Sbf1/S1pr1/Lims1/Pbxip1/Rasal3/Map2k1/Dynlt1c/Tmed2/Bcar3/Arrb2/Cblb/Dennd1b/Rap1a/Rabgap1/Iqgap1/Akt2/Arfgef1/Rasa2/Rcc2/Arhgap15/Rhog/Rabgap1l/Bin1/Dock8/Tbc1d1/Gapvd1/Arhgap25/	40
Hsp90aa1/Wnk1/Hsph1/Hspd1/Klf4/Smad7/Syk/Itga4/Rc3h2/H2-Ab1/Rps3/Pag1/Laptm5/Tnfaip8l2/Hspb1/Sart1/Malt1/Dhps/Rasal3/Ets1/Itch/Nfkbid/Cblb/H2-Eb2/Tnfrsf13c/Ptpn2/Btla/Cd55/Nfat5/Cd86/Dock8/Il6st/Il4ra/	40
Ubb/Dnaja1/Hsph1/Shisa5/Rps7/Gpx1/Atf2/Rps3/Plaur/Tpt1/Pmaip1/Casp2/Hspb1/Aldh2/Map2k1/Myc/Bcl2/Parp1/Sirt1/Nfe2l2/Ep300/Fis1/Eno1/Ptpn2/Snw1/Herpud1/Ivns1abp/Zfp622/Eif2ak3/Pttg1ip/Pik3r1/Sfn/Stk11/	38
Trim25/Stt3b/Ccdc47/Aup1/Os9/Erlin1/Ube2j2/Nrros/Dnajb9/Herpud1/Ube4b/Eif2ak3/Dnajb2/Derl2/Tmub1/Sel1l/Hsp90b1/Usp19/Calr	19
Hspa8/Hspa1a/Hspa1b/Hspd1/Smad7/Syk/Mul1/Rps3/Laptm5/Pmaip1/Casp2/Pdcd2/Arih1/Malt1/Tmem259/Myc/Sart3/Sirt1/Csnk1d/Ist1/Fis1/Gabarap/Ddrgk1/Eno1/Ogt/Herpud1/Anp32b/Zfand2a/Eif2ak3/Dnajb2/Chfr/St	41
Nfkbia/Klf4/Trim25/Smad7/Syk/Trappc9/Kdm5a/Id3/Atf2/Rps3/Ifrd1/Malt1/Prdx3/Crebbp/Sp100/Hes6/Sirt1/Itch/Cd84/Nfkbid/Arrb2/Traf1/Ep300/Prkcb/Ikbkb/Kras/Npm1/Med13/Ddrgk1/Sri/Usp7/Tax1bp1/Cebpg/Ppp3ca/M	46
Jun/Dnaja1/Ppp1r15a/Wnk1/Birc3/Smad7/Dynll1/Dusp1/Irs2/Hspb1/Prdx3/Ppp1r15b/Gadd45b/Hipk3/Sirt1/Tmed2/Arrb2/Ptprj/Cblb/Sh2b3/Npm1/Pik3ip1/Mical1/Inpp5k/Ogt/Ptpn2/Ube2b/Gadd45g/Stk38/Stap1/Rock1/Ra	44
Hsp90ab1/Hsp90aa1/Ppp1r15a/Wnk1/Smad7/Syk/Clk1/Sgk3/Cab39/Bcl2/Hipk3/Csnk1d/Bcar3/Arrb2/Prkcb/Ikbkb/Csnk1g3/Smg1/Csnk2a1/Camk2d/Akt2/Inpp5k/Ogt/Stk38/Rock1/Eif2ak3/Hax1/Tlk1/Fnip1/Mast4/Sbk1/Pt	40
Sertad1/Pcid2/Nr3c1/Setd2/Map2k1/Ubtf/Ldb1/Ccnt2/Wdr43/Zfp326/Ccnt1/Zmynd11/Gtf2h5/Rtf1/Ctnnb1/Paf1/Scaf8/Supt16/Zmiz2	19
Hspa1a/Hspa1b/Ubb/Hsp90ab1/Hsp90aa1/Rps7/Smad7/Gpx1/Laptm5/Arih1/Tmem259/Csnk1d/Xpo1/Csnk2a1/Gabarap/Ddrgk1/Smarcc1/Ogt/Usp7/Herpud1/Zfand2a/Mylip/Dnajb2/Pttg1ip/Chfr/Pten/Faf1/Arih2/Cd81/Pml/	33
Ppp1r15a/Eif1/Rps2/Eif3f/Rpl13a/Eif3k/Eif3a/Ppp1r15b/Tmed2/Eif4g2/Eif3e/Npm1/Bank1/Eif2b2/Eif1ax/Eif2ak3/Larp1/Rps6kb1/Pml/Dnajc3/Eif2s2/Eif2s3x	22
Hsp90aa1/Wnk1/Hsph1/Hspd1/Klf4/Smad7/Syk/Itga4/Rc3h2/H2-Ab1/Rps3/Pag1/Laptm5/Tnfaip8l2/Hspb1/Sart1/Malt1/Dhps/Rasal3/Ets1/Itch/Nfkbid/Cblb/H2-Eb2/Tnfrsf13c/Ptpn2/Btla/Cd55/Rock1/Nfat5/Cd86/Dock8/Il6s	42
Kdm5a/Kmt2e/Arid5a/Sart3/Sirt1/Daxx/Mtf2/Ogt/Ube2b/Snw1/Cdk9/Mecp2/Iws1/Atrx/Pax5/Rtf1/Ctnnb1/Paf1/Akap8l/Flcn/Trip12/Pml/Kmt2a/Nsd1/Chtop/Bcl6/Nipbl	27
Hspa8/Hsp90ab1/Nfkbia/Syk/Atf2/Sp100/Mbtps1/Nup50/Parp1/Sirt1/Chchd10/Xpo1/Npm1/Ipo7/Akt2/Nup88/Rrs1/Ogt/Kpnb1/Ppp3ca/Eif2ak3/Cd2ap/Pttg1ip/Ing1/Pik3r1/Stk11/Rbm22/Paf1/Ppp2ca/Ranbp2/Tnpo1/Flcn/P	38
Nr4a1/Atp2b1/Irs2/Myo1c/Parp1/Sirt1/Bcar3/Foxo1/Zfp106/Ptprj/Nfe2l2/Pde3b/Prkcb/Lmbrd1/Sik2/Smarcc1/Akt2/Inpp5k/Ogt/Ptpn2/Nfkb1/Rock1/Vamp2/Pik3r1/Ccnd3/Ctnnb1/Pten/Rps6kb1/Sp1/Phip/Nucks1/Socs3/Osb	34
Zfp36/Rc3h2/Pcid2/Dhx36/Pabpc4/Zfp36l2/Celf1/Hnrnpa0/Npm1/Taf15/Rock1/Tnrc6a/Cnot1/Pan3/Myd88/Cnot2/Ythdf3/Fus/Larp1/Tnrc6c/Zc3h12a/Pum2/Larp4b/Vim/Tnrc6b	25
Klf2/Pde4b/Rps24/Mef2c/Zfp36/Rps19/Kmt2e/Tsc22d3/Rc3h2/Rps17/Pmaip1/Inpp5d/Tmod3/Tcea1/Mir142hg/Tcirg1/Cited2/Bcl2/Sart3/Ets1/Ldb1/Ikbkb/Kras/Sh2b3/Tnfrsf13c/Stat4/Ptpn2/Cebpg/Stat1/Ptbp3/Hspa9/Ccr7/	41
Phf14/Arid5a/Atf2/Naa35/Morf4l2/Gm16286/Naa50/Pbxip1/Crebbp/Naa10/Irf4/Sirt1/Ldb1/Phf20/Ep300/Dip2b/Ogt/Mecp2/Iws1/Naa15/Per1/Brd8/Epc1/Ep400/Flcn/Kmt2a/Phf20l1/Kat6a/Esco1	29
Hspa8/Hsp90ab1/Hsp90aa1/Gapdh/Hspd1/Ifi30/Rps7/Smad7/Mul1/Ptges3/Crebbp/Bcl2/Sirt1/Tbl1x/Fbxl3/Usp36/Ep300/Kras/Npm1/Ddrgk1/Ctsa/Ube2b/Usp7/Mylip/Naa15/Usp4/Chfr/Pik3r1/Pten/Golga7/Cd81/Pml/Rab3	37
Nr4a1/Atp2b1/Itga4/Irs2/Myo1c/Parp1/Sirt1/Bcar3/Foxo1/Zfp106/Ptprj/Nfe2l2/Pde3b/Prkcb/Fis1/Lmbrd1/Sik2/Smarcc1/Akt2/Inpp5k/Ogt/Ptpn2/Nfkb1/Rock1/Vamp2/Pik3r1/Ccnd3/Ctnnb1/Pten/Rps6kb1/Sp1/Phip/Nucks1/	37
Zfp36/Rc3h2/Pcid2/Dhx36/Pabpc4/Zfp36l2/Celf1/Hnrnpa0/Npm1/Taf15/Rock1/Tnrc6a/Cnot1/Pan3/Myd88/Cnot2/Ythdf3/Fus/Larp1/Tnrc6c/Zc3h12a/Pum2/Zc3h18/Larp4b/Vim/Tnrc6b	26
Akap13/Pcid2/Malt1/Sp100/Setd2/Rae1/Xpo1/Npm1/Nup88/Rrs1/Anp32b/Iws1/Xpo6/Poldip3/Sfn/Eny2/Hspa9/Rbm22/Nemf/Akap8l/Pabpn1/Chtop/Nxf1/Calr/Cse1l	25
Hsp90aa1/Wnk1/Hsph1/Hspd1/Klf4/Smad7/Syk/Itga4/Rc3h2/H2-Ab1/Rps3/Plaur/Pag1/Laptm5/Tnfaip8l2/Hspb1/Sart1/Malt1/Cited2/Dhps/Rasal3/Map2k1/Ets1/Itch/Nfkbid/Cblb/H2-Eb2/Sh2b3/Tnfrsf13c/Jak1/Ptpn2/Btla/	47
Atp2b1/Irs2/Myo1c/Egr1/Parp1/Sirt1/Bcar3/Foxo1/Zfp106/Ptprj/Pde3b/Prkcb/Lmbrd1/Sik2/Smarcc1/Akt2/Inpp5k/Ogt/Ptpn2/Vamp2/Hadha/Pik3r1/Ccnd3/Pten/Rps6kb1/Sp1/Phip/Nucks1/Socs3/Osbpl8/Ptpn1/Srsf5	32
Foxp1/Syk/Ms4a1/H2-Ab1/Pcid2/Laptm5/Inpp5d/Tcirg1/Malt1/Bcl11a/Bcl2/Zfp36l2/Ptprj/Nfatc1/Ep300/Dclre1c/Dnajb9/Ptpn2/Cebpg/Fnip1/Pik3r1/Cd19/Irf2bp2/Ptprc/Bcl6/Dock11	26
Atp2b1/Irs2/Myo1c/Parp1/Sirt1/Bcar3/Foxo1/Zfp106/Ptprj/Pde3b/Prkcb/Lmbrd1/Sik2/Smarcc1/Akt2/Inpp5k/Ogt/Ptpn2/Vamp2/Pik3r1/Ccnd3/Pten/Rps6kb1/Sp1/Phip/Nucks1/Socs3/Osbpl8/Ptpn1	29
Nap1l1/Setd2/Smarca5/Sart3/Rsf1/Daxx/Smarce1/Npm1/Smarcc1/Cabin1/Anp32b/Cebpg/Bptf/Atrx/Arid1a/Ubn1/Smarca2/Paf1/Nap1l4/Nasp/Supt16/Kat6a	22
Rps8/Rps21/Rps16/Rps19/Rps28/Fcf1/Bms1/Abt1/Rrs1/Riok1/Gtf2h5/Nol11/Riok3/Ddx52	14
Hspa8/Hsp90aa1/Hspd1/Macf1/Vps37b/Naca/Myo1c/Laptm5/Pmpcb/Zdhhc3/Syngr2/Exoc4/Os9/Zdhhc18/Arl6ip1/Fis1/Akt2/Inpp5k/Ogt/Pik3r4/Herpud1/Zfand2a/Vps13a/Srpr/Pex7/Pan3/Rabgef1/Tram1/Golga7/Zfand6/P	34
Birc3/Mul1/Rc3h2/Zfp91/Rnf6/Rnf125/Arih1/Ube3b/Rnf4/Nmi/Bcl2/Itch/Ube2j2/Traf1/Ube2e1/Ube2d1/Fbxo38/Birc2/Gabarap/Ube2b/Cul1/Ube2h/Ube4b/Chfr/Rnf34/Ube2g1/Tnks2/Ctnnb1/Trim12a/Arih2/Peli1/Trip12	32
Rps8/Rps24/Rps21/Rps7/Rps27/Rps16/Rps19/Rpl35/Rpl14/Rpl35a/Rps17/Rps28/Fcf1/Rpl7a/Sart1/Bms1/Ints4/Dus3l/Wdr74/Pop7/Rrp1/Usp36/Nifk/Npm1/Abt1/Exosc1/Rrs1/Wdr43/Nhp2/Mecp2/Riok1/Pes1/Ddx47/Gtf2h	40
Irs2/Sirt1/Bcar3/Foxo1/Zfp106/Ptprj/Prkcb/Lmbrd1/Sik2/Smarcc1/Akt2/Inpp5k/Ogt/Ptpn2/Pik3r1/Ccnd3/Rps6kb1/Phip/Nucks1/Socs3/Osbpl8/Ptpn1	22
Dnaja1/Foxp1/Akap13/Ptges3/Rnf6/Nr3c1/Fkbp4/Parp1/Sirt1/Daxx/Ep300/Ddrgk1/Lbh/Cnot1/Arid1a/Per1/Ncoa2/Cnot2/Crebrf/Safb/Calr	21
Rps26/Rps13/Srsf7/Sap18/Acin1/Hnrnpl/Npm1/Akt2/C1qbp/Ptbp3/Srsf10	11
Ubb/Dnaja1/Hsph1/Rps7/Gpx1/Rps3/Plaur/Tpt1/Pmaip1/Hspb1/Map2k1/Bcl2/Parp1/Sirt1/Nfe2l2/Fis1/Eno1/Ptpn2/Herpud1/Ivns1abp/Eif2ak3/Pttg1ip/Bclaf1/Flcn/Bcl2l11/Ptpn1	26
Hsp90aa1/Hsph1/Hspd1/Smad7/Syk/Rc3h2/H2-Ab1/Rps3/Pag1/Laptm5/Tnfaip8l2/Hspb1/Sart1/Malt1/Dhps/Rasal3/Itch/Nfkbid/Cblb/H2-Eb2/Sh2b3/Tnfrsf13c/Ptpn2/Btla/Cd55/Cd86/Dock8/Il6st/Clptm1/Il4ra/Ctnnb1/Ccr7/	38
Gapdh/Trim25/Akap13/Traf4/Hspb1/Dhx36/Trim59/Sirt1/Nfkbid/Card6/Traf1/Prkcb/Ikbkb/Zc3hav1/Ankrd17/Per1/Zmynd11/Stat1/Myd88/Ctnnb1/Trim12a/Zfand6/Rhoh/Peli1/Riok3/Chuk/Zc3h12a	27
Hspa8/Hspa1a/Hspa1b/Hsp90aa1/Hspd1/St13/Dnajb2/Hspa9/Hspa14	9
Hsp90aa1/Foxp1/Smad7/Syk/Satb1/Rc3h2/Atf2/Nfatc3/Hspb1/Sart1/Tcirg1/Malt1/Bcl11a/Egr1/Irf4/Bcl2/Zfp36l2/Nfkbid/Sh2b3/Ptpn2/Gadd45g/Ly9/Clptm1/Il4ra/Ctnnb1/Psap/Ccr7/Dock2/Stk11/Rhoh/Relb/Zc3h12a/Ptprc/	35
Hspa1b/Nr4a1/Gapdh/Hspd1/Klf4/Syk/Gpx1/Mul1/Rps3/Plaur/Pcid2/Laptm5/Pmaip1/Casp2/Pdcd2/Malt1/Myc/Sirt1/Arl6ip1/Arrb2/Fis1/Siah2/Csnk2a1/Akt2/Mical1/Herpud1/Anp32b/Eif2ak3/Bin1/Fnip1/Stat1/Nrd1/Rnf34/S	39
Rnf6/Rnf4/Nmi/Itch/Ube2e1/Ube2d1/Fbxo38/Birc2/Gabarap/Ube2b/Cul1/Ube2h/Rnf34/Ube2g1/Arih2/Peli1	16
Jun/Cyth1/P2ry10/Dennd4a/Akap13/Cdc42se2/Dgkz/Rasal3/Map2k1/Dynlt1c/Brap/Rala/Kras/Gabarap/Rap1a/Camk2d/Ogt/Elmo1/Kpnb1/Arfgef1/Rasa2/Rock1/Rhog/Nisch/Rabgef1/Dock2/Ralgps2/Dnm2/Gpr65/Rhoh/Flc	37
Hsp90aa1/Gapdh/Hspd1/Syk/Rel/Rnf125/G3bp1/Crebbp/Dhx36/Nmi/Setd2/Zbtb20/Zc3hav1/Ifnar1/Stat1/Myd88/Relb/Riok3/Chuk	19
Rps8/Rps24/Rps21/Rps7/Rps27/Rps16/Rps19/Rpl35/Rpl14/Rc3h2/Rpl35a/Rps17/Rps28/Fcf1/Rpl7a/Sart1/Bms1/Ints4/Dus3l/Wdr74/Pop7/Rrp1/Usp36/Nifk/Npm1/Abt1/Exosc1/Rrs1/Wdr43/Nhp2/Mecp2/Nfkb1/Riok1/Pes1	45
Hspa1a/Hspa1b/Hsp90aa1/Nsf/Smad7/Laptm5/Arih1/Tmem259/Csnk1d/Itch/Foxo1/Cblb/Csnk2a1/Gabarap/Ddrgk1/Herpud1/Zfand2a/Mylip/Dnajb2/Pttg1ip/Chfr/Nrd1/Pten/Faf1/Arih2/Cd81/Msn/Apoe/Crebrf	29
Phf14/Arid5a/Atf2/Morf4l2/Naa50/Pbxip1/Crebbp/Irf4/Sirt1/Ldb1/Phf20/Ep300/Dip2b/Ogt/Mecp2/Iws1/Per1/Brd8/Epc1/Ep400/Flcn/Kmt2a/Phf20l1/Kat6a/Esco1	25
Hspa8/Hspd1/Foxp1/Smad7/Syk/Rps19/Arid5a/Rc3h2/Laptm5/Clec2d/Malt1/Myo18a/Dhx36/Cr1l/Sirt1/Cd84/Nfkbid/Arrb2/H2-Q7/Siglecg/Ptprj/Fbxo38/Dennd1b/Rap1a/H2-M3/Dnajb9/Cd55/Stap1/Ankrd17/Cd86/C1qbp/	43
Hsp90ab1/Pgap1/Myo1c/Pmaip1/Sp100/Setd2/Map2k1/Os9/Snx12/Xpo1/Sec24b/Fis1/Akt2/Inpp5k/Ogt/Usp7/Mecp2/Anp32b/Iws1/Pcm1/Vamp2/Derl2/Preb/Tmem30a/Pik3r1/Sfn/Stk11/Rbm22/Ppp2ca/Cd81/Akap8l/Zc3h	37
Klf4/Med15/Tpt1/Klf10/Crebbp/Myc/Ddx6/Zfp36l2/Ldb1/Jmjd1c/Foxo1/Mtf2/Lbh/Wdr43/Pcm1/Med21/Cnot1/Arid1a/Cnot2/Rtf1/Zc3h13/Paf1/Gnl3/Kat6a/Nipbl	25
Phf14/Arid5a/Atf2/Morf4l2/Naa50/Pbxip1/Crebbp/Irf4/Sirt1/Ldb1/Phf20/Ep300/Ogt/Mecp2/Iws1/Per1/Brd8/Epc1/Ep400/Flcn/Kmt2a/Phf20l1/Kat6a	23
Hsp90aa1/Gapdh/Hspd1/Syk/Rel/Rnf125/G3bp1/Crebbp/Dhx36/Nmi/Setd2/Zbtb20/Zc3hav1/Ifnar1/Stat1/Myd88/Relb/Riok3/Chuk	19
Zfp36/Srsf7/Sap18/Acin1/Hnrnpl/Hnrnpa0/Npm1/Cdk9/Taf15/Ccnt1/C1qbp/Ptbp3/Myd88/Fus/Larp1/Secisbp2/Srsf10/Larp4b	18
Zfp36/Rc3h2/Pcid2/Dhx36/Lsm2/Pabpc4/Kynu/Ahcy/Zfp36l2/Celf1/Hnrnpa0/Nfe2l2/Eif3e/Smg5/Smg1/Npm1/Zc3hav1/Nt5c/Csde1/Taf15/Rock1/Casc3/Magoh/Tnrc6a/Cnot1/Pan3/Myd88/Sidt2/Cnot2/Ythdf3/Fus/Rnaset2a	42
Foxp1/Rps3/Laptm5/Dgkz/Malt1/Elf1/Ptprj/Prkcb/Cblb/Ptpn2/Stap1/Cd19/Ccr7/Cd81/Ptprc	15
Birc3/Traf4/Rc3h2/Rps3/Zfp91/Laptm5/Rel/Lims1/Malt1/Crebbp/Nmi/Ep300/Birc2/Nfat5/Cd86/Nfkb2/Relb/Chuk/Zc3h12a/Calr	20
Rps5/Rpsa/Rpl38/Rps27/Rps2/Rps19/Rps28/Abt1/Mrps7	9
Zfp36/Rc3h2/Pcid2/Dhx36/Lsm2/Pabpc4/Ahcy/Zfp36l2/Celf1/Hnrnpa0/Eif3e/Smg5/Smg1/Npm1/Zc3hav1/Nt5c/Csde1/Taf15/Rock1/Casc3/Magoh/Tnrc6a/Cnot1/Pan3/Myd88/Sidt2/Cnot2/Ythdf3/Fus/Rnaset2a/Larp1/Tnrc6	40
Hsp90aa1/Trim25/Mul1/Pmaip1/Ifngr1/Rnf125/G3bp1/Dhx36/Setd2/Bcl2/Itch/Tspan32/Tagap/Zc3hav1/Ifnar1/Ankrd17/Cd86/C1qbp/Zmynd11/Stat1/Myd88/Usp15/Bst2/Trim12a/Pml/Trim11/Riok3/Chuk/Zc3h12a/Pum2/Ptpr	31
Hsp90aa1/Trim25/Mul1/Pmaip1/Ifngr1/Rnf125/G3bp1/Dhx36/Setd2/Bcl2/Itch/Tspan32/Tagap/Zc3hav1/Ifnar1/Ankrd17/Cd86/C1qbp/Zmynd11/Stat1/Myd88/Usp15/Bst2/Trim12a/Pml/Trim11/Riok3/Chuk/Zc3h12a/Pum2/Ptpr	31
Klf4/Med15/Tpt1/Klf10/Crebbp/Myc/Ddx6/Zfp36l2/Ldb1/Jmjd1c/Foxo1/Mtf2/Lbh/Wdr43/Pcm1/Med21/Cnot1/Arid1a/Cnot2/Rtf1/Zc3h13/Paf1/Gnl3/Kat6a/Nipbl	25
Hsp90aa1/Macf1/Vps37b/Atad1/Nsf/Naca/Myo1c/Pmaip1/Sptbn1/Zdhhc3/Ccdc47/Exoc4/Zdhhc18/Arl6ip1/Fis1/Vamp3/Golga4/Akt2/Inpp5k/Ogt/Vamp2/Srpr/Atp2c1/Rabgef1/Tram1/Golga7/Sys1/Sec63/Rab3gap1/Hspa4/	31
Dnaja1/Foxp1/Zfp36/Akap13/Ptges3/Rnf6/Nr3c1/Fkbp4/Parp1/Sirt1/Zfp36l2/Daxx/Ep300/Ddrgk1/Lbh/Cnot1/Arid1a/Per1/Ncoa2/Cnot2/Rps6kb1/Crebrf/Safb/Calr	24
Ppp1r15a/Atf3/Tmed2/Nfe2l2/Ep300/Ddrgk1/Ptpn2/Herpud1/Eif2ak3/Derl2/Pik3r1/Atf6/Dnajc3/Crebrf/Ptpn1	15
Dnajb1/Hspa8/Hspe1/Hspa1a/Hspa1b/Hsph1/Ptges3/Hspb1/Fkbp4/St13/Fkbp1a/Dnajb2/Hspa9/Hspa14	14
Kmt2e/Arid5a/Sart3/Sirt1/Daxx/Mtf2/Ogt/Snw1/Cdk9/Mecp2/Rtf1/Ctnnb1/Paf1/Akap8l/Pml/Kmt2a/Chtop/Bcl6/Nipbl	19
Macf1/Nsf/Pgap1/Tmed5/Laptm5/Sptbn1/Myo18a/Exoc4/Vapa/Snx12/Tmed2/Cux1/Sec24b/Vamp3/Golga4/Arcn1/Vamp2/Rer1/Ergic3/Copb2/Tmed9/Preb/Atp2c1/Uvrag/Ergic2/Dnm2/Golga7/Vti1b/Trappc6b/Sys1/Copa/S	32
Hsp90ab1/Hsp90aa1/Macf1/Foxp1/Smad7/Mul1/Akap13/Itga4/Zeb2/Ifrd1/Rnf6/Bcl11a/Nr3c1/Sirt1/Ist1/Atp5g1/Zfyve27/Dip2b/Golga4/Eif2b2/Iqgap1/Camk2d/Mecp2/Fubp1/Ctnnb1/Stk11/Dnm2/Arih2/Dbnl/Cyfip2/Apoe/G	32
Hspd1/Mef2c/Syk/Satb1/Irs2/Rc3h2/H2-Ab1/Rps3/Laptm5/Inpp5d/Malt1/Dhps/Rasal3/St6gal1/Bcl2/Itch/Siglecg/Cblb/Tnfrsf13c/Fkbp1a/Btla/Cd55/Cd86/Ccnd3/Dock8/Myd88/Il6st/Cd19/Ctnnb1/Ccr7/Dock2/Cd81/Peli1/M	36

Dnaja1/Foxp1/Ptges3/Rnf6/Nr3c1/Fkbp4/Parp1/Sirt1/Daxx/Ep300/Ddrgk1/Lbh/Cnot1/Arid1a/Per1/Ncoa2/Cnot2/Safb/Calr	19
Klf2/Hsp90ab1/Klf4/Zfp36/Smad7/Mul1/Atp2b1/Laptm5/Rel/Inpp5d/Rnf125/Crebbp/Nmi/Parp1/Sirt1/Cd84/Arrb2/Bank1/Nfkb1/Tspo/Cd2ap/Sars/C1qbp/Rabgef1/Ccr7/Cd200/Bst2/Irak3/Relb/Pml/Zc3h12a/Ptprc/Bcl6	33
Jun/Zfp36/Eif3f/Eif3a/Chd1/Sp100/Rsf1/Ep300/Ccnt2/Inpp5k/Snw1/Csde1/Sp1/Trim11/Nucks1	15
Gapdh/Zfp36/Rpl13a/Rc3h2/Rps3/Dhx36/Ddx6/Zfp36l2/Celf1/Bank1/Rock1/Eif2ak3/Tnrc6a/Bin1/Cnot1/Pan3/Cnot2/Rtn3/Ythdf3/Eif4e2/Larp1/Tnrc6c/Pml/Zc3h12a/Apoe/Calr/Tnrc6b	27
Jun/Hsp90aa1/Fos/Nfkbia/Mef2c/Foxp1/Zfp36/Gpr171/Smad7/Syk/Rc3h2/Pcid2/Clec2d/Inpp5d/Hspb1/Sart1/Klf10/Malt1/Myc/Ets1/Zfp36l2/Nfkbid/Ldb1/Sh2b3/Ptpn2/Hax1/Stat1/Pik3r1/Clptm1/Il4ra/Hspa9/Ctnnb1/Ccr7/F	40
Phf14/Arid5a/Atf2/Morf4l2/Naa50/Pbxip1/Crebbp/Irf4/Sirt1/Ldb1/Phf20/Ep300/Ogt/Mecp2/Iws1/Per1/Brd8/Epc1/Ep400/Flcn/Kmt2a/Phf20l1/Kat6a	23
Phf14/Arid5a/Atf2/Morf4l2/Naa50/Pbxip1/Crebbp/Irf4/Sirt1/Ldb1/Phf20/Ep300/Ogt/Mecp2/Iws1/Per1/Brd8/Epc1/Ep400/Flcn/Kmt2a/Phf20l1/Kat6a	23
Zfp36/Rc3h2/Pcid2/Dhx36/Lsm2/Pabpc4/Kynu/Ahcy/Zfp36l2/Celf1/Hnrnpa0/Eif3e/Smg5/Smg1/Npm1/Zc3hav1/Nt5c/Csde1/Taf15/Rock1/Casc3/Magoh/Tnrc6a/Cnot1/Pan3/Myd88/Sidt2/Cnot2/Ythdf3/Fus/Rnaset2a/Larp1/	41
Ppp1r15a/Eif1/Rpl13a/Eif3k/Ppp1r15b/Tmed2/Eif4g2/Npm1/Bank1/Eif2ak3/Larp1/Rps6kb1/Pml/Dnajc3	14
Zfp36/Rc3h2/Pcid2/Dhx36/Zfp36l2/Eif3e/Smg5/Smg1/Csde1/Casc3/Magoh/Tnrc6a/Cnot1/Pan3/Cnot2/Tnrc6c/Secisbp2/Zc3h12a/Tnrc6b	19
Jun/Zfp36/Atf3/Atf2/Pmaip1/Klf10/Hnrnpl/Bcl2/Sirt1/Foxo1/Pik3c3/Nfe2l2/Gabarap/Sik2/Prkag1/Pik3r4/Eif2ak3/Tnrc6a/Fnip1/Higd1a/Larp1/Wipi2/Map1lc3a/Flcn/Zc3h12a	25
Dnaja1/Foxp1/Zfp36/Akap13/Ptges3/Rnf6/Nr3c1/Fkbp4/Map2k1/Bcl2/Parp1/Sirt1/Zfp36l2/Tbl1x/Daxx/Ep300/Ddrgk1/Lbh/Cnot1/Arid1a/Per1/Ncoa2/Pik3r1/Cnot2/Rps6kb1/Crebrf/Safb/Calr	28
Hspd1/Mef2c/Syk/Satb1/Irs2/Rc3h2/H2-Ab1/Rps3/Laptm5/Inpp5d/Tcirg1/Malt1/Dhps/Rasal3/St6gal1/Bcl2/Itch/Siglecg/Cblb/Tnfrsf13c/Fkbp1a/Btla/Cd55/Cd86/Ccnd3/Dock8/Myd88/Il6st/Cd19/Ctnnb1/Ccr7/Dock2/Cd81/F	37
Hspa1a/Hspa1b/Ubb/Hsp90ab1/Rps7/Smad7/Gpx1/Laptm5/Arih1/Tmem259/Csnk1d/Xpo1/Csnk2a1/Gabarap/Ddrgk1/Smarcc1/Ogt/Usp7/Herpud1/Zfand2a/Dnajb2/Chfr/Pten/Arih2/Pml/Apoe/Usp19	27
Zfp36/Rc3h2/Pcid2/Dhx36/Lsm2/Pabpc4/Kynu/Ahcy/Zfp36l2/Celf1/Hnrnpa0/Eif3e/Ephx1/Smg5/Smg1/Npm1/Zc3hav1/Nt5c/Csde1/Taf15/Rock1/Casc3/Magoh/Tnrc6a/Cnot1/Pan3/Myd88/Sidt2/Cnot2/Ythdf3/Fus/Rnaset2a	42
Dnaja1/Hsph1/Gpx1/Plaur/Tpt1/Hspb1/Sgk3/Map2k1/Bcl2/Sirt1/Arrb2/Rb1cc1/Nfe2l2/Siah2/Eno1/Ghitm/Herpud1/Ivns1abp/Pttg1ip/Zmynd11/Birc6/Higd1a/Rnf34/Cdk11b/Ctnnb1/Fcmr/Rps6kb1/Phip/Ptpn1	29
Rpl38/Rpl35/Rpl24/Rpl14/Rpl35a/Rpl7a/Wdr74/Nifk/Npm1/Rrs1/Dhx30/Nhp2/Zfp622/Pes1/Ebna1bp2	15
Hspd1/Mef2c/Syk/Satb1/Irs2/Rc3h2/H2-Ab1/Rps3/Laptm5/Inpp5d/Malt1/Dhps/Rasal3/Bcl2/Itch/Siglecg/Cblb/Tnfrsf13c/Fkbp1a/Btla/Cd55/Cd86/Ccnd3/Dock8/Myd88/Il6st/Cd19/Ctnnb1/Ccr7/Dock2/Cd81/Peli1/Msn/Ptprc	35
Jun/Atf3/Atf2/Pmaip1/Klf10/Hnrnpl/Bcl2/Sirt1/Foxo1/Pik3c3/Nfe2l2/Gabarap/Sik2/Prkag1/Pik3r4/Eif2ak3/Tnrc6a/Fnip1/Higd1a/Wipi2/Map1lc3a/Flcn/Zc3h12a	23
Hspa1b/Nr4a1/Klf4/Gpx1/Plaur/Pcid2/Arl6ip1/Arrb2/Siah2/Csnk2a1/Akt2/Mical1/Herpud1/Fnip1/Rnf34/Sfn/Fcmr	17
Zfp36/Myo1c/Pcid2/Srsf7/Setd2/Rae1/Xpo1/Nup88/Nup210/Iws1/Casc3/Magoh/Poldip3/Eny2/Ranbp2/Akap8l/Pabpn1/Chtop/Nxf1	19
Hspa1b/Nr4a1/Klf4/Gpx1/Plaur/Arl6ip1/Arrb2/Siah2/Csnk2a1/Akt2/Mical1/Herpud1/Fnip1/Rnf34/Sfn/Fcmr	16
Hspa8/Zfp36/Rc3h2/Dazap1/Dhx36/Zfp36l2/Celf1/Snrnp70/Zc3hav1/Snw1/Rock1/Tnrc6a/Cnot1/Pan3/Cnot2/Ythdf3/Tnrc6c/Paf1/Zc3h12a/Tnrc6b	20
Dnajb1/Klf2/Jun/Ppp1r15a/Atf3/Cited2/Egr1/Arnt/Hif1an/Nfe2l2/Eif2ak3/Bclaf1/Atf6	13
Hspa8/Hsp90ab1/Nfkbia/Syk/Bach2/Atf2/Mbtps1/Nup50/Ipo7/Nup88/Prkag1/Kpnb1/Ppp3ca/Pttg1ip/Ing1/Pik3r1/Rbm22/Ppp2ca/Ranbp2/Tnpo1/Phip/Pml/Zc3h12a/Cse1l	24
Cyth1/P2ry10/Dennd4a/Ralgapa1/Akap13/Cdc42se2/Dgkz/Rasal3/Map2k1/Dynlt1c/Kras/Gabarap/Camk2d/Ogt/Arfgef1/Rasa2/Cd2ap/Arhgap25/Rabgef1/Dock2/Ralgps2/Dnm2/Gpr65/Flcn/Apoe/Bcl6/Arhgap26/Iqsec1	28
Kmt2e/Arid4b/Satb1/Crebbp/Setd2/Sirt1/Mtf2/Kmt2c/Ogt/Snw1/Mecp2/Ehmt1/Iws1/Atrx/Pax5/Rtf1/Ctnnb1/Paf1/Kmt2a/Nsd1/Chtop/Prmt1	22
Nfkbia/Klf4/Smad7/Id3/Sp100/Hes6/Sirt1/Itch/Nfkbid/Arrb2/Med13/Sri/Usp7/Tax1bp1/Cebpg/Bhlhe40/Faf1/Cd200/Irak3/Peli1/Chuk/Zc3h12a/Kat6a	23
Zfp36/Rc3h2/Pcid2/Dhx36/Lsm2/Pabpc4/Kynu/Ahcy/Zfp36l2/Celf1/Hnrnpa0/Nfe2l2/Eif3e/Smg5/Smg1/Npm1/Zc3hav1/Nt5c/Csde1/Taf15/Rock1/Casc3/Magoh/Tnrc6a/Cnot1/Pan3/Myd88/Sidt2/Cnot2/Ythdf3/Fus/Rnaset2a	43
Klf2/Rps24/Zfp36/Rps19/Kmt2e/Rps17/Inpp5d/Tmod3/Tcea1/Cited2/Ets1/Ldb1/Sh2b3/Ptpn2/Cebpg/Stat1/Ptbp3/Hspa9/Sp1/Bcl6/Dnase2a/Prmt1	22
Hspa1a/Hspa1b/Hsp90aa1/Smad7/Laptm5/Arih1/Tmem259/Csnk1d/Gabarap/Ddrgk1/Herpud1/Zfand2a/Dnajb2/Pttg1ip/Chfr/Pten/Faf1/Arih2/Cd81/Msn/Apoe	21
Sertad1/Macf1/Foxp1/Smad7/Mul1/Sbf1/Ifrd1/Rnf6/Adam10/Bcl11a/Nr3c1/Lamtor2/Bcl2/Sirt1/Eif4g2/Ist1/Ptprj/Atp5g1/Zfyve27/Dip2b/Golga4/Npm1/Csnk2a1/Eif2b2/Eno1/Mecp2/Dnajb2/Derl2/Mndal/Sfn/Stk11/Bst2/Dnm2	42
Hspd1/Mef2c/Foxp1/Syk/Ms4a1/Irs2/H2-Ab1/Pcid2/Laptm5/Inpp5d/Tcirg1/Sh3kbp1/Malt1/Bcl11a/Bcl2/Zfp36l2/Siglecg/Ptprj/Nfatc1/Ep300/Prkcb/Dclre1c/Bank1/Tnfrsf13c/Dnajb9/Ptpn2/Btla/Cebpg/Cd86/Fnip1/Pik3r1/Bln	41
Hspa8/Hspd1/Foxp1/Syk/Rps19/Arid5a/Laptm5/Malt1/Myo18a/Dhx36/Sirt1/Cd84/Nfkbid/H2-Q7/Ptprj/Fbxo38/Dennd1b/Rap1a/H2-M3/Dnajb9/Cd55/Stap1/Ankrd17/Cd86/H2-Q6/Il4ra/Rabgef1/Ccr7/H2-Q4/Cd81/Ptprc/H2-	32
Cenpa/Nap1l1/Senp6/Rnf4/Smarca5/Sart3/Rsf1/Daxx/Tcf4/Npm1/Gtf2a2/Cabin1/Anp32b/Atrx/Gtf2h5/Bdp1/Ubn1/Smarca2/Nap1l4/Nasp/Kat6a	21
Srsf7/Sap18/Acin1/Hnrnpl/Npm1/Cdk9/Ccnt1/C1qbp/Ptbp3/Srsf10	10
Dnajb1/Hspa8/Hspe1/Hspa1a/Hspa1b/Hsph1/Ptges3/St13/Hspa9/Hspa14	10
Hsp90aa1/Hsph1/Hspd1/Smad7/Syk/Itga4/H2-Ab1/Rps3/Plaur/Lims1/Sart1/Malt1/Cited2/Dhps/Rasal3/Ets1/Nfkbid/Ldb1/Ptprj/H2-Eb2/Plekha2/Tnfrsf13c/Jak1/Iqgap1/Cd55/Ppp3ca/Rock1/Nfat5/Cd86/C1qbp/Dock8/Il6st/I	43
Dynlt1c/Kras/Gabarap/Camk2d/Ogt/Elmo1/Rhog/Nisch/Dock2/Dnm2/Rhoh/Dbnl	12
Zfp36/Myo1c/Pcid2/Srsf7/Setd2/Rae1/Xpo1/Npm1/Nup88/Nup210/Iws1/Casc3/Magoh/Poldip3/Sidt2/Eny2/Ranbp2/Akap8l/Pabpn1/Chtop/Nxf1	21
Zfp36/Myo1c/Pcid2/Srsf7/Setd2/Rae1/Xpo1/Npm1/Nup88/Nup210/Iws1/Casc3/Magoh/Poldip3/Sidt2/Eny2/Ranbp2/Akap8l/Pabpn1/Chtop/Nxf1	21
Gapdh/Zfp36/Rpl13a/Rc3h2/Rps3/Dhx36/Ddx6/Zfp36l2/Celf1/Bank1/Rock1/Eif2ak3/Tnrc6a/Cnot1/Pan3/Cnot2/Ythdf3/Eif4e2/Larp1/Tnrc6c/Pml/Zc3h12a/Calr/Tnrc6b	24
Hsph1/Smad7/Syk/Rc3h2/H2-Ab1/Malt1/Rasal3/Itch/Nfkbid/Cblb/Btla/Cd55/Il4ra/Ccr7/Cd81/Zc3h12a/Ptprc/Bcl6	18
Ubb/Birc3/Rps2/Smad7/Laptm5/Nmi/Ube2d1/Cblb/Npm1/Birc2/Gabarap/Cdk9/Dnajb2/Pttg1ip/Chfr/Fbxo33/Pten/Mycbp2/Peli1	19
Hspa1b/Nr4a1/Gapdh/Hspd1/Klf4/Syk/Gpx1/Mul1/Rps3/Plaur/Pcid2/Laptm5/Pmaip1/Casp2/Pdcd2/Malt1/Myc/Sirt1/Arl6ip1/Arrb2/Fis1/Siah2/Csnk2a1/Ddrgk1/Akt2/Mical1/Herpud1/Anp32b/Eif2ak3/Bin1/Fnip1/Stat1/Birc6/	41
Chchd2/Atf2/Nfatc3/Limd1/Cited2/Egr1/Map2k1/Myc/Bcl2/Arnt/Cr1l/Sirt1/Hif1an/Nfe2l2/Ep300/Prkcb/Birc2/Eno1/Camk2d/Ogt/Mecp2/Pgk1/Higd1a/Pten/Pml/Usp19	26
Dnaja1/Foxp1/Akap13/Ptges3/Rnf6/Nr3c1/Fkbp4/Parp1/Sirt1/Daxx/Ep300/Ddrgk1/Lbh/Cnot1/Arid1a/Per1/Ncoa2/Cnot2/Prcp/Crebrf/Safb/Calr	22
Nr4a1/Fos/Mef2c/Cfl2/Mbnl1/Foxp1/Naca/Mef2d/Gpx1/Atf3/Tsc22d3/Nfatc3/Cited2/Egr1/Gpcpd1/Myc/Bcl2/Ep300/Ccnt2/Snw1/Ppp3ca/Pax5/Ctnnb1/Rps6kb1/Usp19	25
Sirt1/Ptprj/Prkcb/Lmbrd1/Sik2/Inpp5k/Ogt/Ptpn2/Pik3r1/Ccnd3/Rps6kb1/Nucks1/Socs3/Osbpl8/Ptpn1	15
Hspa8/Hsp90ab1/Hsp90aa1/Nfkbia/Hspd1/Syk/Atf2/Mbtps1/Nup50/Ipo7/Nup88/Kpnb1/Ppp3ca/Pex7/Pttg1ip/Ing1/Pik3r1/Rbm22/Ppp2ca/Ranbp2/Tnpo1/Phip/Pml/Zc3h12a/Apoe/Hspa4/Cse1l	27
Jun/Zfp36/Chd1/Sp100/Rsf1/Ep300/Ccnt2/Inpp5k/Snw1/Sp1/Trim11/Nucks1	12
Dnajb1/Klf2/Jun/Ppp1r15a/Atf3/Cited2/Egr1/Arnt/Hif1an/Nfe2l2/Eif2ak3/Atf6	12
Zfp36/Limd1/Map2k1/Ddx6/Mecp2/Nfkb1/Ppp3ca/Eif4e2/Tnrc6c/Zc3h12a/Pum2/Tnrc6b	12
Hsp90aa1/Gapdh/Hspd1/Syk/G3bp1/Dhx36/Setd2/Zbtb20/Zc3hav1/Ifnar1/Stat1/Myd88/Riok3/Chuk	14
Hspa1a/Hspa1b/Hsp90ab1/Rps7/Smad7/Laptm5/Arih1/Csnk1d/Xpo1/Csnk2a1/Gabarap/Ddrgk1/Smarcc1/Ogt/Usp7/Herpud1/Zfand2a/Dnajb2/Chfr/Pten/Arih2/Pml	22
Jun/Dnaja1/Ppp1r15a/Smad7/Dusp1/Hspb1/Ppp1r15b/Gadd45b/Hipk3/Sirt1/Tmed2/Arrb2/Ptprj/Cblb/Sh2b3/Npm1/Mical1/Inpp5k/Ogt/Ptpn2/Ube2b/Gadd45g/Stk38/Rabgef1/Pten/Ppp2ca/Irak3/Zc3h12a/Socs3/Ptprc/Dnaj	35
Dnaja1/Wnk1/Rps7/Zfp36/Smad7/Dusp1/Irs2/Hspb1/Prdx3/Gadd45b/Hipk3/Sirt1/Ptprj/Cblb/Sh2b3/Npm1/Pik3ip1/Inpp5k/Ptpn2/Gadd45g/Stk38/Pten/Ppp2r5a/Irak3/Pml/Ptprc/Dnajc3/Apoe/Ptpn1	29
Dnaja1/Hsph1/Klf4/Atf3/Dusp1/Dusp5/Eif3a/Hipk3/Itch/Foxo1/Ptprj/Sh2b3/Inpp5k/Ptpn2/Stk38/Per1/Zmynd11/Dusp16/Pten/Irak3/Flcn/Ptprc/Apoe/Ptpn1	24
Zfp36/Myo1c/Pcid2/Srsf7/Setd2/Rae1/Xpo1/Npm1/Nup88/Nup210/Iws1/Casc3/Magoh/Poldip3/Sidt2/Eny2/Ranbp2/Akap8l/Pabpn1/Chtop/Nxf1	21
Jun/Fos/Atf3/Itga4/Atf2/Pmaip1/Klf10/Hnrnpl/Bcl2/Sirt1/Foxo1/Pik3c3/Nfe2l2/Gabarap/Sik2/Prkag1/Snw1/Pik3r4/Eif2ak3/Tnrc6a/Fnip1/Higd1a/Wipi2/Map1lc3a/Flcn/Zc3h12a/Ptprc	27
Hsp90ab1/Hsp90aa1/Gapdh/Hspd1/Ifi30/Rps7/Smad7/Mul1/Ptges3/Tbl1x/Usp36/Ep300/Npm1/Ube2b/Usp7/Naa15/Pik3r1/Pten/Golga7/Pml/Rab3gap1/Usp19/Calr/Hypk	24
Hsp90aa1/Hspd1/Foxp1/Syk/Mul1/Rps19/Clec2d/Rnf125/Matr3/Nmi/Itch/Cd84/Arrb2/Fbxo38/Tspan32/H2-M3/Zc3hav1/Ptpn2/Cd55/Pspc1/Ankrd17/C1qbp/Stat1/Mndal/Ythdf3/Usp15/Trim12a/Irak3/Pml/Riok3/Zc3h12a/Pu	34
Kdm5a/Id3/Crem/Egr1/Sirt1/Csnk1d/Ogt/Per1/Ncoa2/Bhlhe40/Mycbp2/Relb/Pml/Kmt2a	14
Smad7/Gpx1/Mul1/Rps19/Rc3h2/Clec2d/Inpp5d/Rnf125/Nmi/Cr1l/Itch/Cd84/Arrb2/Ptpn2/Cd55/C1qbp/Ythdf3/Il4ra/Usp15/Rabgef1/Irak3/Riok3/Zc3h12a/Ptprc/Bcl6	25
Hspa8/Cfl2/Nsf/Mul1/Capg/Sptbn1/Tmod3/Pbxip1/Atg12/Acin1/Myc/Dedd2/Ets1/Rb1cc1/Smarce1/Snapin/Fis1/Gabarap/Ddrgk1/Smarcc1/Mical1/Cebpg/Tspo/Plek/Arid1a/Pik3r1/Uvrag/Flii/Wipi2/Map1lc3a/Irak3/Vrk1/Scaf	38
Ccdc47/Ppp1r15b/Erlin1/Tmed2/Nfe2l2/Ptpn2/Eif2ak3/Tmco1/Atf6/Bcl2l11/Ptpn1	11
Nap1l1/Ubtf/Smarca5/Sart3/Rsf1/Sirt1/Daxx/Npm1/Cabin1/Anp32b/Atrx/Ubn1/Smarca2/Paf1/Nap1l4/Nasp/Kat6a	17
Hsp90ab1/Hsp90aa1/Hsph1/Syk/Akap13/Traf4/Rps3/Zfp91/Zeb2/Dgkz/Malt1/Egr1/Map2k1/Cab39/Sirt1/Arrb2/Daxx/Kras/Cdc25b/Npm1/Rap1a/Iqgap1/Pik3r4/Stap1/Map4k2/Zfp622/Cd86/Higd1a/Ccnd3/Cd19/Ccr7/Stk11	40
Syk/Satb1/Rc3h2/H2-Ab1/Rps3/Laptm5/Malt1/Dhps/Rasal3/Itch/Cblb/Tnfrsf13c/Fkbp1a/Btla/Cd55/Cd86/Ccnd3/Dock8/Il6st/Ctnnb1/Ccr7/Dock2/Cd81/Peli1/Msn/Ptprc	26
Nr4a1/Fos/Mef2c/Cfl2/Cacybp/Mbnl1/Foxp1/Naca/Smad7/Mef2d/Gpx1/Akap13/Atf3/Tsc22d3/Nfatc3/S1pr1/Ifrd1/Kdm6b/Cited2/Egr1/Nr3c1/Gpcpd1/Myc/Bcl2/Csrp1/Sirt1/Arrb2/Ep300/Ccnt2/Fkbp1a/Camk2d/Snw1/Ube4	43
Foxp1/Smad7/Rc3h2/Malt1/Irf4/Nfkbid/H2-M3/Gadd45g/Ly9/Il4ra/Ccr7/Relb/Zc3h12a/Bcl6	14
Hsp90aa1/Atf2/Rnf125/Crebbp/Wdfy4/Zc3hav1/Nfkb1/Ankrd17/C1qbp/Usp15/Riok3/Chuk/Zc3h12a/Pum2	14
Chchd2/Atf2/Cited2/Egr1/Map2k1/Myc/Bcl2/Cr1l/Sirt1/Hif1an/Nfe2l2/Eno1/Ogt/Pgk1/Higd1a/Pten/Usp19	17
Ppp1r15a/Ppp1r15b/Tmed2/Npm1/Eif2ak3/Pml/Dnajc3	7
Hsp90ab1/Rps7/Zfp36/Azin1/Il10ra/Herc1/Dhx36/Snx12/Bcl2/Eif4g2/Hnrnpa0/Usp36/Pde3b/Smg1/Csnk2a1/Ddrgk1/Ctsa/Smarcc1/Ogt/Usp7/Taf15/Tspo/Myd88/Fus/Dbi/Larp1/Secisbp2/Irak3/Mycbp2/Pml/Usp19/Larp4b	32
Zfp36/Limd1/Map2k1/Ddx6/Mecp2/Nfkb1/Ppp3ca/Eif4e2/Tnrc6c/Zc3h12a/Pum2/Tnrc6b	12
Smad7/Rc3h2/H2-Ab1/Pag1/Laptm5/Tnfaip8l2/Inpp5d/Hspb1/Itch/Cd84/Nfkbid/Cblb/Sh2b3/Bank1/Tspan32/Ptpn2/Btla/Cd86/Il4ra/Rabgef1/Cnr2/Cd200/Peli1/Zc3h12a/Apoe/Bcl6	26
Rps5/Rpsa/Rpl38/Rps27/Rps2/Rps19/Rpl24/Rps28/Abt1/Rrs1/Dhx30/Mrps7/C1qbp	13
Klf2/Pde4b/Rps24/Zfp36/Rps19/Kmt2e/Rps17/Inpp5d/Tmod3/Tcea1/Cited2/Ets1/Ldb1/Sh2b3/Ptpn2/Cebpg/Stat1/Ptbp3/Hspa9/Sp1/Bcl2l11/Bcl6/Dnase2a/Prmt1	24
Nfkbia/Birc3/Zfp36/Syk/Rps3/Laptm5/Lims1/Adam10/Sirt1/Zfp36l2/Traf1/Nfe2l2/Ikbkb/Birc2/Tnfrsf13c/Inpp5k/Ptpn2/Nfkb1/Stat1/Rps6kb1/Zfand6/Chuk/Zc3h12a/Prpf8	24
Klf2/Zfp36/Rps19/Kmt2e/Inpp5d/Tmod3/Tcea1/Cited2/Ets1/Ldb1/Sh2b3/Ptpn2/Cebpg/Stat1/Ptbp3/Hspa9/Sp1/Bcl6/Dnase2a/Prmt1	20
Nr4a1/Fos/Mef2c/Cfl2/Mbnl1/Foxp1/Naca/Smad7/Mef2d/Gpx1/Id3/Atf3/Tsc22d3/Nfatc3/S1pr1/Cited2/Egr1/Gpcpd1/Myc/Bcl2/Sirt1/Ep300/Ccnt2/Fkbp1a/Chkb/Snw1/Ube4b/Ppp3ca/Pax5/Mtss1/Ctnnb1/Rps6kb1/Chd2/Cl	35
Macf1/Vps37b/Naca/Myo1c/Zdhhc3/Exoc4/Zdhhc18/Arl6ip1/Fis1/Akt2/Inpp5k/Ogt/Srpr/Rabgef1/Tram1/Golga7/Sec63/Gdi1	18
Ppp1r15a/Wnk1/Smad7/Atf2/Clk1/Map2k1/Cab39/Bcl2/Hipk3/Prkcb/Csnk2a1/Camk2d/Inpp5k/Ogt/Rock1/Sbk1/Vrk1/Phip	18
Ubb/Rps7/Atf3/Rps3/Pmaip1/Casp2/Myc/Dedd2/Sirt1/Daxx/Fis1/Ptpn2/Eif2ak3/Trp53inp1/Pten/Faf1/Bclaf1/Flcn/Pml/Bcl2l11/Ptprc	21
Zfp36/Limd1/Map2k1/Ddx6/Mecp2/Nfkb1/Ppp3ca/Eif4e2/Tnrc6c/Zc3h12a/Pum2/Tnrc6b	12
Gapdh/Atf3/Irs2/Ptges3/B3gnt7/Nr3c1/Sirt1/Chst12/Foxo1/Ep300/Got1/Akt2/Inpp5k/Ogt/Ptpn2/Usp7/Nfkb1/Plek/B3gnt2/Supt20/Pgk1/Cltc/Stk11/Ranbp2	24
Kmt2e/Sirt1/Mtf2/Ogt/Snw1/Mecp2/Iws1/Atrx/Pax5/Rtf1/Ctnnb1/Paf1/Kmt2a/Nsd1/Chtop	15
Nr4a1/Fos/Mef2c/Cfl2/Mbnl1/Foxp1/Naca/Mef2d/Gpx1/Atf3/Tsc22d3/Nfatc3/Cited2/Egr1/Gpcpd1/Myc/Bcl2/Ep300/Ccnt2/Snw1/Ppp3ca/Pax5/Ctnnb1/Rps6kb1/Usp19	25
Nr4a1/Fos/Mef2c/Cfl2/Cacybp/Mbnl1/Foxp1/Naca/Smad7/Mef2d/Gpx1/Akap13/Atf3/Tsc22d3/Nfatc3/S1pr1/Ifrd1/Kdm6b/Cited2/Egr1/Nr3c1/Gpcpd1/Myc/Bcl2/Sirt1/Arrb2/Ep300/Ccnt2/Fkbp1a/Camk2d/Snw1/Ube4b/Ppp3	41
Macf1/Nsf/Sptbn1/Zdhhc3/Tmed2/Zfyve27/Ikbkb/Vamp3/Golga4/Bsg/Csnk2a1/Tspan32/Rap1a/Camk2d/Akt2/Inpp5k/Pgrmc1/Rock1/Vamp2/Rer1/Rhog/Atp2c1/Pik3r1/Cltc/Ppp2r5a/Golga7/Vti1b/Cd81/Sys1/Myadm/Rapge	31
Jun/Atf3/Atf2/Pmaip1/Klf10/Hnrnpl/Bcl2/Sirt1/Foxo1/Pik3c3/Nfe2l2/Gabarap/Sik2/Prkag1/Snw1/Pik3r4/Eif2ak3/Tnrc6a/Fnip1/Higd1a/Wipi2/Map1lc3a/Flcn/Zc3h12a	24
Dnajb1/Hspa8/Hspe1/Hspa1a/Hspa1b/Hsph1/Ptges3/St13/Hspa9/Hspa14	10
Dnajb1/Hspa8/Hspe1/Hspa1a/Hspa1b/Hsph1/Ptges3/St13/Hspa9/Hspa14	10
Jun/Dnaja1/Fos/Chchd2/Hsph1/Rhob/Foxp1/Lrrc8c/Gpx1/Atf2/Rps3/Hspb1/Prdx3/Kdm6b/Aldh2/Bcl2/Arnt/Parp1/Sirt1/Slc12a6/Foxo1/Nfe2l2/Zfp622/Rcsd1/Trp53inp1/Oser1/Ctnnb1/Psap/Setx/Relb/Pml/Chuk/Zc3h12a	33
Hspa8/Hsp90ab1/Vps37b/Trim25/Top2b/Rab43/Pcbp1/Trim59/Vapa/Bcl2/Ddx6/Arl8b/Ist1/Bsg/Zc3hav1/Inpp5k/Atg16l2/Fam111a/Uvrag/Larp1/Bst2/Trim12a/Pml/Trim11/Zc3h12a/Nucks1/Apoe/Srpk2	28
Ifngr1/Dhx36/Hnrnpl/Nfe2l2/Eif3e/Npm1/Eif2ak3/C1qbp/Poldip3/Ythdf3/Larp1/Rps6kb1/Usp16/Secisbp2/Sp1/Dnajc3/Apoe/Larp4/Larp4b/Vim/Eif2s3x	21
Jun/Hsp90ab1/Klf4/Itga4/Plaur/Pbxip1/Sp100/Irf4/Myc/Hnrnpl/Hipk3/Rsf1/Parp1/Sirt1/Arrb2/Eif3e/Krit1/Ep300/Snapin/Cblb/Ddrgk1/Eno1/Sri/Cdk9/Mecp2/Ppp3ca/Rock1/Epb41/Eif2ak3/Dnajb2/Ctnnb1/Ppp2ca/Btaf1/Nsd1	37
Hsp90ab1/Dnaja1/Syk/Akap13/Dusp1/Rps3/Zeb2/Map2k1/Cab39/Hipk3/Sirt1/Ptprj/Kras/Sh2b3/Iqgap1/Inpp5k/Prkag1/Cnppd1/Stk38/Map4k2/Higd1a/Ccnd3/Uvrag/Sfn/Pten/Ccr7/Stk11/Ppp2ca/Irak3/Ccnl1/Ptprc/Apoe/Pt	34
Zfp36/Rc3h2/Dhx36/Zfp36l2/Celf1/Rock1/Tnrc6a/Cnot1/Pan3/Cnot2/Ythdf3/Tnrc6c/Zc3h12a/Zc3h18/Tnrc6b	15
Zfp36/Rc3h2/Dhx36/Zfp36l2/Celf1/Zc3hav1/Rock1/Tnrc6a/Cnot1/Pan3/Cnot2/Ythdf3/Tnrc6c/Zc3h12a/Tnrc6b	15
Smad7/Rc3h2/H2-Ab1/Pag1/Laptm5/Tnfaip8l2/Inpp5d/Hspb1/Itch/Cd84/Nfkbid/Cblb/Bank1/Tspan32/Ptpn2/Btla/Cd86/Il4ra/Rabgef1/Cnr2/Cd200/Peli1/Zc3h12a/Bcl6	24
Cyth1/P2ry10/Dennd4a/Akap13/Cdc42se2/Dgkz/Rasal3/Map2k1/Dynlt1c/Kras/Gabarap/Camk2d/Ogt/Arfgef1/Rasa2/Rabgef1/Dock2/Ralgps2/Dnm2/Gpr65/Flcn/Apoe/Bcl6/Iqsec1	24
Mbnl1/Sf3b1/Isy1/Rbm5/Scaf11/Celf1/Celf2/Luc7l2/Srsf10/Setx/Srpk2	11
Hsp90aa1/Hsph1/Hspd1/Smad7/Syk/Itga4/H2-Ab1/Rps3/Plaur/Sart1/Malt1/Cited2/Dhps/Rasal3/Ets1/Nfkbid/H2-Eb2/Tnfrsf13c/Jak1/Cd55/Nfat5/Cd86/Dock8/Il6st/Il4ra/Ccr7/Rhoh/Cd81/Ptprc/Bcl6	30
Pde4b/Wnk1/Rc3h2/Rps3/Laptm5/Dgkz/Malt1/Elf1/Nfkbid/Ptprj/Cblb/Eif2b2/Dennd1b/Ptpn2/Ccr7/Stk11/Cd81/Zc3h12a/Ptprc	19
Zfp36/Myo1c/Pcid2/Srsf7/Dhx36/Setd2/Rae1/Xpo1/Npm1/Nup88/Nup210/Iws1/Casc3/Magoh/Poldip3/Sidt2/Eny2/Ranbp2/Akap8l/Pabpn1/Chtop/Nxf1	22
Vps37b/Trim25/Zfp36/Top2b/Crebbp/Sp100/Trim59/Vapa/Bcl2/Rsf1/Dynlt1c/Bsg/Zc3hav1/Inpp5k/Fam111a/Stat1/Larp1/Bst2/Trim12a/Pml/Trim11/Srpk2	22
Nfkbia/Birc3/Zfp36/Syk/Rps3/Laptm5/Lims1/Sirt1/Zfp36l2/Traf1/Nfe2l2/Ikbkb/Birc2/Tnfrsf13c/Inpp5k/Ptpn2/Nfkb1/Stat1/Zfand6/Chuk/Zc3h12a/Prpf8	22
Hsp90ab1/Rps7/Zfp36/Il10ra/Herc1/Dhx36/Bcl2/Eif4g2/Hnrnpa0/Usp36/Smg1/Csnk2a1/Ddrgk1/Ctsa/Smarcc1/Ogt/Usp7/Taf15/Tspo/Myd88/Fus/Dbi/Larp1/Secisbp2/Pml/Usp19/Larp4b	27
Sertad1/Pcid2/Nr3c1/Setd2/Map2k1/Ldb1/Wdr43/Zmynd11/Rtf1/Ctnnb1/Paf1/Supt16/Zmiz2	13
Hsp90ab1/Ppp1r15a/Macf1/Cfl2/Ssh2/Capg/Myo1c/Sptbn1/Tmod3/Ano6/Ifrd1/Rnf6/Anxa7/Bcl11a/Arpc5/Slc12a6/Ist1/Rb1cc1/Atp5g1/Zfyve27/Dip2b/Fchsd2/Golga4/Npm1/Eif2b2/Ogt/Arfgef1/Plek/Hax1/Bin1/Flii/Pten/Dnr	38
Ppp1r15a/H13/Casp2/Naa35/Pmpcb/Tmem208/Gm16286/Naa50/Adam10/Crebbp/Cpm/Dhps/Naa10/Myc/Snx12/Sec11c/Parp1/Pik3c3/Ep300/Snapin/Dnajb11/Tspan32/Eno1/Ogt/Naa15/Isca1/Pgk1/Fam111a/Sde2/Fcmr/C	32
Macf1/Nsf/Sptbn1/Zdhhc3/Adam10/Map2k1/Tmed2/Zfyve27/Ikbkb/Vamp3/Golga4/Bsg/Csnk2a1/Tspan32/Rap1a/Camk2d/Akt2/Inpp5k/Pgrmc1/Rock1/Epb41/Vamp2/Rer1/Rhog/Atp2c1/Pik3r1/Cltc/Ppp2r5a/Golga7/Vti1b/C	35
Hspa8/Hsp90ab1/Nfkbia/Syk/Atf2/Mbtps1/Nup50/Ipo7/Nup88/Kpnb1/Ppp3ca/Pttg1ip/Ing1/Pik3r1/Rbm22/Ppp2ca/Ranbp2/Tnpo1/Phip/Pml/Zc3h12a/Cse1l	22
Atf2/Morf4l2/Irf4/Sirt1/Phf20/Ep300/Ogt/Iws1/Per1/Brd8/Epc1/Ep400/Kmt2a	13
Hsp90ab1/Hsp90aa1/Klf4/Dusp1/Ptges3/Rps3/Smchd1/Dhx36/Setd2/Myc/Sub1/Parp1/Sirt1/Arrb2/Smg5/Smg1/Npm1/Camk2d/Pcna/Ube2b/Usp7/Cdk9/Mecp2/Cebpg/Atrx/Fus/Tnks2/Ctnnb1/Flcn/Trip12/Pml/Kmt2a/Nuck	36
Hspa1a/Hsp90ab1/Hsp90aa1/Ptges3/Dhx36/Sp100/Myc/Parp1/Dclre1c/Smg5/Smg1/Pcna/Nhp2/Atrx/Tnks2/Ctnnb1/Pml/Gnl3	18
Kmt2e/Sirt1/Mtf2/Ogt/Snw1/Mecp2/Rtf1/Ctnnb1/Paf1/Kmt2a/Chtop	11
Hspa8/Hspd1/Mef2c/Smad7/Arid5a/Rc3h2/H2-Ab1/Malt1/Cr1l/Nfkbid/H2-Q7/Fbxo38/Tnfrsf13c/Dennd1b/H2-M3/Cd55/H2-Q6/Il4ra/Ccr7/H2-Q4/Cd81/Zc3h12a/Ptprc/H2-T23/Bcl6	25
Hsp90ab1/Hsp90aa1/Hsph1/Syk/Akap13/Traf4/Rps3/Zfp91/Zeb2/Malt1/Egr1/Map2k1/Cab39/Sirt1/Arrb2/Daxx/Kras/Cdc25b/Npm1/Rap1a/Iqgap1/Stap1/Map4k2/Cd86/Higd1a/Ccnd3/Ccr7/Stk11/Ppp2ca/Ptprc/Nedd9/Osb	34
Atad1/Smad7/Dynll1/Pcid2/Zdhhc3/Adam10/Fkbp4/Setd2/Map2k1/Rae1/Xpo1/Snapin/Npm1/Rap1a/Nup88/Rrs1/Iws1/Pcm1/Rcc2/Vamp2/Poldip3/Eny2/Akap9/Akap8l/Pabpn1/Chtop/Nxf1/Iqsec1	28
Hspa8/Jun/Hsp90ab1/Gapdh/Hspd1/Vps37b/Trim25/Gpx1/Chd1/Trim59/Vapa/Arl8b/Dynlt1c/Ist1/Ep300/Bsg/Inpp5k/Snw1/Myd88/Uvrag/Trim12a/Sp1/Pml/Trim11/Zc3h12a/Nucks1/Bcl2l11/Apoe	28
Pcid2/Setd2/Rae1/Xpo1/Npm1/Nup88/Rrs1/Iws1/Poldip3/Eny2/Akap8l/Pabpn1/Chtop/Nxf1	14
Gapdh/Actg1/Rpl13a/Capg/Myo1c/H2-Ab1/Rab43/Myo18a/Sp100/Nmi/Kynu/H2-Q7/Vamp3/Ptpn2/Stat1/Bst2/Rps6kb1/Zyx/Vim	19
Hspa8/Hspd1/Mef2c/Smad7/Arid5a/Rc3h2/H2-Ab1/Malt1/Cr1l/Sirt1/Nfkbid/H2-Q7/Fbxo38/Tnfrsf13c/Dennd1b/H2-M3/Cd55/H2-Q6/Il4ra/Ccr7/H2-Q4/Cd81/Zc3h12a/Ptprc/H2-T23/Bcl6	26
Srsf7/Sap18/Acin1/Hnrnpl/Npm1/C1qbp/Ptbp3/Srsf10	8
Hsp90ab1/Hsp90aa1/Klf4/Ptges3/Rps3/Smchd1/Dhx36/Myc/Parp1/Sirt1/Arrb2/Npm1/Camk2d/Pcna/Ube2b/Usp7/Mecp2/Cebpg/Atrx/Fus/Tnks2/Ctnnb1/Pml/Ptprc/Gnl3	25
Ppp1r15a/Wnk1/Smad7/Atf2/Clk1/Map2k1/Cab39/Bcl2/Hipk3/Prkcb/Csnk2a1/Camk2d/Inpp5k/Ogt/Rock1/Sbk1/Vrk1/Phip	18
Hspd1/Syk/Mul1/Rps3/Laptm5/Pmaip1/Casp2/Pdcd2/Malt1/Myc/Sirt1/Fis1/Anp32b/Eif2ak3/Stat1/Ppp2ca/Pml/Bcl2l11	18
Foxp1/Smad7/Rc3h2/Malt1/Irf4/Nfkbid/Gadd45g/Ly9/Il4ra/Ccr7/Relb/Zc3h12a/Bcl6	13
Ppp1r15a/Macf1/Vapa/Os9/Ankrd13c/Ddrgk1/Herpud1/Zfand2a/Rer1/Srpr/Tram1/Sec63/Rab3gap1	13
Kmt2e/Arid4b/Satb1/Crebbp/Snrpd3/Setd2/Sirt1/Mtf2/Kmt2c/Ogt/Snw1/Mecp2/Ehmt1/Iws1/Atrx/Pax5/Rtf1/Ctnnb1/Paf1/Kmt2a/Nsd1/Chtop/Prmt1	23
Kmt2e/Arid4b/Satb1/Crebbp/Snrpd3/Setd2/Sirt1/Mtf2/Kmt2c/Ogt/Snw1/Mecp2/Ehmt1/Iws1/Atrx/Pax5/Rtf1/Ctnnb1/Paf1/Kmt2a/Nsd1/Chtop/Prmt1	23
Hsp90ab1/Ppp1r15a/Mef2c/Ssh2/Dusp1/Dusp5/Inpp5d/Bod1l/Ppp1r15b/Ssu72/Bcl2/Ptprj/Ikbkb/Smg5/Cdc25b/Iqgap1/Inpp5k/Ptpn2/Nt5c/Ppp3ca/Rock1/Plek/Ppp2r2a/Dusp16/Ptp4a2/Pten/Ppp2r5a/Stk11/Ppm1g/Ppp2c	33
Foxp1/Smad7/Satb1/Rc3h2/Tcirg1/Malt1/Irf4/Nfkbid/Gadd45g/Cd55/Ly9/Il4ra/Ccr7/Cd81/Relb/Zc3h12a/Bcl6	17
Nfkbia/Klf4/Sirt1/Itch/Nfkbid/Arrb2/Usp7/Tax1bp1/Faf1/Cd200/Irak3/Peli1/Chuk/Zc3h12a	14
Pcid2/Setd2/Rae1/Xpo1/Npm1/Nup88/Rrs1/Iws1/Poldip3/Eny2/Akap8l/Pabpn1/Chtop/Nxf1	14
Jun/Fos/Mef2c/Foxp1/Junb/Clec2d/Inpp5d/Klf10/Tcirg1/Cited2/Sbno2/Irf4/Myc/Sirt1/Nfatc1/Nrros/Ptpn2/Hax1/Pik3r1/Ctnnb1/Ccr7/Cd81/Relb/Lbr/Chuk/Tob2	26

Gpx1/Atf3/Pmaip1/Casp2/Zdhhc3/Sgk3/Sp100/Bcl2/Dedd2/Rb1cc1/Traf1/Siah2/Gabarap/Zmynd11/Birc6/Ddx47/Pik3r1/Rnf34/Pten/Fcmr/Faf1/Rps6kb1/Phip/Pml/Bcl2l11/Ptprc	26
Jun/Cacybp/Id3/Pds5a/Orc3/Bod1l/Pold4/Polg/Bcar3/Topbp1/Npm1/Rbms1/Pcna/Cdk9/Atrx/Rfc1/Ankrd17/Fam111a/Sde2/Tonsl/Faf1/Nasp/Supt16/Nucks1/Donson/Esco1/Srpk2	27
Hspa1a/Hspa1b/Ubb/Hsp90ab1/Smad7/Gpx1/Arih1/Tmem259/Csnk1d/Xpo1/Gabarap/Ddrgk1/Smarcc1/Ogt/Usp7/Herpud1/Zfand2a/Dnajb2/Chfr/Arih2/Apoe/Usp19	22
Zfp36/Lrrc8c/Myo1c/Pcid2/Srsf7/Setd2/Rae1/Xpo1/Npm1/Nup88/Nup210/Iws1/Casc3/Magoh/Slc25a51/Poldip3/Sidt2/Eny2/Ranbp2/Akap8l/Pabpn1/Chtop/Nxf1	23
Hsp90ab1/Zeb2/Cited2/Crebbp/Ep300/Got1/Snw1/Stk11/Flcn	9
Hsp90ab1/Zeb2/Cited2/Crebbp/Ep300/Got1/Snw1/Stk11/Flcn	9
Ppp1r15a/Ppp1r15b/Tmed2/Npm1/Eif2ak3/Dnajc3	6
Foxp1/Smad7/Rc3h2/Malt1/Irf4/Nfkbid/Gadd45g/Ly9/Il4ra/Ccr7/Relb/Zc3h12a/Bcl6	13
Hspa8/Hspd1/Arid5a/Malt1/H2-Q7/Fbxo38/Dennd1b/H2-M3/Cd55/H2-Q6/H2-Q4/Cd81/Ptprc/H2-T23	14
Nap1l1/Acin1/Ubtf/Smarca5/Sart3/Rsf1/Sirt1/Daxx/Npm1/Smarcc1/Cabin1/Anp32b/Atrx/Ubn1/Smarca2/Paf1/Nap1l4/Akap8l/Nasp/Kat6a	20
Foxp1/Smad7/Syk/Satb1/Rc3h2/Atf2/Malt1/Irf4/Bcl2/Nfkbid/Gadd45g/Ly9/Il4ra/Psap/Ccr7/Relb/Zc3h12a/Bcl6	18
Hsp90aa1/Hsph1/Hspd1/Syk/Itga4/H2-Ab1/Rps3/Sart1/Malt1/Dhps/Rasal3/Ets1/Nfkbid/H2-Eb2/Tnfrsf13c/Cd55/Nfat5/Cd86/Dock8/Il6st/Il4ra/Ccr7/Rhoh/Cd81/Ptprc/Bcl6	26
Naa35/Gm16286/Naa50/Crebbp/Naa10/Ep300/Naa15	7
Dnaja1/Wnk1/Dusp1/Irs2/Hspb1/Prdx3/Gadd45b/Hipk3/Sirt1/Ptprj/Cblb/Sh2b3/Npm1/Pik3ip1/Inpp5k/Ptpn2/Gadd45g/Stk38/Pten/Ppp2r5a/Irak3/Ptprc/Dnajc3/Apoe/Ptpn1	25
Jun/Fos/Chchd2/Hsph1/Rhob/Foxp1/Gpx1/Atf2/Rps3/Hspb1/Prdx3/Kdm6b/Aldh2/Bcl2/Arnt/Parp1/Sirt1/Foxo1/Nfe2l2/Zfp622/Trp53inp1/Oser1/Ctnnb1/Psap/Setx/Pml/Chuk/Zc3h12a	28
Chchd2/Atf2/Nfatc3/Limd1/Cited2/Egr1/Map2k1/Myc/Bcl2/Arnt/Cr1l/Sirt1/Hif1an/Nfe2l2/Ep300/Prkcb/Birc2/Eno1/Camk2d/Ogt/Mecp2/Pgk1/Higd1a/Pten/Pml/Usp19	26
Hspa1a/Hsp90ab1/Hsp90aa1/Ptges3/Dhx36/Sp100/Myc/Parp1/Dclre1c/Smg5/Smg1/Pcna/Nhp2/Atrx/Tnks2/Ctnnb1/Pml/Gnl3	18
Foxp1/Smad7/Rc3h2/Malt1/Irf4/Nfkbid/Gadd45g/Ly9/Il4ra/Ccr7/Relb/Zc3h12a/Bcl6	13
Nap1l1/Smarca5/Sart3/Rsf1/Daxx/Npm1/Cabin1/Anp32b/Atrx/Ubn1/Smarca2/Nap1l4/Nasp/Kat6a	14
Zfp36/Rc3h2/Dhx36/Zfp36l2/Celf1/Rock1/Tnrc6a/Cnot1/Pan3/Cnot2/Ythdf3/Tnrc6c/Zc3h12a/Tnrc6b	14
Nfkbia/Fth1/Ftl1/Sp100/Diaph1/Aup1/Os9/Ankrd13c/Sirt1/Chchd10/Nrros/Fkbp1a/Camk2d/Sri/Ptpn2/Tspo/Rer1/Vps13a/Tnrc6a/Uvrag/Cd19/Dbi/Akap9/Faf1/Pml/Zc3h12a/Hexa/Ptprc/Apoe/Chd7/Osbpl8/Cherp	32
Macf1/Rhob/Mul1/Cdc42se2/Plekho1/Lims1/Anxa7/Sh3kbp1/Limd1/Dhx36/Bcl11a/St6gal1/Diaph1/Cux1/Zranb1/Mecp2/Rcc2/Epb41/Arhgap15/Rhog/C1qbp/Dnm2/Rhoh/Phip/Dbnl/Brwd1/Gna13/Msn/Myadm/Nedd9/Larp	32
Wnk1/Klf4/Smad7/Rc3h2/H2-Ab1/Pag1/Laptm5/Tnfaip8l2/Hspb1/Itch/Nfkbid/Cblb/Ptpn2/Btla/Cd86/Il4ra/Peli1/Zc3h12a/Bcl6	19
Jun/Hsp90ab1/Fos/Mef2c/Smad7/Zeb2/Cited2/Crebbp/Nr3c1/Parp1/Sirt1/Zfp36l2/Arrb2/Ep300/Got1/Nrros/Snw1/Rock1/Usp15/Stk11/Dnm2/Flcn/Pml/Zyx/Glg1	25
Mef2c/Syk/Irs2/H2-Ab1/Rps3/Laptm5/Inpp5d/Dhps/Rasal3/St6gal1/Bcl2/Itch/Siglecg/Cblb/Tnfrsf13c/Btla/Cd55/Cd86/Myd88/Il6st/Ctnnb1/Ccr7/Cd81/Peli1/Ptprc/Bcl6	26
Irs2/Nr3c1/Sirt1/Foxo1/Ep300/Akt2/Inpp5k/Ogt/Ptpn2/Usp7/Nfkb1/Plek/Supt20/Cltc/Stk11/Ranbp2	16
Hsp90aa1/Pde4b/Wnk1/Mef2c/Foxp1/Syk/Ms4a1/Rc3h2/Rps3/Laptm5/Dgkz/Malt1/Matr3/Elf1/Bcl2/Cr1l/Nfkbid/Ptprj/Prkcb/Cblb/Eif2b2/Dennd1b/Rap1a/Ptpn2/Cd55/Stap1/Pspc1/C1qbp/Mndal/Blnk/Cd19/Rabgef1/Ccr7/	38
Klf4/Kmt2e/Pcid2/Casp2/Tmod3/Dgkz/Crebbp/Bcl2/Zfp36l2/Zfp655/Cdc25b/Topbp1/Camk2d/Mecp2/Anp32b/Rcc2/Ankrd17/Chfr/Ccnd3/Sde2/Pten/Ik/Pml/Rad21/Donson/Lmnb1/Gpr132/Foxn3/Ccnd2	29
Top2b/Nap1l1/Dhx36/Acin1/Ubtf/Smarca5/Sart3/Rsf1/Sirt1/Daxx/Npm1/Smarcc1/Cabin1/Anp32b/Atrx/Top3b/Ubn1/Smarca2/Paf1/Nap1l4/Akap8l/Nasp/Kat6a	23
Hspa1b/Hsp90ab1/Ppp1r15a/Nr4a1/Gapdh/Klf4/Rps7/Gpx1/Plaur/Pcid2/Snx12/Arl6ip1/Arrb2/Siah2/Csnk2a1/Ddrgk1/Smarcc1/Akt2/Mical1/Ogt/Usp7/Herpud1/Dnajb2/Bin1/Fnip1/Birc6/Pik3r1/Rnf34/Sfn/Fcmr/Pml/Glg1/Us	33
Dnaja1/Hsph1/Zfp36/Dusp1/Traf4/Atf2/Rell1/Gadd45b/Map2k1/Hipk3/Itch/Foxo1/Rb1cc1/Traf1/Gadd45g/Nfkb1/Map4k2/Zfp622/Per1/Zmynd11/Myd88/Spag9/Zc3h12a/Ccdc88c/Lmnb1/Prmt1	26
Hspd1/Syk/Mul1/Rps3/Laptm5/Pmaip1/Casp2/Pdcd2/Malt1/Myc/Sirt1/Fis1/Anp32b/Eif2ak3/Stat1/Ppp2ca/Pml/Cyfip2/Bcl2l11	19
Jun/Fos/Atf3/Itga4/Atf2/Pmaip1/Klf10/Hnrnpl/Bcl2/Sirt1/Foxo1/Pik3c3/Nfe2l2/Got1/Gabarap/Sik2/Prkag1/Snw1/Pik3r4/Eif2ak3/Tnrc6a/Fnip1/Higd1a/Ctnnb1/Wipi2/Map1lc3a/Flcn/Zc3h12a/Ptprc	29
Rps8/Rps21/Rps16/Rps19/Fcf1/Bms1/Abt1/Rrs1/Gtf2h5	9
Hsp90ab1/Hsp90aa1/Klf4/Gpx1/Mul1/Sirt1/Arrb2/Ptprj/Sh2b3/Bank1/Lmbrd1/Akt2/Inpp5k/Arfgef1/Hax1/C1qbp/Pten/Stk11/Rps6kb1/Flcn/Setx/P2rx4/Osbpl8	23
G3bp1/Ddx6/Prrc2c/Csde1/Ythdf3/Ubap2l/Atxn2l/Pum2	8
Mef2c/Cfl2/Cacybp/Actg1/Foxp1/Naca/Gpx1/Akap13/Nfatc3/Plekho1/Tmod3/Ifrd1/Kdm6b/Nr3c1/Bcl2/Csrp1/Sirt1/Arrb2/Daxx/Nfatc1/Kras/Ccnt2/Camk2d/Cdk9/Mecp2/Ppp3ca/Bin1/Ankrd17/Arid1a/Epc1/Il4ra/Flii/Ctnnb1/	37
Wnk1/Klf4/Smad7/Rc3h2/H2-Ab1/Pag1/Laptm5/Tnfaip8l2/Hspb1/Map2k1/Itch/Nfkbid/Cblb/Sh2b3/Ptpn2/Btla/Cd86/Pik3r1/Il4ra/Peli1/Zc3h12a/Myadm/Bcl6	23
Nfkbia/Zfp36/Gpr171/Clec2d/Inpp5d/Myc/Ldb1/Ptpn2/Pik3r1/Hspa9/Ctnnb1/Paf1/Tob2/Prmt1	14
Kmt2e/Arid4b/Setd2/Sirt1/Mtf2/Kmt2c/Ogt/Snw1/Mecp2/Ehmt1/Iws1/Atrx/Pax5/Rtf1/Ctnnb1/Kmt2a/Nsd1	17
Kdm5a/Id3/Btbd9/Crem/Klf10/Egr1/Ahcy/Sirt1/Csnk1d/Fbxl3/Ep300/Siah2/Ogt/Usp7/Pspc1/Per1/Ncoa2/Bhlhe40/Pten/Mycbp2/Setx/Relb/Pml/Kmt2a	24
Rab43/Atp6v0a1/Tcirg1/Atg12/Aup1/Arl8b/Sirt1/Usp36/Rb1cc1/Pik3c3/Snapin/Smg1/Gabarap/Ddrgk1/Nsfl1c/Pik3r4/Plekhm1/Trp53inp1/Uvrag/Larp1/Wipi2/Map1lc3a/Vti1b/Rab3gap1	24
Ralgapa1/S1pr1/Lims1/Map2k1/Bcar3/Dennd1b/Rap1a/Rabgap1/Akt2/Rcc2/Rhog/Rabgap1l/Bin1/Dock8/Tbc1d1/Arhgap25/Ccr7/Gpr65/Flcn/Arap2/Rapgef6/Rab3gap1/Nedd9/Iqsec1/Dock11	25
Gapdh/Actg1/Rpl13a/Capg/Myo1c/H2-Ab1/Rab43/Myo18a/Nmi/H2-Q7/Vamp3/Ptpn2/Stat1/Rps6kb1/Zyx/Vim	16
Dnaja1/Hsph1/Zfp36/Dusp1/Traf4/Atf2/Rell1/Gadd45b/Map2k1/Hipk3/Itch/Foxo1/Rb1cc1/Traf1/Gadd45g/Nfkb1/Map4k2/Zfp622/Per1/Zmynd11/Myd88/Spag9/Zc3h12a/Lmnb1/Prmt1	25
Hsp90ab1/Ppp1r15a/Ssh2/Dusp1/Dusp5/Bod1l/Ppp1r15b/Ssu72/Bcl2/Ptprj/Ikbkb/Cdc25b/Ptpn2/Ppp3ca/Rock1/Ppp2r2a/Ptp4a2/Pten/Ppp2r5a/Stk11/Ppm1g/Ppp2ca/Ptprc/Hsp90b1/Ptpn1	25
Hsp90aa1/Ssh2/Syk/Rpl13a/Capg/Myo1c/Rps3/Sptbn1/Tmod3/Senp6/Fkbp4/Rnf4/Arpc5/Parp1/1810058I24Rik/Ep300/Fchsd2/Birc2/Mecp2/Arfgef1/Plek/Hax1/Bin1/Fnip1/Preb/Myd88/Flii/Akap9/Faf1/Dbnl/Riok3/Msn/Cyfi	38
Dnaja1/Hsph1/Gpx1/Plaur/Tpt1/Hspb1/Map2k1/Bcl2/Sirt1/Nfe2l2/Eno1/Herpud1/Ivns1abp/Pttg1ip/Ptpn1	15
Hsph1/Gpx1/Hspb1/Aldh2/Bcl2/Parp1/Sirt1/Nfe2l2/Zfp622/Pml	10
Klf4/Mul1/Sirt1/Ptprj/Sh2b3/Bank1/Lmbrd1/Inpp5k/Pten/Flcn	10
Foxp1/Smad7/Rc3h2/Malt1/Irf4/Nfkbid/Gadd45g/Ly9/Il4ra/Ccr7/Relb/Zc3h12a/Bcl6	13
Mef2c/Syk/Irs2/H2-Ab1/Rps3/Laptm5/Inpp5d/Dhps/Rasal3/Bcl2/Itch/Siglecg/Cblb/Tnfrsf13c/Btla/Cd55/Cd86/Myd88/Il6st/Ctnnb1/Ccr7/Cd81/Peli1/Ptprc/Bcl6	25
Hsp90ab1/Hsp90aa1/Cyth1/Macf1/Rhob/Cenpa/Lims1/Tcirg1/Myo18a/Exoc4/Arpc5/Krit1/Map4/Nsfl1c/Arfgef1/Rhog/Dock8/Ccr7/Dock2/Stk11/Rhoh/Msn/Numa1/Parvg	24
Jun/Hsp90ab1/Fos/Mef2c/Smad7/Zeb2/Cited2/Crebbp/Nr3c1/Parp1/Sirt1/Zfp36l2/Arrb2/Ep300/Got1/Nrros/Snw1/Usp15/Stk11/Dnm2/Flcn/Pml/Zyx/Glg1	24
Rnf125/Zc3hav1/Ankrd17/C1qbp/Usp15/Riok3/Pum2	7
Ppp1r15a/Ppp1r15b/Tmed2/Npm1/Eif2ak3/Pml/Dnajc3	7
Gpx1/Atf3/Pmaip1/Sgk3/Sp100/Bcl2/Dedd2/Rb1cc1/Traf1/Siah2/Zmynd11/Birc6/Rnf34/Pten/Fcmr/Faf1/Rps6kb1/Phip/Pml/Ptprc	20
Hspa8/Jun/Chd1/Vapa/Ep300/Inpp5k/Snw1/Sp1/Zc3h12a/Nucks1/Bcl2l11/Apoe	12
Jun/Hsp90aa1/Fos/Foxp1/Smad7/Syk/Rc3h2/Pcid2/Clec2d/Inpp5d/Hspb1/Sart1/Klf10/Malt1/Myc/Zfp36l2/Nfkbid/Sh2b3/Ptpn2/Hax1/Pik3r1/Clptm1/Il4ra/Ctnnb1/Ccr7/Rhoh/Zc3h12a/Ptprc/Bcl6/Tob2	30
Naa35/Gm16286/Naa50/Crebbp/Naa10/Ep300/Nmt2/Naa15	8
Rps21/Fcf1/Bms1/Pop7/Npm1/Abt1/Rrs1/Zc3h12a	8
Gapdh/Atf3/Nr3c1/Sirt1/Foxo1/Ep300/Ogt/Ptpn2/Usp7/Supt20/Pgk1/Stk11/Ranbp2	13
Foxp1/Rnf6/Nr3c1/Parp1/Sirt1/Ep300/Ddrgk1/Lbh/Cnot1/Per1/Ncoa2/Cnot2/Calr	13
Hsp90ab1/Macf1/Ano6/Ifrd1/Rnf6/Anxa7/Bcl11a/Slc12a6/Ist1/Rb1cc1/Atp5g1/Zfyve27/Dip2b/Golga4/Npm1/Eif2b2/Ogt/Plek/Pten/Dnm2/Dbnl/Apoe/Gdi1	23
Hspa8/Gapdh/Chchd2/Atp5e/Map2k1/Atp5c1/Myc/Ndufv1/Parp1/Ak2/Chchd10/Ep300/Atp5g1/Fis1/Zbtb20/Eno1/Ndufs6/Ogt/Tspo/Pgk1/Ogdh/Ndufs8/Cox7a2/Flcn/Atp6v1b2/Atp5a1	26
Klf4/Kmt2e/Rpl24/Atf2/Pcid2/Casp2/Tmod3/Dgkz/Crebbp/Bcl2/Zfp36l2/Fbxl3/Nfatc1/Zfp655/Cdc25b/Topbp1/Npm1/Camk2d/Mecp2/Anp32b/Ppp3ca/Rcc2/Ankrd17/Chfr/Ccnd3/Sde2/Pten/Ik/Rps6kb1/Paf1/Akap8l/Pml/Ra	40
Myo1c/Pmaip1/Sptbn1/Adam10/Map2k1/Fis1/Csnk2a1/Rap1a/Camk2d/Akt2/Inpp5k/Ogt/Pgrmc1/Rer1/Rhog/Atp2c1/Pik3r1/Cltc/Ppp2r5a/Vti1b/Cd81/Gdi1	22
Klf4/Kmt2e/Rpl24/Pcid2/Casp2/Tmod3/Dgkz/Crebbp/Bcl2/Zfp36l2/Fbxl3/Nfatc1/Zfp655/Cdc25b/Topbp1/Camk2d/Mecp2/Anp32b/Ppp3ca/Rcc2/Ankrd17/Chfr/Ccnd3/Sde2/Pten/Ik/Rps6kb1/Pml/Rad21/Nasp/Donson/Lmnb	35
Kdm5a/Id3/Btbd9/Crem/Klf10/Crebbp/Egr1/Ahcy/Sirt1/Csnk1d/Arrb2/Fbxl3/Ep300/Siah2/Csnk2a1/Ogt/Usp7/Pspc1/Per1/Ncoa2/Bhlhe40/Pten/Sp1/Mycbp2/Nfkb2/Setx/Relb/Pml/Kmt2a	29
Jun/Fos/Chchd2/Hsph1/Rhob/Foxp1/Gpx1/Atf2/Rps3/Hspb1/Prdx3/Kdm6b/Neil1/Ppp1r15b/Aldh2/Map2k1/Bcl2/Arnt/Parp1/Sirt1/Foxo1/Nfe2l2/Nfkb1/Zfp622/Ndufs8/Trp53inp1/Oser1/Ctnnb1/Psap/Sp1/Setx/Pml/Chuk/Zc	35
Rps21/Fcf1/Bms1/Npm1/Abt1/Rrs1	6
Rps21/Fcf1/Bms1/Npm1/Abt1/Rrs1	6
Jun/Chd1/Ep300/Snw1/Sp1/Nucks1	6
Zfp36/Dhx36/Zfp36l2/Cnot1/Tnrc6c/Tnrc6b	6
Chchd2/Atf2/Cited2/Egr1/Map2k1/Myc/Bcl2/Cr1l/Sirt1/Hif1an/Nfe2l2/Eno1/Ogt/Pgk1/Higd1a/Pten/Usp19	17
Foxp1/Smad7/Satb1/Rc3h2/Malt1/Irf4/Nfkbid/Gadd45g/Ly9/Il4ra/Ccr7/Relb/Zc3h12a/Bcl6	14
Mef2c/Syk/Irs2/H2-Ab1/Rps3/Laptm5/Inpp5d/Dhps/Rasal3/St6gal1/Bcl2/Itch/Siglecg/Cblb/Tnfrsf13c/Btla/Cd55/Cd86/Myd88/Il6st/Ctnnb1/Ccr7/Cd81/Peli1/Ptprc/Bcl6	26
Macf1/Actg1/Lims1/Lamtor2/Ldb1/Ptprj/Iqgap1/Rock1/Rcc2/Pik3r1/Pten/Iqsec1	12
Rps3/Laptm5/Dgkz/Malt1/Elf1/Ptprj/Cblb/Ptpn2/Ccr7/Cd81	10
Jun/Hsp90ab1/Fos/Smad7/Zeb2/Cited2/Crebbp/Parp1/Sirt1/Arrb2/Ep300/Got1/Nrros/Snw1/Usp15/Stk11/Dnm2/Flcn/Pml/Zyx/Glg1	21
Hsp90ab1/Myo1c/Pmaip1/Sptbn1/Parp1/Fis1/Npm1/Iqgap1/Ddrgk1/Akt2/Ogt/Pgrmc1/Epb41/Eif2ak3/Rer1/Cd2ap/Rhog/Atp2c1/Pik3r1/Stk11/Rbm22/Cd81/Pml/Zc3h12a/Msn/Gnl3/Numa1	27
Hsp90aa1/Hsph1/Hspd1/Syk/H2-Ab1/Rps3/Sart1/Malt1/Dhps/Rasal3/Nfkbid/H2-Eb2/Tnfrsf13c/Cd55/Cd86/Dock8/Il6st/Il4ra/Ccr7/Rhoh/Cd81/Ptprc/Bcl6	23
Smad7/Rc3h2/H2-Ab1/Pag1/Laptm5/Tnfaip8l2/Hspb1/Itch/Nfkbid/Cblb/Ptpn2/Btla/Cd86/Il4ra/Peli1/Zc3h12a/Bcl6	17
Wnk1/Klf4/Smad7/Dusp1/Rc3h2/H2-Ab1/Pag1/Laptm5/Tnfaip8l2/Hspb1/Adam10/Map2k1/Itch/Nfkbid/Pde3b/Cblb/Sh2b3/Ptpn2/Btla/Rcc2/Cd86/Pik3r1/Il4ra/Pten/Peli1/Zc3h12a/Ptprc/Myadm/Bcl6	29
Klf4/Kmt2e/Rpl24/Atf2/Pcid2/Casp2/Tmod3/Dgkz/Crebbp/Myc/Bcl2/Sirt1/Zfp36l2/Zfp655/Cdc25b/Topbp1/Iqgap1/Camk2d/Mecp2/Anp32b/Rcc2/Ankrd17/Chfr/Ccnd3/Cdk11b/Sde2/Ctnnb1/Pten/Ik/Rps6kb1/Phip/Pml/Rad	38
Mef2c/Syk/Irs2/Rps3/Dhps/Rasal3/St6gal1/Bcl2/Tnfrsf13c/Cd55/Cd86/Myd88/Il6st/Ccr7/Cd81/Peli1/Ptprc/Bcl6	18
Ubb/Shisa5/Rps7/Rpl37/Rps20/Pmaip1/Casp2/Sp100/Sirt1/Ep300/Npm1/Rrs1/Snw1/Atrx/Pttg1ip/Rnf34/Stk11/Pml	18
Hsp90ab1/Hsp90aa1/Klf4/Gpx1/Mul1/Sirt1/Arrb2/Ptprj/Sh2b3/Bank1/Lmbrd1/Inpp5k/Arfgef1/Hax1/C1qbp/Pten/Stk11/Flcn/P2rx4/Osbpl8	20
Mbnl1/Malat1/Fam172a/Sap18/Hnrnpl/Rbm5/Celf1/Celf2/Magoh/Nsrp1/Mbnl2	11
Klf4/Kmt2e/Rpl24/Atf2/Pcid2/Casp2/Tmod3/Dgkz/Crebbp/Bcl2/Zfp36l2/Zfp655/Cdc25b/Topbp1/Npm1/Camk2d/Mecp2/Anp32b/Rcc2/Ankrd17/Chfr/Ccnd3/Sde2/Pten/Ik/Paf1/Pml/Rad21/Donson/Lmnb1/Gpr132/Foxn3/Ccr	33
Pmaip1/Bcl2/Pik3c3/Nfe2l2/Sik2/Prkag1/Pik3r4/Eif2ak3/Higd1a/Zc3h12a	10
Hsp90ab1/Hsp90aa1/Macf1/Mul1/Itga4/Ifrd1/Rnf6/Bcl11a/Atp5g1/Zfyve27/Dip2b/Golga4/Eif2b2/Iqgap1/Mecp2/Ctnnb1/Stk11/Dnm2/Dbnl/Cyfip2/Apoe/Gdi1	22
Chchd2/Atf2/Cited2/Egr1/Map2k1/Myc/Bcl2/Cr1l/Sirt1/Hif1an/Foxo1/Nfe2l2/Eno1/Ogt/Pgk1/Higd1a/Pten/Usp19	18
Gpx1/Atf3/Pmaip1/Zdhhc3/Sp100/Bcl2/Dedd2/Gabarap/Ddx47/Pik3r1/Rnf34/Pten/Faf1	13
Gapdh/Atf3/Nr3c1/Sirt1/Foxo1/Ep300/Ogt/Ptpn2/Usp7/Supt20/Pgk1/Stk11/Ranbp2	13
Jun/Rhob/Gpx1/Pmaip1/Crebbp/Dhx36/Egr1/Rnf4/Myc/Bcl2/Parp1/Sirt1/Fbxl3/Ep300/Dclre1c/Kras/Topbp1/Npm1/Akt2/Pcna/Ube2b/Mecp2/Ube4b/Per1/Trp53inp1/Pik3r1/Bhlhe40/Sde2/Gtf2h5/Stk11/Pml/Kmt2a/Nucks1/	36
Rhob/Gpx1/Pmaip1/Egr1/Rnf4/Myc/Bcl2/Sirt1/Dclre1c/Topbp1/Gtf2h5/Stk11/Pml/Nucks1/Ptprc/Nipbl/Ccnd2	17
Hspa1a/Hspa1b/Hsp90ab1/Smad7/Arih1/Csnk1d/Xpo1/Gabarap/Ddrgk1/Smarcc1/Ogt/Usp7/Herpud1/Zfand2a/Dnajb2/Chfr/Arih2	17
Wnk1/Klf6/Nfkbia/Syk/Mul1/Il10ra/Laptm5/Lims1/Ifngr1/Egr1/Nmi/Sirt1/Traf1/Ikbkb/Kras/Sh2b3/Ugcg/Tnfrsf13c/Jak1/Stat4/Fkbp1a/Ptpn2/Stap1/Ifnar1/Stat1/Myd88/Il6st/Ythdf3/Rabgef1/Irak3/Chuk/Ptprc/Crebrf/Il17ra	34
Pcid2/Setd2/Rae1/Nup88/Iws1/Poldip3/Eny2/Akap8l/Pabpn1/Chtop/Nxf1	11
Pcid2/Setd2/Rae1/Nup88/Iws1/Poldip3/Eny2/Akap8l/Pabpn1/Chtop/Nxf1	11
Zfp36/Limd1/Map2k1/Ddx6/Nfkb1/Eif4e2/Pum2/Tnrc6b	8
Fth1/Ftl1/Sp100/Diaph1/Os9/Ankrd13c/Chchd10/Fkbp1a/Camk2d/Sri/Tspo/Rer1/Vps13a/Tnrc6a/Uvrag/Cd19/Dbi/Akap9/Pml/Ptprc/Apoe/Chd7/Cherp	23
Hspa8/Dazap1/Polr2a/Snrnp70/Snw1/Pik3r1/Rbm22/Setx/Slc38a2/Srsf5	10
Pde4b/Mef2c/Rhob/Cfl2/Actg1/Ssh2/Capg/Akap13/Myo1c/Sptbn1/S1pr1/Tmod3/Arpc5/Fchsd2/Iqgap1/Inpp5k/Sri/Stap1/Arfgef1/Rock1/Plek/Cd2ap/Hax1/Rhog/Bin1/Pik3r1/Mtss1/Flii/Akap9/Bst2/Gpr65/Rhoh/Dbnl/Cyfip2/	35
Zfp36/Gpx1/Mul1/Rps19/Tnfaip8l2/Clec2d/Rnf125/Nmi/Ets1/Itch/Arrb2/Siglecg/Ptpn2/Nfkb1/C1qbp/Ythdf3/Usp15/Rabgef1/Cnr2/Cd200/Irak3/Riok3/Socs3/Apoe	24
Macf1/Smad7/Mul1/Adam10/Bcl11a/Nr3c1/Bcl2/Sirt1/Eif4g2/Ist1/Atp5g1/Zfyve27/Golga4/Csnk2a1/Eif2b2/Mecp2/Derl2/Sfn/Cd81/Dbnl/Apoe/Gdi1	22
Hspa8/Jun/Chd1/Vapa/Ep300/Inpp5k/Snw1/Sp1/Zc3h12a/Nucks1/Apoe	11
Tmed5/Rab43/Plekhj1/Sptbn1/Ano6/Lysmd3/Ccdc47/Myo18a/Syngr2/Map2k1/Arl8b/Csnk1d/Tmed2/Arl6ip1/Tmem127/Pik3c3/Snapin/Zfyve27/Ugcg/Akt2/Nsfl1c/Reep3/Arfgef1/Eif2ak3/Bin1/Tmed9/Cltc/Rtn3/Mtss1/Pten/A	39
Marcksl1/Rhob/Cfl2/Actg1/Ssh2/Capg/Myo1c/Sptbn1/S1pr1/Tmod3/Sh3kbp1/Diaph1/Pstpip1/Bcl2/Arpc5/Fchsd2/Mical1/Inpp5k/Elmo1/Arfgef1/Plek/Cd2ap/Hax1/Rhog/Bin1/Pik3r1/Arhgap25/Mtss1/Flii/Gpr65/Rhoh/Dbnl/Z	37
Pde4b/Wnk1/Mef2c/Foxp1/Syk/Ms4a1/Rc3h2/Rps3/Laptm5/Dgkz/Malt1/Elf1/Bcl2/Nfkbid/Ptprj/Prkcb/Cblb/Eif2b2/Dennd1b/Rap1a/Ptpn2/Btla/Stap1/Blnk/Cd19/Rabgef1/Ccr7/Stk11/Cd81/Zc3h12a/Ptprc	31
Mef2c/Rhob/Cfl2/Actg1/Ssh2/Capg/Akap13/Myo1c/Sptbn1/S1pr1/Tmod3/Arpc5/Fchsd2/Iqgap1/Inpp5k/Stap1/Arfgef1/Rock1/Plek/Cd2ap/Hax1/Rhog/Bin1/Pik3r1/Mtss1/Flii/Bst2/Gpr65/Rhoh/Dbnl/Cyfip2/Myadm	32
Zfp36/Limd1/Map2k1/Ddx6/Nfkb1/Eif4e2/Pum2/Tnrc6b	8
Klf2/Sertad1/Eif1/Gpx1/Arid4b/Fam172a/Xist/Smchd1/Nr3c1/Ubtf/Smarca5/Sirt1/Ep300/Mecp2/Epc1/Zmiz2	16
Klf2/Klf4/Atf2/Tcf4/Pde3b/Krit1/Sh2b3/Mecp2/Rock1/Sars/Pgk1/Stat1/Ctnnb1/Flcn/Pml	15
Hspd1/Syk/Mul1/Rps3/Laptm5/Pmaip1/Casp2/Pdcd2/Malt1/Myc/Sirt1/Fis1/Ddrgk1/Anp32b/Eif2ak3/Stat1/Ppp2ca/Pml/Cyfip2/Bcl2l11	20
Hsp90ab1/Myo1c/Pmaip1/Sp100/Os9/Xpo1/Fis1/Akt2/Inpp5k/Ogt/Anp32b/Pcm1/Vamp2/Derl2/Tmem30a/Pik3r1/Sfn/Rbm22/Ppp2ca/Cd81/Zc3h12a/Ptpn1/Gdi1	23
Kmt2e/Azin1/Pcid2/Tmod3/Cited2/Crebbp/Myc/Cdc25b/Npm1/Camk2d/Ube2b/Nsfl1c/Mecp2/Anp32b/Rcc2/Atrx/Ankrd17/Ccnd3/Cxcr5/Rps6kb1/Paf1/Phip/Rad21/Bcl2l11/Usp19/Lmnb1/Srpk2/Calr/Numa1/Ccnd2	30
Klf2/Klf4/Mef2c/Cited2/Nfe2l2/Mtss1/P2rx4	7
Pde4b/Wnk1/Mef2c/Foxp1/Syk/Ms4a1/Rc3h2/Rps3/Laptm5/Dgkz/Malt1/Elf1/Bcl2/Nfkbid/Ptprj/Prkcb/Cblb/Eif2b2/Dennd1b/Ptpn2/Stap1/Blnk/Cd19/Ccr7/Stk11/Cd81/Zc3h12a/Ptprc	28
Hspa8/Hspd1/Smad7/Arid5a/Malt1/H2-Q7/Fbxo38/Dennd1b/H2-M3/Cd55/H2-Q6/H2-Q4/Cd81/Ptprc/H2-T23	15
Smad7/Rc3h2/Clec2d/Inpp5d/Hspb1/Myc/Nfkbid/Ptpn2/Pik3r1/Il4ra/Hspa9/Ctnnb1/Zc3h12a/Bcl6/Tob2	15
Zfp36/Limd1/Map2k1/Ddx6/Mecp2/Nfkb1/Ppp3ca/Eif4e2/Tnrc6c/Zc3h12a/Pum2/Tnrc6b	12
Zfp36/Rc3h2/Dhx36/Zfp36l2/Tnrc6a/Cnot1/Pan3/Cnot2/Tnrc6c/Tnrc6b	10
H13/Aup1/Os9/Herpud1/Derl2/Preb/Tmem30a/Sel1l/Cd81	9
Macf1/Nsf/Sptbn1/Vamp3/Golga4/Vamp2/Atp2c1/Golga7/Sys1	9
Smad7/Arid5a/Rc3h2/Malt1/Irf4/Nfkbid/Ly9/Zc3h12a/Il17ra	9
Macf1/Nsf/Sptbn1/Exoc4/Vamp3/Golga4/Vamp2/Atp2c1/Dnm2/Golga7/Sys1	11
Rps19/Ubtf/Smarca5/Sirt1/Usp36/Pes1	6
Klf2/Klf4/Atf2/Tcf4/Pde3b/Krit1/Sh2b3/Mecp2/Rock1/Sars/Pgk1/Stat1/Ctnnb1/Flcn/Pml	15
Pde4b/Wnk1/Mef2c/Foxp1/Syk/Ms4a1/Rc3h2/Rps3/Laptm5/Dgkz/Malt1/Elf1/Bcl2/Nfkbid/Ptprj/Prkcb/Cblb/Eif2b2/Dennd1b/Rap1a/Ptpn2/Stap1/Blnk/Cd19/Rabgef1/Ccr7/Stk11/Cd81/Zc3h12a/Ptprc	30
Jun/Fos/Zfp36/Atf3/Itga4/Atf2/Pmaip1/Klf10/Kynu/Hnrnpl/Bcl2/Ahcy/Sirt1/Foxo1/Pik3c3/Nfe2l2/Gabarap/Sik2/Prkag1/Snw1/Pik3r4/Eif2ak3/Tnrc6a/Fnip1/Higd1a/Larp1/Wipi2/Map1lc3a/Rps6kb1/Flcn/Zc3h12a/Ptprc/Apoe	33
Mbnl1/Malat1/Fam172a/Sap18/Rbm17/Hnrnpl/Rbm5/Celf1/Celf2/Magoh/Nsrp1/Mbnl2	12
Ppp1r15a/Tmem259/Sirt1/Ddrgk1/Ptpn2/Herpud1/Eif2ak3/Pik3r1/Atf6/Usp19/Crebrf/Ptpn1	12
Smad7/Rc3h2/H2-Ab1/Pag1/Laptm5/Tnfaip8l2/Inpp5d/Hspb1/Itch/Nfkbid/Cblb/Bank1/Ptpn2/Btla/Cd86/Il4ra/Peli1/Zc3h12a/Bcl6	19
Pde4b/Wnk1/Mef2c/Foxp1/Syk/Ms4a1/Rc3h2/Rps3/Laptm5/Dgkz/Malt1/Elf1/Bcl2/Nfkbid/Ptprj/Prkcb/Cblb/Eif2b2/Dennd1b/Rap1a/Ptpn2/Stap1/Blnk/Cd19/Rabgef1/Ccr7/Stk11/Cd81/Zc3h12a/Ptprc	30
Map2k1/Ldb1/Ccnt2/Wdr43/Zfp326/Ccnt1/Zmynd11/Ctnnb1/Scaf8/Supt16	10
Hsph1/Zfp36/Dusp1/Atf2/Rell1/Gadd45b/Gadd45g/Per1/Zc3h12a/Prmt1	10
Jun/Gpx1/Plaur/Pmaip1/Bcl2/Arrb2/Fis1/Ghitm/Higd1a/Sfn/Bcl2l11	11

Zfp91/Rnf4/Itch/Traf1/Birc2/Ube2b/Ube2g1/Trim12a/Arih2/Peli1/Trip12	11
Hspd1/Foxp1/Zfp36/Syk/Arid5a/Ifngr1/Hspb1/Cd2/Sirt1/Cd84/Arrb2/Ptprj/Zbtb20/Tspo/Pik3r1/Myd88/Ccr7/Irak3/Zc3h12a/Ptprc	20
Klf2/Klf4/Atf2/Tcf4/Pde3b/Krit1/Sh2b3/Mecp2/Rock1/Sars/Pgk1/Stat1/Ctnnb1/Flcn/Pml	15
Hspa8/Hspd1/Mef2c/Smad7/Arid5a/Bach2/Rc3h2/H2-Ab1/Inpp5d/Tcirg1/Malt1/Irf4/Cr1l/Nfkbid/H2-Q7/Fbxo38/H2-Eb2/Tnfrsf13c/Dennd1b/H2-M3/Gadd45g/Cd55/C1qbp/H2-Q6/Ly9/Myd88/Cd19/Il4ra/Ccr7/H2-Q4/Cd81/N	38
Dnaja1/Hsph1/Dusp1/Traf4/Rell1/Gadd45b/Map2k1/Hipk3/Itch/Foxo1/Rb1cc1/Traf1/Gadd45g/Map4k2/Zfp622/Per1/Zmynd11/Myd88/Zc3h12a/Lmnb1/Prmt1	21
Syk/Rasal3/Itch/Cblb/Btla/Cd55/Dock2/Cd81/Ptprc	9
Hspd1/Syk/Mul1/Rps3/Laptm5/Pmaip1/Casp2/Pdcd2/Malt1/Myc/Sirt1/Fis1/Anp32b/Eif2ak3/Stat1/Ppp2ca/Pml/Cyfip2/Bcl2l11	19
Jun/Klf4/Plaur/Sp100/Irf4/Myc/Hipk3/Rsf1/Parp1/Sirt1/Ep300/Ddrgk1/Sri/Ctnnb1/Nsd1/Hspa4	16
Kmt2e/Arid4b/Setd2/Sirt1/Mtf2/Kmt2c/Ogt/Snw1/Mecp2/Ehmt1/Iws1/Atrx/Pax5/Rtf1/Ctnnb1/Kmt2a/Nsd1	17
Hspa8/Jun/Gapdh/Hspd1/Gpx1/Chd1/Vapa/Ep300/Inpp5k/Snw1/Myd88/Sp1/Zc3h12a/Nucks1/Apoe	15
Nr3c1/Sirt1/Foxo1/Ep300/Ogt/Ptpn2/Usp7/Supt20/Stk11/Ranbp2	10
Nfkbia/Mul1/Pmaip1/Dhx36/Npm1/Zc3hav1/Nfkb1/Stat1/Irak3/Peli1/Riok3	11
Gapdh/Atf3/Nr3c1/Sirt1/Foxo1/Ep300/Ogt/Ptpn2/Usp7/Supt20/Pgk1/Stk11/Ranbp2	13
Ubb/Hsp90ab1/Junb/Arid4b/Itga4/Plaur/Dazap1/Xist/Prdx3/Cited2/Fkbp4/Setd2/Map2k1/Bcl2/Arnt/Sirt1/Tmed2/Jmjd1c/Arrb2/Birc2/Eif2b2/Ndufs6/Fndc3a/Bptf/Atrx/Arid1a/Birc6/Ctnnb1/Pten/Akap9/Psap/Stk11/Sp1/Socs	38
Eif3f/Stambpl1/Sart3/Itch/Usp36/Zranb1/Usp7/Usp4/Dnajb2/Usp15/Eny2/Usp16/Usp24/Zc3h12a/Usp19	15
Jun/Dhx36/Myc/Sub1/Ubtf/Smarca5/Rsf1/Med13/Gtf2a2/Gtf2h5/Tfam/Bdp1/Bclaf1/Setx	14
Trim25/Zfp36/Crebbp/Sp100/Trim59/Zc3hav1/Inpp5k/Fam111a/Stat1/Bst2/Trim12a/Pml/Trim11/Srpk2	14
Macf1/Actg1/Lims1/Lamtor2/Bcl2/Ldb1/Ptprj/Iqgap1/Rock1/Rcc2/Pik3r1/Pten/Tln1/Iqsec1	14
Hspa1a/Hspa1b/Smad7/Laptm5/Arih1/Csnk1d/Gabarap/Ddrgk1/Herpud1/Zfand2a/Dnajb2/Chfr/Pten/Arih2	14
Neat1/Ets1/Daxx/Serbp1/Pml/Srpk2	6
Rnf125/Zc3hav1/Ankrd17/C1qbp/Usp15/Pum2	6
Zfp36/Dhx36/Zfp36l2/Cnot1/Tnrc6c/Tnrc6b	6
Dnaja1/Hsph1/Dusp1/Traf4/Rell1/Gadd45b/Map2k1/Hipk3/Itch/Foxo1/Rb1cc1/Traf1/Gadd45g/Map4k2/Zfp622/Per1/Zmynd11/Myd88/Zc3h12a/Lmnb1/Prmt1	21
Hsp90ab1/Pgap1/Myo1c/Pmaip1/Fis1/Akt2/Mecp2/Anp32b/Pcm1/Vamp2/Tmem30a/Pik3r1/Sfn/Stk11/Rbm22/Cd81/Zc3h12a/Msn/Crebrf/Numa1	20
Hspd1/Foxp1/Zfp36/Syk/Arid5a/Ifngr1/Hspb1/Cd2/Sirt1/Cd84/Arrb2/Ptprj/Zbtb20/Tspo/Pik3r1/Myd88/Ccr7/Irak3/Zc3h12a/Ptprc	20
Chchd2/Atf2/Nfatc3/Limd1/Cited2/Egr1/Map2k1/Myc/Bcl2/Arnt/Cr1l/Sirt1/Hif1an/Foxo1/Nfe2l2/Ep300/Prkcb/Birc2/Eno1/Camk2d/Ogt/Mecp2/Pgk1/Higd1a/Pten/Pml/Usp19	27
Mef2c/Syk/Irs2/Rps3/Dhps/Rasal3/St6gal1/Bcl2/Tnfrsf13c/Cd55/Cd86/Myd88/Il6st/Ccr7/Cd81/Peli1/Ptprc/Bcl6	18
Jun/Fos/Atf2/Pmaip1/Casp2/Egr1/Nr3c1/Parp1/Daxx/Atp5g1/Ikbkb/Fis1/Ctnnb1/Bcl2l11/Apoe/Srpk2	16
Mef2c/Syk/Irs2/Rps3/Dhps/Rasal3/Bcl2/Tnfrsf13c/Cd55/Cd86/Myd88/Il6st/Ccr7/Cd81/Peli1/Ptprc/Bcl6	17
Rhob/Crebbp/Dhx36/Egr1/Rnf4/Parp1/Sirt1/Ep300/Npm1/Akt2/Pcna/Trp53inp1/Pik3r1/Sde2/Gtf2h5/Stk11/Nucks1/Nipbl/Ccnd2	19
Mul1/Egr1/Arnt/Uba2/Ctnnb1/Sae1/Gnl3	7
Dynlt1c/Kras/Gabarap/Camk2d/Ogt/Dock2/Dnm2	7
Dhx36/Nr3c1/Rnf4/Daxx/Zfand2a/Zc3h12a/Slc38a2	7
Foxp1/Rps3/Prkcb/Stap1/Ccr7/Cd81/Ptprc	7
Sertad1/Foxp1/Mul1/Ifrd1/Rnf6/Bcl11a/Bcl2/Sirt1/Ptprj/Dip2b/Eno1/Mecp2/Dnajb2/Mndal/Stk11/Bst2/Smarca2/Flcn/Pml/Rrad/Bcl6	21
Kmt2e/Azin1/Pcid2/Tmod3/Crebbp/Cdc25b/Npm1/Camk2d/Ube2b/Nsfl1c/Mecp2/Anp32b/Rcc2/Atrx/Ankrd17/Ccnd3/Cxcr5/Paf1/Phip/Rad21/Usp19/Lmnb1/Numa1/Ccnd2	24
Hspd1/Mul1/Laptm5/Pmaip1/Casp2/Pdcd2/Myc/Anp32b/Eif2ak3/Stat1/Pml/Bcl2l11	12
Smad7/Rps19/Rc3h2/Clec2d/Cr1l/Cd84/Arrb2/Siglecg/Cd55/Il4ra/Rabgef1/Bst2/Irak3/Zc3h12a/Ptprc/Bcl6	16
Hspd1/Foxp1/Zfp36/Syk/Arid5a/Ifngr1/Hspb1/Cd2/Sirt1/Cd84/Arrb2/Ptprj/Zbtb20/Tspo/Pik3r1/Myd88/Ccr7/Irak3/Zc3h12a/Ptprc	20
Jun/Hspd1/Gpx1/Mul1/Atf2/Plaur/Pmaip1/Bcl2/Arrb2/Chchd10/Fis1/Ghitm/Higd1a/Sfn/Bcl2l11	15
Jun/Fos/Nfkbia/Mef2c/Foxp1/Zfp36/Gpr171/Clec2d/Inpp5d/Klf10/Myc/Ets1/Ldb1/Ptpn2/Hax1/Stat1/Pik3r1/Hspa9/Ctnnb1/Paf1/Tob2/Prmt1	22
Hspa8/Hspd1/Arid5a/H2-Ab1/Malt1/Sirt1/Nfkbid/H2-Q7/Fbxo38/Tnfrsf13c/Dennd1b/H2-M3/Cd55/H2-Q6/H2-Q4/Cd81/Ptprc/H2-T23	18
Dhx36/Hnrnpl/Eif3e/Npm1/C1qbp/Poldip3/Ythdf3/Larp1/Rps6kb1/Usp16/Secisbp2/Dnajc3/Larp4/Larp4b/Vim/Eif2s3x	16
Trim25/Trappc9/Atf2/Rps3/Ifrd1/Malt1/Prdx3/Crebbp/Sp100/Cd84/Traf1/Ep300/Prkcb/Ikbkb/Kras/Npm1/Ddrgk1/Ppp3ca/Myd88/Ctnnb1/Pten/Cd200/Trim12a/Irak3/Chuk	25
Klf4/Mef2c/Rhob/Foxp1/Hspb1/Sp100/Sirt1/Ets1/Nfe2l2/Krit1/Bsg/Mecp2/Sp1/Zc3h12a/Prcp/Apoe/P2rx4/Calr/Atp5a1	19
Pcid2/Setd2/Rae1/Npm1/Nup88/Iws1/Poldip3/Eny2/Akap8l/Pabpn1/Chtop/Nxf1	12
Kmt2e/Arid4b/Setd2/Sirt1/Ogt/Mecp2/Iws1/Atrx/Rtf1/Kmt2a	10
Ppp1r15a/Ppp1r15b/Tmed2/Eif2ak3/Dnajc3	5
Dnajb1/Jun/Ppp1r15a/Cited2/Hif1an	5
Nr4a1/Klf4/Mef2c/Rhob/Foxp1/Gpx1/Hspb1/Sp100/Sirt1/Ets1/Nfe2l2/Krit1/Bsg/Mecp2/Pten/Sp1/Zc3h12a/Prcp/Apoe/P2rx4/Calr/Atp5a1	22
Macf1/Mef2c/Naca/Smad7/Mul1/Bcl11a/Nr3c1/Bcl2/Sirt1/Celf1/Ist1/Atp5g1/Zfyve27/Golga4/Eif2b2/Mecp2/Rps6kb1/Dbnl/Apoe/Chd7/Nipbl/Gdi1	22
Zfp36/Tnrc6a/Cnot1/Pan3/Cnot2/Tnrc6c/Tnrc6b	7
Rps21/Fcf1/Bms1/Npm1/Abt1/Rrs1/Nhp2	7
Mbnl1/Isy1/Celf1/Celf2/Luc7l2/Srsf10/Setx	7
Atp2b1/Myo1c/Sirt1/Ptprj/Prkcb/Lmbrd1/Inpp5k/Ptpn2/Rps6kb1/Nucks1/Socs3/Osbpl8	12
Hspd1/Foxp1/Zfp36/Syk/Arid5a/Ifngr1/Hspb1/Cd2/Sirt1/Cd84/Arrb2/Ptprj/Zbtb20/Tspo/Pik3r1/Myd88/Ccr7/Irak3/Zc3h12a/Ptprc	20
Pde4b/Nfkbia/Mef2c/Foxp1/Zfp36/Rpl13a/Arid5a/Malt1/Prdx3/Sbno2/Cd84/Hnrnpa0/Ogt/Cd55/Stap1/Nfkb1/Ifnar1/Cd86/Stat1/Myd88/Ccr7/Cnr2/Trim12a/Paf1/Irak3/Pabpn1/Peli1/Chuk/Zc3h12a/Prpf8/Ly86/Vim	32
Hspa8/Mef2c/Cfl2/Actg1/Ssh2/Capg/Akap13/Myo1c/Rps3/Sptbn1/S1pr1/Tmod3/Fkbp4/Arpc5/Fchsd2/Inpp5k/Mecp2/Arfgef1/Plek/Hax1/Bin1/Pik3r1/Mtss1/Flii/Akap9/Gpr65/Eml4/Dbnl/Cyfip2/Myadm/Apoe/Numa1	32
Mef2c/Cfl2/Cacybp/Actg1/Foxp1/Naca/Gpx1/Akap13/Nfatc3/Plekho1/Tmod3/Kdm6b/Nr3c1/Bcl2/Csrp1/Sirt1/Arrb2/Daxx/Kras/Camk2d/Ppp3ca/Arid1a/Epc1/Il4ra/Flii/Spag9/Cd81/Chuk	28
Hspa1a/Rps3/Pds5a/Morf4l2/Bod1l/Smchd1/Neil1/Pold4/Setd2/Emsy/Parp1/Sirt1/Tdp2/Dclre1c/Smg1/Topbp1/Npm1/Pcna/Ube2b/Usp7/Cdk9/Cebpg/Atrx/Nsmce4a/Rfc1/Fam111a/Uvrag/Gtf2h5/Tonsl/Fus/Setx/Trip12/Pml	38
Macf1/Mef2c/Foxp1/Naca/Smad7/Mul1/Ifrd1/Rnf6/Cited2/Bcl11a/Nr3c1/Bcl2/Sirt1/Daxx/Celf1/Ist1/Atp5g1/Zfyve27/Dip2b/Golga4/Eif2b2/Mecp2/Tnks2/Pten/Rps6kb1/Dnm2/Plac8/Dbnl/Bcl2l11/Apoe/Chd7/Nipbl/Gdi1	33
Hspa8/Atf2/Rab43/Laptm5/Tpp1/Tcirg1/Atg12/Mbtps1/Arl8b/Rb1cc1/Pik3c3/Snapin/Gabarap/Nsfl1c/Trp53inp1/Wipi2/Map1lc3a/Hexa/Rab3gap1	19
Nr4a1/Pde4b/Klf4/Mef2c/Foxp1/Smad7/Atp2b1/Nr3c1/Map2k1/Parp1/Sirt1/Foxo1/Nfatc1/Eno1/Camk2d/Sri/Cdk9/Ppp3ca/Grcc10/Rock1/Bin1/Akap9/Zc3h12a/P2rx4	24
Macf1/Mef2c/Naca/Smad7/Mul1/Adam10/Bcl11a/Nr3c1/Bcl2/Sirt1/Eif4g2/Celf1/Ist1/Atp5g1/Zfyve27/Golga4/Csnk2a1/Eif2b2/Mecp2/Derl2/Sfn/Rps6kb1/Cd81/Dbnl/Apoe/Chd7/Nipbl/Gdi1	28
Hsp90ab1/Klf4/Plaur/Irf4/Myc/Hnrnpl/Hipk3/Parp1/Eif3e/Krit1/Ep300/Ddrgk1/Eno1/Cdk9/Epb41/Eif2ak3/Ctnnb1/Ppp2ca/Apoe/Hspa4	20
Wnk1/Klf4/Macf1/Dusp1/Dusp5/Kdm6b/Exoc4/Setd2/Ldb1/Lmbrd1/Supt20/Arid1a/Rtf1/Ctnnb1/Tnrc6c/Paf1/Kdm6a/Nsd1/Myadm	19
Vps37b/Trim25/Vapa/Arl8b/Ist1/Trim12a/Pml/Trim11	8
Vps37b/Trim25/Vapa/Arl8b/Ist1/Trim12a/Pml/Trim11	8
Vps37b/Trim25/Vapa/Arl8b/Ist1/Trim12a/Pml/Trim11	8
Ubb/Hsp90ab1/Junb/Arid4b/Itga4/Plaur/Dazap1/Xist/Prdx3/Cited2/Fkbp4/Setd2/Map2k1/Bcl2/Arnt/Sirt1/Tmed2/Jmjd1c/Arrb2/Birc2/Eif2b2/Fndc3a/Bptf/Atrx/Arid1a/Birc6/Ctnnb1/Pten/Akap9/Psap/Stk11/Sp1/Socs3/Bcl2l1	37
Gpx1/Pmaip1/Crebbp/Dhx36/Bcl2/Parp1/Sirt1/Ep300/Npm1/Pcna/Ube2b/Ube4b/Trp53inp1/Pik3r1/Sde2/Stk11/Pml	17
Pde4b/Nfkbia/Mef2c/Foxp1/Zfp36/Rpl13a/Arid5a/Malt1/Prdx3/Sbno2/Cd84/Hnrnpa0/H2-M3/Ogt/Cd55/Stap1/Nfkb1/Ifnar1/Cd86/Stat1/Myd88/Ccr7/Cnr2/Trim12a/Paf1/Irak3/Pabpn1/Peli1/Chuk/Zc3h12a/Prpf8/Ly86/Vim	33
Jun/Nr4a1/Klf4/Macf1/Mef2c/Rhob/Foxp1/Gpx1/Zeb2/Hspb1/Sp100/Sirt1/Ets1/Nfe2l2/Krit1/Bsg/Mecp2/Rock1/Pten/Sp1/Zc3h12a/Prcp/Apoe/P2rx4/Iqsec1/Calr/Atp5a1	27
Klf2/Ppp1r15a/Klf4/Rhob/Atf2/Hspb1/Adam10/Sp100/Sirt1/Ets1/Nfe2l2/Tcf4/Pde3b/Krit1/Prkcb/Sh2b3/Jak1/Mecp2/Rock1/Sars/Pgk1/Stat1/Ctnnb1/Sp1/Flcn/Pml/Zc3h12a	27
Ccdc47/Bcl2/Fis1/Camk2d/Herpud1/Tmco1/Pml	7
Gapdh/Atf3/Irs2/Pmaip1/Nr3c1/Myc/Sirt1/Foxo1/Ep300/Eno1/Akt2/Inpp5k/Ogt/Ptpn2/Usp7/Supt20/Pgk1/Ncoa2/Nisch/Pik3r1/Stk11/Ranbp2	22
Klf4/Kmt2e/Casp2/Dgkz/Crebbp/Bcl2/Nfatc1/Zfp655/Camk2d/Anp32b/Ppp3ca/Ankrd17/Ccnd3/Sde2/Pten/Rps6kb1/Pml/Nasp/Gpr132/Ccnd2	20
Jun/Klf4/Macf1/Mef2c/Rhob/Foxp1/Hspb1/Sp100/Sirt1/Ets1/Nfe2l2/Krit1/Bsg/Mecp2/Pten/Sp1/Zc3h12a/Prcp/Apoe/P2rx4/Iqsec1/Calr/Atp5a1	23
Ubb/Rps7/Rps3/Pmaip1/Sirt1/Fis1/Ptpn2/Eif2ak3/Bclaf1/Flcn/Bcl2l11	11
Jun/Zfp36/Atf3/Atf2/Pmaip1/Klf10/Kynu/Hnrnpl/Bcl2/Ahcy/Sirt1/Foxo1/Pik3c3/Nfe2l2/Gabarap/Sik2/Prkag1/Snw1/Pik3r4/Eif2ak3/Tnrc6a/Fnip1/Higd1a/Larp1/Wipi2/Map1lc3a/Rps6kb1/Flcn/Zc3h12a/Apoe	30
Ubb/Jun/Hsp90ab1/Fos/Gapdh/Hsph1/Hspd1/Mef2c/Gpx1/Atf2/Pmaip1/Casp2/Prdx3/Egr1/Nr3c1/Tmem259/Bcl2/Parp1/Sirt1/Arrb2/Daxx/Atp5g1/Ikbkb/Fis1/Kras/Npm1/Akt2/Mecp2/Rock1/Ctnnb1/Cd200/Bcl2l11/Apoe/Sr	34
Jun/Nr4a1/Klf4/Macf1/Mef2c/Rhob/Foxp1/Gpx1/Zeb2/Hspb1/Sp100/Sirt1/Ets1/Nfe2l2/Krit1/Bsg/Mecp2/Rock1/Pten/Sp1/Zc3h12a/Prcp/Apoe/P2rx4/Iqsec1/Calr/Atp5a1	27
Hspa8/Gapdh/Naca/Bcl2/Sirt1/Arrb2/Nfe2l2/Npm1/Pik3r1/Zc3h12a	10
Ccdc47/Ppp1r15b/Eif2ak3/Tmco1/Bcl2l11	5
Zfp36/Tnrc6a/Cnot1/Tnrc6c/Tnrc6b	5
Smad7/Rc3h2/Clec2d/Inpp5d/Hspb1/Myc/Nfkbid/Ptpn2/Pik3r1/Il4ra/Ctnnb1/Zc3h12a/Bcl6/Tob2	14
Hsp90ab1/Hsp90aa1/Cyth1/Cenpa/Tcirg1/Myo18a/Exoc4/Krit1/Map4/Nsfl1c/Arfgef1/Dock8/Ccr7/Dock2/Stk11/Msn/Numa1	17
Jun/Hsp90ab1/Gapdh/Hsph1/Hspd1/Mef2c/Gpx1/Atf2/Pmaip1/Casp2/Prdx3/Egr1/Nr3c1/Bcl2/Parp1/Sirt1/Arrb2/Fis1/Kras/Npm1/Akt2/Mecp2/Rock1/Ctnnb1/Bcl2l11/Apoe/Srpk2	27
Smad7/Rc3h2/Malt1/Nfkbid/Il4ra/Ccr7/Zc3h12a/Bcl6	8
Hspa1a/Hspa1b/Hsp90ab1/Hsp90aa1/Dnaja1/Pde4b/Ier5/Dhx36/Daxx/Eif2b2/Xylt1/Trp53inp1/Hspa4	13
Birc3/Rc3h2/Rps3/Laptm5/Lims1/Crebbp/Nmi/Ep300/Birc2/Nfat5/Cd86/Zc3h12a/Calr	13
Mef2c/Irs2/Inpp5d/Bcl2/Siglecg/Tnfrsf13c/Btla/Cd81/Peli1/Ptprc/Bcl6	11
Eif3f/Stambpl1/Senp6/Sart3/Itch/Usp36/Zranb1/Usp7/Usp4/Dnajb2/Usp15/Eny2/Usp16/Usp24/Zc3h12a/Usp19	16
Hspa1a/Hspa1b/Smad7/Laptm5/Arih1/Tmem259/Csnk1d/Gabarap/Ddrgk1/Herpud1/Zfand2a/Dnajb2/Chfr/Pten/Arih2	15
Macf1/Smad7/Nap1l1/Zeb2/Lims1/Dhx36/Bcl11a/Map2k1/Myc/Bcl2/Ist1/Cux1/Atp5g1/Zfyve27/Golga4/Eif2b2/Snw1/Tspo/Bin1/C1qbp/Il6st/Ctnnb1/Stk11/Dnm2/Dbnl/Myadm/Apoe/Nedd9/Vim/Calr/Gdi1	31
Jun/Nr4a1/Klf4/Macf1/Mef2c/Rhob/Foxp1/Gpx1/Zeb2/Hspb1/Sp100/Sirt1/Ets1/Nfe2l2/Krit1/Bsg/Mecp2/Rock1/Pten/Sp1/Zc3h12a/Prcp/Apoe/P2rx4/Iqsec1/Calr/Atp5a1	27
Irs2/Pmaip1/Nr3c1/Myc/Sirt1/Foxo1/Ep300/Zbtb20/Akt2/Inpp5k/Ogt/Ptpn2/Usp7/Nfkb1/Plek/Supt20/Ncoa2/Cltc/Stk11/Ranbp2	20
Hspa8/Hspd1/Arid5a/H2-Ab1/Malt1/Nfkbid/H2-Q7/Fbxo38/Tnfrsf13c/Dennd1b/H2-M3/Cd55/H2-Q6/H2-Q4/Cd81/Ptprc/H2-T23	17
Trim25/Trappc9/Rps3/Malt1/Prdx3/Cd84/Traf1/Ep300/Prkcb/Ikbkb/Kras/Npm1/Ddrgk1/Myd88/Trim12a/Irak3/Chuk	17
Jun/Cacybp/Id3/Pds5a/Orc3/Bcar3/Npm1/Pcna/Atrx/Ankrd17/Faf1/Nucks1/Donson/Esco1	14
Hspd1/Dhx36/Nmi/Setd2/Zc3hav1/Stat1/Chuk	7
Hspa1a/Hspa1b/Hsp90ab1/Hsp90aa1/Dnaja1/Pde4b/Ier5/Dhx36/Arrb2/Daxx/Eif2b2/Xylt1/Eif2ak3/Trp53inp1/Rnf34/Plac8/Dnajc3/Hspa4	18
Jun/Hspd1/Jund/Foxp1/Syk/Kmt2e/Ifngr1/Myo18a/Adam10/Sbno2/Nmi/Irf4/Cd84/Tspan32/Stap1/Vamp2/Myd88/Il4ra/Rabgef1/Cnr2/Cd200/Dock2/Rhoh/Relb	24
Ubb/Syk/Itga4/Myo1c/Cenpa/Ccdc186/Tcirg1/Cep83/Exoc4/Map2k1/Cd84/Nudc/Xpo1/Snapin/Npm1/Map4/Nup88/Rrs1/Ube2b/Nsfl1c/Kpnb1/Mecp2/Map4k2/Pcm1/Tmed9/Preb/Il4ra/Rabgef1/Ctnnb1/Stk11/Eml4/Rab3ga	33
Foxp1/Smad7/Rc3h2/Malt1/Irf4/Nfkbid/H2-M3/Gadd45g/Ly9/Il4ra/Ccr7/Cd81/Relb/Zc3h12a/Bcl6	15
Pgap1/Zdhhc3/Atg12/Zdhhc18/Pik3c3/Ugcg/Nmt2/Dbi/Wipi2/Golga7/Dpm1/Apoe/Rab3gap1	13
Macf1/Nap1l1/Zeb2/Ifrd1/Rnf6/Dhx36/Bcl11a/Hes6/Map2k1/Myc/Ist1/Cux1/Atp5g1/Zfyve27/Dip2b/Golga4/Dnajb11/Eif2b2/Snw1/Mecp2/Ppp3ca/Pcm1/Tspo/Bin1/Il6st/Bhlhe40/Ctnnb1/Pten/Stk11/Trim11/Dbnl/Apoe/Chd7/	35
Trim59/Sirt1/Nfkbid/Per1/Zmynd11/Stat1/Rhoh/Riok3/Zc3h12a	9
Kmt2e/Tmod3/Crebbp/Cdc25b/Npm1/Camk2d/Mecp2/Anp32b/Rcc2/Ankrd17/Ccnd3/Paf1/Lmnb1/Ccnd2	14
Ssh2/Gpx1/Mul1/Capg/Pcid2/Sptbn1/S1pr1/Tmod3/Fkbp4/Naa10/Parp1/Arrb2/Npm1/Map4/Inpp5k/Ghitm/Arfgef1/Tspo/Atrx/Higd1a/Pik3r1/Tnks2/Flii/Ik/Dnm2/Flcn/Eml4/Pml/Rad21/Myadm	30
Syk/Rasal3/Itch/Cblb/Btla/Cd55/Cd81/Ptprc	8
Hsp90aa1/Smad7/Syk/Rc3h2/Pcid2/Inpp5d/Hspb1/Sart1/Malt1/Zfp36l2/Nfkbid/Sh2b3/Ptpn2/Clptm1/Il4ra/Ccr7/Rhoh/Zc3h12a/Ptprc/Bcl6	20
Hsp90ab1/Hsp90aa1/Macf1/Mul1/Itga4/Zeb2/S1pr1/Ifrd1/Rnf6/Bcl11a/Ist1/Atp5g1/Zfyve27/Dip2b/Golga4/Eif2b2/Iqgap1/Mecp2/Ctnnb1/Stk11/Dnm2/Dbnl/Cyfip2/Apoe/Gdi1	25
Hspa8/Hspd1/Smad7/Syk/Arid5a/Clec2d/Malt1/Dhx36/Cr1l/Cd84/Arrb2/H2-Q7/Fbxo38/Dennd1b/H2-M3/Cd55/Stap1/H2-Q6/Il4ra/Rabgef1/Bst2/H2-Q4/Cd81/Ptprc/H2-T23/Bcl6	26
Kmt2e/Arid4b/Satb1/Crebbp/Snrpd3/Setd2/Myc/Cmtr1/Parp1/Sirt1/Mtf2/Kmt2c/Ogt/Snw1/Mecp2/Ehmt1/Iws1/Atrx/Pax5/Rtf1/Zc3h13/Ctnnb1/Paf1/Kmt2a/Nsd1/Chtop/Prmt1	27
Hsph1/Syk/H2-Ab1/Malt1/Rasal3/Nfkbid/Cd55/Il4ra/Ccr7/Cd81/Ptprc	11
Klf4/Kmt2e/Casp2/Dgkz/Crebbp/Bcl2/Nfatc1/Zfp655/Camk2d/Anp32b/Ppp3ca/Ankrd17/Ccnd3/Sde2/Pten/Rps6kb1/Paf1/Pml/Nasp/Gpr132/Ccnd2	21
Ubb/Jun/Hsp90ab1/Fos/Hsph1/Hspd1/Mef2c/Atf2/Pmaip1/Casp2/Prdx3/Egr1/Nr3c1/Tmem259/Bcl2/Parp1/Sirt1/Arrb2/Daxx/Atp5g1/Ikbkb/Fis1/Kras/Npm1/Mecp2/Rock1/Ctnnb1/Cd200/Bcl2l11/Apoe/Srpk2	31
Rnf125/Zc3hav1/Ankrd17/C1qbp/Usp15/Riok3/Pum2	7
Neat1/Rps19/Ubtf/Smarca5/Sirt1/Ets1/Daxx/Usp36/Nsfl1c/Reep3/Pes1/Bin1/Serbp1/Akap8l/Pml/Srpk2	16
Sptbn1/Adam10/Map2k1/Csnk2a1/Rap1a/Camk2d/Pgrmc1/Epb41/Rer1/Rhog/Atp2c1/Pik3r1/Cltc/Ppp2r5a/Vti1b/Numa1	16
Shisa5/Rps3/Tpt1/Pmaip1/Casp2/Myc/Bcl2/Sirt1/Ep300/Snw1/Pik3r1/Sfn/Pml/Bcl2l11	14
Dnaja1/Foxp1/Rnf6/Fkbp4/Sirt1/Daxx/Ep300/Arid1a	8
Klf4/Mef2c/Foxp1/Nfatc3/Nr3c1/Parp1/Sirt1/Foxo1/Nfatc1/Camk2d/Cdk9/Ppp3ca/Rock1/Tbce/P2rx4	15
Sertad1/Foxp1/Mul1/Ifrd1/Rnf6/Cited2/Bcl11a/Bcl2/Sirt1/Ptprj/Dip2b/Eno1/Mecp2/Dnajb2/Mndal/Pten/Stk11/Bst2/Smarca2/Plac8/Flcn/Pml/Rrad/Bcl6	24
Tmed2/Nfe2l2/Ptpn2/Eif2ak3/Ptpn1	5
Laptm5/Itch/Mylip/Apoe/Ptpn1	5
Dnaja1/Dusp1/Hipk3/Ptprj/Sh2b3/Inpp5k/Stk38/Irak3/Apoe/Ptpn1	10
Mef2c/Foxp1/Nr3c1/Parp1/Sirt1/Foxo1/Camk2d/Cdk9/Ppp3ca/Rock1/P2rx4	11
Hsp90aa1/Mul1/Rnf125/Itch/Tspan32/Zc3hav1/C1qbp/Stat1/Pml/Riok3/Zc3h12a	11
Hspa8/Cfl2/Nsf/Capg/Sptbn1/Tmod3/Myc/Smarce1/Snapin/Smarcc1/Mical1/Cebpg/Plek/Arid1a/Uvrag/Flii/Map1lc3a/Irak3/Scaf8/Setx/Eml4	21
Macf1/Nsf/Laptm5/Sptbn1/Exoc4/Vamp3/Golga4/Vamp2/Atp2c1/Dnm2/Golga7/Vti1b/Sys1	13
Klf2/Foxp1/Tmod3/Cited2/Pabpc4/Ep300/Sh2b3/Nrros/Rabgap1l/Ptbp3/Fli1/Bcl6	12
Hspa1b/Nr4a1/Gapdh/Klf4/Gpx1/Plaur/Pcid2/Arl6ip1/Arrb2/Siah2/Csnk2a1/Akt2/Mical1/Herpud1/Bin1/Fnip1/Rnf34/Sfn/Fcmr	19
Macf1/Dynll1/Zeb2/Hspb1/Dhx36/Bcl11a/Map2k1/Sirt1/Dynlt1c/Ist1/Nfe2l2/Cux1/Ep300/Atp5g1/Zfyve27/Rala/Ikbkb/Fbxo38/Golga4/Eif2b2/Rap1a/Iqgap1/Kidins220/Plekhm1/Tmem30a/Pik3r1/Ccr7/Stk11/Dnm2/Setx/Dbnl/	34
Dnaja1/Dusp1/Hspb1/Gadd45b/Hipk3/Sirt1/Ptprj/Cblb/Sh2b3/Npm1/Inpp5k/Ptpn2/Gadd45g/Stk38/Pten/Irak3/Ptprc/Dnajc3/Apoe/Ptpn1	20
Pcid2/Iws1/Poldip3/Eny2/Pabpn1/Nxf1	6
Klf2/Ppp1r15a/Klf4/Rhob/Atf2/Hspb1/Sp100/Sirt1/Ets1/Nfe2l2/Tcf4/Pde3b/Krit1/Prkcb/Sh2b3/Jak1/Mecp2/Rock1/Sars/Pgk1/Stat1/Ctnnb1/Sp1/Flcn/Pml/Zc3h12a	26
Hspd1/Foxp1/Syk/Laptm5/Rel/Arrb2/Nfkb1/C1qbp/Ccr7/Irak3	10

Hsph1/Foxp1/Gpx1/Hspb1/Aldh2/Bcl2/Parp1/Sirt1/Nfe2l2/Zfp622/Ctnnb1/Psap/Pml	13
Jun/Hspd1/Jund/Foxp1/Syk/Ifngr1/Myo18a/Sbno2/Nmi/Cd84/Stap1/Il4ra/Cd200	13
Klf4/Mef2c/Foxp1/Nr3c1/Parp1/Sirt1/Foxo1/Nfatc1/Camk2d/Cdk9/Ppp3ca/Rock1/P2rx4	13
Nfkbia/Sp100/Os9/Ankrd13c/Chchd10/Nrros/Sri/Tspo/Rer1/Vps13a/Tnrc6a/Faf1/Pml	13
Macf1/Zeb2/Bcl11a/Map2k1/Ist1/Atp5g1/Zfyve27/Golga4/Eif2b2/Stk11/Dbnl/Apoe/Gdi1	13
Hspd1/Dhx36/Nmi/Setd2/Zc3hav1/Stat1/Chuk	7
Laptm5/Itch/Pik3r4/Mylip/Uvrag/Apoe/Ptpn1	7
Smad7/Rc3h2/Malt1/Irf4/Nfkbid/Ly9/Zc3h12a	7
Hspa1a/Hspa1b/Smad7/Arih1/Csnk1d/Gabarap/Ddrgk1/Herpud1/Zfand2a/Dnajb2/Chfr/Arih2	12
Mef2c/Nr3c1/Parp1/Sirt1/Camk2d/Cdk9/Ppp3ca/Rock1	8
Pde4b/Nfkbia/Mef2c/Zfp36/Syk/Arid5a/Malt1/Ccdc47/Ppp1r15b/Sbno2/Cd84/Ogt/Cd55/Stap1/Nfkb1/Eif2ak3/Cd86/Stat1/Myd88/Trim12a/Paf1/Pabpn1/Tmco1/Zc3h12a/Bcl2l11/Prpf8/Ly86/Vim	28
Jun/Nr4a1/Klf4/Macf1/Mef2c/Rhob/Foxp1/Gpx1/Itga4/Zeb2/Hspb1/Sp100/Lamtor2/Sirt1/Ets1/Nfe2l2/Krit1/Bsg/Iqgap1/Mecp2/Rock1/Rcc2/C1qbp/Ythdf3/Pten/Sp1/Pml/Zc3h12a/Prcp/Apoe/P2rx4/Iqsec1/Calr/Atp5a1	34
Klf2/Inpp5d/Cd84/Arrb2/Bank1/Rabgef1/Cd200/Irak3/Zc3h12a	9
Dnaja1/Hsph1/Klf7/Sp100/Os9/Snx12/Pde3b/Inpp5k/Ogt/Ppp3ca/Derl2/Eny2/Cd200/Ppp2ca/Apoe/Gdi1	16
Pde4b/Wnk1/Syk/Rpl13a/Rps19/Itga4/Dusp1/S1pr1/Gpsm3/Ano6/Adam10/Ptprj/Bsg/Stap1/Rock1/Rhog/C1qbp/Dock8/Myd88/Cxcr5/Rabgef1/Ccr7/Cnr2/Cd200/Rhoh/Cd81/Msn/P2rx4/Calr/Il17ra	30
Hsp90aa1/Wnk1/Nfkbia/Hspd1/Syk/Rps19/S1pr1/Gpsm3/Ano6/Hspb1/Adam10/Matr3/Nmi/Ets1/Ptprj/H2-M3/Akt2/Zc3hav1/Stap1/Pspc1/Ankrd17/C1qbp/Mndal/Usp15/Ccr7/Trim12a/Cd81/Riok3/Pum2/Ly86/P2rx4/Calr/Il1	33
Fos/Foxp1/Junb/Clec2d/Inpp5d/Klf10/Tcirg1/Sbno2/Nfatc1/Pik3r1/Ctnnb1/Cd81/Chuk/Tob2	14
Nr4a1/Klf4/Mef2c/Gpx1/Hspb1/Sirt1/Ets1/Nfe2l2/Mecp2/Sp1/Prcp/Apoe/P2rx4/Atp5a1	14
Hspa1b/Ppp1r15a/Nr4a1/Gapdh/Wnk1/Klf4/Gpx1/Kdm5a/Plaur/Pcid2/Bod1l/Neil1/Tmed2/Arl6ip1/Arrb2/Ikbkb/Siah2/Csnk2a1/Akt2/Mical1/Herpud1/Arfgef1/Rock1/Rcc2/Bin1/Fnip1/Birc6/Rnf34/Sfn/Fcmr	30
Hspa8/Hspd1/Smad7/Arid5a/Malt1/Cr1l/H2-Q7/Fbxo38/Dennd1b/H2-M3/Cd55/H2-Q6/H2-Q4/Cd81/Ptprc/H2-T23	16
Irs2/Pmaip1/Nr3c1/Sirt1/Foxo1/Ep300/Akt2/Inpp5k/Ogt/Ptpn2/Usp7/Supt20/Ncoa2/Stk11/Ranbp2	15
Mef2c/Nr3c1/Parp1/Sirt1/Camk2d/Cdk9/Ppp3ca/Rock1	8
Hsph1/Dusp1/Rell1/Gadd45b/Gadd45g/Per1/Zc3h12a/Prmt1	8
Pgap1/Tmed5/Vapa/Tmed2/Sec24b/Arcn1/Ergic3/Copb2/Tmed9/Preb/Ergic2/Trappc6b/Copa/Sec24c	14
Mef2c/Naca/Plekho1/Egr1/Myc/Bcl2/Arpc5/Nfe2l2/Iqgap1/Camk2d/Rock1/Il6st/Rps6kb1/Gna13	14
Mef2c/Foxp1/Nr3c1/Parp1/Sirt1/Foxo1/Camk2d/Cdk9/Ppp3ca/Rock1/P2rx4	11
Pdcd2/Dhx36/N4bp2l2/Nfe2l2/Fnip1/Hspa9/Flcn	7
Tmem259/Sirt1/Ptpn2/Herpud1/Eif2ak3/Pik3r1/Atf6	7
Mul1/Arnt/Uba2/Sae1/Gnl3	5
Zfp36/Tnrc6a/Cnot1/Tnrc6c/Tnrc6b	5
Gapdh/Atf3/Irs2/Pmaip1/Nr3c1/Myc/Sirt1/Foxo1/Ep300/Man2b1/Eno1/Akt2/Inpp5k/Ogt/Ptpn2/Usp7/Supt20/Pgk1/Ncoa2/Nisch/Pik3r1/Stk11/Ranbp2	23
Kmt2e/Tmod3/Crebbp/Cdc25b/Camk2d/Mecp2/Anp32b/Rcc2/Ankrd17/Ccnd3/Lmnb1/Ccnd2	12
Hspd1/Foxp1/Smad7/Syk/Rc3h2/Malt1/Sbno2/Nmi/Irf4/Cd84/Nfkbid/H2-M3/Gadd45g/Vamp2/Ly9/Myd88/Cd19/Il4ra/Rabgef1/Ccr7/Dock2/Cd81/Relb/Zc3h12a/Ptprc/Bcl6/Dock11	27
Fkbp4/Fkbp1a/Nktr/Ppig/Ppwd1/Ranbp2	6
Ddrgk1/Ptpn2/Pik3r1/Atf6/Crebrf/Ptpn1	6
Hspd1/Foxp1/Syk/Laptm5/Rel/Arrb2/Nfkb1/C1qbp/Ccr7/Irak3	10
Nfkbia/Pik3ap1/Hspd1/Foxp1/Zfp36/Gpx1/Rps19/Tnfaip8l2/Gpsm3/Sbno2/Nmi/Aldh2/Ets1/Celf1/Siglecg/Ptpn2/Stap1/Nfkb1/Myd88/Rabgef1/Ccr7/Cnr2/Cd200/Cd81/Socs3/Apoe/Bcl6/Il17ra	28
Tmed5/Rab43/Lysmd3/Myo18a/Map2k1/Csnk1d/Tmed2/Nsfl1c/Arfgef1/Tmed9/Cltc/Akap9/Dnm2/Vrk1/Vti1b	15
Foxp1/Smad7/Arid5a/Laptm5/Malt1/Dhx36/Sirt1/Siglecg/Dennd1b/Dnajb9/Cd55/Cd86/Il4ra/Bst2/Irak3/Cd81/Ptprc/Bcl6	18
Mef2c/Foxp1/Akap13/Nfatc3/Nr3c1/Parp1/Sirt1/Foxo1/Camk2d/Cdk9/Ppp3ca/Rock1/P2rx4	13
Irs2/Ptges3/Pmaip1/B3gnt7/Nr3c1/Sirt1/Foxo1/Rb1cc1/Ep300/Got1/Akt2/Inpp5k/Ogt/Coq2/Ptpn2/Usp7/Mecp2/Plek/B3gnt2/Supt20/Ncoa2/Il6st/Pten/Stk11/Ranbp2	25
Hsp90ab1/Ppp1r15a/Mef2c/Bod1l/Ppp1r15b/Ikbkb/Smg5/Iqgap1/Inpp5k/Rock1/Plek/Ppp2r5a/Ppp2ca/Ptprc/Hsp90b1	15
Mul1/Bcl11a/Egr1/Arnt/Uba2/Ctnnb1/Sae1/Gnl3	8
Smad7/Rc3h2/Itch/Cblb/Btla/Il4ra/Zc3h12a/Bcl6	8
Klf4/Mul1/Sh2b3/Ptpn2/Stap1/Il6st/Ythdf3/Rabgef1/Irak3/Ptprc	10
Pde4b/Atp2b1/Ms4a1/Nfatc3/Ano6/Ahnak/Diaph1/Mcur1/Bcl2/Cd84/Arrb2/Nfatc1/Prkcb/Fkbp1a/Camk2d/Inpp5k/Sri/Ppp3ca/Tspo/Epb41/Bin1/Atp2c1/Cd19/Dbi/Ctnnb1/Ppp2ca/Tmco1/Pml/Ptprc/Rrad/Chd7/P2rx4/Cherp	33
Zfp36/Gpx1/Mul1/Rps19/Dusp1/Tnfaip8l2/Clec2d/Rnf125/Chd8/Nmi/St6gal1/Ets1/Itch/Arrb2/Siglecg/Sh2b3/Ptpn2/Stap1/Nfkb1/Xylt1/C1qbp/Ythdf3/Usp15/Rabgef1/Pten/Cnr2/Cd200/Irak3/Riok3/Socs3/Apoe	31
Jun/Hsp90ab1/Hsph1/Hspd1/Mef2c/Atf2/Pmaip1/Casp2/Prdx3/Egr1/Nr3c1/Bcl2/Parp1/Sirt1/Arrb2/Fis1/Kras/Npm1/Mecp2/Rock1/Ctnnb1/Bcl2l11/Apoe/Srpk2	24
Mef2c/Naca/Tsc22d3/Bcl2/Ctnnb1/Rps6kb1/Usp19	7
Zfp36/Fam172a/Smchd1/Limd1/Map2k1/Ubtf/Smarca5/Ddx6/Sirt1/Celf1/Celf2/Mecp2/Nfkb1/Ppp3ca/Tnrc6a/Cnot1/Cnot2/Eif4e2/Tnrc6c/Zc3h12a/Pum2/Tnrc6b	22
Hspd1/Foxp1/Smad7/Syk/Rc3h2/Malt1/Sbno2/Nmi/Irf4/Cd84/Nfkbid/H2-M3/Gadd45g/Vamp2/Ly9/Myd88/Cd19/Il4ra/Rabgef1/Ccr7/Dock2/Cd81/Relb/Zc3h12a/Ptprc/Bcl6/Dock11	27
Pgap1/Zdhhc3/Atg12/Zdhhc18/Pik3c3/Ugcg/Nmt2/Dbi/Wipi2/Golga7/Dpm1/Rab3gap1	12
Rps21/Fcf1/Npm1/Abt1/Rrs1/Pes1	6
Sart3/Usp36/Usp7/Usp15/Eny2/Usp16	6
Hspd1/Dhx36/Setd2/Zc3hav1/Stat1/Chuk	6
Hsp90aa1/Smad7/Syk/Rc3h2/Hspb1/Sart1/Malt1/Nfkbid/Sh2b3/Ptpn2/Clptm1/Il4ra/Ccr7/Rhoh/Zc3h12a/Ptprc/Bcl6	17
Foxp1/Hspb1/Malt1/Egr1/1810058I24Rik/Arrb2/Ifnar1/Myd88/Mndal/Ccr7/Pml/Zc3h12a	12
Klf4/Mef2c/Hspb1/Sirt1/Ets1/Nfe2l2/Mecp2/Sp1/Prcp/Apoe/P2rx4/Atp5a1	12
Dock8/Ccr7/Dock2/Cd81/Msn	5
Ptges3/Nr3c1/Arid1a/Per1/Ncoa2	5
Hsp90ab1/Smad7/Zeb2/Cited2/Crebbp/Sirt1/Ep300/Got1/Nrros/Snw1/Stk11/Dnm2/Flcn/Glg1	14
Hspa1a/Hspa1b/Smad7/Arih1/Tmem259/Csnk1d/Gabarap/Ddrgk1/Herpud1/Zfand2a/Dnajb2/Chfr/Arih2	13
Klf2/Hspd1/Syk/Arid5a/Laptm5/Inpp5d/Cd84/Arrb2/Bsg/Bank1/Zbtb20/Myd88/Rabgef1/Cd200/Irak3/Zc3h12a/Il17ra	17
Hsp90ab1/Ppp1r15a/Mef2c/Ppp1r15b/Plek/Ppp2ca/Ptprc	7
Rc3h2/Rps3/Laptm5/Lims1/Crebbp/Nmi/Ep300/Nfat5/Cd86/Calr	10
Dnaja1/Rps7/Smad7/Rps3/Arrb2/Ogt/Ivns1abp/Tspo/Usp4/Dnajb2/Ctnnb1/Trip12	12
Dnaja1/Syk/Akap13/Dusp1/Rps3/Zeb2/Hipk3/Ptprj/Kras/Sh2b3/Iqgap1/Inpp5k/Stk38/Map4k2/Ccr7/Irak3/Ptprc/Apoe/Ptpn1	19
Klf4/Mef2c/Rhob/Dusp1/Tmod3/Cited2/Sp100/Bcl2/Clic4/Ptprj/Nfe2l2/Krit1/Mecp2/Stap1/Bin1/Trp53inp1/Nisch/Rabgef1/Pten/Cd200/Bst2/Flcn/Gna13/Apoe/Osbpl8/Calr	26
Hsp90ab1/Rps7/Azin1/Snx12/Csnk2a1/Ddrgk1/Ctsa/Smarcc1/Ogt/Usp7/Irak3/Mycbp2/Pml/Usp19	14
Hsph1/Rhob/Foxp1/Gpx1/Rps3/Prdx3/Kdm6b/Ppp1r15b/Bcl2/Sirt1/Nfe2l2/Oser1/Psap/Setx	14
Mef2c/Foxp1/Akap13/Nfatc3/Nr3c1/Parp1/Sirt1/Foxo1/Camk2d/Cdk9/Ppp3ca/Rock1/P2rx4	13
Irs2/Pmaip1/Myc/Sirt1/Foxo1/Zbtb20/Akt2/Ptpn2/Nfkb1/Supt20/Stk11	11
Syk/H2-Ab1/Rps3/Laptm5/Dhps/Rasal3/Itch/Cblb/Tnfrsf13c/Btla/Cd55/Cd86/Il6st/Ctnnb1/Ccr7/Cd81/Peli1/Ptprc	18
Irs2/Pmaip1/Egr1/Nr3c1/Map2k1/Myc/Erlin1/Parp1/Sirt1/Foxo1/Ep300/Zbtb20/Eno1/Akt2/Inpp5k/Ogt/Ptpn2/Usp7/Nfkb1/Plek/Eif2ak3/Supt20/Ncoa2/Dbi/Stk11/Lpgat1/Ranbp2/Flcn/Apoe	29
Laptm5/Dgkz/Elf1/Ptprj/Cblb/Ptpn2	6
Hspa8/Gapdh/Naca/Bcl2/Sirt1/Arrb2/Nfe2l2/Npm1/Camk2d/Pik3r1/Pten/Zc3h12a	12
Vps37b/Trim25/Top2b/Trim59/Vapa/Bcl2/Bsg/Zc3hav1/Inpp5k/Fam111a/Larp1/Bst2/Trim12a/Pml/Trim11/Srpk2	16
Gpx1/Arid4b/Clic4/Jmjd1c/Sec24b/Ikbkb/Rap1a/Ccm2/Whrn/Fndc3a/Rock1/Atrx/Myd88/Sidt2/Il6st/Sfn/Akap9/Psap/Slc4a7/Msn/Myadm/Ccdc88c/Vim	23
Mul1/Crebbp/Sirt1/Fbxl3/Ep300/Npm1/Mylip/Chfr/Ccdc88c	9
Hsp90ab1/Ppp1r15a/Bod1l/Ppp1r15b/Ikbkb/Rock1/Ppp2ca/Ptprc/Hsp90b1	9
Klf4/Pcid2/Casp2/Dgkz/Bcl2/Zfp36l2/Zfp655/Topbp1/Chfr/Sde2/Pten/Ik/Pml/Rad21/Donson/Gpr132/Foxn3	17
Hsp90ab1/Smad7/Zeb2/Cited2/Crebbp/Sirt1/Ep300/Got1/Nrros/Snw1/Stk11/Dnm2/Flcn/Glg1	14
Pcid2/Dhx36/Senp6/Naa10/Rnf4/Myc/Parp1/Smg5/Smg1/Atrx/Chfr/Tnks2/Ctnnb1/Ik/Pml/Rad21/Gnl3/Numa1	18
Macf1/Zeb2/Ifrd1/Rnf6/Bcl11a/Map2k1/Ist1/Atp5g1/Zfyve27/Dip2b/Golga4/Eif2b2/Pten/Stk11/Dbnl/Apoe/Vim/Gdi1	18
Cenpa/Tpp1/Setd2/Daxx/Rcc2/Atrx/Tnks2/Ik/Pml/Rad21/Gnl3/Nipbl	12
Hspa8/Hsp90ab1/Syk/Clec2d/Cr1l/Arrb2/H2-Q7/H2-M3/Cd55/Stap1/H2-Q6/H2-Q4/Bcl2l11/Ptprc/H2-T23	15
Mef2c/Foxp1/Akap13/Nfatc3/Nr3c1/Parp1/Sirt1/Foxo1/Camk2d/Cdk9/Ppp3ca/Rock1/P2rx4	13
Hspd1/Syk/Arid5a/Ifngr1/Hspb1/Cd2/Cd84/Ptprj/Zbtb20/Pik3r1/Myd88/Ccr7/Ptprc	13
Irs2/Pmaip1/Sirt1/Foxo1/Akt2/Ptpn2/Supt20/Stk11	8
Mef2c/Irs2/Bcl2/Tnfrsf13c/Cd81/Peli1/Ptprc/Bcl6	8
Bod1l/Pcna/Cdk9/Atrx/Fam111a/Tonsl/Nucks1/Donson	8
Smad7/Rc3h2/Malt1/Nfkbid/Cd55/Il4ra/Ccr7/Cd81/Zc3h12a/Bcl6	10
Ptges3/Nr3c1/Arid1a/Per1/Ncoa2	5
Myc/Smarce1/Smarcc1/Cebpg/Arid1a	5
Ppp1r15a/Dusp1/Ppp1r15b/Ppp3ca/Ppp2r2a	5
Dhx36/Daxx/Zfand2a/Zc3h12a/Slc38a2	5
Klf2/Klf4/Mef2c/Nfe2l2/Mtss1	5
Birc3/Nmi/Ube2d1/Birc2/Gabarap	5
Atf2/Pdcd2/Cited2/Dhx36/N4bp2l2/Bcl2/Siglecg/Nfe2l2/Prrc2c/Rrs1/Fnip1/Hspa9/Chd2/Flcn/Samd9l/Ptprc/Pld4	17
Dnaja1/Traf4/Atf2/Gadd45b/Hipk3/Itch/Rb1cc1/Traf1/Gadd45g/Nfkb1/Map4k2/Zfp622/Per1/Zmynd11/Myd88/Spag9/Lmnb1	17
Cfl2/Ssh2/Capg/Myo1c/Sptbn1/Tmod3/Diaph1/Pstpip1/Arpc5/Fchsd2/Mical1/Arfgef1/Plek/Hax1/Bin1/Mtss1/Flii/Dbnl/Cyfip2/Myadm	20
Top2b/Rps3/Pcid2/Pds5a/Naa50/Naa10/Arl8b/Nudc/Csnk2a1/Rrs1/Ube2b/Kpnb1/Rcc2/Atrx/Tlk1/Chfr/Top3b/Uvrag/Ctnnb1/Ik/Akap8l/Eml4/Rad21/Pum2/Esco1/Nipbl/Numa1	27
Atp5e/Map2k1/Atp5c1/Myc/Parp1/Atp5g1/Eno1/Flcn/Atp5a1	9
Syk/Irf4/Bcl2/Ptpn2/Ly9/Ccr7/Dock2/Stk11/Ptprc	9
Jun/Fos/Hsph1/Rhob/Foxp1/Rps3/Prdx3/Kdm6b/Parp1/Sirt1/Nfe2l2/Oser1/Psap/Setx/Chuk	15
Mef2c/Foxp1/Tsc22d3/Irs2/Plekho2/St6gal1/Myc/Sirt1/Nfkbid/Fnip1/Dock8/Pten/Fcmr/Ccr7/Bcl2l11	15
Smad7/Arid5a/Laptm5/Malt1/Dhx36/Sirt1/Dennd1b/Cd55/Bst2/Irak3/Cd81/Bcl6	12
Zc3hav1/Ankrd17/Usp15/Pum2	4
Laptm5/Itch/Apoe/Ptpn1	4
Nfkbia/Pik3ap1/Hspd1/Rnf125/Nmi/Irf4/Arrb2/Zc3hav1/Ankrd17/Cd86/C1qbp/Myd88/Usp15/Irak3/Peli1/Riok3/Pum2	17
H13/Aup1/Os9/Herpud1/Derl2/Sel1l	6
Zfp36/Ddx6/Tnrc6a/Eif4e2/Tnrc6c/Tnrc6b	6
Rnf125/Zc3hav1/Ankrd17/C1qbp/Usp15/Pum2	6
H13/Aup1/Os9/Herpud1/Derl2/Sel1l	6
Smad7/Arid5a/Rc3h2/Malt1/Nfkbid/Zc3h12a	6
Hspa8/Top2b/Pcbp1/Vapa/Bcl2/Zc3hav1/Inpp5k/Atg16l2/Fam111a/Larp1/Bst2/Zc3h12a/Nucks1/Srpk2	14
Ifi30/H2-Ab1/Arl8b/H2-Q7/Wdfy4/H2-Eb2/H2-M3/H2-Q6/Psap/Ccr7/H2-Q4/Relb/H2-T23/Calr	14
Rhob/Lrrc8c/Crebbp/Dhx36/Egr1/Rnf4/Parp1/Sirt1/Slc12a6/Ep300/Got1/Npm1/Akt2/Pcna/Rcsd1/Trp53inp1/Pik3r1/Sde2/Gtf2h5/Ctnnb1/Pten/Stk11/Relb/Nucks1/Nipbl/Ccnd2	26
Rhob/Lrrc8c/Crebbp/Dhx36/Egr1/Rnf4/Parp1/Sirt1/Slc12a6/Ep300/Got1/Npm1/Akt2/Pcna/Rcsd1/Trp53inp1/Pik3r1/Sde2/Gtf2h5/Ctnnb1/Pten/Stk11/Relb/Nucks1/Nipbl/Ccnd2	26
Hspa8/Ano6/Myo18a/Ptprj/Rap1a/Stap1/Ccr7/Dock2/Dnm2/Ptprc/Calr	11
Rc3h2/Pcid2/Cited2/Bcl2/Nfkb2/Kmt2a/Samd9l/Bcl2l11	8
Gpx1/Klf7/Irs2/Ccdc186/Egr1/Sirt1/Nadk/Pde3b/Prkcb/Eif2b2/Zbtb20/Sri/Ogt/Nfkb1/Ppp3ca/Vamp2/Sidt2/Eny2/Ppp2ca	19
Hspd1/Syk/Arid5a/Ifngr1/Hspb1/Cd2/Cd84/Ptprj/Zbtb20/Pik3r1/Myd88/Ccr7/Ptprc	13
Mef2c/Foxp1/Tsc22d3/Irs2/St6gal1/Myc/Sirt1/Nfkbid/Fnip1/Dock8/Pten/Fcmr/Ccr7	13
Top2b/Pcid2/Pds5a/Naa50/Naa10/Nudc/Rrs1/Kpnb1/Atrx/Chfr/Ctnnb1/Ik/Akap8l/Eml4/Rad21/Esco1/Nipbl/Numa1	18
Hsp90ab1/Hsp90aa1/Macf1/Mef2c/Foxp1/Klf7/Itga4/Top2b/Rpl24/Zeb2/Ifrd1/Rnf6/Bcl11a/Map2k1/Bcl2/Ist1/Atp5g1/Zfyve27/Dip2b/Golga4/Bsg/Eif2b2/Grcc10/B3gnt2/Rhog/Tbce/Pten/Stk11/Dnm2/Rhoh/Mycbp2/Dbnl/Ap	35
Klf4/Plaur/Irf4/Myc/Hipk3/Parp1/Ep300/Ddrgk1/Ctnnb1	9
Gpx1/Cept1/Pgap1/Inpp5d/Dgkz/Gpcpd1/Sirt1/Pnpla7/Pik3c3/Scd1/Mboat7/Smg1/Chkb/Inpp5k/Mecp2/Plek/Hadha/Pik3r1/Il6st/Sel1l/Abhd16a/Dbi/Pten/Etnk1/Lpgat1/Dpm1/Socs3/Apoe/Dgka	29
Hsp90aa1/Hspd1/Syk/Rps19/Matr3/Nmi/Fbxo38/H2-M3/Zc3hav1/Pspc1/Ankrd17/Mndal/Usp15/Trim12a/Riok3/Pum2/Ly86	17
Jun/Fos/Hsph1/Rhob/Foxp1/Gpx1/Rps3/Prdx3/Kdm6b/Ppp1r15b/Bcl2/Parp1/Sirt1/Nfe2l2/Oser1/Psap/Setx/Chuk	18
Hsp90ab1/Ppp1r15a/Mef2c/Bod1l/Ppp1r15b/Ikbkb/Rock1/Plek/Ppp2ca/Ptprc/Hsp90b1	11
Ubb/Mul1/Pmaip1/Prdx3/Myc/Bcl2/Parp1/Atp5g1/Got1/Akt2/Tspo	11
Rps21/Fcf1/Bms1/Pop7/Npm1/Abt1/Fip1l1/Rrs1/Nhp2/Zc3h12a	10
Foxp1/Hspb1/Malt1/Egr1/1810058I24Rik/Arrb2/Ifnar1/Myd88/Mndal/Ccr7/Pml/Zc3h12a	12
Pik3ap1/Rnf125/Irf4/Arrb2/Zc3hav1/Ankrd17/C1qbp/Usp15/Irak3/Peli1/Riok3/Pum2	12
Gapdh/Atf3/Irs2/Pmaip1/Nr3c1/Myc/Sirt1/Foxo1/Ep300/Man2b1/Eno1/Akt2/Inpp5k/Ogt/Ptpn2/Usp7/Supt20/Pgk1/Ncoa2/Nisch/Pik3r1/Stk11/Ranbp2	23
Cept1/Pgap1/Inpp5d/Dgkz/Gpcpd1/Pnpla7/Pik3c3/Mboat7/Smg1/Chkb/Inpp5k/Mecp2/Plek/Hadha/Pik3r1/Abhd16a/Dbi/Pten/Etnk1/Lpgat1/Dpm1/Socs3/Dgka	23
Hsp90ab1/Syk/Akap13/Rps3/Zeb2/Map2k1/Cab39/Sirt1/Kras/Iqgap1/Map4k2/Higd1a/Ccnd3/Ccr7/Stk11/Ppp2ca/Ptprc/Ptpn1/Ccnd2	19
Macf1/Ifrd1/Rnf6/Bcl11a/Ist1/Atp5g1/Zfyve27/Dip2b/Golga4/Eif2b2/Dnm2/Dbnl/Apoe/Gdi1	14

Klf4/Kmt2e/Casp2/Dgkz/Crebbp/Bcl2/Zfp655/Anp32b/Ankrd17/Ccnd3/Sde2/Pten/Pml/Ccnd2	14
Hsp90ab1/Junb/Itga4/Dazap1/Xist/Prdx3/Cited2/Setd2/Map2k1/Arnt/Tmed2/Birc2/Bptf/Arid1a/Birc6/Sp1/Socs3	17
Klf2/Hspd1/Syk/Arid5a/Laptm5/Inpp5d/Cd84/Arrb2/Bsg/Bank1/Zbtb20/Myd88/Rabgef1/Cd200/Irak3/Zc3h12a/Il17ra	17
Foxp1/Prkcb/Stap1/Cd19/Cd81	5
Chchd2/Map2k1/Eno1/Ogt/Usp19	5
Hsph1/Gpx1/Hspb1/Sirt1/Nfe2l2	5
Smad7/Rc3h2/Malt1/Nfkbid/Zc3h12a	5
Wnk1/Itga4/Dusp1/Gpsm3/Ano6/Adam10/Ptprj/Stap1/Rhog/C1qbp/Dock8/Myd88/Rabgef1/Ccr7/Cd200/Rhoh/Cd81/Msn/P2rx4/Calr	20
Pds5a/Naa50/Naa10/Atrx/Rad21/Nipbl	6
Dnajb1/Ahsa1/Dnajb11/Zc3hav1/Dnajb2/Chtop	6
Zfp36/Ddx6/Tnrc6a/Eif4e2/Tnrc6c/Tnrc6b	6
Zfp36/Ddx6/Tnrc6a/Eif4e2/Tnrc6c/Tnrc6b	6
Hspa8/Gapdh/Naca/Bcl2/Sirt1/Arrb2/Nfe2l2/Npm1/Camk2d/Pik3r1/Pten/Zc3h12a	12
Nfkbia/Syk/Laptm5/Lims1/Traf1/Ikbkb/Tnfrsf13c/Ptpn2/Stat1/Chuk	10
Klf4/Mef2c/Rhob/Dusp1/Cited2/Sp100/Bcl2/Clic4/Ptprj/Nfe2l2/Krit1/Mecp2/Stap1/Trp53inp1/Nisch/Rabgef1/Pten/Cd200/Bst2/Flcn/Gna13/Apoe/Osbpl8/Calr	24
Hsp90aa1/Ssh2/Capg/Myo1c/Rps3/Sptbn1/Tmod3/Fkbp4/Diaph1/Pstpip1/Arpc5/Csnk1d/Fchsd2/Mecp2/Arfgef1/Hax1/Bin1/Mtss1/Flii/Akap9/Dbnl/Cyfip2/Myadm/Numa1	24
Mul1/Rps19/Clec2d/Rnf125/Nmi/Itch/Arrb2/Ptpn2/C1qbp/Ythdf3/Usp15/Irak3/Riok3	13
Hspa8/Syk/Ano6/Myo18a/Ptprj/Rap1a/Stap1/Pten/Ccr7/Dock2/Dnm2/Ptprc/Calr	13
Itga4/Crebbp/Map2k1/Ankrd11/Ep300/Rab3gap1/Chd7/Kat6a/Nipbl	9
Gpx1/Egr1/Rnf4/Myc/Bcl2/Gtf2h5/Pml/Ptprc	8
Dnaja1/Hsph1/Dusp1/Hipk3/Itch/Foxo1/Per1/Zmynd11	8
Dnaja1/Hsph1/Dusp1/Hipk3/Itch/Foxo1/Per1/Zmynd11	8
Il10ra/Atp6v0a1/Herc1/Map2k1/Bcl2/Sirt1/Eif4g2/Foxo1/Usp36/Smg1/Ctsa/Rock1/Tspo/Trp53inp1/Larp1/Stk11/Trim12a/Flcn/Atf6/Zc3h12a/Bcl2l11/Rab3gap1	22
Zfp36/Limd1/Map2k1/Ddx6/Celf1/Celf2/Mecp2/Nfkb1/Ppp3ca/Tnrc6a/Cnot1/Eif4e2/Tnrc6c/Zc3h12a/Pum2/Tnrc6b	16
Jun/Dhx36/Myc/Sub1/Med13/Bclaf1/Setx	7
Nr4a1/Klf4/Zfp36/Lrrc8c/Gpx1/Atf2/Sirt1/Zfp36l2/Tbl1x/Foxo1/Scd1/Ep300/Zadh2/Eif2ak3/Lamb3/Opa3/Arl4a/Plac8/Flcn/Zc3h12a/Osbpl8/Nipbl	22
Mef2c/Plekho2/St6gal1/Sirt1	4
Dnaja1/Hsph1/Sp100/Os9/Sirt1/Inpp5k/Ogt/Nsfl1c/Derl2/Cltc/Ppp2r5a/Ppp2ca/Flcn/Gdi1	14
Kdm5a/Kdm6b/Jmjd1c/Ube2b/Kdm6a/Kdm7a	6
Kdm5a/Kdm6b/Jmjd1c/Ube2b/Kdm6a/Kdm7a	6
Hsph1/Gpx1/Hspb1/Parp1/Sirt1/Nfe2l2	6
Macf1/Dusp1/Dusp5/Kdm6b/Exoc4/Setd2/Arid1a/Rtf1/Ctnnb1/Tnrc6c/Paf1/Kdm6a	12
Kdm5a/Morf4l2/Sirt1/Tbl1x/Ep300/Fry/Per1/Akap8l/Pml/Elk4/Bcl6/Nipbl	12
Atad1/Nsf/Syk/Laptm5/Plekhj1/Itch/Arrb2/Vamp3/Lmbrd1/Pik3r4/Mylip/Cltc/Uvrag/Dnm2/Cd81/Apoe/Ptpn1/Iqsec1	18
Mul1/Sh2b3/Ptpn2/Stap1/Il6st/Ythdf3/Rabgef1/Irak3/Ptprc	9
Dusp1/Dusp5/Setd2/Ldb1/Bptf/Ankrd17/Rtf1/Ctnnb1/Tnrc6c/Paf1	10
Ubb/Rps7/Rpl37/Rps20/Pmaip1/Sirt1/Npm1/Rrs1/Pttg1ip/Rnf34	10
Nfkbia/Rnf125/Zc3hav1/Ankrd17/C1qbp/Usp15/Riok3/Pum2	8
Tpp1/Tcirg1/Vps13a/Psap/Cd81	5
Kmt2e/Snw1/Rtf1/Ctnnb1/Kmt2a	5
Arl6ip1/Zfyve27/Reep3/Rtn3/Rab3gap1	5
Tmed2/Nfe2l2/Ptpn2/Eif2ak3/Ptpn1	5
Hspd1/Mef2c/Irs2/Inpp5d/Bcl2/Siglecg/Tnfrsf13c/Btla/Cd19/Cd81/Peli1/Ptprc/Bcl6	13
Jun/Hsp90ab1/Fos/Smad7/Atf2/Zeb2/Sptbn1/Cited2/Crebbp/Egr1/Sub1/Parp1/Sirt1/Arrb2/Ep300/Got1/Nrros/Snw1/Myd88/Usp15/Stk11/Dnm2/Flcn/Pml/Zyx/Glg1/Hexa/Vim/Numa1	29
Crebbp/Dhx36/Parp1/Sirt1/Ep300/Npm1/Pcna/Trp53inp1/Pik3r1/Sde2/Stk11	11
Pde4b/Wnk1/Atp2b1/Fxyd5/Nfatc3/Ahnak/Diaph1/Bcl2/Cd84/Arrb2/Ikbkb/Fkbp1a/Camk2d/Akt2/Inpp5k/Sri/Tspo/Epb41/Vamp2/Bin1/Per1/Cd19/Dbi/Ctnnb1/Pten/Akap9/Dnm2/Ppp2ca/Pml/Rrad/Chd7/P2rx4	32
Dusp1/Dusp5/Setd2/Rtf1/Ctnnb1/Tnrc6c/Paf1	7
Mef2c/Rc3h2/Bcl2/Ikbkb/Tnfrsf13c/Bcl2l11/Dock11	7
Fkbp4/P4ha1/Fkbp1a/Nktr/Ppig/Ppwd1/Ranbp2	7
Bod1l/Pcna/Cdk9/Atrx/Fam111a/Tonsl/Nucks1	7
Nr4a1/Pde4b/Klf4/Mef2c/Foxp1/Smad7/Atp2b1/Akap13/Nfatc3/Tmod3/Nr3c1/Map2k1/Parp1/Sirt1/Foxo1/Nfatc1/Fkbp1a/Eno1/Camk2d/Ndufs6/Sri/Cdk9/Ppp3ca/Grcc10/Rock1/Rcsd1/Bin1/Tbce/Akap9/Zc3h12a/P2rx4	31
Rhob/Foxp1/Gpx1/Kdm6b/Clic4/Ikbkb/Bsg/Rap1a/Ccm2/Rock1/Myd88/Ctnnb1/Msn/Myadm	14
Irs2/Pmaip1/Nr3c1/Sirt1/Foxo1/Ep300/Akt2/Inpp5k/Ogt/Ptpn2/Usp7/Plek/Supt20/Ncoa2/Stk11/Ranbp2	16
Rhob/Ikbkb/Rock1/Epb41/Plek/Rhog/Rhoh/Tln1/Calr	9
Phf14/Pbxip1/Irf4/Sirt1/Ldb1/Ep300/Per1/Kmt2a/Kat6a	9
Smad7/Clec2d/Cr1l/Cd84/Arrb2/Cd55/Rabgef1/Bst2/Ptprc/Bcl6	10
Hsp90ab1/Hsp90aa1/Klf4/Ptges3/Myc/Arrb2/Npm1/Camk2d/Pcna/Ctnnb1	10
Klf4/Rpl24/Atf2/Pcid2/Casp2/Dgkz/Bcl2/Zfp36l2/Zfp655/Topbp1/Chfr/Sde2/Ctnnb1/Pten/Ik/Pml/Rad21/Donson/Gpr132/Foxn3	20
Gpx1/Klf7/Ccdc186/Dhps/Map2k1/Sirt1/Nadk/Foxo1/Ptprj/Pde3b/Zbtb20/Inpp5k/Sri/Ogt/Ptpn2/Ppp3ca/Pik3r1/Sidt2/Eny2/Stk11/Ppp2ca/Nucks1/Prcp	23
Mef2c/Foxp1/Naca/Nfatc3/Nr3c1/Bcl2/Sirt1/Arrb2/Daxx/Nfatc1/Ccnt2/Cdk9/Mecp2/Ankrd17/Epc1	15
Klf4/Kmt2e/Casp2/Dgkz/Crebbp/Bcl2/Zfp655/Anp32b/Ankrd17/Ccnd3/Sde2/Pten/Paf1/Pml/Ccnd2	15
Macf1/Actg1/Lims1/Ldb1/Ptprj/Iqgap1/Rock1/Rcc2/Pten	9
Macf1/Actg1/Lims1/Ldb1/Ptprj/Iqgap1/Rock1/Rcc2/Pten	9
Foxp1/Sirt1/Lbh/Cnot1/Per1/Cnot2/Calr	7
Hspa1a/Hspa1b/Senp6/Rnf4/Rae1/Cltc/Numa1	7
Cdc25b/Topbp1/Npm1/Camk2d/Mecp2/Rcc2/Chfr/Rad21/Donson/Lmnb1/Gpr132/Foxn3	12
Hsp90ab1/Hsp90aa1/Ppp1r15a/Smad7/Bcl2/Bcar3/Arrb2/Akt2/Inpp5k/Ogt/Hax1/Fnip1/Pten/Akap9/Phip/Nsd1	16
Map2k1/Myc/Parp1/Ep300/Fis1/Zbtb20/Eno1/Ogt/Tspo/Cox7a2/Flcn	11
Pde4b/Hspd1/Arid5a/Laptm5/Tcirg1/Cd2/Tnfrsf13c/H2-M3/Gadd45g/Cebpg/Ifnar1/C1qbp/Ccr7/Zc3h12a	14
Gpx1/Klf7/Ccdc186/Dhps/Map2k1/Sirt1/Nadk/Foxo1/Ptprj/Pde3b/Zbtb20/Inpp5k/Sri/Ogt/Ptpn2/Ppp3ca/Pik3r1/Sidt2/Eny2/Stk11/Ppp2ca/Nucks1/Prcp	23
Pde4b/Mef2c/Tsc22d3/Rc3h2/Pmaip1/Mir142hg/Tcirg1/Bcl2/Ikbkb/Sh2b3/Tnfrsf13c/Bcl2l11/Dock11	13
Itch/Cblb/Cd86/Ccr7/Irak3	5
Rhob/Foxp1/Bsg/Ccm2/Ctnnb1	5
Arrb2/Nfkb1/C1qbp/Ccr7/Irak3	5
Limd1/Ddx6/Cnot1/Pan3/Cnot2	5
Smad7/Rc3h2/Il4ra/Zc3h12a/Bcl6	5
Sirt1/Foxo1/Ptpn2/Supt20/Stk11	5
Rhob/Foxp1/Bsg/Ccm2/Ctnnb1	5
B3gnt7/Chst12/Nfkb1/Xylt1/B3gnt2/Ccnd3/Cltc/Hexa	8
Smad7/Rc3h2/Hspb1/Nfkbid/Ptpn2/Il4ra/Zc3h12a/Bcl6	8
Hsp90ab1/Rps7/Csnk2a1/Ddrgk1/Smarcc1/Ogt/Usp7/Pml	8
Wnk1/Klf4/Macf1/Rps12/Rnf213/Zeb2/G3bp1/Cmah/Pbxip1/Limd1/Chd8/Egr1/Myc/Csnk1d/Ldb1/Foxo1/Zranb1/Csnk1g3/Siah2/Csnk2a1/Ube2b/Nfkb1/Rtf1/Tnks2/Ctnnb1/Pten/Stk11/Csnk1a1/Paf1/Kdm6a/Apoe/Ccdc88c	32
Hspa1a/Hspa1b/Tubb5/Rps3/Senp6/Rnf4/Csnk1d/Nudc/Rae1/Map4/Kpnb1/Mecp2/Atrx/Tbce/Cltc/Uvrag/Numa1	17
Cited2/Egr1/Hif1an/Nfe2l2	4
Kdm5a/Morf4l2/Sirt1/Tbl1x/Per1/Akap8l/Pml/Elk4/Bcl6/Nipbl	10
Jun/Fos/Wnk1/Mef2c/Jund/Junb/Nfatc3/Clic4/Iqgap1/Fus	10
Gpx1/Mul1/Plaur/Pmaip1/Arrb2/Chchd10/Ep300/Rala/Fis1/Ghitm/Tspo/Higd1a/Flcn/Bcl2l11	14
Sp100/Xpo1/Anp32b/Xpo6/Sfn/Hspa9/Rbm22/Calr/Cse1l	9
Pde4b/Atp2b1/Nfatc3/Ahnak/Diaph1/Bcl2/Cd84/Arrb2/Fkbp1a/Camk2d/Inpp5k/Sri/Tspo/Epb41/Bin1/Cd19/Dbi/Ctnnb1/Ppp2ca/Pml/Rrad/Chd7/P2rx4	23
Hsp90aa1/Ssh2/Capg/Myo1c/Rps3/Sptbn1/Tmod3/Fkbp4/Arpc5/Fchsd2/Mecp2/Arfgef1/Hax1/Bin1/Flii/Akap9/Dbnl/Cyfip2/Myadm/Numa1	20
Rpl35/Rpl7a/Nifk/Npm1/Nhp2/Pes1	6
Cdc25b/Npm1/Camk2d/Mecp2/Rcc2/Lmnb1	6
Birc3/Nmi/Ube2d1/Birc2/Gabarap/Trip12	6
Jun/Klf4/Mef2c/Foxp1/Mef2d/S1pr1/Naa35/Egr1/Myc/Camk2d/Pik3r1/Myd88/Ctnnb1/Pten/Rps6kb1/Gna13/Apoe	17
Wnk1/Klf4/Macf1/Rps12/Rnf213/Zeb2/G3bp1/Cmah/Pbxip1/Limd1/Chd8/Egr1/Myc/Csnk1d/Ldb1/Foxo1/Zranb1/Csnk1g3/Siah2/Csnk2a1/Ube2b/Nfkb1/Rtf1/Tnks2/Ctnnb1/Pten/Stk11/Csnk1a1/Paf1/Kdm6a/Apoe/Ccdc88c	32
Irf4/Bcl2/Ptpn2/Ly9/Dock2/Stk11/Ptprc	7
Ptprj/Prkcb/Lmbrd1/Inpp5k/Ptpn2/Rps6kb1/Socs3	7
P2ry10/Atp2b1/Ms4a1/S1pr1/Tcirg1/Anxa7/Ccdc47/Diaph1/Mcur1/Bcl2/Prkcb/Fis1/Got1/Fkbp1a/Camk2d/Sri/Cd55/Herpud1/Atp2c1/Il6st/Cd19/Cxcr5/Dbi/Ccr7/Cul5/Gpr65/Tmco1/Pml/Gna13/Ptprc/Apoe/Chd7/P2rx4/Che	34
Mef2c/Fbxl20/Bcl2/Mecp2/Ppp3ca/Tspo/Git2/Dbi/Pten/Rps6kb1/Apoe/P2rx4	12
Klf4/Mef2c/Rhob/Dusp1/Cited2/Sp100/St6gal1/Bcl2/Clic4/Ptprj/Nfe2l2/Krit1/Ptpn2/Mecp2/Stap1/Trp53inp1/Nisch/Rabgef1/Pten/Cd200/Bst2/Flcn/Gna13/Apoe/Osbpl8/Calr	26
Smad7/Rc3h2/Malt1/Nfkbid/Il4ra/Ccr7/Zc3h12a/Bcl6	8
Klf4/Mef2c/Rhob/Dusp1/Cited2/Sp100/Bcl2/Clic4/Ptprj/Nfe2l2/Krit1/Mecp2/Stap1/Trp53inp1/Nisch/Rabgef1/Pten/Cd200/Bst2/Flcn/Gna13/Apoe/Osbpl8/Calr	24
Hsp90ab1/Rps7/Csnk2a1/Ddrgk1/Smarcc1/Ogt/Usp7/Pml/Usp19	9
Hspd1/Foxp1/Smad7/Rc3h2/Malt1/Irf4/Nfkbid/H2-M3/Gadd45g/Ly9/Cd19/Il4ra/Ccr7/Cd81/Relb/Zc3h12a/Ptprc/Bcl6/Dock11	19
Gpx1/Klf7/Irs2/Ccdc186/Egr1/Sirt1/Nadk/Pde3b/Eif2b2/Zbtb20/Sri/Ogt/Ppp3ca/Vamp2/Sidt2/Eny2/Ppp2ca	17
Hsp90aa1/Mul1/Atf2/Pmaip1/Myc/Chchd3/Bcl2/Dynlt1c/Chchd10/Ghitm/Bcl2l11/Hspa4	12
Dnaja1/Dusp1/Hipk3/Sirt1/Ptprj/Sh2b3/Inpp5k/Stk38/Pten/Irak3/Apoe/Ptpn1	12
Hsp90aa1/Hsph1/Hspd1/Mef2c/Syk/Irs2/H2-Ab1/Rps3/Pcid2/Inpp5d/Sart1/Sh3kbp1/Malt1/Dhps/Rasal3/Bcl2/Nfkbid/H2-Eb2/Tnfrsf13c/Cd55/Cd86/Dock8/Myd88/Il6st/Il4ra/Ccr7/Rhoh/Cd81/Peli1/Ptprc/Bcl6	31
Hspa8/Jun/Stk17b/Wnk1/Syk/Clk1/Csnk1g3/Smg1/Csnk2a1/Iqgap1/Sik2/Camk2d/Eif2ak3/Hspa9/Stk11/Ppp2ca/Irak3/Vrk1/Ptprc/Clk4	20
Klf2/Rc3h2/Bcl2/Tmed2/Clic4/Daxx/Celf1/Ankrd11/Ep300/Ndufs6/Ccm2/Kmt2c/Atrx/Lncpint/Tnks2/Plac8/Kdm6a/Chd7/Nipbl	19
Hsp90ab1/Ppp1r15a/Ppp1r15b/Ppp2ca/Ptprc	5
Cabin1/Atrx/Ubn1/Nasp/Supt16	5
Jun/Fos/Nfkbia/Nfkb1/Chuk	5
Dhx36/Myc/Parp1/Smg5/Smg1/Atrx/Tnks2/Ctnnb1/Pml/Gnl3	10
Btbd9/Chd8/Crebbp/Egr1/Mecp2/Apoe/Ccnd2	7
Sp100/Os9/Ankrd13c/Chchd10/Tspo/Rer1/Pml	7
Smad7/Rc3h2/Cr1l/Cd55/Il4ra/Zc3h12a/Ptprc/Bcl6	8
Macf1/Atp5g1/Zfyve27/Golga4/Eif2b2/Dbnl/Apoe/Gdi1	8
Kdm5a/Sirt1/Ep300/Fry/Akap8l/Pml/Bcl6/Nipbl	8
Laptm5/Dgkz/Elf1/Ptprj/Cblb/Ptpn2	6
Vps37b/Trim25/Vapa/Trim12a/Pml/Trim11	6
Klf7/Sp100/Os9/Snx12/Pde3b/Inpp5k/Ogt/Ppp3ca/Derl2/Eny2/Cd200/Ppp2ca/Apoe/Gdi1	14
Kmt2e/Arid4b/Satb1/Crebbp/Snrpd3/Setd2/Myc/Cmtr1/Parp1/Sirt1/Mtf2/Kmt2c/Ogt/Snw1/Mecp2/Ehmt1/Iws1/Atrx/Pax5/Rtf1/Zc3h13/Ctnnb1/Paf1/Kmt2a/Nsd1/Chtop/Prmt1	27
Rhob/Foxp1/Hspb1/Sirt1/Ets1/Nfe2l2/Bsg/Sp1/Zc3h12a/P2rx4/Calr/Atp5a1	12
Itga4/Lims1/St6gal1/Vamp3/Rcc2/C1qbp/Lamb3/Dnm2/Myadm/Nedd9/Calr/Parvg	12
Macf1/Nsf/Sptbn1/Vamp3/Golga4/Vamp2/Atp2c1/Golga7/Sys1	9
Rab43/Tcirg1/Atg12/Rb1cc1/Pik3c3/Gabarap/Nsfl1c/Trp53inp1/Wipi2/Map1lc3a/Rab3gap1	11
Rps3/Tpt1/Pmaip1/Smchd1/Setd2/Myc/Parp1/Sirt1/Smg1/Npm1/Pcna/Cdk9/Cebpg/Nsmce4a/Pttg1ip/Fus/Bclaf1/Trip12/Pml	19
Jun/Fos/Wnk1/Mef2c/Jund/Junb/Nfatc3/Slc30a5/Tcirg1/Anxa7/Bcl2/Clic4/Daxx/Nfe2l2/Iqgap1/Mecp2/Ppp3ca/Fus/Ppp2ca/Chuk/Glud1/P2rx4/Gdi1	23
Foxp1/Arid5a/Laptm5/Malt1/Dhx36/Sirt1/Dennd1b/Dnajb9/Cd55/Cd86/Il4ra/Cd81/Ptprc	13
Kmt2e/Tmod3/Crebbp/Cdc25b/Camk2d/Mecp2/Anp32b/Rcc2/Ankrd17/Ccnd3/Rps6kb1/Lmnb1/Ccnd2	13
Rps2/Rpl13a/Eif2s2/Eif2s3x	4
Klf2/Snw1/Cnot1/Calr	4
Ptprj/Rap1a/Rabgef1/Ptprc	4
Sart3/Itch/Dnajb2/Zc3h12a	4
Erlin1/Nfkb1/Plek/Apoe	4
Hnrnpl/Eif3e/Cdk9/Nucks1	4
Gpx1/Klf7/Irs2/Ccdc186/Egr1/Sirt1/Nadk/Pde3b/Eif2b2/Zbtb20/Sri/Ogt/Ppp3ca/Vamp2/Sidt2/Eny2/Ppp2ca	17
Pds5a/Naa50/Naa10/Atrx/Ctnnb1/Rad21/Esco1/Nipbl	8

Hsp90aa1/Rel/Crebbp/Nmi/Zbtb20/Zc3hav1/Ifnar1/Relb/Riok3	9
Hsp90aa1/Rel/Crebbp/Nmi/Zbtb20/Zc3hav1/Ifnar1/Relb/Riok3	9
Klf4/Junb/Rpl13/Sf3b1/Matr3/Cited2/N4bp2l2/Wdr74/Med21/Cnot1/Cnot2/Cdk11b/Rtf1/Sp1/Nasp	15
Rhob/Cdc42se2/Plekho1/Anxa7/Sh3kbp1/Limd1/Diaph1/Epb41/Arhgap15/Rhog/Rhoh/Phip/Brwd1/Gna13/Msn	15
Gpx1/Klf7/Ccdc186/Sirt1/Nadk/Pde3b/Prkcb/Zbtb20/Sri/Ogt/Nfkb1/Ppp3ca/Sidt2/Eny2/Ppp2ca	15
Macf1/Actg1/Lims1/Bcl2/Ldb1/Ptprj/Iqgap1/Rock1/Rcc2/Pten/Tln1	11
Gpx1/Klf7/Irs2/Ccdc186/Egr1/Sirt1/Nadk/Pde3b/Eif2b2/Zbtb20/Sri/Ogt/Ppp3ca/Vamp2/Sidt2/Eny2/Ppp2ca	17
Mef2c/Tsc22d3/Rc3h2/Pmaip1/Tcirg1/Bcl2/Ikbkb/Tnfrsf13c/Bcl2l11/Dock11	10
Ubb/Shisa5/Rps7/Pmaip1/Sirt1/Ep300/Snw1/Pttg1ip/Stk11/Pml	10
Kdm5a/Kdm6b/Jmjd1c/Ube2b/Kdm6a/Kdm7a	6
Kdm5a/Kdm6b/Jmjd1c/Ube2b/Kdm6a/Kdm7a	6
Atf2/Rere/Herc1/Map2k1/Bcl2/Ldb1/Sec24b/Whrn/Arcn1/Mecp2/Ogdh/Psap/Slc4a7	13
Gapdh/Trim25/Akap13/Dhx36/Prkcb/Ikbkb/Zc3hav1/Ankrd17/Myd88/Ctnnb1/Trim12a/Peli1/Chuk	13
Nr4a1/Pde4b/Wnk1/Syk/Rpl13a/Rps19/Dusp1/S1pr1/Gpsm3/Ano6/Hspb1/Adam10/Arrb2/Ptprj/Bsg/Stap1/Rhog/C1qbp/Cxcr5/Ccr7/Cnr2/P2rx4/Calr/Il17ra	24
Smad7/Arid5a/Laptm5/Malt1/Dhx36/Sirt1/Dennd1b/Cd55/Bst2/Irak3/Cd81/Bcl6	12
Crebbp/Dhx36/Parp1/Sirt1/Ep300/Npm1/Akt2/Pcna/Trp53inp1/Pik3r1/Sde2/Stk11	12
Atp6v0a1/Tcirg1/Clic4/Snapin/Dmxl1	5
Orc3/Bod1l/Pold4/Polg/Topbp1/Npm1/Pcna/Cdk9/Atrx/Fam111a/Tonsl/Nucks1/Donson/Srpk2	14
Hsp90ab1/Hsp90aa1/Macf1/Ifrd1/Rnf6/Atp5g1/Zfyve27/Dip2b/Golga4/Eif2b2/Dnm2/Dbnl/Apoe/Gdi1	14
Laptm5/Plekhj1/Map2k1/Snx12/Arl8b/Pik3c3/Vamp3/Usp7/Mecp2/Cltc/Srsf10/Vti1b/Spag9/Sys1/Msn	15
Ssh2/Capg/Myo1c/Sptbn1/Tmod3/Diaph1/Pstpip1/Arpc5/Fchsd2/Arfgef1/Hax1/Bin1/Mtss1/Flii/Dbnl/Cyfip2/Myadm	17
Hspa8/Hspd1/Smad7/Arid5a/Clec2d/Malt1/Cr1l/Arrb2/H2-Q7/Fbxo38/Dennd1b/H2-M3/Cd55/H2-Q6/H2-Q4/Cd81/Ptprc/H2-T23/Bcl6	19
Atp5e/Map2k1/Atp5c1/Myc/Parp1/Atp5g1/Eno1/Flcn/Atp5a1	9
Sp100/Os9/Ankrd13c/Chchd10/Tspo/Rer1/Vps13a/Tnrc6a/Pml	9
Mef2c/Fbxl20/Bcl2/Ep300/Mecp2/Dbi/Rps6kb1/Apoe	8
Mul1/Sp100/Setd2/Ptpn2/Ifnar1/Stat1/Myd88/Ythdf3	8
Mef2c/Naca/Tsc22d3/Bcl2/Ctnnb1/Rps6kb1/Flcn/Usp19	8
Cdc25b/Topbp1/Camk2d/Mecp2/Rcc2/Chfr/Rad21/Donson/Lmnb1/Gpr132/Foxn3	11
Hspa8/Hsp90ab1/Gapdh/Rpl30/Syk/Rps19/Clec2d/Cr1l/Arl8b/Arrb2/H2-Q7/H2-M3/Cd55/Stap1/Cebpg/H2-Q6/Myd88/H2-Q4/Bcl2l11/Ptprc/H2-T23	21
Hspa1a/Hspa1b/Rpl24/Pcid2/Pds5a/Naa50/Naa10/Nudc/Rrs1/Nsfl1c/Kpnb1/Reep3/Atrx/Cltc/Cdk11b/Ik/Akap8l/Phip/Eml4/Rad21/Nipbl/Numa1	22
Dnaja1/Rps7/Smad7/Rps3/Arrb2/Ogt/Ivns1abp/Tspo/Usp4/Trip12	10
Mef2c/Foxp1/Mef2d/Rpl13/Akap13/Atf2/Prpsap2/Ano6/Cited2/Ankrd11/Ep300/Sh2b3/Xylt1/Rabgap1l/Pex7/Fli1/Opa3/Glg1/Ptprc/Foxn3	20
Dnaja1/Traf4/Gadd45b/Hipk3/Itch/Rb1cc1/Traf1/Gadd45g/Map4k2/Zfp622/Per1/Zmynd11/Myd88/Lmnb1	14
Mef2c/Ep300/Sh2b3/Rabgap1l/Fli1/Sp1/Prmt1	7
Macf1/Herpud1/Zfand2a/Srpr/Tram1/Sec63/Rab3gap1	7
Macf1/Actg1/Lims1/St6gal1/Bcl2/Ldb1/Ptprj/Plekha2/Iqgap1/Rock1/Rcc2/C1qbp/Pik3r1/Pten/Dnm2/Myadm/Bcl6/Nedd9/Calr	19
Pde4b/Wnk1/Syk/Rpl13a/Rps19/Dusp1/S1pr1/Gpsm3/Ano6/Adam10/Ptprj/Bsg/Stap1/C1qbp/Cxcr5/Ccr7/Cnr2/Calr/Il17ra	19
Cfl2/Actg1/Ssh2/Capg/Myo1c/Sptbn1/S1pr1/Tmod3/Arpc5/Fchsd2/Inpp5k/Arfgef1/Plek/Hax1/Bin1/Pik3r1/Mtss1/Flii/Gpr65/Dbnl/Cyfip2/Myadm	22
Macf1/Actg1/Lims1/Bcl2/Ldb1/Ptprj/Plekha2/Iqgap1/Rock1/Rcc2/Pik3r1/Pten/Bcl6	13
Macf1/Ifrd1/Rnf6/Atp5g1/Zfyve27/Dip2b/Golga4/Eif2b2/Dnm2/Dbnl/Apoe/Gdi1	12
Atp5e/Map2k1/Atp5c1/Myc/Parp1/Atp5g1/Eno1/Flcn/Atp5a1	9
Hsp90ab1/Myo1c/Pmaip1/Fis1/Akt2/Anp32b/Pcm1/Vamp2/Tmem30a/Pik3r1/Sfn/Rbm22/Cd81/Zc3h12a	14
Map2k1/Ccnt2/Ccnt1/Ctnnb1/Scaf8/Supt16	6
Lims1/Ptprj/Iqgap1/Rock1/Pik3r1/Iqsec1	6
Prdm2/Inpp5d/Map2k1/Bcl2/Polg/Sirt1/Jmjd1c/Kras/Npm1/Ndufs6/Pax5/Lncpint/Baz2b/Pten/Pml/Gna13/Sec63/Apoe/Bcl6/Kat6a/Calr	21
Hspa1a/Hspa1b/Hsph1/Macf1/Azin1/Rps3/Senp6/Fkbp4/Rnf4/Diaph1/Sirt1/Rae1/Xpo1/Npm1/Mecp2/Rock1/Cltc/Akap9/Stk11/Eml4/Numa1	21
Macf1/Nap1l1/Zeb2/Dhx36/Bcl11a/Map2k1/Myc/Ist1/Cux1/Atp5g1/Zfyve27/Golga4/Eif2b2/Snw1/Tspo/Bin1/Il6st/Ctnnb1/Stk11/Dbnl/Apoe/Vim/Gdi1	23
Tcirg1/Inpp5k/Stap1/Rcc2/Plek/Rhog/Plekhm1/Dbnl	8
Klf2/Hsp90aa1/Klf4/Dynll1/H2-M3/Akt2/Tspo/Dnm2/Zc3h12a/P2rx4	10
Pgap1/Zdhhc3/Atg12/Zdhhc18/Pik3c3/Ugcg/Nmt2/Dbi/Wipi2/Golga7/Dpm1/Apoe/Rab3gap1	13
Wnk1/Macf1/Rps12/Rnf213/Zeb2/G3bp1/Cmah/Pbxip1/Limd1/Chd8/Egr1/Csnk1d/Foxo1/Zranb1/Csnk1g3/Siah2/Csnk2a1/Ube2b/Nfkb1/Tnks2/Stk11/Csnk1a1/Apoe/Ccdc88c	24
Hsp90ab1/Irs2/Myo1c/Pmaip1/Myo18a/Sirt1/Nadk/Fis1/Bsg/Akt2/Sri/Anp32b/Pcm1/Vamp2/Cd2ap/Atp2c1/Tmem30a/Pik3r1/Sfn/Rbm22/Cd81/Zc3h12a/Glud1/Crebrf	24
Rps8/Uba52/Rps5/Rpl18/Rplp2/Rpsa/Rpl19/Rpl9-ps6/Rps24/Rps26/Rps4x/Fau/Rpl8/Rpl38/Rps21/Rps7/Rpl37/Rps27/Rps20/Rpl30/Rps16/Rps2/Rpl13a/Rpl18a/Rpl13/Rps19/Rps13/Rpl35/Rpl21/Rps12/Rps27a/Rpl28/Rpl2	46
Rps8/Uba52/Rps5/Rpl18/Rplp2/Rpsa/Rpl19/Rpl9-ps6/Rps24/Rps26/Rps4x/Fau/Rpl8/Rpl38/Rps21/Rps7/Rpl37/Rps27/Rps20/Rpl30/Rps16/Rps2/Rpl13a/Rpl18a/Rpl13/Mrps6/Rps19/Rps13/Rpl35/Rpl21/Rps12/Rps27a/Rpl	57
Rps8/Uba52/Rps5/Rpl18/Rplp2/Rpsa/Rpl19/Rpl9-ps6/Rps24/Rps26/Rps4x/Fau/Rpl8/Rpl38/Rps21/Rps7/Rpl37/Rps27/Rps20/Rpl30/Rps16/Rps2/Rpl13a/Rpl18a/Rpl13/Mrps6/Rps19/Rps13/Rpl35/Rpl21/Rps12/Rps27a/Rpl	52
Rps8/Uba52/Rps5/Rpsa/Rps24/Rps26/Rps4x/Fau/Rps21/Rps7/Rps27/Rps20/Rps16/Rps2/Rps19/Rps13/Rps12/Rps27a/Rps3/Rps10/Rps17/Rps28/Larp4	23
Rps8/Uba52/Rps5/Rpsa/Rps24/Rps26/Rps4x/Fau/Rps21/Rps7/Rps27/Rps20/Rps16/Rps2/Mrps6/Rps19/Rps13/Rps12/Rps27a/Rps3/Rps10/Rps17/Rps28/Mrps12/Npm1/Mrps7/Mrps23/Larp4	28
Uba52/Rpl18/Rplp2/Rpl19/Rpl9-ps6/Rpl8/Rpl38/Rpl37/Rpl30/Rpl13a/Rpl18a/Rpl13/Rpl35/Rpl21/Rps12/Rpl28/Rpl24/Rpl14/Rpl31/Rpl35a/Rpl15/Rpl36a/Rpl7a/Rpl39	24
Rpl18/Rpl19/Rps26/Rps4x/Rpl8/Rpl38/Rps21/Rpl30/Rpl18a/Rpl24/Rpl31/Rps3/Rps28/Rpl36a/Rpl7a/Naa35/Rpl39/Naa38/Fus/Larp1/Larp4/Larp4b/Vim/Calr	24
Hspa1a/Hspa1b/Mef2c/Malat1/Rbm39/Pnisr/Son/Atp6v0a1/Pnn/Sf3b1/Srsf7/Sap18/Pcbp1/Sart1/Dgkz/Dhx36/Srrm2/Nr3c1/Acin1/Usp36/Npm1/Snrnp70/Dennd1b/Tcf12/Snw1/Grcc10/Luc7l2/Poldip3/Srrm1/Sde2/Zc3h13/	50
Rps4x/Tuba1a/Mbnl1/Trim25/Tubb5/Zfp36/Rpl28/Rc3h2/Pcbp1/G3bp1/Limd1/Dhx36/Lsm2/Pabpc4/Hnrnpl/Ddx6/Celf1/Iqgap1/Dhx30/Cdk9/Kpnb1/Rock1/Tnrc6a/Cnot1/Poldip3/Pan3/Cnot2/Blnk/Ythdf3/Eif4e2/Larp1/Tnrc6	40
Rpl18/Rpl19/Rps26/Rpl8/Rpl38/Rps21/Rpl30/Rpl18a/Rpl24/Rpl31/Rps28/Rpl36a/Rpl7a/Rpl39/Larp1/Larp4b	16
Rps4x/Tuba1a/Mbnl1/Trim25/Tubb5/Zfp36/Rpl28/Rc3h2/Pcbp1/G3bp1/Limd1/Dhx36/Lsm2/Pabpc4/Ddx6/Celf1/Iqgap1/Cdk9/Kpnb1/Rock1/Tnrc6a/Cnot1/Poldip3/Pan3/Cnot2/Blnk/Ythdf3/Eif4e2/Larp1/Tnrc6c/Trim12a/Atxn	38
Uba52/Rpl18/Rplp2/Rpl19/Rpl9-ps6/Rpl8/Rpl38/Rpl37/Rpl30/Rpl13a/Rpl18a/Rpl13/Rpl35/Rpl21/Rps12/Rpl28/Rpl24/Rpl14/Rpl31/Rpl35a/Rpl15/Rpl36a/Rpl7a/Rpl39/Mrpl52/Npm1/Mrpl15	27
Nr4a1/Gapdh/Cacybp/Shisa5/Tubb5/Myo1c/Pcid2/Rnf6/Anxa7/Vapa/Bcl2/Nup50/Parp1/Sirt1/Brap/Phf20/Rb1cc1/Rae1/Xpo1/Ipo7/Unc50/Nup88/Nup210/Nrm/Kpnb1/Mrps23/Pcm1/Casc3/Dnajb2/Bin1/Ankrd17/Epc1/Eny2	49
Phf14/Atf2/Naa35/Morf4l2/Gm16286/Naa50/Crebbp/Naa10/Phf20/Ep300/Naa38/Ogt/Naa15/Supt20/Brd8/Epc1/Eny2/Ep400/Phf20l1/Kat6a	20
Phf14/Atf2/Naa35/Morf4l2/Gm16286/Naa50/Crebbp/Naa10/Phf20/Ep300/Naa38/Ogt/Naa15/Supt20/Brd8/Epc1/Eny2/Ep400/Phf20l1/Kat6a	20
Hspa8/Ubb/Hsp90aa1/Gapdh/Uba52/Hspd1/Tuba1a/Actg1/Nsf/Rps27a/Prdx3/Exoc4/Tuba1b/Atp5c1/Ubc/Bcl2/Gdi2/Rala/Tufm/Rap1a/Eno1/Cltc/Hspa9/Pten/Aco2/Msn/Atp6v1b2/Slc25a3/Gdi1/Atp5a1	30
Jun/Nr4a1/Fos/Klf4/Jund/Smad7/Junb/Arid5a/Hnrnpab/Crem/Atf2/Nfatc3/Sap18/Tcea1/Pbxip1/Limd1/Crebbp/Hes6/N4bp2l2/Sub1/Arnt/Parp1/Ets1/Tbl1x/Ldb1/Mxd4/Nfe2l2/Tcf4/Nfatc1/Ep300/Csnk2a1/Gtf2a2/Abt1/Tcf12/	48
Mbnl1/Trim25/Zfp36/Rc3h2/G3bp1/Dhx36/Pabpc4/Ddx6/Celf1/Kpnb1/Rock1/Ythdf3/Larp1/Atxn2l/Pum2/Larp4/Larp4b	17
Hspa8/Hsp90ab1/Laptm5/Atp6v0a1/Tcirg1/Lamtor2/Arl8b/H2-Q7/Rb1cc1/Man2b1/H2-Eb2/Gabarap/H2-M3/Lmbrd1/Cyb561a3/Atg16l2/Vps13a/Fnip1/H2-Q6/Sidt2/Uvrag/Lamtor3/Map1lc3a/Vti1b/H2-Q4/Spag9/Flcn/Atp6v	30
Satb1/Sf3b1/Matr3/Sp100/Clic4/Npm1/Pcna/Snw1/Nrm/Zfp326/Srrm1/Ppig/Scaf8/Akap8l/Lbr/Pml/Rad21/Ebna1bp2/Lmnb1/Numa1	20
Nr4a1/Gapdh/Rnf6/Vapa/Bcl2/Nup50/Sirt1/Brap/Phf20/Rb1cc1/Xpo1/Unc50/Nrm/Kpnb1/Mrps23/Pcm1/Casc3/Dnajb2/Ankrd17/Epc1/Tnrc18/Ctnnb1/Ranbp2/Lbr/Pml/Pum2/P2rx4/Osbpl8/Lmnb1/Ccnd2	30
Satb1/Rnf6/Crebbp/Sp100/Rnf4/Hipk3/Sirt1/Daxx/Tdp2/Eif3e/Topbp1/Cdk9/Atrx/Chfr/Pml/Kat6a	16
Fth1/Ftl1/Actg1/Syk/Capg/Myo1c/Rab43/Tcirg1/Sh3kbp1/Myo18a/Arrb2/H2-Q7/Pik3c3/Rala/Vamp3/Rap1a/Lmbrd1/Cd2ap/Cltc/Uvrag/Dnm2/Zyx/Rapgef6/H2-T23/Pld4/Vim/Calr	27
Mul1/Atp2b1/H13/Atp6v0a1/Ccdc47/Aup1/Mcur1/Chchd3/Arl6ip1/Chchd10/H2-Q7/Scd1/Cux1/Zfyve27/Fis1/Unc50/Ktn1/Coq2/Ghitm/Tvp23b/Sfxn3/Rer1/Ergic3/Dnajb2/Derl2/Preb/Atp2c1/Tram1/Golga7/Tmco1/Sys1/Atf6/	37
Prpsap2/Tcea1/Pold4/Polg/Rb1cc1/Pik3c3/Ikbkb/Ccnt2/Polr2a/Gtf2a2/Prkag1/Pcna/Snw1/Cnppd1/Pik3r4/Cdk9/Polr1d/Ccnt1/Pik3r1/Ccnd3/Uvrag/Prps1/Gtf2h5/Rtf1/Paf1/Chuk/Socs3/Ccnd2	28
Mul1/Atp2b1/H13/Atp6v0a1/Ccdc47/Aup1/Mcur1/Chchd3/Arl6ip1/Chchd10/H2-Q7/Scd1/Cux1/Zfyve27/Fis1/Unc50/Ktn1/Coq2/Ghitm/Tvp23b/Sfxn3/Rer1/Ergic3/Derl2/Preb/Atp2c1/Tram1/Tmco1/Sys1/Atf6/Slc25a3/H2-T2	35
Hspa8/Fth1/Ftl1/Atg12/Aup1/Rb1cc1/Pik3c3/Gabarap/Atg16l2/Plekhm1/Trp53inp1/Uvrag/Wipi2/Map1lc3a	14
Naa35/Gm16286/Naa50/Naa10/Naa38/Naa15	6
Satb1/Sf3b1/Matr3/Clic4/Npm1/Snw1/Zfp326/Srrm1/Ppig/Scaf8/Akap8l/Pml/Rad21/Lmnb1/Numa1	15
Actg1/Syk/Capg/Myo1c/Rab43/Tcirg1/Myo18a/H2-Q7/Pik3c3/Vamp3/Rap1a/Uvrag/Zyx/H2-T23/Pld4/Vim/Calr	17
Hspa8/Pnn/Sf3b1/Sart1/Srrm2/Snrpd3/Isy1/Lsm2/Rbm17/Rbm5/Snrnp70/Snw1/Prpf38b/Casc3/Magoh/Luc7l2/Srrm1/Lsm8/Ik/Rbm22/Ppwd1/Hnrnph1/Mfap1a/Prpf8	24
Phf14/Atf2/Morf4l2/Crebbp/Phf20/Ep300/Ogt/Supt20/Brd8/Epc1/Eny2/Ep400/Phf20l1/Kat6a	14
Cyth1/Sla/Syk/Atp2b1/H13/Rasal3/Gng5/Cd2/Traf1/Ikbkb/Kras/Birc2/Iqgap1/Ppp3ca/Gng2/Mtss1/Pten/Cnr2/Gna13/Chuk/Msn/Ptprc/Ptpn1	23
Klf6/Smad7/Sp140/Traf4/Aff4/Malt1/Znrf2/Ubtf/Smarca5/Sirt1/Rae1/Herc4/Wdr43/Pspc1/Cd2ap/Fam111a/Mri1/Pax5/Nop56	19
Zfp36/Limd1/Lsm2/Ddx6/Tnrc6a/Cnot1/Pan3/Cnot2/Ythdf3/Eif4e2/Tnrc6c/Trim12a/Zc3h12a/Tnrc6b	14
Sf3b1/Sart1/Srrm2/Snrpd3/Isy1/Lsm2/Snrnp70/Snw1/Casc3/Luc7l2/Lsm8/Ik/Rbm22/Mfap1a/Prpf8	15
Aff4/Ccnt2/Snw1/Cdk9/Aff1/Ccnt1/Tonsl/Rtf1/Paf1/Ccnl1/Supt16	11
Arid1b/Smarca5/Rsf1/Smarce1/Brd9/Csnk2a1/Smarcc1/Bptf/Arid1a/Brd8/Ep400/Smarca2/Chd4	13
Arid1b/Smarca5/Rsf1/Smarce1/Brd9/Csnk2a1/Smarcc1/Bptf/Arid1a/Brd8/Ep400/Smarca2/Chd4	13
Hspd1/Rps3/Timm8b/Atp5e/Pmpcb/Ndufa3/Ndufc1/Atp5c1/Mcur1/Chchd3/Bcl2/Ndufv1/Polg/Cox7b/Sirt1/Ak2/Chchd10/Atp5g1/Tufm/Unc50/Ndufs6/Coq2/Nrm/Ghitm/Csde1/Sfxn3/Slc25a51/Ndufs8/Cox7a2/Hadha/Higd1	41
Tex10/Pelp1/Kdm6b/Chd8/Snrpd3/Sirt1/Phf20/Mtf2/Kmt2c/Riok1/Zc3h13/Kdm6a/Kmt2a/Nsd1/Prmt1	15
Hsp90aa1/Macf1/Myo1c/Rps3/Plekho1/Tmod3/Diaph1/Ptprj/Iqgap1/Akt2/Inpp5k/Rock1/Plek/Cd2ap/Dnm2/Dbnl/Atp6v1b2/Tln1/Myadm	19
Hspa8/Pde4b/Macf1/Rpl8/Nsf/Rpl38/Rps27/Rpl30/Rpl18a/Rps19/Rps13/Rpl14/Rps3/Sptbn1/Ifngr1/Hspb1/Adam10/Eif3a/Bcl11a/Nr3c1/Exoc4/Eif4g2/Arrb2/Map4/Ncoa2/Myd88/Ctnnb1/Ppp2ca/Dbnl/P2rx4/Iqsec1	31
Hspa1a/Hspa1b/Hsp90ab1/Gpx1/Arih1/Tdp2/Cabin1/Herpud1/Dnajb2/Psap/Ranbp2/Pabpn1/Nxf1	13
Hspd1/Rhob/H2-Ab1/Plekhj1/Prdx3/Map2k1/Snx12/Uhrf1bp1l/Itch/Tmem127/H2-Q7/Vamp3/Siah2/Mon2/Rap1a/H2-M3/Rcc2/Rabgap1l/Derl2/H2-Q6/Uvrag/Rabgef1/Vti1b/H2-Q4/Samd9l/H2-T23/Apoe/Pld4/Ptpn1/Tmem1	30
Rps7/Fcf1/Bms1/Wdr74/Rrp1/Rrs1/Zfp622/Riok1/Pes1/Mphosph10/Riok3/Nop56/Ebna1bp2	13
Ganab/Stt3b/H13/Rnf125/P4ha1/Aup1/Sec11c/Arl6ip1/Dnajb11/Krtcap2/Srpr/Derl2/Sel1l/Faf1/Sec63/Hsp90b1/Calr	17
Jun/Fos/Jund/Smad7/Junb/Hnrnpab/Crem/Atf2/Tcea1/Crebbp/Arnt/Ldb1/Mxd4/Tcf4/Gtf2a2/Tcf12/Cebpz/Gtf2h5/Ctnnb1/Chd4	20
Pde4b/Macf1/Rpl8/Nsf/Rpl38/Rps27/Rpl30/Rpl18a/Rps19/Rps13/Rpl14/Rps3/Sptbn1/Ifngr1/Hspb1/Adam10/Eif3a/Bcl11a/Nr3c1/Exoc4/Eif4g2/Arrb2/Map4/Ncoa2/Myd88/Ctnnb1/Ppp2ca/Dbnl/P2rx4/Iqsec1	30
Pnn/Sf3b1/Sart1/Srrm2/Snrpd3/Isy1/Lsm2/Snw1/Magoh/Srrm1/Rbm22/Ppwd1/Hnrnph1/Prpf8	14
Rnf125/B3gnt7/St6gal1/Mbtps1/H2-Q7/B3gnt5/Cux1/Ugcg/H2-M3/Unc50/Tvp23b/Xylt1/Arfgef1/Rer1/B3gnt2/Ergic3/Copb2/H2-Q6/Atp2c1/Dnm2/Golga7/H2-Q4/Sys1/Glg1/H2-T23/Numa1	26
Cyth1/Sla/Syk/Atp2b1/Gng5/Cd2/Traf1/Ikbkb/Kras/Birc2/Iqgap1/Ppp3ca/Gng2/Mtss1/Pten/Cnr2/Gna13/Chuk/Msn/Ptprc	20
Rb1cc1/Ikbkb/Ccnt2/Pcna/Snw1/Cnppd1/Cdk9/Ccnt1/Ccnd3/Uvrag/Gtf2h5/Chuk/Ccnd2	13
Hspa8/Pde4b/Macf1/Rpl8/Nsf/Rpl38/Rps27/Rpl30/Rpl18a/Rps19/Rps13/Rpl14/Rps3/Sptbn1/Ifngr1/Hspb1/Adam10/Eif3a/Bcl11a/Nr3c1/Exoc4/Eif4g2/Arrb2/Map4/Ncoa2/Myd88/Ctnnb1/Ppp2ca/Dbnl/P2rx4/Iqsec1	31
Arid1b/Smarce1/Brd9/Smarcc1/Arid1a/Smarca2	6
Hspa8/Gapdh/Rhob/H2-Ab1/Tpt1/Tcirg1/Map2k1/Lamtor2/Pik3c3/H2-Eb2/Rap1a/Kidins220/Zc3hav1/Pik3r4/Cyb561a3/Derl2/Ccz1/Uvrag/Lamtor3/Psap/Map1lc3a/Bst2/Vti1b/Apoe	24
Vps37b/Capg/Hepacam2/Arl8b/Clic4/Ist1/Nudc/Pik3c3/Celf2/Iqgap1/Mical1/Rcc2/Birc6/Uvrag/Flcn/Eml4/Hsp90b1/Gnl3/Safb/Gdi1	20
Hspa8/Pde4b/Macf1/Rpl8/Nsf/Rpl38/Rps27/Rpl30/Rpl18a/Rps19/Rps13/Rpl14/Rps3/Sptbn1/Ifngr1/Hspb1/Adam10/Eif3a/Bcl11a/Nr3c1/Exoc4/Eif4g2/Arrb2/Map4/Ncoa2/Myd88/Ctnnb1/Ppp2ca/Dbnl/P2rx4/Iqsec1	31
Rb1cc1/Ikbkb/Ccnt2/Prkag1/Pcna/Snw1/Cnppd1/Cdk9/Ccnt1/Ccnd3/Uvrag/Gtf2h5/Chuk/Ccnd2	14
Sf3b1/Sart1/Srrm2/Snrpd3/Lsm2/Prpf38b/Lsm8/Ik/Mfap1a/Prpf8	10
Hspa8/Hsp90ab1/Laptm5/Arl8b/H2-Q7/H2-Eb2/H2-M3/Lmbrd1/Cyb561a3/Vps13a/Fnip1/H2-Q6/Sidt2/Vti1b/H2-Q4/Spag9/Flcn/H2-T23/Uba1	19
Hspa8/Hsp90ab1/Laptm5/Arl8b/H2-Q7/H2-Eb2/H2-M3/Lmbrd1/Cyb561a3/Vps13a/Fnip1/H2-Q6/Sidt2/Vti1b/H2-Q4/Spag9/Flcn/H2-T23/Uba1	19
Sf3b1/Sart1/Snrpd3/Lsm2/Sart3/Snrnp70/Arfgef1/Luc7l2/Lsm8/U2surp/Prpf8	11
Myo1c/Pcid2/Nup50/Rae1/Nup88/Nup210/Kpnb1/Eny2/Ranbp2/Lbr/Nxf1	11
Sf3b1/Sart1/Snrpd3/Lsm2/Sart3/Snrnp70/Luc7l2/Lsm8/U2surp/Prpf8	10
Hspa8/Hsph1/Macf1/Tuba1a/Tubb5/Dynll1/Tpt1/Bod1l/Eif3a/Pbxip1/Kif21b/Fkbp4/Tuba1b/Rnf4/Map2k1/Csnk1d/Dynlt1c/Daxx/Nudc/Gabarap/Iqgap1/Map4/Reep3/Rcc2/Tbca/Rassf3/Ncoa2/Cltc/Map1lc3a/Dnm2/Eml4/Bcl	34
Fth1/Ftl1/Pik3c3/Plekhm1/Map1lc3a	5
Hsp90aa1/Macf1/Myo1c/Rps3/Plekho1/Diaph1/Ptprj/Akt2/Inpp5k/Plek/Dnm2	11
Tex10/Pelp1/Kdm6b/Chd8/Sirt1/Phf20/Mtf2/Kmt2c/Kdm6a/Kmt2a/Nsd1	11
Hspd1/Rps3/Timm8b/Atp5e/Pmpcb/Ndufa3/Ndufc1/Atp5c1/Mcur1/Chchd3/Bcl2/Ndufv1/Polg/Cox7b/Ak2/Chchd10/Atp5g1/Tufm/Ndufs6/Coq2/Ghitm/Csde1/Sfxn3/Slc25a51/Ndufs8/Cox7a2/Hadha/Higd1a/Ndufb5/Mtx1/H	35
Satb1/Top2b/Cenpa/Ddx6/Sirt1/Daxx/Ube2b/Mecp2/Cbx1/Atrx/Uhrf2/Pml/Uba1	13
Sf3b1/Sart1/Srrm2/Snrpd3/Lsm2/Lsm8/Ik/Mfap1a/Prpf8	9
Cytip/Macf1/Rhob/Akap13/Sptbn1/Inpp5d/Fryl/Exoc4/Map2k1/Pstpip1/Itch/Daxx/Prkcb/Iqgap1/Eno1/Akt2/Epb41/Cd2ap/Rhog/Fry/Ctnnb1/Rhoh/Dbnl/Myadm/Osbpl8/Numa1	26
H13/Ccdc47/Aup1/Arl6ip1/H2-Q7/Scd1/Zfyve27/Ktn1/Ergic3/Dnajb2/Derl2/Preb/Tram1/Tmco1/Atf6/H2-T23/Calr	17
Arid1b/Smarce1/Smarcc1/Arid1a/Smarca2	5
Hspa8/Hspa1a/Hspa1b/Hspd1/Birc3/Tuba1a/Tubb5/Atp2b1/Ms4a1/Myo1c/Pag1/Tpp1/Ahnak/Tuba1b/Lamtor2/Cd2/Cr1l/Cblb/Kras/Bsg/Birc2/Iqgap1/Cd55/Pgk1/Il6st/Lamtor3/Ctnnb1/Ppp2ca/Ptprc/Myadm/Atp5a1	31
Hspa8/Hspa1a/Hspa1b/Hspd1/Birc3/Tuba1a/Tubb5/Atp2b1/Ms4a1/Myo1c/Pag1/Tpp1/Ahnak/Tuba1b/Lamtor2/Cd2/Cr1l/Cblb/Kras/Bsg/Birc2/Iqgap1/Cd55/Pgk1/Il6st/Lamtor3/Ctnnb1/Ppp2ca/Ptprc/Myadm/Atp5a1	31
Hsp90aa1/Macf1/Atp2b1/Adgre5/Myo1c/Rps3/Sptbn1/Plekho1/Tmod3/Dgkz/Exoc4/Diaph1/Arpc5/Dynlt1c/Ldb1/Ptprj/Iqgap1/Akt2/Inpp5k/Rock1/Plek/Cd2ap/Hax1/Dock8/Ctnnb1/Dnm2/Dbnl/Atp6v1b2/Tln1/Myadm/Vim/A	32
Hspd1/Nsf/Trappc9/Plekhj1/Ccdc186/Arl5c/Myo18a/Adam10/Aftph/St6gal1/Mbtps1/Inpp5k/Xylt1/Arfgef1/Vamp2/Atp11b/Birc6/Atp2c1/Akap9/Dnm2/Golga7/Vrk1/Trappc6b/Sys1/Glg1/Pld4	26
Sf3b1/Sart1/Snrpd3/Lsm2/Sart3/Snrnp70/Arfgef1/Luc7l2/Lsm8/U2surp/Prpf8	11
Ccnt2/Pcna/Snw1/Cnppd1/Cdk9/Ccnt1/Ccnd3/Ccnd2	8
Atf2/Morf4l2/Phf20/Brd8/Epc1/Ep400/Phf20l1	7
Mrps6/Tomm6/Mrps12/Atp5e/Mrpl52/Pmpcb/Ndufa3/Ndufc1/Atp5c1/Chchd3/Ndufv1/Polg/Chchd10/Atp5g1/Ndufs6/Mrps7/Mrps23/Ndufs8/Hadha/Ndufb5/Mtx1/Mrpl15/Atp5a1	23
Dynll1/Capg/Rps3/Tpt1/Hepacam2/Bod1l/Diaph1/Myc/Arl8b/Csnk1d/Tbl1x/Nudc/Rae1/Cdc25b/Npm1/Map4/Nsfl1c/Cbx1/Rcc2/Epb41/Birc6/Cltc/Ik/Flcn/Eml4/Nedd9/Numa1	27
H13/Ccdc47/Aup1/Arl6ip1/H2-Q7/Scd1/Zfyve27/Ktn1/Ergic3/Derl2/Preb/Tram1/Tmco1/Atf6/H2-T23/Calr	16
Wdr74/Rrp1/Rrs1/Zfp622/Pes1/Ebna1bp2	6
Hspd1/Tmed5/Aftph/Tmed2/Sec24b/Vamp3/Lmbrd1/Arcn1/Vamp2/Ergic3/Copb2/Hax1/Tmed9/Cltc/Ergic2/Dnm2/Vti1b/Copa/Sec24c	19

Jun/Klf4/Myc/Sirt1/Polr2a/Ogt/Ctnnb1/Sp1	8
Fth1/Ftl1/Pik3c3/Plekhm1/Map1lc3a	5
Tmed5/H2-Q7/H2-M3/H2-Q6/Atp2c1/Pik3r1/Akap9/H2-Q4/Trappc6b/H2-T23	10
Ppp1r15a/Ier5/Ppp1r15b/Ppp3ca/Ppp2r2a/Ppp2r5a/Ppp2ca/Tox4	8
Ppp1r15a/Ier5/Ppp1r15b/Ppp3ca/Ppp2r2a/Ppp2r5a/Ppp2ca/Tox4	8
H2-Ab1/H2-Q7/H2-Eb2/H2-M3/H2-Q6/H2-Q4/H2-T23	7
Dynll1/Capg/Rps3/Hepacam2/Diaph1/Tbl1x/Nudc/Rae1/Map4/Rcc2/Epb41/Cltc/Ik/Flcn/Eml4/Numa1	16
Hspb1/Psmb3/Sec11c/Psmd3/Ubr1/Psmb6/Psmd2/Zfand2a/Supt20/Dnajb2/Eny2/Psmd11	12
Lamtor2/Pde3b/Rap1a/Elmo1/Lamtor3	5
Lamtor2/Pde3b/Rap1a/Elmo1/Lamtor3	5
Fos/Jund/Kdm5a/Cenpa/Orc3/Pold4/Irf4/Parp1/Nfe2l2/Npm1/Tonsl/Ctnnb1/Sp1/Chd4/Donson/Kat6a	16
Hspe1/Hspa1b/Hspd1/Mrps6/Rps3/Mrps12/Mrpl52/Pmpcb/Acss1/Aldh2/Polg/Tufm/Dhx30/Mrps7/Mrps23/Clpp/C1qbp/Hadha/Tfam/Hspa9/Glud1/Ptpn1/Mrpl15	23
H2-Q7/H2-M3/H2-Q6/Atp2c1/H2-Q4/H2-T23	6
Xist/Smchd1/Daxx/Birc2/Smarcc1/Ube2b/Usp7	7
Hspb1/Psmb3/Psmd3/Ubr1/Psmb6/Psmd2/Zfand2a/Dnajb2/Psmd11	9
Smarce1/Smarcc1/Arid1a/Smarca2	4
Ccnt2/Snw1/Cdk9/Ccnt1/Gtf2h5	5
Cux1/Unc50/Tvp23b/Rer1/Ergic3/Atp2c1/Golga7/Sys1	8
Pold4/Smarca5/Pcna/Ube2b/Rfc1/Tonsl/Donson/Zmiz2/Bcl6	9
Ccnt2/Snw1/Cdk9/Ccnt1	4
Cyth1/Sla/Syk/Gng5/Rb1cc1/Pik3c3/Kras/Nmt2/Iqgap1/Eno1/Pik3r4/Vps13a/Gng2/Pik3r1/Ctnnb1/Wipi2/Cnr2/Pml/Gna13/Zc3h12a/Socs3/Bcl2l11/Apoe/Numa1	24
Tmed5/Tmed2/Sec24b/Ergic3/Tmed9/Ergic2/Vti1b/Sec24c	8
Daxx/Birc2/Smarcc1/Ube2b/Usp7	5
Pnn/Sap18/Acin1/Casc3/Magoh	5
Hsp90aa1/Macf1/Atp2b1/Adgre5/Myo1c/Rps3/Sptbn1/Plekho1/Diaph1/Ptprj/Akt2/Inpp5k/Plek/Dock8/Dnm2	15
Hspb1/Psmb3/Psmd3/Ubr1/Psmb6/Psmd2/Zfand2a/Dnajb2/Psmd11	9
Aftph/Sec24b/Vamp3/Arcn1/Vamp2/Copb2/Cltc/Vti1b/Copa/Sec24c	10
Hspa8/Hsp90ab1/Hsp90aa1/Actg1/Itga4/Fkbp4/Exoc4/Arpc5/Polg/Sirt1/Dynlt1c/Atp5g1/Zfyve27/Prkcb/Got1/Iqgap1/Fkbp1a/Mical1/Sri/Bin1/Git2/Dnm2/Ppp2ca/Srsf10/Setx/Copa/P2rx4	27
Pcna/Nrm/Lbr/Lmnb1	4
Rps8/Uba52/Rps5/Rpl18/Rplp2/Rpsa/Rpl19/Rpl9-ps6/Rps24/Rps26/Rps4x/Fau/Rpl8/Rpl38/Rps21/Rps7/Rpl37/Rps27/Rps20/Rpl30/Rps16/Rps2/Rpl13a/Rpl18a/Rpl13/Mrps6/Rps19/Rps13/Rpl35/Rpl21/Rps12/Rps27a/Rpl	52
Hspa8/Hspa1a/Hspa1b/Ubb/Jun/Hsp90ab1/Hsp90aa1/Dnaja1/Uba52/Nfkbia/Hspd1/Cacybp/Actg1/Tubb5/Smad7/Syk/Mul1/Rps27a/H13/Hltf/Traf4/H2-Ab1/Laptm5/Arih1/Sh3kbp1/Tuba1b/Myc/Aup1/Ubc/Bcl2/Erlin1/Itch/A	65
Hspa8/Hspa1a/Hspa1b/Ubb/Jun/Hsp90ab1/Hsp90aa1/Dnaja1/Uba52/Nfkbia/Hspd1/Cacybp/Actg1/Tubb5/Smad7/Syk/Mul1/Rps27a/H13/Hltf/Traf4/H2-Ab1/Laptm5/Arih1/Sh3kbp1/Tuba1b/Myc/Aup1/Ubc/Bcl2/Erlin1/Arrb2/	62
Jun/Dnaja1/Nr4a1/Fos/Chchd2/Nfkbia/Klf4/Mef2c/Jund/Foxp1/Mef2d/Arid5a/Satb1/Tsc22d3/Atf2/Rps3/Ifrd1/Rnf6/Hspb1/Cited2/Crebbp/Fkbp4/Rnf4/Hes6/Myc/Bcl2/Arnt/Parp1/Sirt1/Nfkbid/Ldb1/Hif1an/Daxx/Nfe2l2/Tcf4/	67
Rps5/Rps26/Rps7/Rpl30/Rps2/Zfp36/Rpl13a/Rps13/Rpl35/Rpl24/Rc3h2/Rps3/Dazap1/Sf3b1/Pcbp1/G3bp1/Eif3a/Dhx36/Srrm2/Pabpc4/Hnrnpl/Rbm5/Ddx6/Zfp36l2/Eif4g2/Celf1/Hnrnpa0/Purb/Snrnp70/Celf2/Rbms1/Nhp2	59
Jun/Dnaja1/Nr4a1/Fos/Nfkbia/Klf4/Jund/Foxp1/Arid5a/Atf2/Ifrd1/Rnf6/Hspb1/Cited2/Crebbp/Fkbp4/Rnf4/Hes6/Arnt/Parp1/Sirt1/Nfkbid/Ldb1/Hif1an/Daxx/Nfe2l2/Smarce1/Nfatc1/Ep300/Prkcb/Npm1/Med13/Gtf2a2/Ddrgk1	55
Dnajb1/Hspa1a/Trim25/Naca/Kdm5a/Arid5a/Rbm39/Med15/Sap18/Rere/Pbxip1/Limd1/Cited2/Crebbp/Bcl11a/Nr3c1/Nmi/Irf4/N4bp2l2/Sub1/Hipk3/Rsf1/Sirt1/Ets1/Tbl1x/Ldb1/Jmjd1c/Daxx/Tcf4/Ep300/Prkcb/Npm1/Med13	61
Jun/Cyth1/Dennd4a/Eef1b2/Ralgapa1/Akap13/Sbf1/Gpsm3/Rasal3/Lamtor2/Bcar3/Gdi2/Krit1/Prr5/Eif2b2/Dennd1b/Rap1a/Rabgap1/Iqgap1/Tagap/Elmo1/Arfgef1/Rasa2/Rcc2/Arhgap15/Rabgap1l/Git2/Ccz1/Fnip1/Preb/Do	52
Jun/Cyth1/Dennd4a/Eef1b2/Ralgapa1/Akap13/Sbf1/Gpsm3/Rasal3/Lamtor2/Bcar3/Gdi2/Krit1/Prr5/Eif2b2/Dennd1b/Rap1a/Rabgap1/Iqgap1/Tagap/Elmo1/Arfgef1/Rasa2/Rcc2/Arhgap15/Rabgap1l/Git2/Ccz1/Fnip1/Preb/Do	52
Dnajb1/Hspa8/Hspe1/Hspa1a/Hspa1b/Hsp90ab1/Hsp90aa1/Dnaja1/Naca/Ptges3/Nudcd3/Nudc/St13/Npm1/Dnajb11/Ube4b/Dnajb2/Tbce/Hspa9/Hspa14/Srsf10/Hsp90b1/Calr	23
Birc3/Trim25/Mul1/Hltf/Rc3h2/Zfp91/Rnf213/Rnf6/Rnf125/Arih1/Malt1/Ube3b/Rnf4/Trim59/Neurl3/Znrf2/Ubr1/Itch/Brap/Fbxl3/Ube2j2/Ube2e1/Ube2d1/Herc4/Cblb/Siah2/Birc2/Ube2b/Cul1/Ube2h/Mylip/Ube4b/Med21/Ube2	53
Birc3/Trim25/Mul1/Hltf/Rc3h2/Zfp91/Rnf213/Rnf6/Rnf125/Arih1/Malt1/Ube3b/Rnf4/Trim59/Neurl3/Znrf2/Ubr1/Itch/Brap/Fbxl3/Ube2j2/Ube2e1/Ube2d1/Herc4/Cblb/Siah2/Birc2/Ube2b/Cul1/Ube2h/Mylip/Ube4b/Med21/Chfr/	51
Jun/Cyth1/Dennd4a/Eef1b2/Ralgapa1/Akap13/Sbf1/Rasal3/Lamtor2/Bcar3/Gdi2/Prr5/Eif2b2/Dennd1b/Rap1a/Rabgap1/Iqgap1/Tagap/Elmo1/Arfgef1/Rasa2/Rcc2/Arhgap15/Rabgap1l/Git2/Ccz1/Fnip1/Preb/Dock8/Rasgef1b	50
Phf14/Kdm5a/Kmt2e/Nap1l1/Chd1/Chd8/Sbno2/Smarca5/Sart3/Rsf1/Sirt1/Tbl1x/Daxx/Mtf2/Brd9/Prkcb/Npm1/Ipo7/Smarcc1/Kmt2c/Anp32b/Bptf/Atrx/Zmynd11/Stat1/Sbno1/Ing1/Usp15/Tonsl/Uhrf2/Usp16/Smarca2/Nap1	40
Nr4a1/Jund/Foxp1/Arid5a/Rnf6/Fkbp4/Rnf4/Parp1/Sirt1/Daxx/Smarce1/Ep300/Prkcb/Med13/Pcna/Snw1/Cnot1/Arid1a/Brd8/Stat1/Ncoa2/Pik3r1/Fus/Ctnnb1/Trip12/Nsd1/Tob2/Calr	28
Cyth1/Dennd4a/Eef1b2/Akap13/Sbf1/Lamtor2/Bcar3/Eif2b2/Dennd1b/Rap1a/Tagap/Elmo1/Arfgef1/Rcc2/Ccz1/Fnip1/Preb/Dock8/Rasgef1b/Gapvd1/Lamtor3/Rabgef1/Dock2/Ralgps2/Mycbp2/Flcn/Rapgef6/Rab3gap1/Ccd	31
Dnajb1/Hspa8/Hspa1a/Hspa1b/Hsp90ab1/Dnaja1/Nfkbia/Zfp36/Ptges3/Rps3/Ahsa1/Nr3c1/Fkbp4/St13/Fkbp1a/Dnajb9/Kpnb1/Nfkb1/Eif2ak3/Dnajb2/Cltc/Tfam/Hspa9/Faf1/Hspa14/Usp19	26
Hsp90aa1/Nsf/Akap13/Myo1c/Cdc42se2/Sptbn1/Ccdc186/Exoc4/Lsm2/Map2k1/Diaph1/Tmem127/Gdi2/Xpo1/Golga4/Ipo7/Dennd1b/Rap1a/Rabgap1/Iqgap1/Eno1/Mical1/Kpnb1/Xpo6/Rock1/Rcc2/Rabgap1l/Bin1/Preb/Ra	39
Trim25/Naca/Kdm5a/Rbm39/Rere/Cited2/Crebbp/Nr3c1/Irf4/Sub1/Sirt1/Ets1/Daxx/Ep300/Prkcb/Npm1/Med13/Birc2/Kmt2c/Snw1/Med21/Cebpz/Brd8/Ncoa2/Eny2/Fus/Ctnnb1/Usp16/Smarca2/Trim12a/Pml/Nucks1/Zmiz2/	34
Rps7/Zfp36/Dazap1/Dhx36/Pabpc4/Hnrnpl/Zfp36l2/Celf1/Hnrnpa0/Celf2/Rbms1/Nhp2/Taf15/Fus/Larp1/Serbp1/Secisbp2/Zc3h12a/Pum2/Larp4/Larp4b	21
Birc3/Trim25/Mul1/Hltf/Rc3h2/Rnf6/Rnf125/Arih1/Ube3b/Rnf4/Trim59/Neurl3/Znrf2/Ubr1/Itch/Brap/Ube2d1/Herc4/Cblb/Siah2/Birc2/Cul1/Mylip/Ube4b/Med21/Chfr/Rnf34/Rabgef1/Uhrf2/Trim12a/Ranbp2/Arih2/Mycbp2/Peli1	38
Birc3/Trim25/Mul1/Hltf/Rc3h2/Rnf6/Rnf125/Arih1/Ube3b/Rnf4/Trim59/Neurl3/Znrf2/Ubr1/Itch/Brap/Ube2d1/Herc4/Cblb/Siah2/Birc2/Cul1/Mylip/Ube4b/Med21/Chfr/Rnf34/Rabgef1/Uhrf2/Trim12a/Arih2/Mycbp2/Peli1/Trip12/	37
Nsf/Akap13/Myo1c/Cdc42se2/Ccdc186/Exoc4/Lsm2/Map2k1/Diaph1/Tmem127/Gdi2/Xpo1/Golga4/Ipo7/Dennd1b/Rap1a/Rabgap1/Iqgap1/Mical1/Kpnb1/Xpo6/Rock1/Rcc2/Rabgap1l/Rabgef1/Ranbp2/Tnpo1/Mycbp2/Cyfi	34
Nsf/Dynll1/Top2b/Myo1c/Dgkz/Prdx3/Neil1/Map2k1/Sirt1/Dynlt1c/Tbl1x/Tcf4/Ep300/Polr2a/Prrc2c/Pcna/Usp7/Epb41/Vamp2/Cd2ap/Pik3r1/Cltc/Ctnnb1/Sae1/Ppp2ca/Sp1/Dbnl/Nipbl/Numa1/Foxn3	30
Hltf/Chd1/Chd8/Smarca5/Atrx/Arid1a/Ep400/Smarca2/Chd2/Chd4/Chd7	11
Rpsa/Eif1/Rpl35/Xist/Eif3k/Ccdc47/Snrpd3/Naa10/Itch/Npm1/Snrnp70/Csnk2a1/Naa15/Srpr/C1qbp/Derl2/Ythdf3/Larp1/Serbp1/Secisbp2/Nemf/Tmco1/Zc3h12a/Prmt1	24
Hspa1a/Hspa1b/Rps21/Pelp1/Srrm2/Exoc4/Map2k1/Parp1/Daxx/Smarce1/Eif3e/Npm1/Birc2/Csnk2a1/Smarcc1/Mecp2/Epb41/Hax1/Tnks2/Nipbl/Hypk	21
Top2b/Hltf/Ptges3/Rps3/G3bp1/Neil1/Pold4/Chd1/Chd8/Dhx36/Smarca5/Polg/Tdp2/Dclre1c/Pcna/Bptf/Atrx/Rfc1/Arid1a/Top3b/Ep400/Smarca2/Chd2/Btaf1/Chd4/Chd7/Pld4/Dnase2a	28
Klf4/Crebbp/Foxo1/Mtf2/Nfe2l2/Ep300/Ccnt2/Cdk9/Ehmt1/Nfkb1/Per1/Stat1/Cnot2/Tfam/Ctnnb1/Sp1/Chd4	17
Hspd1/Mul1/Ptges3/Rnf125/Chd8/Crebbp/Sirt1/Daxx/Ep300/Npm1/Usp7/Ehmt1/Pttg1ip/Rnf34/Stk11	15
Hspa8/Hspa1a/Hspa1b/Dnajb11/Dnajb9/Derl2/Hspa9/Hspa14/Dnajc3	9
Foxp1/Arid5a/Rnf6/Rnf4/Daxx/Ep300/Prkcb/Snw1/Nsd1/Calr	10
Hspa8/Phlda1/Capg/Myo1c/Sptbn1/Tpp1/Plekhb2/Anxa7/Sgk3/Snx12/Krit1/Fchsd2/Pxdc1/Npm1/Gltp/Dennd1b/Iqgap1/Ogt/Nsfl1c/Rasa2/Epb41/Vamp2/Snx30/Bin1/Ncf4/Nisch/Rnf34/Mtss1/Flii/Psap/Wipi2/Map1lc3a/Ara	41
Stk17b/Wnk1/Syk/Akap13/Clk1/Sgk3/Map2k1/Hipk3/Csnk1d/Prkcb/Ikbkb/Csnk1g3/Smg1/Csnk2a1/Sik2/Camk2d/Akt2/Prkag1/Pik3r4/Stk38/Cdk9/Map4k2/Rock1/Eif2ak3/Riok1/Tlk1/Mast4/Ccnd3/Map3k2/Cdk11b/Sbk1/S	40
Jun/Fos/Smad7/Zeb2/Cited2/Crebbp/Parp1/Purb/Ipo7/Fkbp1a/Tcf12/Snw1/Usp15/Ctnnb1/Pml	15
Dnajb1/Hspa1a/Arid5a/Sap18/Rere/Pbxip1/Limd1/Cited2/Crebbp/Bcl11a/N4bp2l2/Hipk3/Sirt1/Tbl1x/Daxx/Tcf4/Snw1/Mecp2/C1qbp/Zmynd11/Irf2bp2/Nsd1/Tob2	23
Top2b/Hltf/G3bp1/Chd1/Chd8/Dhx36/Smarca5/Bptf/Atrx/Rfc1/Arid1a/Ep400/Smarca2/Chd2/Btaf1/Chd4/Chd7	17
Dnajb1/Hspa8/Hspa1a/Hspa1b/Hsp90ab1/Hsp90aa1/Hspb1/Ccdc47/Hspa9/Hspa14	10
Hsp90ab1/Wnk1/Klf4/Hspb1/Malt1/Map2k1/Cab39/Daxx/Ccnt2/Npm1/Iqgap1/Kidins220/Prkag1/Cnppd1/Ccnt1/Pik3r1/Ccnd3/Lamtor3/Stk11/Rhoh/Ccnl1/Socs3/Dnajc3/Ccnl2/Ccnd2	25
Stk17b/Wnk1/Sgk3/Hipk3/Csnk1d/Ikbkb/Csnk1g3/Smg1/Csnk2a1/Sik2/Akt2/Pik3r4/Stk38/Map4k2/Rock1/Eif2ak3/Riok1/Tlk1/Mast4/Sbk1/Stk11/Rps6kb1/Csnk1a1/Vrk1/Riok3/Srpk2	26
Hspa1a/Hsp90ab1/Hspd1/Mbnl1/Rc3h2/Dhx36/Tuba1b/Strbp/Dhx30/Cltc/Sidt2/Ilf2/Msn/Vim	14
Eif1/Eif3f/Eef1b2/Pcbp1/Eif3k/Eif3a/Eif4g2/Eif3e/Tufm/Purb/Eif2b2/Eif1ax/Eif4e2/Larp1/Eif2s2/Eif2s3x	16
Dnajb1/Hspa8/Hspe1/Dnaja1/Hspd1/Ptges3/Ahsa1/St13/Birc2/Dnajb9/Dnajb2/Bin1/Fnip1/Hspa9/Lbr/Dnajc3	16
Atf2/Gm16286/Naa50/Crebbp/Naa10/Sirt1/Smarce1/Ep300/Nmt2/Ogt/Naa15/Epc1/Cers4/Kat6a/Esco1	15
Hltf/G3bp1/Chd1/Chd8/Dhx36/Smarca5/Ddx6/Ddx50/Dhx30/Atrx/Ddx47/Ep400/Smarca2/Chd2/Setx/Chd4/Ddx24/Chd7/Ddx52	19
Bod1l/Bcl2/Foxo1/Smg5/Ppp2r2a/Rps6kb1/Ppp2ca/Ptpn1/Vim	9
Nr4a1/Mef2c/Mef2d/Ralgapa1/Atf3/Top2b/Cenpa/Atf2/Bcl11a/Hes6/Vapa/Bcl2/Arnt/Tcf4/Ikbkb/Gtf2a2/Sri/Tcf12/Zfp318/Uba2/Pik3r1/Bhlhe40/Sae1/Ppp2ca/Irak3/Pml/Atf6/Chuk/Hexa	29
Ppp1r15a/Wnk1/Syk/Irs2/Bod1l/Pstpip1/Chchd3/Bcl2/Foxo1/Siglecg/Ikbkb/Smg5/Jak1/Iqgap1/Cabin1/Eif2ak3/Pik3r1/Ppp2r2a/Ctnnb1/Rps6kb1/Ppp2ca/Hsp90b1/Ptpn1/Vim	24
Hsp90ab1/Hsp90aa1/Fkbp4/Bin1/Ppp2r2a/Ppp2ca/Apoe	7
Hspd1/Kdm5a/Kmt2e/Laptm5/Chd1/Chd8/Daxx/Mtf2/Zranb1/Brd9/Bptf/Atrx/Dnajb2/Zmynd11/Ing1/Usp15/Zfand6/Phip/Pml/Kmt2a/Prpf8/Kdm7a	22
Pelp1/Hspb1/Rnf4/Aup1/Rae1/Birc2/Nsfl1c/Tax1bp1/Uba2/Dnajb2/Faf1/Usp16/Serbp1/Pml/Herc2	15
Fos/Foxp1/Klf10/Egr1/Nr3c1/Myc/Ubtf/Polr2a/Npm1/Stat1	10
Arid5a/Rnf4/Parp1/Pcna/Cnot1/Ncoa2/Pik3r1/Fus/Ctnnb1/Nsd1	10
Atf2/Gm16286/Naa50/Crebbp/Naa10/Ep300/Naa15/Epc1/Kat6a/Esco1	10
Eif1/Eif3f/Eif3k/Eif3a/Eif4g2/Eif3e/Eif2b2/Eif1ax/Eif4e2/Eif2s2/Eif2s3x	11
Zfp36/Ccnt2/Wdr43/Anp32b/Zfp326/Ccnt1/Bin1/Rtf1/Paf1/Scaf8/Pabpn1	11
Ppp1r15a/Irs2/Bod1l/Pstpip1/Bcl2/Foxo1/Ikbkb/Smg5/Jak1/Iqgap1/Cabin1/Eif2ak3/Pik3r1/Ppp2r2a/Ctnnb1/Rps6kb1/Ppp2ca/Hsp90b1/Ptpn1/Vim	20
Pelp1/Rnf4/Uba2/Serbp1/Pml/Herc2	6
Sirt1/Arrb2/Pde3b/Npm1/Ppp2ca/Srsf5	6
Dusp1/Sirt1/Arrb2/Ptprj/Nfatc1/Iqgap1/Spag9/Prmt1	8
Atf2/Gm16286/Naa50/Crebbp/Naa10/Smarce1/Ep300/Ogt/Naa15/Epc1/Kat6a/Esco1	12
Hsp90aa1/Eif3a/Nr3c1/Cd2/Cblb/Sh2b3/Bank1/Ptpn2/Pcna/Stap1/Pik3r1/Blnk/Pten/Nedd9/Ptpn1	15
Eif1/Eif3f/Eef1b2/Pcbp1/Eif3k/Eif3a/Eif4g2/Eif3e/Tufm/Purb/Eif2b2/Eif1ax/Eif2ak3/Eif4e2/Larp1/Eif2s2/Eif2s3x	17
Eif1/Eif3f/Eef1b2/Eif3k/Eif3a/Eif4g2/Eif3e/Tufm/Eif2b2/Eif1ax/Eif4e2/Eif2s2/Eif2s3x	13
Hspa1a/Hspa1b/Hsp90ab1/Hsp90aa1/Klf4/Mef2c/Mef2d/Top2b/Dhx36/Parp1/Smg5/Mecp2/Sp1/Akap8l/Chd4/Nipbl	16
Hspa8/Hspe1/Hspa1a/Hspa1b/Hsp90ab1/Hsp90aa1/Hsph1/Hspd1/Atad1/Nsf/Rnf213/Atp5e/Kif21b/Atp5c1/Smarca5/Rfc1/Hspa9/Hspa14/Smarca2/Hsp90b1/Hspa4/Atp5a1	22
Hspa1a/Hsp90ab1/Hsp90aa1/Gapdh/Crebbp/Cltc/Ctnnb1/Numa1	8
Arid5a/Med13/Brd8/Ncoa2/Fus/Trip12/Nsd1	7
Jun/Foxp1/Atf3/Satb1/Crem/Bach2/Nfatc3/Prdm2/Zeb2/Mynn/Bcl11a/Nr3c1/Hes6/Myc/Foxp4/Foxo1/Mxd4/Nfatc1/Purb/Zbtb20/Zbtb21/Eno1/Nfkb1/Zfp148/Etv3/Bhlhe40/Cc2d1b/Bcl6	28
Kdm5a/Kdm6b/Crebbp/Sbno2/Jmjd1c/Foxo1/Ep300/Mecp2/Per1/Sbno1/Kdm6a/Tox4/Bcl6	13
Jun/Foxp1/Atf3/Satb1/Crem/Bach2/Nfatc3/Prdm2/Zeb2/Mynn/Bcl11a/Nr3c1/Hes6/Myc/Foxp4/Foxo1/Mxd4/Nfatc1/Purb/Zbtb20/Zbtb21/Eno1/Nfkb1/Zfp148/Etv3/Bhlhe40/Cc2d1b/Bcl6	28
Phlda1/Capg/Plekhb2/Sgk3/Snx12/Krit1/Fchsd2/Pxdc1/Npm1/Dennd1b/Iqgap1/Ogt/Epb41/Snx30/Ncf4/Nisch/Rnf34/Flii/Wipi2/Arap2/Tln1/Osbpl8/Snx29/Numa1	24
Rps5/Rpl19/Rps4x/Rpl8/Rpl37/Mrps6/Rps13/Rps3/Npm1/Rrs1/Mrps7	11
Snrpd3/Sart3/Ccnt2/Snrnp70/Cdk9/Ccnt1/Rbm22/Prpf8	8
Rps3/Rnf125/Arih1/Rnf4/Aup1/Siah2/Uba2/Arih2	8
Tsc22d3/Crebbp/Sirt1/Tcf4/Tcf12/Bhlhe40/Sp1	7
Laptm5/Daxx/Dnajb2/Usp15/Pml	5
Ube2j2/Ube2e1/Ube2d1/Ube2b/Ube2h/Ube2f/Birc6/Ube2g1	8
H2-Q7/H2-Eb2/H2-M3/H2-Q6/Dock2/H2-Q4/H2-T23	7
Zfp36/Irs2/Arrb2/H2-Q7/H2-M3/H2-Q6/H2-Q4/H2-T23/Srpk2	9
Hsp90ab1/Wnk1/Hspb1/Map2k1/Cab39/Daxx/Ccnt2/Npm1/Iqgap1/Kidins220/Prkag1/Cnppd1/Ccnt1/Ccnd3/Stk11/Ccnl1/Dnajc3/Ccnl2/Ccnd2	19
Klf4/Smad7/Kdm6b/Chd8/Tbl1x/Foxo1/Ptprj/Tcf4/Ep300/Csnk2a1/Cd2ap/Ctnnb1	12
Rpsa/Eif3k/Ccdc47/Naa10/Naa15/C1qbp/Ythdf3/Serbp1/Tmco1/Zc3h12a	10
Hspa8/Hsp90ab1/Nfkbia/H2-Ab1/Tpp1/Fkbp4/Brap/H2-Q7/H2-Eb2/Gltp/H2-M3/Fkbp1a/Ogt/Kpnb1/Ppp3ca/Nktr/Pex7/H2-Q6/Hadha/Pik3r1/Cltc/Dbi/Psap/Rps6kb1/Ppig/Csnk1a1/Ppwd1/Tnpo1/H2-Q4/Lbr/H2-T23/Apoe/	33
Crebbp/Foxo1/Ep300/Ccnt2/Cdk9/Nfkb1/Tfam	7
Syk/Fkbp4/Bcar3/Pde3b/Cblb/Stap1/Epb41/Pik3r1/Sfn/Rtf1/Csnk1a1/Scaf8/Socs3	13
Arid5a/Rc3h2/Dazap1/Csde1/Zc3h12a	5
Fkbp4/Fkbp3/Fkbp1a/Nktr/Ppig/Ppwd1/Ranbp2	7
Klf4/Atf2/Egr1/Sirt1/Foxo1/Polr2a/Mecp2/Stat1/Chd7/Nipbl	10
Cited2/Egr1/Ets1/Pcna/Stat1/Sp1	6
Atf2/Gm16286/Naa50/Crebbp/Naa10/Smarce1/Ep300/Ogt/Naa15/Epc1/Kat6a/Esco1	12
Foxp1/Malt1/Cd2/Ahcy/Ldb1/Tcf4/Zfyve27/Myd88/Pabpn1/Ccdc88c	10
Atf2/Zdhhc3/Gm16286/Naa50/Crebbp/Naa10/Zdhhc18/Sirt1/Smarce1/Ep300/Cblb/Mboat7/Nmt2/Kmt2c/Ogt/Naa15/Epc1/Hadha/Lpgat1/Cers4/Kat6a/Esco1	22
Rps24/Eif3f/Rps19/Celf1/Eif2ak3/Larp1/Eif2s2	7
Atf2/Zdhhc3/Gm16286/Naa50/Crebbp/Naa10/Zdhhc18/Sirt1/Smarce1/Ep300/Mboat7/Nmt2/Ogt/Naa15/Epc1/Hadha/Lpgat1/Cers4/Kat6a/Esco1	20
Dnajb1/Dnaja1/Rps3/Nr3c1/St13/Fkbp1a/Dnajb9/Dnajb2	8
Arid1b/Rnf4/Smarca5/Cabin1/Arid1a/Nap1l4/Vrk1/Supt16	8
Ube2j2/Ube2e1/Ube2d1/Ube2b/Ube2h/Birc6/Ube2g1	7
Rps7/Rps13/Dhx36/Celf1/Larp1/Gnl3	6
Fkbp4/Fkbp3/Fkbp1a/Nktr/Ppig/Ppwd1/Ranbp2	7
Hsp90ab1/Rhob/Tuba1a/Tubb5/Rab43/Arl5c/Bms1/Fkbp4/Tuba1b/Rasd1/Arl8b/Rala/Tufm/Kras/Gimap6/Eif2b2/Rap1a/Srpr/Rhog/Dnm2/Arl4a/Rhoh/Gna13/Glud1/Rrad/Gnl3/Eif2s3x	27
Gm16286/Naa50/Naa10/Naa15	4
Ubb/Uba52/Rps27a/Ubc	4
Atf2/Naa50/Crebbp/Ep300/Epc1/Kat6a/Esco1	7
Ssh2/Dusp1/Dusp5/Ssu72/Ptprj/Cdc25b/Ptpn2/Ppp3ca/Dusp16/Ptp4a2/Pten/Ppp2r5a/Ppm1g/Ppp2ca/Ptprc/Ptpn1	16
Eif3a/Nr3c1/Cd2/Cblb/Sh2b3/Ptpn2/Pcna/Stap1/Pik3r1/Ptpn1	10
Wnk1/Rps7/Rpl37/Rps20/Smad7/Dynll1/Id3/Dgkz/Bcl2/Daxx/Camk2d/Cd55/Fry/Fnip1	14
Nfkbia/Nfkbid/Hif1an/Ep300/Npm1/Faf1	6
Akap13/Map2k1/Iqgap1/Spag9	4
Ppp3ca/Nktr/Ppig/Ppwd1	4

Hsp90ab1/Cbx1/Ctnnb1/Nop56	4
Itga4/H2-Ab1/Ep400/Ppp2ca	4
Tubb5/H2-Q7/H2-M3/H2-Q6/H2-Q4/H2-T23/Atp5a1	7
Hsp90ab1/Hsp90aa1/Ptges3/Pcna/Ncoa2	5
