# Supplementary material for: The Regulation Network of Glycerolipid Metabolism as Coregulators of Immunotherapy-Related Myocarditis
Source: Cardiovasc Ther. 2023 Jun 21;2023:8774971. doi: 10.1155/2023/8774971 (PMC10307211; doi:10.1155/2023/8774971)
Supplement: Supplementary 4 — Table S4: the Kyoto Encyclopedia of Genes and Genomes (KEGG) pathway enrichment analysis for PTT-related DEGs of T cell subpopulation. [file 8774971.f4.pdf]

Table S4

	ID	Description	GeneRatio	BgRatio	pvalue	p.adjust	qvalue	geneID	Count
**mmu05171**	mmu05171	Coronavirus disease - COVID-19	59/579	248/9010	2.62E-19	7.89E-17	5.27E-17	Jun/Rps8/Fos/Uba52/Rps5/Rpl18/Rplp2/Rpsa/Nfkbia/Rpl19/Rps24/Rps26/Rps4x/Fau/Rpl8/Rpl38/Rps21/Rps7/Rpl37/Rps27/Rps20/Rpl30/Rps16/Rps2/Syk/Rpl13a/Rpl18a/Rpl13/Rps19/Rps13/Rpl35/Rpl21/Rps12/Rps27a/	59
**mmu03010**	mmu03010	Ribosome	49/579	179/9010	6.34E-19	9.54E-17	6.37E-17	Rps8/Uba52/Rps5/Rpl18/Rplp2/Rpsa/Rpl19/Rps24/Rps26/Rps4x/Fau/Rpl8/Rpl38/Rps21/Rps7/Rpl37/Rps27/Rps20/Rpl30/Rps16/Rps2/Rpl13a/Rpl18a/Rpl13/Mrps6/Rps19/Rps13/Rpl35/Rpl21/Rps12/Rps27a/Rpl28/Rpl24/	49
**mmu04141**	mmu04141	Protein processing in endoplasmic reticulum	37/579	172/9010	4.06E-11	4.08E-09	2.72E-09	Dnajb1/Hspa8/Hspa1a/Hspa1b/Hsp90ab1/Hsp90aa1/Dnaja1/Ppp1r15a/Hsph1/Ganab/Stt3b/Mbtps1/Os9/Bcl2/Ube2j2/Ube2d1/Nfe2l2/Sec24b/Dnajb11/Nsfl1c/Cul1/Herpud1/Ube4b/Eif2ak3/Dnajb2/Derl2/Preb/Ube2g1/Sel1	37
**mmu04662**	mmu04662	B cell receptor signaling pathway	21/579	81/9010	2.31E-08	1.43E-06	9.56E-07	Jun/Fos/Nfkbia/Pik3ap1/Syk/Nfatc3/Inpp5d/Malt1/Map2k1/Nfatc1/Prkcb/Ikbkb/Kras/Akt2/Nfkb1/Ppp3ca/Pik3r1/Blnk/Cd19/Cd81/Chuk	21
**mmu05167**	mmu05167	Kaposi sarcoma-associated herpesvirus infection	38/579	224/9010	2.65E-08	1.43E-06	9.56E-07	Ubb/Jun/Fos/Uba52/Nfkbia/Zfp36/Syk/Rps27a/Nfatc3/Ifngr1/Crebbp/Map2k1/Myc/Gng5/Ubc/H2-Q7/Pik3c3/Nfatc1/Ep300/Ikbkb/Kras/H2-M3/Jak1/Akt2/Nfkb1/Ppp3ca/Gng2/Ifnar1/Cd86/H2-Q6/Stat1/Pik3r1/Il6st/Ctnnb1/I	38
**mmu05132**	mmu05132	Salmonella infection	41/579	253/9010	2.85E-08	1.43E-06	9.56E-07	Jun/Hsp90ab1/Hsp90aa1/Cyth1/Fos/Gapdh/Nfkbia/Birc3/Rhob/Tuba1a/Actg1/Tubb5/Dynll1/Rps3/Ahnak/Exoc4/Tuba1b/Map2k1/Myc/Bcl2/Arpc5/Arl8b/Dynlt1c/Pik3c3/Rala/Ikbkb/Birc2/Akt2/Elmo1/Nfkb1/Rhog/Plekhm1/M	41
**mmu04120**	mmu04120	Ubiquitin mediated proteolysis	29/579	147/9010	4.26E-08	1.81E-06	1.21E-06	Ubb/Uba52/Birc3/Rps27a/Ube3b/Herc1/Ubc/Itch/Ube2j2/Ube2e1/Ube2d1/Herc4/Cblb/Birc2/Ube2b/Cul1/Ube2h/Ube4b/Uba2/Ube2f/Birc6/Ube2g1/Cul5/Sae1/Trip12/Pml/Herc2/Socs3/Uba1	29
**mmu04068**	mmu04068	FoxO signaling pathway	27/579	131/9010	4.81E-08	1.81E-06	1.21E-06	Klf2/S1pr4/Irs2/S1pr1/Sgk3/Crebbp/Atg12/Gadd45b/Map2k1/Sirt1/Foxo1/Ep300/Ikbkb/Kras/Gabarap/Akt2/Prkag1/Usp7/Gadd45g/Pik3r1/Pten/Stk11/Chuk/Bcl2l11/Bcl6/Prmt1/Ccnd2	27
**mmu05235**	mmu05235	PD-L1 expression and PD-1 checkpoint pathway in cancer	21/579	88/9010	1.1E-07	3.67E-06	2.45E-06	Jun/Fos/Nfkbia/Nfatc3/Ifngr1/Map2k1/Nfatc1/Ikbkb/Kras/Csnk2a1/Jak1/Akt2/Nfkb1/Ppp3ca/Stat1/Pik3r1/Myd88/Pten/Rps6kb1/Eml4/Chuk	21
**mmu05166**	mmu05166	Human T-cell leukemia virus 1 infection	39/579	250/9010	1.78E-07	5.13E-06	3.43E-06	Jun/Fos/Nfkbia/Zfp36/H2-Ab1/Atf2/Nfatc3/Crebbp/Egr1/Map2k1/Myc/Ets1/H2-Q7/Xpo1/Nfatc1/Ep300/Ikbkb/H2-Eb2/Kras/Tnfrsf13c/H2-M3/Jak1/Akt2/Nfkb1/Ppp3ca/Tspo/H2-Q6/Pik3r1/Ccnd3/Pten/H2-Q4/Nfkb2/Relb/Ch	39
**mmu05169**	mmu05169	Epstein-Barr virus infection	37/579	231/9010	1.88E-07	5.13E-06	3.43E-06	Jun/Nfkbia/Syk/H2-Ab1/Gadd45b/Myc/Bcl2/Psmd3/H2-Q7/Ikbkb/H2-Eb2/H2-M3/Jak1/Akt2/Usp7/Snw1/Gadd45g/Nfkb1/Psmd2/Ifnar1/H2-Q6/Stat1/Pik3r1/Ccnd3/Myd88/Blnk/Cd19/Psmd11/H2-Q4/Nfkb2/Relb/Chuk/Bcl2	37
**mmu04210**	mmu04210	Apoptosis	26/579	136/9010	4.09E-07	1.03E-05	6.86E-06	Jun/Fos/Nfkbia/Birc3/Tuba1a/Actg1/Pmaip1/Casp2/Tuba1b/Gadd45b/Map2k1/Bcl2/Parp1/Daxx/Traf1/Ikbkb/Kras/Birc2/Akt2/Gadd45g/Nfkb1/Eif2ak3/Pik3r1/Chuk/Bcl2l11/Lmnb1	26
**mmu04064**	mmu04064	NF-kappa B signaling pathway	21/579	105/9010	2.51E-06	5.8E-05	3.88E-05	Nfkbia/Birc3/Trim25/Syk/Malt1/Gadd45b/Bcl2/Parp1/Traf1/Prkcb/Ikbkb/Birc2/Csnk2a1/Tnfrsf13c/Gadd45g/Nfkb1/Myd88/Blnk/Nfkb2/Relb/Chuk	21
**mmu03040**	mmu03040	Spliceosome	24/579	134/9010	3.83E-06	8.23E-05	5.5E-05	Hspa8/Hspa1a/Hspa1b/Sf3b1/Srsf7/Pcbp1/Sart1/Snrpd3/Isy1/Lsm2/Acin1/Rbm17/Snrnp70/Snw1/Prpf38b/Magoh/Fus/Lsm8/Rbm22/Srsf10/U2surp/Prpf8/Srsf5/Cherp	24
**mmu05164**	mmu05164	Influenza A	28/579	173/9010	4.81E-06	9.64E-05	6.44E-05	Dnajb1/Nfkbia/Actg1/Trim25/H2-Ab1/Ifngr1/Crebbp/Map2k1/Rae1/Xpo1/Ep300/Prkcb/Ikbkb/H2-Eb2/Jak1/Akt2/Nfkb1/Ifnar1/Stat1/Pik3r1/Ccnd3/Myd88/Pabpn1/Pml/Chuk/Socs3/Dnajc3/Nxf1	28
**mmu04380**	mmu04380	Osteoclast differentiation	23/579	128/9010	5.75E-06	0.00010809	7.22E-05	Jun/Fos/Nfkbia/Jund/Junb/Syk/Ifngr1/Map2k1/Nfatc1/Ikbkb/Jak1/Akt2/Nfkb1/Ppp3ca/Ifnar1/Ncf4/Stat1/Pik3r1/Blnk/Nfkb2/Relb/Chuk/Socs3	23
**mmu04137**	mmu04137	Mitophagy - animal	15/579	68/9010	2.01E-05	0.000352334	0.000235341	Ubb/Jun/Uba52/Rps27a/Cited2/Ubc/Fis1/Kras/Csnk2a1/Gabarap/Tax1bp1/Eif2ak3/Usp15/Map1lc3a/Sp1	15
**mmu05014**	mmu05014	Amyotrophic lateral sclerosis	45/579	369/9010	2.11E-05	0.000352334	0.000235341	Tuba1a/Actg1/Tubb5/Gpx1/Srsf7/Atp5e/Anxa7/Ndufa3/Matr3/Psmb3/Tuba1b/Ndufc1/Atp5c1/Bcl2/Psmd3/Ndufv1/Nup50/Cox7b/Psmb6/Daxx/Chchd10/Rb1cc1/Pik3c3/Rae1/Atp5g1/Ndufs6/Nup88/Nup210/Pik3r4/Psmd2/F	45
**mmu04010**	mmu04010	MAPK signaling pathway	38/579	294/9010	2.59E-05	0.000409562	0.000273567	Hspa8/Hspa1a/Hspa1b/Jun/Nr4a1/Fos/Mef2c/Jund/Dusp1/Atf2/Nfatc3/Dusp5/Hspb1/Gadd45b/Map2k1/Myc/Arrb2/Daxx/Nfatc1/Prkcb/Ikbkb/Kras/Cdc25b/Rap1a/Akt2/Gadd45g/Nfkb1/Rasa2/Map4k2/Ppp3ca/Myd88/Map	38
**mmu05140**	mmu05140	Leishmaniasis	15/579	70/9010	2.91E-05	0.00043721	0.000292034	Jun/Fos/Nfkbia/Marcksl1/Itga4/H2-Ab1/Ifngr1/Cr1l/Prkcb/H2-Eb2/Jak1/Nfkb1/Ncf4/Stat1/Myd88	15
**mmu04659**	mmu04659	Th17 cell differentiation	19/579	105/9010	3.35E-05	0.000479601	0.000320349	Jun/Hsp90ab1/Hsp90aa1/Fos/Nfkbia/H2-Ab1/Nfatc3/Ifngr1/Irf4/Nfatc1/Ikbkb/H2-Eb2/Jak1/Nfkb1/Ppp3ca/Stat1/Il6st/Il4ra/Chuk	19
**mmu05145**	mmu05145	Toxoplasmosis	19/579	110/9010	6.51E-05	0.000890699	0.000594942	Hspa8/Hspa1a/Hspa1b/Nfkbia/Birc3/Il10ra/H2-Ab1/Ifngr1/Bcl2/Ikbkb/H2-Eb2/Birc2/Jak1/Akt2/Nfkb1/Lamb3/Stat1/Myd88/Chuk	19
**mmu03015**	mmu03015	mRNA surveillance pathway	18/579	103/9010	8.62E-05	0.00112781	0.000753319	Dazap1/Pnn/Sap18/Acin1/Ssu72/Pabpc4/Smg5/Smg1/Fip1l1/Casc3/Magoh/Srrm1/Ppp2r2a/Fus/Ppp2r5a/Ppp2ca/Pabpn1/Nxf1	18
**mmu05161**	mmu05161	Hepatitis B	24/579	163/9010	0.000108884	0.001365585	0.000912141	Jun/Fos/Nfkbia/Atf2/Nfatc3/Crebbp/Map2k1/Myc/Bcl2/Nfatc1/Ep300/Prkcb/Ikbkb/Kras/Jak1/Stat4/Akt2/Pcna/Nfkb1/Ifnar1/Stat1/Pik3r1/Myd88/Chuk	24
**mmu04658**	mmu04658	Th1 and Th2 cell differentiation	16/579	88/9010	0.000130247	0.00156818	0.001047464	Jun/Fos/Nfkbia/H2-Ab1/Nfatc3/Ifngr1/Nfatc1/Ikbkb/H2-Eb2/Jak1/Stat4/Nfkb1/Ppp3ca/Stat1/Il4ra/Chuk	16
**mmu05162**	mmu05162	Measles	22/579	146/9010	0.000148179	0.001715459	0.001145839	Hspa8/Hspa1a/Hspa1b/Jun/Fos/Nfkbia/Bcl2/Ikbkb/Cblb/Csnk2a1/Jak1/Akt2/Nfkb1/Eif2ak3/Ifnar1/Stat1/Pik3r1/Ccnd3/Myd88/Chuk/Msn/Ccnd2	22
**mmu05012**	mmu05012	Parkinson disease	33/579	264/9010	0.000167678	0.001808327	0.00120787	Ubb/Uba52/Tuba1a/Tubb5/Rps27a/Dusp1/Atp5e/Ndufa3/Psmb3/Tuba1b/Ndufc1/Atp5c1/Ubc/Psmd3/Ndufv1/Cox7b/Psmb6/Daxx/Ube2j2/Nfe2l2/Atp5g1/Camk2d/Ndufs6/Psmd2/Eif2ak3/Ndufs8/Cox7a2/Ndufb5/Ube2g1/Ps	33
**mmu05215**	mmu05215	Prostate cancer	17/579	99/9010	0.000168216	0.001808327	0.00120787	Hsp90ab1/Hsp90aa1/Nfkbia/Crebbp/Map2k1/Bcl2/Foxo1/Ep300/Ikbkb/Kras/Akt2/Nfkb1/Pik3r1/Ctnnb1/Pten/Chuk/Hsp90b1	17
**mmu05202**	mmu05202	Transcriptional misregulation in cancer	29/579	224/9010	0.000226171	0.002347502	0.001568012	Birc3/Mef2c/Rel/Klf3/Gadd45b/Myc/Ldb1/Jmjd1c/Foxo1/Traf1/Ccnt2/Birc2/Gadd45g/Cdk9/Taf15/Nfkb1/Aff1/Ccnt1/Cd86/Pax5/Fli1/Fus/Kdm6a/Sp1/Pml/Kmt2a/Elk4/Bcl6/Ccnd2	29
**mmu05222**	mmu05222	Small cell lung cancer	16/579	93/9010	0.000253942	0.002547887	0.001701859	Nfkbia/Birc3/Traf4/Gadd45b/Myc/Bcl2/Traf1/Ikbkb/Birc2/Akt2/Gadd45g/Nfkb1/Lamb3/Pik3r1/Pten/Chuk	16
**mmu04140**	mmu04140	Autophagy - animal	21/579	142/9010	0.000271591	0.002622654	0.001751799	Irs2/Atg12/Map2k1/Bcl2/Rb1cc1/Pik3c3/Kras/Gabarap/Akt2/Pik3r4/Atg16l2/Eif2ak3/Supt20/Pik3r1/Uvrag/Pten/Wipi2/Map1lc3a/Stk11/Rps6kb1/Ppp2ca	21
**mmu05417**	mmu05417	Lipid and atherosclerosis	28/579	216/9010	0.000282753	0.002622654	0.001751799	Hspa8/Hspa1a/Hspa1b/Jun/Hsp90ab1/Hsp90aa1/Fos/Nfkbia/Hspd1/Nfatc3/Bcl2/Nfe2l2/Nfatc1/Ikbkb/Kras/Rap1a/Camk2d/Akt2/Nfkb1/Ppp3ca/Eif2ak3/Ncf4/Pik3r1/Myd88/Atf6/Chuk/Hsp90b1/Hspa4	28
**mmu04218**	mmu04218	Cellular senescence	25/579	184/9010	0.000287534	0.002622654	0.001751799	Nfatc3/Lin54/Gadd45b/Map2k1/Myc/Hipk3/Sirt1/Ets1/Zfp36l2/Foxo1/H2-Q7/Nfatc1/Kras/H2-M3/Akt2/Gadd45g/Nfkb1/Ppp3ca/H2-Q6/Pik3r1/Ccnd3/Pten/H2-Q4/H2-T23/Ccnd2	25
**mmu05203**	mmu05203	Viral carcinogenesis	29/579	229/9010	0.000330323	0.00292433	0.001953303	Jun/Nfkbia/Syk/Atf2/Pmaip1/Rel/Crebbp/Sp100/H2-Q7/Traf1/Ep300/Kras/Gtf2a2/H2-M3/Jak1/Usp7/Snw1/Nfkb1/Rasa2/H2-Q6/Pik3r1/Ccnd3/Il6st/H2-Q4/Nfkb2/Chd4/H2-T23/Ubr4/Ccnd2	29
**mmu05010**	mmu05010	Alzheimer disease	42/579	383/9010	0.000427303	0.003557899	0.002376495	Gapdh/Tuba1a/Tubb5/Irs2/Atp5e/Ndufa3/Adam10/Psmb3/Tuba1b/Ndufc1/Map2k1/Atp5c1/Psmd3/Ndufv1/Cox7b/Psmb6/Rb1cc1/Pik3c3/Atp5g1/Ikbkb/Kras/Csnk2a1/Akt2/Ndufs6/Pik3r4/Nfkb1/Psmd2/Ppp3ca/Eif2ak3/Ndu	42
**mmu05210**	mmu05210	Colorectal cancer	15/579	88/9010	0.000435729	0.003557899	0.002376495	Jun/Fos/Pmaip1/Gadd45b/Map2k1/Myc/Bcl2/Rala/Kras/Akt2/Gadd45g/Pik3r1/Ctnnb1/Rps6kb1/Bcl2l11	15
**mmu05208**	mmu05208	Chemical carcinogenesis - reactive oxygen species	28/579	222/9010	0.000445044	0.003557899	0.002376495	Jun/Fos/Nfkbia/Atp5e/Ndufa3/Ndufc1/Map2k1/Atp5c1/Ndufv1/Arnt/Cox7b/Ptprj/Nfe2l2/Atp5g1/Ikbkb/Ephx1/Kras/Akt2/Ndufs6/Nfkb1/Ndufs8/Cox7a2/Pik3r1/Ndufb5/Pten/Chuk/Ptpn1/Atp5a1	28
**mmu03013**	mmu03013	Nucleocytoplasmic transport	18/579	117/9010	0.00044917	0.003557899	0.002376495	Pnn/Sap18/Acin1/Nup50/Rae1/Xpo1/Ipo7/Nup88/Nup210/Kpnb1/Xpo6/Casc3/Magoh/Srrm1/Ranbp2/Tnpo1/Nxf1/Cse1l	18
**mmu04612**	mmu04612	Antigen processing and presentation	15/579	90/9010	0.00055822	0.004308311	0.002877732	Hspa8/Hspa1a/Hspa1b/Hsp90ab1/Hsp90aa1/Ifi30/H2-Ab1/H2-Q7/H2-Eb2/H2-M3/H2-Q6/H2-Q4/H2-T23/Hspa4/Calr	15
**mmu05170**	mmu05170	Human immunodeficiency virus 1 infection	29/579	240/9010	0.000720076	0.005418576	0.003619332	Jun/Fos/Nfkbia/Cfl2/Nfatc3/Map2k1/Gng5/Bcl2/H2-Q7/Nfatc1/Prkcb/Ikbkb/Kras/H2-M3/Akt2/Cul1/Nfkb1/Ppp3ca/Gng2/H2-Q6/Pik3r1/Myd88/Cul5/Bst2/Rps6kb1/H2-Q4/Chuk/H2-T23/Calr	29
**mmu04625**	mmu04625	C-type lectin receptor signaling pathway	17/579	112/9010	0.000751188	0.005514822	0.003683619	Jun/Nfkbia/Syk/Nfatc3/Malt1/Nfatc1/Ikbkb/Cblb/Kras/Akt2/Nfkb1/Ppp3ca/Stat1/Pik3r1/Nfkb2/Relb/Chuk	17
**mmu04660**	mmu04660	T cell receptor signaling pathway	16/579	103/9010	0.000823728	0.005903381	0.003943157	Jun/Fos/Nfkbia/Nfatc3/Malt1/Map2k1/Nfatc1/Ikbkb/Cblb/Kras/Akt2/Nfkb1/Ppp3ca/Pik3r1/Chuk/Ptprc	16
**mmu05022**	mmu05022	Pathways of neurodegeneration - multiple diseases	48/579	471/9010	0.000893936	0.006257554	0.004179726	Ubb/Uba52/Tuba1a/Tubb5/Gpx1/Rps27a/Atp5e/Ndufa3/Psmb3/Tuba1b/Ndufc1/Map2k1/Atp5c1/Ubc/Bcl2/Psmd3/Ndufv1/Cox7b/Psmb6/Daxx/Ube2j2/Rb1cc1/Pik3c3/Atp5g1/Prkcb/Kras/Csnk2a1/Camk2d/Ndufs6/Pik3r4/N	48
**mmu05163**	mmu05163	Human cytomegalovirus infection	30/579	256/9010	0.00097263	0.006518388	0.00435395	Nfkbia/Akap13/Il10ra/Atf2/Nfatc3/Map2k1/Myc/Gng5/H2-Q7/Nfatc1/Prkcb/Ikbkb/Kras/H2-M3/Jak1/Akt2/Nfkb1/Ppp3ca/Rock1/Gng2/H2-Q6/Pik3r1/Ctnnb1/Rps6kb1/H2-Q4/Sp1/Gna13/Chuk/H2-T23/Calr	30
**mmu05135**	mmu05135	Yersinia infection	19/579	135/9010	0.00097451	0.006518388	0.00435395	Jun/Fos/Nfkbia/Actg1/Itga4/Nfatc3/Map2k1/Arpc5/Nfatc1/Ikbkb/Akt2/Elmo1/Nfkb1/Rock1/Rhog/Git2/Pik3r1/Myd88/Chuk	19
**mmu05212**	mmu05212	Pancreatic cancer	13/579	76/9010	0.000999028	0.006537119	0.004366461	Gadd45b/Map2k1/Rala/Ikbkb/Kras/Jak1/Akt2/Gadd45g/Nfkb1/Stat1/Pik3r1/Rps6kb1/Chuk	13
**mmu05152**	mmu05152	Tuberculosis	23/579	180/9010	0.001176755	0.007536238	0.005033822	Hspd1/Syk/Il10ra/H2-Ab1/Atp6v0a1/Ifngr1/Tcirg1/Malt1/Crebbp/Bcl2/Cr1l/Pik3c3/Ep300/H2-Eb2/Jak1/Camk2d/Akt2/Nfkb1/Cebpg/Ppp3ca/Stat1/Myd88/Hspa9	23
**mmu05134**	mmu05134	Legionellosis	11/579	61/9010	0.001543706	0.009680324	0.006465962	Hspa8/Hspa1a/Hspa1b/Nfkbia/Hspd1/Clk1/Cr1l/Nfkb1/Myd88/Nfkb2/Clk4	11
**mmu04211**	mmu04211	Longevity regulating pathway	14/579	90/9010	0.001687569	0.010366498	0.006924292	Irs2/Atf2/Sirt1/Foxo1/Rb1cc1/Kras/Akt2/Prkag1/Ehmt1/Nfkb1/Pik3r1/Eif4e2/Stk11/Rps6kb1	14
**mmu04213**	mmu04213	Longevity regulating pathway - multiple species	11/579	62/9010	0.001770431	0.010657996	0.007118997	Hspa8/Hspa1a/Hspa1b/Irs2/Sirt1/Foxo1/Kras/Akt2/Prkag1/Pik3r1/Rps6kb1	11
**mmu05020**	mmu05020	Prion disease	30/579	268/9010	0.002011235	0.011870231	0.007928708	Hspa8/Hspa1a/Hspa1b/Tuba1a/Tubb5/Atf2/Atp5e/Ndufa3/Psmb3/Egr1/Tuba1b/Ndufc1/Atp5c1/Psmd3/Ndufv1/Cox7b/Psmb6/Atp5g1/Csnk2a1/Ndufs6/Psmd2/Ppp3ca/Eif2ak3/Ncf4/Ndufs8/Cox7a2/Pik3r1/Ndufb5/Psmd11/	30
**mmu04668**	mmu04668	TNF signaling pathway	16/579	113/9010	0.002241218	0.012973205	0.008665438	Jun/Fos/Nfkbia/Birc3/Junb/Atf2/Map2k1/Itch/Traf1/Ikbkb/Birc2/Akt2/Nfkb1/Pik3r1/Chuk/Socs3	16
**mmu04932**	mmu04932	Non-alcoholic fatty liver disease	20/579	156/9010	0.002310771	0.013123436	0.008765785	Jun/Fos/Irs2/Ndufa3/Ndufc1/Ndufv1/Cox7b/Itch/Ikbkb/Akt2/Ndufs6/Prkag1/Nfkb1/Eif2ak3/Ndufs8/Cox7a2/Pik3r1/Ndufb5/Socs3/Bcl2l11	20
**mmu04066**	mmu04066	HIF-1 signaling pathway	16/579	114/9010	0.002456605	0.013693301	0.009146426	Gapdh/Ifngr1/Crebbp/Map2k1/Bcl2/Arnt/Ep300/Prkcb/Eno1/Camk2d/Akt2/Nfkb1/Pgk1/Pik3r1/Eif4e2/Rps6kb1	16
**mmu03018**	mmu03018	RNA degradation	13/579	84/9010	0.002555519	0.013985659	0.009341706	Hspd1/Dhx36/Lsm2/Pabpc4/Ddx6/Eno1/Exosc1/Cnot1/Pan3/Cnot2/Hspa9/Lsm8/Tob2	13
**mmu04928**	mmu04928	Parathyroid hormone synthesis, secretion and action	15/579	108/9010	0.003666265	0.019706174	0.013162718	Pde4b/Fos/Mef2c/Jund/Naca/Mef2d/Akap13/Atf2/Egr1/Map2k1/Bcl2/Arrb2/Prkcb/Sp1/Gna13	15
**mmu05211**	mmu05211	Renal cell carcinoma	11/579	68/9010	0.003767206	0.019893492	0.013287837	Jun/Crebbp/Map2k1/Arnt/Ets1/Ep300/Kras/Rap1a/Akt2/Pik3r1/Flcn	11
**mmu04915**	mmu04915	Estrogen signaling pathway	17/579	134/9010	0.005310577	0.027560062	0.018408714	Hspa8/Hspa1a/Hspa1b/Jun/Hsp90ab1/Hsp90aa1/Fos/Atf2/Fkbp4/Map2k1/Bcl2/Kras/Akt2/Ncoa2/Pik3r1/Sp1/Hsp90b1	17
**mmu04071**	mmu04071	Sphingolipid signaling pathway	16/579	124/9010	0.005718304	0.029173043	0.019486103	S1pr4/S1pr1/Map2k1/Bcl2/Prkcb/Kras/Akt2/Nfkb1/Rock1/Pik3r1/Ppp2r2a/Pten/Ppp2r5a/Ppp2ca/Cers4/Gna13	16
**mmu04666**	mmu04666	Fc gamma R-mediated phagocytosis	13/579	93/9010	0.006251879	0.031253987	0.020876067	Marcksl1/Cfl2/Syk/Inpp5d/Map2k1/Arpc5/Prkcb/Akt2/Bin1/Pik3r1/Rps6kb1/Dnm2/Ptprc	13
**mmu05016**	mmu05016	Huntington disease	31/579	302/9010	0.006333864	0.031253987	0.020876067	Tuba1a/Tubb5/Gpx1/Atp5e/Ndufa3/Psmb3/Crebbp/Tuba1b/Ndufc1/Atp5c1/Psmd3/Ndufv1/Cox7b/Psmb6/Rb1cc1/Pik3c3/Ep300/Atp5g1/Polr2a/Ndufs6/Pik3r4/Psmd2/Ndufs8/Cox7a2/Ndufb5/Cltc/Tfam/Wipi2/Psmd11/Sp1	31
**mmu04371**	mmu04371	Apelin signaling pathway	17/579	137/9010	0.006633196	0.032203097	0.021510025	Klf2/Mef2c/Mef2d/Egr1/Map2k1/Gng5/Pik3c3/Pde3b/Kras/Akt2/Prkag1/Pik3r4/Gng2/Tfam/Map1lc3a/Rps6kb1/Gna13	17
**mmu04151**	mmu04151	PI3K-Akt signaling pathway	35/579	359/9010	0.008573395	0.040961777	0.027360376	Hsp90ab1/Hsp90aa1/Nr4a1/Pik3ap1/Syk/Itga4/Atf2/Sgk3/Map2k1/Myc/Gng5/Bcl2/Ikbkb/Kras/Jak1/Akt2/Nfkb1/Gng2/Ifnar1/Lamb3/Pik3r1/Ccnd3/Ppp2r2a/Cd19/Il4ra/Eif4e2/Pten/Ppp2r5a/Stk11/Rps6kb1/Ppp2ca/Chuk/Bc	35
**mmu05220**	mmu05220	Chronic myeloid leukemia	11/579	76/9010	0.008856641	0.041653888	0.02782267	Nfkbia/Gadd45b/Map2k1/Myc/Ikbkb/Kras/Akt2/Gadd45g/Nfkb1/Pik3r1/Chuk	11
**mmu05160**	mmu05160	Hepatitis C	19/579	165/9010	0.009422157	0.043579452	0.029108849	Nfkbia/Map2k1/Myc/Eif3e/Ikbkb/Kras/Jak1/Akt2/Nfkb1/Eif2ak3/Ifnar1/Stat1/Pik3r1/Ppp2r2a/Ctnnb1/Ppp2ca/Cd81/Chuk/Socs3	19
**mmu05231**	mmu05231	Choline metabolism in cancer	13/579	98/9010	0.009663818	0.043579452	0.029108849	Jun/Fos/Dgkz/Gpcpd1/Map2k1/Prkcb/Kras/Akt2/Chkb/Pik3r1/Rps6kb1/Sp1/Dgka	13
**mmu05165**	mmu05165	Human papillomavirus infection	35/579	362/9010	0.00970041	0.043579452	0.029108849	Itga4/Atp6v0a1/Tcirg1/Crebbp/Hes6/Map2k1/Foxo1/H2-Q7/Ep300/Ikbkb/Kras/H2-M3/Jak1/Akt2/Nfkb1/Ifnar1/Lamb3/H2-Q6/Stat1/Pik3r1/Ccnd3/Ppp2r2a/Ctnnb1/Pten/Ppp2r5a/Rps6kb1/Csnk1a1/Ppp2ca/H2-Q4/Chd4/Chu	35
**mmu04931**	mmu04931	Insulin resistance	14/579	110/9010	0.010565983	0.046770014	0.031239982	Nfkbia/Irs2/Foxo1/Prkcb/Ikbkb/Akt2/Ogt/Prkag1/Nfkb1/Pik3r1/Pten/Rps6kb1/Socs3/Ptpn1	14
**mmu05213**	mmu05213	Endometrial cancer	9/579	58/9010	0.011055799	0.048228922	0.032214458	Gadd45b/Map2k1/Myc/Kras/Akt2/Gadd45g/Pik3r1/Ctnnb1/Pten	9
**mmu04620**	mmu04620	Toll-like receptor signaling pathway	13/579	100/9010	0.011378926	0.048929384	0.03268233	Jun/Fos/Nfkbia/Map2k1/Ikbkb/Akt2/Nfkb1/Ifnar1/Cd86/Stat1/Pik3r1/Myd88/Chuk	13
**mmu01521**	mmu01521	EGFR tyrosine kinase inhibitor resistance	11/579	79/9010	0.011754673	0.049368419	0.032975583	Map2k1/Bcl2/Prkcb/Kras/Jak1/Akt2/Pik3r1/Eif4e2/Pten/Rps6kb1/Bcl2l11	11
**mmu04150**	mmu04150	mTOR signaling pathway	18/579	157/9010	0.011809057	0.049368419	0.032975583	Map2k1/Lamtor2/Cab39/Prkcb/Ikbkb/Prr5/Kras/Akt2/Fnip1/Pik3r1/Lamtor3/Eif4e2/Pten/Stk11/Rps6kb1/Flcn/Chuk/Atp6v1b2	18
**mmu05418**	mmu05418	Fluid shear stress and atherosclerosis	17/579	147/9010	0.013070893	0.053895054	0.035999144	Klf2/Jun/Hsp90ab1/Hsp90aa1/Fos/Mef2c/Actg1/Dusp1/Bcl2/Nfe2l2/Ikbkb/Akt2/Nfkb1/Pik3r1/Ctnnb1/Chuk/Hsp90b1	17
**mmu05221**	mmu05221	Acute myeloid leukemia	10/579	70/9010	0.013417221	0.054243328	0.036231774	Map2k1/Myc/Ikbkb/Kras/Akt2/Nfkb1/Pik3r1/Rps6kb1/Pml/Chuk	10
**mmu04310**	mmu04310	Wnt signaling pathway	19/579	171/9010	0.013515779	0.054243328	0.036231774	Jun/Cacybp/Nfatc3/Chd8/Crebbp/Myc/Tbl1x/Nfatc1/Ep300/Prkcb/Csnk2a1/Camk2d/Cul1/Ppp3ca/Ccnd3/Ctnnb1/Csnk1a1/Ccdc88c/Ccnd2	19
**mmu05142**	mmu05142	Chagas disease	13/579	103/9010	0.014383215	0.056965101	0.038049779	Jun/Fos/Nfkbia/Ifngr1/Ikbkb/Akt2/Nfkb1/Pik3r1/Ppp2r2a/Myd88/Ppp2ca/Chuk/Calr	13
**mmu04136**	mmu04136	Autophagy - other	6/579	32/9010	0.014822868	0.057201068	0.038207393	Atg12/Pik3c3/Gabarap/Pik3r4/Wipi2/Ppp2ca	6
**mmu04215**	mmu04215	Apoptosis - multiple species	6/579	32/9010	0.014822868	0.057201068	0.038207393	Birc3/Pmaip1/Bcl2/Birc2/Birc6/Bcl2l11	6
**mmu03250**	mmu03250	Viral life cycle - HIV-1	9/579	61/9010	0.015191101	0.057880019	0.038660897	Aff4/Crebbp/Xpo1/Ep300/Ccnt2/Cdk9/Ccnt1/Bst2/Ranbp2	9
**mmu04657**	mmu04657	IL-17 signaling pathway	12/579	93/9010	0.015547727	0.058498324	0.039073893	Jun/Hsp90ab1/Hsp90aa1/Fos/Nfkbia/Jund/Traf4/Ikbkb/Nfkb1/Chuk/Hsp90b1/Il17ra	12
**mmu05225**	mmu05225	Hepatocellular carcinoma	19/579	174/9010	0.016032121	0.059576154	0.039793829	Actg1/Arid1b/Gadd45b/Map2k1/Myc/Nfe2l2/Smarce1/Prkcb/Kras/Smarcc1/Akt2/Gadd45g/Arid1a/Pik3r1/Ctnnb1/Pten/Rps6kb1/Csnk1a1/Smarca2	19
**mmu05415**	mmu05415	Diabetic cardiomyopathy	22/579	211/9010	0.016600712	0.06090362	0.040680509	Gapdh/Atp5e/Ndufa3/Ndufc1/Atp5c1/Ndufv1/Parp1/Cox7b/Atp5g1/Prkcb/Camk2d/Akt2/Ndufs6/Nfkb1/Ncf4/Ndufs8/Cox7a2/Pik3r1/Ndufb5/Pten/Sp1/Atp5a1	22
**mmu05321**	mmu05321	Inflammatory bowel disease	9/579	62/9010	0.016794021	0.06090362	0.040680509	Jun/H2-Ab1/Ifngr1/Nfatc1/H2-Eb2/Stat4/Nfkb1/Stat1/Il4ra	9
**mmu05207**	mmu05207	Chemical carcinogenesis - receptor activation	23/579	225/9010	0.018018604	0.064566663	0.043127234	Jun/Hsp90ab1/Hsp90aa1/Fos/Klf4/Ptges3/Atf2/Map2k1/Myc/Bcl2/Arnt/Arrb2/Prkcb/Ephx1/Kras/Akt2/Kpnb1/Nfkb1/Pik3r1/Ccnd3/Rps6kb1/Hsp90b1/Bcl6	23
**mmu05017**	mmu05017	Spinocerebellar ataxia	16/579	141/9010	0.018586953	0.065061033	0.043457448	Psmb3/Psmd3/Psmb6/Rb1cc1/Pik3c3/Prkcb/Akt2/Pik3r4/Psmd2/Pik3r1/Wipi2/Psmd11/Sp1/Nop56/Atxn2l/Pum2	16
**mmu04510**	mmu04510	Focal adhesion	21/579	201/9010	0.018588867	0.065061033	0.043457448	Jun/Birc3/Actg1/Itga4/Map2k1/Diaph1/Bcl2/Prkcb/Birc2/Rap1a/Akt2/Rock1/Lamb3/Pik3r1/Ccnd3/Ctnnb1/Pten/Zyx/Tln1/Parvg/Ccnd2	21
**mmu04710**	mmu04710	Circadian rhythm	6/579	34/9010	0.019742143	0.068303276	0.045623101	Csnk1d/Fbxl3/Prkag1/Cul1/Per1/Bhlhe40	6
**mmu00310**	mmu00310	Lysine degradation	9/579	64/9010	0.020365223	0.069658321	0.046528202	Kmt2e/Prdm2/Aldh2/Setd2/Kmt2c/Ehmt1/Hadha/Kmt2a/Nsd1	9
**mmu04919**	mmu04919	Thyroid hormone signaling pathway	14/579	120/9010	0.021461351	0.07258277	0.048481584	Actg1/Crebbp/Map2k1/Myc/Foxo1/Ep300/Prkcb/Kras/Med13/Akt2/Stat1/Ncoa2/Pik3r1/Ctnnb1	14
**mmu04630**	mmu04630	JAK-STAT signaling pathway	18/579	168/9010	0.022310042	0.074614695	0.049838807	Il10ra/Ifngr1/Crebbp/Myc/Bcl2/Ep300/Jak1/Stat4/Akt2/Ptpn2/Ifnar1/Stat1/Pik3r1/Ccnd3/Il6st/Il4ra/Socs3/Ccnd2	18
**mmu04722**	mmu04722	Neurotrophin signaling pathway	14/579	121/9010	0.022895717	0.075731988	0.050585101	Jun/Nfkbia/Map2k1/Bcl2/Ikbkb/Kras/Sh2b3/Rap1a/Camk2d/Akt2/Kidins220/Nfkb1/Pik3r1/Irak3	14
**mmu05416**	mmu05416	Viral myocarditis	11/579	88/9010	0.024829792	0.081236601	0.054261901	Actg1/H2-Ab1/Eif4g2/H2-Q7/H2-Eb2/H2-M3/Cd55/Cd86/H2-Q6/H2-Q4/H2-T23	11
**mmu00190**	mmu00190	Oxidative phosphorylation	15/579	135/9010	0.026313343	0.08516469	0.056885665	Atp6v0a1/Atp5e/Tcirg1/Ndufa3/Ndufc1/Atp5c1/Ndufv1/Cox7b/Atp5g1/Ndufs6/Ndufs8/Cox7a2/Ndufb5/Atp6v1b2/Atp5a1	15
**mmu04144**	mmu04144	Endocytosis	26/579	272/9010	0.0268954	0.086122503	0.057525435	Hspa8/Hspa1a/Hspa1b/Cyth1/Vps37b/Sh3kbp1/Snx12/Arpc5/Itch/Arrb2/Ist1/H2-Q7/Zfyve27/Cblb/H2-M3/Arfgef1/Bin1/Git2/H2-Q6/Cltc/Dnm2/H2-Q4/Pml/Arap2/H2-T23/Iqsec1	26
**mmu04933**	mmu04933	AGE-RAGE signaling pathway in diabetic complications	12/579	101/9010	0.02804349	0.088853586	0.059349658	Jun/Egr1/Diaph1/Bcl2/Foxo1/Nfatc1/Prkcb/Kras/Akt2/Nfkb1/Stat1/Pik3r1	12
**mmu05216**	mmu05216	Thyroid cancer	6/579	37/9010	0.029030666	0.091023233	0.060798872	Gadd45b/Map2k1/Myc/Kras/Gadd45g/Ctnnb1	6
**mmu04152**	mmu04152	AMPK signaling pathway	14/579	127/9010	0.033047997	0.102551	0.068498832	Irs2/Cab39/Sirt1/Foxo1/Scd1/Akt2/Prkag1/Pik3r1/Ppp2r2a/Tbc1d1/Ppp2r5a/Stk11/Rps6kb1/Ppp2ca	14
**mmu04922**	mmu04922	Glucagon signaling pathway	12/579	104/9010	0.034226382	0.104347811	0.06969901	Atf2/Crebbp/Sirt1/Foxo1/Pde3b/Ep300/Sik2/Camk2d/Akt2/Prkag1/Ppp3ca/Prmt1	12
**mmu04940**	mmu04940	Type I diabetes mellitus	9/579	70/9010	0.034320376	0.104347811	0.06969901	Hspd1/H2-Ab1/H2-Q7/H2-Eb2/H2-M3/Cd86/H2-Q6/H2-Q4/H2-T23	9
**mmu04935**	mmu04935	Growth hormone synthesis, secretion and action	13/579	117/9010	0.036957603	0.110747858	0.073973915	Fos/Junb/Irs2/Atf2/Crebbp/Map2k1/Ep300/Prkcb/Kras/Akt2/Stat1/Pik3r1/Socs3	13
**mmu04920**	mmu04920	Adipocytokine signaling pathway	9/579	71/9010	0.037161242	0.110747858	0.073973915	Nfkbia/Irs2/Ikbkb/Akt2/Prkag1/Nfkb1/Stk11/Chuk/Socs3	9
**mmu04062**	mmu04062	Chemokine signaling pathway	19/579	192/9010	0.039577911	0.116793641	0.078012189	Nfkbia/Map2k1/Gng5/Arrb2/Prkcb/Ikbkb/Kras/Rap1a/Akt2/Elmo1/Nfkb1/Rock1/Gng2/Stat1/Pik3r1/Cxcr5/Ccr7/Dock2/Chuk	19
**mmu04115**	mmu04115	p53 signaling pathway	9/579	72/9010	0.040158699	0.117356975	0.078388467	Shisa5/Pmaip1/Gadd45b/Bcl2/Gadd45g/Ccnd3/Sfn/Pten/Ccnd2	9
**mmu04714**	mmu04714	Thermogenesis	22/579	231/9010	0.040910117	0.118403318	0.079087371	Actg1/Atf2/Atp5e/Ndufa3/Arid1b/Ndufc1/Atp5c1/Ndufv1/Cox7b/Smarce1/Atp5g1/Kras/Smarcc1/Ndufs6/Prkag1/Arid1a/Ndufs8/Cox7a2/Ndufb5/Rps6kb1/Smarca2/Atp5a1	22
**mmu04012**	mmu04012	ErbB signaling pathway	10/579	84/9010	0.042171575	0.12089185	0.080749583	Jun/Map2k1/Myc/Prkcb/Cblb/Kras/Camk2d/Akt2/Pik3r1/Rps6kb1	10
**mmu04145**	mmu04145	Phagosome	18/579	182/9010	0.044518821	0.126416653	0.08443987	Tuba1a/Actg1/Tubb5/H2-Ab1/Atp6v0a1/Tcirg1/Tuba1b/H2-Q7/Pik3c3/Vamp3/H2-Eb2/H2-M3/Ncf4/H2-Q6/H2-Q4/Atp6v1b2/H2-T23/Calr	18
**mmu04917**	mmu04917	Prolactin signaling pathway	9/579	74/9010	0.046635301	0.129906773	0.086771092	Fos/Map2k1/Kras/Akt2/Nfkb1/Stat1/Pik3r1/Socs3/Ccnd2	9
**mmu05214**	mmu05214	Glioma	9/579	74/9010	0.046635301	0.129906773	0.086771092	Gadd45b/Map2k1/Prkcb/Kras/Camk2d/Akt2/Gadd45g/Pik3r1/Pten	9
**mmu05330**	mmu05330	Allograft rejection	8/579	63/9010	0.047474236	0.129906773	0.086771092	H2-Ab1/H2-Q7/H2-Eb2/H2-M3/Cd86/H2-Q6/H2-Q4/H2-T23	8
**mmu05332**	mmu05332	Graft-versus-host disease	8/579	63/9010	0.047474236	0.129906773	0.086771092	H2-Ab1/H2-Q7/H2-Eb2/H2-M3/Cd86/H2-Q6/H2-Q4/H2-T23	8
**mmu05224**	mmu05224	Breast cancer	15/579	147/9010	0.050244171	0.136247708	0.091006513	Jun/Fos/Gadd45b/Map2k1/Myc/Kras/Akt2/Gadd45g/Pik3r1/Ctnnb1/Pten/Rps6kb1/Csnk1a1/Sp1/Nfkb2	15
**mmu05100**	mmu05100	Bacterial invasion of epithelial cells	9/579	76/9010	0.053771469	0.144510824	0.096525852	Actg1/Arpc5/Elmo1/Cd2ap/Rhog/Pik3r1/Cltc/Ctnnb1/Dnm2	9
**mmu04110**	mmu04110	Cell cycle	13/579	125/9010	0.05770117	0.153699577	0.10266347	Orc3/Crebbp/Gadd45b/Myc/Ep300/Cdc25b/Pcna/Cul1/Gadd45g/Ccnd3/Sfn/Rad21/Ccnd2	13
**mmu04910**	mmu04910	Insulin signaling pathway	14/579	139/9010	0.062458555	0.164912501	0.110153131	Irs2/Map2k1/Foxo1/Pde3b/Ikbkb/Cblb/Kras/Akt2/Prkag1/Pik3r1/Eif4e2/Rps6kb1/Socs3/Ptpn1	14
**mmu04720**	mmu04720	Long-term potentiation	8/579	67/9010	0.064161134	0.167934794	0.112171868	Crebbp/Map2k1/Ep300/Prkcb/Kras/Rap1a/Camk2d/Ppp3ca	8
**mmu05230**	mmu05230	Central carbon metabolism in cancer	8/579	69/9010	0.07371579	0.191279765	0.127765117	Map2k1/Myc/Kras/Akt2/Pik3r1/Pten/Gls/Slc1a5	8
**mmu04340**	mmu04340	Hedgehog signaling pathway	7/579	58/9010	0.076783486	0.19586296	0.130826457	Bcl2/Csnk1d/Arrb2/Csnk1g3/Cul1/Csnk1a1/Ccnd2	7
**mmu04370**	mmu04370	VEGF signaling pathway	7/579	58/9010	0.076783486	0.19586296	0.130826457	Hspb1/Map2k1/Prkcb/Kras/Akt2/Ppp3ca/Pik3r1	7
**mmu05340**	mmu05340	Primary immunodeficiency	5/579	36/9010	0.078243931	0.19791112	0.132194523	Dclre1c/Tnfrsf13c/Blnk/Cd19/Ptprc	5
**mmu04350**	mmu04350	TGF-beta signaling pathway	10/579	95/9010	0.083257071	0.208836485	0.13949211	Smad7/Id3/Crebbp/Myc/Ep300/Cul1/Rock1/Rps6kb1/Ppp2ca/Sp1	10
**mmu04520**	mmu04520	Adherens junction	8/579	71/9010	0.084086412	0.209173637	0.139717309	Actg1/Crebbp/Ptprj/Ep300/Csnk2a1/Iqgap1/Ctnnb1/Ptpn1	8
**mmu03008**	mmu03008	Ribosome biogenesis in eukaryotes	12/579	121/9010	0.087905977	0.21688278	0.144866623	Fcf1/Bms1/Pop7/Xpo1/Csnk2a1/Wdr43/Nhp2/Riok1/Mphosph10/Nop56/Nxf1/Gnl3	12
**mmu05223**	mmu05223	Non-small cell lung cancer	8/579	72/9010	0.089577376	0.219209677	0.146420872	Gadd45b/Map2k1/Prkcb/Kras/Akt2/Gadd45g/Pik3r1/Eml4	8
**mmu04621**	mmu04621	NOD-like receptor signaling pathway	19/579	213/9010	0.091028422	0.220964154	0.147592773	Jun/Hsp90ab1/Hsp90aa1/Nfkbia/Birc3/Atg12/Pstpip1/Bcl2/Card6/Ikbkb/Birc2/Gabarap/Jak1/Nfkb1/Ifnar1/Stat1/Myd88/Map1lc3a/Chuk	19
**mmu00564**	mmu00564	Glycerophospholipid metabolism	10/579	98/9010	0.097637982	0.235112261	0.157042986	Cept1/Dgkz/Gpcpd1/Pnpla7/Mboat7/Chkb/Etnk1/Lpgat1/Dgka/Pld4	10
**mmu04217**	mmu04217	Necroptosis	16/579	176/9010	0.100638255	0.240413609	0.160584016	Hsp90ab1/Hsp90aa1/Fth1/Ftl1/Birc3/Ifngr1/Bcl2/Parp1/Birc2/Jak1/Stat4/Camk2d/Ifnar1/Stat1/Faf1/Glud1	16
**mmu04725**	mmu04725	Cholinergic synapse	11/579	112/9010	0.104560788	0.247817301	0.165529304	Fos/Kcnq5/Map2k1/Gng5/Bcl2/Prkcb/Kras/Camk2d/Akt2/Gng2/Pik3r1	11
**mmu04530**	mmu04530	Tight junction	15/579	167/9010	0.117735698	0.276862852	0.184930249	Jun/Tuba1a/Actg1/Tuba1b/Arpc5/Rap1a/Prkag1/Pcna/Rock1/Ppp2r2a/Stk11/Ppp2ca/Msn/Rapgef6/Hspa4	15
**mmu04936**	mmu04936	Alcoholic liver disease	13/579	141/9010	0.119666728	0.279222366	0.186506284	Nfkbia/Aldh2/Sirt1/Foxo1/Scd1/Ikbkb/Akt2/Prkag1/Nfkb1/Myd88/Ctnnb1/Chuk/Il17ra	13
**mmu04926**	mmu04926	Relaxin signaling pathway	12/579	129/9010	0.125127486	0.289718255	0.193517002	Jun/Fos/Nfkbia/Atf2/Map2k1/Gng5/Arrb2/Kras/Akt2/Nfkb1/Gng2/Pik3r1	12
**mmu01230**	mmu01230	Biosynthesis of amino acids	8/579	79/9010	0.1335942	0.302535829	0.202078487	Gapdh/Got1/Eno1/Pgk1/Prps1/Taldo1/Aco2/Mat2b	8
**mmu05320**	mmu05320	Autoimmune thyroid disease	8/579	79/9010	0.1335942	0.302535829	0.202078487	H2-Ab1/H2-Q7/H2-Eb2/H2-M3/Cd86/H2-Q6/H2-Q4/H2-T23	8
**mmu00220**	mmu00220	Arginine biosynthesis	3/579	20/9010	0.133678622	0.302535829	0.202078487	Got1/Gls/Glud1	3
**mmu04672**	mmu04672	Intestinal immune network for IgA production	5/579	43/9010	0.139733859	0.313879789	0.209655673	Itga4/H2-Ab1/H2-Eb2/Tnfrsf13c/Cd86	5
**mmu01522**	mmu01522	Endocrine resistance	9/579	93/9010	0.142240179	0.317142918	0.211835276	Jun/Fos/Map2k1/Bcl2/Kras/Akt2/Pik3r1/Rps6kb1/Sp1	9
**mmu04640**	mmu04640	Hematopoietic cell lineage	9/579	94/9010	0.148960575	0.329684802	0.220212615	Ms4a1/Itga4/H2-Ab1/Cd2/Cr1l/H2-Eb2/Cd55/Cd19/Il4ra	9

**mmu05031**	mmu05031	Amphetamine addiction	7/579	69/9010	0.15317313	0.335983842	0.224420052	Jun/Fos/Atf2/Sirt1/Prkcb/Camk2d/Ppp3ca	7
**mmu01200**	mmu01200	Carbon metabolism	11/579	121/9010	0.154039104	0.335983842	0.224420052	Gapdh/Acss1/Esd/Got1/Eno1/Pgk1/Ogdh/Prps1/Taldo1/Aco2/Glud1	11
**mmu04142**	mmu04142	Lysosome	12/579	135/9010	0.158041396	0.339789001	0.226961704	Laptm5/Tpp1/Atp6v0a1/Tcirg1/Man2b1/Ctsa/Dmxl1/Cltc/Psap/Gns/Hexa/Dnase2a	12
**mmu04728**	mmu04728	Dopaminergic synapse	12/579	135/9010	0.158041396	0.339789001	0.226961704	Fos/Atf2/Gng5/Arrb2/Prkcb/Camk2d/Akt2/Ppp3ca/Gng2/Ppp2r2a/Ppp2r5a/Ppp2ca	12
**mmu04622**	mmu04622	RIG-I-like receptor signaling pathway	7/579	70/9010	0.161436915	0.34462774	0.230193734	Nfkbia/Trim25/Rnf125/Atg12/Ikbkb/Nfkb1/Chuk	7
**mmu05168**	mmu05168	Herpes simplex virus 1 infection	35/579	459/9010	0.163626393	0.346701158	0.231578671	Nfkbia/Birc3/Syk/H2-Ab1/Srsf7/Ifngr1/Zfp868/Sp100/Bcl2/Daxx/H2-Q7/Ikbkb/H2-Eb2/Birc2/Eif2b2/H2-M3/Jak1/Akt2/Nfkb1/Eif2ak3/Zfp87/Ifnar1/H2-Q6/Stat1/Pik3r1/Myd88/Bst2/H2-Q4/Pml/Chuk/Socs3/Nxf1/H2-T23/Calr	35
**mmu03430**	mmu03430	Mismatch repair	3/579	22/9010	0.164711846	0.346701158	0.231578671	Pold4/Pcna/Rfc1	3
**mmu05218**	mmu05218	Melanoma	7/579	72/9010	0.178546945	0.373212712	0.249287036	Gadd45b/Map2k1/Kras/Akt2/Gadd45g/Pik3r1/Pten	7
**mmu03050**	mmu03050	Proteasome	5/579	47/9010	0.182034828	0.377879194	0.252404008	Psmb3/Psmd3/Psmb6/Psmd2/Psmd11	5
**mmu04921**	mmu04921	Oxytocin signaling pathway	13/579	153/9010	0.184337114	0.380037474	0.253845629	Jun/Fos/Mef2c/Actg1/Nfatc3/Map2k1/Nfatc1/Prkcb/Kras/Camk2d/Prkag1/Ppp3ca/Rock1	13
**mmu04540**	mmu04540	Gap junction	8/579	86/9010	0.186660216	0.382209013	0.255296106	Tuba1a/Tubb5/Tuba1b/Map2k1/Csnk1d/Prkcb/Kras/Map3k2	8
**mmu05323**	mmu05323	Rheumatoid arthritis	8/579	87/9010	0.194895118	0.39637453	0.264757948	Jun/Fos/H2-Ab1/Atp6v0a1/Tcirg1/H2-Eb2/Cd86/Atp6v1b2	8
**mmu03410**	mmu03410	Base excision repair	4/579	36/9010	0.198254759	0.400501225	0.26751437	Neil1/Pold4/Parp1/Pcna	4
**mmu04929**	mmu04929	GnRH secretion	6/579	63/9010	0.216780009	0.435005218	0.290561275	Map2k1/Arrb2/Prkcb/Kras/Akt2/Pik3r1	6
**mmu04721**	mmu04721	Synaptic vesicle cycle	7/579	77/9010	0.224380449	0.442511763	0.295575264	Nsf/Atp6v0a1/Tcirg1/Vamp2/Cltc/Dnm2/Atp6v1b2	7
**mmu04960**	mmu04960	Aldosterone-regulated sodium reabsorption	4/579	38/9010	0.225736026	0.442511763	0.295575264	Prkcb/Kras/Pik3r1/Sfn	4
**mmu00533**	mmu00533	Glycosaminoglycan biosynthesis - keratan sulfate	2/579	14/9010	0.225957337	0.442511763	0.295575264	B3gnt7/B3gnt2	2
**mmu04670**	mmu04670	Leukocyte transendothelial migration	10/579	118/9010	0.226401367	0.442511763	0.295575264	Actg1/Itga4/Prkcb/Rap1a/Rock1/Ncf4/Pik3r1/Ctnnb1/Rhoh/Msn	10
**mmu04914**	mmu04914	Progesterone-mediated oocyte maturation	8/579	92/9010	0.238161137	0.462493562	0.308922086	Hsp90ab1/Hsp90aa1/Map2k1/Pde3b/Kras/Cdc25b/Akt2/Pik3r1	8
**mmu04664**	mmu04664	Fc epsilon RI signaling pathway	6/579	66/9010	0.248833206	0.47507967	0.317328963	Syk/Inpp5d/Map2k1/Kras/Akt2/Pik3r1	6
**mmu04966**	mmu04966	Collecting duct acid secretion	3/579	27/9010	0.249438903	0.47507967	0.317328963	Atp6v0a1/Tcirg1/Atp6v1b2	3
**mmu04650**	mmu04650	Natural killer cell mediated cytotoxicity	10/579	121/9010	0.249880254	0.47507967	0.317328963	Syk/Ifngr1/Map2k1/Nfatc1/Prkcb/Kras/Ppp3ca/Ifnar1/Pik3r1/H2-T23	10
**mmu04014**	mmu04014	Ras signaling pathway	18/579	235/9010	0.251993311	0.47507967	0.317328963	Ralgapa1/Rel/Rasal3/Map2k1/Gng5/Ets1/Brap/Prkcb/Rala/Ikbkb/Kras/Rap1a/Akt2/Nfkb1/Rasa2/Gng2/Pik3r1/Chuk	18
**mmu01524**	mmu01524	Platinum drug resistance	7/579	80/9010	0.253659344	0.47507967	0.317328963	Birc3/Top2b/Pmaip1/Bcl2/Birc2/Akt2/Pik3r1	7
**mmu04216**	mmu04216	Ferroptosis	4/579	40/9010	0.254112382	0.47507967	0.317328963	Fth1/Ftl1/Pcbp1/Map1lc3a	4
**mmu00010**	mmu00010	Glycolysis / Gluconeogenesis	6/579	67/9010	0.259807586	0.48272891	0.322438265	Gapdh/Acss1/Aldh2/Minpp1/Eno1/Pgk1	6
**mmu00600**	mmu00600	Sphingolipid metabolism	5/579	54/9010	0.26514341	0.489620653	0.327041597	Ugcg/Cerk/Psap/Cers4/Hexa	5
**mmu01523**	mmu01523	Antifolate resistance	3/579	29/9010	0.285024681	0.519954114	0.347302801	Ikbkb/Nfkb1/Chuk	3
**mmu03060**	mmu03060	Protein export	3/579	29/9010	0.285024681	0.519954114	0.347302801	Sec11c/Srpr/Sec63	3
**mmu04550**	mmu04550	Signaling pathways regulating pluripotency of stem cells	11/579	140/9010	0.288535157	0.523187243	0.349462366	Klf4/Id3/Map2k1/Myc/Kras/Jak1/Akt2/Pik3r1/Il6st/Ctnnb1/Kat6a	11
**mmu03420**	mmu03420	Nucleotide excision repair	4/579	43/9010	0.297873092	0.536885033	0.35861179	Pold4/Pcna/Rfc1/Gtf2h5	4
**mmu04390**	mmu04390	Hippo signaling pathway	12/579	157/9010	0.308816025	0.553295378	0.369573063	Birc3/Actg1/Smad7/Limd1/Myc/Csnk1d/Birc2/Ccnd3/Ppp2r2a/Ctnnb1/Ppp2ca/Ccnd2	12
**mmu04022**	mmu04022	cGMP-PKG signaling pathway	13/579	173/9010	0.319819414	0.569619192	0.380476537	Mef2c/Mef2d/Atp2b1/Irs2/Atf2/Nfatc3/Map2k1/Pde3b/Nfatc1/Akt2/Ppp3ca/Rock1/Gna13	13
**mmu00511**	mmu00511	Other glycan degradation	2/579	18/9010	0.323517761	0.572816742	0.382612337	Man2b1/Hexa	2
**mmu04024**	mmu04024	cAMP signaling pathway	16/579	220/9010	0.340184707	0.598210839	0.399574297	Jun/Pde4b/Fos/Nfkbia/Atp2b1/Crebbp/Map2k1/Pde3b/Nfatc1/Ep300/Rap1a/Camk2d/Akt2/Nfkb1/Rock1/Pik3r1	16
**mmu04730**	mmu04730	Long-term depression	5/579	60/9010	0.341834765	0.598210839	0.399574297	Map2k1/Prkcb/Kras/Ppp2ca/Gna13	5
**mmu04727**	mmu04727	GABAergic synapse	7/579	89/9010	0.346855569	0.603488592	0.403099567	Nsf/Gng5/Prkcb/Gabarap/Gng2/Gls/Slc38a2	7
**mmu04130**	mmu04130	SNARE interactions in vesicular transport	3/579	33/9010	0.356973539	0.61492642	0.410739452	Vamp3/Vamp2/Vti1b	3
**mmu04912**	mmu04912	GnRH signaling pathway	7/579	90/9010	0.357515361	0.61492642	0.410739452	Jun/Egr1/Map2k1/Prkcb/Kras/Camk2d/Map3k2	7
**mmu04072**	mmu04072	Phospholipase D signaling pathway	11/579	149/9010	0.361602397	0.618422281	0.413074508	Cyth1/Syk/Dgkz/Map2k1/Rala/Kras/Akt2/Pik3r1/Dnm2/Gna13/Dgka	11
**mmu05032**	mmu05032	Morphine addiction	7/579	91/9010	0.368204174	0.619094846	0.413523747	Pde4b/Gng5/Pde7a/Arrb2/Pde3b/Prkcb/Gng2	7
**mmu05226**	mmu05226	Gastric cancer	11/579	150/9010	0.369907962	0.619094846	0.413523747	Gadd45b/Map2k1/Myc/Bcl2/Kras/Akt2/Gadd45g/Pik3r1/Ctnnb1/Rps6kb1/Csnk1a1	11
**mmu00532**	mmu00532	Glycosaminoglycan biosynthesis - chondroitin sulfate / derm	2/579	20/9010	0.371344006	0.619094846	0.413523747	Chst12/Xylt1	2
**mmu01210**	mmu01210	2-Oxocarboxylic acid metabolism	2/579	20/9010	0.371344006	0.619094846	0.413523747	Got1/Aco2	2
**mmu04930**	mmu04930	Type II diabetes mellitus	4/579	48/9010	0.372279625	0.619094846	0.413523747	Irs2/Ikbkb/Pik3r1/Socs3	4
**mmu04623**	mmu04623	Cytosolic DNA-sensing pathway	5/579	63/9010	0.380874789	0.629908306	0.420746586	Nfkbia/Ikbkb/Nfkb1/Polr1d/Chuk	5
**mmu05206**	mmu05206	MicroRNAs in cancer	21/579	303/9010	0.391509732	0.638825145	0.42670258	Foxp1/Irs2/Zeb2/Crebbp/Map2k1/Myc/Bcl2/Sirt1/Ep300/Prkcb/Ikbkb/Kras/Cdc25b/Nfkb1/Rock1/Pik3r1/Pten/Gls/Bcl2l11/Vim/Ccnd2	21
**mmu03030**	mmu03030	DNA replication	3/579	35/9010	0.392693599	0.638825145	0.42670258	Pold4/Pcna/Rfc1	3
**mmu00531**	mmu00531	Glycosaminoglycan degradation	2/579	21/9010	0.394755737	0.638825145	0.42670258	Gns/Hexa	2
**mmu00770**	mmu00770	Pantothenate and CoA biosynthesis	2/579	21/9010	0.394755737	0.638825145	0.42670258	Aldh2/Aasdhppt	2
**mmu00510**	mmu00510	N-Glycan biosynthesis	4/579	50/9010	0.402012974	0.643648432	0.429924289	Ganab/Stt3b/St6gal1/Dpm1	4
**mmu04979**	mmu04979	Cholesterol metabolism	4/579	50/9010	0.402012974	0.643648432	0.429924289	Vapa/Mylip/Tspo/Apoe	4
**mmu04964**	mmu04964	Proximal tubule bicarbonate reclamation	2/579	22/9010	0.417760126	0.665321683	0.444400914	Gls/Glud1	2
**mmu04070**	mmu04070	Phosphatidylinositol signaling system	7/579	96/9010	0.421794447	0.668211202	0.446330966	Inpp5d/Dgkz/Pik3c3/Prkcb/Pik3r1/Pten/Dgka	7
**mmu04713**	mmu04713	Circadian entrainment	7/579	98/9010	0.443151463	0.698369583	0.466475224	Fos/Rasd1/Gng5/Prkcb/Camk2d/Gng2/Per1	7
**mmu05205**	mmu05205	Proteoglycans in cancer	14/579	205/9010	0.446787904	0.700433121	0.467853562	Actg1/Plaur/Map2k1/Myc/Prkcb/Kras/Iqgap1/Camk2d/Akt2/Rock1/Pik3r1/Ctnnb1/Rps6kb1/Msn	14
**mmu00270**	mmu00270	Cysteine and methionine metabolism	4/579	54/9010	0.460550302	0.713146163	0.476345225	Ahcy/Got1/Mri1/Mat2b	4
**mmu00250**	mmu00250	Alanine, aspartate and glutamate metabolism	3/579	39/9010	0.462327062	0.713146163	0.476345225	Got1/Gls/Glud1	3
**mmu00534**	mmu00534	Glycosaminoglycan biosynthesis - heparan sulfate / heparin	2/579	24/9010	0.462376874	0.713146163	0.476345225	Hs3st1/Xylt1	2
**mmu04916**	mmu04916	Melanogenesis	7/579	100/9010	0.464374245	0.713146163	0.476345225	Crebbp/Map2k1/Ep300/Prkcb/Kras/Camk2d/Ctnnb1	7
**mmu05310**	mmu05310	Asthma	2/579	25/9010	0.48392187	0.739393314	0.493876982	H2-Ab1/H2-Eb2	2
**mmu00760**	mmu00760	Nicotinate and nicotinamide metabolism	3/579	41/9010	0.495839009	0.742943056	0.496248028	Sirt1/Nadk/Nt5c	3
**mmu03440**	mmu03440	Homologous recombination	3/579	41/9010	0.495839009	0.742943056	0.496248028	Pold4/Topbp1/Top3b	3
**mmu05219**	mmu05219	Bladder cancer	3/579	41/9010	0.495839009	0.742943056	0.496248028	Map2k1/Myc/Kras	3
**mmu00562**	mmu00562	Inositol phosphate metabolism	5/579	72/9010	0.496118121	0.742943056	0.496248028	Inpp5d/Minpp1/Pik3c3/Inpp5k/Pten	5
**mmu04923**	mmu04923	Regulation of lipolysis in adipocytes	4/579	57/9010	0.503123588	0.747595834	0.499355847	Irs2/Pde3b/Akt2/Pik3r1	4
**mmu00601**	mmu00601	Glycosphingolipid biosynthesis - lacto and neolacto series	2/579	26/9010	0.504923345	0.747595834	0.499355847	B3gnt5/B3gnt2	2
**mmu04514**	mmu04514	Cell adhesion molecules	12/579	182/9010	0.506676246	0.747595834	0.499355847	Itga4/H2-Ab1/Cd2/H2-Q7/H2-Eb2/H2-M3/Cd86/H2-Q6/H2-Q4/Glg1/Ptprc/H2-T23	12
**mmu00130**	mmu00130	Ubiquinone and other terpenoid-quinone biosynthesis	1/579	11/9010	0.518590039	0.76144196	0.508604352	Coq2	1
**mmu04810**	mmu04810	Regulation of actin cytoskeleton	15/579	231/9010	0.522334855	0.763217434	0.509790278	Cfl2/Actg1/Ssh2/Itga4/Map2k1/Diaph1/Arpc5/Kras/Iqgap1/Akt2/Rock1/Pik3r1/Gna13/Msn/Cyfip2	15
**mmu03022**	mmu03022	Basal transcription factors	3/579	44/9010	0.544048792	0.787301377	0.525877122	Gtf2a2/Taf15/Gtf2h5	3
**mmu04962**	mmu04962	Vasopressin-regulated water reabsorption	3/579	44/9010	0.544048792	0.787301377	0.525877122	Nsf/Dynll1/Vamp2	3
**mmu05133**	mmu05133	Pertussis	5/579	77/9010	0.556660463	0.795214616	0.531162762	Jun/Fos/Cfl2/Nfkb1/Myd88	5
**mmu04611**	mmu04611	Platelet activation	8/579	125/9010	0.557361978	0.795214616	0.531162762	Actg1/Syk/Rap1a/Akt2/Rock1/Pik3r1/Gna13/Tln1	8
**mmu04961**	mmu04961	Endocrine and other factor-regulated calcium reabsorption	4/579	61/9010	0.557442804	0.795214616	0.531162762	Atp2b1/Prkcb/Cltc/Dnm2	4
**mmu03450**	mmu03450	Non-homologous end-joining	1/579	13/9010	0.578548666	0.821429946	0.548673263	Dclre1c	1
**mmu00561**	mmu00561	Glycerolipid metabolism	4/579	63/9010	0.583375246	0.824394127	0.550653185	Dgkz/Aldh2/Abhd16a/Dgka	4
**mmu04934**	mmu04934	Cushing syndrome	10/579	162/9010	0.599378154	0.833899377	0.557002207	Nr4a1/Atf2/Rasd1/Map2k1/Arnt/Rap1a/Camk2d/Ctnnb1/Sp1/Kmt2a	10
**mmu00640**	mmu00640	Propanoate metabolism	2/579	31/9010	0.601259802	0.833899377	0.557002207	Acss1/Hadha	2
**mmu00565**	mmu00565	Ether lipid metabolism	3/579	48/9010	0.603953701	0.833899377	0.557002207	Cept1/Agps/Pld4	3
**mmu04973**	mmu04973	Carbohydrate digestion and absorption	3/579	48/9010	0.603953701	0.833899377	0.557002207	Prkcb/Akt2/Pik3r1	3
**mmu05030**	mmu05030	Cocaine addiction	3/579	48/9010	0.603953701	0.833899377	0.557002207	Jun/Atf2/Nfkb1	3
**mmu04723**	mmu04723	Retrograde endocannabinoid signaling	9/579	148/9010	0.617225369	0.835165875	0.557848163	Ndufa3/Ndufc1/Gng5/Ndufv1/Prkcb/Ndufs6/Gng2/Ndufs8/Ndufb5	9
**mmu00020**	mmu00020	Citrate cycle (TCA cycle)	2/579	32/9010	0.618744153	0.835165875	0.557848163	Ogdh/Aco2	2
**mmu00410**	mmu00410	beta-Alanine metabolism	2/579	32/9010	0.618744153	0.835165875	0.557848163	Aldh2/Hadha	2
**mmu00630**	mmu00630	Glyoxylate and dicarboxylate metabolism	2/579	32/9010	0.618744153	0.835165875	0.557848163	Acss1/Aco2	2
**mmu03020**	mmu03020	RNA polymerase	2/579	32/9010	0.618744153	0.835165875	0.557848163	Polr2a/Polr1d	2
**mmu00604**	mmu00604	Glycosphingolipid biosynthesis - ganglio series	1/579	15/9010	0.63105085	0.844205804	0.563886374	Hexa	1
**mmu00730**	mmu00730	Thiamine metabolism	1/579	15/9010	0.63105085	0.844205804	0.563886374	Ak2	1
**mmu00030**	mmu00030	Pentose phosphate pathway	2/579	33/9010	0.63563258	0.846572595	0.565467269	Prps1/Taldo1	2
**mmu04261**	mmu04261	Adrenergic signaling in cardiomyocytes	9/579	152/9010	0.649142167	0.860756794	0.57494159	Atp2b1/Crem/Atf2/Bcl2/Camk2d/Akt2/Ppp2r2a/Ppp2r5a/Ppp2ca	9
**mmu00603**	mmu00603	Glycosphingolipid biosynthesis - globo and isoglobo series	1/579	16/9010	0.654799773	0.864450577	0.577408849	Hexa	1
**mmu00380**	mmu00380	Tryptophan metabolism	3/579	52/9010	0.658419883	0.865433995	0.578065721	Aldh2/Kynu/Hadha	3
**mmu00910**	mmu00910	Nitrogen metabolism	1/579	17/9010	0.677022469	0.886016362	0.591813692	Glud1	1
**mmu00330**	mmu00330	Arginine and proline metabolism	3/579	54/9010	0.68354937	0.886846381	0.592368102	Aldh2/P4ha1/Got1	3
**mmu04978**	mmu04978	Mineral absorption	3/579	54/9010	0.68354937	0.886846381	0.592368102	Fth1/Ftl1/Atp2b1	3
**mmu04614**	mmu04614	Renin-angiotensin system	2/579	37/9010	0.697339286	0.900854614	0.601724886	Ctsa/Prcp	2
**mmu04918**	mmu04918	Thyroid hormone synthesis	4/579	74/9010	0.709035103	0.912049427	0.60920245	Gpx1/Atf2/Prkcb/Hsp90b1	4
**mmu05144**	mmu05144	Malaria	3/579	57/9010	0.71861772	0.920442271	0.614808441	Cr1l/Myd88/Cd81	3
**mmu05143**	mmu05143	African trypanosomiasis	2/579	39/9010	0.724806882	0.924435896	0.617475979	Prkcb/Myd88	2
**mmu04015**	mmu04015	Rap1 signaling pathway	12/579	214/9010	0.729565369	0.926578802	0.618907331	Actg1/Map2k1/Krit1/Prkcb/Rala/Kras/Rap1a/Akt2/Pik3r1/Ctnnb1/Tln1/Rapgef6	12
**mmu00100**	mmu00100	Steroid biosynthesis	1/579	20/9010	0.735481482	0.929111522	0.620599058	Lbr	1
**mmu00513**	mmu00513	Various types of N-glycan biosynthesis	2/579	40/9010	0.737733069	0.929111522	0.620599058	Stt3b/Hexa	2
**mmu04724**	mmu04724	Glutamatergic synapse	6/579	113/9010	0.741986916	0.930575257	0.621576758	Gng5/Prkcb/Ppp3ca/Gng2/Gls/Slc38a2	6
**mmu04330**	mmu04330	Notch signaling pathway	3/579	60/9010	0.750595528	0.935982845	0.625188751	Crebbp/Ep300/Snw1	3
**mmu00360**	mmu00360	Phenylalanine metabolism	1/579	21/9010	0.752517769	0.935982845	0.625188751	Got1	1
**mmu00230**	mmu00230	Purine metabolism	7/579	134/9010	0.766397466	0.949323611	0.634099701	Pde4b/Pde7a/Ak2/Pde3b/Nt5c/Prps1/Gmps	7
**mmu00514**	mmu00514	Other types of O-glycan biosynthesis	2/579	43/9010	0.773437859	0.954118014	0.637302118	St6gal1/Ogt	2
**mmu05217**	mmu05217	Basal cell carcinoma	3/579	63/9010	0.779594438	0.957787452	0.639753115	Gadd45b/Gadd45g/Ctnnb1	3
**mmu00620**	mmu00620	Pyruvate metabolism	2/579	44/9010	0.784361148	0.959726445	0.641048264	Acss1/Aldh2	2
**mmu04925**	mmu04925	Aldosterone synthesis and secretion	5/579	102/9010	0.793195202	0.965023977	0.644586744	Nr4a1/Atp2b1/Atf2/Prkcb/Camk2d	5
**mmu04977**	mmu04977	Vitamin digestion and absorption	1/579	24/9010	0.797330737	0.965023977	0.644586744	Lmbrd1	1
**mmu04114**	mmu04114	Oocyte meiosis	6/579	121/9010	0.798308871	0.965023977	0.644586744	Map2k1/Camk2d/Cul1/Ppp3ca/Ppp2r5a/Ppp2ca	6
**mmu04911**	mmu04911	Insulin secretion	4/579	86/9010	0.811943365	0.977579812	0.652973401	Atf2/Prkcb/Camk2d/Vamp2	4
**mmu00340**	mmu00340	Histidine metabolism	1/579	26/9010	0.822608084	0.982559655	0.656299682	Aldh2	1
**mmu00563**	mmu00563	Glycosylphosphatidylinositol (GPI)-anchor biosynthesis	1/579	26/9010	0.822608084	0.982559655	0.656299682	Pgap1	1
**mmu05146**	mmu05146	Amoebiasis	5/579	107/9010	0.826126014	0.982861384	0.656501221	Hspb1/Prkcb/Nfkb1/Lamb3/Pik3r1	5
**mmu00650**	mmu00650	Butanoate metabolism	1/579	27/9010	0.834040621	0.984495008	0.657592399	Hadha	1
**mmu04392**	mmu04392	Hippo signaling pathway - multiple species	1/579	27/9010	0.834040621	0.984495008	0.657592399	Limd1	1
**mmu00520**	mmu00520	Amino sugar and nucleotide sugar metabolism	2/579	51/9010	0.848612428	0.991239458	0.662097347	Cmah/Hexa	2
**mmu04927**	mmu04927	Cortisol synthesis and secretion	3/579	72/9010	0.850288613	0.991239458	0.662097347	Nr4a1/Atf2/Sp1	3
**mmu00062**	mmu00062	Fatty acid elongation	1/579	29/9010	0.854746115	0.991239458	0.662097347	Hadha	1
**mmu04726**	mmu04726	Serotonergic synapse	6/579	131/9010	0.854792992	0.991239458	0.662097347	Dusp1/Map2k1/Gng5/Prkcb/Kras/Gng2	6
**mmu00071**	mmu00071	Fatty acid degradation	2/579	52/9010	0.85622013	0.991239458	0.662097347	Aldh2/Hadha	2
**mmu01240**	mmu01240	Biosynthesis of cofactors	7/579	152/9010	0.865229847	0.99458611	0.66433274	Aldh2/Kynu/Umps/Ak2/Nadk/Coq2/Mat2b	7
**mmu04061**	mmu04061	Viral protein interaction with cytokine and cytokine receptor	4/579	95/9010	0.867870534	0.99458611	0.66433274	Il10ra/Il6st/Cxcr5/Ccr7	4
**mmu04971**	mmu04971	Gastric acid secretion	3/579	75/9010	0.869023744	0.99458611	0.66433274	Actg1/Prkcb/Camk2d	3
**mmu00053**	mmu00053	Ascorbate and aldarate metabolism	1/579	31/9010	0.872872243	0.99520661	0.664747202	Aldh2	1
**mmu05412**	mmu05412	Arrhythmogenic right ventricular cardiomyopathy	3/579	77/9010	0.880336476	0.999501235	0.667615793	Actg1/Itga4/Ctnnb1	3
**mmu00240**	mmu00240	Pyrimidine metabolism	2/579	56/9010	0.883280161	0.999501235	0.667615793	Umps/Nt5c	2
**mmu00280**	mmu00280	Valine, leucine and isoleucine degradation	2/579	57/9010	0.889267886	0.999996903	0.667946874	Aldh2/Hadha	2
**mmu01040**	mmu01040	Biosynthesis of unsaturated fatty acids	1/579	34/9010	0.895915916	0.999996903	0.667946874	Scd1	1
**mmu04360**	mmu04360	Axon guidance	8/579	181/9010	0.903081834	0.999996903	0.667946874	Cfl2/Ssh2/Nfatc3/Kras/Camk2d/Ppp3ca/Rock1/Pik3r1	8
**mmu01232**	mmu01232	Nucleotide metabolism	3/579	84/9010	0.913382231	0.999996903	0.667946874	Ak2/Nt5c/Gmps	3
**mmu01212**	mmu01212	Fatty acid metabolism	2/579	62/9010	0.91515199	0.999996903	0.667946874	Scd1/Hadha	2
**mmu04970**	mmu04970	Salivary secretion	3/579	85/9010	0.917359483	0.999996903	0.667946874	Atp2b1/Prkcb/Vamp2	3
**mmu01250**	mmu01250	Biosynthesis of nucleotide sugars	1/579	38/9010	0.920286929	0.999996903	0.667946874	Cmah	1
**mmu04146**	mmu04146	Peroxisome	3/579	86/9010	0.921169704	0.999996903	0.667946874	Ech1/Agps/Pex7	3
**mmu04613**	mmu04613	Neutrophil extracellular trap formation	9/579	208/9010	0.925120245	0.999996903	0.667946874	Actg1/Syk/Map2k1/Cr1l/Prkcb/Akt2/Nfkb1/Ncf4/Pik3r1	9

**mmu00350**	mmu00350	Tyrosine metabolism	1/579	40/9010	0.930243912	0.999996903	0.667946874	Got1	1
**mmu00970**	mmu00970	Aminoacyl-tRNA biosynthesis	2/579	66/9010	0.931651489	0.999996903	0.667946874	Sars/Gars	2
**mmu03320**	mmu03320	PPAR signaling pathway	3/579	89/9010	0.931657127	0.999996903	0.667946874	Ubc/Scd1/Dbi	3
**mmu05410**	mmu05410	Hypertrophic cardiomyopathy	3/579	91/9010	0.937919185	0.999996903	0.667946874	Actg1/Itga4/Prkag1	3
**mmu04610**	mmu04610	Complement and coagulation cascades	3/579	94/9010	0.94632439	0.999996903	0.667946874	Plaur/Cr1l/Cd55	3
**mmu04924**	mmu04924	Renin secretion	2/579	76/9010	0.960623344	0.999996903	0.667946874	Pde3b/Ppp3ca	2
**mmu03460**	mmu03460	Fanconi anemia pathway	1/579	51/9010	0.966531806	0.999996903	0.667946874	Top3b	1
**mmu04974**	mmu04974	Protein digestion and absorption	3/579	108/9010	0.973289932	0.999996903	0.667946874	Slc1a5/Prcp/Slc38a2	3
**mmu00590**	mmu00590	Arachidonic acid metabolism	2/579	85/9010	0.976295746	0.999996903	0.667946874	Ptges3/Zadh2	2
**mmu04260**	mmu04260	Cardiac muscle contraction	2/579	87/9010	0.978850375	0.999996903	0.667946874	Cox7b/Cox7a2	2
**mmu04512**	mmu04512	ECM-receptor interaction	2/579	88/9010	0.980025668	0.999996903	0.667946874	Itga4/Lamb3	2
**mmu04972**	mmu04972	Pancreatic secretion	3/579	114/9010	0.980362955	0.999996903	0.667946874	Atp2b1/Prkcb/Rap1a	3
**mmu00983**	mmu00983	Drug metabolism - other enzymes	2/579	92/9010	0.984125062	0.999996903	0.667946874	Umps/Gmps	2
**mmu04270**	mmu04270	Vascular smooth muscle contraction	4/579	144/9010	0.985319576	0.999996903	0.667946874	Map2k1/Prkcb/Rock1/Gna13	4
**mmu05414**	mmu05414	Dilated cardiomyopathy	2/579	94/9010	0.985855769	0.999996903	0.667946874	Actg1/Itga4	2
**mmu05322**	mmu05322	Systemic lupus erythematosus	4/579	149/9010	0.988498257	0.999996903	0.667946874	H2-Ab1/Snrpd3/H2-Eb2/Cd86	4
**mmu04750**	mmu04750	Inflammatory mediator regulation of TRP channels	3/579	127/9010	0.990066095	0.999996903	0.667946874	Prkcb/Camk2d/Pik3r1	3
**mmu00480**	mmu00480	Glutathione metabolism	1/579	72/9010	0.991785107	0.999996903	0.667946874	Gpx1	1
**mmu00980**	mmu00980	Metabolism of xenobiotics by cytochrome P450	1/579	73/9010	0.992317264	0.999996903	0.667946874	Ephx1	1
**mmu05204**	mmu05204	Chemical carcinogenesis - DNA adducts	1/579	84/9010	0.996324239	0.999996903	0.667946874	Ephx1	1
**mmu04060**	mmu04060	Cytokine-cytokine receptor interaction	9/579	292/9010	0.99680333	0.999996903	0.667946874	Il10ra/Ifngr1/Tnfrsf13c/Ifnar1/Il6st/Cxcr5/Il4ra/Ccr7/Il17ra	9
**mmu05034**	mmu05034	Alcoholism	5/579	205/9010	0.997567467	0.999996903	0.667946874	Atf2/Map2k1/Gng5/Kras/Gng2	5
**mmu05150**	mmu05150	Staphylococcus aureus infection	2/579	128/9010	0.998106334	0.999996903	0.667946874	H2-Ab1/H2-Eb2	2
**mmu04976**	mmu04976	Bile secretion	1/579	100/9010	0.998744206	0.999996903	0.667946874	Ephx1	1
**mmu04020**	mmu04020	Calcium signaling pathway	5/579	240/9010	0.999590311	0.999996903	0.667946874	Atp2b1/Prkcb/Camk2d/Ppp3ca/P2rx4	5
**mmu04080**	mmu04080	Neuroactive ligand-receptor interaction	7/579	386/9010	0.999996903	0.999996903	0.667946874	P2ry10/S1pr4/S1pr1/Nr3c1/Tspo/Cnr2/P2rx4	7
