# Supplementary material for: The Regulation Network of Glycerolipid Metabolism as Coregulators of Immunotherapy-Related Myocarditis
Source: Cardiovasc Ther. 2023 Jun 21;2023:8774971. doi: 10.1155/2023/8774971 (PMC10307211; doi:10.1155/2023/8774971)
Supplement: Supplementary 5 — Table S5: the Gene Set Enrichment Analysis (GSEA) map enrichment results for PTT-related DEGs of the T cell subpopulation. [file 8774971.f5.pdf]

Table S5

ID	Description	setSize	enrichmentScore	NES	pvalue	p.adjust	qvalues	rank	leading_edge
GOBP_AMIDE_BIOSYNTHETIC_PROCESS	GOBP_AMIDE_BIOSYNTHETIC_PROCESS	GOBP_AMIDE_BIOSYNTHETIC_PROCESS	111	0.374134495	3.175533752	1E-10	5.72E-08	5.35E-08	302
GOBP_CYTOPLASMIC_TRANSLATION	GOBP_CYTOPLASMIC_TRANSLATION	GOBP_CYTOPLASMIC_TRANSLATION	50	0.641221364	4.294788495	1E-10	5.72E-08	5.35E-08	284
GOBP_PEPTIDE_BIOSYNTHETIC_PROCESS	GOBP_PEPTIDE_BIOSYNTHETIC_PROCESS	GOBP_PEPTIDE_BIOSYNTHETIC_PROCESS	107	0.388128529	3.288181361	1E-10	5.72E-08	5.35E-08	302
GOBP_PEPTIDE_METABOLIC_PROCESS	GOBP_PEPTIDE_METABOLIC_PROCESS	GOBP_PEPTIDE_METABOLIC_PROCESS	118	0.35512504	3.077042096	1E-10	5.72E-08	5.35E-08	302
GOBP_RESPONSE_TO_TOPOLOGICALLY_INCORRECT_PROTEIN	GOBP_RESPONSE_TO_TOPOLOGICALLY_INCORRECT_PROTEIN	GOBP_RESPONSE_TO_TOPOLOGICALLY_INCORRECT_PROTEIN	42	0.5517403	3.520797721	1E-10	5.72E-08	5.35E-08	20
GOCC_CYTOSOLIC_LARGE_RIBOSOMAL_SUBUNIT	GOCC_CYTOSOLIC_LARGE_RIBOSOMAL_SUBUNIT	GOCC_CYTOSOLIC_LARGE_RIBOSOMAL_SUBUNIT	23	0.774416595	3.881178576	1E-10	5.72E-08	5.35E-08	284
GOCC_CYTOSOLIC_RIBOSOME	GOCC_CYTOSOLIC_RIBOSOME	GOCC_CYTOSOLIC_RIBOSOME	43	0.788038698	5.03783617	1E-10	5.72E-08	5.35E-08	284
GOCC_CYTOSOLIC_SMALL_RIBOSOMAL_SUBUNIT	GOCC_CYTOSOLIC_SMALL_RIBOSOMAL_SUBUNIT	GOCC_CYTOSOLIC_SMALL_RIBOSOMAL_SUBUNIT	21	0.781708369	3.828502731	1E-10	5.72E-08	5.35E-08	274
GOCC_LARGE_RIBOSOMAL_SUBUNIT	GOCC_LARGE_RIBOSOMAL_SUBUNIT	GOCC_LARGE_RIBOSOMAL_SUBUNIT	25	0.741419833	3.913143758	1E-10	5.72E-08	5.35E-08	284
GOCC_RIBOSOMAL_SUBUNIT	GOCC_RIBOSOMAL_SUBUNIT	GOCC_RIBOSOMAL_SUBUNIT	48	0.750756231	4.94887354	1E-10	5.72E-08	5.35E-08	284
GOCC_RIBOSOME	GOCC_RIBOSOME	GOCC_RIBOSOME	52	0.707062785	4.804897614	1E-10	5.72E-08	5.35E-08	294
GOCC_SMALL_RIBOSOMAL_SUBUNIT	GOCC_SMALL_RIBOSOMAL_SUBUNIT	GOCC_SMALL_RIBOSOMAL_SUBUNIT	24	0.740376007	3.860681353	1E-10	5.72E-08	5.35E-08	274
GOMF_STRUCTURAL_CONSTITUENT_OF_RIBOSOME	GOMF_STRUCTURAL_CONSTITUENT_OF_RIBOSOME	GOMF_STRUCTURAL_CONSTITUENT_OF_RIBOSOME	50	0.753584402	5.04737646	1E-10	5.72E-08	5.35E-08	284
GOMF_STRUCTURAL_MOLECULE_ACTIVITY	GOMF_STRUCTURAL_MOLECULE_ACTIVITY	GOMF_STRUCTURAL_MOLECULE_ACTIVITY	77	0.439831648	3.407863359	1E-10	5.72E-08	5.35E-08	284
HP_CENTROCECAL_SCOTOMA	HP_CENTROCECAL_SCOTOMA	HP_CENTROCECAL_SCOTOMA	10	-0.929059829	-2.932944493	1E-10	5.72E-08	5.35E-08	94
HP_MITOCHONDRIAL_INHERITANCE	HP_MITOCHONDRIAL_INHERITANCE	HP_MITOCHONDRIAL_INHERITANCE	12	-0.896316826	-2.983663652	1E-10	5.72E-08	5.35E-08	94
HP_MITOCHONDRIAL_RESPIRATORY_CHAIN_DEFECTS	HP_MITOCHONDRIAL_RESPIRATORY_CHAIN_DEFECTS	HP_MITOCHONDRIAL_RESPIRATORY_CHAIN_DEFECTS	10	-0.929059829	-2.932944493	1E-10	5.72E-08	5.35E-08	94
HP_RAGGED_RED_MUSCLE_FIBERS	HP_RAGGED_RED_MUSCLE_FIBERS	HP_RAGGED_RED_MUSCLE_FIBERS	16	-0.809827366	-2.986223646	1E-10	5.72E-08	5.35E-08	94
HP_VENTRICULAR_PREEXCITATION	HP_VENTRICULAR_PREEXCITATION	HP_VENTRICULAR_PREEXCITATION	11	-0.929854577	-3.016000923	1E-10	5.72E-08	5.35E-08	94
HP_CARDIAC_CONDUCTION_ABNORMALITY	HP_CARDIAC_CONDUCTION_ABNORMALITY	HP_CARDIAC_CONDUCTION_ABNORMALITY	17	-0.801096005	-3.046457207	1.08E-10	5.89E-08	5.51E-08	94
GOCC_RIBONUCLEOPROTEIN_COMPLEX	GOCC_RIBONUCLEOPROTEIN_COMPLEX	GOCC_RIBONUCLEOPROTEIN_COMPLEX	126	0.340876185	3.000522938	1.25E-10	6.46E-08	6.04E-08	300
HP_LEBER_OPTIC_ATROPHY	HP_LEBER_OPTIC_ATROPHY	HP_LEBER_OPTIC_ATROPHY	9	-0.928266439	-2.798747854	1.46E-10	6.6E-08	6.17E-08	94
HP_RETINAL_ARTERIAL_TORTUOSITY	HP_RETINAL_ARTERIAL_TORTUOSITY	HP_RETINAL_ARTERIAL_TORTUOSITY	9	-0.928266439	-2.798747854	1.46E-10	6.6E-08	6.17E-08	94
HP_VASCULAR_TORTUOSITY	HP_VASCULAR_TORTUOSITY	HP_VASCULAR_TORTUOSITY	9	-0.928266439	-2.798747854	1.46E-10	6.6E-08	6.17E-08	94
HP_CENTRAL_SCOTOMA	HP_CENTRAL_SCOTOMA	HP_CENTRAL_SCOTOMA	12	-0.862542663	-2.871236061	1.62E-10	6.86E-08	6.42E-08	94
GOMF_PROTEIN_FOLDING_CHAPERONE	GOMF_PROTEIN_FOLDING_CHAPERONE	GOMF_PROTEIN_FOLDING_CHAPERONE	11	0.884462171	3.288350597	1.64E-10	6.86E-08	6.42E-08	20
GOMF_UNFOLDED_PROTEIN_BINDING	GOMF_UNFOLDED_PROTEIN_BINDING	GOMF_UNFOLDED_PROTEIN_BINDING	22	0.713628477	3.516814537	4.28E-10	1.72E-07	1.61E-07	46
HP_RETINAL_VASCULAR_TORTUOSITY	HP_RETINAL_VASCULAR_TORTUOSITY	HP_RETINAL_VASCULAR_TORTUOSITY	12	-0.85274551	-2.838623249	4.88E-10	1.9E-07	1.77E-07	94
GOBP_PROTEIN_FOLDING	GOBP_PROTEIN_FOLDING	GOBP_PROTEIN_FOLDING	37	0.571922162	3.48416914	1.17E-09	4.26E-07	3.98E-07	46
HP_BLURRED_VISION	HP_BLURRED_VISION	HP_BLURRED_VISION	12	-0.842765908	-2.805403106	1.17E-09	4.26E-07	3.98E-07	94
HP_RETINAL_TELANGIECTASIA	HP_RETINAL_TELANGIECTASIA	HP_RETINAL_TELANGIECTASIA	12	-0.841923604	-2.80259924	1.29E-09	4.54E-07	4.25E-07	94
HP_SLOW_DECREASE_IN_VISUAL_ACUITY	HP_SLOW_DECREASE_IN_VISUAL_ACUITY	HP_SLOW_DECREASE_IN_VISUAL_ACUITY	12	-0.834153744	-2.776734894	2.19E-09	7.44E-07	6.96E-07	94
HP_STROKE_LIKE_EPISODE	HP_STROKE_LIKE_EPISODE	HP_STROKE_LIKE_EPISODE	8	-0.927474403	-2.661982024	3.62E-09	1.19E-06	1.11E-06	94
GOBP_DE_NOVO_PROTEIN_FOLDING	GOBP_DE_NOVO_PROTEIN_FOLDING	GOBP_DE_NOVO_PROTEIN_FOLDING	12	0.8405694	3.25809812	5.54E-09	1.77E-06	1.66E-06	72
HP_OPTIC_NEUROPATHY	HP_OPTIC_NEUROPATHY	HP_OPTIC_NEUROPATHY	13	-0.810887064	-2.779478929	8.37E-09	2.38E-06	2.23E-06	94
HP_MITOCHONDRIAL_MYOPATHY	HP_MITOCHONDRIAL_MYOPATHY	HP_MITOCHONDRIAL_MYOPATHY	15	-0.784885889	-2.810036616	9.29E-09	2.38E-06	2.23E-06	94
HP_MUSCLE_ABNORMALITY_RELATED_TO_MITOCHONDRIAL_DYSFUNCTION	HP_MUSCLE_ABNORMALITY_RELATED_TO_MITOCHONDRIAL_DYSFUNCTION	HP_MUSCLE_ABNORMALITY_RELATED_TO_MITOCHONDRIAL_DYSFUNCTION	15	-0.784885889	-2.810036616	9.29E-09	2.38E-06	2.23E-06	94
HP_ABNORMAL_BRAIN_LACTATE_LEVEL_BY_MRS	HP_ABNORMAL_BRAIN_LACTATE_LEVEL_BY_MRS	HP_ABNORMAL_BRAIN_LACTATE_LEVEL_BY_MRS	7	-0.926683717	-2.537820836	1.16E-08	2.38E-06	2.23E-06	94
HP_ABNORMAL_CIRCULATING_CITRULLINE_CONCENTRATION	HP_ABNORMAL_CIRCULATING_CITRULLINE_CONCENTRATION	HP_ABNORMAL_CIRCULATING_CITRULLINE_CONCENTRATION	7	-0.926683717	-2.537820836	1.16E-08	2.38E-06	2.23E-06	94
HP_ABNORMAL_CIRCULATING_NON_PROTEINOGENIC_AMINO_ACID_CONCENTRATION	HP_ABNORMAL_CIRCULATING_NON_PROTEINOGENIC_AMINO_ACID_CONCENTRATION	HP_ABNORMAL_CIRCULATING_NON_PROTEINOGENIC_AMINO_ACID_CONCENTRATION	7	-0.926683717	-2.537820836	1.16E-08	2.38E-06	2.23E-06	94
HP_ABNORMAL_SPEECH_PROSODY	HP_ABNORMAL_SPEECH_PROSODY	HP_ABNORMAL_SPEECH_PROSODY	7	-0.926683717	-2.537820836	1.16E-08	2.38E-06	2.23E-06	94
HP_CONCENTRIC_HYPERTROPHIC_CARDIOMYOPATHY	HP_CONCENTRIC_HYPERTROPHIC_CARDIOMYOPATHY	HP_CONCENTRIC_HYPERTROPHIC_CARDIOMYOPATHY	7	-0.926683717	-2.537820836	1.16E-08	2.38E-06	2.23E-06	94
HP_EPISODIC_RESPIRATORY_DISTRESS	HP_EPISODIC_RESPIRATORY_DISTRESS	HP_EPISODIC_RESPIRATORY_DISTRESS	7	-0.926683717	-2.537820836	1.16E-08	2.38E-06	2.23E-06	94
HP_FLUCTUATIONS_IN_CONSCIOUSNESS	HP_FLUCTUATIONS_IN_CONSCIOUSNESS	HP_FLUCTUATIONS_IN_CONSCIOUSNESS	7	-0.926683717	-2.537820836	1.16E-08	2.38E-06	2.23E-06	94
HP_LACTICACIDURIA	HP_LACTICACIDURIA	HP_LACTICACIDURIA	7	-0.926683717	-2.537820836	1.16E-08	2.38E-06	2.23E-06	94
HP_LOW_PLASMA_CITRULLINE	HP_LOW_PLASMA_CITRULLINE	HP_LOW_PLASMA_CITRULLINE	7	-0.926683717	-2.537820836	1.16E-08	2.38E-06	2.23E-06	94
HP_MIXED_DEMYELINATING_AND_AXONAL_POLYNEUROPATHY	HP_MIXED_DEMYELINATING_AND_AXONAL_POLYNEUROPATHY	HP_MIXED_DEMYELINATING_AND_AXONAL_POLYNEUROPATHY	7	-0.926683717	-2.537820836	1.16E-08	2.38E-06	2.23E-06	94
HP_MULTIPLE_GLOMERULAR_CYSTS	HP_MULTIPLE_GLOMERULAR_CYSTS	HP_MULTIPLE_GLOMERULAR_CYSTS	7	-0.926683717	-2.537820836	1.16E-08	2.38E-06	2.23E-06	94
HP_NEPHROPATHY	HP_NEPHROPATHY	HP_NEPHROPATHY	7	-0.926683717	-2.537820836	1.16E-08	2.38E-06	2.23E-06	94
HP_PSYCHOTIC_MENTATION	HP_PSYCHOTIC_MENTATION	HP_PSYCHOTIC_MENTATION	7	-0.926683717	-2.537820836	1.16E-08	2.38E-06	2.23E-06	94
HP_RECURRENT_PANCREATITIS	HP_RECURRENT_PANCREATITIS	HP_RECURRENT_PANCREATITIS	7	-0.926683717	-2.537820836	1.16E-08	2.38E-06	2.23E-06	94
HP_STUTTERING	HP_STUTTERING	HP_STUTTERING	7	-0.926683717	-2.537820836	1.16E-08	2.38E-06	2.23E-06	94
HP_WIDENED_CEREBRAL_SUBARACHNOID_SPACE	HP_WIDENED_CEREBRAL_SUBARACHNOID_SPACE	HP_WIDENED_CEREBRAL_SUBARACHNOID_SPACE	7	-0.926683717	-2.537820836	1.16E-08	2.38E-06	2.23E-06	94
GOBP_CHAPERONE_COFACTOR_DEPENDENT_PROTEIN_REFOLDING	GOBP_CHAPERONE_COFACTOR_DEPENDENT_PROTEIN_REFOLDING	GOBP_CHAPERONE_COFACTOR_DEPENDENT_PROTEIN_REFOLDING	10	0.860257267	3.022186839	1.32E-08	2.66E-06	2.49E-06	72
GOBP_CHAPERONE_MEDIATED_PROTEIN_FOLDING	GOBP_CHAPERONE_MEDIATED_PROTEIN_FOLDING	GOBP_CHAPERONE_MEDIATED_PROTEIN_FOLDING	15	0.773459892	3.264136444	1.38E-08	2.72E-06	2.55E-06	72
GOBP_CELLULAR_AMIDE_METABOLIC_PROCESS	GOBP_CELLULAR_AMIDE_METABOLIC_PROCESS	GOBP_CELLULAR_AMIDE_METABOLIC_PROCESS	130	0.307968225	2.699574782	1.4E-08	2.72E-06	2.55E-06	302
HP_EPISODIC_VOMITING	HP_EPISODIC_VOMITING	HP_EPISODIC_VOMITING	15	-0.779681852	-2.791405201	1.53E-08	2.91E-06	2.72E-06	152
HP_ABNORMALITY_OF_THE_THENAR_EMINENCE	HP_ABNORMALITY_OF_THE_THENAR_EMINENCE	HP_ABNORMALITY_OF_THE_THENAR_EMINENCE	14	0.777015437	3.140897811	1.91E-08	3.15E-06	2.95E-06	274
HP_ADENOCARCINOMA_OF_THE_COLON	HP_ADENOCARCINOMA_OF_THE_COLON	HP_ADENOCARCINOMA_OF_THE_COLON	14	0.777015437	3.140897811	1.91E-08	3.15E-06	2.95E-06	274
HP_ERYTHROID_HYPOPLASIA	HP_ERYTHROID_HYPOPLASIA	HP_ERYTHROID_HYPOPLASIA	14	0.777015437	3.140897811	1.91E-08	3.15E-06	2.95E-06	274
HP_MALIGNANT_GENITOURINARY_TRACT_TUMOR	HP_MALIGNANT_GENITOURINARY_TRACT_TUMOR	HP_MALIGNANT_GENITOURINARY_TRACT_TUMOR	14	0.777015437	3.140897811	1.91E-08	3.15E-06	2.95E-06	274
HP_OSTEOSARCOMA	HP_OSTEOSARCOMA	HP_OSTEOSARCOMA	14	0.777015437	3.140897811	1.91E-08	3.15E-06	2.95E-06	274
HP_PARTIAL_DUPLICATION_OF_THE_PHALANX_OF_HAND	HP_PARTIAL_DUPLICATION_OF_THE_PHALANX_OF_HAND	HP_PARTIAL_DUPLICATION_OF_THE_PHALANX_OF_HAND	14	0.777015437	3.140897811	1.91E-08	3.15E-06	2.95E-06	274
HP_PERSISTENCE_OF_HEMOGLOBIN_F	HP_PERSISTENCE_OF_HEMOGLOBIN_F	HP_PERSISTENCE_OF_HEMOGLOBIN_F	14	0.777015437	3.140897811	1.91E-08	3.15E-06	2.95E-06	274
HP_PURE_RED_CELL_APLASIA	HP_PURE_RED_CELL_APLASIA	HP_PURE_RED_CELL_APLASIA	14	0.777015437	3.140897811	1.91E-08	3.15E-06	2.95E-06	274
HP_RETICULOCYTOPENIA	HP_RETICULOCYTOPENIA	HP_RETICULOCYTOPENIA	14	0.777015437	3.140897811	1.91E-08	3.15E-06	2.95E-06	274
GOBP_ORGANONITROGEN_COMPOUND_BIOSYNTHETIC_PROCESS	GOBP_ORGANONITROGEN_COMPOUND_BIOSYNTHETIC_PROCESS	GOBP_ORGANONITROGEN_COMPOUND_BIOSYNTHETIC_PROCESS	176	0.259490562	2.427707667	2.2E-08	3.57E-06	3.34E-06	302
HP_ABNORMAL_MUSCLE_FIBER_MORPHOLOGY	HP_ABNORMAL_MUSCLE_FIBER_MORPHOLOGY	HP_ABNORMAL_MUSCLE_FIBER_MORPHOLOGY	24	-0.66025135	-2.766600773	3.32E-08	5.3E-06	4.96E-06	137
HP_ABNORMAL_MITOCHONDRIA_IN_MUSCLE_TISSUE	HP_ABNORMAL_MITOCHONDRIA_IN_MUSCLE_TISSUE	HP_ABNORMAL_MITOCHONDRIA_IN_MUSCLE_TISSUE	12	-0.801556784	-2.668225981	3.73E-08	5.8E-06	5.42E-06	94
HP_PROXIMAL_TUBULOPATHY	HP_PROXIMAL_TUBULOPATHY	HP_PROXIMAL_TUBULOPATHY	12	-0.801556784	-2.668225981	3.73E-08	5.8E-06	5.42E-06	94
GOBP_CELLULAR_MACROMOLECULE_BIOSYNTHETIC_PROCESS	GOBP_CELLULAR_MACROMOLECULE_BIOSYNTHETIC_PROCESS	GOBP_CELLULAR_MACROMOLECULE_BIOSYNTHETIC_PROCESS	178	0.258778732	2.412620446	4.39E-08	6.72E-06	6.29E-06	302
HP_INCREASED_CSF_LACTATE	HP_INCREASED_CSF_LACTATE	HP_INCREASED_CSF_LACTATE	14	-0.767581938	-2.705129605	4.87E-08	7.36E-06	6.88E-06	94
GOMF_ELECTRON_TRANSFER_ACTIVITY	GOMF_ELECTRON_TRANSFER_ACTIVITY	GOMF_ELECTRON_TRANSFER_ACTIVITY	21	-0.674980062	-2.720858552	5.48E-08	8.17E-06	7.64E-06	94
GOCC_RESPIRASOME	GOCC_RESPIRASOME	GOCC_RESPIRASOME	21	-0.673892514	-2.716474627	5.63E-08	8.28E-06	7.74E-06	94
GOMF_C3HC4_TYPE_RING_FINGER_DOMAIN_BINDING	GOMF_C3HC4_TYPE_RING_FINGER_DOMAIN_BINDING	GOMF_C3HC4_TYPE_RING_FINGER_DOMAIN_BINDING	4	0.988945578	2.279235337	7E-08	1.01E-05	9.49E-06	17
HP_PROGRESSIVE_SENSORINEURAL_HEARING_IMPAIRMENT	HP_PROGRESSIVE_SENSORINEURAL_HEARING_IMPAIRMENT	HP_PROGRESSIVE_SENSORINEURAL_HEARING_IMPAIRMENT	8	-0.895846625	-2.571205851	7.14E-08	1.02E-05	9.55E-06	49
HP_VENTRICULAR_ARRHYTHMIA	HP_VENTRICULAR_ARRHYTHMIA	HP_VENTRICULAR_ARRHYTHMIA	17	-0.73061158	-2.778414694	7.42E-08	1.05E-05	9.8E-06	94
HP_SPRENGEL_ANOMALY	HP_SPRENGEL_ANOMALY	HP_SPRENGEL_ANOMALY	15	0.747639485	3.155169798	7.51E-08	1.05E-05	9.8E-06	309
HP_SCOTOMA	HP_SCOTOMA	HP_SCOTOMA	15	-0.760105604	-2.721318611	8.23E-08	1.13E-05	1.06E-05	94
GOMF_PRIMARY_ACTIVE_TRANSMEMBRANE_TRANSPORTER_ACTIVITY	GOMF_PRIMARY_ACTIVE_TRANSMEMBRANE_TRANSPORTER_ACTIVITY	GOMF_PRIMARY_ACTIVE_TRANSMEMBRANE_TRANSPORTER_ACTIVITY	23	-0.661902244	-2.774123204	9.76E-08	1.33E-05	1.24E-05	94
GOMF_OXIDOREDUCTION_DRIVEN_ACTIVE_TRANSMEMBRANE_TRANSPORTER_ACTIVIT	GOMF_OXIDOREDUCTION_DRIVEN_ACTIVE_TRANSMEMBRANE_TRANSPORTER_ACTIVIT	GOMF_OXIDOREDUCTION_DRIVEN_ACTIVE_TRANSMEMBRANE_TRANSPORTER_ACTIVIT	20	-0.690838085	-2.752785118	1.22E-07	1.64E-05	1.53E-05	94
HP_ABNORMAL_CSF_METABOLITE_LEVEL	HP_ABNORMAL_CSF_METABOLITE_LEVEL	HP_ABNORMAL_CSF_METABOLITE_LEVEL	16	-0.727680089	-2.683307059	1.37E-07	1.82E-05	1.7E-05	125
HP_PROGRESSIVE_EXTERNAL_OPHTHALMOPLEGIA	HP_PROGRESSIVE_EXTERNAL_OPHTHALMOPLEGIA	HP_PROGRESSIVE_EXTERNAL_OPHTHALMOPLEGIA	10	-0.836937804	-2.642124917	1.47E-07	1.93E-05	1.81E-05	94
HP_PIGMENTARY_RETINOPATHY	HP_PIGMENTARY_RETINOPATHY	HP_PIGMENTARY_RETINOPATHY	16	-0.725369073	-2.67478523	1.63E-07	2.11E-05	1.97E-05	94
HP_DUPLICATION_OF_THUMB_PHALANX	HP_DUPLICATION_OF_THUMB_PHALANX	HP_DUPLICATION_OF_THUMB_PHALANX	16	0.716625794	3.107951514	1.76E-07	2.25E-05	2.1E-05	274
HP_BASAL_GANGLIA_CALCIFICATION	HP_BASAL_GANGLIA_CALCIFICATION	HP_BASAL_GANGLIA_CALCIFICATION	8	-0.880868505	-2.528216539	2.08E-07	2.63E-05	2.46E-05	94
HP_VITILIGO	HP_VITILIGO	HP_VITILIGO	8	-0.878821225	-2.52234056	2.72E-07	3.39E-05	3.17E-05	94
HP_EPILEPTIC_SPASM	HP_EPILEPTIC_SPASM	HP_EPILEPTIC_SPASM	28	-0.603363459	-2.652768863	2.83E-07	3.39E-05	3.17E-05	242
HP_ABNORMAL_RETICULOCYTE_MORPHOLOGY	HP_ABNORMAL_RETICULOCYTE_MORPHOLOGY	HP_ABNORMAL_RETICULOCYTE_MORPHOLOGY	16	0.709258143	3.075998574	2.85E-07	3.39E-05	3.17E-05	274
HP_ABNORMALITY_OF_THE_MITOCHONDRION	HP_ABNORMALITY_OF_THE_MITOCHONDRION	HP_ABNORMALITY_OF_THE_MITOCHONDRION	23	-0.647032802	-2.711803331	2.89E-07	3.39E-05	3.17E-05	94
HP_INFANTILE_SPASMS	HP_INFANTILE_SPASMS	HP_INFANTILE_SPASMS	11	-0.80080043	-2.597411355	2.89E-07	3.39E-05	3.17E-05	125
HP_ABNORMALITY_OF_HEAD_BLOOD_VESSEL	HP_ABNORMALITY_OF_HEAD_BLOOD_VESSEL	HP_ABNORMALITY_OF_HEAD_BLOOD_VESSEL	14	-0.744601062	-2.624139883	2.9E-07	3.39E-05	3.17E-05	94
HP_ABNORMAL_RETINAL_ARTERY_MORPHOLOGY	HP_ABNORMAL_RETINAL_ARTERY_MORPHOLOGY	HP_ABNORMAL_RETINAL_ARTERY_MORPHOLOGY	14	-0.744601062	-2.624139883	2.9E-07	3.39E-05	3.17E-05	94
HP_EXTERNAL_OPHTHALMOPLEGIA	HP_EXTERNAL_OPHTHALMOPLEGIA	HP_EXTERNAL_OPHTHALMOPLEGIA	12	-0.765632272	-2.548640296	3.25E-07	3.77E-05	3.52E-05	94
GOMF_HEAT_SHOCK_PROTEIN_BINDING	GOMF_HEAT_SHOCK_PROTEIN_BINDING	GOMF_HEAT_SHOCK_PROTEIN_BINDING	25	0.587124006	3.098784978	3.43E-07	3.93E-05	3.68E-05	125
HP_VISUAL_IMPAIRMENT	HP_VISUAL_IMPAIRMENT	HP_VISUAL_IMPAIRMENT	101	-0.394190371	-2.469415706	3.6E-07	4.08E-05	3.82E-05	253
GOBP_CELLULAR_RESPONSE_TO_CHEMICAL_STRESS	GOBP_CELLULAR_RESPONSE_TO_CHEMICAL_STRESS	GOBP_CELLULAR_RESPONSE_TO_CHEMICAL_STRESS	38	0.490552573	3.048746156	3.73E-07	4.14E-05	3.88E-05	42
GOCC_INNER_MITOCHONDRIAL_MEMBRANE_PROTEIN_COMPLEX	GOCC_INNER_MITOCHONDRIAL_MEMBRANE_PROTEIN_COMPLEX	GOCC_INNER_MITOCHONDRIAL_MEMBRANE_PROTEIN_COMPLEX	29	-0.598847535	-2.674989671	3.73E-07	4.14E-05	3.88E-05	94
HP_RENAL_TUBULAR_DYSFUNCTION	HP_RENAL_TUBULAR_DYSFUNCTION	HP_RENAL_TUBULAR_DYSFUNCTION	14	-0.741246227	-2.612316697	3.83E-07	4.17E-05	3.9E-05	94
GOBP_ATP_SYNTHESIS_COUPLED_ELECTRON_TRANSPORT	GOBP_ATP_SYNTHESIS_COUPLED_ELECTRON_TRANSPORT	GOBP_ATP_SYNTHESIS_COUPLED_ELECTRON_TRANSPORT	20	-0.674290489	-2.686847852	3.84E-07	4.17E-05	3.9E-05	94
HP_POSTURAL_TREMOR	HP_POSTURAL_TREMOR	HP_POSTURAL_TREMOR	16	-0.710530223	-2.62006724	4E-07	4.28E-05	4E-05	94
HP_OPHTHALMOPARESIS	HP_OPHTHALMOPARESIS	HP_OPHTHALMOPARESIS	25	-0.622913307	-2.640042811	4.01E-07	4.28E-05	4E-05	222
HP_TRIPHALANGEAL_THUMB	HP_TRIPHALANGEAL_THUMB	HP_TRIPHALANGEAL_THUMB	15	0.71992504	3.038209976	4.12E-07	4.35E-05	4.07E-05	274
GOBP_RESPIRATORY_ELECTRON_TRANSPORT_CHAIN	GOBP_RESPIRATORY_ELECTRON_TRANSPORT_CHAIN	GOBP_RESPIRATORY_ELECTRON_TRANSPORT_CHAIN	21	-0.645079651	-2.600329379	4.33E-07	4.53E-05	4.24E-05	94
HP_HYPERVENTILATION	HP_HYPERVENTILATION	HP_HYPERVENTILATION	9	-0.837155422	-2.524045731	5.21E-07	5.4E-05	5.05E-05	114
HP_VISUAL_FIELD_DEFECT	HP_VISUAL_FIELD_DEFECT	HP_VISUAL_FIELD_DEFECT	22	-0.63297285	-2.595267694	5.52E-07	5.66E-05	5.29E-05	159
GOBP_CELLULAR_RESPONSE_TO_STRESS	GOBP_CELLULAR_RESPONSE_TO_STRESS	GOBP_CELLULAR_RESPONSE_TO_STRESS	191	0.241313685	2.276370598	5.72E-07	5.82E-05	5.44E-05	57
HP_FOCAL_SEGMENTAL_GLOMERULOSCLEROSIS	HP_FOCAL_SEGMENTAL_GLOMERULOSCLEROSIS	HP_FOCAL_SEGMENTAL_GLOMERULOSCLEROSIS	9	-0.835085022	-2.517803418	5.87E-07	5.86E-05	5.48E-05	94
HP_MOTOR_SEIZURE	HP_MOTOR_SEIZURE	HP_MOTOR_SEIZURE	38	-0.534569886	-2.611512968	5.87E-07	5.86E-05	5.48E-05	242
GOCC_TRANSPORTER_COMPLEX	GOCC_TRANSPORTER_COMPLEX	GOCC_TRANSPORTER_COMPLEX	25	-0.616868863	-2.614425138	5.98E-07	5.87E-05	5.49E-05	94
HP_DISTAL_PERIPHERAL_SENSORY_NEUROPATHY	HP_DISTAL_PERIPHERAL_SENSORY_NEUROPATHY	HP_DISTAL_PERIPHERAL_SENSORY_NEUROPATHY	8	-0.868887353	-2.493828949	6E-07	5.87E-05	5.49E-05	94
GOBP_OXIDATIVE_PHOSPHORYLATION	GOBP_OXIDATIVE_PHOSPHORYLATION	GOBP_OXIDATIVE_PHOSPHORYLATION	28	-0.592562063	-2.605279066	6.1E-07	5.93E-05	5.54E-05	94
HP_HYPOTHERMIA	HP_HYPOTHERMIA	HP_HYPOTHERMIA	8	-0.868440317	-2.492545892	6.2E-07	5.96E-05	5.58E-05	94
HP_ABNORMAL_LEFT_VENTRICLE_MORPHOLOGY	HP_ABNORMAL_LEFT_VENTRICLE_MORPHOLOGY	HP_ABNORMAL_LEFT_VENTRICLE_MORPHOLOGY	11	-0.789070859	-2.559366271	6.33E-07	5.98E-05	5.6E-05	49
HP_LEFT_VENTRICULAR_HYPERTROPHY	HP_LEFT_VENTRICULAR_HYPERTROPHY	HP_LEFT_VENTRICULAR_HYPERTROPHY	11	-0.789070859	-2.559366271	6.33E-07	5.98E-05	5.6E-05	49
GOMF_UBIQUITIN_LIKE_PROTEIN_LIGASE_BINDING	GOMF_UBIQUITIN_LIKE_PROTEIN_LIGASE_BINDING	GOMF_UBIQUITIN_LIKE_PROTEIN_LIGASE_BINDING	60	0.389261722	2.788668669	6.48E-07	6.08E-05	5.68E-05	66
HP_IMPAIRED_VISUOSPATIAL_CONSTRUCTIVE_COGNITION	HP_IMPAIRED_VISUOSPATIAL_CONSTRUCTIVE_COGNITION	HP_IMPAIRED_VISUOSPATIAL_CONSTRUCTIVE_COGNITION	8	-0.864250943	-2.480521801	7.88E-07	7.32E-05	6.85E-05	94
HP_ABNORMAL_METABOLIC_BRAIN_IMAGING_BY_MRS	HP_ABNORMAL_METABOLIC_BRAIN_IMAGING_BY_MRS	HP_ABNORMAL_METABOLIC_BRAIN_IMAGING_BY_MRS	9	-0.828331725	-2.49744205	9.02E-07	8.25E-05	7.72E-05	94
HP_BILATERAL_TONIC_CLONIC_SEIZURE	HP_BILATERAL_TONIC_CLONIC_SEIZURE	HP_BILATERAL_TONIC_CLONIC_SEIZURE	37	-0.540092203	-2.623844505	9.03E-07	8.25E-05	7.72E-05	114
GOCC_POLYSOMAL_RIBOSOME	GOCC_POLYSOMAL_RIBOSOME	GOCC_POLYSOMAL_RIBOSOME	16	0.681672824	2.956363145	1.07E-06	9.62E-05	9E-05	294
HP_RECURRENT_PAROXYSMAL_HEADACHE	HP_RECURRENT_PAROXYSMAL_HEADACHE	HP_RECURRENT_PAROXYSMAL_HEADACHE	8	-0.858705192	-2.464604714	1.07E-06	9.62E-05	9E-05	94
GOBP_REGULATION_OF_DNA_TEMPLATED_TRANSCRIPTION_IN_RESPONSE_TO_STRESS	GOBP_REGULATION_OF_DNA_TEMPLATED_TRANSCRIPTION_IN_RESPONSE_TO_STRESS	GOBP_REGULATION_OF_DNA_TEMPLATED_TRANSCRIPTION_IN_RESPONSE_TO_STRESS	12	0.759463537	2.943726864	1.21E-06	0.0001082	0.0001012	75
HP_DEVELOPMENTAL_GLAUCOMA	HP_DEVELOPMENTAL_GLAUCOMA	HP_DEVELOPMENTAL_GLAUCOMA	15	0.701539832	2.960621175	1.32E-06	0.0001165	0.0001090	274
HP_SEGMENTAL_PERIPHERAL_DEMYELINATION_REMYELINATION	HP_SEGMENTAL_PERIPHERAL_DEMYELINATION_REMYELINATION	HP_SEGMENTAL_PERIPHERAL_DEMYELINATION_REMYELINATION	9	-0.822223873	-2.479026716	1.39E-06	0.0001219	0.0001140	94
HP_HEMIANOPIA	HP_HEMIANOPIA	HP_HEMIANOPIA	10	-0.803087148	-2.535261943	1.42E-06	0.0001232	0.0001152	49
HP_ABNORMALITY_OF_KREBS_CYCLE_METABOLISM	HP_ABNORMALITY_OF_KREBS_CYCLE_METABOLISM	HP_ABNORMALITY_OF_KREBS_CYCLE_METABOLISM	8	-0.852855747	-2.447815984	1.47E-06	0.0001272	0.0001190	94
HP_APLASIA_HYPOPLASIA_OF_THE_CEREBRAL_WHITE_MATTER	HP_APLASIA_HYPOPLASIA_OF_THE_CEREBRAL_WHITE_MATTER	HP_APLASIA_HYPOPLASIA_OF_THE_CEREBRAL_WHITE_MATTER	9	-0.8200019	-2.472327408	1.5E-06	0.0001280	0.0001198	152
GOMF_ACTIVE_TRANSMEMBRANE_TRANSPORTER_ACTIVITY	GOMF_ACTIVE_TRANSMEMBRANE_TRANSPORTER_ACTIVITY	GOMF_ACTIVE_TRANSMEMBRANE_TRANSPORTER_ACTIVITY	28	-0.576071425	-2.532775752	1.66E-06	0.0001406	0.0001315	94
HP_PERIPHERAL_DEMYELINATION	HP_PERIPHERAL_DEMYELINATION	HP_PERIPHERAL_DEMYELINATION	13	-0.734509029	-2.517677811	1.79E-06	0.0001509	0.0001411	125
HP_MACROCYTIC_ANEMIA	HP_MACROCYTIC_ANEMIA	HP_MACROCYTIC_ANEMIA	18	0.649058938	2.978780192	1.93E-06	0.0001612	0.0001508	304
HP_VENTRICULAR_HYPERTROPHY	HP_VENTRICULAR_HYPERTROPHY	HP_VENTRICULAR_HYPERTROPHY	13	-0.728864176	-2.49832894	2.33E-06	0.0001935	0.0001810	49
GOBP_RESPONSE_TO_TEMPERATURE_STIMULUS	GOBP_RESPONSE_TO_TEMPERATURE_STIMULUS	GOBP_RESPONSE_TO_TEMPERATURE_STIMULUS	26	0.540520491	2.874297916	2.47E-06	0.0002031	0.0001900	46
GOBP_ELECTRON_TRANSPORT_CHAIN	GOBP_ELECTRON_TRANSPORT_CHAIN	GOBP_ELECTRON_TRANSPORT_CHAIN	23	-0.613684019	-2.572034001	2.49E-06	0.0002035	0.0001904	94
HP_ABNORMAL_ERYTHROCYTE_ENZYME_LEVEL	HP_ABNORMAL_ERYTHROCYTE_ENZYME_LEVEL	HP_ABNORMAL_ERYTHROCYTE_ENZYME_LEVEL	16	0.66042755	2.86422401	2.7E-06	0.0002173	0.0002032	274
HP_NONIMMUNE_HYDROPS_FETALIS	HP_NONIMMUNE_HYDROPS_FETALIS	HP_NONIMMUNE_HYDROPS_FETALIS	16	0.660526666	2.864653867	2.7E-06	0.0002173	0.0002032	274
HP_ABNORMAL_HEMOGLOBIN	HP_ABNORMAL_HEMOGLOBIN	HP_ABNORMAL_HEMOGLOBIN	16	0.659295103	2.859312673	2.78E-06	0.0002223	0.0002080	274

HP_PHENOTYPIC_VARIABILITY	HP_PHENOTYPIC_VARIABILITY	HP_PHENOTYPIC_VARIABILITY	38	-0.516745819	-2.524437764	2.84E-06	0.0002243	0.0002098	160
HP_ABNORMAL_CIRCULATING_PYRUVATE_FAMILY_AMINO_ACID_CONCENTRATION	HP_ABNORMAL_CIRCULATING_PYRUVATE_FAMILY_AMINO_ACID_CONCENTRATION	HP_ABNORMAL_CIRCULATING_PYRUVATE_FAMILY_AMINO_ACID_CONCENTRATION	9	-0.812344378	-2.449239776	2.85E-06	0.0002243	0.0002098	94
GOBP_AEROBIC_RESPIRATION	GOBP_AEROBIC_RESPIRATION	GOBP_AEROBIC_RESPIRATION	30	-0.563862461	-2.543671352	3.25E-06	0.0002545	0.0002380	94
HP_APHASIA	HP_APHASIA	HP_APHASIA	27	-0.569126406	-2.465024783	3.46E-06	0.0002689	0.0002515	177
HP_ENCEPHALOPATHY	HP_ENCEPHALOPATHY	HP_ENCEPHALOPATHY	36	-0.519322986	-2.503471852	3.63E-06	0.0002801	0.0002620	139
HP_ABNORMAL_URINE_PH	HP_ABNORMAL_URINE_PH	HP_ABNORMAL_URINE_PH	9	-0.806044112	-2.430244307	3.7E-06	0.0002832	0.0002649	94
HP_SEVERE_GLOBAL_DEVELOPMENTAL_DELAY	HP_SEVERE_GLOBAL_DEVELOPMENTAL_DELAY	HP_SEVERE_GLOBAL_DEVELOPMENTAL_DELAY	22	-0.599697112	-2.458833015	3.8E-06	0.0002875	0.0002689	113
HP_GENERALIZED_ONSET_SEIZURE	HP_GENERALIZED_ONSET_SEIZURE	HP_GENERALIZED_ONSET_SEIZURE	40	-0.501252958	-2.526124061	3.81E-06	0.0002875	0.0002689	242
HP_PROGRESSIVE_VISUAL_LOSS	HP_PROGRESSIVE_VISUAL_LOSS	HP_PROGRESSIVE_VISUAL_LOSS	17	-0.66835566	-2.541664053	4.31E-06	0.0003232	0.0003023	94
HP_NAUSEA_AND_VOMITING	HP_NAUSEA_AND_VOMITING	HP_NAUSEA_AND_VOMITING	49	-0.465948915	-2.468988463	4.63E-06	0.0003447	0.0003224	361
GOBP_CELLULAR_RESPIRATION	GOBP_CELLULAR_RESPIRATION	GOBP_CELLULAR_RESPIRATION	32	-0.539657856	-2.478913791	4.91E-06	0.0003607	0.0003374	94
HP_VARIABLE_EXPRESSIVITY	HP_VARIABLE_EXPRESSIVITY	HP_VARIABLE_EXPRESSIVITY	26	-0.580775608	-2.483369887	4.92E-06	0.0003607	0.0003374	160
HP_ELEVATED_PULMONARY_ARTERY_PRESSURE	HP_ELEVATED_PULMONARY_ARTERY_PRESSURE	HP_ELEVATED_PULMONARY_ARTERY_PRESSURE	17	-0.665064276	-2.529147373	4.94E-06	0.0003607	0.0003374	211
HP_PROGRESSIVE_HEARING_IMPAIRMENT	HP_PROGRESSIVE_HEARING_IMPAIRMENT	HP_PROGRESSIVE_HEARING_IMPAIRMENT	10	-0.77824601	-2.456841076	5.04E-06	0.0003655	0.0003419	123
HP_ERYTHEMA	HP_ERYTHEMA	HP_ERYTHEMA	14	-0.701015239	-2.47053374	5.84E-06	0.0004207	0.0003936	214
HP_LACTIC_ACIDOSIS	HP_LACTIC_ACIDOSIS	HP_LACTIC_ACIDOSIS	21	-0.603546189	-2.432907138	5.98E-06	0.0004278	0.0004002	94
HP_FEVER	HP_FEVER	HP_FEVER	52	-0.455122669	-2.448259884	6.13E-06	0.0004356	0.0004075	277
GOCC_POLYSOME	GOCC_POLYSOME	GOCC_POLYSOME	20	0.595609422	2.848260249	6.25E-06	0.0004415	0.0004130	294
HP_ABSENT_THUMB	HP_ABSENT_THUMB	HP_ABSENT_THUMB	15	0.67179011	2.83507213	6.29E-06	0.0004415	0.0004130	274
HP_PANCREATITIS	HP_PANCREATITIS	HP_PANCREATITIS	12	-0.714000292	-2.376767519	6.57E-06	0.0004581	0.0004286	114
GOBP_RESPONSE_TO_HEAT	GOBP_RESPONSE_TO_HEAT	GOBP_RESPONSE_TO_HEAT	15	0.668894683	2.822852918	6.94E-06	0.0004806	0.0004496	20
HP_ABNORMAL_CARDIAC_VENTRICULAR_FUNCTION	HP_ABNORMAL_CARDIAC_VENTRICULAR_FUNCTION	HP_ABNORMAL_CARDIAC_VENTRICULAR_FUNCTION	11	-0.750152794	-2.43313479	7.39E-06	0.0005031	0.0004706	49
HP_ABNORMAL_BASAL_GANGLIA_MRI_SIGNAL_INTENSITY	HP_ABNORMAL_BASAL_GANGLIA_MRI_SIGNAL_INTENSITY	HP_ABNORMAL_BASAL_GANGLIA_MRI_SIGNAL_INTENSITY	10	-0.771607034	-2.435882525	7.4E-06	0.0005031	0.0004706	94
HP_FOCAL_T2_HYPERINTENSE_BASAL_GANGLIA_LESION	HP_FOCAL_T2_HYPERINTENSE_BASAL_GANGLIA_LESION	HP_FOCAL_T2_HYPERINTENSE_BASAL_GANGLIA_LESION	10	-0.771607034	-2.435882525	7.4E-06	0.0005031	0.0004706	94
HP_TYPE_II_DIABETES_MELLITUS	HP_TYPE_II_DIABETES_MELLITUS	HP_TYPE_II_DIABETES_MELLITUS	13	-0.710317801	-2.4347575	7.65E-06	0.0005166	0.0004832	94
HP_GENERALIZED_ONSET_MOTOR_SEIZURE	HP_GENERALIZED_ONSET_MOTOR_SEIZURE	HP_GENERALIZED_ONSET_MOTOR_SEIZURE	27	-0.553366443	-2.396764554	7.74E-06	0.0005194	0.0004858	242
HP_ABNORMALITY_OF_THE_BASAL_GANGLIA	HP_ABNORMALITY_OF_THE_BASAL_GANGLIA	HP_ABNORMALITY_OF_THE_BASAL_GANGLIA	29	-0.554834894	-2.47838978	7.82E-06	0.0005218	0.0004881	234
GOBP_ENERGY_DERIVATION_BY_OXIDATION_OF_ORGANIC_COMPOUNDS	GOBP_ENERGY_DERIVATION_BY_OXIDATION_OF_ORGANIC_COMPOUNDS	GOBP_ENERGY_DERIVATION_BY_OXIDATION_OF_ORGANIC_COMPOUNDS	38	-0.501992741	-2.452365138	8.26E-06	0.0005477	0.0005123	94
HP_GLOMERULAR_SCLEROSIS	HP_GLOMERULAR_SCLEROSIS	HP_GLOMERULAR_SCLEROSIS	11	-0.747273826	-2.423796801	8.86E-06	0.0005839	0.0005462	94
HP_ADENOCARCINOMA_OF_THE_INTESTINES	HP_ADENOCARCINOMA_OF_THE_INTESTINES	HP_ADENOCARCINOMA_OF_THE_INTESTINES	15	0.662563804	2.796135496	9.52E-06	0.0006220	0.0005818	274
HP_MYOPATHY	HP_MYOPATHY	HP_MYOPATHY	31	-0.527997349	-2.409382815	9.55E-06	0.0006220	0.0005818	95
HP_ABNORMAL_CIRCULATING_PROTEINOGENIC_AMINO_ACID_DERIVATIVE_CONCENTR	HP_ABNORMAL_CIRCULATING_PROTEINOGENIC_AMINO_ACID_DERIVATIVE_CONCENTR	HP_ABNORMAL_CIRCULATING_PROTEINOGENIC_AMINO_ACID_DERIVATIVE_CONCENTR	10	-0.767250823	-2.422130422	1E-05	0.0006464	0.0006046	94
HP_ABNORMALITY_OF_RETINAL_PIGMENTATION	HP_ABNORMALITY_OF_RETINAL_PIGMENTATION	HP_ABNORMALITY_OF_RETINAL_PIGMENTATION	25	-0.574755519	-2.435939577	1E-05	0.0006464	0.0006046	94
HP_ABNORMAL_RENAL_TUBULE_MORPHOLOGY	HP_ABNORMAL_RENAL_TUBULE_MORPHOLOGY	HP_ABNORMAL_RENAL_TUBULE_MORPHOLOGY	10	-0.765902739	-2.417874664	1.03E-05	0.0006616	0.0006189	94
HP_RETINAL_DYSTROPHY	HP_RETINAL_DYSTROPHY	HP_RETINAL_DYSTROPHY	22	-0.584379731	-2.396029839	1.11E-05	0.0007028	0.0006574	94
HP_VOMITING	HP_VOMITING	HP_VOMITING	32	-0.524641584	-2.409936675	1.12E-05	0.0007085	0.0006628	152
HP_VISUAL_LOSS	HP_VISUAL_LOSS	HP_VISUAL_LOSS	32	-0.523526782	-2.404815838	1.16E-05	0.0007285	0.0006815	125
HP_HYPOPARATHYROIDISM	HP_HYPOPARATHYROIDISM	HP_HYPOPARATHYROIDISM	12	-0.69907621	-2.327088164	1.22E-05	0.0007595	0.0007105	94
GOBP_RESPONSE_TO_ABIOTIC_STIMULUS	GOBP_RESPONSE_TO_ABIOTIC_STIMULUS	GOBP_RESPONSE_TO_ABIOTIC_STIMULUS	96	0.295304081	2.463990789	1.23E-05	0.0007617	0.0007125	82
HP_DEMYELINATING_PERIPHERAL_NEUROPATHY	HP_DEMYELINATING_PERIPHERAL_NEUROPATHY	HP_DEMYELINATING_PERIPHERAL_NEUROPATHY	11	-0.740422688	-2.401575007	1.23E-05	0.0007617	0.0007125	143
HP_POLYNEUROPATHY	HP_POLYNEUROPATHY	HP_POLYNEUROPATHY	19	-0.628702925	-2.459834875	1.33E-05	0.0008158	0.0007631	125
GOCC_OXIDOREDUCTASE_COMPLEX	GOCC_OXIDOREDUCTASE_COMPLEX	GOCC_OXIDOREDUCTASE_COMPLEX	18	-0.632359305	-2.45580702	1.4E-05	0.0008581	0.0008026	94
HP_ACTION_TREMOR	HP_ACTION_TREMOR	HP_ACTION_TREMOR	28	-0.54094485	-2.378337026	1.44E-05	0.0008755	0.0008189	143
HP_SENSORY_NEUROPATHY	HP_SENSORY_NEUROPATHY	HP_SENSORY_NEUROPATHY	14	-0.682791028	-2.406307566	1.46E-05	0.0008822	0.0008252	94
HP_ABNORMAL_MEAN_CORPUSCULAR_VOLUME	HP_ABNORMAL_MEAN_CORPUSCULAR_VOLUME	HP_ABNORMAL_MEAN_CORPUSCULAR_VOLUME	16	0.62412288	2.706773419	1.52E-05	0.0009061	0.0008476	274
HP_INCREASED_MEAN_CORPUSCULAR_VOLUME	HP_INCREASED_MEAN_CORPUSCULAR_VOLUME	HP_INCREASED_MEAN_CORPUSCULAR_VOLUME	16	0.62412288	2.706773419	1.52E-05	0.0009061	0.0008476	274
HP_HEMIPARESIS	HP_HEMIPARESIS	HP_HEMIPARESIS	14	-0.680469895	-2.398127375	1.58E-05	0.0009405	0.0008798	211
HP_SENSORIMOTOR_NEUROPATHY	HP_SENSORIMOTOR_NEUROPATHY	HP_SENSORIMOTOR_NEUROPATHY	20	-0.617954153	-2.46236424	1.59E-05	0.0009426	0.0008817	143
HP_INTESTINAL_PSEUDO_OBSTRUCTION	HP_INTESTINAL_PSEUDO_OBSTRUCTION	HP_INTESTINAL_PSEUDO_OBSTRUCTION	10	-0.758870596	-2.395674925	1.64E-05	0.0009601	0.0008981	159
HP_DYSTONIA	HP_DYSTONIA	HP_DYSTONIA	47	-0.457725795	-2.391487509	1.64E-05	0.0009601	0.0008981	242
GOBP_REGULATION_OF_CELL_DEATH	GOBP_REGULATION_OF_CELL_DEATH	GOBP_REGULATION_OF_CELL_DEATH	153	0.229949439	2.080227051	1.81E-05	0.0010550	0.0009868	57
HP_ABNORMAL_NUMBER_OF_ERYTHROID_PRECURSORS	HP_ABNORMAL_NUMBER_OF_ERYTHROID_PRECURSORS	HP_ABNORMAL_NUMBER_OF_ERYTHROID_PRECURSORS	15	0.647707835	2.733440704	1.91E-05	0.0011011	0.0010300	274
HP_NORMOCHROMIC_ANEMIA	HP_NORMOCHROMIC_ANEMIA	HP_NORMOCHROMIC_ANEMIA	15	0.647707835	2.733440704	1.91E-05	0.0011011	0.0010300	274
GOBP_ION_TRANSMEMBRANE_TRANSPORT	GOBP_ION_TRANSMEMBRANE_TRANSPORT	GOBP_ION_TRANSMEMBRANE_TRANSPORT	57	-0.416172229	-2.297654288	1.93E-05	0.0011011	0.0010300	170
HP_MANIA	HP_MANIA	HP_MANIA	12	-0.687913658	-2.289930208	1.93E-05	0.0011011	0.0010300	94
GOBP_CATION_TRANSMEMBRANE_TRANSPORT	GOBP_CATION_TRANSMEMBRANE_TRANSPORT	GOBP_CATION_TRANSMEMBRANE_TRANSPORT	52	-0.436885089	-2.350153731	2.11E-05	0.0011947	0.0011175	170
HP_BULBAR_SIGNS	HP_BULBAR_SIGNS	HP_BULBAR_SIGNS	11	-0.726696336	-2.357053325	2.22E-05	0.0012529	0.0011719	210
HP_FOCAL_ONSET_SEIZURE	HP_FOCAL_ONSET_SEIZURE	HP_FOCAL_ONSET_SEIZURE	36	-0.494250653	-2.382607034	2.27E-05	0.0012716	0.0011894	242
GOBP_PROGRAMMED_CELL_DEATH	GOBP_PROGRAMMED_CELL_DEATH	GOBP_PROGRAMMED_CELL_DEATH	186	0.210697528	1.984854075	2.28E-05	0.0012716	0.0011894	98
HP_ABNORMALITY_OF_CARDIOVASCULAR_SYSTEM_ELECTROPHYSIOLOGY	HP_ABNORMALITY_OF_CARDIOVASCULAR_SYSTEM_ELECTROPHYSIOLOGY	HP_ABNORMALITY_OF_CARDIOVASCULAR_SYSTEM_ELECTROPHYSIOLOGY	36	-0.493582383	-2.37938554	2.42E-05	0.0013423	0.0012556	94
HP_ABNORMAL_PERIPHERAL_MYELINATION	HP_ABNORMAL_PERIPHERAL_MYELINATION	HP_ABNORMAL_PERIPHERAL_MYELINATION	19	-0.616605071	-2.412501356	2.43E-05	0.0013423	0.0012556	125
HP_ABNORMAL_NEPHRON_MORPHOLOGY	HP_ABNORMAL_NEPHRON_MORPHOLOGY	HP_ABNORMAL_NEPHRON_MORPHOLOGY	22	-0.572650117	-2.347936957	2.46E-05	0.0013428	0.0012560	94
HP_ABNORMAL_RENAL_CORTEX_MORPHOLOGY	HP_ABNORMAL_RENAL_CORTEX_MORPHOLOGY	HP_ABNORMAL_RENAL_CORTEX_MORPHOLOGY	22	-0.57278348	-2.348483763	2.46E-05	0.0013428	0.0012560	94
GOBP_INORGANIC_ION_TRANSMEMBRANE_TRANSPORT	GOBP_INORGANIC_ION_TRANSMEMBRANE_TRANSPORT	GOBP_INORGANIC_ION_TRANSMEMBRANE_TRANSPORT	49	-0.440432098	-2.333778947	2.5E-05	0.0013531	0.0012657	170
GOBP_CELLULAR_RESPONSE_TO_HEAT	GOBP_CELLULAR_RESPONSE_TO_HEAT	GOBP_CELLULAR_RESPONSE_TO_HEAT	11	0.707238483	2.629448906	2.5E-05	0.0013531	0.0012657	20
HP_ABNORMAL_GASTROINTESTINAL_MOTILITY	HP_ABNORMAL_GASTROINTESTINAL_MOTILITY	HP_ABNORMAL_GASTROINTESTINAL_MOTILITY	13	-0.683963358	-2.344422333	2.54E-05	0.0013691	0.0012806	210
HP_ABNORMALITY_OF_TEMPERATURE_REGULATION	HP_ABNORMALITY_OF_TEMPERATURE_REGULATION	HP_ABNORMALITY_OF_TEMPERATURE_REGULATION	60	-0.414472579	-2.321603697	2.57E-05	0.0013744	0.0012856	167
HP_ABNORMALITY_OF_THE_MUSCULATURE_OF_THE_HAND	HP_ABNORMALITY_OF_THE_MUSCULATURE_OF_THE_HAND	HP_ABNORMALITY_OF_THE_MUSCULATURE_OF_THE_HAND	19	0.56110468	2.636699428	2.66E-05	0.00142	0.0013282	302
HP_DILATED_CARDIOMYOPATHY	HP_DILATED_CARDIOMYOPATHY	HP_DILATED_CARDIOMYOPATHY	20	-0.606677925	-2.417431814	2.77E-05	0.0014680	0.0013732	94
HP_ABNORMALITY_OF_THE_OPTIC_NERVE	HP_ABNORMALITY_OF_THE_OPTIC_NERVE	HP_ABNORMALITY_OF_THE_OPTIC_NERVE	74	-0.3766465	-2.20312471	2.95E-05	0.0015557	0.0014553	177
HP_MYOCLONUS	HP_MYOCLONUS	HP_MYOCLONUS	35	-0.483912177	-2.310057233	3.06E-05	0.0016062	0.0015024	320
HP_PERSONALITY_CHANGES	HP_PERSONALITY_CHANGES	HP_PERSONALITY_CHANGES	13	-0.679166584	-2.327980422	3.09E-05	0.0016153	0.0015110	94
HP_CEREBRAL_CORTICAL_ATROPHY	HP_CEREBRAL_CORTICAL_ATROPHY	HP_CEREBRAL_CORTICAL_ATROPHY	44	-0.45334393	-2.335618331	3.29E-05	0.0017140	0.0016033	120
GOCC_MEMBRANE_PROTEIN_COMPLEX	GOCC_MEMBRANE_PROTEIN_COMPLEX	GOCC_MEMBRANE_PROTEIN_COMPLEX	93	-0.349932105	-2.161955844	3.38E-05	0.0017516	0.0016385	94
HP_FUNCTIONAL_ABNORMALITY_OF_THE_GASTROINTESTINAL_TRACT	HP_FUNCTIONAL_ABNORMALITY_OF_THE_GASTROINTESTINAL_TRACT	HP_FUNCTIONAL_ABNORMALITY_OF_THE_GASTROINTESTINAL_TRACT	110	-0.344454127	-2.200773892	3.48E-05	0.0017874	0.0016720	431
HP_INCREASED_SERUM_LACTATE	HP_INCREASED_SERUM_LACTATE	HP_INCREASED_SERUM_LACTATE	23	-0.563647565	-2.36232435	3.48E-05	0.0017874	0.0016720	94
GOBP_PROTEIN_REFOLDING	GOBP_PROTEIN_REFOLDING	GOBP_PROTEIN_REFOLDING	10	0.723014157	2.540035352	3.59E-05	0.0018286	0.0017105	46
HP_MYOCLONIC_SEIZURE	HP_MYOCLONIC_SEIZURE	HP_MYOCLONIC_SEIZURE	26	-0.550134555	-2.352350149	3.6E-05	0.0018286	0.0017105	114
GOMF_CHAPERONE_BINDING	GOMF_CHAPERONE_BINDING	GOMF_CHAPERONE_BINDING	18	0.584906379	2.684359516	3.71E-05	0.0018783	0.0017570	46
HP_ABNORMAL_SUBARACHNOID_SPACE_MORPHOLOGY	HP_ABNORMAL_SUBARACHNOID_SPACE_MORPHOLOGY	HP_ABNORMAL_SUBARACHNOID_SPACE_MORPHOLOGY	18	-0.613171128	-2.381288534	3.85E-05	0.0019291	0.0018045	242
HP_WIDENED_SUBARACHNOID_SPACE	HP_WIDENED_SUBARACHNOID_SPACE	HP_WIDENED_SUBARACHNOID_SPACE	18	-0.613171128	-2.381288534	3.85E-05	0.0019291	0.0018045	242
HP_LEUKOPENIA	HP_LEUKOPENIA	HP_LEUKOPENIA	22	0.537133806	2.64703559	3.94E-05	0.0019669	0.0018399	311
HP_INCOMPLETE_PENETRANCE	HP_INCOMPLETE_PENETRANCE	HP_INCOMPLETE_PENETRANCE	17	-0.624045233	-2.373157631	4.17E-05	0.0020717	0.0019379	94
GOCC_MITOCHONDRIAL_PROTEIN_CONTAINING_COMPLEX	GOCC_MITOCHONDRIAL_PROTEIN_CONTAINING_COMPLEX	GOCC_MITOCHONDRIAL_PROTEIN_CONTAINING_COMPLEX	37	-0.479535634	-2.329652107	4.21E-05	0.0020800	0.0019457	94
GOBP_RESPONSE_TO_OXIDATIVE_STRESS	GOBP_RESPONSE_TO_OXIDATIVE_STRESS	GOBP_RESPONSE_TO_OXIDATIVE_STRESS	39	0.400899446	2.500305705	4.29E-05	0.0021127	0.0019763	42
GOBP_MITOCHONDRIAL_ELECTRON_TRANSPORT_NADH_TO_UBIQUINONE	GOBP_MITOCHONDRIAL_ELECTRON_TRANSPORT_NADH_TO_UBIQUINONE	GOBP_MITOCHONDRIAL_ELECTRON_TRANSPORT_NADH_TO_UBIQUINONE	13	-0.673918268	-2.309990761	4.44E-05	0.0021380	0.0019999	94
GOCC_NADH_DEHYDROGENASE_COMPLEX	GOCC_NADH_DEHYDROGENASE_COMPLEX	GOCC_NADH_DEHYDROGENASE_COMPLEX	13	-0.673918268	-2.309990761	4.44E-05	0.0021380	0.0019999	94
GOMF_NADH_DEHYDROGENASE_ACTIVITY	GOMF_NADH_DEHYDROGENASE_ACTIVITY	GOMF_NADH_DEHYDROGENASE_ACTIVITY	13	-0.673918268	-2.309990761	4.44E-05	0.0021380	0.0019999	94
GOMF_OXIDOREDUCTASE_ACTIVITY_ACTING_ON_NAD_P_H_QUINONE_OR_SIMILAR_CO	GOMF_OXIDOREDUCTASE_ACTIVITY_ACTING_ON_NAD_P_H_QUINONE_OR_SIMILAR_CO	GOMF_OXIDOREDUCTASE_ACTIVITY_ACTING_ON_NAD_P_H_QUINONE_OR_SIMILAR_CO	13	-0.673918268	-2.309990761	4.44E-05	0.0021380	0.0019999	94
HP_OPTIC_ATROPHY	HP_OPTIC_ATROPHY	HP_OPTIC_ATROPHY	53	-0.421481383	-2.291293026	4.44E-05	0.0021380	0.0019999	430
GOBP_REGULATION_OF_INTRINSIC_APOPTOTIC_SIGNALING_PATHWAY	GOBP_REGULATION_OF_INTRINSIC_APOPTOTIC_SIGNALING_PATHWAY	GOBP_REGULATION_OF_INTRINSIC_APOPTOTIC_SIGNALING_PATHWAY	26	0.486680887	2.587997835	4.58E-05	0.0021918	0.0020502	42
HP_ABNORMAL_RETINAL_VASCULAR_MORPHOLOGY	HP_ABNORMAL_RETINAL_VASCULAR_MORPHOLOGY	HP_ABNORMAL_RETINAL_VASCULAR_MORPHOLOGY	26	-0.545091585	-2.330786638	4.6E-05	0.0021918	0.0020502	94
GOBP_NUCLEOTIDE_BINDING_OLIGOMERIZATION_DOMAIN_CONTAINING_2_SIGNALING	GOBP_NUCLEOTIDE_BINDING_OLIGOMERIZATION_DOMAIN_CONTAINING_2_SIGNALING	GOBP_NUCLEOTIDE_BINDING_OLIGOMERIZATION_DOMAIN_CONTAINING_2_SIGNALING	3	0.980458794	2.00762713	5.67E-05	0.0026926	0.0025187	26
GOBP_CHAPERONE_MEDIATED_PROTEIN_COMPLEX_ASSEMBLY	GOBP_CHAPERONE_MEDIATED_PROTEIN_COMPLEX_ASSEMBLY	GOBP_CHAPERONE_MEDIATED_PROTEIN_COMPLEX_ASSEMBLY	6	0.850754417	2.383586355	6.35E-05	0.0030022	0.0028083	46
HP_ABNORMAL_CIRCULATING_AMINO_ACID_CONCENTRATION	HP_ABNORMAL_CIRCULATING_AMINO_ACID_CONCENTRATION	HP_ABNORMAL_CIRCULATING_AMINO_ACID_CONCENTRATION	13	-0.665927503	-2.282600803	6.46E-05	0.0030228	0.0028275	94
GOBP_REGULATION_OF_APOPTOTIC_SIGNALING_PATHWAY	GOBP_REGULATION_OF_APOPTOTIC_SIGNALING_PATHWAY	GOBP_REGULATION_OF_APOPTOTIC_SIGNALING_PATHWAY	47	0.378664593	2.506017878	6.47E-05	0.0030228	0.0028275	42
HP_MIGRAINE	HP_MIGRAINE	HP_MIGRAINE	17	-0.615127739	-2.339245636	6.48E-05	0.0030228	0.0028275	230
HP_ABNORMALITY_OF_ACID_BASE_HOMEOSTASIS	HP_ABNORMALITY_OF_ACID_BASE_HOMEOSTASIS	HP_ABNORMALITY_OF_ACID_BASE_HOMEOSTASIS	34	-0.489406791	-2.317974821	6.64E-05	0.0030873	0.0028879	94
HP_INCREASED_BLOOD_PRESSURE	HP_INCREASED_BLOOD_PRESSURE	HP_INCREASED_BLOOD_PRESSURE	24	-0.535135183	-2.242336058	6.7E-05	0.0030986	0.0028985	114
GOBP_POSITIVE_REGULATION_OF_TELOMERASE_ACTIVITY	GOBP_POSITIVE_REGULATION_OF_TELOMERASE_ACTIVITY	GOBP_POSITIVE_REGULATION_OF_TELOMERASE_ACTIVITY	6	0.847708435	2.375052327	6.8E-05	0.0031204	0.0029189	43
GOBP_REGULATION_OF_TELOMERASE_ACTIVITY	GOBP_REGULATION_OF_TELOMERASE_ACTIVITY	GOBP_REGULATION_OF_TELOMERASE_ACTIVITY	6	0.847708435	2.375052327	6.8E-05	0.0031204	0.0029189	43
GOBP_INTRINSIC_APOPTOTIC_SIGNALING_PATHWAY	GOBP_INTRINSIC_APOPTOTIC_SIGNALING_PATHWAY	GOBP_INTRINSIC_APOPTOTIC_SIGNALING_PATHWAY	39	0.394908144	2.462939507	6.94E-05	0.0031378	0.0029351	42
HP_CEREBRAL_CALCIFICATION	HP_CEREBRAL_CALCIFICATION	HP_CEREBRAL_CALCIFICATION	17	-0.613256433	-2.332129319	7.03E-05	0.0031378	0.0029351	253
GOBP_HEAT_ACCLIMATION	GOBP_HEAT_ACCLIMATION	GOBP_HEAT_ACCLIMATION	2	0.995755518	1.73829474	7.04E-05	0.0031378	0.0029351	7
GOBP_POSITIVE_REGULATION_OF_NUCLEOTIDE_BINDING_OLIGOMERIZATION_DOMAIN	GOBP_POSITIVE_REGULATION_OF_NUCLEOTIDE_BINDING_OLIGOMERIZATION_DOMAIN	GOBP_POSITIVE_REGULATION_OF_NUCLEOTIDE_BINDING_OLIGOMERIZATION_DOMAIN_	2	0.995755518	1.73829474	7.04E-05	0.0031378	0.0029351	7
GOBP_POSITIVE_REGULATION_OF_NUCLEOTIDE_BINDING_OLIGOMERIZATION_DOMAIN	GOBP_POSITIVE_REGULATION_OF_NUCLEOTIDE_BINDING_OLIGOMERIZATION_DOMAIN	GOBP_POSITIVE_REGULATION_OF_NUCLEOTIDE_BINDING_OLIGOMERIZATION_DOMAIN_	2	0.995755518	1.73829474	7.04E-05	0.0031378	0.0029351	7
GOBP_REGULATION_OF_MITOTIC_SPINDLE_ASSEMBLY	GOBP_REGULATION_OF_MITOTIC_SPINDLE_ASSEMBLY	GOBP_REGULATION_OF_MITOTIC_SPINDLE_ASSEMBLY	2	0.995755518	1.73829474	7.04E-05	0.0031378	0.0029351	7
GOBP_REGULATION_OF_NUCLEOTIDE_BINDING_OLIGOMERIZATION_DOMAIN_CONTAIN	GOBP_REGULATION_OF_NUCLEOTIDE_BINDING_OLIGOMERIZATION_DOMAIN_CONTAINI	GOBP_REGULATION_OF_NUCLEOTIDE_BINDING_OLIGOMERIZATION_DOMAIN_CONTAINI	2	0.995755518	1.73829474	7.04E-05	0.0031378	0.0029351	7
GOCC_TRANSCRIPTION_FACTOR_AP_1_COMPLEX	GOCC_TRANSCRIPTION_FACTOR_AP_1_COMPLEX	GOCC_TRANSCRIPTION_FACTOR_AP_1_COMPLEX	4	0.933673469	2.151849011	7.53E-05	0.0033405	0.0031247	82
HP_AUTISTIC_BEHAVIOR	HP_AUTISTIC_BEHAVIOR	HP_AUTISTIC_BEHAVIOR	79	-0.362831421	-2.148068217	7.82E-05	0.0034569	0.0032336	442
HP_ABNORMALITY_OF_THE_CEREBROSPINAL_FLUID	HP_ABNORMALITY_OF_THE_CEREBROSPINAL_FLUID	HP_ABNORMALITY_OF_THE_CEREBROSPINAL_FLUID	46	-0.426198584	-2.216255972	7.88E-05	0.0034709	0.0032467	262
GOMF_OXIDOREDUCTASE_ACTIVITY_ACTING_ON_NAD_P_H	GOMF_OXIDOREDUCTASE_ACTIVITY_ACTING_ON_NAD_P_H	GOMF_OXIDOREDUCTASE_ACTIVITY_ACTING_ON_NAD_P_H	14	-0.645010512	-2.273160617	8.13E-05	0.0035633	0.0033332	94
HP_INVOLUNTARY_MOVEMENTS	HP_INVOLUNTARY_MOVEMENTS	HP_INVOLUNTARY_MOVEMENTS	108	-0.328107017	-2.087853418	8.8E-05	0.0038417	0.0035936	243
HP_TELANGIECTASIA	HP_TELANGIECTASIA	HP_TELANGIECTASIA	27	-0.511630764	-2.215997185	9.13E-05	0.0039724	0.0037158	114
HP_CEREBRAL_VISUAL_IMPAIRMENT	HP_CEREBRAL_VISUAL_IMPAIRMENT	HP_CEREBRAL_VISUAL_IMPAIRMENT	34	-0.479986014	-2.273355246	9.69E-05	0.0041982	0.0039270	242
HP_ABNORMALITY_OF_PULMONARY_CIRCULATION	HP_ABNORMALITY_OF_PULMONARY_CIRCULATION	HP_ABNORMALITY_OF_PULMONARY_CIRCULATION	20	-0.581816667	-2.318367063	9.89E-05	0.0042691	0.0039933	211
GOCC_BLOOD_MICROPARTICLE	GOCC_BLOOD_MICROPARTICLE	GOCC_BLOOD_MICROPARTICLE	6	0.836779495	2.344432361	0.0001019	0.0043819	0.0040989	58
HP_UPPER_MOTOR_NEURON_DYSFUNCTION	HP_UPPER_MOTOR_NEURON_DYSFUNCTION	HP_UPPER_MOTOR_NEURON_DYSFUNCTION	105	-0.325159804	-2.067210555	0.0001023	0.0043819	0.0040989	483
HP_ABNORMALITY_OF_PANCREAS_PHYSIOLOGY	HP_ABNORMALITY_OF_PANCREAS_PHYSIOLOGY	HP_ABNORMALITY_OF_PANCREAS_PHYSIOLOGY	16	-0.601322742	-2.217366645	0.0001049	0.0044599	0.0041718	211
HP_HYPERTROPHIC_CARDIOMYOPATHY	HP_HYPERTROPHIC_CARDIOMYOPATHY	HP_HYPERTROPHIC_CARDIOMYOPATHY	23	-0.541583445	-2.269850595	0.0001049	0.0044599	0.0041718	222
HP_ABNORMAL_SCAPULA_MORPHOLOGY	HP_ABNORMAL_SCAPULA_MORPHOLOGY	HP_ABNORMAL_SCAPULA_MORPHOLOGY	19	0.532493706	2.502252964	0.0001076	0.0045203	0.0042283	309
HP_ABNORMAL_CORPUS_CALLOSUM_MORPHOLOGY	HP_ABNORMAL_CORPUS_CALLOSUM_MORPHOLOGY	HP_ABNORMAL_CORPUS_CALLOSUM_MORPHOLOGY	96	-0.335388008	-2.078737865	0.0001076	0.0045203	0.0042283	446
GOBP_PROTON_TRANSMEMBRANE_TRANSPORT	GOBP_PROTON_TRANSMEMBRANE_TRANSPORT	GOBP_PROTON_TRANSMEMBRANE_TRANSPORT	18	-0.590754842	-2.29423348	0.0001076	0.0045203	0.0042283	94
HP_EXERCISE_INTOLERANCE	HP_EXERCISE_INTOLERANCE	HP_EXERCISE_INTOLERANCE	15	-0.645296169	-2.310279606	0.0001084	0.0045360	0.0042431	94
HP_HETEROGENEOUS	HP_HETEROGENEOUS	HP_HETEROGENEOUS	24	-0.523646992	-2.194197973	0.0001122	0.0046729	0.0043711	253
GOBP_BIOLOGICAL_PROCESS_INVOLVED_IN_SYMBIOTIC_INTERACTION	GOBP_BIOLOGICAL_PROCESS_INVOLVED_IN_SYMBIOTIC_INTERACTION	GOBP_BIOLOGICAL_PROCESS_INVOLVED_IN_SYMBIOTIC_INTERACTION	34	0.410658455	2.423542528	0.0001125	0.0046729	0.0043711	46
GOBP_POSITIVE_REGULATION_OF_CELL_DEATH	GOBP_POSITIVE_REGULATION_OF_CELL_DEATH	GOBP_POSITIVE_REGULATION_OF_CELL_DEATH	61	0.320557686	2.33049597	0.0001138	0.0046897	0.0043868	107
HP_ABNORMALITY_OF_CENTRAL_NERVOUS_SYSTEM_ELECTROPHYSIOLOGY	HP_ABNORMALITY_OF_CENTRAL_NERVOUS_SYSTEM_ELECTROPHYSIOLOGY	HP_ABNORMALITY_OF_CENTRAL_NERVOUS_SYSTEM_ELECTROPHYSIOLOGY	53	-0.406486332	-2.209775653	0.0001143	0.0046897	0.0043868	173
HP_ROD_CONE_DYSTROPHY	HP_ROD_CONE_DYSTROPHY	HP_ROD_CONE_DYSTROPHY	13	-0.652221495	-2.235620696	0.0001146	0.0046897	0.0043868	94
HP_HYPOGONADOTROPIC_HYPOGONADISM	HP_HYPOGONADOTROPIC_HYPOGONADISM	HP_HYPOGONADOTROPIC_HYPOGONADISM	16	-0.597950719	-2.204932374	0.0001147	0.0046897	0.0043868	159
HP_CONSTIPATION	HP_CONSTIPATION	HP_CONSTIPATION	47	-0.432301325	-2.258651862	0.0001184	0.0048237	0.0045121	242
GOBP_PROTEIN_STABILIZATION	GOBP_PROTEIN_STABILIZATION	GOBP_PROTEIN_STABILIZATION	25	0.476492135	2.514880421	0.0001215	0.0049303	0.0046119	46
GOBP_MITOCHONDRIAL_ELECTRON_TRANSPORT_CYTOCHROME_C_TO_OXYGEN	GOBP_MITOCHONDRIAL_ELECTRON_TRANSPORT_CYTOCHROME_C_TO_OXYGEN	GOBP_MITOCHONDRIAL_ELECTRON_TRANSPORT_CYTOCHROME_C_TO_OXYGEN	4	-0.891182772	-1.994885815	0.0001273	0.0051493	0.0048168	25
GOBP_NEGATIVE_REGULATION_OF_TRANSCRIPTION_FROM_RNA_POLYMERASE_II_PRO	GOBP_NEGATIVE_REGULATION_OF_TRANSCRIPTION_FROM_RNA_POLYMERASE_II_PRO	GOBP_NEGATIVE_REGULATION_OF_TRANSCRIPTION_FROM_RNA_POLYMERASE_II_PRO	5	0.882838039	2.23551205	0.0001286	0.0051833	0.0048485	73
GOBP_REGULATION_OF_CELLULAR_RESPONSE_TO_STRESS	GOBP_REGULATION_OF_CELLULAR_RESPONSE_TO_STRESS	GOBP_REGULATION_OF_CELLULAR_RESPONSE_TO_STRESS	78	0.289082452	2.259615271	0.0001411	0.0056623	0.0052966	51
HP_APLASIA_OF_THE_FINGERS	HP_APLASIA_OF_THE_FINGERS	HP_APLASIA_OF_THE_FINGERS	17	0.566358989	2.509434516	0.0001418	0.0056727	0.0053063	274
HP_GENERALIZED_MYOCLONIC_SEIZURE	HP_GENERALIZED_MYOCLONIC_SEIZURE	HP_GENERALIZED_MYOCLONIC_SEIZURE	25	-0.525840669	-2.228627743	0.0001464	0.0058356	0.0054587	242
GOBP_REGULATION_OF_BONE_MINERALIZATION	GOBP_REGULATION_OF_BONE_MINERALIZATION	GOBP_REGULATION_OF_BONE_MINERALIZATION	4	-0.888605442	-1.98911654	0.0001472	0.0058441	0.0054667	136
GOBP_RESPONSE_TO_GROWTH_FACTOR	GOBP_RESPONSE_TO_GROWTH_FACTOR	GOBP_RESPONSE_TO_GROWTH_FACTOR	52	0.337720683	2.295005961	0.0001486	0.0058784	0.0054987	130

**GOBP_CELLULAR_RESPONSE_TO_UNFOLDED_PROTEIN**	GOBP_CELLULAR_RESPONSE_TO_UNFOLDED_PROTEIN	GOBP_CELLULAR_RESPONSE_TO_UNFOLDED_PROTEIN	25	0.472523982	2.493936886	0.0001506	0.0059363	0.0055529	46
**GOBP_APOPTOTIC_SIGNALING_PATHWAY**	GOBP_APOPTOTIC_SIGNALING_PATHWAY	GOBP_APOPTOTIC_SIGNALING_PATHWAY	67	0.303733559	2.282677205	0.0001544	0.0060633	0.0056717	42
**HP_ABNORMAL_VASCULAR_PHYSIOLOGY**	HP_ABNORMAL_VASCULAR_PHYSIOLOGY	HP_ABNORMAL_VASCULAR_PHYSIOLOGY	30	-0.494646088	-2.231425514	0.0001612	0.0063065	0.0058992	331
**GOMF_TRANSPORTER_ACTIVITY**	GOMF_TRANSPORTER_ACTIVITY	GOMF_TRANSPORTER_ACTIVITY	59	-0.391810237	-2.177671049	0.0001778	0.0069327	0.0064849	222
**HP_ABNORMAL_RETINAL_MORPHOLOGY**	HP_ABNORMAL_RETINAL_MORPHOLOGY	HP_ABNORMAL_RETINAL_MORPHOLOGY	85	-0.345455218	-2.095509343	0.0001790	0.0069532	0.0065041	483
**HP_GLUCOSE_INTOLERANCE**	HP_GLUCOSE_INTOLERANCE	HP_GLUCOSE_INTOLERANCE	41	-0.434494315	-2.186747896	0.0001871	0.0072409	0.0067733	161
**HP_ABNORMALITY_OF_THE_CEREBRAL_SUBCORTEX**	HP_ABNORMALITY_OF_THE_CEREBRAL_SUBCORTEX	HP_ABNORMALITY_OF_THE_CEREBRAL_SUBCORTEX	115	-0.310924768	-2.004495001	0.0001880	0.0072500	0.0067818	446
**GOBP_GENERATION_OF_PRECURSOR_METABOLITES_AND_ENERGY**	GOBP_GENERATION_OF_PRECURSOR_METABOLITES_AND_ENERGY	GOBP_GENERATION_OF_PRECURSOR_METABOLITES_AND_ENERGY	50	-0.401475387	-2.14658631	0.0001936	0.0074407	0.0069602	117
**HP_ABNORMAL_PERIPHERAL_NERVOUS_SYSTEM_MORPHOLOGY**	HP_ABNORMAL_PERIPHERAL_NERVOUS_SYSTEM_MORPHOLOGY	HP_ABNORMAL_PERIPHERAL_NERVOUS_SYSTEM_MORPHOLOGY	55	-0.390892915	-2.140455562	0.0001997	0.0076481	0.0071542	239
**HP_PSYCHOSIS**	HP_PSYCHOSIS	HP_PSYCHOSIS	13	-0.638755299	-2.189462592	0.0002030	0.0077379	0.0072382	94
**GOBP_POSITIVE_REGULATION_OF_MIRNA_TRANSCRIPTION**	GOBP_POSITIVE_REGULATION_OF_MIRNA_TRANSCRIPTION	GOBP_POSITIVE_REGULATION_OF_MIRNA_TRANSCRIPTION	8	0.735834953	2.356305094	0.0002035	0.0077379	0.0072382	75
**HP_DYSKINESIA**	HP_DYSKINESIA	HP_DYSKINESIA	31	-0.476769656	-2.175618148	0.0002073	0.0078470	0.0073402	320
**GOBP_REGULATION_OF_PROTEIN_STABILITY**	GOBP_REGULATION_OF_PROTEIN_STABILITY	GOBP_REGULATION_OF_PROTEIN_STABILITY	38	0.389770408	2.422392825	0.0002078	0.0078470	0.0073402	46
**GOBP_INCLUSION_BODY_ASSEMBLY**	GOBP_INCLUSION_BODY_ASSEMBLY	GOBP_INCLUSION_BODY_ASSEMBLY	4	0.916155944	2.111476149	0.0002171	0.0081152	0.0075911	7
**GOBP_NEGATIVE_REGULATION_OF_INCLUSION_BODY_ASSEMBLY**	GOBP_NEGATIVE_REGULATION_OF_INCLUSION_BODY_ASSEMBLY	GOBP_NEGATIVE_REGULATION_OF_INCLUSION_BODY_ASSEMBLY	4	0.916155944	2.111476149	0.0002171	0.0081152	0.0075911	7
**GOBP_REGULATION_OF_INCLUSION_BODY_ASSEMBLY**	GOBP_REGULATION_OF_INCLUSION_BODY_ASSEMBLY	GOBP_REGULATION_OF_INCLUSION_BODY_ASSEMBLY	4	0.916155944	2.111476149	0.0002171	0.0081152	0.0075911	7
**HP_ABNORMAL_GLUCOSE_HOMEOSTASIS**	HP_ABNORMAL_GLUCOSE_HOMEOSTASIS	HP_ABNORMAL_GLUCOSE_HOMEOSTASIS	53	-0.397327869	-2.159987633	0.0002210	0.0082314	0.0076998	161
**HP_STROKE**	HP_STROKE	HP_STROKE	20	-0.563912337	-2.247023613	0.0002279	0.0084512	0.0079053	271
**GOMF_HYDROLASE_ACTIVITY_ACTING_ON_ACID_ANHYDRIDES**	GOMF_HYDROLASE_ACTIVITY_ACTING_ON_ACID_ANHYDRIDES	GOMF_HYDROLASE_ACTIVITY_ACTING_ON_ACID_ANHYDRIDES	66	0.30560989	2.262936078	0.0002284	0.0084512	0.0079053	72
**HP_ABNORMAL_URINE_PROTEIN_LEVEL**	HP_ABNORMAL_URINE_PROTEIN_LEVEL	HP_ABNORMAL_URINE_PROTEIN_LEVEL	19	-0.566969834	-2.218300755	0.0002295	0.0084611	0.0079147	94
**HP_VASCULAR_SKIN_ABNORMALITY**	HP_VASCULAR_SKIN_ABNORMALITY	HP_VASCULAR_SKIN_ABNORMALITY	63	-0.370702635	-2.088459281	0.0002307	0.0084786	0.0079310	304
**GOBP_CYTOSKELETON_DEPENDENT_INTRACELLULAR_TRANSPORT**	GOBP_CYTOSKELETON_DEPENDENT_INTRACELLULAR_TRANSPORT	GOBP_CYTOSKELETON_DEPENDENT_INTRACELLULAR_TRANSPORT	13	0.61626165	2.456874287	0.0002328	0.0085270	0.0079762	30
**GOBP_TELOMERASE_HOLOENZYME_COMPLEX_ASSEMBLY**	GOBP_TELOMERASE_HOLOENZYME_COMPLEX_ASSEMBLY	GOBP_TELOMERASE_HOLOENZYME_COMPLEX_ASSEMBLY	3	0.966015293	1.978052034	0.0002348	0.0085716	0.0080180	43
**HP_DEMENTIA**	HP_DEMENTIA	HP_DEMENTIA	22	-0.530892488	-2.17672547	0.0002378	0.0086493	0.0080907	137
**HP_CHOREA**	HP_CHOREA	HP_CHOREA	34	-0.463132199	-2.193530607	0.0002438	0.0088385	0.0082677	242
**HP_DEPRESSION**	HP_DEPRESSION	HP_DEPRESSION	37	-0.445107862	-2.162397111	0.0002526	0.0091292	0.0085396	239
**HP_TYPE_I_DIABETES_MELLITUS**	HP_TYPE_I_DIABETES_MELLITUS	HP_TYPE_I_DIABETES_MELLITUS	15	-0.629607734	-2.254112107	0.0002545	0.0091441	0.0085535	114
**HP_RESPIRATORY_DISTRESS**	HP_RESPIRATORY_DISTRESS	HP_RESPIRATORY_DISTRESS	23	-0.520551306	-2.181702009	0.0002547	0.0091441	0.0085535	94
**GOBP_NADH_DEHYDROGENASE_COMPLEX_ASSEMBLY**	GOBP_NADH_DEHYDROGENASE_COMPLEX_ASSEMBLY	GOBP_NADH_DEHYDROGENASE_COMPLEX_ASSEMBLY	9	-0.713766142	-2.152023787	0.0002578	0.0092230	0.0086274	94
**HP_ABNORMALITY_OF_THE_PARATHYROID_GLAND**	HP_ABNORMALITY_OF_THE_PARATHYROID_GLAND	HP_ABNORMALITY_OF_THE_PARATHYROID_GLAND	16	-0.579392056	-2.13649764	0.0002665	0.0095030	0.0088893	94
**HP_HEMIPLEGIA_HEMIPARESIS**	HP_HEMIPLEGIA_HEMIPARESIS	HP_HEMIPLEGIA_HEMIPARESIS	22	-0.527538322	-2.162972968	0.0002697	0.0095858	0.0089667	94
**HP_HYPOPLASIA_OF_THE_CORPUS_CALLOSUM**	HP_HYPOPLASIA_OF_THE_CORPUS_CALLOSUM	HP_HYPOPLASIA_OF_THE_CORPUS_CALLOSUM	64	-0.376511318	-2.127058915	0.0002748	0.0097369	0.0091080	253
**GOCC_CYTOCHROME_COMPLEX**	GOCC_CYTOCHROME_COMPLEX	GOCC_CYTOCHROME_COMPLEX	6	-0.815156655	-2.112280369	0.0002935	0.0103665	0.0096969	49
**GOCC_FICOLIN_1_RICH_GRANULE_LUMEN**	GOCC_FICOLIN_1_RICH_GRANULE_LUMEN	GOCC_FICOLIN_1_RICH_GRANULE_LUMEN	18	0.530654867	2.435378536	0.0003015	0.0106111	0.0099257	55
**GOCC_CELL_SUBSTRATE_JUNCTION**	GOCC_CELL_SUBSTRATE_JUNCTION	GOCC_CELL_SUBSTRATE_JUNCTION	70	0.291766601	2.216223427	0.0003032	0.0106384	0.0099513	225
**GOMF_DISORDERED_DOMAIN_SPECIFIC_BINDING**	GOMF_DISORDERED_DOMAIN_SPECIFIC_BINDING	GOMF_DISORDERED_DOMAIN_SPECIFIC_BINDING	7	0.751130126	2.260333345	0.0003080	0.0107735	0.0100777	28
**HP_ABNORMALITY_OF_THE_THYROID_GLAND**	HP_ABNORMALITY_OF_THE_THYROID_GLAND	HP_ABNORMALITY_OF_THE_THYROID_GLAND	48	-0.404917396	-2.136759515	0.0003171	0.0110181	0.0103065	451
**HP_APNEA**	HP_APNEA	HP_APNEA	30	-0.4803057	-2.166733792	0.0003177	0.0110181	0.0103065	351
**GOBP_CELLULAR_RESPONSE_TO_TOPOLOGICALLY_INCORRECT_PROTEIN**	GOBP_CELLULAR_RESPONSE_TO_TOPOLOGICALLY_INCORRECT_PROTEIN	GOBP_CELLULAR_RESPONSE_TO_TOPOLOGICALLY_INCORRECT_PROTEIN	27	0.451634944	2.43794649	0.0003201	0.0110181	0.0103065	46
**GOMF_GLUTAMATE_RECEPTOR_BINDING**	GOMF_GLUTAMATE_RECEPTOR_BINDING	GOMF_GLUTAMATE_RECEPTOR_BINDING	2	-0.985568761	-1.674740687	0.0003201	0.0110181	0.0103065	20
**GOMF_IONOTROPIC_GLUTAMATE_RECEPTOR_BINDING**	GOMF_IONOTROPIC_GLUTAMATE_RECEPTOR_BINDING	GOMF_IONOTROPIC_GLUTAMATE_RECEPTOR_BINDING	2	-0.985568761	-1.674740687	0.0003201	0.0110181	0.0103065	20
**HP_ABNORMAL_CELLULAR_PHENOTYPE**	HP_ABNORMAL_CELLULAR_PHENOTYPE	HP_ABNORMAL_CELLULAR_PHENOTYPE	70	-0.359732268	-2.070141881	0.0003275	0.0112109	0.0104868	266
**HP_ATROPHY_DEGENERATION_AFFECTING_THE_CENTRAL_NERVOUS_SYSTEM**	HP_ATROPHY_DEGENERATION_AFFECTING_THE_CENTRAL_NERVOUS_SYSTEM	HP_ATROPHY_DEGENERATION_AFFECTING_THE_CENTRAL_NERVOUS_SYSTEM	93	-0.320152668	-1.977972075	0.0003278	0.0112109	0.0104868	365
**GOBP_REGULATION_OF_RESPONSE_TO_STRESS**	GOBP_REGULATION_OF_RESPONSE_TO_STRESS	GOBP_REGULATION_OF_RESPONSE_TO_STRESS	130	0.217828424	1.909431145	0.0003390	0.0115578	0.0108113	58
**GOMF_MISFOLDED_PROTEIN_BINDING**	GOMF_MISFOLDED_PROTEIN_BINDING	GOMF_MISFOLDED_PROTEIN_BINDING	9	0.709333846	2.383105266	0.0003548	0.0120214	0.0112450	7
**GOBP_POSITIVE_REGULATION_OF_NEURON_DIFFERENTIATION**	GOBP_POSITIVE_REGULATION_OF_NEURON_DIFFERENTIATION	GOBP_POSITIVE_REGULATION_OF_NEURON_DIFFERENTIATION	6	-0.810051107	-2.099050583	0.0003548	0.0120214	0.0112450	230
**GOBP_CATION_TRANSPORT**	GOBP_CATION_TRANSPORT	GOBP_CATION_TRANSPORT	73	-0.352084622	-2.055498676	0.0003617	0.0122189	0.0114298	131
**HP_DIARRHEA**	HP_DIARRHEA	HP_DIARRHEA	41	-0.420889351	-2.118276054	0.0003638	0.0122493	0.0114582	218
**GOMF_MHC_CLASS_II_PROTEIN_COMPLEX_BINDING**	GOMF_MHC_CLASS_II_PROTEIN_COMPLEX_BINDING	GOMF_MHC_CLASS_II_PROTEIN_COMPLEX_BINDING	6	0.80659997	2.259877402	0.0003691	0.0123508	0.0115531	49
**GOMF_MHC_PROTEIN_COMPLEX_BINDING**	GOMF_MHC_PROTEIN_COMPLEX_BINDING	GOMF_MHC_PROTEIN_COMPLEX_BINDING	6	0.80659997	2.259877402	0.0003691	0.0123508	0.0115531	49
**HP_ABNORMALITY_OF_THYROID_PHYSIOLOGY**	HP_ABNORMALITY_OF_THYROID_PHYSIOLOGY	HP_ABNORMALITY_OF_THYROID_PHYSIOLOGY	38	-0.4363741	-2.131801007	0.0003764	0.0125594	0.0117483	451
**HP_ABNORMAL_CSF_PROTEIN_LEVEL**	HP_ABNORMAL_CSF_PROTEIN_LEVEL	HP_ABNORMAL_CSF_PROTEIN_LEVEL	16	-0.570913873	-2.105234496	0.0003845	0.0127875	0.0119616	94
**HP_ABNORMAL_ESOPHAGUS_PHYSIOLOGY**	HP_ABNORMAL_ESOPHAGUS_PHYSIOLOGY	HP_ABNORMAL_ESOPHAGUS_PHYSIOLOGY	77	-0.346371865	-2.048672099	0.0003892	0.0129059	0.0120724	451
**GOBP_POSITIVE_REGULATION_OF_NITRIC_OXIDE_METABOLIC_PROCESS**	GOBP_POSITIVE_REGULATION_OF_NITRIC_OXIDE_METABOLIC_PROCESS	GOBP_POSITIVE_REGULATION_OF_NITRIC_OXIDE_METABOLIC_PROCESS	6	0.804372151	2.25363565	0.0003935	0.0130073	0.0121672	20
**GOBP_REGULATION_OF_CELL_CYCLE**	GOBP_REGULATION_OF_CELL_CYCLE	GOBP_REGULATION_OF_CELL_CYCLE	106	0.242779675	2.052245801	0.0003971	0.0130891	0.0122438	98
**HP_IMPAIRED_SOCIAL_INTERACTIONS**	HP_IMPAIRED_SOCIAL_INTERACTIONS	HP_IMPAIRED_SOCIAL_INTERACTIONS	14	-0.601779771	-2.120805862	0.0004119	0.0135343	0.0126602	298
**GOBP_MICROTUBULE_BASED_PROCESS**	GOBP_MICROTUBULE_BASED_PROCESS	GOBP_MICROTUBULE_BASED_PROCESS	49	0.327278273	2.180980118	0.0004365	0.0143009	0.0133773	30
**HP_DEVELOPMENTAL_REGRESSION**	HP_DEVELOPMENTAL_REGRESSION	HP_DEVELOPMENTAL_REGRESSION	40	-0.421899693	-2.126213818	0.0004380	0.0143048	0.0133809	423
**HP_SHORT_THUMB**	HP_SHORT_THUMB	HP_SHORT_THUMB	19	0.494716216	2.324731925	0.0004808	0.0156559	0.0146448	274
**HP_MENTAL_DETERIORATION**	HP_MENTAL_DETERIORATION	HP_MENTAL_DETERIORATION	31	-0.458252399	-2.091119312	0.0004855	0.0157638	0.0147457	137
**HP_BILATERAL_SENSORINEURAL_HEARING_IMPAIRMENT**	HP_BILATERAL_SENSORINEURAL_HEARING_IMPAIRMENT	HP_BILATERAL_SENSORINEURAL_HEARING_IMPAIRMENT	15	-0.609987972	-2.183869732	0.0005250	0.0169934	0.0158959	49
**GOMF_OXIDOREDUCTASE_ACTIVITY**	GOMF_OXIDOREDUCTASE_ACTIVITY	GOMF_OXIDOREDUCTASE_ACTIVITY	45	-0.406215467	-2.101104384	0.0005267	0.0169996	0.0159017	94
**GOBP_POSITIVE_REGULATION_OF_BIOMINERALIZATION**	GOBP_POSITIVE_REGULATION_OF_BIOMINERALIZATION	GOBP_POSITIVE_REGULATION_OF_BIOMINERALIZATION	3	-0.926083263	-1.844624638	0.0005451	0.0174601	0.0163324	91
**GOBP_POSITIVE_REGULATION_OF_BONE_MINERALIZATION**	GOBP_POSITIVE_REGULATION_OF_BONE_MINERALIZATION	GOBP_POSITIVE_REGULATION_OF_BONE_MINERALIZATION	3	-0.926083263	-1.844624638	0.0005451	0.0174601	0.0163324	91
**GOBP_REGULATION_OF_DNA_BIOSYNTHETIC_PROCESS**	GOBP_REGULATION_OF_DNA_BIOSYNTHETIC_PROCESS	GOBP_REGULATION_OF_DNA_BIOSYNTHETIC_PROCESS	10	0.648856138	2.279509348	0.0005458	0.0174601	0.0163324	43
**GOCC_MITOCHONDRION**	GOCC_MITOCHONDRION	GOCC_MITOCHONDRION	139	0.215777156	1.908429787	0.0005684	0.0179141	0.0167571	112
**GOMF_G_PROTEIN_COUPLED_RECEPTOR_BINDING**	GOMF_G_PROTEIN_COUPLED_RECEPTOR_BINDING	GOMF_G_PROTEIN_COUPLED_RECEPTOR_BINDING	15	0.563662081	2.378752875	0.0005709	0.0179141	0.0167571	17
**GOCC_DENDRITE_TERMINUS**	GOCC_DENDRITE_TERMINUS	GOCC_DENDRITE_TERMINUS	2	0.984719864	1.719029752	0.0005716	0.0179141	0.0167571	20
**GOCC_DENDRITIC_GROWTH_CONE**	GOCC_DENDRITIC_GROWTH_CONE	GOCC_DENDRITIC_GROWTH_CONE	2	0.984719864	1.719029752	0.0005716	0.0179141	0.0167571	20
**GOCC_SPERM_HEAD**	GOCC_SPERM_HEAD	GOCC_SPERM_HEAD	2	0.984719864	1.719029752	0.0005716	0.0179141	0.0167571	20
**GOMF_NITRIC_OXIDE_SYNTHASE_REGULATOR_ACTIVITY**	GOMF_NITRIC_OXIDE_SYNTHASE_REGULATOR_ACTIVITY	GOMF_NITRIC_OXIDE_SYNTHASE_REGULATOR_ACTIVITY	2	0.984719864	1.719029752	0.0005716	0.0179141	0.0167571	20
**GOMF_TPR_DOMAIN_BINDING**	GOMF_TPR_DOMAIN_BINDING	GOMF_TPR_DOMAIN_BINDING	2	0.984719864	1.719029752	0.0005716	0.0179141	0.0167571	20
**HP_ABNORMAL_FEAR_ANXIETY_RELATED_BEHAVIOR**	HP_ABNORMAL_FEAR_ANXIETY_RELATED_BEHAVIOR	HP_ABNORMAL_FEAR_ANXIETY_RELATED_BEHAVIOR	51	-0.394253164	-2.122814155	0.0005813	0.0181676	0.0169942	458
**HP_HEADACHE**	HP_HEADACHE	HP_HEADACHE	29	-0.475566419	-2.124305745	0.0005852	0.0182354	0.0170576	257
**GOBP_REGULATION_OF_PROTEOLYSIS**	GOBP_REGULATION_OF_PROTEOLYSIS	GOBP_REGULATION_OF_PROTEOLYSIS	73	0.278627929	2.12795412	0.0005992	0.0186181	0.0174156	46
**GOBP_RESPONSE_TO_ORGANIC_CYCLIC_COMPOUND**	GOBP_RESPONSE_TO_ORGANIC_CYCLIC_COMPOUND	GOBP_RESPONSE_TO_ORGANIC_CYCLIC_COMPOUND	78	0.264304744	2.065940123	0.0006118	0.0189564	0.0177321	82
**GOBP_POSITIVE_REGULATION_OF_DNA_BIOSYNTHETIC_PROCESS**	GOBP_POSITIVE_REGULATION_OF_DNA_BIOSYNTHETIC_PROCESS	GOBP_POSITIVE_REGULATION_OF_DNA_BIOSYNTHETIC_PROCESS	9	0.691769865	2.324096639	0.0006169	0.0190599	0.0178289	43
**HP_CONGESTIVE_HEART_FAILURE**	HP_CONGESTIVE_HEART_FAILURE	HP_CONGESTIVE_HEART_FAILURE	25	-0.490865561	-2.080395588	0.0006237	0.0192165	0.0179754	125
**HP_BRAIN_IMAGING_ABNORMALITY**	HP_BRAIN_IMAGING_ABNORMALITY	HP_BRAIN_IMAGING_ABNORMALITY	23	-0.49910353	-2.091811436	0.0006377	0.0195911	0.0183258	94
**GOBP_G_PROTEIN_COUPLED_PURINERGIC_NUCLEOTIDE_RECEPTOR_SIGNALING_PATH**	GOBP_G_PROTEIN_COUPLED_PURINERGIC_NUCLEOTIDE_RECEPTOR_SIGNALING_PATH	GOBP_G_PROTEIN_COUPLED_PURINERGIC_NUCLEOTIDE_RECEPTOR_SIGNALING_PATH	2	-0.977928693	-1.661758201	0.0006434	0.0196569	0.0183873	29
**GOMF_G_PROTEIN_COUPLED_PURINERGIC_NUCLEOTIDE_RECEPTOR_ACTIVITY**	GOMF_G_PROTEIN_COUPLED_PURINERGIC_NUCLEOTIDE_RECEPTOR_ACTIVITY	GOMF_G_PROTEIN_COUPLED_PURINERGIC_NUCLEOTIDE_RECEPTOR_ACTIVITY	2	-0.977928693	-1.661758201	0.0006434	0.0196569	0.0183873	29
**GOBP_TISSUE_DEVELOPMENT**	GOBP_TISSUE_DEVELOPMENT	GOBP_TISSUE_DEVELOPMENT	116	0.218266507	1.86804108	0.0006632	0.0202034	0.0188985	75
**GOBP_POSITIVE_REGULATION_OF_GENE_EXPRESSION**	GOBP_POSITIVE_REGULATION_OF_GENE_EXPRESSION	GOBP_POSITIVE_REGULATION_OF_GENE_EXPRESSION	127	0.213021645	1.876837901	0.0006711	0.0203867	0.0190700	83
**HP_ECTOPIC_CALCIFICATION**	HP_ECTOPIC_CALCIFICATION	HP_ECTOPIC_CALCIFICATION	21	-0.504711875	-2.034503977	0.0007007	0.0212260	0.0198551	253
**GOBP_REACTIVE_NITROGEN_SPECIES_METABOLIC_PROCESS**	GOBP_REACTIVE_NITROGEN_SPECIES_METABOLIC_PROCESS	GOBP_REACTIVE_NITROGEN_SPECIES_METABOLIC_PROCESS	8	0.692890442	2.218787341	0.0007108	0.0214127	0.0200298	20
**GOBP_REGULATION_OF_NITRIC_OXIDE_METABOLIC_PROCESS**	GOBP_REGULATION_OF_NITRIC_OXIDE_METABOLIC_PROCESS	GOBP_REGULATION_OF_NITRIC_OXIDE_METABOLIC_PROCESS	8	0.692890442	2.218787341	0.0007108	0.0214127	0.0200298	20
**GOBP_REGULATION_OF_CELL_POPULATION_PROLIFERATION**	GOBP_REGULATION_OF_CELL_POPULATION_PROLIFERATION	GOBP_REGULATION_OF_CELL_POPULATION_PROLIFERATION	125	0.21627814	1.907934071	0.0007137	0.0214415	0.0200567	135
**GOBP_NEGATIVE_REGULATION_OF_PROTEIN_METABOLIC_PROCESS**	GOBP_NEGATIVE_REGULATION_OF_PROTEIN_METABOLIC_PROCESS	GOBP_NEGATIVE_REGULATION_OF_PROTEIN_METABOLIC_PROCESS	111	0.226931747	1.926123982	0.0007543	0.0225987	0.0211391	57
**GOBP_ION_TRANSPORT**	GOBP_ION_TRANSPORT	GOBP_ION_TRANSPORT	82	-0.321999828	-1.931874623	0.0007668	0.0228490	0.0213733	170
**HP_SPASTICITY**	HP_SPASTICITY	HP_SPASTICITY	82	-0.321630427	-1.92965836	0.0007668	0.0228490	0.0213733	361
**HP_INCREASED_BODY_WEIGHT**	HP_INCREASED_BODY_WEIGHT	HP_INCREASED_BODY_WEIGHT	46	-0.382662473	-1.989865809	0.0007741	0.0230028	0.0215171	479
**GOMF_PROTON_TRANSMEMBRANE_TRANSPORTER_ACTIVITY**	GOMF_PROTON_TRANSMEMBRANE_TRANSPORTER_ACTIVITY	GOMF_PROTON_TRANSMEMBRANE_TRANSPORTER_ACTIVITY	16	-0.551854183	-2.034952239	0.0007860	0.0232282	0.0217280	61
**HP_FULL_CHEEKS**	HP_FULL_CHEEKS	HP_FULL_CHEEKS	16	-0.551850646	-2.034939195	0.0007860	0.0232282	0.0217280	164
**HP_IMPAIRMENT_IN_PERSONALITY_FUNCTIONING**	HP_IMPAIRMENT_IN_PERSONALITY_FUNCTIONING	HP_IMPAIRMENT_IN_PERSONALITY_FUNCTIONING	77	-0.335846791	-1.986419858	0.0007939	0.0233988	0.0218875	442
**HP_GAIT_ATAXIA**	HP_GAIT_ATAXIA	HP_GAIT_ATAXIA	31	-0.448424622	-2.046272732	0.0007996	0.0235043	0.0219862	143
**HP_ABNORMAL_NERVOUS_SYSTEM_ELECTROPHYSIOLOGY**	HP_ABNORMAL_NERVOUS_SYSTEM_ELECTROPHYSIOLOGY	HP_ABNORMAL_NERVOUS_SYSTEM_ELECTROPHYSIOLOGY	67	-0.352717307	-2.013053885	0.0008383	0.0245734	0.0229863	173
**HP_ABNORMALITY_OF_THE_VASCULATURE_OF_THE_EYE**	HP_ABNORMALITY_OF_THE_VASCULATURE_OF_THE_EYE	HP_ABNORMALITY_OF_THE_VASCULATURE_OF_THE_EYE	36	-0.424194226	-2.044889853	0.0008441	0.0246240	0.0230337	94
**HP_ATROPHY_DEGENERATION_AFFECTING_THE_CEREBRUM**	HP_ATROPHY_DEGENERATION_AFFECTING_THE_CEREBRUM	HP_ATROPHY_DEGENERATION_AFFECTING_THE_CEREBRUM	79	-0.326866742	-1.935146791	0.0008445	0.0246240	0.0230337	365
**GOBP_CELL_ADHESION_MEDIATED_BY_INTEGRIN**	GOBP_CELL_ADHESION_MEDIATED_BY_INTEGRIN	GOBP_CELL_ADHESION_MEDIATED_BY_INTEGRIN	4	-0.840152061	-1.880655105	0.0008728	0.0253788	0.0237397	76
**HP_ABNORMAL_CEREBRAL_CORTEX_MORPHOLOGY**	HP_ABNORMAL_CEREBRAL_CORTEX_MORPHOLOGY	HP_ABNORMAL_CEREBRAL_CORTEX_MORPHOLOGY	77	-0.334697866	-1.979624355	0.0008866	0.0257133	0.0240526	262
**HP_SPEECH_APRAXIA**	HP_SPEECH_APRAXIA	HP_SPEECH_APRAXIA	11	-0.636802578	-2.065481218	0.0009098	0.0263157	0.0246161	243
**HP_TREMOR**	HP_TREMOR	HP_TREMOR	51	-0.383934026	-2.067251852	0.0009287	0.0267782	0.0250487	162
**GOBP_NEGATIVE_REGULATION_OF_INTRINSIC_APOPTOTIC_SIGNALING_PATHWAY**	GOBP_NEGATIVE_REGULATION_OF_INTRINSIC_APOPTOTIC_SIGNALING_PATHWAY	GOBP_NEGATIVE_REGULATION_OF_INTRINSIC_APOPTOTIC_SIGNALING_PATHWAY	14	0.545920086	2.206750498	0.0009307	0.0267782	0.0250487	42
**HP_NEOPLASM_OF_THE_SKELETAL_SYSTEM**	HP_NEOPLASM_OF_THE_SKELETAL_SYSTEM	HP_NEOPLASM_OF_THE_SKELETAL_SYSTEM	20	0.474680075	2.269964741	0.0009351	0.0268319	0.0250990	274
**HP_DYSPHAGIA**	HP_DYSPHAGIA	HP_DYSPHAGIA	49	-0.384428066	-2.037022578	0.0009532	0.0272799	0.0255181	242
**HP_ABNORMAL_CEREBRAL_VASCULAR_MORPHOLOGY**	HP_ABNORMAL_CEREBRAL_VASCULAR_MORPHOLOGY	HP_ABNORMAL_CEREBRAL_VASCULAR_MORPHOLOGY	32	-0.439620782	-2.019394341	0.0009660	0.0275068	0.0257303	271
**HP_WEBBED_NECK**	HP_WEBBED_NECK	HP_WEBBED_NECK	23	0.441894712	2.214663659	0.0009668	0.0275068	0.0257303	274
**HP_ABNORMALITY_OF_THE_MUSCULATURE_OF_THE_UPPER_LIMBS**	HP_ABNORMALITY_OF_THE_MUSCULATURE_OF_THE_UPPER_LIMBS	HP_ABNORMALITY_OF_THE_MUSCULATURE_OF_THE_UPPER_LIMBS	26	0.418244363	2.22407646	0.0009687	0.0275068	0.0257303	302
**GOBP_MITOCHONDRIAL_RESPIRATORY_CHAIN_COMPLEX_ASSEMBLY**	GOBP_MITOCHONDRIAL_RESPIRATORY_CHAIN_COMPLEX_ASSEMBLY	GOBP_MITOCHONDRIAL_RESPIRATORY_CHAIN_COMPLEX_ASSEMBLY	10	-0.667495638	-2.107213761	0.0009808	0.0277283	0.0259374	94
**GOBP_POSITIVE_REGULATION_OF_TAU_PROTEIN_KINASE_ACTIVITY**	GOBP_POSITIVE_REGULATION_OF_TAU_PROTEIN_KINASE_ACTIVITY	GOBP_POSITIVE_REGULATION_OF_TAU_PROTEIN_KINASE_ACTIVITY	3	0.938827528	1.922381264	0.0009841	0.0277283	0.0259374	75
**GOBP_REGULATION_OF_TAU_PROTEIN_KINASE_ACTIVITY**	GOBP_REGULATION_OF_TAU_PROTEIN_KINASE_ACTIVITY	GOBP_REGULATION_OF_TAU_PROTEIN_KINASE_ACTIVITY	3	0.938827528	1.922381264	0.0009841	0.0277283	0.0259374	75
**GOBP_NEGATIVE_REGULATION_OF_APOPTOTIC_SIGNALING_PATHWAY**	GOBP_NEGATIVE_REGULATION_OF_APOPTOTIC_SIGNALING_PATHWAY	GOBP_NEGATIVE_REGULATION_OF_APOPTOTIC_SIGNALING_PATHWAY	26	0.4150483	2.207080921	0.0010297	0.0289373	0.0270684	42
**HP_ABNORMALITY_OF_HEPATOBILIARY_SYSTEM_PHYSIOLOGY**	HP_ABNORMALITY_OF_HEPATOBILIARY_SYSTEM_PHYSIOLOGY	HP_ABNORMALITY_OF_HEPATOBILIARY_SYSTEM_PHYSIOLOGY	26	-0.4764526	-2.037289485	0.0010759	0.0301558	0.0282082	114
**HP_ABNORMAL_SYSTEMIC_ARTERIAL_MORPHOLOGY**	HP_ABNORMAL_SYSTEMIC_ARTERIAL_MORPHOLOGY	HP_ABNORMAL_SYSTEMIC_ARTERIAL_MORPHOLOGY	68	-0.337308012	-1.928399229	0.0011385	0.0318296	0.0297739	177
**GOBP_CELL_CYCLE**	GOBP_CELL_CYCLE	GOBP_CELL_CYCLE	161	0.190247475	1.754204402	0.0011613	0.0323161	0.0302290	39
**GOBP_TRANSMEMBRANE_TRANSPORT**	GOBP_TRANSMEMBRANE_TRANSPORT	GOBP_TRANSMEMBRANE_TRANSPORT	75	-0.330725206	-1.941496403	0.0011618	0.0323161	0.0302290	94
**GOBP_CELL_MIGRATION_INVOLVED_IN_SPROUTING_ANGIOGENESIS**	GOBP_CELL_MIGRATION_INVOLVED_IN_SPROUTING_ANGIOGENESIS	GOBP_CELL_MIGRATION_INVOLVED_IN_SPROUTING_ANGIOGENESIS	2	0.978777589	1.708656296	0.0011716	0.0325031	0.0304038	27
**GOCC_FICOLIN_1_RICH_GRANULE**	GOCC_FICOLIN_1_RICH_GRANULE	GOCC_FICOLIN_1_RICH_GRANULE	25	0.425049246	2.243369719	0.0012232	0.0338491	0.0316629	76
**HP_SPECIFIC_LEARNING_DISABILITY**	HP_SPECIFIC_LEARNING_DISABILITY	HP_SPECIFIC_LEARNING_DISABILITY	28	-0.456412413	-2.00667876	0.0012432	0.0343149	0.0320987	125
**GOBP_DNA_BIOSYNTHETIC_PROCESS**	GOBP_DNA_BIOSYNTHETIC_PROCESS	GOBP_DNA_BIOSYNTHETIC_PROCESS	15	0.543627203	2.294202176	0.0012472	0.0343396	0.0321218	174
**GOBP_MICROTUBULE_BASED_TRANSPORT**	GOBP_MICROTUBULE_BASED_TRANSPORT	GOBP_MICROTUBULE_BASED_TRANSPORT	7	0.706754055	2.126794949	0.0012692	0.0347682	0.0325227	14
**GOBP_TRANSPORT_ALONG_MICROTUBULE**	GOBP_TRANSPORT_ALONG_MICROTUBULE	GOBP_TRANSPORT_ALONG_MICROTUBULE	7	0.706754055	2.126794949	0.0012692	0.0347682	0.0325227	14
**HP_ABNORMAL_INVOLUNTARY_EYE_MOVEMENTS**	HP_ABNORMAL_INVOLUNTARY_EYE_MOVEMENTS	HP_ABNORMAL_INVOLUNTARY_EYE_MOVEMENTS	83	-0.313681106	-1.889118879	0.0012815	0.0350174	0.0327558	437
**GOCC_INCLUSION_BODY**	GOCC_INCLUSION_BODY	GOCC_INCLUSION_BODY	11	0.596386146	2.217309912	0.0012870	0.0350802	0.0328146	42
**GOBP_BIOLOGICAL_PROCESS_INVOLVED_IN_INTERSPECIES_INTERACTION_BETWEEN_**	GOBP_BIOLOGICAL_PROCESS_INVOLVED_IN_INTERSPECIES_INTERACTION_BETWEEN_	GOBP_BIOLOGICAL_PROCESS_INVOLVED_IN_INTERSPECIES_INTERACTION_BETWEEN_	155	0.199180708	1.809384425	0.0012956	0.0352260	0.0329509	58
**GOBP_RIBOSOME_ASSEMBLY**	GOBP_RIBOSOME_ASSEMBLY	GOBP_RIBOSOME_ASSEMBLY	13	0.562275912	2.241647245	0.0013061	0.0354232	0.0331353	313
**GOBP_TUBE_DEVELOPMENT**	GOBP_TUBE_DEVELOPMENT	GOBP_TUBE_DEVELOPMENT	70	0.269279809	2.045416508	0.0013246	0.0358341	0.0335197	73
**GOCC_NUCLEAR_ENVELOPE_LUMEN**	GOCC_NUCLEAR_ENVELOPE_LUMEN	GOCC_NUCLEAR_ENVELOPE_LUMEN	2	0.977079796	1.705692452	0.0013535	0.0365270	0.0341679	29
**HP_ABNORMAL_SOCIAL_BEHAVIOR**	HP_ABNORMAL_SOCIAL_BEHAVIOR	HP_ABNORMAL_SOCIAL_BEHAVIOR	18	-0.526589645	-2.045043912	0.0013663	0.0367809	0.0344054	298
**GOMF_DNA_BINDING_TRANSCRIPTION_ACTIVATOR_ACTIVITY**	GOMF_DNA_BINDING_TRANSCRIPTION_ACTIVATOR_ACTIVITY	GOMF_DNA_BINDING_TRANSCRIPTION_ACTIVATOR_ACTIVITY	39	0.336866243	2.100947252	0.0013736	0.0368845	0.0345023	82
**HP_WEAKNESS_DUE_TO_UPPER_MOTOR_NEURON_DYSFUNCTION**	HP_WEAKNESS_DUE_TO_UPPER_MOTOR_NEURON_DYSFUNCTION	HP_WEAKNESS_DUE_TO_UPPER_MOTOR_NEURON_DYSFUNCTION	50	-0.366425287	-1.959182379	0.0014015	0.0375402	0.0351156	479
**GOBP_POTASSIUM_ION_TRANSPORT**	GOBP_POTASSIUM_ION_TRANSPORT	GOBP_POTASSIUM_ION_TRANSPORT	10	-0.653527206	-2.063116886	0.0014400	0.0384774	0.0359923	91
**GOBP_CELL_CELL_ADHESION_MEDIATED_BY_INTEGRIN**	GOBP_CELL_CELL_ADHESION_MEDIATED_BY_INTEGRIN	GOBP_CELL_CELL_ADHESION_MEDIATED_BY_INTEGRIN	2	-0.967741935	-1.64444822	0.0014463	0.0385521	0.0360622	41
**GOBP_POSITIVE_REGULATION_OF_SIGNAL_TRANSDUCTION_BY_P53_CLASS_MEDIATOR**	GOBP_POSITIVE_REGULATION_OF_SIGNAL_TRANSDUCTION_BY_P53_CLASS_MEDIATOF	GOBP_POSITIVE_REGULATION_OF_SIGNAL_TRANSDUCTION_BY_P53_CLASS_MEDIATOR	8	0.661709522	2.118939189	0.0014783	0.0393074	0.0367687	164
**HP_ABNORMAL_MYELINATION**	HP_ABNORMAL_MYELINATION	HP_ABNORMAL_MYELINATION	70	-0.330729828	-1.903242296	0.0015375	0.0407823	0.0381484	581
**GOBP_CELLULAR_RESPONSE_TO_REACTIVE_OXYGEN_SPECIES**	GOBP_CELLULAR_RESPONSE_TO_REACTIVE_OXYGEN_SPECIES	GOBP_CELLULAR_RESPONSE_TO_REACTIVE_OXYGEN_SPECIES	14	0.529812612	2.141639912	0.0015565	0.0411867	0.0385267	270
**HP_PUBERTY_AND_GONADAL_DISORDERS**	HP_PUBERTY_AND_GONADAL_DISORDERS	HP_PUBERTY_AND_GONADAL_DISORDERS	56	-0.34424357	-1.89319754	0.0015674	0.0413023	0.0386348	442
**GOCC_CHAPERONE_COMPLEX**	GOCC_CHAPERONE_COMPLEX	GOCC_CHAPERONE_COMPLEX	4	0.869123785	2.003080539	0.0015685	0.0413023	0.0386348	43
**GOBP_MYELOID_CELL_DIFFERENTIATION**	GOBP_MYELOID_CELL_DIFFERENTIATION	GOBP_MYELOID_CELL_DIFFERENTIATION	52	0.293525338	1.994673215	0.0016060	0.0421871	0.0394625	73

HP_INFANTILE_MUSCULAR_HYPOTONIA	HP_INFANTILE_MUSCULAR_HYPOTONIA	HP_INFANTILE_MUSCULAR_HYPOTONIA	24	-0.464077246	-1.944587419	0.0016243	0.0425659	0.0398167	164
HP_GASTROESOPHAGEAL_REFLUX	HP_GASTROESOPHAGEAL_REFLUX	HP_GASTROESOPHAGEAL_REFLUX	48	-0.367948947	-1.941676056	0.0016474	0.0430664	0.0402849	441
HP_POOR_EYE_CONTACT	HP_POOR_EYE_CONTACT	HP_POOR_EYE_CONTACT	10	-0.64815051	-2.046143219	0.0017196	0.0448466	0.0419502	239
HP_APRAXIA	HP_APRAXIA	HP_APRAXIA	18	-0.521677973	-2.025969125	0.0017307	0.0450285	0.0421203	304
GOBP_RESPONSE_TO_WOUNDING	GOBP_RESPONSE_TO_WOUNDING	GOBP_RESPONSE_TO_WOUNDING	37	0.352006853	2.144437645	0.0017564	0.0455875	0.0426432	58
HP_SHORT_ATTENTION_SPAN	HP_SHORT_ATTENTION_SPAN	HP_SHORT_ATTENTION_SPAN	64	-0.344971958	-1.948880798	0.0018020	0.0466611	0.0436474	177
GOBP_REGULATION_OF_ION_TRANSMEMBRANE_TRANSPORT	GOBP_REGULATION_OF_ION_TRANSMEMBRANE_TRANSPORT	GOBP_REGULATION_OF_ION_TRANSMEMBRANE_TRANSPORT	23	-0.475940583	-1.994732345	0.0018147	0.0468772	0.0438496	170
GOBP_REGENERATION	GOBP_REGENERATION	GOBP_REGENERATION	12	0.573144358	2.221542395	0.0018522	0.0477331	0.0446502	174
GOCC_RESPIRATORY_CHAIN_COMPLEX_IV	GOCC_RESPIRATORY_CHAIN_COMPLEX_IV	GOCC_RESPIRATORY_CHAIN_COMPLEX_IV	5	-0.799987301	-1.934929625	0.0019169	0.0492837	0.0461007	25
HP_COGNITIVE_IMPAIRMENT	HP_COGNITIVE_IMPAIRMENT	HP_COGNITIVE_IMPAIRMENT	46	-0.365942037	-1.902918628	0.0019235	0.0493356	0.0461492	239
GOBP_RESPONSE_TO_MECHANICAL_STIMULUS	GOBP_RESPONSE_TO_MECHANICAL_STIMULUS	GOBP_RESPONSE_TO_MECHANICAL_STIMULUS	16	0.484745274	2.102303351	0.0019497	0.0497822	0.0465669	82
HP_MOTOR_DELAY	HP_MOTOR_DELAY	HP_MOTOR_DELAY	76	-0.327371213	-1.922099895	0.0019500	0.0497822	0.0465669	253
GOMF_OXIDOREDUCTASE_ACTIVITY_ACTING_ON_A_HEME_GROUP_OF_DONORS	GOMF_OXIDOREDUCTASE_ACTIVITY_ACTING_ON_A_HEME_GROUP_OF_DONORS	GOMF_OXIDOREDUCTASE_ACTIVITY_ACTING_ON_A_HEME_GROUP_OF_DONORS	5	-0.798697011	-1.9318088	0.0020061	0.0510750	0.0477763	25
GOBP_POSITIVE_REGULATION_OF_OSTEOCLAST_DIFFERENTIATION	GOBP_POSITIVE_REGULATION_OF_OSTEOCLAST_DIFFERENTIATION	GOBP_POSITIVE_REGULATION_OF_OSTEOCLAST_DIFFERENTIATION	2	0.971137521	1.695318996	0.0020133	0.0510750	0.0477763	36
GOBP_SUPRAMOLECULAR_FIBER_ORGANIZATION	GOBP_SUPRAMOLECULAR_FIBER_ORGANIZATION	GOBP_SUPRAMOLECULAR_FIBER_ORGANIZATION	60	0.276646545	1.981894209	0.0020148	0.0510750	0.0477763	63
GOCC_RESPIRATORY_CHAIN_COMPLEX_III	GOCC_RESPIRATORY_CHAIN_COMPLEX_III	GOCC_RESPIRATORY_CHAIN_COMPLEX_III	2	-0.960950764	-1.632908232	0.0020456	0.0516800	0.0483422	49
GOCC_PERINUCLEAR_REGION_OF_CYTOPLASM	GOCC_PERINUCLEAR_REGION_OF_CYTOPLASM	GOCC_PERINUCLEAR_REGION_OF_CYTOPLASM	55	0.292611683	2.033085202	0.0020481	0.0516800	0.0483422	28
GOBP_REGULATION_OF_BIOMINERALIZATION	GOBP_REGULATION_OF_BIOMINERALIZATION	GOBP_REGULATION_OF_BIOMINERALIZATION	5	-0.797599422	-1.929154063	0.0020952	0.0527459	0.0493393	136
GOBP_NEGATIVE_REGULATION_OF_PROTEIN_MODIFICATION_PROCESS	GOBP_NEGATIVE_REGULATION_OF_PROTEIN_MODIFICATION_PROCESS	GOBP_NEGATIVE_REGULATION_OF_PROTEIN_MODIFICATION_PROCESS	58	0.28301771	2.0188828	0.0021512	0.0540305	0.0505409	31
GOBP_BIOLOGICAL_PROCESS_INVOLVED_IN_INTERACTION_WITH_SYMBIONT	GOBP_BIOLOGICAL_PROCESS_INVOLVED_IN_INTERACTION_WITH_SYMBIONT	GOBP_BIOLOGICAL_PROCESS_INVOLVED_IN_INTERACTION_WITH_SYMBIONT	17	0.49168477	2.178566525	0.0021599	0.0541226	0.0506271	46
GOMF_ATP_HYDROLYSIS_ACTIVITY	GOMF_ATP_HYDROLYSIS_ACTIVITY	GOMF_ATP_HYDROLYSIS_ACTIVITY	41	0.324707823	2.032402061	0.0021666	0.0541668	0.0506684	72
GOBP_MITOTIC_SPINDLE_ASSEMBLY	GOBP_MITOTIC_SPINDLE_ASSEMBLY	GOBP_MITOTIC_SPINDLE_ASSEMBLY	3	0.912772101	1.869029118	0.0021913	0.0546573	0.0511272	7
GOMF_INORGANIC_MOLECULAR_ENTITY_TRANSMEMBRANE_TRANSPORTER_ACTIVITY	GOMF_INORGANIC_MOLECULAR_ENTITY_TRANSMEMBRANE_TRANSPORTER_ACTIVITY	GOMF_INORGANIC_MOLECULAR_ENTITY_TRANSMEMBRANE_TRANSPORTER_ACTIVITY	30	-0.42998494	-1.939729011	0.0022747	0.0566079	0.0529518	162
GOBP_RESPONSE_TO_CYCLOHEXIMIDE	GOBP_RESPONSE_TO_CYCLOHEXIMIDE	GOBP_RESPONSE_TO_CYCLOHEXIMIDE	2	0.968590832	1.69087323	0.0022863	0.0567663	0.0531000	39
GOBP_ADAPTIVE_IMMUNE_RESPONSE	GOBP_ADAPTIVE_IMMUNE_RESPONSE	GOBP_ADAPTIVE_IMMUNE_RESPONSE	44	-0.370581154	-1.909226258	0.0023186	0.0574382	0.0537285	276
GOBP_INTERLEUKIN_8_PRODUCTION	GOBP_INTERLEUKIN_8_PRODUCTION	GOBP_INTERLEUKIN_8_PRODUCTION	7	0.682789056	2.054678434	0.0023536	0.0580832	0.0543318	16
HP_MEMORY_IMPAIRMENT	HP_MEMORY_IMPAIRMENT	HP_MEMORY_IMPAIRMENT	18	-0.512559852	-1.990558333	0.0023553	0.0580832	0.0543318	211
HP_ABNORMALITY_OF_COORDINATION	HP_ABNORMALITY_OF_COORDINATION	HP_ABNORMALITY_OF_COORDINATION	92	-0.298306969	-1.836702393	0.0023857	0.0586984	0.0549074	152
GOCC_AXONAL_GROWTH_CONE	GOCC_AXONAL_GROWTH_CONE	GOCC_AXONAL_GROWTH_CONE	3	0.909090909	1.86149136	0.0024045	0.0590276	0.0552153	110
GOBP_REGULATION_OF_NEURON_DIFFERENTIATION	GOBP_REGULATION_OF_NEURON_DIFFERENTIATION	GOBP_REGULATION_OF_NEURON_DIFFERENTIATION	12	-0.579133052	-1.927820817	0.0024503	0.0600168	0.0561406	259
HP_PERIPHERAL_AXONAL_DEGENERATION	HP_PERIPHERAL_AXONAL_DEGENERATION	HP_PERIPHERAL_AXONAL_DEGENERATION	24	-0.452520338	-1.896161388	0.0024560	0.0600204	0.0561439	159
GOMF_UBIQUITIN_PROTEIN_TRANSFERASE_REGULATOR_ACTIVITY	GOMF_UBIQUITIN_PROTEIN_TRANSFERASE_REGULATOR_ACTIVITY	GOMF_UBIQUITIN_PROTEIN_TRANSFERASE_REGULATOR_ACTIVITY	4	0.857142857	1.975467944	0.0024683	0.0601857	0.0562986	172
GOBP_CELLULAR_RESPONSE_TO_ACID_CHEMICAL	GOBP_CELLULAR_RESPONSE_TO_ACID_CHEMICAL	GOBP_CELLULAR_RESPONSE_TO_ACID_CHEMICAL	7	0.681229838	2.049986369	0.0025014	0.0606252	0.0567097	142
GOBP_FIBROBLAST_PROLIFERATION	GOBP_FIBROBLAST_PROLIFERATION	GOBP_FIBROBLAST_PROLIFERATION	7	0.680306698	2.047208415	0.0025014	0.0606252	0.0567097	55
HP_PERIPHERAL_NEUROPATHY	HP_PERIPHERAL_NEUROPATHY	HP_PERIPHERAL_NEUROPATHY	52	-0.360264797	-1.937987075	0.0025030	0.0606252	0.0567097	159
HP_HYDROPS_FETALIS	HP_HYDROPS_FETALIS	HP_HYDROPS_FETALIS	21	0.435979918	2.135259609	0.0025348	0.0612592	0.0573028	274
GOCC_CENTRIOLE	GOCC_CENTRIOLE	GOCC_CENTRIOLE	7	0.679563623	2.044972321	0.0025510	0.0615128	0.0575400	34
HP_ABNORMAL_CIRCULATING_CARBOXYLIC_ACID_CONCENTRATION	HP_ABNORMAL_CIRCULATING_CARBOXYLIC_ACID_CONCENTRATION	HP_ABNORMAL_CIRCULATING_CARBOXYLIC_ACID_CONCENTRATION	20	-0.499076707	-1.988672834	0.0025854	0.0622062	0.0581886	125
HP_ABNORMALITY_OF_SKIN_PIGMENTATION	HP_ABNORMALITY_OF_SKIN_PIGMENTATION	HP_ABNORMALITY_OF_SKIN_PIGMENTATION	51	-0.360561269	-1.941403735	0.0026384	0.0633408	0.0592499	125
GOCC_ORGANELLE_INNER_MEMBRANE	GOCC_ORGANELLE_INNER_MEMBRANE	GOCC_ORGANELLE_INNER_MEMBRANE	57	-0.333473059	-1.841078645	0.0026556	0.0636125	0.0595041	94
HP_SLEEP_DISTURBANCE	HP_SLEEP_DISTURBANCE	HP_SLEEP_DISTURBANCE	59	-0.342000422	-1.900829405	0.0026625	0.0636372	0.0595271	451
GOBP_SPINDLE_ASSEMBLY	GOBP_SPINDLE_ASSEMBLY	GOBP_SPINDLE_ASSEMBLY	9	0.6470419	2.17382685	0.0026731	0.0637518	0.0596343	92
GOBP_HINDBRAIN_DEVELOPMENT	GOBP_HINDBRAIN_DEVELOPMENT	GOBP_HINDBRAIN_DEVELOPMENT	14	-0.554738164	-1.955020767	0.0026922	0.0640668	0.0599290	351
GOBP_NEGATIVE_REGULATION_OF_B_CELL_APOPTOTIC_PROCESS	GOBP_NEGATIVE_REGULATION_OF_B_CELL_APOPTOTIC_PROCESS	GOBP_NEGATIVE_REGULATION_OF_B_CELL_APOPTOTIC_PROCESS	3	-0.86491079	-1.722777873	0.0027115	0.0642712	0.0601202	163
GOMF_DNA_POLYMERASE_BINDING	GOMF_DNA_POLYMERASE_BINDING	GOMF_DNA_POLYMERASE_BINDING	4	0.855442177	1.971548365	0.0027126	0.0642712	0.0601202	174
HP_PRIMARY_MICROCEPHALY	HP_PRIMARY_MICROCEPHALY	HP_PRIMARY_MICROCEPHALY	5	-0.789787234	-1.91025872	0.0027788	0.0656955	0.0614525	253
GOBP_REGULATION_OF_EXTRINSIC_APOPTOTIC_SIGNALING_PATHWAY	GOBP_REGULATION_OF_EXTRINSIC_APOPTOTIC_SIGNALING_PATHWAY	GOBP_REGULATION_OF_EXTRINSIC_APOPTOTIC_SIGNALING_PATHWAY	20	0.438228429	2.095649544	0.0028573	0.0672593	0.0629153	42
HP_APLASIA_HYPOPLASIA_OF_THE_THUMB	HP_APLASIA_HYPOPLASIA_OF_THE_THUMB	HP_APLASIA_HYPOPLASIA_OF_THE_THUMB	20	0.438624068	2.097541529	0.0028573	0.0672593	0.0629153	274
HP_FUNCTIONAL_ABNORMALITY_OF_THE_INNER_EAR	HP_FUNCTIONAL_ABNORMALITY_OF_THE_INNER_EAR	HP_FUNCTIONAL_ABNORMALITY_OF_THE_INNER_EAR	78	-0.30780534	-1.817705861	0.0029486	0.0691132	0.0646495	430
HP_POSTERIOR_UVEITIS	HP_POSTERIOR_UVEITIS	HP_POSTERIOR_UVEITIS	3	-0.861512319	-1.716008609	0.0029512	0.0691132	0.0646495	167
GOMF_PROTEIN_DOMAIN_SPECIFIC_BINDING	GOMF_PROTEIN_DOMAIN_SPECIFIC_BINDING	GOMF_PROTEIN_DOMAIN_SPECIFIC_BINDING	85	0.23137398	1.884476235	0.0029551	0.0691132	0.0646495	51
GOBP_POSITIVE_REGULATION_OF_WNT_SIGNALING_PATHWAY	GOBP_POSITIVE_REGULATION_OF_WNT_SIGNALING_PATHWAY	GOBP_POSITIVE_REGULATION_OF_WNT_SIGNALING_PATHWAY	13	-0.568325214	-1.948049273	0.0029795	0.0693858	0.0649044	250
HP_ACUTE_MYELOID_LEUKEMIA	HP_ACUTE_MYELOID_LEUKEMIA	HP_ACUTE_MYELOID_LEUKEMIA	20	0.43697879	2.089673652	0.0029796	0.0693858	0.0649044	277
GOCC_CELL_BODY	GOCC_CELL_BODY	GOCC_CELL_BODY	28	0.371183579	2.021675508	0.0029966	0.0696327	0.0651355	22
GOMF_CATION_TRANSMEMBRANE_TRANSPORTER_ACTIVITY	GOMF_CATION_TRANSMEMBRANE_TRANSPORTER_ACTIVITY	GOMF_CATION_TRANSMEMBRANE_TRANSPORTER_ACTIVITY	29	-0.431342813	-1.926763496	0.0030563	0.0708687	0.0662916	91
GOBP_NEGATIVE_REGULATION_OF_CELL_DEATH	GOBP_NEGATIVE_REGULATION_OF_CELL_DEATH	GOBP_NEGATIVE_REGULATION_OF_CELL_DEATH	92	0.23521834	1.948223913	0.0030641	0.0709002	0.0663210	46
HP_CROHN_S_DISEASE	HP_CROHN_S_DISEASE	HP_CROHN_S_DISEASE	4	-0.798473701	-1.78735935	0.0031109	0.0718300	0.0671908	76
GOBP_REGULATION_OF_MIRNA_TRANSCRIPTION	GOBP_REGULATION_OF_MIRNA_TRANSCRIPTION	GOBP_REGULATION_OF_MIRNA_TRANSCRIPTION	9	0.638962147	2.146681799	0.0031403	0.0723538	0.0676808	75
HP_OPTIC_NERVE_HYPOPLASIA	HP_OPTIC_NERVE_HYPOPLASIA	HP_OPTIC_NERVE_HYPOPLASIA	3	-0.856414613	-1.705854712	0.0031615	0.0726893	0.0679946	173
GOMF_ACTIVE_ION_TRANSMEMBRANE_TRANSPORTER_ACTIVITY	GOMF_ACTIVE_ION_TRANSMEMBRANE_TRANSPORTER_ACTIVITY	GOMF_ACTIVE_ION_TRANSMEMBRANE_TRANSPORTER_ACTIVITY	13	-0.563212229	-1.930523488	0.0032023	0.0730033	0.0682883	49
GOBP_BONE_RESORPTION	GOBP_BONE_RESORPTION	GOBP_BONE_RESORPTION	7	-0.678405839	-1.857885751	0.0032038	0.0730033	0.0682883	265
GOBP_ADENYLATE_CYCLASE_ACTIVATING_G_PROTEIN_COUPLED_RECEPTOR_SIGNALI	GOBP_ADENYLATE_CYCLASE_ACTIVATING_G_PROTEIN_COUPLED_RECEPTOR_SIGNALI	GOBP_ADENYLATE_CYCLASE_ACTIVATING_G_PROTEIN_COUPLED_RECEPTOR_SIGNALI	9	-0.641061039	-1.932815981	0.0032914	0.0730033	0.0682883	144
HP_TUBE_FEEDING	HP_TUBE_FEEDING	HP_TUBE_FEEDING	27	-0.433639396	-1.878197616	0.0033076	0.0730033	0.0682883	320
GOBP_RIBOSOMAL_SMALL_SUBUNIT_ASSEMBLY	GOBP_RIBOSOMAL_SMALL_SUBUNIT_ASSEMBLY	GOBP_RIBOSOMAL_SMALL_SUBUNIT_ASSEMBLY	8	0.633823069	2.029640641	0.0033207	0.0730033	0.0682883	274
GOMF_R_SMAD_BINDING	GOMF_R_SMAD_BINDING	GOMF_R_SMAD_BINDING	3	0.899660183	1.842180624	0.0033567	0.0730033	0.0682883	13
HP_HEPATIC_FAILURE	HP_HEPATIC_FAILURE	HP_HEPATIC_FAILURE	21	-0.468424203	-1.888227623	0.0033596	0.0730033	0.0682883	114
HP_DEEPLY_SET_EYE	HP_DEEPLY_SET_EYE	HP_DEEPLY_SET_EYE	24	-0.444339414	-1.861881486	0.0033692	0.0730033	0.0682883	441
GOBP_NEGATIVE_REGULATION_OF_DIGESTIVE_SYSTEM_PROCESS	GOBP_NEGATIVE_REGULATION_OF_DIGESTIVE_SYSTEM_PROCESS	GOBP_NEGATIVE_REGULATION_OF_DIGESTIVE_SYSTEM_PROCESS	1	-1	-1.326958239	0.0033699	0.0730033	0.0682883	2
GOBP_POSITIVE_REGULATION_OF_BLOOD_PRESSURE	GOBP_POSITIVE_REGULATION_OF_BLOOD_PRESSURE	GOBP_POSITIVE_REGULATION_OF_BLOOD_PRESSURE	1	-1	-1.326958239	0.0033699	0.0730033	0.0682883	2
GOBP_POSITIVE_REGULATION_OF_POTASSIUM_ION_IMPORT_ACROSS_PLASMA_MEMB	GOBP_POSITIVE_REGULATION_OF_POTASSIUM_ION_IMPORT_ACROSS_PLASMA_MEMB	GOBP_POSITIVE_REGULATION_OF_POTASSIUM_ION_IMPORT_ACROSS_PLASMA_MEMB	1	-1	-1.326958239	0.0033699	0.0730033	0.0682883	2
GOBP_POSITIVE_REGULATION_OF_SYSTEMIC_ARTERIAL_BLOOD_PRESSURE	GOBP_POSITIVE_REGULATION_OF_SYSTEMIC_ARTERIAL_BLOOD_PRESSURE	GOBP_POSITIVE_REGULATION_OF_SYSTEMIC_ARTERIAL_BLOOD_PRESSURE	1	-1	-1.326958239	0.0033699	0.0730033	0.0682883	2
GOBP_REGULATION_OF_CELL_CELL_ADHESION_MEDIATED_BY_INTEGRIN	GOBP_REGULATION_OF_CELL_CELL_ADHESION_MEDIATED_BY_INTEGRIN	GOBP_REGULATION_OF_CELL_CELL_ADHESION_MEDIATED_BY_INTEGRIN	1	-1	-1.326958239	0.0033699	0.0730033	0.0682883	2
GOBP_REGULATION_OF_PANCREATIC_JUICE_SECRETION	GOBP_REGULATION_OF_PANCREATIC_JUICE_SECRETION	GOBP_REGULATION_OF_PANCREATIC_JUICE_SECRETION	1	-1	-1.326958239	0.0033699	0.0730033	0.0682883	2
GOBP_REGULATION_OF_POTASSIUM_ION_IMPORT	GOBP_REGULATION_OF_POTASSIUM_ION_IMPORT	GOBP_REGULATION_OF_POTASSIUM_ION_IMPORT	1	-1	-1.326958239	0.0033699	0.0730033	0.0682883	2
GOMF_POTASSIUM_CHANNEL_INHIBITOR_ACTIVITY	GOMF_POTASSIUM_CHANNEL_INHIBITOR_ACTIVITY	GOMF_POTASSIUM_CHANNEL_INHIBITOR_ACTIVITY	1	-1	-1.326958239	0.0033699	0.0730033	0.0682883	2
HP_ABNORMALITY_OF_TASTE_SENSATION	HP_ABNORMALITY_OF_TASTE_SENSATION	HP_ABNORMALITY_OF_TASTE_SENSATION	1	-1	-1.326958239	0.0033699	0.0730033	0.0682883	2
HP_ABNORMAL_BLOOD_CHLORIDE_CONCENTRATION	HP_ABNORMAL_BLOOD_CHLORIDE_CONCENTRATION	HP_ABNORMAL_BLOOD_CHLORIDE_CONCENTRATION	1	-1	-1.326958239	0.0033699	0.0730033	0.0682883	2
HP_ACRAL_ULCERATION	HP_ACRAL_ULCERATION	HP_ACRAL_ULCERATION	1	-1	-1.326958239	0.0033699	0.0730033	0.0682883	2
HP_AUTOAMPUTATION	HP_AUTOAMPUTATION	HP_AUTOAMPUTATION	1	-1	-1.326958239	0.0033699	0.0730033	0.0682883	2
HP_AUTOAMPUTATION_OF_DIGITS	HP_AUTOAMPUTATION_OF_DIGITS	HP_AUTOAMPUTATION_OF_DIGITS	1	-1	-1.326958239	0.0033699	0.0730033	0.0682883	2
HP_DECREASED_CORNEAL_REFLEX	HP_DECREASED_CORNEAL_REFLEX	HP_DECREASED_CORNEAL_REFLEX	1	-1	-1.326958239	0.0033699	0.0730033	0.0682883	2
HP_FOOT_ACROOSTEOLYSIS	HP_FOOT_ACROOSTEOLYSIS	HP_FOOT_ACROOSTEOLYSIS	1	-1	-1.326958239	0.0033699	0.0730033	0.0682883	2
HP_HYPERCHLOREMIC_ACIDOSIS	HP_HYPERCHLOREMIC_ACIDOSIS	HP_HYPERCHLOREMIC_ACIDOSIS	1	-1	-1.326958239	0.0033699	0.0730033	0.0682883	2
HP_HYPERKALEMIA	HP_HYPERKALEMIA	HP_HYPERKALEMIA	1	-1	-1.326958239	0.0033699	0.0730033	0.0682883	2
HP_OSTEOLYSIS_INVOLVING_BONES_OF_THE_FEET	HP_OSTEOLYSIS_INVOLVING_BONES_OF_THE_FEET	HP_OSTEOLYSIS_INVOLVING_BONES_OF_THE_FEET	1	-1	-1.326958239	0.0033699	0.0730033	0.0682883	2
HP_OSTEOLYSIS_INVOLVING_BONES_OF_THE_UPPER_LIMBS	HP_OSTEOLYSIS_INVOLVING_BONES_OF_THE_UPPER_LIMBS	HP_OSTEOLYSIS_INVOLVING_BONES_OF_THE_UPPER_LIMBS	1	-1	-1.326958239	0.0033699	0.0730033	0.0682883	2
HP_PAINLESS_FRACTURES_DUE_TO_INJURY	HP_PAINLESS_FRACTURES_DUE_TO_INJURY	HP_PAINLESS_FRACTURES_DUE_TO_INJURY	1	-1	-1.326958239	0.0033699	0.0730033	0.0682883	2
HP_PSEUDOHYPOALDOSTERONISM	HP_PSEUDOHYPOALDOSTERONISM	HP_PSEUDOHYPOALDOSTERONISM	1	-1	-1.326958239	0.0033699	0.0730033	0.0682883	2
HP_ABNORMAL_PATTERN_OF_RESPIRATION	HP_ABNORMAL_PATTERN_OF_RESPIRATION	HP_ABNORMAL_PATTERN_OF_RESPIRATION	63	-0.32678703	-1.841048165	0.0033821	0.0731220	0.0683994	378
GOBP_REGULATION_OF_SPINDLE_ASSEMBLY	GOBP_REGULATION_OF_SPINDLE_ASSEMBLY	GOBP_REGULATION_OF_SPINDLE_ASSEMBLY	5	0.78017381	1.9755469	0.0034972	0.0754604	0.0705867	92
GOBP_POSITIVE_REGULATION_OF_B_CELL_DIFFERENTIATION	GOBP_POSITIVE_REGULATION_OF_B_CELL_DIFFERENTIATION	GOBP_POSITIVE_REGULATION_OF_B_CELL_DIFFERENTIATION	3	-0.852166525	-1.697393131	0.0035830	0.0771607	0.0721772	178
GOBP_POSITIVE_REGULATION_OF_MOLECULAR_FUNCTION	GOBP_POSITIVE_REGULATION_OF_MOLECULAR_FUNCTION	GOBP_POSITIVE_REGULATION_OF_MOLECULAR_FUNCTION	129	0.19143726	1.678518008	0.0035995	0.0773618	0.0723653	56
HP_FETAL_DISTRESS	HP_FETAL_DISTRESS	HP_FETAL_DISTRESS	7	-0.669572221	-1.833693959	0.0036153	0.0775483	0.0725398	222
GOBP_POSITIVE_REGULATION_OF_PROTEIN_POLYMERIZATION	GOBP_POSITIVE_REGULATION_OF_PROTEIN_POLYMERIZATION	GOBP_POSITIVE_REGULATION_OF_PROTEIN_POLYMERIZATION	10	0.587122888	2.062633045	0.0036262	0.0776285	0.0726148	19
HP_ABNORMAL_SYSTEMIC_BLOOD_PRESSURE	HP_ABNORMAL_SYSTEMIC_BLOOD_PRESSURE	HP_ABNORMAL_SYSTEMIC_BLOOD_PRESSURE	33	-0.403540263	-1.886788539	0.0036681	0.0783706	0.0733090	114
HP_HYPERTONIA	HP_HYPERTONIA	HP_HYPERTONIA	98	-0.273413326	-1.705442706	0.0037529	0.0800262	0.0748577	576
GOBP_NEGATIVE_REGULATION_OF_CELLULAR_COMPONENT_ORGANIZATION	GOBP_NEGATIVE_REGULATION_OF_CELLULAR_COMPONENT_ORGANIZATION	GOBP_NEGATIVE_REGULATION_OF_CELLULAR_COMPONENT_ORGANIZATION	57	0.277057907	1.956452237	0.0038401	0.0817244	0.0764462	42
GOBP_REGULATION_OF_RESPONSE_TO_CYTOKINE_STIMULUS	GOBP_REGULATION_OF_RESPONSE_TO_CYTOKINE_STIMULUS	GOBP_REGULATION_OF_RESPONSE_TO_CYTOKINE_STIMULUS	16	0.465089586	2.01705813	0.0038588	0.0819624	0.0766688	20
GOBP_NEURON_DEATH	GOBP_NEURON_DEATH	GOBP_NEURON_DEATH	27	0.383887051	2.072240204	0.0038942	0.0825545	0.0772227	98
GOBP_POSITIVE_REGULATION_OF_B_CELL_ACTIVATION	GOBP_POSITIVE_REGULATION_OF_B_CELL_ACTIVATION	GOBP_POSITIVE_REGULATION_OF_B_CELL_ACTIVATION	12	-0.566116082	-1.884489866	0.0039117	0.0827534	0.0774087	259
GOBP_LYSOSOMAL_TRANSPORT	GOBP_LYSOSOMAL_TRANSPORT	GOBP_LYSOSOMAL_TRANSPORT	9	0.62889898	2.112873197	0.0039188	0.0827534	0.0774087	23
HP_GASTROSTOMY_TUBE_FEEDING_IN_INFANCY	HP_GASTROSTOMY_TUBE_FEEDING_IN_INFANCY	HP_GASTROSTOMY_TUBE_FEEDING_IN_INFANCY	18	-0.494438671	-1.920183587	0.0039493	0.0832354	0.0778595	242
GOBP_REGULATION_OF_CATION_TRANSMEMBRANE_TRANSPORT	GOBP_REGULATION_OF_CATION_TRANSMEMBRANE_TRANSPORT	GOBP_REGULATION_OF_CATION_TRANSMEMBRANE_TRANSPORT	20	-0.486895531	-1.940134456	0.0039831	0.0837844	0.0783731	170
HP_DEVELOPMENTAL_CATARACT	HP_DEVELOPMENTAL_CATARACT	HP_DEVELOPMENTAL_CATARACT	28	-0.426234868	-1.873999109	0.0039919	0.0838082	0.0783954	49
GOBP_BLOOD_VESSEL_MORPHOGENESIS	GOBP_BLOOD_VESSEL_MORPHOGENESIS	GOBP_BLOOD_VESSEL_MORPHOGENESIS	50	0.285997173	1.915559023	0.0040074	0.0839709	0.0785476	73
GOBP_TISSUE_MIGRATION	GOBP_TISSUE_MIGRATION	GOBP_TISSUE_MIGRATION	27	0.382198089	2.063123105	0.0040209	0.0840917	0.0786606	42
GOBP_POSITIVE_REGULATION_OF_EPITHELIAL_CELL_MIGRATION	GOBP_POSITIVE_REGULATION_OF_EPITHELIAL_CELL_MIGRATION	GOBP_POSITIVE_REGULATION_OF_EPITHELIAL_CELL_MIGRATION	13	0.525128548	2.093550402	0.0041282	0.0861696	0.0806042	23
HP_DELAYED_ABILITY_TO_SIT	HP_DELAYED_ABILITY_TO_SIT	HP_DELAYED_ABILITY_TO_SIT	6	-0.718409707	-1.861584164	0.0041444	0.0863421	0.0807656	239
HP_THROMBOCYTOSIS	HP_THROMBOCYTOSIS	HP_THROMBOCYTOSIS	23	0.3938479	1.973865286	0.0041727	0.0867006	0.0811010	304
GOBP_PURINERGIC_NUCLEOTIDE_RECEPTOR_SIGNALING_PATHWAY	GOBP_PURINERGIC_NUCLEOTIDE_RECEPTOR_SIGNALING_PATHWAY	GOBP_PURINERGIC_NUCLEOTIDE_RECEPTOR_SIGNALING_PATHWAY	4	-0.788309755	-1.764607662	0.0041775	0.0867006	0.0811010	91
GOBP_REGULATION_OF_OSSIFICATION	GOBP_REGULATION_OF_OSSIFICATION	GOBP_REGULATION_OF_OSSIFICATION	8	-0.65797455	-1.88847953	0.0042417	0.0878657	0.0821908	136
GOBP_REGULATION_OF_SPINDLE_ORGANIZATION	GOBP_REGULATION_OF_SPINDLE_ORGANIZATION	GOBP_REGULATION_OF_SPINDLE_ORGANIZATION	7	0.652701058	1.964136326	0.0042846	0.0885852	0.0828639	92
GOBP_REGULATION_OF_ION_TRANSPORT	GOBP_REGULATION_OF_ION_TRANSPORT	GOBP_REGULATION_OF_ION_TRANSPORT	37	-0.380770751	-1.849838302	0.0043232	0.0892135	0.0834516	170
GOBP_IMMUNE_SYSTEM_DEVELOPMENT	GOBP_IMMUNE_SYSTEM_DEVELOPMENT	GOBP_IMMUNE_SYSTEM_DEVELOPMENT	111	0.204932301	1.739399728	0.0044250	0.0911404	0.0852541	75
HP_GASTROINTESTINAL_CARCINOMA	HP_GASTROINTESTINAL_CARCINOMA	HP_GASTROINTESTINAL_CARCINOMA	20	0.424239649	2.028753881	0.0045145	0.0928079	0.0868139	301
GOBP_REGULATION_OF_OXIDATIVE_STRESS_INDUCED_INTRINSIC_APOPTOTIC_SIGNAL	GOBP_REGULATION_OF_OXIDATIVE_STRESS_INDUCED_INTRINSIC_APOPTOTIC_SIGNAL	GOBP_REGULATION_OF_OXIDATIVE_STRESS_INDUCED_INTRINSIC_APOPTOTIC_SIGNAL	5	0.765957447	1.939548395	0.0045584	0.0935332	0.0874923	280
GOBP_POSITIVE_REGULATION_OF_RESPONSE_TO_CYTOKINE_STIMULUS	GOBP_POSITIVE_REGULATION_OF_RESPONSE_TO_CYTOKINE_STIMULUS	GOBP_POSITIVE_REGULATION_OF_RESPONSE_TO_CYTOKINE_STIMULUS	3	0.886647208	1.815534729	0.0045947	0.0939243	0.0878582	7
GOBP_POSITIVE_REGULATION_OF_TUMOR_NECROSIS_FACTOR_MEDIATED_SIGNALING_	GOBP_POSITIVE_REGULATION_OF_TUMOR_NECROSIS_FACTOR_MEDIATED_SIGNALING_	GOBP_POSITIVE_REGULATION_OF_TUMOR_NECROSIS_FACTOR_MEDIATED_SIGNALING_	3	0.886647208	1.815534729	0.0045947	0.0939243	0.0878582	7
GOMF_ION_TRANSMEMBRANE_TRANSPORTER_ACTIVITY	GOMF_ION_TRANSMEMBRANE_TRANSPORTER_ACTIVITY	GOMF_ION_TRANSMEMBRANE_TRANSPORTER_ACTIVITY	33	-0.395462498	-1.849020224	0.0046293	0.0944552	0.0883547	162
GOCC_NEURON_PROJECTION_MEMBRANE	GOCC_NEURON_PROJECTION_MEMBRANE	GOCC_NEURON_PROJECTION_MEMBRANE	3	-0.845369584	-1.683854601	0.0047272	0.0962713	0.0900536	186
HP_ABNORMALITY_OF_THE_PANCREAS	HP_ABNORMALITY_OF_THE_PANCREAS	HP_ABNORMALITY_OF_THE_PANCREAS	28	-0.423053413	-1.860011417	0.0048161	0.0977575	0.0914438	479
GOBP_POSITIVE_REGULATION_OF_I_KAPPAB_KINASE_NF_KAPPAB_SIGNALING	GOBP_POSITIVE_REGULATION_OF_I_KAPPAB_KINASE_NF_KAPPAB_SIGNALING	GOBP_POSITIVE_REGULATION_OF_I_KAPPAB_KINASE_NF_KAPPAB_SIGNALING	22	-0.457042907	-1.873932968	0.0048182	0.0977575	0.0914438	261
GOMF_RIBONUCLEOPROTEIN_COMPLEX_BINDING	GOMF_RIBONUCLEOPROTEIN_COMPLEX_BINDING	GOMF_RIBONUCLEOPROTEIN_COMPLEX_BINDING	19	0.424994463	1.997100893	0.0048408	0.0980347	0.0917031	603
HP_AGENESIS_OF_CORPUS_CALLOSUM	HP_AGENESIS_OF_CORPUS_CALLOSUM	HP_AGENESIS_OF_CORPUS_CALLOSUM	35	-0.391161625	-1.867292837	0.0049061	0.0991716	0.0927665	159
GOBP_RESPONSE_TO_CALCIUM_ION	GOBP_RESPONSE_TO_CALCIUM_ION	GOBP_RESPONSE_TO_CALCIUM_ION	11	0.542352922	2.016419257	0.0049837	0.1004487	0.0939612	82
HP_B_CELL_LYMPHOMA	HP_B_CELL_LYMPHOMA	HP_B_CELL_LYMPHOMA	5	-0.766641142	-1.854275258	0.0049878	0.1004487	0.0939612	167
GOMF_CADHERIN_BINDING	GOMF_CADHERIN_BINDING	GOMF_CADHERIN_BINDING	50	0.280989105	1.88201586	0.0050560	0.1016358	0.0950716	239
GOBP_POSITIVE_REGULATION_OF_PROTEIN_CONTAINING_COMPLEX_ASSEMBLY	GOBP_POSITIVE_REGULATION_OF_PROTEIN_CONTAINING_COMPLEX_ASSEMBLY	GOBP_POSITIVE_REGULATION_OF_PROTEIN_CONTAINING_COMPLEX_ASSEMBLY	19	0.423738273	1.991197901	0.0050907	0.1021434	0.0955464	34
GOBP_FC_EPSILON_RECEPTOR_SIGNALING_PATHWAY	GOBP_FC_EPSILON_RECEPTOR_SIGNALING_PATHWAY	GOBP_FC_EPSILON_RECEPTOR_SIGNALING_PATHWAY	2	-0.934634975	-1.588190781	0.0051008	0.1021576	0.0955597	80
HP_CONGENITAL_NYSTAGMUS	HP_CONGENITAL_NYSTAGMUS	HP_CONGENITAL_NYSTAGMUS	10	-0.617320842	-1.948817187	0.0051422	0.1027291	0.0960943	177
GOBP_GERMINAL_CENTER_FORMATION	GOBP_GERMINAL_CENTER_FORMATION	GOBP_GERMINAL_CENTER_FORMATION	3	-0.841971113	-1.677085336	0.0051487	0.1027291	0.0960943	190
GOBP_RESPONSE_TO_LIPID	GOBP_RESPONSE_TO_LIPID	GOBP_RESPONSE_TO_LIPID	75	0.237218524	1.818886453	0.0051577	0.1027291	0.0960943	57
GOBP_NUCLEOTIDE_BINDING_DOMAIN_LEUCINE_RICH_REPEAT_CONTAINING_RECEPT	GOBP_NUCLEOTIDE_BINDING_DOMAIN_LEUCINE_RICH_REPEAT_CONTAINING_RECEPT	GOBP_NUCLEOTIDE_BINDING_DOMAIN_LEUCINE_RICH_REPEAT_CONTAINING_RECEPT	7	0.641102989	1.929234913	0.0054258	0.1078731	0.1009060	26
GOCC_B_WICH_COMPLEX	GOCC_B_WICH_COMPLEX	GOCC_B_WICH_COMPLEX	2	-0.932937182	-1.585305784	0.0054718	0.1085873	0.1015741	82
GOCC_VACUOLAR_LUMEN	GOCC_VACUOLAR_LUMEN	GOCC_VACUOLAR_LUMEN	16	0.45353418	1.966943211	0.0055339	0.1096206	0.1025407	116
GOMF_G_PROTEIN_COUPLED_RECEPTOR_ACTIVITY	GOMF_G_PROTEIN_COUPLED_RECEPTOR_ACTIVITY	GOMF_G_PROTEIN_COUPLED_RECEPTOR_ACTIVITY	11	-0.572991651	-1.858509268	0.0055833	0.1103756	0.1032469	136
GOMF_DNA_BINDING_TRANSCRIPTION_FACTOR_BINDING	GOMF_DNA_BINDING_TRANSCRIPTION_FACTOR_BINDING	GOMF_DNA_BINDING_TRANSCRIPTION_FACTOR_BINDING	71	0.240871861	1.833129501	0.0055923	0.1103756	0.1032469	39
HP_ABNORMAL_BLOOD_GLUCOSE_CONCENTRATION	HP_ABNORMAL_BLOOD_GLUCOSE_CONCENTRATION	HP_ABNORMAL_BLOOD_GLUCOSE_CONCENTRATION	24	-0.430896256	-1.80555165	0.0056168	0.1106585	0.1035116	249
GOBP_CELLULAR_RESPONSE_TO_ORGANIC_CYCLIC_COMPOUND	GOBP_CELLULAR_RESPONSE_TO_ORGANIC_CYCLIC_COMPOUND	GOBP_CELLULAR_RESPONSE_TO_ORGANIC_CYCLIC_COMPOUND	54	0.274986458	1.879652044	0.0057091	0.1121704	0.1049258	75

**GOBP_REGULATION_OF_MICROTUBULE_POLYMERIZATION**	GOBP_REGULATION_OF_MICROTUBULE_POLYMERIZATION	GOBP_REGULATION_OF_MICROTUBULE_POLYMERIZATION	7	0.637654427	1.918857353	0.005724	0.112170	0.104925	7
**GOBP_REGULATION_OF_MICROTUBULE_POLYMERIZATION_OR_DEPOLYMERIZATION**	GOBP_REGULATION_OF_MICROTUBULE_POLYMERIZATION_OR_DEPOLYMERIZATION	GOBP_REGULATION_OF_MICROTUBULE_POLYMERIZATION_OR_DEPOLYMERIZATION	7	0.637654427	1.918857353	0.005724	0.112170	0.104925	7
**GOBP_NEGATIVE_REGULATION_OF_TRANSCRIPTION_BY_RNA_POLYMERASE_II**	GOBP_NEGATIVE_REGULATION_OF_TRANSCRIPTION_BY_RNA_POLYMERASE_II	GOBP_NEGATIVE_REGULATION_OF_TRANSCRIPTION_BY_RNA_POLYMERASE_II	89	0.222258553	1.790320548	0.005867	0.114763	0.107351	82
**GOBP_POSITIVE_REGULATION_OF_VASCULATURE_DEVELOPMENT**	GOBP_POSITIVE_REGULATION_OF_VASCULATURE_DEVELOPMENT	GOBP_POSITIVE_REGULATION_OF_VASCULATURE_DEVELOPMENT	10	0.563535693	1.979768404	0.005884	0.114816	0.107401	98
**GOCC_ORGANELLE_ENVELOPE_LUMEN**	GOCC_ORGANELLE_ENVELOPE_LUMEN	GOCC_ORGANELLE_ENVELOPE_LUMEN	9	0.613174922	2.060046049	0.005894	0.114816	0.107401	97
**GOBP_POSITIVE_REGULATION_OF_MYELOID_CELL_DIFFERENTIATION**	GOBP_POSITIVE_REGULATION_OF_MYELOID_CELL_DIFFERENTIATION	GOBP_POSITIVE_REGULATION_OF_MYELOID_CELL_DIFFERENTIATION	11	0.536338839	1.994059438	0.005901	0.114816	0.107401	36
**GOMF_FK506_BINDING**	GOMF_FK506_BINDING	GOMF_FK506_BINDING	4	0.827380952	1.906875307	0.005943	0.115211	0.107770	207
**GOMF_MACROLIDE_BINDING**	GOMF_MACROLIDE_BINDING	GOMF_MACROLIDE_BINDING	4	0.827380952	1.906875307	0.005943	0.115211	0.107770	207
**GOMF_ATP_DEPENDENT_ACTIVITY**	GOMF_ATP_DEPENDENT_ACTIVITY	GOMF_ATP_DEPENDENT_ACTIVITY	52	0.26312553	1.788089063	0.006027	0.116626	0.109093	72
**GOMF_CHONDROITIN_SULFATE_BINDING**	GOMF_CHONDROITIN_SULFATE_BINDING	GOMF_CHONDROITIN_SULFATE_BINDING	1	-0.999151824	-1.325832744	0.006039	0.116651	0.109117	3
**GOMF_PROTEIN_TAG**	GOMF_PROTEIN_TAG	GOMF_PROTEIN_TAG	4	0.825680272	1.902955728	0.006238	0.120281	0.112513	209
**GOBP_TRANSFORMING_GROWTH_FACTOR_BETA_RECEPTOR_SIGNALING_PATHWAY**	GOBP_TRANSFORMING_GROWTH_FACTOR_BETA_RECEPTOR_SIGNALING_PATHWAY	GOBP_TRANSFORMING_GROWTH_FACTOR_BETA_RECEPTOR_SIGNALING_PATHWAY	23	0.38132121	1.911084709	0.006309	0.121299	0.113465	20
**GOCC_CHROMOSOME**	GOCC_CHROMOSOME	GOCC_CHROMOSOME	170	-0.230743342	-1.606007399	0.006323	0.121299	0.113465	331
**GOBP_METENCEPHALON_DEVELOPMENT**	GOBP_METENCEPHALON_DEVELOPMENT	GOBP_METENCEPHALON_DEVELOPMENT	13	-0.542595816	-1.859856576	0.006324	0.121299	0.113465	351
**GOBP_POSITIVE_REGULATION_OF_INTRINSIC_APOPTOTIC_SIGNALING_PATHWAY_BY_P**	GOBP_POSITIVE_REGULATION_OF_INTRINSIC_APOPTOTIC_SIGNALING_PATHWAY_BY_P	GOBP_POSITIVE_REGULATION_OF_INTRINSIC_APOPTOTIC_SIGNALING_PATHWAY_BY_P	3	0.871707732	1.784944051	0.006363	0.121844	0.113975	154
**HP_AORTIC_ROOT_ANEURYSM**	HP_AORTIC_ROOT_ANEURYSM	HP_AORTIC_ROOT_ANEURYSM	10	-0.609275665	-1.923419408	0.006381	0.121973	0.114096	177
**GOBP_REGULATION_OF_LEUKOCYTE_MEDIATED_IMMUNITY**	GOBP_REGULATION_OF_LEUKOCYTE_MEDIATED_IMMUNITY	GOBP_REGULATION_OF_LEUKOCYTE_MEDIATED_IMMUNITY	23	-0.44351089	-1.858815047	0.006408	0.122259	0.114363	266
**HP_ARNOLD_CHIARI_TYPE_I_MALFORMATION**	HP_ARNOLD_CHIARI_TYPE_I_MALFORMATION	HP_ARNOLD_CHIARI_TYPE_I_MALFORMATION	5	-0.751489362	-1.81762764	0.006423	0.122332	0.114431	298
**GOBP_DECIDUALIZATION**	GOBP_DECIDUALIZATION	GOBP_DECIDUALIZATION	3	0.870858114	1.78320434	0.006459	0.122812	0.114880	155
**HP_HYPERTRICHOSIS**	HP_HYPERTRICHOSIS	HP_HYPERTRICHOSIS	43	-0.357757041	-1.816573792	0.006479	0.122970	0.115028	177
**GOBP_SKELETAL_MUSCLE_CELL_DIFFERENTIATION**	GOBP_SKELETAL_MUSCLE_CELL_DIFFERENTIATION	GOBP_SKELETAL_MUSCLE_CELL_DIFFERENTIATION	8	0.595650278	1.907402984	0.006574	0.124551	0.116507	75
**HP_RECURRENT_FEVER**	HP_RECURRENT_FEVER	HP_RECURRENT_FEVER	4	-0.766301928	-1.715343804	0.006609	0.125014	0.116940	125
**GOBP_NEGATIVE_REGULATION_OF_OXIDATIVE_STRESS_INDUCED_INTRINSIC_APOPTOT**	GOBP_NEGATIVE_REGULATION_OF_OXIDATIVE_STRESS_INDUCED_INTRINSIC_APOPTOT	GOBP_NEGATIVE_REGULATION_OF_OXIDATIVE_STRESS_INDUCED_INTRINSIC_APOPTOT	4	0.823897062	1.898845941	0.006630	0.125088	0.117009	98
**GOBP_EXPORT_ACROSS_PLASMA_MEMBRANE**	GOBP_EXPORT_ACROSS_PLASMA_MEMBRANE	GOBP_EXPORT_ACROSS_PLASMA_MEMBRANE	2	-0.925297114	-1.572323298	0.006648	0.125088	0.117009	91
**GOBP_METAL_ION_EXPORT**	GOBP_METAL_ION_EXPORT	GOBP_METAL_ION_EXPORT	2	-0.925297114	-1.572323298	0.006648	0.125088	0.117009	91
**GOBP_REGULATION_OF_DNA_BINDING**	GOBP_REGULATION_OF_DNA_BINDING	GOBP_REGULATION_OF_DNA_BINDING	16	0.444712291	1.928683352	0.006793	0.127409	0.119180	67
**HP_GAIT_DISTURBANCE**	HP_GAIT_DISTURBANCE	HP_GAIT_DISTURBANCE	113	-0.266381936	-1.710923122	0.006795	0.127409	0.119180	211
**GOBP_POSITIVE_REGULATION_OF_MICROTUBULE_POLYMERIZATION_OR_DEPOLYMERI**	GOBP_POSITIVE_REGULATION_OF_MICROTUBULE_POLYMERIZATION_OR_DEPOLYMERI	GOBP_POSITIVE_REGULATION_OF_MICROTUBULE_POLYMERIZATION_OR_DEPOLYMERI	6	0.692956257	1.94147811	0.006819	0.127497	0.119263	7
**GOMF_PROTEIN_DISAGGREGASE_ACTIVITY**	GOMF_PROTEIN_DISAGGREGASE_ACTIVITY	GOMF_PROTEIN_DISAGGREGASE_ACTIVITY	4	0.823142946	1.89710792	0.006827	0.127497	0.119263	7
**GOBP_RESPONSE_TO_MUSCLE_STRETCH**	GOBP_RESPONSE_TO_MUSCLE_STRETCH	GOBP_RESPONSE_TO_MUSCLE_STRETCH	5	0.747797134	1.893563066	0.006835	0.127497	0.119263	26
**GOBP_NEGATIVE_REGULATION_OF_PROTEIN_MODIFICATION_BY_SMALL_PROTEIN_CON**	GOBP_NEGATIVE_REGULATION_OF_PROTEIN_MODIFICATION_BY_SMALL_PROTEIN_CON	GOBP_NEGATIVE_REGULATION_OF_PROTEIN_MODIFICATION_BY_SMALL_PROTEIN_CON	15	0.47774012	2.016147125	0.006886	0.128233	0.119951	164
**GOBP_TUBE_MORPHOGENESIS**	GOBP_TUBE_MORPHOGENESIS	GOBP_TUBE_MORPHOGENESIS	61	0.24884473	1.809133478	0.006949	0.129192	0.120848	73
**GOBP_GRANULOCYTE_CHEMOTAXIS**	GOBP_GRANULOCYTE_CHEMOTAXIS	GOBP_GRANULOCYTE_CHEMOTAXIS	7	-0.642969825	-1.760840498	0.007030	0.130465	0.122038	76
**HP_ABNORMALITY_OF_THE_PULMONARY_ARTERY**	HP_ABNORMALITY_OF_THE_PULMONARY_ARTERY	HP_ABNORMALITY_OF_THE_PULMONARY_ARTERY	13	-0.53830556	-1.845150858	0.007107	0.131682	0.123177	177
**HP_HIGH_FOREHEAD**	HP_HIGH_FOREHEAD	HP_HIGH_FOREHEAD	29	-0.404778422	-1.808103121	0.007175	0.132704	0.124133	437
**HP_GASTRITIS**	HP_GASTRITIS	HP_GASTRITIS	3	-0.827527613	-1.648315961	0.007201	0.132817	0.124239	207
**GOBP_POSITIVE_REGULATION_OF_PROTEOLYSIS**	GOBP_POSITIVE_REGULATION_OF_PROTEOLYSIS	GOBP_POSITIVE_REGULATION_OF_PROTEOLYSIS	42	0.271965042	1.735479358	0.007205	0.132817	0.124239	46
**GOBP_REGULATION_OF_MYELOID_CELL_DIFFERENTIATION**	GOBP_REGULATION_OF_MYELOID_CELL_DIFFERENTIATION	GOBP_REGULATION_OF_MYELOID_CELL_DIFFERENTIATION	25	0.372185745	1.964361164	0.007368	0.135590	0.126833	57
**GOBP_EMBRYONIC_PROCESS_INVOLVED_IN_FEMALE_PREGNANCY**	GOBP_EMBRYONIC_PROCESS_INVOLVED_IN_FEMALE_PREGNANCY	GOBP_EMBRYONIC_PROCESS_INVOLVED_IN_FEMALE_PREGNANCY	2	0.939728353	1.640487875	0.007397	0.135897	0.127120	73
**GOMF_HISTONE_DEACETYLASE_BINDING**	GOMF_HISTONE_DEACETYLASE_BINDING	GOMF_HISTONE_DEACETYLASE_BINDING	15	0.475422869	2.006367918	0.007464	0.136899	0.128057	20
**HP_DUPLICATION_OF_HAND_BONES**	HP_DUPLICATION_OF_HAND_BONES	HP_DUPLICATION_OF_HAND_BONES	25	0.371808051	1.962367729	0.007496	0.137046	0.128195	309
**GOBP_NEGATIVE_REGULATION_OF_MITOCHONDRIAL_OUTER_MEMBRANE_PERMEABIL**	GOBP_NEGATIVE_REGULATION_OF_MITOCHONDRIAL_OUTER_MEMBRANE_PERMEABILI	GOBP_NEGATIVE_REGULATION_OF_MITOCHONDRIAL_OUTER_MEMBRANE_PERMEABILI	1	0.999151824	1.331775251	0.007498	0.137046	0.128195	2
**HP_PERIPHERAL_AXONAL_NEUROPATHY**	HP_PERIPHERAL_AXONAL_NEUROPATHY	HP_PERIPHERAL_AXONAL_NEUROPATHY	21	-0.439313454	-1.770881596	0.007603	0.138731	0.129771	94
**GOBP_POSITIVE_REGULATION_OF_CELL_PROLIFERATION_INVOLVED_IN_KIDNEY_DEVEL**	GOBP_POSITIVE_REGULATION_OF_CELL_PROLIFERATION_INVOLVED_IN_KIDNEY_DEVEL	GOBP_POSITIVE_REGULATION_OF_CELL_PROLIFERATION_INVOLVED_IN_KIDNEY_DEVEL	2	0.93803056	1.637524031	0.007673	0.139781	0.130753	75
**GOBP_MITOCHONDRIAL_MEMBRANE_ORGANIZATION**	GOBP_MITOCHONDRIAL_MEMBRANE_ORGANIZATION	GOBP_MITOCHONDRIAL_MEMBRANE_ORGANIZATION	15	0.473001708	1.996150196	0.007696	0.139791	0.130763	97
**GOMF_GUANYL_NUCLEOTIDE_BINDING**	GOMF_GUANYL_NUCLEOTIDE_BINDING	GOMF_GUANYL_NUCLEOTIDE_BINDING	27	0.361558831	1.951711432	0.007699	0.139791	0.130763	161
**GOBP_RESPONSE_TO_AXON_INJURY**	GOBP_RESPONSE_TO_AXON_INJURY	GOBP_RESPONSE_TO_AXON_INJURY	7	0.619286559	1.863583965	0.007715	0.139846	0.130814	131
**GOBP_NEGATIVE_REGULATION_OF_NUCLEOBASE_CONTAINING_COMPOUND_METABOL**	GOBP_NEGATIVE_REGULATION_OF_NUCLEOBASE_CONTAINING_COMPOUND_METABOL	GOBP_NEGATIVE_REGULATION_OF_NUCLEOBASE_CONTAINING_COMPOUND_METABOL	144	0.177831117	1.59455392	0.007775	0.140684	0.131598	111
**GOBP_BLOOD_VESSEL_ENDOTHELIAL_CELL_MIGRATION**	GOBP_BLOOD_VESSEL_ENDOTHELIAL_CELL_MIGRATION	GOBP_BLOOD_VESSEL_ENDOTHELIAL_CELL_MIGRATION	14	0.473621097	1.914499242	0.007787	0.140684	0.131598	98
**GOMF_HSP70_PROTEIN_BINDING**	GOMF_HSP70_PROTEIN_BINDING	GOMF_HSP70_PROTEIN_BINDING	5	0.741926267	1.878696925	0.007804	0.140753	0.131662	17
**GOBP_NEGATIVE_REGULATION_OF_CELL_POPULATION_PROLIFERATION**	GOBP_NEGATIVE_REGULATION_OF_CELL_POPULATION_PROLIFERATION	GOBP_NEGATIVE_REGULATION_OF_CELL_POPULATION_PROLIFERATION	57	0.262196895	1.851510781	0.007879	0.141875	0.132712	55
**HP_PHARYNGITIS**	HP_PHARYNGITIS	HP_PHARYNGITIS	3	-0.824129142	-1.641546697	0.007927	0.142416	0.133218	211
**GOBP_LEUKOCYTE_MEDIATED_IMMUNITY**	GOBP_LEUKOCYTE_MEDIATED_IMMUNITY	GOBP_LEUKOCYTE_MEDIATED_IMMUNITY	35	-0.377946412	-1.804207217	0.007939	0.142416	0.133218	266
**GOBP_MICROTUBULE_BASED_MOVEMENT**	GOBP_MICROTUBULE_BASED_MOVEMENT	GOBP_MICROTUBULE_BASED_MOVEMENT	13	0.490265219	1.954559413	0.007967	0.142416	0.133218	14
**GOCC_ION_CHANNEL_COMPLEX**	GOCC_ION_CHANNEL_COMPLEX	GOCC_ION_CHANNEL_COMPLEX	7	-0.634816771	-1.738512503	0.007975	0.142416	0.133218	157
**HP_BROAD_EYEBROW**	HP_BROAD_EYEBROW	HP_BROAD_EYEBROW	7	-0.6342711	-1.737018125	0.007975	0.142416	0.133218	437
**GOBP_NEURON_PROJECTION_REGENERATION**	GOBP_NEURON_PROJECTION_REGENERATION	GOBP_NEURON_PROJECTION_REGENERATION	6	0.685695182	1.921134521	0.008014	0.142878	0.133650	131
**GOBP_REGULATION_OF_MICROTUBULE_CYTOSKELETON_ORGANIZATION**	GOBP_REGULATION_OF_MICROTUBULE_CYTOSKELETON_ORGANIZATION	GOBP_REGULATION_OF_MICROTUBULE_CYTOSKELETON_ORGANIZATION	12	0.506795919	1.964371808	0.008128	0.144674	0.135330	110
**GOBP_REGULATION_OF_PROTEIN_CONTAINING_COMPLEX_ASSEMBLY**	GOBP_REGULATION_OF_PROTEIN_CONTAINING_COMPLEX_ASSEMBLY	GOBP_REGULATION_OF_PROTEIN_CONTAINING_COMPLEX_ASSEMBLY	40	0.276752358	1.712313952	0.008171	0.145209	0.135831	34
**GOBP_ATP_METABOLIC_PROCESS**	GOBP_ATP_METABOLIC_PROCESS	GOBP_ATP_METABOLIC_PROCESS	48	-0.333998236	-1.762517283	0.008232	0.145913	0.136489	117
**GOBP_RESPONSE_TO_XENOBIOTIC_STIMULUS**	GOBP_RESPONSE_TO_XENOBIOTIC_STIMULUS	GOBP_RESPONSE_TO_XENOBIOTIC_STIMULUS	23	0.373123845	1.870001603	0.008252	0.145913	0.136489	185
**GOBP_NEGATIVE_REGULATION_OF_SIGNAL_TRANSDUCTION_IN_ABSENCE_OF_LIGAND**	GOBP_NEGATIVE_REGULATION_OF_SIGNAL_TRANSDUCTION_IN_ABSENCE_OF_LIGAND	GOBP_NEGATIVE_REGULATION_OF_SIGNAL_TRANSDUCTION_IN_ABSENCE_OF_LIGAND	3	0.863281972	1.767691125	0.008277	0.145913	0.136489	7
**GOBP_REGULATION_OF_EXTRINSIC_APOPTOTIC_SIGNALING_PATHWAY_IN_ABSENCE_O**	GOBP_REGULATION_OF_EXTRINSIC_APOPTOTIC_SIGNALING_PATHWAY_IN_ABSENCE_C	GOBP_REGULATION_OF_EXTRINSIC_APOPTOTIC_SIGNALING_PATHWAY_IN_ABSENCE_C	3	0.863281972	1.767691125	0.008277	0.145913	0.136489	7
**GOBP_POSITIVE_REGULATION_OF_RNA_SPLICING**	GOBP_POSITIVE_REGULATION_OF_RNA_SPLICING	GOBP_POSITIVE_REGULATION_OF_RNA_SPLICING	11	0.522141267	1.941274148	0.008284	0.145913	0.136489	41
**GOBP_IMPORT_INTO_CELL**	GOBP_IMPORT_INTO_CELL	GOBP_IMPORT_INTO_CELL	10	-0.5962926	-1.882433231	0.008302	0.145913	0.136489	41
**HP_LANGUAGE_IMPAIRMENT**	HP_LANGUAGE_IMPAIRMENT	HP_LANGUAGE_IMPAIRMENT	97	-0.262773359	-1.635422276	0.008305	0.145913	0.136489	442
**HP_CLINODACTYLY**	HP_CLINODACTYLY	HP_CLINODACTYLY	57	-0.308103753	-1.701016693	0.008318	0.145913	0.136489	442
**GOBP_MULTI_MULTICELLULAR_ORGANISM_PROCESS**	GOBP_MULTI_MULTICELLULAR_ORGANISM_PROCESS	GOBP_MULTI_MULTICELLULAR_ORGANISM_PROCESS	14	0.468598159	1.894195225	0.008364	0.146008	0.136578	155
**GOBP_NEGATIVE_REGULATION_OF_UBIQUITIN_PROTEIN_LIGASE_ACTIVITY**	GOBP_NEGATIVE_REGULATION_OF_UBIQUITIN_PROTEIN_LIGASE_ACTIVITY	GOBP_NEGATIVE_REGULATION_OF_UBIQUITIN_PROTEIN_LIGASE_ACTIVITY	3	0.863211555	1.767546936	0.008373	0.146008	0.136578	164
**GOMF_UBIQUITIN_PROTEIN_TRANSFERASE_INHIBITOR_ACTIVITY**	GOMF_UBIQUITIN_PROTEIN_TRANSFERASE_INHIBITOR_ACTIVITY	GOMF_UBIQUITIN_PROTEIN_TRANSFERASE_INHIBITOR_ACTIVITY	3	0.863211555	1.767546936	0.008373	0.146008	0.136578	164
**HP_ABNORMAL_MYOCARDIUM_MORPHOLOGY**	HP_ABNORMAL_MYOCARDIUM_MORPHOLOGY	HP_ABNORMAL_MYOCARDIUM_MORPHOLOGY	38	-0.357063378	-1.744347494	0.008377	0.146008	0.136578	222
**GOBP_EXTRINSIC_APOPTOTIC_SIGNALING_PATHWAY**	GOBP_EXTRINSIC_APOPTOTIC_SIGNALING_PATHWAY	GOBP_EXTRINSIC_APOPTOTIC_SIGNALING_PATHWAY	27	0.356098256	1.92223499	0.008717	0.151449	0.141668	42
**GOBP_RIBOSOMAL_SMALL_SUBUNIT_BIOGENESIS**	GOBP_RIBOSOMAL_SMALL_SUBUNIT_BIOGENESIS	GOBP_RIBOSOMAL_SMALL_SUBUNIT_BIOGENESIS	22	0.384719473	1.895926351	0.008717	0.151449	0.141668	277
**GOBP_CELLULAR_RESPONSE_TO_CALCIUM_ION**	GOBP_CELLULAR_RESPONSE_TO_CALCIUM_ION	GOBP_CELLULAR_RESPONSE_TO_CALCIUM_ION	8	0.58154497	1.86223469	0.008782	0.152320	0.142482	82
**GOBP_SIGNAL_TRANSDUCTION_IN_ABSENCE_OF_LIGAND**	GOBP_SIGNAL_TRANSDUCTION_IN_ABSENCE_OF_LIGAND	GOBP_SIGNAL_TRANSDUCTION_IN_ABSENCE_OF_LIGAND	5	0.734197199	1.85912547	0.008870	0.153534	0.143618	7
**HP_GASTROINTESTINAL_INFLAMMATION**	HP_GASTROINTESTINAL_INFLAMMATION	HP_GASTROINTESTINAL_INFLAMMATION	12	-0.541383197	-1.802158923	0.008880	0.153534	0.143618	370
**GOMF_TRANSCRIPTION_REGULATOR_ACTIVITY**	GOMF_TRANSCRIPTION_REGULATOR_ACTIVITY	GOMF_TRANSCRIPTION_REGULATOR_ACTIVITY	141	0.176272452	1.586097242	0.009024	0.155771	0.145710	82
**GOBP_REGULATION_OF_PEPTIDASE_ACTIVITY**	GOBP_REGULATION_OF_PEPTIDASE_ACTIVITY	GOBP_REGULATION_OF_PEPTIDASE_ACTIVITY	41	0.288872699	1.808103861	0.009041	0.155831	0.145767	46
**HP_ABNORMAL_LATERAL_VENTRICLE_MORPHOLOGY**	HP_ABNORMAL_LATERAL_VENTRICLE_MORPHOLOGY	HP_ABNORMAL_LATERAL_VENTRICLE_MORPHOLOGY	18	-0.470684736	-1.827933694	0.009094	0.156270	0.146178	320
**GOBP_HISTONE_MODIFICATION**	GOBP_HISTONE_MODIFICATION	GOBP_HISTONE_MODIFICATION	64	-0.31431096	-1.775664893	0.009111	0.156270	0.146178	559
**GOBP_NEGATIVE_REGULATION_OF_MACROAUTOPHAGY**	GOBP_NEGATIVE_REGULATION_OF_MACROAUTOPHAGY	GOBP_NEGATIVE_REGULATION_OF_MACROAUTOPHAGY	2	-0.911714771	-1.549243323	0.009133	0.156270	0.146178	107
**GOBP_POSITIVE_REGULATION_OF_MICROTUBULE_NUCLEATION**	GOBP_POSITIVE_REGULATION_OF_MICROTUBULE_NUCLEATION	GOBP_POSITIVE_REGULATION_OF_MICROTUBULE_NUCLEATION	3	0.859747487	1.760453771	0.009139	0.156270	0.146178	7
**GOBP_REGULATION_OF_MICROTUBULE_NUCLEATION**	GOBP_REGULATION_OF_MICROTUBULE_NUCLEATION	GOBP_REGULATION_OF_MICROTUBULE_NUCLEATION	3	0.859747487	1.760453771	0.009139	0.156270	0.146178	7
**GOBP_AMEBOIDAL_TYPE_CELL_MIGRATION**	GOBP_AMEBOIDAL_TYPE_CELL_MIGRATION	GOBP_AMEBOIDAL_TYPE_CELL_MIGRATION	34	0.314703032	1.857251867	0.009258	0.157753	0.147564	42
**HP_HYPERREFLEXIA**	HP_HYPERREFLEXIA	HP_HYPERREFLEXIA	58	-0.314141214	-1.74565016	0.009280	0.157753	0.147564	304
**GOBP_MICROTUBULE_POLYMERIZATION**	GOBP_MICROTUBULE_POLYMERIZATION	GOBP_MICROTUBULE_POLYMERIZATION	8	0.578102768	1.851212003	0.009283	0.157753	0.147564	7
**GOBP_MICROTUBULE_POLYMERIZATION_OR_DEPOLYMERIZATION**	GOBP_MICROTUBULE_POLYMERIZATION_OR_DEPOLYMERIZATION	GOBP_MICROTUBULE_POLYMERIZATION_OR_DEPOLYMERIZATION	8	0.578102768	1.851212003	0.009283	0.157753	0.147564	7
**HP_NARROW_FACE**	HP_NARROW_FACE	HP_NARROW_FACE	14	-0.508822196	-1.793202676	0.009345	0.158121	0.147909	177
**HP_THORACIC_AORTIC_ANEURYSM**	HP_THORACIC_AORTIC_ANEURYSM	HP_THORACIC_AORTIC_ANEURYSM	14	-0.50868949	-1.79273499	0.009345	0.158121	0.147909	437
**GOBP_REGULATION_OF_SIGNALING_RECEPTOR_ACTIVITY**	GOBP_REGULATION_OF_SIGNALING_RECEPTOR_ACTIVITY	GOBP_REGULATION_OF_SIGNALING_RECEPTOR_ACTIVITY	4	-0.751505525	-1.682222502	0.009349	0.158121	0.147909	133
**GOBP_REGULATION_OF_CELLULAR_PROTEIN_CATABOLIC_PROCESS**	GOBP_REGULATION_OF_CELLULAR_PROTEIN_CATABOLIC_PROCESS	GOBP_REGULATION_OF_CELLULAR_PROTEIN_CATABOLIC_PROCESS	36	0.307283319	1.846391854	0.009402	0.158774	0.148519	42
**GOBP_INNATE_IMMUNE_RESPONSE_ACTIVATING_SIGNAL_TRANSDUCTION**	GOBP_INNATE_IMMUNE_RESPONSE_ACTIVATING_SIGNAL_TRANSDUCTION	GOBP_INNATE_IMMUNE_RESPONSE_ACTIVATING_SIGNAL_TRANSDUCTION	4	-0.75085034	-1.68075589	0.009468	0.159146	0.148867	298
**HP_ADDUCTED_THUMB**	HP_ADDUCTED_THUMB	HP_ADDUCTED_THUMB	4	-0.75085034	-1.68075589	0.009468	0.159146	0.148867	298
**HP_SYRINGOMYELIA**	HP_SYRINGOMYELIA	HP_SYRINGOMYELIA	4	-0.75085034	-1.68075589	0.009468	0.159146	0.148867	298
**HP_UVEITIS**	HP_UVEITIS	HP_UVEITIS	7	-0.625681196	-1.71349377	0.009629	0.161605	0.151167	325
**GOBP_REGULATION_OF_PROTEIN_MODIFICATION_BY_SMALL_PROTEIN_CONJUGATION**	GOBP_REGULATION_OF_PROTEIN_MODIFICATION_BY_SMALL_PROTEIN_CONJUGATION	GOBP_REGULATION_OF_PROTEIN_MODIFICATION_BY_SMALL_PROTEIN_CONJUGATION	48	0.266601126	1.757395014	0.009671	0.162055	0.151588	20
**GOBP_POSITIVE_REGULATION_OF_INTERFERON_GAMMA_PRODUCTION**	GOBP_POSITIVE_REGULATION_OF_INTERFERON_GAMMA_PRODUCTION	GOBP_POSITIVE_REGULATION_OF_INTERFERON_GAMMA_PRODUCTION	6	-0.687013675	-1.780228977	0.009865	0.165061	0.154400	211
**GOBP_CELLULAR_RESPONSE_TO_LAMINAR_FLUID_SHEAR_STRESS**	GOBP_CELLULAR_RESPONSE_TO_LAMINAR_FLUID_SHEAR_STRESS	GOBP_CELLULAR_RESPONSE_TO_LAMINAR_FLUID_SHEAR_STRESS	3	0.857092899	1.755018128	0.009905	0.165474	0.154787	16
**GOBP_REGULATION_OF_PROTEASOMAL_PROTEIN_CATABOLIC_PROCESS**	GOBP_REGULATION_OF_PROTEASOMAL_PROTEIN_CATABOLIC_PROCESS	GOBP_REGULATION_OF_PROTEASOMAL_PROTEIN_CATABOLIC_PROCESS	24	0.362929618	1.892491917	0.009922	0.165500	0.154811	42
**HP_WORMIAN_BONES**	HP_WORMIAN_BONES	HP_WORMIAN_BONES	3	-0.813914593	-1.621200784	0.009985	0.166294	0.155554	57
**GOCC_ANCHORING_JUNCTION**	GOCC_ANCHORING_JUNCTION	GOCC_ANCHORING_JUNCTION	89	0.214528556	1.728054452	0.010038	0.166925	0.156144	215
**GOBP_VACUOLAR_TRANSPORT**	GOBP_VACUOLAR_TRANSPORT	GOBP_VACUOLAR_TRANSPORT	13	0.483121079	1.926077593	0.010055	0.166952	0.156169	23
**GOBP_DNA_SYNTHESIS_INVOLVED_IN_DNA_REPAIR**	GOBP_DNA_SYNTHESIS_INVOLVED_IN_DNA_REPAIR	GOBP_DNA_SYNTHESIS_INVOLVED_IN_DNA_REPAIR	3	0.854715378	1.75014982	0.010098	0.167402	0.156590	174
**GOBP_REGULATION_OF_EPITHELIAL_CELL_MIGRATION**	GOBP_REGULATION_OF_EPITHELIAL_CELL_MIGRATION	GOBP_REGULATION_OF_EPITHELIAL_CELL_MIGRATION	22	0.382009307	1.882570451	0.010117	0.167475	0.156659	23
**HP_MEDIASTINAL_LYMPHADENOPATHY**	HP_MEDIASTINAL_LYMPHADENOPATHY	HP_MEDIASTINAL_LYMPHADENOPATHY	8	-0.622929309	-1.787894759	0.010171	0.168112	0.157254	259
**GOBP_CELLULAR_RESPONSE_TO_STEROID_HORMONE_STIMULUS**	GOBP_CELLULAR_RESPONSE_TO_STEROID_HORMONE_STIMULUS	GOBP_CELLULAR_RESPONSE_TO_STEROID_HORMONE_STIMULUS	27	0.349010136	1.88397299	0.010245	0.169067	0.158148	110
**HP_LOCALIZED_SKIN_LESION**	HP_LOCALIZED_SKIN_LESION	HP_LOCALIZED_SKIN_LESION	71	-0.294641865	-1.69909933	0.010354	0.170476	0.159466	464
**GOBP_TRANSMEMBRANE_RECEPTOR_PROTEIN_SERINE_THREONINE_KINASE_SIGNALI**	GOBP_TRANSMEMBRANE_RECEPTOR_PROTEIN_SERINE_THREONINE_KINASE_SIGNALI	GOBP_TRANSMEMBRANE_RECEPTOR_PROTEIN_SERINE_THREONINE_KINASE_SIGNALI	30	0.34215041	1.93621352	0.010361	0.170476	0.159466	111
**GOCC_NEURON_PROJECTION**	GOCC_NEURON_PROJECTION	GOCC_NEURON_PROJECTION	75	0.224445973	1.7209522	0.010543	0.173204	0.162017	20
**GOCC_AGGRESOME**	GOCC_AGGRESOME	GOCC_AGGRESOME	4	0.807074991	1.860075902	0.010563	0.173266	0.162076	7
**HP_ABNORMALITY_OF_THE_VOICE**	HP_ABNORMALITY_OF_THE_VOICE	HP_ABNORMALITY_OF_THE_VOICE	48	-0.326813549	-1.724603507	0.010601	0.173629	0.162415	177
**GOBP_ADENYLATE_CYCLASE_MODULATING_G_PROTEIN_COUPLED_RECEPTOR_SIGNA**	GOBP_ADENYLATE_CYCLASE_MODULATING_G_PROTEIN_COUPLED_RECEPTOR_SIGNA	GOBP_ADENYLATE_CYCLASE_MODULATING_G_PROTEIN_COUPLED_RECEPTOR_SIGNA	10	-0.590001463	-1.862572772	0.010674	0.174556	0.163283	144
**GOBP_NEGATIVE_REGULATION_OF_MOLECULAR_FUNCTION**	GOBP_NEGATIVE_REGULATION_OF_MOLECULAR_FUNCTION	GOBP_NEGATIVE_REGULATION_OF_MOLECULAR_FUNCTION	95	0.199540058	1.659589029	0.010736	0.174733	0.163448	67
**HP_ERUPTION_FAILURE**	HP_ERUPTION_FAILURE	HP_ERUPTION_FAILURE	2	-0.905772496	-1.539145834	0.010749	0.174733	0.163448	114
**HP_FAILURE_OF_ERUPTION_OF_PERMANENT_TEETH**	HP_FAILURE_OF_ERUPTION_OF_PERMANENT_TEETH	HP_FAILURE_OF_ERUPTION_OF_PERMANENT_TEETH	2	-0.905772496	-1.539145834	0.010749	0.174733	0.163448	114
**HP_ULCERATIVE_COLITIS**	HP_ULCERATIVE_COLITIS	HP_ULCERATIVE_COLITIS	2	-0.905772496	-1.539145834	0.010749	0.174733	0.163448	114
**GOBP_SPROUTING_ANGIOGENESIS**	GOBP_SPROUTING_ANGIOGENESIS	GOBP_SPROUTING_ANGIOGENESIS	5	0.721388021	1.826690222	0.010814	0.175537	0.164200	27
**GOCC_PROTON_TRANSPORTING_ATP_SYNTHASE_COMPLEX_COUPLING_FACTOR_F_O**	GOCC_PROTON_TRANSPORTING_ATP_SYNTHASE_COMPLEX_COUPLING_FACTOR_F_O	GOCC_PROTON_TRANSPORTING_ATP_SYNTHASE_COMPLEX_COUPLING_FACTOR_F_O	3	-0.806449817	-1.606332024	0.010832	0.175564	0.164226	61
**HP_SKIN_NODULE**	HP_SKIN_NODULE	HP_SKIN_NODULE	9	-0.599487617	-1.807471015	0.010872	0.175942	0.164579	479
**HP_CLEFT_SOFT_PALATE**	HP_CLEFT_SOFT_PALATE	HP_CLEFT_SOFT_PALATE	24	0.358833956	1.871135138	0.010933	0.176675	0.165264	279
**HP_GENERALIZED_ABNORMALITY_OF_SKIN**	HP_GENERALIZED_ABNORMALITY_OF_SKIN	HP_GENERALIZED_ABNORMALITY_OF_SKIN	91	-0.274058014	-1.681059926	0.010989	0.177319	0.165867	351
**GOBP_REGULATION_OF_CELL_ADHESION_MEDIATED_BY_INTEGRIN**	GOBP_REGULATION_OF_CELL_ADHESION_MEDIATED_BY_INTEGRIN	GOBP_REGULATION_OF_CELL_ADHESION_MEDIATED_BY_INTEGRIN	3	-0.805990008	-1.605416152	0.011074	0.178160	0.166654	76
**GOBP_RESPONSE_TO_VITAMIN_D**	GOBP_RESPONSE_TO_VITAMIN_D	GOBP_RESPONSE_TO_VITAMIN_D	3	-0.805973625	-1.605383519	0.011074	0.178160	0.166654	34
**GOMF_RNA_POLYMERASE_II_SPECIFIC_DNA_BINDING_TRANSCRIPTION_FACTOR_BINDI**	GOMF_RNA_POLYMERASE_II_SPECIFIC_DNA_BINDING_TRANSCRIPTION_FACTOR_BINDI	GOMF_RNA_POLYMERASE_II_SPECIFIC_DNA_BINDING_TRANSCRIPTION_FACTOR_BINDI	54	0.261053249	1.7844125	0.011158	0.179237	0.167661	27
**HP_ABNORMAL_UVEA_MORPHOLOGY**	HP_ABNORMAL_UVEA_MORPHOLOGY	HP_ABNORMAL_UVEA_MORPHOLOGY	39	-0.352061079	-1.742123512	0.011251	0.180464	0.168809	451
**GOBP_TRANSCRIPTION_BY_RNA_POLYMERASE_III**	GOBP_TRANSCRIPTION_BY_RNA_POLYMERASE_III	GOBP_TRANSCRIPTION_BY_RNA_POLYMERASE_III	7	-0.617900297	-1.69218496	0.011401	0.182611	0.170817	353
**HP_DEVIATION_OF_TOES**	HP_DEVIATION_OF_TOES	HP_DEVIATION_OF_TOES	22	-0.427824951	-1.754135699	0.011467	0.183401	0.171556	479
**HP_OCULAR_ANTERIOR_SEGMENT_DYSGENESIS**	HP_OCULAR_ANTERIOR_SEGMENT_DYSGENESIS	HP_OCULAR_ANTERIOR_SEGMENT_DYSGENESIS	33	-0.368331296	-1.72216587	0.011547	0.184406	0.172496	49
**GOBP_APOPTOTIC_MITOCHONDRIAL_CHANGES**	GOBP_APOPTOTIC_MITOCHONDRIAL_CHANGES	GOBP_APOPTOTIC_MITOCHONDRIAL_CHANGES	14	0.452739178	1.830089115	0.011595	0.184865	0.172925	97
**GOCC_LYSOSOMAL_LUMEN**	GOCC_LYSOSOMAL_LUMEN	GOCC_LYSOSOMAL_LUMEN	10	0.529381891	1.859782003	0.011610	0.184865	0.172925	21
**GOBP_RESPONSE_TO_TRANSFORMING_GROWTH_FACTOR_BETA**	GOBP_RESPONSE_TO_TRANSFORMING_GROWTH_FACTOR_BETA	GOBP_RESPONSE_TO_TRANSFORMING_GROWTH_FACTOR_BETA	28	0.330693388	1.801143048	0.011712	0.185663	0.173672	20
**GOMF_FERRIC_IRON_BINDING**	GOMF_FERRIC_IRON_BINDING	GOMF_FERRIC_IRON_BINDING	2	0.922750424	1.610849431	0.011728	0.185663	0.173672	93
**HP_ABNORMAL_CIRCULATING_FERRITIN_CONCENTRATION**	HP_ABNORMAL_CIRCULATING_FERRITIN_CONCENTRATION	HP_ABNORMAL_CIRCULATING_FERRITIN_CONCENTRATION	2	0.922750424	1.610849431	0.011728	0.185663	0.173672	93
**HP_HYPERREFLEXIA_IN_UPPER_LIMBS**	HP_HYPERREFLEXIA_IN_UPPER_LIMBS	HP_HYPERREFLEXIA_IN_UPPER_LIMBS	2	0.922750424	1.610849431	0.011728	0.185663	0.173672	93
**GOBP_ENDOTHELIAL_CELL_CHEMOTAXIS**	GOBP_ENDOTHELIAL_CELL_CHEMOTAXIS	GOBP_ENDOTHELIAL_CELL_CHEMOTAXIS	3	0.845423511	1.731123417	0.011829	0.186978	0.174902	27
**HP_HYPOGONADISM**	HP_HYPOGONADISM	HP_HYPOGONADISM	30	-0.381642013	-1.721646538	0.012071	0.190538	0.178232	464
**GOBP_POSITIVE_REGULATION_OF_PATTERN_RECOGNITION_RECEPTOR_SIGNALING_PA**	GOBP_POSITIVE_REGULATION_OF_PATTERN_RECOGNITION_RECEPTOR_SIGNALING_PA	GOBP_POSITIVE_REGULATION_OF_PATTERN_RECOGNITION_RECEPTOR_SIGNALING_PA	6	0.662845621	1.857116161	0.012105	0.190800	0.178477	7
**GOBP_AXO_DENDRITIC_TRANSPORT**	GOBP_AXO_DENDRITIC_TRANSPORT	GOBP_AXO_DENDRITIC_TRANSPORT	4	0.799719536	1.843123693	0.012142	0.191104	0.178761	6
**GOMF_EXOGENOUS_PROTEIN_BINDING**	GOMF_EXOGENOUS_PROTEIN_BINDING	GOMF_EXOGENOUS_PROTEIN_BINDING	8	0.563645526	1.804916739	0.012204	0.191795	0.179408	155

GOBP_POSITIVE_REGULATION_OF_APOPTOTIC_SIGNALING_PATHWAY	GOBP_POSITIVE_REGULATION_OF_APOPTOTIC_SIGNALING_PATHWAY	GOBP_POSITIVE_REGULATION_OF_APOPTOTIC_SIGNALING_PATHWAY	18	0.416260631	1.910379551	0.012282	0.191914	0.179519	98
HP_FRONTAL_LOBE_DEMENTIA	HP_FRONTAL_LOBE_DEMENTIA	HP_FRONTAL_LOBE_DEMENTIA	2	0.919354839	1.604921742	0.012282	0.191914	0.179519	97
GOBP_AXO_DENDRITIC_PROTEIN_TRANSPORT	GOBP_AXO_DENDRITIC_PROTEIN_TRANSPORT	GOBP_AXO_DENDRITIC_PROTEIN_TRANSPORT	1	0.998303647	1.330644712	0.012406	0.191914	0.179519	3
GOBP_POSITIVE_REGULATION_OF_ENDOTHELIAL_CELL_CHEMOTAXIS_BY_VEGF_ACTIV	GOBP_POSITIVE_REGULATION_OF_ENDOTHELIAL_CELL_CHEMOTAXIS_BY_VEGF_ACTIVA	GOBP_POSITIVE_REGULATION_OF_ENDOTHELIAL_CELL_CHEMOTAXIS_BY_VEGF_ACTIVA	1	0.998303647	1.330644712	0.012406	0.191914	0.179519	3
GOBP_PROTEIN_LOCALIZATION_TO_PRESYNAPSE	GOBP_PROTEIN_LOCALIZATION_TO_PRESYNAPSE	GOBP_PROTEIN_LOCALIZATION_TO_PRESYNAPSE	1	0.998303647	1.330644712	0.012406	0.191914	0.179519	3
GOBP_PROTEIN_TRANSPORT_ALONG_MICROTUBULE	GOBP_PROTEIN_TRANSPORT_ALONG_MICROTUBULE	GOBP_PROTEIN_TRANSPORT_ALONG_MICROTUBULE	1	0.998303647	1.330644712	0.012406	0.191914	0.179519	3
HP_DISTAL_LOWER_LIMB_AMYOTROPHY	HP_DISTAL_LOWER_LIMB_AMYOTROPHY	HP_DISTAL_LOWER_LIMB_AMYOTROPHY	1	0.998303647	1.330644712	0.012406	0.191914	0.179519	3
HP_IMPAIRED_TEMPERATURE_SENSATION	HP_IMPAIRED_TEMPERATURE_SENSATION	HP_IMPAIRED_TEMPERATURE_SENSATION	1	0.998303647	1.330644712	0.012406	0.191914	0.179519	3
HP_LIMB_FASCICULATIONS	HP_LIMB_FASCICULATIONS	HP_LIMB_FASCICULATIONS	1	0.998303647	1.330644712	0.012406	0.191914	0.179519	3
HP_ULNAR_CLAW	HP_ULNAR_CLAW	HP_ULNAR_CLAW	1	0.998303647	1.330644712	0.012406	0.191914	0.179519	3
HP_MUSCLE_FIBRILLATION	HP_MUSCLE_FIBRILLATION	HP_MUSCLE_FIBRILLATION	8	0.561601734	1.798372068	0.012406	0.191914	0.179519	408
HP_SUBMUCOUS_CLEFT_OF_SOFT_AND_HARD_PALATE	HP_SUBMUCOUS_CLEFT_OF_SOFT_AND_HARD_PALATE	HP_SUBMUCOUS_CLEFT_OF_SOFT_AND_HARD_PALATE	5	-0.725828264	-1.755561131	0.012427	0.191968	0.179569	164
GOBP_REGULATION_OF_POTASSIUM_ION_TRANSPORT	GOBP_REGULATION_OF_POTASSIUM_ION_TRANSPORT	GOBP_REGULATION_OF_POTASSIUM_ION_TRANSPORT	7	-0.614019134	-1.681555987	0.012467	0.192316	0.179895	91
GOBP_RESPONSE_TO_TUMOR_NECROSIS_FACTOR	GOBP_RESPONSE_TO_TUMOR_NECROSIS_FACTOR	GOBP_RESPONSE_TO_TUMOR_NECROSIS_FACTOR	27	0.339166892	1.830838698	0.012535	0.193033	0.180566	135
GOBP_RESPONSE_TO_STEROID_HORMONE	GOBP_RESPONSE_TO_STEROID_HORMONE	GOBP_RESPONSE_TO_STEROID_HORMONE	33	0.331833692	1.931037335	0.012550	0.193033	0.180566	131
GOBP_POSITIVE_REGULATION_OF_SMALL_GTPASE_MEDIATED_SIGNAL_TRANSDUCTIO	GOBP_POSITIVE_REGULATION_OF_SMALL_GTPASE_MEDIATED_SIGNAL_TRANSDUCTION	GOBP_POSITIVE_REGULATION_OF_SMALL_GTPASE_MEDIATED_SIGNAL_TRANSDUCTION	4	-0.738162017	-1.652353461	0.012567	0.193033	0.180566	159
GOBP_VASCULATURE_DEVELOPMENT	GOBP_VASCULATURE_DEVELOPMENT	GOBP_VASCULATURE_DEVELOPMENT	62	0.245470634	1.799322572	0.012585	0.193043	0.180575	128
HP_STEREOTYPY	HP_STEREOTYPY	HP_STEREOTYPY	42	-0.336768244	-1.70609095	0.012751	0.194796	0.182215	441
HP_DELAYED_ABILITY_TO_ROLL_OVER	HP_DELAYED_ABILITY_TO_ROLL_OVER	HP_DELAYED_ABILITY_TO_ROLL_OVER	3	-0.800339847	-1.594161844	0.012771	0.194796	0.182215	239
HP_DEVELOPMENTAL_STAGNATION	HP_DEVELOPMENTAL_STAGNATION	HP_DEVELOPMENTAL_STAGNATION	3	-0.800339847	-1.594161844	0.012771	0.194796	0.182215	239
HP_REPETITIVE_COMPULSIVE_BEHAVIOR	HP_REPETITIVE_COMPULSIVE_BEHAVIOR	HP_REPETITIVE_COMPULSIVE_BEHAVIOR	3	-0.800339847	-1.594161844	0.012771	0.194796	0.182215	239
GOBP_PEROXISOME_PROLIFERATOR_ACTIVATED_RECEPTOR_SIGNALING_PATHWAY	GOBP_PEROXISOME_PROLIFERATOR_ACTIVATED_RECEPTOR_SIGNALING_PATHWAY	GOBP_PEROXISOME_PROLIFERATOR_ACTIVATED_RECEPTOR_SIGNALING_PATHWAY	2	0.918505942	1.60343982	0.012837	0.194975	0.182383	98
GOBP_PROSTANOID_BIOSYNTHETIC_PROCESS	GOBP_PROSTANOID_BIOSYNTHETIC_PROCESS	GOBP_PROSTANOID_BIOSYNTHETIC_PROCESS	2	0.918505942	1.60343982	0.012837	0.194975	0.182383	98
GOBP_REGULATION_OF_PEROXISOME_PROLIFERATOR_ACTIVATED_RECEPTOR_SIGNAL	GOBP_REGULATION_OF_PEROXISOME_PROLIFERATOR_ACTIVATED_RECEPTOR_SIGNAL	GOBP_REGULATION_OF_PEROXISOME_PROLIFERATOR_ACTIVATED_RECEPTOR_SIGNAL	2	0.918505942	1.60343982	0.012837	0.194975	0.182383	98
GOCC_MEMBRANE_MICRODOMAIN	GOCC_MEMBRANE_MICRODOMAIN	GOCC_MEMBRANE_MICRODOMAIN	27	-0.392619268	-1.700529468	0.012940	0.195602	0.182969	265
GOBP_POSITIVE_REGULATION_OF_INTERLEUKIN_2_PRODUCTION	GOBP_POSITIVE_REGULATION_OF_INTERLEUKIN_2_PRODUCTION	GOBP_POSITIVE_REGULATION_OF_INTERLEUKIN_2_PRODUCTION	7	-0.612152717	-1.676444607	0.012942	0.195602	0.182969	167
HP_SUPERNUMERARY_NIPPLE	HP_SUPERNUMERARY_NIPPLE	HP_SUPERNUMERARY_NIPPLE	7	-0.612046404	-1.676153457	0.012942	0.195602	0.182969	159
GOBP_POSITIVE_REGULATION_OF_INTRINSIC_APOPTOTIC_SIGNALING_PATHWAY	GOBP_POSITIVE_REGULATION_OF_INTRINSIC_APOPTOTIC_SIGNALING_PATHWAY	GOBP_POSITIVE_REGULATION_OF_INTRINSIC_APOPTOTIC_SIGNALING_PATHWAY	11	0.505888424	1.880847542	0.012950	0.195602	0.182969	301
GOBP_REGULATION_OF_SECRETION	GOBP_REGULATION_OF_SECRETION	GOBP_REGULATION_OF_SECRETION	33	-0.365325684	-1.708112859	0.012978	0.195754	0.183111	165
HP_ABNORMAL_NASOLACRIMAL_SYSTEM_MORPHOLOGY	HP_ABNORMAL_NASOLACRIMAL_SYSTEM_MORPHOLOGY	HP_ABNORMAL_NASOLACRIMAL_SYSTEM_MORPHOLOGY	11	-0.541257741	-1.75557973	0.013034	0.196051	0.183389	407
HP_PSYCHOGENIC_NON_EPILEPTIC_SEIZURE	HP_PSYCHOGENIC_NON_EPILEPTIC_SEIZURE	HP_PSYCHOGENIC_NON_EPILEPTIC_SEIZURE	11	-0.540908751	-1.754447774	0.013034	0.196051	0.183389	339
GOBP_BIOMINERALIZATION	GOBP_BIOMINERALIZATION	GOBP_BIOMINERALIZATION	10	-0.582481101	-1.838831778	0.013053	0.196078	0.183414	315
GOMF_NUCLEOTIDE_RECEPTOR_ACTIVITY	GOMF_NUCLEOTIDE_RECEPTOR_ACTIVITY	GOMF_NUCLEOTIDE_RECEPTOR_ACTIVITY	3	-0.799052616	-1.591597864	0.013257	0.198869	0.186025	29
HP_HYPOPLASIA_OF_TEETH	HP_HYPOPLASIA_OF_TEETH	HP_HYPOPLASIA_OF_TEETH	12	-0.5193371	-1.728771774	0.013301	0.199240	0.186372	119
GOBP_BONE_REMODELING	GOBP_BONE_REMODELING	GOBP_BONE_REMODELING	8	-0.613894294	-1.761962993	0.013353	0.199754	0.186852	265
HP_ABNORMALITY_OF_THE_PULMONARY_VASCULATURE	HP_ABNORMALITY_OF_THE_PULMONARY_VASCULATURE	HP_ABNORMALITY_OF_THE_PULMONARY_VASCULATURE	14	-0.494501274	-1.742732561	0.013510	0.201827	0.188792	177
HP_ABNORMAL_INTESTINE_MORPHOLOGY	HP_ABNORMAL_INTESTINE_MORPHOLOGY	HP_ABNORMAL_INTESTINE_MORPHOLOGY	83	-0.272764837	-1.642703988	0.013592	0.202506	0.189427	430
GOBP_CHONDROCYTE_PROLIFERATION	GOBP_CHONDROCYTE_PROLIFERATION	GOBP_CHONDROCYTE_PROLIFERATION	2	-0.899830221	-1.529048345	0.013612	0.202506	0.189427	121
HP_NOCTURNAL_SEIZURES	HP_NOCTURNAL_SEIZURES	HP_NOCTURNAL_SEIZURES	2	-0.896434635	-1.523278351	0.013612	0.202506	0.189427	125
HP_CLEFT_LIP	HP_CLEFT_LIP	HP_CLEFT_LIP	29	0.324746102	1.796457751	0.013642	0.202678	0.189588	324
GOBP_CIRCULATORY_SYSTEM_DEVELOPMENT	GOBP_CIRCULATORY_SYSTEM_DEVELOPMENT	GOBP_CIRCULATORY_SYSTEM_DEVELOPMENT	80	0.209717177	1.657324964	0.013733	0.203748	0.190589	75
GOBP_BONE_MINERALIZATION	GOBP_BONE_MINERALIZATION	GOBP_BONE_MINERALIZATION	8	-0.612435928	-1.757777278	0.013826	0.204852	0.191621	315
GOBP_CARDIOCYTE_DIFFERENTIATION	GOBP_CARDIOCYTE_DIFFERENTIATION	GOBP_CARDIOCYTE_DIFFERENTIATION	7	-0.607666108	-1.664157556	0.013892	0.205547	0.192272	153
GOCC_SOMATODENDRITIC_COMPARTMENT	GOCC_SOMATODENDRITIC_COMPARTMENT	GOCC_SOMATODENDRITIC_COMPARTMENT	41	0.279190755	1.74750291	0.013960	0.206070	0.192761	20
HP_ABNORMALITY_OF_URINE_HOMEOSTASIS	HP_ABNORMALITY_OF_URINE_HOMEOSTASIS	HP_ABNORMALITY_OF_URINE_HOMEOSTASIS	49	-0.320265372	-1.697034764	0.013969	0.206070	0.192761	94
GOBP_VIRAL_PROCESS	GOBP_VIRAL_PROCESS	GOBP_VIRAL_PROCESS	53	0.244175908	1.662288666	0.013985	0.206070	0.192761	155
GOMF_SH2_DOMAIN_BINDING	GOMF_SH2_DOMAIN_BINDING	GOMF_SH2_DOMAIN_BINDING	4	-0.733215964	-1.641281869	0.014003	0.206070	0.192761	172
GOBP_CENTRAL_NERVOUS_SYSTEM_NEURON_DEVELOPMENT	GOBP_CENTRAL_NERVOUS_SYSTEM_NEURON_DEVELOPMENT	GOBP_CENTRAL_NERVOUS_SYSTEM_NEURON_DEVELOPMENT	7	0.590294558	1.77633998	0.014106	0.206564	0.193223	20
GOBP_NEGATIVE_REGULATION_OF_RESPONSE_TO_ENDOPLASMIC_RETICULUM_STRES	GOBP_NEGATIVE_REGULATION_OF_RESPONSE_TO_ENDOPLASMIC_RETICULUM_STRES	GOBP_NEGATIVE_REGULATION_OF_RESPONSE_TO_ENDOPLASMIC_RETICULUM_STRES	7	0.589502819	1.773957443	0.014106	0.206564	0.193223	9
HP_IMMUNOLOGIC_HYPERSENSITIVITY	HP_IMMUNOLOGIC_HYPERSENSITIVITY	HP_IMMUNOLOGIC_HYPERSENSITIVITY	23	-0.419450289	-1.757973765	0.014115	0.206564	0.193223	370
GOBP_REGULATION_OF_INTRACELLULAR_SIGNAL_TRANSDUCTION	GOBP_REGULATION_OF_INTRACELLULAR_SIGNAL_TRANSDUCTION	GOBP_REGULATION_OF_INTRACELLULAR_SIGNAL_TRANSDUCTION	166	0.164328226	1.528719091	0.014122	0.206564	0.193223	42
GOBP_POSITIVE_REGULATION_OF_DEVELOPMENTAL_PROCESS	GOBP_POSITIVE_REGULATION_OF_DEVELOPMENTAL_PROCESS	GOBP_POSITIVE_REGULATION_OF_DEVELOPMENTAL_PROCESS	89	0.209061237	1.684014506	0.014131	0.206564	0.193223	133
HP_FEEDING_DIFFICULTIES	HP_FEEDING_DIFFICULTIES	HP_FEEDING_DIFFICULTIES	109	-0.253036748	-1.613654254	0.014200	0.206979	0.193611	242
GOCC_CELL_PROJECTION_MEMBRANE	GOCC_CELL_PROJECTION_MEMBRANE	GOCC_CELL_PROJECTION_MEMBRANE	21	-0.415945648	-1.676685487	0.014212	0.206979	0.193611	253
HP_MUSCLE_WEAKNESS	HP_MUSCLE_WEAKNESS	HP_MUSCLE_WEAKNESS	85	-0.270498874	-1.640828934	0.014217	0.206979	0.193611	479
GOBP_INTRACELLULAR_RECEPTOR_SIGNALING_PATHWAY	GOBP_INTRACELLULAR_RECEPTOR_SIGNALING_PATHWAY	GOBP_INTRACELLULAR_RECEPTOR_SIGNALING_PATHWAY	38	0.280514053	1.743373058	0.014250	0.207190	0.193809	110
GOBP_POLYAMINE_METABOLIC_PROCESS	GOBP_POLYAMINE_METABOLIC_PROCESS	GOBP_POLYAMINE_METABOLIC_PROCESS	2	0.913412564	1.594548287	0.014314	0.207562	0.194157	104
GOBP_NEGATIVE_REGULATION_OF_FIBROBLAST_PROLIFERATION	GOBP_NEGATIVE_REGULATION_OF_FIBROBLAST_PROLIFERATION	GOBP_NEGATIVE_REGULATION_OF_FIBROBLAST_PROLIFERATION	4	0.788639966	1.817588468	0.014314	0.207562	0.194157	55
GOMF_DEACETYLASE_ACTIVITY	GOMF_DEACETYLASE_ACTIVITY	GOMF_DEACETYLASE_ACTIVITY	2	0.912563667	1.593066365	0.014499	0.209587	0.196050	105
GOMF_ATPASE_ACTIVATOR_ACTIVITY	GOMF_ATPASE_ACTIVATOR_ACTIVITY	GOMF_ATPASE_ACTIVATOR_ACTIVITY	4	0.787883441	1.815844895	0.014512	0.209587	0.196050	17
GOMF_MOLECULAR_FUNCTION_ACTIVATOR_ACTIVITY	GOMF_MOLECULAR_FUNCTION_ACTIVATOR_ACTIVITY	GOMF_MOLECULAR_FUNCTION_ACTIVATOR_ACTIVITY	4	0.787883441	1.815844895	0.014512	0.209587	0.196050	17
HP_AORTIC_ANEURYSM	HP_AORTIC_ANEURYSM	HP_AORTIC_ANEURYSM	19	-0.446722611	-1.747826861	0.014706	0.212117	0.198418	211
GOBP_ACTIVATION_OF_PHOSPHOLIPASE_C_ACTIVITY	GOBP_ACTIVATION_OF_PHOSPHOLIPASE_C_ACTIVITY	GOBP_ACTIVATION_OF_PHOSPHOLIPASE_C_ACTIVITY	2	-0.89049236	-1.513180862	0.014860	0.212777	0.199035	132
GOBP_GRANULOCYTE_MACROPHAGE_COLONY_STIMULATING_FACTOR_PRODUCTION	GOBP_GRANULOCYTE_MACROPHAGE_COLONY_STIMULATING_FACTOR_PRODUCTION	GOBP_GRANULOCYTE_MACROPHAGE_COLONY_STIMULATING_FACTOR_PRODUCTION	2	-0.891341256	-1.51462336	0.014860	0.212777	0.199035	131
GOBP_MONOCYTE_CHEMOTACTIC_PROTEIN_1_PRODUCTION	GOBP_MONOCYTE_CHEMOTACTIC_PROTEIN_1_PRODUCTION	GOBP_MONOCYTE_CHEMOTACTIC_PROTEIN_1_PRODUCTION	2	-0.891341256	-1.51462336	0.014860	0.212777	0.199035	131
GOBP_POSITIVE_REGULATION_OF_MONOCYTE_CHEMOTACTIC_PROTEIN_1_PRODUCTI	GOBP_POSITIVE_REGULATION_OF_MONOCYTE_CHEMOTACTIC_PROTEIN_1_PRODUCTI	GOBP_POSITIVE_REGULATION_OF_MONOCYTE_CHEMOTACTIC_PROTEIN_1_PRODUCTI	2	-0.891341256	-1.51462336	0.014860	0.212777	0.199035	131
GOBP_REGULATION_OF_PHOSPHOLIPASE_C_ACTIVITY	GOBP_REGULATION_OF_PHOSPHOLIPASE_C_ACTIVITY	GOBP_REGULATION_OF_PHOSPHOLIPASE_C_ACTIVITY	2	-0.89049236	-1.513180862	0.014860	0.212777	0.199035	132
HP_INFLAMMATION_OF_THE_LARGE_INTESTINE	HP_INFLAMMATION_OF_THE_LARGE_INTESTINE	HP_INFLAMMATION_OF_THE_LARGE_INTESTINE	10	-0.573117342	-1.809271372	0.014869	0.212777	0.199035	370
GOBP_NEGATIVE_REGULATION_OF_DNA_BINDING	GOBP_NEGATIVE_REGULATION_OF_DNA_BINDING	GOBP_NEGATIVE_REGULATION_OF_DNA_BINDING	5	0.710027482	1.7979232	0.014906	0.213023	0.199264	67
GOMF_TELOMERIC_DNA_BINDING	GOMF_TELOMERIC_DNA_BINDING	GOMF_TELOMERIC_DNA_BINDING	3	-0.789528682	-1.572627558	0.014960	0.213515	0.199725	67
GOBP_MUSCLE_CELL_DIFFERENTIATION	GOBP_MUSCLE_CELL_DIFFERENTIATION	GOBP_MUSCLE_CELL_DIFFERENTIATION	27	-0.387502575	-1.678367827	0.015044	0.214434	0.200585	266
HP_BROAD_TOE	HP_BROAD_TOE	HP_BROAD_TOE	11	-0.536282115	-1.739441193	0.015074	0.214480	0.200628	177
GOBP_REGULATION_OF_OXIDATIVE_STRESS_INDUCED_CELL_DEATH	GOBP_REGULATION_OF_OXIDATIVE_STRESS_INDUCED_CELL_DEATH	GOBP_REGULATION_OF_OXIDATIVE_STRESS_INDUCED_CELL_DEATH	6	0.645183868	1.807632653	0.015107	0.214480	0.200628	98
GOBP_REGULATION_OF_RESPONSE_TO_OXIDATIVE_STRESS	GOBP_REGULATION_OF_RESPONSE_TO_OXIDATIVE_STRESS	GOBP_REGULATION_OF_RESPONSE_TO_OXIDATIVE_STRESS	6	0.645183868	1.807632653	0.015107	0.214480	0.200628	98
HP_POOR_SPEECH	HP_POOR_SPEECH	HP_POOR_SPEECH	32	-0.36291382	-1.667041558	0.015146	0.214752	0.200882	406
GOBP_REGULATION_OF_PEPTIDYL_THREONINE_PHOSPHORYLATION	GOBP_REGULATION_OF_PEPTIDYL_THREONINE_PHOSPHORYLATION	GOBP_REGULATION_OF_PEPTIDYL_THREONINE_PHOSPHORYLATION	3	-0.78795306	-1.569489145	0.015204	0.215292	0.201387	37
GOBP_REGULATION_OF_LYMPHOCYTE_DIFFERENTIATION	GOBP_REGULATION_OF_LYMPHOCYTE_DIFFERENTIATION	GOBP_REGULATION_OF_LYMPHOCYTE_DIFFERENTIATION	21	-0.413501948	-1.666834883	0.015281	0.215820	0.201881	276
GOBP_INTRINSIC_APOPTOTIC_SIGNALING_PATHWAY_IN_RESPONSE_TO_ENDOPLASMIC	GOBP_INTRINSIC_APOPTOTIC_SIGNALING_PATHWAY_IN_RESPONSE_TO_ENDOPLASMIC	GOBP_INTRINSIC_APOPTOTIC_SIGNALING_PATHWAY_IN_RESPONSE_TO_ENDOPLASMIC	11	0.497643119	1.850192242	0.015344	0.215820	0.201881	98
HP_MACROTIA	HP_MACROTIA	HP_MACROTIA	32	-0.362433005	-1.66483294	0.015352	0.215820	0.201881	177
GOBP_SPHINGOLIPID_MEDIATED_SIGNALING_PATHWAY	GOBP_SPHINGOLIPID_MEDIATED_SIGNALING_PATHWAY	GOBP_SPHINGOLIPID_MEDIATED_SIGNALING_PATHWAY	2	-0.887096774	-1.507410868	0.015360	0.215820	0.201881	136
GOMF_BIOACTIVE_LIPID_RECEPTOR_ACTIVITY	GOMF_BIOACTIVE_LIPID_RECEPTOR_ACTIVITY	GOMF_BIOACTIVE_LIPID_RECEPTOR_ACTIVITY	2	-0.887096774	-1.507410868	0.015360	0.215820	0.201881	136
GOMF_SPHINGOSINE_1_PHOSPHATE_RECEPTOR_ACTIVITY	GOMF_SPHINGOSINE_1_PHOSPHATE_RECEPTOR_ACTIVITY	GOMF_SPHINGOSINE_1_PHOSPHATE_RECEPTOR_ACTIVITY	2	-0.887096774	-1.507410868	0.015360	0.215820	0.201881	136
HP_NARROW_FOREHEAD	HP_NARROW_FOREHEAD	HP_NARROW_FOREHEAD	16	-0.4613923	-1.701375695	0.015413	0.216285	0.202316	164
HP_DELAYED_ABILITY_TO_WALK	HP_DELAYED_ABILITY_TO_WALK	HP_DELAYED_ABILITY_TO_WALK	28	-0.3866753	-1.700070132	0.015501	0.217244	0.203213	239
HP_CAFE_AU_LAIT_SPOT	HP_CAFE_AU_LAIT_SPOT	HP_CAFE_AU_LAIT_SPOT	18	-0.454066915	-1.763397342	0.015531	0.217378	0.203338	479
HP_DEVIATION_OF_THE_HALLUX	HP_DEVIATION_OF_THE_HALLUX	HP_DEVIATION_OF_THE_HALLUX	16	-0.460821124	-1.69926949	0.015629	0.218465	0.204355	206
GOBP_REGULATION_OF_CYSTEINE_TYPE_ENDOPEPTIDASE_ACTIVITY	GOBP_REGULATION_OF_CYSTEINE_TYPE_ENDOPEPTIDASE_ACTIVITY	GOBP_REGULATION_OF_CYSTEINE_TYPE_ENDOPEPTIDASE_ACTIVITY	34	0.299891117	1.769837845	0.016052	0.224091	0.209618	46
GOBP_MICROTUBULE_NUCLEATION	GOBP_MICROTUBULE_NUCLEATION	GOBP_MICROTUBULE_NUCLEATION	5	0.704078469	1.782859178	0.016075	0.224134	0.209658	7
HP_ESOPHAGITIS	HP_ESOPHAGITIS	HP_ESOPHAGITIS	2	-0.884550085	-1.503083373	0.016109	0.224318	0.209831	139
GOBP_ENDOTHELIAL_CELL_MIGRATION	GOBP_ENDOTHELIAL_CELL_MIGRATION	GOBP_ENDOTHELIAL_CELL_MIGRATION	22	0.370866393	1.827657336	0.016242	0.225640	0.211067	42
GOBP_HISTONE_DEUBIQUITINATION	GOBP_HISTONE_DEUBIQUITINATION	GOBP_HISTONE_DEUBIQUITINATION	7	-0.600035212	-1.643259546	0.016266	0.225640	0.211067	228
HP_SUBCUTANEOUS_NODULE	HP_SUBCUTANEOUS_NODULE	HP_SUBCUTANEOUS_NODULE	7	-0.598465473	-1.63896065	0.016266	0.225640	0.211067	479
GOCC_LEADING_EDGE_MEMBRANE	GOCC_LEADING_EDGE_MEMBRANE	GOCC_LEADING_EDGE_MEMBRANE	15	-0.501605583	-1.795840738	0.016342	0.226404	0.211782	251
HP_VASCULAR_DILATATION	HP_VASCULAR_DILATATION	HP_VASCULAR_DILATATION	23	-0.413678445	-1.733783175	0.016382	0.226668	0.212028	479
GOBP_RETINA_HOMEOSTASIS	GOBP_RETINA_HOMEOSTASIS	GOBP_RETINA_HOMEOSTASIS	3	0.831202258	1.702003404	0.016445	0.227244	0.212567	58
GOBP_NEGATIVE_REGULATION_OF_CELL_ACTIVATION	GOBP_NEGATIVE_REGULATION_OF_CELL_ACTIVATION	GOBP_NEGATIVE_REGULATION_OF_CELL_ACTIVATION	25	-0.395747298	-1.677263588	0.016471	0.227319	0.212637	628
GOBP_SPINDLE_ORGANIZATION	GOBP_SPINDLE_ORGANIZATION	GOBP_SPINDLE_ORGANIZATION	17	0.409223721	1.813196488	0.016527	0.227668	0.212964	22
GOBP_NEGATIVE_REGULATION_OF_BIOSYNTHETIC_PROCESS	GOBP_NEGATIVE_REGULATION_OF_BIOSYNTHETIC_PROCESS	GOBP_NEGATIVE_REGULATION_OF_BIOSYNTHETIC_PROCESS	147	0.1775488	1.601267285	0.016538	0.227668	0.212964	82
GOBP_ERYTHROCYTE_HOMEOSTASIS	GOBP_ERYTHROCYTE_HOMEOSTASIS	GOBP_ERYTHROCYTE_HOMEOSTASIS	23	0.345929779	1.733711874	0.016586	0.228036	0.213308	73
GOBP_RESPONSE_TO_CADMIUM_ION	GOBP_RESPONSE_TO_CADMIUM_ION	GOBP_RESPONSE_TO_CADMIUM_ION	5	0.701949788	1.777468957	0.016660	0.228765	0.213990	13
HP_INTELLECTUAL_DISABILITY_SEVERE	HP_INTELLECTUAL_DISABILITY_SEVERE	HP_INTELLECTUAL_DISABILITY_SEVERE	51	-0.317746531	-1.710872341	0.016720	0.229015	0.214224	491
GOBP_EYELID_DEVELOPMENT_IN_CAMERA_TYPE_EYE	GOBP_EYELID_DEVELOPMENT_IN_CAMERA_TYPE_EYE	GOBP_EYELID_DEVELOPMENT_IN_CAMERA_TYPE_EYE	1	0.997455471	1.329514172	0.016735	0.229015	0.214224	4
GOBP_REGULATION_OF_RHO_PROTEIN_SIGNAL_TRANSDUCTION	GOBP_REGULATION_OF_RHO_PROTEIN_SIGNAL_TRANSDUCTION	GOBP_REGULATION_OF_RHO_PROTEIN_SIGNAL_TRANSDUCTION	7	-0.598007794	-1.637707248	0.016741	0.229015	0.214224	163
GOMF_GLUCOCORTICOID_RECEPTOR_BINDING	GOMF_GLUCOCORTICOID_RECEPTOR_BINDING	GOMF_GLUCOCORTICOID_RECEPTOR_BINDING	2	0.908319185	1.585656754	0.016900	0.230660	0.215762	110
GOBP_REGULATION_OF_SUPRAMOLECULAR_FIBER_ORGANIZATION	GOBP_REGULATION_OF_SUPRAMOLECULAR_FIBER_ORGANIZATION	GOBP_REGULATION_OF_SUPRAMOLECULAR_FIBER_ORGANIZATION	34	0.298490491	1.76157191	0.016904	0.230660	0.215762	58
HP_ABNORMAL_RENAL_PHYSIOLOGY	HP_ABNORMAL_RENAL_PHYSIOLOGY	HP_ABNORMAL_RENAL_PHYSIOLOGY	52	-0.323012921	-1.737596543	0.016982	0.230840	0.215931	222
GOBP_POSITIVE_REGULATION_OF_OSSIFICATION	GOBP_POSITIVE_REGULATION_OF_OSSIFICATION	GOBP_POSITIVE_REGULATION_OF_OSSIFICATION	5	-0.709498211	-1.716063626	0.017011	0.230840	0.215931	91
GOCC_GLUTAMATERGIC_SYNAPSE	GOCC_GLUTAMATERGIC_SYNAPSE	GOCC_GLUTAMATERGIC_SYNAPSE	14	0.442602988	1.789116007	0.017043	0.230840	0.215931	6
GOBP_NEGATIVE_REGULATION_OF_EXTRINSIC_APOPTOTIC_SIGNALING_PATHWAY	GOBP_NEGATIVE_REGULATION_OF_EXTRINSIC_APOPTOTIC_SIGNALING_PATHWAY	GOBP_NEGATIVE_REGULATION_OF_EXTRINSIC_APOPTOTIC_SIGNALING_PATHWAY	11	0.491096435	1.82585226	0.017085	0.230840	0.215931	42
GOBP_RESPONSE_TO_ACID_CHEMICAL	GOBP_RESPONSE_TO_ACID_CHEMICAL	GOBP_RESPONSE_TO_ACID_CHEMICAL	11	0.489858797	1.821250834	0.017085	0.230840	0.215931	174
GOBP_ADENYLATE_CYCLASE_ACTIVATING_ADRENERGIC_RECEPTOR_SIGNALING_PATH	GOBP_ADENYLATE_CYCLASE_ACTIVATING_ADRENERGIC_RECEPTOR_SIGNALING_PATH	GOBP_ADENYLATE_CYCLASE_ACTIVATING_ADRENERGIC_RECEPTOR_SIGNALING_PATH	2	-0.880305603	-1.495870881	0.017108	0.230840	0.215931	144
GOBP_ADENYLATE_CYCLASE_ACTIVATING_ADRENERGIC_RECEPTOR_SIGNALING_PATH	GOBP_ADENYLATE_CYCLASE_ACTIVATING_ADRENERGIC_RECEPTOR_SIGNALING_PATH	GOBP_ADENYLATE_CYCLASE_ACTIVATING_ADRENERGIC_RECEPTOR_SIGNALING_PATH	2	-0.880305603	-1.495870881	0.017108	0.230840	0.215931	144
GOBP_ADRENERGIC_RECEPTOR_SIGNALING_PATHWAY	GOBP_ADRENERGIC_RECEPTOR_SIGNALING_PATHWAY	GOBP_ADRENERGIC_RECEPTOR_SIGNALING_PATHWAY	2	-0.880305603	-1.495870881	0.017108	0.230840	0.215931	144
GOBP_G_PROTEIN_COUPLED_RECEPTOR_SIGNALING_PATHWAY_INVOLVED_IN_HEART_	GOBP_G_PROTEIN_COUPLED_RECEPTOR_SIGNALING_PATHWAY_INVOLVED_IN_HEART_	GOBP_G_PROTEIN_COUPLED_RECEPTOR_SIGNALING_PATHWAY_INVOLVED_IN_HEART_	2	-0.880305603	-1.495870881	0.017108	0.230840	0.215931	144
GOMF_IDENTICAL_PROTEIN_BINDING	GOMF_IDENTICAL_PROTEIN_BINDING	GOMF_IDENTICAL_PROTEIN_BINDING	134	0.17579319	1.552368543	0.017159	0.231240	0.216306	63
GOMF_RRNA_BINDING	GOMF_RRNA_BINDING	GOMF_RRNA_BINDING	9	0.55626959	1.868864707	0.017298	0.232823	0.217786	270
HP_FOCAL_T2_HYPERINTENSE_BRAINSTEM_LESION	HP_FOCAL_T2_HYPERINTENSE_BRAINSTEM_LESION	HP_FOCAL_T2_HYPERINTENSE_BRAINSTEM_LESION	8	-0.602348623	-1.728825293	0.017371	0.232922	0.217879	227
GOBP_MELANOCYTE_DIFFERENTIATION	GOBP_MELANOCYTE_DIFFERENTIATION	GOBP_MELANOCYTE_DIFFERENTIATION	3	-0.783347494	-1.56031552	0.017393	0.232922	0.217879	259
GOBP_NEGATIVE_REGULATION_OF_OSSIFICATION	GOBP_NEGATIVE_REGULATION_OF_OSSIFICATION	GOBP_NEGATIVE_REGULATION_OF_OSSIFICATION	3	-0.783347494	-1.56031552	0.017393	0.232922	0.217879	259
GOBP_PIGMENT_CELL_DIFFERENTIATION	GOBP_PIGMENT_CELL_DIFFERENTIATION	GOBP_PIGMENT_CELL_DIFFERENTIATION	3	-0.783347494	-1.56031552	0.017393	0.232922	0.217879	259
HP_WEIGHT_LOSS	HP_WEIGHT_LOSS	HP_WEIGHT_LOSS	32	-0.358101098	-1.64493436	0.017412	0.232922	0.217879	361
GOBP_NEGATIVE_REGULATION_OF_OXIDATIVE_STRESS_INDUCED_CELL_DEATH	GOBP_NEGATIVE_REGULATION_OF_OXIDATIVE_STRESS_INDUCED_CELL_DEATH	GOBP_NEGATIVE_REGULATION_OF_OXIDATIVE_STRESS_INDUCED_CELL_DEATH	5	0.698964215	1.769908926	0.017634	0.235236	0.220043	98
HP_CUTANEOUS_ABSCESS	HP_CUTANEOUS_ABSCESS	HP_CUTANEOUS_ABSCESS	3	-0.782619461	-1.558865383	0.017636	0.235236	0.220043	80
GOBP_PROTEIN_LOCALIZATION_TO_CELL_JUNCTION	GOBP_PROTEIN_LOCALIZATION_TO_CELL_JUNCTION	GOBP_PROTEIN_LOCALIZATION_TO_CELL_JUNCTION	8	0.543114238	1.739171046	0.017650	0.235236	0.220043	128
HP_NON_MOTOR_SEIZURE	HP_NON_MOTOR_SEIZURE	HP_NON_MOTOR_SEIZURE	25	-0.393713551	-1.668644126	0.017730	0.236008	0.220765	242
HP_ABNORMALITY_OF_REFRACTION	HP_ABNORMALITY_OF_REFRACTION	HP_ABNORMALITY_OF_REFRACTION	63	-0.292231809	-1.646371444	0.018119	0.240646	0.225104	442
GOBP_VASCULAR_ENDOTHELIAL_CELL_PROLIFERATION	GOBP_VASCULAR_ENDOTHELIAL_CELL_PROLIFERATION	GOBP_VASCULAR_ENDOTHELIAL_CELL_PROLIFERATION	3	-0.780343345	-1.554331687	0.018123	0.240646	0.225104	71
HP_ABSENT_SPEECH	HP_ABSENT_SPEECH	HP_ABSENT_SPEECH	41	-0.334672829	-1.684360601	0.018149	0.240697	0.225151	593
GOBP_LUNG_EPITHELIUM_DEVELOPMENT	GOBP_LUNG_EPITHELIUM_DEVELOPMENT	GOBP_LUNG_EPITHELIUM_DEVELOPMENT	2	0.906621392	1.582692909	0.018193	0.240697	0.225151	112
HP_HEAT_INTOLERANCE	HP_HEAT_INTOLERANCE	HP_HEAT_INTOLERANCE	2	0.906621392	1.582692909	0.018193	0.240697	0.225151	112
HP_ABNORMALITY_OF_THE_CHEEK	HP_ABNORMALITY_OF_THE_CHEEK	HP_ABNORMALITY_OF_THE_CHEEK	28	-0.381790112	-1.678591744	0.018414	0.243330	0.227614	173
GOCC_MITOCHONDRIAL_MATRIX	GOCC_MITOCHONDRIAL_MATRIX	GOCC_MITOCHONDRIAL_MATRIX	32	0.293877493	1.717060376	0.018474	0.243819	0.228072	678
GOCC_INTRINSIC_COMPONENT_OF_PLASMA_MEMBRANE	GOCC_INTRINSIC_COMPONENT_OF_PLASMA_MEMBRANE	GOCC_INTRINSIC_COMPONENT_OF_PLASMA_MEMBRANE	52	-0.31975718	-1.720082801	0.018553	0.244570	0.228774	268
GOBP_POSITIVE_REGULATION_OF_TRANSCRIPTION_FROM_RNA_POLYMERASE_II_PRO	GOBP_POSITIVE_REGULATION_OF_TRANSCRIPTION_FROM_RNA_POLYMERASE_II_PRON	GOBP_POSITIVE_REGULATION_OF_TRANSCRIPTION_FROM_RNA_POLYMERASE_II_PRON	4	0.775645225	1.787639324	0.018658	0.245653	0.229787	10
HP_OVERWEIGHT	HP_OVERWEIGHT	HP_OVERWEIGHT	5	-0.704680851	-1.704411875	0.018700	0.245783	0.229909	353
GOBP_MITOTIC_SPINDLE_ORGANIZATION	GOBP_MITOTIC_SPINDLE_ORGANIZATION	GOBP_MITOTIC_SPINDLE_ORGANIZATION	10	0.511243657	1.796060217	0.018771	0.245783	0.229909	7
GOBP_REGULATION_OF_B_CELL_DIFFERENTIATION	GOBP_REGULATION_OF_B_CELL_DIFFERENTIATION	GOBP_REGULATION_OF_B_CELL_DIFFERENTIATION	4	-0.722913908	-1.618221028	0.018790	0.245783	0.229909	178
HP_APLASIA_HYPOPLASIA_OF_THE_OPTIC_NERVE	HP_APLASIA_HYPOPLASIA_OF_THE_OPTIC_NERVE	HP_APLASIA_HYPOPLASIA_OF_THE_OPTIC_NERVE	4	-0.723453808	-1.619429578	0.018790	0.245783	0.229909	173
GOBP_OVULATION_CYCLE	GOBP_OVULATION_CYCLE	GOBP_OVULATION_CYCLE	5	0.695319149	1.760678932	0.018803	0.245783	0.229909	363

GOBP_REGULATION_OF_PROTEIN_IMPORT	GOBP_REGULATION_OF_PROTEIN_IMPORT	GOBP_REGULATION_OF_PROTEIN_IMPORT	5	0.694982511	1.7598265	0.0188038	0.2457835	0.2299094	20
HP_NEOPLASM_OF_THE_COLON	HP_NEOPLASM_OF_THE_COLON	HP_NEOPLASM_OF_THE_COLON	21	0.357228027	1.749563558	0.0188555	0.2458796	0.2299993	301
GOBP_NEGATIVE_REGULATION_OF_ALPHA_BETA_T_CELL_ACTIVATION	GOBP_NEGATIVE_REGULATION_OF_ALPHA_BETA_T_CELL_ACTIVATION	GOBP_NEGATIVE_REGULATION_OF_ALPHA_BETA_T_CELL_ACTIVATION	7	-0.590006479	-1.615794804	0.0188790	0.2458796	0.2299993	276
HP_SPINAL_CORD_LESION	HP_SPINAL_CORD_LESION	HP_SPINAL_CORD_LESION	6	-0.650676356	-1.686069647	0.0188790	0.2458796	0.2299993	298
GOBP_REGULATION_OF_T_HELPER_2_CELL_DIFFERENTIATION	GOBP_REGULATION_OF_T_HELPER_2_CELL_DIFFERENTIATION	GOBP_REGULATION_OF_T_HELPER_2_CELL_DIFFERENTIATION	3	-0.77740017	-1.548469307	0.0190962	0.2479349	0.2319218	266
GOBP_T_HELPER_2_CELL_DIFFERENTIATION	GOBP_T_HELPER_2_CELL_DIFFERENTIATION	GOBP_T_HELPER_2_CELL_DIFFERENTIATION	3	-0.77740017	-1.548469307	0.0190962	0.2479349	0.2319218	266
GOBP_REGULATION_OF_TRANSMEMBRANE_TRANSPORT	GOBP_REGULATION_OF_TRANSMEMBRANE_TRANSPORT	GOBP_REGULATION_OF_TRANSMEMBRANE_TRANSPORT	34	-0.358430498	-1.697632491	0.0191052	0.2479349	0.2319218	170
GOBP_CELLULAR_IRON_ION_HOMEOSTASIS	GOBP_CELLULAR_IRON_ION_HOMEOSTASIS	GOBP_CELLULAR_IRON_ION_HOMEOSTASIS	5	0.693691292	1.756556891	0.0191935	0.2487843	0.2327164	162
GOBP_CHROMOSOME_CONDENSATION	GOBP_CHROMOSOME_CONDENSATION	GOBP_CHROMOSOME_CONDENSATION	3	-0.776054594	-1.545789113	0.0193394	0.2494106	0.2333023	82
GOBP_RESPONSE_TO_REACTIVE_OXYGEN_SPECIES	GOBP_RESPONSE_TO_REACTIVE_OXYGEN_SPECIES	GOBP_RESPONSE_TO_REACTIVE_OXYGEN_SPECIES	21	0.355756067	1.742354471	0.0193484	0.2494106	0.2333023	42
HP_AGENESIS_OF_CEREBELLAR_VERMIS	HP_AGENESIS_OF_CEREBELLAR_VERMIS	HP_AGENESIS_OF_CEREBELLAR_VERMIS	2	-0.873514431	-1.484330893	0.0193565	0.2494106	0.2333023	152
HP_HAPPY_DEMEANOR	HP_HAPPY_DEMEANOR	HP_HAPPY_DEMEANOR	2	-0.873514431	-1.484330893	0.0193565	0.2494106	0.2333023	152
HP_PELVIC_KIDNEY	HP_PELVIC_KIDNEY	HP_PELVIC_KIDNEY	2	-0.873514431	-1.484330893	0.0193565	0.2494106	0.2333023	152
GOBP_RESPONSE_TO_ENDOPLASMIC_RETICULUM_STRESS	GOBP_RESPONSE_TO_ENDOPLASMIC_RETICULUM_STRESS	GOBP_RESPONSE_TO_ENDOPLASMIC_RETICULUM_STRESS	44	0.263690245	1.685916884	0.0194246	0.2499915	0.2338457	32
GOBP_REGULATION_OF_MITOCHONDRION_ORGANIZATION	GOBP_REGULATION_OF_MITOCHONDRION_ORGANIZATION	GOBP_REGULATION_OF_MITOCHONDRION_ORGANIZATION	16	0.400468953	1.736803362	0.0194548	0.2500851	0.2339332	133
GOBP_NEGATIVE_REGULATION_OF_AUTOPHAGY	GOBP_NEGATIVE_REGULATION_OF_AUTOPHAGY	GOBP_NEGATIVE_REGULATION_OF_AUTOPHAGY	9	-0.570315311	-1.71951574	0.0195391	0.2508713	0.2346687	354
GOBP_NEGATIVE_REGULATION_OF_LYMPHOCYTE_ACTIVATION	GOBP_NEGATIVE_REGULATION_OF_LYMPHOCYTE_ACTIVATION	GOBP_NEGATIVE_REGULATION_OF_LYMPHOCYTE_ACTIVATION	17	-0.447234135	-1.700769502	0.0195737	0.2510199	0.2348076	618
GOBP_PROTEIN_LOCALIZATION_TO_MITOCHONDRION	GOBP_PROTEIN_LOCALIZATION_TO_MITOCHONDRION	GOBP_PROTEIN_LOCALIZATION_TO_MITOCHONDRION	14	0.436725888	1.765359246	0.0198264	0.2539604	0.2375582	265
HP_NARROW_MOUTH	HP_NARROW_MOUTH	HP_NARROW_MOUTH	29	-0.37344259	-1.668129218	0.0200236	0.2560985	0.2395582	437
GOBP_CELLULAR_RESPONSE_TO_CADMIUM_ION	GOBP_CELLULAR_RESPONSE_TO_CADMIUM_ION	GOBP_CELLULAR_RESPONSE_TO_CADMIUM_ION	4	0.770575345	1.775954708	0.0200404	0.2560985	0.2395582	13
GOBP_CARTILAGE_DEVELOPMENT	GOBP_CARTILAGE_DEVELOPMENT	GOBP_CARTILAGE_DEVELOPMENT	10	-0.562688956	-1.77635005	0.0200914	0.2564485	0.2398856	315
HP_ABNORMAL_ARACHNOID_MATER_MORPHOLOGY	HP_ABNORMAL_ARACHNOID_MATER_MORPHOLOGY	HP_ABNORMAL_ARACHNOID_MATER_MORPHOLOGY	4	-0.72038052	-1.612550115	0.0202269	0.2578753	0.2412202	164
GOMF_TRANSCRIPTION_COREPRESSOR_ACTIVITY	GOMF_TRANSCRIPTION_COREPRESSOR_ACTIVITY	GOMF_TRANSCRIPTION_COREPRESSOR_ACTIVITY	23	0.338221423	1.695079562	0.0203620	0.2592945	0.2425478	107
HP_ABNORMALITY_OF_CRANIAL_SUTURES	HP_ABNORMALITY_OF_CRANIAL_SUTURES	HP_ABNORMALITY_OF_CRANIAL_SUTURES	37	-0.338826774	-1.646068514	0.0204927	0.2606532	0.2438187	433
HP_DECREASED_ACTIVITY_OF_MITOCHONDRIAL_COMPLEX_I	HP_DECREASED_ACTIVITY_OF_MITOCHONDRIAL_COMPLEX_I	HP_DECREASED_ACTIVITY_OF_MITOCHONDRIAL_COMPLEX_I	7	-0.586518789	-1.606243398	0.0205413	0.2609659	0.2441112	222
GOCC_TORC1_COMPLEX	GOCC_TORC1_COMPLEX	GOCC_TORC1_COMPLEX	1	-0.995759118	-1.321330765	0.0206215	0.2616798	0.2447791	7
HP_PAROXYSMAL_INVOLUNTARY_EYE_MOVEMENTS	HP_PAROXYSMAL_INVOLUNTARY_EYE_MOVEMENTS	HP_PAROXYSMAL_INVOLUNTARY_EYE_MOVEMENTS	6	-0.645824675	-1.673497694	0.0207787	0.2633674	0.2463577	222
GOBP_REGULATION_OF_DEOXYRIBONUCLEASE_ACTIVITY	GOBP_REGULATION_OF_DEOXYRIBONUCLEASE_ACTIVITY	GOBP_REGULATION_OF_DEOXYRIBONUCLEASE_ACTIVITY	4	0.767857143	1.769690034	0.0208341	0.2635076	0.2464888	277
HP_HYPERACTIVITY	HP_HYPERACTIVITY	HP_HYPERACTIVITY	63	-0.2893177	-1.629953981	0.0208383	0.2635076	0.2464888	781
GOMF_CELL_ADHESION_MOLECULE_BINDING	GOMF_CELL_ADHESION_MOLECULE_BINDING	GOMF_CELL_ADHESION_MOLECULE_BINDING	61	0.223364635	1.62388988	0.0210154	0.2654389	0.2482954	37
HP_LONG_FINGERS	HP_LONG_FINGERS	HP_LONG_FINGERS	28	-0.376690026	-1.656168527	0.0211197	0.2664472	0.2492385	442
HP_MACRODONTIA	HP_MACRODONTIA	HP_MACRODONTIA	9	-0.566644215	-1.708447289	0.0211577	0.2666169	0.2493973	177
HP_ERYTHROCYTE_INCLUSION_BODIES	HP_ERYTHROCYTE_INCLUSION_BODIES	HP_ERYTHROCYTE_INCLUSION_BODIES	2	0.899830221	1.570837532	0.0213461	0.2686792	0.2513264	120
GOBP_POSITIVE_REGULATION_OF_CELL_GROWTH	GOBP_POSITIVE_REGULATION_OF_CELL_GROWTH	GOBP_POSITIVE_REGULATION_OF_CELL_GROWTH	14	-0.47577581	-1.676739857	0.0216392	0.2717260	0.2541764	354
GOBP_POSITIVE_REGULATION_OF_DEVELOPMENTAL_GROWTH	GOBP_POSITIVE_REGULATION_OF_DEVELOPMENTAL_GROWTH	GOBP_POSITIVE_REGULATION_OF_DEVELOPMENTAL_GROWTH	14	-0.47585734	-1.677027186	0.0216392	0.2717260	0.2541764	354
GOBP_REGULATION_OF_BONE_RESORPTION	GOBP_REGULATION_OF_BONE_RESORPTION	GOBP_REGULATION_OF_BONE_RESORPTION	4	-0.717573029	-1.606265629	0.0216631	0.2717260	0.2541764	136
HP_MYELODYSPLASIA	HP_MYELODYSPLASIA	HP_MYELODYSPLASIA	23	0.334480895	1.676332988	0.0217105	0.2720070	0.2544392	274
GOBP_POSITIVE_REGULATION_OF_EPITHELIAL_CELL_PROLIFERATION	GOBP_POSITIVE_REGULATION_OF_EPITHELIAL_CELL_PROLIFERATION	GOBP_POSITIVE_REGULATION_OF_EPITHELIAL_CELL_PROLIFERATION	8	0.525452879	1.682615497	0.0220966	0.2765260	0.2586664	98
GOMF_TRANSCRIPTION_FACTOR_BINDING	GOMF_TRANSCRIPTION_FACTOR_BINDING	GOMF_TRANSCRIPTION_FACTOR_BINDING	87	0.199094109	1.621917747	0.0224050	0.2799684	0.2618864	39
GOBP_INTRINSIC_APOPTOTIC_SIGNALING_PATHWAY_IN_RESPONSE_TO_OXIDATIVE_STI	GOBP_INTRINSIC_APOPTOTIC_SIGNALING_PATHWAY_IN_RESPONSE_TO_OXIDATIVE_STI	GOBP_INTRINSIC_APOPTOTIC_SIGNALING_PATHWAY_IN_RESPONSE_TO_OXIDATIVE_STI	8	0.523597299	1.676673524	0.0225020	0.2799684	0.2618864	98
HP_EPILEPTIC_ENCEPHALOPATHY	HP_EPILEPTIC_ENCEPHALOPATHY	HP_EPILEPTIC_ENCEPHALOPATHY	9	-0.56336521	-1.698561002	0.0225451	0.2799684	0.2618864	113
GOBP_NEGATIVE_REGULATION_OF_DNA_RECOMBINATION	GOBP_NEGATIVE_REGULATION_OF_DNA_RECOMBINATION	GOBP_NEGATIVE_REGULATION_OF_DNA_RECOMBINATION	2	-0.86417657	-1.46846341	0.0226034	0.2799684	0.2618864	163
GOBP_REGULATION_OF_GERMINAL_CENTER_FORMATION	GOBP_REGULATION_OF_GERMINAL_CENTER_FORMATION	GOBP_REGULATION_OF_GERMINAL_CENTER_FORMATION	2	-0.86417657	-1.46846341	0.0226034	0.2799684	0.2618864	163
HP_ABNORMAL_MONOCYTE_MORPHOLOGY	HP_ABNORMAL_MONOCYTE_MORPHOLOGY	HP_ABNORMAL_MONOCYTE_MORPHOLOGY	2	-0.865025467	-1.469905909	0.0226034	0.2799684	0.2618864	162
HP_CRANIOFACIAL_HYPEROSTOSIS	HP_CRANIOFACIAL_HYPEROSTOSIS	HP_CRANIOFACIAL_HYPEROSTOSIS	2	-0.865025467	-1.469905909	0.0226034	0.2799684	0.2618864	162
HP_MONOCYTOSIS	HP_MONOCYTOSIS	HP_MONOCYTOSIS	2	-0.865025467	-1.469905909	0.0226034	0.2799684	0.2618864	162
HP_RECURRENT_EAR_INFECTIONS	HP_RECURRENT_EAR_INFECTIONS	HP_RECURRENT_EAR_INFECTIONS	2	-0.865025467	-1.469905909	0.0226034	0.2799684	0.2618864	162
GOBP_REGULATION_OF_INTRINSIC_APOPTOTIC_SIGNALING_PATHWAY_BY_P53_CLASS_	GOBP_REGULATION_OF_INTRINSIC_APOPTOTIC_SIGNALING_PATHWAY_BY_P53_CLASS_	GOBP_REGULATION_OF_INTRINSIC_APOPTOTIC_SIGNALING_PATHWAY_BY_P53_CLASS_	6	0.621220503	1.740493714	0.0227227	0.2809863	0.2628387	154
GOBP_MYELOID_LEUKOCYTE_DIFFERENTIATION	GOBP_MYELOID_LEUKOCYTE_DIFFERENTIATION	GOBP_MYELOID_LEUKOCYTE_DIFFERENTIATION	24	0.334500753	1.744249959	0.0227372	0.2809863	0.2628387	98
HP_ABNORMALITY_OF_THE_THORACIC_CAVITY	HP_ABNORMALITY_OF_THE_THORACIC_CAVITY	HP_ABNORMALITY_OF_THE_THORACIC_CAVITY	9	-0.563080613	-1.697702934	0.0227763	0.2811496	0.2629914	451
GOBP_POSITIVE_REGULATION_OF_INTERLEUKIN_8_PRODUCTION	GOBP_POSITIVE_REGULATION_OF_INTERLEUKIN_8_PRODUCTION	GOBP_POSITIVE_REGULATION_OF_INTERLEUKIN_8_PRODUCTION	5	0.680529541	1.72322886	0.0229112	0.2824937	0.2642486	7
GOBP_POSITIVE_REGULATION_OF_LYMPHOCYTE_MIGRATION	GOBP_POSITIVE_REGULATION_OF_LYMPHOCYTE_MIGRATION	GOBP_POSITIVE_REGULATION_OF_LYMPHOCYTE_MIGRATION	4	-0.712110879	-1.594038772	0.0230993	0.2827144	0.2644551	41
GOBP_POSITIVE_REGULATION_OF_T_CELL_MIGRATION	GOBP_POSITIVE_REGULATION_OF_T_CELL_MIGRATION	GOBP_POSITIVE_REGULATION_OF_T_CELL_MIGRATION	4	-0.712110879	-1.594038772	0.0230993	0.2827144	0.2644551	41
GOBP_ADRENAL_GLAND_DEVELOPMENT	GOBP_ADRENAL_GLAND_DEVELOPMENT	GOBP_ADRENAL_GLAND_DEVELOPMENT	2	0.89049236	1.554536387	0.0232022	0.2827144	0.2644551	131
GOBP_C21_STEROID_HORMONE_BIOSYNTHETIC_PROCESS	GOBP_C21_STEROID_HORMONE_BIOSYNTHETIC_PROCESS	GOBP_C21_STEROID_HORMONE_BIOSYNTHETIC_PROCESS	2	0.89049236	1.554536387	0.0232022	0.2827144	0.2644551	131
GOBP_C21_STEROID_HORMONE_METABOLIC_PROCESS	GOBP_C21_STEROID_HORMONE_METABOLIC_PROCESS	GOBP_C21_STEROID_HORMONE_METABOLIC_PROCESS	2	0.89049236	1.554536387	0.0232022	0.2827144	0.2644551	131
GOBP_CELLULAR_HORMONE_METABOLIC_PROCESS	GOBP_CELLULAR_HORMONE_METABOLIC_PROCESS	GOBP_CELLULAR_HORMONE_METABOLIC_PROCESS	2	0.89049236	1.554536387	0.0232022	0.2827144	0.2644551	131
GOBP_GLIAL_CELL_PROLIFERATION	GOBP_GLIAL_CELL_PROLIFERATION	GOBP_GLIAL_CELL_PROLIFERATION	2	0.89049236	1.554536387	0.0232022	0.2827144	0.2644551	131
GOBP_POSITIVE_REGULATION_OF_GLIAL_CELL_PROLIFERATION	GOBP_POSITIVE_REGULATION_OF_GLIAL_CELL_PROLIFERATION	GOBP_POSITIVE_REGULATION_OF_GLIAL_CELL_PROLIFERATION	2	0.89049236	1.554536387	0.0232022	0.2827144	0.2644551	131
GOBP_REGULATION_OF_GLIAL_CELL_PROLIFERATION	GOBP_REGULATION_OF_GLIAL_CELL_PROLIFERATION	GOBP_REGULATION_OF_GLIAL_CELL_PROLIFERATION	2	0.89049236	1.554536387	0.0232022	0.2827144	0.2644551	131
GOBP_STEROID_HORMONE_BIOSYNTHETIC_PROCESS	GOBP_STEROID_HORMONE_BIOSYNTHETIC_PROCESS	GOBP_STEROID_HORMONE_BIOSYNTHETIC_PROCESS	2	0.89049236	1.554536387	0.0232022	0.2827144	0.2644551	131
GOBP_ANTIMICROBIAL_HUMORAL_IMMUNE_RESPONSE_MEDIATED_BY_ANTIMICROBIA	GOBP_ANTIMICROBIAL_HUMORAL_IMMUNE_RESPONSE_MEDIATED_BY_ANTIMICROBIA	GOBP_ANTIMICROBIAL_HUMORAL_IMMUNE_RESPONSE_MEDIATED_BY_ANTIMICROBIA	4	0.761904762	1.755971506	0.0232150	0.2827144	0.2644551	284
GOMF_RIBOSOME_BINDING	GOMF_RIBOSOME_BINDING	GOMF_RIBOSOME_BINDING	10	0.495726496	1.741546569	0.0233354	0.2837549	0.2654284	600
HP_METOPIC_SYNOSTOSIS	HP_METOPIC_SYNOSTOSIS	HP_METOPIC_SYNOSTOSIS	2	-0.863327674	-1.467020912	0.0233527	0.2837549	0.2654284	164
GOMF_THIOESTERASE_BINDING	GOMF_THIOESTERASE_BINDING	GOMF_THIOESTERASE_BINDING	2	0.887945671	1.550090621	0.0235734	0.2850957	0.2666827	134
GOBP_NEURON_APOPTOTIC_PROCESS	GOBP_NEURON_APOPTOTIC_PROCESS	GOBP_NEURON_APOPTOTIC_PROCESS	19	0.356696471	1.676160287	0.0235924	0.2850957	0.2666827	42
GOBP_CELLULAR_RESPONSE_TO_EPINEPHRINE_STIMULUS	GOBP_CELLULAR_RESPONSE_TO_EPINEPHRINE_STIMULUS	GOBP_CELLULAR_RESPONSE_TO_EPINEPHRINE_STIMULUS	1	-0.994910941	-1.320205271	0.0238562	0.2850957	0.2666827	8
GOBP_NEGATIVE_REGULATION_OF_ADENYLATE_CYCLASE_ACTIVATING_ADRENERGIC_F	GOBP_NEGATIVE_REGULATION_OF_ADENYLATE_CYCLASE_ACTIVATING_ADRENERGIC_F	GOBP_NEGATIVE_REGULATION_OF_ADENYLATE_CYCLASE_ACTIVATING_ADRENERGIC_F	1	-0.994910941	-1.320205271	0.0238562	0.2850957	0.2666827	8
GOBP_NEGATIVE_REGULATION_OF_ADENYLATE_CYCLASE_ACTIVATING_G_PROTEIN_CO	GOBP_NEGATIVE_REGULATION_OF_ADENYLATE_CYCLASE_ACTIVATING_G_PROTEIN_CO	GOBP_NEGATIVE_REGULATION_OF_ADENYLATE_CYCLASE_ACTIVATING_G_PROTEIN_CO	1	-0.994910941	-1.320205271	0.0238562	0.2850957	0.2666827	8
GOBP_NEGATIVE_REGULATION_OF_RELAXATION_OF_MUSCLE	GOBP_NEGATIVE_REGULATION_OF_RELAXATION_OF_MUSCLE	GOBP_NEGATIVE_REGULATION_OF_RELAXATION_OF_MUSCLE	1	-0.994910941	-1.320205271	0.0238562	0.2850957	0.2666827	8
GOBP_PLASMA_MEMBRANE_FUSION	GOBP_PLASMA_MEMBRANE_FUSION	GOBP_PLASMA_MEMBRANE_FUSION	1	-0.994062765	-1.319079776	0.0238562	0.2850957	0.2666827	9
GOBP_REGULATION_OF_ADENYLATE_CYCLASE_ACTIVATING_G_PROTEIN_COUPLED_RE	GOBP_REGULATION_OF_ADENYLATE_CYCLASE_ACTIVATING_G_PROTEIN_COUPLED_RE	GOBP_REGULATION_OF_ADENYLATE_CYCLASE_ACTIVATING_G_PROTEIN_COUPLED_RE	1	-0.994910941	-1.320205271	0.0238562	0.2850957	0.2666827	8
GOBP_RESPONSE_TO_EPINEPHRINE	GOBP_RESPONSE_TO_EPINEPHRINE	GOBP_RESPONSE_TO_EPINEPHRINE	1	-0.994910941	-1.320205271	0.0238562	0.2850957	0.2666827	8
GOCC_DENDRITIC_SHAFT	GOCC_DENDRITIC_SHAFT	GOCC_DENDRITIC_SHAFT	1	-0.994062765	-1.319079776	0.0238562	0.2850957	0.2666827	9
GOCC_EXCITATORY_SYNAPSE	GOCC_EXCITATORY_SYNAPSE	GOCC_EXCITATORY_SYNAPSE	1	-0.994910941	-1.320205271	0.0238562	0.2850957	0.2666827	8
GOCC_GAMMA_TUBULIN_COMPLEX	GOCC_GAMMA_TUBULIN_COMPLEX	GOCC_GAMMA_TUBULIN_COMPLEX	1	-0.994910941	-1.320205271	0.0238562	0.2850957	0.2666827	8
GOMF_CAMP_BINDING	GOMF_CAMP_BINDING	GOMF_CAMP_BINDING	1	-0.994910941	-1.320205271	0.0238562	0.2850957	0.2666827	8
GOMF_CYCLIC_NUCLEOTIDE_BINDING	GOMF_CYCLIC_NUCLEOTIDE_BINDING	GOMF_CYCLIC_NUCLEOTIDE_BINDING	1	-0.994910941	-1.320205271	0.0238562	0.2850957	0.2666827	8
GOMF_GAMMA_TUBULIN_BINDING	GOMF_GAMMA_TUBULIN_BINDING	GOMF_GAMMA_TUBULIN_BINDING	1	-0.994910941	-1.320205271	0.0238562	0.2850957	0.2666827	8
GOBP_REGULATION_OF_NUCLEASE_ACTIVITY	GOBP_REGULATION_OF_NUCLEASE_ACTIVITY	GOBP_REGULATION_OF_NUCLEASE_ACTIVITY	6	0.619831271	1.736601456	0.0239292	0.2856540	0.2672048	277
GOBP_RESPONSE_TO_STARVATION	GOBP_RESPONSE_TO_STARVATION	GOBP_RESPONSE_TO_STARVATION	24	0.331577289	1.729005594	0.0240075	0.2862738	0.2677846	98
GOBP_RIBOSOMAL_LARGE_SUBUNIT_BIOGENESIS	GOBP_RIBOSOMAL_LARGE_SUBUNIT_BIOGENESIS	GOBP_RIBOSOMAL_LARGE_SUBUNIT_BIOGENESIS	15	0.427667544	1.804832071	0.0240776	0.2864820	0.2679794	313
GOCC_PIGMENT_GRANULE	GOCC_PIGMENT_GRANULE	GOCC_PIGMENT_GRANULE	15	0.428445933	1.808117006	0.0240776	0.2864820	0.2679794	34
GOBP_REGULATION_OF_ENDOPLASMIC_RETICULUM_STRESS_INDUCED_INTRINSIC_AP	GOBP_REGULATION_OF_ENDOPLASMIC_RETICULUM_STRESS_INDUCED_INTRINSIC_AP	GOBP_REGULATION_OF_ENDOPLASMIC_RETICULUM_STRESS_INDUCED_INTRINSIC_AP	8	0.519053461	1.662123156	0.0241238	0.2867175	0.2681996	98
HP_ARNOLD_CHIARI_MALFORMATION	HP_ARNOLD_CHIARI_MALFORMATION	HP_ARNOLD_CHIARI_MALFORMATION	11	-0.517137442	-1.677345083	0.0243745	0.2888681	0.2702114	190
GOBP_REGULATION_OF_ALPHA_BETA_T_CELL_DIFFERENTIATION	GOBP_REGULATION_OF_ALPHA_BETA_T_CELL_DIFFERENTIATION	GOBP_REGULATION_OF_ALPHA_BETA_T_CELL_DIFFERENTIATION	10	-0.548816246	-1.732555359	0.0244114	0.2888681	0.2702114	276
GOBP_CELLULAR_RESPONSE_TO_EXTRACELLULAR_STIMULUS	GOBP_CELLULAR_RESPONSE_TO_EXTRACELLULAR_STIMULUS	GOBP_CELLULAR_RESPONSE_TO_EXTRACELLULAR_STIMULUS	27	0.317654956	1.714716268	0.0244283	0.2888681	0.2702114	36
HP_FINGER_CLINODACTYLY	HP_FINGER_CLINODACTYLY	HP_FINGER_CLINODACTYLY	36	-0.340383266	-1.640866953	0.0244515	0.2888681	0.2702114	441
GOBP_POSITIVE_REGULATION_OF_CATALYTIC_ACTIVITY	GOBP_POSITIVE_REGULATION_OF_CATALYTIC_ACTIVITY	GOBP_POSITIVE_REGULATION_OF_CATALYTIC_ACTIVITY	99	0.181525293	1.51737609	0.0244637	0.2888681	0.2702114	56
GOBP_CHONDROCYTE_DIFFERENTIATION	GOBP_CHONDROCYTE_DIFFERENTIATION	GOBP_CHONDROCYTE_DIFFERENTIATION	8	-0.588296581	-1.688493955	0.0244641	0.2888681	0.2702114	315
GOBP_VIRION_ASSEMBLY	GOBP_VIRION_ASSEMBLY	GOBP_VIRION_ASSEMBLY	4	-0.709176608	-1.587470494	0.0247772	0.2922483	0.2733732	145
GOBP_LEUKOCYTE_CHEMOTAXIS	GOBP_LEUKOCYTE_CHEMOTAXIS	GOBP_LEUKOCYTE_CHEMOTAXIS	18	-0.443248198	-1.721382175	0.0248454	0.2927353	0.2738288	136
GOBP_CELLULAR_RESPONSE_TO_OXYGEN_LEVELS	GOBP_CELLULAR_RESPONSE_TO_OXYGEN_LEVELS	GOBP_CELLULAR_RESPONSE_TO_OXYGEN_LEVELS	15	0.426135574	1.798366885	0.0250126	0.2942816	0.2752753	242
HP_IMPAIRED_TANDEM_GAIT	HP_IMPAIRED_TANDEM_GAIT	HP_IMPAIRED_TANDEM_GAIT	7	-0.578724243	-1.584897213	0.0250579	0.2942816	0.2752753	177
HP_PULMONARY_ARTERY_DILATATION	HP_PULMONARY_ARTERY_DILATATION	HP_PULMONARY_ARTERY_DILATATION	7	-0.578724243	-1.584897213	0.0250579	0.2942816	0.2752753	177
GOBP_OLEFINIC_COMPOUND_METABOLIC_PROCESS	GOBP_OLEFINIC_COMPOUND_METABOLIC_PROCESS	GOBP_OLEFINIC_COMPOUND_METABOLIC_PROCESS	4	0.756667517	1.743901163	0.0251992	0.2956219	0.2765290	75
GOBP_REGULATION_OF_PROTEIN_COMPLEX_STABILITY	GOBP_REGULATION_OF_PROTEIN_COMPLEX_STABILITY	GOBP_REGULATION_OF_PROTEIN_COMPLEX_STABILITY	1	0.995759118	1.327253094	0.0256039	0.2981190	0.2788648	6
GOBP_VESICLE_UNCOATING	GOBP_VESICLE_UNCOATING	GOBP_VESICLE_UNCOATING	1	0.995759118	1.327253094	0.0256039	0.2981190	0.2788648	6
GOCC_GLYCINERGIC_SYNAPSE	GOCC_GLYCINERGIC_SYNAPSE	GOCC_GLYCINERGIC_SYNAPSE	1	0.995759118	1.327253094	0.0256039	0.2981190	0.2788648	6
GOCC_PHOTORECEPTOR_RIBBON_SYNAPSE	GOCC_PHOTORECEPTOR_RIBBON_SYNAPSE	GOCC_PHOTORECEPTOR_RIBBON_SYNAPSE	1	0.995759118	1.327253094	0.0256039	0.2981190	0.2788648	6
GOCC_POSTSYNAPTIC_CYTOSOL	GOCC_POSTSYNAPTIC_CYTOSOL	GOCC_POSTSYNAPTIC_CYTOSOL	1	0.995759118	1.327253094	0.0256039	0.2981190	0.2788648	6
GOCC_RIBBON_SYNAPSE	GOCC_RIBBON_SYNAPSE	GOCC_RIBBON_SYNAPSE	1	0.995759118	1.327253094	0.0256039	0.2981190	0.2788648	6
GOMF_PROTEIN_CARRIER_CHAPERONE	GOMF_PROTEIN_CARRIER_CHAPERONE	GOMF_PROTEIN_CARRIER_CHAPERONE	1	0.995759118	1.327253094	0.0256039	0.2981190	0.2788648	6
GOBP_ANTERIOR_POSTERIOR_AXIS_SPECIFICATION	GOBP_ANTERIOR_POSTERIOR_AXIS_SPECIFICATION	GOBP_ANTERIOR_POSTERIOR_AXIS_SPECIFICATION	4	-0.707482993	-1.583679389	0.0257394	0.2983518	0.2790825	349
GOMF_NON_MEMBRANE_SPANNING_PROTEIN_TYROSINE_KINASE_ACTIVITY	GOMF_NON_MEMBRANE_SPANNING_PROTEIN_TYROSINE_KINASE_ACTIVITY	GOMF_NON_MEMBRANE_SPANNING_PROTEIN_TYROSINE_KINASE_ACTIVITY	4	-0.70811267	-1.585088901	0.0257394	0.2983518	0.2790825	142
HP_APLASIA_HYPOPLASIA_OF_THE_EAR	HP_APLASIA_HYPOPLASIA_OF_THE_EAR	HP_APLASIA_HYPOPLASIA_OF_THE_EAR	26	0.321024243	1.707094049	0.0257687	0.2983518	0.2790825	274
GOBP_POSITIVE_REGULATION_OF_NUCLEOBASE_CONTAINING_COMPOUND_METABOLI	GOBP_POSITIVE_REGULATION_OF_NUCLEOBASE_CONTAINING_COMPOUND_METABOLI	GOBP_POSITIVE_REGULATION_OF_NUCLEOBASE_CONTAINING_COMPOUND_METABOLI	205	0.145470854	1.402807824	0.0258178	0.2983518	0.2790825	75
GOBP_TISSUE_REMODELING	GOBP_TISSUE_REMODELING	GOBP_TISSUE_REMODELING	12	-0.484608421	-1.613166785	0.0258195	0.2983518	0.2790825	265
GOBP_REGULATION_OF_DEACETYLASE_ACTIVITY	GOBP_REGULATION_OF_DEACETYLASE_ACTIVITY	GOBP_REGULATION_OF_DEACETYLASE_ACTIVITY	2	-0.858234295	-1.458365921	0.0258527	0.2983518	0.2790825	170
GOBP_REGULATION_OF_HISTONE_DEACETYLASE_ACTIVITY	GOBP_REGULATION_OF_HISTONE_DEACETYLASE_ACTIVITY	GOBP_REGULATION_OF_HISTONE_DEACETYLASE_ACTIVITY	2	-0.858234295	-1.458365921	0.0258527	0.2983518	0.2790825	170
GOBP_REGULATION_OF_RELAXATION_OF_CARDIAC_MUSCLE	GOBP_REGULATION_OF_RELAXATION_OF_CARDIAC_MUSCLE	GOBP_REGULATION_OF_RELAXATION_OF_CARDIAC_MUSCLE	2	-0.858234295	-1.458365921	0.0258527	0.2983518	0.2790825	170
GOBP_RESPONSE_TO_EXTRACELLULAR_STIMULUS	GOBP_RESPONSE_TO_EXTRACELLULAR_STIMULUS	GOBP_RESPONSE_TO_EXTRACELLULAR_STIMULUS	38	0.261705753	1.626480938	0.0258727	0.2983518	0.2790825	57
GOBP_REGULATION_OF_CELLULAR_COMPONENT_BIOGENESIS	GOBP_REGULATION_OF_CELLULAR_COMPONENT_BIOGENESIS	GOBP_REGULATION_OF_CELLULAR_COMPONENT_BIOGENESIS	78	0.202455905	1.582498186	0.0258983	0.2983518	0.2790825	34
HP_RENAL_AGENESIS	HP_RENAL_AGENESIS	HP_RENAL_AGENESIS	22	0.356688007	1.75778519	0.0259630	0.2987812	0.2794842	274
HP_HIGH_ANTERIOR_HAIRLINE	HP_HIGH_ANTERIOR_HAIRLINE	HP_HIGH_ANTERIOR_HAIRLINE	7	-0.576119029	-1.577762561	0.0260124	0.2990334	0.2797202	68
GOBP_POSITIVE_REGULATION_OF_TRANSCRIPTION_BY_RNA_POLYMERASE_II	GOBP_POSITIVE_REGULATION_OF_TRANSCRIPTION_BY_RNA_POLYMERASE_II	GOBP_POSITIVE_REGULATION_OF_TRANSCRIPTION_BY_RNA_POLYMERASE_II	116	0.169185392	1.44797874	0.0262321	0.3012404	0.2817846	82
GOBP_NEGATIVE_REGULATION_OF_MITOCHONDRION_ORGANIZATION	GOBP_NEGATIVE_REGULATION_OF_MITOCHONDRION_ORGANIZATION	GOBP_NEGATIVE_REGULATION_OF_MITOCHONDRION_ORGANIZATION	7	0.561752164	1.69044897	0.0263422	0.3016923	0.2822074	131
GOBP_POSITIVE_REGULATION_OF_ERYTHROCYTE_DIFFERENTIATION	GOBP_POSITIVE_REGULATION_OF_ERYTHROCYTE_DIFFERENTIATION	GOBP_POSITIVE_REGULATION_OF_ERYTHROCYTE_DIFFERENTIATION	7	0.561257379	1.688960043	0.0263422	0.3016923	0.2822074	7
GOBP_NEGATIVE_REGULATION_OF_SODIUM_ION_TRANSPORT	GOBP_NEGATIVE_REGULATION_OF_SODIUM_ION_TRANSPORT	GOBP_NEGATIVE_REGULATION_OF_SODIUM_ION_TRANSPORT	2	-0.858234295	-1.458365921	0.0263547	0.3016923	0.2822074	170
GOBP_REGULATION_OF_TRANSFERASE_ACTIVITY	GOBP_REGULATION_OF_TRANSFERASE_ACTIVITY	GOBP_REGULATION_OF_TRANSFERASE_ACTIVITY	82	0.196318478	1.572217471	0.0264302	0.3022387	0.2827184	57
GOBP_POSITIVE_REGULATION_OF_INTRACELLULAR_SIGNAL_TRANSDUCTION	GOBP_POSITIVE_REGULATION_OF_INTRACELLULAR_SIGNAL_TRANSDUCTION	GOBP_POSITIVE_REGULATION_OF_INTRACELLULAR_SIGNAL_TRANSDUCTION	92	0.194659783	1.612292834	0.0264706	0.3023830	0.2828534	51
GOBP_CELLULAR_RESPONSE_TO_LIPID	GOBP_CELLULAR_RESPONSE_TO_LIPID	GOBP_CELLULAR_RESPONSE_TO_LIPID	51	0.244697001	1.647665277	0.0265394	0.3028504	0.2832906	57
GOBP_REGULATION_OF_TRANSCRIPTION_BY_RNA_POLYMERASE_III	GOBP_REGULATION_OF_TRANSCRIPTION_BY_RNA_POLYMERASE_III	GOBP_REGULATION_OF_TRANSCRIPTION_BY_RNA_POLYMERASE_III	4	-0.704081633	-1.576065546	0.0267016	0.3039138	0.2842853	353
GOMF_CYSTEINE_TYPE_ENDOPEPTIDASE_INHIBITOR_ACTIVITY	GOMF_CYSTEINE_TYPE_ENDOPEPTIDASE_INHIBITOR_ACTIVITY	GOMF_CYSTEINE_TYPE_ENDOPEPTIDASE_INHIBITOR_ACTIVITY	4	-0.701848681	-1.57106715	0.0267016	0.3039138	0.2842853	209
GOBP_DEVELOPMENT_OF_PRIMARY_FEMALE_SEXUAL_CHARACTERISTICS	GOBP_DEVELOPMENT_OF_PRIMARY_FEMALE_SEXUAL_CHARACTERISTICS	GOBP_DEVELOPMENT_OF_PRIMARY_FEMALE_SEXUAL_CHARACTERISTICS	7	0.560739001	1.687400115	0.0267444	0.3039138	0.2842853	152
GOBP_REGULATION_OF_EXTRINSIC_APOPTOTIC_SIGNALING_PATHWAY_VIA_DEATH_DO	GOBP_REGULATION_OF_EXTRINSIC_APOPTOTIC_SIGNALING_PATHWAY_VIA_DEATH_DO	GOBP_REGULATION_OF_EXTRINSIC_APOPTOTIC_SIGNALING_PATHWAY_VIA_DEATH_DO	7	0.560729835	1.687372535	0.0267444	0.3039138	0.2842853	42
GOCC_ORGANELLAR_RIBOSOME	GOCC_ORGANELLAR_RIBOSOME	GOCC_ORGANELLAR_RIBOSOME	5	0.66950526	1.695313308	0.0268275	0.3042362	0.2845869	52
HP_CERVICAL_RIBS	HP_CERVICAL_RIBS	HP_CERVICAL_RIBS	2	-0.855687606	-1.454038426	0.0268567	0.3042362	0.2845869	173
HP_VERTEBRAL_FUSION	HP_VERTEBRAL_FUSION	HP_VERTEBRAL_FUSION	2	-0.855687606	-1.454038426	0.0268567	0.3042362	0.2845869	173
GOBP_REGULATION_OF_RAS_PROTEIN_SIGNAL_TRANSDUCTION	GOBP_REGULATION_OF_RAS_PROTEIN_SIGNAL_TRANSDUCTION	GOBP_REGULATION_OF_RAS_PROTEIN_SIGNAL_TRANSDUCTION	19	-0.422083271	-1.651424083	0.0270071	0.3056218	0.2858830	163
GOBP_REGULATION_OF_RESPONSE_TO_ENDOPLASMIC_RETICULUM_STRESS	GOBP_REGULATION_OF_RESPONSE_TO_ENDOPLASMIC_RETICULUM_STRESS	GOBP_REGULATION_OF_RESPONSE_TO_ENDOPLASMIC_RETICULUM_STRESS	17	0.384739545	1.704711521	0.0271324	0.3059555	0.2861951	123
GOBP_MATERNAL_PLACENTA_DEVELOPMENT	GOBP_MATERNAL_PLACENTA_DEVELOPMENT	GOBP_MATERNAL_PLACENTA_DEVELOPMENT	6	0.614298884	1.721101188	0.0271465	0.3059555	0.2861951	251
GOBP_MATERNAL_PROCESS_INVOLVED_IN_FEMALE_PREGNANCY	GOBP_MATERNAL_PROCESS_INVOLVED_IN_FEMALE_PREGNANCY	GOBP_MATERNAL_PROCESS_INVOLVED_IN_FEMALE_PREGNANCY	6	0.614298884	1.721101188	0.0271465	0.3059555	0.2861951	251
HP_PULMONARY_INFILTRATES	HP_PULMONARY_INFILTRATES	HP_PULMONARY_INFILTRATES	10	-0.539663198	-1.703660147	0.0271491	0.3059555	0.2861951	218
GOBP_NEGATIVE_REGULATION_OF_ATP_METABOLIC_PROCESS	GOBP_NEGATIVE_REGULATION_OF_ATP_METABOLIC_PROCESS	GOBP_NEGATIVE_REGULATION_OF_ATP_METABOLIC_PROCESS	4	0.74744898	1.722655082	0.0271833	0.3060240	0.2862592	301
HP_ABNORMAL_HAIR_WHORL	HP_ABNORMAL_HAIR_WHORL	HP_ABNORMAL_HAIR_WHORL	13	-0.489001365	-1.676150787	0.0273563	0.3076529	0.2877830	437
GOBP_REGULATION_OF_VASCULATURE_DEVELOPMENT	GOBP_REGULATION_OF_VASCULATURE_DEVELOPMENT	GOBP_REGULATION_OF_VASCULATURE_DEVELOPMENT	25	0.326501959	1.72324646	0.0276957	0.3111476	0.2910519	23
GOBP_MODULATION_BY_HOST_OF_SYMBIONT_PROCESS	GOBP_MODULATION_BY_HOST_OF_SYMBIONT_PROCESS	GOBP_MODULATION_BY_HOST_OF_SYMBIONT_PROCESS	13	0.429327045	1.711614828	0.0277463	0.3113942	0.2912826	6
GOBP_REGULATION_OF_RIBONUCLEASE_ACTIVITY	GOBP_REGULATION_OF_RIBONUCLEASE_ACTIVITY	GOBP_REGULATION_OF_RIBONUCLEASE_ACTIVITY	2	0.874426967	1.526490961	0.0280282	0.3142338	0.2939388	2

**GOBP_REGULATION_OF_ERYTHROCYTE_DIFFERENTIATION**	GOBP_REGULATION_OF_ERYTHROCYTE_DIFFERENTIATION	GOBP_REGULATION_OF_ERYTHROCYTE_DIFFERENTIATION	10	0.486045893	1.707537453	0.0281332	0.3150868	0.2947367	57
**HP_COLITIS**	HP_COLITIS	HP_COLITIS	8	-0.579390789	-1.66293308	0.0282642	0.3162285	0.2958047	331
**GOBP_RIBOSOME_BIOGENESIS**	GOBP_RIBOSOME_BIOGENESIS	GOBP_RIBOSOME_BIOGENESIS	49	0.240644301	1.603651935	0.0284785	0.3182982	0.2977408	287
**GOBP_CYTOKINE_PRODUCTION**	GOBP_CYTOKINE_PRODUCTION	GOBP_CYTOKINE_PRODUCTION	86	0.191397917	1.563776108	0.0287594	0.3211076	0.3003687	28
**GOCC_DENDRITIC_TREE**	GOCC_DENDRITIC_TREE	GOCC_DENDRITIC_TREE	30	0.305318235	1.727781928	0.0288955	0.3220762	0.3012747	20
**GOBP_REGULATION_OF_CELLULAR_MACROMOLECULE_BIOSYNTHETIC_PROCESS**	GOBP_REGULATION_OF_CELLULAR_MACROMOLECULE_BIOSYNTHETIC_PROCESS	GOBP_REGULATION_OF_CELLULAR_MACROMOLECULE_BIOSYNTHETIC_PROCESS	85	0.19371884	1.57778567	0.0289054	0.3220762	0.3012747	57
**GOBP_REGULATION_OF_NEURON_PROJECTION_ARBORIZATION**	GOBP_REGULATION_OF_NEURON_PROJECTION_ARBORIZATION	GOBP_REGULATION_OF_NEURON_PROJECTION_ARBORIZATION	1	-0.991518236	-1.315703292	0.0289858	0.3226424	0.3018043	12
**HP_ABNORMAL_LARYNX_MORPHOLOGY**	HP_ABNORMAL_LARYNX_MORPHOLOGY	HP_ABNORMAL_LARYNX_MORPHOLOGY	18	-0.43291978	-1.681271116	0.0290924	0.3234977	0.3026044	430
**GOBP_REGULATION_OF_PROTEASOMAL_UBIQUITIN_DEPENDENT_PROTEIN_CATABOLIC**	GOBP_REGULATION_OF_PROTEASOMAL_UBIQUITIN_DEPENDENT_PROTEIN_CATABOLIC	GOBP_REGULATION_OF_PROTEASOMAL_UBIQUITIN_DEPENDENT_PROTEIN_CATABOLIC	19	0.345321641	1.622708572	0.0291609	0.3239277	0.3030067	20
**HP_TAPERED_FINGER**	HP_TAPERED_FINGER	HP_TAPERED_FINGER	18	-0.432663912	-1.680277439	0.0295171	0.3275504	0.3063953	433
**GOBP_CELLULAR_RESPONSE_TO_ENDOGENOUS_STIMULUS**	GOBP_CELLULAR_RESPONSE_TO_ENDOGENOUS_STIMULUS	GOBP_CELLULAR_RESPONSE_TO_ENDOGENOUS_STIMULUS	110	0.18203391	1.542673553	0.0295556	0.3276423	0.3064814	111
**GOBP_POSITIVE_REGULATION_OF_HYDROLASE_ACTIVITY**	GOBP_POSITIVE_REGULATION_OF_HYDROLASE_ACTIVITY	GOBP_POSITIVE_REGULATION_OF_HYDROLASE_ACTIVITY	60	0.214959546	1.53996891	0.0302083	0.3345374	0.3129311	46
**HP_ECTRODACTYLY**	HP_ECTRODACTYLY	HP_ECTRODACTYLY	2	0.870416135	1.519489234	0.0302555	0.3347197	0.3131016	3
**GOMF_CATION_CHANNEL_ACTIVITY**	GOMF_CATION_CHANNEL_ACTIVITY	GOMF_CATION_CHANNEL_ACTIVITY	10	-0.53102381	-1.676386504	0.0303431	0.3350464	0.3134072	91
**GOBP_REGULATION_OF_SPROUTING_ANGIOGENESIS**	GOBP_REGULATION_OF_SPROUTING_ANGIOGENESIS	GOBP_REGULATION_OF_SPROUTING_ANGIOGENESIS	3	0.804057078	1.646419834	0.0303467	0.3350464	0.3134072	16
**GOBP_RRNA_METABOLIC_PROCESS**	GOBP_RRNA_METABOLIC_PROCESS	GOBP_RRNA_METABOLIC_PROCESS	39	0.258481854	1.612084177	0.0308850	0.3406444	0.3186437	274
**HP_ABNORMALITY_OF_THE_ABDOMINAL_ORGANS**	HP_ABNORMALITY_OF_THE_ABDOMINAL_ORGANS	HP_ABNORMALITY_OF_THE_ABDOMINAL_ORGANS	115	-0.246292369	-1.587817613	0.0309892	0.3414471	0.3193945	607
**GOBP_NUCLEOSIDE_PHOSPHATE_CATABOLIC_PROCESS**	GOBP_NUCLEOSIDE_PHOSPHATE_CATABOLIC_PROCESS	GOBP_NUCLEOSIDE_PHOSPHATE_CATABOLIC_PROCESS	4	-0.69085333	-1.546454388	0.0310315	0.3415670	0.3195067	183
**GOBP_ALPHA_BETA_T_CELL_DIFFERENTIATION**	GOBP_ALPHA_BETA_T_CELL_DIFFERENTIATION	GOBP_ALPHA_BETA_T_CELL_DIFFERENTIATION	15	-0.470571202	-1.684731916	0.0310833	0.3417913	0.3197165	276
**GOBP_CELLULAR_RESPONSE_TO_VASCULAR_ENDOTHELIAL_GROWTH_FACTOR_STIMU**	GOBP_CELLULAR_RESPONSE_TO_VASCULAR_ENDOTHELIAL_GROWTH_FACTOR_STIMU	GOBP_CELLULAR_RESPONSE_TO_VASCULAR_ENDOTHELIAL_GROWTH_FACTOR_STIMU	3	0.803282646	1.644834076	0.0311198	0.3418472	0.3197688	27
**GOBP_T_CELL_MIGRATION**	GOBP_T_CELL_MIGRATION	GOBP_T_CELL_MIGRATION	7	-0.568315495	-1.556391763	0.0312625	0.3430680	0.3209107	136
**GOBP_RESPONSE_TO_CYTOKINE**	GOBP_RESPONSE_TO_CYTOKINE	GOBP_RESPONSE_TO_CYTOKINE	96	0.187353513	1.563260922	0.0313104	0.3432470	0.3210782	58
**GOBP_REGULATION_OF_ATP_DEPENDENT_ACTIVITY**	GOBP_REGULATION_OF_ATP_DEPENDENT_ACTIVITY	GOBP_REGULATION_OF_ATP_DEPENDENT_ACTIVITY	8	0.504690767	1.616130656	0.0314216	0.3441190	0.3218938	125
**GOMF_RIBONUCLEOTIDE_BINDING**	GOMF_RIBONUCLEOTIDE_BINDING	GOMF_RIBONUCLEOTIDE_BINDING	143	0.159593484	1.422841451	0.0314923	0.3445467	0.3222939	58
**GOBP_CARDIAC_MUSCLE_CELL_DIFFERENTIATION**	GOBP_CARDIAC_MUSCLE_CELL_DIFFERENTIATION	GOBP_CARDIAC_MUSCLE_CELL_DIFFERENTIATION	5	-0.675503194	-1.633839863	0.0317668	0.3465117	0.3241320	144
**GOBP_REGULATION_OF_MAST_CELL_ACTIVATION_INVOLVED_IN_IMMUNE_RESPONSE**	GOBP_REGULATION_OF_MAST_CELL_ACTIVATION_INVOLVED_IN_IMMUNE_RESPONSE	GOBP_REGULATION_OF_MAST_CELL_ACTIVATION_INVOLVED_IN_IMMUNE_RESPONSE	5	-0.673384883	-1.628716304	0.0317668	0.3465117	0.3241320	266
**GOBP_VIRAL_LIFE_CYCLE**	GOBP_VIRAL_LIFE_CYCLE	GOBP_VIRAL_LIFE_CYCLE	35	0.268885822	1.611581884	0.0317777	0.3465117	0.3241320	20
**GOBP_ESTABLISHMENT_OF_PROTEIN_LOCALIZATION_TO_ORGANELLE**	GOBP_ESTABLISHMENT_OF_PROTEIN_LOCALIZATION_TO_ORGANELLE	GOBP_ESTABLISHMENT_OF_PROTEIN_LOCALIZATION_TO_ORGANELLE	48	0.227164686	1.49743586	0.0317994	0.3465117	0.3241320	46
**GOBP_POSITIVE_REGULATION_OF_CYTOKINE_PRODUCTION**	GOBP_POSITIVE_REGULATION_OF_CYTOKINE_PRODUCTION	GOBP_POSITIVE_REGULATION_OF_CYTOKINE_PRODUCTION	53	0.22407161	1.525423616	0.0318786	0.3468368	0.3244361	98
**GOBP_HYPOTHALAMUS_DEVELOPMENT**	GOBP_HYPOTHALAMUS_DEVELOPMENT	GOBP_HYPOTHALAMUS_DEVELOPMENT	3	0.802462585	1.643154885	0.0318930	0.3468368	0.3244361	39
**HP_RECURRENT_SINOPULMONARY_INFECTIONS**	HP_RECURRENT_SINOPULMONARY_INFECTIONS	HP_RECURRENT_SINOPULMONARY_INFECTIONS	4	-0.687849862	-1.539731215	0.0319937	0.3475192	0.3250745	207
**GOBP_POSITIVE_REGULATION_OF_IMMUNE_SYSTEM_PROCESS**	GOBP_POSITIVE_REGULATION_OF_IMMUNE_SYSTEM_PROCESS	GOBP_POSITIVE_REGULATION_OF_IMMUNE_SYSTEM_PROCESS	89	-0.259380906	-1.595060637	0.0320197	0.3475192	0.3250745	178
**GOBP_REGULATION_OF_RAB_PROTEIN_SIGNAL_TRANSDUCTION**	GOBP_REGULATION_OF_RAB_PROTEIN_SIGNAL_TRANSDUCTION	GOBP_REGULATION_OF_RAB_PROTEIN_SIGNAL_TRANSDUCTION	1	-0.990670059	-1.314577797	0.0322365	0.3493484	0.3267856	13
**HP_HORSESHOE_KIDNEY**	HP_HORSESHOE_KIDNEY	HP_HORSESHOE_KIDNEY	23	0.31862141	1.596849293	0.0322524	0.3493484	0.3267856	274
**GOMF_CHROMATIN_DNA_BINDING**	GOMF_CHROMATIN_DNA_BINDING	GOMF_CHROMATIN_DNA_BINDING	15	-0.466094421	-1.66870421	0.0324060	0.3506623	0.3280146	638
**GOBP_NEGATIVE_REGULATION_OF_INTRACELLULAR_SIGNAL_TRANSDUCTION**	GOBP_NEGATIVE_REGULATION_OF_INTRACELLULAR_SIGNAL_TRANSDUCTION	GOBP_NEGATIVE_REGULATION_OF_INTRACELLULAR_SIGNAL_TRANSDUCTION	66	0.213987255	1.584501988	0.0325864	0.3522636	0.3295125	42
**GOBP_REGULATION_OF_PROTEIN_POLYMERIZATION**	GOBP_REGULATION_OF_PROTEIN_POLYMERIZATION	GOBP_REGULATION_OF_PROTEIN_POLYMERIZATION	22	0.345936195	1.704799454	0.0331605	0.3581139	0.3349849	34
**HP_COARCTATION_OF_AORTA**	HP_COARCTATION_OF_AORTA	HP_COARCTATION_OF_AORTA	26	0.306608906	1.630438354	0.0332739	0.3589827	0.3357976	274
**HP_KYPHOSIS**	HP_KYPHOSIS	HP_KYPHOSIS	45	-0.320925703	-1.659952552	0.0333778	0.3597462	0.3365118	239
**GOBP_REGULATION_OF_EXTENT_OF_CELL_GROWTH**	GOBP_REGULATION_OF_EXTENT_OF_CELL_GROWTH	GOBP_REGULATION_OF_EXTENT_OF_CELL_GROWTH	11	-0.49851337	-1.616937555	0.0334862	0.3605573	0.3372704	471
**GOBP_MICROTUBULE_CYTOSKELETON_ORGANIZATION**	GOBP_MICROTUBULE_CYTOSKELETON_ORGANIZATION	GOBP_MICROTUBULE_CYTOSKELETON_ORGANIZATION	37	0.263576879	1.605719256	0.0335328	0.3607024	0.3374062	30
**GOBP_POSITIVE_REGULATION_OF_FIBROBLAST_PROLIFERATION**	GOBP_POSITIVE_REGULATION_OF_FIBROBLAST_PROLIFERATION	GOBP_POSITIVE_REGULATION_OF_FIBROBLAST_PROLIFERATION	3	0.793853851	1.625527293	0.0338259	0.3633916	0.3399217	39
**GOBP_PATTERN_RECOGNITION_RECEPTOR_SIGNALING_PATHWAY**	GOBP_PATTERN_RECOGNITION_RECEPTOR_SIGNALING_PATHWAY	GOBP_PATTERN_RECOGNITION_RECEPTOR_SIGNALING_PATHWAY	21	0.332934021	1.630580993	0.0339342	0.3633916	0.3399217	46
**GOBP_CYTOPLASMIC_PATTERN_RECOGNITION_RECEPTOR_SIGNALING_PATHWAY**	GOBP_CYTOPLASMIC_PATTERN_RECOGNITION_RECEPTOR_SIGNALING_PATHWAY	GOBP_CYTOPLASMIC_PATTERN_RECOGNITION_RECEPTOR_SIGNALING_PATHWAY	13	0.416744434	1.661451243	0.0339613	0.3633916	0.3399217	26
**GOMF_OXIDOREDUCTASE_ACTIVITY_ACTING_ON_METAL_IONS**	GOMF_OXIDOREDUCTASE_ACTIVITY_ACTING_ON_METAL_IONS	GOMF_OXIDOREDUCTASE_ACTIVITY_ACTING_ON_METAL_IONS	2	0.86417657	1.508596799	0.0339678	0.3633916	0.3399217	162
**GOMF_PROTEIN_PHOSPHATASE_1_BINDING**	GOMF_PROTEIN_PHOSPHATASE_1_BINDING	GOMF_PROTEIN_PHOSPHATASE_1_BINDING	1	0.993214589	1.323861476	0.0339711	0.3633916	0.3399217	9
**GOBP_REGULATION_OF_MITOCHONDRIAL_MEMBRANE_PERMEABILITY**	GOBP_REGULATION_OF_MITOCHONDRIAL_MEMBRANE_PERMEABILITY	GOBP_REGULATION_OF_MITOCHONDRIAL_MEMBRANE_PERMEABILITY	7	0.542989629	1.633987936	0.0339833	0.3633916	0.3399217	97
**GOBP_REGULATION_OF_CARBOHYDRATE_BIOSYNTHETIC_PROCESS**	GOBP_REGULATION_OF_CARBOHYDRATE_BIOSYNTHETIC_PROCESS	GOBP_REGULATION_OF_CARBOHYDRATE_BIOSYNTHETIC_PROCESS	14	-0.454110673	-1.600387093	0.0342197	0.3655600	0.3419501	456
**HP_ABNORMAL_SOFT_PALATE_MORPHOLOGY**	HP_ABNORMAL_SOFT_PALATE_MORPHOLOGY	HP_ABNORMAL_SOFT_PALATE_MORPHOLOGY	26	0.305565638	1.624890624	0.0342746	0.3657873	0.3421627	279
**GOBP_ADAPTIVE_IMMUNE_RESPONSE_BASED_ON_SOMATIC_RECOMBINATION_OF_IMM**	GOBP_ADAPTIVE_IMMUNE_RESPONSE_BASED_ON_SOMATIC_RECOMBINATION_OF_IMM	GOBP_ADAPTIVE_IMMUNE_RESPONSE_BASED_ON_SOMATIC_RECOMBINATION_OF_IMM	31	-0.360655722	-1.645761481	0.0343406	0.3661320	0.3424851	276
**HP_ABNORMAL_CIRCULATING_CARBOHYDRATE_CONCENTRATION**	HP_ABNORMAL_CIRCULATING_CARBOHYDRATE_CONCENTRATION	HP_ABNORMAL_CIRCULATING_CARBOHYDRATE_CONCENTRATION	8	-0.569870923	-1.635609726	0.0344395	0.3664677	0.3427992	239
**HP_INCREASED_SERUM_PYRUVATE**	HP_INCREASED_SERUM_PYRUVATE	HP_INCREASED_SERUM_PYRUVATE	8	-0.569870923	-1.635609726	0.0344395	0.3664677	0.3427992	239
**GOBP_REGULATION_OF_TRANSPORTER_ACTIVITY**	GOBP_REGULATION_OF_TRANSPORTER_ACTIVITY	GOBP_REGULATION_OF_TRANSPORTER_ACTIVITY	15	-0.463204156	-1.65835653	0.0346105	0.3679268	0.3441640	77
**GOBP_REGULATION_OF_TUMOR_NECROSIS_FACTOR_MEDIATED_SIGNALING_PATHWAY**	GOBP_REGULATION_OF_TUMOR_NECROSIS_FACTOR_MEDIATED_SIGNALING_PATHWAY	GOBP_REGULATION_OF_TUMOR_NECROSIS_FACTOR_MEDIATED_SIGNALING_PATHWAY	6	0.594525127	1.665700409	0.0347877	0.3694496	0.3455884	7
**HP_ABNORMALITY_OF_THE_FONTANELLES_OR_CRANIAL_SUTURES**	HP_ABNORMALITY_OF_THE_FONTANELLES_OR_CRANIAL_SUTURES	HP_ABNORMALITY_OF_THE_FONTANELLES_OR_CRANIAL_SUTURES	42	-0.322331669	-1.632954278	0.0348993	0.3702733	0.3463590	433
**HP_ABNORMAL_INFLAMMATORY_RESPONSE**	HP_ABNORMAL_INFLAMMATORY_RESPONSE	HP_ABNORMAL_INFLAMMATORY_RESPONSE	109	-0.243996186	-1.556001199	0.0350665	0.3711945	0.3472207	218
**GOMF_MDM2_MDM4_FAMILY_PROTEIN_BINDING**	GOMF_MDM2_MDM4_FAMILY_PROTEIN_BINDING	GOMF_MDM2_MDM4_FAMILY_PROTEIN_BINDING	2	0.862478778	1.505632955	0.0350815	0.3711945	0.3472207	164
**HP_INCOMITANT_STRABISMUS**	HP_INCOMITANT_STRABISMUS	HP_INCOMITANT_STRABISMUS	9	-0.545839051	-1.645719171	0.0350885	0.3711945	0.3472207	177
**HP_UPSLANTED_PALPEBRAL_FISSURE**	HP_UPSLANTED_PALPEBRAL_FISSURE	HP_UPSLANTED_PALPEBRAL_FISSURE	35	-0.339605142	-1.621177049	0.0353260	0.3733442	0.3492315	670
**GOBP_RIG_I_SIGNALING_PATHWAY**	GOBP_RIG_I_SIGNALING_PATHWAY	GOBP_RIG_I_SIGNALING_PATHWAY	8	-0.565664349	-1.623536267	0.0353895	0.3736521	0.3495196	249
**HP_REDUCED_CONSCIOUSNESS_CONFUSION**	HP_REDUCED_CONSCIOUSNESS_CONFUSION	HP_REDUCED_CONSCIOUSNESS_CONFUSION	47	-0.31373478	-1.639175279	0.0356200	0.3757207	0.3514545	94
**HP_ROUND_FACE**	HP_ROUND_FACE	HP_ROUND_FACE	11	-0.495648519	-1.607645355	0.0357641	0.3768754	0.3525347	173
**HP_FRONTAL_CORTICAL_ATROPHY**	HP_FRONTAL_CORTICAL_ATROPHY	HP_FRONTAL_CORTICAL_ATROPHY	10	-0.525982495	-1.660471602	0.0358184	0.3770819	0.3527279	177
**GOBP_RESPONSE_TO_HORMONE**	GOBP_RESPONSE_TO_HORMONE	GOBP_RESPONSE_TO_HORMONE	83	0.182196645	1.464981392	0.0359408	0.3774181	0.3530423	82
**GOBP_NEGATIVE_REGULATION_OF_SPROUTING_ANGIOGENESIS**	GOBP_NEGATIVE_REGULATION_OF_SPROUTING_ANGIOGENESIS	GOBP_NEGATIVE_REGULATION_OF_SPROUTING_ANGIOGENESIS	1	0.992366412	1.322730937	0.0359892	0.3774181	0.3530423	10
**GOBP_POSITIVE_REGULATION_OF_RETINOIC_ACID_RECEPTOR_SIGNALING_PATHWAY**	GOBP_POSITIVE_REGULATION_OF_RETINOIC_ACID_RECEPTOR_SIGNALING_PATHWAY	GOBP_POSITIVE_REGULATION_OF_RETINOIC_ACID_RECEPTOR_SIGNALING_PATHWAY	1	0.992366412	1.322730937	0.0359892	0.3774181	0.3530423	10
**GOBP_TYPE_I_PNEUMOCYTE_DIFFERENTIATION**	GOBP_TYPE_I_PNEUMOCYTE_DIFFERENTIATION	GOBP_TYPE_I_PNEUMOCYTE_DIFFERENTIATION	1	0.992366412	1.322730937	0.0359892	0.3774181	0.3530423	10
**GOBP_TRANSLATIONAL_INITIATION**	GOBP_TRANSLATIONAL_INITIATION	GOBP_TRANSLATIONAL_INITIATION	27	0.304099115	1.641541202	0.0362451	0.3797362	0.3552107	277
**GOBP_POSITIVE_REGULATION_OF_MULTICELLULAR_ORGANISMAL_PROCESS**	GOBP_POSITIVE_REGULATION_OF_MULTICELLULAR_ORGANISMAL_PROCESS	GOBP_POSITIVE_REGULATION_OF_MULTICELLULAR_ORGANISMAL_PROCESS	118	0.165411953	1.433240362	0.0363158	0.3800357	0.3554909	46
**GOCC_SECRETORY_VESICLE**	GOCC_SECRETORY_VESICLE	GOCC_SECRETORY_VESICLE	81	0.190197573	1.51107085	0.0363436	0.3800357	0.3554909	141
**HP_MACULE**	HP_MACULE	HP_MACULE	25	-0.389196642	-1.649500479	0.0364656	0.3807420	0.3561516	437
**GOBP_MITOCHONDRIAL_TRANSPORT**	GOBP_MITOCHONDRIAL_TRANSPORT	GOBP_MITOCHONDRIAL_TRANSPORT	24	0.312818759	1.631189476	0.0365083	0.3807420	0.3561516	206
**HP_CONGENITAL_STATIONARY_NIGHT_BLINDNESS**	HP_CONGENITAL_STATIONARY_NIGHT_BLINDNESS	HP_CONGENITAL_STATIONARY_NIGHT_BLINDNESS	1	-0.988973707	-1.312326808	0.0365707	0.3807420	0.3561516	15
**HP_MULTIFOCAL_CEREBRAL_WHITE_MATTER_ABNORMALITIES**	HP_MULTIFOCAL_CEREBRAL_WHITE_MATTER_ABNORMALITIES	HP_MULTIFOCAL_CEREBRAL_WHITE_MATTER_ABNORMALITIES	1	-0.988973707	-1.312326808	0.0365707	0.3807420	0.3561516	15
**GOBP_TUMOR_NECROSIS_FACTOR_MEDIATED_SIGNALING_PATHWAY**	GOBP_TUMOR_NECROSIS_FACTOR_MEDIATED_SIGNALING_PATHWAY	GOBP_TUMOR_NECROSIS_FACTOR_MEDIATED_SIGNALING_PATHWAY	14	0.406741663	1.644155234	0.0365862	0.3807420	0.3561516	26
**GOBP_POSITIVE_REGULATION_OF_CELLULAR_COMPONENT_BIOGENESIS**	GOBP_POSITIVE_REGULATION_OF_CELLULAR_COMPONENT_BIOGENESIS	GOBP_POSITIVE_REGULATION_OF_CELLULAR_COMPONENT_BIOGENESIS	35	0.265607008	1.591930134	0.0368395	0.3830114	0.3582744	34
**GOBP_ALDEHYDE_CATABOLIC_PROCESS**	GOBP_ALDEHYDE_CATABOLIC_PROCESS	GOBP_ALDEHYDE_CATABOLIC_PROCESS	2	0.859083192	1.499705266	0.0369377	0.3832994	0.3585438	168
**GOBP_AMINE_BIOSYNTHETIC_PROCESS**	GOBP_AMINE_BIOSYNTHETIC_PROCESS	GOBP_AMINE_BIOSYNTHETIC_PROCESS	2	0.859083192	1.499705266	0.0369377	0.3832994	0.3585438	168
**GOBP_CELL_JUNCTION_ASSEMBLY**	GOBP_CELL_JUNCTION_ASSEMBLY	GOBP_CELL_JUNCTION_ASSEMBLY	20	-0.414395531	-1.651243432	0.0370370	0.3839635	0.3591650	318
**GOBP_REGULATION_OF_MEMBRANE_PERMEABILITY**	GOBP_REGULATION_OF_MEMBRANE_PERMEABILITY	GOBP_REGULATION_OF_MEMBRANE_PERMEABILITY	8	0.494985368	1.585051836	0.0370977	0.3842263	0.3594108	97
**GOBP_REGULATION_OF_ORGANELLE_ASSEMBLY**	GOBP_REGULATION_OF_ORGANELLE_ASSEMBLY	GOBP_REGULATION_OF_ORGANELLE_ASSEMBLY	20	0.352259387	1.684537503	0.0371525	0.3844281	0.3595996	34
**GOBP_CELLULAR_RESPONSE_TO_EXTERNAL_STIMULUS**	GOBP_CELLULAR_RESPONSE_TO_EXTERNAL_STIMULUS	GOBP_CELLULAR_RESPONSE_TO_EXTERNAL_STIMULUS	31	0.285818797	1.644658518	0.0374019	0.3866408	0.3616694	36
**GOBP_IMMUNE_EFFECTOR_PROCESS**	GOBP_IMMUNE_EFFECTOR_PROCESS	GOBP_IMMUNE_EFFECTOR_PROCESS	55	-0.294545508	-1.612875411	0.0378096	0.3904842	0.3652645	288
**GOBP_EPITHELIAL_CELL_PROLIFERATION**	GOBP_EPITHELIAL_CELL_PROLIFERATION	GOBP_EPITHELIAL_CELL_PROLIFERATION	23	0.313291416	1.570136718	0.0382150	0.3942962	0.3688303	112
**GOCC_CLATHRIN_SCULPTED_GAMMA_AMINOBUTYRIC_ACID_TRANSPORT_VESICLE**	GOCC_CLATHRIN_SCULPTED_GAMMA_AMINOBUTYRIC_ACID_TRANSPORT_VESICLE	GOCC_CLATHRIN_SCULPTED_GAMMA_AMINOBUTYRIC_ACID_TRANSPORT_VESICLE	2	0.858234295	1.498223344	0.0384263	0.3957254	0.3701673	169
**GOCC_CLATHRIN_SCULPTED_VESICLE**	GOCC_CLATHRIN_SCULPTED_VESICLE	GOCC_CLATHRIN_SCULPTED_VESICLE	2	0.858234295	1.498223344	0.0384263	0.3957254	0.3701673	169
**GOBP_MONOUBIQUITINATED_PROTEIN_DEUBIQUITINATION**	GOBP_MONOUBIQUITINATED_PROTEIN_DEUBIQUITINATION	GOBP_MONOUBIQUITINATED_PROTEIN_DEUBIQUITINATION	5	-0.660922757	-1.598574155	0.0385565	0.3964610	0.3708553	228
**GOBP_POSITIVE_REGULATION_OF_SUPRAMOLECULAR_FIBER_ORGANIZATION**	GOBP_POSITIVE_REGULATION_OF_SUPRAMOLECULAR_FIBER_ORGANIZATION	GOBP_POSITIVE_REGULATION_OF_SUPRAMOLECULAR_FIBER_ORGANIZATION	15	0.40628183	1.714580604	0.0385706	0.3964610	0.3708553	42
**GOBP_POSITIVE_REGULATION_OF_MONONUCLEAR_CELL_MIGRATION**	GOBP_POSITIVE_REGULATION_OF_MONONUCLEAR_CELL_MIGRATION	GOBP_POSITIVE_REGULATION_OF_MONONUCLEAR_CELL_MIGRATION	8	-0.56309237	-1.616154323	0.0387147	0.3968653	0.3712335	91
**GOBP_RNA_DEPENDENT_DNA_BIOSYNTHETIC_PROCESS**	GOBP_RNA_DEPENDENT_DNA_BIOSYNTHETIC_PROCESS	GOBP_RNA_DEPENDENT_DNA_BIOSYNTHETIC_PROCESS	8	0.488110113	1.563035761	0.0387194	0.3968653	0.3712335	132
**GOBP_SMAD_PROTEIN_SIGNAL_TRANSDUCTION**	GOBP_SMAD_PROTEIN_SIGNAL_TRANSDUCTION	GOBP_SMAD_PROTEIN_SIGNAL_TRANSDUCTION	8	0.490614615	1.571055727	0.0387194	0.3968653	0.3712335	13
**HP_PROMINENT_FOREHEAD**	HP_PROMINENT_FOREHEAD	HP_PROMINENT_FOREHEAD	29	-0.363934676	-1.625658354	0.0388349	0.3976742	0.3719902	177
**GOBP_POSITIVE_REGULATION_OF_LYMPHOCYTE_DIFFERENTIATION**	GOBP_POSITIVE_REGULATION_OF_LYMPHOCYTE_DIFFERENTIATION	GOBP_POSITIVE_REGULATION_OF_LYMPHOCYTE_DIFFERENTIATION	13	-0.472131421	-1.618325653	0.0391125	0.4001405	0.3742972	266
**GOMF_UBIQUITIN_LIKE_PROTEIN_LIGASE_ACTIVITY**	GOMF_UBIQUITIN_LIKE_PROTEIN_LIGASE_ACTIVITY	GOMF_UBIQUITIN_LIKE_PROTEIN_LIGASE_ACTIVITY	34	-0.34090307	-1.614617427	0.0391891	0.4005473	0.3746778	665
**GOCC_ADHERENS_JUNCTION**	GOCC_ADHERENS_JUNCTION	GOCC_ADHERENS_JUNCTION	10	-0.501915401	-1.584494308	0.0394537	0.4010177	0.3751177	383
**HP_ABNORMAL_INCISOR_MORPHOLOGY**	HP_ABNORMAL_INCISOR_MORPHOLOGY	HP_ABNORMAL_INCISOR_MORPHOLOGY	10	-0.506765887	-1.599806784	0.0394537	0.4010177	0.3751177	177
**HP_DISPROPORTIONATE_TALL_STATURE**	HP_DISPROPORTIONATE_TALL_STATURE	HP_DISPROPORTIONATE_TALL_STATURE	10	-0.504984687	-1.594183721	0.0394537	0.4010177	0.3751177	177
**HP_DOWN_SLOPING_SHOULDERS**	HP_DOWN_SLOPING_SHOULDERS	HP_DOWN_SLOPING_SHOULDERS	10	-0.505233731	-1.594969929	0.0394537	0.4010177	0.3751177	177
**HP_LARYNGOMALACIA**	HP_LARYNGOMALACIA	HP_LARYNGOMALACIA	10	-0.506785884	-1.599869912	0.0394537	0.4010177	0.3751177	430
**HP_BROAD_BASED_GAIT**	HP_BROAD_BASED_GAIT	HP_BROAD_BASED_GAIT	8	-0.539409362	-1.54818076	0.0394944	0.4010177	0.3751177	265
**GOBP_CARDIAC_MUSCLE_TISSUE_DEVELOPMENT**	GOBP_CARDIAC_MUSCLE_TISSUE_DEVELOPMENT	GOBP_CARDIAC_MUSCLE_TISSUE_DEVELOPMENT	12	-0.481231233	-1.601924784	0.0395136	0.4010177	0.3751177	144
**GOBP_REGULATION_OF_CELL_SIZE**	GOBP_REGULATION_OF_CELL_SIZE	GOBP_REGULATION_OF_CELL_SIZE	18	-0.414813397	-1.610953842	0.0395480	0.4010177	0.3751177	479
**GOBP_PEPTIDYL_THREONINE_MODIFICATION**	GOBP_PEPTIDYL_THREONINE_MODIFICATION	GOBP_PEPTIDYL_THREONINE_MODIFICATION	15	-0.45237506	-1.619586363	0.0395894	0.4010177	0.3751177	38
**GOBP_STRIATED_MUSCLE_CELL_DIFFERENTIATION**	GOBP_STRIATED_MUSCLE_CELL_DIFFERENTIATION	GOBP_STRIATED_MUSCLE_CELL_DIFFERENTIATION	19	-0.419142219	-1.639917055	0.0396039	0.4010177	0.3751177	266
**GOBP_ENDOTHELIAL_CELL_APOPTOTIC_PROCESS**	GOBP_ENDOTHELIAL_CELL_APOPTOTIC_PROCESS	GOBP_ENDOTHELIAL_CELL_APOPTOTIC_PROCESS	6	-0.599802366	-1.554242066	0.0396825	0.4014396	0.3755124	227
**GOBP_MITOTIC_NUCLEAR_DIVISION**	GOBP_MITOTIC_NUCLEAR_DIVISION	GOBP_MITOTIC_NUCLEAR_DIVISION	23	0.312063002	1.563980221	0.0398411	0.4026696	0.3766630	60
**GOBP_NEGATIVE_REGULATION_OF_ALPHA_BETA_T_CELL_DIFFERENTIATION**	GOBP_NEGATIVE_REGULATION_OF_ALPHA_BETA_T_CELL_DIFFERENTIATION	GOBP_NEGATIVE_REGULATION_OF_ALPHA_BETA_T_CELL_DIFFERENTIATION	5	-0.655410783	-1.58524234	0.0400114	0.4028933	0.3768722	276
**GOBP_NEGATIVE_REGULATION_OF_CD4_POSITIVE_ALPHA_BETA_T_CELL_ACTIVATION**	GOBP_NEGATIVE_REGULATION_OF_CD4_POSITIVE_ALPHA_BETA_T_CELL_ACTIVATION	GOBP_NEGATIVE_REGULATION_OF_CD4_POSITIVE_ALPHA_BETA_T_CELL_ACTIVATION	5	-0.655410783	-1.58524234	0.0400114	0.4028933	0.3768722	276
**GOBP_NEGATIVE_REGULATION_OF_CD4_POSITIVE_ALPHA_BETA_T_CELL_DIFFERENTIA**	GOBP_NEGATIVE_REGULATION_OF_CD4_POSITIVE_ALPHA_BETA_T_CELL_DIFFERENTIAT	GOBP_NEGATIVE_REGULATION_OF_CD4_POSITIVE_ALPHA_BETA_T_CELL_DIFFERENTIAT	5	-0.655410783	-1.58524234	0.0400114	0.4028933	0.3768722	276
**GOBP_NEGATIVE_REGULATION_OF_T_HELPER_CELL_DIFFERENTIATION**	GOBP_NEGATIVE_REGULATION_OF_T_HELPER_CELL_DIFFERENTIATION	GOBP_NEGATIVE_REGULATION_OF_T_HELPER_CELL_DIFFERENTIATION	5	-0.655410783	-1.58524234	0.0400114	0.4028933	0.3768722	276
**GOBP_CENTRAL_NERVOUS_SYSTEM_DEVELOPMENT**	GOBP_CENTRAL_NERVOUS_SYSTEM_DEVELOPMENT	GOBP_CENTRAL_NERVOUS_SYSTEM_DEVELOPMENT	68	0.201915178	1.528403729	0.0401309	0.4037221	0.3776475	39
**GOCC_PROTON_TRANSPORTING_TWO_SECTOR_ATPASE_COMPLEX_PROTON_TRANSPO**	GOCC_PROTON_TRANSPORTING_TWO_SECTOR_ATPASE_COMPLEX_PROTON_TRANSP	GOCC_PROTON_TRANSPORTING_TWO_SECTOR_ATPASE_COMPLEX_PROTON_TRANSP	5	-0.641234162	-1.550953342	0.0403225	0.4052754	0.3791005	317
**HP_INTELLECTUAL_DISABILITY_MILD**	HP_INTELLECTUAL_DISABILITY_MILD	HP_INTELLECTUAL_DISABILITY_MILD	43	-0.323023957	-1.640210495	0.0404858	0.4060667	0.3798407	253
**GOBP_POSITIVE_REGULATION_OF_CELLULAR_PROTEIN_CATABOLIC_PROCESS**	GOBP_POSITIVE_REGULATION_OF_CELLULAR_PROTEIN_CATABOLIC_PROCESS	GOBP_POSITIVE_REGULATION_OF_CELLULAR_PROTEIN_CATABOLIC_PROCESS	24	0.30692632	1.600463426	0.0405931	0.4060667	0.3798407	19
**HP_DELAYED_CRANIAL_SUTURE_CLOSURE**	HP_DELAYED_CRANIAL_SUTURE_CLOSURE	HP_DELAYED_CRANIAL_SUTURE_CLOSURE	13	-0.454668581	-1.558468244	0.0406015	0.4060667	0.3798407	380
**GOBP_POSITIVE_REGULATION_OF_ERK1_AND_ERK2_CASCADE**	GOBP_POSITIVE_REGULATION_OF_ERK1_AND_ERK2_CASCADE	GOBP_POSITIVE_REGULATION_OF_ERK1_AND_ERK2_CASCADE	8	0.485140831	1.553527467	0.0407466	0.4060667	0.3798407	128
**GOBP_ERK1_AND_ERK2_CASCADE**	GOBP_ERK1_AND_ERK2_CASCADE	GOBP_ERK1_AND_ERK2_CASCADE	17	0.362519223	1.606257286	0.0408250	0.4060667	0.3798407	128
**GOBP_REGULATION_OF_HEMOPOIESIS**	GOBP_REGULATION_OF_HEMOPOIESIS	GOBP_REGULATION_OF_HEMOPOIESIS	44	0.244697812	1.56448781	0.0408292	0.4060667	0.3798407	57
**HP_ABNORMAL_CHOROID_MORPHOLOGY**	HP_ABNORMAL_CHOROID_MORPHOLOGY	HP_ABNORMAL_CHOROID_MORPHOLOGY	18	-0.409209913	-1.589192362	0.0409604	0.4060667	0.3798407	517
**GOBP_NEGATIVE_REGULATION_OF_SIGNALING**	GOBP_NEGATIVE_REGULATION_OF_SIGNALING	GOBP_NEGATIVE_REGULATION_OF_SIGNALING	117	0.165801229	1.421226826	0.0410759	0.4060667	0.3798407	42
**GOBP_FEMALE_SEX_DIFFERENTIATION**	GOBP_FEMALE_SEX_DIFFERENTIATION	GOBP_FEMALE_SEX_DIFFERENTIATION	8	0.484454044	1.551328225	0.0411520	0.4060667	0.3798407	152
**GOBP_PROTEIN_IMPORT**	GOBP_PROTEIN_IMPORT	GOBP_PROTEIN_IMPORT	25	0.312885351	1.651379288	0.0411549	0.4060667	0.3798407	46
**GOBP_RESPONSE_TO_ENDOGENOUS_STIMULUS**	GOBP_RESPONSE_TO_ENDOGENOUS_STIMULUS	GOBP_RESPONSE_TO_ENDOGENOUS_STIMULUS	129	0.16183469	1.418963275	0.0412146	0.4060667	0.3798407	131
**GOBP_REGULATION_OF_LYMPHOCYTE_MIGRATION**	GOBP_REGULATION_OF_LYMPHOCYTE_MIGRATION	GOBP_REGULATION_OF_LYMPHOCYTE_MIGRATION	6	-0.594110295	-1.539492445	0.0412698	0.4060667	0.3798407	41
**HP_SARCOMA**	HP_SARCOMA	HP_SARCOMA	26	0.300554415	1.598242699	0.0412796	0.4060667	0.3798407	301
**GOBP_ESTROUS_CYCLE**	GOBP_ESTROUS_CYCLE	GOBP_ESTROUS_CYCLE	2	0.853989813	1.490813733	0.0414107	0.4060667	0.3798407	174
**GOBP_RESPONSE_TO_L_GLUTAMATE**	GOBP_RESPONSE_TO_L_GLUTAMATE	GOBP_RESPONSE_TO_L_GLUTAMATE	2	0.853989813	1.490813733	0.0414107	0.4060667	0.3798407	174
**HP_DISINHIBITION**	HP_DISINHIBITION	HP_DISINHIBITION	21	-0.398558691	-1.606598305	0.0414285	0.4060667	0.3798407	298
**GOBP_HYDROGEN_PEROXIDE_BIOSYNTHETIC_PROCESS**	GOBP_HYDROGEN_PEROXIDE_BIOSYNTHETIC_PROCESS	GOBP_HYDROGEN_PEROXIDE_BIOSYNTHETIC_PROCESS	1	-0.986429177	-1.308950324	0.0414468	0.4060667	0.3798407	18
**GOBP_POSITIVE_REGULATION_OF_HYDROGEN_PEROXIDE_METABOLIC_PROCESS**	GOBP_POSITIVE_REGULATION_OF_HYDROGEN_PEROXIDE_METABOLIC_PROCESS	GOBP_POSITIVE_REGULATION_OF_HYDROGEN_PEROXIDE_METABOLIC_PROCESS	1	-0.986429177	-1.308950324	0.0414468	0.4060667	0.3798407	18
**GOBP_POSITIVE_REGULATION_OF_REACTIVE_OXYGEN_SPECIES_BIOSYNTHETIC_PROC**	GOBP_POSITIVE_REGULATION_OF_REACTIVE_OXYGEN_SPECIES_BIOSYNTHETIC_PROC	GOBP_POSITIVE_REGULATION_OF_REACTIVE_OXYGEN_SPECIES_BIOSYNTHETIC_PROC	1	-0.986429177	-1.308950324	0.0414468	0.4060667	0.3798407	18
**GOBP_REACTIVE_OXYGEN_SPECIES_BIOSYNTHETIC_PROCESS**	GOBP_REACTIVE_OXYGEN_SPECIES_BIOSYNTHETIC_PROCESS	GOBP_REACTIVE_OXYGEN_SPECIES_BIOSYNTHETIC_PROCESS	1	-0.986429177	-1.308950324	0.0414468	0.4060667	0.3798407	18
**GOBP_REGULATION_OF_HYDROGEN_PEROXIDE_BIOSYNTHETIC_PROCESS**	GOBP_REGULATION_OF_HYDROGEN_PEROXIDE_BIOSYNTHETIC_PROCESS	GOBP_REGULATION_OF_HYDROGEN_PEROXIDE_BIOSYNTHETIC_PROCESS	1	-0.986429177	-1.308950324	0.0414468	0.4060667	0.3798407	18
**GOBP_REGULATION_OF_HYDROGEN_PEROXIDE_METABOLIC_PROCESS**	GOBP_REGULATION_OF_HYDROGEN_PEROXIDE_METABOLIC_PROCESS	GOBP_REGULATION_OF_HYDROGEN_PEROXIDE_METABOLIC_PROCESS	1	-0.986429177	-1.308950324	0.0414468	0.4060667	0.3798407	18
**GOBP_REGULATION_OF_REACTIVE_OXYGEN_SPECIES_BIOSYNTHETIC_PROCESS**	GOBP_REGULATION_OF_REACTIVE_OXYGEN_SPECIES_BIOSYNTHETIC_PROCESS	GOBP_REGULATION_OF_REACTIVE_OXYGEN_SPECIES_BIOSYNTHETIC_PROCESS	1	-0.986429177	-1.308950324	0.0414468	0.4060667	0.3798407	18
**HP_CAESARIAN_SECTION**	HP_CAESARIAN_SECTION	HP_CAESARIAN_SECTION	1	-0.987277354	-1.310075819	0.0414468	0.4060667	0.3798407	17
**HP_DEATH_IN_ADOLESCENCE**	HP_DEATH_IN_ADOLESCENCE	HP_DEATH_IN_ADOLESCENCE	1	-0.987277354	-1.310075819	0.0414468	0.4060667	0.3798407	17
**HP_FOCAL_MYOCLONIC_SEIZURE**	HP_FOCAL_MYOCLONIC_SEIZURE	HP_FOCAL_MYOCLONIC_SEIZURE	1	-0.987277354	-1.310075819	0.0414468	0.4060667	0.3798407	17
**HP_LACTOSE_INTOLERANCE**	HP_LACTOSE_INTOLERANCE	HP_LACTOSE_INTOLERANCE	1	-0.987277354	-1.310075819	0.0414468	0.4060667	0.3798407	17

**HP_YELLOW_BROWN_DISCOLORATION_OF_THE_TEETH**	HP_YELLOW_BROWN_DISCOLORATION_OF_THE_TEETH	HP_YELLOW_BROWN_DISCOLORATION_OF_THE_TEETH	1	-0.987277354	-1.310075819	0.0414468	0.4060667	0.3798407	17
**HP_ABNORMAL_HAIR_MORPHOLOGY**	HP_ABNORMAL_HAIR_MORPHOLOGY	HP_ABNORMAL_HAIR_MORPHOLOGY	127	-0.23021694	-1.511145051	0.0415183	0.4064018	0.3801541	177
**HP_ABNORMALITY_OF_THE_CURVATURE_OF_THE_VERTEBRAL_COLUMN**	HP_ABNORMALITY_OF_THE_CURVATURE_OF_THE_VERTEBRAL_COLUMN	HP_ABNORMALITY_OF_THE_CURVATURE_OF_THE_VERTEBRAL_COLUMN	101	-0.247906153	-1.553014461	0.0415647	0.4064902	0.3802367	600
**HP_GENERALIZED_HYPOTONIA**	HP_GENERALIZED_HYPOTONIA	HP_GENERALIZED_HYPOTONIA	94	-0.245954927	-1.522585037	0.0417690	0.4081206	0.3817619	491
**HP_ABNORMAL_AORTIC_VALVE_PHYSIOLOGY**	HP_ABNORMAL_AORTIC_VALVE_PHYSIOLOGY	HP_ABNORMAL_AORTIC_VALVE_PHYSIOLOGY	13	-0.468873889	-1.607159805	0.0418255	0.4082207	0.3818555	325
**GOBP_CELL_CELL_SIGNALING**	GOBP_CELL_CELL_SIGNALING	GOBP_CELL_CELL_SIGNALING	73	-0.262334239	-1.531528633	0.0418781	0.4082207	0.3818555	456
**HP_FUNCTIONAL_ABNORMALITY_OF_THE_MIDDLE_EAR**	HP_FUNCTIONAL_ABNORMALITY_OF_THE_MIDDLE_EAR	HP_FUNCTIONAL_ABNORMALITY_OF_THE_MIDDLE_EAR	34	-0.33528219	-1.58799528	0.0418918	0.4082207	0.3818555	464
**GOBP_POSITIVE_REGULATION_OF_CANONICAL_WNT_SIGNALING_PATHWAY**	GOBP_POSITIVE_REGULATION_OF_CANONICAL_WNT_SIGNALING_PATHWAY	GOBP_POSITIVE_REGULATION_OF_CANONICAL_WNT_SIGNALING_PATHWAY	9	-0.53600444	-1.616067556	0.0420596	0.4094890	0.3830419	230
**HP_LETHARGY**	HP_LETHARGY	HP_LETHARGY	26	0.29854118	1.587537023	0.0422803	0.4112688	0.3847068	276
**GOBP_CELLULAR_RESPONSE_TO_STARVATION**	GOBP_CELLULAR_RESPONSE_TO_STARVATION	GOBP_CELLULAR_RESPONSE_TO_STARVATION	22	0.335625141	1.653985811	0.0424144	0.4122048	0.3855823	36
**HP_DIALEPTIC_SEIZURE**	HP_DIALEPTIC_SEIZURE	HP_DIALEPTIC_SEIZURE	26	-0.379179985	-1.621356241	0.0426742	0.4142857	0.3875288	242
**GOMF_MOLECULAR_FUNCTION_REGULATOR**	GOMF_MOLECULAR_FUNCTION_REGULATOR	GOMF_MOLECULAR_FUNCTION_REGULATOR	136	0.161743389	1.437588978	0.0427133	0.4142857	0.3875288	50
**GOBP_REGULATION_OF_DNA_BINDING_TRANSCRIPTION_FACTOR_ACTIVITY**	GOBP_REGULATION_OF_DNA_BINDING_TRANSCRIPTION_FACTOR_ACTIVITY	GOBP_REGULATION_OF_DNA_BINDING_TRANSCRIPTION_FACTOR_ACTIVITY	42	0.232455915	1.483361384	0.0427866	0.4142857	0.3875288	26
**GOBP_ANATOMICAL_STRUCTURE_FORMATION_INVOLVED_IN_MORPHOGENESIS**	GOBP_ANATOMICAL_STRUCTURE_FORMATION_INVOLVED_IN_MORPHOGENESIS	GOBP_ANATOMICAL_STRUCTURE_FORMATION_INVOLVED_IN_MORPHOGENESIS	79	0.193891035	1.529580277	0.0427948	0.4142857	0.3875288	73
**HP_ABNORMALITY_OF_THE_LARYNX**	HP_ABNORMALITY_OF_THE_LARYNX	HP_ABNORMALITY_OF_THE_LARYNX	21	-0.396210916	-1.597134375	0.0428571	0.4142857	0.3875288	458
**HP_INTERICTAL_EEG_ABNORMALITY**	HP_INTERICTAL_EEG_ABNORMALITY	HP_INTERICTAL_EEG_ABNORMALITY	21	-0.396668326	-1.598978207	0.0428571	0.4142857	0.3875288	270
**GOCC_RNA_POLYMERASE_II_TRANSCRIPTION_REGULATOR_COMPLEX**	GOCC_RNA_POLYMERASE_II_TRANSCRIPTION_REGULATOR_COMPLEX	GOCC_RNA_POLYMERASE_II_TRANSCRIPTION_REGULATOR_COMPLEX	28	0.292083767	1.590853237	0.0430848	0.4161166	0.3892415	82
**GOBP_REGULATION_OF_B_CELL_ACTIVATION**	GOBP_REGULATION_OF_B_CELL_ACTIVATION	GOBP_REGULATION_OF_B_CELL_ACTIVATION	16	-0.435096932	-1.604412004	0.0432276	0.4171258	0.3901855	178
**GOBP_CAMP_CATABOLIC_PROCESS**	GOBP_CAMP_CATABOLIC_PROCESS	GOBP_CAMP_CATABOLIC_PROCESS	2	-0.847198642	-1.439613441	0.0434056	0.4173622	0.3904066	183
**GOBP_CAMP_METABOLIC_PROCESS**	GOBP_CAMP_METABOLIC_PROCESS	GOBP_CAMP_METABOLIC_PROCESS	2	-0.847198642	-1.439613441	0.0434056	0.4173622	0.3904066	183
**GOBP_CYCLIC_NUCLEOTIDE_CATABOLIC_PROCESS**	GOBP_CYCLIC_NUCLEOTIDE_CATABOLIC_PROCESS	GOBP_CYCLIC_NUCLEOTIDE_CATABOLIC_PROCESS	2	-0.847198642	-1.439613441	0.0434056	0.4173622	0.3904066	183
**GOBP_CYCLIC_NUCLEOTIDE_METABOLIC_PROCESS**	GOBP_CYCLIC_NUCLEOTIDE_METABOLIC_PROCESS	GOBP_CYCLIC_NUCLEOTIDE_METABOLIC_PROCESS	2	-0.847198642	-1.439613441	0.0434056	0.4173622	0.3904066	183
**HP_ABNORMAL_BLOOD_ION_CONCENTRATION**	HP_ABNORMAL_BLOOD_ION_CONCENTRATION	HP_ABNORMAL_BLOOD_ION_CONCENTRATION	22	-0.39859479	-1.634288394	0.0436005	0.4182242	0.3912129	517
**HP_ANEMIA_OF_INADEQUATE_PRODUCTION**	HP_ANEMIA_OF_INADEQUATE_PRODUCTION	HP_ANEMIA_OF_INADEQUATE_PRODUCTION	28	0.291809465	1.589359232	0.0436134	0.4182242	0.3912129	304
**GOBP_PHOSPHATIDYLCHOLINE_ACYL_CHAIN_REMODELING**	GOBP_PHOSPHATIDYLCHOLINE_ACYL_CHAIN_REMODELING	GOBP_PHOSPHATIDYLCHOLINE_ACYL_CHAIN_REMODELING	2	0.848896435	1.4819222	0.0436491	0.4182242	0.3912129	180
**HP_SHORT_FACE**	HP_SHORT_FACE	HP_SHORT_FACE	2	0.846349745	1.477476433	0.0436491	0.4182242	0.3912129	183
**GOBP_MYELOID_CELL_HOMEOSTASIS**	GOBP_MYELOID_CELL_HOMEOSTASIS	GOBP_MYELOID_CELL_HOMEOSTASIS	25	0.309987609	1.636085285	0.0437433	0.4187169	0.3916738	73
**GOBP_REGULATION_OF_MULTICELLULAR_ORGANISMAL_DEVELOPMENT**	GOBP_REGULATION_OF_MULTICELLULAR_ORGANISMAL_DEVELOPMENT	GOBP_REGULATION_OF_MULTICELLULAR_ORGANISMAL_DEVELOPMENT	102	0.185263545	1.561880241	0.0437775	0.4187169	0.3916738	39
**HP_ABNORMAL_TESTIS_MORPHOLOGY**	HP_ABNORMAL_TESTIS_MORPHOLOGY	HP_ABNORMAL_TESTIS_MORPHOLOGY	70	-0.269665026	-1.551834276	0.0439844	0.4201056	0.3929729	491
**HP_RELATIVE_MACROCEPHALY**	HP_RELATIVE_MACROCEPHALY	HP_RELATIVE_MACROCEPHALY	12	-0.479508061	-1.596188681	0.0440729	0.4201056	0.3929729	206
**GOMF_TRANSCRIPTION_COACTIVATOR_ACTIVITY**	GOMF_TRANSCRIPTION_COACTIVATOR_ACTIVITY	GOMF_TRANSCRIPTION_COACTIVATOR_ACTIVITY	36	-0.325791213	-1.570523844	0.0443548	0.4201056	0.3929729	495
**GOBP_EPITHELIUM_DEVELOPMENT**	GOBP_EPITHELIUM_DEVELOPMENT	GOBP_EPITHELIUM_DEVELOPMENT	65	0.209388371	1.550911853	0.0443642	0.4201056	0.3929729	73
**GOCC_NEURON_PROJECTION_TERMINUS**	GOCC_NEURON_PROJECTION_TERMINUS	GOCC_NEURON_PROJECTION_TERMINUS	8	0.479600111	1.535784868	0.0444115	0.4201056	0.3929729	178
**GOCC_ROUGH_ENDOPLASMIC_RETICULUM_MEMBRANE**	GOCC_ROUGH_ENDOPLASMIC_RETICULUM_MEMBRANE	GOCC_ROUGH_ENDOPLASMIC_RETICULUM_MEMBRANE	8	0.480375427	1.538267597	0.0444115	0.4201056	0.3929729	617
**HP_LARGE_BASAL_GANGLIA**	HP_LARGE_BASAL_GANGLIA	HP_LARGE_BASAL_GANGLIA	7	-0.557977666	-1.528080533	0.0444444	0.4201056	0.3929729	234
**GOBP_ESTABLISHMENT_OF_MITOCHONDRION_LOCALIZATION**	GOBP_ESTABLISHMENT_OF_MITOCHONDRION_LOCALIZATION	GOBP_ESTABLISHMENT_OF_MITOCHONDRION_LOCALIZATION	1	0.988973707	1.31820878	0.0447340	0.4201056	0.3929729	14
**GOBP_FAT_PAD_DEVELOPMENT**	GOBP_FAT_PAD_DEVELOPMENT	GOBP_FAT_PAD_DEVELOPMENT	1	0.988973707	1.31820878	0.0447340	0.4201056	0.3929729	14
**GOBP_HYPOTHALAMUS_CELL_DIFFERENTIATION**	GOBP_HYPOTHALAMUS_CELL_DIFFERENTIATION	GOBP_HYPOTHALAMUS_CELL_DIFFERENTIATION	1	0.988973707	1.31820878	0.0447340	0.4201056	0.3929729	14
**GOBP_HYPOTHALAMUS_GONADOTROPHIN_RELEASING_HORMONE_NEURON_DIFFERE**	GOBP_HYPOTHALAMUS_GONADOTROPHIN_RELEASING_HORMONE_NEURON_DIFFERE	GOBP_HYPOTHALAMUS_GONADOTROPHIN_RELEASING_HORMONE_NEURON_DIFFERE	1	0.988973707	1.31820878	0.0447340	0.4201056	0.3929729	14
**GOBP_MALE_MEIOSIS_I**	GOBP_MALE_MEIOSIS_I	GOBP_MALE_MEIOSIS_I	1	0.988973707	1.31820878	0.0447340	0.4201056	0.3929729	14
**GOBP_MALE_MEIOTIC_NUCLEAR_DIVISION**	GOBP_MALE_MEIOTIC_NUCLEAR_DIVISION	GOBP_MALE_MEIOTIC_NUCLEAR_DIVISION	1	0.988973707	1.31820878	0.0447340	0.4201056	0.3929729	14
**GOBP_RESPONSE_TO_GRAVITY**	GOBP_RESPONSE_TO_GRAVITY	GOBP_RESPONSE_TO_GRAVITY	1	0.989821883	1.319339319	0.0447340	0.4201056	0.3929729	13
**GOBP_RESPONSE_TO_IMMOBILIZATION_STRESS**	GOBP_RESPONSE_TO_IMMOBILIZATION_STRESS	GOBP_RESPONSE_TO_IMMOBILIZATION_STRESS	1	0.989821883	1.319339319	0.0447340	0.4201056	0.3929729	13
**HP_INCREASED_C_PEPTIDE_LEVEL**	HP_INCREASED_C_PEPTIDE_LEVEL	HP_INCREASED_C_PEPTIDE_LEVEL	1	0.989821883	1.319339319	0.0447340	0.4201056	0.3929729	13
**HP_NASAL_REGURGITATION**	HP_NASAL_REGURGITATION	HP_NASAL_REGURGITATION	1	0.988973707	1.31820878	0.0447340	0.4201056	0.3929729	14
**HP_OVERGROWTH_OF_EXTERNAL_GENITALIA**	HP_OVERGROWTH_OF_EXTERNAL_GENITALIA	HP_OVERGROWTH_OF_EXTERNAL_GENITALIA	1	0.989821883	1.319339319	0.0447340	0.4201056	0.3929729	13
**HP_PRECOCIOUS_PUBERTY_IN_FEMALES**	HP_PRECOCIOUS_PUBERTY_IN_FEMALES	HP_PRECOCIOUS_PUBERTY_IN_FEMALES	1	0.989821883	1.319339319	0.0447340	0.4201056	0.3929729	13
**HP_PROPORTIONATE_TALL_STATURE**	HP_PROPORTIONATE_TALL_STATURE	HP_PROPORTIONATE_TALL_STATURE	1	0.989821883	1.319339319	0.0447340	0.4201056	0.3929729	13
**HP_VELOPHARYNGEAL_INSUFFICIENCY**	HP_VELOPHARYNGEAL_INSUFFICIENCY	HP_VELOPHARYNGEAL_INSUFFICIENCY	1	0.988973707	1.31820878	0.0447340	0.4201056	0.3929729	14
**GOMF_ATPASE_BINDING**	GOMF_ATPASE_BINDING	GOMF_ATPASE_BINDING	7	0.526606504	1.584687125	0.0448616	0.4209411	0.3937544	1
**HP_CRYPTORCHIDISM**	HP_CRYPTORCHIDISM	HP_CRYPTORCHIDISM	60	-0.281233483	-1.575285624	0.0449293	0.4212130	0.3940087	442
**GOMF_CYTOSKELETAL_MOTOR_ACTIVITY**	GOMF_CYTOSKELETAL_MOTOR_ACTIVITY	GOMF_CYTOSKELETAL_MOTOR_ACTIVITY	2	-0.844651952	-1.435285946	0.0450751	0.4222153	0.3949462	186
**GOBP_REGULATION_OF_PROTEIN_CATABOLIC_PROCESS**	GOBP_REGULATION_OF_PROTEIN_CATABOLIC_PROCESS	GOBP_REGULATION_OF_PROTEIN_CATABOLIC_PROCESS	51	0.23117039	1.556583951	0.0451348	0.4224104	0.3951288	47
**HP_ABNORMAL_CARDIAC_VENTRICLE_MORPHOLOGY**	HP_ABNORMAL_CARDIAC_VENTRICLE_MORPHOLOGY	HP_ABNORMAL_CARDIAC_VENTRICLE_MORPHOLOGY	67	-0.271770445	-1.551068063	0.0453955	0.4244858	0.3970701	173
**GOBP_POSITIVE_REGULATION_OF_PEPTIDYL_LYSINE_ACETYLATION**	GOBP_POSITIVE_REGULATION_OF_PEPTIDYL_LYSINE_ACETYLATION	GOBP_POSITIVE_REGULATION_OF_PEPTIDYL_LYSINE_ACETYLATION	3	-0.768054376	-1.529853829	0.0455284	0.4249973	0.3975486	277
**GOBP_POSITIVE_REGULATION_OF_PROTEIN_ACETYLATION**	GOBP_POSITIVE_REGULATION_OF_PROTEIN_ACETYLATION	GOBP_POSITIVE_REGULATION_OF_PROTEIN_ACETYLATION	3	-0.768054376	-1.529853829	0.0455284	0.4249973	0.3975486	277
**HP_ABNORMAL_ACTIVITY_OF_MITOCHONDRIAL_RESPIRATORY_CHAIN**	HP_ABNORMAL_ACTIVITY_OF_MITOCHONDRIAL_RESPIRATORY_CHAIN	HP_ABNORMAL_ACTIVITY_OF_MITOCHONDRIAL_RESPIRATORY_CHAIN	8	-0.530744796	-1.523312237	0.0458135	0.4267251	0.3991648	75
**HP_HEMORRHAGE_OF_THE_EYE**	HP_HEMORRHAGE_OF_THE_EYE	HP_HEMORRHAGE_OF_THE_EYE	8	-0.530643998	-1.523022933	0.0458135	0.4267251	0.3991648	471
**GOBP_REGULATION_OF_T_CELL_MIGRATION**	GOBP_REGULATION_OF_T_CELL_MIGRATION	GOBP_REGULATION_OF_T_CELL_MIGRATION	5	-0.648366533	-1.56820441	0.0458312	0.4267251	0.3991648	41
**GOBP_REGULATION_OF_PATTERN_RECOGNITION_RECEPTOR_SIGNALING_PATHWAY**	GOBP_REGULATION_OF_PATTERN_RECOGNITION_RECEPTOR_SIGNALING_PATHWAY	GOBP_REGULATION_OF_PATTERN_RECOGNITION_RECEPTOR_SIGNALING_PATHWAY	14	0.394379186	1.5941829	0.0459074	0.4270690	0.3994865	7
**HP_ABNORMAL_VASCULAR_MORPHOLOGY**	HP_ABNORMAL_VASCULAR_MORPHOLOGY	HP_ABNORMAL_VASCULAR_MORPHOLOGY	103	-0.244788905	-1.541930135	0.0461165	0.428647	0.4009625	211
**GOMF_UBIQUITIN_LIKE_PROTEIN_TRANSFERASE_ACTIVITY**	GOMF_UBIQUITIN_LIKE_PROTEIN_TRANSFERASE_ACTIVITY	GOMF_UBIQUITIN_LIKE_PROTEIN_TRANSFERASE_ACTIVITY	50	-0.291884063	-1.5606295	0.0462962	0.4293715	0.4016403	665
**HP_FUNCTIONAL_MOTOR_DEFICIT**	HP_FUNCTIONAL_MOTOR_DEFICIT	HP_FUNCTIONAL_MOTOR_DEFICIT	50	-0.293011609	-1.566658203	0.0462962	0.4293715	0.4016403	270
**GOBP_GRANULOCYTE_MIGRATION**	GOBP_GRANULOCYTE_MIGRATION	GOBP_GRANULOCYTE_MIGRATION	10	-0.509323773	-1.607881762	0.0463129	0.4293715	0.4016403	76
**GOCC_CATION_CHANNEL_COMPLEX**	GOCC_CATION_CHANNEL_COMPLEX	GOCC_CATION_CHANNEL_COMPLEX	4	-0.674979059	-1.510920311	0.0464	0.4298126	0.4020529	70
**GOBP_CHAPERONE_MEDIATED_AUTOPHAGY**	GOBP_CHAPERONE_MEDIATED_AUTOPHAGY	GOBP_CHAPERONE_MEDIATED_AUTOPHAGY	4	0.714726747	1.647239741	0.0466594	0.4304379	0.4026378	19
**GOCC_SECRETORY_GRANULE**	GOCC_SECRETORY_GRANULE	GOCC_SECRETORY_GRANULE	74	0.208380883	1.597794624	0.0466634	0.4304379	0.4026378	141
**HP_ABNORMAL_BRAINSTEM_MRI_SIGNAL_INTENSITY**	HP_ABNORMAL_BRAINSTEM_MRI_SIGNAL_INTENSITY	HP_ABNORMAL_BRAINSTEM_MRI_SIGNAL_INTENSITY	9	-0.529910649	-1.597694615	0.0467071	0.4304379	0.4026378	227
**GOBP_DETERMINATION_OF_ADULT_LIFESPAN**	GOBP_DETERMINATION_OF_ADULT_LIFESPAN	GOBP_DETERMINATION_OF_ADULT_LIFESPAN	2	-0.842105263	-1.430958451	0.0467445	0.4304379	0.4026378	189
**GOMF_ARYL_HYDROCARBON_RECEPTOR_BINDING**	GOMF_ARYL_HYDROCARBON_RECEPTOR_BINDING	GOMF_ARYL_HYDROCARBON_RECEPTOR_BINDING	2	-0.84295416	-1.432400949	0.0467445	0.4304379	0.4026378	188
**GOMF_INTRONIC_TRANSCRIPTION_REGULATORY_REGION_SEQUENCE_SPECIFIC_DNA_**	GOMF_INTRONIC_TRANSCRIPTION_REGULATORY_REGION_SEQUENCE_SPECIFIC_DNA_	GOMF_INTRONIC_TRANSCRIPTION_REGULATORY_REGION_SEQUENCE_SPECIFIC_DNA_	2	-0.84295416	-1.432400949	0.0467445	0.4304379	0.4026378	188
**HP_RECURRENT_HYPOGLYCEMIA**	HP_RECURRENT_HYPOGLYCEMIA	HP_RECURRENT_HYPOGLYCEMIA	2	-0.841256367	-1.429515952	0.0467445	0.4304379	0.4026378	190
**GOBP_REGULATION_OF_SMALL_GTPASE_MEDIATED_SIGNAL_TRANSDUCTION**	GOBP_REGULATION_OF_SMALL_GTPASE_MEDIATED_SIGNAL_TRANSDUCTION	GOBP_REGULATION_OF_SMALL_GTPASE_MEDIATED_SIGNAL_TRANSDUCTION	30	-0.346396819	-1.562649982	0.0468965	0.4314720	0.4036051	471
**HP_INTELLECTUAL_DISABILITY_MODERATE**	HP_INTELLECTUAL_DISABILITY_MODERATE	HP_INTELLECTUAL_DISABILITY_MODERATE	26	-0.370331574	-1.583520839	0.0469416	0.4315221	0.4036520	242
**HP_SELF_INJURIOUS_BEHAVIOR**	HP_SELF_INJURIOUS_BEHAVIOR	HP_SELF_INJURIOUS_BEHAVIOR	29	-0.35581836	-1.589403616	0.0471567	0.4329064	0.4049469	632
**GOBP_ORGANOPHOSPHATE_CATABOLIC_PROCESS**	GOBP_ORGANOPHOSPHATE_CATABOLIC_PROCESS	GOBP_ORGANOPHOSPHATE_CATABOLIC_PROCESS	9	-0.528557341	-1.593614356	0.0471718	0.4329064	0.4049469	199
**GOBP_REGULATION_OF_METAL_ION_TRANSPORT**	GOBP_REGULATION_OF_METAL_ION_TRANSPORT	GOBP_REGULATION_OF_METAL_ION_TRANSPORT	28	-0.360193384	-1.583638816	0.0472879	0.4336053	0.4056006	277
**HP_NON_HODGKIN_LYMPHOMA**	HP_NON_HODGKIN_LYMPHOMA	HP_NON_HODGKIN_LYMPHOMA	7	-0.552421414	-1.512864151	0.0476190	0.4362739	0.4080969	167
**GOBP_POSITIVE_REGULATION_OF_NUCLEASE_ACTIVITY**	GOBP_POSITIVE_REGULATION_OF_NUCLEASE_ACTIVITY	GOBP_POSITIVE_REGULATION_OF_NUCLEASE_ACTIVITY	3	0.773152082	1.583137511	0.0477846	0.4370102	0.4087856	270
**GOMF_SMALL_RIBOSOMAL_SUBUNIT_RRNA_BINDING**	GOMF_SMALL_RIBOSOMAL_SUBUNIT_RRNA_BINDING	GOMF_SMALL_RIBOSOMAL_SUBUNIT_RRNA_BINDING	3	0.773152082	1.583137511	0.0477846	0.4370102	0.4087856	270
**GOBP_ALPHA_BETA_T_CELL_ACTIVATION**	GOBP_ALPHA_BETA_T_CELL_ACTIVATION	GOBP_ALPHA_BETA_T_CELL_ACTIVATION	27	-0.364641708	-1.579351857	0.0478199	0.4370102	0.4087856	276
**HP_ABNORMAL_EYEBROW_MORPHOLOGY**	HP_ABNORMAL_EYEBROW_MORPHOLOGY	HP_ABNORMAL_EYEBROW_MORPHOLOGY	68	-0.268451957	-1.534747261	0.0481144	0.4391369	0.4107750	442
**GOBP_ANTERIOR_POSTERIOR_PATTERN_SPECIFICATION**	GOBP_ANTERIOR_POSTERIOR_PATTERN_SPECIFICATION	GOBP_ANTERIOR_POSTERIOR_PATTERN_SPECIFICATION	8	-0.551107812	-1.581756957	0.0482151	0.4391369	0.4107750	437
**GOBP_REGULATION_OF_MYELOID_LEUKOCYTE_MEDIATED_IMMUNITY**	GOBP_REGULATION_OF_MYELOID_LEUKOCYTE_MEDIATED_IMMUNITY	GOBP_REGULATION_OF_MYELOID_LEUKOCYTE_MEDIATED_IMMUNITY	8	-0.550002006	-1.578583137	0.0482151	0.4391369	0.4107750	266
**HP_CHEST_PAIN**	HP_CHEST_PAIN	HP_CHEST_PAIN	8	-0.549755801	-1.577876495	0.0482151	0.4391369	0.4107750	339
**GOBP_POSITIVE_REGULATION_OF_GLIOGENESIS**	GOBP_POSITIVE_REGULATION_OF_GLIOGENESIS	GOBP_POSITIVE_REGULATION_OF_GLIOGENESIS	4	0.713435374	1.644263497	0.0482545	0.4391369	0.4107750	341
**HP_SMALL_FOR_GESTATIONAL_AGE**	HP_SMALL_FOR_GESTATIONAL_AGE	HP_SMALL_FOR_GESTATIONAL_AGE	31	0.2773798	1.596098839	0.0483220	0.4393831	0.4110053	443
**HP_HYPOREFLEXIA**	HP_HYPOREFLEXIA	HP_HYPOREFLEXIA	36	-0.323089598	-1.557500315	0.0483870	0.4394845	0.4111001	160
**GOMF_CHLORIDE_CHANNEL_INHIBITOR_ACTIVITY**	GOMF_CHLORIDE_CHANNEL_INHIBITOR_ACTIVITY	GOMF_CHLORIDE_CHANNEL_INHIBITOR_ACTIVITY	2	-0.833935067	-1.417075137	0.0484140	0.4394845	0.4111001	2
**GOBP_MYELOID_CELL_ACTIVATION_INVOLVED_IN_IMMUNE_RESPONSE**	GOBP_MYELOID_CELL_ACTIVATION_INVOLVED_IN_IMMUNE_RESPONSE	GOBP_MYELOID_CELL_ACTIVATION_INVOLVED_IN_IMMUNE_RESPONSE	11	-0.477472764	-1.54869195	0.0484848	0.4397604	0.4113582	288
**HP_ABNORMAL_PULMONARY_THORACIC_IMAGING_FINDING**	HP_ABNORMAL_PULMONARY_THORACIC_IMAGING_FINDING	HP_ABNORMAL_PULMONARY_THORACIC_IMAGING_FINDING	12	-0.474550847	-1.579687084	0.0486322	0.4407294	0.4122647	451
**HP_ABNORMALITY_OF_MOUTH_SHAPE**	HP_ABNORMALITY_OF_MOUTH_SHAPE	HP_ABNORMALITY_OF_MOUTH_SHAPE	25	-0.369506658	-1.56605002	0.0490883	0.4444928	0.4157850	164
**GOBP_REGULATION_OF_MAST_CELL_ACTIVATION**	GOBP_REGULATION_OF_MAST_CELL_ACTIVATION	GOBP_REGULATION_OF_MAST_CELL_ACTIVATION	6	-0.583874436	-1.512968704	0.0492063	0.4449993	0.4162588	266
**HP_NEOPLASM_OF_HEAD_AND_NECK**	HP_NEOPLASM_OF_HEAD_AND_NECK	HP_NEOPLASM_OF_HEAD_AND_NECK	5	-0.644255319	-1.558260615	0.0492261	0.4449993	0.4162588	424
**GOBP_PROTEIN_POLYMERIZATION**	GOBP_PROTEIN_POLYMERIZATION	GOBP_PROTEIN_POLYMERIZATION	25	0.30650841	1.6177224	0.0494376	0.4465098	0.4176717	34
**GOBP_NEGATIVE_REGULATION_OF_LEUKOCYTE_CELL_CELL_ADHESION**	GOBP_NEGATIVE_REGULATION_OF_LEUKOCYTE_CELL_CELL_ADHESION	GOBP_NEGATIVE_REGULATION_OF_LEUKOCYTE_CELL_CELL_ADHESION	17	-0.415646837	-1.580647381	0.0495049	0.4465098	0.4176717	276
**GOBP_CELLULAR_RESPONSE_TO_HORMONE_STIMULUS**	GOBP_CELLULAR_RESPONSE_TO_HORMONE_STIMULUS	GOBP_CELLULAR_RESPONSE_TO_HORMONE_STIMULUS	66	0.205278518	1.520016789	0.0495367	0.4465098	0.4176717	57
**GOBP_MITOCHONDRION_ORGANIZATION**	GOBP_MITOCHONDRION_ORGANIZATION	GOBP_MITOCHONDRION_ORGANIZATION	52	0.212856037	1.44647899	0.0495574	0.4465098	0.4176717	46
**GOMF_CHANNEL_INHIBITOR_ACTIVITY**	GOMF_CHANNEL_INHIBITOR_ACTIVITY	GOMF_CHANNEL_INHIBITOR_ACTIVITY	4	-0.671331278	-1.502754862	0.0496	0.4465231	0.4176842	259
**HP_ABNORMALITY_OF_THE_URINARY_SYSTEM_PHYSIOLOGY**	HP_ABNORMALITY_OF_THE_URINARY_SYSTEM_PHYSIOLOGY	HP_ABNORMALITY_OF_THE_URINARY_SYSTEM_PHYSIOLOGY	78	-0.256849917	-1.516794997	0.0496815	0.4467752	0.4179199	125
**GOMF_PROTEIN_SERINE_THREONINE_TYROSINE_KINASE_ACTIVITY**	GOMF_PROTEIN_SERINE_THREONINE_TYROSINE_KINASE_ACTIVITY	GOMF_PROTEIN_SERINE_THREONINE_TYROSINE_KINASE_ACTIVITY	36	-0.320704504	-1.546002626	0.0497311	0.4467752	0.4179199	439
**GOBP_REGULATION_OF_IMMUNE_RESPONSE**	GOBP_REGULATION_OF_IMMUNE_RESPONSE	GOBP_REGULATION_OF_IMMUNE_RESPONSE	86	-0.248359277	-1.511592906	0.0497512	0.4467752	0.4179199	276

core_enrichment	
tags=61%, list=26%, signal=50%	Hspb1/Ppp1r15a/Gapdh/Mrps6/Mrpl52/Mrps12/Zfp36/Rplp2/Rps17/Rps5/Eif3f/Rps8/Rpl31/Rps26/Rpl18/Uba52/Dhps/Rpsa/Rps4x/Eef1b2/Rpl19/Rps24/Vapa/Rpl22l1/Rps20/Eif2b2/Rpl8/Rps7/Rpl37/Rpl21/Rpl38/Rpl35/Rp
tags=88%, list=24%, signal=70%	Rplp2/Rps17/Rps5/Rps8/Rpl31/Rps26/Rpl18/Uba52/Rpsa/Rps4x/Rpl19/Rps24/Rpl22l1/Rps20/Rpl8/Rps7/Rpl37/Rpl21/Rpl38/Rpl35/Rps27/Rpl30/Rps21/Rpl18a/Rps12/Rpl7a/Rpl13/Rps16/Rps27a/Rps2/Rpl24/Rps13/Rpl1
tags=63%, list=26%, signal=51%	Hspb1/Ppp1r15a/Gapdh/Mrps6/Mrpl52/Mrps12/Zfp36/Rplp2/Rps17/Rps5/Eif3f/Rps8/Rpl31/Rps26/Rpl18/Uba52/Dhps/Rpsa/Rps4x/Eef1b2/Rpl19/Rps24/Rpl22l1/Rps20/Eif2b2/Rpl8/Rps7/Rpl37/Rpl21/Rpl38/Rpl35/Rps27/R
tags=59%, list=26%, signal=49%	Hspb1/Ppp1r15a/Gapdh/Mrps6/Gpx1/Mrpl52/Mrps12/Zfp36/Rplp2/Rps17/Rps5/Eif3f/Rps8/Rpl31/Rps26/Rpl18/Uba52/Dhps/Rpsa/Rps4x/Eef1b2/Rpl19/Rps24/Rpl22l1/Rps20/Eif2b2/Rpl8/Rps7/Rpl37/Rpl21/Rpl38/Rpl35/R
tags=26%, list=2%, signal=27%	Dnajb1/Hspa1a/Hspb1/Hspe1/Hspa8/Hspa1b/Atf3/Ppp1r15a/Dnaja1/Hsp90aa1/Hsp90ab1
tags=100%, list=24%, signal=77%	Rplp2/Rpl31/Rpl18/Uba52/Rpl19/Rpl8/Rpl37/Rpl21/Rpl38/Rpl35/Rpl30/Rpl18a/Rpl7a/Rpl13/Rpl24/Rpl13a/Rpl34/Rpl15/Rpl14/Rpl28/Rpl36a/Rpl35a/Rpl39
tags=100%, list=24%, signal=79%	Rplp2/Rps17/Rps5/Rps8/Rpl31/Rps26/Rpl18/Uba52/Rpsa/Rps4x/Rpl19/Rps24/Rps20/Rpl8/Rps7/Rpl37/Rpl21/Rpl38/Rpl35/Rps27/Rpl30/Rps21/Rpl18a/Rps12/Rpl7a/Rpl13/Rps16/Rps27a/Rps2/Rpl24/Rps13/Rpl13a/Rpl3
tags=100%, list=23%, signal=78%	Rps17/Rps5/Rps8/Rps26/Uba52/Rpsa/Rps4x/Rps24/Rps20/Rps7/Rps27/Rps21/Rps12/Rps16/Rps27a/Rps2/Rps13/Rps10/Rps3/Rps19/Rps28
tags=96%, list=24%, signal=74%	Mrpl52/Rplp2/Rpl31/Rpl18/Uba52/Rpl19/Rpl8/Rpl37/Rpl21/Rpl38/Rpl35/Rpl30/Rpl18a/Rpl7a/Rpl13/Rpl24/Rpl13a/Rpl34/Rpl15/Rpl14/Rpl28/Rpl36a/Rpl35a/Rpl39
tags=96%, list=24%, signal=76%	Mrps6/Mrpl52/Mrps12/Rplp2/Rps17/Rps5/Rps8/Rpl31/Rps26/Rpl18/Uba52/Rpsa/Rps4x/Rpl19/Rps24/Rps20/Rpl8/Rps7/Rpl37/Rpl21/Rpl38/Rpl35/Rps27/Rpl30/Rps21/Rpl18a/Rps12/Rpl7a/Rpl13/Rps16/Rps27a/Rps2/Rpl
tags=92%, list=25%, signal=73%	Mrps6/Mrpl52/Mrps12/Rplp2/Rps17/Rps5/Rps8/Rpl31/Rps26/Rpl18/Uba52/Rpsa/Rps4x/Rpl19/Rps24/Rpl22l1/Rps20/Rpl8/Rps7/Rpl37/Rpl21/Rpl38/Rpl35/Rps27/Rpl30/Rps21/Rpl18a/Rps12/Rpl7a/Rpl13/Rps16/Rps27a/R
tags=96%, list=23%, signal=75%	Mrps6/Mrps12/Rps17/Rps5/Rps8/Rps26/Uba52/Rpsa/Rps4x/Rps24/Rps20/Rps7/Rps27/Rps21/Rps12/Rps16/Rps27a/Rps2/Rps13/Rps10/Rps3/Rps19/Rps28
tags=96%, list=24%, signal=76%	Mrps6/Mrpl52/Mrps12/Rplp2/Rps17/Rps5/Rps8/Rpl31/Rps26/Rpl18/Uba52/Rpsa/Rps4x/Rpl19/Rps24/Rpl22l1/Rps20/Rpl8/Rps7/Rpl37/Rpl21/Rpl38/Rpl35/Rps27/Rpl30/Rps21/Rpl18a/Rps12/Rpl7a/Rpl13/Rps16/Rps27a/R
tags=69%, list=24%, signal=56%	Tubb5/Tuba1a/Mrps6/Mrpl52/Csrp1/Mrps12/Actg1/Rplp2/Rps17/Rps5/Rps8/Rpl31/Rps26/Rpl18/Uba52/Rpsa/Rps4x/Rpl19/Rps24/Rpl22l1/Rps20/Rpl8/Rps7/Tuba1b/Rpl37/Rpl21/Rpl38/Rpl35/Rps27/Rpl30/Rps21/Rpl18a/R
tags=100%, list=8%, signal=93%	mt-Atp6/mt-Cytb/mt-Nd1/mt-Co1/mt-Co3/mt-Nd2/mt-Nd5/mt-Nd4l/mt-Nd6
tags=92%, list=8%, signal=85%	mt-Nd4/mt-Atp6/mt-Cytb/mt-Nd1/mt-Co1/mt-Co3/mt-Nd2/mt-Co2/mt-Nd5/mt-Nd4l/mt-Nd6
tags=100%, list=8%, signal=93%	mt-Atp6/mt-Cytb/mt-Nd1/mt-Co1/mt-Co3/mt-Nd2/mt-Nd5/mt-Nd4l/mt-Nd6
tags=75%, list=8%, signal=70%	mt-Nd4/mt-Nd3/mt-Atp6/mt-Cytb/mt-Nd1/mt-Co1/mt-Atp8/mt-Co3/mt-Nd2/mt-Co2/mt-Nd5/mt-Nd6
tags=100%, list=8%, signal=93%	mt-Atp6/mt-Cytb/mt-Nd1/mt-Co1/mt-Co3/mt-Nd2/mt-Co2/mt-Nd5/mt-Nd4l/mt-Nd6
tags=76%, list=8%, signal=71%	mt-Nd4/Crebbp/mt-Nd3/mt-Atp6/mt-Cytb/mt-Nd1/mt-Co1/mt-Atp8/mt-Co3/mt-Nd2/mt-Co2/mt-Nd5/mt-Nd6
tags=57%, list=25%, signal=48%	Hspa1a/Hspa8/Hspa1b/Gapdh/Mrps6/Dazap1/Ptges3/Mrpl52/Mrps12/Pop7/Zfp36/Rplp2/Lsm2/Fcf1/Rps17/Hnrnpab/Rps5/Eif3f/Rps8/Rpl31/Rbm17/Rps26/Rpl18/Uba52/Rpsa/Rps4x/Rpl19/Rps24/Nhp2/Rps20/Rpl8/Rps7/
tags=100%, list=8%, signal=93%	mt-Atp6/mt-Cytb/mt-Nd1/mt-Co3/mt-Nd2/mt-Nd5/mt-Nd4l/mt-Nd6
tags=100%, list=8%, signal=93%	mt-Atp6/mt-Cytb/mt-Nd1/mt-Co3/mt-Nd2/mt-Nd5/mt-Nd4l/mt-Nd6
tags=100%, list=8%, signal=93%	mt-Atp6/mt-Cytb/mt-Nd1/mt-Co3/mt-Nd2/mt-Nd5/mt-Nd4l/mt-Nd6
tags=83%, list=8%, signal=77%	mt-Nd4/mt-Atp6/mt-Cytb/mt-Nd1/mt-Co1/mt-Co3/mt-Nd2/mt-Nd5/mt-Nd4l/mt-Nd6
tags=64%, list=2%, signal=63%	Dnajb1/Hspa1a/Hspb1/Hspa8/Hspa1b/Hsp90aa1/Hsp90ab1
tags=45%, list=4%, signal=45%	Dnajb1/Hspa1a/Hspe1/Hspa8/Hspa1b/Dnaja1/Hsp90aa1/Hsp90ab1/Ptges3/Hspd1
tags=83%, list=8%, signal=77%	mt-Nd4/mt-Atp6/mt-Cytb/mt-Nd1/mt-Co1/mt-Co3/mt-Nd2/mt-Nd5/mt-Nd4l/mt-Nd6
tags=30%, list=4%, signal=29%	Dnajb1/Hspa1a/Hspb1/Hspe1/Hspa8/Hspa1b/Dnaja1/Hsp90aa1/Hsp90ab1/Ptges3/Hspd1
tags=83%, list=8%, signal=77%	mt-Nd4/mt-Atp6/mt-Cytb/mt-Nd1/mt-Co1/mt-Co3/mt-Nd2/mt-Nd5/mt-Nd4l/mt-Nd6
tags=83%, list=8%, signal=77%	mt-Nd4/mt-Atp6/mt-Cytb/mt-Nd1/mt-Co1/mt-Co3/mt-Nd2/mt-Nd5/mt-Nd4l/mt-Nd6
tags=83%, list=8%, signal=77%	mt-Nd4/mt-Atp6/mt-Cytb/mt-Nd1/mt-Co1/mt-Co3/mt-Nd2/mt-Nd5/mt-Nd4l/mt-Nd6
tags=100%, list=8%, signal=93%	mt-Cytb/mt-Nd1/mt-Co1/mt-Co3/mt-Co2/mt-Nd5/mt-Nd6
tags=67%, list=6%, signal=63%	Dnajb1/Hspa1a/Hspe1/Hspa8/Hspa1b/Ptges3/Hspd1/Hsph1
tags=77%, list=8%, signal=72%	mt-Nd4/mt-Nd3/mt-Atp6/mt-Cytb/mt-Nd1/mt-Co3/mt-Nd2/mt-Nd5/mt-Nd4l/mt-Nd6
tags=73%, list=8%, signal=68%	mt-Nd4/mt-Nd3/mt-Atp6/mt-Cytb/mt-Nd1/mt-Co1/mt-Co3/mt-Nd2/mt-Co2/mt-Nd5/mt-Nd6
tags=73%, list=8%, signal=68%	mt-Nd4/mt-Nd3/mt-Atp6/mt-Cytb/mt-Nd1/mt-Co1/mt-Co3/mt-Nd2/mt-Co2/mt-Nd5/mt-Nd6
tags=100%, list=8%, signal=93%	mt-Nd1/mt-Co1/mt-Co3/mt-Co2/mt-Nd5/mt-Nd6
tags=100%, list=8%, signal=93%	mt-Nd3/mt-Atp6/mt-Nd1/mt-Nd2/mt-Nd5/mt-Nd6
tags=100%, list=8%, signal=93%	mt-Nd3/mt-Atp6/mt-Nd1/mt-Nd2/mt-Nd5/mt-Nd6
tags=100%, list=8%, signal=93%	mt-Nd3/mt-Atp6/mt-Nd1/mt-Nd2/mt-Nd5/mt-Nd6
tags=100%, list=8%, signal=93%	mt-Nd1/mt-Co1/mt-Co3/mt-Co2/mt-Nd5/mt-Nd6
tags=100%, list=8%, signal=93%	mt-Nd3/mt-Atp6/mt-Nd1/mt-Nd2/mt-Nd5/mt-Nd6
tags=100%, list=8%, signal=93%	mt-Nd1/mt-Co1/mt-Co3/mt-Co2/mt-Nd5/mt-Nd6
tags=100%, list=8%, signal=93%	mt-Nd3/mt-Atp6/mt-Nd1/mt-Nd2/mt-Nd5/mt-Nd6
tags=100%, list=8%, signal=93%	mt-Nd3/mt-Atp6/mt-Nd1/mt-Nd2/mt-Nd5/mt-Nd6
tags=100%, list=8%, signal=93%	mt-Nd1/mt-Co1/mt-Co3/mt-Co2/mt-Nd5/mt-Nd6
tags=100%, list=8%, signal=93%	mt-Nd3/mt-Atp6/mt-Nd1/mt-Nd2/mt-Nd5/mt-Nd6
tags=100%, list=8%, signal=93%	mt-Nd1/mt-Co1/mt-Co3/mt-Co2/mt-Nd5/mt-Nd6
tags=100%, list=8%, signal=93%	mt-Nd1/mt-Co1/mt-Co3/mt-Co2/mt-Nd5/mt-Nd6
tags=100%, list=8%, signal=93%	mt-Nd1/mt-Co1/mt-Co3/mt-Co2/mt-Nd5/mt-Nd6
tags=100%, list=8%, signal=93%	mt-Nd1/mt-Co1/mt-Co3/mt-Co2/mt-Nd5/mt-Nd6
tags=100%, list=8%, signal=93%	mt-Nd1/mt-Co1/mt-Co3/mt-Co2/mt-Nd5/mt-Nd6
tags=70%, list=6%, signal=66%	Dnajb1/Hspa1a/Hspe1/Hspa8/Hspa1b/Ptges3/Hsph1
tags=53%, list=6%, signal=51%	Dnajb1/Hspa1a/Hspb1/Hspe1/Hspa8/Hspa1b/Ptges3/Hsph1
tags=55%, list=26%, signal=46%	Hspb1/Ppp1r15a/Gapdh/Mrps6/Gpx1/Mrpl52/Mrps12/Zfp36/Rplp2/Rps17/Rps5/Eif3f/Rps8/Rpl31/Rps26/Rpl18/Uba52/Dhps/Rpsa/Rps4x/Eef1b2/Rpl19/Rps24/Vapa/Rpl22l1/Rps20/Eif2b2/Rpl8/Rps7/Rpl37/Rpl21/Rpl38/Rp
tags=80%, list=13%, signal=71%	Zeb2/mt-Nd4/mt-Nd3/mt-Atp6/mt-Cytb/mt-Nd1/mt-Co1/mt-Co3/mt-Nd2/mt-Co2/mt-Nd5/mt-Nd6
tags=100%, list=23%, signal=78%	Rps17/Rpl31/Rps26/Rpl18/Rps24/Rps20/Rps7/Rpl35/Rps27/Rpl15/Rps10/Rpl35a/Rps19/Rps28
tags=100%, list=23%, signal=78%	Rps17/Rpl31/Rps26/Rpl18/Rps24/Rps20/Rps7/Rpl35/Rps27/Rpl15/Rps10/Rpl35a/Rps19/Rps28
tags=100%, list=23%, signal=78%	Rps17/Rpl31/Rps26/Rpl18/Rps24/Rps20/Rps7/Rpl35/Rps27/Rpl15/Rps10/Rpl35a/Rps19/Rps28
tags=100%, list=23%, signal=78%	Rps17/Rpl31/Rps26/Rpl18/Rps24/Rps20/Rps7/Rpl35/Rps27/Rpl15/Rps10/Rpl35a/Rps19/Rps28
tags=100%, list=23%, signal=78%	Rps17/Rpl31/Rps26/Rpl18/Rps24/Rps20/Rps7/Rpl35/Rps27/Rpl15/Rps10/Rpl35a/Rps19/Rps28
tags=100%, list=23%, signal=78%	Rps17/Rpl31/Rps26/Rpl18/Rps24/Rps20/Rps7/Rpl35/Rps27/Rpl15/Rps10/Rpl35a/Rps19/Rps28
tags=100%, list=23%, signal=78%	Rps17/Rpl31/Rps26/Rpl18/Rps24/Rps20/Rps7/Rpl35/Rps27/Rpl15/Rps10/Rpl35a/Rps19/Rps28
tags=100%, list=23%, signal=78%	Rps17/Rpl31/Rps26/Rpl18/Rps24/Rps20/Rps7/Rpl35/Rps27/Rpl15/Rps10/Rpl35a/Rps19/Rps28
tags=100%, list=23%, signal=78%	Rps17/Rpl31/Rps26/Rpl18/Rps24/Rps20/Rps7/Rpl35/Rps27/Rpl15/Rps10/Rpl35a/Rps19/Rps28
tags=49%, list=26%, signal=43%	Hspb1/Ppp1r15a/Phlda1/Klf4/Gapdh/Myc/Mrps6/Mrpl52/Azin1/Mrps12/Dbi/Zfp36/Rplp2/Rps17/Umps/Rps5/Eif3f/Rps8/Rpl31/Rps26/Rpl18/Uba52/Dhps/Rpsa/Rps4x/Eef1b2/Rpl19/Rps24/Tspo/Atg12/Vapa/Rpl22l1/Rps20/
tags=58%, list=12%, signal=53%	Matr3/Cfl2/mt-Nd4/mt-Nd3/mt-Atp6/mt-Cytb/mt-Nd1/mt-Co1/mt-Atp8/mt-Co3/mt-Nd2/mt-Co2/mt-Nd5/mt-Nd6
tags=75%, list=8%, signal=70%	mt-Nd4/mt-Nd3/mt-Nd1/mt-Co1/mt-Co3/mt-Nd2/mt-Co2/mt-Nd5/mt-Nd6
tags=75%, list=8%, signal=70%	mt-Nd4/mt-Nd3/mt-Nd1/mt-Co1/mt-Co3/mt-Nd2/mt-Co2/mt-Nd5/mt-Nd6
tags=48%, list=26%, signal=42%	Hspb1/Ppp1r15a/Phlda1/Klf4/Hsp90aa1/Hsp90ab1/Id3/Gapdh/Myc/Mrps6/Ptges3/Mrpl52/Mrps12/Pold4/Dbi/Zfp36/Rplp2/Rps17/Rps5/Eif3f/Rps8/Rpl31/Rps26/Rpl18/Sirt1/Uba52/Dhps/Nap1l1/Rpsa/Rps4x/Eef1b2/Rpl19/
tags=71%, list=8%, signal=67%	mt-Nd4/mt-Nd3/mt-Atp6/mt-Nd1/mt-Co1/mt-Co3/mt-Nd2/mt-Co2/mt-Nd5/mt-Nd6
tags=52%, list=8%, signal=49%	mt-Nd4/mt-Nd3/mt-Cytb/mt-Nd1/mt-Co1/mt-Co3/mt-Nd2/mt-Co2/mt-Nd5/mt-Nd4l/mt-Nd6
tags=52%, list=8%, signal=49%	mt-Nd4/mt-Nd3/mt-Cytb/mt-Nd1/mt-Co1/mt-Co3/mt-Nd2/mt-Co2/mt-Nd5/mt-Nd4l/mt-Nd6
tags=100%, list=1%, signal=99%	Hspa1a/Hspa8/Hspa1b/Dnaja1
tags=88%, list=4%, signal=84%	mt-Cytb/mt-Nd1/mt-Co1/mt-Co3/mt-Co2/mt-Nd5/mt-Nd6
tags=65%, list=8%, signal=60%	mt-Nd4/mt-Atp6/mt-Cytb/mt-Nd1/mt-Co1/mt-Co3/mt-Nd2/mt-Co2/mt-Nd5/mt-Nd4l/mt-Nd6
tags=100%, list=26%, signal=75%	Rps17/Rpl31/Rps26/Rpl18/Rps24/Rps20/Rps7/Rpl35/Rps27/Rpl15/Rps10/Rpl35a/Rps19/Rps28/Tmco1
tags=67%, list=8%, signal=62%	mt-Nd4/mt-Atp6/mt-Cytb/mt-Nd1/mt-Co1/mt-Co3/mt-Nd2/mt-Nd5/mt-Nd4l/mt-Nd6
tags=52%, list=8%, signal=49%	mt-Nd4/mt-Nd3/mt-Cytb/Atp2b1/mt-Nd1/mt-Co1/mt-Co3/mt-Nd2/mt-Co2/mt-Nd5/mt-Nd4l/mt-Nd6
tags=55%, list=8%, signal=51%	mt-Nd4/mt-Nd3/mt-Cytb/mt-Nd1/mt-Co1/mt-Co3/mt-Nd2/mt-Co2/mt-Nd5/mt-Nd4l/mt-Nd6
tags=81%, list=11%, signal=74%	Mecp2/Ndufv1/Sptbn1/mt-Nd4/mt-Nd3/mt-Atp6/mt-Nd1/mt-Co1/mt-Co3/mt-Nd2/mt-Co2/mt-Nd5/mt-Nd6
tags=80%, list=8%, signal=74%	mt-Nd4/mt-Nd1/mt-Co1/mt-Atp8/mt-Co3/mt-Co2/mt-Nd5/mt-Nd6
tags=62%, list=8%, signal=58%	mt-Nd4/mt-Nd3/mt-Atp6/mt-Nd1/mt-Co1/mt-Co3/mt-Nd2/mt-Co2/mt-Nd5/mt-Nd6
tags=94%, list=23%, signal=73%	Actg1/Rps17/Rpl31/Rps26/Rpl18/Rps24/Rps20/Rps7/Rpl35/Rps27/Rpl15/Rps10/Rpl35a/Rps19/Rps28
tags=88%, list=8%, signal=81%	mt-Nd4/mt-Nd1/mt-Co1/mt-Co3/mt-Co2/mt-Nd5/mt-Nd6
tags=88%, list=8%, signal=81%	mt-Nd4/mt-Nd1/mt-Co1/mt-Co3/mt-Co2/mt-Nd5/mt-Nd6
tags=64%, list=21%, signal=52%	Hivep2/Mecp2/Sars/Cux1/Sptbn1/Tcf4/mt-Nd4/mt-Nd3/Kcnq5/mt-Atp6/Ralgapa1/Pgap1/mt-Nd1/mt-Nd2/mt-Nd5/Trappc9/Nsf/mt-Nd6
tags=94%, list=23%, signal=73%	Rps17/Rpl31/Rps26/Rpl18/Rps24/Rps20/Rps7/Rpl35/Rps27/Rpl15/Rps10/Pgk1/Rpl35a/Rps19/Rps28
tags=52%, list=8%, signal=49%	mt-Nd4/mt-Nd3/mt-Atp6/mt-Cytb/mt-Nd1/mt-Co1/mt-Co3/mt-Nd2/mt-Co2/mt-Nd5/mt-Nd4l/mt-Nd6
tags=82%, list=11%, signal=74%	Sptbn1/mt-Nd4/mt-Nd3/mt-Atp6/Ralgapa1/mt-Nd1/mt-Nd2/mt-Nd5/mt-Nd6
tags=64%, list=8%, signal=60%	mt-Nd4/mt-Atp6/mt-Cytb/mt-Nd1/mt-Co3/mt-Nd2/mt-Nd5/mt-Nd4l/mt-Nd6
tags=64%, list=8%, signal=60%	mt-Nd4/mt-Atp6/mt-Cytb/mt-Nd1/mt-Co3/mt-Nd2/mt-Nd5/mt-Nd4l/mt-Nd6
tags=67%, list=8%, signal=62%	mt-Nd4/mt-Nd1/mt-Co1/mt-Atp8/mt-Co3/mt-Co2/mt-Nd5/mt-Nd6
tags=44%, list=11%, signal=40%	Dnajb1/Hspa1a/Hspa8/Hspa1b/Dnaja1/Hsp90ab1/Ptges3/Zfp36/Fkbp4/Dnajb9/Ahsa1
tags=43%, list=21%, signal=37%	Ctnnb1/Hivep2/Sars/Polr2a/Ndufv1/H2-Q7/Cux1/Med13/Ankrd11/Malt1/Son/Tcirg1/Foxp1/Kras/Zeb2/Tcf12/Sptbn1/Diaph1/Tcf4/Kmt2e/Ddx6/mt-Nd4/Crebbp/mt-Nd3/Kcnq5/mt-Atp6/Sf3b1/Aff4/Smchd1/mt-Cytb/Birc3/Ral
tags=29%, list=4%, signal=29%	Hspa1a/Hspb1/Jun/Hspa1b/Klf2/Fos/Dnaja1/Rhob/Chchd2/Myc/Gpx1
tags=45%, list=8%, signal=42%	mt-Nd4/mt-Nd3/mt-Atp6/mt-Cytb/mt-Nd1/mt-Co1/mt-Atp8/mt-Co3/mt-Nd2/mt-Co2/mt-Nd5/mt-Nd4l/mt-Nd6
tags=64%, list=8%, signal=60%	mt-Nd4/mt-Nd3/mt-Nd1/mt-Co1/mt-Co3/mt-Nd2/mt-Co2/mt-Nd5/mt-Nd6
tags=55%, list=8%, signal=51%	mt-Nd4/mt-Nd3/mt-Cytb/mt-Nd1/mt-Co1/mt-Co3/mt-Nd2/mt-Co2/mt-Nd5/mt-Nd4l/mt-Nd6
tags=62%, list=8%, signal=58%	mt-Nd4/mt-Atp6/mt-Cytb/mt-Nd1/mt-Co1/mt-Co3/mt-Nd2/mt-Nd5/mt-Nd4l/mt-Nd6
tags=64%, list=19%, signal=53%	Ndufv1/H2-Q7/Tcirg1/Kras/mt-Nd4/mt-Nd3/mt-Atp6/mt-Cytb/mt-Nd1/mt-Co1/mt-Atp8/mt-Co3/mt-Nd2/mt-Co2/mt-Nd5/mt-Nd6
tags=93%, list=23%, signal=73%	Rps17/Rpl31/Rps26/Rpl18/Rps24/Rps20/Rps7/Rpl35/Rps27/Rpl15/Rps10/Rpl35a/Rps19/Rps28
tags=52%, list=8%, signal=49%	mt-Nd4/mt-Nd3/mt-Cytb/mt-Nd1/mt-Co1/mt-Co3/mt-Nd2/mt-Co2/mt-Nd5/mt-Nd4l/mt-Nd6
tags=89%, list=10%, signal=81%	Tcf4/mt-Nd4/mt-Nd3/mt-Atp6/mt-Nd1/mt-Nd2/mt-Nd5/mt-Nd6
tags=59%, list=13%, signal=52%	Kras/Tcf12/mt-Nd4/mt-Atp6/mt-Cytb/mt-Nd1/mt-Co1/mt-Co3/mt-Nd2/mt-Co2/mt-Nd5/mt-Nd4l/mt-Nd6
tags=14%, list=5%, signal=15%	Dnajb1/Hspa1a/Hspb1/Jun/Hspa8/Hspa1b/Atf3/Ppp1r15a/Klf2/Fos/Klf4/Dnaja1/Hsp90aa1/Hsp90ab1/Rhob/Nfkbia/Dusp1/Pmaip1/Chchd2/Klf10/Myc/Gpx1/Hspd1/Traf4/Pold4/Zfp36
tags=78%, list=8%, signal=72%	mt-Nd4/mt-Nd1/mt-Co1/mt-Co3/mt-Co2/mt-Nd5/mt-Nd6
tags=55%, list=21%, signal=45%	Hivep2/Mecp2/Sars/Cux1/Med13/Sptbn1/Tcf4/mt-Nd4/mt-Nd3/Kcnq5/Setd2/mt-Atp6/Ralgapa1/Pgap1/mt-Nd1/mt-Nd2/Satb1/mt-Nd5/Trappc9/Nsf/mt-Nd6
tags=48%, list=8%, signal=45%	mt-Nd4/Ano6/mt-Nd3/Kcnq5/mt-Cytb/mt-Nd1/mt-Co1/mt-Nd2/mt-Nd5/mt-Nd4l/Pde4b/mt-Nd6
tags=88%, list=8%, signal=81%	mt-Nd4/mt-Nd1/mt-Co1/mt-Co3/mt-Co2/mt-Nd5/mt-Nd6
tags=46%, list=8%, signal=44%	mt-Nd4/mt-Nd3/mt-Atp6/mt-Cytb/mt-Nd1/mt-Co1/mt-Atp8/mt-Co3/mt-Nd2/mt-Co2/mt-Nd5/mt-Nd4l/mt-Nd6
tags=88%, list=8%, signal=81%	mt-Nd4/mt-Nd3/mt-Atp6/mt-Nd1/mt-Nd2/mt-Nd5/mt-Nd6
tags=64%, list=4%, signal=62%	mt-Cytb/mt-Nd1/mt-Co1/mt-Co3/mt-Co2/mt-Nd5/mt-Nd6
tags=64%, list=4%, signal=62%	mt-Cytb/mt-Nd1/mt-Co1/mt-Co3/mt-Co2/mt-Nd5/mt-Nd6
tags=25%, list=6%, signal=25%	Hspa1a/Jun/Hspa8/Hspa1b/Ubb/Dnaja1/Hsp90aa1/Hsp90ab1/Tubb5/Nfkbia/Cacybp/Hspd1/Traf4/Actg1/Ubc
tags=88%, list=8%, signal=81%	mt-Nd4/mt-Nd1/mt-Co1/mt-Co3/mt-Co2/mt-Nd5/mt-Nd6
tags=78%, list=8%, signal=72%	mt-Nd4/mt-Nd1/mt-Co1/mt-Co3/mt-Co2/mt-Nd5/mt-Nd6
tags=43%, list=10%, signal=40%	Tcf4/mt-Nd4/Herc1/mt-Nd3/Kcnq5/Setd2/mt-Atp6/mt-Cytb/Pgap1/mt-Nd1/mt-Co1/mt-Co3/mt-Nd2/mt-Co2/mt-Nd5/mt-Nd6
tags=94%, list=25%, signal=71%	Rpl31/Rps26/Rpl18/Rpl19/Rpl8/Rpl38/Rpl30/Rps21/Rpl18a/Rpl7a/Rpl24/Rpl36a/Rps28/Rpl39/Larp4b
tags=88%, list=8%, signal=81%	mt-Nd4/mt-Nd1/mt-Co1/mt-Co3/mt-Co2/mt-Nd5/mt-Nd6
tags=58%, list=6%, signal=55%	Dnajb1/Hspa1a/Jun/Atf3/Klf2/Cited2/Egr1
tags=93%, list=23%, signal=73%	Rps17/Rpl31/Rps26/Rpl18/Rps24/Rps20/Rps7/Rpl35/Rps27/Rpl15/Rps10/Rpl35a/Rps19/Rps28
tags=78%, list=8%, signal=72%	mt-Nd4/mt-Nd3/mt-Atp6/mt-Nd1/mt-Nd2/mt-Nd5/mt-Nd6
tags=70%, list=4%, signal=68%	mt-Cytb/mt-Nd1/mt-Co1/mt-Co3/mt-Co2/mt-Nd5/mt-Nd6
tags=88%, list=8%, signal=81%	mt-Nd4/mt-Nd3/mt-Atp6/mt-Nd1/mt-Nd2/mt-Nd5/mt-Nd6
tags=89%, list=13%, signal=78%	Zeb2/mt-Nd4/mt-Nd1/mt-Co1/mt-Co3/mt-Co2/mt-Nd5/mt-Nd6
tags=43%, list=8%, signal=40%	mt-Nd4/mt-Nd3/mt-Cytb/Atp2b1/mt-Nd1/mt-Co1/mt-Co3/mt-Nd2/mt-Co2/mt-Nd5/mt-Nd4l/mt-Nd6
tags=69%, list=11%, signal=63%	Sptbn1/Ubtf/mt-Nd4/mt-Nd3/mt-Atp6/mt-Nd1/mt-Nd2/mt-Nd5/mt-Nd6
tags=89%, list=26%, signal=67%	Rps17/Umps/Rpl31/Rps26/Rpl18/Rps24/Rps20/Rps7/Rpl35/Rps27/Rpl15/Rps10/Rpl35a/Rps19/Rps28/H2-Ab1
tags=54%, list=4%, signal=52%	mt-Cytb/mt-Nd1/mt-Co1/mt-Co3/mt-Co2/mt-Nd5/mt-Nd6
tags=35%, list=4%, signal=34%	Dnajb1/Hspa1a/Hspa1b/Fos/Dnaja1/Hsp90aa1/Hsp90ab1/Nfkbia/Hspd1
tags=48%, list=8%, signal=45%	mt-Nd4/mt-Nd3/mt-Cytb/mt-Nd1/mt-Co1/mt-Co3/mt-Nd2/mt-Co2/mt-Nd5/mt-Nd4l/mt-Nd6
tags=88%, list=23%, signal=68%	Rps17/Rpl31/Rps26/Rpl18/Rps24/Rps20/Rps7/Rpl35/Rps27/Rpl15/Rps10/Rpl35a/Rps19/Rps28
tags=88%, list=23%, signal=68%	Rps17/Rpl31/Rps26/Rpl18/Rps24/Rps20/Rps7/Rpl35/Rps27/Rpl15/Rps10/Rpl35a/Rps19/Rps28
tags=88%, list=23%, signal=68%	Rps17/Rpl31/Rps26/Rpl18/Rps24/Rps20/Rps7/Rpl35/Rps27/Rpl15/Rps10/Rpl35a/Rps19/Rps28

tags=47%, list=14%, signal=42%	Fbxo38/Fbxo11/Matr3/Sh3kbp1/mt-Nd4/Ganab/Crebbp/Kcnq5/mt-Atp6/mt-Cytb/mt-Nd1/mt-Co1/mt-Co3/mt-Nd2/mt-Co2/mt-Nd5/mt-Nd4l/mt-Nd6
tags=78%, list=8%, signal=72%	mt-Nd4/mt-Nd3/mt-Atp6/mt-Nd1/mt-Nd2/mt-Nd5/mt-Nd6
tags=43%, list=8%, signal=41%	mt-Nd4/mt-Nd3/mt-Atp6/mt-Cytb/mt-Nd1/mt-Co1/mt-Atp8/mt-Co3/mt-Nd2/mt-Co2/mt-Nd5/mt-Nd4l/mt-Nd6
tags=52%, list=15%, signal=45%	Med13/Kras/Ubtf/Tcf4/Kmt2e/Ddx6/mt-Nd4/mt-Nd1/mt-Co1/mt-Co3/mt-Co2/Satb1/mt-Nd5/mt-Nd6
tags=44%, list=12%, signal=40%	Atad1/Tcf4/Kmt2e/mt-Nd4/mt-Nd3/Mef2c/Kcnq5/mt-Cytb/mt-Nd1/mt-Co1/mt-Co3/mt-Nd2/mt-Co2/mt-Nd5/Nsf/mt-Nd6
tags=78%, list=8%, signal=72%	mt-Nd4/mt-Nd3/mt-Atp6/mt-Nd1/mt-Nd2/mt-Nd5/mt-Nd6
tags=50%, list=10%, signal=46%	Kmt2e/Ddx6/mt-Nd4/mt-Nd3/mt-Atp6/Ralgapa1/mt-Nd1/mt-Nd2/Satb1/mt-Nd5/mt-Nd6
tags=52%, list=21%, signal=43%	Hivep2/Mecp2/Sars/Cux1/Nfkb2/Med13/Son/Zeb2/Tcf4/mt-Nd4/mt-Nd3/Kcnq5/Setd2/mt-Atp6/Pgap1/mt-Nd1/mt-Nd2/Satb1/mt-Nd5/Trappc9/mt-Nd6
tags=76%, list=8%, signal=71%	Tpp1/Ctnnb1/Son/mt-Nd4/mt-Atp6/mt-Cytb/mt-Nd1/mt-Co1/mt-Co3/mt-Nd2/mt-Nd5/mt-Nd4l/mt-Nd6
tags=63%, list=31%, signal=46%	Polg/Ranbp2/Tmem127/Stat4/Daxx/Rnf6/Ctnnb1/Krit1/Ndufv1/H2-Q7/Fus/Malt1/Foxp1/Kras/Zeb2/Matr3/Sptbn1/Kmt2e/mt-Nd4/Syk/mt-Nd3/mt-Atp6/mt-Cytb/Birc3/mt-Nd1/mt-Co1/mt-Co3/mt-Nd2/mt-Co2/mt-Nd5/mt-N
tags=41%, list=8%, signal=38%	mt-Nd4/mt-Nd3/mt-Atp6/mt-Cytb/mt-Nd1/mt-Co1/mt-Atp8/mt-Co3/mt-Nd2/mt-Co2/mt-Nd5/mt-Nd4l/mt-Nd6
tags=54%, list=14%, signal=48%	Fbxo38/Fbxo11/Matr3/Sh3kbp1/Ganab/Crebbp/Kcnq5/mt-Cytb/mt-Nd1/mt-Co1/mt-Co3/mt-Co2/mt-Nd5/mt-Nd6
tags=71%, list=18%, signal=59%	H2-Q7/Tcirg1/Foxp1/Kras/mt-Nd4/Aff4/mt-Nd1/mt-Co1/mt-Co3/mt-Co2/mt-Nd5/mt-Nd6
tags=80%, list=10%, signal=72%	Diaph1/mt-Cytb/mt-Nd1/mt-Co1/mt-Co3/mt-Co2/mt-Nd5/mt-Nd6
tags=71%, list=18%, signal=59%	Atp2c1/H2-Q7/mt-Nd4/mt-Nd1/mt-Co1/mt-Co3/mt-Co2/mt-Nd5/mt-Nd6/Adgre5
tags=48%, list=8%, signal=45%	mt-Nd4/mt-Nd3/mt-Cytb/mt-Nd1/mt-Co1/mt-Co3/mt-Nd2/mt-Co2/mt-Nd5/mt-Nd6
tags=50%, list=23%, signal=40%	Pml/Bcl2/Sh2b3/Ifngr1/H2-Q7/Malt1/Bcl6/Tcirg1/Foxp1/Sptbn1/Tcf4/Sh3kbp1/mt-Nd4/Syk/mt-Nd3/Bach2/mt-Atp6/mt-Cytb/Birc3/mt-Nd1/mt-Co1/mt-Co3/mt-Nd2/mt-Co2/mt-Nd5/mt-Nd6
tags=85%, list=25%, signal=65%	Rpl31/Rps26/Rpl18/Rps4x/Rpl19/Rpl8/Rpl38/Rpl30/Rps21/Rpl18a/Rpl7a/Rpl24/Rpl36a/Rps3/Rps28/Rpl39/Larp4b
tags=93%, list=23%, signal=73%	Rps17/Rpl31/Rps26/Rpl18/Rps24/Rps20/Rps7/Rpl35/Rps27/Rpl15/Rps10/Rpl35a/Rps19/Rps28
tags=67%, list=10%, signal=61%	Tcf4/mt-Nd4/mt-Nd1/mt-Co1/mt-Co3/mt-Co2/mt-Nd5/mt-Nd6
tags=40%, list=2%, signal=40%	Dnajb1/Hspa1a/Hspa1b/Dnaja1/Hsp90aa1/Hsp90ab1
tags=64%, list=4%, signal=62%	mt-Cytb/mt-Nd1/mt-Co1/mt-Co3/mt-Co2/mt-Nd5/mt-Nd6
tags=70%, list=8%, signal=65%	mt-Nd4/mt-Nd3/mt-Atp6/mt-Nd1/mt-Nd2/mt-Nd5/mt-Nd6
tags=70%, list=8%, signal=65%	mt-Nd4/mt-Nd3/mt-Atp6/mt-Nd1/mt-Nd2/mt-Nd5/mt-Nd6
tags=62%, list=8%, signal=57%	mt-Nd4/mt-Nd1/mt-Co1/mt-Co3/Irs2/mt-Co2/mt-Nd5/mt-Nd6
tags=59%, list=21%, signal=48%	Hivep2/Mecp2/Cux1/Med13/Tcf4/mt-Nd4/mt-Nd3/Kcnq5/Setd2/mt-Atp6/mt-Nd1/mt-Nd2/Satb1/mt-Nd5/Trappc9/mt-Nd6
tags=55%, list=20%, signal=45%	Sars/Polr2a/Ndufv1/Zeb2/mt-Nd4/mt-Nd3/mt-Atp6/Pgap1/mt-Nd1/mt-Co1/mt-Co3/mt-Nd2/mt-Co2/mt-Nd5/Trappc9/mt-Nd6
tags=37%, list=8%, signal=35%	mt-Nd4/mt-Nd3/mt-Atp6/mt-Cytb/mt-Nd1/mt-Co1/mt-Atp8/mt-Co3/mt-Nd2/Irs2/mt-Co2/mt-Nd5/mt-Nd4l/mt-Nd6
tags=64%, list=8%, signal=59%	mt-Nd4/mt-Nd1/mt-Co1/mt-Co3/mt-Co2/mt-Nd5/mt-Nd6
tags=93%, list=23%, signal=73%	Rps17/Rpl31/Rps26/Rpl18/Rps24/Rps20/Rps7/Rpl35/Rps27/Rpl15/Rps10/Rpl35a/Rps19/Rps28
tags=39%, list=8%, signal=37%	Cfl2/mt-Nd4/mt-Atp6/mt-Cytb/mt-Nd1/mt-Co1/mt-Co3/mt-Nd2/mt-Co2/mt-Nd5/mt-Nd4l/mt-Nd6
tags=70%, list=8%, signal=65%	mt-Nd4/mt-Nd3/mt-Atp6/mt-Nd1/mt-Nd2/mt-Nd5/mt-Nd6
tags=44%, list=8%, signal=41%	mt-Nd4/mt-Nd3/mt-Atp6/mt-Nd1/mt-Co1/mt-Atp8/mt-Co3/mt-Nd2/mt-Co2/mt-Nd5/mt-Nd6
tags=70%, list=8%, signal=65%	mt-Nd4/mt-Nd3/mt-Atp6/mt-Nd1/mt-Nd2/mt-Nd5/mt-Nd6
tags=45%, list=8%, signal=43%	mt-Nd4/Crebbp/mt-Nd3/mt-Atp6/Pgap1/mt-Nd1/mt-Nd2/mt-Nd5/Trappc9/mt-Nd6
tags=56%, list=13%, signal=50%	Ctnnb1/Krit1/Ndufv1/Zeb2/Sptbn1/Kmt2e/mt-Nd4/Syk/mt-Nd3/mt-Atp6/mt-Cytb/mt-Nd1/mt-Co1/mt-Co3/mt-Nd2/mt-Co2/mt-Nd5/mt-Nd6
tags=44%, list=11%, signal=40%	Sptbn1/mt-Nd4/mt-Atp6/Sf3b1/Smchd1/mt-Cytb/mt-Nd1/mt-Co1/mt-Co3/mt-Nd2/mt-Co2/mt-Nd5/mt-Nd4l/mt-Nd6
tags=58%, list=8%, signal=54%	mt-Nd4/mt-Nd1/mt-Co1/mt-Co3/mt-Co2/mt-Nd5/mt-Nd6
tags=21%, list=7%, signal=21%	Dnajb1/Hspa1a/Jun/Hspa1b/Fos/Dnaja1/Hsp90aa1/Hsp90ab1/Rhob/Nfkbia/Nr4a1/Pmaip1/Chchd2/Myc/Gpx1/Hspd1/Actg1/Cited2/Egr1/Jund
tags=73%, list=12%, signal=65%	Rfc1/mt-Nd4/mt-Nd3/mt-Atp6/mt-Nd1/mt-Nd2/mt-Nd5/mt-Nd6
tags=53%, list=11%, signal=48%	Sptbn1/mt-Nd4/mt-Atp6/mt-Cytb/mt-Nd1/mt-Co3/mt-Nd2/mt-Nd5/mt-Nd4l/mt-Nd6
tags=50%, list=8%, signal=47%	mt-Nd4/mt-Nd3/mt-Cytb/mt-Nd1/mt-Co1/mt-Nd2/mt-Nd5/mt-Nd4l/mt-Nd6
tags=46%, list=12%, signal=42%	Rfc1/Sptbn1/Ppp1r15b/mt-Nd4/mt-Atp6/mt-Cytb/mt-Nd1/mt-Co1/mt-Co3/mt-Nd2/mt-Nd5/mt-Nd4l/mt-Nd6
tags=57%, list=8%, signal=53%	mt-Nd4/mt-Atp6/mt-Nd1/mt-Co1/mt-Co3/mt-Co2/mt-Nd5/mt-Nd6
tags=88%, list=23%, signal=68%	Rps17/Rpl31/Rps26/Rpl18/Rps24/Rps20/Rps7/Rpl35/Rps27/Rpl15/Rps10/Rpl35a/Rps19/Rps28
tags=88%, list=23%, signal=68%	Rps17/Rpl31/Rps26/Rpl18/Rps24/Rps20/Rps7/Rpl35/Rps27/Rpl15/Rps10/Rpl35a/Rps19/Rps28
tags=71%, list=18%, signal=59%	H2-Q7/Kras/mt-Nd4/mt-Cytb/mt-Nd1/mt-Co1/mt-Co3/mt-Co2/mt-Nd5/mt-Nd6
tags=55%, list=12%, signal=49%	Rfc1/mt-Nd4/mt-Nd3/mt-Atp6/mt-Nd1/mt-Co1/mt-Co3/mt-Nd2/mt-Co2/mt-Nd5/mt-Nd6
tags=80%, list=13%, signal=70%	Kras/mt-Nd4/mt-Nd1/mt-Co1/mt-Co3/mt-Co2/mt-Nd5/mt-Nd6
tags=47%, list=21%, signal=39%	Hivep2/Mecp2/Sars/Ndufv1/Fus/Cux1/Sptbn1/Ubtf/Tcf4/mt-Nd4/mt-Nd3/Kcnq5/mt-Atp6/mt-Cytb/Pgap1/mt-Nd1/mt-Co3/mt-Nd2/mt-Nd5/Trappc9/mt-Nd4l/mt-Nd6
tags=14%, list=5%, signal=15%	Hspa1a/Hspb1/Jun/Hspa1b/Atf3/Phlda1/Fos/Ubb/Klf4/Dnaja1/Hsp90aa1/Hsp90ab1/Rhob/Id3/Nr4a1/Pmaip1/Myc/Gpx1/Hspd1/Traf4/Zfp36
tags=93%, list=23%, signal=73%	Rps17/Rpl31/Rps26/Rpl18/Rps24/Rps20/Rps7/Rpl35/Rps27/Rpl15/Rps10/Rpl35a/Rps19/Rps28
tags=93%, list=23%, signal=73%	Rps17/Rpl31/Rps26/Rpl18/Rps24/Rps20/Rps7/Rpl35/Rps27/Rpl15/Rps10/Rpl35a/Rps19/Rps28
tags=35%, list=14%, signal=32%	Camk2d/Tcirg1/Lrrc8c/Diaph1/mt-Nd4/Ano6/Ahnak/Mef2c/Kcnq5/mt-Atp6/mt-Cytb/Cab39/Atp2b1/mt-Co1/mt-Atp8/mt-Co3/mt-Co2/mt-Nd5/Pde4b/Wnk1
tags=58%, list=8%, signal=54%	mt-Nd4/mt-Nd1/mt-Co1/mt-Co3/mt-Co2/mt-Nd5/mt-Nd6
tags=37%, list=14%, signal=33%	Camk2d/Tcirg1/Diaph1/mt-Nd4/Ano6/Ahnak/Mef2c/Kcnq5/mt-Atp6/mt-Cytb/Cab39/Atp2b1/mt-Co1/mt-Atp8/mt-Co3/mt-Co2/mt-Nd5/Pde4b/Wnk1
tags=82%, list=18%, signal=68%	Fus/Matr3/mt-Nd4/mt-Nd3/mt-Atp6/mt-Nd1/mt-Nd2/mt-Nd5/mt-Nd6
tags=50%, list=21%, signal=41%	Hivep2/Sars/Cux1/Zeb2/Sptbn1/Tcf4/mt-Nd4/Kcnq5/Setd2/Pgap1/mt-Nd1/mt-Co1/mt-Co3/mt-Co2/Satb1/mt-Nd5/Trappc9/mt-Nd6
tags=17%, list=8%, signal=18%	Hspa1a/Hspb1/Jun/Hspa1b/Atf3/Ppp1r15a/Phlda1/Ubb/Klf4/Dnaja1/Hsp90aa1/Hsp90ab1/Rhob/Id3/Nfkbia/Nr4a1/Gapdh/Pmaip1/Myc/Gpx1/Hspd1/Traf4/Zfp36/Fam32a/Cited2/Egr1/Dynll1/Gadd45b/Dedd2/Chchd10/Sirt1
tags=39%, list=8%, signal=37%	mt-Nd4/Crebbp/mt-Nd3/mt-Atp6/mt-Cytb/mt-Nd1/mt-Co1/mt-Atp8/mt-Co3/mt-Nd2/mt-Co2/mt-Nd5/mt-Nd4l/mt-Nd6
tags=53%, list=11%, signal=48%	Sptbn1/Ubtf/mt-Nd4/mt-Nd3/mt-Atp6/mt-Nd1/mt-Nd2/mt-Nd5/mt-Nd6/Wnk1
tags=45%, list=8%, signal=43%	mt-Nd4/mt-Nd3/mt-Atp6/mt-Nd1/mt-Co1/mt-Co3/mt-Nd2/mt-Co2/mt-Nd5/mt-Nd6
tags=45%, list=8%, signal=43%	mt-Nd4/mt-Nd3/mt-Atp6/mt-Nd1/mt-Co1/mt-Co3/mt-Nd2/mt-Co2/mt-Nd5/mt-Nd6
tags=37%, list=14%, signal=33%	Camk2d/Tcirg1/Diaph1/mt-Nd4/Ano6/Ahnak/Kcnq5/mt-Atp6/mt-Cytb/Cab39/Atp2b1/mt-Co1/mt-Atp8/mt-Co3/mt-Co2/mt-Nd5/Pde4b/Wnk1
tags=45%, list=2%, signal=45%	Dnajb1/Hspa1a/Hspa1b/Hsp90aa1/Hsp90ab1
tags=77%, list=18%, signal=64%	Fus/Son/Kras/mt-Nd4/mt-Nd1/mt-Co1/mt-Co3/mt-Co2/mt-Nd5/mt-Nd6
tags=37%, list=14%, signal=33%	Malt1/Bcl6/Tcirg1/Foxp1/Sptbn1/Tcf4/Sh3kbp1/mt-Nd4/Syk/mt-Nd3/Bach2/mt-Atp6/mt-Cytb/Birc3/mt-Nd1/mt-Co1/mt-Co3/mt-Nd2/mt-Co2/mt-Nd5/Trappc9/mt-Nd6
tags=84%, list=26%, signal=64%	Rps17/Rpl31/Rps26/Rpl18/Chchd10/Rps24/Rps20/Rps7/Rpl35/Rps27/Rpl15/Rps10/Rpl35a/Rps19/Rps28/Gars
tags=50%, list=8%, signal=47%	mt-Nd4/mt-Nd3/mt-Atp6/mt-Nd1/mt-Co1/mt-Co3/mt-Nd2/mt-Co2/mt-Nd5/mt-Nd6
tags=65%, list=15%, signal=59%	Plekhm1/Dpm1/Pten/Prpf8/Smarce1/Bptf/Xylt1/Chd7/Ube3b/Jmjd1c/Cltc/Tbce/Kat6a/Polg/Rere/Ube4b/Stat4/Minpp1/Celf2/Ctnnb1/Ndufv1/H2-Q7/Med13/Ankrd11/Son/Tcirg1/Kras/Zeb2/Rfc1/Diaph1/Kmt2e/Ddx6/mt-Nd4/
tags=60%, list=27%, signal=45%	Zmynd11/Minpp1/Celf2/Tpp1/Hivep2/Mecp2/Ndufv1/Cux1/Tnrc6a/Atad1/Tcf4/mt-Nd4/Kcnq5/mt-Atp6/mt-Nd1/mt-Co1/mt-Co3/mt-Co2/Satb1/mt-Nd5/mt-Nd6
tags=54%, list=8%, signal=50%	mt-Nd4/mt-Nd1/mt-Co1/mt-Co3/mt-Co2/mt-Nd5/mt-Nd6
tags=41%, list=10%, signal=38%	Med13/Kras/Ubtf/Kmt2e/Ddx6/mt-Nd4/Herc1/Mef2c/mt-Atp6/Pgap1/mt-Nd1/mt-Co1/mt-Co3/mt-Co2/Satb1/mt-Nd5/Trappc9/mt-Nd6
tags=44%, list=8%, signal=44%	Arcn1/Pik3r4/Atp6v0a1/Uvrag/Adam10/H13/Os9/Snx12/Bcl2/Ctnnb1/Rnf125/Sec24b/Ndufv1/H2-Q7/Birc2/Chchd3/Tcirg1/Lrrc8c/mt-Nd4/Ano6/Aftph/Ikbkb/Ahnak/Syk/mt-Nd3/Kcnq5/Stt3b/mt-Atp6/Vps37b/mt-Cytb/Itga4/
tags=56%, list=37%, signal=39%	Xylt1/Chd7/Stk11/Jmjd1c/Rad21/Cltc/Kmt2c/Tbce/Kat6a/Jak1/Polg/Rere/Ube4b/Stat1/Stat4/Zmynd11/Setx/Ep300/Daxx/Ivns1abp/Celf2/Ctnnb1/Rnf125/Hivep2/Mecp2/Polr2a/Ifngr1/H2-Q7/Fus/Usp7/Cux1/Nfkb2/Ankrd11/
tags=43%, list=8%, signal=41%	mt-Nd4/mt-Nd3/mt-Atp6/mt-Nd1/mt-Co1/mt-Co3/mt-Nd2/mt-Co2/mt-Nd5/mt-Nd6
tags=50%, list=4%, signal=48%	Hspa1a/Hspa8/Hspa1b/Hsp90aa1/Hspd1
tags=58%, list=10%, signal=53%	Hivep2/Mecp2/Cux1/Tcf4/mt-Nd4/mt-Nd3/Kcnq5/Setd2/mt-Atp6/mt-Nd1/mt-Nd2/Satb1/mt-Nd5/Trappc9/mt-Nd6
tags=33%, list=4%, signal=33%	Dnajb1/Hspe1/Hspa8/Dnaja1/Ptges3/Hspd1
tags=67%, list=21%, signal=54%	Hivep2/Mecp2/Cux1/Tcf4/mt-Nd4/Kcnq5/mt-Nd1/mt-Co1/mt-Co3/mt-Co2/mt-Nd5/mt-Nd6
tags=67%, list=21%, signal=54%	Hivep2/Mecp2/Cux1/Tcf4/mt-Nd4/Kcnq5/mt-Nd1/mt-Co1/mt-Co3/mt-Co2/mt-Nd5/mt-Nd6
tags=82%, list=26%, signal=61%	Rps17/Rpl31/Rps26/Rpl18/Rps24/Rps20/Rps7/Rpl35/Rps27/Rpl15/Rps10/Fip1l1/Rpl35a/Rps19/Rps28/Npm1/Ak2/Arhgap31
tags=53%, list=8%, signal=49%	mt-Nd4/mt-Atp6/mt-Cytb/mt-Nd1/mt-Co3/mt-Nd2/mt-Nd5/mt-Nd4l/mt-Nd6
tags=35%, list=8%, signal=33%	mt-Nd4/mt-Nd3/mt-Atp6/mt-Cytb/mt-Nd1/mt-Co1/mt-Atp8/mt-Co3/mt-Nd2/mt-Co2/mt-Nd5/mt-Nd4l/mt-Nd6
tags=23%, list=4%, signal=23%	Hspa1a/Hspb1/Jun/Hspa1b/Klf2/Fos/Rhob/Chchd2/Gpx1
tags=54%, list=8%, signal=50%	mt-Nd4/mt-Nd3/mt-Nd1/mt-Nd2/mt-Nd5/mt-Nd4l/mt-Nd6
tags=54%, list=8%, signal=50%	mt-Nd4/mt-Nd3/mt-Nd1/mt-Nd2/mt-Nd5/mt-Nd4l/mt-Nd6
tags=54%, list=8%, signal=50%	mt-Nd4/mt-Nd3/mt-Nd1/mt-Nd2/mt-Nd5/mt-Nd4l/mt-Nd6
tags=54%, list=8%, signal=50%	mt-Nd4/mt-Nd3/mt-Nd1/mt-Nd2/mt-Nd5/mt-Nd4l/mt-Nd6
tags=64%, list=36%, signal=43%	Chd7/Ube3b/Jmjd1c/Cltc/Tbce/Kat6a/Polg/Rere/Ube4b/Minpp1/Celf2/Ctnnb1/Ndufv1/H2-Q7/Son/Tcirg1/Kras/Zeb2/Rfc1/Diaph1/mt-Nd4/mt-Nd3/Stt3b/mt-Atp6/Aff4/mt-Cytb/mt-Nd1/mt-Co1/mt-Co3/mt-Nd2/mt-Co2/mt-N
tags=27%, list=4%, signal=27%	Hspa1a/Hspb1/Ubb/Dnaja1/Pmaip1/Myc/Gpx1
tags=42%, list=8%, signal=40%	mt-Nd4/mt-Atp6/Smchd1/mt-Cytb/mt-Nd1/mt-Co1/mt-Co3/mt-Nd2/mt-Nd5/mt-Nd4l/mt-Nd6
tags=100%, list=2%, signal=98%	Hspa1a/Hspa1b/Nfkbia
tags=83%, list=4%, signal=80%	Hspa1a/Hsp90aa1/Hsp90ab1/Ptges3/Hspd1
tags=54%, list=8%, signal=50%	mt-Nd4/mt-Nd3/mt-Atp6/mt-Nd1/mt-Nd2/mt-Nd5/mt-Nd6
tags=19%, list=4%, signal=19%	Hspa1a/Hspb1/Hspa1b/Atf3/Ubb/Dnaja1/Pmaip1/Myc/Gpx1
tags=65%, list=19%, signal=53%	Csnk1d/H2-Q7/Kras/mt-Nd4/mt-Cytb/mt-Nd1/mt-Co1/mt-Co3/mt-Co2/mt-Nd5/mt-Nd6
tags=35%, list=8%, signal=33%	mt-Nd4/mt-Nd3/mt-Atp6/mt-Cytb/mt-Nd1/mt-Co1/mt-Co3/mt-Nd2/mt-Co2/mt-Nd5/mt-Nd6/Wnk1
tags=42%, list=10%, signal=38%	Tcf4/Ganab/mt-Cytb/mt-Nd1/mt-Co1/mt-Co3/mt-Co2/mt-Nd5/mt-Nd6/Wnk1
tags=83%, list=4%, signal=81%	Klf4/Hsp90aa1/Hsp90ab1/Myc/Ptges3
tags=83%, list=4%, signal=81%	Klf4/Hsp90aa1/Hsp90ab1/Myc/Ptges3
tags=21%, list=4%, signal=20%	Hspa1a/Hspb1/Ppp1r15a/Ubb/Dnaja1/Pmaip1/Myc/Gpx1
tags=65%, list=21%, signal=52%	Ctnnb1/Krit1/Tcirg1/Kras/mt-Nd4/mt-Nd1/mt-Co1/mt-Co3/mt-Co2/mt-Nd5/mt-Nd6
tags=100%, list=1%, signal=100%	Hspa1a/Hspa1b
tags=100%, list=1%, signal=100%	Hspa1a/Hspa1b
tags=100%, list=1%, signal=100%	Hspa1a/Hspa1b
tags=100%, list=1%, signal=100%	Hspa1a/Hspa1b
tags=100%, list=1%, signal=100%	Hspa1a/Hspa1b
tags=100%, list=7%, signal=93%	Jun/Fos/Junb/Jund
tags=59%, list=37%, signal=40%	Smarce1/Bptf/Chd7/Jmjd1c/Rad21/Cltc/Ppp2ca/Kmt2c/Kat6a/Chd8/Rere/Phf21a/Ube4b/Zmynd11/Ep300/Celf2/Ctnnb1/Chd1/Hivep2/Mecp2/Sars/Polr2a/Ndufv1/Usp7/Cux1/Med13/Ankrd11/Son/Foxp1/Tcf12/Sptbn1/Ubtf
tags=48%, list=22%, signal=39%	Ccnd2/Ctnnb1/Rnf125/Mecp2/Ndufv1/Cep83/Usp7/Tcirg1/Kras/Sptbn1/mt-Nd4/Herc1/mt-Nd3/mt-Atp6/mt-Cytb/mt-Nd1/mt-Co1/mt-Co3/mt-Nd2/mt-Co2/mt-Nd5/mt-Nd6
tags=50%, list=8%, signal=47%	mt-Nd4/mt-Nd3/mt-Nd1/mt-Nd2/mt-Nd5/mt-Nd4l/mt-Nd6
tags=37%, list=21%, signal=32%	Chd1/Hivep2/Mecp2/Sars/Ndufv1/Fus/Cux1/Zfyve27/Tnrc6a/Tcirg1/Foxp1/Fbxo38/Zeb2/Rfc1/Atad1/Sptbn1/Ubtf/Ppp1r15b/Tcf4/Ddx6/mt-Nd4/Crebbp/mt-Nd3/Mef2c/Kcnq5/Setd2/mt-Atp6/mt-Cytb/Pgap1/mt-Nd1/mt-Co
tags=41%, list=10%, signal=38%	Tcf4/mt-Nd4/mt-Atp6/mt-Cytb/mt-Nd1/mt-Co1/mt-Co3/mt-Nd2/mt-Nd5/mt-Nd4l/mt-Nd6
tags=50%, list=21%, signal=41%	Hivep2/Sars/Cux1/Son/Diaph1/Tcf4/Kcnq5/mt-Cytb/Ralgapa1/Pgap1/mt-Nd1/mt-Co1/mt-Co3/mt-Co2/mt-Nd5/Trappc9/mt-Nd6
tags=60%, list=18%, signal=50%	H2-Q7/Tcirg1/Foxp1/Kras/mt-Nd4/Aff4/mt-Nd1/mt-Co1/mt-Co3/mt-Co2/mt-Nd5/mt-Nd6
tags=67%, list=5%, signal=64%	Hspa1a/Hspa8/Hspa1b/Actg1
tags=59%, list=41%, signal=38%	Dpm1/Pten/Pex7/Taf15/Apoe/Copb2/Smarce1/Slc12a6/Chd7/Nacc1/Cnot1/Cltc/Tbce/Polg/Ranbp2/Rere/Tufm/Ube4b/Stat4/Zmynd11/Setx/Minpp1/Celf2/Tpp1/Ctnnb1/Hivep2/Mecp2/Sars/Ndufv1/H2-Q7/Fus/Cux1/Zfyve2
tags=62%, list=18%, signal=52%	H2-Q7/Kras/Tcf4/mt-Nd4/mt-Nd1/mt-Co1/mt-Co3/mt-Co2/mt-Nd5/mt-Nd6
tags=57%, list=19%, signal=47%	Ndufv1/H2-Q7/Kras/mt-Nd4/mt-Nd3/mt-Atp6/mt-Nd1/mt-Co1/mt-Co3/mt-Nd2/mt-Co2/mt-Nd5/mt-Nd6
tags=84%, list=26%, signal=63%	Rps17/Rpl31/Rps26/Rpl18/Rps24/Rps20/Rps7/Rpl35/Rps27/Rpl15/Rps10/Rpl35a/Rps19/Rps28/Bin1/Tmco1
tags=56%, list=38%, signal=38%	Copb2/Smarce1/Flii/Bptf/Slc12a6/Ube3b/Cnot1/Cltc/Ppp2ca/Kmt2c/Tbce/Nrros/Rere/Ube4b/Exosc1/Zmynd11/Ep300/Minpp1/Celf2/Ctnnb1/Hivep2/Sars/Polr2a/Usp7/Cux1/Med13/Ankrd11/Son/Kras/Zeb2/Diaph1/Ubtf/Pp
tags=50%, list=8%, signal=47%	Tcirg1/mt-Nd4/mt-Atp6/mt-Cytb/mt-Co1/mt-Atp8/mt-Co3/mt-Co2/mt-Nd5
tags=53%, list=8%, signal=50%	mt-Nd4/mt-Cytb/mt-Nd1/mt-Co1/mt-Co3/mt-Co2/mt-Nd5/mt-Nd6
tags=67%, list=21%, signal=53%	Ctnnb1/Mecp2/Krit1/H2-Q7/Kras/mt-Nd4/Crebbp/mt-Atp6/mt-Cytb/Smad7/mt-Nd1/mt-Co3/mt-Nd2/mt-Nd5/mt-Nd4l/mt-Nd6
tags=24%, list=4%, signal=23%	Hspa1a/Jun/Hspa8/Hspa1b/Hsp90ab1/Gapdh/Gpx1/Hspd1
tags=28%, list=9%, signal=27%	Jun/Atf3/Phlda1/Fos/Ubb/Dnaja1/Rhob/Id3/Nr4a1/Pmaip1/Myc/Hspd1/Egr1/Gadd45b/Dedd2/Sirt1/Sap18
tags=36%, list=15%, signal=32%	Ankrd11/Foxp1/Kras/Zeb2/Atad1/Sptbn1/Ubtf/mt-Nd4/Crebbp/Pgap1/mt-Nd1/mt-Co1/mt-Co3/mt-Co2/Satb1/mt-Nd5/Trappc9/Nsf/mt-Nd6
tags=54%, list=8%, signal=50%	mt-Nd4/mt-Nd3/mt-Atp6/mt-Nd1/mt-Nd2/mt-Nd5/mt-Nd6
tags=56%, list=13%, signal=49%	Kras/mt-Nd4/Smchd1/mt-Nd1/mt-Co1/mt-Co3/mt-Co2/mt-Nd5/mt-Nd6
tags=47%, list=21%, signal=39%	Hivep2/Mecp2/Usp7/Med13/Malt1/Foxp1/Kras/Zeb2/Sptbn1/Tcf4/mt-Nd4/Crebbp/Setd2/Aff4/Birc3/mt-Nd1/mt-Co1/mt-Co3/mt-Co2/Satb1/mt-Nd5/mt-Nd6
tags=32%, list=4%, signal=31%	Hspa1a/Hspa1b/Hsp90aa1/Hsp90ab1/Ifi30/Gapdh/Ptges3/Hspd1
tags=75%, list=2%, signal=74%	mt-Co1/mt-Co3/mt-Co2
tags=80%, list=6%, signal=75%	Dnajb1/Hspa1a/Jun/Cited2
tags=15%, list=4%, signal=16%	Dnajb1/Hspa1a/Hspb1/Ppp1r15a/Klf4/Dnaja1/Dusp1/Pmaip1/Chchd2/Myc/Gpx1/Traf4
tags=82%, list=23%, signal=64%	Rps17/Rpl31/Rps26/Rpl18/Rps24/Rps20/Rps7/Rpl35/Rps27/Rpl15/Rps10/Rpl35a/Rps19/Rps28
tags=56%, list=21%, signal=45%	Hivep2/Mecp2/Cux1/Tcf4/mt-Nd4/mt-Nd3/Kcnq5/Setd2/mt-Atp6/mt-Nd1/mt-Nd2/mt-Nd5/Trappc9/mt-Nd6
tags=100%, list=12%, signal=89%	Ano6/Mef2c/Atp2b1
tags=29%, list=11%, signal=27%	Hspa1a/Hspb1/Jun/Fos/Klf4/Hsp90ab1/Nr4a1/Myc/Zfp36/Cited2/Egr1/Sirt1/Ube2d1/Rap1a/Ier2

tags=24%, list=4%, signal=24%	Hspa1a/Hspa8/Hspa1b/Atf3/Ppp1r15a/Hspd1
tags=15%, list=4%, signal=15%	Hspa1a/Hspb1/Hspa1b/Atf3/Ppp1r15a/Ubb/Dnaja1/Pmaip1/Myc/Gpx1
tags=63%, list=28%, signal=47%	Stat1/Stat4/Rnf213/Sh2b3/Ctnnb1/Rnf125/H2-Q7/Son/Tcirg1/Foxp1/Kras/mt-Nd4/Aff4/mt-Nd1/mt-Co1/mt-Co3/mt-Co2/mt-Nd5/mt-Nd6
tags=37%, list=19%, signal=32%	Ndufv1/Slc25a51/Atp2c1/Tcirg1/Lrrc8c/Atad1/mt-Nd4/Ano6/mt-Nd3/Kcnq5/mt-Atp6/mt-Cytb/Atp2b1/mt-Nd1/mt-Co1/mt-Atp8/mt-Co3/mt-Nd2/mt-Co2/mt-Nd5/mt-Nd4l/mt-Nd6
tags=61%, list=41%, signal=39%	Dpm1/Pten/Pex7/Dnm2/Prpf8/Apoe/Flii/Bptf/Slc12a6/Xylt1/Chd7/Ube3b/Jmjd1c/Cltc/Polg/Rere/Tmem127/Stat4/Ep300/Ivns1abp/Celf2/Tpp1/Ctnnb1/Krit1/Ndufv1/Cep83/H2-Q7/Med13/Kras/Zeb2/Sptbn1/Kmt2e/Ddx6/mt-
tags=39%, list=14%, signal=35%	Foxp1/Kras/Ppp1r15b/Tcf4/mt-Nd4/mt-Nd3/mt-Atp6/mt-Cytb/mt-Nd1/mt-Co1/mt-Co3/mt-Nd2/Irs2/mt-Co2/mt-Nd5/mt-Nd6
tags=53%, list=38%, signal=37%	Copb2/Smarce1/Flii/Bptf/Slc12a6/Ube3b/Nacc1/Cnot1/Cltc/Ppp2ca/Kmt2c/Tbce/Nrros/Polg/Ranbp2/Rere/Ube4b/Exosc1/Zmynd11/Ep300/Minpp1/Celf2/Ctnnb1/Hivep2/Sars/Polr2a/Ndufv1/Usp7/Cux1/Med13/Ankrd11/So
tags=30%, list=10%, signal=28%	Arnt/mt-Nd4/mt-Nd3/mt-Atp6/mt-Cytb/mt-Nd1/mt-Co1/mt-Atp8/mt-Co3/mt-Nd2/Irs2/mt-Co2/mt-Nd5/mt-Nd4l/mt-Nd6
tags=44%, list=20%, signal=36%	Mecp2/Krit1/Fus/Bms1/Tcirg1/Fbxo38/Kras/Rfc1/Matr3/Sptbn1/Ubtf/Cfl2/mt-Nd4/mt-Nd3/mt-Atp6/Smchd1/mt-Nd1/mt-Co1/mt-Co3/mt-Nd2/mt-Co2/mt-Nd5/mt-Nd6/Wnk1
tags=54%, list=8%, signal=50%	mt-Nd4/mt-Nd1/mt-Co1/mt-Co3/mt-Co2/mt-Nd5/mt-Nd6
tags=62%, list=6%, signal=59%	Jun/Fos/Klf4/Myc/Egr1
tags=58%, list=27%, signal=43%	Zmynd11/Wipi2/Celf2/Hivep2/Sars/Cux1/Kras/Tcf4/mt-Nd4/mt-Nd3/Kcnq5/mt-Atp6/Pgap1/mt-Nd1/mt-Nd2/mt-Nd5/Trappc9/mt-Nd6
tags=24%, list=4%, signal=24%	Hspa1a/Hspa8/Hspa1b/Hsp90aa1/Hsp90ab1/Ifi30/Gapdh/Ptges3/Hspd1
tags=75%, list=1%, signal=75%	Dnajb1/Hspa1a/Hspa1b
tags=75%, list=1%, signal=75%	Dnajb1/Hspa1a/Hspa1b
tags=75%, list=1%, signal=75%	Dnajb1/Hspa1a/Hspa1b
tags=36%, list=14%, signal=32%	Foxp1/Kras/Ppp1r15b/Tcf4/mt-Nd4/Herc1/mt-Nd3/Setd2/mt-Atp6/Aff4/mt-Cytb/mt-Nd1/mt-Co1/mt-Co3/mt-Nd2/Irs2/mt-Co2/mt-Nd5/mt-Nd6
tags=65%, list=23%, signal=51%	Rnf213/Sh2b3/Mecp2/Son/Kras/mt-Nd4/mt-Cytb/mt-Nd1/mt-Co1/mt-Co3/mt-Co2/mt-Nd5/mt-Nd6
tags=21%, list=6%, signal=21%	Hspa1a/Hspe1/Hspa8/Hspa1b/Rasd1/Hsp90aa1/Hsp90ab1/Tubb5/Rhob/Tuba1a/Hspd1/Rrad/Rsf1/Hsph1
tags=42%, list=8%, signal=39%	mt-Nd4/Ganab/mt-Nd1/mt-Co1/mt-Co3/mt-Co2/mt-Nd5/mt-Nd6
tags=46%, list=26%, signal=36%	Setx/Daxx/Hps3/Pml/Rnf213/Sh2b3/Ctnnb1/Krit1/Atp2c1/H2-Q7/Nfkb1/Nfkb2/Tcirg1/Kras/Ppp1r15b/Tcf4/mt-Nd4/Syk/mt-Atp6/mt-Cytb/mt-Nd1/mt-Co1/mt-Co3/mt-Nd2/mt-Co2/mt-Nd5/mt-Nd4l/mt-Nd6/Adgre5
tags=38%, list=3%, signal=38%	Hspb1/Hspa8/Ubb/Tubb5/Tuba1a
tags=100%, list=4%, signal=97%	Hsp90aa1/Hsp90ab1/Ptges3
tags=45%, list=12%, signal=41%	Matr3/mt-Nd4/mt-Atp6/mt-Cytb/mt-Nd1/mt-Co1/mt-Co3/mt-Co2/mt-Nd5/mt-Nd6
tags=50%, list=21%, signal=41%	Hivep2/Mecp2/Sars/Fus/Cux1/Ubtf/Tcf4/mt-Nd4/mt-Nd3/Kcnq5/mt-Atp6/Pgap1/mt-Nd1/mt-Nd2/mt-Nd5/Trappc9/mt-Nd6
tags=46%, list=20%, signal=38%	Mecp2/Sars/H2-Q7/Fus/Kras/Matr3/Sptbn1/Tcf4/mt-Nd4/Pgap1/mt-Nd1/mt-Co1/mt-Co3/mt-Co2/mt-Nd5/Trappc9/mt-Nd6
tags=53%, list=10%, signal=49%	Tcf4/mt-Nd4/mt-Nd1/mt-Co1/mt-Co3/mt-Co2/mt-Nd5/mt-Nd6
tags=39%, list=8%, signal=37%	mt-Nd4/Crebbp/mt-Nd3/Stt3b/mt-Atp6/mt-Nd1/mt-Nd2/mt-Nd5/mt-Nd6
tags=56%, list=8%, signal=52%	mt-Nd4/mt-Nd1/mt-Nd2/mt-Nd5/mt-Nd6
tags=44%, list=8%, signal=41%	mt-Nd4/mt-Nd1/mt-Co1/mt-Co3/mt-Co2/mt-Nd5/mt-Nd6
tags=82%, list=8%, signal=77%	Pten/Pex7/Smarce1/Slc12a6/Rere/Ube4b/Stat4/H2-Q7/Kras/mt-Nd4/mt-Cytb/mt-Nd1/mt-Co1/mt-Atp8/mt-Co3/mt-Co2/mt-Nd5/mt-Nd6
tags=42%, list=21%, signal=35%	Ctnnb1/Hivep2/Sars/Polr2a/Usp7/Cux1/Ankrd11/Son/Zeb2/Diaph1/Ubtf/Ppp1r15b/Tcf4/Ddx6/mt-Nd4/Mef2c/Kcnq5/Ralgapa1/Pgap1/mt-Nd1/mt-Co1/mt-Co3/mt-Co2/mt-Nd5/Trappc9/Macf1/mt-Nd6
tags=67%, list=4%, signal=64%	mt-Cytb/mt-Co1/mt-Co3/mt-Co2
tags=33%, list=5%, signal=32%	Hspa1a/Hspa8/Hspa1b/Hsp90aa1/Hsp90ab1/Fth1
tags=40%, list=19%, signal=34%	Hspa1a/Hspb1/Hspa8/Hspa1b/Rhob/Csrp1/Actg1/Rplp2/Rps17/Rps5/Rps8/Rpl31/Rpl18/Map2k1/Rps4x/Rpl19/Rala/Rpl8/Rps7/Bsg/Rpl38/Rpl30/Bcar3/Rpl7a/Rps16/Rps2/Rps13/Rpl13a
tags=57%, list=2%, signal=56%	Hspa1a/Hsp90aa1/Hsp90ab1/Gapdh
tags=65%, list=38%, signal=42%	Apoe/Smarce1/Flii/Chd7/Jmjd1c/Ubr1/Jak1/Polg/Rere/Phf21a/Ube4b/Stat1/Eif2ak3/Minpp1/Ctnnb1/Malt1/Foxp1/Kras/Ppp1r15b/Tcf4/mt-Nd4/Setd2/Aff4/Birc3/mt-Nd1/mt-Co1/mt-Co3/mt-Co2/mt-Nd5/Trappc9/mt-Nd6
tags=63%, list=30%, signal=46%	Rere/Arcn1/Ep300/Ctnnb1/Rnf125/Mecp2/Ndufv1/Usp7/Tcirg1/Tcf4/mt-Nd4/Crebbp/mt-Nd3/mt-Atp6/Aff4/mt-Nd1/mt-Nd2/mt-Nd5/mt-Nd6
tags=22%, list=4%, signal=22%	Hspa1a/Hspa8/Hspa1b/Atf3/Ppp1r15a/Hspd1
tags=100%, list=2%, signal=98%	Nsf
tags=100%, list=2%, signal=98%	Nsf
tags=40%, list=23%, signal=33%	Il4ra/Tpp1/Mecp2/Ndufv1/Ifngr1/Fus/Nfkb1/Nfkb2/Son/Kras/Zeb2/Tcf4/Sh3kbp1/mt-Nd4/Ikbkb/Syk/mt-Nd3/Bach2/mt-Atp6/mt-Cytb/mt-Nd1/mt-Co1/mt-Co3/mt-Nd2/mt-Co2/mt-Nd5/mt-Nd4l/mt-Nd6
tags=46%, list=31%, signal=35%	Nrros/Polg/Rere/Ube4b/Exosc1/Zmynd11/Wipi2/Setx/Celf2/Tpp1/Rnf125/Hivep2/Mecp2/Sars/Ndufv1/Fus/Cux1/Med13/Son/Kras/Atad1/Matr3/Sptbn1/Ubtf/Tcf4/Kmt2e/Ddx6/mt-Nd4/Herc1/Mef2c/Kcnq5/Setd2/mt-Atp6/Ra
tags=13%, list=5%, signal=14%	Dnajb1/Hspa1a/Hspb1/Ppp1r15a/Klf4/Dnaja1/Hsp90aa1/Hsp90ab1/Nfkbia/Dusp1/Pmaip1/Chchd2/Myc/Gpx1/Traf4/Zfp36/Actg1
tags=33%, list=1%, signal=33%	Hspa1a/Hspa8/Hspa1b
tags=100%, list=19%, signal=81%	Bcl6/Spag9/Tcf12/Tcf4/Mef2c
tags=27%, list=11%, signal=26%	Cd84/Diaph1/mt-Nd4/Ano6/Ahnak/Syk/Mef2c/Kcnq5/mt-Atp6/mt-Cytb/Cab39/Atp2b1/mt-Co1/mt-Atp8/mt-Co3/mt-Co2/mt-Nd5/Nsf/Pde4b/Wnk1
tags=41%, list=18%, signal=35%	Ifngr1/H2-Q7/Usp7/Son/Tcirg1/Kras/mt-Nd4/Ikbkb/Syk/Bach2/Il10ra/mt-Nd1/mt-Co1/mt-Co3/mt-Co2/mt-Nd5/mt-Nd6
tags=67%, list=4%, signal=64%	Hspa8/Hsp90aa1/Hsp90ab1/Ms4a1
tags=67%, list=4%, signal=64%	Hspa8/Hsp90aa1/Hsp90ab1/Ms4a1
tags=68%, list=38%, signal=44%	Apoe/Smarce1/Flii/Chd7/Jmjd1c/Ubr1/Jak1/Polg/Rere/Phf21a/Ube4b/Stat1/Eif2ak3/Ctnnb1/Foxp1/Ppp1r15b/mt-Nd4/Setd2/Aff4/mt-Nd1/mt-Co1/mt-Co3/mt-Co2/mt-Nd5/Trappc9/mt-Nd6
tags=44%, list=8%, signal=41%	mt-Nd4/mt-Nd1/mt-Co1/mt-Co3/mt-Co2/mt-Nd5/mt-Nd6
tags=58%, list=38%, signal=39%	Apoe/Flii/Chd7/Jmjd1c/Rad21/Cltc/Kmt2c/Kat6a/Polg/Rere/Ube4b/Zmynd11/Setx/Ep300/Ivns1abp/Celf2/Rnf125/Hivep2/Mecp2/Polr2a/H2-Q7/Fus/Usp7/Cux1/Ankrd11/Son/Zeb2/Atad1/Matr3/Ubtf/Tcf4/mt-Nd4/Crebbp/mt
tags=67%, list=2%, signal=66%	Klf2/Klf4/Hsp90aa1/Hsp90ab1
tags=18%, list=8%, signal=18%	Hspa1a/Jun/Hspa8/Hspa1b/Ppp1r15a/Sertad1/Klf4/Hsp90ab1/Rhob/Id3/Nr4a1/Dusp1/Myc/Junb/Cited2/Jund/Gadd45b/Senp6/Sirt1
tags=71%, list=25%, signal=54%	Ep300/Mecp2/Ndufv1/Sptbn1/Crebbp/Herc1/mt-Nd3/Mef2c/mt-Nd1/mt-Nd2
tags=16%, list=3%, signal=17%	Hspa1a/Hspb1/Hspa8/Hspa1b/Ubb/Tubb5/Gapdh/Tuba1a
tags=65%, list=36%, signal=43%	Nacc1/Cltc/Tbce/Polg/Ranbp2/Tufm/Stat4/Celf2/Tpp1/Ctnnb1/Mecp2/Ndufv1/H2-Q7/Son/Tcirg1/Kras/Sptbn1/Ubtf/mt-Nd4/mt-Nd3/mt-Atp6/mt-Nd1/mt-Nd2/Satb1/mt-Nd5/mt-Nd6
tags=74%, list=23%, signal=58%	Rps17/Rpl31/Rps26/Rpl18/Rps24/Rps20/Rps7/Rpl35/Rps27/Rpl15/Rps10/Rpl35a/Rps19/Rps28
tags=39%, list=12%, signal=35%	Matr3/Ubtf/mt-Nd4/Crebbp/mt-Atp6/mt-Cytb/mt-Nd1/mt-Co1/mt-Co3/mt-Co2/mt-Nd5/mt-Nd6
tags=53%, list=4%, signal=52%	Sptbn1/mt-Cytb/mt-Nd1/mt-Co1/mt-Co3/mt-Co2/mt-Nd5/mt-Nd6
tags=29%, list=8%, signal=28%	mt-Nd4/mt-Nd3/Kdm5a/mt-Cytb/mt-Nd1/mt-Co1/mt-Co3/mt-Nd2/mt-Co2/mt-Nd5/mt-Nd4l/Kdm6b/mt-Nd6
tags=100%, list=8%, signal=93%	Mef2c/Atp2b1
tags=100%, list=8%, signal=93%	Mef2c/Atp2b1
tags=50%, list=4%, signal=49%	Klf4/Hsp90aa1/Hsp90ab1/Myc/Ptges3
tags=21%, list=9%, signal=21%	Hspa1a/Hspe1/Hspa1b/Ppp1r15a/Ubb/Dnaja1/Hsp90aa1/Hsp90ab1/Nr4a1/Pmaip1/Chchd2/Tomm6/Myc/Mrps6/Gpx1/Mrpl52/Timm8b/Hspd1/Mrps12/P4ha1/Ubc/Ech1/Dynll1/Nol7/Chchd10/Sirt1/Uba52/Fkbp4/Map2k1
tags=27%, list=1%, signal=27%	Hspa1a/Hspa8/Hspa1b/Dnaja1
tags=100%, list=2%, signal=98%	Hsp90aa1/Hsp90ab1
tags=100%, list=2%, signal=98%	Hsp90aa1/Hsp90ab1
tags=100%, list=2%, signal=98%	Dnajb1/Hsp90ab1
tags=100%, list=2%, signal=98%	Hsp90aa1/Hsp90ab1
tags=100%, list=2%, signal=98%	Hsp90aa1/Hsp90ab1
tags=65%, list=39%, signal=41%	Taf15/Flii/Bptf/Chd7/Jmjd1c/Rad21/Phip/Polg/Chd8/Phf21a/Tmem127/Ep300/Rnf125/Hivep2/Mecp2/Fus/Med13/Foxp1/Kras/Matr3/Sptbn1/Kmt2e/Ddx6/mt-Nd4/Crebbp/Setd2/mt-Nd1/mt-Co1/mt-Co3/mt-Co2/Satb1/mt-N
tags=62%, list=22%, signal=50%	Polg/Tmem127/Stat4/Sh2b3/Ctnnb1/Csnk1d/Krit1/H2-Q7/Kras/mt-Nd4/mt-Atp6/mt-Cytb/mt-Nd1/mt-Co1/mt-Co3/mt-Co2/mt-Nd5/mt-Nd6
tags=16%, list=4%, signal=17%	Hspa1a/Hspe1/Hspa1b/Ubb/Klf4/Hsp90ab1/Nr4a1/Gapdh/Pmaip1/Myc/Gpx1/Hspd1
tags=21%, list=7%, signal=20%	Hspa1a/Hspa8/Hspa1b/Klf2/Fos/Hsp90ab1/Id3/Nfkbia/Pmaip1/Myc/Gpx1/Ptges3/Zfp36/Ly6d/Egr1/Jund
tags=56%, list=4%, signal=54%	Klf4/Hsp90aa1/Hsp90ab1/Myc/Ptges3
tags=44%, list=11%, signal=40%	Sptbn1/Tcf4/Sf3b1/Aff4/mt-Cytb/mt-Nd1/mt-Co1/mt-Co3/mt-Co2/mt-Nd5/mt-Nd6
tags=39%, list=8%, signal=37%	mt-Nd4/Crebbp/Setd2/mt-Nd1/mt-Co1/mt-Co3/mt-Co2/mt-Nd5/mt-Nd6
tags=100%, list=2%, signal=98%	Gpr171
tags=100%, list=2%, signal=98%	Gpr171
tags=18%, list=6%, signal=19%	Jun/Atf3/Klf2/Fos/Ubb/Klf4/Tubb5/Rhob/Id3/Nr4a1/Dusp1/Klf10/Myc/Gpx1/Csrp1/Zfp36/Actg1/Junb/Hnrnpab/Cited2/Egr1
tags=15%, list=7%, signal=16%	Hspa1a/Hspb1/Hspa8/Hspa1b/Atf3/Ppp1r15a/Klf4/Hsp90aa1/Id3/Gapdh/Myc/Dazap1/Hspd1/Zfp36/Actg1/Hnrnpab/Cited2/Egr1/Gpsm3
tags=52%, list=21%, signal=42%	Ctnnb1/Krit1/Tcirg1/Kras/mt-Nd4/mt-Nd1/mt-Co1/mt-Co3/mt-Co2/mt-Nd5/mt-Nd6
tags=50%, list=2%, signal=49%	Klf2/Klf4/Hsp90aa1/Hsp90ab1
tags=50%, list=2%, signal=49%	Klf2/Klf4/Hsp90aa1/Hsp90ab1
tags=22%, list=11%, signal=22%	Hspa1a/Jun/Hspa1b/Atf3/Marcksl1/Sertad1/Klf4/Ifi30/Nfkbia/Nr4a1/Dusp1/Klf10/Myc/Gpx1/Fth1/Zfp36/Junb/Cited2/Egr1/Jund/Sirt1/Dhps/Nap1l1/Map2k1/Ccdc117/Rps4x/Tspo/Tnfrsf13c
tags=14%, list=5%, signal=14%	Hspa1a/Hspb1/Jun/Hspa1b/Ppp1r15a/Klf4/Dnaja1/Hsp90ab1/Nr4a1/Gapdh/Dusp1/Gpx1/Azin1/Dbi/Zfp36
tags=28%, list=14%, signal=26%	Camk2d/Tcirg1/Lrrc8c/Cd84/Diaph1/mt-Nd4/Ano6/Ahnak/Syk/Mef2c/Kcnq5/mt-Atp6/mt-Cytb/Cab39/Atp2b1/mt-Co1/mt-Atp8/mt-Co3/mt-Co2/mt-Nd5/Nsf/Pde4b/Wnk1
tags=48%, list=31%, signal=35%	Polg/Ranbp2/Rere/Tufm/Zmynd11/Setx/Minpp1/Celf2/Ctnnb1/Hivep2/Mecp2/Sars/Ndufv1/Fus/Cux1/Zfyve27/Son/Foxp1/Kras/Zeb2/Atad1/Matr3/Sptbn1/Ubtf/Ppp1r15b/Tcf4/mt-Nd4/mt-Nd3/Kcnq5/mt-Atp6/Ralgapa1/Pga
tags=67%, list=41%, signal=42%	Pten/Dnm2/Prpf8/Apoe/Smarce1/Flii/Bptf/Xylt1/Chd7/Jmjd1c/Rad21/Kmt2c/Phip/Chd8/Rere/Phf21a/Ube4b/Ep300/Ctnnb1/Mecp2/Usp7/Foxp1/Sptbn1/Sh3kbp1/Ddx6/Crebbp/Herc1/Setd2/Aff4/Satb1/Trappc9
tags=38%, list=5%, signal=36%	mt-Atp6/mt-Cytb/mt-Co1/mt-Atp8/mt-Co3/mt-Co2
tags=56%, list=14%, signal=49%	Son/Kras/Sptbn1/Ppp1r15b/Tcf4/Kmt2e/Crebbp/Aff4/Kdm6b
tags=56%, list=37%, signal=37%	Smarce1/Flii/Bptf/Chd7/Jmjd1c/Rad21/Cltc/Phip/Polg/Chd8/Phf21a/Tmem127/Ep300/Celf2/Rnf125/Hivep2/Mecp2/Sars/Ndufv1/H2-Q7/Fus/Usp7/Med13/Foxp1/Kras/Matr3/Sptbn1/Ubtf/Tcf4/Kmt2e/Ddx6/mt-Nd4/Crebbp/S
tags=39%, list=12%, signal=35%	Rfc1/Ubtf/Ppp1r15b/Tcf4/mt-Nd4/Herc1/mt-Nd3/mt-Atp6/mt-Nd1/mt-Nd2/mt-Nd5/mt-Nd6
tags=33%, list=15%, signal=30%	Ankrd11/Foxp1/Fbxo38/Kras/Zeb2/Atad1/Matr3/Sptbn1/Ubtf/mt-Nd4/Crebbp/Pgap1/mt-Nd1/mt-Co1/mt-Co3/mt-Co2/Satb1/mt-Nd5/Trappc9/Nsf/mt-Nd6/Wnk1
tags=53%, list=8%, signal=50%	Tmem127/Stat4/Setx/Ivns1abp/Ctnnb1/Rnf125/Krit1/H2-Q7/mt-Nd4/mt-Atp6/Smchd1/mt-Cytb/mt-Nd1/mt-Co1/mt-Co3/mt-Nd2/mt-Nd5/mt-Nd4l/mt-Nd6
tags=47%, list=31%, signal=35%	Nrros/Polg/Rere/Ube4b/Exosc1/Zmynd11/Wipi2/Celf2/Tpp1/Rnf125/Hivep2/Mecp2/Sars/Cux1/Med13/Kras/Atad1/Ubtf/Tcf4/Kmt2e/Ddx6/mt-Nd4/Herc1/Mef2c/Kcnq5/Setd2/mt-Atp6/Ralgapa1/Pgap1/mt-Nd1/mt-Co1/mt-C
tags=75%, list=6%, signal=70%	Syk/Itga4/Wnk1
tags=39%, list=22%, signal=32%	Ccnd2/Rnf125/Hivep2/Mecp2/Sars/Fus/Cux1/Med13/Son/Kras/Zeb2/Ubtf/Tcf4/Kmt2e/Ddx6/mt-Nd4/Herc1/Mef2c/Kcnq5/mt-Atp6/Pgap1/mt-Nd1/mt-Co1/mt-Co3/mt-Co2/Satb1/mt-Nd5/Trappc9/Macf1/mt-Nd6
tags=73%, list=21%, signal=58%	Chd1/Usp7/Med13/Foxp1/Kmt2e/Ddx6/Satb1/Kdm6b
tags=33%, list=14%, signal=30%	Tcirg1/Rfc1/Sptbn1/Ppp1r15b/mt-Nd4/Setd2/mt-Atp6/mt-Cytb/Pgap1/mt-Nd1/mt-Co1/mt-Co3/mt-Nd2/Satb1/mt-Nd5/mt-Nd4l/mt-Nd6
tags=29%, list=4%, signal=28%	Hspa1a/Hspb1/Dnaja1/Gpx1
tags=75%, list=23%, signal=59%	Rps17/Rpl31/Rps26/Rpl18/Rps24/Rps20/Rps7/Rpl35/Rps27/Polr1d/Rpl15/Rps10/Rpl35a/Rps19/Rps28
tags=43%, list=21%, signal=36%	Hivep2/Mecp2/H2-Q7/Fus/Cux1/Ankrd11/Son/Zeb2/Matr3/Ubtf/Tcf4/mt-Nd4/mt-Nd3/Kcnq5/mt-Atp6/mt-Nd1/mt-Nd2/Satb1/mt-Nd5/Macf1/mt-Nd6
tags=50%, list=23%, signal=40%	Rnf213/Sh2b3/Mecp2/Krit1/Son/Kras/Sptbn1/mt-Nd4/Ganab/mt-Cytb/mt-Nd1/mt-Co1/mt-Co3/mt-Co2/mt-Nd5/mt-Nd6
tags=70%, list=23%, signal=54%	Actg1/Rps17/Rpl31/Rps26/Rpl18/Map2k1/Rps24/Rps20/Rps7/Rpl35/Rps27/Rpl15/Rps10/Rpl35a/Rps19/Rps28
tags=69%, list=26%, signal=53%	Hspb1/Rps17/Rpl31/Rps26/Rpl18/Chchd10/Rps24/Rps20/Rps7/Rpl35/Rps27/Rpl15/Rps10/Rpl35a/Rps19/Rps28/Bin1/Gars
tags=50%, list=8%, signal=46%	mt-Nd4/mt-Nd1/mt-Nd2/mt-Nd5/mt-Nd6
tags=100%, list=6%, signal=94%	Hsp90aa1/Hsp90ab1/Egr1
tags=100%, list=6%, signal=94%	Hsp90aa1/Hsp90ab1/Egr1
tags=19%, list=4%, signal=19%	Hspa1a/Hspb1/Hspa1b/Dnaja1/Gpx1
tags=38%, list=10%, signal=36%	Tcf4/mt-Nd4/Ganab/mt-Nd3/Stt3b/mt-Atp6/mt-Nd1/mt-Nd2/mt-Nd5/mt-Nd6
tags=31%, list=15%, signal=28%	Med13/Son/Kras/Zeb2/Sptbn1/Kmt2e/Ddx6/mt-Nd4/Ganab/Crebbp/Setd2/mt-Atp6/Aff4/mt-Cytb/mt-Nd1/mt-Co3/mt-Nd2/Satb1/mt-Nd5/mt-Nd4l/mt-Nd6
tags=10%, list=3%, signal=11%	Hspa1a/Jun/Hspa8/Hspa1b/Ppp1r15a/Ubb/Sertad1/Klf4/Hsp90ab1/Tubb5/Rhob/Id3/Nr4a1/Tuba1a/Dusp1/Myc
tags=28%, list=8%, signal=28%	Camk2d/Tcirg1/Lrrc8c/Diaph1/mt-Nd4/Ano6/Ahnak/Mef2c/Kcnq5/mt-Atp6/mt-Cytb/Cab39/Atp2b1/mt-Co1/mt-Atp8/mt-Co3/Irs2/mt-Co2/mt-Nd5/Pde4b/Wnk1
tags=100%, list=2%, signal=98%	Klf4/Nr4a1
tags=28%, list=6%, signal=27%	Hspa1a/Hspa8/Hspa1b/Hsp90aa1/Hsp90ab1/Fth1/Dynll1
tags=36%, list=11%, signal=33%	Sptbn1/Tcf4/mt-Nd4/Setd2/mt-Nd1/mt-Co1/mt-Co3/mt-Co2/mt-Nd5/mt-Nd6
tags=60%, list=15%, signal=52%	Klf4/Hsp90aa1/Hsp90ab1/Myc/Ptges3/Pold4/Sirt1/Nhp2/Pcna
tags=43%, list=1%, signal=43%	Hspb1/Hspa8/Ubb
tags=43%, list=1%, signal=43%	Hspb1/Hspa8/Ubb
tags=54%, list=37%, signal=37%	Bptf/Slc12a6/Chd7/Ube3b/Rad21/Rasa2/Ogt/Cltc/Phip/Polg/Rere/Phf21a/Tufm/Ube4b/Setx/Ep300/Minpp1/Hps3/Celf2/Tpp1/Ctnnb1/Ndufv1/H2-Q7/Fus/Usp7/Med13/Ankrd11/Son/Tcirg1/Foxp1/Kras/Rfc1/Kmt2e/Ddx6/mt-
tags=36%, list=4%, signal=35%	Hspa1a/Hspa1b/Hsp90ab1/Gpx1
tags=13%, list=5%, signal=14%	Hspa1a/Hspb1/Jun/Hspa8/Hspa1b/Fos/Hsp90aa1/Hsp90ab1/Tubb5/Nfkbia/Nr4a1/Gapdh/Pmaip1/Myc/Gpx1/Hspd1/Ms4a1/Traf4/Zfp36/Actg1
tags=85%, list=27%, signal=63%	Rps5/Rpsa/Rpl38/Rps27/Rpl24/Abt1/Rps19/Rps28/Npm1/C1qbp/Dhx30
tags=20%, list=6%, signal=20%	Hspb1/Jun/Klf2/Ubb/Klf4/Rhob/Nr4a1/Myc/Gpx1/Ptges3/Traf4/Actg1/Junb/Cited2
tags=100%, list=2%, signal=98%	Tubb5/Cacybp
tags=61%, list=25%, signal=46%	Ep300/Mecp2/Ndufv1/Sptbn1/Crebbp/Herc1/mt-Nd3/Mef2c/Setd2/mt-Nd1/mt-Nd2
tags=28%, list=7%, signal=27%	Jun/Atf3/Fos/Klf4/Klf6/Nr4a1/Klf10/Myc/Junb/Egr1/Jund
tags=64%, list=41%, signal=40%	Pten/Pex7/Apoe/Smarce1/Slc12a6/Chd7/Cltc/Tbce/Polg/Ranbp2/Rere/Ube4b/Stat4/Setx/Mecp2/Ndufv1/H2-Q7/Fus/Zfyve27/Kras/Ppp1r15b/mt-Nd4/mt-Atp6/mt-Cytb/Pgap1/mt-Nd1/mt-Co1/mt-Atp8/mt-Co3/mt-Co2/mt-N
tags=50%, list=8%, signal=47%	Ano6/Kcnq5/Cab39/Nsf/Wnk1
tags=100%, list=3%, signal=97%	Wnk1
tags=75%, list=14%, signal=65%	Ubb/Pmaip1/Myc/Rps20/Rps7/Rpl37
tags=70%, list=49%, signal=38%	Dcaf8/Lmnb1/Vps13a/Ppp3ca/Arid1b/Isca1/Psap/Sbf1/Dpm1/Pten/Pex7/Dnm2/Copb2/Slc12a6/Nacc1/Cltc/Ppp2ca/Tbce/Nrros/Polg/Rere/Tufm/Exosc1/Ep300/Celf2/Ctnnb1/Polr2a/Ndufv1/Fus/Foxp1/Rfc1/Sptbn1/Ubtf/Pp
tags=71%, list=23%, signal=56%	Jun/Klf2/Fos/Rhob/Sirt1/Pcna/Nfe2l2/Oser1/Prdx3/Rps3
tags=59%, list=37%, signal=39%	Smarce1/Flii/Bptf/Chd7/Stk11/Rad21/Rasa2/Kmt2c/Polg/Rere/Phf21a/Ube4b/Stat1/Ep300/Ctnnb1/Mecp2/Usp7/Med13/Kras/Sptbn1/Ppp1r15b/Kmt2e/Ddx6/mt-Nd4/Crebbp/Smchd1/mt-Nd1/mt-Co1/mt-Co3/mt-Co2/Satb
tags=75%, list=4%, signal=73%	Hspa8/Hsp90ab1/Ptges3
tags=21%, list=6%, signal=21%	Hspa1a/Jun/Hspa1b/Klf2/Fos/Nfkbia/Klf10/Myc/Zfp36/Junb/Cited2

tags=42%, list=14%, signal=37%	Son/Zeb2/Ubtf/mt-Nd4/mt-Nd3/mt-Atp6/mt-Nd1/mt-Nd2/mt-Nd5/mt-Nd6
tags=60%, list=37%, signal=39%	Flii/Chd7/Jmjd1c/Rad21/Cltc/Kmt2c/Kat6a/Polg/Rere/Ube4b/Zmynd11/Ep300/Celf2/Rnf125/Hivep2/Mecp2/Polr2a/Usp7/Cux1/Son/Atad1/Tcf4/Crebbp/Kcnq5/Setd2/mt-Atp6/Aff4/Satb1/Wnk1
tags=70%, list=20%, signal=56%	Mecp2/Ndufv1/Sptbn1/mt-Nd3/Mef2c/mt-Nd1/mt-Nd2
tags=67%, list=26%, signal=50%	Setx/Tpp1/Chd1/Mecp2/Fus/Usp7/Med13/Foxp1/Kmt2e/Ddx6/Satb1/Kdm6b
tags=24%, list=5%, signal=24%	Hspb1/Jun/Klf4/Id3/Myc/Gpx1/Csrp1/Zfp36/Actg1
tags=31%, list=15%, signal=28%	Med13/Foxp1/Kras/Fbxo11/Sptbn1/Kmt2e/Sh3kbp1/Ddx6/mt-Nd4/Crebbp/Setd2/Pgap1/mt-Nd1/mt-Co1/mt-Co3/mt-Co2/Satb1/mt-Nd5/Trappc9/mt-Nd6
tags=43%, list=14%, signal=38%	Camk2d/Tcirg1/Diaph1/Ano6/Ahnak/Mef2c/Kcnq5/Cab39/Pde4b/Wnk1
tags=58%, list=15%, signal=50%	Jun/Klf4/Myc/Gpx1/Map2k1/Tspo/Pcna
tags=60%, list=2%, signal=59%	mt-Co1/mt-Co3/mt-Co2
tags=39%, list=20%, signal=32%	Mecp2/Krit1/H2-Q7/Fus/Kras/Matr3/Sptbn1/Ubtf/mt-Nd4/Crebbp/mt-Atp6/mt-Cytb/mt-Nd1/mt-Co1/mt-Co3/mt-Co2/mt-Nd5/mt-Nd6
tags=38%, list=7%, signal=35%	Jun/Fos/Nfkbia/Actg1/Cited2/Jund
tags=37%, list=21%, signal=31%	Ctnnb1/Mecp2/Sars/Polr2a/Fus/Med13/Son/Foxp1/Fbxo11/Zeb2/Sptbn1/Tcf4/Kmt2e/Ddx6/Cfl2/mt-Nd4/Crebbp/Mef2c/Ralgapa1/Pgap1/mt-Nd1/mt-Co1/mt-Co3/mt-Co2/Satb1/mt-Nd5/Trappc9/mt-Nd6
tags=60%, list=2%, signal=59%	mt-Co1/mt-Co3/mt-Co2
tags=100%, list=3%, signal=97%	Fos/Klf10
tags=18%, list=5%, signal=18%	Hspa1a/Hspa8/Hspa1b/Marcksl1/Hsp90ab1/Rhob/Capg/Gpx1/Csrp1/Actg1/P4ha1
tags=100%, list=4%, signal=96%	mt-Co1
tags=15%, list=2%, signal=15%	Hspa1a/Hspa8/Hspa1b/Dnaja1/Rasd1/Hsp90aa1/Hsp90ab1/Gapdh
tags=80%, list=12%, signal=71%	S1pr1/Ano6/Mef2c/Atp2b1
tags=12%, list=3%, signal=12%	Hspa1a/Hspb1/Jun/Hspa1b/Ppp1r15a/Dnaja1/Dusp1
tags=29%, list=4%, signal=29%	Jun/Hspa8/Gapdh/Gpx1/Hspd1
tags=22%, list=6%, signal=21%	Hspa1a/Hspe1/Hspa8/Hspa1b/Hsp90aa1/Hsp90ab1/Hspd1/Rsf1/Hsph1
tags=67%, list=1%, signal=66%	Hspa1a/Hspa1b
tags=37%, list=14%, signal=32%	Tcirg1/Lrrc8c/Ano6/Kcnq5/mt-Atp6/mt-Cytb/Atp2b1/mt-Co1/mt-Atp8/mt-Co3/mt-Co2
tags=100%, list=3%, signal=97%	Klf2/Myc
tags=45%, list=23%, signal=36%	Rc3h2/Il4ra/Rnf125/Cr1l/H2-Q7/Nfkb2/Siglecg/Malt1/Bcl6/Tcirg1/Fbxo38/Cd86/Cd84/Inpp5d/Arid5a/Syk/Mef2c/Bach2/Pag1/Smad7
tags=43%, list=1%, signal=43%	Hspa1a/Hspa1b/Klf4
tags=50%, list=18%, signal=42%	H2-Q7/Kras/mt-Nd4/mt-Nd1/mt-Co1/mt-Co3/mt-Co2/mt-Nd5/mt-Nd6
tags=26%, list=13%, signal=25%	Zeb2/Rfc1/Atad1/Sptbn1/Ubtf/Ppp1r15b/Tcf4/mt-Nd4/Crebbp/Herc1/mt-Nd3/Setd2/mt-Atp6/mt-Cytb/mt-Nd1/mt-Co1/mt-Atp8/mt-Co3/mt-Nd2/mt-Co2/Satb1/mt-Nd5/mt-Nd4l/mt-Nd6
tags=100%, list=9%, signal=91%	Hsp90aa1/Hsp90ab1/Fkbp4
tags=67%, list=22%, signal=53%	Bcl2/Csnk1d/Bcl6/Spag9/Tcf12/Tcf4/Ddx6/Mef2c
tags=46%, list=13%, signal=40%	Kras/Rfc1/Ubtf/mt-Nd4/mt-Atp6/mt-Nd1/mt-Co1/mt-Co3/mt-Co2/mt-Nd5/mt-Nd6
tags=100%, list=15%, signal=86%	Rps20/Rps7/Rpl37/Aup1
tags=71%, list=12%, signal=63%	Klf2/Myc/Hnrnpab/Lamtor2/Bcl11a
tags=57%, list=5%, signal=55%	Jun/Ifi30/Myc/Fth1
tags=31%, list=13%, signal=28%	Kras/Rfc1/Sptbn1/mt-Nd4/mt-Nd3/mt-Atp6/mt-Cytb/mt-Nd1/mt-Co1/mt-Co3/mt-Nd2/mt-Co2/mt-Nd5/mt-Nd4l/mt-Nd6/Wnk1
tags=71%, list=23%, signal=56%	Rps17/Rpl31/Rps26/Rpl18/Rps24/Rps20/Rps7/Rpl35/Rps27/Ahcy/Rpl15/Rps10/Rpl35a/Rps19/Rps28
tags=43%, list=3%, signal=42%	Hspa1a/Hspa1b/Capg
tags=40%, list=11%, signal=36%	Sptbn1/mt-Nd4/mt-Nd3/mt-Atp6/mt-Nd1/mt-Nd2/mt-Nd5/mt-Nd6
tags=33%, list=11%, signal=31%	Med13/Kras/Sptbn1/Tcf4/Kmt2e/Ddx6/mt-Nd4/Crebbp/Setd2/mt-Nd1/mt-Co1/mt-Co3/mt-Co2/Satb1/mt-Nd5/Kdm6b/mt-Nd6
tags=23%, list=8%, signal=22%	mt-Nd4/mt-Nd3/mt-Atp6/mt-Cytb/mt-Nd1/mt-Co1/mt-Atp8/mt-Co3/mt-Nd2/mt-Co2/mt-Nd5/mt-Nd4l/mt-Nd6
tags=59%, list=38%, signal=39%	Apoe/Smarce1/Flii/Bptf/Nacc1/Rad21/Cnot1/Cltc/Kat6a/Chd8/Rere/Arcn1/Zmynd11/Ep300/Ctnnb1/Hivep2/Mecp2/Sars/Polr2a/Csnk1d/Usp7/Cux1/Med13/Zeb2/Atad1/Sptbn1/Tcf4/Kmt2e/Ddx6/Crebbp/Kcnq5/Aff4/Pgap1/
tags=44%, list=8%, signal=41%	Hspa1a/Hspa1b/Tubb5/Senp6
tags=79%, list=30%, signal=56%	Rere/Arcn1/Ldb1/Ogdh/Bcl2/Ctnnb1/Mecp2/Sec24b/mt-Nd4/Herc1/mt-Co1
tags=100%, list=14%, signal=86%	Foxp1/Irs2
tags=100%, list=15%, signal=86%	Hsp90aa1/Hsp90ab1/Ptges3/Pcna
tags=100%, list=21%, signal=79%	Mecp2/Zeb2/Ppp1r15b/Nsf
tags=25%, list=4%, signal=25%	Hspa1a/Hspa1b/Atf3/Pmaip1/Gpx1
tags=70%, list=23%, signal=55%	Rps17/Rpl31/Rps26/Rpl18/Rps24/Rps20/Rps7/Rpl35/Rps27/Rpl15/Rps10/Rpl35a/Rps19/Rps28
tags=51%, list=36%, signal=35%	Chd7/Rad21/Rasa2/Cnot1/Ubr1/Hax1/Polg/Rere/Tmem127/Ube4b/Stat4/Ep300/Ildr1/Pml/Sh2b3/Ctnnb1/Mecp2/Ndufv1/H2-Q7/Fus/Kras/Zeb2/Rfc1/Sptbn1/Diaph1/Ppp1r15b/mt-Nd4/Crebbp/mt-Nd3/mt-Atp6/Aff4/Smchd
tags=100%, list=14%, signal=86%	Foxp1/Birc3
tags=15%, list=4%, signal=16%	Hspa1a/Hspa8/Hspa1b/Dnaja1/Hsp90aa1/Hsp90ab1/Tubb5/Id3/Gapdh/Cacybp/Capg/Gpx1/Traf4
tags=62%, list=21%, signal=49%	Zranb1/Csnk1d/Csnk1g3/Nfkb1/Zeb2/Pbxip1/Macf1/Wnk1
tags=75%, list=23%, signal=58%	Rps17/Rpl31/Rps26/Rpl18/Rps24/Rps20/Rps7/Rpl35/Rps27/Rpl15/Rps10/Rpl35a/Rps19/Rps28/Npm1
tags=18%, list=2%, signal=18%	Dnajb1/Ubb/Hsp90aa1/Hsp90ab1/Tubb5
tags=31%, list=8%, signal=29%	Ano6/Kcnq5/mt-Atp6/mt-Cytb/Atp2b1/mt-Co1/mt-Atp8/mt-Co3/mt-Co2
tags=12%, list=4%, signal=12%	Hspa1a/Hspb1/Jun/Hspa1b/Klf4/Dnaja1/Hsp90ab1/Nr4a1/Myc/Gpx1/Hspd1
tags=75%, list=6%, signal=70%	Syk/Bach2/Il10ra
tags=56%, list=6%, signal=52%	Jun/Fos/Klf4/Myc/Egr1
tags=100%, list=15%, signal=86%	Son/Mef2c
tags=38%, list=4%, signal=37%	mt-Cytb/Atp2b1/mt-Co1/mt-Co3/mt-Co2
tags=86%, list=22%, signal=67%	Tpp1/Ctnnb1/Tcirg1/S1pr1/Inpp5d/Syk
tags=56%, list=12%, signal=49%	Akap13/S1pr1/S1pr4/Pde4b/Adgre5
tags=56%, list=27%, signal=41%	Zmynd11/Ep300/Hivep2/Sars/Fus/Cux1/Son/Ubtf/Tcf4/Crebbp/Kcnq5/Aff4/Ralgapa1/Pgap1/Trappc9
tags=88%, list=23%, signal=68%	Rps5/Rpsa/Rpl38/Rps27/Abt1/Rps19/Rps28
tags=67%, list=1%, signal=66%	Jun/Fos
tags=38%, list=10%, signal=35%	Tcf4/mt-Nd4/mt-Nd3/mt-Atp6/mt-Nd1/mt-Nd2/mt-Nd5/mt-Nd6
tags=71%, list=37%, signal=45%	Flii/Bptf/Tbce/Kat6a/Rere/Ube4b/Ep300/Son/Fbxo11/Zeb2/Sptbn1/Tcf4/Kmt2e/Crebbp/Mef2c/Ralgapa1/Pgap1
tags=100%, list=0%, signal=100%	
tags=100%, list=0%, signal=100%	
tags=100%, list=0%, signal=100%	
tags=100%, list=0%, signal=100%	
tags=100%, list=0%, signal=100%	
tags=100%, list=0%, signal=100%	
tags=100%, list=0%, signal=100%	
tags=100%, list=0%, signal=100%	
tags=100%, list=0%, signal=100%	
tags=100%, list=0%, signal=100%	
tags=100%, list=0%, signal=100%	
tags=100%, list=0%, signal=100%	
tags=100%, list=0%, signal=100%	
tags=100%, list=0%, signal=100%	
tags=100%, list=0%, signal=100%	
tags=100%, list=0%, signal=100%	
tags=100%, list=0%, signal=100%	
tags=100%, list=0%, signal=100%	
tags=100%, list=0%, signal=100%	
tags=100%, list=0%, signal=100%	
tags=100%, list=0%, signal=100%	
tags=49%, list=32%, signal=35%	Kat6a/Polg/Ranbp2/Rere/Arcn1/Stat4/Ep300/Pml/Sh2b3/Ctnnb1/Rnf125/Mecp2/Ndufv1/H2-Q7/Fus/Usp7/Tcirg1/Matr3/Tcf4/Cfl2/mt-Nd4/Crebbp/mt-Nd3/Stt3b/mt-Atp6/Aff4/mt-Cytb/mt-Nd1/mt-Nd2/mt-Nd5/mt-Nd6
tags=60%, list=8%, signal=56%	Hspa1a/Hspa1b/Senp6
tags=100%, list=15%, signal=85%	Inpp5d/Syk
tags=11%, list=5%, signal=12%	Dnajb1/Hspa1a/Hspe1/Hspa1b/Ppp1r15a/Klf4/Hsp90aa1/Hsp90ab1/Pmaip1/Myc/Ptges3/Hspd1/Traf4/Dbi
tags=71%, list=19%, signal=58%	Ndufv1/Son/mt-Nd3/mt-Nd1/mt-Nd2
tags=30%, list=2%, signal=30%	Hspa1a/Hspa1b/Hsp90aa1
tags=30%, list=10%, signal=28%	Tcf4/Ganab/mt-Cytb/mt-Nd1/mt-Co1/mt-Co3/mt-Co2/mt-Nd5/mt-Nd6/Wnk1
tags=64%, list=49%, signal=36%	Lmnb1/Vps13a/Ppp3ca/Arl6ip1/Erlin1/Atrx/Isca1/Hnrnph2/Psap/Inpp5k/Kynu/Dpm1/Pex7/Taf15/Apoe/Copb2/Nacc1/Rad21/Cnot1/Cltc/Tbce/Kat6a/Polg/Ranbp2/Rere/Tufm/Zmynd11/Ogdh/Eif2ak3/Setx/Minpp1/Celf2/Ctnn
tags=12%, list=4%, signal=12%	Dnajb1/Hspa1a/Hspa8/Hspa1b/Dusp1/Capg/Gpx1
tags=25%, list=2%, signal=25%	Hspa1a/Hspa1b/Klf4/Hsp90ab1
tags=30%, list=8%, signal=28%	Jun/Fos/Ubb/Hsp90ab1/Gapdh/Gpx1/Egr1/Sirt1
tags=67%, list=22%, signal=53%	Bcl2/Pcid2/Bcl6/Sh3kbp1/Inpp5d/Syk/Mef2c/Irs2
tags=33%, list=2%, signal=33%	Hspa1a/Hspa8/Rhob
tags=56%, list=21%, signal=45%	Hivep2/Fus/Cux1/Son/Ubtf/Tcf4/Kcnq5/Aff4/Ralgapa1/Pgap1
tags=45%, list=14%, signal=39%	Camk2d/Tcirg1/Diaph1/Ano6/Ahnak/Mef2c/Cab39/Pde4b/Wnk1
tags=25%, list=4%, signal=25%	mt-Cytb/mt-Nd1/mt-Co1/mt-Co3/mt-Co2/mt-Nd5/mt-Nd6
tags=20%, list=6%, signal=20%	Hspb1/Jun/Klf2/Klf4/Rhob/Nr4a1/Gpx1/Actg1/Junb/Cited2
tags=22%, list=4%, signal=22%	Hspb1/Jun/Klf4/Rhob/Nr4a1/Gpx1
tags=23%, list=2%, signal=23%	Hspb1/Jun/Rhob
tags=83%, list=20%, signal=67%	Mecp2/Polr2a/Son/Pgap1/Satb1
tags=70%, list=26%, signal=53%	Rps17/Rpl31/Rps26/Rpl18/Rpsa/Rps24/Rps20/Rps7/Rpl35/Rps27/Rpl15/Rps10/Rpl35a/Rps19/Rps28/H2-Ab1
tags=75%, list=8%, signal=69%	Ano6/P2ry10/Gpr171
tags=75%, list=12%, signal=67%	Bcl2/S1pr1/Ano6/Mef2c/Atp2b1/Smad7
tags=43%, list=8%, signal=40%	Hspa1a/Hspa1b/Senp6
tags=35%, list=14%, signal=31%	Camk2d/Tcirg1/Cd84/Diaph1/Ano6/Ahnak/Syk/Mef2c/Kcnq5/Cab39/Atp2b1/Pde4b/Wnk1
tags=16%, list=6%, signal=17%	Hspa1a/Jun/Hspa1b/Klf2/Fos/Klf4/Klf6/Nfkbia/Nfkbid/Klf10/Myc/Hspd1/Ms4a1/Zfp36/Ly6d/Junb/Cited2/Egr1
tags=75%, list=26%, signal=57%	Rps17/Rpl31/Rps26/Rpl18/Rps24/Rps20/Rps7/Rpl35/Rps27/Rpl15/Rps10/Rpl35a/Rps19/Rps28/Flcn
tags=100%, list=24%, signal=77%	Hspb1/Gpx1/Sirt1/Nfe2l2/Parp1
tags=67%, list=1%, signal=66%	Hspa1a/Hspa1b
tags=67%, list=1%, signal=66%	Hspa1a/Hspa1b
tags=33%, list=14%, signal=30%	Tcirg1/Lrrc8c/Ano6/Kcnq5/mt-Atp6/mt-Cytb/Atp2b1/mt-Co1/mt-Atp8/mt-Co3/mt-Co2
tags=100%, list=16%, signal=84%	Sptbn1/Atp2b1
tags=71%, list=41%, signal=43%	Pten/Apoe/Stk11/Cnot1/Ubr1/Rere/Ube4b/Stat4/Eif2ak3/H2-Q7/Kras/Tcf4/mt-Nd4/Ganab/mt-Nd1/mt-Co1/mt-Co3/mt-Co2/mt-Nd5/mt-Nd6
tags=55%, list=22%, signal=43%	Shisa5/Ctnnb1/Atp2c1/Birc2/Mul1/Malt1/Dhx36/Akap13/Rel/Ikbkb/Birc3/Trim25
tags=95%, list=51%, signal=47%	Rpsa/Eif3k/Pes1/Eif1/Npm1/C1qbp/Secisbp2/Tmco1/Serbp1/Derl2/Nemf/Ythdf3/Zc3h12a/Naa15/Naa10/Itch/Ccdc47/Srpr
tags=34%, list=13%, signal=31%	Kras/Zeb2/mt-Nd4/Crebbp/mt-Cytb/Pgap1/mt-Nd1/mt-Co1/mt-Co3/mt-Co2/mt-Nd5/mt-Nd6
tags=45%, list=7%, signal=43%	Jun/Fos/Actg1/Junb/Jund
tags=80%, list=14%, signal=69%	Malt1/Foxp1/Syk/Birc3
tags=42%, list=20%, signal=35%	Dnajb1/Hspa1a/Hspa8/Hsp90ab1/Capg/Fxyd5/Rps26/Ahsa1/Vapa/Bsg/Psmb6/Nudc/Rpl7a/Eno1/Pcbp1/Rps2/Rpl24/Rpl34/Eif3e/Rpl15/Rpl14
tags=21%, list=3%, signal=21%	Hspa1a/Hspa1b/Hsp90aa1/Capg
tags=100%, list=7%, signal=93%	Syk
tags=60%, list=15%, signal=51%	Med13/Kmt2e/Ddx6/mt-Atp6/Pgap1/Satb1
tags=100%, list=16%, signal=84%	Bcl6/Mef2c
tags=16%, list=5%, signal=16%	Hspa1a/Hspa8/Hspa1b/Fos/Klf4/Id3/Nfkbia/Nr4a1/Myc/Gpx1/Ptges3/Zfp36
tags=43%, list=2%, signal=42%	Hspa1a/Hspa1b/Nfkbia
tags=100%, list=7%, signal=93%	Sf3b1
tags=38%, list=10%, signal=34%	Hspa8/Hsp90aa1/Ifi30/Tubb5/Ftl1/Man2b1
tags=45%, list=12%, signal=41%	S1pr1/S1pr4/P2ry10/Gpr171/Adgre5
tags=14%, list=3%, signal=14%	Hspb1/Jun/Fos/Klf4/Dnaja1/Id3/Nfkbia/Nr4a1/Chchd2/Myc
tags=46%, list=21%, signal=37%	Rnf125/Ndufv1/Nfkb2/Ppp1r15b/Herc1/mt-Nd3/Setd2/Aff4/mt-Cytb/mt-Nd1/mt-Nd2
tags=19%, list=6%, signal=18%	Hspa1a/Hspa8/Hspa1b/Klf2/Hsp90ab1/Id3/Myc/Ptges3/Zfp36/Egr1

tags=29%, list=1%, signal=29%	Hspa1a/Hspa1b
tags=29%, list=1%, signal=29%	Hspa1a/Hspa1b
tags=16%, list=7%, signal=16%	Dnajb1/Hspa1a/Jun/Atf3/Klf2/Klf4/Id3/Klf10/Myc/Zfp36/Cited2/Egr1/Foxp4/Jund
tags=40%, list=8%, signal=37%	Hspb1/Klf4/Rhob/Sirt1
tags=56%, list=8%, signal=51%	Tubb5/Cacybp/Chchd2/Timm8b/Chchd10
tags=36%, list=3%, signal=36%	Hspa1a/Hspa1b/Fos/Klf10
tags=100%, list=18%, signal=83%	Fkbp4/Nfatc1/Fkbp3/Fkbp1a
tags=100%, list=18%, signal=83%	Fkbp4/Nfatc1/Fkbp3/Fkbp1a
tags=17%, list=6%, signal=17%	Hspa1a/Hspe1/Hspa8/Hspa1b/Hsp90aa1/Hsp90ab1/Hspd1/Rsf1/Hsph1
tags=100%, list=0%, signal=100%	
tags=100%, list=18%, signal=83%	Ubb/Ubc/Uba52/Rps27a
tags=17%, list=2%, signal=17%	Hspa1a/Jun/Fos/Hsp90ab1
tags=40%, list=28%, signal=34%	Stat1/Stat4/Zmynd11/Orc3/Ldb1/Uvrag/Ist1/Setx/Ep300/Daxx/Pml/Ccnd2/Rbm34/Ctnnb1/Ppp2r5a/Brd9/Arpc5/Chd1/Mecp2/Sp100/Polr2a/Birc2/Nfkb1/Usp7/Nifk/Bms1/Cux1/Cebpg/Nasp/Nfkb2/Ncoa2/Neil1/Klf3/Epc1/B
tags=77%, list=30%, signal=55%	Rere/Arcn1/Ldb1/Ogdh/Bcl2/Mecp2/Sec24b/mt-Nd4/Herc1/mt-Co1
tags=100%, list=13%, signal=87%	Ubb/Myc/Rps7
tags=60%, list=15%, signal=51%	Med13/Kmt2e/Ddx6/Ganab/Aff4/Satb1
tags=52%, list=23%, signal=41%	Il4ra/Cr1l/H2-Q7/Malt1/Bcl6/Fbxo38/Dhx36/Cd84/Arid5a/Syk/Smad7/Adgre5
tags=100%, list=25%, signal=75%	Nfkb2/Kras/Zeb2/Crebbp
tags=100%, list=13%, signal=87%	Junb/Cited2/Bsg
tags=33%, list=15%, signal=29%	Med13/Ankrd11/Sptbn1/Ppp1r15b/mt-Nd4/Aff4/Smchd1/mt-Nd1/mt-Co1/mt-Co3/mt-Co2/mt-Nd5/Trappc9/mt-Nd6
tags=62%, list=6%, signal=59%	Atf3/Fos/Nr4a1/Cited2/Egr1
tags=75%, list=11%, signal=67%	Sptbn1/Sh3kbp1/Syk
tags=75%, list=8%, signal=69%	Hspb1/Gpx1/Sirt1
tags=100%, list=8%, signal=92%	Atp2b1
tags=100%, list=8%, signal=92%	Atp2b1
tags=31%, list=6%, signal=30%	Jun/Klf4/Nfkbia/Myc/Rsf1
tags=30%, list=18%, signal=27%	H2-Q7/Fus/Usp7/Zfyve27/Med13/Fbxo38/Kras/Zeb2/Rfc1/Atad1/Matr3/Sptbn1/Ubtf/Ppp1r15b/Tcf4/Kmt2e/Ddx6/Cfl2/mt-Nd4/Crebbp/Herc1/mt-Nd3/Mef2c/Kcnq5/mt-Atp6/Pgap1/mt-Nd1/mt-Co1/mt-Co3/mt-Nd2/mt-Co2/
tags=33%, list=1%, signal=33%	Hspa1a/Hspa1b
tags=75%, list=1%, signal=75%	Hspa1a/Hspa8/Hspa1b
tags=60%, list=2%, signal=59%	Jun/Fos/Nfkbia
tags=47%, list=14%, signal=41%	Hspa1a/Hspa1b/Dnaja1/Tspo/Rps20/Rps7/Rpl37
tags=18%, list=6%, signal=18%	Hspb1/Jun/Klf2/Klf4/Rhob/Nr4a1/Myc/Gpx1/Actg1/Junb/Cited2
tags=43%, list=6%, signal=40%	Syk/Pde4b/Adgre5
tags=54%, list=15%, signal=46%	Med13/Tcirg1/Kras/Zeb2/Kmt2e/Ddx6/Satb1
tags=66%, list=37%, signal=42%	Bptf/Rasa2/Cltc/Tbce/Phip/Exosc1/Celf2/Ccnd2/Polr2a/Med13/Ankrd11/Kras/Fbxo11/Sptbn1/Kmt2e/Ddx6/Mef2c/Setd2/Satb1
tags=100%, list=18%, signal=83%	Kras/Syk
tags=14%, list=4%, signal=14%	Hspa1a/Hspe1/Hspa1b/Pmaip1/Myc/Hspd1
tags=28%, list=5%, signal=27%	Hspa1a/Hspa1b/Fos/Nfkbia/Klf10/Myc/Zfp36
tags=100%, list=6%, signal=94%	Junb/Cited2
tags=27%, list=2%, signal=27%	Hspa1a/Hspa1b/Hsp90aa1/Hsp90ab1
tags=64%, list=26%, signal=48%	Actg1/Rps17/Rpl31/Rps26/Rpl18/Rps24/Rps20/Rps7/Rpl35/Rps27/Rpl15/Rps10/Rpl35a/Rps19/Rps28/Tmco1
tags=100%, list=0%, signal=100%	Hspa1a
tags=38%, list=8%, signal=36%	mt-Nd4/mt-Atp6/mt-Nd1/mt-Co1/mt-Co3/mt-Co2/mt-Nd5/mt-Nd6
tags=100%, list=6%, signal=94%	Myc/Egr1
tags=40%, list=8%, signal=37%	Hspa1a/Hsp90aa1/Pmaip1/Myc/Timm8b/Chchd10
tags=41%, list=14%, signal=36%	Rasd1/Hsp90ab1/Tubb5/Rhob/Tuba1a/Rrad/Fkbp4/Rap1a/Rala/Eif2b2/Tuba1b
tags=57%, list=11%, signal=51%	Jun/Klf4/Map2k1/Tspo
tags=16%, list=9%, signal=16%	Dnajb1/Hspa1a/Jun/Hspa8/Atf3/Klf2/Klf4/Rasd1/Id3/Klf10/Myc/Zfp36/Rsf1/Hnrnpab/Cited2/Egr1/Foxp4/Jund/Rps26/Dedd2/Sirt1/Sap18/Ube2d1
tags=36%, list=8%, signal=33%	Hspb1/Klf4/Nr4a1/Gpx1/Sirt1
tags=40%, list=1%, signal=40%	Dnajb1/Dnaja1
tags=16%, list=5%, signal=16%	Hspa1a/Jun/Hspa1b/Klf4/Ifi30/Dusp1/Klf10/Myc/Fth1
tags=100%, list=18%, signal=82%	Nfkb2/Tcirg1
tags=46%, list=23%, signal=36%	Il4ra/Cr1l/H2-Q7/Cebpg/Malt1/Bcl6/Tcirg1/Fbxo38/Dhx36/Cd84/Kmt2e/Inpp5d/Arid5a/Syk/Smad7/Adgre5
tags=23%, list=1%, signal=23%	Hspb1/Hspa8/Ubb
tags=57%, list=13%, signal=50%	Lrrc8c/Ano6/Kcnq5/Pde4b
tags=100%, list=37%, signal=63%	Rere/Chd1/Son/Zeb2/Sptbn1/Herc1
tags=67%, list=11%, signal=60%	Jun/Klf4/Map2k1/Tspo
tags=33%, list=9%, signal=31%	Hspa1a/Hspa1b/Senp6/Fkbp4
tags=12%, list=3%, signal=13%	Hspa1a/Hspa8/Hspa1b/Hsp90aa1/Capg
tags=29%, list=10%, signal=27%	Arnt/mt-Nd4/mt-Nd3/mt-Atp6/mt-Cytb/mt-Nd1/mt-Co1/mt-Atp8/mt-Co3/mt-Nd2/mt-Co2/mt-Nd5/mt-Nd4l/mt-Nd6
tags=43%, list=16%, signal=37%	Jun/Fos/Hsp90ab1/Myc/Gpx1/Umps/Tspo/Cd69/Pcna/Bcar3
tags=67%, list=1%, signal=66%	Hspa1a/Hspa1b
tags=67%, list=1%, signal=66%	Hspa1a/Hspa1b
tags=27%, list=3%, signal=27%	Hspa1a/Hspa8/Dazap1
tags=50%, list=3%, signal=49%	Lrrc8c/Itga4/Il10ra/Irs2/Wnk1
tags=51%, list=37%, signal=34%	Smarce1/Flii/Bptf/Xylt1/Ube3b/Nacc1/Cnot1/Cltc/Ppp2ca/Kmt2c/Tbce/Kat6a/Rere/Phf21a/Ube4b/Stat4/Zmynd11/Wipi2/Ep300/Celf2/Tpp1/Ccnd2/Rnf125/Hivep2/Mecp2/Sars/Usp7/Med13/Ankrd11/Foxp1/Fbxo11/Zeb2/Rf
tags=56%, list=37%, signal=37%	Smarce1/Flii/Bptf/Xylt1/Chd7/Ube3b/Rad21/Rasa2/Ogt/Ubr1/Phip/Rere/Phf21a/Ube4b/Wipi2/Ep300/Rnf6/Mecp2/Usp7/Med13/Ankrd11/Foxp1/Kras/Fbxo11/Ppp1r15b/Tcf4/Kmt2e/Ddx6/Crebbp/Satb1/Trappc9/Kdm6b
tags=50%, list=13%, signal=44%	Fos/Dazap1/Junb/Umps/Cited2/Fkbp4/Bsg
tags=100%, list=14%, signal=86%	Rps20/Rps7/Rpl37
tags=100%, list=14%, signal=86%	Rps20/Rps7/Rpl37
tags=37%, list=19%, signal=31%	Ndufv1/H2-Q7/Ankrd11/Kras/mt-Nd4/mt-Nd3/mt-Atp6/mt-Nd1/mt-Co1/mt-Co3/mt-Nd2/mt-Co2/mt-Nd5/mt-Nd6
tags=19%, list=4%, signal=18%	Hspa1a/Hspa1b/Atf3/Pmaip1/Gpx1
tags=64%, list=23%, signal=50%	Rps17/Rps5/Rps8/Rpsa/Rps24/Rps7/Rpl38/Rps27/Rps21/Rps16/Abt1/Rps19/Rps28/Npm1
tags=50%, list=7%, signal=47%	Jun/Fos/Junb/Jund
tags=40%, list=1%, signal=40%	Hspa1a/Hspa1b
tags=75%, list=31%, signal=52%	Jak1/Stat1/Ivns1abp/H2-Q7/Atad1/Tcf4/Syk/Bach2/Il10ra
tags=13%, list=7%, signal=14%	Dnajb1/Hspa1a/Jun/Atf3/Klf2/Fos/Sertad1/Klf4/Id3/Klf6/Nr4a1/Klf10/Myc/Rsf1/Junb/Cited2/Egr1/Foxp4/Jund
tags=20%, list=4%, signal=19%	Hspe1/Klf4/Nr4a1/Gapdh/Pmaip1/Myc/Gpx1/Hspd1
tags=61%, list=27%, signal=45%	Zmynd11/Ep300/Hivep2/Cux1/Ankrd11/Son/Zeb2/Tcf4/Crebbp/Kcnq5/Satb1
tags=69%, list=47%, signal=38%	Sart3/Atrx/Iws1/Ep400/Kdm7a/Eny2/Phf20l1/Phf20/Ube2b/Atf2/Snw1/Brd8/Prkcb/Jmjd1c/Pax5/Ogt/Rtf1/Kmt2c/Paf1/Kat6a/Per1/Ldb1/Ep300/Ctnnb1/Chtop/Mecp2/Usp16/Usp15/Usp7/Prdm2/Epc1/Camk2d/Bcl6/Supt20/
tags=100%, list=9%, signal=91%	Smg1
tags=67%, list=1%, signal=66%	Hspa1a/Hspa1b
tags=67%, list=1%, signal=66%	Hspa1a/Hspa1b
tags=18%, list=4%, signal=18%	Hspb1/Jun/Klf4/Rhob/Nr4a1/Gpx1
tags=41%, list=26%, signal=32%	Setx/Ep300/Minpp1/Tpp1/Mecp2/Ndufv1/H2-Q7/Fus/Zfyve27/Kras/Zeb2/Atad1/Matr3/Sptbn1/Ppp1r15b/mt-Nd4/Crebbp/mt-Nd3/mt-Atp6/Pgap1/mt-Nd1/mt-Nd2/mt-Nd5/mt-Nd6
tags=25%, list=1%, signal=25%	Hspa1a/Hspa1b
tags=25%, list=1%, signal=25%	Hspa1a/Hspa1b
tags=50%, list=15%, signal=43%	Med13/Ppp1r15b/Kmt2e/Ddx6/Cfl2/Setd2/Satb1
tags=79%, list=37%, signal=50%	Bptf/Chd7/Rere/Ube4b/H2-Q7/Med13/Kmt2e/Ddx6/Ganab/Aff4/Satb1
tags=75%, list=11%, signal=67%	Ankrd13c/Cblb/Mef2c
tags=17%, list=4%, signal=17%	Hspa1a/Hspa1b/Ubb/Hsp90aa1/Hsp90ab1/Gpx1
tags=100%, list=25%, signal=75%	Crebbp/Ikbkb/Syk
tags=100%, list=25%, signal=75%	Zeb2/Atad1/Crebbp
tags=100%, list=25%, signal=75%	Ctnnb1/Crebbp/Setd2
tags=100%, list=28%, signal=73%	Stat4/H2-Q7/Malt1/Foxp1/Tcf4/Birc3
tags=12%, list=2%, signal=13%	Hspa1a/Hspa1b/Ubb/Dnaja1/Hsp90aa1/Hsp90ab1
tags=83%, list=18%, signal=69%	H2-Q7/Cd2/Cebpg/Arid5a/Pde4b
tags=67%, list=1%, signal=66%	Klf2/Klf4
tags=21%, list=4%, signal=21%	Hspa1a/Hspa1b/Ubb/Hsp90ab1/Gpx1
tags=67%, list=5%, signal=64%	Aff4/Wnk1
tags=33%, list=18%, signal=29%	Hspa1a/Hspb1/Hspa8/Hspa1b/Rhob/Csrp1/Traf4/Actg1/Rplp2/Rps17/Rps5/Rps8/Rpl31/Rpl18/Map2k1/Rps4x/Rpl19/Rala/Vapa/Rpl8/Rps7/Bsg/Rpl38/Rpl30/Bcar3/Rpl7a/Rps16/Rps2/Rps13
tags=23%, list=2%, signal=23%	Hspa1a/Hspa8/Rhob
tags=100%, list=15%, signal=85%	Pold4/Sirt1/Pcna
tags=18%, list=2%, signal=18%	Hspb1/Jun/Klf4/Rhob
tags=75%, list=22%, signal=59%	Bcl2/H2-Q7/Malt1/Bcl6/Foxp1/Birc3
tags=26%, list=9%, signal=24%	Hspa1a/Hspa8/Hspa1b/Ptges3/Zfp36/Sirt1/Fkbp4
tags=56%, list=39%, signal=36%	Pten/Prpf8/Apoe/Bptf/Xylt1/Chd7/Ube3b/Stk11/Jmjd1c/Rasa2/Ubr1/Phip/Kat6a/Rere/Tmem127/Ube4b/Stat4/Eif2ak3/Ep300/Bcl2/Ctnnb1/Atp2c1/H2-Q7/Fus/Nfkb1/Bms1/Med13/Ankrd11/Bcl6/Tcirg1/Kras/Sptbn1/Tcf4/Km
tags=27%, list=9%, signal=25%	Hspa1a/Jun/Fos/Hsp90ab1/Cited2/Egr1/Sirt1/Ube2d1
tags=9%, list=2%, signal=10%	Dnajb1/Hspb1/Hspa8/Fos/Ubb/Hsp90aa1/Hsp90ab1
tags=50%, list=1%, signal=50%	Hspa1a/Hspa1b
tags=31%, list=15%, signal=28%	Med13/Foxp1/Matr3/Sptbn1/Ppp1r15b/Kmt2e/Ddx6/mt-Nd4/mt-Nd3/mt-Atp6/mt-Nd1/mt-Nd2/Satb1/mt-Nd5/mt-Nd6
tags=50%, list=12%, signal=44%	Akap13/S1pr1/S1pr4/Pde4b/Adgre5
tags=15%, list=6%, signal=15%	Hspb1/Jun/Ppp1r15a/Klf4/Dnaja1/Id3/Nfkbia/Nr4a1/Gapdh/Dusp1/Gpx1/Rrad/Zfp36/Rsf1
tags=100%, list=10%, signal=90%	Smchd1
tags=100%, list=10%, signal=90%	Smchd1
tags=100%, list=10%, signal=90%	Bach2
tags=60%, list=2%, signal=59%	Klf2/Klf4/Nr4a1
tags=67%, list=5%, signal=63%	mt-Atp6/mt-Atp8
tags=100%, list=41%, signal=60%	Apoe/Stat4/Bcl2/Ctnnb1/H2-Q7/Fus/Bcl6/Kras
tags=67%, list=24%, signal=52%	Ubb/Rps17/Rpl31/Rps26/Rpl18/Rps24/Rps20/Rps7/Rpl35/Rps27/Rpl15/Rps10/Rpl35a/Rps19/Rps28/Bin1
tags=41%, list=30%, signal=31%	Rere/Tmem127/Ube4b/Eif2ak3/Setx/Daxx/Hps3/Pml/Rnf213/Sh2b3/Ctnnb1/Krit1/Atp2c1/H2-Q7/Nfkb1/Nfkb2/Tcirg1/Kras/Sptbn1/Ppp1r15b/Tcf4/Kmt2e/Ddx6/mt-Nd4/Syk/Setd2/mt-Atp6/mt-Cytb/mt-Nd1/mt-Co1/mt-Co3/
tags=67%, list=6%, signal=63%	Syk/Wnk1
tags=67%, list=3%, signal=65%	Atp2b1/Trim25
tags=13%, list=2%, signal=13%	Hspb1/Jun/Fos/Klf4/Dnaja1/Nfkbia/Nr4a1
tags=62%, list=38%, signal=39%	Apoe/Ptpn2/Xylt1/Chd7/Ube3b/Rere/Tmem127/Ube4b/Stat4/Ep300/Ctnnb1/Krit1/H2-Q7/Malt1/Foxp1/Kras/Zeb2/Sptbn1/Tcf4/Ddx6/Crebbp/Sf3b1/Smchd1/Birc3
tags=86%, list=30%, signal=60%	Chd8/Ivns1abp/Bdp1/Dhx36/Smarca5/Sf3b1
tags=73%, list=41%, signal=44%	Pten/Bptf/Xylt1/Kat6a/Ep300/Rnf6/Mecp2/Usp7/Med13/Zeb2/Tcf12/Ppp1r15b/Kmt2e/Ddx6/Crebbp/Satb1
tags=21%, list=4%, signal=21%	mt-Cytb/mt-Nd1/mt-Co1/mt-Co3/mt-Co2/mt-Nd5/mt-Nd6
tags=36%, list=8%, signal=33%	Hspa1a/Pmaip1/Gpx1/Hspd1/Chchd10
tags=30%, list=2%, signal=30%	Hspa8/Hsp90aa1/Ifi30
tags=14%, list=2%, signal=14%	Hspa1a/Jun/Fos/Hsp90ab1
tags=100%, list=8%, signal=92%	Fth1/Ftl1
tags=100%, list=8%, signal=92%	Fth1/Ftl1
tags=100%, list=8%, signal=92%	Hspd1/Ftl1
tags=67%, list=2%, signal=65%	Hspb1/Nr4a1
tags=63%, list=39%, signal=39%	Prpf8/Smarce1/Chd7/Rasa2/Polg/Rere/Ube4b/Ctnnb1/Mecp2/Usp7/Kras/mt-Nd4/Smchd1/mt-Nd1/mt-Co1/mt-Co3/mt-Co2/mt-Nd5/mt-Nd6
tags=33%, list=1%, signal=33%	Hspa1a/Hspa1b
tags=50%, list=1%, signal=50%	Hspb1/Hspa8
tags=50%, list=13%, signal=44%	Hspa1a/Hspa1b/Rpsa/Bsg

tags=33%, list=8%, signal=31%	Atf3/Ubb/Pmaip1/Myc/Dedd2/Sirt1
tags=100%, list=8%, signal=92%	Ftl1/Chchd10
tags=100%, list=0%, signal=100%	Hspb1
tags=100%, list=0%, signal=100%	Hspb1
tags=100%, list=0%, signal=100%	Hspb1
tags=100%, list=0%, signal=100%	Hspb1
tags=100%, list=0%, signal=100%	Hspb1
tags=100%, list=0%, signal=100%	Hspb1
tags=100%, list=0%, signal=100%	Hspb1
tags=100%, list=0%, signal=100%	Hspb1
tags=88%, list=35%, signal=58%	Hspb1/Chchd10/Ifrd1/Bin1/Gars/Uba1/Nop56
tags=80%, list=14%, signal=69%	Son/Kras/Zeb2/Smchd1
tags=43%, list=8%, signal=40%	Ano6/Cab39/Wnk1
tags=30%, list=11%, signal=27%	Hspa1a/Hspa1b/Klf2/Nfkbia/Zfp36/Sirt1/Traf1/Tnfrsf13c
tags=27%, list=11%, signal=25%	Hspa1a/Hspa8/Hspa1b/Fos/Ptges3/Zfp36/Sirt1/Fkbp4/Tspo
tags=75%, list=13%, signal=65%	Kras/Akap13/P2ry10
tags=24%, list=11%, signal=23%	Hspb1/Jun/Klf2/Klf4/Rhob/Nr4a1/Gpx1/Actg1/Junb/Cited2/Egr1/Sirt1/Map2k1/Nfatc1/Rap1a
tags=57%, list=37%, signal=37%	Flii/Nacc1/Cltc/Kat6a/Rere/Ube4b/Zmynd11/Ep300/Chd1/Hivep2/Mecp2/Sars/Cux1/Foxp1/Zeb2/Sptbn1/Tcf4/Ddx6/Crebbp/Mef2c/Kcnq5/Pgap1/Satb1/Trappc9
tags=100%, list=20%, signal=80%	Son/Pgap1
tags=100%, list=20%, signal=80%	Polr2a/Satb1
tags=100%, list=20%, signal=80%	Foxp1/Sptbn1
tags=100%, list=8%, signal=92%	Cited2/Sirt1
tags=100%, list=8%, signal=92%	Ptges3/Sirt1
tags=100%, list=8%, signal=92%	Cited2/Sirt1
tags=48%, list=22%, signal=38%	Tpp1/Ctnnb1/Birc2/Cd2/Myo1c/S1pr1/Cblb/Inpp5d/Ikbkb/Ahnak/Pag1/Birc3/Atp2b1
tags=57%, list=14%, signal=49%	Malt1/Cd86/Sptbn1/Pde4b
tags=57%, list=13%, signal=50%	Kras/Zeb2/Tcf4/Ddx6
tags=73%, list=26%, signal=55%	Ubb/Pmaip1/Myc/Sirt1/Rps7/Fis1/Rps3/Flcn
tags=30%, list=14%, signal=27%	Fbxl20/Tcirg1/Cd84/Klf7/Syk/Mef2c/Irs2/Nsf/Adgre5/Wnk1
tags=82%, list=34%, signal=54%	Ubr1/Kat6a/Ep300/Malt1/Foxp1/Kras/Crebbp/Smchd1/Birc3
tags=73%, list=29%, signal=52%	Tmem127/Ep300/Mecp2/Med13/Kmt2e/Ddx6/Crebbp/Satb1
tags=80%, list=27%, signal=59%	Eif2ak3/Minpp1/Sbno2/Tcirg1/S1pr1/Ano6/Mef2c/Atp2b1
tags=100%, list=2%, signal=98%	P2ry10/Gpr171
tags=42%, list=10%, signal=38%	Ppp1r15b/Crebbp/Smchd1/Pgap1/Satb1
tags=75%, list=22%, signal=59%	Tpp1/Ctnnb1/Tcirg1/S1pr1/Inpp5d/Syk
tags=50%, list=15%, signal=43%	Med13/Tcirg1/Kras/Zeb2/Kmt2e/Ddx6/Satb1
tags=48%, list=36%, signal=33%	Chd7/Ube3b/Stk11/Jmjd1c/Rad21/Ubr1/Tbce/Kat6a/Jak1/Polg/Rere/Ube4b/Stat1/Stat4/Ep300/Minpp1/Ivns1abp/Ctnnb1/Ptprj/Mecp2/H2-Q7/Son/Kras/Zeb2/Tcf4/Sh3kbp1/mt-Nd4/Crebbp/Syk/Setd2/Bach2/Il10ra/Smad7/
tags=100%, list=10%, signal=90%	Smad7
tags=100%, list=11%, signal=90%	Satb1
tags=66%, list=27%, signal=49%	Ubb/Rps17/Rpl31/Rps26/Rpl18/Rps24/Rps20/Rps7/Rpl35/Rps27/Polr1d/Rpl15/Rps10/Rpl35a/Rps19/Rps28/Tmco1/Arhgap31/Chuk
tags=16%, list=6%, signal=16%	Hspb1/Jun/Klf2/Klf4/Rhob/Id3/Nr4a1/Cacybp/Gpx1/Actg1/Junb/Cited2/Egr1
tags=88%, list=27%, signal=65%	Eif2ak3/Minpp1/Sbno2/S1pr1/Ano6/Mef2c/Atp2b1
tags=57%, list=13%, signal=50%	Dhx36/Akap13/Mef2c/Kdm6b
tags=12%, list=2%, signal=12%	Dnajb1/Hspa8/Ubb/Hsp90aa1/Hsp90ab1
tags=24%, list=8%, signal=24%	Sptbn1/mt-Nd4/Ganab/mt-Nd3/mt-Atp6/mt-Nd1/mt-Co1/mt-Co3/mt-Nd2/mt-Co2/mt-Nd5/mt-Nd6
tags=25%, list=13%, signal=22%	Hspa1a/Jun/Hspa8/Hspa1b/Hsp90ab1/Zfp36/Rsf1/Eif3f/Rpsa/Atg12/Vapa/Trim59/Bsg
tags=75%, list=15%, signal=64%	Siglecg/Syk/Pag1
tags=43%, list=2%, signal=42%	Ubb/Hsp90aa1/Hsp90ab1
tags=29%, list=1%, signal=29%	Hspa1a/Ppp1r15a
tags=65%, list=31%, signal=46%	Jmjd1c/Jak1/Stat4/Ep300/Ivns1abp/Il4ra/Chd1/H2-Q7/Usp7/Nfkb2/Foxp1/Sptbn1/Tcf4/Crebbp/Aff4
tags=9%, list=4%, signal=10%	Hspa1a/Hspb1/Jun/Hspa1b/Atf3/Ubb/Dnaja1/Hsp90aa1/Hsp90ab1/Nfkbia/Gapdh/Dusp1/Pmaip1/Myc/Gpx1
tags=22%, list=11%, signal=22%	Hspa1a/Hspb1/Hspa1b/Fos/Klf4/Hsp90ab1/Rhob/Nfkbid/Klf10/Myc/Zfp36/Junb/Cited2/Jund/Sirt1/Nap1l1/Map2k1/Rap1a/Tspo/Rala
tags=30%, list=21%, signal=27%	Hivep2/Mecp2/Sars/Polr2a/Ndufv1/Fus/Usp7/Cux1/Med13/Ankrd11/Son/Foxp1/Kras/Sptbn1/Ubtf/Tcf4/Kmt2e/Ddx6/Cfl2/Crebbp/mt-Nd3/Mef2c/Kcnq5/Setd2/Stt3b/Aff4/Ralgapa1/Pgap1/mt-Nd1/mt-Nd2/Satb1/Trappc9/W
tags=48%, list=21%, signal=38%	Ctnnb1/Ptprj/H2-Q7/Myo1c/Sptbn1/Diaph1/Plekho1/Atp2b1/Macf1/Adgre5
tags=55%, list=41%, signal=35%	Pten/Pex7/Dnm2/Taf15/Smarce1/Slc12a6/Chd7/Ube3b/Nacc1/Rasa2/Cltc/Tbce/Polg/Ranbp2/Stat4/Eif2ak3/Setx/Minpp1/Pml/Celf2/Mecp2/Sars/Dnajb2/Ndufv1/H2-Q7/Fus/Bms1/Zfyve27/Tcirg1/Fbxo38/Kras/Rfc1/Matr3/P
tags=24%, list=9%, signal=22%	Hspa1a/Hspa1b/Klf2/Nfkbia/Nr4a1/Ptges3/Cited2/Sirt1/Fkbp4
tags=100%, list=9%, signal=91%	Azin1/Dhps
tags=75%, list=5%, signal=72%	Ifi30/Myc/Fth1
tags=100%, list=9%, signal=91%	Sirt1/Esd
tags=50%, list=1%, signal=49%	Dnajb1/Dnaja1
tags=50%, list=1%, signal=49%	Dnajb1/Dnaja1
tags=74%, list=18%, signal=61%	Pten/Bptf/Chd7/Rere/Ube4b/H2-Q7/Med13/Sptbn1/Kmt2e/Ddx6/Ganab/Setd2/Aff4/Satb1
tags=100%, list=11%, signal=89%	S1pr4
tags=100%, list=11%, signal=89%	Syk
tags=100%, list=11%, signal=89%	Syk
tags=100%, list=11%, signal=89%	Syk
tags=100%, list=11%, signal=89%	S1pr4
tags=80%, list=31%, signal=55%	Jak1/Stat1/Ivns1abp/H2-Q7/Tcf4/Syk/Bach2/Il10ra
tags=60%, list=6%, signal=57%	Jun/Nfkbia/Rsf1
tags=67%, list=6%, signal=63%	Zbtb10/Smg1
tags=48%, list=23%, signal=38%	Il4ra/Bcl2/Ctnnb1/Mecp2/Kras/Spag9/Akap13/Plekho1/Cfl2/Tmod3/Mef2c/Nfatc3/Kdm6b
tags=55%, list=15%, signal=47%	Med13/Zeb2/Kmt2e/Ddx6/Crebbp/Satb1
tags=50%, list=8%, signal=46%	Hspb1/Gpx1/Sirt1
tags=50%, list=8%, signal=46%	Hspb1/Gpx1/Sirt1
tags=56%, list=34%, signal=38%	Cltc/Kat6a/Rere/Ube4b/Zmynd11/Wipi2/Ctnnb1/Hivep2/Mecp2/Sars/Cux1/Zeb2/Diaph1/Ppp1r15b/Tcf4/Kcnq5/Pgap1/Trappc9
tags=67%, list=3%, signal=65%	Cab39/Smad7
tags=52%, list=23%, signal=41%	Rc3h2/Il4ra/H2-Q7/Cd2/Pcid2/Malt1/Bcl6/Cd86/Inpp5d/Syk/Smad7
tags=36%, list=8%, signal=34%	Hspa1a/Ppp1r15a/Pmaip1/Sirt1
tags=34%, list=15%, signal=30%	Med13/Ankrd11/Kras/Ppp1r15b/Kmt2e/Ddx6/Herc1/Pgap1/Satb1/Trappc9/Kdm6b
tags=100%, list=12%, signal=89%	S1pr4
tags=100%, list=12%, signal=89%	S1pr4
tags=100%, list=12%, signal=89%	S1pr4
tags=44%, list=14%, signal=38%	Son/Sptbn1/Ppp1r15b/Tcf4/Ddx6/Aff4/Trappc9
tags=43%, list=20%, signal=35%	Mecp2/Polr2a/Fus/Son/Foxp1/Fbxo11/Zeb2/Kmt2e/Crebbp/Pgap1/Satb1/Trappc9
tags=78%, list=41%, signal=47%	Pten/Bptf/Ubr1/Phip/Rere/Tmem127/Ep300/Med13/Kras/Kmt2e/Ddx6/Crebbp/Satb1/Kdm6b
tags=50%, list=17%, signal=42%	Usp7/Med13/Zeb2/Tcf12/Kmt2e/Ddx6/Crebbp/Satb1
tags=21%, list=4%, signal=20%	Hspe1/Klf4/Nr4a1/Pmaip1/Myc/Gpx1/Hspd1
tags=40%, list=1%, signal=40%	Hspa1a/Hspa1b
tags=100%, list=12%, signal=88%	Tcf4
tags=23%, list=4%, signal=22%	Hspb1/Klf4/Rhob/Nr4a1/Gpx1
tags=71%, list=19%, signal=58%	Usp16/Usp15/Usp7/Supt20/Usp36
tags=100%, list=41%, signal=60%	Apoe/Stat4/Ctnnb1/H2-Q7/Fus/Kras
tags=53%, list=21%, signal=43%	Ptprj/Myo1c/Sptbn1/Diaph1/Plekho1/Atp2b1/Macf1/Adgre5
tags=70%, list=41%, signal=42%	Pten/Bptf/Chd7/Ubr1/Rere/Ube4b/Stat1/H2-Q7/Med13/Sptbn1/Kmt2e/Ddx6/Ganab/Setd2/Aff4/Satb1
tags=67%, list=5%, signal=64%	Hspb1/Actg1
tags=88%, list=53%, signal=42%	Rabgef1/Peli1/Ptprc/Itch/Cnr2/H2-M3/Tnfaip8l2/Laptm5/Apoe/Ptpn2/Bank1/Rc3h2/Il4ra/Sh2b3/H2-Q7/Bcl6/Cd86/Cd84/Cblb/Inpp5d/Pag1/Smad7
tags=18%, list=2%, signal=18%	Hspa1a/Hspa1b/Tubb5
tags=13%, list=7%, signal=14%	Dnajb1/Hspa1a/Jun/Hspa8/Atf3/Klf2/Klf4/Rasd1/Id3/Gapdh/Klf10/Myc/Dbi/Zfp36/Rsf1/Cited2/Egr1/Foxp4/Jund
tags=26%, list=6%, signal=25%	Hspa1a/Hspa1b/Klf2/Zfp36/Rps17/Cited2
tags=40%, list=1%, signal=40%	Jun/Fos
tags=61%, list=42%, signal=37%	Arid1a/Pten/Pex7/Copb2/Smarce1/Ube3b/Nacc1/Rad21/Cltc/Kat6a/Zmynd11/Wipi2/Hivep2/Mecp2/Sars/Ndufv1/Cux1/Son/Foxp1/Zeb2/Ubtf/Ppp1r15b/Tcf4/Herc1/Mef2c/Kcnq5/Setd2/mt-Atp6/Pgap1/Satb1/Trappc9
tags=100%, list=0%, signal=100%	Jun
tags=57%, list=14%, signal=50%	Bcl6/Akap13/Cdc42se2/P2ry10
tags=100%, list=9%, signal=91%	Nr4a1/Fkbp4
tags=18%, list=5%, signal=17%	Hspa1a/Hspa8/Hspa1b/Capg/Gpx1/Actg1
tags=33%, list=19%, signal=28%	Ndufv1/Cep83/H2-Q7/Son/Sptbn1/Tcf4/mt-Nd4/Ganab/mt-Nd3/Setd2/mt-Nd1/mt-Co1/mt-Co3/mt-Nd2/mt-Co2/mt-Nd5/mt-Nd6
tags=60%, list=8%, signal=56%	Ano6/Mef2c/Atp2b1
tags=14%, list=1%, signal=14%	Dnajb1/Hspa8
tags=27%, list=4%, signal=27%	Hspa1a/Hspa1b/Gpx1
tags=55%, list=15%, signal=47%	Klf2/Myc/Hnrnpab/Lamtor2/Bcl11a/Pcna
tags=100%, list=12%, signal=88%	Pde4b
tags=100%, list=12%, signal=88%	Pde4b
tags=100%, list=12%, signal=88%	Pde4b
tags=100%, list=12%, signal=88%	Pde4b
tags=13%, list=5%, signal=14%	Hspb1/Jun/Atf3/Fos/Hsp90aa1/Hsp90ab1/Nfkbia/Nr4a1/Gapdh/Cacybp/Tuba1a/Ms4a1/Traf4/Fth1/Dbi/Actg1/P4ha1
tags=78%, list=23%, signal=60%	Mrps6/Rps5/Rps4x/Rpl8/Rpl37/Rps13/Rps3
tags=62%, list=19%, signal=51%	Krit1/Ndufv1/mt-Nd3/mt-Nd1/mt-Nd2
tags=100%, list=22%, signal=78%	Zeb2/Mef2c
tags=100%, list=22%, signal=78%	Mef2c/Smad7
tags=100%, list=22%, signal=78%	Zeb2/Mef2c
tags=53%, list=31%, signal=38%	Polg/Tmem127/Stat4/Daxx/Pml/Bcl2/Mecp2/Ifngr1/H2-Q7/Fus/Malt1/Bcl6/Foxp1/Kras/Tcf4/Syk/Birc3
tags=60%, list=8%, signal=55%	Hspb1/Gpx1/Sirt1
tags=67%, list=7%, signal=62%	Ikbkb/Il10ra
tags=50%, list=11%, signal=45%	Hspb1/Actg1/Lamtor2/Rap1a
tags=44%, list=21%, signal=36%	Hivep2/Mecp2/Cux1/Nfkb2/Son/Zeb2/Tcf4/Kcnq5/Setd2/Pgap1/Satb1
tags=54%, list=37%, signal=36%	Smarce1/Flii/Bptf/Slc12a6/Xylt1/Ube3b/Rad21/Ogt/Kmt2c/Tbce/Phip/Kat6a/Rere/Arcn1/Ube4b/Ep300/Ctnnb1/Usp7/Med13/Ankrd11/Son/Foxp1/Kras/Fbxo11/Zeb2/Tcf4/Kmt2e/Ddx6/Crebbp/Herc1/Setd2/Sf3b1/Aff4/Satb1
tags=67%, list=6%, signal=63%	Mef2c/Itga4
tags=73%, list=50%, signal=38%	Gns/Ccdc47/Lmnb1/Ppp3ca/Arid1b/Hnrnph2/Arid1a/Smarce1/Ube3b/Nacc1/Tbce/Kat6a/Rere/Ube4b/Ep300/Ccnd2/Hivep2/Mecp2/Usp7/Zeb2/Sptbn1/Ubtf/Tcf4/Crebbp/Herc1/Kcnq5/Ralgapa1/Pgap1/Satb1/Macf1
tags=100%, list=9%, signal=91%	Klf2/Map2k1
tags=100%, list=9%, signal=91%	Nfkbia/Map2k1
tags=39%, list=15%, signal=34%	Ankrd11/Son/Kras/Sptbn1/Ppp1r15b/Tcf4/Kmt2e/Crebbp/Syk/Aff4/Kdm6b
tags=88%, list=57%, signal=38%	Hspe1/Mrps6/Gpx1/Mrpl52/Hspd1/Mrps12/Aldh2/Atp5e/Atp5c1/Prdx3/Rps3/Ndufs8/C1qbp/Gars/Dhx30/Gls/Mrpl15/Clpp/Glud1/Aco2/Atp5a1/Hspa9/Tfam/Mrps7/Isca1/Acss1/Hadha/Nr3c1
tags=38%, list=23%, signal=31%	Ktn1/Il4ra/Ptprj/Ifngr1/Plaur/H2-Q7/Birc2/Cd2/Tcirg1/Lrrc8c/S1pr1/Cd84/S1pr4/Ikbkb/Kcnq5/Itga4/Atp2b1/Il10ra/P2ry10/Adgre5
tags=50%, list=1%, signal=50%	Atf3/Klf2
tags=100%, list=30%, signal=70%	Usp7/Foxp1/Sptbn1/Satb1
tags=20%, list=1%, signal=20%	Hspa1a/Hspa1b
tags=75%, list=15%, signal=64%	Pcid2/Inpp5d/Syk
tags=75%, list=15%, signal=64%	Ankrd11/Son/Mef2c
tags=100%, list=31%, signal=70%	Egr1/Sirt1/Pcna/Arrb2/Casp2

tags=40%, list=2%, signal=39%	Hspa8/Hsp90ab1
tags=71%, list=26%, signal=54%	Rps17/Rpl31/Rps26/Rpl18/Rps24/Rps20/Rps7/Rpl35/Rps27/Rpl15/Rps10/Rpl35a/Rps19/Rps28/Flcn
tags=71%, list=23%, signal=55%	Rc3h2/Il4ra/Bcl6/Cblb/Smad7
tags=83%, list=25%, signal=63%	Ep300/Ctnnb1/Krit1/Crebbp/Setd2
tags=100%, list=23%, signal=78%	Bcl6/Cd86
tags=100%, list=23%, signal=78%	Bcl6/Cd86
tags=32%, list=14%, signal=29%	Camk2d/Tcirg1/Diaph1/Ano6/Ahnak/Mef2c/Kcnq5/Cab39/Irs2/Pde4b/Wnk1
tags=80%, list=14%, signal=69%	Myc/Fth1/Ftl1/Cyb561a3
tags=67%, list=7%, signal=62%	Smarca5/Acin1
tags=24%, list=4%, signal=23%	Jun/Klf2/Fos/Rhob/Gpx1
tags=100%, list=13%, signal=87%	Mef2c
tags=100%, list=13%, signal=87%	Tcf4
tags=100%, list=13%, signal=87%	Ddx6
tags=11%, list=3%, signal=11%	Hspa1a/Jun/Atf3/Ppp1r15a/Pmaip1
tags=38%, list=11%, signal=34%	Hspa1a/Pmaip1/Gpx1/Chchd10/Tspo/Rala
tags=78%, list=30%, signal=55%	Eif4g2/Bcl2/Snrnp70/Usp36/Herc1/Il10ra/Smg1
tags=94%, list=52%, signal=45%	Peli1/Itch/H2-M3/Tnfaip8l2/Laptm5/Ptpn2/Bank1/Rc3h2/Il4ra/H2-Q7/Bcl6/Cd86/Cblb/Inpp5d/Pag1/Smad7
tags=64%, list=22%, signal=50%	Dnaja1/Hsp90aa1/Timm8b/Hspd1/Tspo/Rala/Ube2j2/Fis1/Sae1
tags=66%, list=37%, signal=42%	Bptf/Chd7/Ube3b/Jmjd1c/Kat6a/Rere/Ube4b/Ep300/Ccnd2/Hivep2/Mecp2/Med13/Son/Fbxo11/Ppp1r15b/Kmt2e/Ddx6/Crebbp/Satb1
tags=50%, list=1%, signal=50%	Jun/Fos
tags=70%, list=27%, signal=52%	Eif2ak3/Ctnnb1/Pbxip1/Arid5a/Mef2c/Mef2d/Smad7
tags=75%, list=14%, signal=65%	Son/Kras/Ganab
tags=22%, list=9%, signal=20%	Dnajb1/Hspa1a/Cited2/Sirt1/Sap18
tags=57%, list=37%, signal=37%	Slc12a6/Jmjd1c/Tbce/Kat6a/Rere/Ube4b/Ep300/Rnf125/Usp7/Ankrd11/Son/Tcirg1/Kras/Zeb2/Tcf12/Ddx6/Crebbp/Herc1/Setd2/Aff4/Wnk1
tags=57%, list=19%, signal=47%	Ndufv1/mt-Nd3/mt-Nd1/mt-Nd2
tags=100%, list=1%, signal=99%	
tags=67%, list=19%, signal=54%	Ndufv1/mt-Nd3/mt-Nd1/mt-Nd2
tags=100%, list=23%, signal=77%	Sirt1/Pcna/Rps3/Npm1
tags=90%, list=66%, signal=32%	Kmt2a/Nop56/Glud1/Sec24c/Tnrc6b/Nemf/Gdi1/Csnk2a1/Nipbl/Chd2/Iqsec1/Cyfip2/Nsd1/Zfp292/Trip12/Tbl1x/Naa15/Herc2/Gns/Vps13a/Ppp3ca/Ankrd17/Atrx/Arid1b/Hnrnph2/Arid1a/Smarce1/Flii/Bptf/Chd7/Jmjd1c/Rad2[illegible]
tags=10%, list=3%, signal=10%	Dnajb1/Hspa1a/Hspa8/Hsp90ab1/Capg/Fxyd5
tags=64%, list=37%, signal=41%	Smarce1/Slc12a6/Ube3b/Jmjd1c/Cnot1/Kmt2c/Phip/Phf21a/Hivep2/Son/Fbxo11/Zeb2/Tcf4/Kmt2e/Ddx6/Herc1/Trappc9/Wnk1
tags=56%, list=15%, signal=48%	Med13/Ankrd11/Kmt2e/Ddx6/Satb1
tags=100%, list=10%, signal=90%	Gpx1/Rpsa
tags=64%, list=30%, signal=46%	Eif4g2/Golga4/Adam10/Ist1/Bcl2/Zfyve27/Mul1/Smad7/Macf1
tags=64%, list=30%, signal=46%	Eif4g2/Golga4/Ist1/Bcl2/Zfyve27/Mul1/Mef2c/Smad7/Macf1
tags=75%, list=12%, signal=67%	S1pr1/Inpp5d/Syk
tags=61%, list=23%, signal=48%	Rps17/Rpl31/Rps26/Rpl18/Rps24/Rps20/Rps7/Rpl35/Rps27/Rpl15/Rps10/Rpl35a/Rps19/Rps28
tags=50%, list=8%, signal=46%	Jun/Nr4a1/Myc/Sirt1
tags=11%, list=3%, signal=12%	Hspb1/Jun/Fos/Klf4/Dnaja1/Id3/Nfkbia/Nr4a1/Chchd2/Myc
tags=38%, list=8%, signal=35%	Hspb1/Gpx1/Sirt1
tags=44%, list=10%, signal=40%	Kmt2e/Mef2c/Kcnq5/Nsf
tags=100%, list=14%, signal=86%	Smchd1
tags=100%, list=14%, signal=86%	Mef2c
tags=100%, list=14%, signal=86%	Kras
tags=100%, list=14%, signal=86%	Kras
tags=100%, list=14%, signal=86%	Kras
tags=100%, list=14%, signal=86%	Sptbn1
tags=67%, list=13%, signal=58%	Ubb/Myc/Sirt1/Rps7
tags=29%, list=8%, signal=27%	Jun/Fos/Klf10/Myc/Junb/Cited2/Sirt1
tags=89%, list=38%, signal=55%	Apoe/Smarce1/Bcl2/H2-Q7/Malt1/Bcl6/Foxp1/Birc3
tags=40%, list=1%, signal=40%	Hspa1a/Hspa1b
tags=75%, list=3%, signal=73%	Adam10/Itga4/Wnk1
tags=75%, list=3%, signal=73%	Adam10/Itga4/Wnk1
tags=100%, list=11%, signal=89%	Cited2/Tspo
tags=100%, list=11%, signal=89%	Egr1/Tspo
tags=100%, list=11%, signal=89%	Egr1/Tspo
tags=100%, list=11%, signal=89%	Egr1/Tspo
tags=100%, list=11%, signal=89%	Myc/Tspo
tags=100%, list=11%, signal=89%	Myc/Tspo
tags=100%, list=11%, signal=89%	Myc/Tspo
tags=100%, list=11%, signal=89%	Egr1/Tspo
tags=100%, list=24%, signal=76%	Gapdh/Rpl30/Rps19/Rpl39
tags=100%, list=51%, signal=50%	Rpsa/Eif3k/C1qbp/Tmco1/Serbp1/Ythdf3/Zc3h12a/Naa15/Naa10/Ccdc47
tags=100%, list=14%, signal=86%	Herc1
tags=100%, list=11%, signal=89%	Traf4/Traf1
tags=21%, list=4%, signal=21%	Jun/Hsp90ab1/Gapdh/Gpx1
tags=100%, list=1%, signal=99%	
tags=100%, list=1%, signal=99%	
tags=100%, list=1%, signal=99%	
tags=100%, list=1%, signal=99%	
tags=100%, list=1%, signal=99%	
tags=100%, list=1%, signal=99%	
tags=100%, list=1%, signal=99%	
tags=100%, list=1%, signal=99%	
tags=100%, list=1%, signal=99%	
tags=100%, list=1%, signal=99%	
tags=100%, list=1%, signal=99%	
tags=100%, list=1%, signal=99%	
tags=100%, list=1%, signal=99%	
tags=83%, list=23%, signal=64%	Hspa1a/Sirt1/Pcna/Rps3/Npm1
tags=25%, list=8%, signal=23%	Hspa8/Atf3/Pmaip1/Klf10/Zfp36/Sirt1
tags=73%, list=27%, signal=55%	Nhp2/Wdr74/Rpl38/Rpl35/Rpl7a/Rpl24/Pes1/Rpl14/Rpl35a/Npm1/Dhx30
tags=27%, list=3%, signal=26%	Hspa8/Hsp90aa1/Hsp90ab1/Capg
tags=38%, list=8%, signal=35%	Hspa1a/Pmaip1/Sirt1
tags=64%, list=16%, signal=54%	Ep300/Nfkb2/Son/Kras/Zeb2/Crebbp/Setd2
tags=70%, list=23%, signal=54%	Rc3h2/Il4ra/Malt1/Bcl6/Cd86/Syk/Smad7
tags=19%, list=3%, signal=18%	Hspa8/Atf3/Fos/Pmaip1/Klf10
tags=61%, list=37%, signal=39%	Flii/Bptf/Xylt1/Chd7/Ube3b/Rad21/Rasa2/Ogt/Ubr1/Rere/Phf21a/Ube4b/Wipi2/Ep300/Mecp2/Usp7/Ankrd11/Kras/Tcf4/Crebbp/Trappc9/Kdm6b
tags=12%, list=5%, signal=13%	Hspa1a/Hspe1/Ppp1r15a/Klf4/Hsp90aa1/Hsp90ab1/Pmaip1/Myc/Ptges3/Hspd1/Traf4/Dbi
tags=75%, list=27%, signal=55%	Eif2ak3/Ctnnb1/Arid5a/Mef2c/Mef2d/Smad7
tags=75%, list=12%, signal=66%	Rab43/Ddx6/Vps37b
tags=33%, list=12%, signal=30%	S1pr1/Ano6/Syk/Pde4b/Adgre5/Wnk1
tags=60%, list=21%, signal=48%	Pmaip1/Chchd2/Myc/Cited2/Egr1/Sirt1/Eno1/Nfe2l2/Pgk1
tags=57%, list=15%, signal=49%	Med13/Kmt2e/Ddx6/Satb1
tags=57%, list=15%, signal=49%	Med13/Kmt2e/Ddx6/Satb1
tags=75%, list=6%, signal=70%	Gpx1/Ptges3/Egr1
tags=100%, list=1%, signal=100%	Hspa8
tags=100%, list=1%, signal=100%	Hspa8
tags=100%, list=1%, signal=100%	Hspa8
tags=100%, list=1%, signal=100%	Hspa8
tags=100%, list=1%, signal=100%	Hspa8
tags=100%, list=1%, signal=100%	Hspa8
tags=100%, list=1%, signal=100%	Hspa8
tags=100%, list=30%, signal=71%	Ldb1/Ctnnb1/Pgap1
tags=100%, list=12%, signal=88%	Sla/Syk/Clk1
tags=58%, list=23%, signal=45%	Rps17/Rpl31/Rps26/Rpl18/Rps24/Rps20/Rps7/Rpl35/Rps27/Polr1d/Rpl15/Rps10/Rpl35a/Rps19/Rps28
tags=12%, list=6%, signal=13%	Hspa1a/Jun/Hspa8/Atf3/Klf2/Fos/Sertad1/Klf4/Hsp90aa1/Hsp90ab1/Klf6/Nfkbia/Nr4a1/Chchd2/Klf10/Myc/Dazap1/Ptges3/Zfp36/Rsf1/Junb/Hnrnpab/Cited2/Egr1
tags=58%, list=22%, signal=46%	Tpp1/Ctnnb1/Tcirg1/S1pr1/Inpp5d/Syk/Mef2c
tags=100%, list=14%, signal=86%	Kdm5a
tags=100%, list=14%, signal=86%	Kdm5a
tags=100%, list=14%, signal=86%	Pde4b
tags=18%, list=5%, signal=18%	Hspa8/Atf3/Fos/Pmaip1/Klf10/Gpx1/Zfp36
tags=8%, list=3%, signal=8%	Dnajb1/Hspa1a/Hspa8/Hspa1b/Hsp90aa1/Capg
tags=64%, list=23%, signal=50%	Rps17/Rpl31/Rps26/Rpl18/Rps24/Rps20/Rps7/Rpl35/Rps27/Rpl15/Rps10/Rpl35a/Rps19/Rps28
tags=43%, list=6%, signal=41%	Setd2/Ralgapa1/Satb1
tags=15%, list=7%, signal=15%	Jun/Atf3/Klf2/Fos/Sertad1/Klf4/Klf6/Nfkbia/Nr4a1/Chchd2/Klf10/Myc/Rsf1/Junb/Cited2/Egr1/Jund
tags=43%, list=11%, signal=38%	Hspa1a/Gpx1/Tspo
tags=29%, list=1%, signal=29%	Hspa1a/Hspa1b
tags=100%, list=14%, signal=86%	Wnk1
tags=15%, list=5%, signal=15%	Hspb1/Sertad1/Klf4/Dnaja1/Hsp90aa1/Hsp90ab1/Dusp1/Myc/Ptges3/Traf4/Dbi/Zfp36
tags=12%, list=4%, signal=12%	Hspa1a/Jun/Hspa1b/Ubb/Hsp90aa1/Hsp90ab1/Gapdh/Pmaip1/Myc/Gpx1/Traf4
tags=16%, list=5%, signal=16%	Hspa1a/Hspa8/Hspa1b/Id3/Nfkbia/Myc/Ptges3/Zfp36
tags=100%, list=30%, signal=70%	Dhx36/Smarca5/Sf3b1
tags=75%, list=18%, signal=62%	Birc2/Birc6/Birc3
tags=57%, list=13%, signal=50%	Ubb/Myc/Sirt1/Eif2b2
tags=43%, list=4%, signal=42%	Atf3/Pmaip1/Gpx1
tags=60%, list=4%, signal=58%	Mrps6/Mrpl52/Mrps12
tags=100%, list=15%, signal=85%	Son
tags=100%, list=15%, signal=85%	Aff4
tags=37%, list=14%, signal=32%	Bcl6/Kras/Akap13/Cdc42se2/P2ry10/Dennd4a/Cyth1
tags=29%, list=10%, signal=27%	Hspa1a/Ppp1r15a/Pmaip1/Sirt1/Dnajb9
tags=83%, list=21%, signal=66%	Dazap1/Junb/Cited2/Bsg/Prdx3
tags=83%, list=21%, signal=66%	Dazap1/Junb/Cited2/Bsg/Prdx3
tags=70%, list=18%, signal=58%	Stat4/Ifngr1/H2-Q7/Malt1/Foxp1/Bach2/Birc3
tags=100%, list=26%, signal=75%	Tspo/Fis1/Parp1/Flcn
tags=77%, list=37%, signal=49%	Bptf/Ogt/Ubr1/Ep300/Med13/Foxp1/Kmt2e/Ddx6/Crebbp/Satb1
tags=16%, list=2%, signal=16%	Hspb1/Klf2/Klf4/Rhob
tags=15%, list=1%, signal=15%	Jun/Hspa8
tags=50%, list=0%, signal=50%	Hspa1a

tags=30%, list=5%, signal=29%	Hspa1a/Hspa1b/Zfp36
tags=75%, list=28%, signal=54%	Stat1/Ivns1abp/Tcf4/Syk/Bach2/Il10ra
tags=51%, list=24%, signal=40%	Pop7/Fcf1/Rps17/Rps5/Rps8/Rpsa/Rps24/Nhp2/Rps7/Wdr74/Rpl38/Rpl35/Rps27/Rps21/Rpl7a/Rps16/Rpl24/Pes1/Rpl14/Abt1/Rpl35a/Rps19/Rps28/Npm1/C1qbp
tags=9%, list=2%, signal=10%	Hspa1a/Hspb1/Hspa1b/Klf2/Klf4/Hsp90aa1/Hsp90ab1/Gapdh
tags=13%, list=2%, signal=13%	Dnajb1/Hspa8/Hsp90aa1/Hsp90ab1
tags=13%, list=5%, signal=13%	Hspb1/Ppp1r15a/Klf4/Hsp90aa1/Hsp90ab1/Id3/Gapdh/Myc/Ptges3/Dbi/Zfp36
tags=100%, list=1%, signal=99%	
tags=67%, list=36%, signal=43%	Chd7/Ube3b/Jmjd1c/Kat6a/Ep300/Kras/Matr3/Sptbn1/Crebbp/Aff4/mt-Cytb/Ralgapa1
tags=16%, list=2%, signal=16%	Hspa1a/Hspa1b/Hsp90ab1
tags=67%, list=37%, signal=43%	Slc12a6/Cnot1/Kmt2c/Phip/Phf21a/Hivep2/Fbxo11/Tcf4/Kmt2e/Ddx6/Trappc9/Wnk1
tags=16%, list=9%, signal=16%	Hspa1a/Jun/Hspa8/Hspa1b/Klf2/Fos/Hsp90ab1/Nr4a1/Myc/Ptges3/Zfp36/Hnrnpab/Cited2/Egr1/Sirt1/Lamtor2/Fkbp4/Ube2d1
tags=12%, list=4%, signal=12%	Hspa1a/Hspe1/Ppp1r15a/Hsp90ab1/Pmaip1/Myc/Hspd1
tags=50%, list=0%, signal=50%	Hspb1
tags=40%, list=8%, signal=37%	Ano6/Kcnq5/mt-Atp6/mt-Atp8
tags=67%, list=1%, signal=66%	Klf2/Klf4
tags=51%, list=23%, signal=41%	Pop7/Fcf1/Rps17/Rps8/Dedd2/Rps24/Nhp2/Rps7/Wdr74/Rpl35/Rps27/Rps21/Rpl7a/Rps16/Pes1/Rpl14/Abt1/Rpl35a/Rps19/Rps28
tags=65%, list=51%, signal=35%	Tfam/Ptprc/Lmbrd1/Itch/Gns/Cd55/Ccdc47/Vps13a/Pstpip1/Atrx/Arid1b/Mrps7/Psap/Epb41/Hadha/Cd81/Arid1a/Plekhm1/Dpm1/Pten/Pex7/Apoe/Ptpn2/Smarce1/Xylt1/Chd7/Stk11/Jmjd1c/Rasa2/Cnot1/Ubr1/Jak1/Polg/Re
tags=75%, list=16%, signal=64%	Pde7a/Neil1/Pde4b
tags=60%, list=23%, signal=47%	Rc3h2/Il4ra/Bcl2/Malt1/Bcl6/Foxp1/Cd86/Syk/Smad7
tags=67%, list=2%, signal=65%	Hspb1/Nr4a1
tags=43%, list=12%, signal=38%	S1pr1/Itga4/Wnk1
tags=14%, list=5%, signal=14%	Hspa1a/Hspa1b/Klf2/Fos/Klf4/Hsp90ab1/Nfkbia/Gapdh/Cacybp/Dusp1/Myc/Zfp36/Actg1
tags=38%, list=11%, signal=34%	Dnajb1/Sirt1/Ahsa1
tags=10%, list=5%, signal=11%	Hspa1a/Hspe1/Hspa8/Hspa1b/Dnaja1/Rasd1/Hsp90aa1/Hsp90ab1/Tubb5/Rhob/Tuba1a/Hspd1/Rrad/Dbi/Actg1
tags=60%, list=12%, signal=53%	Akap13/Mef2c/Kdm6b
tags=80%, list=23%, signal=62%	Il4ra/Cd84/Syk/Adgre5
tags=11%, list=2%, signal=12%	Hspa1a/Hspa8/Hspa1b/Hsp90ab1
tags=15%, list=4%, signal=15%	Hspa8/Dnaja1/Hsp90aa1/Hsp90ab1/Nfkbia/Timm8b/Hspd1
tags=17%, list=8%, signal=16%	Hspa1a/Hspb1/Hspa1b/Hsp90aa1/Gapdh/Hspd1/Egr1/Gpsm3/Sirt1
tags=67%, list=3%, signal=65%	Ubb/Myc
tags=75%, list=18%, signal=62%	Nfkb1/Tcirg1/Bach2
tags=35%, list=15%, signal=32%	Rc3h2/Il4ra/Bcl2/Ptprj/Cr1l/H2-Q7/Cd2/Pcid2/Malt1/Bcl6/Foxp1/Fbxo38/Dhx36/Matr3/Cd86/Cd84/Cblb/Sh3kbp1/Inpp5d/Ano6/Crebbp/Arid5a/Ikbkb/Syk/Mef2c/Acin1/Itga4/Irs2/Pde4b/Adgre5/Wnk1
tags=100%, list=1%, signal=99%	
tags=61%, list=23%, signal=48%	Rps17/Rpl31/Rps26/Rpl18/Rps24/Rps20/Rps7/Rpl35/Rps27/Rpl15/Rps10/Rpl35a/Rps19/Rps28
tags=100%, list=54%, signal=47%	Chd4/Kdm6a/Tox4/Foxo1/Smarce1/Sbno1/Jmjd1c/Per1/Ep300/Sbno2/Bcl6/Crebbp/Kdm5a/Kdm6b
tags=11%, list=4%, signal=11%	Hspa1a/Hspb1/Atf3/Dnaja1/Dusp1/Myc/Gpx1
tags=18%, list=3%, signal=18%	Hspa1a/Hspa1b/Hsp90aa1/Capg
tags=58%, list=23%, signal=45%	Rps17/Rpl31/Rps26/Rpl18/Map2k1/Rps24/Rps20/Rps7/Rpl35/Rps27/Rpl15/Rps10/Rpl35a/Rps19/Rps28
tags=38%, list=20%, signal=31%	Mecp2/H2-Q7/Usp7/Med13/Ankrd11/Son/Kras/Atad1/Sptbn1/Ubtf/Ppp1r15b/Kmt2e/Ddx6/Cfl2/Herc1/Setd2/Satb1
tags=82%, list=40%, signal=50%	Dnm2/Apoe/Eif4g2/Golga4/Ist1/Rnf6/Zfyve27/Dip2b/Macf1
tags=14%, list=3%, signal=14%	Hspa1a/Hspa1b/Tubb5/Gapdh/Tuba1a
tags=67%, list=3%, signal=65%	Jun/Myc
tags=19%, list=4%, signal=19%	Hspa1a/Hspa1b/Nfkbia/Hspd1
tags=23%, list=2%, signal=23%	Hspa1a/Hspa1b/Nfkbia
tags=100%, list=14%, signal=86%	Fth1/Cyb561a3
tags=100%, list=1%, signal=99%	Ppp1r15a
tags=43%, list=8%, signal=40%	Hspa1a/Pmaip1/Chchd10
tags=86%, list=39%, signal=53%	Inpp5k/Foxo1/Ptpn2/Stk11/Ogt/Cltc/Plek/Ranbp2/Ep300/Nfkb1/Usp7/Irs2
tags=62%, list=24%, signal=48%	Ubb/Rps17/Rpl31/Rps26/Rpl18/Rps24/Rps20/Rps7/Rpl35/Rps27/Rpl15/Rps10/Rpl35a/Rps19/Rps28/Bin1
tags=45%, list=23%, signal=36%	Rc3h2/Il4ra/Cr1l/H2-Q7/Nfkb2/Malt1/Bcl6/Tcirg1/Fbxo38/Inpp5d/Arid5a/Mef2c/Bach2/Smad7
tags=75%, list=20%, signal=60%	Polg/Mecp2/Ndufv1/mt-Nd3/mt-Nd1/mt-Nd2
tags=75%, list=20%, signal=60%	Polg/Mecp2/Ndufv1/mt-Nd3/mt-Nd1/mt-Nd2
tags=33%, list=7%, signal=32%	Ahnak/Mef2c/Cab39/Pde4b/Wnk1
tags=33%, list=1%, signal=33%	Hspa1a/Hspa1b
tags=55%, list=37%, signal=36%	Slc12a6/Jmjd1c/Ubr1/Tbce/Kat6a/Rere/Ube4b/Ep300/Rnf125/Usp7/Ankrd11/Son/Tcirg1/Kras/Zeb2/Tcf12/Sptbn1/Ddx6/Crebbp/Herc1/Setd2/Aff4/Wnk1
tags=28%, list=18%, signal=25%	Ifngr1/H2-Q7/Nfkb1/Nfkb2/Ankrd11/Malt1/Tcirg1/Foxp1/Zeb2/Atad1/Sptbn1/Tcf4/Sh3kbp1/mt-Nd4/Ganab/Crebbp/Ikbkb/Syk/Setd2/Bach2/Sf3b1/Aff4/Birc3/Il10ra/mt-Nd1/mt-Co1/mt-Co3/mt-Co2/mt-Nd5/mt-Nd6
tags=100%, list=14%, signal=86%	Rps20/Rpl37
tags=67%, list=15%, signal=57%	Ep300/Med13/Kmt2e/Ddx6/Crebbp/Satb1
tags=89%, list=57%, signal=39%	Zfp292/Trip12/Zfp148/Chd4/Donson/Ehmt1/Herc2/Lmnb1/Ankrd17/Atrx/Arid1b/Pex7/Copb2/Flii/Bptf/Ube3b/Jmjd1c/Cltc/Phip/Rere/Eif2ak3/Ep300/Minpp1/Mecp2/Ankrd11/Sptbn1/Tcf4/Crebbp/Herc1/Mef2c/Pgap1
tags=62%, list=21%, signal=50%	Rnf125/Usp15/Birc2/Birc3/Trim25
tags=21%, list=8%, signal=20%	mt-Nd4/mt-Nd3/mt-Cytb/mt-Nd1/mt-Co1/mt-Co3/mt-Nd2/mt-Co2/mt-Nd5/mt-Nd6
tags=45%, list=15%, signal=39%	Ankrd11/Ppp1r15b/Aff4/Trappc9/Kdm6b
tags=50%, list=15%, signal=43%	Med13/Kmt2e/Ddx6/Mef2c/Satb1
tags=14%, list=7%, signal=14%	Hspa1a/Hspa8/Hspa1b/Fos/Nr4a1/Myc/Gpx1/Ptges3/Zfp36/Cited2/Egr1/Jund
tags=100%, list=1%, signal=99%	Klf2
tags=100%, list=1%, signal=99%	Klf2
tags=100%, list=1%, signal=99%	Klf2
tags=48%, list=23%, signal=38%	Hspb1/Ppp1r15a/Rps17/Rps5/Eif3f/Eif2b2/Eif3k/Rpl13a/Eif1ax/Eif1/Eif3e/Rps3/Npm1
tags=10%, list=4%, signal=11%	Hspa1a/Hspb1/Hspa1b/Fos/Klf4/Hsp90aa1/Rhob/Gapdh/Nfkbid/Klf10/Myc/Hspd1
tags=21%, list=12%, signal=20%	Hspa1a/Hspa8/Hspa1b/Hsp90aa1/Hsp90ab1/Tubb5/Hspd1/Fth1/Dynll1/Ftl1/Syngr2/Lamtor2/Man2b1/Rap1a/Ostf1/Plekho2/Vapa
tags=64%, list=37%, signal=41%	Bptf/Stk11/Ubr1/Phip/Rere/Tmem127/Ube4b/Ep300/H2-Q7/Med13/Kras/Kmt2e/Ddx6/Crebbp/Satb1/Kdm6b
tags=42%, list=17%, signal=35%	Hspa1a/Hsp90aa1/Pmaip1/Timm8b/Hspd1/Chchd10/Tspo/Atp5e/Atp5c1/Ube2j2
tags=100%, list=1%, signal=99%	
tags=100%, list=1%, signal=99%	
tags=21%, list=2%, signal=21%	Hspa1a/Hspa1b/Nfkbia
tags=11%, list=3%, signal=11%	Hspa1a/Hspa1b/Hsp90aa1/Capg
tags=100%, list=14%, signal=86%	Esd/Aldh2
tags=100%, list=14%, signal=86%	Azin1/Aldh2
tags=55%, list=27%, signal=41%	Ldb1/Ildr1/Bcl2/Ctnnb1/Ptprj/Mecp2/Myo1c/Ikbkb/Mef2c/Smad7/Macf1
tags=38%, list=8%, signal=35%	Hspa1a/Pmaip1/Chchd10
tags=15%, list=3%, signal=15%	Hspa1a/Hspa1b/Capg
tags=16%, list=3%, signal=16%	Hspa8/Atf3/Fos/Pmaip1/Klf10
tags=40%, list=24%, signal=32%	Sbno2/Rc3h2/Il4ra/Ptprj/Cr1l/Dock2/H2-Q7/Cebpg/Malt1/Bcl6/Tcirg1/Foxp1/Fbxo38/Dhx36/Cd86/Cd84/Kmt2e/Inpp5d/Arid5a/Syk/Smad7/Adgre5
tags=30%, list=9%, signal=28%	Jun/Nr4a1/Myc/Gpx1/Zfp36/Sirt1/Map2k1
tags=100%, list=14%, signal=86%	Hspa8/Vamp2
tags=100%, list=14%, signal=86%	Hspa8/Vamp2
tags=80%, list=19%, signal=65%	Usp16/Usp15/Usp7/Supt20
tags=20%, list=4%, signal=20%	Hspa1a/Hspa1b/Gpx1
tags=38%, list=8%, signal=35%	Ano6/Itga4/Wnk1
tags=50%, list=11%, signal=45%	Hsp90aa1/Hsp90ab1/Ptges3/Nhp2
tags=25%, list=1%, signal=25%	Jun/Fos
tags=34%, list=15%, signal=30%	Med13/Son/Foxp1/Kmt2e/Ddx6/Crebbp/Herc1/Setd2/Satb1/Kdm6b
tags=62%, list=23%, signal=48%	Il4ra/H2-Q7/Pcid2/Malt1/Bcl6/Cd86/Inpp5d/Syk
tags=85%, list=56%, signal=38%	Ubr4/Trip12/Arih2/Trim11/Rabgef1/Peli1/Rnf34/Herc2/Itch/Brap/Ube3b/Znrf2/Ubr1/Ranbp2/Ube4b/Rnf6/Rc3h2/Rnf213/Rnf125/Chfr/Mycbp2/Birc2/Hltf/Mul1/Arih1/Neurl3/Cblb/Birc3/Trim25
tags=70%, list=32%, signal=48%	Limd1/Eif4g2/Adam10/Ctnnb1/Tmod3/Smad7/Cyth1
tags=60%, list=15%, signal=51%	Ep300/Med13/Kmt2e/Ddx6/Crebbp/Satb1
tags=50%, list=15%, signal=43%	Med13/Kmt2e/Ddx6/Herc1/Satb1
tags=50%, list=15%, signal=43%	Med13/Ppp1r15b/Kmt2e/Ddx6/Satb1
tags=80%, list=36%, signal=51%	Chd7/Ube3b/Jmjd1c/Kat6a/Ep300/Crebbp/Aff4/Ralgapa1
tags=62%, list=22%, signal=49%	Tpp1/Mecp2/Zeb2/Sptbn1/Ppp1r15b
tags=42%, list=12%, signal=37%	Akap13/S1pr1/Mef2c/Smad7/Kdm6b
tags=78%, list=41%, signal=47%	Pten/Dnm2/Apoe/Slc12a6/Rb1cc1/Plek/Eif4g2/Golga4/Ist1/Rnf6/Zfyve27/Ano6/Dip2b/Macf1
tags=73%, list=3%, signal=72%	Inpp5k/Atf2/Prkcb/Stk11/Hipk3/Bcl2/Camk2d/Clk1/Cab39/Smad7/Wnk1
tags=53%, list=23%, signal=41%	Il4ra/Bcl2/Kras/Spag9/Akap13/Plekho1/Cfl2/Tmod3/Mef2c/Kdm6b
tags=67%, list=19%, signal=54%	Krit1/H2-Q7/Ano6/Itga4
tags=17%, list=5%, signal=17%	Hspa1a/Hspa1b/Dusp1/Naa50
tags=80%, list=23%, signal=62%	Rc3h2/Il4ra/Bcl6/Smad7
tags=80%, list=23%, signal=62%	Rc3h2/Il4ra/Bcl6/Smad7
tags=80%, list=23%, signal=62%	Rc3h2/Il4ra/Bcl6/Smad7
tags=80%, list=23%, signal=62%	Rc3h2/Il4ra/Bcl6/Smad7
tags=12%, list=3%, signal=12%	Dnajb1/Marcksl1/Ubb/Hsp90aa1/Hsp90ab1/Id3/Capg/Myc
tags=80%, list=27%, signal=59%	Atp6v0a1/Tcirg1/mt-Atp6/mt-Atp8
tags=37%, list=21%, signal=30%	Ctnnb1/Hivep2/Mecp2/Sars/Cux1/Med13/Foxp1/Tcf4/Kmt2e/Ddx6/Kcnq5/Setd2/mt-Atp6/Pgap1/Satb1/Trappc9
tags=12%, list=2%, signal=13%	Hspa1a/Hspa1b/Hsp90aa1
tags=69%, list=32%, signal=47%	Tbce/Rere/Ube4b/Ep300/Rnf125/Usp7/Ankrd11/Ddx6/Crebbp
tags=38%, list=11%, signal=34%	Jun/Map2k1/Rap1a
tags=29%, list=11%, signal=27%	Jun/Atf3/Myc/Map2k1/Rap1a
tags=18%, list=5%, signal=18%	Hspa1a/Hspa1b/Fos/Nfkbia/Nfkbid/Klf10/Myc/Zfp36
tags=78%, list=44%, signal=44%	Hadha/Pten/Apoe/Xylt1/Chd7/Ube3b/Rere/Ep300/Ctnnb1/Krit1/H2-Q7/Zeb2/Sptbn1/Crebbp
tags=9%, list=4%, signal=9%	Hspa1a/Hspb1/Hspa1b/Atf3/Ppp1r15a/Dnaja1/Nfkbia/Dusp1/Myc/Gpx1
tags=50%, list=13%, signal=44%	Ubb/Myc/Sirt1/Eif2b2
tags=20%, list=4%, signal=20%	Hspa8/Hsp90aa1/Hsp90ab1/Nfkbia/Hspd1
tags=18%, list=11%, signal=18%	Hspa1a/Jun/Hspa8/Hspa1b/Klf2/Fos/Hsp90ab1/Nr4a1/Myc/Gpx1/Ptges3/Zfp36/Hnrnpab/Cited2/Egr1/Jund/Sirt1/Lamtor2/Fkbp4/Ube2d1/Rap1a/Ier2/Tspo
tags=33%, list=3%, signal=32%	Itga4/Wnk1
tags=62%, list=26%, signal=47%	Rps17/Rpl31/Rps26/Rpl18/Map2k1/Rps24/Rps20/Rps7/Rpl35/Rps27/Rpl15/Rps10/Rpl35a/Rps19/Rps28/Flcn
tags=100%, list=15%, signal=85%	Egr1/Pcna
tags=100%, list=15%, signal=85%	Bcl11a/Pcna
tags=52%, list=25%, signal=40%	Ep300/Celf2/Hivep2/Sars/Fus/Usp7/Sptbn1/Ubtf/Crebbp/Pgap1/Trappc9
tags=100%, list=2%, signal=99%	
tags=100%, list=2%, signal=99%	
tags=100%, list=2%, signal=99%	
tags=100%, list=2%, signal=99%	
tags=100%, list=2%, signal=99%	
tags=100%, list=2%, signal=99%	
tags=100%, list=2%, signal=99%	
tags=100%, list=1%, signal=99%	
tags=100%, list=1%, signal=99%	
tags=100%, list=1%, signal=99%	
tags=100%, list=1%, signal=99%	

tags=100%, list=1%, signal=99%	
tags=24%, list=15%, signal=23%	Med13/Ankrd11/Son/Tcirg1/Foxp1/Kras/Fbxo11/Zeb2/Tcf12/Sptbn1/Ppp1r15b/Kmt2e/Ddx6/mt-Nd4/Crebbp/Herc1/Mef2c/Setd2/Aff4/Smchd1/Ralgapa1/Pgap1/mt-Nd1/mt-Co1/mt-Co3/mt-Co2/Satb1/mt-Nd5/Trappc9/mt-
tags=64%, list=51%, signal=35%	Herc2/Itch/Kdm6a/Ccdc47/Lmnb1/Ppp3ca/Ankrd17/Erlin1/Atrx/Arid1b/Hnrnph2/Inpp5k/Sbf1/Mbtps1/Arid1a/Pten/Pex7/Dnm2/Smarce1/Flii/Bptf/Slc12a6/Xylt1/Chd7/Nacc1/Jmjd1c/Rasa2/Cltc/Kmt2c/Tbce/Rere/Ube4b/Zm
tags=53%, list=42%, signal=34%	Arid1a/Dpm1/Pten/Tdp2/Dnm2/Bptf/Slc12a6/Ube3b/Nacc1/Cnot1/Ubr1/Cltc/Ppp2ca/Kmt2c/Tbce/Phip/Kat6a/Polg/Rere/Phf21a/Ogdh/Ep300/Celf2/Ctnnb1/Rnf125/Chd1/Hivep2/Sars/Polr2a/Ndufv1/Usp7/Cux1/Foxp1/Fbx
tags=62%, list=28%, signal=45%	Stat4/Ep300/H2-Q7/Son/Kras/Zeb2/Sptbn1/Crebbp
tags=53%, list=39%, signal=35%	Foxo1/G3bp1/Apoe/Prkcb/Slc12a6/Chd7/Stk11/Rtf1/Ppp2ca/Limd1/Paf1/Chd8/Btbd9/Ldb1/Adam10/Zdhhc3/Ildr1/Rnf213/Ctnnb1/Zranb1/Mecp2/Csnk1d/Csnk1g3/Nfkb1/Fbxl20/Tcirg1/Kras/Zeb2/Atad1/Pbxip1/Sh3kbp1/
tags=65%, list=39%, signal=40%	Prpf8/Flii/Bptf/Chd7/Jmjd1c/Rad21/Cnot1/Hax1/Rere/Tmem127/Ube4b/Ep300/H2-Q7/Med13/Ankrd11/Kmt2e/Ddx6/Crebbp/Setd2/Aff4/Smchd1/Satb1
tags=56%, list=19%, signal=45%	Csnk1d/Csnk1g3/Nfkb1/Zeb2/Wnk1
tags=65%, list=23%, signal=51%	Rps17/Rpl31/Rps26/Rpl18/Rps24/Rps20/Eif2b2/Rps7/Ndufs6/Rpl35/Rps27/Rpl15/Rps10/Rpl35a/Rps19/Rps28/Ndufs8
tags=18%, list=3%, signal=18%	Hspa8/Atf3/Pmaip1/Klf10
tags=42%, list=21%, signal=34%	Hivep2/Mecp2/Cux1/Nfkb2/Son/Zeb2/Tcf4/Kcnq5/Setd2/Pgap1/Satb1
tags=9%, list=4%, signal=10%	Dnajb1/Hspb1/Jun/Ppp1r15a/Dnaja1/Hsp90aa1/Hsp90ab1/Id3/Gapdh/Fxyd5/Azin1/Rrad
tags=12%, list=2%, signal=12%	Hspa1a/Hspa1b/Klf4/Id3/Nfkbia
tags=15%, list=6%, signal=15%	Hspb1/Jun/Klf2/Klf4/Rhob/Nr4a1/Dusp1/Gpx1/Csrp1/Actg1/Junb/Cited2
tags=67%, list=39%, signal=42%	Taf15/Chd7/Ube3b/Jmjd1c/Kat6a/Ep300/Fus/Kras/Matr3/Sptbn1/Crebbp/Aff4/mt-Cytb/Ralgapa1
tags=43%, list=23%, signal=34%	Celf2/Mecp2/Sars/Atad1/Sptbn1/Pgap1/Satb1/Trappc9/Nsf
tags=25%, list=7%, signal=24%	Jun/Atf3/Fos/Myc/Junb/Hnrnpab/Jund
tags=56%, list=15%, signal=48%	Bank1/Bcl2/Pcid2/Bcl6/Sh3kbp1/Inpp5d/Syk/Mef2c/Irs2
tags=100%, list=16%, signal=85%	Pde4b
tags=100%, list=16%, signal=85%	Pde4b
tags=100%, list=16%, signal=85%	Pde4b
tags=100%, list=16%, signal=85%	Pde4b
tags=73%, list=44%, signal=42%	Hadha/Nr3c1/Tdp2/Chd7/Jmjd1c/Ubr1/Tbce/Tmem127/Eif2ak3/Nfkb2/Tcirg1/Foxp1/Kras/Syk/Setd2/Wnk1
tags=57%, list=26%, signal=43%	Rps17/Umps/Rpl31/Rps26/Rpl18/Rps24/Rps20/Rps7/Rpl35/Rps27/Rpl15/Rps10/Rpl35a/Rps19/Rps28/H2-Ab1
tags=100%, list=15%, signal=85%	Dbi/Mboat7
tags=100%, list=16%, signal=85%	Ubb/Polr1d
tags=24%, list=6%, signal=23%	Hspa1a/Hspa1b/Klf2/Zfp36/Rps17/Cited2
tags=11%, list=3%, signal=11%	Hspa1a/Hspb1/Hspa1b/Klf2/Fos/Klf4/Rhob/Nfkbia/Nfkbid/Klf10/Myc
tags=57%, list=42%, signal=35%	Arid1a/Pten/Dnm2/Prpf8/Smarce1/Bptf/Chd7/Stk11/Jmjd1c/Rad21/Rasa2/Ogt/Ubr1/Tbce/Phip/Kat6a/Polg/Rere/Ube4b/Stat4/Ep300/Minpp1/Mecp2/H2-Q7/Usp7/Med13/Ankrd11/Kras/Zeb2/Ppp1r15b/Tcf4/Kmt2e/Ddx6/C
tags=50%, list=17%, signal=42%	Usp7/Med13/Son/Kmt2e/Ddx6/Satb1
tags=64%, list=42%, signal=38%	Snw1/Arid1a/Brd8/Smarce1/Nucks1/Prkcb/Ets1/Kmt2c/Kat6a/Rere/Cebpz/Ep300/Daxx/Ctnnb1/Usp16/Fus/Birc2/Ncoa2/Med13/Pbxip1/Crebbp/Kdm5a/Trim25
tags=17%, list=6%, signal=17%	Jun/Klf2/Klf4/Tubb5/Rhob/Id3/Myc/Gpx1/Zfp36/Actg1/Cited2
tags=50%, list=15%, signal=43%	Hspa8/Actg1/Vamp2/Mical1
tags=100%, list=52%, signal=48%	Rps26/Rps28/Sec63/Uba1/Suco/Zc3h12a/Ccdc47/Arl6ip1
tags=57%, list=20%, signal=46%	Sars/Zeb2/Pgap1/Trappc9
tags=100%, list=1%, signal=99%	Ubb
tags=100%, list=1%, signal=99%	Ubb
tags=100%, list=1%, signal=99%	Ubb
tags=100%, list=1%, signal=99%	Ubb
tags=100%, list=1%, signal=99%	Ubb
tags=100%, list=1%, signal=99%	Ubb
tags=100%, list=1%, signal=99%	Fos
tags=100%, list=1%, signal=99%	Fos
tags=100%, list=1%, signal=99%	Fos
tags=100%, list=1%, signal=99%	Ubb
tags=100%, list=1%, signal=99%	Fos
tags=100%, list=1%, signal=99%	Fos
tags=100%, list=1%, signal=99%	Fos
tags=100%, list=1%, signal=99%	Ubb
tags=14%, list=0%, signal=14%	Dnajb1
tags=53%, list=37%, signal=35%	Smarce1/Bptf/Chd7/Jmjd1c/Rad21/Rasa2/Ogt/Ubr1/Tbce/Phip/Kat6a/Rere/Ube4b/Ep300/Minpp1/Mecp2/Usp7/Med13/Ankrd11/Kras/Zeb2/Ppp1r15b/Tcf4/Kmt2e/Ddx6/Crebbp/Setd2/Stt3b/Aff4/Smchd1/Satb1/Kdm6b
tags=100%, list=16%, signal=84%	Kif21b
tags=14%, list=4%, signal=14%	Hspa1a/Hspa1b/Ubb/Hsp90aa1/Hsp90ab1/Gpx1/Azin1
tags=25%, list=15%, signal=23%	Ankrd11/Son/Tcirg1/Kras/Zeb2/Sptbn1/Crebbp/Setd2/Aff4/mt-Cytb/mt-Nd1/mt-Co1/mt-Co3/mt-Co2/Satb1/mt-Nd5/mt-Nd6
tags=100%, list=23%, signal=77%	Arid5a/Dip2b
tags=100%, list=23%, signal=77%	Arid5a/Dip2b
tags=50%, list=6%, signal=47%	Ndufv1/mt-Nd3/mt-Nd1/mt-Nd2
tags=88%, list=40%, signal=53%	Dnm2/Apoe/Xylt1/Ranbp2/Ctnnb1/Zeb2/Sf3b1
tags=60%, list=3%, signal=58%	Adam10/Itga4/Wnk1
tags=14%, list=1%, signal=14%	Hspa1a/Hspa1b
tags=27%, list=18%, signal=24%	H2-Q7/Nfkb1/Nfkb2/Med13/Son/Tcirg1/Kras/Zeb2/Sptbn1/Ppp1r15b/Tcf4/Kmt2e/Ddx6/mt-Nd4/Ganab/Crebbp/Syk/Setd2/mt-Atp6/Aff4/mt-Cytb/mt-Nd1/mt-Co3/mt-Nd2/Satb1/mt-Nd5/mt-Nd4l/mt-Nd6
tags=78%, list=56%, signal=36%	Ubr4/Fbxl3/Trip12/Arih2/Trim11/Rabgef1/Peli1/Rnf34/Herc2/Itch/Cul5/Brap/Ube2g1/Ube2b/Ube3b/Znrf2/Ubr1/Ranbp2/Ube4b/Rnf6/Zfp91/Pml/Rc3h2/Rnf213/Rnf125/Chfr/Mycbp2/Birc2/Hltf/Mul1/Malt1/Fbxo11/Arih1/Neurl
tags=38%, list=23%, signal=31%	Celf2/Tpp1/Mecp2/Polr2a/Fus/Fbxo38/Matr3/Ddx6/mt-Nd4/Herc1/mt-Atp6/mt-Cytb/Pgap1/mt-Nd1/mt-Co1/mt-Co3/mt-Co2/mt-Nd5/mt-Nd6
tags=30%, list=6%, signal=28%	Syk/Pde4b/Adgre5
tags=50%, list=6%, signal=47%	Kcnq5/Pde4b
tags=50%, list=2%, signal=49%	Hspa8/Hsp90aa1
tags=22%, list=12%, signal=20%	Hspa1a/Hspa8/Hspa1b/Hsp90aa1/Hsp90ab1/Tubb5/Hspd1/Fth1/Dynll1/Ftl1/Lamtor2/Man2b1/Rap1a/Ostf1/Plekho2/Vapa
tags=56%, list=19%, signal=45%	Krit1/Ndufv1/mt-Nd3/mt-Nd1/mt-Nd2
tags=100%, list=16%, signal=84%	Inpp5d
tags=100%, list=16%, signal=84%	Arnt
tags=100%, list=16%, signal=84%	Bcl6
tags=100%, list=16%, signal=84%	Ppp1r15b
tags=60%, list=40%, signal=37%	Dnm2/Apoe/Rasa2/Ogt/Arhgap26/Ralgps2/Arhgap30/Arhgap15/Arhgap25/Dock2/Bcl6/Kras/Akap13/Cdc42se2/Ralgapa1/P2ry10/Dennd4a/Cyth1
tags=42%, list=21%, signal=34%	Hivep2/Mecp2/Sars/Cux1/Ankrd11/Foxp1/Tcf4/Kcnq5/Setd2/Pgap1/Trappc9
tags=83%, list=54%, signal=39%	Zbtb20/Naa10/Ehmt1/Herc2/Vps13a/Ppp3ca/Atrx/Hnrnph2/Flii/Cltc/Kmt2c/Rere/Ube4b/Zmynd11/Ep300/Ctnnb1/Hivep2/Usp7/Cux1/Foxp1/Tcf4/Kmt2e/Crebbp/Kcnq5
tags=56%, list=17%, signal=47%	Pnpla7/Pde7a/Neil1/Gpcpd1/Pde4b
tags=43%, list=23%, signal=34%	Pml/Bcl2/Ctnnb1/Camk2d/Cd84/Diaph1/Ano6/Ahnak/Cab39/Atp2b1/Pde4b/Wnk1
tags=57%, list=14%, signal=49%	Malt1/Foxp1/Syk/Birc3
tags=100%, list=23%, signal=77%	Hspa1a/Pcna/Rps3
tags=100%, list=23%, signal=77%	Mrps6/Rps13/Rps3
tags=48%, list=23%, signal=38%	Rc3h2/Il4ra/Bcl2/Dock2/H2-Q7/Malt1/Bcl6/Tcirg1/Foxp1/Cd86/Cblb/Syk/Smad7
tags=50%, list=37%, signal=33%	Smarce1/Flii/Bptf/Xylt1/Chd7/Ube3b/Rad21/Ogt/Kmt2c/Phip/Kat6a/Rere/Ube4b/Ep300/Rnf125/Chd1/Med13/Ankrd11/Son/Kras/Zeb2/Sptbn1/Ppp1r15b/Kmt2e/Ddx6/Crebbp/Herc1/Mef2c/Aff4/Smchd1/Ralgapa1/Pgap1/Sa
tags=88%, list=37%, signal=55%	Bptf/Hes6/Tmed2/Ldb1/Ep300/Ctnnb1/Pgap1
tags=62%, list=23%, signal=49%	Il4ra/Dhx36/Cd84/Syk/Adgre5
tags=75%, list=29%, signal=54%	Tmem127/Stat4/Rnf6/Sh2b3/Ctnnb1/H2-Q7
tags=100%, list=29%, signal=71%	Myc/Tspo/Bin1/Il6st
tags=74%, list=38%, signal=48%	Rps17/Rpl31/Rps26/Rpl18/Rps24/Rps20/Rps7/Rpl35/Rps27/Fam111a/Rpl15/Rps10/Rpl35a/Rps19/Rps28/Tonsl/Flcn/Taldo1/Pik3r1/Cyb5a/Eif2s3x/Gdi1/Ngly1
tags=31%, list=14%, signal=27%	Fbxo38/Rfc1/Cfl2/mt-Nd4/mt-Nd3/mt-Atp6/mt-Nd1/mt-Nd2/mt-Nd5/mt-Nd6/Wnk1
tags=100%, list=0%, signal=100%	Wnk1
tags=55%, list=24%, signal=42%	Sbno2/Il4ra/Dock2/Cd84/Syk/Adgre5
tags=75%, list=38%, signal=47%	Apoe/Jmjd1c/Stat4/Ifngr1/H2-Q7/Malt1/Foxp1/Bach2/Birc3
tags=36%, list=14%, signal=32%	Son/Foxp1/Fbxo11/Zeb2/Ppp1r15b/Tcf4/Mef2c/Aff4/Trappc9
tags=67%, list=23%, signal=52%	Il4ra/Cd84/Syk/Adgre5
tags=100%, list=36%, signal=64%	Rad21/Stat1/Minpp1/Rnf6
tags=16%, list=3%, signal=16%	Hspa1a/Hspa1b/Hsp90aa1/Capg
tags=53%, list=23%, signal=41%	Rc3h2/Il4ra/H2-Q7/Bcl6/Cd86/Cblb/Pag1/Smad7/Wnk1
tags=12%, list=5%, signal=12%	Hspa1a/Hspa8/Hspa1b/Fos/Nr4a1/Myc/Ptges3/Zfp36
tags=15%, list=4%, signal=15%	Hspa1a/Hsp90aa1/Pmaip1/Chchd2/Myc/Gpx1/Timm8b/Hspd1
tags=75%, list=22%, signal=59%	Bcl2/Camk2d/Wnk1
tags=21%, list=11%, signal=20%	Sptbn1/Tcf4/mt-Nd4/Ganab/Crebbp/mt-Nd3/Setd2/mt-Atp6/mt-Nd1/mt-Co1/mt-Co3/mt-Nd2/mt-Co2/Satb1/mt-Nd5/mt-Nd6
tags=53%, list=37%, signal=34%	Prkcb/Stk11/Hipk3/Jak1/Map4k2/Sik2/Pik3r4/Eif2ak3/Csnk1d/Csnk1g3/Riok1/Camk2d/Stk38/Ikbkb/Syk/Stk17b/Clk1/Smg1/Wnk1
tags=36%, list=23%, signal=30%	Rc3h2/Il4ra/Bcl2/Ptprj/Rnf125/Cr1l/Usp15/H2-Q7/Birc2/Mul1/Malt1/Bcl6/Foxp1/Fbxo38/Matr3/Cd86/Cd84/Cblb/Inpp5d/Crebbp/Arid5a/Ikbkb/Syk/Mef2c/Pik3ap1/Birc3/Smad7/Pde4b/Trim25/Adgre5/Wnk1
