# Supplementary material for: The Regulation Network of Glycerolipid Metabolism as Coregulators of Immunotherapy-Related Myocarditis
Source: Cardiovasc Ther. 2023 Jun 21;2023:8774971. doi: 10.1155/2023/8774971 (PMC10307211; doi:10.1155/2023/8774971)
Supplement: Supplementary 6 — Table S6: the differentially expressed metabolite results of immunotherapy-related myocarditis via UPLC-MS/MS detection platform. [file 8774971.f6.pdf]

Table S6

Index	Formula	Compounds	__	Class I	_____	Class II	_____	CAS	Cont1	Cont2	Cont3	DCM2	MyoCs2	MyoCs3	VIP	Fold_Change
MEDN0097	C8H8O3	PÐHydroxyphenyl Acetic Acid	_-_____	Benzene and substituted derivatives	_____	Phenolic acids	___	156-38-7	92509	11740	8063.9	25454	9	9	1.076006571	0.226794963
MEDN0105	C26H45NO7S	Taurocholic acid	____	Bile acids	___	Bile acids	___	81-24-3	9	9	9	44092	17905	9	1.233534511	2296.518519
MEDN0198	C6H8O7	Citric Acid	___	Organic acid And Its derivatives	_______	Organicacid and Its derivatives	_______	77-92-9	1233800	424350	1011800	4186000	3564500	2649200	1.543660243	3.895091668
MEDN0283	C4H8O3	2-Hydroxybutanoic Acid	2-____	Organic acid And Its derivatives	_______	Organicacid and Its derivatives	_______	600-15-7	78565	9	9	87629	165740	200490	1.256626396	5.775536694
MEDN0334	C8H8O3	Mandelic Acid	___	Organic acid And Its derivatives	_______	Organicacid and Its derivatives	_______	90-64-2	92509	11740	8063.9	25454	9	9	1.076006571	0.226794963
MEDN0352	C2H8NO4P	O-Phosphorylethanolamine	______	GP	_____	PE	______	1071-23-4	527690	307090	466430	2059500	664650	681400	1.26581687	2.61721782
MEDN0356	C28H44NO9P	Lysops 22:6	_______22:6	GP	_____	LPS	_______	-	1654900	1485500	517810	213730	326790	698240	1.275622716	0.338624628
MEDN0368	C19H40NO7P	Lysope 14:0	_______14:0	GP	_____	LPE	_______	-	6478.8	1894.3	1422	1181	473.9	851.64	1.22053116	0.255897336
MEDN0391	C22H32O2	DHA [4Z,7Z,10Z,13Z,16Z,19Z-docosahexaenoic acid]	______	Oxidized lipids	____	Oxidized lipids	____	6217-54-5	6214400	7799800	3177000	2304900	2045800	2520800	1.433873493	0.399710317
MEDN0394	C20H36O2	Cis-11,14-Eicosadienoic Acid(C20:2)	_-11,14-______	FA	____	FFA	_____	2091-39-6	13514	14982	5146.9	7996.3	3254.3	2892.1	1.124902528	0.420376959
MEDN0396	C10H20O2	Capric Acid(C10:0)	__	FA	____	FFA	_____	334-48-5	2318.4	2231.5	1831.9	2132.3	5075.3	5727.1	1.111892016	2.026810618
MEDN0399	C20H40O2	Arachidic Acid(C20:0)	___	FA	____	FFA	_____	506-30-9	4699.8	2039.6	3110.9	21941	30077	35811	1.624858438	8.916378181
MEDN0502	C5H11O7P	2-Deoxyribose 1-Phosphate	2-____ 1-__	Carboxylic acids and derivatives	_________	Phosphate sugars	___	17210-42-3	1032600	500660	826840	145430	659280	334770	1.118569386	0.48281005
MEDN0506	C8H16NO9P	N-Acetylglucosamine 1-Phosphate	N-______ 1-__	Carboxylic acids and derivatives	_________	Phosphate sugars	___	6866-69-9	205130	92674	89775	1213900	315830	174580	1.225980695	4.397322868
MEDN0561	C11H16N2O8	N-Acetylaspartylglutamic acid	N-_________	Amino acid and Its metabolomics	_______	Amino acid derivatives	_____	3106-85-2	36137	18086	19707	123780	142340	115130	1.628389015	5.156905181
MEDN0593	C4H6O5	D-Malic acid	D-(+)-___	Organic acid And Its derivatives	_______	Organicacid and Its derivatives	_______	636-61-3	32689000	12392000	18235000	5988400	11896000	9554200	1.249540109	0.433359656
MEDN0658	C16H30O4	Hexadecanedioic acid	_____	FA	____	FFA	_____	505-54-4	14519	17686	24184	33283	45113	39464	1.511755496	2.09012396
MEDN0780	C20H34O5	5-iPF2_-VI [(8_)-5,9_,11_-trihydroxy-prosta-6E,14Z-dien-1-oic acid]	(8_)-5,9_,11_-___-prosta-6E,14Z-__-1-_	Oxidized lipids	____	Oxidized lipids	____	180469-63-0	9	9	9	70872	15287	35663	1.666965092	4511.925926
MEDN0781	C20H34O6	6 keto-PGF1_ [6-oxo-9_,11_,15S-trihydroxy-prost-13E-en-1-oic acid]	6-_____F1_	Oxidized lipids	____	Oxidized lipids	____	58962-34-8	9	9	9	49378	2153.1	4262.1	1.627000565	2066.414815
MEDN1041	C18H30O4	9(S)-HpOTrE	9(S)-HpOTrE	Oxidized lipids	____	Oxidized lipids	____	111004-08-1	2285.1	9	9	3604.7	3238	3955.3	1.273480992	4.688463375
MEDN1054	C16H26O3	Tetranor-12(R) -HETE	Tetranor-12(R)-HETE	Oxidized lipids	____	Oxidized lipids	____	135271-51-1	23857	21911	12008	9	9	9	1.671183414	0.000467322
MEDN1055	C18H34N2O10	D-Calcium Pantothenate	D-______B5_	CoEnzyme and vitamins	_____	CoEnzyme and vitamins	_____	137-08-6	19225	33988	36140	2446.6	7634.7	11633	1.450968894	0.243017022
MEDN1063	C20H34O2	Homo-Gamma-Linolenic Acid	Homo-Gamma-Linolenic Acid	Organic acid And Its derivatives	_______	Organicacid and Its derivatives	_______	1783-84-2	13669000	16789000	5738200	8839700	2503100	3050000	1.146878829	0.397632901
MEDN1075	C18H32O4	13-HpODE	13-HpODE	Oxidized lipids	____	Oxidized lipids	____	23017-93-8	8716.3	3022.6	3235.3	25558	28980	42529	1.565154238	6.48228286
MEDN1079	C22H32O2	(4E,7E,10Z,13E,16E,19E) -docosa-4,7,10,13,16,19-hexanoic acid	(4E,7E,10Z,13E,16E,19E)-docosa-4,7,10,13,16,19-___	Oxidized lipids	____	Oxidized lipids	____	25167-62-8	6214400	7799800	3177000	2304900	2045800	2520800	1.433873493	0.399710317
MEDN1098	C6H12O3	2-ethyl-2-hydroxybutyric acid	2-__-2-____	Organic acid And Its derivatives	_______	Organicacid and Its derivatives	_______	3639-21-2	15925	73681	21711	11518	3716.6	5849.5	1.360575838	0.189405931
MEDN1126	C24H40O5	Gamma-Mercholic Acid	_-___	Bile acids	___	Bile acids	___	547-75-1	45566	425590	10318	13566	9	3443.6	1.110771222	0.035346872
MEDN1148	C16H32O3	2-hydroxyhexadecanoic acid	2-_____	FA	____	FFA	_____	764-67-0	53733	87720	46868	209930	121420	117070	1.424410592	2.381147084
MEDN1239	C10H13O10P	5-O-(1-carboxyvinyl) -3-phosphate	5-O-(1-_____)-3-___	Organic acid And Its derivatives	_______	Phosphoric acids	_____	89771-75-5	225460	133510	150130	25666	170900	19387	1.133680701	0.424185818
MEDN1266	C27H46NO7P	PysoPE 22:5(2n isomer3)	_______ 22:5(2n isomer3)	GP	_____	LPE	_______	-	226940	234400	47686	93324	37519	29098	1.067815429	0.314209883
MEDN1267	C27H46NO7P	PysoPE 22:5(2n isomer2)	_______ 22:5(2n isomer2)	GP	_____	LPE	_______	-	226940	234400	47686	93324	37519	29098	1.067815429	0.314209883
MEDN1268	C27H46NO7P	PysoPE 22:5(2n isomer1)	_______ 22:5(2n isomer1)	GP	_____	LPE	_______	-	226940	234400	47686	93324	37519	29098	1.067815429	0.314209883
MEDN1269	C27H44NO7P	PysoPE 22:6(2n isomer1)	_______ 22:6 (2n isomer1)	GP	_____	LPE	_______	-	3432800	2125600	908620	97638	213430	666630	1.435559374	0.151182152
MEDN1270	C27H44NO7P	PysoPE 22:6	_______ 22:6	GP	_____	LPE	_______	-	3432800	2125600	908620	97638	213430	666630	1.435559374	0.151182152
MEDN1276	C25H44NO7P	PysoPE 20:4(2n isomer1)	_______ 20:4 (2n isomer1)	GP	_____	LPE	_______	-	22735000	19918000	5756500	465370	1797900	6213300	1.338577838	0.175101375
MEDN1277	C25H42NO7P	PysoPE 20:5(2n isomer1)	_______ 20:5(2n isomer1)	GP	_____	LPE	_______	-	267130	124070	52701	3254.3	7906.3	24709	1.485768198	0.080805405
MEDN1278	C25H42NO7P	PysoPE 20:5	_______ 20:5	GP	_____	LPE	_______	-	267130	124070	52701	3254.3	7906.3	24709	1.485768198	0.080805405
MEDP0010	C6H14N4O2	L-Arginine	L-___	Amino acid and Its metabolomics	_______	Amino acids	___	74-79-3	1703100	1611600	1787900	7108300	1941200	2333500	1.078732294	2.230823502
MEDP0039	C5H11NO2	Betaine	___	Others	_	Others	_	107-43-7	2734900	544940	1002100	2606700	4227800	2879000	1.237323006	2.268481109
MEDP0083	C5H9NO3	Trans-4-Hydroxy-L-Proline	_-4-__-L-___	Amino acid and Its metabolomics	_______	Amino acid derivatives	_____	51-35-4	10556000	5315300	7249900	1485300	3635700	4179000	1.334435581	0.402228258
MEDP0127	C4H12N2	Putrescine	__	Alcohol and amines	____	Polyamines	_	110-60-1	264610	187450	204830	728890	322470	477420	1.441117787	2.327299852
MEDP0151	C5H5N5O	2-Hydroxy-6-Aminopurine	2-__-6-____	Nucleotide And Its metabolomics	_______	Nucleotide and Its metabolomics	_______	3373-53-3	57937	59778	53314	1805500	48504	307740	1.073080512	12.63963421
MEDP0185	C9H13NO3	Epinephrine	____	Hormones and hormone related compunds	________	Hormones and hormone related compunds	________	51-43-4	9	9	68740	116190	76352	57698	1.223686527	3.639431048
MEDP0272	C10H9NO3	5-Hydroxyindole-3-Acetic Acid	5-____-3-__	Heterocyclic compounds	_____	Indole and Its derivatives	_____	54-16-0	7123.7	9	9	7099.8	8856.5	13283	1.238035057	4.094165255
MEDP0295	C6H11NO3	4-Acetamidobutyric Acid	4-______	Organic acid And Its derivatives	_______	Organicacid and Its derivatives	_______	3025-96-5	38037	9	12995	112770	65621	53887	1.043635666	4.550812092
MEDP0336	C22H46NO7P	Lysopc 14:0	______14:0	GP	_____	LPC	______	20559-16-4	1021200	364670	207070	175210	127310	176290	1.192438296	0.300582571
MEDP0340	C24H48NO7P	Lysopc 16:1	______16:1	GP	_____	LPC	______	-	4715000	1817300	900590	746040	416890	746260	1.245498233	0.256857023
MEDP0344	C26H52NO7P	Lysopc 18:1	______18:1	GP	_____	LPC	______	3542-29-8	60754000	53691000	28547000	11083000	9306400	22967000	1.471309791	0.303208571
MEDP0346	C26H50NO7P	Lysopc 18:2	______18:2	GP	_____	LPC	______	-	53378000	51878000	41048000	6809100	8722500	13407000	1.640583092	0.197797736
MEDP0350	C28H56NO7P	Lysopc 20:1	______20:1	GP	_____	LPC	______	-	328440	169500	64199	112460	10683	34469	1.091739816	0.280379052
MEDP0352	C28H54NO7P	Lysopc 20:2	______20:2	GP	_____	LPC	______	-	1344100	806820	219200	247770	58726	120910	1.275358227	0.180330954
MEDP0373	C4H7NO3	N-Acetylglycine	N-______	Amino acid and Its metabolomics	_______	Amino acid derivatives	_____	543-24-8	5107500	771300	1806800	5552600	9247200	5606700	1.266156282	2.6551603
MEDP0408	C20H30O	11-Cis-Retinol	11-____	CoEnzyme and vitamins	_____	CoEnzyme and vitamins	_____	22737-96-8	788.38	547.62	322.85	2196.9	2434.6	4919.8	1.522803133	5.757784007
MEDP0442	C8H20NO6P	Sn-Glycero-3-Phosphocholine	sn-__-3-____	Tryptamines,Cholines,Pigments	_______	Cholines	___	28319-77-9	8220100	19214000	10178000	782080	2813700	3432900	1.491618964	0.186872841
MEDP0495	C26H50NO7P	Lysope 18:2 (2N Isomer)	_______ 18:2(2n__)	GP	_____	LPE	_______	-	53378000	51878000	41048000	6809100	8722500	13407000	1.640583092	0.197797736
MEDP0514	C12H17N4OS+	Thiamine	___	CoEnzyme and vitamins	_____	CoEnzyme and vitamins	_____	59-43-8	86077	112040	45565	32576	7655.8	28602	1.331092782	0.28247388
MEDP0576	C12H28N4O	N1-Acetylspermine	N1-____	Organic acid And Its derivatives	_______	Organicacid and Its derivatives	_______	25593-72-0	9	9	9	14994	8212	15585	1.669476054	1436.703704
MEDP0689	C8H15NO2	TranexamicAcid	__-4-(___)_____	Organic acid And Its derivatives	_______	Organicacid and Its derivatives	_______	1197-18-8	9	9	9	23116000	23961000	9	1.233353606	1743592.926
MEDP0878	C6H11NO4	N-Methyl-L-Glutamate	N-__-L-___	Amino acid and Its metabolomics	_______	Amino acid derivatives	_____	35989-16-3	1510800	1187600	797500	843010	443040	343490	1.259568005	0.466128894
MEDP0886	C5H9N3O4	N-Amidino-L-Aspartate	N-__-L-____	Amino acid and Its metabolomics	_______	Amino acid derivatives	_____	6133-30-8	60532	18110	26048	92638	101310	34181	1.119084587	2.179090649
MEDP1001	C6H10O2	Delta-Hexalactone	_-___	Aldehyde,Ketones,Esters	_____	Esters	_	823-22-3	108210	127770	56551	23013	46726	46929	1.387457477	0.398822689
MEDP1002	C7H15NO2	Acetylcholine	____	Alcohol and amines	____	Polyamines	_	51-84-3	588890	778320	486040	213240	318820	347180	1.518844035	0.474431404
MEDP1054	C42H62O16	Glycyrrhizinate	___	Organic acid And Its derivatives	_______	Organicacid and Its derivatives	_______	1405-86-3	210850	290330	9	9	9	9	1.207637096	5.38719E-05
MEDP1096	C28H56NO8P	1,2-dodecanoyl PC	1,2-____PC	GP	_____	PC	_____	3436-44-0	68080	53714	40163	2594.6	34235	33469	1.059513737	0.434057188
MEDP1097	C21H38O4	2-Linoleoyl Glycerol	2-______	GL	____	MG	____	3443-82-1	62455	49309	25840	19299	13084	11091	1.44648206	0.315935583
MEDP1109	C26H43NO6	Glycocholic Acid	____	Bile acids	___	Bile acids	___	475-31-0	147380	54153	88446	48747	1542.4	26743	1.084346685	0.265648202
MEDP1118	C21H28O5	Aldosterone	___	Aldehyde,Ketones,Esters	_____	Ketones	_	52-39-1	9	9	9	5571.3	24050	20554	1.650485637	1858.344444
MEDP1124	C20H32O3	(±)15-HETE [(±)15-hydroxy-5Z,8Z,11Z,13E-eicosatetraenoic acid]	(±)15-__-5Z,8Z,11Z,13E-______	Oxidized lipids	____	Oxidized lipids	____	54845-95-3	1884.9	2642.4	1274	7019.8	3918.6	10748	1.426642416	3.738196611
MEDP1132	C7H15NO2	3-Carboxypropyltrimethylammonium	3-_______	Organic acid And Its derivatives	_______	Organicacid and Its derivatives	_______	6249-56-5	588890	778320	486040	213240	318820	347180	1.518844035	0.474431404
MEDP1139	C20H37NO2	Linoleylethanolamide	_______	Alcohol and amines	____	Amines	_	68171-52-8	95982	237520	156840	76205	50362	56709	1.437758265	0.373771776
MEDP1162	C6H7N5O	1-Methylguanine	1-_____	Nucleotide And Its metabolomics	_______	Nucleotide and Its metabolomics	_______	938-85-2	74229	27402	6439.5	130130	70448	63465	1.097292386	2.443247695
MEDP1177	C5H11NO2	N-Methyl-_-aminoisobutyric acid	N-__-_-_____	Amino acid and Its metabolomics	_______	Amino acid derivatives	_____	2566-34-9	32055	4764	965.5	140100	434840	105330	1.428351641	18.00394342
MEDP1188	C27H40O3	Testosterone	___	Hormones and hormone related compunds	________	Hormones and hormone related compunds	________	58-22-0	757.14	1231.8	9	3057.4	2167.6	2948.7	1.033363137	4.091063796
MEDP1190	C14H18N5O11P	Adenylocuccinic Acid	_______	Organic acid And Its derivatives	_______	Organicacid and Its derivatives	_______	19046-78-7	9014200	4417100	3406300	188920	4318700	1172200	1.126536475	0.337329548
MEDP1201	C20H36O6	8,8a-deoxy oleane	8,8a-____	Others	_	Others	_	53428-54-9	25654	269680	4930.4	7272.5	9	9	1.254900791	0.024280268
MEDP1228	C11H19NO6	Heterodendrin	2-_______-3-____	Others	_	Others	_	66465-22-3	4626000	4720800	3037500	537450	3218200	1246100	1.252876582	0.403878298
MEDP1255	C33H42N4O6	Urobilin	___	Tryptamines,Cholines,Pigments	_______	Pigments	_	1856-98-0	31365	9022.4	54126	495430	105660	24585	1.07033529	6.619960767
MEDP1276	C5H5N5O	Guanine	___	Nucleotide And Its metabolomics	_______	Nucleotide and Its metabolomics	_______	73-40-5	57937	59778	53314	1805500	48504	307740	1.073080512	12.63963421
MEDP1298	C21H36O3	Pregnanetriol	___	Alcohol and amines	____	Alcohols	_	1098-45-9	54557	37310	8033.6	5716	3274.5	5676.4	1.361303125	0.146814934
MEDP1318	C30H55NO7P	LysoPC 22:4 (2n isomer1)	_______ 22:4 (2n isomer1)	GP	_____	LPC	______	-	2573900	1792300	273400	291670	102170	177070	1.289794188	0.123051556
MEDP1319	C30H55NO7P	LysoPC 22:4	_______ 22:4	GP	_____	LPC	______	-	2573900	1792300	273400	291670	102170	177070	1.289794188	0.123051556
MEDP1320	C30H53NO7P	LysoPC 22:5 (2n isomer3)	_______ 22:5 (2n isomer3)	GP	_____	LPC	______	-	1454200	953330	389870	94181	94037	143990	1.561099059	0.118755988
MEDP1321	C30H53NO7P	LysoPC 22:5 (2n isomer2)	_______ 22:5 (2n isomer2)	GP	_____	LPC	______	-	1454200	953330	389870	94181	94037	143990	1.561099059	0.118755988
MEDP1322	C30H53NO7P	LysoPC 22:5 (2n isomer1)	_______ 22:5 (2n isomer1)	GP	_____	LPC	______	-	1515300	1004800	393750	86202	96931	123440	1.570936681	0.105212348
MEDP1325	C30H51NO7P	LysoPC 22:6	_______ 22:6	GP	_____	LPC	______	-	8392600	7020800	3189400	721920	790020	1596100	1.558504539	0.167073774
MEDP1331	C28H53NO7P	LysoPC 20:3(2n isomer)	_______ 20:3(2n__)	GP	_____	LPC	______	-	7121800	5344600	1591900	656220	184540	728060	1.452838408	0.111593863
MEDP1332	C28H53NO7P	LysoPC 20:3	_______ 20:3	GP	_____	LPC	______	-	7121800	5344600	1591900	656220	184540	728060	1.452838408	0.111593863
MEDP1333	C28H51NO7P	LysoPC 20:4(2n isomer)	_______ 20:4(2n__)	GP	_____	LPC	______	-	37234000	38268000	18834000	3005700	5309300	13544000	1.470359469	0.231714298
MEDP1334	C28H51NO7P	LysoPC 20:4	_______ 20:4	GP	_____	LPC	______	-	37234000	38268000	18834000	3005700	5309300	13544000	1.470359469	0.231714298
MEDP1335	C28H49NO7P	LysoPC 20:5	_______ 20:5	GP	_____	LPC	______	-	1135000	552850	416490	61150	60807	160690	1.549596977	0.134316223
MEDP1336	C28H49NO7P	LysoPC 20:5(2n isomer)	_______ 20:5(2n__)	GP	_____	LPC	______	-	1121300	546630	423330	60462	54863	155340	1.552846789	0.129426757
MEDP1341	C26H51NO7P	LysoPC 18:2(2n isomer1)	_______ 18:2(2n__1)	GP	_____	LPC	______	-	43150000	45488000	43903000	7975800	10403000	16768000	1.620690222	0.265176813
MEDP1343	C26H49NO7P	LysoPC 18:3(2n isomer3)	_______ 18:3(2n__3)	GP	_____	LPC	______	-	567700	414100	155160	14697	16914	31629	1.608121197	0.05562201
MEDP1345	C26H49NO7P	LysoPC 18:3(2n isomer2)	_______ 18:3(2n__2)	GP	_____	LPC	______	-	1941600	473210	188150	81985	81680	105660	1.338618206	0.103468743
MEDP1348	C24H49NO7P	LysoPC 16:1(2n isomer)	_______ 16:1(2n__)	GP	_____	LPC	______	-	1562900	625710	318510	356900	133200	291980	1.133901511	0.311943585
MEDP1377	C27H51NO5	Carnitine C20:1-OH	__ C20:1-OH	FA	____	CAR	____	-	29283	21837	30032	4210.9	8628.1	11792	1.516417071	0.303516857
MEDP1385	C24H43NO6	Carnitine C17:1:DC	__ C17:1:DC	FA	____	CAR	____	-	16864	15246	37082	8294.4	9849.8	13735	1.250602495	0.460735345
MEDP1409	C19H37NO5	Carnitine C12-OH	__ C12-OH	FA	____	CAR	____	-	28271	39245	27115	11609	15193	16969	1.583176793	0.462543987
MEDP1410	C18H33NO6	Carnitine C11:DC	__ C11:DC	FA	____	CAR	____	-	20093	42050	33912	17359	13342	14578	1.418683597	0.471386185
MEDP1423	C14H25NO6	Carnitine C7:DC	__ C7:DC	FA	____	CAR	____	-	45001	195060	220470	58852	35017	47902	1.156988537	0.307842469
MEDP1429	C12H21NO6	Carnitine C5:DC	__ C5:DC	FA	____	CAR	____	-	573970	768800	788730	131780	277680	264420	1.56378877	0.316152944
MEDP1449	C25H38O4	MG(22:6)isomer	MG(22:6) ___	GL	____	MG	____	-	131080	181820	43887	15135	13903	11024	1.546662867	0.112285481
MEDP1450	C25H38O4	MG(22:6)	MG(22:6)	GL	____	MG	____	-	131080	181820	43887	15135	13903	11024	1.546662867	0.112285481
MEDP1451	C25H40O4	MG(22:5) isomer	MG(22:5) ___	GL	____	MG	____	-	19199	21982	9195.6	3569.8	3070.8	2360.6	1.577799736	0.178678196
MEDP1452	C25H40O4	MG(22:5)	MG(22:5)	GL	____	MG	____	-	19199	21982	9195.6	3569.8	3070.8	2360.6	1.577799736	0.178678196
MEDP1457	C24H30O3	Tetracosaenoic acid	_______	FA	____	FFA	_____	-	13621	30758	5301.4	6229.1	6858	4566	1.060064441	0.35533329
MEDP1466	C5H8N2O5	N-Carbamoyl-L-aspartate	N-_____L-____	Amino acid and Its metabolomics	_______	Amino acid derivatives	_____	13184-27-5	61525	25564	29757	13220	17770	25516	1.208438164	0.48359379
MEDP1490	C5H9NO3	Cis-4-Hydroxy-D-Proline	__4-__-D-___	Amino acid and Its metabolomics	_______	Amino acid derivatives	_____	2584-71-6	1845100	654800	1697100	458680	689750	841590	1.101449556	0.474152966
LIPID-N-000	C24H40O4	Chenodeoxycholic acid	_____	FA	____	BA	___	474-25-9	121250	47687	38086	25554	26356	39857	1.132597937	0.443269588
LIPID-N-002	C20H30O2	FFA(20:5)	_____FFA(20:5)	FA	____	Eicosanoid	_____	10417-94-4	1117400	549880	582360	509770	197450	308900	1.217307675	0.451681158
LIPID-N-005	C20H34O5	5-iso PGF2VI	_____5-iso PGF2VI	FA	____	Eicosanoid	____	-	4816.5	10806	9849.7	66278	19928	40805	1.461788873	4.98625953
LIPID-N-008	C20H38O2	FFA(20:1)	_____	FA	____	FFA	_____	-	1996800	1844800	1488100	946130	508360	809460	1.509109494	0.424780006
LIPID-N-008	C16H28O2	FFA(16:2)	______	FA	____	FFA	_____	-	283130	606710	482060	95973	252660	215400	1.351530229	0.411132736
LIPID-N-009	C18H32O2	FFA(18:2)	___	FA	____	FFA	_____	-	9707100	9701200	11550000	3753800	4772100	4793000	1.637312096	0.430220652
LIPID-N-009	C20H36O2	FFA(20:2)	______	FA	____	FFA	_____	-	1841200	2163600	1578000	931710	589780	615280	1.568071293	0.382741635
LIPID-N-009	C20H34O2	FFA(20:3)	______	FA	____	FFA	_____	-	1509300	2010800	1425400	749490	398010	462980	1.555875935	0.325645536
LIPID-N-009	C22H38O2	FFA(22:3)	_______	FA	____	FFA	_____	-	234260	301970	218560	210370	71323	25478	1.130988753	0.406962201
LIPID-N-009	C20H32O2	FFA(20:4)	_____	FA	____	FFA	_____	-	16601000	10896000	5961300	4057700	2542400	3536000	1.442499991	0.302947251
LIPID-N-009	C22H36O2	FFA(22:4)	_______	FA	____	FFA	_____	-	4060400	5410700	3490500	2264000	1579800	1412400	1.534395468	0.405520923
LIPID-N-010	C22H34O2	FFA(22:5)	_______	FA	____	FFA	_____	-	3064400	3729100	2862400	774610	1033700	851330	1.658118495	0.275441958
LIPID-N-010	C24H38O2	FFA(24:5)	_______	FA	____	FFA	_____	-	1431600	1010700	921940	1135300	242200	207990	1.08635032	0.471277317
LIPID-N-010	C22H32O2	FFA(22:6)	_______	FA	____	FFA	_____	-	1780400	2136200	1232700	505170	549420	666370	1.611968531	0.334212417
LIPID-N-010	C23H48NO7P	LPC(0:0/16:0)	_______LPC(0:0/16:0)	GP	_____	LPC	______	-	2355200	1390900	991780	868580	528440	807350	1.284389104	0.465265055
LIPID-N-010	C24H50NO7P	LPC(16:0/0:0)	_______LPC(16:0/0:0)	GP	_____	LPC	______	-	16623000	8625500	5975500	3968300	2674900	3704200	1.405265848	0.331392519
LIPID-N-011	C24H48NO7P	LPC(16:1/0:0)	_______LPC(16:1/0:0)	GP	_____	LPC	______	-	2694600	1057600	865030	522020	435230	735160	1.26981772	0.366542278
LIPID-N-012	C26H52NO7P	LPC(0:0/18:1)	_______LPC(0:0/18:1)	GP	_____	LPC	______	-	11034000	11621000	6573700	1380900	2038900	4125500	1.528194676	0.258146958
LIPID-N-012	C26H50NO7P	LPC(18:2/0:0)	_______LPC(18:2/0:0)	GP	_____	LPC	______	-	30703000	21832000	13254000	2852500	3153500	4418600	1.610857689	0.158455061
LIPID-N-014	C21H44NO7P	LPE(16:0/0:0)	________LPE(16:0/0:0)	GP	_____	LPE	_______	-	16752000	4716600	4421000	3145900	1499900	1850500	1.274542188	0.250923151
LIPID-N-014	C23H48NO7P	LPE(0:0/18:0)	________LPE(0:0/18:0)	GP	_____	LPE	_______	-	11149000	4516100	3176300	2582200	1675400	2052500	1.272484919	0.334906111
LIPID-N-015	C23H44NO7P	LPE(18:2/0:0)	________LPE(18:2/0:0)	GP	_____	LPE	_______	-	5582100	3156500	2310100	1299800	777710	1003700	1.490054545	0.278875343
LIPID-N-016	C25H44NO7P	LPE(20:4/0:0)	________LPE(20:4/0:0)	GP	_____	LPE	_______	-	13638000	9210700	4893800	1108400	2140600	4391100	1.391970575	0.27539335
LIPID-N-016	C27H48NO7P	LPE(22:4/0:0)	________LPE(22:4/0:0)	GP	_____	LPE	_______	-	308500	189900	134230	105400	66186	106100	1.345622544	0.438939032
LIPID-N-016	C27H46NO7P	LPE(22:5/0:0)	________LPE(22:5/0:0)	GP	_____	LPE	_______	-	587090	351490	338430	80811	107390	200010	1.505375036	0.303999969
LIPID-N-016	C27H44NO7P	LPE(22:6/0:0)	________LPE(22:6/0:0)	GP	_____	LPE	_______	-	1740300	1033900	801710	148650	233430	588790	1.433509673	0.271502918
LIPID-N-016	C24H49O9P	LPG(18:0/0:0)	_______LPG(18:0/0:0)	GP	_____	LPG	______	-	692920	317480	140780	158300	51236	97954	1.212151856	0.267108532
LIPID-N-017	C27H49O12P	LPI(18:2/0:0)	_______LPI(18:2/0:0)	GP	_____	LPI	______	-	334690	266030	1561800	123960	43469	58806	1.381320081	0.104616373

LIPID-N-018	C29H51O12P	LPI(20:3/0:0)	_______LPI(20:3/0:0)	GP	____	LPI	______	-	36497	25153	201740	19750	4651.9	9	1.008218787	0.092679677
LIPID-N-018	C29H49O12P	LPI(20:4/0:0)	_______LPI(20:4/0:0)	GP	____	LPI	______	-	351780	331530	1340600	188260	82962	168800	1.302383572	0.217411841
LIPID-N-018	C24H48NO9P	LPS(18:0/0:0)	________LPS(18:0/0:0)	GP	____	LPS	_______	119786-67-3	740910	420090	216060	144310	145810	191750	1.327227069	0.349926655
LIPID-N-018	C26H52NO9P	LPS(20:0/0:0)	________LPS(20:0/0:0)	GP	____	LPS	_______	-	83483	84644	26292	31817	23199	34508	1.086153947	0.460469399
LIPID-N-019	C26H50NO9P	LPS(20:1/0:0)	________LPS(20:1/0:0)	GP	____	LPS	_______	-	100250	65827	38114	22849	32497	41532	1.249502667	0.474447943
LIPID-N-020	C28H46NO9P	LPS(22:5/0:0)	________LPS(22:5/0:0)	GP	____	LPS	_______	-	91812	75123	81191	40987	5803.1	15921	1.354707282	0.252738931
LIPID-N-020	C28H44NO9P	LPS(22:6/0:0)	________LPS(22:6/0:0)	GP	____	LPS	_______	-	546450	279370	283410	178400	123350	138950	1.430287581	0.397302633
LIPID-N-022	C40H76NO8P	PC(16:1_16:1)	_____PC(16:1_16:1)	GP	____	PC	____	-	285150	38441	140290	898360	294160	407390	1.273387871	3.448966438
LIPID-N-023	C42H80NO8P	PC(16:0_18:2)	_____PC(16:0_18:2)	GP	____	PC	____	-	8194800	8306300	9601100	3369200	5129400	3981300	1.584303222	0.478116787
LIPID-N-024	C46H88NO8P	PC(18:2_20:0)	_____PC(18:2_20:0)	GP	____	PC	____	-	90522	106720	284160	94941	46346	67286	1.086296418	0.433261598
LIPID-N-032	C48H80NO8P	PC(18:2_22:6)	_____PC(18:2_22:6)	GP	____	PC	____	-	608620	440650	994100	320930	307620	268050	1.375685825	0.438784948
LIPID-N-039	C45H82NO8P	PE(20:4_20:0)	______PE(20:4_20:0)	GP	____	PE	_____	-	361520	960090	1289900	138040	127280	337730	1.413216422	0.230920042
LIPID-N-039	C41H72NO8P	PE(16:0_20:5)	______PE(16:0_20:5)	GP	____	PE	_____	-	328380	131490	348050	106910	56189	175590	1.146603742	0.419211061
LIPID-N-040	C43H76NO8P	PE(18:0_20:5)	______PE(18:0_20:5)	GP	____	PE	_____	-	2032500	1552600	1859500	708600	614760	823090	1.61881406	0.394234655
LIPID-N-040	C43H76NO8P	PE(16:0_22:5)	______PE(16:0_22:5)	GP	____	PE	_____	-	1059200	697630	1187100	474280	509840	362490	1.454062715	0.457419164
LIPID-N-041	C44H76NO8P	PE(17:0_22:6)	______PE(17:0_22:6)	GP	____	PE	_____	-	272920	269700	165160	58558	104960	115480	1.494475448	0.394187459
LIPID-N-046	C46H79O10P	PG(18:0_22:6)	_____PG(18:0_22:6)	GP	____	PG	____	-	5813.8	6696.5	2385.9	80252	37603	4911.7	1.192625031	8.241477692
LIPID-N-047	C44H83O13P	PI(18:0_17:1)	_____PI(18:0_17:1)	GP	____	PI	____	-	121400	61946	125850	36576	20117	9635.3	1.411937323	0.214518622
LIPID-N-050	C47H87O13P	PI(18:0_20:2)	_____PI(18:0_20:2)	GP	____	PI	____	-	202200	131420	134910	64196	75225	14934	1.245440564	0.329445286
LIPID-N-051	C47H85O13P	PI(20:3_18:0)	_____PI(20:3_18:0)	GP	____	PI	____	-	884580	1167500	1378900	742620	409430	170470	1.252501918	0.385464211
LIPID-N-055	C49H83O13P	PI(18:0_22:6)	_____PI(18:0_22:6)	GP	____	PI	____	-	90410	148090	199210	46338	61654	94354	1.340755176	0.462283247
LIPID-N-057	C42H80NO10P	PS(18:0_18:1)	______PS(18:0_18:1)	GP	____	PS	_____	-	1505900	1329600	1672100	6375500	2538000	1616600	1.144449715	2.336076848
LIPID-N-057	C43H80NO10P	PS(18:1_19:1)	______PS(18:1_19:1)	GP	____	PS	_____	-	31537	19866	73455	215410	42755	65208	1.020453307	2.589926156
LIPID-N-058	C42H76NO10P	PS(18:2_18:1)	______PS(18:2_18:1)	GP	____	PS	_____	-	866580	788160	1054200	319300	475590	330120	1.573047667	0.415295282
LIPID-N-060	C34H68NO6P	CerP(d18:1/16:0)	1-______CerP(d18:1/16:0)	SL	___	CerP	1-_____	-	50387	45922	71258	263250	57003	105090	1.102055611	2.538345856
LIPID-N-060	C36H70NO6P	CerP(d18:1/18:1)	1-______CerP(d18:1/18:1)	SL	___	CerP	1-_____	-	960800	846870	605440	3706900	996590	1221000	1.132649745	2.455126372
LIPID-N-061	C19H39O7P	LPA(0:0/16:0)	_____LPA(0:0/16:0)	GP	____	LPA	____	-	97956	32806	102020	28985	10414	35324	1.195649778	0.320999905
LIPID-N-061	C21H39O7P	LPA(0:0/18:2)	_____LPA(0:0/18:2)	GP	____	LPA	____	-	85308	72270	82669	39094	16420	3650	1.268435388	0.246263221
LIPID-N-061	C23H39O7P	LPA(0:0/20:4)	_____LPA(0:0/20:4)	GP	____	LPA	____	-	36403	34144	90584	20689	8058.9	21531	1.300573495	0.312037411
LIPID-N-062	C35H69O8P	PA(16:0_16:0)	___PA(16:0_16:0)	GP	____	PA	__	-	60214	15257	80997	352340	165970	62117	1.201220169	3.709557226
LIPID-N-063	C39H69O8P	PA(18:2_18:2)	___PA(18:2_18:2)	GP	____	PA	__	-	19398	9709.5	24568	117420	73931	11301	1.006346181	3.77550279
LIPID-P-000	C11H21NO5	Carnitine C4-OH	__ C4-OH	FA	___	CAR	___	-	14188000	31580000	20679000	5788500	7098100	5771700	1.578917322	0.280799735
LIPID-P-000	C12H23NO5	Carnitine C5-OH	__ C5-OH	FA	___	CAR	___	-	1008600	907560	626790	126890	535330	450700	1.247540576	0.437649187
LIPID-P-002	C10H17NO6	Carnitine C3:1-2OH	__ C3:1-OH	FA	___	CAR	___	910825-21-7	12714000	27377000	19307000	5334500	6252000	5118500	1.58552767	0.281238426
LIPID-P-002	C11H19NO6	Carnitine C4:1-2OH	__ C4:1-OH	FA	___	CAR	___	-	1136600	998320	694730	149500	626640	493550	1.237757373	0.448709204
LIPID-P-002	C12H21NO4	Carnitine C5:1	__ C5:1	FA	___	CAR	___	64681-36-3	123490	266270	173470	30923	134780	115780	1.112805303	0.499765637
LIPID-P-003	C15H27NO4	Carnitine C8:1	__ C8:1	FA	___	CAR	___	-	6010.8	23932	32921	100720	92348	20253	1.095284739	3.393383792
LIPID-P-004	C29H55NO4	Carnitine C22:1	__ C22:1	FA	___	CAR	___	-	395170	115960	85278	53423	45861	47058	1.284089826	0.245372295
LIPID-P-004	C21H37NO4	Carnitine C14:2	__ C14:2	FA	___	CAR	___	-	160020	196590	783750	103440	167840	113100	1.036985338	0.337068996
LIPID-P-004	C23H41NO4	Carnitine C16:2	__ C16:2	FA	___	CAR	___	1911579-97-9	327250	287530	1004400	177510	154340	187070	1.265989936	0.320483208
LIPID-P-004	C25H45NO4	Carnitine C18:2	__ C18:2	FA	___	CAR	___	36816-10-1	2924800	2012100	5105000	644850	623140	667560	1.563400634	0.192747388
LIPID-P-006	C45H78O2	CE(18:1)	____CE(18:1)	SL	__	CE	___	303-43-5	8396	2267.7	3854.5	72021	23162	11530	1.425761521	7.350291358
LIPID-P-012	C36H71NO3	Cer(d16:1/20:0)	____Cer(d16:1/20:0)	SL	__	Cer	___	-	1733.1	5018.4	2847.3	12023	6740.3	10206	1.409320699	3.018012668
LIPID-P-013	C34H65NO3	Cer(d18:2/16:0)	____Cer(d18:2/16:0)	SL	__	Cer	___	-	31184	16887	21254	75416	55588	70242	1.590348435	2.902935449
LIPID-P-015	C34H66NO6P	CerP(d18:1/16:1)	1-______CerP(d18:1/16:1)	SL	__	CerP	1-_____	-	28606	14695	13574	116690	40289	62921	1.470603797	3.866373626
LIPID-P-017	C37H72O5	DG(16:0_18:0)	____DG(16:0_18:0)	GL	___	DG	___	-	1789700	2529200	980110	406130	758130	1275600	1.216114149	0.46043695
LIPID-P-018	C40H78O5	DG(21:0_16:0)	____DG(21:0_16:0)	GL	___	DG	___	-	76027	48712	27339	367370	133410	1645600	1.338894369	14.11367851
LIPID-P-020	C39H74O5	DG(16:0_20:1)	____DG(16:0_20:1)	GL	___	DG	___	-	37911	39068	58291	19840	15558	12011	1.532996978	0.350476824
LIPID-P-020	C39H74O5	DG(18:0_18:1)	____DG(18:0_18:1)	GL	___	DG	___	-	990070	1255600	1036900	179990	254830	419790	1.60517969	0.260347837
LIPID-P-021	C35H64O5	DG(16:1_16:1)	____DG(16:1_16:1)	GL	___	DG	___	-	23106	4541.5	15067	45452	17340	156490	1.128137669	5.133666554
LIPID-P-021	C39H72O5	DG(18:0_18:2)	____DG(18:0_18:2)	GL	___	DG	___	-	977780	1344000	859720	289960	504300	407880	1.57191702	0.377853214
LIPID-P-022	C41H76O5	DG(18:0_20:2)	____DG(18:0_20:2)	GL	___	DG	___	-	14733	4503.1	10074	696.71	3337.4	2095.5	1.367406636	0.209129617
LIPID-P-023	C41H74O5	DG(18:2_20:1)	____DG(18:2_20:1)	GL	___	DG	___	-	48022	60825	79557	17383	37841	31038	1.413129988	0.457856521
LIPID-P-023	C41H74O5	DG(18:1_20:2)	____DG(18:1_20:2)	GL	___	DG	___	-	40013	46594	73578	21900	14066	33764	1.366290417	0.435309174
LIPID-P-023	C37H64O5	DG(16:1_18:3)	____DG(16:1_18:3)	GL	___	DG	___	-	9	9	9	13670	7043.9	51285	1.644511183	2666.625926
LIPID-P-025	C41H70O5	DG(16:0_22:5)	____DG(16:0_22:5)	GL	___	DG	___	-	39205	52807	117370	9617.2	47575	22370	1.161465141	0.379985863
LIPID-P-025	C43H74O5	DG(18:0_22:5)	____DG(18:0_22:5)	GL	___	DG	___	-	21395	47734	28391	7180.5	24978	5816.2	1.24455022	0.389404225
LIPID-P-025	C43H74O5	DG(18:1_22:4)	____DG(18:1_22:4)	GL	___	DG	___	-	41932	123290	75561	21079	33707	37335	1.365333628	0.382589302
LIPID-P-026	C41H68O5	DG(16:0_22:6)	____DG(16:0_22:6)	GL	___	DG	___	-	160090	706670	483500	108420	187720	181600	1.198531923	0.35381334
LIPID-P-026	C41H68O5	DG(16:1_22:5)	____DG(16:1_22:5)	GL	___	DG	___	-	16601	44933	43190	6761.3	18745	18665	1.19616933	0.421787747
LIPID-P-026	C43H72O5	DG(18:2_22:4)	____DG(18:2_22:4)	GL	___	DG	___	-	9837.9	92053	43188	5808.1	21741	11883	1.032909699	0.271797622
LIPID-P-026	C43H72O5	DG(18:1_22:5)	____DG(18:1_22:5)	GL	___	DG	___	-	37290	80731	125970	19315	31615	21945	1.388159873	0.29867905
LIPID-P-026	C43H72O5	DG(18:0_22:6)	____DG(18:0_22:6)	GL	___	DG	___	-	126990	276490	179000	61177	77963	54220	1.535349948	0.331959896
LIPID-P-026	C43H70O5	DG(18:1_22:6)	____DG(18:1_22:6)	GL	___	DG	___	-	232990	1001400	763250	169850	126900	182680	1.383238086	0.239998198
LIPID-P-027	C43H68O5	DG(18:2_22:6)	____DG(18:2_22:6)	GL	___	DG	___	-	89641	424160	329340	28479	185700	111330	1.013623647	0.386067099
LIPID-P-028	C21H40O4	MG(18:1)	____MG(18:1)	GL	___	MG	___	-	11653	6460.4	14158	2776.9	1669.3	4199.4	1.466080821	0.267902849
LIPID-P-028	C21H38O4	MG(18:2)	____MG(18:2)	GL	___	MG	___	-	1943.1	919.3	4124.5	697.56	464.61	549.15	1.311989374	0.24493266
LIPID-P-032	C52H97NO18	Hex3Cer(d18:1/16:0)	___Hex3Cer(d18:1/16:0)	SL	__	HexCer	__	-	25843	8484.9	4423.7	7171.2	1198.7	3451.6	1.012608311	0.305058372
LIPID-P-035	C22H46NO7P	LPC(14:0/0:0)	_______LPC(14:0/0:0)	GP	____	LPC	______	20559-16-4	1532400	472210	411480	377440	172730	245590	1.161827148	0.329358592
LIPID-P-035	C23H48NO7P	LPC(0:0/15:0)	_______LPC(0:0/15:0)	GP	____	LPC	______	-	217430	103700	88180	106100	47163	48250	1.075170419	0.492323667
LIPID-P-035	C23H48NO7P	LPC(15:0/0:0)	_______LPC(15:0/0:0)	GP	____	LPC	______	-	465650	217120	159370	219020	60620	92191	1.053906939	0.441531099
LIPID-P-035	C25H52NO7P	LPC(17:0/0:0)	_______LPC(17:0/0:0)	GP	____	LPC	______	50930-23-9	1891900	763400	459160	612260	251910	359610	1.050935773	0.392934891
LIPID-P-036	C23H46NO7P	LPC(15:1/0:0)	_______LPC(15:1/0:0)	GP	____	LPC	______	-	73728	27536	29338	8594.9	4676.9	22561	1.316330412	0.274366396
LIPID-P-036	C25H50NO7P	LPC(17:1/0:0)	_______LPC(17:1/0:0)	GP	____	LPC	______	-	523590	304810	192030	72364	61777	117480	1.500091205	0.246583303
LIPID-P-037	C26H52NO7P	LPC(18:1/0:0)	_______LPC(18:1/0:0)	GP	____	LPC	______	-	6887200	6528400	3851800	745330	1087300	2249900	1.538401445	0.23642992
LIPID-P-037	C27H54NO7P	LPC(19:1/0:0)	_______LPC(19:1/0:0)	GP	____	LPC	______	-	344670	190530	118090	34221	34664	51285	1.551950854	0.183945874
LIPID-P-037	C28H56NO7P	LPC(0:0/20:1)	_______LPC(0:0/20:1)	GP	____	LPC	______	-	1032000	447370	320940	104240	72292	129460	1.515797308	0.169966284
LIPID-P-037	C30H60NO7P	LPC(0:0/22:1)	_______LPC(0:0/22:1)	GP	____	LPC	______	-	18006	6976.5	6544.9	7232.4	1628.4	3545.1	1.090791379	0.393495816
LIPID-P-037	C26H50NO7P	LPC(0:0/18:2)	_______LPC(0:0/18:2)	GP	____	LPC	______	-	22104000	14307000	7831300	828700	1289600	2006000	1.60374454	0.093220741
LIPID-P-038	C28H54NO7P	LPC(0:0/20:2)	_______LPC(0:0/20:2)	GP	____	LPC	______	-	3854900	1847400	999730	318870	228840	462470	1.506017501	0.150727466
LIPID-P-038	C28H54NO7P	LPC(20:2/0:0)	_______LPC(20:2/0:0)	GP	____	LPC	______	-	3950900	2054200	980140	292280	248820	426060	1.523135206	0.138457662
LIPID-P-038	C26H48NO7P	LPC(18:3/0:0)	_______LPC(18:3/0:0)	GP	____	LPC	______	-	9328600	2605100	1931500	531390	519710	757930	1.459488332	0.130472694
LIPID-P-038	C28H52NO7P	LPC(20:3/0:0)	_______LPC(20:3/0:0)	GP	____	LPC	______	-	19311000	11861000	8974400	1375800	1132400	2783500	1.593937113	0.131810075
LIPID-P-038	C26H46NO7P	LPC(18:4/0:0)	_______LPC(18:4/0:0)	GP	____	LPC	______	-	55856	9149.4	7505.2	3185.7	4753.3	3511.1	1.216720072	0.157909326
LIPID-P-038	C28H50NO7P	LPC(20:4/0:0)	_______LPC(20:4/0:0)	GP	____	LPC	______	60701-99-7	3568000	3579500	2022400	253400	620200	1607500	1.401885011	0.270570017
LIPID-P-038	C30H54NO7P	LPC(0:0/22:4)	_______LPC(0:0/22:4)	GP	____	LPC	______	-	4971000	3134300	1274900	549540	521050	716760	1.483686047	0.190544978
LIPID-P-038	C30H54NO7P	LPC(22:4/0:0)	_______LPC(22:4/0:0)	GP	____	LPC	______	-	5243100	3389600	1281700	740370	520790	754260	1.442912111	0.203282095
LIPID-P-038	C28H48NO7P	LPC(20:5/0:0)	_______LPC(20:5/0:0)	GP	____	LPC	______	-	3791700	1614700	1920700	216570	244880	644870	1.540757131	0.15099016
LIPID-P-039	C30H52NO7P	LPC(0:0/22:5)	_______LPC(0:0/22:5)	GP	____	LPC	______	-	1183200	982970	631060	99927	141170	344640	1.534127376	0.209398941
LIPID-P-039	C30H52NO7P	LPC(22:5/0:0)	_______LPC(22:5/0:0)	GP	____	LPC	______	-	11657000	9463400	4316400	457520	1262400	2041900	1.512564645	0.147888885
LIPID-P-039	C30H50NO7P	LPC(22:6/0:0)	_______LPC(22:6/0:0)	GP	____	LPC	______	-	10865000	8565500	5690200	982750	1441400	2734200	1.565358499	0.205342606
LIPID-P-039	C24H50NO6P	LPC(O-16:1)	_______LPC(O-16:1)	GP	____	LPC-O	______	-	46381000	21662000	13606000	7448500	7027900	9926800	1.383377873	0.298879349
LIPID-P-041	C23H48NO7P	LPE(18:0/0:0)	________LPE(18:0/0:0)	GP	____	LPE	_______	-	45144000	14731000	9444100	7379900	4402300	5761000	1.263136291	0.253078877
LIPID-P-041	C25H52NO7P	LPE(20:0/0:0)	________LPE(20:0/0:0)	GP	____	LPE	_______	-	57543	47690	41943	21639	5455.7	10774	1.44744041	0.257302142
LIPID-P-041	C27H56NO7P	LPE(22:0/0:0)	________LPE(22:0/0:0)	GP	____	LPE	_______	-	26935	37391	17274	20657	2680.2	8797.8	1.136148751	0.393811275
LIPID-P-042	C23H44NO7P	LPE(0:0/18:2)	________LPE(0:0/18:2)	GP	____	LPE	_______	-	4573300	2443600	1610200	500120	407390	560600	1.564255676	0.170174218
LIPID-P-042	C25H48NO7P	LPE(0:0/20:2)	________LPE(0:0/20:2)	GP	____	LPE	_______	-	60334	21978	18452	14516	11682	8751	1.235728256	0.346840141
LIPID-P-042	C25H46NO7P	LPE(0:0/20:3)	________LPE(0:0/20:3)	GP	____	LPE	_______	-	315390	204410	172200	76054	34905	99179	1.449881097	0.30366763
LIPID-P-043	C27H50NO7P	LPE(20:3/0:0)	________LPE(20:3/0:0)	GP	____	LPE	_______	-	336470	198980	183440	75982	34441	95481	1.458585812	0.286419341
LIPID-P-043	C25H42NO7P	LPE(20:5/0:0)	________LPE(20:5/0:0)	GP	____	LPE	_______	-	196820	53233	73654	5976.4	8761.1	28384	1.456217378	0.133211515
LIPID-P-043	C27H46NO7P	LPE(0:0/22:5)	________LPE(0:0/22:5)	GP	____	LPE	_______	-	1122600	839490	549140	113820	143190	306780	1.541901999	0.224507512
LIPID-P-043	C21H44NO6P	LPE(P-16:0)	________LPE(P-16:0)	GP	____	LPE-P	_______	174062-72-7	27466000	9078300	6865200	5370500	2873100	3762700	1.267694637	0.276582315
LIPID-P-044	C23H48NO6P	LPE(P-18:0)	________LPE(P-18:0)	GP	____	LPE-P	_______	174062-73-8	8296600	3854300	2007900	2679600	1262800	1455400	1.097446658	0.381232873
LIPID-P-044	C22H44NO6P	LPE(P-17:1)	________LPE(P-17:1)	GP	____	LPE-P	_______	-	12972	4353.9	3253.4	1550.5	993.37	2090.6	1.363827484	0.225200566
LIPID-P-044	C23H46NO6P	LPE(P-18:1)	________LPE(P-18:1)	GP	____	LPE-P	_______	-	6822100	2562000	2071500	1704100	1099300	1402600	1.224199938	0.367156674
LIPID-P-044	C22H45O9P	LPG(16:0/0:0)	_______LPG(16:0/0:0)	GP	____	LPG	______	-	588670	378150	176570	31564	53086	156230	1.40839739	0.210671774
LIPID-P-045	C22H44NO9P	LPS(16:0/0:0)	________LPS(16:0/0:0)	GP	____	LPS	_______	116947-34-3	64970	30930	22101	14584	10939	14820	1.371975099	0.341886933
LIPID-P-051	C44H78NO8P	PC(16:0_20:5)	_____PC(16:0_20:5)	GP	____	PC	____	-	8979400	6288600	13618000	4408300	3927400	4629600	1.402049182	0.448843731
LIPID-P-053	C40H80NO7P	PC(O-16:0_16:1)	_____PC(O-16:0_16:1)	GP	____	PC-O	____	-	2974500	1964500	1664300	7256600	3509800	2910200	1.256370737	2.071176533
LIPID-P-054	C42H82NO7P	PC(O-16:0_18:2)	_____PC(O-16:0_18:2)	GP	____	PC-O	____	88542-95-4	125440000(	116490000(	97037000	31121000	56431000	38564000	1.578279601	0.372059817
LIPID-P-054	C46H90NO7P	PC(O-18:1_20:1)	_____PC(O-18:1_20:1)	GP	____	PC-O	____	-	337730	205490	107290	708420	388490	328500	1.228165377	2.191219197
LIPID-P-054	C42H80NO7P	PC(O-16:1_18:2)	_____PC(O-16:1_18:2)	GP	____	PC-O	____	-	760500000(	726150000(	686710000(	183630000(	436160000(	323740000(	1.479752512	0.434134244
LIPID-P-055	C42H78NO7P	PC(O-14:0_20:4)	_____PC(O-14:0_20:4)	GP	____	PC-O	____	-	2507600	2047100	3360800	230210	962520	628880	1.478911157	0.230132019
LIPID-P-056	C44H78NO7P	PC(O-16:1_20:5)	_____PC(O-16:1_20:5)	GP	____	PC-O	____	-	14615000	10368000	18371000	6408100	6643700	7810700	1.480257355	0.481212806
LIPID-P-058	C39H74NO8P	PE(18:1_16:1)	______PE(18:1_16:1)	GP	____	PE	_____	-	48116000	23573000	38903000	20139000	15223000	10654000	1.34554275	0.416087963
LIPID-P-059	C39H72NO8P	PE(16:1_18:2)	______PE(16:1_18:2)	GP	____	PE	_____	-	181870	62366	130720	510080	161190	175590	1.106138221	2.258558338
LIPID-P-060	C44H80NO8P	PE(19:0_20:4)	______PE(19:0_20:4)	GP	____	PE	_____	-	1366300	1385000	1482000	494940	597280	728950	1.63533099	0.430201025
LIPID-P-061	C41H72NO8P	PE(16:1_20:4)	______PE(16:1_20:4)	GP	____	PE	_____	-	1456600	454400	1607500	432910	244690	314490	1.280465234	0.281963905
LIPID-P-061	C45H80NO8P	PE(18:0_22:5)	______PE(18:0_22:5)	GP	____	PE	_____	-	31889000	29035000	39629000	15864000	19324000	14351000	1.555220699	0.492665559
LIPID-P-061	C42H72NO8P	PE(15:0_22:6)	______PE(15:0_22:6)	GP	____	PE	_____	-	62655	37952	73827	41872	24353	20078	1.217665482	0.494760196
LIPID-P-062	C46H80NO8P	PE(19:0_22:6)	______PE(19:0_22:6)	GP	____	PE	_____	-	324890	287220	317330	122000	139880	170350	1.628422464	0.465043467
LIPID-P-062	C47H82NO8P	PE(22:6_20:0)	______PE(22:6_20:0)	GP	____	PE	_____	-	281070	437040	779410	126930	85690	203300	1.437826758	0.277739195
LIPID-P-063	C43H86NO7P	PE(P-18:0_20:0)	______PE(P-18:0_20:0)	GP	____	PE-P	_____	-	58240	42382	37371	157410	59504	81089	1.28453627	2.159551571
LIPID-P-069	C40H76NO10P	PS(16:0_18:1)	______PS(16:0_18:1)	GP	____	PS	_____	-	499930	148530	287760	4362000	969460	822420	1.398718105	6.573113157
LIPID-P-070	C46H86NO10P	PS(18:1_22:1)	______PS(18:1_22:1)	GP	____	PS	_____	-	181360	142030	176340	977080	436750	337090	1.499258652	3.503732015
LIPID-P-072	C37H77N2O6P	SM(d18:0/14:0)	___SM(d18:0/14:0)	SL	__	SM	__	-	208170	89610	72113	502650	209970	136610	1.098597673	2.29588016
LIPID-P-073	C39H81N2O6P	SM(d18:0/16:0)	___SM(d18:0/16:0)	SL	__	SM	__	-	383030	256360	251790	1100600	365570	398110	1.122163285	2.091923068
LIPID-P-073	C39H79N2O6P	SM(d18:1/16:0)	___SM(d18:1/16:0)	SL	__	SM	__	-	4973800	3612000	3488700	14177000	4628200	5459500	1.097157832	2.009582177
LIPID-P-074	C37H73N2O6P	SM(d18:2/14:0)	___SM(d18:2/14:0)	SL	__	SM	__	2292199-25-6	254110	95935	132270	665350	220570	172110	1.021083324	2.193649379
LIPID-P-075	C45H89N2O6P	SM(d18:1/22:1)	___SM(d18:1/22:1)	SL	__	SM	__	222403-68-1	1523400	1014600	532030	4086500	1910100	1434100	1.217578547	2.420399801
LIPID-P-075	C46H91N2O6P	SM(d18:1/23:1)	___SM(d18:1/23:1)	SL	__	SM	__	2124199-71-7	743880	474200	266490	1703600	867780	702850	1.221191936	2.205507319
LIPID-P-075	C48H95N2O6P	SM(d18:1/25:1)	___SM(d18:1/25:1)	SL	__	SM	__	-	62006	33918	20031	201620	59266	39587	1.003782481	2.591289724
LIPID-P-076	C49H97N2O6P	SM(d18:1/26:1)	___SM(d18:1/26:1)	SL	__	SM	__	-	183090	90585	62815	625240	171850	170490	1.167040454	2.87550893
LIPID-P-076	C39H74O6	TG(10:0_12:0_14:0)	____TG(10:0_12:0_14:0)	GL	___	TG	___	-	17972	16591	15215	186240	34318	15567	1.028247276	4.743561413
LIPID-P-077	C41H78O6	TG(8:0_14:0_16:0)	____TG(8:0_14:0_16:0)	GL	___	TG	___	-	8247	3734.7	17731	1685400	49603	108450	1.408008424	62.04259458
LIPID-P-077	C41H78O6	TG(10:0_16:0_12:0)	____TG(10:0_16:0_12:0)	GL	___	TG	___	-	94121	19120	14363	109010	512210	53805	1.143137737	5.289998746
LIPID-P-077	C43H82O6	TG(8:0_16:0_16:0)	____TG(8:0_16:0_16:0)	GL	___	TG	___	-	102720	18304	32466	3250900	1401000	506220	1.580349775	33.60557691
LIPID-P-077	C43H82O6	TG(10:0_14:0_16:0)	____TG(10:0_14:0_16:0)	GL	___	TG	___	-	110770	41072	25516	496650	705290	184240	1.484561534	7.815717363
LIPID-P-077	C43H82O6	TG(8:0_14:0_18:0)	____TG(8:0_14:0_18:0)	GL	___	TG	___	-	21148	9	7353.8	1886900	62688	183100	1.272317331	74.80281157

LIPID-P-077	C45H86O6	TG(12:0_14:0_16:0)	____TG(12:0_14:0_16:0)	GL	___	TG	___	-	176090	22394	61129	1397400	5295100	1265800	1.558352117	30.65447416
LIPID-P-077	C45H86O6	TG(12:0_12:0_18:0)	____TG(12:0_12:0_18:0)	GL	___	TG	___	-	35907	2989.8	15153	206540	244860	90136	1.480954803	10.01920451
LIPID-P-078	C47H90O6	TG(14:0_14:0_16:0)	____TG(14:0_14:0_16:0)	GL	___	TG	___	-	618900	91252	283610	1195900	25657000	4054700	1.371312093	31.10161185
LIPID-P-078	C47H90O6	TG(12:0_14:0_18:0)	____TG(12:0_14:0_18:0)	GL	___	TG	___	-	57141	4241.3	14401	179630	915280	161050	1.442929761	16.57304446
LIPID-P-078	C47H90O6	TG(12:0_16:0_16:0)	____TG(12:0_16:0_16:0)	GL	___	TG	___	-	253240	31549	135880	476530	9477400	1665800	1.362226186	27.62202587
LIPID-P-078	C48H92O6	TG(14:0_15:0_16:0)	____TG(14:0_15:0_16:0)	GL	___	TG	___	-	35447	33304	26380	79187	651450	488030	1.400091388	12.8104088
LIPID-P-078	C49H94O6	TG(14:0_16:0_16:0)	____TG(14:0_16:0_16:0)	GL	___	TG	___	-	2353000	513760	2141100	6872900	17111000(	71284000	1.408464365	49.77513349
LIPID-P-078	C50H96O6	TG(15:0_16:0_16:0)	____TG(15:0_16:0_16:0)	GL	___	TG	___	-	215330	148900	144550	396200	14145000	10172000	1.310859121	48.57345021
LIPID-P-078	C51H98O6	TG(16:0_16:0_16:0)	____TG(16:0_16:0_16:0)	GL	___	TG	___	555-44-2	10871000	2485300	14389000	10855000	64447000(	51484000(	1.19082793	42.1752513
LIPID-P-078	C51H98O6	TG(14:0_16:0_18:0)	____TG(14:0_16:0_18:0)	GL	___	TG	___	-	829400	82208	1109300	616950	68008000	26695000	1.147331043	47.16689231
LIPID-P-079	C52H100O6	TG(16:0_16:0_17:0)	____TG(16:0_16:0_17:0)	GL	___	TG	___	-	54227	25346	101000	96213	14107000	5274500	1.229702402	107.8661428
LIPID-P-079	C53H102O6	TG(16:0_16:0_18:0)	____TG(16:0_16:0_18:0)	GL	___	TG	___	-	4311700	1816100	6288100	3340000	34869000(	21808000(	1.105244477	45.91773452
LIPID-P-079	C55H106O6	TG(16:0_18:0_18:0)	____TG(16:0_18:0_18:0)	GL	___	TG	___	-	640010	340570	672030	617430	62403000	64018000	1.162111618	76.87139131
LIPID-P-079	C55H106O6	TG(14:0_18:0_20:0)	____TG(14:0_18:0_20:0)	GL	___	TG	___	-	24063	10435	53817	30426	6364900	6368600	1.162853092	144.5272717
LIPID-P-080	C56H108O6	TG(17:0_18:0_18:0)	____TG(17:0_18:0_18:0)	GL	___	TG	___	-	20994	12606	18241	14161	734420	284520	1.073235467	19.92826141
LIPID-P-080	C57H110O6	TG(18:0_18:0_18:0)	____TG(18:0_18:0_18:0)	GL	___	TG	___	-	229770	148670	273330	170590	12065000	10359000	1.08326167	34.66650812
LIPID-P-080	C59H114O6	TG(16:0_18:0_22:0)	____TG(16:0_18:0_22:0)	GL	___	TG	___	-	6309.7	3601.7	9660.1	3985	242880	308790	1.024636993	28.39102777
LIPID-P-081	C41H76O6	TG(10:0_14:0_14:1)	____TG(10:0_14:0_14:1)	GL	___	TG	___	-	9	9	9	430820	19205	133140	1.648100363	21598.7037
LIPID-P-081	C43H80O6	TG(8:0_16:0_16:1)	____TG(8:0_16:0_16:1)	GL	___	TG	___	-	60410	6288.9	84123	4153100	502840	3077100	1.51620084	51.27266
LIPID-P-082	C43H80O6	TG(8:0_14:0_18:1)	____TG(8:0_14:0_18:1)	GL	___	TG	___	-	13764	9	16198	973970	101090	253810	1.276355857	44.33852724
LIPID-P-082	C43H80O6	TG(10:0_12:0_18:1)	____TG(10:0_12:0_18:1)	GL	___	TG	___	-	14309	9	7198.1	407320	189950	77158	1.291648884	31.34527168
LIPID-P-082	C45H84O6	TG(8:0_16:0_18:1)	____TG(8:0_16:0_18:1)	GL	___	TG	___	-	170320	13245	28679	6740700	2078700	1402800	1.57512345	48.16249223
LIPID-P-082	C45H84O6	TG(12:0_14:0_16:1)	____TG(12:0_14:0_16:1)	GL	___	TG	___	-	60228	9	34569	666490	907570	361730	1.207114665	20.41843343
LIPID-P-082	C45H84O6	TG(10:0_16:0_16:1)	____TG(10:0_16:0_16:1)	GL	___	TG	___	-	35999	2680.7	26885	1935100	582160	512480	1.566046938	46.20992699
LIPID-P-082	C46H86O6	TG(12:0_15:0_16:1)	____TG(12:0_15:0_16:1)	GL	___	TG	___	-	10330	4848.9	4881.7	25807	91830	16954	1.410508608	6.70922106
LIPID-P-082	C47H88O6	TG(12:0_16:0_16:1)	____TG(12:0_16:0_16:1)	GL	___	TG	___	-	134740	20649	80482	695620	3252900	1574300	1.539600959	23.41457831
LIPID-P-082	C47H88O6	TG(10:0_16:0_18:1)	____TG(10:0_16:0_18:1)	GL	___	TG	___	-	112780	8207.1	109610	3965800	2718200	4144900	1.576429947	46.96026099
LIPID-P-082	C47H88O6	TG(12:0_14:0_18:1)	____TG(12:0_14:0_18:1)	GL	___	TG	___	-	204030	54585	113020	821930	3716600	1888300	1.548939245	17.29339271
LIPID-P-083	C48H90O6	TG(12:0_15:0_18:1)	____TG(12:0_15:0_18:1)	GL	___	TG	___	-	23364	9969.8	23264	96261	261320	159130	1.576703749	9.129524469
LIPID-P-083	C48H90O6	TG(14:0_15:0_16:1)	____TG(14:0_15:0_16:1)	GL	___	TG	___	-	54059	89912	74417	128000	326040	260680	1.417484987	3.272707292
LIPID-P-083	C49H92O6	TG(12:0_16:0_18:1)	____TG(12:0_16:0_18:1)	GL	___	TG	___	-	1042100	33379	590570	2489200	39069000	9804900	1.362086038	30.82928533
LIPID-P-083	C49H92O6	TG(14:0_16:0_16:1)	____TG(14:0_16:0_16:1)	GL	___	TG	___	-	524810	65752	610490	3544200	27666000	22051000	1.496608554	44.34545715
LIPID-P-083	C50H94O6	TG(15:0_16:0_16:1)	____TG(15:0_16:0_16:1)	GL	___	TG	___	-	135480	70975	175570	285380	2448700	2806800	1.362970518	14.50397225
LIPID-P-083	C50H94O6	TG(14:0_15:0_18:1)	____TG(14:0_15:0_18:1)	GL	___	TG	___	-	80676	45866	97412	136850	1429300	804330	1.327146699	10.58467364
LIPID-P-083	C51H96O6	TG(16:0_16:0_16:1)	____TG(16:0_16:0_16:1)	GL	___	TG	___	-	6697100	608520	12526000	27543000	19015000(	17510000(	1.390331086	19.80640008
LIPID-P-083	C51H96O6	TG(14:0_16:0_18:1)	____TG(14:0_16:0_18:1)	GL	___	TG	___	-	2591200	341980	2876900	11913000	90480000	67068000	1.469973225	29.16672404
LIPID-P-084	C52H98O6	TG(15:0_16:0_18:1)	____TG(15:0_16:0_18:1)	GL	___	TG	___	-	211080	76839	284030	713390	13960000	9583200	1.40714271	42.41040722
LIPID-P-084	C53H100O6	TG(16:0_16:0_18:1)	____TG(16:0_16:0_18:1)	GL	___	TG	___	-	3966200	331050	4498900	6731100	16614000(	12845000(	1.305508388	34.2560211
LIPID-P-084	C54H102O6	TG(16:0_17:0_18:1)	____TG(16:0_17:0_18:1)	GL	___	TG	___	-	216200	60034	360060	501640	24898000	16775000	1.311397779	66.2816874
LIPID-P-084	C55H104O6	TG(16:0_18:0_18:1)	____TG(16:0_18:0_18:1)	GL	___	TG	___	-	7272500	970250	15446000	8538800	19466000(	16316000(	1.172098022	15.46551844
LIPID-P-084	C55H104O6	TG(16:0_16:1_20:0)	____TG(16:0_16:1_20:0)	GL	___	TG	___	-	33829	3211	114610	89918	1188600	1221500	1.257966729	16.48544675
LIPID-P-084	C56H106O6	TG(17:0_18:0_18:1)	____TG(17:0_18:0_18:1)	GL	___	TG	___	-	27458	15996	58416	38852	5255200	3075200	1.175614483	82.15619908
LIPID-P-085	C56H106O6	TG(17:0_17:0_19:1)	____TG(17:0_17:0_19:1)	GL	___	TG	___	-	3629.3	871.53	5703.9	4222.7	595030	410450	1.198162418	98.94457766
LIPID-P-085	C57H108O6	TG(16:0_18:0_20:1)	____TG(16:0_18:0_20:1)	GL	___	TG	___	-	44536	17745	49769	74901	5432800	3354300	1.274412614	79.08970103
LIPID-P-085	C57H108O6	TG(18:0_18:0_18:1)	____TG(18:0_18:0_18:1)	GL	___	TG	___	-	1571800	291830	1563200	2055400	79152000	83090000	1.268793581	47.94442677
LIPID-P-085	C57H108O6	TG(16:0_20:0_18:1)	____TG(16:0_20:0_18:1)	GL	___	TG	___	-	112130	26764	358390	218340	6606000	8711500	1.223505634	31.24138319
LIPID-P-085	C58H110O6	TG(16:0_18:1_21:0)	____TG(16:0_18:1_21:0)	GL	___	TG	___	-	7443.3	1388.6	6417.2	7922.3	196550	130560	1.25438806	21.97062777
LIPID-P-086	C61H116O6	TG(16:0_24:0_18:1)	____TG(16:0_24:0_18:1)	GL	___	TG	___	-	7853.2	8731.2	20496	11066	177000	331780	1.095141295	14.01942805
LIPID-P-087	C43H78O6	TG(8:0_14:0_18:2)	____TG(8:0_14:0_18:2)	GL	___	TG	___	-	3218.6	1171.2	3224.5	235830	27602	55624	1.576204059	41.90221031
LIPID-P-087	C43H78O6	TG(8:0_16:1_16:1)	____TG(8:0_16:1_16:1)	GL	___	TG	___	-	9	9	9	603660	27649	50493	1.651703564	25251.92593
LIPID-P-087	C43H78O6	TG(10:0_12:0_18:2)	____TG(10:0_12:0_18:2)	GL	___	TG	___	-	9131.3	9	4536.7	80049	98804	10481	1.177467941	13.84324048
LIPID-P-087	C45H82O6	TG(8:0_16:1_18:1)	____TG(8:0_16:1_18:1)	GL	___	TG	___	-	18758	9	10584	1467700	142970	418920	1.320239508	69.14892167
LIPID-P-088	C45H82O6	TG(8:0_16:0_18:2)	____TG(8:0_16:0_18:2)	GL	___	TG	___	-	75727	5717.3	64802	2214300	1333900	756080	1.552906456	29.43171896
LIPID-P-088	C46H84O6	TG(9:0_16:0_18:2)	____TG(9:0_16:0_18:2)	GL	___	TG	___	-	9538.6	3850.3	25021	10922	141580	150190	1.099037533	7.880572457
LIPID-P-088	C47H86O6	TG(14:0_14:1_16:1)	____TG(14:0_14:1_16:1)	GL	___	TG	___	-	95085	61510	57844	688840	1018900	1360900	1.634301516	14.31008352
LIPID-P-088	C47H86O6	TG(12:0_14:0_18:2)	____TG(12:0_14:0_18:2)	GL	___	TG	___	-	107210	13443	54268	300420	2319000	386990	1.421712838	17.18724453
LIPID-P-088	C47H86O6	TG(10:0_16:0_18:2)	____TG(10:0_16:0_18:2)	GL	___	TG	___	-	103870	11359	107470	1776600	3508600	1141400	1.559887877	28.85778562
LIPID-P-089	C48H88O6	TG(15:0_14:1_16:1)	____TG(15:0_14:1_16:1)	GL	___	TG	___	-	11477	7301.7	4612.1	41174	160330	39019	1.502050821	10.2828035
LIPID-P-089	C49H90O6	TG(14:1_16:0_16:1)	____TG(14:1_16:0_16:1)	GL	___	TG	___	-	622140	26201	346130	1460500	26367000	11936000	1.384313028	39.98457471
LIPID-P-089	C49H90O6	TG(12:0_16:0_18:2)	____TG(12:0_16:0_18:2)	GL	___	TG	___	-	507530	33589	412500	1153500	34434000	7629000	1.352348903	45.31841333
LIPID-P-089	C49H90O6	TG(14:0_14:1_18:1)	____TG(14:0_14:1_18:1)	GL	___	TG	___	-	448420	42482	299040	2379300	8868700	9056900	1.511925719	25.70429221
LIPID-P-089	C50H92O6	TG(14:0_15:0_18:2)	____TG(14:0_15:0_18:2)	GL	___	TG	___	-	12997	9291.2	30397	83667	1053300	478420	1.462503991	30.66111546
LIPID-P-089	C50H92O6	TG(15:0_16:1_16:1)	____TG(15:0_16:1_16:1)	GL	___	TG	___	-	50411	20456	66110	95859	790340	694970	1.356044912	11.54331749
LIPID-P-090	C50H92O6	TG(14:0_16:1_17:1)	____TG(14:0_16:1_17:1)	GL	___	TG	___	-	16690	7598.7	13292	87233	766270	593820	1.518405158	38.51240131
LIPID-P-090	C51H94O6	TG(16:0_16:1_16:1)	____TG(16:0_16:1_16:1)	GL	___	TG	___	-	2239000	246430	3643100	9361500	11533000(	86449000	1.410191017	34.45206273
LIPID-P-090	C51H94O6	TG(14:0_16:0_18:2)	____TG(14:0_16:0_18:2)	GL	___	TG	___	-	1527900	189420	3026100	4164800	69695000	48566000	1.334794	25.80960573
LIPID-P-090	C51H94O6	TG(14:0_16:1_18:1)	____TG(14:0_16:1_18:1)	GL	___	TG	___	-	1414600	237040	3070100	6729200	97268000	70734000	1.409144142	37.00568011
LIPID-P-090	C52H96O6	TG(16:0_16:1_17:1)	____TG(16:0_16:1_17:1)	GL	___	TG	___	-	76251	25393	77007	248490	2212700	3120600	1.438981025	31.24410163
LIPID-P-090	C52H96O6	TG(15:0_16:0_18:2)	____TG(15:0_16:0_18:2)	GL	___	TG	___	-	84482	12606	122340	252460	7298000	4185600	1.370280939	53.48478772
LIPID-P-090	C52H96O6	TG(15:0_16:1_18:1)	____TG(15:0_16:1_18:1)	GL	___	TG	___	-	109440	35336	138110	572540	8671400	5691900	1.470298493	52.79808828
LIPID-P-091	C53H98O6	TG(16:0_16:1_18:1)	____TG(16:0_16:1_18:1)	GL	___	TG	___	-	3979700	538750	7951900	8754000	22607000(	18888000(	1.298471464	33.97691324
LIPID-P-091	C53H98O6	TG(14:0_18:0_18:2)	____TG(14:0_18:0_18:2)	GL	___	TG	___	-	482590	24397	773830	2381100	19817000	19558000	1.399251558	32.60114443
LIPID-P-091	C54H100O6	TG(15:0_17:0_19:2)	____TG(15:0_17:0_19:2)	GL	___	TG	___	-	110640	30207	191310	693260	8486500	8773700	1.464432164	54.05112643
LIPID-P-091	C54H100O6	TG(16:0_17:1_18:1)	____TG(16:0_17:1_18:1)	GL	___	TG	___	-	195560	39176	252280	697320	15235000	16370000	1.400812034	66.32702006
LIPID-P-091	C55H102O6	TG(16:0_18:1_18:1)	____TG(16:0_18:1_18:1)	GL	___	TG	___	-	11859000	1113000	28744000	25428000	38197000(	37660000(	1.264758369	18.79370026
LIPID-P-091	C55H102O6	TG(16:0_18:0_18:2)	____TG(16:0_18:0_18:2)	GL	___	TG	___	-	4503600	946450	11580000	5922800	16038000(	15894000(	1.173656828	19.09817059
LIPID-P-091	C55H102O6	TG(16:0_16:1_20:1)	____TG(16:0_16:1_20:1)	GL	___	TG	___	-	41916000	4298900	68371000	68869000	71656000(	73579000(	1.262884218	13.27579571
LIPID-P-091	C55H102O6	TG(14:0_18:0_20:2)	____TG(14:0_18:0_20:2)	GL	___	TG	___	-	74566	11587	184010	335410	1460500	2062800	1.391882414	14.28289588
LIPID-P-092	C56H104O6	TG(17:0_18:1_18:1)	____TG(17:0_18:1_18:1)	GL	___	TG	___	-	129740	21794	410670	227190	11975000	22498000	1.205961703	61.721706
LIPID-P-092	C56H104O6	TG(16:0_18:1_19:1)	____TG(16:0_18:1_19:1)	GL	___	TG	___	-	46461	15493	103980	198760	8225700	8566100	1.363236493	102.3934817
LIPID-P-092	C57H106O6	TG(16:0_18:1_20:1)	____TG(16:0_18:1_20:1)	GL	___	TG	___	-	525480	94858	1987100	1586300	34059000	42176000	1.280181525	29.84588704
LIPID-P-092	C57H106O6	TG(16:1_18:0_20:1)	____TG(16:1_18:0_20:1)	GL	___	TG	___	-	693570	67383	2092400	1547200	42587000	45452000	1.252227162	31.39681631
LIPID-P-092	C57H106O6	TG(18:0_18:1_18:1)	____TG(18:0_18:1_18:1)	GL	___	TG	___	-	10069000	1491800	22869000	16252000	26701000(	25675000(	1.228457251	15.68443616
LIPID-P-092	C57H106O6	TG(16:0_16:1_22:1)	____TG(16:0_16:1_22:1)	GL	___	TG	___	-	1387.8	1332.4	10367	40663	328840	203350	1.506949451	43.77200624
LIPID-P-093	C58H108O6	TG(18:0_18:1_19:1)	____TG(18:0_18:1_19:1)	GL	___	TG	___	-	8069.4	1980.4	21670	11703	896130	798020	1.19098669	53.77880693
LIPID-P-093	C59H110O6	TG(18:0_18:1_20:1)	____TG(18:0_18:1_20:1)	GL	___	TG	___	-	78854	18937	142740	152890	4555200	7440100	1.275594155	50.50571444
LIPID-P-093	C59H110O6	TG(16:0_18:1_22:1)	____TG(16:0_18:1_22:1)	GL	___	TG	___	-	12570	3960	28854	51006	2372800	1213800	1.362139034	80.15172748
LIPID-P-093	C61H114O6	TG(22:0_18:1_18:1)	____TG(22:0_18:1_18:1)	GL	___	TG	___	-	11812	7365.8	55732	16258	280110	792570	1.036777108	14.53665609
LIPID-P-094	C61H114O6	TG(18:0_22:0_18:2)	____TG(18:0_22:0_18:2)	GL	___	TG	___	-	2765.4	542.37	1756.6	2723	184740	116880	1.246433294	60.09493777
LIPID-P-094	C61H114O6	TG(16:0_18:1_24:1)	____TG(16:0_18:1_24:1)	GL	___	TG	___	-	5091.4	596.33	3217.6	26273	293240	190930	1.496318162	57.31881918
LIPID-P-095	C45H80O6	TG(8:0_16:1_18:2)	____TG(8:0_16:1_18:2)	GL	___	TG	___	-	3841.1	3085.9	3544.4	350390	21022	120780	1.515703839	47.00345704
LIPID-P-095	C45H80O6	TG(10:0_14:0_18:3)	____TG(10:0_14:0_18:3)	GL	___	TG	___	-	9	9	9	85456	36660	38928	1.673529094	5964.592593
LIPID-P-095	C45H80O6	TG(8:0_16:0_18:3)	____TG(8:0_16:0_18:3)	GL	___	TG	___	-	9	9	9	274300	28098	60544	1.662658559	13442.2963
LIPID-P-095	C47H84O6	TG(14:1_14:1_16:1)	____TG(14:1_14:1_16:1)	GL	___	TG	___	-	34868	7582.7	5536.4	580930	274150	509370	1.595131104	28.43368322
LIPID-P-095	C47H84O6	TG(8:0_18:1_18:2)	____TG(8:0_18:1_18:2)	GL	___	TG	___	-	50229	6050.3	57446	1610700	855880	1180600	1.585686137	32.07008467
LIPID-P-095	C47H84O6	TG(10:0_16:0_18:3)	____TG(10:0_16:0_18:3)	GL	___	TG	___	-	29361	9	37967	1180500	712370	604470	1.278690727	37.08718832
LIPID-P-095	C48H86O6	TG(9:0_18:1_18:2)	____TG(9:0_18:1_18:2)	GL	___	TG	___	-	9	9	17411	20410	154500	117250	1.312963966	16.76286649
LIPID-P-095	C48H86O6	TG(14:1_15:1_16:1)	____TG(14:1_15:1_16:1)	GL	___	TG	___	-	11358	13748	10952	29909	34954	40631	1.613368835	2.925675301
LIPID-P-096	C49H88O6	TG(14:1_16:1_16:1)	____TG(14:1_16:1_16:1)	GL	___	TG	___	-	9	9	9	330980	549990	769730	1.66693882	61137.03704
LIPID-P-096	C49H88O6	TG(10:0_18:1_18:2)	____TG(10:0_18:1_18:2)	GL	___	TG	___	-	90413	16468	63521	2468300	2480300	2508900	1.636685979	43.7641577
LIPID-P-096	C49H88O6	TG(12:0_16:1_18:2)	____TG(12:0_16:1_18:2)	GL	___	TG	___	-	74797	3129.4	27547	287780	4129100	922990	1.446640528	50.6276464
LIPID-P-096	C51H92O6	TG(14:1_16:1_18:1)	____TG(14:1_16:1_18:1)	GL	___	TG	___	-	335170	164100	248040	2946400	14549000	33340000	1.538817181	68.02451459
LIPID-P-096	C51H92O6	TG(14:0_16:1_18:2)	____TG(14:0_16:1_18:2)	GL	___	TG	___	-	863300	72875	768240	3441500	48521000	20775000	1.439421233	42.6759328
LIPID-P-096	C51H92O6	TG(16:0_14:1_18:2)	____TG(16:0_14:1_18:2)	GL	___	TG	___	-	212760	35400	260020	1243000	10767000	10214000	1.485433548	43.73253572
LIPID-P-097	C52H94O6	TG(16:1_16:1_17:1)	____TG(16:1_16:1_17:1)	GL	___	TG	___	-	52964	53562	62812	206380	1676700	1962200	1.443221685	22.70772065
LIPID-P-097	C52H94O6	TG(15:0_16:1_18:2)	____TG(15:0_16:1_18:2)	GL	___	TG	___	-	47247	25407	49557	134890	2590200	2318500	1.389310972	41.26952566
LIPID-P-097	C52H94O6	TG(14:0_17:1_18:2)	____TG(14:0_17:1_18:2)	GL	___	TG	___	-	6830.8	3458.7	9938.9	35275	880150	540490	1.435760626	71.9738091
LIPID-P-097	C53H96O6	TG(16:1_16:1_18:1)	____TG(16:1_16:1_18:1)	GL	___	TG	___	-	2571500	230570	3152400	9659500	10014000(	15934000(	1.405133577	45.19957276
LIPID-P-097	C53H96O6	TG(16:0_16:1_18:2)	____TG(16:0_16:1_18:2)	GL	___	TG	___	-	2820800	341350	4246800	13857000	16381000(	28132000(	1.421271544	61.95034384
LIPID-P-097	C54H98O6	TG(16:1_17:1_18:1)	____TG(16:1_17:1_18:1)	GL	___	TG	___	-	47369	9	77136	215180	3470700	7336400	1.215928377	88.52241515
LIPID-P-097	C54H98O6	TG(15:0_18:1_18:2)	____TG(15:0_18:1_18:2)	GL	___	TG	___	-	152760	17903	202960	386350	8540900	15907000	1.339641887	66.46873988
LIPID-P-097	C54H98O6	TG(16:0_17:1_18:2)	____TG(16:0_17:1_18:2)	GL	___	TG	___	-	104890	16142	204990	208730	9369200	17409000	1.268346782	82.77640773
LIPID-P-098	C55H100O6	TG(16:1_18:1_18:1)	____TG(16:1_18:1_18:1)	GL	___	TG	___	-	4844300	310960	7064200	18360000	10661000(	24569000(	1.375144483	30.33358266
LIPID-P-098	C55H100O6	TG(16:0_18:1_18:2)	____TG(16:0_18:1_18:2)	GL	___	TG	___	-	19795000	2617900	30724000	22716000	48196000(	44564000(	1.207286919	17.88429509
LIPID-P-098	C55H100O6	TG(14:0_18:1_20:2)	____TG(14:0_18:1_20:2)	GL	___	TG	___	-	59914	12912	134280	215270	1694800	1854000	1.384590543	18.17460624
LIPID-P-098	C56H102O6	TG(17:0_18:1_18:2)	____TG(17:0_18:1_18:2)	GL	___	TG	___	-	237160	45023	457140	383500	20020000	16469000	1.246484538	49.87333006
LIPID-P-098	C56H102O6	TG(17:1_18:1_18:1)	____TG(17:1_18:1_18:1)	GL	___	TG	___	-	98825	19115	194430	524400	10542000	18558000	1.402814613	94.83753241
LIPID-P-098	C57H104O6	TG(18:1_18:1_18:1)	____TG(18:1_18:1_18:1)	GL	___	TG	___	-	37895000	4868800	82182000	72710000	71410000(	75297000(	1.260809793	12.32358351
LIPID-P-098	C57H104O6	TG(18:0_18:1_18:2)	____TG(18:0_18:1_18:2)	GL	___	TG	___	-	6249200	1117400	20443000	6330700	17098000(	16596000(	1.077425692	12.34360437
LIPID-P-099	C57H104O6	TG(16:0_18:1_20:2)	____TG(16:0_18:1_20:2)	GL	___	TG	___	-	686930	94556	1704900	1099900	24519000	26309000	1.218012391	20.88489076
LIPID-P-099	C58H106O6	TG(17:1_18:1_20:1)	____TG(17:1_18:1_20:1)	GL	___	TG	___	-	9	9	9	13656	465470	667920	1.602194129	42483.18519
LIPID-P-099	C58H106O6	TG(18:1_18:1_19:1)	____TG(18:1_18:1_19:1)	GL	___	TG	___	-	40449	8498.4	69196	139220	2954100	4413900	1.369781589	63.54328723
LIPID-P-099	C59H108O6	TG(18:1_18:2_20:0)	____TG(18:1_18:2_20:0)	GL	___	TG	___	-	572000	113340	2291600	2031000	44125000	57795000	1.29846211	34.91874206
LIPID-P-099	C59H108O6	TG(18:1_18:1_20:1)	____TG(18:1_18:1_20:1)	GL	___	TG	___	-	495930	100870	1507500	1250100	23692000	36969000	1.273346598	29.42123271
LIPID-P-100	C59H108O6	TG(16:1_18:1_22:1)	____TG(16:1_18:1_22:1)	GL	___	TG	___	-	9	9	39575	48539	1415700	682810	1.335329165	54.22799485
LIPID-P-100	C59H108O6	TG(20:1_18:2_18:0)	____TG(20:1_18:2_18:0)	GL	___	TG	___	-	168930	40644	688580	264180	9002000	5742700	1.147439888	16.71080906
LIPID-P-100	C61H112O6	TG(16:1_18:1_24:1)	____TG(16:1_18:1_24:1)	GL	___	TG	___	-	9	9	9	9	38112	36671	1.130309614	2770.074074
LIPID-P-100	C61H112O6	TG(18:1_18:1_22:1)	____TG(18:1_18:1_22:1)	GL	___	TG	___	-	16652	2642.7	32160	41419	1030700	806920	1.322994309	36.51831611
LIPID-P-101	C61H112O6	TG(18:1_18:2_22:0)	____TG(18:1_18:2_22:0)	GL	___	TG	___	-	17304	12899	58925	41851	815810	1121100	1.232684181	22.20133965
LIPID-P-101	C61H112O6	TG(18:1_20:1_20:1)	____TG(18:1_20:1_20:1)	GL	___	TG	___	-	6741.2	9	15038	14362	667810	915970	1.195123127	73.34896871
LIPID-P-101	C63H116O6	TG(18:1_20:1_22:1)	____TG(18:1_20:1_22:1)	GL	___	TG	___	-	11443	1959.4	10213	45187	212580	141900	1.499488511	16.92399875
LIPID-P-101	C63H116O6	TG(18:1_18:1_24:1)	____TG(18:1_18:1_24:1)	GL	___	TG	___	-	3986.9	9	7416.5	19835	94798	76765	1.222681249	16.77105604
LIPID-P-102	C63H116O6	TG(24:0_18:1_18:2)	____TG(24:0_18:1_18:2)	GL	___	TG	___	-	5746.6	4618.3	20437	6379.9	119970	137420	1.001482779	8.563429529
LIPID-P-102	C47H82O6	TG(10:0_16:2_18:2)	____TG(10:0_16:2_18:2)	GL	___	TG	___	-	4257.2	8701.4	8595.9	389580	103920	198430	1.620691767	32.10141734
LIPID-P-102	C49H86O6	TG(14:1_16:1_16:2)	____TG(14:1_16:1_16:2)	GL	___	TG	___	-	9	9	9	77165	213830	179940	1.664499538	17442.03704
LIPID-P-102	C49H86O6	TG(10:0_18:1_18:3)	____TG(10:0_18:1_18:3)	GL	___	TG	___	-	19532	9	14090	415540	270850	564650	1.298701782	37.19901282
LIPID-P-102	C51H90O6	TG(16:1_16:1_16:2)	____TG(16:1_16:1_16:2)	GL	___	TG	___	-	9637.4	9	10746	28448	348130	344320	1.220967215	35.35130735
LIPID-P-103	C51H90O6	TG(14:1_16:1_18:2)	____TG(14:1_16:1_18:2)	GL	___	TG	___	-	34832	9	16570	207800	1075400	1401400	1.275595652	52.21839684
LIPID-P-103	C51H90O6	TG(12:0_18:2_18:2)	____TG(12:0_18:2_18:2)	GL	___	TG	___	-	186050	16027	161520	242940	10368000	1468000	1.248337444	33.22068114

**LIPID-P-103**	C51H90O6	TG(12:0_18:1_18:3)	_____TG(12:0_18:1_18:3)	GL	____	TG	____	-	81082	7727.9	65008	346510	2252200	900540	1.470344343	22.74930291
**LIPID-P-103**	C51H90O6	TG(14:0_16:1_18:3)	_____TG(14:0_16:1_18:3)	GL	____	TG	____	-	29802	9	9	223600	1202400	1884400	1.419254676	111.0127431
**LIPID-P-103**	C53H94O6	TG(14:0_18:2_18:2)	_____TG(14:0_18:2_18:2)	GL	____	TG	____	-	597580	106860	1028000	1245600	24175000	17719000	1.312753646	24.90106439
**LIPID-P-103**	C53H94O6	TG(16:1_16:1_18:2)	_____TG(16:1_16:1_18:2)	GL	____	TG	____	-	1147100	215200	1859300	3214300	38628000	58542000	1.361700725	31.15976533
**LIPID-P-104**	C53H94O6	TG(14:0_16:0_20:4)	_____TG(14:0_16:0_20:4)	GL	____	TG	____	-	41527	5883.9	107780	251860	3901600	2535800	1.41424362	43.10342939
**LIPID-P-104**	C53H94O6	TG(16:0_16:1_18:3)	_____TG(16:0_16:1_18:3)	GL	____	TG	____	-	337700	22416	204390	692960	14840000	10804000	1.374874361	46.65488055
**LIPID-P-104**	C54H96O6	TG(15:0_18:2_18:2)	_____TG(15:0_18:2_18:2)	GL	____	TG	____	-	70872	20823	105390	212470	5212600	4587300	1.371756549	50.80229343
**LIPID-P-104**	C54H96O6	TG(16:1_17:1_18:2)	_____TG(16:1_17:1_18:2)	GL	____	TG	____	-	21206	4612.7	15358	85144	1066000	1820900	1.45017772	72.1778093
**LIPID-P-104**	C55H98O6	TG(16:1_18:1_18:2)	_____TG(16:1_18:1_18:2)	GL	____	TG	____	-	421390	56207	836800	1380000	36618000	45706000	1.347571436	63.68243385
**LIPID-P-104**	C55H98O6	TG(16:0_16:0_20:4)	_____TG(16:0_16:0_20:4)	GL	____	TG	____	-	76845	48362	529970	845710	10534000	10075000	1.407514567	32.74643341
**LIPID-P-104**	C55H98O6	TG(16:0_18:2_18:2)	_____TG(16:0_18:2_18:2)	GL	____	TG	____	-	3234600	515030	9936900	2687900	21142000(	14531000(	1.074681731	26.26070304
**LIPID-P-105**	C56H100O6	TG(15:0_19:2_19:2)	_____TG(15:0_19:2_19:2)	GL	____	TG	____	-	9	9	10523	7503.9	423910	463410	1.322187612	84.88984916
**LIPID-P-105**	C56H100O6	TG(17:1_18:1_18:2)	_____TG(17:1_18:1_18:2)	GL	____	TG	____	-	108660	27679	166640	266760	6710500	15298000	1.316050519	73.52080507
**LIPID-P-105**	C57H102O6	TG(18:1_18:1_18:2)	_____TG(18:1_18:1_18:2)	GL	____	TG	____	-	3311500	454210	5842000	3948500	83292000	11555000(	1.198072692	21.10705881
**LIPID-P-105**	C57H102O6	TG(16:0_18:0_20:4)	_____TG(16:0_18:0_20:4)	GL	____	TG	____	-	207520	59037	460280	125420	11279000	6999300	1.018411361	25.32028502
**LIPID-P-105**	C58H104O6	TG(17:1_18:1_20:2)	_____TG(17:1_18:1_20:2)	GL	____	TG	____	-	43242	12968	97442	156100	3683600	5511600	1.351350371	60.86025564
**LIPID-P-105**	C58H104O6	TG(17:0_19:2_19:2)	_____TG(17:0_19:2_19:2)	GL	____	TG	____	-	4636.8	9	9408	12085	507760	522710	1.226611593	74.18313908
**LIPID-P-106**	C59H106O6	TG(16:0_18:1_22:3)	_____TG(16:0_18:1_22:3)	GL	____	TG	____	-	1341400	230080	3393600	2930800	36102000	37536000	1.274580415	15.4214635
**LIPID-P-106**	C59H106O6	TG(18:1_18:2_20:1)	_____TG(18:1_18:2_20:1)	GL	____	TG	____	-	696550	112850	1501300	1309200	20139000	19845000	1.266698414	17.87042887
**LIPID-P-106**	C59H106O6	TG(18:0_18:1_20:3)	_____TG(18:0_18:1_20:3)	GL	____	TG	____	-	45550	27993	363830	119010	4476600	2294300	1.139568139	15.75293857
**LIPID-P-106**	C59H106O6	TG(16:1_18:1_22:2)	_____TG(16:1_18:1_22:2)	GL	____	TG	____	-	12378	9	15227	28140	358460	223560	1.166043772	22.09603824
**LIPID-P-106**	C61H110O6	TG(18:1_18:1_22:2)	_____TG(18:1_18:1_22:2)	GL	____	TG	____	-	48261	9925.3	140530	124950	1859200	1248300	1.29105239	16.26665754
**LIPID-P-106**	C61H110O6	TG(18:1_22:1_18:2)	_____TG(18:1_22:1_18:2)	GL	____	TG	____	-	23239	6438.4	48515	43842	846710	522590	1.27452446	18.07262598
**LIPID-P-107**	C63H114O6	TG(18:1_24:1_18:2)	_____TG(18:1_24:1_18:2)	GL	____	TG	____	-	4770.1	2691.1	18836	52316	452490	316500	1.480721727	31.23169007
**LIPID-P-108**	C51H88O6	TG(16:0_16:2_16:3)	_____TG(16:0_16:2_16:3)	GL	____	TG	____	-	1843.4	4619.9	4886	2744.2	92489	144350	1.037401682	21.10995392
**LIPID-P-108**	C51H88O6	TG(12:0_18:2_18:3)	_____TG(12:0_18:2_18:3)	GL	____	TG	____	-	26102	9261.6	12548	98561	975420	314270	1.502945921	28.9752586
**LIPID-P-108**	C51H88O6	TG(14:1_16:1_18:3)	_____TG(14:1_16:1_18:3)	GL	____	TG	____	-	9	9	9	27965	124260	120590	1.654550603	10104.25926
**LIPID-P-108**	C53H92O6	TG(16:1_16:1_18:3)	_____TG(16:1_16:1_18:3)	GL	____	TG	____	-	20755	9	15470	153710	1443200	2024200	1.306421103	99.93679969
**LIPID-P-108**	C53H92O6	TG(14:0_18:2_18:3)	_____TG(14:0_18:2_18:3)	GL	____	TG	____	-	83570	13997	124240	262230	3520400	3345600	1.391014252	32.13708314
**LIPID-P-108**	C53H92O6	TG(14:1_18:2_18:2)	_____TG(14:1_18:2_18:2)	GL	____	TG	____	-	24334	9	43717	138920	1640500	1195000	1.222020509	43.7029092
**LIPID-P-108**	C53H92O6	TG(12:0_16:0_22:5)	_____TG(12:0_16:0_22:5)	GL	____	TG	____	-	10002	9	9	41698	976390	539960	1.419622754	155.4938124
**LIPID-P-109**	C53H92O6	TG(14:0_16:1_20:4)	_____TG(14:0_16:1_20:4)	GL	____	TG	____	-	23160	9	26754	260160	1944400	1186300	1.293743635	67.92179957
**LIPID-P-109**	C54H94O6	TG(15:0_18:2_18:3)	_____TG(15:0_18:2_18:3)	GL	____	TG	____	-	9	9	9	7433.9	349210	256450	1.596474489	22707.18148
**LIPID-P-109**	C54H94O6	TG(15:0_16:1_20:4)	_____TG(15:0_16:1_20:4)	GL	____	TG	____	-	9	9	9	9	102430	71685	1.130840739	6449.037037
**LIPID-P-109**	C54H94O6	TG(16:1_17:1_18:3)	_____TG(16:1_17:1_18:3)	GL	____	TG	____	-	3444.6	9	9	12798	383830	201860	1.429035765	172.843528
**LIPID-P-109**	C55H96O6	TG(16:1_18:2_18:2)	_____TG(16:1_18:2_18:2)	GL	____	TG	____	-	1724500	297790	2882800	2922700	10368000(	10932000(	1.269545318	44.02013011
**LIPID-P-109**	C55H96O6	TG(16:0_18:2_18:3)	_____TG(16:0_18:2_18:3)	GL	____	TG	____	-	502090	73462	743070	663730	55610000	29150000	1.236052254	64.78257605
**LIPID-P-109**	C55H96O6	TG(16:1_18:1_18:3)	_____TG(16:1_18:1_18:3)	GL	____	TG	____	-	861690	54251	1086400	1002300	70546000	36613000	1.231817932	54.01742261
**LIPID-P-109**	C55H96O6	TG(16:0_16:1_20:4)	_____TG(16:0_16:1_20:4)	GL	____	TG	____	-	100470	26157	319130	777920	11656000	14797000	1.425392686	61.08915844
**LIPID-P-110**	C56H98O6	TG(17:1_18:2_18:2)	_____TG(17:1_18:2_18:2)	GL	____	TG	____	-	48632	5201.1	54437	67047	3675200	4560700	1.26510802	76.68734951
**LIPID-P-110**	C56H98O6	TG(15:0_18:1_20:4)	_____TG(15:0_18:1_20:4)	GL	____	TG	____	-	12184	9	8868.5	29105	550340	550540	1.236807563	53.65168673
**LIPID-P-110**	C57H100O6	TG(18:1_18:2_18:2)	_____TG(18:1_18:2_18:2)	GL	____	TG	____	-	1451100	247850	2795800	1160400	75468000	86648000	1.117331416	36.32602481
**LIPID-P-110**	C57H100O6	TG(16:0_18:1_20:4)	_____TG(16:0_18:1_20:4)	GL	____	TG	____	-	650600	207770	3832100	2659900	53122000	49915000	1.28087185	22.53439421
**LIPID-P-110**	C57H100O6	TG(16:1_18:1_20:3)	_____TG(16:1_18:1_20:3)	GL	____	TG	____	-	77100	9158.4	181550	247870	4945100	4898400	1.336024402	37.68130499
**LIPID-P-110**	C58H102O6	TG(16:0_17:0_22:5)	_____TG(16:0_17:0_22:5)	GL	____	TG	____	-	4239.8	1040.9	5594.8	6882.9	139510	150670	1.291526251	27.31487288
**LIPID-P-110**	C58H102O6	TG(17:0_18:1_20:4)	_____TG(17:0_18:1_20:4)	GL	____	TG	____	-	9	9	40384	57794	604640	507460	1.329363998	28.9563388
**LIPID-P-110**	C58H102O6	TG(19:1_18:2_18:2)	_____TG(19:1_18:2_18:2)	GL	____	TG	____	-	13583	9	15728	35413	1519500	1284000	1.235737503	96.82513643
**LIPID-P-111**	C59H104O6	TG(18:0_18:3_20:2)	_____TG(18:0_18:3_20:2)	GL	____	TG	____	-	337570	75342	877220	455980	13421000	10012000	1.177280699	18.51669442
**LIPID-P-111**	C59H104O6	TG(16:0_18:1_22:4)	_____TG(16:0_18:1_22:4)	GL	____	TG	____	-	92128	50465	561840	522400	24282000	16927000	1.313780242	59.24112016
**LIPID-P-111**	C59H104O6	TG(18:1_18:2_20:2)	_____TG(18:1_18:2_20:2)	GL	____	TG	____	-	560800	237010	2061700	1120500	37938000	33710000	1.204944591	25.44789142
**LIPID-P-111**	C59H104O6	TG(18:0_18:1_20:4)	_____TG(18:0_18:1_20:4)	GL	____	TG	____	-	346010	159890	1361300	671530	10804000	6246200	1.174734311	9.491072194
**LIPID-P-111**	C59H104O6	TG(18:2_18:2_20:1)	_____TG(18:2_18:2_20:1)	GL	____	TG	____	-	96221	37029	358060	161040	5226400	5015100	1.168078982	21.17306792
**LIPID-P-112**	C61H108O6	TG(18:2_18:2_22:1)	_____TG(18:2_18:2_22:1)	GL	____	TG	____	-	17708	3569.3	81298	63340	863910	537320	1.283527857	14.2779987
**LIPID-P-112**	C61H108O6	TG(18:1_18:2_22:2)	_____TG(18:1_18:2_22:2)	GL	____	TG	____	-	9	9	21636	25134	384540	253690	1.327034853	30.63470952
**LIPID-P-112**	C61H108O6	TG(18:0_18:1_22:4)	_____TG(18:0_18:1_22:4)	GL	____	TG	____	-	20677	18800	68826	49919	2257000	1300600	1.222407938	33.30950205
**LIPID-P-112**	C61H108O6	TG(16:0_18:1_24:4)	_____TG(16:0_18:1_24:4)	GL	____	TG	____	-	1454.2	9	7317.6	5017.8	393010	127120	1.214512959	59.806373
**LIPID-P-112**	C63H112O6	TG(24:1_18:2_18:2)	_____TG(24:1_18:2_18:2)	GL	____	TG	____	-	3893.6	1863.5	9638.8	17709	211080	104600	1.418148172	21.6544015
**LIPID-P-112**	C53H90O6	TG(14:1_18:2_18:3)	_____TG(14:1_18:2_18:3)	GL	____	TG	____	-	5927.5	2771.7	28043	13648	479550	219740	1.193345028	19.40379183
**LIPID-P-112**	C53H90O6	TG(16:0_18:2_16:4)	_____TG(16:0_18:2_16:4)	GL	____	TG	____	-	9	9	9	12133	278880	52890	1.629179928	12737.14815
**LIPID-P-113**	C53H90O6	TG(14:0_16:1_20:5)	_____TG(14:0_16:1_20:5)	GL	____	TG	____	-	5268.5	700.34	347.51	5634.3	78642	30062	1.339958584	18.10195762
**LIPID-P-113**	C53H90O6	TG(12:0_16:0_22:6)	_____TG(12:0_16:0_22:6)	GL	____	TG	____	-	16528	3929.1	16376	90450	645720	136790	1.501147569	23.70042163
**LIPID-P-113**	C55H94O6	TG(16:0_16:1_20:5)	_____TG(16:0_16:1_20:5)	GL	____	TG	____	-	8731.3	2722.5	9	104100	737800	1137300	1.35558667	172.6628747
**LIPID-P-113**	C55H94O6	TG(16:1_18:2_18:3)	_____TG(16:1_18:2_18:3)	GL	____	TG	____	-	70255	10670	79672	97204	4021900	3606700	1.270497254	48.10677659
**LIPID-P-113**	C55H94O6	TG(16:0_18:2_18:4)	_____TG(16:0_18:2_18:4)	GL	____	TG	____	-	9	9	25681	34339	774540	641370	1.344395871	56.43211798
**LIPID-P-113**	C56H96O6	TG(17:1_18:2_18:3)	_____TG(17:1_18:2_18:3)	GL	____	TG	____	-	9	9	9	9	393060	247590	1.130877917	23728.11111
**LIPID-P-114**	C57H98O6	TG(16:0_18:2_20:4)	_____TG(16:0_18:2_20:4)	GL	____	TG	____	-	2659200	927770	6196600	3072800	99786000	11009000(	1.146434237	21.76596069
**LIPID-P-114**	C57H98O6	TG(18:1_18:2_18:3)	_____TG(18:1_18:2_18:3)	GL	____	TG	____	-	530980	118340	681150	532660	35877000	27197000	1.194709471	47.80766195
**LIPID-P-114**	C57H98O6	TG(16:0_16:1_22:5)	_____TG(16:0_16:1_22:5)	GL	____	TG	____	-	9	9	107460	168280	4072900	4667000	1.344764877	82.88375295
**LIPID-P-114**	C57H98O6	TG(16:0_16:0_22:6)	_____TG(16:0_16:0_22:6)	GL	____	TG	____	-	76952	82734	406410	386360	4909200	7022600	1.310137386	21.75984285
**LIPID-P-114**	C59H102O6	TG(16:0_18:1_22:5)	_____TG(16:0_18:1_22:5)	GL	____	TG	____	-	57509	41575	397260	209800	5965900	6293400	1.226902262	25.12189127
**LIPID-P-115**	C59H102O6	TG(18:0_18:2_20:4)	_____TG(18:0_18:2_20:4)	GL	____	TG	____	-	485660	242980	2243500	1416700	26388000	32869000	1.245792014	20.41414604
**LIPID-P-115**	C59H102O6	TG(16:0_18:0_22:6)	_____TG(16:0_18:0_22:6)	GL	____	TG	____	-	19521	22162	136940	198380	1966700	2816600	1.38712218	27.88935355
**LIPID-P-115**	C59H102O6	TG(18:2_18:2_20:2)	_____TG(18:2_18:2_20:2)	GL	____	TG	____	-	117690	58858	698840	381670	5918600	5993900	1.232601762	14.04425238
**LIPID-P-115**	C61H106O6	TG(18:1_18:1_22:4)	_____TG(18:1_18:1_22:4)	GL	____	TG	____	-	50756	27704	279510	217560	7633000	6536400	1.287404496	40.19040702
**LIPID-P-115**	C61H106O6	TG(18:0_18:0_22:6)	_____TG(18:0_18:0_22:6)	GL	____	TG	____	-	2968.2	7335.6	44496	29202	237490	490030	1.238269233	13.80884602
**LIPID-P-116**	C63H110O6	TG(18:1_18:1_24:4)	_____TG(18:1_18:1_24:4)	GL	____	TG	____	-	2975.7	9	12480	19620	314690	223530	1.248643452	36.0718281
**LIPID-P-116**	C55H92O6	TG(14:0_18:3_20:4)	_____TG(14:0_18:3_20:4)	GL	____	TG	____	-	5541.1	9	3279.9	11083	422770	137950	1.257589025	64.75685164
**LIPID-P-116**	C55H92O6	TG(16:1_16:1_20:5)	_____TG(16:1_16:1_20:5)	GL	____	TG	____	-	9	9	9	21917	83919	104170	1.653413988	7778
**LIPID-P-117**	C57H96O6	TG(16:0_18:2_20:5)	_____TG(16:0_18:2_20:5)	GL	____	TG	____	-	26731	18235	135440	80018	2831200	2442100	1.230361886	29.67372482
**LIPID-P-117**	C57H96O6	TG(16:0_16:1_22:6)	_____TG(16:0_16:1_22:6)	GL	____	TG	____	-	81315	44739	293570	962890	3478300	6455500	1.499793189	25.96774732
**LIPID-P-117**	C57H96O6	TG(18:1_18:2_18:4)	_____TG(18:1_18:2_18:4)	GL	____	TG	____	-	19606	9	9	14519	803030	492360	1.322114982	66.75035671
**LIPID-P-117**	C57H96O6	TG(18:2_18:2_18:3)	_____TG(18:2_18:2_18:3)	GL	____	TG	____	-	110520	16163	175920	81873	10020000	4011800	1.12429085	46.64088922
**LIPID-P-117**	C58H98O6	TG(15:0_18:1_22:6)	_____TG(15:0_18:1_22:6)	GL	____	TG	____	-	9	9	9	27008	269390	373310	1.635146408	24804
**LIPID-P-117**	C59H100O6	TG(18:1_18:2_20:4)	_____TG(18:1_18:2_20:4)	GL	____	TG	____	-	260880	210240	1097600	633540	20435000	25932000	1.201549536	29.96107655
**LIPID-P-117**	C59H100O6	TG(16:0_18:1_22:6)	_____TG(16:0_18:1_22:6)	GL	____	TG	____	-	273160	213570	1855100	1918700	20352000	34141000	1.339560623	24.08872548
**LIPID-P-117**	C59H100O6	TG(16:1_16:1_24:5)	_____TG(16:1_16:1_24:5)	GL	____	TG	____	-	9	9	33876	39035	1571400	1235000	1.341510453	83.95099428
**LIPID-P-118**	C61H104O6	TG(18:0_18:1_22:6)	_____TG(18:0_18:1_22:6)	GL	____	TG	____	-	72142	127040	615150	204830	2436100	3512400	1.05722524	7.556291537
**LIPID-P-118**	C61H104O6	TG(18:1_18:1_22:5)	_____TG(18:1_18:1_22:5)	GL	____	TG	____	-	63237	57996	454990	330490	2368600	2774900	1.285869903	9.499776996
**LIPID-P-118**	C61H104O6	TG(18:1_18:2_22:4)	_____TG(18:1_18:2_22:4)	GL	____	TG	____	-	39447	38103	175610	130860	3889900	4728700	1.248092013	34.5609891
**LIPID-P-118**	C61H104O6	TG(16:0_20:2_22:5)	_____TG(16:0_20:2_22:5)	GL	____	TG	____	-	7212	1901.9	18740	15436	697840	199250	1.253940807	32.76115732
**LIPID-P-119**	C55H90O6	TG(14:1_16:1_22:6)	_____TG(14:1_16:1_22:6)	GL	____	TG	____	-	6837.5	9	9	58082	304970	56581	1.427793856	61.21114434
**LIPID-P-119**	C57H94O6	TG(18:2_18:3_18:3)	_____TG(18:2_18:3_18:3)	GL	____	TG	____	-	12222	2610.2	6519.3	18004	325060	111340	1.352653504	21.28206449
**LIPID-P-119**	C57H94O6	TG(16:1_16:1_22:6)	_____TG(16:1_16:1_22:6)	GL	____	TG	____	-	12055	6794.8	52751	151400	527760	643780	1.501337649	18.4766092
**LIPID-P-120**	C59H98O6	TG(16:0_18:2_22:6)	_____TG(16:0_18:2_22:6)	GL	____	TG	____	-	217900	102330	1134000	788450	15477000	16841000	1.272766378	22.76562167
**LIPID-P-120**	C59H98O6	TG(16:1_18:1_22:6)	_____TG(16:1_18:1_22:6)	GL	____	TG	____	-	5789.4	8197.2	26833	59795	256820	504070	1.446306306	20.10517007
**LIPID-P-120**	C59H98O6	TG(16:1_16:1_24:6)	_____TG(16:1_16:1_24:6)	GL	____	TG	____	-	35818	21880	156620	76606	3033700	2960600	1.18515302	28.32662679
**LIPID-P-120**	C60H100O6	TG(17:0_18:2_22:6)	_____TG(17:0_18:2_22:6)	GL	____	TG	____	-	9	9	8552.5	8396.2	218110	212350	1.335501081	51.20543726
**LIPID-P-120**	C61H102O6	TG(18:1_18:2_22:5)	_____TG(18:1_18:2_22:5)	GL	____	TG	____	-	76987	76920	641900	244370	6221200	3893600	1.153156752	13.01718884
**LIPID-P-120**	C61H102O6	TG(18:1_18:1_22:6)	_____TG(18:1_18:1_22:6)	GL	____	TG	____	-	57224	46283	319000	238300	803550	2995700	1.23198382	9.556173034
**LIPID-P-120**	C61H102O6	TG(18:0_18:2_22:6)	_____TG(18:0_18:2_22:6)	GL	____	TG	____	-	191470	151090	1233900	635250	5498500	13342000	1.190618953	12.3541035
**LIPID-P-120**	C61H102O6	TG(18:2_18:2_22:4)	_____TG(18:2_18:2_22:4)	GL	____	TG	____	-	5102.7	13749	61493	21922	1468300	833310	1.122488132	28.91954292
**LIPID-P-120**	C63H106O6	TG(18:1_20:1_22:6)	_____TG(18:1_20:1_22:6)	GL	____	TG	____	-	7789.9	4162.7	17766	12893	155970	287460	1.208953147	15.35479464
**LIPID-P-121**	C57H92O6	TG(16:0_18:4_20:5)	_____TG(16:0_18:4_20:5)	GL	____	TG	____	-	18951	9	20781	40284	337310	541680	1.154331261	23.13162729
**LIPID-P-121**	C57H92O6	TG(16:1_16:2_22:6)	_____TG(16:1_16:2_22:6)	GL	____	TG	____	-	4550.7	1668.5	4575	22798	103140	74096	1.549722225	18.53161883
**LIPID-P-121**	C59H96O6	TG(16:1_18:2_22:6)	_____TG(16:1_18:2_22:6)	GL	____	TG	____	-	11419	9	50550	71543	1277400	1540800	1.213021202	46.62530253
**LIPID-P-121**	C59H96O6	TG(18:2_18:3_20:4)	_____TG(18:2_18:3_20:4)	GL	____	TG	____	-	3756.6	2640.3	31825	9853.5	568860	286210	1.136074522	22.62900327
**LIPID-P-121**	C61H100O6	TG(18:1_18:2_22:6)	_____TG(18:1_18:2_22:6)	GL	____	TG	____	-	123520	84128	709010	390020	4059900	5820900	1.218831973	11.20463684
**LIPID-P-121**	C61H100O6	TG(18:2_20:3_20:4)	_____TG(18:2_20:3_20:4)	GL	____	TG	____	-	4793.1	9	43092	39422	350890	412040	1.178216864	16.75262715
**LIPID-P-121**	C61H100O6	TG(16:0_20:4_22:5)	_____TG(16:0_20:4_22:5)	GL	____	TG	____	-	18019	20551	144710	38129	1750100	954030	1.060717718	14.96212898
**LIPID-P-122**	C63H104O6	TG(20:1_18:2_22:6)	_____TG(20:1_18:2_22:6)	GL	____	TG	____	-	10257	5194.1	38894	15960	215410	270490	1.119494094	9.234687212
**LIPID-P-122**	C61H98O6	TG(18:2_18:2_22:6)	_____TG(18:2_18:2_22:6)	GL	____	TG	____	-	20182	25046	89616	71361	1665300	858620	1.257899287	19.24654415
**LIPID-P-122**	C61H98O6	TG(18:2_20:4_20:4)	_____TG(18:2_20:4_20:4)	GL	____	TG	____	-	6261	10806	33158	29274	508800	344300	1.277904522	17.5684221
**LIPID-P-123**	C61H96O6	TG(18:2_18:3_22:6)	_____TG(18:2_18:3_22:6)	GL	____	TG	____	-	3727.4	2042.9	9198.6	27341	142440	79306	1.514750824	16.64030089
**LIPID-P-123**	C63H100O6	TG(18:1_20:4_22:6)	_____TG(18:1_20:4_22:6)	GL	____	TG	____	-	6014.9	20418	26085	17515	147360	72775	1.055508674	4.525123815
**LIPID-P-124**	C63H98O6	TG(16:0_22:6_22:6)	_____TG(16:0_22:6_22:6)	GL	____	TG	____	-	1180.6	5026.2	7679.7	13746	88034	172460	1.342665453	19.74867677
**LIPID-P-124**	C65H100O6	TG(18:2_22:5_22:6)	_____TG(18:2_22:5_22:6)	GL	____	TG	____	-	2295.8	8613.5	8107.2	12066	13960	22255	1.201284325	2.538900429

Log2FC	Type	hmdb_ID	primary_SMPDB_ID
-2.140539496	down	HMDB0000020	SMP0000006;SMP0000169;SMP0000190;SMP0000218;SMP0000429;SMP0000494;SMP0000498;SMP0000533
11.1652327	up	HMDB0000036	SMP0000035;SMP0000314;SMP0000318;SMP0000317;SMP0000316;SMP0000315;SMP0000720
1.961657282	up	HMDB0000094	SMP0000057;SMP0000466;SMP0000546;SMP0000547;SMP0000548;SMP0000549;SMP0000550;SMP0000551;SMP0000654;SMP0002291;SMP0002292;SMP0002295;SMP0002298;SMP0002358;SMP0002359;SMP0083
2.529955016	up	HMDB0000008	SMP0000016;SMP0000198;SMP0000201;SMP0000502
-2.140539496	down	HMDB0000703	--
1.388033999	up	HMDB0000224	SMP0000034;SMP0000349;SMP0000348;SMP0000347;SMP0000525;SMP0000526;SMP0000649;SMP0014212;SMP0029731
-1.562241192	down	--	--
-1.966362964	down	--	--
-1.322973284	down	HMDB0002183	SMP0000018
-1.250244497	down	HMDB0005060	--
1.019211292	up	HMDB0000511	SMP0000456
3.156457809	up	HMDB0002212	--
-1.050472387	down	HMDB0001351	SMP0000050;SMP0000031;SMP0000144;SMP0000167;SMP0000365;SMP0000364;SMP0000203;SMP0000220;SMP0000210;SMP0000168;SMP0000046;SMP0000172;SMP0000219;SMP0000178;SMP0000202;SMP0000
2.136625465	up	HMDB0001367	SMP0000045;SMP0000216;SMP0000240;SMP0000390;SMP0000534
2.366505519	up	HMDB0001067	--
-1.206363243	down	HMDB0031518	--
1.063588508	up	HMDB0000672	--
12.13952767	up	--	--
11.01291418	up	--	--
2.229115163	up	--	--
-11.0632952	down	--	--
-2.040870722	down	--	--
-1.330490962	down	--	--
2.696501975	up	--	--
-1.322973284	down	--	--
-2.400446589	down	HMDB0001975	--
-4.822273627	down	--	--
1.251656739	up	HMDB0031057	--
-1.237231707	down	--	--
-1.670199535	down	--	--
-1.670199535	down	--	--
-1.670199535	down	--	--
-2.725640265	down	--	--
-2.725640265	down	--	--
-2.513737684	down	--	--
-3.629404396	down	--	--
-3.629404396	down	--	--
1.157576375	up	HMDB0000517	SMP0000067;SMP0000020;SMP0000207;SMP0000362;SMP0000360;SMP0000361;SMP0000208;SMP0000188;SMP0000363;SMP0000175;SMP0000192;SMP0000004;SMP0000059;SMP0000357;SMP0000003;SMP0000
1.181726646	up	HMDB0000043	SMP0000123;SMP0000033;SMP0000177;SMP0000341;SMP0000214;SMP0000222;SMP0000340;SMP0000221;SMP0000004;SMP0000242;SMP0000179;SMP0000244;SMP0000223;SMP0000484;SMP0000485;SMP0000
-1.313913655	down	HMDB0000725	SMP0000020;SMP0000207;SMP0000362;SMP0000360;SMP0000361;SMP0000208;SMP0000188;SMP0000363;SMP0000504;SMP0000505;SMP0000506;SMP0000507;SMP0002292;SMP0002295
1.218657101	up	HMDB0001414	SMP0000445;SMP0000033;SMP0000177;SMP0000341;SMP0000214;SMP0000222;SMP0000340;SMP0000221;SMP0000570
3.659882808	up	HMDB0000403	--
1.863712932	up	HMDB0000068	SMP0000012;SMP0000170;SMP0000006;SMP0000169;SMP0000190;SMP0000218;SMP0000429;SMP0000494;SMP0000497;SMP0000498;SMP0000533;SMP0000661
2.033569336	up	HMDB0000763	SMP0000063
2.186124017	up	HMDB0003681	--
-1.734166738	down	HMDB0010379	SMP0000075;SMP0000353;SMP0000077;SMP0000083;SMP0000084;SMP0000085;SMP0000086;SMP0000087;SMP0000093;SMP0000094;SMP0000096;SMP0000098;SMP0000101;SMP0000102;SMP0000104;SMP0000
-1.960962573	down	HMDB0010383	--
-1.721617559	down	HMDB0010385	--
-2.33790218	down	HMDB0010386	--
-1.834549529	down	HMDB0010391	--
-2.471281039	down	HMDB0010392	--
1.408798963	up	HMDB0000532	--
2.52551367	up	HMDB0006216	SMP0000074;SMP0000336
-2.41987118	down	HMDB0000086	SMP0000074;SMP0000336
-2.33790218	down	--	--
-1.823810626	down	HMDB0000235	SMP0000076
10.48854685	up	HMDB0001186	--
20.73363182	up	--	--
-1.101199151	down	HMDB0062660	--
1.123726213	up	HMDB0003157	--
-1.326180608	down	HMDB0000453	--
-1.075728585	down	HMDB0000895	SMP0000025;SMP0000225;SMP0000226;SMP0000227;SMP0000228;SMP0000229;SMP0000230;SMP0000231;SMP0000232;SMP0000233;SMP0000246;SMP0000589;SMP0000734;SMP0000735;SMP0000736;SMP0051
-14.18010772	down	--	--
-1.204042961	down	--	--
-1.66229766	down	--	--
-1.912411146	down	HMDB0000138	SMP0000035;SMP0000314;SMP0000318;SMP0000317;SMP0000316;SMP0000315;SMP0000720
10.85980221	up	HMDB0000037	SMP0000130;SMP0000483;SMP0000373;SMP0000371;SMP0000372;SMP0000184;SMP0000189;SMP0000193;SMP0000197;SMP0000145;SMP0000146;SMP0000147;SMP0000148;SMP0000149;SMP0000150;SMP0000
1.90234245	up	HMDB0003876	SMP0000075;SMP0000353;SMP0000077;SMP0000083;SMP0000084;SMP0000085;SMP0000086;SMP0000087;SMP0000093;SMP0000094;SMP0000096;SMP0000098;SMP0000101;SMP0000102;SMP0000104;SMP0000
-1.075728585	down	--	--
-1.419770463	down	--	--
1.28880013	up	HMDB0003282	--
4.170241031	up	HMDB0002141	--
2.032476035	up	HMDB0000234	SMP0000068;SMP0000356;SMP0000565;SMP0030406
-1.567769399	down	HMDB0000536	SMP0000067;SMP0000050;SMP0000144;SMP0000167;SMP0000365;SMP0000364;SMP0000203;SMP0000220;SMP0000210;SMP0000168;SMP0000175;SMP0000192;SMP0000427;SMP0000428;SMP0000430;SMP0000
-5.364071864	down	--	--
-1.30800747	down	--	--
2.726822667	up	--	--
3.659882808	up	HMDB0000132	SMP0000050;SMP0000144;SMP0000167;SMP0000365;SMP0000364;SMP0000203;SMP0000220;SMP0000210;SMP0000168;SMP0000427;SMP0000428;SMP0000430;SMP0000512;SMP0000513;SMP0000535;SMP0000
-2.767929368	down	--	--
-3.022665192	down	--	--
-3.022665192	down	--	--
-3.073927839	down	--	--
-3.073927839	down	--	--
-3.248624063	down	--	--
-2.581442809	down	--	--
-3.163670409	down	--	--
-3.163670409	down	--	--
-2.109581027	down	--	--
-2.109581027	down	--	--
-2.896294531	down	--	--
-2.949792191	down	--	--
-1.914973462	down	--	--
-4.168200321	down	--	--
-3.272733083	down	--	--
-1.680642955	down	--	--
-1.720151449	down	--	--
-1.117989817	down	--	--
-1.112337526	down	--	--
-1.085018617	down	--	--
-1.699735821	down	--	--
-1.661305441	down	--	--
-3.154756699	down	--	--
-3.154756699	down	--	--
-2.484564502	down	--	--
-2.484564502	down	--	--
-1.492755237	down	--	--
-1.048132376	down	HMDB0000828	SMP0000067;SMP0000175;SMP0000192;SMP0000046;SMP0000172;SMP0000219;SMP0000178;SMP0000202
-1.076575533	down	HMDB0060460	--
-1.173743709	down	HMDB0000518	SMP0000035;SMP0000314;SMP0000318;SMP0000317;SMP0000316;SMP0000315;SMP0000720
-1.146623363	down	HMDB0001999	SMP0000018
2.317957976	up	HMDB0010203	--
-1.235212232	down	HMDB0002231	--
-1.282323847	down	--	--
-1.216851316	down	HMDB0012535	--
-1.385557248	down	HMDB0005060	--
-1.618625643	down	HMDB0002925	SMP0000018
-1.297033291	down	HMDB0002823	--
-1.722861482	down	HMDB0001043	SMP0000018;SMP0000075;SMP0000353;SMP0000077;SMP0000083;SMP0000084;SMP0000085;SMP0000086;SMP0000087;SMP0000093;SMP0000094;SMP0000096;SMP0000098;SMP0000101;SMP0000102;SMP0000
-1.302151741	down	HMDB0034295	--
-1.860179755	down	HMDB0004708	--
-1.085351851	down	--	--
-1.581162759	down	HMDB0002183	SMP0000018
-1.10387526	down	--	--
-1.593387062	down	HMDB0010382	SMP0000025
-1.447948484	down	HMDB0010383	--
-1.953735496	down	--	--
-2.657854356	down	HMDB0010386	--
-1.994682512	down	HMDB0011503	SMP0000025
-1.578171394	down	HMDB0011129	--
-1.842307713	down	HMDB0011507	--
-1.860434374	down	HMDB0011487	--
-1.187907529	down	HMDB0011493	--
-1.71785692	down	HMDB0011524	--
-1.880960391	down	HMDB0011496	--
-1.904502035	down	--	--
-3.25681943	down	--	--

-3.431603181	down	--	--
-2.201497575	down	--	--
-1.51487553	down	HMDB0240606	--
-1.118822813	down	--	--
-1.075678292	down	--	--
-1.984280186	down	--	--
-1.331689739	down	--	--
1.78616409	up	HMDB0008002	--
-1.064565034	down	HMDB0007973	--
-1.206689724	down	HMDB0008143	--
-1.188414057	down	HMDB0008156	--
-2.114534699	down	HMDB0009394	--
-1.254251313	down	HMDB0008939	--
-1.342873495	down	HMDB0009005	--
-1.128411284	down	HMDB0009005	--
-1.343046215	down	--	--
3.042903035	up	--	--
-2.2208252	down	--	--
-1.60188921	down	--	--
-1.375331176	down	HMDB0009880	--
-1.113151014	down	HMDB0009821	--
1.224087734	up	HMDB0010163	--
1.372910964	up	--	--
-1.267790614	down	HMDB0012401	--
1.343888653	up	HMDB0010700	--
1.295797286	up	--	--
-1.639355222	down	HMDB0007849	--
-2.021726916	down	HMDB07849	--
-1.680209089	down	HMDB07849	--
1.891246996	up	HMDB0000674	SMP0000025;SMP0000039;SMP0000187;SMP0000529;SMP0000530
1.916668783	up	--	--
-1.83238652	down	--	--
-1.192153205	down	HMDB0062555	--
-1.830134372	down	HMDB0002095	--
-1.156147317	down	HMDB0061717	--
-1.000676386	down	HMDB0002366	--
1.762724608	up	HMDB0013324	--
-2.026955729	down	--	--
-1.568884163	down	HMDB0013331	--
-1.641679329	down	HMDB0013334	--
-2.375216782	down	HMDB0006469	--
2.877801438	up	HMDB0000918	--
1.593598862	up	--	--
1.537512492	up	--	--
1.950981059	up	--	--
-1.118924482	down	HMDB0007100	--
3.819022147	up	HMDB0094301	--
-1.512609048	down	HMDB0007108	--
-1.941487673	down	HMDB0007160	--
2.359989592	up	HMDB0007128	--
-1.404102201	down	HMDB0007161	--
-2.257530701	down	HMDB0007167	--
-1.127032524	down	HMDB0007253	--
-1.199887671	down	HMDB0007225	--
11.38079974	up	HMDB0007134	--
-1.395982349	down	HMDB0007119	--
-1.360659558	down	HMDB0007177	--
-1.38613156	down	HMDB0007234	--
-1.498939653	down	HMDB0007121	--
-1.24541091	down	HMDB0007148	--
-1.879395262	down	HMDB0007263	--
-1.743332049	down	HMDB0007236	--
-1.590919136	down	HMDB0007179	--
-2.058904522	down	HMDB0007208	--
-1.373076483	down	HMDB0007266	--
-1.900218172	down	HMDB0011566	--
-2.029542937	down	--	--
-1.712842771	down	HMDB0004879	--
-1.60226891	down	HMDB0010379	SMP0000075;SMP0000353;SMP0000077;SMP0000083;SMP0000084;SMP0000085;SMP0000086;SMP0000087;SMP0000093;SMP0000094;SMP0000096;SMP0000098;SMP0000101;SMP0000102;SMP0000104;SMP0000
-1.022321001	down	--	--
-1.179413037	down	HMDB0010381	--
-1.347637817	down	HMDB0012108	--
-1.865824304	down	--	--
-2.019852981	down	--	--
-2.080515476	down	HMDB0010385	--
-2.442646779	down	--	--
-2.556679509	down	--	--
-1.345579798	down	--	--
-3.423205205	down	--	--
-2.72998576	down	--	--
-2.852483201	down	HMDB0010392	--
-2.938180189	down	HMDB0010388	--
-2.923467446	down	HMDB0010393	--
-2.662831713	down	HMDB0010389	--
-1.885926117	down	HMDB0010395	--
-2.391796512	down	--	--
-2.298444948	down	HMDB0010401	--
-2.727473564	down	HMDB0010397	--
-2.255673948	down	--	--
-2.757414464	down	HMDB0010402	--
-2.283895096	down	HMDB0010404	--
-1.742364875	down	--	--
-1.982340994	down	HMDB0011130	--
-1.958464629	down	HMDB0011481	--
-1.34442368	down	HMDB0011490	--
-2.554915611	down	HMDB0011477	--
-1.527657217	down	HMDB0011483	--
-1.719434965	down	--	--
-1.803799179	down	HMDB0011516	--
-2.908209295	down	--	--
-2.155164375	down	HMDB0011494	--
-1.854219183	down	HMDB0011152	--
-1.39125557	down	HMDB0240598	--
-2.150717644	down	--	--
-1.445532268	down	--	--
-2.246931059	down	--	--
-1.548408811	down	HMDB0240605	--
-1.155714851	down	HMDB0007984	--
1.050450524	up	HMDB0013404	--
-1.426393509	down	HMDB0011151	--
1.131733811	up	--	--
-1.203786871	down	--	--
-2.119466368	down	--	--
-1.055253058	down	--	--
-1.265039542	down	HMDB0009056	--
1.175402178	up	HMDB0008961	--
-1.216917132	down	--	--
-1.826417604	down	HMDB0008970	--
-1.021319474	down	HMDB0009010	--
-1.015198657	down	HMDB0008913	--
-1.104562525	down	--	--
-1.848197305	down	HMDB0009691	--
1.110731769	up	HMDB0011380	--
2.716576821	up	HMDB0012357	--
1.808892434	up	HMDB0112417	--
1.199047338	up	HMDB0012085	--
1.064829797	up	HMDB0010168	--
1.006895574	up	HMDB0010169	--
1.133332951	up	HMDB0240637	--
1.275245371	up	HMDB0012104	--
1.141110548	up	HMDB0240614	--
1.373670328	up	--	--
1.523817318	up	HMDB0013461	--
2.245970625	up	HMDB0011188	--
5.955187115	up	HMDB0071936	--
2.40326738	up	HMDB0071588	--
5.070628766	up	HMDB0071914	--
2.966378297	up	HMDB0071634	--
6.225020592	up	HMDB0072111	--

4.938025752	up	HMDB0095276	--
3.324696063	up	HMDB0095006	--
4.958917445	up	HMDB0042063	--
4.050766744	up	HMDB0095296	--
4.787747229	up	HMDB0095566	--
3.67924461	up	HMDB0042093	--
5.63735328	up	HMDB0010411	--
5.602096061	up	HMDB0043019	--
5.39832476	up	HMDB0005356	SMP0000039;SMP0000187;SMP0000529;SMP0000530
5.559702644	up	HMDB0042124	--
6.75309829	up	HMDB0104223	--
5.520979559	up	HMDB0005357	--
6.264374875	up	HMDB0005365	--
7.175197939	up	HMDB0042155	--
4.316743947	up	HMDB0106781	--
5.115470619	up	HMDB0005393	--
4.827363171	up	HMDB0043916	--
14.39865711	up	--	--
5.680117841	up	--	--
5.470488945	up	--	--
4.970175929	up	--	--
5.589838143	up	--	--
4.351800277	up	--	--
5.530130905	up	--	--
2.746145279	up	--	--
4.54933515	up	--	--
5.553368521	up	--	--
4.112149027	up	--	--
3.190539716	up	--	--
1.710484575	up	HMDB0042099	--
4.946229541	up	--	--
5.470714414	up	HMDB0042129	--
3.858376165	up	HMDB0043025	--
3.403904882	up	HMDB0042100	--
4.307894782	up	HMDB0005359	--
4.866251449	up	HMDB0042131	--
5.406346431	up	HMDB0043027	--
5.098285684	up	HMDB0005360	--
6.050538426	up	--	--
3.950983292	up	HMDB0005367	--
4.043121079	up	HMDB0005375	--
6.360297532	up	--	--
6.628548742	up	--	--
6.305417936	up	HMDB0005368	--
5.583291216	up	HMDB0005395	--
4.965386424	up	HMDB0005368	--
4.457504188	up	--	--
3.809355588	up	HMDB0044004	--
5.388954442	up	--	--
14.6241058	up	--	--
3.791109789	up	--	--
6.111634849	up	--	--
4.8792999	up	--	--
2.978300433	up	--	--
3.838960187	up	HMDB0042279	--
4.103266364	up	--	--
4.850888695	up	--	--
3.362161748	up	HMDB0043170	--
5.321371638	up	HMDB0047771	--
5.502025446	up	--	--
4.683937382	up	HMDB0042281	--
4.938338278	up	HMDB0042106	--
3.528986002	up	HMDB0043199	--
5.267251175	up	--	--
5.106518458	up	HMDB0010415	--
4.689836198	up	HMDB0010420	--
5.209674826	up	HMDB0010420	--
4.965511953	up	--	--
5.74105671	up	HMDB0043032	--
5.722413788	up	HMDB0043201	--
5.086482886	up	HMDB0005377	--
5.026850704	up	HMDB0010430	--
5.756252779	up	--	--
6.051524805	up	--	--
4.232177239	up	HMDB0005382	--
4.255362544	up	HMDB0005369	--
3.73072643	up	HMDB0005378	--
3.836216613	up	HMDB0042168	--
5.947706034	up	HMDB0062701	--
6.677980068	up	--	--
4.899460227	up	HMDB0005383	--
4.97254637	up	HMDB0005424	--
3.971261761	up	HMDB0005403	--
5.451936605	up	HMDB0044064	--
5.748965845	up	--	--
5.658374725	up	HMDB0005404	--
6.324661709	up	HMDB0044120	--
3.861623534	up	HMDB0046485	--
5.909171562	up	HMDB0044815	--
5.840936984	up	HMDB0044093	--
5.554694964	up	--	--
12.54220788	up	--	--
13.71449199	up	--	--
4.829529089	up	HMDB0047886	--
5.003156258	up	--	--
5.212848992	up	--	--
4.067196969	up	--	--
1.548769664	up	--	--
15.89975901	up	HMDB0047909	--
5.451677899	up	--	--
5.661853512	up	--	--
6.087982852	up	HMDB0047911	--
5.415350782	up	HMDB0042316	--
5.450635097	up	HMDB0044038	--
4.505110994	up	--	--
5.367004951	up	--	--
6.169400108	up	--	--
5.498237231	up	HMDB0048590	--
5.953040385	up	HMDB0005379	--
6.467970907	up	--	--
6.054604098	up	HMDB0011708	--
6.371147736	up	--	--
4.922843998	up	HMDB0005438	--
4.160621351	up	HMDB0005384	--
4.183852203	up	HMDB0042378	--
5.64019663	up	--	--
6.567386221	up	--	--
3.623349925	up	HMDB0005453	--
3.625691822	up	HMDB0005405	--
4.384387692	up	HMDB0044096	--
15.37460432	up	--	--
5.98966782	up	--	--
5.125929682	up	HMDB0005459	--
4.87878579	up	HMDB0005454	--
5.760965921	up	HMDB0048638	--
4.062709679	up	HMDB0005410	--
11.43570884	up	--	--
5.190548338	up	HMDB0010466	--
4.472574827	up	--	--
6.196704777	up	HMDB0005457	--
4.080998579	up	HMDB0049924	--
4.067901631	up	HMDB0049266	--
3.098188691	up	HMDB0047230	--
5.004565091	up	--	--
14.09028092	up	--	--
5.217192431	up	--	--
5.143691664	up	--	--
5.706486261	up	HMDB0047916	--
5.054009749	up	--	--

4.507750433	up	--	--
6.794581482	up	HMDB0042317	--
4.638135506	up	HMDB0042526	--
4.961612463	up	HMDB0005435	--
5.429730752	up	HMDB0042140	--
5.543956104	up	HMDB0044074	--
5.666821723	up	HMDB0011711	--
6.173483451	up	--	--
5.992823569	up	HMDB0005440	--
5.033265878	up	HMDB0005363	--
4.714833635	up	HMDB0005390	--
6.407520146	up	--	--
6.200080659	up	--	--
4.399653653	up	HMDB0005455	--
4.662221739	up	HMDB0005370	--
5.927428489	up	--	--
6.213019412	up	--	--
3.946867779	up	--	--
4.159502353	up	HMDB0005460	--
3.97754907	up	HMDB0044953	--
4.465715816	up	HMDB0048645	--
4.023845933	up	HMDB0049911	--
4.175734242	up	HMDB0049351	--
4.964938736	up	HMDB0049372	--
4.399851524	up	--	--
4.856749632	up	--	--
13.30267594	up	HMDB0047917	--
6.642944114	up	HMDB0048604	--
5.006167086	up	HMDB0010471	--
5.449657415	up	HMDB0048077	--
7.280713362	up	--	--
6.085802778	up	HMDB0042320	--
14.47086102	up	HMDB0043410	--
12.65486804	up	HMDB0043210	--
7.433322774	up	--	--
5.460091504	up	--	--
6.017533932	up	HMDB0044263	--
5.755352899	up	HMDB0048648	--
5.932844461	up	HMDB0005380	--
6.260916703	up	--	--
5.745551623	up	HMDB0043268	--
5.182931594	up	HMDB0005461	--
4.494056761	up	HMDB0005385	--
5.235777025	up	HMDB0048643	--
4.771614804	up	--	--
4.855807296	up	--	--
6.597309724	up	--	--
4.210754668	up	HMDB0045114	--
5.888527015	up	HMDB0044128	--
4.669474216	up	HMDB0049395	--
3.246571076	up	HMDB0005406	--
4.404158422	up	HMDB0005473	--
3.835721871	up	HMDB0052591	--
4.937095267	up	HMDB0049398	--
5.057861882	up	HMDB0044956	--
5.902227322	up	--	--
4.436588393	up	HMDB0052158	--
4.278266702	up	HMDB0048078	--
13.63675467	up	--	--
4.178073819	up	--	--
4.56684082	up	--	--
7.431814103	up	HMDB0044077	--
5.588168229	up	HMDB0048751	--
5.818444592	up	HMDB0044271	--
14.53430964	up	--	--
4.444001793	up	HMDB0005391	--
5.579169947	up	HMDB0010481	--
6.373017424	up	HMDB0044073	--
4.443596233	up	HMDB0010418	--
4.650873175	up	HMDB0044134	--
4.351497313	up	HMDB0005412	--
4.80164259	up	HMDB0043939	--
3.811907922	up	HMDB0010491	--
5.328779283	up	HMDB0010467	--
3.787520856	up	--	--
5.172800635	up	--	--
6.016960941	up	HMDB0042560	--
12.92518352	up	HMDB0048607	--
4.891114129	up	HMDB0044273	--
4.698648962	up	HMDB0044079	--
6.060703641	up	HMDB0049402	--
5.543523389	up	HMDB0010489	--
14.59828517	up	HMDB0043248	--
4.905017558	up	HMDB0005462	--
4.590286159	up	HMDB0044135	--
6.391475507	up	--	--
2.917678365	up	HMDB0044936	--
3.247893647	up	HMDB0010468	--
5.111072601	up	HMDB0049399	--
5.033914418	up	HMDB0044325	--
5.935722434	up	HMDB0047929	--
4.411566203	up	HMDB0052637	--
4.207628115	up	HMDB0048609	--
4.508784951	up	HMDB0044275	--
4.329494635	up	HMDB0048631	--
4.824086907	up	--	--
5.678225106	up	--	--
3.702346016	up	HMDB0049400	--
3.256432977	up	HMDB0010465	--
3.626918417	up	HMDB0045098	--
4.853972845	up	HMDB0010494	--
3.940617312	up	HMDB0049322	--
4.531794857	up	HMDB0044525	--
4.211917009	up	--	--
5.543041181	up	HMDB0048763	--
4.500101135	up	HMDB0005448	--
3.486023985	up	HMDB0049406	--
4.066315452	up	HMDB0052580	--
3.903243568	up	HMDB0044381	--
3.207063097	up	HMDB0050602	--
4.266527518	up	HMDB0010496	--
4.134912717	up	HMDB0005476	--
4.056609615	up	HMDB0052649	--
2.177957267	up	HMDB0049490	--
4.303684086	up	HMDB0044639	--
1.344203816	up	HMDB0052739	--
