# Supplementary material for: The Regulation Network of Glycerolipid Metabolism as Coregulators of Immunotherapy-Related Myocarditis
Source: Cardiovasc Ther. 2023 Jun 21;2023:8774971. doi: 10.1155/2023/8774971 (PMC10307211; doi:10.1155/2023/8774971)
Supplement: Supplementary 7 — Table S7: The KEGG functional enrichment results for differentially expressed metabolites. [file 8774971.f7.pdf]

Table S7

name	Total Cmpd	Hits	Pvalue	Holm p	FDR	foldEnrichment	hitCompound
**Glycerophospholipid metabolism**	36	2	0.028259	1	0.31238	3.6463	Choline,Acetylcholine
**Steroid hormone biosynthesis**	85	4	0.03611	1	0.31238	3.5306	Cholesterol,Aldosterone,Cortisol,Testosterone
**Steroid biosynthesis**	42	2	0.036124	1	0.31238	3.53235	Cholesterol,Calcitriol
**Retinol metabolism**	17	1	0.040496	1	0.31238	3.4516	11-cis-Retinol
**Drug metabolism - other enzymes**	39	1	0.042473	1	0.31238	3.4167	Isonicotinic acid
**Ascorbate and aldarate metabolism**	8	1	0.045556	1	0.31238	3.36415	myo-Inositol
**Phosphatidylinositol signaling system**	28	1	0.045556	1	0.31238	3.36415	myo-Inositol
**Caffeine metabolism**	10	1	0.079947	1	0.39593	2.8825	7-Methyluric acid
**Histidine metabolism**	16	3	0.084442	1	0.39593	2.8283	L-Histidine,Carnosine,Histamine
**beta-Alanine metabolism**	21	4	0.084456	1	0.39593	2.82935	Carnosine,Uracil,L-Histidine,Spermidine
**Glycine, serine and threonine metabolism**	33	8	0.090733	1	0.39593	2.7155	L-Serine,Choline,Betaine,Glycine,Sarcosine,L-Threonine,Creatine,L-Cysteine
**Thiamine metabolism**	7	2	0.12929	1	0.51716	1.87775	L-Cysteine,Thiamine
**Starch and sucrose metabolism**	18	1	0.16479	1	0.60845	2.094	D-Fructose
**Aminoacyl-tRNA biosynthesis**	48	16	0.25936	1	0.85143	1.32375	L-Histidine,L-Phenylalanine,L-Arginine,L-Glutamine,L-Cysteine,Glycine,L-Serine,L-Methionine,L-Valine,L-Alanine,L-Lysine,L-Isoleucine,L-Threonine,L-Tryptophan,L-Tyrosine,L-Proline
**Galactose metabolism**	27	4	0.26607	1	0.85143	1.41255	D-Fructose,Galactitol,D-Mannose,myo-Inositol
**Fructose and mannose metabolism**	20	5	0.32512	1	0.88552	1.1663	D-Fructose,D-Mannose,D-Mannose 1-phosphate,D-Glyceraldehyde 3-phosphate,GDP-L-fucose
**Lysine degradation**	25	1	0.35214	1	0.88552	1.08365	L-Lysine
**Biotin metabolism**	10	2	0.35214	1	0.88552	1.08365	Biotin,L-Lysine
**Porphyrin and chlorophyll metabolism**	30	3	0.36497	1	0.88552	1.0342	Glycine,Biliverdin,Porphobilinogen
**Glutathione metabolism**	28	5	0.40723	1	0.88552	0.88315	Glutathione,Glycine,L-Cysteine,Putrescine,Spermidine
**Amino sugar and nucleotide sugar metabolism**	37	4	0.42611	1	0.88552	0.8462	N-Acetyl-D-glucosamine,D-Mannose 1-phosphate,GDP-L-fucose,D-Mannose
**Riboflavin metabolism**	4	2	0.47847	1	0.88552	0.67305	Riboflavin,FAD
**Arginine biosynthesis**	14	5	0.50428	1	0.88552	0.6452	L-Arginine,N-Acetylornithine,Carbamoyl phosphate,L-Glutamine,Urea
**Arginine and proline metabolism**	38	6	0.50935	1	0.88552	0.83705	L-Arginine,Creatine,Putrescine,Spermidine,L-Proline,cis-4-Hydroxy-D-proline
**Sphingolipid metabolism**	21	1	0.52558	1	0.88552	0.5381	L-Serine
**Pyrimidine metabolism**	39	7	0.52929	1	0.88552	0.5361	L-Glutamine,Carbamoyl phosphate,Uridine,Cytidine,dCDP,dCMP,Uracil
**Nitrogen metabolism**	6	2	0.53082	1	0.88552	0.52985	Carbamoyl phosphate,L-Glutamine
**D-Glutamine and D-glutamate metabolism**	6	1	0.53129	1	0.88552	0.5242	L-Glutamine
**Glyoxylate and dicarboxylate metabolism**	32	3	0.535	1	0.88552	0.52445	L-Serine,Glycine,L-Glutamine
**Tryptophan metabolism**	41	3	0.58285	1	0.92637	0.408685	L-Tryptophan,N-Acetylserotonin,5-Methoxyindoleacetate
**Pantothenate and CoA biosynthesis**	19	4	0.60058	1	0.92637	0.373485	Pantetheine,L-Valine,L-Cysteine,Uracil
**Selenocompound metabolism**	20	1	0.61758	1	0.92637	0.34025	L-Alanine
**alpha-Linolenic acid metabolism**	13	1	0.70922	1	0.952	0.19282	Stearidonic acid
**Inositol phosphate metabolism**	30	2	0.74667	1	0.952	0.18451	myo-Inositol,D-Glyceraldehyde 3-phosphate
**Alanine, aspartate and glutamate metabolism**	28	3	0.75657	1	0.952	0.40632	L-Alanine,L-Glutamine,Carbamoyl phosphate
**Cysteine and methionine metabolism**	33	3	0.76517	1	0.952	0.14933	L-Serine,L-Methionine,L-Cysteine
**Purine metabolism**	65	13	0.79882	1	0.952	0.41902	Xanthine,L-Glutamine,ADP,Adenosine,Deoxyinosine,Xanthosine,Hypoxanthine,Inosine,Guanine,Deoxyguanosine,Guanosine,Adenine,Urea
**Glycolysis / Gluconeogenesis**	26	1	0.81592	1	0.952	0.076075	D-Glyceraldehyde 3-phosphate
**Pentose phosphate pathway**	22	1	0.81592	1	0.952	0.076075	D-Glyceraldehyde 3-phosphate
**Vitamin B6 metabolism**	9	1	0.83046	1	0.952	0.06443	Pyridoxal
**Primary bile acid biosynthesis**	46	3	0.85052	1	0.952	0.06832	Cholesterol,Glycine,Taurine
**Tyrosine metabolism**	42	2	0.8863	1	0.952	0.028838	L-Tyrosine,Thyroxine
**Ubiquinone and other terpenoid-quinone biosynthesis**	9	1	0.8863	1	0.952	0.028838	L-Tyrosine
**Valine, leucine and isoleucine degradation**	40	2	0.8898	1	0.952	0.09873	L-Valine,L-Isoleucine
**Valine, leucine and isoleucine biosynthesis**	8	3	0.8925	1	0.952	0.098745	L-Threonine,L-Isoleucine,L-Valine
**Phenylalanine metabolism**	10	2	0.95973	1	0.96368	0.02528	L-Phenylalanine,L-Tyrosine
**Phenylalanine, tyrosine and tryptophan biosynthesis**	4	2	0.95973	1	0.96368	0.02528	L-Phenylalanine,L-Tyrosine
**Taurine and hypotaurine metabolism**	8	3	0.96368	1	0.96368	0.00316665	L-Cysteine,Taurine,Hypotaurine

member	reference
Phosphatidylethanolamine; Phosphatidylcholine; Glycerone phosphate; 1-Acyl-sn-glycero-3-phosphocholine; CDP-choline; 1,2-Diacyl-sn-glycerol; Choline phosphate; Choline; Acetylcholine; Ethanolamine phosphate; Ethanc	http://www.genome.jp/kegg-bin/show_pathway?hsa00564
Cholesterol; 20alpha,22beta-Dihydroxycholesterol; 20alpha-Hydroxycholesterol; 17alpha,20alpha-Dihydroxycholesterol; Pregnenolone; 21-Hydroxypregnenolone; 17alpha-Hydroxypregnenolone; 17alpha,21-Dihydroxypregne	http://www.genome.jp/kegg-bin/show_pathway?hsa00140
Vitamin D3; Delta7-Avenasterol; Zymosterol; Calcidiol; 7-Dehydrocholesterol; Lathosterol; 5alpha-Cholest-8-en-3beta-ol; Episterol; 24-Methylenecholesterol; 5-Dehydroepisterol; Cholesterol; 7-Dehydrodesmosterol; 14-Deme	http://www.genome.jp/kegg-bin/show_pathway?hsa00100
Retinol; Retinal; beta-Carotene; 11-cis-Retinal; Retinyl ester; 9-cis-Retinol; 11-cis-Retinol; Retinoate; 9-cis-Retinal; all-trans-13,14-Dihydroretinol; 11-cis-Retinyl palmitate; all-trans-4-Hydroxyretinoic acid; all-trans-18-Hydrox	http://www.genome.jp/kegg-bin/show_pathway?hsa00830
Mercaptopurine (INN); 6-Thioinosine-5'-monophosphate; 6-Thioguanosine monophosphate; 6-Methylmercaptopurine; Tioguanine (INN); 6-Thioxanthine 5'-monophosphate; 6-Mercaptopurine ribonucleoside triphosphate; Iso	http://www.genome.jp/kegg-bin/show_pathway?hsa00983
myo-Inositol; UDP-glucose; L-Gulono-1,4-lactone; D-Glucuronolactone; UDP-glucuronate; D-Glucuronate; L-Gulonate; D-Glucarate	http://www.genome.jp/kegg-bin/show_pathway?hsa00053
1-Phosphatidyl-1D-myo-inositol 3-phosphate; 1D-myo-Inositol 1,3,4,5,6-pentakisphosphate; 1-Phosphatidyl-D-myo-inositol; 1-Phosphatidyl-D-myo-inositol 4,5-bisphosphate; myo-Inositol; CDP-diacylglycerol; 1-Phosphatid	http://www.genome.jp/kegg-bin/show_pathway?hsa04070
1,7-Dimethylxanthine; 1-Methylxanthine; Theobromine; 7-Methylxanthine; Caffeine; 1-Methyluric acid; 3,7-Dimethyluric acid; 1,7-Dimethyluric acid; 5-Acetylamino-6-formylamino-3-methyluracil; 7-Methyluric acid	http://www.genome.jp/kegg-bin/show_pathway?hsa00232
L-Glutamate; 4-Imidazolone-5-propanoate; Urocanate; L-Histidine; Carnosine; Imidazole-4-acetaldehyde; N-Methylhistamine; Methylimidazole acetaldehyde; Histamine; N(pi)-Methyl-L-histidine; N-Formyl-L-aspartate; N-Forr	http://www.genome.jp/kegg-bin/show_pathway?hsa00340
Propanoyl-CoA; 3-Hydroxypropionyl-CoA; 3-Hydroxypropanoate; Malonyl-CoA; 3-Oxopropanoate; beta-Alanine; L-Aspartate; 1,3-Diaminopropane; 3-Aminopropanal; 3-Ureidopropionate; 5,6-Dihydrouracil; Carnosine; beta-A	http://www.genome.jp/kegg-bin/show_pathway?hsa00410
L-Serine; Choline; Betaine aldehyde; Betaine; Guanidinoacetate; 3-Phospho-D-glycerate; N,N-Dimethylglycine; L-Cystathionine; Glycine; O-Phospho-L-serine; Sarcosine; 5,10-Methylenetetrahydrofolate; L-Threonine; Lipoylp	http://www.genome.jp/kegg-bin/show_pathway?hsa00260
[Enzyme]-cysteine; L-Cysteine; Thiamine; Thiamin triphosphate; Thiamin diphosphate; Thiamin monophosphate; [Enzyme]-S-sulfanylcysteine	http://www.genome.jp/kegg-bin/show_pathway?hsa00730
Cellodextrin; Cellobiose; D-Fructose; Sucrose; beta-D-Glucoside; UDP-glucose; D-Glucose 1-phosphate; D-Glucose 6-phosphate; D-Glucose; Amylose; alpha,alpha-Trehalose; Maltodextrin; Starch; Maltose; Dextrin; Isomalt	http://www.genome.jp/kegg-bin/show_pathway?hsa00500
L-Asparagine; L-Histidine; L-Phenylalanine; L-Arginine; L-Glutamine; L-Cysteine; Glycine; L-Aspartate; L-Serine; L-Methionine; L-Valine; L-Alanine; L-Lysine; L-Isoleucine; L-Leucine; L-Threonine; L-Tryptophan; L-Methionyl-t	http://www.genome.jp/kegg-bin/show_pathway?hsa00970
Stachyose; D-Tagatose 6-phosphate; D-Gal alpha 1->6D-Gal alpha 1->6D-Glucose; Sucrose; Raffinose; Melibiose; D-Galactose; 3-beta-D-Galactosyl-sn-glycerol; Epimelibiose; Melibiitol; alpha-D-Galactosyl-(1->3)-1D-myo-i	http://www.genome.jp/kegg-bin/show_pathway?hsa00052
D-Sorbitol; D-Fructose; D-Mannose; beta-D-Fructose 2,6-bisphosphate; D-Mannose 6-phosphate; L-Fucose 1-phosphate; GDP-4-dehydro-6-deoxy-D-mannose; GDP-mannose; D-Mannose 1-phosphate; beta-D-Fructose 6	http://www.genome.jp/kegg-bin/show_pathway?hsa00051
L-Lysine; 4-Trimethylammoniobutanoate; 4-Trimethylammoniobutanal; N6,N6,N6-Trimethyl-L-lysine; Protein N6-methyl-L-lysine; Protein lysine; Procollagen 5-hydroxy-L-lysine; Protein N6,N6-dimethyl-L-lysine; Crotonoyl-CoA	http://www.genome.jp/kegg-bin/show_pathway?hsa00310
Biotin; Biotinyl-5'-AMP; Biocytin; Glutaryl-[acp] methyl ester; Malonyl-[acyl-carrier protein]; Malonyl-[acp] methyl ester; Holo-[carboxylase]; L-Lysine; 3-Ketopimeloyl-[acp] methyl ester; 3-Ketoglutaryl-[acp] methyl ester	http://www.genome.jp/kegg-bin/show_pathway?hsa00780
Cobinamide; Heme O; Heme; Glycine; Bilirubin beta-diglucuronide; Cob(I)yrinate a,c diamide; Fe2+; Biliverdin; Cob(I)alamin; Protoporphyrinogen IX; Coproporphyrinogen III; Uroporphyrinogen III; Hydroxymethylbilane; Porphc	http://www.genome.jp/kegg-bin/show_pathway?hsa00860
R-S-Cysteinylglycine; R-S-Glutathione; Glutathione; NADP+; Glutathione disulfide; NADPH; Glycine; gamma-L-Glutamyl-L-cysteine; L-Glutamate; L-Cysteine; Cys-Gly; 5-Oxoproline; L-Amino acid; (5-L-Glutamyl)-L-amino ac	http://www.genome.jp/kegg-bin/show_pathway?hsa00480
N-Acetyl-D-glucosamine; N-Acetyl-D-glucosamine 6-phosphate; N-Acetyl-alpha-D-glucosamine 1-phosphate; UDP-N-acetyl-alpha-D-glucosamine; N-Acetyl-D-mannosamine; N-Acetylneuraminate 9-phosphate; N-Acetylneu	http://www.genome.jp/kegg-bin/show_pathway?hsa00520
Riboflavin; FMN; FAD; Reduced riboflavin	http://www.genome.jp/kegg-bin/show_pathway?hsa00740
L-Glutamate; L-Arginine; N-Acetylornithine; N-(L-Arginino)succinate; L-Citrulline; L-Aspartate; Carbamoyl phosphate; L-Ornithine; Ammonia; L-Glutamine; 2-Oxoglutarate; N-Acetyl-L-glutamate; Urea; Fumarate	http://www.genome.jp/kegg-bin/show_pathway?hsa00220
L-Arginine; N(omega)-Hydroxyarginine; Guanidinoacetate; Creatine; 4-Aminobutanoate; Agmatine; Putrescine; 4-Aminobutyraldehyde; S-Adenosylmethioninamine; S-Adenosyl-L-methionine; Spermidine; N-Acetylputrescine;	http://www.genome.jp/kegg-bin/show_pathway?hsa00330
Dihydroceramide; Ceramide 1-phosphate; Sphingosine 1-phosphate; Sphinganine 1-phosphate; Phytoceramide; Sphinganine; Sphingomyelin; Sphingosine; N-Acylsphingosine; L-Serine; Palmitoyl-CoA; 3-Dehydrosphinganir	http://www.genome.jp/kegg-bin/show_pathway?hsa00600
UDP; L-Glutamine; Carbamoyl phosphate; (S)-Dihydroorotate; Orotidine 5'-phosphate; UMP; UTP; Uridine; 5,6-Dihydrouracil; 3-Ureidopropionate; CTP; CDP; CMP; Cytidine; dCDP; dCMP; dCTP; Deoxycytidine; dUTP; dUDF	http://www.genome.jp/kegg-bin/show_pathway?hsa00240
CO2; Ammonia; L-Glutamate; HCO3-; Carbamoyl phosphate; L-Glutamine	http://www.genome.jp/kegg-bin/show_pathway?hsa00910
D-Glutamate; L-Glutamate; D-Glutamine; L-Glutamine; 5-Oxo-D-proline; 2-Oxoglutarate	http://www.genome.jp/kegg-bin/show_pathway?hsa00471
Hydroxypyruvate; cis-Aconitate; 4-Hydroxy-2-oxoglutarate; N-Formylderivatives; Glycolate; Citrate; (S)-Malate; 2-Phosphoglycolate; Acetyl-CoA; (S)-Methylmalonyl-CoA; Propanoyl-CoA; (R)-Methylmalonyl-CoA; Glyoxylate; I	http://www.genome.jp/kegg-bin/show_pathway?hsa00630
L-Tryptophan; Melatonin; N-Acetylserotonin; Serotonin; 5-Hydroxyindoleacetate; 5-Hydroxykynurenamine; 5-Hydroxykynurenine; 5-Hydroxy-L-tryptophan; L-Formylkynurenine; Acetoacetyl-CoA; (S)-3-Hydroxybutanoyl-CoA;	http://www.genome.jp/kegg-bin/show_pathway?hsa00380
Dephospho-CoA; Pantetheine 4'-phosphate; Pantetheine; (R)-4'-Phosphopantothenoyl-L-cysteine; N-((R)-Pantothenoyl)-L-cysteine; Pantothenate; 3-Ureidopropionate; 5,6-Dihydrouracil; L-Valine; L-Aspartate; Apo-[acyl-carri	http://www.genome.jp/kegg-bin/show_pathway?hsa00770
Selenite; Hydrogen selenide; L-Selenocysteine; L-Selenomethionine; Se-Methyl-L-selenocysteine; O-Phosphoseryl-tRNA(Sec); Selenophosphoric acid; L-Seryl-tRNA(Sec); Selenate; L-Selenocystathionine; Selenohomocystei	http://www.genome.jp/kegg-bin/show_pathway?hsa00450
OPC4-CoA; OPC6-CoA; 3-Oxo-OPC4-CoA; 3-Oxo-OPC6-CoA; 3-Oxo-OPC8-CoA; OPC8-CoA; Phosphatidylcholine; (9Z,12Z,15Z)-Octadecatrienoic acid; trans-2-Enoyl-OPC4-CoA; trans-2-Enoyl-OPC6-CoA; (+)-7-Isojasmo	http://www.genome.jp/kegg-bin/show_pathway?hsa00592
1-Phosphatidyl-1D-myo-inositol 5-phosphate; 1-Phosphatidyl-D-myo-inositol 4,5-bisphosphate; D-myo-Inositol 1,4,5-trisphosphate; 1D-myo-Inositol 1,4,5,6-tetrakisphosphate; Phosphatidylinositol-3,4,5-trisphosphate; 1D-r	http://www.genome.jp/kegg-bin/show_pathway?hsa00562
N-Acetyl-L-aspartate; 2-Oxosuccinamate; L-Aspartate; L-Asparagine; D-Aspartate; N-(L-Arginino)succinate; N6-(1,2-Dicarboxyethyl)-AMP; L-Alanine; Succinate semialdehyde; L-Glutamate; 4-Aminobutanoate; L-Glutamine; /	http://www.genome.jp/kegg-bin/show_pathway?hsa00250
4-Methylthio-2-oxobutanoic acid; 1,2-Dihydroxy-5-(methylthio)pent-1-en-3-one; S-Methyl-5-thio-D-ribulose 1-phosphate; S-Methyl-5-thio-D-ribose 1-phosphate; 5'-Methylthioadenosine; S-Adenosylmethioninamine; S-Aden	http://www.genome.jp/kegg-bin/show_pathway?hsa00270
GDP; Xanthine; D-Ribose 5-phosphate; L-Glutamine; 5-Phospho-alpha-D-ribose 1-diphosphate; 5-Phosphoribosylamine; 5'-Phosphoribosylglycinamide; 2-(Formamido)-N1-(5'-phosphoribosyl)acetamidine; 1-(5'-Phosphoribc	http://www.genome.jp/kegg-bin/show_pathway?hsa00230
Acetaldehyde; Ethanol; Thiamin diphosphate; Pyruvate; 2-(alpha-Hydroxyethyl)thiamine diphosphate; Enzyme N6-(lipoyl)lysine; Acetyl-CoA; Enzyme N6-(dihydrolipoyl)lysine; (S)-Lactate; Phosphoenolpyruvate; 2-Phospho-D-(	http://www.genome.jp/kegg-bin/show_pathway?hsa00010
alpha-D-Glucose 6-phosphate; Deoxyribose; 2-Deoxy-D-ribose 1-phosphate; 2-Deoxy-D-ribose 5-phosphate; D-Ribose 5-phosphate; alpha-D-Ribose 1-phosphate; D-Ribose; Sedoheptulose 7-phosphate; D-Glyceraldehyde	http://www.genome.jp/kegg-bin/show_pathway?hsa00030
O-Phospho-4-hydroxy-L-threonine; Pyridoxine phosphate; Pyridoxamine phosphate; Pyridoxine; Pyridoxal phosphate; Pyridoxal; Pyridoxamine; 2-Oxo-3-hydroxy-4-phosphobutanoate; 4-Pyridoxate	http://www.genome.jp/kegg-bin/show_pathway?hsa00750
Cholesterol; Cholest-5-ene-3beta,26-diol; 25-Hydroxycholesterol; 4-Cholesten-7alpha,12alpha-diol-3-one; 7alpha-Hydroxycholest-4-en-3-one; 7alpha,12alpha-Dihydroxy-5beta-cholestan-3-one; 3alpha,7alpha,12alpha-Trihy	http://www.genome.jp/kegg-bin/show_pathway?hsa00120
L-Dopachrome; L-Normetanephrine; 3-Methoxy-4-hydroxyphenylglycolaldehyde; L-Noradrenaline; L-Adrenaline; 3,4-Dihydroxymandelate; 3,4-Dihydroxymandelaldehyde; 3,4-Dihydroxyphenylethyleneglycol; L-Metanephrine;	http://www.genome.jp/kegg-bin/show_pathway?hsa00350
3-(4-Hydroxyphenyl)pyruvate; L-Tyrosine; Menaquinone; 2,3-Epoxymenaquinone; Phylloquinol; Phylloquinone; Menaquinol; Vitamin K1 epoxide; Homogentisate	http://www.genome.jp/kegg-bin/show_pathway?hsa00130
Enzyme N6-(lipoyl)lysine; 2-Methyl-1-hydroxybutyl-ThPP; Enzyme N6-(dihydrolipoyl)lysine; 2-Methyl-1-hydroxypropyl-ThPP; 3-Methyl-1-hydroxybutyl-ThPP; Acetyl-CoA; Acetoacetyl-CoA; Acetoacetate; (S)-3-Hydroxy-3-met	http://www.genome.jp/kegg-bin/show_pathway?hsa00280
L-Threonine; (S)-3-Methyl-2-oxopentanoic acid; L-Leucine; 3-Methyl-2-oxobutanoic acid; 2-Oxobutanoate; L-Isoleucine; 4-Methyl-2-oxopentanoate; L-Valine	http://www.genome.jp/kegg-bin/show_pathway?hsa00290
Phenylacetaldehyde; L-Phenylalanine; Phenethylamine; Phenylpyruvate; Benzoyl-CoA; Phenylacetic acid; 2-Hydroxyphenylacetate; 2-Hydroxy-3-phenylpropenoate; Hippurate; L-Tyrosine	http://www.genome.jp/kegg-bin/show_pathway?hsa00360
Phenylpyruvate; L-Phenylalanine; L-Tyrosine; 3-(4-Hydroxyphenyl)pyruvate	http://www.genome.jp/kegg-bin/show_pathway?hsa00400
L-Cysteate; L-Cysteine; Cysteamine; 3-Sulfino-L-alanine; Taurine; Hypotaurine; 5-L-Glutamyl-taurine; Taurocholate	http://www.genome.jp/kegg-bin/show_pathway?hsa00430
